# Flexible open conformation of the AP-3 complex explains its role in cargo recruitment at the Golgi

**DOI:** 10.1016/j.jbc.2021.101334

**Published:** 2021-10-22

**Authors:** Jannis Schoppe, Evelyn Schubert, Amir Apelbaum, Erdal Yavavli, Oliver Birkholz, Heike Stephanowitz, Yaping Han, Angela Perz, Oliver Hofnagel, Fan Liu, Jacob Piehler, Stefan Raunser, Christian Ungermann

**Affiliations:** 1 Department of Biology/Chemistry, Biochemistry Section, Osnabrück University, Osnabrück, Germany; 2 Department of Structural Biochemistry, Max-Planck Institute of Molecular Physiology, Dortmund, Germany; 3 Department of Biology/Chemistry, Biophysics Section, Osnabrück University, Osnabrück, Germany; 4 Leibniz-Forschungsinstitut für Molekulare Pharmakologie (FMP), Campus Berlin-Buch, Berlin, Germany; 5 Center of Cellular Nanoanalytics Osnabrück (CellNanOs), Osnabrück University, Osnabrück, Germany

**Keywords:** AP, adapter protein, DOPC, di-oleoyl-phosphatidyl-choline, DSSO, disuccinimidyl sulfoxide, GEF, guanine nucleotide exchange factor, PEG, polyethyleneglycol, PI4P, phosphatidylinositol-4-phosphate, PSM, polymer-supported lipid membrane, TGN, trans-Golgi network

## Abstract

Vesicle formation at endomembranes requires the selective concentration of cargo by coat proteins. Conserved adapter protein complexes at the Golgi (AP-3), the endosome (AP-1), or the plasma membrane (AP-2) with their conserved core domain and flexible ear domains mediate this function. These complexes also rely on the small GTPase Arf1 and/or specific phosphoinositides for membrane binding. The structural details that influence these processes, however, are still poorly understood. Here we present cryo-EM structures of the full-length stable 300 kDa yeast AP-3 complex. The structures reveal that AP-3 adopts an open conformation in solution, comparable to the membrane-bound conformations of AP-1 or AP-2. This open conformation appears to be far more flexible than AP-1 or AP-2, resulting in compact, intermediate, and stretched subconformations. Mass spectrometrical analysis of the cross-linked AP-3 complex further indicates that the ear domains are flexibly attached to the surface of the complex. Using biochemical reconstitution assays, we also show that efficient AP-3 recruitment to the membrane depends primarily on cargo binding. Once bound to cargo, AP-3 clustered and immobilized cargo molecules, as revealed by single-molecule imaging on polymer-supported membranes. We conclude that its flexible open state may enable AP-3 to bind and collect cargo at the Golgi and could thus allow coordinated vesicle formation at the *trans*-Golgi upon Arf1 activation.

Eukaryotic cells have membrane-enclosed organelles, which carry out specialized functions, including compartmentalized biochemical reactions, metabolic channeling, and regulated signaling, inside a single cell. The transport of proteins, lipids, and other molecules between these organelles is mediated largely by small vesicular carriers that bud off at a donor compartment and fuse with the target membrane to deliver their cargo. The generation of these vesicles has been subject to extensive studies and has led to the identification of numerous coat proteins that are required for their formation at different sites (1, 2). Coat proteins can be monomers, but in most cases, they consist of several proteins, which form a heteromeric complex.

Heterotetrameric adapter protein (AP) complexes are required at several endomembranes for cargo binding. Five well-conserved AP-complexes with differing functions have been identified in mammalian cells, named AP-1–AP-5, of which three (AP-1–AP-3) are conserved from yeast to human (3, 4). The three conserved adapter complexes function at different membranes along the endomembrane system. AP-1 is required for cargo transport between the Golgi and the endosome, AP-2 is required for cargo recognition and transport between the plasma membrane and the early endosome. Finally, AP-3 functions between the trans Golgi and the vacuole in yeast, whereas mammalian AP-3 localizes to a tubular endosomal compartment, in addition to or instead of the TGN (2, 5, 6).

Each of the complexes consists of four different subunits: two large adaptins (named α−ζ and β1-5 respectively), a medium-sized subunit (μ1-5), and a small subunit (σ1-5). While μ- and σ-subunits together with the N-termini of the large adaptins build the membrane-binding core of the complex, the C-termini of both adaptins contain the ear domains, which are connected *via* flexible linkers (2). The recruitment of these complexes to membranes is not entirely conserved. They all require cargo binding, yet AP-1 binds Arf1-GTP with the γ and β1 subunit and phosphatidylinositol-4-phosphate (PI4P) *via* a proposed conserved site on its γ-subunit (7, 8). AP-2, on the other hand, interacts with PI(4,5)P_2_ at the plasma membrane *via* its α, β2, and μ2 subunits (9, 10, 11).

Several studies have uncovered how AP-3 functions in cargo sorting in yeast. AP-3 recognizes cargo at the Golgi *via* two sorting motifs in the cytosolic segments of membrane proteins: a Yxxφ sorting motif, as found in yeast in the SNARE Nyv1 or the Yck3 casein kinase, which binds to a site in μ3, as shown for mammalian AP-3, which is similar to μ2 in AP-2 (12, 13, 14), and dileucine motifs as found in the yeast SNARE Vam3 or the alkaline phosphatase Pho8, potentially also at a site comparable to AP-1 and AP-2 (15, 16). Unlike AP-1 and AP-2-coated vesicles, which depend on clathrin for their formation (2, 17), AP-3 vesicle formation in yeast does not require clathrin or the HOPS subunit Vps41 (18), yet Vps41 is required at the vacuole to bind AP-3 vesicles prior to fusion (19, 20, 21, 22). Studies in metazoan cells revealed that Vps41 and AP-3 function in regulated secretion (23, 24, 25), and AP-3 is required for biogenesis of lysosome-related organelles (26). This suggests that the AP-3 complex has features that are quite different from AP-1 and AP-2 complexes, which cooperate with clathrin in vesicle formation (2).

Among the three conserved AP complexes, the function of the AP-3 complex is the least understood. Arf1 is necessary for efficient AP-3 vesicle generation in mammalian cells and shows a direct interaction with the β3 and δ subunits of AP-3 (27, 28). In addition, *in vitro* experiments on mammalian AP-3 using liposomes or enriched Golgi membranes suggest Arf1 as an important factor in AP-3 recruitment, whereas acidic lipids do not have a major effect, in contrast to what was found for AP-1 and AP-2 (7, 11, 29, 30). Another study showed that membrane recruitment of AP-3 depends on the recognition of sorting signals in cargo tails and PI3P (31), similar to AP-1 recruitment *via* cargo tails, Arf1 and PI4P (32).

However, since AP-1 and AP-3 are both recruited to the trans-Golgi network (TGN) in yeast (33), the mechanism of their recruitment likely differs. Even though Arf1 is required, yeast AP-3 seems to be present at the TGN *before* the arrival of the Arf1 guanine nucleotide exchange factor (GEF) Sec7 (33). This implies the necessity for additional factors at the TGN and a distinct mechanism to allow for spatial and temporal separation of AP-1 and AP-3 recruitment to membranes. Structural data on mammalian AP-1 and AP-2 “core” complexes without the hinge and ear domains of their large subunits revealed that both exist in at least two very defined conformational states: a “closed” cytosolic state, where the cargo-binding sites are buried within the complex, and an “open” state, where the same sites are available to bind cargo (7, 8, 10, 34, 35). Binding of Arf1 to AP-1 or PI(4,5)P_2_ in case of AP-2 induces a conformational change in the complexes that enables them to bind cargo molecules carrying a conserved acidic di-Leucine or a Tyrosine-based motif, as for all three AP complexes in yeast (8, 34). Additional conformational states and intermediates have been reported for both, mammalian AP-1 and AP-2 complex. AP-1, for example, can be hijacked by the human immunodeficiency virus-1 (HIV-1) proteins viral protein u (Vpu) and negative factor (Nef), resulting in a hyper-open conformation of AP-1 (36, 37).

An emerging model over the past years has suggested that APs have several binding sites that allow for the stabilization of membrane binding and the open conformation of the complexes, but there are initial interactions required that dictate their recruitment to the target membrane. Although these interaction sites for mammalian AP-1 and AP-2 have been identified in great detail based on interaction analyses and structural studies (8, 10, 11, 35, 36, 38, 39), structural data for AP-3 is largely missing. The C-terminal part of the μ-subunit of mammalian AP-3 has been crystallized together with a Yxxφ motif-containing a cargo peptide, which revealed a similar fold and cargo-binding site as shown for AP-1 and AP-2 (14). However, positively charged binding surfaces required for PIP-interaction were not well conserved. Although the “trunk” segment of AP-1 and AP-2 is known quite well by now, information on hinge and ear domains in context of these complexes is largely missing. Crystal structures of the isolated ear domains of α-, γ- and β2-adaptin have been published (40, 41, 42), and a study on mammalian AP-3 suggested a direct interaction between δ-ear and δ3 that interfered with Arf1-binding (43). Furthermore, during tethering of AP-3 vesicles with the yeast vacuole, the δ−subunit Apl5 of the yeast AP-3 complex binds to the Vps41 subunit of the HOPS complex as a prerequisite of fusion (18, 19, 21, 22).

In this study, we applied single particle electron cryo-microscopy (cryo-EM) to analyze the purified full-length AP-3 complex from yeast and unraveled the factors required for AP-3 recruitment to membranes by biochemical reconstitution. Our data reveal that a surprisingly flexible AP-3 complex requires a combination of cargo, PI4P, and Arf1 for membrane binding, which explains its function in selective cargo sorting at the Golgi.

## Results

### Isolation of full-length AP-3 and AP-1 complex

**Figure 1:**
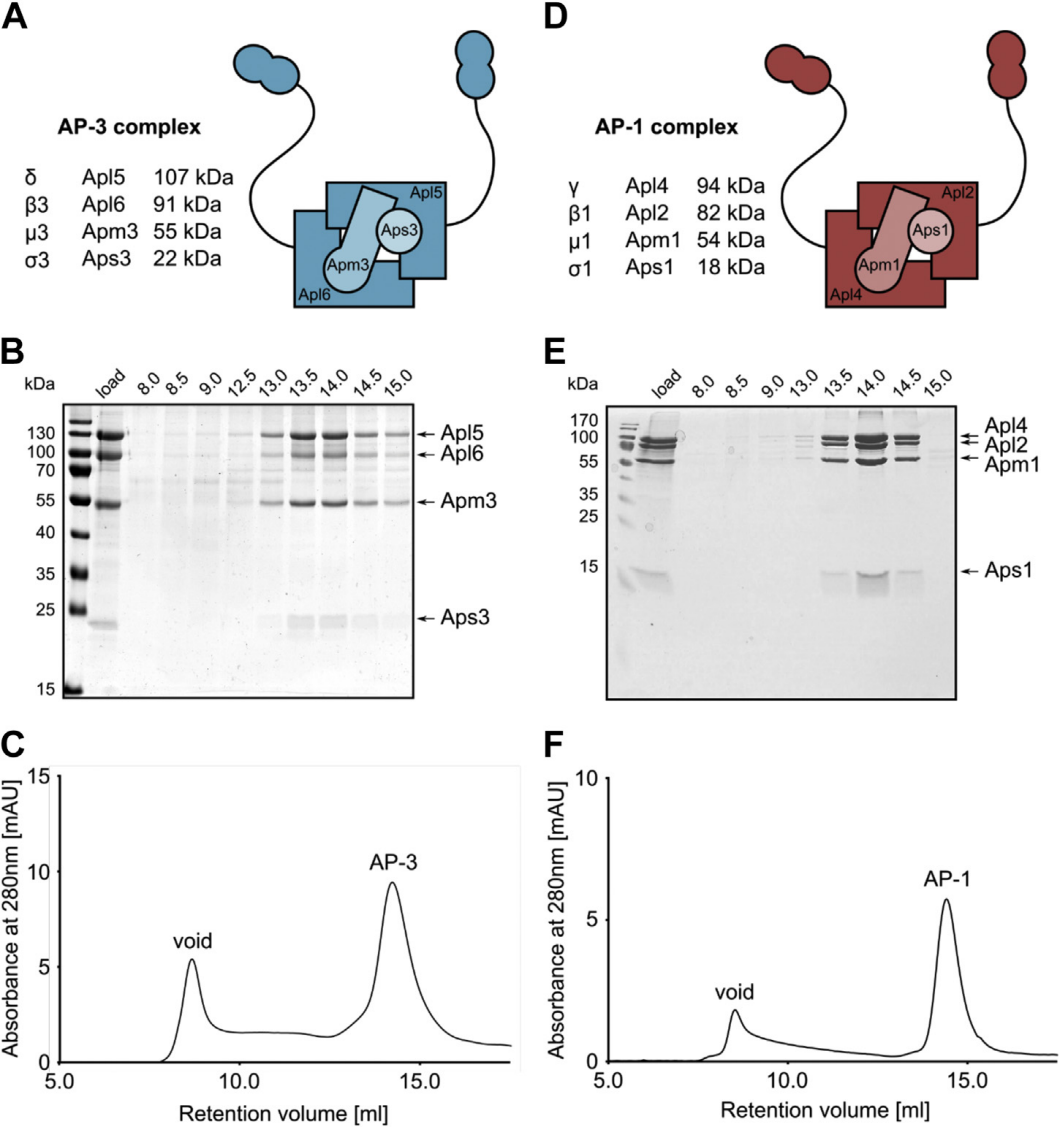
**Purification of full-length AP-3 and AP-1 complexes from yeast**. *A*, scheme of AP-3 composition and list of single subunits with their respective molecular weight in kDa. *B* and *C*, affinity chromatography and size-exclusion chromatography. Overexpressed AP-3 was purified *via* a C-terminal 3xFLAG-tag on Apl5 and subsequently run on a Superose 6 Increase column. *B*, eluted fractions were boiled in sample buffer and analyzed by Coomassie staining following SDS-PAGE. Load corresponds to the sample before gel filtration. *C*, chromatogram with the absorption over the retention volume. Elution of AP-3 is indicated. Void corresponds to the exclusion volume of the column. *D*, scheme of AP-1 composition and list of single subunits with their respective molecular weight in kDa. *E* and *F*, affinity chromatography and size-exclusion chromatography. Overexpressed AP-1 was purified *via* a C-terminal 3xFLAG-tag on Apl4 and analyzed by gel filtration as AP-3. *E*, eluted fractions were boiled in sample buffer and analyzed by Coomassie staining following SDS-PAGE. Load corresponds to the sample before gel filtration. *F*, chromatogram reflects absorption over the retention volume, and elution of AP-1 is indicated.

The mammalian adapter protein complexes AP-1 and AP-2 have been analyzed in the past in some detail by structural and functional analyses. However, little is known about the AP-3 complex. AP-3 vesicle biogenesis requires Arf1 (27), though it is not clear which other factors are required for AP-3 recruitment and function at the Golgi. We therefore generated a yeast strain to overproduce and purify the heterotetrameric full-length AP-3 complex for further analyses regarding its structure and membrane targeting (Fig. 1*A*). For this, all subunits, the δ-subunit Apl5, the β3-subunit Apl6, the μ3-subunit Apm3, and the σ-subunit Aps3, were placed under the control of the inducible *GAL1*-promoter. The AP-3 complex was then purified by affinity purification *via* a C-terminal 3xFLAG-tag on Apl5, which does not impair function *in vivo* (19, 21), and subsequent gel filtration. The complex appeared as a stable heterotetramer with an apparent equimolar stoichiometry of the subunits and eluted as a single peak, corresponding to about 300 kDa (Fig. 1, *B* and *C*). During the purification process, we did not have any evidence for aggregation or proteolysis, indicating that the full-length complex includes the ear domains. As a control for functional assays, we further overproduced all subunits and purified the full-length AP-1 complex *via* a 3xFLAG-tag on the respective γ-adaptin Apl4 (Fig. 1*D*). As for the AP-3 complex, AP-1 forms a full-length heterotetrameric complex in solution (Fig. 1, *E* and *F*).

### Structural analysis of the full-length yeast AP-3 complex

To understand the mechanistic differences between AP-3 and the other adaptor protein complexes at the molecular level, we analyzed the full-length yeast AP-3 complex by single particle cryo-EM (Fig. S1*A*). We selected ∼1 million single particles and processed them in SPHIRE (44, 45). The 2D class averages and the 3D reconstruction suggested that AP-3 adapts an open conformation (Fig. S1). Although the 2D class averages of the AP-3 complex showed distinct secondary structure elements suggesting a potential for high resolution, the 3D reconstruction was limited to ∼15 Å, indicating a high flexibility or heterogeneity of the complex.

Indeed, 3D sorting in SPHIRE (44) resulted in five classes, particularly in the positions of the N-terminal domains of the Apl5 and Apl6 subunits vary (Fig. S1*A*, Movie S1). Although each class contained between 100k–150k particles, the resolution of the 3D reconstructions did not improve after 3D sorting, indicating that the particles in the five classes were still too heterogenous. Therefore, we applied the neural network-based approach of cryoDRGN (46, 47) to better separate different conformations of the AP-3 complex (see Experimental procedures). The 20 reconstructions that we obtained at resolutions from 7.9 Å to 13.5 Å differ considerably in their conformations (Movie S2), indicating that the AP-3 complex is highly flexible in solution. The position and conformation of almost all subunits in the complex are highly variable, which becomes especially apparent when morphing between the different maps (Movie S2).

**Figure 2:**
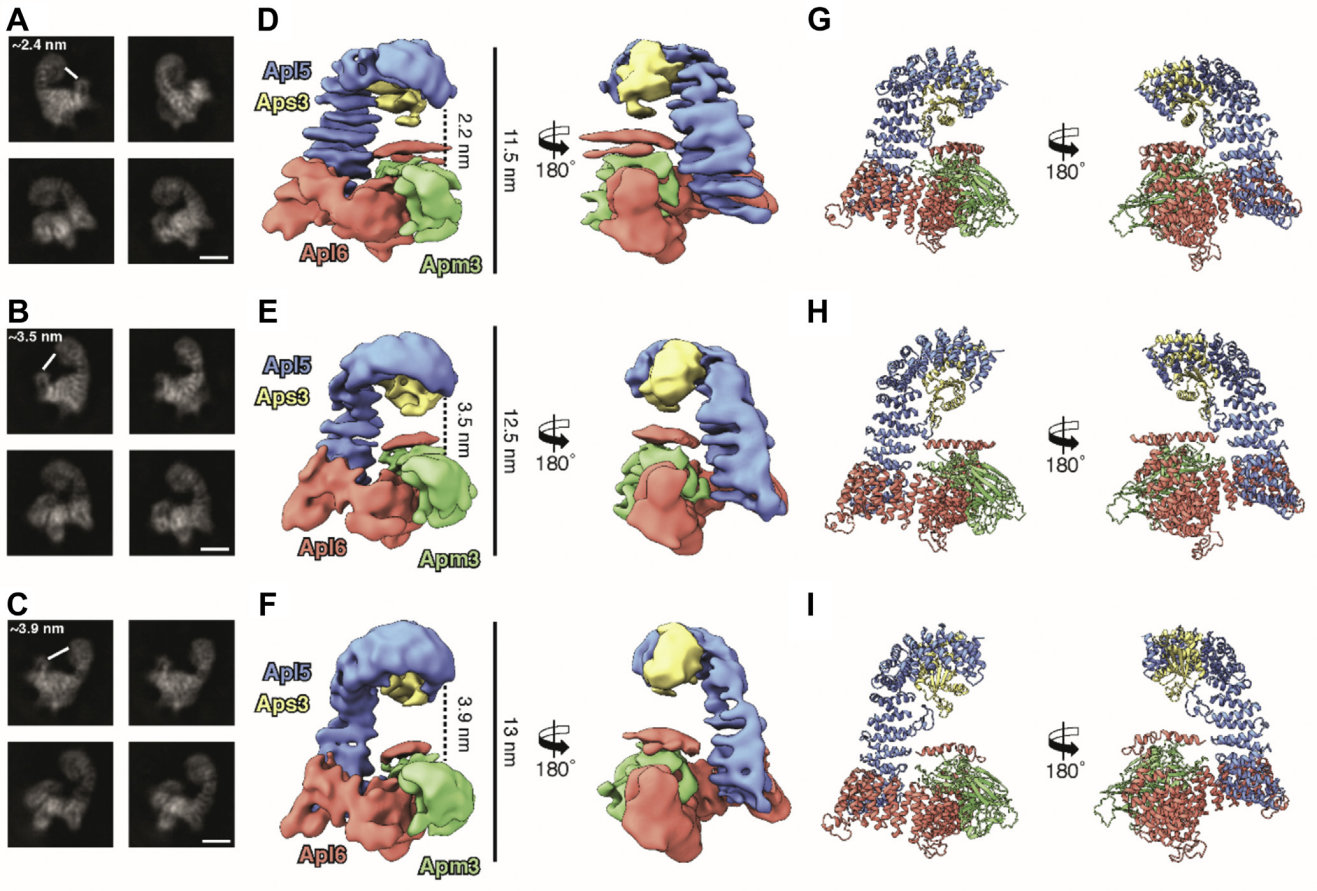
**Representative cryo-EM structures of the compact, intermediate, and stretched conformations of the AP-3 complex**. *A–C*, representative 2D class averages of the cryoDRGN classes corresponding to putative compact (*A*), intermediate (*B*), and stretched (*C*) conformation of the full-length AP-3 complex in solution. Scale bar: 5 nm. *D–F*, rotated side views of representative cryo-EM density maps of the compact (*D*), intermediate (*E*), and stretched (*F*) conformation of the AP-3 complex. The maps were segmented into the four subunits Apl5, Apl6, Apm3, and Aps3 that are depicted in *blue, red, green*, and *gold*, respectively. Segmented maps were generated in UCSF Chimera and are shown at different thresholds for visualization with thresholds of 0.016 for Apl5, 0.016 for Apl6, 0.0182 for Apm3, and 0.015 for Aps3. *G–I*, rotated side views of representative compact (*G*), intermediate (*H*), and stretched (*I*) AP-3 homology models generated by flexible fitting of trRosetta calculated structures of the four subunits into the 3D reconstructions.

We selected three classes, which showed the most distinct conformational differences based on the gap created between the N-Apl5 and C-Apm3 subunits as well as the overall length of the AP-3 complex in the 2D class averages and the 3D reconstructions (Fig. 2, *A*–*F*). Indeed, the overall length of the AP-3 complex varied from 11.5 nm in its most compact to 13 nm in its stretched form. Similarly, the distance between the N-Apl5 and C-Apm3 subunits increases from ∼2.2 nm to ∼3.9 nm, demonstrating the variability of the “open” AP-3 complex in solution.

Next, we fitted the calculated homology models of the individual AP-3 subunits Apl5, Apl6, Apm3, and Aps3 into the maps (Fig. 2, S2*B*, and 3). After fitting of the four subunits into the maps, it became apparent that the ear domains of Apl5 (aa 639–932) and Apl6 (aa 631–809) and the large flexible linker (aa 139–211) connecting the N- and C-terminal domains of Apm3 are not resolved in the reconstructions. The latter one was also missing in the previously reported crystal structures of AP-1 and AP-2 (7, 10).

Apl5 and Apl6 both contain extended α-solenoid domains that are oriented perpendicular to each other, forming the L-shaped backbone of the AP-3 complex. Aps3 is completely surrounded by Apl5 and the resolved regions of Apm3 bind exclusively to Apl6. Intriguingly, the N-terminal domain of Apl6 was less defined than the rest of the subunits, indicating a higher degree of flexibility in this area (Fig. 2). It is tempting to speculate whether this flexibility of the N-terminal domain plays a crucial role in the activity of the protein as it appears to be wrapped around the N-terminal domain of Apm3. The same applies for the smaller N-terminal domain of Apm3 (aa 1–25, 39–138) as well as the Aps3 (aa 1–168). These domains are composed of a five-stranded ß-sheet surrounded on each side by α-helices and show a high structural similarity to each other (10).

**Figure 3:**
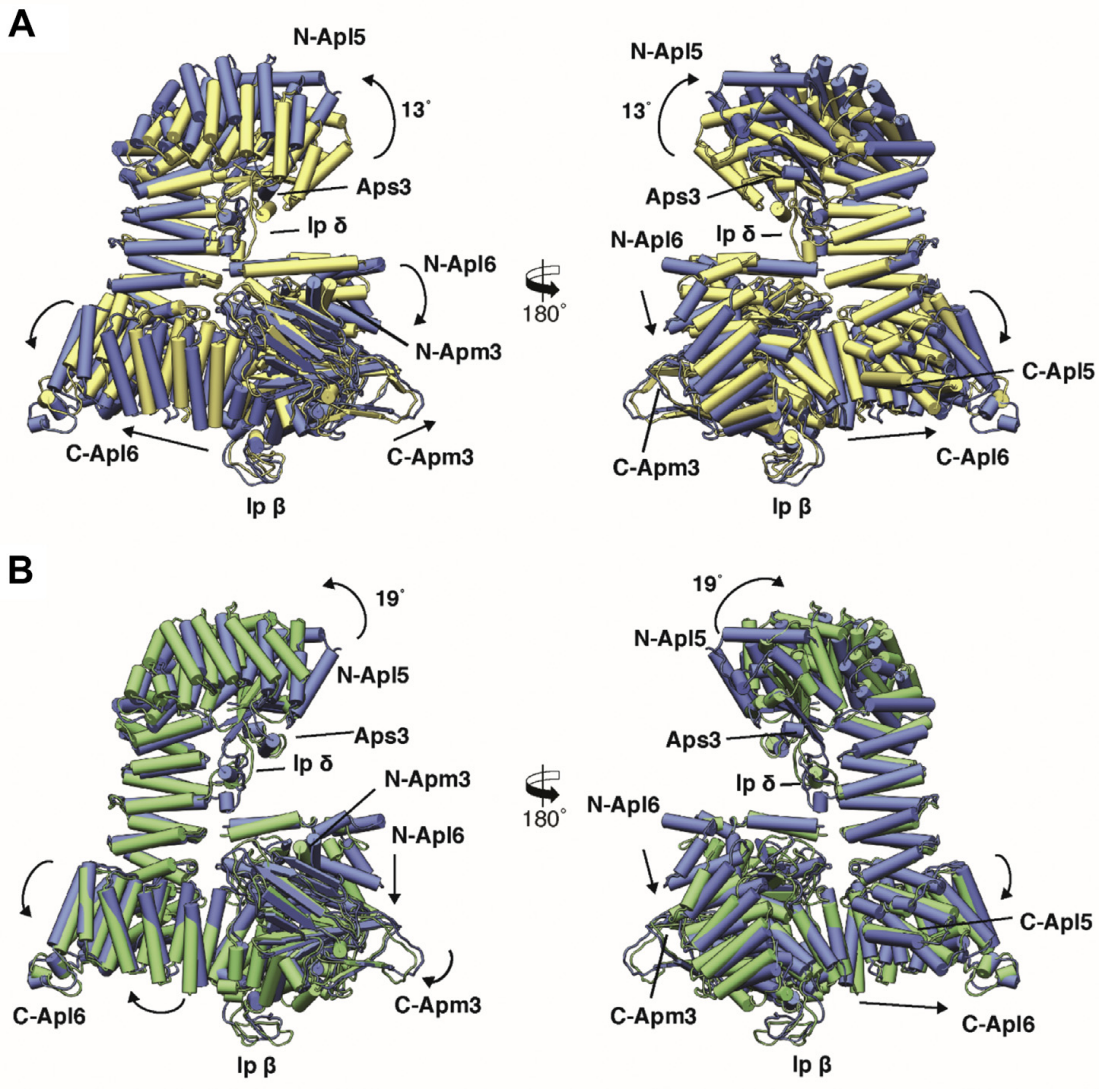
**AP-3 complex in solution shows high variability in its “open” conformation**. *A*, superposition of the homology models of the compact conformation (*gold*) with the intermediate conformation (*blue*) of the open AP-3 complex. *B*, superposition of the homology models of the intermediate conformation (*blue*) with the stretched conformation (*green*) of the open AP-3 complex. The models are shown in pipes and planks representation. *Arrows* represent the directional movement of the individual domains observed in the AP3 complex. See also Movies S1 and S2.

The high degree of intramolecular flexibility among the greatest part of the AP-3 complex becomes especially apparent when the homology models of the three classes are superimposed (Fig. 3, Movie S3). The large α-solenoid domains of Apl5 and Apl6 contain several hinge regions that allow an accordion-like expansion and partial rotation of the domains. It is especially the N-terminal regions of the two proteins that move, whereas the C-terminal domains that interact with each other are less flexible. This results in compact, intermediate, and stretched appearances of the complex, which can be seen as snapshots of a continuously moving complex. These appearances are mainly contributed to the movement of the N-terminal region of Apl5, which rotates ∼13° upward from the compact to the intermediate conformation and then tilts 19° backward generating the stretched conformation. Apm3 and Aps3 also contain hinges that allow the subunits to adapt to the changes in Apl5 and Apl6. Strikingly, we did not observe a strong movement of the C-terminal half of Apm3 in any of the generated classes, which is probably due to its “free” position in the complex as it is not involved in the conformational flexibility of the large adaptins Apl5 and Apl6 (Fig. 3). Thus, the inherent flexibility of Apl5 and Apl6 rotates and repositions Aps3. It also introduces a deformation of the N-terminal domain of Apm3. Such movements may support the transition between a more closed and open state of the complex.

**Figure 4:**
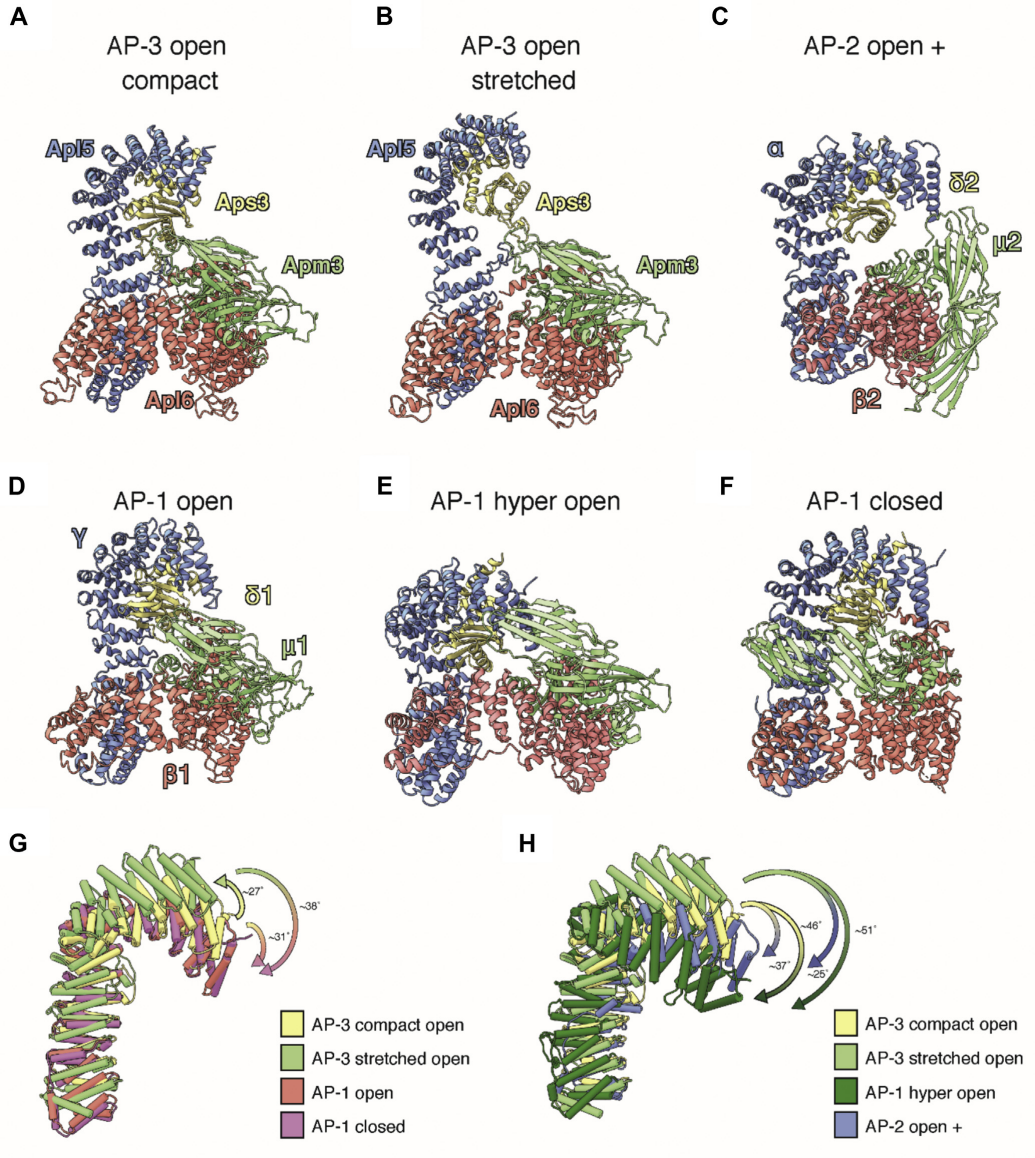
**Structure comparison of the AP-3 complex with the AP-1 and AP-2 complexes**. *A* and *B*, compact (*A*) and stretched (*B*) AP-3 form of the AP-3 open conformation. *C*, crystal structures of the AP-2 complex in its open+ (pdb-ID: 6QH6). *D–F*, crystal structures of the AP-1 complex in its open (pdb-ID: 4HMY) (*D*), hyper open (pdb-ID: 4P6Z) (*E*), and closed (pdb-ID: 1W63) (*F*) conformation. The different subunits of the adaptor protein complexes are depicted in the colors as described in Figure 2 with Apl5 depicted in *blue*, Apl6 in *red*, Apm3 in *green*, and Aps3 in *gold*. *G*, superposition of the Apl5 subunit in the compact (*yellow*) and stretched (*green*) conformation with its homologous structures in the open (*red*) and closed (*purple*) conformations of AP-1. *H*, superposition of the Apl5 subunit in the compact (*yellow*) and stretched (*green*) conformation with its homologous structures in the hyper open conformation of AP-1 (*dark green*) and open+ conformation of AP-2 (*blue*). Superposition of the Apl5 subunits was superimposed on the basis of the beta (Apl6) subunits to highlight the change in rotation of the N-terminal part of Apl5.

The general organization of both the compact and stretched conformation of the yeast AP-3 complex is similar to that of mammalian AP-1 and AP-2. We superimposed both structures of the compact and stretched AP-3 complex with the published structures of AP-1 and AP-2 in their “open,” “hyper open,” “open+,” and “closed” states (7, 8, 10, 34, 35, 36, 37) (Fig. 4). A direct comparison with the open and closed conformation of AP-1 revealed that both conformations of AP-3 represent a more open state of adaptor proteins than previously observed for membrane-bound AP-1 or AP-2, in which the N-terminal region of Apl5 is rotated ∼31° (compact) and ∼38° (stretched) upward and tilted away from the core of the complex (Fig. 4*G*). This open conformation is in direct contrast with the hyper open state of AP-1 and the open+ state of AP-1, in which the N-terminal region of γ and α is rotated toward the core of the molecule (Fig. 4*H*). We thus conclude that AP-3 in solution does not exist in a defined closed state, but rather shows an equilibrium of various “open” states owing to its high flexibility.

**Figure 5:**
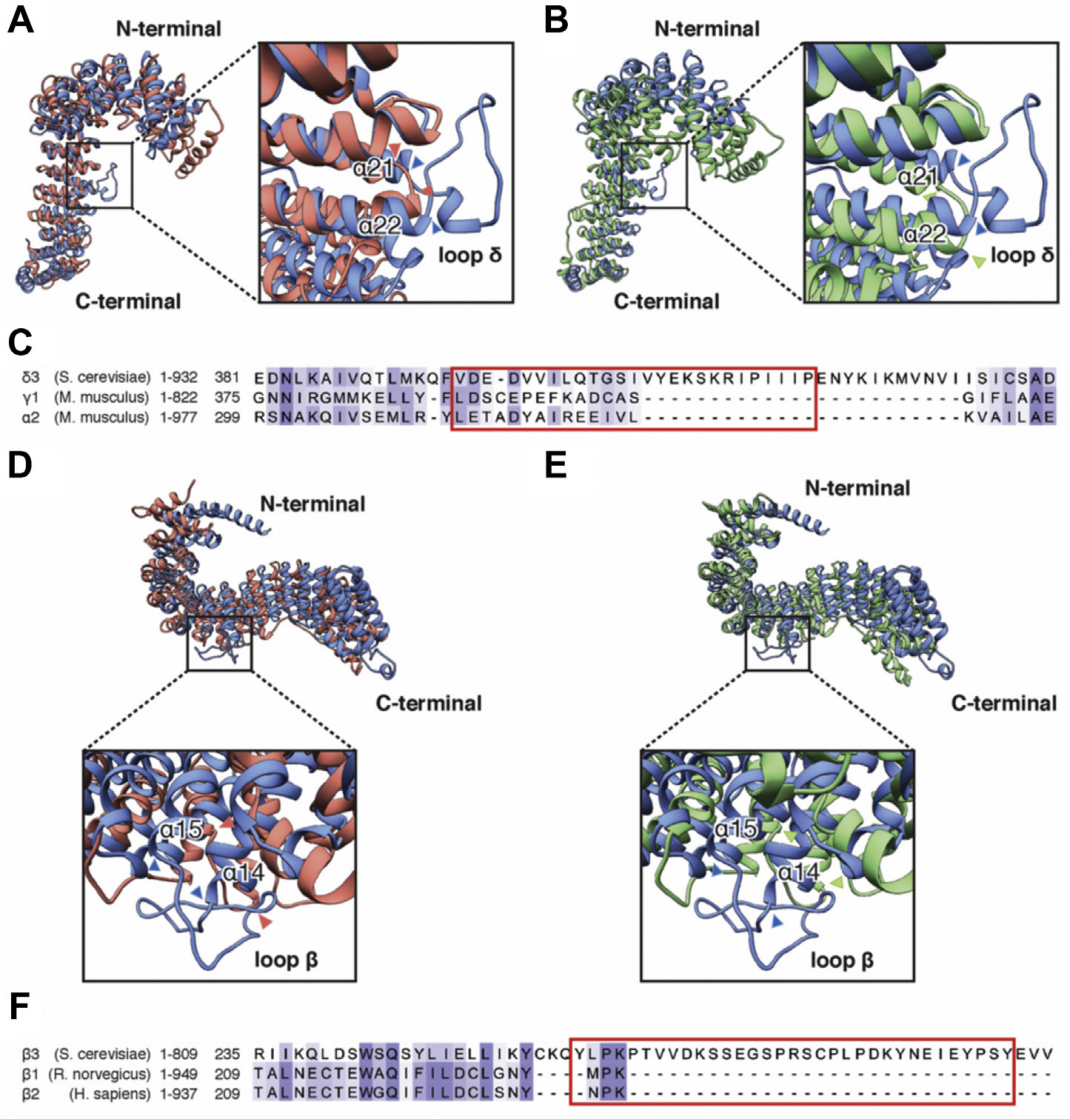
**Predicted large loops in Apl5 and Apl6 in the AP-3 complex might result in increased flexibility compared to other adaptor protein complexes**. *A* and *B*, superposition of the Apl5 subunit in the AP3 complex (*blue*) with the alpha subunits of the AP-1 (*A*, pdb-ID: 4HMY, *red*) and AP-2 (*B*, pdb-ID: 2XA7, *green*) complexes. Insets show a close up of one identified flexible region of the Apl5 subunit. This region features a large loop (lp δ,aa: 399–421) connecting helices α21 (aa: 381–398) and α22 (aa:422–438), which correspond to helices α20 (aa: 375–391) and α21 (aa: 393–411) of the AP-1 complex (A) and helices α20 (aa: 399–415) and α21 (aa: 419–436) of the AP-2 complex (*B*). The sequence corresponding to the predicted loop is highlighted in *red*. (C) Section of the sequence alignment of the Apl5 subunit (*S. cerevisiae*) with the homologous subunits of AP-1 and AP-2 (*M. musculus*). The sequence corresponding to the predicted loop is highlighted in *red*. Alignments were generated with T-Coffee and prepared for presentation using Jalview 2. Sequences were scored for conservation using the BLOSUM62 Matrix as implemented in Jalview 2 (77, 78). *D* and *E*, superposition of the Apl6 subunit in the AP3 complex (*blue*) with the beta subunits of the AP-1 (*A*, *red*) and AP-2 (*B*, *green*) complexes. Insets show a close up of one identified flexible region of the Apl5 subunit. This region features a large loop (lp β,aa: 259–291) connecting helices α14 (aa: 243–258) and α15 (aa: 290–312), which correspond to helices α12 (aa: 215–226) and α13 (aa: 234–245) of the AP-1 complex (*A*) and helices α12 (aa: 216–227) and α13 (aa: 235–245) of the AP-2 complex (*B*). *F*, section of the sequence alignment of the Apl6 subunit (*S. cerevisiae*) with the homologous subunits of AP-1 and AP-2 (*M. musculus*). The sequence corresponding to the predicted loop is highlighted in *red*. Alignments were generated with T-Coffee and prepared for presentation using Jalview 2. Sequences were scored for conservation using the BLOSUM62 Matrix as implemented in Jalview 2 (77, 78).

To define unique features among the AP-3 adaptins Apl5 and Apl6, we compared their structure with those of the equivalent subunits in AP-1 (γ and β1) and AP-2 (α and β2). A superposition of the δ-subunit Apl5 over its homologues revealed a high similarity with minor shifts and differences in the overall assembly (Fig. 5, *A* and *B*). Notably, the trRosetta homology model of Apl5 contained an extended loop (lp δ) in an α-helical hairpin between helices α21 and α22 (aa 399–421) that is not present in the respective subunits of AP-1 and AP-2. A subsequent sequence alignment revealed that this loop is unique to Apl5 (Fig. 5*C*) in this setup. Interestingly, this α-helical hairpin appears to be at the junction, where the movement of the N-terminal part of Apl5 originates from (Movie S3). We found a comparable situation for the β3-subunit Apl6, the second large adaptin of AP-3. The arrangement of the Apl6 α-solenoid did not overlay as well with β1 and β2, as Apl5 did with γ and α (Fig. 5, *D* and *E*). Interestingly, we also found an elongated loop (lp β) in between helices α14 and α15 of Apl6 that occupies an extra density at the bottom of the solenoid (Fig. S4) and does not exist in the mammalian AP-1 or AP-2 structure, yet is conserved in the β3 subunit of mammalian AP-3 (Fig. 5, *D* and *E* and Movie S1). This loop of 32 residues (aa 259–291) extends even further than the one found in Apl5, and sequence alignment revealed as well that this stretch is not found in the homologues of AP-1 and AP-2 (Fig. 5*F*). Although the density corresponding to this loop is not well-defined, we observed a distinct motion in this region, corresponding to the extension and retraction of the N-terminal domain of Apl6 (Movies S2 and S3).

### Positioning of the ear domains in the full-length AP-3 complex

Our analysis did not reveal the position of the ear domains of Apl5 (aa: 639–932) and Apl6 (aa: 631–809), as we could not detect extra density for these domains (Figs. 2 and S1). *De novo* structural prediction of Apl5 and Apl6 suggested multiple possible locations of the ear domains relative to the conserved α-solenoid part (Fig. S3, *A* and *B*). To identify possible locations of the ear domains in the context of the full-length complex, we used a combinatorial approach of cross-linking and mass spectrometry. Purified AP-3 complex was incubated with the cross-linker disuccinimidyl sulfoxide (DSSO), and cross-linked peptides were identified by mass spectrometry (48) (Fig. S5). To simplify the obtained results, we analyzed only the most confident cross-links of the ear domains (Fig. S5, *A* and *C*). We found that the ear domains of Apl5 and Apl6 cross-link with all subunits of AP-3, yet the ear domain of Apl5 predominantly interacts with one side of AP-3, whereas the ear domain of Apl6 has contacts mostly with the other side (Fig. S5, *B* and *D*). If one follows the Apl5 cross-links on AP-3 starting at the origin of the ear domain, one can track a path of interaction on AP-3. This is less obvious for the ear domain of Apl6. Both ear domains are cross-linked to residues close to their origin in the Apl5 and Apl6 subunits, but also to remote residues of Aps3 and Apm3, which are up to 10 nm away from the origin of the domains. In addition, we found several cross-links between the ear domains. Taken together, this indicates that although the ear domains are very flexible, they are in close proximity to the AP-3 complex, a possible prerequisite of the interaction with the HOPS complex at the vacuole during tethering (18, 19, 21).

### Interaction with cargo and Arf1-GTP stabilizes AP-3 on membranes

**Figure 6:**
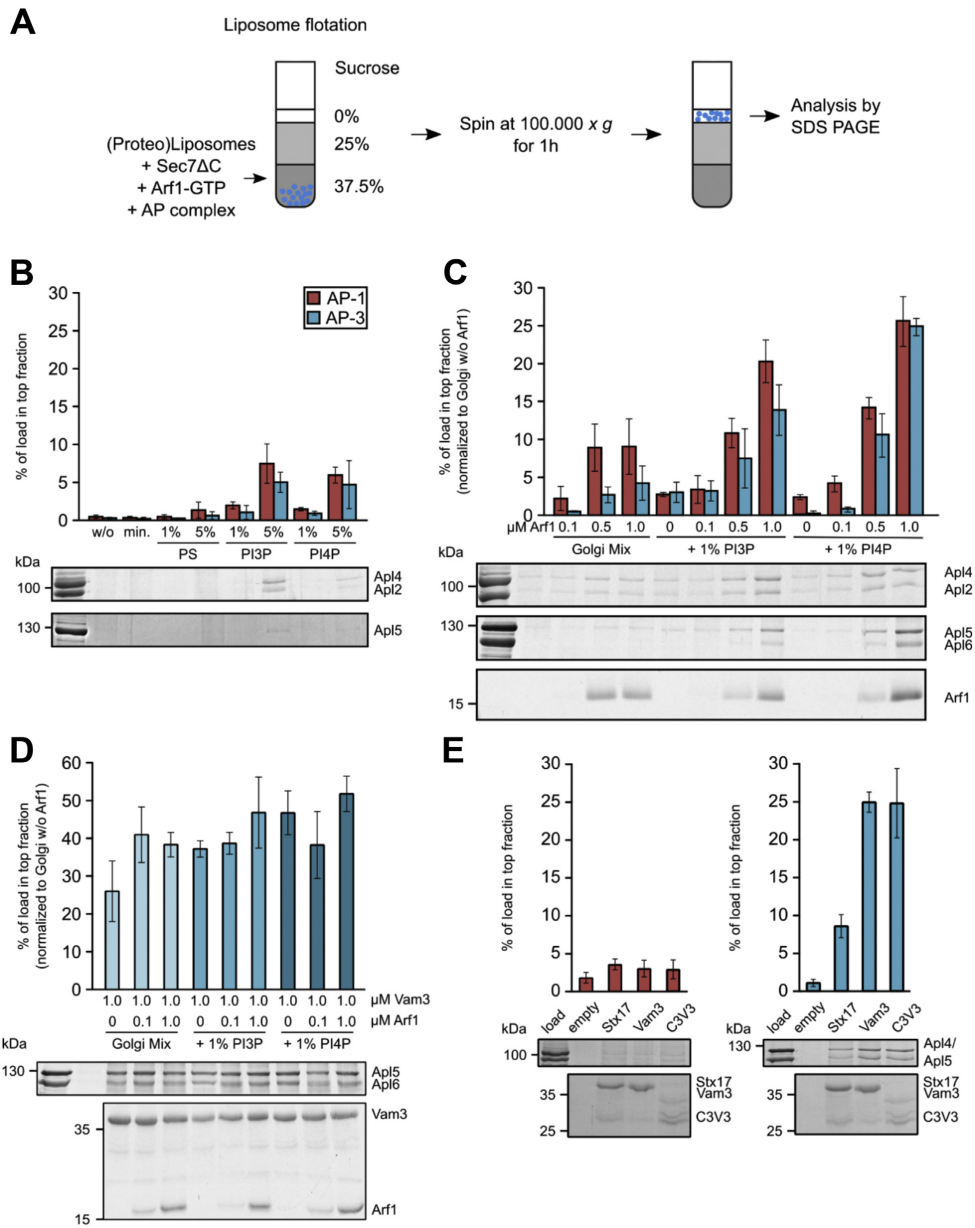
**Cargo, Arf1, and PI4P are required for recruitment of AP-3 to membranes**. *A*, scheme for AP complex flotation assay. Indicated components were incubated with proteoliposomes in the presence of GTP and added to the bottom fraction of a sucrose gradient. Samples were centrifuged and the top fraction was removed for further analyses (see Experimental procedures for details). *B*, lipids alone have a minor influence on AP-1 and AP-3 recruitment. 0.5 mM of empty liposomes prepared from a minimal lipid mix (73 % DOPC, 18 % DOPE, 8 % Ergosterol, 1 % DAG) with indicated amounts of acidic lipids were incubated with purified AP-1 and AP-3 complex and floated on a sucrose step gradient. The top fraction was removed, proteins were precipitated by TCA, boiled in SDS sample buffer, and analyzed by SDS PAGE. The first lane indicates a sample without liposomes. Band intensity corresponding to Apl4 or Apl5 was determined using FIJI (79). Graphs show the mean percentage of the load in the top fractions. Error bars indicate standard deviation (n = 3). *C*, Arf1 and PIPs result in a coincidence detection of AP complexes. 0.5 mM of empty liposomes prepared from a Golgi-mimicking lipid mix (see Experimental procedures) and indicated amounts of PIPs were incubated with indicated amounts of Arf1 and purified AP-1 and AP-3 complex and floated on a sucrose step gradient. The top fraction was removed, proteins were precipitated by TCA, boiled in SDS sample buffer, and analyzed by SDS PAGE. Band intensity corresponding to Apl4 or Apl5 was determined using FIJI (79). Graphs show the mean percentage of the load in the top fractions, normalized to a sample using Golgi-Mix liposomes without Arf1 (first band on the gel). Error bars indicate standard deviation (n = 3). *D*, AP-3 is recruited by Vam3. Empty liposomes or proteoliposomes carrying a total of 1 μM Vam3 were incubated with or without indicated concentrations of Arf1 and purified AP-3 complex and floated on a sucrose step gradient. The top fraction was removed, proteins were precipitated by TCA, boiled in SDS sample buffer, and analyzed by SDS PAGE. Band intensity corresponding to Apl5 was determined using FIJI (79). Graphs show the mean percentage of the load in the top fractions, normalized to a sample using Golgi-Mix liposomes without Arf1 or Vam3 (first band on the gel). Error bars indicate standard deviation (n = 3). *E*, AP-3 recruitment by cargo is specific. Empty liposomes or proteoliposomes carrying a total of 1 μM Stx17, Vam3 or GST-Chs3(aa1-38)-Vam3(TMD) (C3V3) were incubated with purified AP-1 or AP-3 complex and floated on a sucrose step gradient. The top fraction was removed, proteins were precipitated by TCA, boiled in SDS sample buffer, and analyzed by SDS PAGE. Band intensity corresponding to Apl4 or Apl5 was determined using FIJI (79). Graphs show the mean percentage of the load in the top fractions. Error bars indicate standard deviation (n = 3).

To determine the requirements of AP-3 interaction with its postulated ligands on membranes in a reconstituted system, we set up a liposome floatation assay using specific lipid mixtures, purified N-myristoylated Arf1, and cargo proteins such as the SNARE Vam3 (Fig. 6*A*). For this, liposomes were incubated with or without different components and purified AP-3 complex. The flotation was then carried out in a 37.5–0% sucrose step gradient, and proteins in the top fraction were analyzed. As the factors required for membrane association of AP-1 are well known—at least in metazoans—we used this complex as an internal control for our assays.

To assess the influence of acidic lipids and PI3P or PI4P on recruitment, liposomes were generated from a minimal lipid mix. Without any relevant charge on the lipid surface, neither AP-3 nor AP-1 was recovered in the top fraction. While the addition of 1 or 5 % PS did not result in a major increase, liposomes containing either 5 % PI3P or PI4P were able to recruit some AP-1 and AP-3 complexes (Fig. 6*B*), indicating a specific effect of PIPs.

To decipher the recruitment of AP-3 in more detail, we next focused on Arf1, a protein that has been reported to be required for AP-3 functionality (27). First we analyzed the Arf1-dependent membrane association of AP-1, as a control. We thus generated liposomes from a Golgi mimicking lipid mix with our without PI3P or PI4P and performed the flotation assay in the presence of increasing concentrations of Arf1. Whereas liposomes without any PIPs and Arf1 did not recruit any significant amount of AP-1, the addition of PI3P or PI4P slightly promoted its binding (Fig. 6*C*) as already observed with the minimal lipid mix. In the presence of Arf1, which we recruited to membranes in the presence of GTP by a hyperactive version of its guanine nucleotide exchange factor (GEF) Sec7 (49), about 10% of the complex was bound. However, in the presence of both Arf1-GTP and PI3P or PI4P, we observed a 2- to 3-fold increase in membrane-bound AP-1 with 1 μM of Arf1 that was even more pronounced on PI4P-containing liposomes (Fig. 6*C*), in agreement with earlier findings on AP-1 recruitment (8, 50).

Next, we analyzed the role of Arf1 for AP-3 recruitment to membranes. Neither AP-1 nor AP-3 complex showed significant binding to liposomes containing a basic Golgi-mimicking lipid mix (see Experimental procedures) lacking PI3P or PI4P. Addition of 1% PIPs did not significantly increase the amount of recruited AP-3 complex (Fig. 6*C*), as expected (Fig. 6*B*). In the absence of PIPs, the presence of Arf1-GTP only caused a slight increase in membrane binding of AP-3, unlike our observation of AP-1. Only when both Arf1 and PI3P or PI4P were at the membrane surface an increase in AP-3 recruitment was observed (Fig. 6*C*). In all analyses, AP-1 binding was more efficient except at the highest tested concentration of PI4P and Arf1.

We reasoned that cargo binding is the most critical factor in AP-3 recruitment and therefore analyzed its effect in the presence of increasing Arf1 concentrations. For this, we generated proteoliposomes carrying Vam3 without or with PI3P or PI4P. The presence of Vam3 alone already led to a drastic increase to more than 25% recruitment of AP-3, which was even further promoted upon addition of low amounts of Arf1 and PIPs, respectively, to around 50% (Fig. 6*D*). Also in this case, PI4P has a slightly stronger effect than PI3P. To determine that the interaction of AP-3 and Vam3 is a specific cargo–adaptor interaction, we generated proteoliposomes carrying Vam3, Stx17, a mammalian SNARE protein that is unrelated to AP trafficking (51, 52), or a synthetic cargo construct consisting of a GST-tag, the N-terminus of Chs3, which can bind AP-1 or AP-3 (53), followed by the Vam3 transmembrane domain. To determine the role of cargo, PIPs or Arf1 was excluded in these assays. Strikingly, proteoliposomes carrying the dileucine sorting motif-containing Vam3 and Chs3 clearly recruited some 25% AP-3. The nonspecific cargo Stx17 just recruited some 10%. However, AP-1 did not bind any of the proteoliposomes (Fig. 6*E*), indicating that the “closed” cytosolic AP-1 complex requires the activation by Arf1 (7). We thus conclude that AP-3 relies on a coincidence detection of cargo, Arf1-GTP and PI4P to bind to membranes, whereas AP-1 depends primarily on Arf1 and PI4P before binding to cargo (Fig. 6, *C* and *D*).

As cargo appears to be a major factor in AP-3 recruitment, we decided to investigate whether the cargo composition had any influence on the efficiency of AP-3 recruitment. We focused on the two SNAREs Vam3 and Nyv1 (a cargo with a Yxxφ sorting motif), which were both able to recruit AP-3 onto proteoliposomes (Fig. S7*A*). To compare the relative contribution of each cargo, we generated proteoliposomes with different molar compositions of Nyv1 and Vam3 (carrying a dileucine motif). However, we observed that it was not the composition, but rather the total amount of cargo on liposomes that determined AP-3 recruitment (Fig. S7*B*). We thus conclude that AP-3 is recruited to membranes primarily by cargo and that Arf1 further enhances this process.

### Recruitment and clustering of AP-3 on cargo-loaded membranes

**Figure 7:**
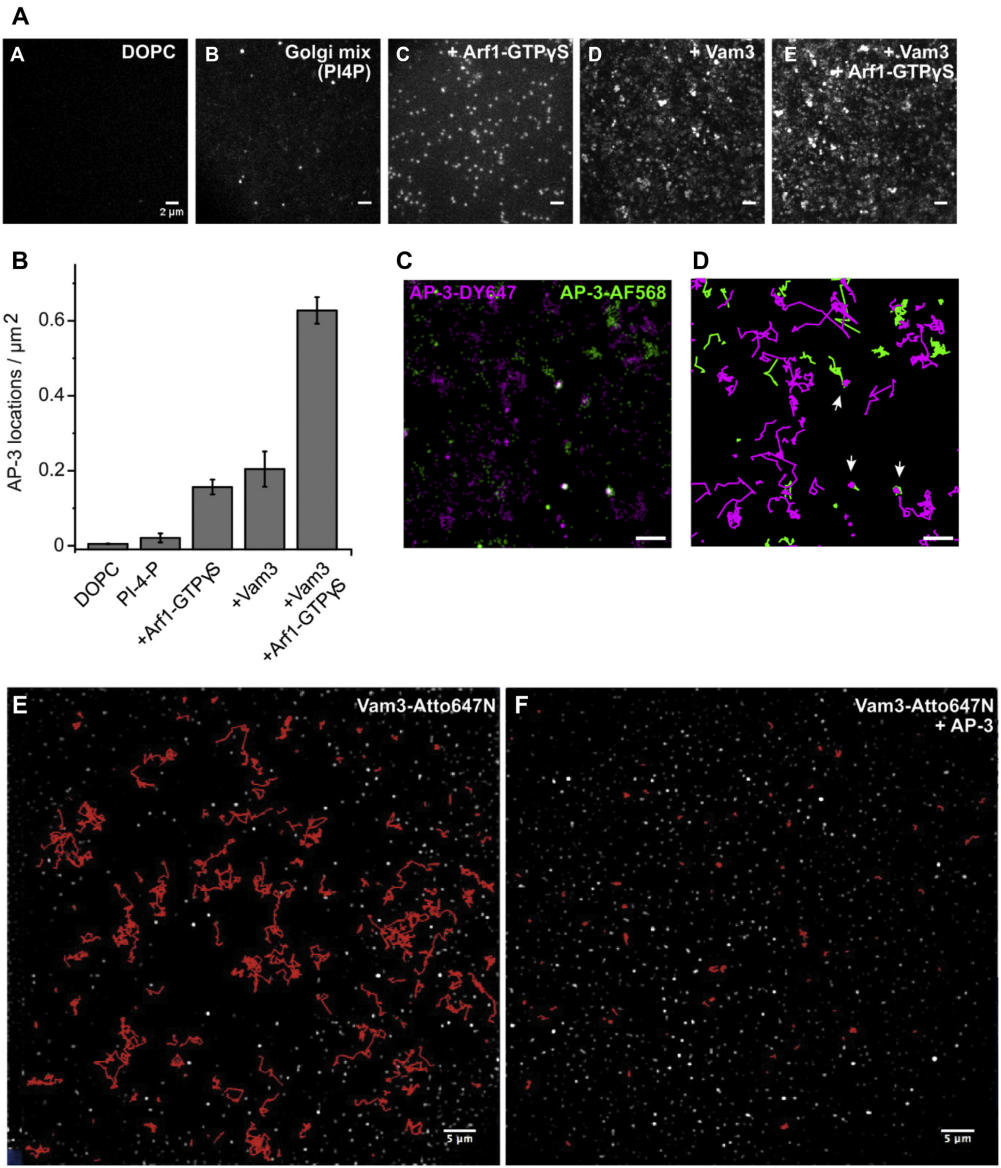
**AP-3 interaction with cargo drives self-assembly and traps cargo**. Polymer-supported membranes were reconstituted from liposomes and monitored using fluorophores on AP-3 and Vam3. *A*, recruitment of AP-3 in the presence of PI4P, GTP, Arf1, and Vam3. Polymer supported lipid bilayers containing the indicated lipid composition were incubated with or without Arf1 (10 nM), Sec7ΔC (10 nM), and 400 μM GTPgS for 10 min at room temperature prior to the addition of GFP-tagged AP-3 (2 nM) and then monitored by total internal reflection fluorescence microscopy for 45 min. Maximum intensity projection of 30 frames from representative movies of 2 nM AP-3 labeled with a αGFP Nanobody (tagged with Dy647) is shown: (*a*) binding to DOPC membranes, (*b*) a Golgi mixture including PI4P (see Experimental procedures), (*c*) Arf1, Sec7ΔC, and GTPγS, (*d*) reconstituted Vam3, and (*e*) coreconstituted Vam3 and activated Arf1. Scale bar = 2 μm. *B*, single-molecule localizations per area for conditions in (*A*). Median values of the localizations within the first ten frames of the experiment were averaged over a minimum of three experiments. Error bars indicate standard deviation. *C*, AP-3 self-assembles into immobile clusters. AP-3 was monitored on supported lipid bilayer membranes carrying Vam3. Dual-color localizations of 100 consecutive frames of GFP-AP-3 equimolar labeled with either αGFPnb-AlexaFluor568 (*magenta*) or αGFPnbDy647 (*green*). The colocalized fraction (colocalization divided by the number of localizations of the limiting fluorescence channel) of AP-3 was 5.3 ± 2 %. *D*, trajectories of the localizations highlighted in (*C*) showing restricted mobility of colocalized AP-3 (*bottom*). Scale bar, 2 μm. *E*, mobility of Vam3 is monitored before and (*F*) after the addition of AP-3. Trajectories from 30 frames of the respective movies (Movies S6 and S7) are shown in *red*.

We next inquired whether AP-3 recruitment to membranes might be sufficient to sequester cargo into clusters prior to vesicle formation. To test this, we used a polymer-supported lipid membrane (PSM). These bilayers were generated out of liposomes or proteoliposomes that were captured onto a polyethyleneglycol (PEG) polymer brush functionalized with palmitic acid moieties (54). After fusion of captured liposomes into a contiguous PSM, recruitment of AP-3 and interaction with cargo within the membrane were monitored by total internal reflection fluorescence microscopy (TIRFM). To trace AP-3 at single molecule level, we generated a strain with Apl6 fused to GFP, which was labeled with a photostable fluorescence dye *via* an anti-GFP nanobody. The labeled complex was then added to PSM of a different composition. While no signal was observed on simple bilayers made of di-oleoyl-phosphatidyl-choline (DOPC), a change to a more Golgi-like phospholipid mixture (see Experimental procedures) including 1 mol% PI4P resulted in formation of some AP-3 positive, diffraction-limited signals on the membrane (Fig. 7*A*, *a* and *b*, Movies S4 and S5). This observation was comparable to what was observed in the flotation assay (Fig. 6*C*). In the presence of myristoylated Arf1, Sec7, and the nonhydrolyzable GTPγS or just the cargo Vam3, more AP-3 signals accumulated on the bilayer (Fig. 7*A*, *c* and *d*, Movie S6). However, the strongest recruitment was observed when both cargo and Arf1 were combined (Fig. 7, *Ae* and *B*). Only upon cargo addition did we observe multiple bright signals on membranes that could not be attributed to single AP-3 complexes, but rather to clusters of AP-3. To address the possible clustering, we labeled surface-bound AP-3 with an equimolar amount of GFP nanobodies that carried either DY647 (DY647NB) or Alexa Fluor (AF) 568 (AF568NB). Localization of individual DY647 and AF568 signals beyond the diffraction limit (median localization precision ∼20 nm) revealed AP-3 colocalization as suggested by our initial observations (Fig. 7*C*, Movie S7). To further validate this interaction, we used a cotracking algorithm to follow individual AP-3 complexes over time and observed several events where AP-3 was immobilized upon colocalization (Fig. 7*D*, right panel, white arrowheads). This suggested that AP-3 interacts with itself on membrane surfaces in the presence of cargo.

We then analyzed whether cargo was indeed trapped by AP-3 clusters. Here, we took advantage of the fact that cargo itself is sufficient to recruit AP-3 (Figs. 6*D* and 7*Ac*) and therefore left out Arf1 and Sec7 in these experiments. We followed multiple Vam3 tracks before (Fig. 7*E*, Movie S8) and after (Fig. 7*F*, Movie S9) the addition of AP-3. Strikingly, AP-3 addition strongly diminished the mobility of Vam3 under these conditions. This indicates that AP-3 does indeed trap its cargo when recruited to the membrane surface.

In summary, our data reveal that full-length AP-3 complex takes advantage of its open conformation to capture and immobilize cargo on membranes without the need to bind further factors, explaining how the complex can be recruited to the Golgi even without the presence of Arf1.

## Discussion

In this study, we established the purification of the full-length AP-3 complex from yeast and examined its structure and function in detail. We showed that the complex AP-3 forms a stable heterotetramer as revealed by gel filtration and single particle cryo-EM.

Using proteoliposomes of different composition, we revealed that AP-3 strongly depends on cargo for its membrane recruitment, with contributions by Arf1-GTP and PI4P, whereas AP-1 relies on Arf1-GTP and PI4P (Fig. 6). This was also observed on supported lipid bilayers, where AP-3 complexes captured the Vam3 cargo, restricted its movement, and formed oligomers, which may suggest a nucleus in vesicle formation (Fig. 7). In agreement with this interpretation, time-course analyses in yeast revealed that AP-3 arrives at the TGN before the Arf1-GEF Sec7 (33). We conclude from these findings that the recruitment of AP-3 to membranes is initiated by cargo binding, possibly aided by PI4P interactions on membranes, which is then followed by binding to Arf1-GTP and subsequent vesicle formation.

This interpretation is strongly supported by our cryo-EM analysis of the full-length AP-3 complex. The overall structure of the entire AP-3 complex revealed several conformational states, which correspond to the previously described “open” conformation of the AP-1 and AP-2 complexes (Fig. 4). As this “open” AP-3 complex is neither stabilized by cargo nor Arf1, it is not surprising that we observe a high flexibility in almost all domains. Such flexibility results in highly extended “open” conformations, allowing to have easier access to the cargo-binding sites. Therefore, AP-3 may be primed to bind cargo even in the absence of Arf1-GTP.

Crystal and cryo-EM structures of truncated AP-1 and AP-2 complexes revealed that they both exist in two or more distinct conformational states in a switch-like behavior. The “closed” cytosolic complexes undergo a large conformational change upon binding to Arf1 (for AP-1) or PI(4,5)P_2_ (for AP-2) at a membrane surface (7, 8, 10, 34). This “open” structure involves a movement of the C-terminal part of μ1 or μ2 from the center of the complex (activation), which reveals the cargo-binding sites for tyrosine-based Yxxφ or acidic dileucine [D/E]XXL[L/I] sorting motifs (8, 34). Contrary conformational changes were proposed for metazoan AP-3 based on extensive yeast-two-hybrid interaction studies, which suggested that the binding of Arf1 to AP-3 is inhibited by an interaction of the δ-ear domain with the σ3 subunit, whereas cargo binding was not affected by this ear–core interaction, further supporting an initial recruitment of AP-3 to membranes by cargo rather than Arf1 (43).

Our structural and functional analyses suggest that the full-length AP-3 complex does not depend on Arf1-GTP to bind cargo. It rather seems as if its inherent “open” conformation and the resulting higher flexibility render these sites immediately available to capture cargo at the TGN, in agreement with *in vivo* analyses (33). Such flexibilities may also be partially present in AP-1 and AP-2. These may have been occluded due to the natural restrictions in protein crystallization, which require truncations of the ear domains of the two large adaptins in each complex. Likewise, crystal contacts and additional binding partners such as Arf1, phosphoinositol, or peptides carrying sorting motifs may have stabilized a certain conformation that is favored over others in these setups. As we directly vitrified freshly purified full-length AP-3, we uncovered the more dynamic and flexible behavior of the complex. We can, of course, not exclude the possibility that the yeast AP-3 complex is more flexible, whereas those from higher eukaryotes have evolved more defined steps and conformational states. Comparative studies of full-length AP-complexes from different organisms, both in solution and on membranes, will be necessary to clarify this question in the future.

How can we explain the flexibility of the AP-3 complex? We identified two extended loops within an α-helical hairpin in both large adaptins, Apl5 and Apl6, which are located close to the middle of the polypeptide chain at the trunk part (Fig. 5). The loop in Apl6 is conserved in the human β3-subunit, whereas it is lost in δ, but they are only present in the AP-3 subunits and are not found in those of metazoan or yeast AP-1 and AP-2. These elongated loops might enhance overall flexibility of the large adaptins by providing less restriction than for tightly connected α-helices. Interestingly, the N-terminal parts of Apl5 and Apl6 appear to be more flexible than the C-terminal trunk part. These extended loops may act as a hinge that allows for increased movement of N-Apl5 and N-Apl6 and consequently the modulation of the N-terminal Aps3 and Apm3 domain. As we and others did not find evidence for a clathrin lattice and could exclude the HOPS Vps41 subunit as a putative coat (18, 55), we speculate that such a movement may facilitate vesicle formation even in the absence of an outer coat. Reconstitution of AP-3 vesicle formation will be necessary to clarify this issue.

Several studies uncovered key roles of the C-terminal ear domains of the two large adaptins in AP-1 and AP-2, which are connected *via* putative flexible linkers to the trunk part (40, 42, 56, 57, 58). These domains are also critical for AP-3 function in yeast (18). So far, the localization of the ear domain relative to the trunk part is only speculative. Yet, our analysis of the full-length complex makes it unlikely that the ear domains extend away from the core. Indeed, when β2-hinge domain was included in crystallization of the core part of the AP-2 complex, it was found in close contact with the core (35). As we obtain multiple cross-links of the ear domains of the δ- and β3-subunits with all other AP-3 subunits (Fig. S6), we suspect that they associate dynamically with the core part of the complex. This is line with our structural analysis, where we could neither identify the ear domains in our 2D class averages nor in our 3D maps. Such flexible association of the ear domain of Apl5 with the core of the AP-3 complex is likely needed to allow its interaction with the HOPS subunit Vps41 during tethering at the vacuole (18, 19, 21). For VAMP7, the mammalian homologue of Nyv1, a direct interaction of its Longin domain with the hinge region of the δ-subunit, has been shown (56, 59). An interaction like this in the yeast AP-3 complex could potentially explain how cargo binding results in relocalization of the hinge and ear regions. As a result, this relocalization could expose the Arf1-binding site that was previously blocked by the δ-ear domain and thus result in stabilization of membrane interaction (43).

AP-3 is one of many sorting factors at the TGN, where cargo is sorted to either the plasma membrane, early or late endosome/vacuole (60, 61, 62). Given that some cargoes have similar sorting motifs and are recognized by both AP-1, Gga1 and 2, and AP-3, the question thus arises: how is specificity achieved at last? Our structural and functional analysis of the AP-3 complex suggests that AP-3's open conformation and preference for cargo even in the absence of Arf1 may explain how it can compete with other cargo adaptors. We suggest that the TGN provides a platform, where the coincidence detection code of each adaptor or sorting complex, consisting of cargo recognition, Arf1-GTP, and lipid preference (such as PI4P or sphingolipids and cholesterol) determines the specific sorting into vesicles. Further analyses of full-length AP complexes in solution and at membranes combined with *in vivo* tracing of complexes at membranes will be necessary to understand how cargo sorting and vesicle formation are coordinated.

## Experimental procedures

### Strains and plasmids

The strains for AP-1 and AP-3 overproduction were generated by inserting the *GAL1* promoter in front of each of the four subunits. Overproduction was confirmed by tagging of each respective subunit with a 3xFLAG-tag, followed by Western blotting. The final strain only harbored the 3xFLAG tag on Apl5 or Apl4, respectively. Where indicated, Apl6 was tagged C-terminally with mGFP to mark the AP-3 complex for PSM experiments. Tagging of the indicated strains with epitope tags was done as published (63, 64). See Table S2. Plasmids for overproduction of Vam3 and Nyv1 have been published. Syntaxin 17 was cloned into a pET28a-His-Sumo vector for N-terminal tagging, and the hybrid construct of Chs3 (aa 1–38) and the Vam3 transmembrane domain (aa 263–283) was cloned into a pGEX4T1 vector, adding an N-terminal GST-tag.

### Purification of the full-length AP-1 and AP-3 complex

Two liters of yeast peptone (YP) medium containing 2 % galactose (v/v) was inoculated with 6 ml of an overnight culture. The cells were grown for 24 h and harvested by centrifugation in a JLA 8.1000 rotor (4800*g*, 6 min, 4 °C). Pellets were washed with 100 ml of cold H_2_O and centrifuged again. The pellet was resuspended in 25 ml of AP-3 purification buffer (APB, 150 mM NaCl, 20 mM HEPES/NaOH, pH 7.4, 1.5 mM MgCl_2_) supplemented with 0.5 mM Phenylmethylsulfonylfluoride (PMSF), 1× FY protease inhibitor mix (Serva), and 0.5 mM Dithiothreitol (AppliChem GmbH), and lysed by glass bead lysis. Glass beads were removed by filtration, and the lysate was centrifuged for 15 min at 3000*g* and 4 °C, and then 100,000*g* for 70 min at 4 °C to generate a cleared lysate. After centrifugation, the supernatant was added to 500 μl of anti-FLAG M2 affinity gel (Sigma-Aldrich) for 60 min at 4 °C on a nutator. The beads were briefly centrifuged (900*g*, 1 min, 4 °C), and the supernatant was removed. Beads were transferred to a 1 ml MoBiCol column (MoBiTec) and washed with 20 ml of APB. Then 25 μl of 5 μg/μl FLAG-peptide was added to the beads and incubated on a turning wheel for 20 min. The eluate was collected by centrifugation (900*g*, 2 min, 4 °C). After addition of Glycerol to a final concentration of 5% (v/v), AP-3 was flash frozen and stored at −80 °C. For Cryo-EM AP-3 was prepared without Glycerol. After purification, the sample was loaded onto a Superose 6 Increase column (GE Healthcare). Peak fractions were pooled and concentrated in a Vivaspin 100 kDa MWCO concentrator (Sartorius) to the desired concentration. For cryo electron microscopy, samples were freshly prepared and imaged at the same day.

### Cross-linking of purified AP-3 complex

Purified AP-3 complex was centrifuged (21,000*g*, 10 min, 4 °C) to remove any aggregates. Freshly prepared DSSO was added to 200 μg of purified AP-3 complex to a final concentration of 0.5 mM and incubated at room temperature for 20 min. This step was repeated and afterward 1 M Tris-HCl pH 8.0 was added to a final concentration of 20 mM and incubated for 30 min at room temperature to quench the reaction. Samples were flash frozen and stored at −80 °C until further preparation. Cross-linked AP-3 complex was denatured in 8 M urea, reduced with 5 mM DTT at 56 °C for 30 min, alkylated with 40 mM chloroacetamide at room temperature in the dark for 20 min, and digested with trypsin at 1:100 protein to trypsin ratio (w/w) at 37 °C overnight. On the next day, the peptides were desalted using C8 Sep-Pak desalting columns. Strong cation exchange (SCX) fractionation was performed on an Agilent 1260 Infinity II UPLC system equipped with a PolySULFOETHYL-A column (100 × 2.1 mm, 3 μm particles, PolyLC Inc). Eight late SCX fractions were collected, desalted, and subjected to LC/MS analysis. LC/MS was performed using Thermo Scientific Dionex UltiMate 3000 system connected an Orbitrap Fusion Lumos mass spectrometer. A mass-difference triggered MS2-MS3 acquisition strategy was applied for cross-link identification (48). Data analysis was performed using XlinkX standalone (peaklist-generating software: Proteome Discoverer Daemon 2.3.352) with the following parameters: minimum peptide length = 6; maximal peptide length = 35; missed cleavages = 3; fix modification: Cys carbamidomethyl = 57.021 Da; variable modification: Met oxidation = 15.995 Da; DSSO cross-linker=158.0038 Da (short arm = 54.0106 Da, long arm = 85.9824 Da); precursor mass tolerance = 10 ppm; fragment mass tolerance = 20 ppm. MS2 spectra were searched against a reduced database (HS1028_AP-3_Komplex_Osnbrück.fasta, containing four sequences, 2450 residues [Data Sheet S1]). Results were reported at 1% FDR at CSM level based on target decoy. Cross-links were additionally filtered using a score cutoff of 1 × 10^−15^ for both cross-linked peptides. A list of all identified peptides is provided in Data Sheet S2.

### Purification of myristoylated Arf1 and Sec7ΔC

Myristoylated Arf1 was prepared according to the published protocol with the indicated alterations (65). In brief, plasmids expressing Arf1 and NMT-1 (N-myristoyl transferase) were cotransformed into BL21 DE3 Rosetta. Coexpression was induced with 1 mM IPTG for 5 h at 37 °C in the presence of 0.5 mM myristic acid at an OD_600_ = 0.5. Cells were harvested, washed once with STE buffer (50 mM Tris/HCl, pH 8.0, 40 mM EDTA, 25 % (w/v) sucrose), flash frozen, and stored at −80 °C. On the next day, the pellet was thawed and resuspended in 20 ml of STE buffer supplemented with 1 mM PMSF, 0.1× protease inhibitor cocktail (PIC; protease inhibitor cocktail; 1× = 0.1 mg/ml of leupeptin, 1 mM *o*-phenanthroline, 0.5 mg/ml of pepstatin A, 0.1 mM Pefabloc), and 1 mg/ml lysozyme. After addition of 8 ml TB (50 mM Tris/HCl, pH 8.0, 0.2 % (v/v), Triton X-100, 100 mM MgCl_2_), cells were lysed by sonication. The lysate was then centrifuged at 100,000*g* for 60 min at 4 °C. The supernatant was loaded onto a 50 ml DEAE Sephacel column (GE), which was washed with 6 ml fractions of DEAE buffer (20 mM Tris/HCl, pH 7.4, 50 mM NaCl, 1 mM EDTA, 1 mM DTT). Eluted proteins were analyzed by SDS-PAGE and concentrated to 5 ml (Sartorius Vivaspin, 10,000 MWCO PES). Arf1 was further purified on a Superdex 75 prep grade column (GE Healthcare) in 20 mM HEPES/NaOH, pH 7.4, 100 mM NaCl, 1 mM EDTA, 1 mM DTT, 2 mM MgCl_2_. Protein-containing fractions were concentrated to 1 mg/ml, aliquoted, flash frozen, and stored at −80 °C.

His-tagged Sec7ΔC was purified as published (49). The activity of Sec7 on Arf1 was confirmed by activity-induced changes in tryptophan fluorescence. Arf1 myristoylation was confirmed by mass spectrometry.

### Purification of the full-length SNAREs and hybrid constructs for reconstitution

Vam3 and Nyv1 were purified as previously reported (66). The hybrid cargo consisting of Chs3 (aa 1–38) and Vam3 (aa 263–283) was purified in the same way. Syntaxin 17 was expressed in *Escherichia coli* BL21 Rosetta, induced with 1 mM isopropyl 1-thio-β-D-galactopyranoside for 16 h at 16 °C. Cells were taken up in resuspension buffer (300 mM NaCl, 20 mM HEPES/NaOH pH 7.4, 1 mM DTT) supplemented with 1 mM PMSF and 0.5 × PIC and lysed in a Microfluidizer (Model M-110L, Microfluidics, Newton, MA). Membranes were pelleted by centrifugation (125,000*g*, 50 min, 4 °C). The pellet was resuspended in lysis buffer (300 mM NaCl, 20 mM HEPES/NaOH, pH 7.4, 100 mM n-octyl-β-D-glycopyranoside (β-OG), 1 mM DTT, 1 mM PMSF, 0.5 × PIC) on a nutator over night at 4 °C. The sample was centrifuged as before, the supernatant was added to 1 ml Ni-NTA resin pre-equilibrated with resuspension buffer, and incubated on a nutator for 2 h at 4 °C. The resin was washed with 40 column volumes of washing buffer (300 mM NaCl, 20 mM HEPES/NaOH, pH 7.4, 100 mM β-OG, 1 mM DTT, 25 mM Imidazole) and eluted with elution buffer (300 mM NaCl, 20 mM HEPS/NaOH, pH 7.4, 100 mM β-OG, 1 mM DTT, 300 mM Imidazole). Protein-containing fractions were aliquoted, flash-frozen, and stored at −80 °C.

### Liposome flotation assays

Liposomes containing the SNAREs Vam3, Syntaxin 17, or Nyv1 were generated as published (67), with a Golgi-mimicking lipid composition (46 mol% dioleoyl PC, 18 mol% dioleoyl PE, 18 mol% Soy phosphatidylinositol, 4.4 mol% dioleoyl PS, 2 mol% dioleoyl PA, 1.6 mol% cardiolipin, 8 mol% ergosterol, 1 mol% diacylglycerol), including PIPs as indicated. Liposomes (0.5 μM) were incubated in polycarbonate tubes (11 × 34 mm, Beckman Coulter) for 30 min at room temperature with 100 nM Sec7ΔC+HDS1, 500 nM myristoylated Arf1, and 400 μM GTPγS in 50 μl. Then 20 μg of AP-3 and RB150 (Reconstitution buffer; 150 mM NaCl, 20 mM HEPES/NaOH, pH 7.4, 1 mM MgCl_2_, 10 % (v/v) glycerol) were added to a final volume of 150 μl. The reaction was incubated for 60 min at 4 °C, mixed with 150 μl of 80 % (w/v) Histodenz, in RB150, and then overlayed with 300 μl of 30 % (v/v) Histodenz in RB150, and 100 μl RB150. Reactions were centrifuged at 100,000*g* for 1 h at 4 °C, and 200 μl of the top fraction was collected. Proteins were then TCA-precipitated and analyzed by SDS and Western Blot. Where indicated, a sucrose gradient of 75 % (w/v), 35 % (w/v), and 0 % was used.

### Labeling of Vam3

Before labeling, the buffer of Vam3 (500 μg) was exchanged by size-exclusion chromatography (Superdex 75 10/300) to PBS +35 mM n-octyl-β-D-glycopyranoside. The protein-containing fractions were concentrated to 300 μl. For labeling of Vam3, 2-fold molar excess of Atto 647N (Maleimide) (ATTO-TEC) was added. The labeling reaction was performed for 1 h in the dark. Excess dye was removed by gel filtration with a Superdex 75 10/100 column. Vam3-containing fractions were concentrated to 0.3 mg/ml.

### Reconstitution into PSM and single-molecule imaging

PSMs were generated as described (54). Briefly, a Golgi mimicking lipid mixture (46 mol% dioleoyl PC, 18 mol% dioleoyl PE, 18 mol% Soy phosphatidylinositol, 4.4 mol% dioleoyl PS, 2 mol% dioleoyl PA, 1.6 mol% cardiolipin, 8 mol% ergosterol, 1 mol% diacylglycerol, 1 mol% PI4P) was evaporated and dissolved in 35 mM n-octyl-β-D-glycopyranoside (in H_2_O) to a final lipid concentration of 2 mM. In total, 62.5 μl of this lipid solution was incubated with prelabeled transmembrane cargo (also in 35 mM n-octyl-β-D-glycopyranoside) to obtain a protein:lipid ratio of 1:1,000,000. To generate liposomes, β-cyclodextrin was added in a 2-fold molarity (70 mM) and incubated for 5 min at room temperature. Finally, HBS buffer (20 mM HEPES/NaOH, pH 7.4, 150 mM NaCl) was added to final volume of 500 μl. The liposomes were added to surfaces that were chemically modified with poly(ethylene)glycol and palmitic acid (PEG-PA). After incubation for 30 min at 15 room temperature, excess liposomes were removed by washing 2× with 2 ml HBS buffer. To fuse the liposomes to the surface, 2 ml of 10 % PEG8000 in HBS was added. After 15 min, the bilayer was washed extensively with 10 ml of HBS buffer. Single-molecule imaging of PSMs was performed using a TIRF microscope (Olympus) as described (68). Single-molecule localization, tracking, and cotracking were carried out using standard algorithms implemented into a self-written Matlab software package (69).

### Sample vitrification

For Cryo-EM sample preparation, AP-3 complexes were concentrated to a final concentration of 0.25 mg/ml, and 3 μl was applied onto freshly glow-discharged UltraAuFoil R 1.2/1.3300 mesh gold grids (Quantifoil GmbH). The sample was blotted using a 2.5 s blotting time and 0 blotting force with 100 % humidity at 4 °C and plunged into liquid ethane using the Vitrobot Mark III (FEI). The quality of the grids was screened using an Arctica microscope (FEI), operated at 200 kV. The grids were then stored in liquid nitrogen until data collection.

### Cryo-EM data acquisition

Cryo-EM datasets of AP-3 were collected on a C_s_-corrected Titan Krios transmission electron microscope (FEI), equipped with a high-brightness field-emission gun (XFEG) operated at an acceleration voltage of 300 kV. The images were acquired on a K2 summit direct electron detector (Gatan) operated in counting mode with a calibrated pixel size of 1.07 Å/pixel on the sample level with a post column GIF BioQuantum LS energy filter (Gatan) using a slit width of 20 eV. Two datasets were collected with a total of 6572 images. Images were collected for both datasets with 50 frames, an exposure time of 15 s resulting in a total dose of ∼81 e^−^ Å^−2^ and a defocus range of 1.5– 3.6 μm. All images were collected automatically using the automated data collection software EPU (FEI). Motion correction was performed using the MotionCor2 program (70).

### Single-particle cryo-EM data processing

All image-processing steps were carried out using the SPHIRE software package (44). Initially micrographs were manually evaluated in regard of ice quality high drift and were discarded accordingly. The remaining motion-corrected sums without dose weighting were evaluated in aspect of defocus and astigmatism in CTER, and low-quality images were discarded using the graphical CTF assessment tool in SPHIRE (44). In total, 958,892 single particles were automatically selected based on a trained model using the deep learning particle picker software crYOLO implemented in SPHIRE (44, 71). The particles were extracted from the dose-weighted motion-corrected sums with a final window size of 264 × 264. Precleaning of the dataset and reference-free 2D classification were performed with the iterative stable alignment and clustering approach ISAC2 in SPHIRE. Refined and sharpened 2D class averages with the original pixel size and enhanced high-resolution features were generated with the Beautifier tool implemented in SPHIRE. The best class averages were used to generate an initial 3D model of the AP-3 complex using RVIPER (44). This model was used for the initial 3D refinement step in MERIDIEN implemented in SPHIRE (44), using the remaining 647,557 particles after the precleaning step in ISAC. Afterward the obtained projection parameters during the initial 3D refinement as well as the optimized resulting cryo-EM density at an estimated resolution of ∼15 Å were used for second 3D refinement step. The optimized projection parameters were used for further heterogeneity analysis of the AP-3 complexes. Initial 3D Sorting using SORT3D implemented in SPHIRE (44) resulted in five different classes, showing high flexibility in all four domains. For further analysis of the inherent flexibility of the AP-3 complex, the cryoDRGN program (46, 47) was used to further separate different classes of AP-3 conformations. To assess heterogeneity of the AP-3 complex, we trained a 10D latent variable model, which resulted in 20 distinct 3D reconstructions, representing individual conformations of the open state of AP-3. These reconstructions were used as references for subsequent 3D refinements (Fig. S2*A*, Movie S2). Each class corresponding to the cryoDRGN reconstructions encompassed approximately 20k–30k particles, with the exception of class 19 (∼5k particles), which was disregarded for further analysis. For analysis of the open conformations of the AP-3 full-length complex, the cryoDRGN classes were sorted from a compact to a stretched conformation (Movie S2), and three maps were chosen representing putative compact, intermediate, and stretched conformations of the AP-3 complex. The estimated global resolution according to the gold standard FSC@0.5/0.143 criterion between the two masked half-maps was 9.1/7.9 Å for the compact, 10.1/8.6 Å for the intermediate, and 10.5/8.8 Å for the stretched conformation (Fig. S1, *F* and *G*). The local resolution and the angular distribution of the three maps were calculated in SPHIRE (Fig. S1, *D* and *E*). The resulting colored density maps showed a local resolution of up to 6 Å in the core of the molecule (6–10 Å), whereas the exterior of the molecules shows the lowest resolution (38–45 Å) for all three conformations. Directional FSC plots for the three cryo-EM maps were generated using the 3DFSC server (72) (Fig. S1*F*). The angular distribution plot as well as the calculated FSC plot indicates minor overrepresentation of views in x-direction as well as missing views due to the limited number of particles (20k) in the final reconstructions, resulting in minor elongation of the helical structure elements, but overall do not result in any deformation in y- and z-direction of the whole molecule as indicated by comparable measurements of the molecule length in the 2D classes and the 3D reconstructions. The workflow of the image processing strategy including the obtained 3D classes by Sort3D, and the number of particles they contained is described in detail in Figure S2*A*.

### Model building and structure analysis

Three cryoDRGN maps after 3D refinement representing the compact, intermediate, and stretched open conformation were chosen for modeling of the AP-3 complex. A representative workflow of the model building strategy for the compact conformation is depicted in Figure S2*B*. In the initial step, the crystal structure of AP-1 in its open conformation (pdb-ID: 4HMY) was rigid body fitted into the cryo-EM map using the “Fit to Map” function of UCSF Chimera. To build the final homology model of the AP-3 core complex and analyze the position of the appendages of Apl5 and Apl6, the structures of the four subunits Apl5, Apl5, Apm3, and Aps3 were generated by the *de novo* structure prediction server trRosetta (73) (Fig. S3). These models were superpositioned with the crystal structure of AP-1 using the “Matchmaker” function in UCSF Chimera. Since trRosetta uses the best homologous pdb template for structure prediction, the predicted models of the subunits showed high structural similarity to the AP-1 and AP-2 subunits apart from the overall curvature of the α-solenoid. Importantly, these models showed additional structural features (*e.g.*, loops) that are missing in the AP-1 and AP-2 as indicated by the sequence alignment of the Apl5 and Apl6 with their corresponding homologs (Fig. 5, *C* and *F*). We therefore opted to use these models for final model building instead of the available crystal structures of AP-1 or AP-2. After initial positioning using the crystal structure of AP-1, all subunits were rigid-body fitted into the cryo-EM density maps of the putative compact, intermediate, and stretched form of AP-3 using the “Fit to Map” function of UCSF Chimera (74) (Fig. S2*B*). For the rigid-body fitting step, Apl5 and Aps3 were combined to maintain the relative position of Aps3 to Apl5, since the putative density corresponding to the Aps3 subunit was less defined compared with the rest of the molecule. Furthermore, Apm3 was split into its N-terminal and C-terminal domain. To improve the quality of the fit of the adaptins, the rigid-body fitted subunits were combined and used for flexible fitting using iMODFIT, which is suitable for flexible fitting of structures at lower resolutions (75). In the final flexible fitting step, the ear domains of Apl5 (aa: 639–932) and Apl6 (aa: 631–809) were deleted from the model since no extra density for these domains could be observed. The flexible fitted models of the Apl5 and Apl6 subunits, which mainly consist of α-helices, were mostly well placed in the visible secondary structure features in the cryo-EM densities with average resolutions of ∼7.9 Å, 8.6 Å, and 8.8 Å. Furthermore, the large C-terminal ß-sheet domain of Apm3 could also be flexibly fitted into the cryo-EM densities. In contrast, the densities corresponding to the small ß-sheet subunits Aps3 and the N-terminal domain of Apm3 (aa:1–138) were not well defined. Thus, these domains were only rigid-body fitted into the densities under consideration of their relative orientation and position of the corresponding domains in the AP-1 and AP-2 complexes. To complete the model, the flexible fitted subunits Apl5, Apl6, and the C-terminal domain of Apm3 were combined with the rigid-body fitted N-terminal domain of Apm3 and the Aps3 subunit. Morphing between the two conformers was calculated using the tool “Morph Conformations” in Chimera and recorded as a movie.

## Data availability

The mass spectrometry proteomics data have been deposited to the ProteomeXchange Consortium *via* the PRIDE (76) partner repository with the dataset identifier PXD028005. All remaining data are contained within the article.

## Supporting information

This article contains supporting information.

## Conflict of interest

The authors declare that they have no conflicts of interest with the contents of this article.

## Supporting information

Supporting information

Data Sheet S1

Data Sheet S2
