# Supplementary material for: Flexible open conformation of the AP-3 complex explains its role in cargo recruitment at the Golgi
Source: J Biol Chem. 2021 Oct 22;297(5):101334. doi: 10.1016/j.jbc.2021.101334 (PMC8591511; doi:10.1016/j.jbc.2021.101334)
Supplement: Figures S1–S4 and Tables S1 and S [file mmc4.pdf]

## Supplemental information

# Flexible open conformation of the AP-3 complex explains its role in cargo recruitment at the Golgi

Jannis Schoppe, Evelyn Schubert, Amir Apelbaum, Erdal Yavavli, Oliver Birkholz, Heike Stephanowitz, Yaping Han, Angela Perz, Oliver Hofnagel, Fan Liu, Jacob Piehler, Stefan Raunser and Christian Ungermann

Figure S1 - Cryo-EM of the full-length AP-3 complex.

Figure S2 - Flowchart of image processing strategy in SPHIRE and model building of the AP-3 complex.

Figure S3 - *De novo* structure prediction of the four AP-3 subunits generated by trRosetta.

Figure S4 - Homology models of the three open conformations of the AP-3 complex flexibly fitted into the cryo-EM density maps.

Figure S5 - Crosslinking-MS on purified AP-3 complex shows potential ear domain localization.

Figure S6 - Both the SNAREs Nyv1 (a YxxΦcargo) and the dileucine containing Vam3 can recruit AP-3 to liposomes.

Movie S1 - 3D Sorting reveals high flexibility within all four subunits of the AP-3 complex.

Movie S2 - Heterogeneity analysis of the AP-3 complex by cryoDRGN.

Movie S3 - Transitions between the ‘open’ state conformations of the AP-3 complex.

Movie S4 - Association of AP-3 with polymer supported membranes (related to Figure 3A a)

Movie S5 - Association of AP-3 with membranes containing a Golgi lipid mix (related to Figure 7A b)

Movie S6 - Association of AP-3 with membranes containing a Golgi lipid mix and Arf1 (related to Figure 7A c)

Movie S7 - Mobility of Vam3 and AP-3 on membranes reveals co-mobility (Related to Figure 7C).

Movie S8 - Mobility of Vam3 on membranes containing a Golgi lipid mix (related to Figure 7E).

Movie S9 - Mobility of Vam3 upon AP-3 addition to on membranes (related to Figure 7E).

Table S1 - EM Data collection parameters of the AP-3 complex.

Table S2 - Yeast strains used in this study.

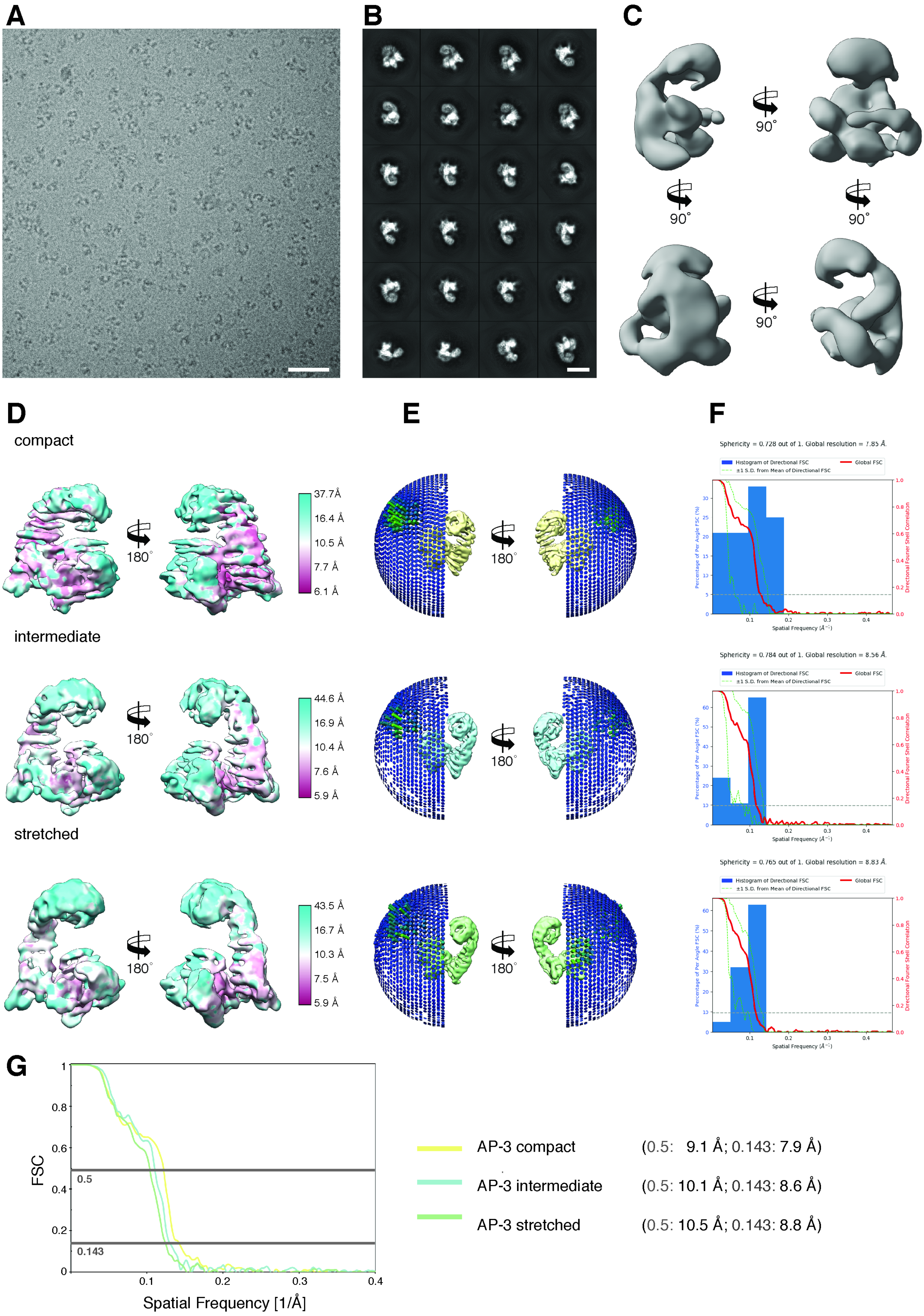
A
B
C
90°
90°
90°
90°
D
compact
180°
37.7 Å
16.4 Å
10.5 Å
7.7 Å
6.1 Å
intermediate
180°
44.6 Å
16.9 Å
10.4 Å
7.6 Å
5.9 Å
stretched
180°
43.5 Å
16.7 Å
10.3 Å
7.5 Å
5.9 Å
E
180°
180°
180°
F
Sphericity = 0.728 out of 1. Global resolution = 7.85 Å.
Histogram of Directional FSC
Global FSC
±1 S.D. from Mean of Directional FSC
Percentage of Per Angle FSC (%)
Directional Fourier Shell Correlation
Spatial Frequency (Å⁻¹)
Sphericity = 0.784 out of 1. Global resolution = 8.56 Å.
Histogram of Directional FSC
Global FSC
±1 S.D. from Mean of Directional FSC
Spatial Frequency (Å⁻¹)
Sphericity = 0.765 out of 1. Global resolution = 8.83 Å.
Histogram of Directional FSC
Global FSC
±1 S.D. from Mean of Directional FSC
Spatial Frequency (Å⁻¹)
G
FSC
0.5
0.143
Spatial Frequency [1/Å]
AP-3 compact (0.5: 9.1 Å; 0.143: 7.9 Å)
AP-3 intermediate (0.5: 10.1 Å; 0.143: 8.6 Å)
AP-3 stretched (0.5: 10.5 Å; 0.143: 8.8 Å)

**Figure S1. Cryo-EM of the full-length AP-3 complex.**

**(A)** Representative digital micrograph of vitrified AP-3 complex. Scale bar: 50 nm. **(B)** Representative 2D class averages corresponding to A. Scale bar: 10 nm. (C) Rotated views of the initial 3D model generated by RVIPER implemented in SPHIRE. **(D)** Rotated views of the 3D reconstructions of compact (top, threshold 0.014), intermediate (middle, threshold 0.017) and stretched (bottom, threshold 0.012) conformation of the full-length AP-3 complex colored by local resolution. **(E)** 3D angular distribution of the three selected 3D reconstructions, corresponding to the compact (yellow), intermediate (blue) and stretched open conformation of full-length AP-3 in solution. The relative height of the bars represents the number of containing particles. **(F)** Histograms and directional FSC plots for the cryoEM density maps of the compact (top) intermediate (middle) and stretched (bottom) conformation of the AP-3 complex. **(G)** FSC curves between two independently refined half-maps of AP3 in its compact (yellow), intermediate (blue) and stretched conformation (green).

Figure S2 (Schoppe, Schubert et al.)

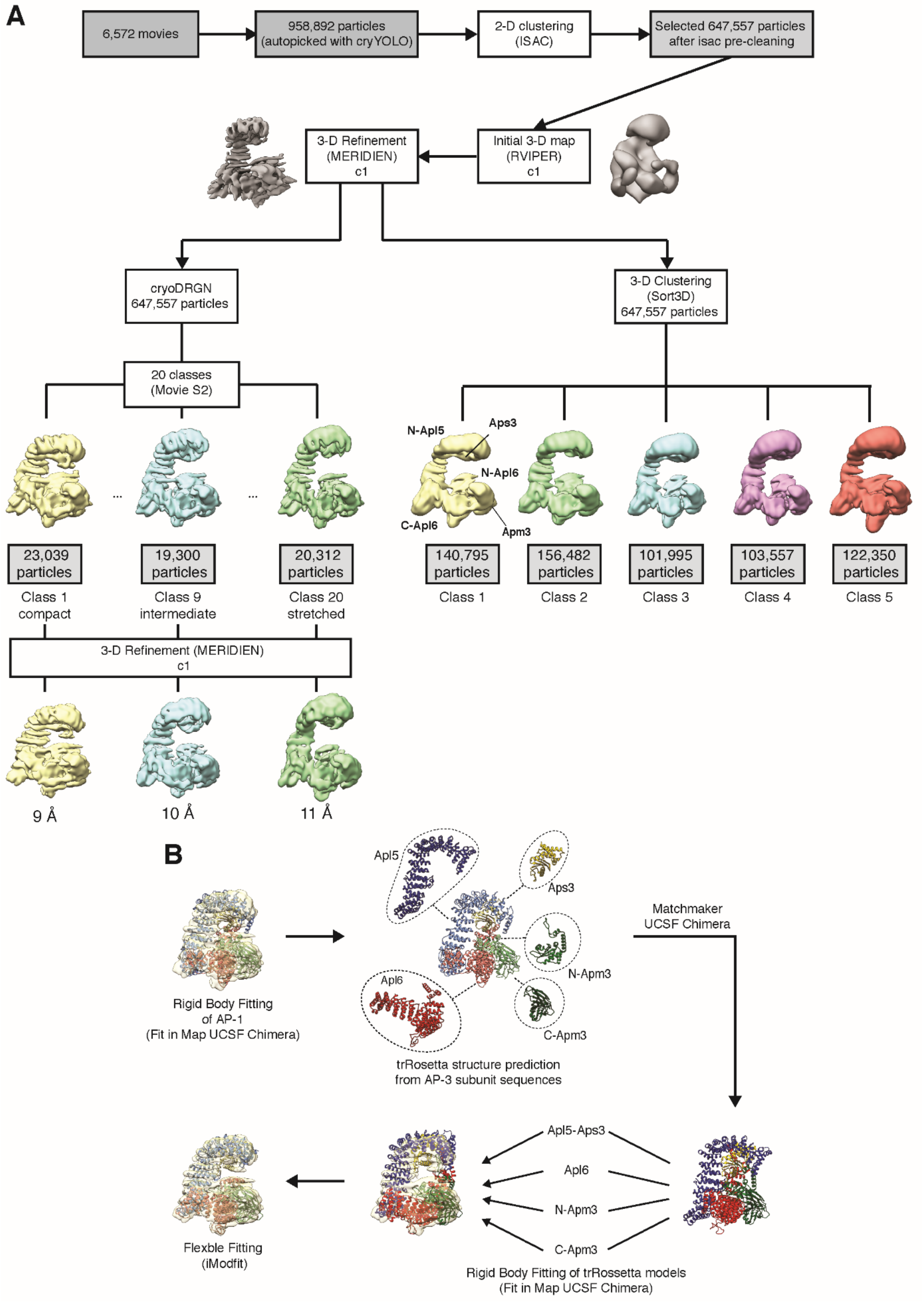

**Figure S2. Flowchart of image processing strategy in SPHIRE and model building of the AP-3 complex.**

**(A)** Single particle processing workflow of the initial refinement and 3D classification of the AP-3 complex in SPHIRE, including 3D heterogeneity analysis in cryoDRGN. Number of particles after automated particle picking, 2D classification and in each class after 3D Sorting is provided as a grey box. Three representative cryoDRGN maps of putative compact (c), intermediate (i) and stretched (s) 'open' conformations before (thresholds: c: 0.108, i: 0.135, s: 0.137) and after (thresholds: c: 0.014, i: 0.017, s: 0.012) refinement in MERIDIEN are depicted in yellow, blue and green, respectively. The five obtained maps from Sort3D (right) were sorted according to their conformation ranging from a compact to a more stretched conformation (threshold: 741). **(B)** Model Building strategy for the AP-3 homology model shown representative for the compact conformation. First, the crystal structure of AP-1 in its open conformation (pdb-ID: 4HMY) was rigid body fitted into the cryo-EM map (Fit in Map) and the trRosetta homology models of the AP-3 subunits were superimposed with the AP-1 structure the 'Matchmaker' function of UCSF Chimera. The pre-positioned subunits were then rigid-body fitted into the cryo-EM density (Fit in Map), combined into one molecule and subjected to flexible fitting in iMODFIT.

Figure S3 (Schoppe, Schubert et al.)

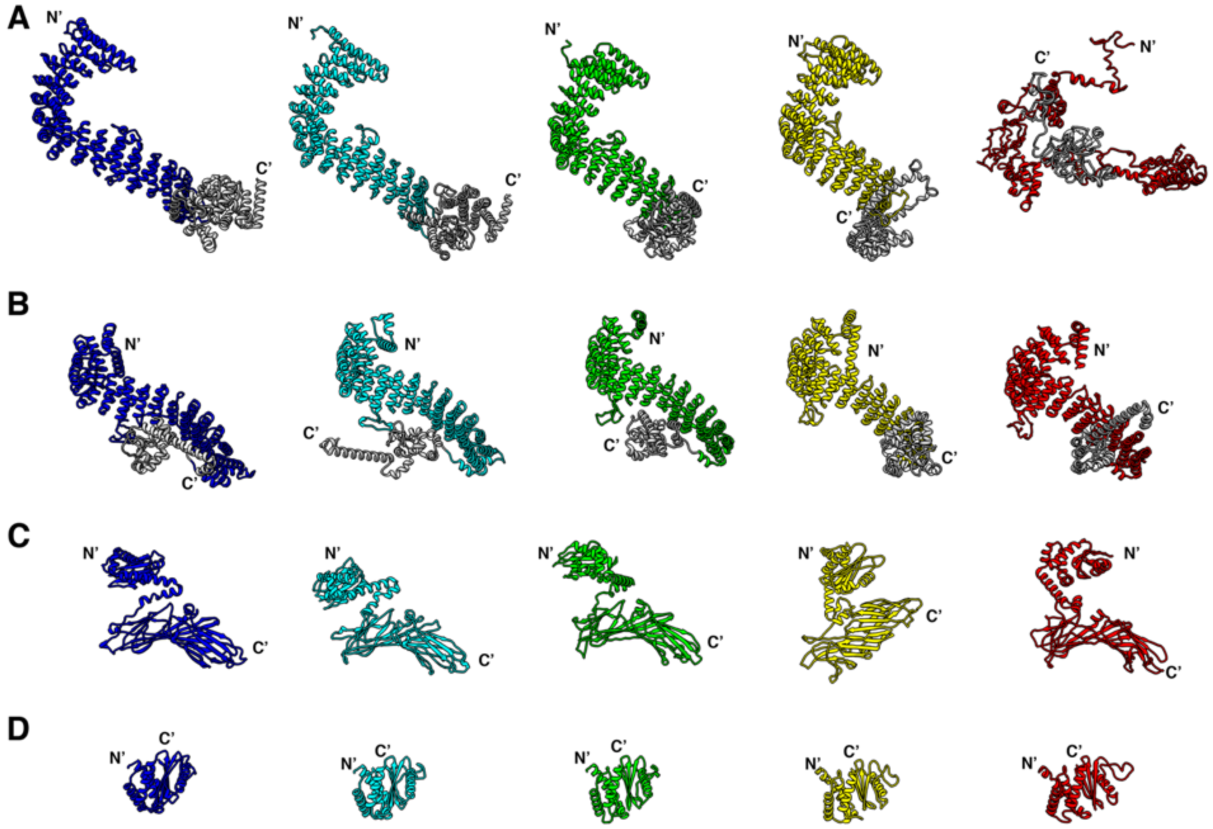

**Figure S3:** ***De novo*** **structure prediction of the four AP-3 subunits generated by trRosetta.**

**(A)** Predicted structures of the Apl5 subunit of the AP-3 complex. The ear domain (grey) is connected to the core of the Apl5 subunit via a large flexible linker, resulting in multiple orientations of the ear domain compared to the conserved core structure in the first four depicted molecules. **(B)** Predicted structures of the Apl6 subunit of the AP-3 complex. The ear domain of Apl6 (grey) is also connected to the core structure of Apl5 via a large flexible linker and therefore adapts different orientations similar to Apl6 (A). **(C)** Predicted structures of the Apm3 subunit of the AP-3 complex. The N-terminal domain of Apm3 is connected to the C-terminal domain via a large flexible linker resulting in different orientations of the N-terminal domain. **(D)** Predicted structures of the Aps3 subunit of the AP-3 complex. trRosetta predicted five different models for each subunit, which are depicted in blue, cyan, green, gold and red for each subunit. Almost all models show high structural similarities with the exception of model five of the Apl5 subunit (red), which is more unstructured than the other four predicted Apl5 structures. The ear domains of Apl5 and Apl6 are shown in grey.

Figure S4 (Schoppe, Schubert et al.)

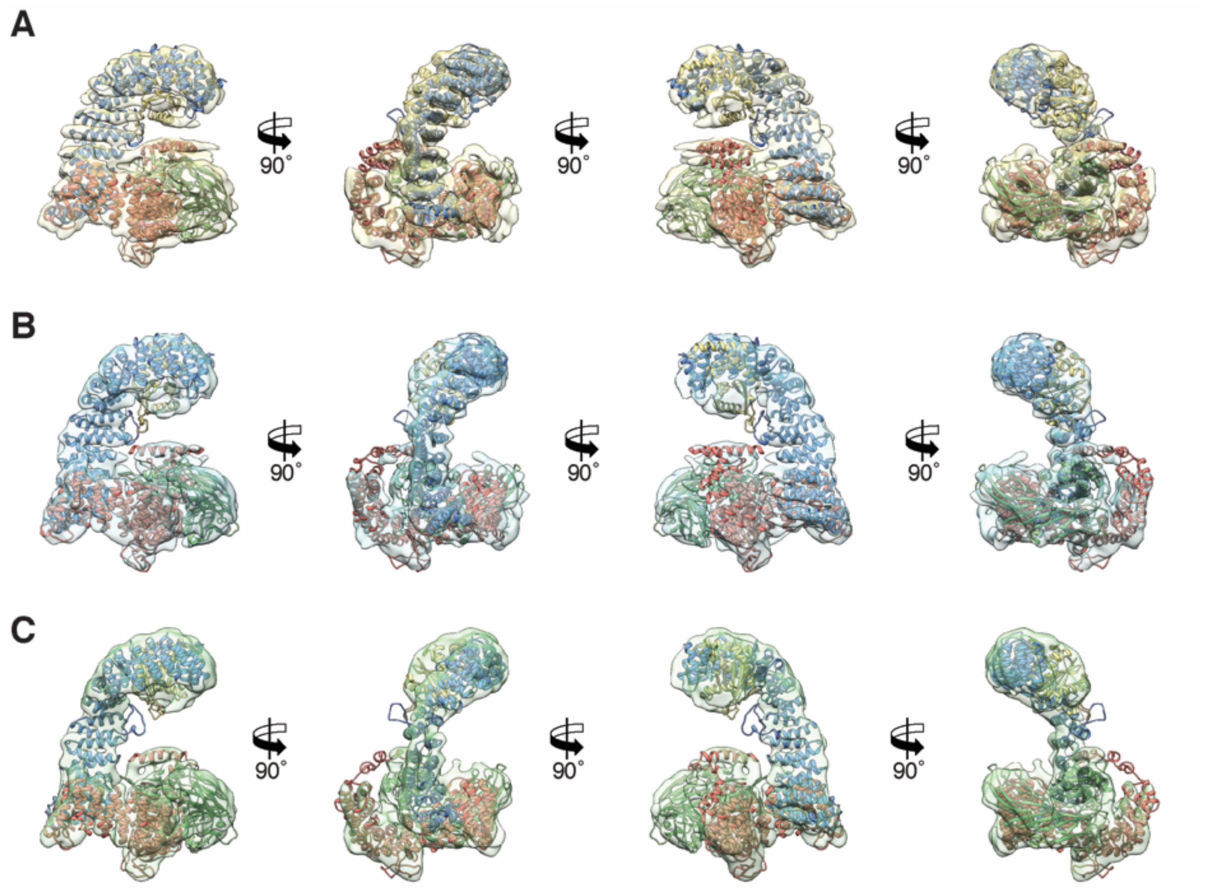

**Figure S4: Homology models of the three open conformations of the AP-3 complex flexibly fitted into the cryo-EM density maps.**

Rotated views of the cryo-EM density maps of the compact (threshold: 0.108) **(A)**, intermediate (threshold: 0.135) **(B)** and stretched (threshold: 0.137) **(C)** conformation of the open AP-3 complex with the homology model flexibly fitted. The four subunits Apl5, Apl6, Apm3 and Aps3 are depicted in blue, red, green and gold.

Figure S5 (Schoppe, Schubert et al.)

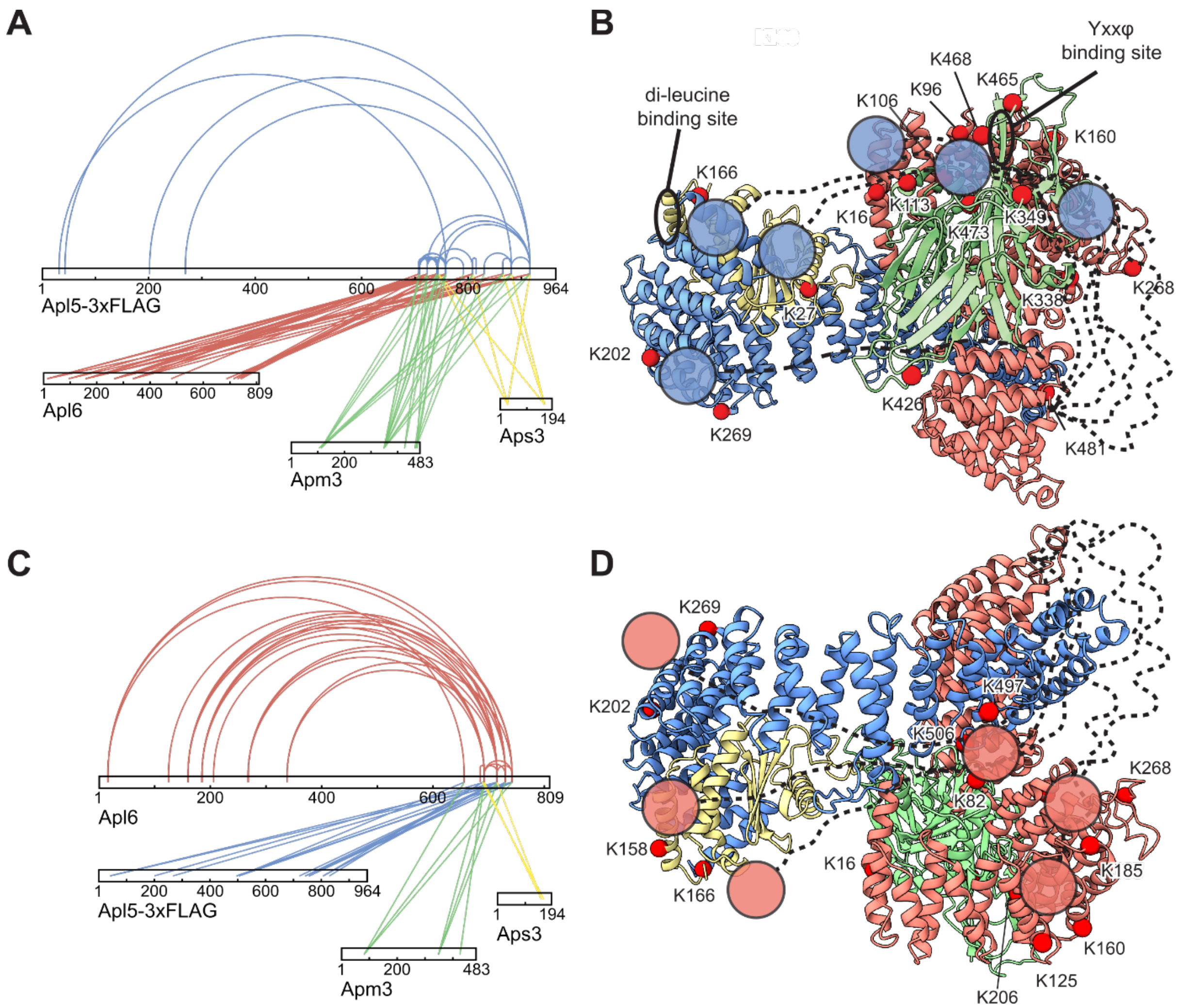

**Figure S5: Crosslinking-MS on purified AP-3 complex shows potential ear domain localization.** The purified AP-3 complex was crosslinked with disuccinimidyl sulfoxide (DSSO) and subsequently analyzed by mass spectrometry. Links to Apl5, Apl6, Apm3 and Aps3 are shown in blue, red, green, and gold, respectively. **(A)** All crosslinks of the Apl5 hinge and ear-domain (aa 639 – 932). The original dataset was filtered for confident crosslinks limited to this region of Apl5. **(B)** Putative path and locations of the flexible Apl5 ear domain based on the obtained crosslinks. **(C)** All crosslinks of the Apl6 hinge and ear-domain (aa 631 – 809). The original data set was filtered for confident crosslinks limited to this region of Apl6. **(D)** Putative paths and locations of the flexible Apl6 ear domain based on the obtained crosslinks. The crosslinks and the different paths of the ear domains are depicted as a red spheres and dashed lines, respectively.

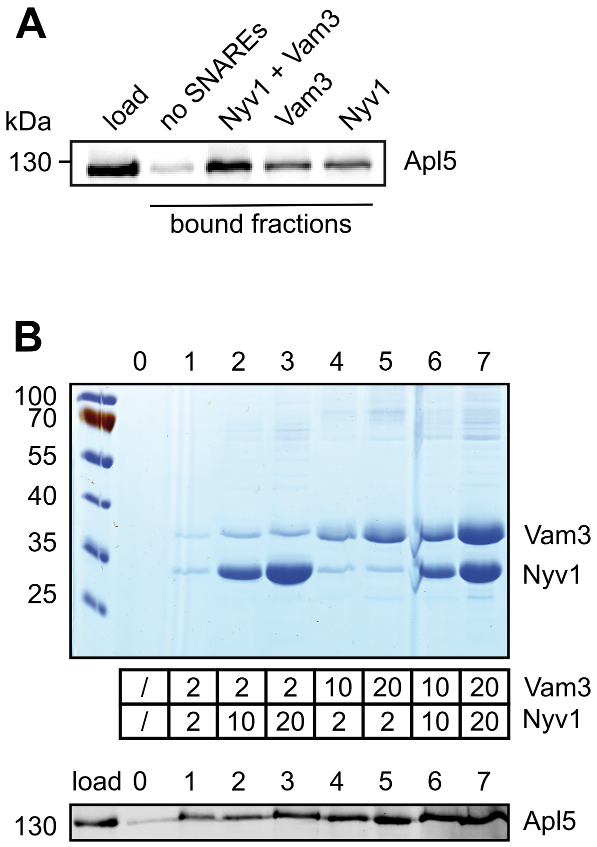
A
kDa
load
no SNAREs
Nyv1 + Vam3
Vam3
Nyv1
130
Apl5
bound fractions
B
0 1 2 3 4 5 6 7
100
70
55
40
35
25
Vam3
Nyv1
/ 2 2 2 10 20 10 20 Vam3
/ 2 10 20 2 2 10 20 Nyv1
load 0 1 2 3 4 5 6 7
130
Apl5

**Figure S6: Both the SNAREs Nyv1 (a Yxxϕ cargo) and the dileucine containing Vam3 can recruit AP-3 to liposomes. (A)** 0.5 mM of empty liposomes or proteoliposomes (Golgi-mimetic lipid composition, see methods) carrying of 500 nM of Vam3, Nyv1 or both were incubated with purified AP-3 and floated on a sucrose gradient. The top fraction was removed, proteins were precipitated by TCA, boiled in SDS sample buffer, and analyzed by SDS PAGE and Western blotting using an antibody against Apl5. **(B)** Effect of cargo composition on recruitment of the AP-3 complex. Proteoliposomes with Golgi-mimetic lipid compositions containing indicated amounts of Vam3 and Nyv1 (µM) were incubated with AP-3 and separated on a sucrose gradient. Top fractions were analyzed by SDS-PAGE and subsequent Coomassie staining to show SNARE composition and by Western Blot against Apl5 to reveal AP-3 binding.

## Supplemental Movies

**Movie S1. 3D Sorting reveals high flexibility within all four subunits of the AP-3 complex.**

The movie shows a morph between the five classes of the AP-3 complex obtained by 3D sorting in SORT3D implemented in SPHIRE. All classes appear to be in the ‘open’ conformation, but show conformational flexibility within the whole complex. This flexibility results in the up and down movement of the N-Apl5 and Aps3 subunits, which strongly interacts with the N-terminal domain of Apl5. In parallel, the helices of the N-Apl6 domain appear to wrap around the N-terminal domain of Apm3 causing the deformation of N-Apm3 upon extension and retraction of N-Apl6.

**Movie S2. Heterogeneity analysis of the AP-3 complex by cryoDRGN.**

The movie shows a morph between nineteen refined classes of AP-3 obtained during the heterogeneity analysis performed in cryoDRGN. Each class shows a distinct conformation of the AP-3 complex in its ‘open’ state. The classes were ordered from the compact conformation of the AP-3 complex to the stretched conformation. For visualization purposes the cryo-EM density maps were filtered to 12 Å, due to the mixed resolutions obtained for each class in the final refinements.

**Movie S3. Transitions between the ‘open’ state conformations of the AP-3 complex.**

We generated homology models of three observed ‘open’ conformations of the AP-3 complex obtained in cryoDRGN and created a continuous morph between all three conformations. The conformational transitions show that the N-terminal half of the $\delta$-subunit Apl5 can extend from or retract towards the trunk portion of the complex. We identified a putative hinge region for

this movement containing helices α21 and α22, which are connected by a large loop (lp δ aa: 399-421). At the same time, we observe two directional movements in the N- and C-terminal domain of Apl6, which diverges at a putative hinge region marked by a large loop (lp β). The N-terminal part of Apl6 twists either away (conformation 1-2) or towards (conformation 2-3) the Ams3 subunit, which results in a minor repositioning of N-Ams3. On the other site, C-Apl6 moves away from its N-terminal part and Ams3 subunit. Both identified loops in the putative hinge regions are unique for AP-3 and probably responsible for the higher flexibility compared to AP-1 and AP-2.

**Movie S4. Association of AP-3 with polymer supported membranes (related to Figure 3A a)**

The purified AP-3 complex with C-terminally GFP-tagged Apl6 was incubated with a nanobody labeled with Atto647 and added to PSM membranes containing dioleoyl-PC (DOPC). Recording was done for 15 sec by TIRF microscopy (for details see Methods).

**Movie S5. Association of AP-3 with membranes containing a Golgi lipid mix (related to Figure 7A b)**

Experiment was done as in Movie S1 except that PSM membranes were generated from a Golgi mimicking lipid mix (see Methods).

**Movie S6. Association of AP-3 with membranes containing a Golgi lipid mix and Arf1 (related to Figure 7A c)**

Purified AP-3 complex was added to PSM membranes containing a Golgi-mimicking lipid mix and Arf1-GTP, and mobility of AP-3 was monitored by TIRF microscopy (see Methods).

**Movie S7. Mobility of Vam3 and AP-3 on membranes reveals co-mobility (Related to Figure 7C).**

Purified AP-3 complex with C-terminally GFP-tagged Apl6 was incubated with a nanobody labeled with Alexa568 (blue) and added to PSM containing a Golgi-mimicking lipid mix and Vam3 labeled with Atto647 (red) and both were monitored over time by TIRF microscopy (see methods). Events of co-tracking is indicated by white lines.

**Movie S8. Mobility of Vam3 on membranes containing a Golgi lipid mix (related to Figure 7E).**

Membranes containing a Golgi-mimicking mix and Vam3, labeled with Atto647N (see Methods) were analyzed by TIRF microscopy. Mobility of Vam3 is shown by red lines (see Movie S1 and methods).

**Movie S9. Mobility of Vam3 upon AP-3 addition to on membranes (related to Figure 7E).**

Membranes containing a Golgi lipid mix and Vam3 labeled with Atto647 (red) were incubated with purified unlabeled AP-3 complex and Vam3 was monitored over time by TIRF microscopy (see methods). Mobility of Vam3 is shown by red lines.

**Supplemental Tables**

**Supplementary Table 1. EM Data collection parameters of the AP-3 complex.**

| | AP-3 compact<br>PDB-ID: 7P3X<br>EMDB ID: 13187 | AP-3 intermediate<br>PDB-ID: 7P3Y<br>EMDB ID: 13188 | AP-3 stretched<br>PDB-ID: 7P3Z<br>EMDB ID: 13189 |
|---|---|---|---|
| Data collectio | | | |
| Microscope | Titan Krios (Cs corrected, XFEG) | | |
| Voltage (kV) | 300 | | |
| Camera | K2 summit (Gatan) | | |
| Camera mode | Counting | | |
| Magnification | 130k | | |
| Pixel size (Å) | 1.07 | | |
| No. of frames | 50 | | |
| Total electron dose ($e^-$/Å$^2$) | 81 | | |
| Exposure time (s) | 1.62 | | |
| Defocus range (µm) | 1.5-3.6 | | |
| Movies | 6,572 | | |
| Data processing | | | |
| No. of picked particles (2D class.) | 958,892 | | |
| No. of particles (3Dref, 3D class.) | 647,557 | | |
| Symmetry imposed | C1 | | |
| Box size | 264 | | |
| No. of particles (final) | 23,039 | 19,300 | 20,312 |
| Global Resolution (0.5/0.143) (Å) | 9.1/7.9 | 10.1/8.6 | 10.5/8.8 |
| Map sharpening B-factor | -122 | -323 | -345 |

**Supplementary Table 2.** Yeast strains used in this study.

| | Genotype | Reference |
|---|---|---|
| CUY9659 | MATa *his3*Δ200 *leu2*Δ0 *met15*Δ0 *trp1*Δ63 *ura3*Δ0 *APS3*::TRP1-GAL1pr *APL6*::natNT2-Gal1pr x MATalpha *his3*Δ200 *leu2*Δ0 *lys2*Δ0 *met15*Δ0 *trp1*Δ63 *ura3*Δ0 *APL5*::kanMX-GAL1pr *APL5*::3xFLAG-hphNT1 *APM3*::TRP1-GAL1pr | This study |
| CUY9913 | MATa *his3*Δ200 *leu2*Δ0 *met15*Δ0 *ura3*Δ0 *APS3*::TRP1-GAL1pr *APL6*::natNT2-Gal1pr *APL6*::mGFP-hphNT1 x MATalpha *his3*Δ200 *leu2*Δ0 *lys2*Δ0 *met15*Δ0 *trp1*Δ63 *ura3*Δ0 *APL5*::kanMX-GAL1pr *APL5*::3xFLAG-hphNT1 *APM3*::TRP1-GAL1pr | This study |
| CUY11125 | MATalpha *his3*Δ200 *leu2*Δ0 *lys2*Δ0 *met15*Δ0 *trp1*Δ63 *ura3*Δ0 *APL4*::3xFLAG-hphNT1 *APL4*::natNT2-GAL1pr *APM1*::TRP1-GAL1pr x MATa *his3*Δ200 *leu2*Δ0 *met15*Δ0 *trp1*Δ63 *ura3*Δ0 *APS1*::URA3-GAL1pr *APL2*::TRP1-GAL1pr | This study |
