# Supplementary material for: Flexible open conformation of the AP-3 complex explains its role in cargo recruitment at the Golgi
Source: J Biol Chem. 2021 Oct 22;297(5):101334. doi: 10.1016/j.jbc.2021.101334 (PMC8591511; doi:10.1016/j.jbc.2021.101334)
Supplement: Supporting information [file mmc5.pdf]

Full wwPDB EM Validation Report (i)

Jul 9, 2021 – 02:54 pm BST

PDB ID	:	7P3X
EMDB ID	:	EMD-13187
Title	:	Homology model of the full-length AP-3 complex in a compact open conformation
Deposited on	:	2021-07-09
Resolution	:	9.10 Å(reported)

This is a Full wwPDB EM Validation Report.

This report is produced by the wwPDB biocuration pipeline after annotation of the structure.

We welcome your comments at *validation@mail.wwpdb.org*
A user guide is available at
https://www.wwpdb.org/validation/2017/EMValidationReportHelp
with specific help available everywhere you see the (i) symbol.

The following versions of software and data (see references (i)) were used in the production of this report:

EMDB validation analysis	:	0.0.0.dev84
MolProbity	:	4.02b-467
Percentile statistics	:	20191225.v01 (using entries in the PDB archive December 25th 2019)
Ideal geometry (proteins)	:	Engh & Huber (2001)
Ideal geometry (DNA, RNA)	:	Parkinson et al. (1996)
Validation Pipeline (wwPDB-VP)	:	2.22

1 Overall quality at a glance (i)

The following experimental techniques were used to determine the structure:
ELECTRON MICROSCOPY

The reported resolution of this entry is 9.10 Å.

Percentile scores (ranging between 0-100) for global validation metrics of the entry are shown in the following graphic. The table shows the number of entries on which the scores are based.

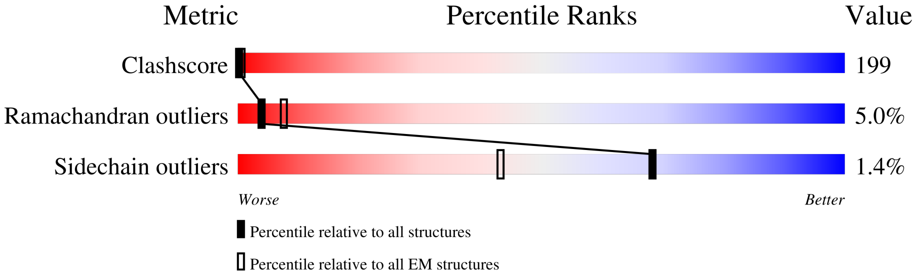

Metric	Whole archive (#Entries)	EM structures (#Entries)
Clashscore	158937	4297
Ramachandran outliers	154571	4023
Sidechain outliers	154315	3826

The table below summarises the geometric issues observed across the polymeric chains and their fit to the map. The red, orange, yellow and green segments of the bar indicate the fraction of residues that contain outliers for >=3, 2, 1 and 0 types of geometric quality criteria respectively. A grey segment represents the fraction of residues that are not modelled. The numeric value for each fraction is indicated below the corresponding segment, with a dot representing fractions <=5% The upper red bar (where present) indicates the fraction of residues that have poor fit to the EM map (all-atom inclusion < 40%). The numeric value is given above the bar.

Mol	Chain	Length	Quality of chain
1	A	964	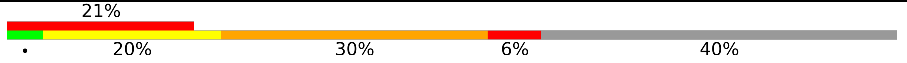
2	B	809	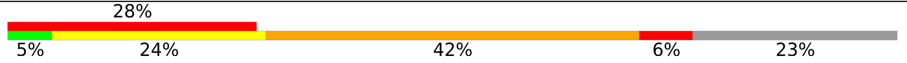
3	S	194	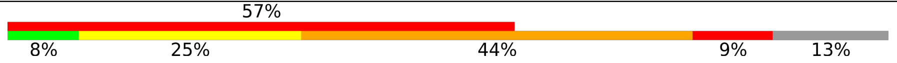
4	M	483	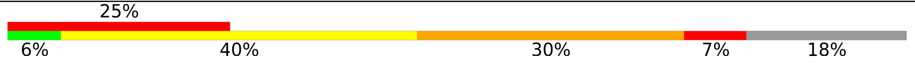

2 Entry composition (i)

There are 4 unique types of molecules in this entry. The entry contains 14104 atoms, of which 0 are hydrogens and 0 are deuteriums.

In the tables below, the AltConf column contains the number of residues with at least one atom in alternate conformation and the Trace column contains the number of residues modelled with at most 2 atoms.

- Molecule 1 is a protein called AP-3 complex subunit delta.

Mol	Chain	Residues	Atoms					AltConf	Trace
			Total	C	N	O	S		
1	A	576	4625	2978	738	881	28	0	0

There are 32 discrepancies between the modelled and reference sequences:

Chain	Residue	Modelled	Actual	Comment	Reference
A	933	ARG	-	expression tag	UNP A0A7I9C4X2
A	934	THR	-	expression tag	UNP A0A7I9C4X2
A	935	LEU	-	expression tag	UNP A0A7I9C4X2
A	936	GLN	-	expression tag	UNP A0A7I9C4X2
A	937	VAL	-	expression tag	UNP A0A7I9C4X2
A	938	ASP	-	expression tag	UNP A0A7I9C4X2
A	939	GLY	-	expression tag	UNP A0A7I9C4X2
A	940	SER	-	expression tag	UNP A0A7I9C4X2
A	941	ASP	-	expression tag	UNP A0A7I9C4X2
A	942	TYR	-	expression tag	UNP A0A7I9C4X2
A	943	LYS	-	expression tag	UNP A0A7I9C4X2
A	944	ASP	-	expression tag	UNP A0A7I9C4X2
A	945	ASP	-	expression tag	UNP A0A7I9C4X2
A	946	ASP	-	expression tag	UNP A0A7I9C4X2
A	947	ASP	-	expression tag	UNP A0A7I9C4X2
A	948	LYS	-	expression tag	UNP A0A7I9C4X2
A	949	ASP	-	expression tag	UNP A0A7I9C4X2
A	950	TYR	-	expression tag	UNP A0A7I9C4X2
A	951	LYS	-	expression tag	UNP A0A7I9C4X2
A	952	ASP	-	expression tag	UNP A0A7I9C4X2
A	953	ASP	-	expression tag	UNP A0A7I9C4X2
A	954	ASP	-	expression tag	UNP A0A7I9C4X2
A	955	ASP	-	expression tag	UNP A0A7I9C4X2
A	956	LYS	-	expression tag	UNP A0A7I9C4X2
A	957	ASP	-	expression tag	UNP A0A7I9C4X2
A	958	TYR	-	expression tag	UNP A0A7I9C4X2
A	959	LYS	-	expression tag	UNP A0A7I9C4X2
A	960	ASP	-	expression tag	UNP A0A7I9C4X2

Continued on next page...

Continued from previous page...

Chain	Residue	Modelled	Actual	Comment	Reference
A	961	ASP	-	expression tag	UNP A0A7I9C4X2
A	962	ASP	-	expression tag	UNP A0A7I9C4X2
A	963	ASP	-	expression tag	UNP A0A7I9C4X2
A	964	LYS	-	expression tag	UNP A0A7I9C4X2

- Molecule 2 is a protein called Y55_G0035830.mRNA.1.CDS.1.

Mol	Chain	Residues	Atoms					AltConf	Trace
2	B	621	Total 4963	C 3168	N 830	O 937	S 28	0	0

- Molecule 3 is a protein called AP complex subunit sigma.

Mol	Chain	Residues	Atoms					AltConf	Trace
3	S	168	Total 1358	C 867	N 215	O 272	S 4	0	0

- Molecule 4 is a protein called AP-3 complex subunit mu.

Mol	Chain	Residues	Atoms					AltConf	Trace
4	M	397	Total 3158	C 2018	N 516	O 612	S 12	0	0

3 Residue-property plots

These plots are drawn for all protein, RNA, DNA and oligosaccharide chains in the entry. The first graphic for a chain summarises the proportions of the various outlier classes displayed in the second graphic. The second graphic shows the sequence view annotated by issues in geometry and atom inclusion in map density. Residues are color-coded according to the number of geometric quality criteria for which they contain at least one outlier: green = 0, yellow = 1, orange = 2 and red = 3 or more. A red diamond above a residue indicates a poor fit to the EM map for this residue (all-atom inclusion < 40%). Stretches of 2 or more consecutive residues without any outlier are shown as a green connector. Residues present in the sample, but not in the model, are shown in grey.

- Molecule 1: AP-3 complex subunit delta

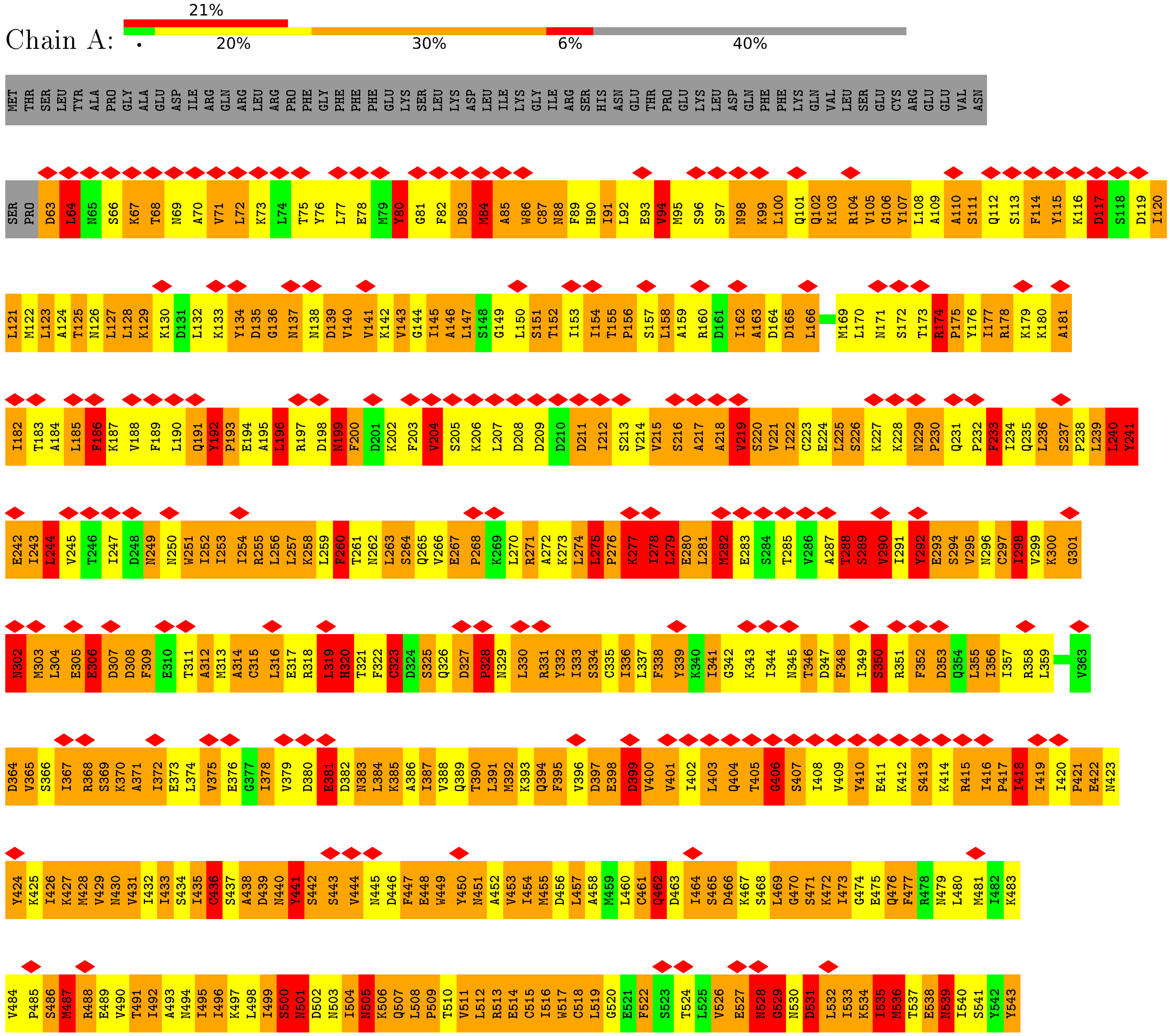

S544 H545 S546 V547 Q548 E549 V550 L551 I552 L553 A554 L555 V556 K557 V558 F559 S560 N561 W562 C563 N564 N565 F566 Q567 E568 D569 K570 R571 F572 E573 I574 K575 M576 V577 L578 K579 E580 L581 I582 E583 F584 F585 E586 N587 L588 S589 Y590 T593 F594 E595 V596 Q597 E598 R599 S600 V601 E602 V603 L604

E605 F606 L607 R608 L609 S610 L611 E612 A613 L614 E615 E616 D617 T618 E619 G620 L621 P622 M623 L624 L625 S626 E627 V628 L629 P630 S631 F632 F633 N634 A635 Y636 E637 L638 ALA PRO ILE ALA ARG GLY THR GLN LEU LYS LEU ALA VAL ASP GLU ASN LEU ASP LEU GLU THR PRO PHE LEU THR LYS

GLU ALA ALA ASP GLU LEU LEU ASP GLU GLN LYS SER ASP ALA ILE SER ASP LEU MET SER ASP ILE SER MET ASP GLU GLN VAL GLU LEU LYS PHE VAL ASP ASP SER ASP THR SER TYR GLU GLU LYS GLU LYS LEU ASP ASP PHE GLU ASN PRO PHE GLU ILE GLU ARG GLU LYS GLU

ARG MET SER ASN PRO TYR THR LEU GLY GLU GLU ASP GLU ARG THR LYS ASN SER LYS ASP LEU LEU ASP LEU ASN GLU GLU SER SER ASP LYS LYS PRO GLU THR ILE ARG LEU ASN ARG THR ASP ASN SER LEU ASN SER LEU SER LEU SER THR THR GLU ILE SER ARG LYS

LYS LYS LYS GLY LYS LYS LYS ASN ARG VAL GLN VAL LEU SER ASP GLU PRO VAL ILE GLU ALA ALA PRO LYS ARG LYS ASP ALA PHE GLN LYS PRO HIS ASP ASN HIS SER THR GLN ASN PRO LEU LYS LYS ASP LYS ILE ASN LEU ARG MET HIS SER GLN LEU GLU ASN PHE ASP PHE

SER ASN PHE GLY GLN SER SER ASN ALA GLY ARG GLY SER GLN GLU GLU GLY ASN LEU ARG LYS GLU ASP GLU LEU GLU LEU SER ARG LEU GLU ALA ASN LEU ILE VAL LYS ASP GLU LYS ASP ASN ASN LEU SER ASP THR GLU GLU VAL ILE VAL ILE LYS LYS LYS LYS LYS GLY LYS LYS

SER LYS SER LYS ASN LYS LEU LYS THR LYS ALA LYS ASN SER PRO GLU PRO ASN GLU PHE LEU ARG ASP GLN SER THR ASP ILE ARG THR LEU GLN VAL ASP GLY SER ASP TYR LYS ASP ASP ASP LYS ASP TYR LYS ASP ASP ASP LYS ASP TYR LYS ASP ASP ASP LYS

- Molecule 2: Y55_G0035830.mRNA.1.CDS.1

Chain B: 28% | 5% | 24% | 42% | 6% | 23%

M1 V2 I5 H6 R7 I8 A9 S10 A11 L12 D13 T14 A15 K16 V17 I18 T19 R20 E21 A22 A23 A24 V25 A26 T27 S28 K29 L30 G31 E32 S33 S34 Y35 T36 Y37 Y38 S39 Q40 N41 I42 N43 P44 Q45 Q46 L47 V48 T49 L50 L51 N52 S53 R54 N55 S56 R57 E58 V59 R60 D61

A62 M63 K64 R65 I66 I67 S68 I69 M70 A71 S72 D73 D74 D75 S76 I77 D78 V79 Q80 L81 Y82 F83 A84 D85 V86 V87 K88 N89 I90 T91 T92 N93 D94 T95 K96 V97 K98 R99 L100 I101 H102 L103 Y104 L105 L106 R107 F108 A109 E110 N111 D112 L115 T116 L117 L118 S119 I120 N121 S122

L123 Q124 K125 S126 L127 S128 D129 S130 N131 S132 E133 L134 R135 C136 F137 A138 L139 S140 A141 L142 S143 D144 M145 K146 M147 S148 S149 L150 A151 P152 I153 I154 L155 H156 T157 V158 K159 K160 L161 V162 T163 D164 P165 S166 A167 M168 V169 R170 G171 E172 V173 A174 L175 A176 I177 I178 K179 L180 Y181 R182

A183 G184 K185 N186 D187 Y188 H189 E190 E191 L192 L193 D194 I195 L196 K197 E198 L199 M200 A201 D202 T203 D204 P205 K206 V207 I208 S209 C210 A211 V212 L213 A214 Y215 K216 E217 C218 Y219 A220 D221 H222 L223 E224 L225 L226 H227 G228 H229 F230 R231 R232 Y233 C234 R235 I236 I237 K238 Q239 L240 D241 S242

W243 S244 Q245 S246 Y247 L248 I249 E250 L251 L252 I253 K254 Y255 C256 K257 Q258 Y259 L260 P261 K262 P263 T264 V265 V266 D267 K268 S269 S270 E271 G272 S273 P274 R275 S276 C277 P278 L279 P280 D281 K282 Y283 N284 E285 I286 E287 Y288 P289 S290 Y291 E292 V293 V294 N295 D296 P297 D298 L299 D300 L301 F302

L303 Q304 S305 L306 N307 C308 L309 I310 Y311 S312 S313 N314 P315 T316 V317 I318 L319 S320 C321 C322 N323 A324 L325 Y326 Q327 L328 A329 S330 P331 L332 Q333 M334 K335 N336 T337 K338 F339 I340 E341 A342 L343 V344 R345 T346 V347 T348 M349 T350 E351 N352 Q353 G354 N355 K356 E357 M358 L359 L360 Q361 A362

I363 H364 F365 L366 S367 I368 L369 D370 Q371 T372 L373 F374 L375 P376 Y377 T378 K379 K380 F381 Y382 V383 F384 P385 K386 D387 P388 I389 V390 A391 S392 I393 W394 K395 I396 Q397 I398 L399 S400 T401 L402 I403 N404 E405 S406 N407 V408 K409 E410 I411 F412 K413 E414 L415 K416 Y417 Y418 V419 A420 S421 A422

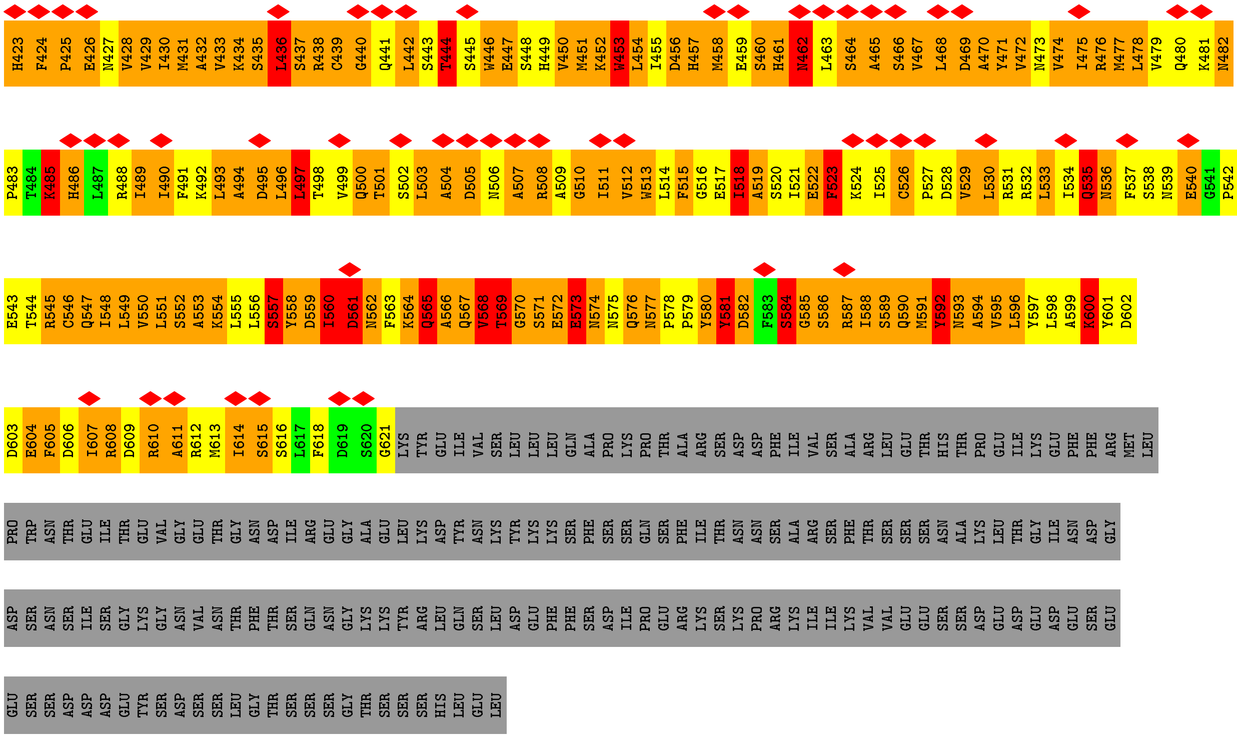

- Molecule 3: AP complex subunit sigma

Chain S:

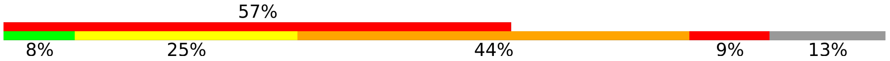

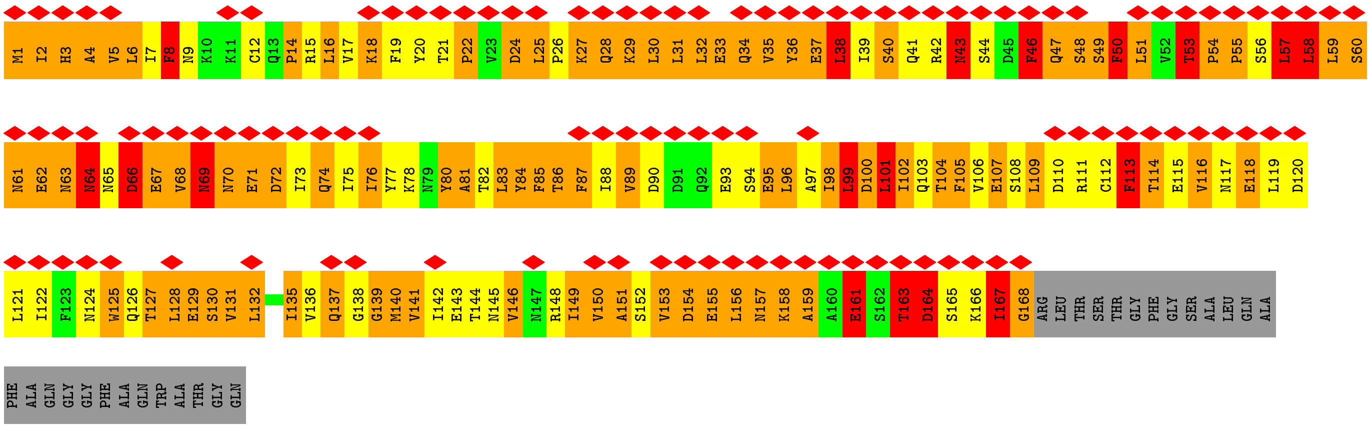

- Molecule 4: AP-3 complex subunit mu

Chain M:

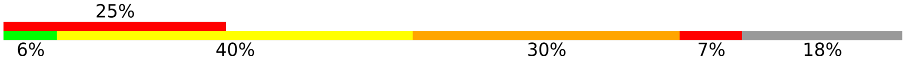

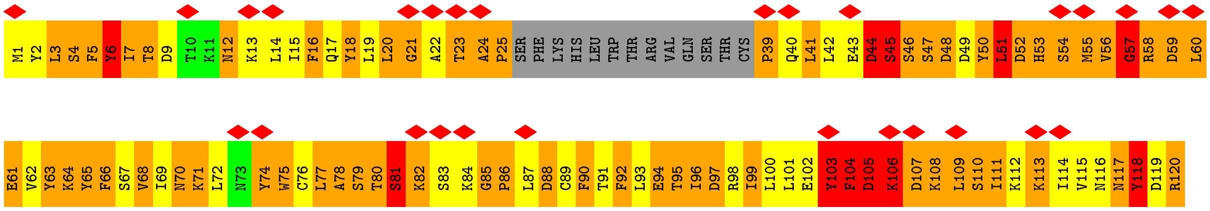

I121 S122 L123 I124 F125 N126 C127 C128 V129 E130 A131 G132 E133 P134 N135 V136 S137 D138 MET LEU TYR VAL ASN LYS ILE LYS GLU ALA VAL PRO GLU ARG SER ASP LEU SER LYS PHE ILE SER SER THR ALA HIS ASN LEU GLN GLN ALA VAL GLN LEU PRO GLN GLN ARG GLN GLN GLN LEU

GLN GLN ASN GLN ILE SER ARG GLY SER ASN SER LEU ILE GLU ASN GLU GLU ILE VAL PRO TRP ARG THR SER ARG ALA SER LYS HIS GLU ASN N212 E213 L214 Y215 V216 D217 L218 L219 E220 T221 F222 H223 V224 V225 F226 E227 K228 K229 K230 S231 H232 L233 R234 L235 L236 T237 G238 S239 I240

H241 G242 I243 V244 D245 V246 R247 S248 Y249 L250 N251 D252 N253 P254 L255 V256 A257 V258 K259 L260 N261 T262 M263 G264 N265 D266 I267 G268 I269 P270 S271 L272 H273 D274 C275 V276 E277 I278 N279 D280 G281 V282 F283 S284 P285 S286 N287 I288 T289 F290 I291 P292 P293 D294 G295 K296 F297 R298 L299 L300

E301 Y302 S303 V304 D305 L306 S307 S308 Q309 V310 K311 Q312 S313 G314 V315 R316 M317 N318 S319 I320 G321 L322 M323 S324 L325 H326 F327 Q328 N329 G330 L331 G332 K333 D334 S335 D336 E337 F338 E339 L340 S341 L342 N343 I344 E345 N346 F347 K348 K349 V350 S351 Q352 V353 D354 D355 L356 K357 I358 D359 L360

Q361 F362 N363 V364 E365 N366 A367 D368 P369 N370 E371 I372 A373 Y374 K375 I376 K377 I378 L379 R380 N381 T382 H383 G384 R385 F386 E387 N388 S389 I390 I391 M392 G393 Q394 G395 Q396 W397 I398 F399 D400 K401 S402 T403 A404 T405 G406 T407 V408 P409 V410 L411 R412 G413 C414 I415 E416 Y417 E418 N419 T420

G421 P422 N423 F424 T425 K426 K427 V428 D429 L430 Q431 T432 V433 S434 L435 E436 Y437 S438 Y439 I440 G441 Q442 S443 A444 S445 G446 I447 Y448 V449 E450 A451 I452 D453 I454 V455 S456 G457 L458 T459 I460 G461 K462 N463 T464 K465 L466 Y467 K468 G469 A470 K471 Y472 K473 T474 Q475 T476 G477 N478 F479 Q480

V481 R482 L483

4 Experimental information (i)

Property	Value	Source
EM reconstruction method	SINGLE PARTICLE	Depositor
Imposed symmetry	POINT, C1	Depositor
Number of particles used	23039	Depositor
Resolution determination method	FSC 0.5 CUT-OFF	Depositor
CTF correction method	PHASE FLIPPING ONLY	Depositor
Microscope	FEI TITAN KRIOS	Depositor
Voltage (kV)	300	Depositor
Electron dose $(e^-/\text{Å}^2)$	81	Depositor
Minimum defocus (nm)	1500	Depositor
Maximum defocus (nm)	3600	Depositor
Magnification	130000	Depositor
Image detector	GATAN K2 SUMMIT (4k x 4k)	Depositor
Maximum map value	0.049	Depositor
Minimum map value	-0.014	Depositor
Average map value	0.000	Depositor
Map value standard deviation	0.002	Depositor
Recommended contour level	0.015	Depositor
Map size (Å)	282.48, 282.48, 282.48	wwPDB
Map dimensions	264, 264, 264	wwPDB
Map angles (°)	90.0, 90.0, 90.0	wwPDB
Pixel spacing (Å)	1.07, 1.07, 1.07	Depositor

5 Model quality (i)

5.1 Standard geometry (i)

The Z score for a bond length (or angle) is the number of standard deviations the observed value is removed from the expected value. A bond length (or angle) with $|Z| > 5$ is considered an outlier worth inspection. RMSZ is the root-mean-square of all Z scores of the bond lengths (or angles).

Mol	Chain	Bond lengths		Bond angles	
		RMSZ	#|Z| >5	RMSZ	#|Z| >5
1	A	1.85	106/4699 (2.3%)	2.69	616/6358 (9.7%)
2	B	1.71	91/5057 (1.8%)	2.45	596/6855 (8.7%)
3	S	2.51	75/1379 (5.4%)	2.79	167/1874 (8.9%)
4	M	2.39	156/3217 (4.8%)	2.55	283/4346 (6.5%)
All	All	2.01	428/14352 (3.0%)	2.59	1662/19433 (8.6%)

Chiral center outliers are detected by calculating the chiral volume of a chiral center and verifying if the center is modelled as a planar moiety or with the opposite hand.A planarity outlier is detected by checking planarity of atoms in a peptide group, atoms in a mainchain group or atoms of a sidechain that are expected to be planar.

Mol	Chain	#Chirality outliers	#Planarity outliers
1	A	0	54
2	B	0	31
3	S	1	18
4	M	1	24
All	All	2	127

All (428) bond length outliers are listed below:

Mol	Chain	Res	Type	Atoms	Z	Observed(Å)	Ideal(Å)
4	M	104	PHE	N-CA	-22.48	1.01	1.46
4	M	64	LYS	N-CA	-18.25	1.09	1.46
4	M	135	ASN	C-N	-17.55	0.93	1.34
4	M	132	GLY	CA-C	-17.17	1.24	1.51
4	M	281	GLY	CA-C	16.88	1.78	1.51
4	M	107	ASP	N-CA	-15.73	1.14	1.46
4	M	136	VAL	N-CA	-15.49	1.15	1.46
1	A	438	ALA	CA-CB	-15.46	1.20	1.52
3	S	49	SER	N-CA	-15.21	1.16	1.46
4	M	133	GLU	N-CA	-14.60	1.17	1.46
3	S	53	THR	N-CA	-14.44	1.17	1.46
3	S	68	VAL	C-N	14.43	1.67	1.34

Continued on next page...

Continued from previous page...

Mol	Chain	Res	Type	Atoms	Z	Observed(Å)	Ideal(Å)
4	M	132	GLY	N-CA	-13.97	1.25	1.46
4	M	65	TYR	CA-C	-13.82	1.17	1.52
4	M	63	TYR	CA-C	-13.76	1.17	1.52
3	S	48	SER	C-N	-13.29	1.03	1.34
4	M	131	ALA	CA-CB	13.14	1.80	1.52
4	M	293	PRO	N-CD	12.65	1.65	1.47
4	M	131	ALA	CA-C	-12.47	1.20	1.52
4	M	66	PHE	N-CA	-11.96	1.22	1.46
3	S	48	SER	CA-C	-11.71	1.22	1.52
1	A	406	GLY	CA-C	11.69	1.70	1.51
2	B	581	TYR	N-CA	-11.65	1.23	1.46
3	S	163	THR	CA-C	11.62	1.83	1.52
4	M	282	VAL	N-CA	11.60	1.69	1.46
4	M	3	LEU	C-N	-11.30	1.08	1.34
3	S	46	PHE	CA-C	-11.12	1.24	1.52
4	M	81	SER	C-N	-11.06	1.08	1.34
4	M	137	SER	N-CA	-11.02	1.24	1.46
4	M	295	GLY	CA-C	10.85	1.69	1.51
4	M	63	TYR	N-CA	-10.82	1.24	1.46
4	M	61	GLU	CA-C	-10.75	1.25	1.52
4	M	20	LEU	C-N	-10.60	1.14	1.33
3	S	64	ASN	CA-C	10.50	1.80	1.52
3	S	55	PRO	N-CA	10.50	1.65	1.47
4	M	56	VAL	CA-C	-10.34	1.26	1.52
4	M	105	ASP	C-N	-10.33	1.10	1.34
4	M	135	ASN	CA-C	-10.24	1.26	1.52
3	S	58	LEU	CA-C	-10.13	1.26	1.52
4	M	80	THR	C-N	-9.95	1.11	1.34
3	S	124	ASN	N-CA	-9.89	1.26	1.46
1	A	218	ALA	CA-C	-9.82	1.27	1.52
3	S	50	PHE	CA-C	-9.82	1.27	1.52
3	S	49	SER	C-N	9.81	1.56	1.34
4	M	65	TYR	N-CA	-9.77	1.26	1.46
4	M	131	ALA	N-CA	-9.65	1.27	1.46
1	A	404	GLN	N-CA	-9.64	1.27	1.46
2	B	274	PRO	N-CD	9.59	1.61	1.47
4	M	62	VAL	N-CA	-9.57	1.27	1.46
1	A	407	SER	N-CA	9.57	1.65	1.46
3	S	71	GLU	N-CA	-9.57	1.27	1.46
1	A	509	PRO	N-CD	9.36	1.60	1.47
4	M	62	VAL	CA-C	-9.34	1.28	1.52
4	M	130	GLU	C-N	-9.29	1.12	1.34

Continued on next page...

Continued from previous page...

Mol	Chain	Res	Type	Atoms	Z	Observed(Å)	Ideal(Å)
3	S	51	LEU	N-CA	-9.27	1.27	1.46
3	S	54	PRO	N-CA	9.26	1.62	1.47
2	B	284	ASN	C-N	-9.13	1.13	1.34
1	A	175	PRO	N-CD	9.12	1.60	1.47
2	B	293	VAL	C-N	-9.08	1.13	1.34
4	M	21	GLY	N-CA	-9.03	1.32	1.46
2	B	337	THR	N-CA	-8.98	1.28	1.46
4	M	41	LEU	CA-C	-8.95	1.29	1.52
4	M	63	TYR	C-N	-8.92	1.13	1.34
4	M	21	GLY	C-N	-8.91	1.13	1.34
3	S	4	ALA	CA-CB	8.84	1.71	1.52
1	A	467	LYS	N-CA	-8.79	1.28	1.46
3	S	67	GLU	N-CA	-8.68	1.28	1.46
4	M	61	GLU	C-N	-8.67	1.14	1.34
4	M	351	SER	C-N	-8.63	1.14	1.34
2	B	289	PRO	CA-C	-8.54	1.35	1.52
1	A	138	ASN	CA-C	-8.50	1.30	1.52
3	S	144	THR	N-CA	-8.48	1.29	1.46
4	M	349	LYS	N-CA	8.48	1.63	1.46
2	B	501	THR	N-CA	-8.41	1.29	1.46
3	S	72	ASP	N-CA	-8.40	1.29	1.46
3	S	63	ASN	CA-C	-8.39	1.31	1.52
4	M	231	SER	N-CA	8.39	1.63	1.46
2	B	342	ALA	N-CA	-8.34	1.29	1.46
2	B	581	TYR	C-N	-8.32	1.15	1.34
4	M	52	ASP	C-N	-8.31	1.15	1.34
4	M	64	LYS	CA-C	-8.29	1.31	1.52
2	B	260	LEU	N-CA	-8.26	1.29	1.46
4	M	106	LYS	C-N	-8.25	1.15	1.34
4	M	281	GLY	C-N	8.24	1.53	1.34
1	A	508	LEU	C-N	-8.21	1.18	1.34
4	M	406	GLY	N-CA	-8.20	1.33	1.46
1	A	87	CYS	N-CA	-8.20	1.29	1.46
4	M	59	ASP	N-CA	8.17	1.62	1.46
3	S	60	SER	N-CA	-8.11	1.30	1.46
1	A	513	ARG	N-CA	-8.10	1.30	1.46
3	S	167	ILE	C-N	8.10	1.47	1.33
4	M	348	LYS	C-N	8.10	1.52	1.34
1	A	477	PHE	N-CA	-8.06	1.30	1.46
3	S	83	LEU	CA-C	8.05	1.73	1.52
1	A	394	GLN	CA-C	-8.02	1.32	1.52
4	M	56	VAL	C-N	-7.99	1.18	1.33

Continued on next page...

Continued from previous page...

Mol	Chain	Res	Type	Atoms	Z	Observed(Å)	Ideal(Å)
2	B	185	LYS	N-CA	-7.99	1.30	1.46
4	M	225	VAL	CA-C	-7.98	1.32	1.52
1	A	440	ASN	N-CA	-7.97	1.30	1.46
1	A	139	ASP	N-CA	-7.93	1.30	1.46
1	A	217	ALA	C-N	7.90	1.52	1.34
4	M	138	ASP	N-CA	-7.89	1.30	1.46
2	B	212	VAL	C-O	7.88	1.38	1.23
4	M	294	ASP	N-CA	-7.88	1.30	1.46
4	M	348	LYS	CA-C	7.87	1.73	1.52
2	B	183	ALA	CA-CB	-7.84	1.35	1.52
4	M	335	SER	CA-C	-7.83	1.32	1.52
4	M	239	SER	N-CA	-7.80	1.30	1.46
4	M	352	GLN	C-N	-7.75	1.16	1.34
2	B	522	GLU	CD-OE2	-7.74	1.17	1.25
4	M	354	ASP	CA-C	7.74	1.73	1.52
1	A	616	GLU	CD-OE1	-7.72	1.17	1.25
3	S	8	PHE	C-N	-7.71	1.16	1.34
4	M	44	ASP	C-N	7.71	1.51	1.34
4	M	453	ASP	C-N	-7.67	1.16	1.34
4	M	279	ASN	C-N	-7.67	1.16	1.34
1	A	405	THR	CA-C	-7.62	1.33	1.52
4	M	136	VAL	CA-C	-7.62	1.33	1.52
1	A	406	GLY	C-N	7.54	1.51	1.34
4	M	134	PRO	C-N	-7.51	1.16	1.34
2	B	464	SER	CA-CB	-7.50	1.41	1.52
1	A	515	CYS	CA-C	-7.49	1.33	1.52
4	M	103	TYR	CA-C	7.48	1.72	1.52
3	S	62	GLU	CD-OE2	-7.46	1.17	1.25
4	M	283	PHE	N-CA	-7.46	1.31	1.46
4	M	319	SER	C-N	-7.45	1.17	1.34
3	S	55	PRO	CA-C	-7.44	1.38	1.52
3	S	140	MET	N-CA	-7.44	1.31	1.46
4	M	91	THR	CA-C	-7.42	1.33	1.52
4	M	293	PRO	CA-C	-7.42	1.38	1.52
4	M	45	SER	CA-C	7.41	1.72	1.52
2	B	466	SER	CA-CB	-7.38	1.41	1.52
1	A	218	ALA	CA-CB	-7.35	1.37	1.52
1	A	507	GLN	C-N	-7.34	1.17	1.34
1	A	534	LYS	CA-C	-7.29	1.33	1.52
4	M	62	VAL	C-N	-7.27	1.17	1.34
3	S	116	VAL	CA-C	-7.27	1.34	1.52
3	S	103	GLN	CA-C	-7.25	1.34	1.52

Continued on next page...

Continued from previous page...

Mol	Chain	Res	Type	Atoms	Z	Observed(Å)	Ideal(Å)
4	M	280	ASP	C-N	-7.22	1.20	1.33
3	S	70	ASN	C-N	-7.21	1.17	1.34
3	S	129	GLU	CG-CD	-7.18	1.41	1.51
2	B	330	SER	N-CA	-7.18	1.31	1.46
1	A	287	ALA	CA-CB	-7.17	1.37	1.52
4	M	109	LEU	N-CA	-7.15	1.32	1.46
4	M	450	GLU	C-N	-7.12	1.17	1.34
4	M	403	THR	C-N	-7.11	1.17	1.34
4	M	55	MET	C-N	7.08	1.50	1.34
4	M	230	LYS	CA-C	7.05	1.71	1.52
4	M	442	GLN	N-CA	-7.05	1.32	1.46
1	A	125	THR	N-CA	-7.03	1.32	1.46
2	B	311	TYR	CD2-CE2	-7.02	1.28	1.39
4	M	70	ASN	C-N	-7.01	1.18	1.34
1	A	136	GLY	CA-C	-7.00	1.40	1.51
4	M	136	VAL	C-N	-6.99	1.18	1.34
3	S	115	GLU	CA-C	-6.98	1.34	1.52
2	B	307	ASN	N-CA	-6.98	1.32	1.46
1	A	289	SER	C-N	-6.97	1.18	1.34
3	S	22	PRO	C-N	-6.97	1.18	1.34
3	S	55	PRO	N-CD	6.96	1.57	1.47
2	B	285	GLU	N-CA	-6.96	1.32	1.46
4	M	420	THR	N-CA	-6.94	1.32	1.46
4	M	81	SER	N-CA	-6.92	1.32	1.46
1	A	529	GLY	N-CA	-6.90	1.35	1.46
1	A	509	PRO	CA-C	-6.89	1.39	1.52
4	M	322	LEU	C-O	6.88	1.36	1.23
4	M	307	SER	CB-OG	-6.87	1.33	1.42
3	S	6	LEU	C-N	-6.84	1.18	1.34
2	B	334	MET	N-CA	6.83	1.60	1.46
2	B	329	ALA	CA-C	-6.82	1.35	1.52
4	M	441	GLY	N-CA	-6.81	1.35	1.46
4	M	441	GLY	CA-C	-6.81	1.41	1.51
3	S	22	PRO	N-CA	-6.80	1.35	1.47
4	M	64	LYS	C-N	-6.78	1.18	1.34
4	M	95	THR	N-CA	6.78	1.59	1.46
2	B	330	SER	C-N	-6.74	1.21	1.34
2	B	458	MET	C-O	6.73	1.36	1.23
3	S	120	ASP	N-CA	-6.70	1.32	1.46
4	M	392	MET	N-CA	-6.70	1.32	1.46
4	M	379	LEU	C-N	-6.68	1.18	1.34
3	S	81	ALA	CA-CB	-6.67	1.38	1.52

Continued on next page...

Continued from previous page...

Mol	Chain	Res	Type	Atoms	Z	Observed(Å)	Ideal(Å)
4	M	451	ALA	CA-CB	6.66	1.66	1.52
1	A	243	ILE	N-CA	-6.64	1.33	1.46
3	S	62	GLU	CD-OE1	-6.64	1.18	1.25
2	B	363	ILE	N-CA	-6.64	1.33	1.46
3	S	114	THR	C-N	-6.60	1.18	1.34
4	M	55	MET	N-CA	-6.60	1.33	1.46
1	A	552	ILE	CA-C	-6.60	1.35	1.52
4	M	440	ILE	C-N	-6.59	1.21	1.33
2	B	582	ASP	N-CA	-6.59	1.33	1.46
4	M	440	ILE	N-CA	-6.58	1.33	1.46
1	A	298	ILE	C-O	6.58	1.35	1.23
4	M	288	ILE	N-CA	6.57	1.59	1.46
3	S	138	GLY	C-N	6.55	1.44	1.33
2	B	382	TYR	N-CA	-6.51	1.33	1.46
4	M	105	ASP	N-CA	-6.47	1.33	1.46
1	A	288	THR	N-CA	6.47	1.59	1.46
4	M	439	TYR	CA-C	-6.46	1.36	1.52
2	B	334	MET	C-N	-6.44	1.19	1.34
4	M	25	PRO	CA-C	-6.44	1.40	1.52
1	A	378	ILE	C-N	-6.43	1.19	1.34
3	S	113	PHE	C-N	-6.43	1.19	1.34
4	M	282	VAL	CA-C	-6.43	1.36	1.52
4	M	323	MET	N-CA	-6.42	1.33	1.46
1	A	278	ILE	N-CA	-6.39	1.33	1.46
2	B	184	GLY	C-N	-6.37	1.19	1.34
3	S	108	SER	N-CA	-6.37	1.33	1.46
1	A	71	VAL	CA-C	-6.36	1.36	1.52
4	M	82	LYS	C-N	-6.35	1.19	1.34
2	B	585	GLY	N-CA	-6.35	1.36	1.46
4	M	320	ILE	N-CA	-6.34	1.33	1.46
1	A	533	ILE	N-CA	-6.33	1.33	1.46
1	A	221	VAL	N-CA	6.32	1.58	1.46
1	A	439	ASP	C-N	-6.30	1.19	1.34
4	M	377	LYS	C-N	-6.28	1.19	1.34
1	A	230	PRO	N-CD	-6.27	1.39	1.47
3	S	143	GLU	CA-C	-6.25	1.36	1.52
3	S	116	VAL	N-CA	-6.25	1.33	1.46
4	M	212	ASN	N-CA	-6.25	1.33	1.46
3	S	159	ALA	CA-CB	6.25	1.65	1.52
3	S	3	HIS	N-CA	-6.25	1.33	1.46
1	A	387	ILE	CA-C	-6.24	1.36	1.52
1	A	465	SER	N-CA	-6.24	1.33	1.46

Continued on next page...

Continued from previous page...

Mol	Chain	Res	Type	Atoms	Z	Observed(Å)	Ideal(Å)
4	M	23	THR	C-N	-6.24	1.19	1.34
4	M	97	ASP	CA-C	-6.23	1.36	1.52
1	A	535	ILE	C-N	-6.22	1.19	1.34
4	M	288	ILE	C-N	-6.20	1.19	1.34
2	B	345	ARG	CD-NE	-6.19	1.35	1.46
1	A	441	TYR	C-O	-6.19	1.11	1.23
2	B	326	TYR	N-CA	-6.17	1.34	1.46
1	A	281	LEU	N-CA	6.17	1.58	1.46
4	M	402	SER	C-N	6.16	1.48	1.34
4	M	404	ALA	N-CA	-6.15	1.34	1.46
2	B	329	ALA	N-CA	-6.11	1.34	1.46
1	A	589	SER	N-CA	-6.11	1.34	1.46
4	M	6	TYR	C-N	-6.10	1.20	1.34
3	S	60	SER	C-N	-6.09	1.20	1.34
3	S	62	GLU	C-N	-6.09	1.20	1.34
4	M	60	LEU	CA-C	-6.08	1.37	1.52
2	B	259	TYR	C-N	-6.07	1.20	1.34
3	S	48	SER	N-CA	-6.07	1.34	1.46
2	B	382	TYR	CA-C	-6.06	1.37	1.52
1	A	464	ILE	C-N	-6.06	1.20	1.34
2	B	153	ILE	CA-C	-6.05	1.37	1.52
2	B	568	VAL	N-CA	-6.04	1.34	1.46
4	M	252	ASP	C-N	-6.04	1.20	1.34
1	A	444	VAL	N-CA	-6.03	1.34	1.46
1	A	394	GLN	N-CA	-6.00	1.34	1.46
1	A	302	ASN	CA-C	-5.97	1.37	1.52
2	B	82	TYR	N-CA	-5.97	1.34	1.46
3	S	57	LEU	CA-C	-5.96	1.37	1.52
1	A	348	PHE	CA-C	-5.96	1.37	1.52
2	B	301	LEU	N-CA	-5.95	1.34	1.46
1	A	244	LEU	C-N	-5.95	1.20	1.34
3	S	144	THR	C-N	-5.94	1.20	1.34
2	B	187	ASP	CA-C	-5.93	1.37	1.52
4	M	288	ILE	CA-C	-5.93	1.37	1.52
1	A	277	LYS	C-N	-5.93	1.20	1.34
2	B	259	TYR	CA-C	-5.92	1.37	1.52
1	A	263	LEU	N-CA	-5.92	1.34	1.46
1	A	242	GLU	CA-C	-5.92	1.37	1.52
4	M	22	ALA	N-CA	-5.92	1.34	1.46
3	S	139	GLY	CA-C	-5.92	1.42	1.51
1	A	531	ASP	CA-C	-5.90	1.37	1.52
4	M	263	MET	N-CA	-5.90	1.34	1.46

Continued on next page...

Continued from previous page...

Mol	Chain	Res	Type	Atoms	Z	Observed(Å)	Ideal(Å)
2	B	222	HIS	N-CA	5.89	1.58	1.46
4	M	96	ILE	C-N	5.89	1.47	1.34
4	M	18	TYR	N-CA	-5.88	1.34	1.46
4	M	262	THR	CA-C	-5.88	1.37	1.52
2	B	293	VAL	CA-C	-5.87	1.37	1.52
1	A	566	PHE	CA-C	-5.86	1.37	1.52
2	B	543	GLU	CD-OE2	-5.86	1.19	1.25
2	B	334	MET	CA-C	-5.85	1.37	1.52
1	A	401	VAL	CA-C	-5.85	1.37	1.52
4	M	407	THR	N-CA	-5.85	1.34	1.46
2	B	184	GLY	N-CA	5.84	1.54	1.46
1	A	466	ASP	C-N	-5.83	1.20	1.34
1	A	387	ILE	N-CA	-5.83	1.34	1.46
4	M	68	VAL	N-CA	-5.83	1.34	1.46
2	B	395	LYS	CA-C	-5.82	1.37	1.52
1	A	588	LEU	C-N	-5.78	1.20	1.34
2	B	363	ILE	CA-C	-5.78	1.38	1.52
3	S	35	VAL	N-CA	-5.77	1.34	1.46
3	S	59	LEU	N-CA	-5.77	1.34	1.46
4	M	50	TYR	C-N	-5.77	1.20	1.34
1	A	418	ILE	C-N	5.75	1.47	1.34
1	A	407	SER	CB-OG	-5.75	1.34	1.42
4	M	287	ASN	CA-C	5.75	1.67	1.52
4	M	336	ASP	N-CA	-5.75	1.34	1.46
3	S	158	LYS	CA-C	-5.73	1.38	1.52
1	A	301	GLY	CA-C	-5.73	1.42	1.51
1	A	448	GLU	CD-OE1	-5.73	1.19	1.25
1	A	410	TYR	N-CA	-5.72	1.34	1.46
4	M	278	ILE	CA-C	-5.72	1.38	1.52
2	B	219	TYR	C-N	-5.71	1.21	1.34
1	A	381	GLU	CD-OE1	-5.70	1.19	1.25
2	B	567	GLN	CA-C	-5.70	1.38	1.52
2	B	150	LEU	N-CA	-5.69	1.34	1.46
2	B	326	TYR	CG-CD2	-5.67	1.31	1.39
1	A	550	VAL	CA-C	-5.67	1.38	1.52
3	S	95	GLU	CD-OE1	-5.67	1.19	1.25
2	B	522	GLU	CA-C	-5.66	1.38	1.52
4	M	457	GLY	CA-C	-5.65	1.42	1.51
3	S	47	GLN	N-CA	-5.65	1.35	1.46
2	B	83	PHE	C-N	5.65	1.47	1.34
2	B	290	SER	N-CA	-5.65	1.35	1.46
2	B	352	ASN	C-N	-5.65	1.21	1.34

Continued on next page...

Continued from previous page...

Mol	Chain	Res	Type	Atoms	Z	Observed(Å)	Ideal(Å)
2	B	576	GLN	C-N	-5.64	1.21	1.34
1	A	499	ILE	C-O	-5.64	1.12	1.23
2	B	304	GLN	CA-C	-5.64	1.38	1.52
4	M	462	LYS	C-N	-5.64	1.21	1.34
3	S	14	PRO	C-N	-5.62	1.21	1.34
1	A	293	GLU	CD-OE1	-5.61	1.19	1.25
3	S	84	TYR	C-N	-5.60	1.21	1.34
3	S	83	LEU	C-N	-5.59	1.21	1.34
1	A	216	SER	C-N	5.59	1.47	1.34
2	B	584	SER	C-N	-5.59	1.23	1.33
4	M	118	TYR	CA-C	-5.58	1.38	1.52
2	B	500	GLN	C-N	-5.58	1.21	1.34
1	A	84	MET	N-CA	-5.58	1.35	1.46
1	A	615	GLU	N-CA	-5.58	1.35	1.46
3	S	64	ASN	C-N	5.57	1.46	1.34
4	M	223	HIS	C-N	-5.56	1.21	1.34
1	A	535	ILE	N-CA	-5.55	1.35	1.46
1	A	93	GLU	CD-OE1	-5.54	1.19	1.25
1	A	464	ILE	CA-C	-5.54	1.38	1.52
3	S	80	TYR	N-CA	-5.53	1.35	1.46
4	M	24	ALA	N-CA	-5.53	1.35	1.46
3	S	83	LEU	N-CA	-5.51	1.35	1.46
2	B	515	PHE	CA-C	5.51	1.67	1.52
4	M	262	THR	N-CA	-5.50	1.35	1.46
1	A	303	MET	N-CA	-5.50	1.35	1.46
4	M	446	GLY	CA-C	5.50	1.60	1.51
3	S	5	VAL	CA-C	5.50	1.67	1.52
4	M	83	SER	N-CA	-5.49	1.35	1.46
4	M	41	LEU	C-N	5.47	1.46	1.34
1	A	623	MET	CA-C	-5.46	1.38	1.52
1	A	138	ASN	C-N	-5.46	1.21	1.34
4	M	296	LYS	CA-C	-5.46	1.38	1.52
1	A	511	VAL	N-CA	-5.45	1.35	1.46
2	B	172	GLU	CD-OE2	-5.43	1.19	1.25
1	A	634	ASN	CA-C	-5.43	1.38	1.52
4	M	128	CYS	C-O	5.42	1.33	1.23
2	B	526	CYS	N-CA	-5.41	1.35	1.46
1	A	308	ASP	N-CA	-5.41	1.35	1.46
2	B	344	VAL	CA-CB	-5.41	1.43	1.54
4	M	228	LYS	C-N	-5.40	1.21	1.34
2	B	273	SER	N-CA	-5.39	1.35	1.46
1	A	388	VAL	CA-C	-5.38	1.39	1.52

Continued on next page...

Continued from previous page...

Mol	Chain	Res	Type	Atoms	Z	Observed(Å)	Ideal(Å)
4	M	367	ALA	CA-CB	-5.38	1.41	1.52
2	B	557	SER	C-N	-5.37	1.21	1.34
3	S	119	LEU	C-N	5.37	1.46	1.34
3	S	129	GLU	CD-OE1	-5.37	1.19	1.25
1	A	147	LEU	CA-C	-5.36	1.39	1.52
1	A	392	MET	N-CA	-5.36	1.35	1.46
2	B	561	ASP	CG-OD1	-5.35	1.13	1.25
3	S	105	PHE	N-CA	-5.35	1.35	1.46
4	M	296	LYS	N-CA	5.35	1.57	1.46
1	A	406	GLY	N-CA	-5.34	1.38	1.46
4	M	78	ALA	C-N	-5.33	1.21	1.34
4	M	71	LYS	N-CA	-5.33	1.35	1.46
2	B	381	PHE	CE2-CZ	-5.33	1.27	1.37
4	M	253	ASN	N-CA	-5.33	1.35	1.46
1	A	134	TYR	CB-CG	-5.32	1.43	1.51
3	S	70	ASN	CA-C	-5.32	1.39	1.52
4	M	257	ALA	N-CA	-5.32	1.35	1.46
2	B	276	SER	N-CA	-5.32	1.35	1.46
2	B	485	LYS	CA-C	-5.31	1.39	1.52
4	M	61	GLU	N-CA	-5.30	1.35	1.46
2	B	380	LYS	C-O	-5.30	1.13	1.23
4	M	65	TYR	C-N	-5.30	1.21	1.34
4	M	387	GLU	CD-OE1	-5.30	1.19	1.25
2	B	256	CYS	N-CA	-5.29	1.35	1.46
2	B	381	PHE	C-N	-5.28	1.21	1.34
1	A	633	PHE	CA-C	-5.28	1.39	1.52
2	B	289	PRO	C-N	-5.27	1.22	1.34
4	M	354	ASP	C-N	-5.27	1.22	1.34
1	A	489	GLU	CA-C	-5.27	1.39	1.52
1	A	435	ILE	N-CA	-5.26	1.35	1.46
1	A	445	ASN	CA-C	-5.26	1.39	1.52
4	M	113	LYS	CA-C	-5.26	1.39	1.52
1	A	534	LYS	N-CA	-5.25	1.35	1.46
3	S	40	SER	CA-CB	-5.22	1.45	1.52
1	A	399	ASP	N-CA	-5.21	1.35	1.46
3	S	161	GLU	CD-OE2	-5.21	1.20	1.25
2	B	580	TYR	CA-C	-5.21	1.39	1.52
4	M	280	ASP	CA-C	-5.21	1.39	1.52
1	A	192	TYR	CA-C	-5.20	1.39	1.52
4	M	284	SER	C-N	-5.20	1.24	1.34
2	B	35	TYR	CA-C	-5.19	1.39	1.52
4	M	426	LYS	C-N	-5.19	1.22	1.34

Continued on next page...

Continued from previous page...

Mol	Chain	Res	Type	Atoms	Z	Observed(Å)	Ideal(Å)
4	M	130	GLU	CA-C	5.18	1.66	1.52
4	M	75	TRP	C-N	-5.18	1.22	1.34
2	B	443	SER	CA-C	-5.17	1.39	1.52
2	B	290	SER	CA-C	-5.17	1.39	1.52
1	A	403	LEU	C-O	-5.16	1.13	1.23
2	B	579	PRO	N-CD	-5.15	1.40	1.47
1	A	522	PHE	CA-C	-5.14	1.39	1.52
2	B	424	PHE	N-CA	-5.13	1.36	1.46
4	M	51	LEU	N-CA	5.12	1.56	1.46
1	A	281	LEU	C-N	-5.12	1.22	1.34
1	A	302	ASN	N-CA	-5.10	1.36	1.46
4	M	126	ASN	CA-C	-5.10	1.39	1.52
2	B	75	ASP	C-N	-5.09	1.22	1.34
4	M	250	LEU	N-CA	-5.09	1.36	1.46
3	S	67	GLU	CA-C	-5.09	1.39	1.52
4	M	129	VAL	C-O	-5.08	1.13	1.23
1	A	388	VAL	N-CA	-5.08	1.36	1.46
1	A	630	PRO	N-CD	-5.08	1.40	1.47
3	S	143	GLU	C-N	-5.07	1.22	1.34
3	S	2	ILE	CA-C	-5.07	1.39	1.52
1	A	111	SER	CA-CB	-5.06	1.45	1.52
1	A	225	LEU	N-CA	-5.05	1.36	1.46
4	M	253	ASN	C-N	-5.05	1.24	1.34
2	B	582	ASP	C-N	-5.05	1.22	1.34
2	B	422	ALA	CA-C	-5.05	1.39	1.52
2	B	540	GLU	CD-OE1	-5.05	1.20	1.25
1	A	304	LEU	N-CA	-5.04	1.36	1.46
2	B	325	LEU	C-N	-5.03	1.22	1.34
1	A	120	ILE	CA-C	-5.03	1.39	1.52
2	B	109	ALA	CA-CB	-5.03	1.41	1.52
2	B	407	ASN	N-CA	-5.02	1.36	1.46
2	B	574	ASN	N-CA	-5.02	1.36	1.46
2	B	405	GLU	CD-OE2	-5.02	1.20	1.25
2	B	331	PRO	N-CA	-5.02	1.38	1.47
2	B	272	GLY	CA-C	-5.01	1.43	1.51
4	M	477	GLY	N-CA	-5.01	1.38	1.46
1	A	567	GLN	CA-C	-5.01	1.40	1.52
4	M	66	PHE	C-N	-5.00	1.22	1.34

All (1662) bond angle outliers are listed below:

Continued on next page...

Continued from previous page...

Mol	Chain	Res	Type	Atoms	Z	Observed(°)	Ideal(°)
4	M	404	ALA	CB-CA-C	24.95	147.53	110.10
3	S	69	ASN	N-CA-C	23.29	173.89	111.00
1	A	242	GLU	C-N-CA	22.94	179.05	121.70
4	M	130	GLU	N-CA-C	-19.38	58.67	111.00
1	A	265	GLN	N-CA-C	-19.25	59.04	111.00
4	M	404	ALA	N-CA-CB	-18.91	83.63	110.10
3	S	69	ASN	CA-C-N	-18.82	75.79	117.20
3	S	69	ASN	O-C-N	18.81	152.80	122.70
1	A	80	TYR	N-CA-C	-18.32	61.54	111.00
3	S	69	ASN	CA-C-O	-17.85	82.61	120.10
3	S	58	LEU	N-CA-C	-17.79	62.98	111.00
4	M	45	SER	C-N-CA	-17.35	78.33	121.70
4	M	82	LYS	C-N-CA	16.95	164.08	121.70
4	M	103	TYR	N-CA-C	-16.88	65.42	111.00
3	S	69	ASN	CB-CA-C	-16.71	76.99	110.40
1	A	277	LYS	C-N-CA	16.59	163.18	121.70
4	M	354	ASP	N-CA-C	-16.56	66.30	111.00
4	M	354	ASP	C-N-CA	16.38	162.66	121.70
1	A	444	VAL	N-CA-C	-16.20	67.25	111.00
1	A	439	ASP	N-CA-C	-16.11	67.51	111.00
4	M	403	THR	C-N-CA	16.03	161.77	121.70
4	M	373	ALA	CB-CA-C	15.54	133.40	110.10
2	B	580	TYR	C-N-CA	-15.41	83.18	121.70
1	A	98	ASN	C-N-CA	15.19	159.67	121.70
1	A	534	LYS	CA-C-N	15.18	150.60	117.20
4	M	280	ASP	N-CA-C	15.14	151.89	111.00
2	B	584	SER	N-CA-C	15.04	151.61	111.00
2	B	275	ARG	N-CA-C	-14.98	70.56	111.00
1	A	304	LEU	C-N-CA	-14.94	84.35	121.70
4	M	21	GLY	C-N-CA	14.89	158.91	121.70
4	M	103	TYR	O-C-N	-14.66	99.24	122.70
2	B	263	PRO	C-N-CA	14.64	158.29	121.70
2	B	290	SER	C-N-CA	-14.63	85.12	121.70
4	M	131	ALA	CA-C-N	-14.62	86.95	116.20
1	A	534	LYS	C-N-CA	14.54	158.05	121.70
4	M	135	ASN	N-CA-C	-14.31	72.36	111.00
4	M	294	ASP	N-CA-C	-13.97	73.28	111.00
1	A	543	TYR	CB-CG-CD2	13.89	129.34	121.00
4	M	103	TYR	C-N-CA	13.82	156.26	121.70
1	A	418	ILE	C-N-CA	-13.80	87.20	121.70
2	B	600	LYS	C-N-CA	-13.41	88.18	121.70

Continued on next page...

Continued from previous page...

Mol	Chain	Res	Type	Atoms	Z	Observed(°)	Ideal(°)
1	A	532	LEU	C-N-CA	-13.36	88.30	121.70
2	B	579	PRO	C-N-CA	-13.34	88.35	121.70
4	M	80	THR	N-CA-C	13.27	146.83	111.00
2	B	78	ASP	C-N-CA	-13.22	88.65	121.70
1	A	98	ASN	N-CA-C	-13.12	75.59	111.00
1	A	80	TYR	O-C-N	-13.10	100.93	123.20
1	A	405	THR	C-N-CA	-12.96	95.09	122.30
4	M	293	PRO	CA-N-CD	-12.91	93.42	111.50
4	M	59	ASP	C-N-CA	-12.82	89.65	121.70
1	A	536	MET	O-C-N	-12.76	102.29	122.70
1	A	302	ASN	N-CA-C	-12.70	76.72	111.00
1	A	64	LEU	O-C-N	-12.69	102.39	122.70
4	M	51	LEU	N-CA-C	12.65	145.15	111.00
4	M	130	GLU	C-N-CA	12.64	153.30	121.70
1	A	464	ILE	C-N-CA	-12.63	90.11	121.70
1	A	260	PHE	O-C-N	-12.55	102.63	122.70
3	S	46	PHE	C-N-CA	-12.54	90.34	121.70
3	S	168	GLY	N-CA-C	12.40	144.09	113.10
1	A	151	SER	C-N-CA	-12.35	90.82	121.70
4	M	133	GLU	C-N-CD	12.31	154.26	128.40
4	M	405	THR	N-CA-C	12.24	144.04	111.00
2	B	581	TYR	C-N-CA	12.23	152.28	121.70
4	M	61	GLU	N-CA-C	-12.17	78.15	111.00
4	M	54	SER	N-CA-C	12.15	143.81	111.00
1	A	469	LEU	C-N-CA	-12.11	96.88	122.30
4	M	82	LYS	N-CA-C	-12.03	78.53	111.00
4	M	132	GLY	CA-C-N	-12.02	90.77	117.20
4	M	351	SER	N-CA-C	11.96	143.28	111.00
4	M	22	ALA	CB-CA-C	11.92	127.97	110.10
4	M	50	TYR	N-CA-C	11.87	143.05	111.00
1	A	289	SER	O-C-N	-11.78	103.85	122.70
1	A	135	ASP	C-N-CA	-11.74	97.64	122.30
2	B	330	SER	N-CA-C	-11.74	79.30	111.00
1	A	323	CYS	C-N-CA	11.69	150.91	121.70
1	A	233	PHE	C-N-CA	-11.63	92.62	121.70
2	B	336	ASN	C-N-CA	11.61	150.72	121.70
2	B	185	LYS	C-N-CA	-11.61	92.69	121.70
1	A	265	GLN	CA-C-N	11.48	142.45	117.20
2	B	286	ILE	N-CA-C	-11.42	80.16	111.00
1	A	536	MET	C-N-CA	-11.38	93.24	121.70
1	A	136	GLY	C-N-CA	-11.36	93.30	121.70
3	S	53	THR	C-N-CD	11.34	152.22	128.40

Continued on next page...

Continued from previous page...

Mol	Chain	Res	Type	Atoms	Z	Observed(°)	Ideal(°)
2	B	329	ALA	CB-CA-C	-11.32	93.11	110.10
4	M	458	LEU	O-C-N	11.27	140.74	122.70
4	M	23	THR	N-CA-C	-11.26	80.61	111.00
2	B	404	ASN	C-N-CA	-11.21	93.68	121.70
4	M	462	LYS	N-CA-C	-11.19	80.79	111.00
4	M	52	ASP	N-CA-C	11.15	141.11	111.00
3	S	63	ASN	N-CA-C	-11.10	81.03	111.00
2	B	260	LEU	N-CA-C	-11.08	81.07	111.00
1	A	242	GLU	CA-C-N	11.08	141.57	117.20
3	S	54	PRO	CA-N-CD	-11.04	96.05	111.50
2	B	310	ILE	C-N-CA	-10.90	94.44	121.70
1	A	204	VAL	O-C-N	-10.84	105.36	122.70
3	S	109	LEU	O-C-N	-10.76	105.48	122.70
1	A	80	TYR	CA-C-N	10.73	137.66	116.20
1	A	416	ILE	C-N-CD	10.73	150.93	128.40
3	S	43	ASN	O-C-N	-10.70	105.58	122.70
1	A	287	ALA	C-N-CA	10.70	148.45	121.70
4	M	46	SER	C-N-CA	-10.70	94.94	121.70
3	S	163	THR	C-N-CA	10.61	148.23	121.70
1	A	84	MET	C-N-CA	-10.50	95.44	121.70
2	B	428	VAL	O-C-N	10.47	139.46	122.70
1	A	586	GLU	C-N-CA	-10.47	95.52	121.70
2	B	497	LEU	O-C-N	-10.44	106.00	122.70
2	B	287	GLU	C-N-CA	10.39	147.68	121.70
2	B	82	TYR	C-N-CA	-10.38	95.74	121.70
2	B	292	GLU	C-N-CA	10.38	147.64	121.70
4	M	80	THR	C-N-CA	10.37	147.62	121.70
3	S	164	ASP	C-N-CA	-10.32	95.89	121.70
4	M	279	ASN	N-CA-C	10.29	138.79	111.00
1	A	621	LEU	C-N-CD	10.29	150.01	128.40
2	B	335	LYS	N-CA-C	-10.29	83.22	111.00
2	B	584	SER	C-N-CA	-10.29	100.70	122.30
1	A	629	LEU	C-N-CD	10.27	149.97	128.40
1	A	519	LEU	C-N-CA	-10.27	100.73	122.30
1	A	543	TYR	CB-CG-CD1	-10.21	114.88	121.00
1	A	534	LYS	CA-C-O	-10.14	98.81	120.10
1	A	244	LEU	O-C-N	-10.12	106.50	122.70
2	B	577	ASN	C-N-CD	10.12	149.66	128.40
2	B	571	SER	N-CA-C	-10.10	83.73	111.00
2	B	162	VAL	C-N-CA	-10.03	96.63	121.70
2	B	289	PRO	C-N-CA	-10.01	96.67	121.70
4	M	402	SER	C-N-CA	-9.99	96.72	121.70

Continued on next page...

Continued from previous page...

Mol	Chain	Res	Type	Atoms	Z	Observed(°)	Ideal(°)
1	A	504	ILE	O-C-N	-9.99	106.72	122.70
1	A	534	LYS	N-CA-C	-9.98	84.04	111.00
2	B	262	LYS	C-N-CD	9.94	149.28	128.40
1	A	586	GLU	O-C-N	-9.90	106.85	122.70
4	M	83	SER	C-N-CA	9.90	146.46	121.70
1	A	569	ASP	C-N-CA	-9.89	96.97	121.70
3	S	57	LEU	CA-C-N	-9.89	95.44	117.20
2	B	584	SER	N-CA-CB	-9.80	95.80	110.50
1	A	380	ASP	N-CA-C	-9.79	84.58	111.00
1	A	440	ASN	C-N-CA	9.78	146.15	121.70
2	B	576	GLN	N-CA-C	-9.78	84.60	111.00
2	B	570	GLY	N-CA-C	-9.75	88.72	113.10
1	A	320	HIS	O-C-N	-9.75	107.11	122.70
4	M	268	GLY	C-N-CA	-9.75	97.34	121.70
2	B	187	ASP	C-N-CA	-9.72	97.40	121.70
4	M	306	LEU	O-C-N	-9.70	107.18	122.70
1	A	84	MET	O-C-N	-9.69	107.19	122.70
1	A	282	MET	O-C-N	-9.64	107.27	122.70
4	M	3	LEU	O-C-N	-9.64	107.27	122.70
4	M	57	GLY	C-N-CA	9.63	145.77	121.70
1	A	346	THR	C-N-CA	-9.60	97.70	121.70
1	A	462	GLN	O-C-N	-9.60	107.34	122.70
3	S	21	THR	C-N-CD	9.60	148.56	128.40
1	A	365	VAL	C-N-CA	-9.58	97.76	121.70
1	A	212	ILE	C-N-CA	-9.53	97.88	121.70
3	S	68	VAL	N-CA-C	9.52	136.72	111.00
1	A	403	LEU	C-N-CA	-9.52	97.90	121.70
4	M	279	ASN	C-N-CA	9.50	145.44	121.70
2	B	325	LEU	O-C-N	-9.46	107.56	122.70
2	B	230	PHE	C-N-CA	-9.46	98.06	121.70
3	S	6	LEU	O-C-N	-9.46	107.57	122.70
1	A	80	TYR	C-N-CA	9.45	142.13	122.30
1	A	465	SER	C-N-CA	9.44	145.29	121.70
2	B	212	VAL	CA-C-O	9.39	139.82	120.10
1	A	569	ASP	N-CA-C	-9.39	85.65	111.00
1	A	319	LEU	O-C-N	-9.39	107.68	122.70
2	B	577	ASN	N-CA-C	9.38	136.34	111.00
4	M	379	LEU	O-C-N	-9.38	107.69	122.70
4	M	134	PRO	N-CA-C	-9.38	87.72	112.10
3	S	116	VAL	CA-C-N	-9.37	96.60	117.20
1	A	465	SER	CA-C-N	-9.36	96.61	117.20
1	A	305	GLU	N-CA-C	9.35	136.24	111.00

Continued on next page...

Continued from previous page...

Mol	Chain	Res	Type	Atoms	Z	Observed(°)	Ideal(°)
2	B	535	GLN	C-N-CA	-9.34	98.34	121.70
4	M	6	TYR	O-C-N	-9.32	107.78	122.70
3	S	58	LEU	CA-C-N	-9.32	96.70	117.20
1	A	381	GLU	C-N-CA	-9.32	98.41	121.70
2	B	102	HIS	O-C-N	-9.30	107.82	122.70
1	A	242	GLU	N-CA-C	-9.29	85.93	111.00
4	M	406	GLY	N-CA-C	-9.27	89.92	113.10
2	B	366	LEU	O-C-N	-9.26	107.89	122.70
3	S	143	GLU	N-CA-C	-9.26	86.01	111.00
4	M	136	VAL	CA-C-N	-9.25	96.85	117.20
1	A	275	LEU	C-N-CD	9.25	147.82	128.40
4	M	252	ASP	N-CA-C	-9.20	86.16	111.00
1	A	240	LEU	C-N-CA	-9.19	98.72	121.70
2	B	505	ASP	C-N-CA	-9.15	98.82	121.70
2	B	580	TYR	N-CA-C	-9.14	86.31	111.00
2	B	461	HIS	C-N-CA	-9.14	98.85	121.70
4	M	59	ASP	CA-C-N	9.13	137.28	117.20
2	B	560	ILE	C-N-CA	-9.09	98.98	121.70
1	A	504	ILE	C-N-CA	-9.09	98.98	121.70
4	M	41	LEU	O-C-N	9.09	137.24	122.70
2	B	211	ALA	O-C-N	9.07	137.21	122.70
1	A	163	ALA	C-N-CA	-9.06	99.04	121.70
2	B	497	LEU	C-N-CA	-9.05	99.06	121.70
1	A	100	LEU	C-N-CA	-9.05	99.07	121.70
2	B	223	LEU	C-N-CA	-9.05	99.08	121.70
2	B	205	PRO	C-N-CA	-9.04	99.09	121.70
2	B	568	VAL	CA-C-N	-9.03	97.33	117.20
1	A	350	SER	O-C-N	-8.99	108.31	122.70
3	S	100	ASP	O-C-N	8.98	137.06	122.70
1	A	86	TRP	C-N-CA	-8.97	99.26	121.70
4	M	92	PHE	O-C-N	8.97	137.06	122.70
3	S	161	GLU	O-C-N	-8.96	108.36	122.70
2	B	569	THR	N-CA-C	8.95	135.17	111.00
1	A	103	LYS	O-C-N	-8.94	108.39	122.70
1	A	288	THR	N-CA-C	8.93	135.10	111.00
2	B	559	ASP	CA-C-O	-8.93	101.35	120.10
4	M	477	GLY	C-N-CA	-8.90	99.45	121.70
4	M	351	SER	C-N-CA	8.88	143.91	121.70
1	A	196	LEU	C-N-CA	-8.88	99.49	121.70
2	B	472	VAL	O-C-N	-8.88	108.49	122.70
1	A	384	LEU	O-C-N	8.88	136.90	122.70
4	M	63	TYR	C-N-CA	-8.84	99.59	121.70

Continued on next page...

Continued from previous page...

Mol	Chain	Res	Type	Atoms	Z	Observed(°)	Ideal(°)
1	A	529	GLY	CA-C-O	-8.83	104.70	120.60
2	B	287	GLU	N-CA-C	-8.83	87.17	111.00
4	M	99	ILE	O-C-N	8.81	136.80	122.70
3	S	5	VAL	O-C-N	-8.80	108.62	122.70
1	A	461	CYS	O-C-N	-8.80	108.62	122.70
2	B	404	ASN	O-C-N	-8.80	108.62	122.70
3	S	50	PHE	C-N-CA	-8.79	99.74	121.70
4	M	405	THR	CA-C-N	-8.77	98.65	116.20
1	A	432	ILE	O-C-N	8.75	136.71	122.70
2	B	212	VAL	O-C-N	-8.74	108.72	122.70
1	A	138	ASN	N-CA-C	-8.74	87.41	111.00
1	A	110	ALA	C-N-CA	-8.72	99.89	121.70
1	A	154	ILE	N-CA-C	8.71	134.52	111.00
2	B	523	PHE	O-C-N	-8.71	108.76	122.70
3	S	104	THR	O-C-N	8.70	136.62	122.70
1	A	325	SER	N-CA-C	-8.68	87.56	111.00
4	M	335	SER	O-C-N	8.67	136.57	122.70
1	A	88	ASN	C-N-CA	-8.66	100.04	121.70
4	M	279	ASN	CA-C-N	8.66	136.24	117.20
1	A	233	PHE	O-C-N	-8.65	108.86	122.70
4	M	347	PHE	N-CA-C	-8.64	87.67	111.00
2	B	147	MET	O-C-N	-8.62	108.91	122.70
1	A	539	ASN	O-C-N	-8.61	108.92	122.70
4	M	420	THR	C-N-CA	-8.60	104.24	122.30
3	S	8	PHE	O-C-N	-8.58	108.97	122.70
3	S	81	ALA	CB-CA-C	8.58	122.97	110.10
1	A	545	HIS	C-N-CA	-8.58	100.25	121.70
2	B	109	ALA	C-N-CA	-8.56	100.31	121.70
4	M	406	GLY	C-N-CA	-8.55	100.31	121.70
1	A	413	SER	N-CA-C	-8.54	87.93	111.00
3	S	167	ILE	C-N-CA	-8.54	104.36	122.30
1	A	94	VAL	O-C-N	-8.54	109.04	122.70
1	A	461	CYS	CA-C-O	8.50	137.94	120.10
3	S	98	ILE	O-C-N	8.49	136.29	122.70
3	S	37	GLU	O-C-N	8.47	136.26	122.70
4	M	126	ASN	O-C-N	8.46	136.24	122.70
1	A	212	ILE	O-C-N	-8.44	109.19	122.70
1	A	328	PRO	C-N-CA	-8.43	100.63	121.70
2	B	574	ASN	C-N-CA	-8.42	100.66	121.70
2	B	205	PRO	O-C-N	-8.39	109.27	122.70
1	A	504	ILE	CA-C-O	8.38	137.71	120.10
4	M	292	PRO	C-N-CA	8.37	157.16	122.00

Continued on next page...

Continued from previous page...

Mol	Chain	Res	Type	Atoms	Z	Observed(°)	Ideal(°)
1	A	547	VAL	O-C-N	8.37	136.09	122.70
1	A	242	GLU	O-C-N	-8.35	109.34	122.70
1	A	545	HIS	O-C-N	-8.35	109.34	122.70
1	A	391	LEU	C-N-CA	-8.33	100.87	121.70
2	B	146	LYS	C-N-CA	-8.31	100.93	121.70
1	A	431	VAL	O-C-N	8.30	135.99	122.70
1	A	281	LEU	CA-C-O	8.29	137.51	120.10
1	A	573	GLU	O-C-N	-8.29	109.44	122.70
4	M	53	HIS	CA-C-N	8.29	135.44	117.20
4	M	367	ALA	C-N-CA	8.29	142.42	121.70
1	A	637	GLU	C-N-CA	-8.28	101.00	121.70
2	B	444	THR	O-C-N	-8.28	109.46	122.70
1	A	275	LEU	C-N-CA	-8.26	87.31	122.00
2	B	366	LEU	C-N-CA	-8.26	101.06	121.70
2	B	505	ASP	O-C-N	-8.25	109.49	122.70
1	A	631	SER	O-C-N	8.25	135.90	122.70
2	B	339	PHE	O-C-N	8.24	135.89	122.70
1	A	215	VAL	CA-C-O	8.24	137.40	120.10
3	S	59	LEU	CA-C-N	-8.24	99.08	117.20
2	B	83	PHE	O-C-N	8.22	135.85	122.70
4	M	54	SER	O-C-N	8.22	135.85	122.70
1	A	100	LEU	O-C-N	-8.22	109.55	122.70
2	B	295	ASN	C-N-CA	8.22	142.24	121.70
2	B	132	SER	C-N-CA	-8.20	101.21	121.70
4	M	457	GLY	N-CA-C	-8.20	92.61	113.10
2	B	375	LEU	O-C-N	-8.19	105.53	121.10
2	B	526	CYS	O-C-N	8.19	136.67	121.10
3	S	66	ASP	N-CA-C	-8.19	88.88	111.00
4	M	105	ASP	C-N-CA	-8.19	101.22	121.70
4	M	74	TYR	CA-C-O	8.19	137.30	120.10
1	A	192	TYR	CA-C-O	-8.18	102.91	120.10
4	M	104	PHE	O-C-N	-8.18	109.61	122.70
1	A	346	THR	O-C-N	-8.18	109.61	122.70
1	A	120	ILE	O-C-N	8.18	135.78	122.70
3	S	158	LYS	O-C-N	8.17	135.77	122.70
4	M	104	PHE	C-N-CA	8.17	142.12	121.70
1	A	443	SER	N-CA-C	8.17	133.05	111.00
2	B	184	GLY	CA-C-N	-8.16	99.24	117.20
1	A	424	TYR	O-C-N	8.16	135.76	122.70
2	B	108	PHE	CA-C-O	8.16	137.23	120.10
4	M	330	GLY	C-N-CA	8.16	142.09	121.70
2	B	56	SER	O-C-N	-8.15	109.67	122.70

Continued on next page...

Continued from previous page...

Mol	Chain	Res	Type	Atoms	Z	Observed(°)	Ideal(°)
4	M	51	LEU	C-N-CA	-8.15	101.33	121.70
1	A	304	LEU	O-C-N	-8.14	109.68	122.70
2	B	108	PHE	O-C-N	-8.13	109.69	122.70
2	B	296	ASP	CA-C-O	-8.13	103.03	120.10
2	B	30	LEU	C-N-CA	8.12	139.36	122.30
1	A	487	MET	O-C-N	-8.11	109.72	122.70
2	B	105	LEU	O-C-N	-8.11	109.72	122.70
1	A	450	TYR	O-C-N	8.11	135.67	122.70
1	A	330	LEU	O-C-N	8.10	135.66	122.70
4	M	63	TYR	CD1-CG-CD2	8.10	126.81	117.90
2	B	486	HIS	O-C-N	8.10	135.65	122.70
3	S	108	SER	O-C-N	8.10	135.65	122.70
2	B	557	SER	O-C-N	-8.09	109.75	122.70
2	B	35	TYR	O-C-N	8.08	135.63	122.70
1	A	270	LEU	O-C-N	8.08	135.63	122.70
1	A	549	GLU	O-C-N	8.08	135.63	122.70
4	M	81	SER	O-C-N	-8.06	109.80	122.70
1	A	367	ILE	O-C-N	8.06	135.60	122.70
1	A	465	SER	N-CA-C	8.06	132.77	111.00
2	B	273	SER	N-CA-C	8.06	132.76	111.00
1	A	415	ARG	N-CA-C	-8.06	89.25	111.00
4	M	232	HIS	CA-C-O	8.05	137.01	120.10
1	A	103	LYS	CA-C-O	8.05	137.01	120.10
2	B	336	ASN	CA-C-N	-8.05	99.49	117.20
3	S	46	PHE	CA-C-O	-8.05	103.20	120.10
3	S	125	TRP	CA-C-O	-8.04	103.21	120.10
4	M	135	ASN	CA-C-N	-8.04	99.51	117.20
3	S	103	GLN	O-C-N	8.03	135.56	122.70
4	M	63	TYR	CB-CG-CD2	-8.03	116.18	121.00
2	B	317	VAL	O-C-N	8.03	135.54	122.70
1	A	218	ALA	CB-CA-C	8.02	122.12	110.10
4	M	108	LYS	C-N-CA	-8.01	101.69	121.70
3	S	16	LEU	O-C-N	-8.00	109.89	122.70
2	B	274	PRO	C-N-CA	-8.00	101.70	121.70
2	B	494	ALA	O-C-N	8.00	135.50	122.70
1	A	442	SER	N-CA-C	-7.98	89.44	111.00
2	B	240	LEU	N-CA-C	7.98	132.53	111.00
2	B	515	PHE	C-N-CA	-7.97	105.57	122.30
3	S	46	PHE	N-CA-C	-7.96	89.51	111.00
3	S	139	GLY	C-N-CA	-7.96	101.81	121.70
4	M	294	ASP	C-N-CA	7.95	138.99	122.30
4	M	5	PHE	O-C-N	-7.95	109.99	122.70

Continued on next page...

Continued from previous page...

Mol	Chain	Res	Type	Atoms	Z	Observed(°)	Ideal(°)
2	B	126	SER	O-C-N	-7.94	110.00	122.70
2	B	299	LEU	O-C-N	7.94	135.40	122.70
3	S	74	GLN	O-C-N	-7.93	110.00	122.70
1	A	328	PRO	O-C-N	-7.93	110.01	122.70
2	B	174	ALA	O-C-N	-7.93	110.01	122.70
1	A	158	LEU	O-C-N	7.92	135.38	122.70
4	M	262	THR	C-N-CA	-7.92	101.89	121.70
4	M	55	MET	CA-C-N	7.92	134.62	117.20
3	S	113	PHE	N-CA-C	-7.92	89.63	111.00
1	A	264	SER	C-N-CA	-7.91	101.92	121.70
2	B	389	ILE	O-C-N	-7.91	110.05	122.70
2	B	508	ARG	O-C-N	-7.91	110.05	122.70
1	A	281	LEU	C-N-CA	-7.90	101.96	121.70
2	B	584	SER	O-C-N	-7.89	109.78	123.20
1	A	479	ASN	O-C-N	7.89	135.32	122.70
2	B	132	SER	O-C-N	-7.89	110.08	122.70
2	B	293	VAL	C-N-CA	-7.88	102.00	121.70
2	B	566	ALA	C-N-CA	7.87	141.38	121.70
1	A	601	VAL	O-C-N	-7.87	110.11	122.70
1	A	364	ASP	O-C-N	7.86	135.28	122.70
4	M	292	PRO	N-CA-C	-7.86	91.66	112.10
4	M	263	MET	C-N-CA	7.86	138.80	122.30
1	A	588	LEU	O-C-N	-7.84	110.15	122.70
2	B	147	MET	C-N-CA	-7.84	102.11	121.70
1	A	70	ALA	O-C-N	7.83	135.23	122.70
1	A	612	GLU	O-C-N	7.83	135.22	122.70
3	S	25	LEU	O-C-N	-7.82	106.23	121.10
3	S	36	TYR	O-C-N	7.82	135.21	122.70
1	A	434	SER	O-C-N	7.81	135.20	122.70
2	B	416	LYS	O-C-N	7.81	135.19	122.70
1	A	156	PRO	O-C-N	-7.80	110.22	122.70
4	M	75	TRP	O-C-N	-7.80	110.22	122.70
1	A	448	GLU	N-CA-C	-7.80	89.95	111.00
4	M	230	LYS	CA-C-N	7.79	134.33	117.20
2	B	237	ILE	O-C-N	-7.78	110.25	122.70
2	B	142	LEU	O-C-N	-7.78	110.26	122.70
2	B	220	ALA	N-CA-C	-7.75	90.08	111.00
2	B	418	TYR	C-N-CA	-7.75	102.33	121.70
3	S	66	ASP	CA-C-N	-7.75	100.16	117.20
2	B	115	LEU	O-C-N	7.74	135.09	122.70
2	B	287	GLU	CA-C-N	-7.74	100.17	117.20
1	A	461	CYS	C-N-CA	-7.74	102.36	121.70

Continued on next page...

Continued from previous page...

Mol	Chain	Res	Type	Atoms	Z	Observed(°)	Ideal(°)
4	M	97	ASP	O-C-N	7.74	135.08	122.70
1	A	279	LEU	O-C-N	-7.73	110.33	122.70
3	S	62	GLU	N-CA-C	7.71	131.81	111.00
4	M	265	ASN	N-CA-C	-7.68	90.25	111.00
3	S	28	GLN	O-C-N	7.68	134.99	122.70
1	A	508	LEU	CA-C-O	-7.67	103.98	120.10
3	S	60	SER	N-CA-C	-7.66	90.31	111.00
2	B	478	LEU	O-C-N	-7.66	110.44	122.70
1	A	298	ILE	O-C-N	-7.65	110.46	122.70
1	A	469	LEU	CA-C-O	7.65	136.17	120.10
2	B	585	GLY	N-CA-C	-7.64	93.99	113.10
4	M	72	LEU	C-N-CA	7.64	140.81	121.70
1	A	534	LYS	O-C-N	-7.64	110.48	122.70
1	A	281	LEU	O-C-N	-7.64	110.48	122.70
1	A	394	GLN	CA-C-O	-7.63	104.07	120.10
2	B	169	VAL	O-C-N	7.62	134.89	122.70
3	S	31	LEU	O-C-N	7.62	134.88	122.70
2	B	515	PHE	O-C-N	-7.61	110.26	123.20
1	A	527	GLU	C-N-CA	7.60	140.70	121.70
3	S	132	LEU	O-C-N	7.60	134.86	122.70
4	M	104	PHE	N-CA-CB	-7.60	96.92	110.60
2	B	528	ASP	O-C-N	7.60	134.86	122.70
3	S	58	LEU	C-N-CA	-7.59	102.72	121.70
1	A	220	SER	O-C-N	7.58	134.83	122.70
4	M	421	GLY	O-C-N	-7.58	106.69	121.10
2	B	51	LEU	C-N-CA	-7.58	102.76	121.70
4	M	280	ASP	CB-CA-C	-7.57	95.27	110.40
3	S	58	LEU	CA-C-O	7.56	135.98	120.10
1	A	596	VAL	O-C-N	7.54	134.76	122.70
3	S	27	LYS	O-C-N	7.54	134.76	122.70
1	A	218	ALA	O-C-N	7.54	134.76	122.70
4	M	367	ALA	CB-CA-C	7.53	121.39	110.10
4	M	450	GLU	CA-C-O	7.53	135.90	120.10
2	B	252	LEU	O-C-N	7.52	134.73	122.70
2	B	560	ILE	O-C-N	-7.52	110.67	122.70
3	S	153	VAL	O-C-N	7.51	134.72	122.70
3	S	109	LEU	CA-C-O	7.50	135.84	120.10
2	B	408	VAL	O-C-N	7.49	134.68	122.70
4	M	237	THR	C-N-CA	-7.49	106.57	122.30
1	A	365	VAL	O-C-N	-7.49	110.72	122.70
4	M	279	ASN	N-CA-CB	-7.48	97.14	110.60
4	M	105	ASP	O-C-N	-7.48	110.74	122.70

Continued on next page...

Continued from previous page...

Mol	Chain	Res	Type	Atoms	Z	Observed(°)	Ideal(°)
4	M	351	SER	N-CA-CB	-7.47	99.29	110.50
2	B	442	LEU	C-N-CA	-7.46	103.05	121.70
1	A	528	ASN	CA-C-O	7.46	135.76	120.10
1	A	441	TYR	CA-C-N	-7.44	100.83	117.20
1	A	98	ASN	CA-C-N	7.44	133.57	117.20
2	B	554	LYS	O-C-N	7.43	134.59	122.70
2	B	237	ILE	C-N-CA	-7.42	103.14	121.70
2	B	215	TYR	O-C-N	7.42	134.57	122.70
2	B	46	GLN	O-C-N	7.42	134.57	122.70
2	B	328	LEU	C-N-CA	-7.42	103.15	121.70
1	A	601	VAL	C-N-CA	-7.41	103.18	121.70
2	B	277	CYS	C-N-CD	7.41	143.95	128.40
3	S	25	LEU	CA-C-O	7.40	135.63	120.10
1	A	88	ASN	CA-C-O	7.39	135.62	120.10
2	B	220	ALA	CB-CA-C	7.39	121.19	110.10
1	A	311	THR	O-C-N	7.39	134.52	122.70
1	A	439	ASP	CA-C-N	-7.39	100.95	117.20
4	M	407	THR	N-CA-C	-7.38	91.06	111.00
1	A	260	PHE	C-N-CA	-7.38	103.25	121.70
1	A	394	GLN	O-C-N	7.37	134.49	122.70
2	B	270	SER	N-CA-C	-7.37	91.11	111.00
3	S	55	PRO	CA-N-CD	-7.37	101.19	111.50
3	S	25	LEU	C-N-CD	7.36	143.86	128.40
2	B	488	ARG	O-C-N	7.36	134.47	122.70
3	S	54	PRO	C-N-CD	7.36	143.85	128.40
2	B	142	LEU	C-N-CA	-7.34	103.35	121.70
1	A	519	LEU	O-C-N	-7.34	110.72	123.20
3	S	66	ASP	C-N-CA	7.33	140.04	121.70
4	M	130	GLU	CA-C-N	-7.33	101.08	117.20
2	B	183	ALA	N-CA-C	7.33	130.78	111.00
2	B	192	LEU	O-C-N	7.32	134.41	122.70
4	M	61	GLU	O-C-N	7.32	134.41	122.70
1	A	559	PHE	O-C-N	7.31	134.39	122.70
1	A	355	LEU	O-C-N	7.31	134.39	122.70
4	M	132	GLY	CA-C-O	7.30	133.74	120.60
3	S	148	ARG	O-C-N	7.30	134.38	122.70
3	S	96	LEU	O-C-N	7.30	134.38	122.70
1	A	606	PHE	O-C-N	7.29	134.36	122.70
2	B	172	GLU	O-C-N	-7.28	111.05	122.70
1	A	263	LEU	C-N-CA	-7.28	103.51	121.70
1	A	529	GLY	N-CA-C	-7.27	94.92	113.10
2	B	243	TRP	O-C-N	7.26	134.31	122.70

Continued on next page...

Continued from previous page...

Mol	Chain	Res	Type	Atoms	Z	Observed(°)	Ideal(°)
3	S	156	LEU	O-C-N	7.24	134.29	122.70
1	A	405	THR	N-CA-C	7.24	130.54	111.00
2	B	572	GLU	O-C-N	7.23	134.27	122.70
1	A	204	VAL	C-N-CA	-7.22	103.64	121.70
2	B	531	ARG	O-C-N	7.22	134.26	122.70
1	A	511	VAL	O-C-N	7.21	134.24	122.70
1	A	107	TYR	O-C-N	7.21	134.23	122.70
1	A	302	ASN	CA-C-N	-7.20	101.37	117.20
2	B	288	TYR	N-CA-C	-7.20	91.57	111.00
2	B	415	LEU	O-C-N	7.20	134.21	122.70
1	A	573	GLU	C-N-CA	-7.18	103.75	121.70
1	A	265	GLN	CA-C-O	-7.18	105.03	120.10
3	S	167	ILE	N-CA-C	7.17	130.37	111.00
4	M	128	CYS	C-N-CA	-7.17	103.77	121.70
1	A	539	ASN	C-N-CA	-7.17	103.78	121.70
4	M	319	SER	N-CA-C	-7.17	91.64	111.00
3	S	84	TYR	O-C-N	-7.17	111.23	122.70
4	M	129	VAL	C-N-CA	7.16	139.61	121.70
2	B	153	ILE	O-C-N	7.16	134.16	122.70
2	B	213	LEU	O-C-N	7.16	134.15	122.70
2	B	323	ASN	O-C-N	7.16	134.15	122.70
2	B	493	LEU	O-C-N	7.16	134.15	122.70
2	B	325	LEU	CA-C-O	7.15	135.12	120.10
3	S	80	TYR	CA-C-O	7.15	135.12	120.10
4	M	118	TYR	CA-C-O	-7.15	105.08	120.10
1	A	391	LEU	O-C-N	-7.14	111.28	122.70
1	A	607	LEU	O-C-N	7.14	134.12	122.70
3	S	47	GLN	CA-C-N	-7.14	101.50	117.20
4	M	460	ILE	C-N-CA	7.14	137.28	122.30
2	B	185	LYS	O-C-N	-7.13	111.28	122.70
4	M	54	SER	C-N-CA	7.13	139.53	121.70
1	A	508	LEU	N-CA-C	7.13	130.25	111.00
3	S	118	GLU	O-C-N	7.13	134.11	122.70
1	A	383	ASN	O-C-N	7.12	134.09	122.70
2	B	223	LEU	O-C-N	-7.12	111.31	122.70
2	B	102	HIS	CA-C-O	7.12	135.05	120.10
4	M	458	LEU	C-N-CA	7.12	139.50	121.70
2	B	478	LEU	CA-C-O	7.11	135.04	120.10
2	B	511	ILE	O-C-N	-7.11	111.32	122.70
1	A	447	PHE	O-C-N	7.10	134.05	122.70
1	A	453	VAL	O-C-N	7.09	134.05	122.70
3	S	137	GLN	CA-C-N	-7.09	102.02	116.20

Continued on next page...

Continued from previous page...

Mol	Chain	Res	Type	Atoms	Z	Observed(°)	Ideal(°)
1	A	543	TYR	CG-CD2-CE2	7.08	126.97	121.30
2	B	143	SER	C-N-CA	-7.08	103.99	121.70
1	A	474	GLY	O-C-N	7.08	134.03	122.70
1	A	624	LEU	O-C-N	7.07	134.02	122.70
1	A	441	TYR	CA-C-O	7.07	134.95	120.10
1	A	566	PHE	N-CA-C	7.07	130.09	111.00
4	M	110	SER	O-C-N	7.07	134.01	122.70
4	M	53	HIS	O-C-N	-7.06	111.40	122.70
4	M	266	ASP	CA-C-N	-7.06	101.67	117.20
1	A	602	GLU	O-C-N	7.06	133.99	122.70
4	M	403	THR	N-CA-C	-7.05	91.96	111.00
2	B	78	ASP	O-C-N	-7.05	111.42	122.70
2	B	105	LEU	C-N-CA	-7.04	104.10	121.70
2	B	355	ASN	O-C-N	7.04	133.96	122.70
3	S	99	LEU	O-C-N	7.04	133.96	122.70
1	A	488	ARG	O-C-N	7.03	133.95	122.70
2	B	56	SER	C-N-CA	-7.03	104.12	121.70
2	B	226	LEU	O-C-N	7.02	133.94	122.70
2	B	475	ILE	O-C-N	7.02	133.93	122.70
1	A	256	LEU	O-C-N	7.01	133.92	122.70
1	A	300	LYS	N-CA-C	7.01	129.92	111.00
3	S	83	LEU	CA-C-O	7.00	134.81	120.10
4	M	63	TYR	CG-CD2-CE2	-7.00	115.70	121.30
1	A	581	LEU	O-C-N	7.00	133.90	122.70
2	B	267	ASP	O-C-N	-7.00	111.50	122.70
4	M	63	TYR	CA-C-N	-7.00	101.80	117.20
2	B	389	ILE	C-N-CA	-7.00	104.21	121.70
2	B	104	TYR	O-C-N	7.00	133.89	122.70
4	M	426	LYS	C-N-CA	6.99	139.17	121.70
1	A	177	ILE	O-C-N	6.99	133.88	122.70
1	A	102	GLN	O-C-N	6.98	133.87	122.70
2	B	422	ALA	N-CA-CB	6.98	119.87	110.10
2	B	216	LYS	C-N-CA	-6.98	104.26	121.70
2	B	260	LEU	C-N-CD	6.97	143.05	128.40
1	A	436	CYS	O-C-N	-6.97	111.55	122.70
2	B	489	ILE	O-C-N	6.97	133.84	122.70
2	B	97	VAL	O-C-N	6.96	133.84	122.70
1	A	582	ILE	O-C-N	6.96	133.84	122.70
1	A	279	LEU	C-N-CA	-6.95	104.32	121.70
2	B	474	VAL	O-C-N	6.95	133.82	122.70
4	M	106	LYS	CA-C-N	-6.94	101.92	117.20
2	B	253	ILE	O-C-N	6.94	133.80	122.70

Continued on next page...

Continued from previous page...

Mol	Chain	Res	Type	Atoms	Z	Observed(°)	Ideal(°)
2	B	395	LYS	O-C-N	6.94	133.80	122.70
3	S	33	GLU	O-C-N	6.94	133.80	122.70
1	A	182	ILE	O-C-N	6.93	133.80	122.70
1	A	517	TRP	O-C-N	6.93	133.79	122.70
4	M	63	TYR	CB-CG-CD1	-6.93	116.84	121.00
1	A	188	VAL	O-C-N	6.93	133.79	122.70
1	A	569	ASP	O-C-N	-6.93	111.61	122.70
2	B	174	ALA	C-N-CA	-6.93	104.37	121.70
3	S	106	VAL	O-C-N	6.93	133.79	122.70
3	S	143	GLU	CA-C-N	-6.93	101.96	117.20
1	A	514	GLU	O-C-N	6.92	133.78	122.70
1	A	623	MET	O-C-N	6.92	133.77	122.70
1	A	271	ARG	O-C-N	6.91	133.76	122.70
2	B	611	ALA	O-C-N	6.91	133.76	122.70
1	A	301	GLY	N-CA-C	-6.91	95.83	113.10
1	A	621	LEU	C-N-CA	-6.91	93.00	122.00
4	M	91	THR	CA-C-O	-6.91	105.60	120.10
4	M	425	THR	C-N-CA	6.91	138.96	121.70
2	B	521	ILE	C-N-CA	-6.90	104.44	121.70
1	A	306	GLU	O-C-N	-6.90	111.66	122.70
2	B	411	ILE	O-C-N	6.90	133.74	122.70
4	M	47	SER	N-CA-C	-6.90	92.37	111.00
1	A	451	ASN	O-C-N	6.89	133.73	122.70
4	M	317	MET	C-N-CA	-6.89	104.47	121.70
1	A	338	PHE	O-C-N	6.89	133.72	122.70
3	S	99	LEU	CA-C-O	-6.89	105.64	120.10
1	A	166	LEU	O-C-N	6.88	133.71	122.70
1	A	406	GLY	N-CA-C	-6.88	95.89	113.10
2	B	273	SER	N-CA-CB	-6.88	100.18	110.50
2	B	271	GLU	C-N-CA	-6.88	107.86	122.30
2	B	573	GLU	C-N-CA	-6.88	104.51	121.70
3	S	116	VAL	O-C-N	6.88	133.70	122.70
3	S	8	PHE	CA-C-O	6.87	134.53	120.10
3	S	128	LEU	O-C-N	6.87	133.70	122.70
2	B	457	HIS	CA-C-O	6.87	134.52	120.10
2	B	552	SER	O-C-N	6.87	133.69	122.70
4	M	277	GLU	O-C-N	-6.87	111.71	122.70
2	B	356	LYS	O-C-N	6.87	133.69	122.70
1	A	162	ILE	O-C-N	6.86	133.68	122.70
1	A	555	LEU	O-C-N	6.86	133.68	122.70
3	S	68	VAL	C-N-CA	-6.86	104.54	121.70
2	B	158	VAL	O-C-N	6.86	133.68	122.70

Continued on next page...

Continued from previous page...

Mol	Chain	Res	Type	Atoms	Z	Observed(°)	Ideal(°)
1	A	635	ALA	O-C-N	6.86	133.67	122.70
1	A	466	ASP	O-C-N	6.86	133.67	122.70
4	M	455	VAL	C-N-CA	-6.86	104.56	121.70
2	B	555	LEU	O-C-N	6.85	133.66	122.70
1	A	457	LEU	O-C-N	6.85	133.66	122.70
1	A	257	LEU	O-C-N	6.85	133.66	122.70
1	A	601	VAL	CA-C-O	6.85	134.48	120.10
1	A	392	MET	O-C-N	6.84	133.65	122.70
2	B	362	ALA	O-C-N	6.84	133.65	122.70
3	S	24	ASP	O-C-N	6.84	133.65	122.70
4	M	21	GLY	N-CA-C	-6.84	95.99	113.10
4	M	50	TYR	N-CA-CB	-6.84	98.28	110.60
2	B	504	ALA	O-C-N	6.84	133.64	122.70
1	A	140	VAL	CA-C-O	6.84	134.46	120.10
1	A	140	VAL	O-C-N	-6.83	111.76	122.70
2	B	447	GLU	O-C-N	6.83	133.63	122.70
1	A	67	LYS	O-C-N	6.83	133.62	122.70
1	A	298	ILE	CA-C-O	6.82	134.43	120.10
2	B	66	ILE	O-C-N	6.82	133.61	122.70
2	B	579	PRO	CA-C-N	6.82	132.20	117.20
4	M	394	GLN	CA-C-O	6.82	134.42	120.10
2	B	568	VAL	C-N-CA	6.82	138.74	121.70
1	A	388	VAL	O-C-N	6.81	133.59	122.70
2	B	137	PHE	O-C-N	6.81	133.59	122.70
2	B	363	ILE	O-C-N	6.81	133.59	122.70
1	A	399	ASP	O-C-N	6.80	133.59	122.70
1	A	610	SER	O-C-N	6.80	133.59	122.70
1	A	515	CYS	O-C-N	6.80	133.59	122.70
3	S	32	LEU	O-C-N	6.80	133.58	122.70
4	M	5	PHE	CA-C-O	6.80	134.38	120.10
3	S	29	LYS	O-C-N	6.80	133.58	122.70
2	B	139	LEU	O-C-N	6.79	133.57	122.70
2	B	99	ARG	O-C-N	6.79	133.57	122.70
1	A	265	GLN	N-CA-CB	6.79	122.82	110.60
1	A	225	LEU	O-C-N	6.78	133.55	122.70
4	M	60	LEU	O-C-N	6.78	133.55	122.70
1	A	105	VAL	O-C-N	6.78	134.72	123.20
1	A	599	ARG	O-C-N	6.78	133.54	122.70
3	S	157	ASN	O-C-N	6.78	133.54	122.70
2	B	267	ASP	CA-C-O	6.77	134.32	120.10
2	B	581	TYR	N-CA-C	-6.77	92.72	111.00
2	B	521	ILE	N-CA-C	-6.76	92.74	111.00

Continued on next page...

Continued from previous page...

Mol	Chain	Res	Type	Atoms	Z	Observed(°)	Ideal(°)
2	B	589	SER	O-C-N	6.76	133.52	122.70
4	M	96	ILE	O-C-N	6.76	133.52	122.70
4	M	290	PHE	C-N-CA	6.76	138.60	121.70
2	B	544	THR	O-C-N	6.76	133.51	122.70
1	A	603	VAL	O-C-N	6.76	133.51	122.70
1	A	418	ILE	N-CA-C	6.76	129.24	111.00
2	B	559	ASP	O-C-N	6.76	133.51	122.70
4	M	85	GLY	N-CA-C	-6.75	96.21	113.10
2	B	158	VAL	CA-C-O	-6.75	105.92	120.10
2	B	150	LEU	O-C-N	6.75	133.49	122.70
1	A	156	PRO	C-N-CA	-6.74	104.84	121.70
1	A	432	ILE	CA-C-O	-6.74	105.94	120.10
1	A	607	LEU	CA-C-O	-6.74	105.94	120.10
2	B	566	ALA	CB-CA-C	6.74	120.22	110.10
1	A	608	ARG	O-C-N	6.73	133.47	122.70
2	B	594	ALA	O-C-N	6.73	133.47	122.70
2	B	412	PHE	O-C-N	6.73	133.46	122.70
1	A	556	VAL	O-C-N	6.72	133.46	122.70
2	B	267	ASP	C-N-CA	-6.72	104.89	121.70
1	A	605	GLU	O-C-N	6.72	133.46	122.70
1	A	471	SER	O-C-N	6.72	133.45	122.70
2	B	359	LEU	O-C-N	6.72	133.45	122.70
4	M	319	SER	C-N-CA	6.72	138.50	121.70
1	A	196	LEU	CA-C-O	6.72	134.20	120.10
1	A	467	LYS	O-C-N	6.71	133.44	122.70
3	S	150	VAL	O-C-N	6.71	133.43	122.70
4	M	284	SER	O-C-N	-6.71	108.35	121.10
1	A	225	LEU	CA-C-O	-6.71	106.02	120.10
1	A	600	SER	O-C-N	6.71	133.43	122.70
1	A	381	GLU	O-C-N	-6.70	111.98	122.70
2	B	157	THR	O-C-N	6.70	133.42	122.70
3	S	30	LEU	O-C-N	6.69	133.41	122.70
4	M	54	SER	CA-C-N	-6.69	102.49	117.20
2	B	123	LEU	O-C-N	6.68	133.38	122.70
4	M	4	SER	CA-C-O	6.67	134.12	120.10
4	M	59	ASP	CB-CG-OD1	6.67	124.31	118.30
1	A	496	ILE	O-C-N	6.67	133.37	122.70
3	S	54	PRO	N-CA-C	-6.67	94.76	112.10
2	B	306	LEU	O-C-N	6.67	133.37	122.70
2	B	508	ARG	CA-C-O	6.66	134.09	120.10
4	M	389	SER	O-C-N	6.66	133.36	122.70
2	B	513	TRP	O-C-N	6.66	133.36	122.70

Continued on next page...

Continued from previous page...

Mol	Chain	Res	Type	Atoms	Z	Observed(°)	Ideal(°)
4	M	425	THR	N-CA-C	-6.66	93.02	111.00
2	B	507	ALA	O-C-N	6.65	133.34	122.70
1	A	265	GLN	O-C-N	-6.64	112.07	122.70
1	A	477	PHE	O-C-N	6.64	133.33	122.70
2	B	322	CYS	O-C-N	6.64	133.32	122.70
4	M	458	LEU	N-CA-C	-6.64	93.07	111.00
1	A	465	SER	O-C-N	6.63	133.32	122.70
2	B	154	ILE	CA-C-O	-6.63	106.17	120.10
1	A	297	CYS	O-C-N	-6.63	112.09	122.70
1	A	522	PHE	O-C-N	6.63	133.31	122.70
4	M	94	GLU	O-C-N	6.63	133.31	122.70
2	B	591	MET	O-C-N	6.62	133.30	122.70
2	B	302	PHE	O-C-N	6.62	133.29	122.70
2	B	306	LEU	CA-C-O	-6.62	106.19	120.10
4	M	130	GLU	N-CA-CB	6.62	122.52	110.60
2	B	361	GLN	O-C-N	6.61	133.28	122.70
1	A	388	VAL	CA-C-O	-6.61	106.22	120.10
2	B	460	SER	N-CA-C	6.61	128.84	111.00
4	M	78	ALA	CB-CA-C	6.61	120.01	110.10
4	M	113	LYS	O-C-N	6.60	133.26	122.70
2	B	613	MET	O-C-N	6.60	133.26	122.70
2	B	134	LEU	O-C-N	6.60	133.26	122.70
4	M	63	TYR	O-C-N	6.60	133.26	122.70
4	M	385	ARG	O-C-N	-6.60	112.15	122.70
1	A	307	ASP	N-CA-C	-6.59	93.21	111.00
2	B	100	LEU	O-C-N	6.59	133.24	122.70
2	B	548	ILE	O-C-N	6.58	133.23	122.70
1	A	127	LEU	O-C-N	6.58	133.23	122.70
1	A	258	LYS	O-C-N	6.57	133.22	122.70
1	A	296	ASN	O-C-N	6.57	133.22	122.70
1	A	611	LEU	O-C-N	6.57	133.21	122.70
1	A	495	ILE	O-C-N	6.57	133.21	122.70
4	M	424	PHE	N-CA-C	-6.57	93.27	111.00
2	B	343	LEU	O-C-N	6.57	133.20	122.70
2	B	154	ILE	O-C-N	6.56	133.20	122.70
2	B	604	GLU	O-C-N	6.56	133.20	122.70
3	S	18	LYS	O-C-N	-6.56	112.20	122.70
4	M	125	PHE	O-C-N	6.56	133.20	122.70
1	A	240	LEU	O-C-N	-6.56	112.20	122.70
2	B	301	LEU	O-C-N	6.55	133.19	122.70
1	A	146	ALA	O-C-N	6.55	133.18	122.70
1	A	297	CYS	CA-C-O	6.55	133.86	120.10

Continued on next page...

Continued from previous page...

Mol	Chain	Res	Type	Atoms	Z	Observed(°)	Ideal(°)
2	B	63	MET	O-C-N	6.55	133.18	122.70
2	B	170	ARG	O-C-N	-6.55	112.06	123.20
1	A	289	SER	CA-C-O	6.55	133.85	120.10
1	A	236	LEU	O-C-N	6.55	133.17	122.70
3	S	106	VAL	CA-C-O	-6.54	106.36	120.10
2	B	230	PHE	O-C-N	-6.54	112.23	122.70
2	B	453	TRP	O-C-N	6.54	133.16	122.70
2	B	510	GLY	CA-C-O	-6.54	108.84	120.60
4	M	54	SER	CB-CA-C	-6.53	97.69	110.10
1	A	91	ILE	O-C-N	6.53	133.14	122.70
1	A	87	CYS	O-C-N	6.53	133.14	122.70
1	A	475	GLU	O-C-N	6.52	133.14	122.70
2	B	146	LYS	CA-C-N	6.52	131.55	117.20
2	B	425	PRO	O-C-N	6.52	133.13	122.70
3	S	4	ALA	O-C-N	-6.52	112.26	122.70
2	B	181	TYR	C-N-CA	-6.52	105.40	121.70
2	B	414	GLU	O-C-N	6.52	133.13	122.70
4	M	265	ASN	CA-C-N	6.51	131.53	117.20
2	B	325	LEU	C-N-CA	-6.51	105.42	121.70
2	B	337	THR	CA-C-O	-6.51	106.42	120.10
2	B	607	ILE	O-C-N	6.51	133.12	122.70
4	M	281	GLY	N-CA-C	-6.51	96.82	113.10
1	A	613	ALA	O-C-N	6.51	133.11	122.70
2	B	246	SER	O-C-N	6.51	133.11	122.70
2	B	256	CYS	O-C-N	-6.51	112.29	122.70
1	A	522	PHE	CA-C-O	-6.50	106.44	120.10
2	B	344	VAL	O-C-N	6.50	133.10	122.70
2	B	105	LEU	CA-C-O	6.50	133.75	120.10
3	S	4	ALA	CA-C-O	6.50	133.74	120.10
1	A	472	LYS	O-C-N	6.49	133.09	122.70
1	A	562	TRP	O-C-N	6.49	133.09	122.70
2	B	181	TYR	O-C-N	-6.49	112.31	122.70
1	A	107	TYR	CA-C-O	-6.49	106.47	120.10
3	S	101	LEU	O-C-N	6.49	133.08	122.70
2	B	135	ARG	O-C-N	6.49	133.08	122.70
1	A	487	MET	C-N-CA	-6.49	105.49	121.70
2	B	116	THR	O-C-N	6.49	133.08	122.70
2	B	467	VAL	O-C-N	6.49	133.08	122.70
2	B	485	LYS	O-C-N	6.48	133.07	122.70
3	S	129	GLU	O-C-N	6.48	133.07	122.70
4	M	117	ASN	O-C-N	6.48	133.07	122.70
4	M	277	GLU	C-N-CA	-6.48	105.50	121.70

Continued on next page...

Continued from previous page...

Mol	Chain	Res	Type	Atoms	Z	Observed(°)	Ideal(°)
2	B	588	ILE	O-C-N	6.48	133.07	122.70
2	B	285	GLU	N-CA-C	-6.48	93.51	111.00
2	B	582	ASP	CA-C-N	6.48	131.45	117.20
4	M	107	ASP	CA-C-N	6.48	131.45	117.20
1	A	609	LEU	O-C-N	6.47	133.05	122.70
1	A	462	GLN	C-N-CA	-6.47	105.53	121.70
2	B	585	GLY	O-C-N	6.47	133.05	122.70
2	B	305	SER	O-C-N	6.46	133.04	122.70
1	A	385	LYS	O-C-N	6.46	133.04	122.70
2	B	151	ALA	CA-C-O	-6.46	106.53	120.10
3	S	127	THR	O-C-N	6.46	133.04	122.70
4	M	124	ILE	O-C-N	6.46	133.04	122.70
1	A	145	ILE	O-C-N	6.46	133.03	122.70
1	A	386	ALA	O-C-N	6.46	133.03	122.70
2	B	102	HIS	C-N-CA	-6.46	105.55	121.70
2	B	81	LEU	C-N-CA	-6.46	105.56	121.70
4	M	457	GLY	CA-C-N	-6.46	103.00	117.20
1	A	486	SER	N-CA-C	-6.45	93.58	111.00
2	B	450	VAL	O-C-N	6.45	133.03	122.70
2	B	454	LEU	O-C-N	6.45	133.02	122.70
4	M	123	LEU	O-C-N	6.45	133.02	122.70
1	A	80	TYR	N-CA-CB	6.44	122.20	110.60
2	B	244	SER	O-C-N	6.44	133.01	122.70
1	A	407	SER	N-CA-C	-6.44	93.60	111.00
1	A	200	PHE	O-C-N	6.44	133.00	122.70
2	B	62	ALA	O-C-N	6.44	133.00	122.70
2	B	65	ARG	O-C-N	6.44	133.00	122.70
4	M	105	ASP	N-CA-C	6.44	128.38	111.00
2	B	151	ALA	O-C-N	6.43	133.33	121.10
3	S	105	PHE	O-C-N	6.43	132.99	122.70
1	A	415	ARG	C-N-CA	-6.43	105.62	121.70
2	B	296	ASP	O-C-N	6.43	133.31	121.10
1	A	476	GLN	O-C-N	6.43	132.98	122.70
1	A	516	ILE	O-C-N	6.42	132.98	122.70
2	B	472	VAL	CA-C-O	6.42	133.59	120.10
1	A	579	LYS	O-C-N	6.42	132.97	122.70
2	B	417	TYR	O-C-N	6.42	132.97	122.70
4	M	65	TYR	C-N-CA	-6.42	105.65	121.70
1	A	348	PHE	O-C-N	6.42	132.96	122.70
2	B	229	HIS	C-N-CA	6.41	137.73	121.70
4	M	122	SER	O-C-N	6.41	132.95	122.70
1	A	159	ALA	O-C-N	6.41	132.95	122.70

Continued on next page...

Continued from previous page...

Mol	Chain	Res	Type	Atoms	Z	Observed(°)	Ideal(°)
1	A	302	ASN	CA-C-O	6.41	133.55	120.10
1	A	629	LEU	C-N-CA	-6.41	95.09	122.00
2	B	612	ARG	O-C-N	6.41	132.95	122.70
1	A	215	VAL	O-C-N	-6.40	112.46	122.70
2	B	360	LEU	O-C-N	6.40	132.94	122.70
1	A	550	VAL	O-C-N	6.40	132.93	122.70
1	A	83	ASP	CA-C-N	-6.39	103.13	117.20
2	B	145	MET	C-N-CA	-6.39	105.73	121.70
1	A	425	LYS	O-C-N	6.38	132.91	122.70
2	B	191	GLU	O-C-N	6.38	132.92	122.70
2	B	399	LEU	C-N-CA	-6.38	105.74	121.70
2	B	79	VAL	CA-C-O	-6.38	106.70	120.10
1	A	64	LEU	CA-C-O	6.38	133.50	120.10
1	A	558	VAL	O-C-N	6.38	132.91	122.70
1	A	604	LEU	O-C-N	6.38	132.91	122.70
1	A	147	LEU	O-C-N	6.38	132.90	122.70
2	B	139	LEU	CA-C-O	-6.37	106.72	120.10
2	B	342	ALA	O-C-N	6.37	132.90	122.70
1	A	554	ALA	O-C-N	6.37	132.89	122.70
2	B	452	LYS	O-C-N	6.37	132.89	122.70
1	A	290	VAL	CA-C-O	-6.37	106.72	120.10
1	A	141	VAL	O-C-N	6.37	132.89	122.70
1	A	634	ASN	O-C-N	6.37	132.89	122.70
2	B	271	GLU	N-CA-C	6.37	128.19	111.00
2	B	615	SER	O-C-N	6.37	132.89	122.70
2	B	123	LEU	CA-C-O	-6.37	106.73	120.10
2	B	610	ARG	O-C-N	6.37	132.88	122.70
4	M	86	PRO	O-C-N	6.37	132.89	122.70
1	A	211	ASP	O-C-N	6.36	132.88	122.70
1	A	454	ILE	O-C-N	6.36	132.87	122.70
2	B	614	ILE	O-C-N	6.36	132.87	122.70
1	A	202	LYS	O-C-N	6.36	132.87	122.70
1	A	128	LEU	O-C-N	6.35	132.86	122.70
2	B	388	PRO	C-N-CA	6.35	137.57	121.70
2	B	592	TYR	O-C-N	6.34	132.85	122.70
2	B	341	GLU	O-C-N	6.34	132.84	122.70
1	A	278	ILE	C-N-CA	6.34	137.54	121.70
4	M	16	PHE	CA-C-O	6.34	133.41	120.10
1	A	332	TYR	O-C-N	6.33	132.83	122.70
4	M	90	PHE	O-C-N	6.33	132.84	122.70
1	A	331	ARG	O-C-N	6.33	132.83	122.70
3	S	1	MET	O-C-N	-6.33	112.57	122.70

Continued on next page...

Continued from previous page...

Mol	Chain	Res	Type	Atoms	Z	Observed(°)	Ideal(°)
4	M	51	LEU	O-C-N	-6.33	112.57	122.70
4	M	3	LEU	CA-C-O	6.33	133.38	120.10
1	A	186	PHE	O-C-N	6.32	132.81	122.70
2	B	209	SER	O-C-N	6.32	132.81	122.70
4	M	402	SER	CA-C-N	6.32	131.10	117.20
3	S	165	SER	C-N-CA	-6.32	105.91	121.70
1	A	421	PRO	O-C-N	6.31	132.80	122.70
1	A	603	VAL	CA-C-O	-6.31	106.84	120.10
4	M	232	HIS	O-C-N	-6.31	112.60	122.70
1	A	185	LEU	O-C-N	6.31	132.80	122.70
2	B	67	ILE	O-C-N	6.31	132.80	122.70
2	B	177	ILE	O-C-N	6.31	132.79	122.70
2	B	435	SER	O-C-N	6.31	132.79	122.70
1	A	492	ILE	O-C-N	6.31	132.79	122.70
1	A	428	MET	O-C-N	6.30	132.78	122.70
1	A	316	LEU	O-C-N	6.30	132.78	122.70
2	B	302	PHE	CA-C-O	-6.30	106.87	120.10
1	A	110	ALA	O-C-N	-6.30	112.62	122.70
2	B	409	LYS	O-C-N	6.29	132.77	122.70
1	A	155	THR	CA-C-O	-6.29	106.88	120.10
2	B	595	VAL	O-C-N	6.29	132.77	122.70
1	A	395	PHE	C-N-CA	-6.29	105.99	121.70
2	B	101	ILE	O-C-N	6.29	132.76	122.70
2	B	262	LYS	C-N-CA	-6.29	95.60	122.00
3	S	130	SER	O-C-N	6.29	132.76	122.70
2	B	490	ILE	O-C-N	6.28	132.75	122.70
2	B	550	VAL	O-C-N	6.28	132.75	122.70
1	A	139	ASP	O-C-N	6.28	132.74	122.70
1	A	620	GLY	CA-C-O	-6.28	109.30	120.60
1	A	337	LEU	O-C-N	6.28	132.74	122.70
1	A	552	ILE	O-C-N	6.28	132.74	122.70
3	S	80	TYR	O-C-N	-6.28	112.66	122.70
1	A	252	ILE	O-C-N	6.27	132.73	122.70
1	A	298	ILE	C-N-CA	-6.27	106.03	121.70
1	A	473	ILE	O-C-N	6.27	133.86	123.20
2	B	193	LEU	O-C-N	6.27	132.73	122.70
4	M	126	ASN	CA-C-O	-6.27	106.94	120.10
1	A	117	ASP	O-C-N	6.27	132.72	122.70
1	A	625	LEU	O-C-N	-6.27	112.67	122.70
2	B	394	TRP	O-C-N	6.26	132.72	122.70
2	B	587	ARG	O-C-N	6.26	132.72	122.70
1	A	569	ASP	CA-C-O	-6.26	106.95	120.10

Continued on next page...

Continued from previous page...

Mol	Chain	Res	Type	Atoms	Z	Observed(°)	Ideal(°)
2	B	346	THR	O-C-N	6.26	132.71	122.70
2	B	64	LYS	O-C-N	6.25	132.70	122.70
2	B	549	LEU	O-C-N	6.25	132.71	122.70
1	A	387	ILE	O-C-N	6.25	132.70	122.70
1	A	574	ILE	O-C-N	6.25	132.70	122.70
2	B	432	ALA	O-C-N	6.25	132.70	122.70
4	M	55	MET	C-N-CA	-6.25	106.08	121.70
3	S	120	ASP	O-C-N	6.25	132.69	122.70
1	A	333	ILE	O-C-N	6.24	132.69	122.70
1	A	68	THR	O-C-N	6.24	132.69	122.70
1	A	439	ASP	N-CA-CB	6.24	121.83	110.60
2	B	374	PHE	O-C-N	6.24	132.68	122.70
1	A	576	MET	O-C-N	6.23	132.67	122.70
4	M	368	ASP	CA-C-O	-6.23	107.01	120.10
2	B	249	ILE	O-C-N	6.23	132.67	122.70
1	A	335	CYS	O-C-N	6.23	132.66	122.70
1	A	421	PRO	CA-C-O	-6.23	105.26	120.20
3	S	85	PHE	CA-C-O	6.23	133.18	120.10
4	M	394	GLN	O-C-N	-6.23	112.61	123.20
1	A	632	PHE	O-C-N	6.22	132.66	122.70
2	B	426	GLU	O-C-N	6.22	132.66	122.70
2	B	111	ASN	N-CA-C	6.22	127.79	111.00
2	B	272	GLY	N-CA-C	-6.22	97.55	113.10
2	B	522	GLU	O-C-N	6.22	132.65	122.70
1	A	492	ILE	CA-C-O	-6.22	107.04	120.10
1	A	353	ASP	O-C-N	6.22	132.65	122.70
4	M	77	LEU	CA-C-O	6.22	133.15	120.10
2	B	59	VAL	O-C-N	6.21	132.64	122.70
2	B	363	ILE	CA-C-O	-6.21	107.05	120.10
1	A	98	ASN	O-C-N	-6.21	112.76	122.70
1	A	372	ILE	O-C-N	6.21	132.63	122.70
1	A	274	LEU	C-N-CA	-6.20	106.19	121.70
2	B	455	ILE	O-C-N	6.20	132.62	122.70
3	S	149	ILE	O-C-N	6.20	132.62	122.70
4	M	272	LEU	C-N-CA	6.19	137.18	121.70
2	B	155	LEU	O-C-N	6.19	132.60	122.70
1	A	140	VAL	C-N-CA	-6.19	106.23	121.70
2	B	605	PHE	O-C-N	6.19	132.60	122.70
1	A	108	LEU	CA-C-O	-6.18	107.11	120.10
1	A	429	VAL	O-C-N	6.18	132.60	122.70
2	B	119	SER	O-C-N	6.18	132.60	122.70
2	B	430	ILE	O-C-N	6.18	132.59	122.70

Continued on next page...

Continued from previous page...

Mol	Chain	Res	Type	Atoms	Z	Observed(°)	Ideal(°)
1	A	628	VAL	O-C-N	6.18	132.59	122.70
2	B	543	GLU	O-C-N	6.18	132.59	122.70
2	B	593	ASN	O-C-N	6.18	132.59	122.70
2	B	410	GLU	O-C-N	6.18	132.59	122.70
1	A	458	ALA	O-C-N	6.18	132.58	122.70
2	B	251	LEU	O-C-N	6.18	132.58	122.70
4	M	65	TYR	O-C-N	6.17	132.58	122.70
1	A	138	ASN	O-C-N	6.17	132.57	122.70
2	B	495	ASP	O-C-N	6.16	132.56	122.70
2	B	58	GLU	O-C-N	6.16	132.56	122.70
2	B	511	ILE	CA-C-O	6.16	133.03	120.10
4	M	111	ILE	O-C-N	6.16	132.55	122.70
4	M	421	GLY	CA-C-O	6.16	131.68	120.60
1	A	241	TYR	O-C-N	6.16	132.55	122.70
2	B	532	ARG	O-C-N	6.16	132.55	122.70
2	B	248	LEU	O-C-N	6.15	132.55	122.70
2	B	429	VAL	O-C-N	6.15	132.55	122.70
2	B	136	CYS	O-C-N	6.15	132.54	122.70
2	B	145	MET	CA-C-N	6.15	130.73	117.20
3	S	43	ASN	C-N-CA	-6.15	106.33	121.70
1	A	295	VAL	CA-C-O	-6.15	107.19	120.10
1	A	339	TYR	O-C-N	6.15	132.53	122.70
1	A	142	LYS	O-C-N	6.14	132.53	122.70
2	B	233	TYR	O-C-N	6.14	132.53	122.70
4	M	429	ASP	C-N-CA	-6.14	106.34	121.70
2	B	196	LEU	O-C-N	6.14	132.53	122.70
1	A	312	ALA	O-C-N	6.14	132.52	122.70
3	S	83	LEU	O-C-N	-6.14	112.88	122.70
2	B	337	THR	O-C-N	6.14	132.52	122.70
4	M	107	ASP	CA-C-O	-6.14	107.22	120.10
2	B	551	LEU	O-C-N	6.13	132.51	122.70
3	S	70	ASN	CA-C-O	6.13	132.98	120.10
2	B	82	TYR	O-C-N	-6.13	112.89	122.70
2	B	324	ALA	O-C-N	6.13	132.50	122.70
3	S	145	ASN	O-C-N	6.13	132.50	122.70
1	A	143	VAL	O-C-N	6.13	133.62	123.20
2	B	464	SER	O-C-N	6.13	132.50	122.70
3	S	21	THR	C-N-CA	-6.13	96.27	122.00
2	B	195	ILE	O-C-N	6.12	132.49	122.70
2	B	512	VAL	O-C-N	6.12	132.49	122.70
3	S	81	ALA	N-CA-C	-6.12	94.48	111.00
1	A	491	THR	O-C-N	6.11	132.48	122.70

Continued on next page...

Continued from previous page...

Mol	Chain	Res	Type	Atoms	Z	Observed(°)	Ideal(°)
3	S	64	ASN	C-N-CA	-6.11	106.42	121.70
4	M	290	PHE	N-CA-C	-6.11	94.51	111.00
1	A	598	GLU	O-C-N	6.11	132.47	122.70
2	B	510	GLY	O-C-N	6.11	132.47	122.70
1	A	313	MET	O-C-N	6.11	132.47	122.70
1	A	237	SER	CA-C-O	-6.10	107.29	120.10
1	A	584	PHE	O-C-N	6.10	132.46	122.70
4	M	135	ASN	N-CA-CB	6.10	121.57	110.60
3	S	38	LEU	O-C-N	6.10	132.45	122.70
2	B	178	ILE	O-C-N	6.09	132.45	122.70
2	B	553	ALA	O-C-N	6.09	132.44	122.70
4	M	398	ILE	O-C-N	-6.09	112.95	122.70
2	B	244	SER	CA-C-O	-6.09	107.32	120.10
2	B	582	ASP	O-C-N	-6.08	112.97	122.70
1	A	390	THR	O-C-N	6.08	132.42	122.70
2	B	74	ASP	C-N-CA	6.07	136.88	121.70
2	B	44	PRO	O-C-N	6.07	132.41	122.70
2	B	355	ASN	CA-C-O	-6.07	107.36	120.10
3	S	139	GLY	N-CA-C	-6.07	97.93	113.10
2	B	166	SER	O-C-N	6.06	132.40	122.70
1	A	158	LEU	CA-C-O	-6.06	107.38	120.10
1	A	578	LEU	O-C-N	6.06	132.39	122.70
1	A	81	GLY	C-N-CA	6.06	136.84	121.70
1	A	533	ILE	CA-C-O	-6.05	107.39	120.10
1	A	557	LYS	O-C-N	6.05	132.39	122.70
3	S	85	PHE	O-C-N	-6.05	113.01	122.70
4	M	381	ASN	N-CA-C	-6.05	94.65	111.00
1	A	631	SER	CA-C-O	-6.05	107.39	120.10
4	M	91	THR	O-C-N	6.05	132.38	122.70
2	B	131	ASN	O-C-N	6.05	132.38	122.70
2	B	469	ASP	CA-C-O	-6.05	107.40	120.10
2	B	568	VAL	O-C-N	6.05	132.37	122.70
3	S	16	LEU	CA-C-O	6.04	132.79	120.10
2	B	189	HIS	O-C-N	6.04	132.37	122.70
4	M	131	ALA	C-N-CA	6.04	134.98	122.30
1	A	104	ARG	O-C-N	6.04	132.36	122.70
4	M	237	THR	O-C-N	-6.04	112.93	123.20
1	A	122	MET	O-C-N	6.04	132.36	122.70
2	B	207	VAL	O-C-N	6.04	132.36	122.70
2	B	585	GLY	CA-C-O	-6.04	109.73	120.60
1	A	125	THR	O-C-N	6.03	132.35	122.70
2	B	358	MET	O-C-N	6.03	132.35	122.70

Continued on next page...

Continued from previous page...

Mol	Chain	Res	Type	Atoms	Z	Observed(°)	Ideal(°)
3	S	32	LEU	CA-C-O	-6.03	107.43	120.10
2	B	525	ILE	O-C-N	6.03	132.35	122.70
1	A	494	ASN	O-C-N	6.03	132.35	122.70
2	B	155	LEU	CA-C-O	-6.03	107.44	120.10
1	A	296	ASN	CA-C-O	-6.03	107.45	120.10
1	A	633	PHE	CA-C-O	-6.03	107.45	120.10
2	B	150	LEU	CA-C-O	-6.03	107.45	120.10
1	A	229	ASN	C-N-CD	6.02	141.05	128.40
3	S	96	LEU	CA-C-O	-6.02	107.45	120.10
2	B	255	TYR	O-C-N	6.02	132.33	122.70
2	B	184	GLY	CA-C-O	6.01	131.42	120.60
2	B	190	GLU	O-C-N	6.01	132.32	122.70
1	A	71	VAL	CA-C-O	-6.01	107.48	120.10
4	M	426	LYS	N-CA-C	-6.01	94.78	111.00
2	B	103	LEU	O-C-N	6.01	132.31	122.70
2	B	462	ASN	C-N-CA	-6.01	106.68	121.70
1	A	545	HIS	CA-C-O	6.00	132.70	120.10
2	B	209	SER	CA-C-O	-6.00	107.50	120.10
2	B	413	LYS	O-C-N	6.00	132.30	122.70
1	A	336	ILE	O-C-N	6.00	132.30	122.70
1	A	538	GLU	C-N-CA	-6.00	106.71	121.70
2	B	433	VAL	O-C-N	6.00	132.29	122.70
2	B	303	LEU	O-C-N	5.99	132.29	122.70
1	A	154	ILE	C-N-CA	-5.99	106.72	121.70
1	A	108	LEU	O-C-N	5.99	132.28	122.70
2	B	437	SER	O-C-N	5.99	132.28	122.70
1	A	121	LEU	O-C-N	5.98	132.27	122.70
2	B	314	ASN	O-C-N	5.98	132.47	121.10
4	M	6	TYR	CA-C-O	5.98	132.66	120.10
4	M	283	PHE	N-CA-C	-5.98	94.84	111.00
1	A	625	LEU	C-N-CA	-5.98	106.75	121.70
1	A	249	ASN	O-C-N	5.98	132.27	122.70
4	M	117	ASN	CA-C-O	-5.98	107.55	120.10
4	M	131	ALA	N-CA-C	5.98	127.14	111.00
2	B	523	PHE	C-N-CA	-5.98	106.76	121.70
1	A	162	ILE	CA-C-O	-5.97	107.56	120.10
1	A	230	PRO	CA-C-O	-5.97	105.88	120.20
1	A	600	SER	CA-C-O	-5.97	107.56	120.10
1	A	325	SER	CA-C-N	-5.97	104.07	117.20
2	B	98	LYS	O-C-N	5.97	132.25	122.70
1	A	454	ILE	CA-C-O	-5.96	107.58	120.10
2	B	47	LEU	O-C-N	5.96	132.24	122.70

Continued on next page...

Continued from previous page...

Mol	Chain	Res	Type	Atoms	Z	Observed(°)	Ideal(°)
1	A	217	ALA	CA-C-O	-5.95	107.60	120.10
1	A	448	GLU	C-N-CA	5.95	136.58	121.70
1	A	130	LYS	O-C-N	5.95	132.22	122.70
1	A	323	CYS	CA-C-N	5.95	130.29	117.20
1	A	491	THR	CA-C-O	-5.95	107.60	120.10
2	B	204	ASP	O-C-N	5.95	132.41	121.10
1	A	155	THR	O-C-N	5.95	132.40	121.10
1	A	499	ILE	C-N-CA	-5.95	106.83	121.70
2	B	333	GLN	N-CA-C	-5.95	94.94	111.00
1	A	309	PHE	O-C-N	5.95	132.21	122.70
2	B	238	LYS	C-N-CA	5.95	136.56	121.70
1	A	271	ARG	CA-C-O	-5.94	107.62	120.10
1	A	513	ARG	CA-C-O	-5.94	107.62	120.10
4	M	120	ARG	O-C-N	5.94	132.20	122.70
4	M	74	TYR	O-C-N	-5.94	113.20	122.70
1	A	566	PHE	C-N-CA	5.93	136.53	121.70
1	A	369	SER	O-C-N	5.93	132.19	122.70
3	S	107	GLU	O-C-N	5.93	132.19	122.70
1	A	325	SER	C-N-CA	5.92	136.51	121.70
2	B	407	ASN	O-C-N	5.92	132.18	122.70
4	M	367	ALA	N-CA-C	-5.92	95.00	111.00
1	A	371	ALA	O-C-N	5.92	132.18	122.70
1	A	518	CYS	CA-C-O	-5.92	107.66	120.10
1	A	278	ILE	O-C-N	-5.92	113.22	122.70
2	B	402	LEU	N-CA-C	5.92	126.98	111.00
1	A	452	ALA	O-C-N	5.92	132.16	122.70
2	B	586	SER	O-C-N	5.91	132.16	122.70
2	B	247	TYR	O-C-N	5.91	132.16	122.70
3	S	154	ASP	O-C-N	5.91	132.15	122.70
1	A	400	VAL	O-C-N	5.91	132.15	122.70
3	S	152	SER	O-C-N	5.91	132.15	122.70
2	B	174	ALA	CA-C-O	5.90	132.50	120.10
2	B	446	TRP	O-C-N	5.90	132.15	122.70
1	A	287	ALA	N-CA-C	5.90	126.94	111.00
1	A	268	PRO	O-C-N	5.90	132.14	122.70
1	A	500	SER	O-C-N	-5.90	113.26	122.70
2	B	250	GLU	O-C-N	5.90	132.14	122.70
2	B	444	THR	CA-C-O	5.90	132.49	120.10
2	B	239	GLN	C-N-CA	5.90	136.44	121.70
2	B	579	PRO	N-CA-C	5.90	127.43	112.10
1	A	490	VAL	O-C-N	5.90	132.13	122.70
3	S	61	ASN	C-N-CA	-5.89	106.97	121.70

Continued on next page...

Continued from previous page...

Mol	Chain	Res	Type	Atoms	Z	Observed(°)	Ideal(°)
2	B	469	ASP	O-C-N	5.89	132.13	122.70
2	B	590	GLN	O-C-N	5.89	132.13	122.70
2	B	396	ILE	O-C-N	5.89	132.12	122.70
2	B	496	LEU	O-C-N	5.89	132.12	122.70
4	M	280	ASP	CA-C-N	-5.89	104.42	116.20
4	M	306	LEU	CA-C-O	5.89	132.46	120.10
1	A	553	LEU	O-C-N	5.88	132.11	122.70
2	B	214	ALA	O-C-N	5.88	132.12	122.70
2	B	546	CYS	O-C-N	5.88	132.12	122.70
3	S	159	ALA	O-C-N	5.88	132.11	122.70
2	B	530	LEU	O-C-N	5.88	132.11	122.70
1	A	419	ILE	N-CA-C	5.88	126.88	111.00
1	A	257	LEU	CA-C-O	-5.88	107.76	120.10
1	A	106	GLY	CA-C-O	-5.88	110.02	120.60
4	M	121	ILE	O-C-N	5.87	132.10	122.70
1	A	276	PRO	O-C-N	-5.87	113.31	122.70
2	B	393	ILE	O-C-N	5.87	132.09	122.70
2	B	526	CYS	CA-C-O	-5.86	107.79	120.10
1	A	436	CYS	C-N-CA	-5.86	107.05	121.70
4	M	46	SER	O-C-N	-5.86	113.32	122.70
1	A	251	TRP	O-C-N	5.86	132.07	122.70
2	B	412	PHE	CA-C-O	-5.86	107.80	120.10
2	B	471	TYR	O-C-N	5.86	132.07	122.70
2	B	36	THR	O-C-N	5.86	132.07	122.70
2	B	490	ILE	CA-C-O	-5.86	107.80	120.10
3	S	117	ASN	O-C-N	5.85	132.07	122.70
1	A	535	ILE	O-C-N	-5.85	113.34	122.70
2	B	211	ALA	CA-C-O	-5.85	107.81	120.10
2	B	293	VAL	O-C-N	-5.85	113.33	122.70
2	B	96	LYS	O-C-N	5.85	132.06	122.70
2	B	212	VAL	C-N-CA	-5.85	107.07	121.70
2	B	449	HIS	O-C-N	5.85	132.06	122.70
2	B	232	ARG	O-C-N	5.85	132.05	122.70
2	B	476	ARG	O-C-N	5.84	132.05	122.70
2	B	569	THR	CA-C-N	-5.84	104.51	116.20
2	B	189	HIS	CA-C-O	-5.84	107.83	120.10
2	B	545	ARG	O-C-N	5.84	132.05	122.70
1	A	404	GLN	C-N-CA	5.84	136.30	121.70
1	A	417	PRO	N-CA-C	-5.84	96.92	112.10
2	B	79	VAL	O-C-N	5.84	132.04	122.70
1	A	442	SER	N-CA-CB	5.84	119.26	110.50
4	M	60	LEU	N-CA-C	-5.84	95.24	111.00

Continued on next page...

Continued from previous page...

Mol	Chain	Res	Type	Atoms	Z	Observed(°)	Ideal(°)
4	M	278	ILE	CA-C-N	-5.84	104.36	117.20
4	M	367	ALA	N-CA-CB	-5.83	101.93	110.10
1	A	214	VAL	O-C-N	5.83	132.03	122.70
1	A	333	ILE	CA-C-O	-5.83	107.86	120.10
2	B	547	GLN	O-C-N	5.83	132.02	122.70
4	M	59	ASP	O-C-N	-5.83	113.38	122.70
1	A	217	ALA	O-C-N	5.82	132.01	122.70
1	A	474	GLY	CA-C-O	-5.82	110.12	120.60
4	M	133	GLU	C-N-CA	-5.82	97.56	122.00
2	B	245	GLN	O-C-N	5.82	132.01	122.70
2	B	60	ARG	O-C-N	5.82	132.00	122.70
3	S	58	LEU	N-CA-CB	5.82	122.03	110.40
2	B	519	ALA	N-CA-CB	-5.81	101.97	110.10
1	A	265	GLN	C-N-CA	5.81	136.22	121.70
1	A	413	SER	C-N-CA	-5.81	107.18	121.70
3	S	155	GLU	O-C-N	5.81	132.00	122.70
4	M	223	HIS	O-C-N	-5.80	113.41	122.70
2	B	112	ASP	N-CA-C	5.80	126.67	111.00
2	B	418	TYR	O-C-N	-5.80	113.42	122.70
1	A	75	THR	O-C-N	5.80	131.98	122.70
1	A	430	ASN	O-C-N	5.80	131.97	122.70
2	B	448	SER	O-C-N	5.80	131.97	122.70
1	A	317	GLU	O-C-N	5.79	131.97	122.70
1	A	316	LEU	CA-C-O	-5.79	107.94	120.10
2	B	567	GLN	C-N-CA	-5.79	107.22	121.70
3	S	76	ILE	O-C-N	-5.79	113.44	122.70
4	M	401	LYS	C-N-CA	-5.79	107.23	121.70
1	A	105	VAL	C-N-CA	5.79	134.45	122.30
1	A	572	PHE	O-C-N	5.79	131.96	122.70
1	A	599	ARG	CA-C-O	-5.78	107.95	120.10
1	A	422	GLU	CA-C-O	-5.78	107.96	120.10
2	B	468	LEU	O-C-N	5.78	131.95	122.70
2	B	436	LEU	O-C-N	5.78	131.94	122.70
3	S	48	SER	C-N-CA	-5.78	107.26	121.70
2	B	466	SER	O-C-N	5.78	131.94	122.70
2	B	303	LEU	CA-C-O	-5.77	107.98	120.10
3	S	150	VAL	CA-C-O	-5.77	107.98	120.10
4	M	265	ASN	CA-C-O	-5.77	107.98	120.10
1	A	315	CYS	O-C-N	5.77	131.93	122.70
3	S	84	TYR	CA-C-O	5.77	132.21	120.10
1	A	528	ASN	O-C-N	-5.76	113.40	123.20
2	B	457	HIS	O-C-N	-5.76	113.48	122.70

Continued on next page...

Continued from previous page...

Mol	Chain	Res	Type	Atoms	Z	Observed(°)	Ideal(°)
2	B	33	SER	O-C-N	5.76	131.92	122.70
2	B	67	ILE	CA-C-O	-5.76	108.00	120.10
1	A	426	ILE	O-C-N	5.76	131.91	122.70
2	B	55	ASN	O-C-N	5.76	131.91	122.70
3	S	86	THR	O-C-N	-5.76	113.49	122.70
1	A	106	GLY	O-C-N	5.75	131.91	122.70
2	B	423	HIS	N-CA-C	-5.75	95.46	111.00
1	A	145	ILE	CA-C-O	-5.75	108.02	120.10
1	A	216	SER	O-C-N	5.75	131.91	122.70
2	B	36	THR	CA-C-O	-5.75	108.02	120.10
4	M	130	GLU	CB-CA-C	5.75	121.89	110.40
2	B	322	CYS	CA-C-O	-5.74	108.04	120.10
2	B	604	GLU	CA-C-N	-5.74	104.56	117.20
3	S	137	GLN	CA-C-O	5.74	132.16	120.10
4	M	447	ILE	CA-C-O	5.74	132.16	120.10
2	B	529	VAL	O-C-N	5.74	131.89	122.70
2	B	395	LYS	CA-C-O	-5.74	108.05	120.10
1	A	493	ALA	O-C-N	5.73	131.87	122.70
1	A	582	ILE	CA-C-O	-5.73	108.06	120.10
2	B	341	GLU	CA-C-O	-5.73	108.06	120.10
3	S	87	PHE	CA-C-O	5.73	132.13	120.10
4	M	354	ASP	N-CA-CB	5.73	120.91	110.60
3	S	117	ASN	CA-C-O	-5.73	108.07	120.10
2	B	371	GLN	O-C-N	5.73	131.86	122.70
3	S	35	VAL	O-C-N	5.72	131.86	122.70
1	A	152	THR	C-N-CA	-5.72	107.39	121.70
1	A	630	PRO	O-C-N	5.72	131.86	122.70
4	M	135	ASN	O-C-N	5.72	131.86	122.70
1	A	543	TYR	N-CA-C	5.72	126.44	111.00
2	B	216	LYS	O-C-N	-5.72	113.55	122.70
2	B	318	ILE	CA-C-O	-5.72	108.09	120.10
1	A	572	PHE	C-N-CA	5.71	135.98	121.70
2	B	275	ARG	N-CA-CB	5.71	120.89	110.60
4	M	384	GLY	O-C-N	5.71	131.84	122.70
2	B	391	ALA	O-C-N	5.71	131.84	122.70
2	B	388	PRO	O-C-N	5.71	131.84	122.70
1	A	64	LEU	C-N-CA	-5.71	107.43	121.70
1	A	135	ASP	CA-C-N	5.70	127.61	116.20
1	A	163	ALA	O-C-N	-5.70	113.57	122.70
1	A	237	SER	O-C-N	5.70	131.93	121.10
2	B	176	ALA	O-C-N	5.70	131.82	122.70
2	B	265	VAL	CA-C-N	-5.70	104.67	117.20

Continued on next page...

Continued from previous page...

Mol	Chain	Res	Type	Atoms	Z	Observed(°)	Ideal(°)
1	A	427	LYS	O-C-N	5.70	131.81	122.70
3	S	131	VAL	O-C-N	5.70	131.81	122.70
1	A	501	ASN	C-N-CA	5.69	135.93	121.70
2	B	366	LEU	CA-C-O	5.69	132.05	120.10
1	A	350	SER	C-N-CA	-5.69	107.47	121.70
2	B	324	ALA	CA-C-O	-5.69	108.16	120.10
2	B	300	ASP	O-C-N	5.68	131.79	122.70
1	A	397	ASP	O-C-N	-5.68	113.61	122.70
2	B	241	ASP	O-C-N	5.68	131.79	122.70
4	M	306	LEU	C-N-CA	-5.68	107.51	121.70
1	A	123	LEU	O-C-N	5.67	131.78	122.70
4	M	321	GLY	C-N-CA	-5.67	107.51	121.70
1	A	575	LYS	O-C-N	5.67	131.78	122.70
2	B	596	LEU	O-C-N	5.67	131.78	122.70
1	A	275	LEU	O-C-N	-5.67	110.33	121.10
4	M	136	VAL	CA-C-O	5.67	132.00	120.10
1	A	230	PRO	CA-N-CD	5.66	119.63	111.70
1	A	253	ILE	O-C-N	5.66	131.76	122.70
1	A	575	LYS	CA-C-O	-5.66	108.20	120.10
2	B	99	ARG	CA-C-O	-5.66	108.22	120.10
2	B	188	TYR	O-C-N	5.66	131.75	122.70
1	A	173	THR	C-N-CA	5.65	135.83	121.70
1	A	580	GLU	O-C-N	5.65	131.73	122.70
2	B	48	VAL	O-C-N	5.65	131.74	122.70
2	B	522	GLU	N-CA-C	5.65	126.25	111.00
2	B	428	VAL	CA-C-O	-5.64	108.25	120.10
4	M	267	ILE	C-N-CA	-5.64	110.45	122.30
2	B	477	MET	O-C-N	5.64	131.73	122.70
1	A	305	GLU	CA-C-N	5.64	129.61	117.20
2	B	431	MET	O-C-N	5.64	131.72	122.70
1	A	368	ARG	O-C-N	5.64	131.72	122.70
1	A	233	PHE	CA-C-O	5.63	131.93	120.10
4	M	261	ASN	C-N-CA	-5.63	107.63	121.70
2	B	173	VAL	O-C-N	5.63	131.71	122.70
2	B	593	ASN	CA-C-O	-5.63	108.28	120.10
1	A	188	VAL	CA-C-O	-5.63	108.28	120.10
1	A	577	VAL	O-C-N	5.63	131.70	122.70
2	B	569	THR	C-N-CA	5.63	134.12	122.30
1	A	330	LEU	CA-C-O	-5.62	108.29	120.10
3	S	87	PHE	O-C-N	-5.62	113.70	122.70
1	A	109	ALA	O-C-N	5.62	131.69	122.70
1	A	184	ALA	O-C-N	5.62	131.69	122.70

Continued on next page...

Continued from previous page...

Mol	Chain	Res	Type	Atoms	Z	Observed(°)	Ideal(°)
3	S	60	SER	C-N-CA	5.62	135.75	121.70
4	M	61	GLU	CA-C-N	-5.62	104.84	117.20
1	A	88	ASN	O-C-N	-5.62	113.71	122.70
3	S	1	MET	CA-C-O	5.62	131.89	120.10
2	B	156	HIS	O-C-N	5.61	131.68	122.70
2	B	438	ARG	O-C-N	5.61	131.68	122.70
4	M	18	TYR	CA-C-O	5.61	131.88	120.10
1	A	293	GLU	O-C-N	5.61	131.67	122.70
1	A	467	LYS	C-N-CA	-5.61	107.68	121.70
1	A	341	ILE	O-C-N	5.60	132.72	123.20
2	B	122	SER	O-C-N	5.60	131.66	122.70
1	A	551	LEU	CA-C-O	-5.60	108.35	120.10
2	B	179	LYS	O-C-N	5.60	131.65	122.70
1	A	105	VAL	CA-C-O	-5.59	108.35	120.10
2	B	365	PHE	CA-C-O	-5.59	108.35	120.10
1	A	199	ASN	O-C-N	5.59	131.65	122.70
3	S	49	SER	CA-C-N	5.59	129.50	117.20
1	A	124	ALA	O-C-N	5.59	131.64	122.70
1	A	624	LEU	CA-C-O	-5.59	108.37	120.10
2	B	434	LYS	O-C-N	5.59	131.64	122.70
1	A	277	LYS	CA-C-N	5.58	129.49	117.20
2	B	138	ALA	CA-C-O	-5.58	108.37	120.10
2	B	223	LEU	CA-C-O	5.58	131.83	120.10
2	B	554	LYS	CA-C-O	-5.58	108.37	120.10
2	B	169	VAL	CA-C-O	-5.58	108.38	120.10
2	B	172	GLU	C-N-CA	-5.58	107.75	121.70
2	B	319	LEU	O-C-N	5.58	131.63	122.70
4	M	421	GLY	C-N-CD	5.58	140.12	128.40
1	A	160	ARG	O-C-N	5.58	131.62	122.70
1	A	507	GLN	C-N-CA	-5.58	107.75	121.70
4	M	387	GLU	O-C-N	-5.58	113.78	122.70
2	B	458	MET	C-N-CA	-5.58	107.76	121.70
4	M	292	PRO	C-N-CD	-5.58	108.33	120.60
1	A	191	GLN	C-N-CA	5.57	135.63	121.70
2	B	290	SER	N-CA-C	5.57	126.05	111.00
2	B	86	VAL	O-C-N	5.57	131.62	122.70
1	A	583	GLU	O-C-N	5.57	131.61	122.70
2	B	117	LEU	O-C-N	5.57	131.60	122.70
4	M	368	ASP	O-C-N	5.57	131.68	121.10
4	M	225	VAL	O-C-N	5.56	131.60	122.70
1	A	636	TYR	CB-CG-CD2	-5.56	117.66	121.00
1	A	588	LEU	CA-C-O	5.56	131.78	120.10

Continued on next page...

Continued from previous page...

Mol	Chain	Res	Type	Atoms	Z	Observed(°)	Ideal(°)
2	B	120	ILE	O-C-N	5.56	131.60	122.70
4	M	103	TYR	N-CA-CB	5.56	120.61	110.60
2	B	283	TYR	CB-CG-CD2	-5.55	117.67	121.00
1	A	265	GLN	CB-CA-C	5.55	121.50	110.40
4	M	284	SER	C-N-CD	5.55	140.06	128.40
2	B	551	LEU	CA-C-O	-5.55	108.44	120.10
2	B	605	PHE	CA-C-O	-5.55	108.45	120.10
2	B	194	ASP	O-C-N	5.55	131.58	122.70
2	B	492	LYS	O-C-N	5.54	131.57	122.70
1	A	229	ASN	C-N-CA	-5.54	98.71	122.00
1	A	242	GLU	CA-C-O	-5.54	108.46	120.10
2	B	340	ILE	O-C-N	5.54	131.57	122.70
2	B	365	PHE	O-C-N	5.54	131.57	122.70
4	M	385	ARG	CA-C-O	5.54	131.74	120.10
2	B	430	ILE	CA-C-O	-5.54	108.46	120.10
2	B	215	TYR	CA-C-O	-5.54	108.47	120.10
3	S	164	ASP	O-C-N	-5.54	113.84	122.70
1	A	294	SER	O-C-N	5.53	131.55	122.70
4	M	25	PRO	N-CA-C	-5.53	97.72	112.10
4	M	256	VAL	O-C-N	5.53	131.54	122.70
2	B	457	HIS	C-N-CA	-5.53	107.89	121.70
2	B	456	ASP	O-C-N	5.52	131.54	122.70
1	A	126	ASN	O-C-N	5.52	131.53	122.70
1	A	103	LYS	C-N-CA	-5.51	107.91	121.70
1	A	356	ILE	O-C-N	5.51	131.52	122.70
2	B	318	ILE	O-C-N	5.51	131.52	122.70
3	S	151	ALA	O-C-N	5.51	131.52	122.70
1	A	156	PRO	CA-C-O	5.51	133.43	120.20
3	S	109	LEU	C-N-CA	-5.51	107.93	121.70
2	B	204	ASP	CA-C-O	-5.51	108.54	120.10
1	A	165	ASP	O-C-N	5.50	131.50	122.70
1	A	278	ILE	CA-C-O	-5.50	108.55	120.10
2	B	120	ILE	CA-C-O	-5.50	108.55	120.10
1	A	456	ASP	O-C-N	5.50	131.50	122.70
2	B	334	MET	N-CA-C	5.50	125.84	111.00
1	A	558	VAL	CA-C-O	-5.50	108.56	120.10
1	A	125	THR	CA-C-O	-5.49	108.57	120.10
1	A	181	ALA	O-C-N	5.49	131.48	122.70
1	A	341	ILE	CA-C-O	-5.49	108.58	120.10
1	A	416	ILE	C-N-CA	-5.49	98.94	122.00
1	A	501	ASN	N-CA-CB	5.49	120.48	110.60
4	M	230	LYS	O-C-N	-5.49	113.92	122.70

Continued on next page...

Continued from previous page...

Mol	Chain	Res	Type	Atoms	Z	Observed(°)	Ideal(°)
2	B	275	ARG	CA-C-N	-5.49	105.13	117.20
1	A	129	LYS	O-C-N	5.49	131.48	122.70
4	M	441	GLY	CA-C-N	-5.49	105.13	117.20
2	B	451	MET	O-C-N	5.48	131.47	122.70
2	B	540	GLU	N-CA-C	5.48	125.80	111.00
2	B	586	SER	CA-C-O	-5.48	108.59	120.10
1	A	528	ASN	C-N-CA	-5.48	110.79	122.30
2	B	287	GLU	O-C-N	5.48	131.47	122.70
2	B	415	LEU	CA-C-O	-5.48	108.59	120.10
1	A	496	ILE	CA-C-O	-5.48	108.59	120.10
1	A	175	PRO	O-C-N	5.47	131.46	122.70
1	A	604	LEU	CA-C-O	-5.47	108.61	120.10
1	A	433	ILE	CA-C-O	-5.47	108.61	120.10
1	A	593	THR	O-C-N	5.47	131.46	122.70
2	B	178	ILE	CA-C-O	-5.47	108.61	120.10
4	M	90	PHE	CA-C-O	-5.47	108.61	120.10
2	B	225	LEU	CA-C-N	-5.47	105.17	117.20
4	M	343	ASN	C-N-CA	-5.47	108.04	121.70
2	B	321	CYS	O-C-N	5.46	131.44	122.70
2	B	354	GLY	N-CA-C	-5.46	99.44	113.10
2	B	436	LEU	CA-C-O	-5.46	108.62	120.10
3	S	74	GLN	CA-C-O	5.46	131.57	120.10
1	A	505	ASN	O-C-N	-5.46	113.97	122.70
3	S	124	ASN	O-C-N	5.46	131.44	122.70
4	M	85	GLY	CA-C-O	-5.46	110.78	120.60
4	M	103	TYR	CB-CA-C	5.46	121.31	110.40
1	A	319	LEU	CA-C-O	5.45	131.55	120.10
4	M	51	LEU	N-CA-CB	-5.45	99.50	110.40
3	S	98	ILE	CA-C-O	-5.45	108.67	120.10
4	M	88	ASP	O-C-N	5.45	131.41	122.70
1	A	308	ASP	N-CA-C	5.44	125.70	111.00
1	A	470	GLY	O-C-N	5.44	131.41	122.70
2	B	73	ASP	C-N-CA	-5.44	108.09	121.70
1	A	578	LEU	CA-C-O	-5.44	108.68	120.10
4	M	58	ARG	C-N-CA	-5.44	108.11	121.70
2	B	133	GLU	O-C-N	5.44	131.40	122.70
2	B	531	ARG	CA-C-O	-5.44	108.68	120.10
1	A	63	ASP	N-CA-C	5.43	125.67	111.00
2	B	323	ASN	CA-C-O	-5.43	108.69	120.10
3	S	158	LYS	CA-C-O	-5.43	108.69	120.10
4	M	377	LYS	N-CA-C	5.43	125.67	111.00
1	A	370	LYS	CA-C-O	-5.43	108.70	120.10

Continued on next page...

Continued from previous page...

Mol	Chain	Res	Type	Atoms	Z	Observed(°)	Ideal(°)
2	B	245	GLN	CA-C-O	-5.43	108.70	120.10
2	B	475	ILE	CA-C-O	-5.43	108.70	120.10
4	M	354	ASP	CB-CA-C	5.42	121.24	110.40
1	A	137	ASN	C-N-CA	-5.42	108.15	121.70
1	A	335	CYS	CA-C-O	-5.41	108.73	120.10
2	B	387	ASP	N-CA-C	5.41	125.62	111.00
1	A	244	LEU	CA-C-O	5.41	131.46	120.10
1	A	375	VAL	O-C-N	-5.41	114.04	122.70
1	A	419	ILE	CA-C-N	-5.41	105.30	117.20
4	M	57	GLY	O-C-N	-5.41	114.05	122.70
1	A	334	SER	O-C-N	5.41	131.35	122.70
3	S	37	GLU	CA-C-O	-5.41	108.75	120.10
1	A	255	ARG	O-C-N	5.41	131.35	122.70
1	A	466	ASP	N-CA-C	-5.41	96.40	111.00
4	M	87	LEU	O-C-N	5.41	131.35	122.70
4	M	279	ASN	CA-C-O	-5.40	108.76	120.10
2	B	109	ALA	CB-CA-C	-5.40	102.00	110.10
3	S	49	SER	CA-C-O	-5.40	108.76	120.10
1	A	611	LEU	CA-C-O	-5.40	108.77	120.10
1	A	139	ASP	CA-C-O	-5.40	108.77	120.10
1	A	391	LEU	CA-C-O	5.39	131.43	120.10
4	M	130	GLU	O-C-N	5.39	131.33	122.70
1	A	514	GLU	CA-C-O	-5.39	108.78	120.10
2	B	192	LEU	CA-C-O	-5.39	108.78	120.10
1	A	73	LYS	O-C-N	5.39	131.32	122.70
1	A	614	LEU	C-N-CA	-5.38	108.24	121.70
4	M	307	SER	O-C-N	5.38	131.31	122.70
2	B	127	LEU	C-N-CA	-5.38	108.25	121.70
1	A	94	VAL	CA-C-O	5.38	131.39	120.10
1	A	501	ASN	N-CA-C	-5.38	96.48	111.00
4	M	396	GLN	O-C-N	-5.38	114.10	122.70
1	A	80	TYR	CB-CA-C	5.38	121.15	110.40
1	A	312	ALA	CA-C-O	-5.38	108.81	120.10
2	B	420	ALA	N-CA-CB	-5.37	102.58	110.10
1	A	85	ALA	O-C-N	5.37	131.29	122.70
2	B	491	PHE	O-C-N	5.37	131.29	122.70
1	A	313	MET	CA-C-O	-5.37	108.83	120.10
2	B	493	LEU	CA-C-O	-5.37	108.83	120.10
4	M	450	GLU	O-C-N	-5.37	114.12	122.70
1	A	289	SER	C-N-CA	-5.36	108.29	121.70
1	A	346	THR	CA-C-O	5.36	131.36	120.10
2	B	357	GLU	O-C-N	5.36	131.28	122.70

Continued on next page...

Continued from previous page...

Mol	Chain	Res	Type	Atoms	Z	Observed(^{o})	Ideal(^{o})
1	A	90	HIS	O-C-N	5.36	131.27	122.70
2	B	392	SER	O-C-N	5.36	131.27	122.70
4	M	444	ALA	N-CA-CB	-5.36	102.60	110.10
4	M	104	PHE	CA-C-O	5.35	131.34	120.10
1	A	597	GLN	O-C-N	5.35	131.26	122.70
3	S	103	GLN	CA-C-O	-5.35	108.86	120.10
2	B	562	ASN	O-C-N	5.35	131.26	122.70
2	B	273	SER	C-N-CA	5.35	144.46	122.00
4	M	447	ILE	O-C-N	-5.35	114.15	122.70
1	A	518	CYS	O-C-N	5.34	131.25	122.70
2	B	196	LEU	CA-C-O	-5.34	108.88	120.10
1	A	455	MET	O-C-N	5.34	131.24	122.70
1	A	99	LYS	O-C-N	5.33	131.24	122.70
2	B	222	HIS	O-C-N	5.33	131.23	122.70
4	M	405	THR	N-CA-CB	-5.33	100.17	110.30
1	A	352	PHE	O-C-N	5.33	131.22	122.70
1	A	370	LYS	O-C-N	5.33	131.22	122.70
2	B	482	ASN	O-C-N	5.33	131.22	121.10
1	A	134	TYR	C-N-CA	-5.33	108.38	121.70
1	A	380	ASP	O-C-N	5.33	131.22	122.70
2	B	126	SER	CA-C-O	5.32	131.28	120.10
1	A	439	ASP	C-N-CA	5.32	135.00	121.70
4	M	61	GLU	N-CA-CB	5.32	120.17	110.60
1	A	85	ALA	N-CA-CB	-5.32	102.65	110.10
2	B	467	VAL	CA-C-O	-5.31	108.94	120.10
2	B	595	VAL	CA-C-O	-5.31	108.94	120.10
1	A	636	TYR	CD1-CG-CD2	5.31	123.74	117.90
1	A	295	VAL	O-C-N	5.31	131.20	122.70
2	B	472	VAL	C-N-CA	-5.31	108.43	121.70
1	A	216	SER	CA-C-O	-5.31	108.95	120.10
1	A	398	GLU	N-CA-C	5.31	125.33	111.00
2	B	219	TYR	N-CA-C	5.31	125.33	111.00
2	B	34	SER	O-C-N	5.30	131.19	122.70
4	M	296	LYS	C-N-CA	-5.30	108.44	121.70
1	A	193	PRO	CA-C-O	-5.30	107.49	120.20
2	B	383	VAL	CA-C-N	-5.30	105.55	117.20
2	B	489	ILE	CA-C-O	-5.30	108.98	120.10
2	B	283	TYR	CD1-CG-CD2	5.29	123.72	117.90
3	S	34	GLN	O-C-N	5.29	131.17	122.70
1	A	613	ALA	CA-C-O	-5.29	108.99	120.10
2	B	213	LEU	CA-C-O	-5.29	108.99	120.10
2	B	83	PHE	CA-C-N	-5.29	105.56	117.20

Continued on next page...

Continued from previous page...

Mol	Chain	Res	Type	Atoms	Z	Observed(°)	Ideal(°)
2	B	616	SER	O-C-N	5.29	131.16	122.70
1	A	177	ILE	CA-C-O	-5.28	109.00	120.10
1	A	390	THR	CA-C-O	-5.28	109.00	120.10
1	A	633	PHE	O-C-N	5.28	131.15	122.70
2	B	364	HIS	O-C-N	5.28	131.15	122.70
2	B	54	ARG	N-CA-C	5.28	125.26	111.00
1	A	512	LEU	O-C-N	5.28	131.15	122.70
2	B	351	GLU	CA-C-O	5.28	131.19	120.10
1	A	72	LEU	O-C-N	5.28	131.15	122.70
1	A	222	ILE	O-C-N	5.28	131.15	122.70
1	A	292	TYR	O-C-N	5.28	131.15	122.70
2	B	592	TYR	CA-C-O	-5.28	109.01	120.10
2	B	398	ILE	O-C-N	5.28	131.15	122.70
1	A	110	ALA	CA-C-O	5.28	131.18	120.10
1	A	280	GLU	O-C-N	5.28	131.14	122.70
1	A	221	VAL	CA-C-O	-5.27	109.03	120.10
2	B	609	ASP	O-C-N	5.27	131.14	122.70
2	B	494	ALA	CA-C-O	-5.27	109.04	120.10
2	B	566	ALA	N-CA-CB	-5.27	102.72	110.10
4	M	39	PRO	O-C-N	5.27	131.13	122.70
1	A	163	ALA	CA-C-O	5.27	131.16	120.10
1	A	429	VAL	CA-C-O	-5.27	109.04	120.10
3	S	18	LYS	CA-C-O	5.27	131.16	120.10
4	M	1	MET	C-N-CA	-5.27	108.53	121.70
4	M	59	ASP	N-CA-C	-5.27	96.78	111.00
2	B	608	ARG	O-C-N	5.26	131.12	122.70
2	B	555	LEU	CA-C-O	-5.26	109.05	120.10
4	M	7	ILE	CA-C-O	-5.26	109.05	120.10
2	B	399	LEU	O-C-N	-5.25	114.30	122.70
3	S	68	VAL	N-CA-CB	-5.25	99.95	111.50
1	A	254	ILE	O-C-N	5.25	131.10	122.70
2	B	470	ALA	O-C-N	5.25	131.09	122.70
1	A	305	GLU	CA-C-O	-5.25	109.08	120.10
2	B	68	SER	O-C-N	5.25	131.09	122.70
2	B	166	SER	C-N-CA	5.25	134.81	121.70
2	B	222	HIS	N-CA-C	5.24	125.15	111.00
1	A	146	ALA	CA-C-O	-5.24	109.09	120.10
2	B	482	ASN	CA-C-O	-5.24	109.09	120.10
3	S	153	VAL	CA-C-O	-5.24	109.09	120.10
1	A	383	ASN	CA-C-O	-5.24	109.10	120.10
2	B	280	PRO	N-CA-C	-5.24	98.48	112.10
4	M	16	PHE	O-C-N	-5.24	114.32	122.70

Continued on next page...

Continued from previous page...

Mol	Chain	Res	Type	Atoms	Z	Observed(°)	Ideal(°)
1	A	516	ILE	CA-C-O	-5.24	109.11	120.10
2	B	297	PRO	CA-C-O	-5.24	107.64	120.20
2	B	319	LEU	CA-C-O	-5.24	109.11	120.10
2	B	577	ASN	C-N-CA	-5.24	100.01	122.00
4	M	284	SER	CA-C-O	5.24	131.09	120.10
1	A	327	ASP	O-C-N	5.23	131.04	121.10
3	S	89	VAL	C-N-CA	-5.23	108.63	121.70
1	A	91	ILE	CA-C-O	-5.23	109.12	120.10
1	A	559	PHE	CA-C-O	-5.23	109.13	120.10
1	A	304	LEU	CA-C-N	5.22	128.69	117.20
4	M	234	ARG	O-C-N	-5.22	114.34	122.70
1	A	226	SER	C-N-CA	-5.22	108.64	121.70
1	A	337	LEU	CA-C-O	-5.22	109.13	120.10
3	S	78	LYS	CA-C-O	5.22	131.07	120.10
2	B	500	GLN	N-CA-C	5.22	125.10	111.00
3	S	138	GLY	C-N-CA	5.22	133.26	122.30
2	B	286	ILE	CA-C-N	-5.22	105.72	117.20
4	M	21	GLY	CA-C-N	-5.22	105.72	117.20
1	A	172	SER	C-N-CA	-5.22	108.66	121.70
1	A	634	ASN	CA-C-O	-5.21	109.15	120.10
2	B	63	MET	CA-C-O	-5.21	109.16	120.10
1	A	144	GLY	O-C-N	5.21	131.03	122.70
1	A	620	GLY	O-C-N	5.21	131.03	122.70
2	B	248	LEU	CA-C-O	-5.20	109.17	120.10
2	B	350	THR	N-CA-C	5.20	125.05	111.00
4	M	55	MET	O-C-N	-5.20	114.37	122.70
2	B	451	MET	CA-C-O	-5.20	109.18	120.10
2	B	216	LYS	CA-C-O	5.20	131.02	120.10
3	S	154	ASP	CA-C-O	-5.20	109.18	120.10
2	B	118	LEU	O-C-N	5.20	131.01	122.70
2	B	544	THR	CA-C-O	-5.20	109.19	120.10
4	M	60	LEU	CA-C-N	-5.20	105.77	117.20
1	A	562	TRP	CA-C-O	-5.19	109.19	120.10
2	B	48	VAL	CA-C-O	-5.19	109.19	120.10
2	B	533	LEU	O-C-N	5.19	131.01	122.70
4	M	122	SER	CA-C-O	-5.19	109.19	120.10
2	B	112	ASP	CA-C-O	-5.19	109.20	120.10
4	M	77	LEU	O-C-N	-5.19	114.39	122.70
4	M	331	LEU	CA-C-N	-5.19	105.82	116.20
1	A	121	LEU	CA-C-O	-5.19	109.20	120.10
1	A	526	VAL	C-N-CA	5.19	134.67	121.70
1	A	422	GLU	O-C-N	5.19	131.00	122.70

Continued on next page...

Continued from previous page...

Mol	Chain	Res	Type	Atoms	Z	Observed(°)	Ideal(°)
1	A	314	ALA	O-C-N	5.19	131.00	122.70
1	A	502	ASP	O-C-N	5.19	131.00	122.70
2	B	606	ASP	O-C-N	5.18	130.99	122.70
2	B	360	LEU	CA-C-O	-5.18	109.22	120.10
4	M	379	LEU	CA-C-O	5.18	130.97	120.10
2	B	584	SER	CB-CA-C	-5.18	100.26	110.10
1	A	239	LEU	O-C-N	5.17	130.98	122.70
1	A	437	SER	N-CA-C	5.17	124.96	111.00
1	A	115	TYR	C-N-CA	5.17	134.62	121.70
2	B	411	ILE	CA-C-O	-5.17	109.25	120.10
4	M	59	ASP	CA-C-O	-5.17	109.25	120.10
1	A	440	ASN	N-CA-C	-5.16	97.06	111.00
2	B	391	ALA	CA-C-O	-5.16	109.26	120.10
1	A	323	CYS	O-C-N	-5.16	114.44	122.70
1	A	635	ALA	CA-C-O	-5.16	109.27	120.10
1	A	493	ALA	CA-C-O	-5.16	109.27	120.10
3	S	164	ASP	CA-C-N	5.16	128.55	117.20
3	S	54	PRO	CA-C-N	5.16	131.53	117.10
1	A	566	PHE	O-C-N	5.15	130.94	122.70
1	A	230	PRO	O-C-N	5.15	130.94	122.70
2	B	242	SER	N-CA-C	5.15	124.90	111.00
2	B	116	THR	CA-C-O	-5.14	109.30	120.10
4	M	46	SER	CA-C-N	5.14	128.51	117.20
2	B	552	SER	CA-C-O	-5.14	109.31	120.10
2	B	615	SER	CA-C-O	-5.14	109.31	120.10
2	B	60	ARG	CA-C-O	-5.14	109.31	120.10
2	B	84	ALA	O-C-N	5.14	130.92	122.70
2	B	518	ILE	O-C-N	5.14	130.92	122.70
3	S	141	VAL	N-CA-C	-5.14	97.13	111.00
4	M	309	GLN	O-C-N	5.13	130.91	122.70
1	A	71	VAL	O-C-N	5.13	130.91	122.70
2	B	548	ILE	CA-C-O	-5.13	109.32	120.10
2	B	614	ILE	CA-C-O	-5.13	109.32	120.10
1	A	434	SER	CA-C-O	-5.13	109.33	120.10
2	B	345	ARG	O-C-N	5.13	130.91	122.70
3	S	78	LYS	O-C-N	-5.13	114.50	122.70
2	B	530	LEU	CA-C-O	-5.13	109.34	120.10
1	A	389	GLN	O-C-N	5.12	130.90	122.70
2	B	439	CYS	CA-C-O	-5.12	109.34	120.10
1	A	302	ASN	N-CA-CB	5.12	119.82	110.60
1	A	219	VAL	O-C-N	5.12	130.89	122.70
2	B	210	CYS	CA-C-O	-5.12	109.34	120.10

Continued on next page...

Continued from previous page...

Mol	Chain	Res	Type	Atoms	Z	Observed(°)	Ideal(°)
4	M	278	ILE	C-N-CA	-5.12	108.90	121.70
1	A	602	GLU	CA-C-O	-5.12	109.35	120.10
2	B	61	ASP	O-C-N	5.12	130.89	122.70
1	A	178	ARG	O-C-N	5.12	130.89	122.70
2	B	176	ALA	CA-C-O	-5.12	109.36	120.10
4	M	117	ASN	C-N-CA	5.12	134.49	121.70
1	A	193	PRO	O-C-N	5.12	130.88	122.70
2	B	94	ASP	O-C-N	5.12	130.88	122.70
2	B	340	ILE	CA-C-O	-5.11	109.36	120.10
2	B	532	ARG	CA-C-O	-5.11	109.36	120.10
1	A	387	ILE	CA-C-O	-5.11	109.37	120.10
1	A	555	LEU	CA-C-O	-5.11	109.37	120.10
2	B	589	SER	CA-C-O	-5.11	109.37	120.10
1	A	479	ASN	CA-C-O	-5.11	109.38	120.10
2	B	474	VAL	CA-C-O	-5.11	109.38	120.10
2	B	588	ILE	CA-C-O	-5.11	109.38	120.10
3	S	36	TYR	CA-C-O	-5.11	109.38	120.10
3	S	146	VAL	CA-C-O	-5.11	109.38	120.10
4	M	110	SER	CA-C-O	-5.11	109.38	120.10
2	B	274	PRO	CA-N-CD	-5.10	104.36	111.50
2	B	401	THR	C-N-CA	5.10	134.46	121.70
2	B	292	GLU	CA-C-N	-5.10	105.98	117.20
2	B	577	ASN	N-CA-CB	-5.10	101.42	110.60
1	A	560	SER	O-C-N	5.10	130.85	122.70
1	A	445	ASN	N-CA-C	-5.09	97.25	111.00
2	B	317	VAL	CA-C-O	-5.09	109.40	120.10
2	B	443	SER	CA-C-N	-5.09	105.99	117.20
1	A	85	ALA	CA-C-N	-5.09	106.00	117.20
2	B	55	ASN	C-N-CA	5.09	134.43	121.70
2	B	336	ASN	O-C-N	5.09	130.85	122.70
1	A	174	ARG	C-N-CD	-5.09	109.41	120.60
3	S	12	CYS	C-N-CA	-5.09	108.98	121.70
2	B	450	VAL	CA-C-O	-5.08	109.44	120.10
4	M	63	TYR	CG-CD1-CE1	-5.08	117.24	121.30
1	A	441	TYR	N-CA-C	-5.07	97.31	111.00
4	M	24	ALA	C-N-CD	5.07	139.04	128.40
2	B	132	SER	CA-C-O	5.07	130.74	120.10
2	B	69	ILE	C-N-CA	-5.06	109.04	121.70
4	M	282	VAL	C-N-CA	-5.06	109.05	121.70
2	B	502	SER	C-N-CA	-5.06	109.06	121.70
1	A	174	ARG	O-C-N	5.06	130.71	121.10
2	B	426	GLU	CA-C-O	-5.05	109.49	120.10

Continued on next page...

Continued from previous page...

Mol	Chain	Res	Type	Atoms	Z	Observed(°)	Ideal(°)
3	S	132	LEU	CA-C-O	-5.05	109.49	120.10
2	B	138	ALA	O-C-N	5.05	130.78	122.70
4	M	323	MET	C-N-CA	-5.05	109.08	121.70
1	A	253	ILE	CA-C-O	-5.05	109.50	120.10
2	B	596	LEU	CA-C-O	-5.04	109.51	120.10
3	S	63	ASN	N-CA-CB	5.04	119.68	110.60
3	S	89	VAL	CA-C-O	5.04	130.69	120.10
2	B	350	THR	C-N-CA	5.04	134.30	121.70
1	A	375	VAL	CA-C-O	5.04	130.68	120.10
2	B	134	LEU	CA-C-O	-5.04	109.52	120.10
1	A	239	LEU	C-N-CA	-5.04	109.10	121.70
1	A	469	LEU	CA-C-N	-5.04	106.13	116.20
2	B	229	HIS	O-C-N	5.03	130.75	122.70
2	B	435	SER	CA-C-O	-5.03	109.53	120.10
2	B	465	ALA	O-C-N	5.03	130.75	122.70
4	M	405	THR	CA-C-O	5.03	130.66	120.10
1	A	425	LYS	CA-C-O	-5.03	109.55	120.10
3	S	29	LYS	CA-C-O	-5.03	109.55	120.10
4	M	266	ASP	C-N-CA	5.02	134.24	121.70
2	B	444	THR	C-N-CA	-5.02	109.16	121.70
2	B	492	LYS	CA-C-O	-5.02	109.57	120.10
2	B	503	LEU	CA-C-N	-5.01	106.17	117.20
4	M	136	VAL	O-C-N	5.01	130.71	122.70
1	A	535	ILE	CA-C-N	-5.00	106.19	117.20
4	M	103	TYR	CA-C-O	5.00	130.61	120.10

All (2) chirality outliers are listed below:

Mol	Chain	Res	Type	Atom
3	S	69	ASN	CA
4	M	22	ALA	CA

All (127) planarity outliers are listed below:

Mol	Chain	Res	Type	Group
1	A	117	ASP	Mainchain
1	A	174	ARG	Mainchain
1	A	192	TYR	Mainchain
1	A	199	ASN	Mainchain
1	A	204	VAL	Mainchain
1	A	219	VAL	Mainchain
1	A	233	PHE	Mainchain

Continued on next page...

Continued from previous page...

Mol	Chain	Res	Type	Group
1	A	240	LEU	Mainchain
1	A	244	LEU	Mainchain
1	A	260	PHE	Mainchain
1	A	275	LEU	Mainchain
1	A	277	LYS	Mainchain
1	A	278	ILE	Mainchain
1	A	282	MET	Mainchain
1	A	288	THR	Mainchain
1	A	289	SER	Mainchain
1	A	290	VAL	Mainchain
1	A	298	ILE	Mainchain
1	A	302	ASN	Mainchain
1	A	306	GLU	Mainchain
1	A	319	LEU	Mainchain
1	A	320	HIS	Mainchain
1	A	323	CYS	Mainchain
1	A	325	SER	Mainchain
1	A	328	PRO	Mainchain
1	A	350	SER	Mainchain
1	A	381	GLU	Mainchain
1	A	399	ASP	Peptide
1	A	400	VAL	Peptide
1	A	436	CYS	Mainchain
1	A	441	TYR	Mainchain
1	A	462	GLN	Mainchain
1	A	487	MET	Mainchain
1	A	500	SER	Mainchain
1	A	501	ASN	Mainchain
1	A	505	ASN	Mainchain
1	A	506	LYS	Mainchain
1	A	527	GLU	Mainchain
1	A	528	ASN	Mainchain
1	A	529	GLY	Mainchain
1	A	531	ASP	Mainchain
1	A	535	ILE	Mainchain
1	A	536	MET	Mainchain
1	A	538	GLU	Mainchain
1	A	539	ASN	Mainchain
1	A	565	ASN	Mainchain
1	A	569	ASP	Mainchain
1	A	571	ARG	Mainchain
1	A	586	GLU	Mainchain

Continued on next page...

Continued from previous page...

Mol	Chain	Res	Type	Group
1	A	588	LEU	Mainchain
1	A	64	LEU	Mainchain
1	A	80	TYR	Mainchain
1	A	84	MET	Mainchain
1	A	94	VAL	Mainchain
2	B	126	SER	Mainchain
2	B	142	LEU	Mainchain
2	B	147	MET	Mainchain
2	B	181	TYR	Mainchain
2	B	186	ASN	Mainchain
2	B	237	ILE	Mainchain
2	B	267	ASP	Mainchain
2	B	278	PRO	Peptide
2	B	293	VAL	Mainchain
2	B	310	ILE	Mainchain
2	B	326	TYR	Mainchain
2	B	375	LEU	Mainchain
2	B	377	TYR	Mainchain
2	B	384	PHE	Mainchain
2	B	404	ASN	Mainchain
2	B	444	THR	Mainchain
2	B	459	GLU	Mainchain
2	B	485	LYS	Mainchain
2	B	497	LEU	Mainchain
2	B	523	PHE	Mainchain
2	B	536	ASN	Mainchain
2	B	557	SER	Mainchain
2	B	56	SER	Mainchain
2	B	565	GLN	Mainchain
2	B	569	THR	Mainchain
2	B	573	GLU	Mainchain
2	B	581	TYR	Mainchain
2	B	584	SER	Mainchain
2	B	78	ASP	Mainchain
2	B	82	TYR	Mainchain
2	B	88	LYS	Mainchain
4	M	102	GLU	Mainchain
4	M	103	TYR	Mainchain
4	M	104	PHE	Mainchain
4	M	105	ASP	Peptide
4	M	131	ALA	Mainchain
4	M	132	GLY	Mainchain

Continued on next page...

Continued from previous page...

Mol	Chain	Res	Type	Group
4	M	134	PRO	Mainchain
4	M	265	ASN	Mainchain
4	M	284	SER	Mainchain
4	M	292	PRO	Peptide
4	M	40	GLN	Mainchain
4	M	405	THR	Mainchain
4	M	421	GLY	Mainchain
4	M	426	LYS	Peptide
4	M	445	SER	Peptide
4	M	45	SER	Peptide
4	M	456	SER	Mainchain
4	M	462	LYS	Peptide
4	M	477	GLY	Peptide
4	M	51	LEU	Peptide
4	M	57	GLY	Mainchain
4	M	79	SER	Mainchain
4	M	8	THR	Mainchain
4	M	85	GLY	Mainchain
3	S	101	LEU	Mainchain
3	S	102	ILE	Mainchain
3	S	135	ILE	Mainchain
3	S	161	GLU	Mainchain
3	S	163	THR	Peptide
3	S	167	ILE	Peptide
3	S	22	PRO	Mainchain
3	S	43	ASN	Mainchain
3	S	46	PHE	Mainchain
3	S	50	PHE	Mainchain
3	S	53	THR	Mainchain
3	S	56	SER	Mainchain
3	S	57	LEU	Mainchain
3	S	58	LEU	Peptide
3	S	64	ASN	Mainchain
3	S	66	ASP	Mainchain
3	S	69	ASN	Mainchain
3	S	99	LEU	Mainchain

5.2 Too-close contacts (i)

In the following table, the Non-H and H(model) columns list the number of non-hydrogen atoms and hydrogen atoms in the chain respectively. The H(added) column lists the number of hydrogen atoms added and optimized by MolProbity. The Clashes column lists the number of clashes within

the asymmetric unit, whereas Symm-Clashes lists symmetry-related clashes.

Mol	Chain	Non-H	H(model)	H(added)	Clashes	Symm-Clashes
1	A	4625	0	4703	1294	0
2	B	4963	0	4983	2778	0
3	S	1358	0	1335	469	0
4	M	3158	0	3098	1734	0
All	All	14104	0	14119	5611	0

The all-atom clashscore is defined as the number of clashes found per 1000 atoms (including hydrogen atoms). The all-atom clashscore for this structure is 199.

All (5611) close contacts within the same asymmetric unit are listed below, sorted by their clash magnitude.

Atom-1	Atom-2	Interatomic distance (Å)	Clash overlap (Å)
2:B:17:VAL:HG21	2:B:35:TYR:CD2	1.29	1.68
2:B:243:TRP:CH2	4:M:98:ARG:HD3	1.17	1.63
2:B:106:LEU:CD1	2:B:144:ASP:HB3	1.23	1.62
1:A:606:PHE:CZ	1:A:633:PHE:HB2	1.23	1.62
2:B:578:PRO:HD2	2:B:581:TYR:CE2	1.21	1.61
2:B:106:LEU:CD2	4:M:130:GLU:HB3	1.26	1.61
2:B:2:VAL:HG11	4:M:54:SER:CB	1.24	1.60
2:B:106:LEU:HD13	2:B:144:ASP:CB	1.32	1.60
2:B:479:VAL:CG1	2:B:486:HIS:CE1	1.80	1.60
4:M:131:ALA:CA	4:M:131:ALA:CB	1.80	1.59
4:M:344:ILE:CG2	4:M:347:PHE:HB3	1.24	1.58
2:B:523:PHE:HZ	2:B:580:TYR:CE2	1.17	1.58
2:B:73:ASP:HA	4:M:19:LEU:CD2	1.29	1.58
2:B:403:ILE:HD13	2:B:439:CYS:CA	1.34	1.57
2:B:216:LYS:CB	2:B:251:LEU:HD13	1.12	1.57
2:B:226:LEU:HD23	2:B:255:TYR:CD1	1.38	1.55
2:B:17:VAL:CG2	2:B:35:TYR:HD2	1.20	1.54
4:M:282:VAL:N	4:M:282:VAL:CA	1.69	1.54
1:A:594:PHE:HE2	2:B:477:MET:SD	1.28	1.53
4:M:244:VAL:HA	4:M:472:TYR:CD2	1.37	1.52
2:B:106:LEU:HD23	4:M:130:GLU:CB	1.05	1.51
2:B:107:ARG:CZ	4:M:20:LEU:HD23	1.37	1.51
4:M:281:GLY:CA	4:M:281:GLY:C	1.78	1.51
2:B:73:ASP:CA	4:M:19:LEU:HD22	1.11	1.51
2:B:1:MET:SD	4:M:39:PRO:HD2	1.48	1.50
3:S:64:ASN:CA	3:S:64:ASN:C	1.80	1.49
2:B:72:SER:CA	4:M:17:GLN:HE22	1.25	1.49
2:B:512:VAL:HG13	2:B:533:LEU:CD1	1.40	1.47

Continued on next page...

Continued from previous page...

Atom-1	Atom-2	Interatomic distance (Å)	Clash overlap (Å)
2:B:72:SER:HA	4:M:17:GLN:NE2	1.28	1.47
1:A:329:ASN:CA	3:S:50:PHE:CZ	1.82	1.47
2:B:219:TYR:CE2	2:B:226:LEU:HD13	1.45	1.47
2:B:486:HIS:CE1	2:B:518:ILE:HD13	1.45	1.46
1:A:405:THR:HA	2:B:7:ARG:NH2	1.24	1.46
1:A:406:GLY:C	3:S:64:ASN:HD21	1.14	1.45
4:M:432:THR:OG1	4:M:480:GLN:CG	1.63	1.45
2:B:230:PHE:CZ	2:B:252:LEU:HD22	1.49	1.45
2:B:211:ALA:CB	2:B:233:TYR:OH	1.64	1.45
3:S:163:THR:CA	3:S:163:THR:C	1.83	1.45
2:B:73:ASP:CA	4:M:19:LEU:CD2	1.87	1.45
4:M:245:ASP:O	4:M:472:TYR:CE1	1.68	1.44
2:B:12:LEU:HD13	4:M:13:LYS:CG	1.47	1.44
2:B:14:THR:HG22	2:B:36:THR:CG2	1.47	1.44
2:B:106:LEU:HD23	4:M:130:GLU:CA	1.46	1.43
2:B:224:GLU:CB	2:B:259:TYR:OH	1.64	1.43
2:B:374:PHE:CE2	2:B:402:LEU:CD1	1.77	1.43
1:A:328:PRO:C	3:S:50:PHE:HZ	1.20	1.43
1:A:251:TRP:CE3	3:S:97:ALA:CA	1.92	1.43
1:A:506:LYS:NZ	4:M:82:LYS:HD2	1.28	1.43
2:B:215:TYR:CD2	2:B:233:TYR:HE2	1.37	1.43
2:B:423:HIS:NE2	4:M:365:GLU:HB3	1.28	1.43
2:B:523:PHE:CZ	2:B:580:TYR:CE2	2.06	1.43
2:B:256:CYS:SG	2:B:328:LEU:HD22	1.57	1.43
2:B:223:LEU:HD11	2:B:258:GLN:CB	1.46	1.42
2:B:107:ARG:HH22	4:M:20:LEU:CD2	1.31	1.42
2:B:293:VAL:O	2:B:299:LEU:CG	1.65	1.42
2:B:337:THR:CA	2:B:373:LEU:HD11	0.97	1.42
4:M:347:PHE:CE1	4:M:350:VAL:CG1	2.02	1.42
2:B:12:LEU:HD13	4:M:13:LYS:CB	1.48	1.41
2:B:337:THR:HA	2:B:373:LEU:CD1	0.93	1.41
2:B:564:LYS:CD	2:B:621:GLY:O	1.67	1.41
2:B:437:SER:CB	2:B:474:VAL:HG13	1.49	1.41
2:B:20:ARG:NH1	4:M:118:TYR:HB3	1.09	1.41
2:B:158:VAL:HG11	2:B:177:ILE:CG1	1.45	1.41
2:B:223:LEU:HD13	2:B:259:TYR:N	1.15	1.41
2:B:106:LEU:CG	4:M:130:GLU:HB3	1.49	1.40
2:B:178:ILE:CG2	2:B:217:GLU:HB2	1.48	1.40
1:A:595:GLU:OE1	2:B:513:TRP:CZ3	1.73	1.40
2:B:243:TRP:CH2	4:M:98:ARG:CD	2.02	1.40
2:B:20:ARG:HH12	4:M:118:TYR:CB	1.31	1.40

Continued on next page...

Continued from previous page...

Atom-1	Atom-2	Interatomic distance (Å)	Clash overlap (Å)
2:B:11:ALA:HA	2:B:40:GLN:NE2	1.18	1.40
2:B:403:ILE:CD1	2:B:439:CYS:CA	2.00	1.40
2:B:374:PHE:HZ	2:B:381:PHE:CD2	1.40	1.40
2:B:578:PRO:CD	2:B:581:TYR:CE2	2.02	1.40
2:B:259:TYR:O	2:B:261:PRO:CD	1.66	1.39
1:A:595:GLU:CD	2:B:513:TRP:CZ3	1.95	1.39
2:B:178:ILE:HG13	2:B:214:ALA:C	1.41	1.39
2:B:25:VAL:HG22	2:B:30:LEU:C	1.13	1.39
2:B:107:ARG:NH1	4:M:20:LEU:HD23	1.32	1.39
2:B:479:VAL:HG11	2:B:486:HIS:NE2	1.34	1.39
2:B:278:PRO:HD2	2:B:292:GLU:CB	1.54	1.38
1:A:329:ASN:N	3:S:50:PHE:CZ	1.87	1.38
2:B:28:SER:CB	2:B:30:LEU:O	1.69	1.38
2:B:107:ARG:NH2	4:M:20:LEU:HD23	1.36	1.37
2:B:252:LEU:HD13	2:B:302:PHE:CD1	1.59	1.37
2:B:290:SER:O	2:B:292:GLU:N	1.56	1.37
2:B:293:VAL:O	2:B:299:LEU:CD1	1.69	1.37
2:B:403:ILE:CD1	2:B:439:CYS:O	1.69	1.37
2:B:337:THR:CB	2:B:373:LEU:HD11	1.55	1.37
2:B:396:ILE:CG2	2:B:432:ALA:HA	1.54	1.37
1:A:595:GLU:OE2	2:B:513:TRP:CZ3	1.77	1.37
2:B:12:LEU:CD1	4:M:13:LYS:HA	1.54	1.37
2:B:193:LEU:CD2	2:B:225:LEU:CB	2.00	1.37
4:M:219:LEU:CB	4:M:472:TYR:O	1.71	1.36
1:A:407:SER:C	3:S:64:ASN:HD22	1.24	1.36
2:B:12:LEU:CD1	4:M:13:LYS:HG3	1.53	1.36
2:B:38:TYR:CE2	2:B:43:ASN:O	1.78	1.36
4:M:222:PHE:O	4:M:479:PHE:CE1	1.79	1.35
2:B:403:ILE:HD13	2:B:439:CYS:CB	1.56	1.35
4:M:245:ASP:N	4:M:472:TYR:CD1	1.93	1.35
2:B:28:SER:OG	2:B:30:LEU:C	1.64	1.35
2:B:252:LEU:HB2	2:B:302:PHE:CZ	1.60	1.35
1:A:179:LYS:CE	3:S:137:GLN:O	1.74	1.34
2:B:479:VAL:HG13	2:B:486:HIS:ND1	1.41	1.34
1:A:595:GLU:OE2	2:B:513:TRP:CE3	1.78	1.34
1:A:503:ASN:HA	4:M:59:ASP:O	1.24	1.34
1:A:504:ILE:HA	4:M:59:ASP:OD2	1.23	1.34
1:A:594:PHE:CE2	2:B:477:MET:SD	2.18	1.34
2:B:25:VAL:CG2	2:B:30:LEU:C	1.94	1.34
2:B:512:VAL:CG1	2:B:533:LEU:HD13	1.55	1.34
3:S:29:LYS:NZ	3:S:33:GLU:OE2	1.59	1.34

Continued on next page...

Continued from previous page...

Atom-1	Atom-2	Interatomic distance (Å)	Clash overlap (Å)
1:A:606:PHE:HZ	1:A:633:PHE:CB	1.38	1.33
2:B:216:LYS:CB	2:B:251:LEU:CD1	2.03	1.33
2:B:213:LEU:CD1	4:M:135:ASN:OD1	1.74	1.33
2:B:501:THR:O	2:B:508:ARG:NH2	1.59	1.33
1:A:638:LEU:HD21	2:B:561:ASP:N	1.43	1.33
2:B:374:PHE:CE2	2:B:402:LEU:HD11	1.15	1.33
2:B:403:ILE:HD11	2:B:439:CYS:O	1.16	1.33
2:B:162:VAL:HG21	2:B:195:ILE:CG2	1.56	1.32
2:B:437:SER:HB2	2:B:474:VAL:CG1	1.59	1.32
1:A:504:ILE:HD13	4:M:59:ASP:OD2	1.20	1.32
1:A:606:PHE:CZ	1:A:633:PHE:CB	2.12	1.32
2:B:73:ASP:N	4:M:19:LEU:CD2	1.89	1.32
2:B:226:LEU:CD2	2:B:255:TYR:CD1	2.10	1.32
2:B:295:ASN:O	2:B:300:ASP:CB	1.75	1.32
2:B:230:PHE:CE2	2:B:252:LEU:HD23	1.63	1.32
2:B:259:TYR:O	2:B:261:PRO:N	1.60	1.32
2:B:276:SER:O	2:B:295:ASN:HB2	1.19	1.32
2:B:9:ALA:CB	4:M:14:LEU:HB2	1.57	1.32
2:B:193:LEU:HD22	2:B:225:LEU:CB	1.26	1.32
4:M:435:LEU:O	4:M:479:PHE:CD1	1.82	1.32
2:B:106:LEU:CD1	2:B:144:ASP:CB	1.93	1.31
2:B:224:GLU:HA	2:B:259:TYR:CZ	1.10	1.31
2:B:375:LEU:CD1	2:B:404:ASN:HD22	1.40	1.31
2:B:433:VAL:HG11	2:B:471:TYR:CA	1.58	1.31
4:M:41:LEU:CD1	4:M:52:ASP:H	1.39	1.31
2:B:9:ALA:O	4:M:14:LEU:HB3	1.27	1.31
2:B:375:LEU:CD1	2:B:404:ASN:ND2	1.93	1.31
2:B:17:VAL:CG2	2:B:35:TYR:CD2	1.97	1.31
2:B:479:VAL:HG13	2:B:486:HIS:CE1	1.43	1.31
4:M:223:HIS:HA	4:M:479:PHE:CD1	1.62	1.31
1:A:251:TRP:CE3	3:S:97:ALA:HA	1.02	1.31
2:B:38:TYR:OH	2:B:46:GLN:CG	1.78	1.31
2:B:400:SER:HB3	2:B:435:SER:CB	1.60	1.31
4:M:243:ILE:O	4:M:472:TYR:CB	1.77	1.31
2:B:278:PRO:CD	2:B:292:GLU:HB2	1.60	1.31
2:B:396:ILE:HG21	2:B:432:ALA:CA	1.61	1.31
2:B:243:TRP:CZ3	4:M:98:ARG:CD	2.13	1.30
1:A:407:SER:O	3:S:64:ASN:ND2	1.63	1.30
2:B:5:ILE:CG2	4:M:42:LEU:HD11	1.61	1.30
2:B:12:LEU:CG	4:M:13:LYS:HG3	1.59	1.30
2:B:274:PRO:HG2	2:B:295:ASN:CB	1.61	1.30

Continued on next page...

Continued from previous page...

Atom-1	Atom-2	Interatomic distance (Å)	Clash overlap (Å)
1:A:506:LYS:NZ	4:M:82:LYS:CD	1.94	1.30
4:M:344:ILE:CG2	4:M:347:PHE:CB	2.09	1.30
1:A:503:ASN:OD1	4:M:60:LEU:CA	1.79	1.30
2:B:234:CYS:O	2:B:237:ILE:HG22	1.18	1.30
2:B:274:PRO:CB	2:B:295:ASN:OD1	1.80	1.30
1:A:503:ASN:OD1	4:M:60:LEU:HD23	1.31	1.30
2:B:200:MET:SD	2:B:229:HIS:O	0.91	1.30
2:B:337:THR:HA	2:B:373:LEU:CG	1.60	1.30
2:B:75:ASP:OD1	4:M:24:ALA:HB3	1.16	1.29
4:M:355:ASP:OD2	4:M:357:LYS:NZ	1.61	1.29
1:A:402:ILE:CD1	3:S:62:GLU:O	1.79	1.29
2:B:73:ASP:HB3	4:M:25:PRO:O	1.32	1.29
2:B:230:PHE:CE2	2:B:252:LEU:CD2	2.16	1.29
1:A:506:LYS:HE2	4:M:82:LYS:CB	1.62	1.28
4:M:219:LEU:CD1	4:M:472:TYR:O	1.81	1.28
1:A:556:VAL:HG21	1:A:603:VAL:CG2	1.63	1.28
2:B:181:TYR:CE1	2:B:222:HIS:CD2	2.20	1.28
2:B:216:LYS:HE3	4:M:133:GLU:OE2	1.32	1.28
2:B:256:CYS:SG	2:B:328:LEU:CD2	2.21	1.28
2:B:274:PRO:CG	2:B:295:ASN:OD1	1.80	1.28
2:B:433:VAL:CG1	2:B:471:TYR:HA	1.63	1.28
4:M:218:LEU:HA	4:M:472:TYR:CE2	1.67	1.28
2:B:230:PHE:CE1	2:B:234:CYS:SG	2.26	1.28
2:B:296:ASP:OD1	2:B:297:PRO:HD2	1.22	1.28
1:A:462:GLN:O	4:M:58:ARG:HA	1.24	1.28
2:B:25:VAL:CG2	2:B:32:GLU:H	1.47	1.28
2:B:73:ASP:OD1	4:M:19:LEU:HD13	1.26	1.28
2:B:219:TYR:CE2	2:B:226:LEU:CD1	2.14	1.27
1:A:402:ILE:CG2	3:S:62:GLU:O	1.81	1.27
1:A:402:ILE:HG21	3:S:62:GLU:O	1.16	1.27
2:B:155:LEU:HD23	2:B:192:LEU:CD1	1.62	1.27
2:B:274:PRO:HG2	2:B:295:ASN:CG	1.52	1.27
2:B:17:VAL:HG21	2:B:35:TYR:CE2	1.69	1.27
2:B:28:SER:O	2:B:58:GLU:HG2	1.12	1.27
2:B:38:TYR:CE2	2:B:43:ASN:N	2.01	1.27
1:A:186:PHE:CE2	1:A:224:GLU:CB	2.17	1.27
2:B:347:VAL:HG22	2:B:359:LEU:CB	1.64	1.27
1:A:405:THR:CA	2:B:7:ARG:HH21	1.46	1.26
2:B:374:PHE:CZ	2:B:381:PHE:CD2	2.21	1.26
4:M:218:LEU:O	4:M:441:GLY:N	1.64	1.26
2:B:278:PRO:HD2	2:B:292:GLU:CG	1.66	1.26

Continued on next page...

Continued from previous page...

Atom-1	Atom-2	Interatomic distance (Å)	Clash overlap (Å)
4:M:96:ILE:HG21	4:M:125:PHE:CZ	1.68	1.26
4:M:223:HIS:HA	4:M:479:PHE:CE1	1.70	1.26
2:B:231:ARG:NH2	2:B:279:LEU:HD21	1.47	1.26
1:A:333:ILE:CD1	3:S:95:GLU:OE1	1.83	1.26
2:B:403:ILE:CD1	2:B:439:CYS:C	2.02	1.26
2:B:567:GLN:O	2:B:569:THR:N	1.68	1.26
2:B:73:ASP:OD1	4:M:24:ALA:HB1	1.12	1.26
2:B:200:MET:CE	2:B:229:HIS:O	1.81	1.26
2:B:479:VAL:CG1	2:B:486:HIS:NE2	1.92	1.26
2:B:309:LEU:HB3	2:B:317:VAL:CG1	1.66	1.25
2:B:418:TYR:OH	2:B:432:ALA:HB2	1.34	1.25
2:B:74:ASP:O	2:B:77:ILE:HG12	1.15	1.25
2:B:22:ALA:HB2	2:B:33:SER:OG	1.37	1.25
2:B:178:ILE:HG13	2:B:214:ALA:CA	1.66	1.25
2:B:73:ASP:OD1	4:M:19:LEU:CD1	1.83	1.25
2:B:219:TYR:CZ	2:B:226:LEU:HB2	1.71	1.25
2:B:25:VAL:HG23	2:B:32:GLU:N	1.53	1.24
2:B:144:ASP:CG	4:M:131:ALA:HA	1.57	1.24
2:B:219:TYR:CD2	2:B:226:LEU:HD22	1.71	1.24
2:B:243:TRP:CZ3	4:M:98:ARG:HD3	1.72	1.24
2:B:556:LEU:CA	2:B:588:ILE:HD11	1.65	1.24
4:M:65:TYR:CZ	4:M:86:PRO:HB3	1.69	1.24
2:B:230:PHE:CZ	2:B:252:LEU:CD2	2.17	1.24
2:B:337:THR:CB	2:B:373:LEU:CD1	2.13	1.24
2:B:216:LYS:HA	2:B:251:LEU:CD1	1.67	1.24
3:S:32:LEU:O	3:S:35:VAL:HG22	1.33	1.24
2:B:197:LYS:HB2	2:B:229:HIS:NE2	1.51	1.24
2:B:216:LYS:CA	2:B:251:LEU:CD1	2.15	1.23
2:B:347:VAL:CG2	2:B:359:LEU:HB3	1.66	1.23
1:A:329:ASN:HA	3:S:50:PHE:CZ	1.39	1.23
2:B:200:MET:SD	2:B:229:HIS:C	2.14	1.23
2:B:341:GLU:HG3	2:B:377:TYR:CE1	1.50	1.23
2:B:107:ARG:HH12	4:M:20:LEU:CD2	1.49	1.23
2:B:215:TYR:CD2	2:B:233:TYR:CE2	2.27	1.23
4:M:218:LEU:HA	4:M:472:TYR:CD2	1.71	1.23
4:M:222:PHE:CD1	4:M:240:ILE:HG23	1.72	1.23
1:A:422:GLU:OE1	3:S:62:GLU:HG2	1.37	1.23
1:A:450:TYR:OH	1:A:476:GLN:CG	1.87	1.23
2:B:13:ASP:OD1	4:M:14:LEU:C	1.75	1.23
2:B:216:LYS:HB2	2:B:251:LEU:CD1	1.66	1.23
2:B:219:TYR:CE2	2:B:226:LEU:HB2	1.71	1.23

Continued on next page...

Continued from previous page...

Atom-1	Atom-2	Interatomic distance (Å)	Clash overlap (Å)
1:A:258:LYS:NZ	3:S:93:GLU:CA	2.02	1.22
2:B:2:VAL:CG1	4:M:54:SER:CB	2.17	1.22
2:B:73:ASP:OD1	4:M:24:ALA:CB	1.85	1.22
2:B:375:LEU:HD13	2:B:404:ASN:CB	1.67	1.22
3:S:135:ILE:O	3:S:141:VAL:HA	1.33	1.22
1:A:506:LYS:HZ1	4:M:82:LYS:CD	1.50	1.22
2:B:293:VAL:O	2:B:299:LEU:HG	1.21	1.22
2:B:104:TYR:O	2:B:107:ARG:HB2	1.40	1.22
2:B:259:TYR:CD1	2:B:261:PRO:HG3	1.75	1.22
4:M:363:ASN:ND2	4:M:431:GLN:OE1	1.72	1.22
2:B:103:LEU:CD1	4:M:123:LEU:HD11	1.68	1.21
2:B:277:CYS:O	2:B:288:TYR:HB3	1.38	1.21
2:B:563:PHE:O	2:B:566:ALA:HB3	1.32	1.21
4:M:245:ASP:N	4:M:472:TYR:CE1	2.08	1.21
2:B:567:GLN:O	2:B:569:THR:OG1	1.53	1.21
2:B:584:SER:O	2:B:588:ILE:HG22	1.35	1.21
4:M:44:ASP:CB	4:M:50:TYR:CD2	2.23	1.21
2:B:107:ARG:NH2	4:M:20:LEU:CD2	1.96	1.21
2:B:143:SER:OG	2:B:179:LYS:HD2	1.36	1.21
2:B:178:ILE:CG1	2:B:214:ALA:HB1	1.69	1.21
2:B:196:LEU:O	2:B:215:TYR:OH	1.58	1.21
2:B:237:ILE:O	2:B:238:LYS:C	1.69	1.21
2:B:336:ASN:C	2:B:373:LEU:HD21	1.60	1.21
2:B:567:GLN:O	2:B:569:THR:CB	1.89	1.21
2:B:14:THR:CG2	2:B:36:THR:CG2	2.19	1.21
2:B:318:ILE:HD13	2:B:346:THR:OG1	1.08	1.21
1:A:406:GLY:C	3:S:64:ASN:ND2	1.93	1.20
2:B:1:MET:SD	4:M:39:PRO:CD	2.28	1.20
1:A:329:ASN:N	3:S:50:PHE:CE2	2.09	1.20
4:M:350:VAL:HG13	4:M:442:GLN:CB	1.71	1.20
2:B:5:ILE:HG21	4:M:42:LEU:CD1	1.70	1.20
2:B:106:LEU:HD22	2:B:144:ASP:OD2	1.42	1.20
2:B:479:VAL:CG2	2:B:486:HIS:CD2	2.25	1.20
1:A:328:PRO:C	3:S:50:PHE:CZ	2.09	1.20
2:B:72:SER:CB	4:M:17:GLN:OE1	1.89	1.20
2:B:106:LEU:CD2	4:M:130:GLU:CB	1.91	1.20
2:B:139:LEU:CD2	2:B:173:VAL:HA	1.72	1.20
2:B:279:LEU:N	2:B:288:TYR:HB2	1.54	1.20
1:A:606:PHE:CE1	1:A:633:PHE:HB2	1.75	1.20
2:B:12:LEU:HB3	4:M:13:LYS:CG	1.70	1.20
2:B:28:SER:C	2:B:58:GLU:HG2	1.62	1.20

Continued on next page...

Continued from previous page...

Atom-1	Atom-2	Interatomic distance (Å)	Clash overlap (Å)
2:B:313:SER:OG	4:M:269:ILE:O	1.60	1.19
4:M:221:THR:N	4:M:474:THR:OG1	1.73	1.19
1:A:329:ASN:HA	3:S:50:PHE:CE1	1.75	1.19
1:A:563:CYS:HB3	1:A:621:LEU:HD11	1.24	1.19
2:B:523:PHE:CE2	2:B:580:TYR:CD2	2.30	1.19
1:A:410:TYR:CD1	3:S:43:ASN:ND2	2.10	1.19
2:B:375:LEU:HD13	2:B:404:ASN:HB2	1.21	1.19
4:M:121:ILE:O	4:M:125:PHE:CD1	1.95	1.19
2:B:226:LEU:HD23	2:B:255:TYR:CE1	1.77	1.19
2:B:256:CYS:CB	2:B:328:LEU:CD2	2.19	1.19
2:B:268:LYS:O	2:B:273:SER:OG	1.55	1.19
2:B:375:LEU:HD13	2:B:404:ASN:ND2	1.52	1.19
2:B:403:ILE:CD1	2:B:439:CYS:HA	1.62	1.19
2:B:523:PHE:CZ	2:B:580:TYR:CD2	2.30	1.19
1:A:407:SER:C	3:S:64:ASN:ND2	1.95	1.18
2:B:2:VAL:CG1	4:M:54:SER:OG	1.89	1.18
2:B:106:LEU:CD2	4:M:130:GLU:CA	2.14	1.18
2:B:136:CYS:SG	2:B:169:VAL:HA	1.83	1.18
1:A:215:VAL:CG1	1:A:243:ILE:HG21	1.74	1.18
1:A:186:PHE:CE2	1:A:224:GLU:HB2	1.77	1.18
2:B:11:ALA:CA	2:B:40:GLN:NE2	2.06	1.18
2:B:261:PRO:CD	2:B:293:VAL:HG23	1.74	1.18
2:B:274:PRO:HG2	2:B:295:ASN:OD1	1.37	1.18
2:B:351:GLU:HB3	4:M:476:THR:CB	1.74	1.18
2:B:223:LEU:CD1	2:B:259:TYR:N	2.05	1.18
2:B:252:LEU:CD1	2:B:302:PHE:CD1	2.27	1.18
2:B:278:PRO:HA	2:B:288:TYR:CB	1.73	1.18
1:A:503:ASN:HB3	4:M:59:ASP:C	1.63	1.17
2:B:144:ASP:OD1	4:M:131:ALA:HA	1.40	1.17
2:B:219:TYR:CZ	2:B:226:LEU:CA	2.27	1.17
2:B:335:LYS:HA	2:B:370:ASP:OD2	1.43	1.17
2:B:155:LEU:CD2	2:B:192:LEU:HD11	1.75	1.17
2:B:433:VAL:HG12	2:B:474:VAL:CG2	1.73	1.17
2:B:158:VAL:CG1	2:B:177:ILE:CG1	2.22	1.17
2:B:383:VAL:O	2:B:385:PRO:HD3	1.45	1.17
2:B:374:PHE:CE2	2:B:402:LEU:HD13	1.75	1.17
4:M:275:CYS:SG	4:M:293:PRO:HB3	1.85	1.17
4:M:432:THR:OG1	4:M:480:GLN:HG3	1.00	1.17
2:B:28:SER:O	2:B:58:GLU:CG	1.90	1.16
2:B:155:LEU:CD2	2:B:192:LEU:CD1	2.23	1.16
2:B:564:LYS:HD2	2:B:621:GLY:O	1.02	1.16

Continued on next page...

Continued from previous page...

Atom-1	Atom-2	Interatomic distance (Å)	Clash overlap (Å)
2:B:252:LEU:HB2	2:B:302:PHE:CE1	1.79	1.16
2:B:322:CYS:SG	2:B:366:LEU:HD11	1.83	1.16
2:B:400:SER:HB3	2:B:435:SER:HB3	1.18	1.16
3:S:8:PHE:HB3	3:S:36:TYR:OH	1.42	1.16
2:B:16:LYS:HD3	4:M:115:VAL:HG21	1.21	1.16
2:B:296:ASP:OD1	2:B:297:PRO:CD	1.93	1.16
2:B:479:VAL:HG22	2:B:486:HIS:CD2	1.79	1.16
4:M:219:LEU:HD22	4:M:473:LYS:HA	1.16	1.16
2:B:38:TYR:OH	2:B:46:GLN:CB	1.92	1.16
2:B:73:ASP:N	4:M:19:LEU:HD22	1.51	1.16
2:B:106:LEU:CD1	2:B:144:ASP:O	1.92	1.16
2:B:216:LYS:CA	2:B:251:LEU:HD13	1.76	1.16
2:B:245:GLN:CD	2:B:309:LEU:HD11	1.66	1.16
2:B:568:VAL:HG12	2:B:571:SER:OG	1.44	1.16
1:A:258:LYS:NZ	3:S:93:GLU:HA	1.57	1.15
1:A:410:TYR:CE1	3:S:43:ASN:ND2	2.13	1.15
2:B:2:VAL:CG1	4:M:54:SER:HB3	1.74	1.15
2:B:178:ILE:HG12	2:B:214:ALA:HB1	1.19	1.15
2:B:476:ARG:HA	2:B:514:LEU:HD13	1.16	1.15
2:B:158:VAL:HG11	2:B:177:ILE:HG13	1.27	1.15
4:M:302:TYR:CD1	4:M:445:SER:HB3	1.80	1.15
1:A:179:LYS:HE3	3:S:140:MET:CB	1.75	1.15
1:A:411:GLU:OE2	3:S:46:PHE:CE1	1.99	1.15
2:B:64:LYS:NZ	4:M:120:ARG:NH1	1.94	1.15
2:B:219:TYR:CE1	2:B:226:LEU:HB2	1.81	1.15
1:A:186:PHE:CE2	1:A:224:GLU:CG	2.30	1.15
1:A:260:PHE:O	1:A:261:THR:C	1.69	1.15
2:B:219:TYR:CE2	2:B:226:LEU:CB	2.28	1.15
2:B:313:SER:CB	4:M:269:ILE:HB	1.64	1.15
1:A:254:ILE:HG21	3:S:94:SER:OG	1.44	1.15
2:B:16:LYS:CD	4:M:115:VAL:HG21	1.76	1.15
2:B:236:ILE:CG2	2:B:240:LEU:HD11	1.77	1.15
2:B:259:TYR:O	2:B:261:PRO:HD3	1.31	1.15
4:M:241:HIS:O	4:M:474:THR:CB	1.94	1.15
4:M:344:ILE:HG23	4:M:347:PHE:CB	1.71	1.15
2:B:212:VAL:HG21	2:B:248:LEU:HD21	1.29	1.14
4:M:41:LEU:HD12	4:M:52:ASP:H	1.02	1.14
1:A:504:ILE:HA	4:M:59:ASP:CG	1.68	1.14
2:B:13:ASP:O	2:B:17:VAL:HG22	1.46	1.14
2:B:236:ILE:HG22	2:B:240:LEU:CD1	1.77	1.14
2:B:252:LEU:HB3	2:B:302:PHE:CD2	1.81	1.14

Continued on next page...

Continued from previous page...

Atom-1	Atom-2	Interatomic distance (Å)	Clash overlap (Å)
2:B:343:LEU:HD23	2:B:363:ILE:HD13	1.15	1.14
4:M:244:VAL:HA	4:M:472:TYR:CE2	1.81	1.14
4:M:347:PHE:CE1	4:M:350:VAL:HG11	1.69	1.14
2:B:245:GLN:CD	2:B:309:LEU:CD1	2.16	1.14
2:B:375:LEU:O	2:B:377:TYR:N	1.78	1.14
2:B:556:LEU:HD22	2:B:588:ILE:HG12	1.26	1.14
2:B:25:VAL:HG22	2:B:30:LEU:O	1.45	1.14
1:A:186:PHE:CE2	1:A:224:GLU:HG2	1.83	1.14
1:A:638:LEU:HD23	2:B:561:ASP:CG	1.65	1.14
2:B:2:VAL:HG11	4:M:54:SER:OG	1.39	1.14
3:S:17:VAL:HG21	3:S:19:PHE:CZ	1.83	1.14
4:M:95:THR:OG1	4:M:137:SER:O	1.64	1.14
4:M:343:ASN:HA	4:M:408:VAL:HG13	1.15	1.14
1:A:275:LEU:O	1:A:276:PRO:C	1.73	1.13
1:A:406:GLY:O	3:S:64:ASN:ND2	1.76	1.13
1:A:408:ILE:HG21	3:S:41:GLN:HB3	1.14	1.13
2:B:14:THR:CG2	2:B:36:THR:HG22	1.77	1.13
2:B:20:ARG:NH1	4:M:118:TYR:CB	1.99	1.13
2:B:70:MET:HE1	2:B:107:ARG:HG3	1.29	1.13
2:B:216:LYS:HA	2:B:251:LEU:HD11	1.14	1.13
4:M:262:THR:CG2	4:M:265:ASN:H	1.61	1.13
2:B:219:TYR:CD2	2:B:226:LEU:HB2	1.84	1.13
2:B:341:GLU:CG	2:B:377:TYR:CE1	2.25	1.13
2:B:344:VAL:HG13	2:B:381:PHE:CE2	1.82	1.13
4:M:222:PHE:O	4:M:479:PHE:CZ	2.00	1.13
1:A:503:ASN:CB	4:M:59:ASP:C	2.16	1.13
2:B:127:LEU:HD13	2:B:157:THR:HG21	1.30	1.13
2:B:325:LEU:HD13	2:B:339:PHE:HB3	1.18	1.13
4:M:71:LYS:HB3	4:M:74:TYR:CZ	1.83	1.13
1:A:638:LEU:CD1	2:B:557:SER:C	2.17	1.13
1:A:114:PHE:CZ	1:A:153:ILE:HA	1.83	1.12
1:A:530:ASN:ND2	1:A:573:GLU:OE1	1.80	1.13
2:B:103:LEU:HB3	4:M:126:ASN:HD22	1.00	1.12
2:B:469:ASP:OD2	2:B:506:ASN:HB2	1.47	1.12
1:A:333:ILE:HD13	3:S:95:GLU:OE1	1.47	1.12
2:B:143:SER:HB2	2:B:179:LYS:CB	1.79	1.12
4:M:222:PHE:C	4:M:479:PHE:CZ	2.23	1.12
2:B:18:ILE:CD1	2:B:36:THR:HG21	1.76	1.12
2:B:72:SER:HB3	4:M:17:GLN:OE1	1.42	1.12
2:B:486:HIS:CE1	2:B:518:ILE:CD1	2.33	1.12
4:M:302:TYR:CE1	4:M:445:SER:HB3	1.84	1.12

Continued on next page...

Continued from previous page...

Atom-1	Atom-2	Interatomic distance (Å)	Clash overlap (Å)
4:M:350:VAL:HG13	4:M:442:GLN:HB3	1.16	1.12
1:A:503:ASN:CA	4:M:59:ASP:O	1.98	1.12
2:B:73:ASP:CG	4:M:24:ALA:HB1	1.68	1.12
2:B:106:LEU:HG	4:M:130:GLU:OE1	1.47	1.12
2:B:219:TYR:CZ	2:B:226:LEU:CB	2.31	1.12
4:M:219:LEU:HB2	4:M:472:TYR:O	1.43	1.12
1:A:606:PHE:HZ	1:A:633:PHE:CG	1.68	1.12
2:B:12:LEU:CD1	4:M:13:LYS:CA	2.27	1.12
2:B:12:LEU:CB	4:M:13:LYS:HG3	1.80	1.12
2:B:38:TYR:HE2	2:B:43:ASN:O	1.12	1.12
2:B:82:TYR:HB2	2:B:104:TYR:OH	1.50	1.12
2:B:230:PHE:O	2:B:231:ARG:O	1.66	1.12
2:B:261:PRO:HD3	2:B:293:VAL:HG23	1.26	1.12
2:B:422:ALA:HB3	2:B:424:PHE:CE2	1.85	1.12
2:B:181:TYR:CE1	2:B:185:LYS:HG3	1.85	1.11
4:M:219:LEU:HD13	4:M:472:TYR:O	1.40	1.11
2:B:9:ALA:HB1	4:M:14:LEU:CB	1.80	1.11
2:B:293:VAL:C	2:B:299:LEU:HG	1.71	1.11
2:B:223:LEU:CD1	2:B:258:GLN:C	2.19	1.11
1:A:233:PHE:O	1:A:234:ILE:C	1.71	1.11
2:B:12:LEU:HD12	4:M:13:LYS:HA	1.11	1.11
2:B:25:VAL:HG22	2:B:31:GLY:N	1.64	1.11
1:A:556:VAL:HG21	1:A:603:VAL:HG21	1.15	1.11
2:B:16:LYS:HB3	4:M:115:VAL:HG11	1.19	1.11
2:B:29:LYS:N	2:B:30:LEU:HA	1.56	1.11
2:B:107:ARG:NH1	4:M:20:LEU:CD2	2.09	1.11
4:M:244:VAL:CA	4:M:472:TYR:CD2	2.33	1.11
4:M:268:GLY:N	4:M:302:TYR:OH	1.83	1.11
1:A:215:VAL:CG1	1:A:243:ILE:CG2	2.28	1.10
1:A:503:ASN:CG	4:M:60:LEU:HD23	1.71	1.10
2:B:252:LEU:CB	2:B:302:PHE:CZ	2.35	1.10
4:M:2:TYR:OH	4:M:64:LYS:NZ	1.82	1.10
4:M:347:PHE:CE1	4:M:350:VAL:HG12	1.84	1.10
1:A:429:VAL:CB	1:A:469:LEU:HD11	1.81	1.10
1:A:503:ASN:OD1	4:M:60:LEU:HA	0.93	1.10
2:B:211:ALA:C	2:B:233:TYR:OH	1.90	1.10
2:B:303:LEU:HD22	2:B:339:PHE:CZ	1.85	1.10
2:B:348:THR:HG21	2:B:380:LYS:HB3	1.18	1.10
2:B:107:ARG:HH22	4:M:20:LEU:HD21	1.00	1.10
2:B:344:VAL:HG11	2:B:377:TYR:HB3	1.18	1.10
2:B:219:TYR:CD2	2:B:226:LEU:HD13	1.85	1.10

Continued on next page...

Continued from previous page...

Atom-1	Atom-2	Interatomic distance (Å)	Clash overlap (Å)
2:B:280:PRO:HG2	2:B:283:TYR:CD2	1.86	1.10
2:B:318:ILE:CD1	2:B:346:THR:OG1	2.00	1.10
2:B:337:THR:N	2:B:373:LEU:HD21	1.66	1.10
2:B:451:MET:HG3	2:B:489:ILE:HG12	1.32	1.10
4:M:341:SER:OG	4:M:343:ASN:ND2	1.84	1.10
1:A:125:THR:OG1	1:A:158:LEU:HD13	1.48	1.09
1:A:638:LEU:HD12	2:B:557:SER:C	1.71	1.09
2:B:211:ALA:HB3	2:B:233:TYR:OH	1.48	1.09
2:B:310:ILE:CG2	2:B:342:ALA:HB1	1.82	1.09
3:S:16:LEU:HB2	3:S:125:TRP:NE1	1.66	1.09
4:M:44:ASP:HB3	4:M:50:TYR:CD2	1.86	1.09
4:M:51:LEU:HB3	4:M:68:VAL:HG21	1.31	1.09
2:B:11:ALA:CA	2:B:40:GLN:HE22	1.60	1.09
2:B:70:MET:CE	2:B:107:ARG:HG3	1.80	1.09
2:B:106:LEU:HD13	2:B:144:ASP:HB2	1.18	1.09
2:B:158:VAL:HG11	2:B:177:ILE:HG12	1.10	1.09
2:B:274:PRO:CA	2:B:295:ASN:OD1	2.00	1.09
2:B:479:VAL:HG11	2:B:486:HIS:CE1	1.60	1.09
4:M:350:VAL:CG2	4:M:442:GLN:HG2	1.82	1.09
2:B:310:ILE:HD11	2:B:321:CYS:HB2	1.29	1.09
2:B:479:VAL:HG13	2:B:486:HIS:CG	1.87	1.09
2:B:224:GLU:CA	2:B:259:TYR:OH	0.79	1.09
3:S:35:VAL:HG12	3:S:77:TYR:OH	1.53	1.09
4:M:76:CYS:SG	4:M:97:ASP:OD1	2.10	1.09
4:M:262:THR:O	4:M:264:GLY:N	1.85	1.09
1:A:68:THR:HB	3:S:166:LYS:CB	1.83	1.09
1:A:114:PHE:CD1	1:A:153:ILE:HG23	1.88	1.09
2:B:20:ARG:CZ	4:M:118:TYR:HB3	1.82	1.09
2:B:276:SER:O	2:B:295:ASN:CB	1.99	1.09
1:A:179:LYS:HE3	3:S:140:MET:HB3	1.12	1.08
2:B:24:ALA:HB3	2:B:32:GLU:HG2	1.12	1.08
2:B:280:PRO:CG	2:B:283:TYR:CD2	2.36	1.08
4:M:20:LEU:HD22	4:M:129:VAL:HG21	1.29	1.08
4:M:106:LYS:CE	4:M:296:LYS:HE3	1.82	1.08
4:M:350:VAL:HG22	4:M:442:GLN:HG2	1.09	1.08
1:A:429:VAL:HB	1:A:469:LEU:HD11	1.33	1.08
1:A:506:LYS:CE	4:M:82:LYS:CB	2.31	1.08
2:B:340:ILE:HG12	2:B:373:LEU:HD23	1.36	1.08
4:M:44:ASP:HB2	4:M:50:TYR:CD2	1.88	1.08
4:M:106:LYS:HE2	4:M:296:LYS:CE	1.83	1.08
4:M:219:LEU:CG	4:M:472:TYR:O	2.02	1.08

Continued on next page...

Continued from previous page...

Atom-1	Atom-2	Interatomic distance (Å)	Clash overlap (Å)
2:B:18:ILE:HD13	2:B:36:THR:HG21	1.33	1.08
2:B:23:ALA:C	2:B:32:GLU:OE1	1.90	1.08
2:B:375:LEU:HD12	2:B:404:ASN:HD22	1.19	1.08
4:M:69:ILE:O	4:M:75:TRP:HA	1.53	1.08
1:A:595:GLU:OE1	2:B:513:TRP:HZ3	1.14	1.08
2:B:158:VAL:CG1	2:B:177:ILE:HG13	1.82	1.08
2:B:237:ILE:O	2:B:239:GLN:N	1.85	1.08
4:M:106:LYS:HE2	4:M:296:LYS:HE3	1.18	1.08
4:M:306:LEU:CD2	4:M:317:MET:CE	2.32	1.08
2:B:1:MET:HG2	4:M:39:PRO:HD3	1.30	1.08
2:B:104:TYR:O	2:B:107:ARG:CB	2.01	1.08
2:B:140:SER:HB2	2:B:172:GLU:OE1	1.51	1.08
2:B:236:ILE:O	2:B:240:LEU:HG	1.54	1.08
2:B:418:TYR:OH	2:B:432:ALA:CB	2.02	1.08
1:A:111:SER:HB2	1:A:152:THR:OG1	1.54	1.07
1:A:536:MET:CG	1:A:551:LEU:HD11	1.83	1.07
1:A:559:PHE:CE2	1:A:581:LEU:HD22	1.88	1.07
1:A:585:PHE:CE2	1:A:603:VAL:CG1	2.37	1.07
2:B:18:ILE:HD13	2:B:36:THR:CG2	1.81	1.07
2:B:103:LEU:HB3	4:M:126:ASN:ND2	1.68	1.07
2:B:433:VAL:HG21	2:B:471:TYR:CD2	1.88	1.07
2:B:556:LEU:CB	2:B:588:ILE:HD11	1.82	1.07
3:S:8:PHE:CD2	3:S:84:TYR:HB2	1.89	1.07
2:B:197:LYS:HB2	2:B:229:HIS:HE2	1.02	1.07
2:B:231:ARG:HG3	2:B:298:ASP:OD1	1.52	1.07
2:B:256:CYS:HB2	2:B:328:LEU:HD23	1.32	1.07
4:M:9:ASP:HB2	4:M:111:ILE:CG2	1.83	1.07
2:B:106:LEU:CB	4:M:130:GLU:HB3	1.85	1.07
2:B:247:TYR:CD1	4:M:136:VAL:HG12	1.89	1.07
2:B:386:LYS:NZ	4:M:478:ASN:OD1	1.86	1.07
2:B:433:VAL:HG11	2:B:471:TYR:N	1.69	1.07
2:B:556:LEU:HA	2:B:588:ILE:HD11	1.31	1.07
4:M:347:PHE:CD1	4:M:350:VAL:HB	1.89	1.07
2:B:223:LEU:CD1	2:B:258:GLN:CB	2.33	1.07
2:B:348:THR:CG2	2:B:380:LYS:HB3	1.84	1.07
2:B:38:TYR:OH	2:B:46:GLN:HB2	1.52	1.07
2:B:278:PRO:HG2	2:B:292:GLU:OE1	1.54	1.07
2:B:293:VAL:O	2:B:299:LEU:HD12	1.52	1.07
2:B:325:LEU:HD13	2:B:339:PHE:CB	1.84	1.07
2:B:461:HIS:O	2:B:462:ASN:HB3	1.43	1.07
4:M:223:HIS:ND1	4:M:476:THR:OG1	1.81	1.07

Continued on next page...

Continued from previous page...

Atom-1	Atom-2	Interatomic distance (Å)	Clash overlap (Å)
1:A:137:ASN:O	1:A:139:ASP:N	1.88	1.06
2:B:74:ASP:O	2:B:77:ILE:CG1	2.03	1.06
2:B:341:GLU:CG	2:B:377:TYR:HE1	1.61	1.06
2:B:375:LEU:HD13	2:B:404:ASN:CG	1.74	1.06
2:B:400:SER:HB3	2:B:435:SER:CA	1.85	1.06
4:M:375:LYS:HE2	4:M:418:GLU:OE1	1.55	1.06
1:A:225:LEU:HB3	1:A:233:PHE:CE2	1.89	1.06
4:M:261:ASN:HB2	4:M:450:GLU:CG	1.85	1.06
1:A:504:ILE:CA	4:M:59:ASP:OD2	2.01	1.06
2:B:178:ILE:HG23	2:B:217:GLU:HB2	1.16	1.06
2:B:564:LYS:CE	2:B:621:GLY:O	2.02	1.06
4:M:106:LYS:O	4:M:107:ASP:C	1.89	1.06
4:M:443:SER:HB3	4:M:447:ILE:CG1	1.85	1.06
1:A:186:PHE:HE2	1:A:224:GLU:HB2	0.94	1.06
2:B:1:MET:CG	4:M:39:PRO:CD	2.34	1.06
2:B:139:LEU:HG	2:B:176:ALA:HB2	1.37	1.06
2:B:309:LEU:HB3	2:B:317:VAL:HG12	1.35	1.06
2:B:374:PHE:CE1	2:B:381:PHE:CE2	2.44	1.06
2:B:433:VAL:HG12	2:B:474:VAL:HG21	1.36	1.06
2:B:556:LEU:HA	2:B:588:ILE:CD1	1.84	1.06
2:B:569:THR:HG22	2:B:569:THR:O	1.47	1.06
4:M:96:ILE:HG23	4:M:125:PHE:CE1	1.90	1.06
4:M:96:ILE:CG2	4:M:125:PHE:CE1	2.39	1.06
1:A:504:ILE:CD1	4:M:59:ASP:OD2	2.04	1.06
2:B:25:VAL:CG2	2:B:32:GLU:N	2.12	1.06
2:B:199:LEU:O	2:B:201:ALA:N	1.87	1.06
2:B:403:ILE:HG21	2:B:439:CYS:SG	1.95	1.06
4:M:243:ILE:O	4:M:472:TYR:CD2	2.09	1.06
4:M:273:HIS:HB2	4:M:298:ARG:O	1.55	1.06
1:A:200:PHE:CZ	1:A:236:LEU:HD21	1.90	1.05
2:B:73:ASP:H	4:M:19:LEU:HD23	1.20	1.05
2:B:219:TYR:HB3	2:B:223:LEU:HD23	1.33	1.05
4:M:243:ILE:O	4:M:472:TYR:CG	2.08	1.05
4:M:379:LEU:HD23	4:M:411:LEU:HG	1.33	1.05
2:B:70:MET:HE1	2:B:107:ARG:CG	1.85	1.05
2:B:196:LEU:HB3	2:B:215:TYR:CZ	1.91	1.05
2:B:476:ARG:HA	2:B:514:LEU:CD1	1.84	1.05
2:B:73:ASP:CB	4:M:19:LEU:HD22	1.87	1.05
2:B:100:LEU:HD21	4:M:123:LEU:HD22	1.38	1.05
4:M:48:ASP:O	4:M:70:ASN:HB3	1.56	1.05
4:M:323:MET:SD	4:M:342:LEU:HA	1.96	1.05

Continued on next page...

Continued from previous page...

Atom-1	Atom-2	Interatomic distance (Å)	Clash overlap (Å)
1:A:186:PHE:CZ	1:A:224:GLU:HG2	1.92	1.05
1:A:582:ILE:HG23	1:A:604:LEU:HG	1.33	1.05
2:B:70:MET:CE	2:B:107:ARG:CG	2.34	1.05
2:B:396:ILE:HD13	2:B:432:ALA:HB2	1.36	1.05
1:A:585:PHE:CE2	1:A:603:VAL:HG11	1.91	1.05
2:B:12:LEU:CD1	4:M:13:LYS:CG	2.23	1.05
2:B:64:LYS:NZ	4:M:120:ARG:HH12	1.50	1.05
2:B:73:ASP:H	4:M:19:LEU:CD2	1.60	1.05
2:B:106:LEU:HD11	2:B:144:ASP:HB3	1.07	1.05
2:B:162:VAL:HG21	2:B:195:ILE:HG22	1.31	1.05
2:B:310:ILE:HG12	2:B:318:ILE:HA	1.39	1.05
1:A:186:PHE:HE2	1:A:224:GLU:CB	1.62	1.04
2:B:12:LEU:HD13	4:M:13:LYS:CA	1.88	1.04
2:B:69:ILE:O	2:B:71:ALA:N	1.90	1.04
2:B:313:SER:HB3	4:M:269:ILE:HB	1.09	1.04
2:B:461:HIS:O	2:B:462:ASN:CB	1.98	1.04
3:S:32:LEU:O	3:S:35:VAL:CG2	2.04	1.04
1:A:506:LYS:HE2	4:M:82:LYS:HB3	1.04	1.04
2:B:38:TYR:OH	2:B:46:GLN:HG3	1.56	1.04
2:B:143:SER:CB	2:B:179:LYS:HB2	1.86	1.04
2:B:223:LEU:HD13	2:B:258:GLN:C	1.76	1.04
2:B:223:LEU:CD1	2:B:258:GLN:HB3	1.88	1.04
2:B:295:ASN:O	2:B:300:ASP:HB2	0.89	1.04
1:A:225:LEU:HD13	1:A:233:PHE:HZ	1.14	1.04
4:M:41:LEU:CD1	4:M:52:ASP:N	2.20	1.04
4:M:344:ILE:HG22	4:M:347:PHE:HB3	1.09	1.04
1:A:333:ILE:HD11	3:S:95:GLU:OE1	1.52	1.04
1:A:638:LEU:CD2	2:B:561:ASP:H	1.70	1.04
2:B:103:LEU:HD12	4:M:123:LEU:HD11	1.06	1.04
2:B:143:SER:CB	2:B:179:LYS:HD2	1.88	1.04
2:B:178:ILE:HD12	2:B:218:CYS:H	1.23	1.04
2:B:216:LYS:CA	2:B:251:LEU:HD11	1.85	1.04
2:B:299:LEU:O	2:B:302:PHE:HB3	1.58	1.04
2:B:379:LYS:HE2	2:B:410:GLU:HG3	1.06	1.04
2:B:556:LEU:HD22	2:B:588:ILE:CG1	1.88	1.04
4:M:220:GLU:HG2	4:M:439:TYR:HB2	1.38	1.04
1:A:179:LYS:HE2	3:S:137:GLN:O	0.86	1.03
2:B:106:LEU:HD22	2:B:144:ASP:CG	1.77	1.03
2:B:196:LEU:HB3	2:B:215:TYR:CE1	1.92	1.03
2:B:552:SER:O	2:B:556:LEU:HG	1.58	1.03
2:B:9:ALA:CA	4:M:14:LEU:HB2	1.89	1.03

Continued on next page...

Continued from previous page...

Atom-1	Atom-2	Interatomic distance (Å)	Clash overlap (Å)
2:B:512:VAL:C	2:B:551:LEU:HD13	1.78	1.03
1:A:503:ASN:CG	4:M:60:LEU:CA	2.26	1.03
2:B:17:VAL:CB	2:B:35:TYR:CD2	2.40	1.03
2:B:212:VAL:O	2:B:213:LEU:C	1.94	1.03
3:S:53:THR:HG21	3:S:68:VAL:HA	1.36	1.03
3:S:73:ILE:CG2	3:S:88:ILE:HG23	1.87	1.03
1:A:638:LEU:HD13	2:B:557:SER:OG	1.58	1.03
2:B:17:VAL:CB	2:B:35:TYR:HD2	1.71	1.03
2:B:178:ILE:CG1	2:B:214:ALA:C	2.27	1.03
2:B:212:VAL:HG23	2:B:233:TYR:CE1	1.94	1.03
2:B:344:VAL:CG1	2:B:377:TYR:HB3	1.90	1.02
2:B:347:VAL:HG11	2:B:381:PHE:CE1	1.94	1.02
4:M:105:ASP:O	4:M:106:LYS:CB	2.02	1.02
4:M:350:VAL:HA	4:M:442:GLN:HB2	1.38	1.02
4:M:383:HIS:CG	4:M:403:THR:HG1	1.76	1.02
2:B:224:GLU:CA	2:B:259:TYR:CZ	1.87	1.02
2:B:309:LEU:HB3	2:B:317:VAL:HG11	1.38	1.02
1:A:215:VAL:HG11	1:A:243:ILE:HG23	1.40	1.02
1:A:422:GLU:OE1	3:S:62:GLU:CG	2.07	1.02
1:A:462:GLN:OE1	4:M:58:ARG:CB	2.02	1.02
2:B:75:ASP:OD1	4:M:24:ALA:CB	2.07	1.02
2:B:103:LEU:CD1	4:M:123:LEU:CD1	2.37	1.02
2:B:174:ALA:HB1	2:B:211:ALA:HA	1.40	1.02
2:B:245:GLN:OE1	2:B:309:LEU:HD12	1.59	1.02
2:B:337:THR:OG1	2:B:373:LEU:HD13	1.58	1.02
4:M:243:ILE:O	4:M:472:TYR:HB3	1.54	1.02
4:M:245:ASP:O	4:M:472:TYR:HE1	1.11	1.02
1:A:289:SER:O	1:A:290:VAL:C	1.83	1.02
1:A:503:ASN:CG	4:M:60:LEU:HA	1.79	1.02
2:B:313:SER:HB3	4:M:269:ILE:CB	1.89	1.02
4:M:54:SER:H	4:M:66:PHE:HD2	1.05	1.02
4:M:224:VAL:H	4:M:479:PHE:CB	1.73	1.02
1:A:503:ASN:OD1	4:M:60:LEU:CD2	2.08	1.02
2:B:79:VAL:HG23	2:B:108:PHE:CE2	1.94	1.02
2:B:256:CYS:HB2	2:B:328:LEU:CD2	1.84	1.02
2:B:351:GLU:HB3	4:M:476:THR:HB	1.02	1.02
4:M:65:TYR:O	4:M:79:SER:HA	1.58	1.02
1:A:68:THR:HB	3:S:166:LYS:HB3	1.03	1.01
2:B:219:TYR:CD1	2:B:226:LEU:HB2	1.95	1.01
2:B:328:LEU:HB3	2:B:333:GLN:HE22	1.22	1.01
2:B:343:LEU:CD2	2:B:363:ILE:HD13	1.90	1.01

Continued on next page...

Continued from previous page...

Atom-1	Atom-2	Interatomic distance (Å)	Clash overlap (Å)
2:B:389:ILE:CG1	2:B:425:PRO:HG2	1.88	1.01
2:B:397:GLN:HE21	2:B:431:MET:HE3	1.21	1.01
4:M:15:ILE:HG23	4:M:115:VAL:CG2	1.90	1.01
4:M:306:LEU:HD22	4:M:317:MET:CE	1.89	1.01
2:B:12:LEU:CD1	4:M:13:LYS:CB	2.39	1.01
2:B:252:LEU:CB	2:B:302:PHE:CE2	2.44	1.01
2:B:567:GLN:C	2:B:569:THR:N	2.11	1.01
1:A:585:PHE:HE2	1:A:603:VAL:CG1	1.73	1.01
1:A:585:PHE:O	1:A:600:SER:OG	1.79	1.01
2:B:14:THR:HA	2:B:36:THR:HA	1.41	1.01
2:B:38:TYR:CD2	2:B:43:ASN:N	2.09	1.01
2:B:193:LEU:CD2	2:B:225:LEU:HB3	1.75	1.01
2:B:433:VAL:HG11	2:B:471:TYR:HA	1.06	1.01
3:S:127:THR:CG2	3:S:153:VAL:HG13	1.90	1.01
1:A:411:GLU:HG3	3:S:46:PHE:CZ	1.95	1.01
1:A:462:GLN:C	4:M:58:ARG:HA	1.81	1.01
1:A:573:GLU:O	1:A:574:ILE:C	1.77	1.01
2:B:12:LEU:HB3	4:M:13:LYS:HG2	1.40	1.01
2:B:73:ASP:CB	4:M:25:PRO:O	2.07	1.01
2:B:167:ALA:O	2:B:207:VAL:CG2	2.07	1.01
2:B:418:TYR:CD1	2:B:418:TYR:C	2.34	1.01
2:B:423:HIS:NE2	4:M:365:GLU:CB	2.23	1.01
4:M:261:ASN:HB2	4:M:450:GLU:HG3	1.42	1.01
4:M:443:SER:CB	4:M:447:ILE:HG13	1.91	1.01
1:A:556:VAL:CG2	1:A:603:VAL:HG21	1.90	1.01
2:B:219:TYR:CD2	2:B:226:LEU:CD2	2.43	1.01
2:B:259:TYR:HD1	2:B:261:PRO:HG3	1.15	1.01
2:B:375:LEU:CD1	2:B:404:ASN:HB2	1.91	1.01
2:B:447:GLU:OE1	2:B:485:LYS:HG3	1.61	1.01
4:M:218:LEU:O	4:M:441:GLY:CA	2.09	1.01
4:M:302:TYR:CE1	4:M:445:SER:CB	2.44	1.01
2:B:5:ILE:CG2	4:M:42:LEU:CD1	2.32	1.00
2:B:44:PRO:HB3	2:B:82:TYR:OH	1.59	1.00
2:B:278:PRO:CD	2:B:292:GLU:CB	2.28	1.00
2:B:563:PHE:HD1	2:B:584:SER:CB	1.73	1.00
4:M:68:VAL:HA	4:M:76:CYS:O	1.59	1.00
2:B:224:GLU:N	2:B:259:TYR:OH	1.74	1.00
2:B:398:ILE:CG2	2:B:402:LEU:HD11	1.91	1.00
1:A:409:VAL:CG1	3:S:42:ARG:HH12	1.74	1.00
1:A:506:LYS:CE	4:M:82:LYS:HB2	1.92	1.00
1:A:556:VAL:HG22	1:A:603:VAL:HG11	1.40	1.00

Continued on next page...

Continued from previous page...

Atom-1	Atom-2	Interatomic distance (Å)	Clash overlap (Å)
2:B:267:ASP:H	2:B:289:PRO:HG3	1.23	1.00
2:B:174:ALA:CB	2:B:211:ALA:HA	1.90	1.00
2:B:213:LEU:HD13	4:M:135:ASN:CG	1.81	1.00
2:B:245:GLN:NE2	2:B:309:LEU:HD11	1.74	1.00
2:B:279:LEU:HG	2:B:288:TYR:HD2	1.25	1.00
2:B:578:PRO:HD2	2:B:581:TYR:CD2	1.97	1.00
4:M:214:LEU:HD23	4:M:214:LEU:O	1.62	1.00
4:M:245:ASP:CA	4:M:472:TYR:CE1	2.45	1.00
4:M:347:PHE:CZ	4:M:350:VAL:HG12	1.95	1.00
2:B:155:LEU:HD21	2:B:192:LEU:HD12	1.44	1.00
2:B:212:VAL:HG21	2:B:248:LEU:CD2	1.92	1.00
2:B:193:LEU:CD2	2:B:225:LEU:HB2	1.88	1.00
2:B:437:SER:CA	2:B:474:VAL:HG13	1.91	1.00
3:S:8:PHE:CE2	3:S:84:TYR:CB	2.45	1.00
1:A:589:SER:CA	1:A:597:GLN:HG3	1.92	1.00
2:B:136:CYS:HB3	2:B:172:GLU:HG3	1.38	0.99
2:B:243:TRP:CZ3	4:M:98:ARG:HD2	1.96	0.99
2:B:252:LEU:O	2:B:302:PHE:CE2	2.14	0.99
2:B:336:ASN:C	2:B:373:LEU:CD2	2.29	0.99
2:B:344:VAL:HG21	2:B:377:TYR:CB	1.92	0.99
1:A:323:CYS:SG	1:A:334:SER:HB3	2.02	0.99
2:B:219:TYR:CG	2:B:226:LEU:HB2	1.96	0.99
2:B:403:ILE:HD13	2:B:439:CYS:HA	1.19	0.99
2:B:337:THR:CA	2:B:373:LEU:CD1	1.76	0.99
1:A:450:TYR:OH	1:A:476:GLN:HG3	1.57	0.99
2:B:17:VAL:HG11	2:B:35:TYR:CE2	1.98	0.99
2:B:261:PRO:HD3	2:B:293:VAL:CG2	1.91	0.99
4:M:20:LEU:HD22	4:M:129:VAL:CG2	1.91	0.99
2:B:73:ASP:CG	4:M:19:LEU:HD13	1.82	0.99
2:B:252:LEU:HB3	2:B:302:PHE:CE2	1.97	0.99
4:M:74:TYR:CB	4:M:114:ILE:HD11	1.93	0.99
4:M:220:GLU:HG3	4:M:439:TYR:CD2	1.96	0.99
2:B:211:ALA:HB1	2:B:233:TYR:OH	1.58	0.99
2:B:274:PRO:C	2:B:295:ASN:OD1	1.69	0.99
2:B:38:TYR:CE2	2:B:43:ASN:C	2.35	0.99
2:B:106:LEU:CD2	4:M:130:GLU:HA	1.92	0.99
2:B:256:CYS:SG	2:B:299:LEU:HD23	2.03	0.99
3:S:35:VAL:CG1	3:S:77:TYR:OH	2.10	0.99
4:M:245:ASP:CB	4:M:472:TYR:CD1	2.46	0.99
4:M:245:ASP:C	4:M:472:TYR:CE1	2.35	0.99
1:A:402:ILE:CG1	3:S:62:GLU:O	2.09	0.99

Continued on next page...

Continued from previous page...

Atom-1	Atom-2	Interatomic distance (Å)	Clash overlap (Å)
2:B:25:VAL:CG2	2:B:31:GLY:N	2.20	0.99
2:B:211:ALA:CA	2:B:233:TYR:OH	2.09	0.99
4:M:344:ILE:HG23	4:M:347:PHE:HB3	1.29	0.99
2:B:519:ALA:O	2:B:523:PHE:HB3	1.61	0.99
2:B:584:SER:O	2:B:588:ILE:CG2	2.10	0.99
4:M:95:THR:CG2	4:M:137:SER:O	2.09	0.99
4:M:244:VAL:O	4:M:299:LEU:N	1.95	0.99
1:A:288:THR:HG23	3:S:96:LEU:HD21	1.40	0.99
2:B:34:SER:O	2:B:37:TYR:HB3	1.63	0.99
2:B:193:LEU:HB3	2:B:225:LEU:HD12	1.44	0.99
2:B:396:ILE:HG22	2:B:435:SER:OG	1.61	0.99
2:B:14:THR:HG22	2:B:36:THR:HG22	0.99	0.98
2:B:70:MET:HE1	2:B:107:ARG:CB	1.93	0.98
2:B:216:LYS:CE	4:M:133:GLU:OE2	2.10	0.98
2:B:219:TYR:OH	2:B:226:LEU:HA	1.63	0.98
4:M:443:SER:HB3	4:M:447:ILE:HG13	1.41	0.98
2:B:469:ASP:OD1	2:B:507:ALA:N	1.97	0.98
1:A:225:LEU:CD1	1:A:233:PHE:CZ	2.44	0.98
2:B:475:ILE:HG23	2:B:489:ILE:HG21	1.45	0.98
4:M:96:ILE:CG2	4:M:125:PHE:CZ	2.45	0.98
1:A:136:GLY:O	1:A:139:ASP:HB3	1.64	0.98
2:B:143:SER:HB2	2:B:179:LYS:HB2	0.99	0.98
2:B:215:TYR:HD2	2:B:233:TYR:CE2	1.70	0.98
2:B:389:ILE:HG12	2:B:425:PRO:CG	1.92	0.98
2:B:403:ILE:HD13	2:B:439:CYS:HB3	1.44	0.98
1:A:213:SER:HB3	3:S:142:ILE:O	1.62	0.98
2:B:469:ASP:CG	2:B:506:ASN:HB2	1.84	0.98
4:M:375:LYS:CE	4:M:418:GLU:OE1	2.10	0.98
4:M:354:ASP:HB2	4:M:440:ILE:CD1	1.94	0.98
4:M:375:LYS:N	4:M:416:GLU:O	1.96	0.98
2:B:178:ILE:HG13	2:B:214:ALA:CB	1.94	0.97
2:B:178:ILE:CG1	2:B:214:ALA:CB	2.42	0.97
2:B:322:CYS:SG	2:B:366:LEU:CD1	2.51	0.97
2:B:213:LEU:HD13	4:M:135:ASN:OD1	0.80	0.97
2:B:230:PHE:HE2	2:B:252:LEU:HD23	1.17	0.97
4:M:271:SER:O	4:M:300:LEU:HA	1.64	0.97
4:M:354:ASP:HB2	4:M:440:ILE:HD12	1.44	0.97
1:A:508:LEU:HD12	4:M:59:ASP:OD2	1.62	0.97
2:B:5:ILE:HD13	4:M:42:LEU:HD12	1.41	0.97
2:B:24:ALA:CB	2:B:32:GLU:HG2	1.88	0.97
2:B:167:ALA:O	2:B:207:VAL:HG22	1.63	0.97

Continued on next page...

Continued from previous page...

Atom-1	Atom-2	Interatomic distance (Å)	Clash overlap (Å)
2:B:13:ASP:OD1	4:M:14:LEU:O	1.81	0.97
2:B:374:PHE:CZ	2:B:381:PHE:CE2	2.51	0.97
3:S:8:PHE:HB3	3:S:36:TYR:CZ	1.98	0.97
2:B:344:VAL:HG22	2:B:374:PHE:CE1	1.98	0.97
2:B:542:PRO:O	2:B:607:ILE:HD11	1.64	0.97
4:M:96:ILE:HD12	4:M:125:PHE:CD1	1.99	0.97
2:B:162:VAL:CG2	2:B:195:ILE:CG2	2.41	0.97
2:B:400:SER:CB	2:B:435:SER:HA	1.94	0.97
2:B:588:ILE:HG23	2:B:618:PHE:CZ	1.99	0.97
2:B:25:VAL:HG23	2:B:32:GLU:H	1.11	0.97
2:B:341:GLU:HA	2:B:377:TYR:CE1	1.65	0.97
2:B:73:ASP:N	4:M:19:LEU:CB	2.28	0.97
2:B:106:LEU:HD23	4:M:130:GLU:HB2	1.47	0.97
2:B:523:PHE:HE2	2:B:580:TYR:CD2	1.80	0.97
1:A:556:VAL:HG22	1:A:603:VAL:CG1	1.94	0.97
4:M:353:VAL:O	4:M:401:LYS:HA	1.65	0.97
2:B:9:ALA:O	4:M:14:LEU:CB	2.13	0.96
2:B:106:LEU:CD2	2:B:144:ASP:CB	2.43	0.96
2:B:261:PRO:HB2	2:B:290:SER:HB3	1.46	0.96
2:B:328:LEU:CB	2:B:333:GLN:HE22	1.78	0.96
2:B:422:ALA:HB3	2:B:424:PHE:CD2	2.00	0.96
1:A:225:LEU:CB	1:A:233:PHE:CZ	2.47	0.96
2:B:109:ALA:HB2	2:B:145:MET:SD	2.05	0.96
1:A:309:PHE:CZ	1:A:348:PHE:CZ	2.53	0.96
2:B:227:HIS:O	2:B:298:ASP:OD2	1.82	0.96
2:B:318:ILE:HD13	2:B:346:THR:CB	1.93	0.96
2:B:347:VAL:HG11	2:B:381:PHE:HE1	1.30	0.96
4:M:244:VAL:HG13	4:M:472:TYR:CE2	2.00	0.96
4:M:323:MET:HB3	4:M:340:LEU:HD11	1.46	0.96
2:B:143:SER:C	2:B:179:LYS:HD3	1.85	0.96
2:B:475:ILE:HG23	2:B:489:ILE:CG2	1.96	0.96
1:A:263:LEU:O	1:A:266:VAL:N	1.98	0.96
2:B:34:SER:HB3	2:B:65:ARG:NH1	1.80	0.96
2:B:396:ILE:CD1	2:B:432:ALA:HB2	1.94	0.96
4:M:243:ILE:H	4:M:474:THR:CG2	1.77	0.96
2:B:497:LEU:O	2:B:499:VAL:N	1.98	0.96
4:M:405:THR:C	4:M:407:THR:N	2.09	0.96
1:A:528:ASN:O	1:A:529:GLY:C	1.98	0.96
2:B:396:ILE:HD13	2:B:432:ALA:CB	1.95	0.96
2:B:523:PHE:CE2	2:B:580:TYR:CG	2.53	0.96
2:B:69:ILE:O	2:B:70:MET:C	2.00	0.96

Continued on next page...

Continued from previous page...

Atom-1	Atom-2	Interatomic distance (Å)	Clash overlap (Å)
2:B:139:LEU:HD21	2:B:173:VAL:HA	1.45	0.96
2:B:161:LEU:HB3	2:B:173:VAL:HG22	1.46	0.96
2:B:556:LEU:HB3	2:B:588:ILE:HD11	1.45	0.96
4:M:41:LEU:HD12	4:M:52:ASP:N	1.80	0.96
4:M:220:GLU:OE2	4:M:439:TYR:CD2	2.17	0.96
2:B:260:LEU:HA	2:B:293:VAL:HG21	1.48	0.96
2:B:278:PRO:HD3	2:B:289:PRO:O	1.64	0.96
4:M:379:LEU:HA	4:M:412:ARG:O	1.66	0.96
2:B:139:LEU:HD23	2:B:173:VAL:HA	1.44	0.96
2:B:144:ASP:OD2	4:M:131:ALA:HA	1.66	0.96
2:B:556:LEU:CD2	2:B:588:ILE:CG1	2.44	0.96
1:A:215:VAL:HG11	1:A:243:ILE:CG2	1.93	0.95
2:B:16:LYS:NZ	4:M:111:ILE:HD12	1.81	0.95
2:B:223:LEU:HD13	2:B:259:TYR:H	1.14	0.95
3:S:35:VAL:HB	3:S:77:TYR:CE2	2.00	0.95
4:M:265:ASN:HB3	4:M:309:GLN:HG3	1.46	0.95
1:A:68:THR:CB	3:S:166:LYS:HB3	1.96	0.95
2:B:155:LEU:CD2	2:B:192:LEU:HD12	1.95	0.95
2:B:278:PRO:C	2:B:288:TYR:HB2	1.85	0.95
2:B:297:PRO:O	2:B:301:LEU:CG	2.13	0.95
2:B:389:ILE:HG12	2:B:425:PRO:HG2	0.97	0.95
2:B:497:LEU:O	2:B:498:THR:C	1.92	0.95
4:M:350:VAL:HG22	4:M:442:GLN:CG	1.96	0.95
2:B:236:ILE:HG22	2:B:240:LEU:HD11	0.97	0.95
2:B:256:CYS:CB	2:B:328:LEU:HD22	1.88	0.95
2:B:523:PHE:CZ	2:B:580:TYR:CZ	2.55	0.95
1:A:323:CYS:SG	1:A:334:SER:CB	2.55	0.95
1:A:424:TYR:CD1	3:S:63:ASN:ND2	2.34	0.95
1:A:563:CYS:HB3	1:A:621:LEU:CD1	1.95	0.95
1:A:638:LEU:HD21	2:B:561:ASP:H	0.82	0.95
3:S:8:PHE:CE2	3:S:84:TYR:HB2	1.99	0.95
4:M:290:PHE:CE2	4:M:297:PHE:CZ	2.53	0.95
4:M:374:TYR:O	4:M:390:ILE:HD12	1.66	0.95
1:A:213:SER:HB2	3:S:142:ILE:CA	1.96	0.95
1:A:605:GLU:OE1	1:A:636:TYR:OH	1.83	0.95
2:B:208:ILE:HD13	2:B:236:ILE:HG21	1.47	0.95
2:B:560:ILE:HG23	2:B:564:LYS:HB2	1.47	0.95
4:M:41:LEU:HD13	4:M:52:ASP:H	1.27	0.95
2:B:371:GLN:HB3	2:B:401:THR:O	1.67	0.95
2:B:397:GLN:NE2	2:B:431:MET:CE	2.30	0.95
1:A:114:PHE:O	1:A:115:TYR:C	2.03	0.95

Continued on next page...

Continued from previous page...

Atom-1	Atom-2	Interatomic distance (Å)	Clash overlap (Å)
1:A:215:VAL:HG13	1:A:243:ILE:HG21	1.44	0.95
1:A:239:LEU:O	1:A:242:GLU:O	1.84	0.95
1:A:258:LYS:HZ1	3:S:93:GLU:HA	1.17	0.95
1:A:450:TYR:OH	1:A:476:GLN:HG2	1.65	0.95
3:S:130:SER:OG	3:S:156:LEU:HD12	1.66	0.95
4:M:432:THR:OG1	4:M:480:GLN:HG2	1.63	0.95
1:A:581:LEU:HD23	1:A:607:LEU:HD11	1.46	0.95
4:M:220:GLU:CD	4:M:439:TYR:HD2	1.71	0.95
1:A:137:ASN:C	1:A:139:ASP:N	2.12	0.95
1:A:558:VAL:CG1	1:A:562:TRP:CE2	2.49	0.95
2:B:162:VAL:HG21	2:B:195:ILE:HG23	1.48	0.95
2:B:184:GLY:O	2:B:188:TYR:HD2	1.47	0.95
2:B:556:LEU:HD23	2:B:588:ILE:HG13	1.46	0.95
4:M:219:LEU:HD22	4:M:473:LYS:CA	1.97	0.95
2:B:213:LEU:HD21	4:M:136:VAL:HB	1.49	0.94
2:B:216:LYS:CG	2:B:251:LEU:HD13	1.97	0.94
2:B:227:HIS:C	2:B:298:ASP:OD2	2.06	0.94
2:B:234:CYS:O	2:B:237:ILE:CG2	2.14	0.94
2:B:387:ASP:CB	2:B:388:PRO:HD2	1.97	0.94
2:B:566:ALA:HA	2:B:574:ASN:HB3	1.48	0.94
4:M:51:LEU:CB	4:M:68:VAL:HG21	1.97	0.94
4:M:347:PHE:CG	4:M:350:VAL:HB	2.01	0.94
4:M:436:GLU:HA	4:M:479:PHE:CE1	2.00	0.94
1:A:295:VAL:HG22	1:A:315:CYS:HB3	1.49	0.94
2:B:513:TRP:HA	2:B:551:LEU:CD2	1.97	0.94
2:B:519:ALA:O	2:B:523:PHE:CB	2.15	0.94
1:A:225:LEU:CB	1:A:233:PHE:CE2	2.50	0.94
1:A:319:LEU:O	1:A:320:HIS:C	2.02	0.94
2:B:9:ALA:HB1	4:M:14:LEU:HB2	0.94	0.94
2:B:144:ASP:OD2	4:M:131:ALA:CA	2.14	0.94
2:B:310:ILE:O	2:B:311:TYR:C	1.95	0.94
2:B:337:THR:N	2:B:373:LEU:CD2	2.30	0.94
2:B:348:THR:OG1	4:M:305:ASP:OD2	1.84	0.94
3:S:17:VAL:CG2	3:S:19:PHE:CZ	2.49	0.94
2:B:312:SER:C	4:M:269:ILE:HD13	1.73	0.94
4:M:20:LEU:CD2	4:M:129:VAL:HG21	1.97	0.94
4:M:44:ASP:O	4:M:47:SER:N	2.00	0.94
4:M:222:PHE:O	4:M:479:PHE:HE1	1.48	0.94
4:M:245:ASP:CA	4:M:472:TYR:CD1	2.50	0.94
2:B:86:VAL:HG12	2:B:101:ILE:HG23	1.48	0.94
2:B:278:PRO:HA	2:B:288:TYR:C	1.87	0.94

Continued on next page...

Continued from previous page...

Atom-1	Atom-2	Interatomic distance (Å)	Clash overlap (Å)
2:B:476:ARG:CA	2:B:514:LEU:HD13	1.95	0.94
2:B:563:PHE:C	2:B:566:ALA:HB3	1.87	0.94
2:B:25:VAL:HG21	2:B:32:GLU:H	1.31	0.94
2:B:335:LYS:CA	2:B:370:ASP:OD2	2.16	0.94
2:B:405:GLU:O	2:B:446:TRP:NE1	2.01	0.94
3:S:46:PHE:O	3:S:48:SER:N	2.00	0.94
1:A:405:THR:CA	2:B:7:ARG:NH2	2.13	0.94
1:A:179:LYS:NZ	3:S:142:ILE:N	2.16	0.94
1:A:225:LEU:HD13	1:A:233:PHE:CZ	2.01	0.94
1:A:257:LEU:HD22	1:A:278:ILE:CG2	1.97	0.94
1:A:462:GLN:O	4:M:58:ARG:CA	2.15	0.94
2:B:70:MET:CE	2:B:107:ARG:CB	2.46	0.94
2:B:325:LEU:CD1	2:B:339:PHE:HB3	1.96	0.94
4:M:224:VAL:N	4:M:479:PHE:CG	2.35	0.94
4:M:276:VAL:HG22	4:M:290:PHE:HD1	1.30	0.94
1:A:121:LEU:HD21	1:A:158:LEU:HB2	1.49	0.94
2:B:136:CYS:HB3	2:B:172:GLU:CG	1.97	0.94
2:B:200:MET:HG2	2:B:232:ARG:HB3	1.49	0.94
2:B:231:ARG:NH2	2:B:279:LEU:CD2	2.30	0.94
4:M:258:VAL:HG13	4:M:449:VAL:HG13	1.49	0.94
1:A:77:LEU:O	1:A:80:TYR:O	1.86	0.94
2:B:479:VAL:CG1	2:B:486:HIS:CD2	2.50	0.94
2:B:563:PHE:O	2:B:566:ALA:CB	2.15	0.94
1:A:581:LEU:HD11	1:A:585:PHE:CZ	2.03	0.93
2:B:16:LYS:HZ3	4:M:111:ILE:CG1	1.82	0.93
2:B:178:ILE:CG2	2:B:217:GLU:CB	2.44	0.93
2:B:181:TYR:CE1	2:B:222:HIS:HD2	1.75	0.93
2:B:226:LEU:HD23	2:B:255:TYR:HD1	1.17	0.93
2:B:256:CYS:SG	2:B:299:LEU:CD2	2.57	0.93
4:M:435:LEU:O	4:M:479:PHE:CG	2.20	0.93
2:B:106:LEU:HD22	2:B:144:ASP:CB	1.98	0.93
2:B:237:ILE:HG23	2:B:238:LYS:N	1.80	0.93
2:B:325:LEU:HB3	2:B:334:MET:SD	2.08	0.93
3:S:8:PHE:CB	3:S:36:TYR:HE1	1.81	0.93
4:M:95:THR:HG21	4:M:137:SER:O	1.68	0.93
4:M:262:THR:HG22	4:M:265:ASN:H	1.32	0.93
4:M:309:GLN:NE2	4:M:445:SER:O	2.00	0.93
2:B:337:THR:CG2	2:B:373:LEU:CD1	2.45	0.93
2:B:341:GLU:CA	2:B:377:TYR:CE1	2.36	0.93
2:B:400:SER:CB	2:B:435:SER:CA	2.47	0.93
3:S:8:PHE:CB	3:S:36:TYR:CE1	2.52	0.93

Continued on next page...

Continued from previous page...

Atom-1	Atom-2	Interatomic distance (Å)	Clash overlap (Å)
4:M:220:GLU:CG	4:M:439:TYR:HD2	1.78	0.93
4:M:245:ASP:O	4:M:472:TYR:CZ	2.22	0.93
2:B:313:SER:CB	4:M:269:ILE:O	2.16	0.93
2:B:568:VAL:O	2:B:571:SER:HB2	1.66	0.93
1:A:220:SER:O	1:A:223:CYS:HB3	1.69	0.93
2:B:179:LYS:NZ	4:M:131:ALA:HA	1.82	0.93
2:B:259:TYR:HD1	2:B:261:PRO:CG	1.82	0.93
2:B:337:THR:HG23	2:B:373:LEU:HD12	1.49	0.93
2:B:374:PHE:O	2:B:402:LEU:HD22	1.68	0.93
1:A:179:LYS:CE	3:S:140:MET:HB3	1.98	0.93
1:A:225:LEU:CD1	1:A:233:PHE:HZ	1.80	0.93
2:B:152:PRO:HA	2:B:188:TYR:CE1	2.03	0.93
2:B:196:LEU:C	2:B:215:TYR:OH	2.06	0.93
2:B:310:ILE:HG22	2:B:342:ALA:HB1	1.50	0.93
4:M:405:THR:O	4:M:407:THR:HG23	1.68	0.93
2:B:106:LEU:CD2	2:B:144:ASP:CG	2.37	0.93
2:B:231:ARG:HH22	2:B:279:LEU:HD21	1.10	0.93
4:M:241:HIS:O	4:M:474:THR:HB	1.68	0.93
1:A:391:LEU:O	1:A:392:MET:C	1.88	0.93
2:B:340:ILE:HG12	2:B:373:LEU:CD2	1.98	0.93
1:A:429:VAL:HB	1:A:469:LEU:CD1	1.98	0.93
2:B:161:LEU:HB3	2:B:173:VAL:CG2	1.98	0.93
2:B:181:TYR:CD2	2:B:218:CYS:O	2.22	0.93
2:B:219:TYR:CZ	2:B:226:LEU:HA	1.98	0.93
2:B:243:TRP:HH2	4:M:98:ARG:CD	1.59	0.93
2:B:325:LEU:HD13	2:B:339:PHE:CG	2.04	0.93
2:B:5:ILE:HD11	4:M:39:PRO:CG	1.98	0.93
2:B:310:ILE:HG23	2:B:318:ILE:HG23	1.48	0.93
2:B:415:LEU:HD12	2:B:436:LEU:HD21	1.49	0.93
4:M:104:PHE:CZ	4:M:117:ASN:HB3	2.04	0.93
2:B:1:MET:HG2	4:M:39:PRO:CD	1.98	0.92
2:B:73:ASP:HA	4:M:19:LEU:HD21	1.52	0.92
1:A:213:SER:HB2	3:S:142:ILE:HA	1.48	0.92
1:A:462:GLN:OE1	4:M:58:ARG:HB3	1.69	0.92
1:A:506:LYS:HZ3	4:M:82:LYS:CD	1.79	0.92
2:B:513:TRP:HA	2:B:551:LEU:HD22	1.49	0.92
2:B:518:ILE:O	2:B:518:ILE:HD12	1.69	0.92
4:M:74:TYR:HB3	4:M:114:ILE:HD11	1.51	0.92
4:M:218:LEU:CA	4:M:472:TYR:CE2	2.52	0.92
1:A:601:VAL:O	1:A:602:GLU:C	1.91	0.92
2:B:220:ALA:HA	2:B:258:GLN:HG3	1.50	0.92

Continued on next page...

Continued from previous page...

Atom-1	Atom-2	Interatomic distance (Å)	Clash overlap (Å)
1:A:570:LYS:HD3	1:A:618:THR:HG22	1.51	0.92
2:B:1:MET:CG	4:M:39:PRO:HD3	1.98	0.92
2:B:252:LEU:CB	2:B:302:PHE:CE1	2.51	0.92
2:B:336:ASN:O	2:B:373:LEU:HD21	1.69	0.92
2:B:433:VAL:HG12	2:B:474:VAL:HG23	1.48	0.92
2:B:472:VAL:CG1	2:B:510:GLY:HA3	1.99	0.92
1:A:381:GLU:O	1:A:382:ASP:C	1.91	0.92
2:B:16:LYS:HZ3	4:M:111:ILE:HG13	1.35	0.92
2:B:181:TYR:CE2	2:B:218:CYS:O	2.22	0.92
4:M:222:PHE:CD1	4:M:240:ILE:CG2	2.53	0.92
2:B:136:CYS:SG	2:B:169:VAL:CA	2.58	0.92
2:B:224:GLU:C	2:B:259:TYR:OH	2.07	0.92
2:B:374:PHE:CE2	2:B:398:ILE:CG2	2.52	0.92
4:M:9:ASP:CB	4:M:111:ILE:CG2	2.47	0.92
2:B:178:ILE:HG21	2:B:217:GLU:HB2	1.49	0.92
2:B:565:GLN:OE1	2:B:581:TYR:OH	1.87	0.92
1:A:103:LYS:HB3	1:A:107:TYR:CE2	2.03	0.92
1:A:254:ILE:CG2	3:S:94:SER:OG	2.18	0.92
2:B:219:TYR:CD2	2:B:226:LEU:CG	2.53	0.92
2:B:230:PHE:O	2:B:231:ARG:C	1.96	0.92
2:B:119:SER:O	2:B:123:LEU:HG	1.70	0.92
2:B:336:ASN:HB3	2:B:339:PHE:CE2	2.04	0.92
4:M:362:PHE:O	4:M:363:ASN:C	2.06	0.92
1:A:556:VAL:CG2	1:A:603:VAL:CG2	2.44	0.92
2:B:337:THR:HG23	2:B:373:LEU:CD1	2.00	0.92
4:M:344:ILE:HG23	4:M:347:PHE:HB2	1.50	0.92
2:B:107:ARG:HH12	4:M:20:LEU:HB3	1.35	0.91
4:M:351:SER:HB2	4:M:440:ILE:O	1.68	0.91
2:B:5:ILE:HG21	4:M:42:LEU:HD11	0.94	0.91
2:B:237:ILE:CG2	2:B:238:LYS:N	2.34	0.91
2:B:297:PRO:O	2:B:301:LEU:HD12	1.70	0.91
3:S:130:SER:OG	3:S:156:LEU:CD1	2.16	0.91
1:A:309:PHE:CE1	1:A:348:PHE:CE2	2.58	0.91
2:B:72:SER:OG	4:M:17:GLN:OE1	1.87	0.91
2:B:578:PRO:CD	2:B:581:TYR:HE2	1.57	0.91
2:B:124:GLN:HA	2:B:127:LEU:HD12	1.51	0.91
2:B:178:ILE:HD13	2:B:218:CYS:HB2	1.50	0.91
2:B:256:CYS:CB	2:B:328:LEU:HD23	1.94	0.91
4:M:9:ASP:HB2	4:M:111:ILE:HG21	1.50	0.91
2:B:297:PRO:O	2:B:301:LEU:HG	1.71	0.91
2:B:329:ALA:HB2	2:B:334:MET:HG2	1.51	0.91

Continued on next page...

Continued from previous page...

Atom-1	Atom-2	Interatomic distance (Å)	Clash overlap (Å)
4:M:344:ILE:HG21	4:M:347:PHE:HB3	1.50	0.91
1:A:114:PHE:CE1	1:A:153:ILE:HA	2.05	0.91
2:B:12:LEU:HD12	4:M:13:LYS:CA	1.96	0.91
2:B:80:GLN:N	2:B:108:PHE:HE2	1.68	0.91
2:B:219:TYR:CG	2:B:226:LEU:HD22	2.05	0.91
2:B:523:PHE:HZ	2:B:580:TYR:CZ	1.88	0.91
2:B:578:PRO:HD3	2:B:581:TYR:HE2	1.33	0.91
4:M:479:PHE:H	4:M:479:PHE:HD1	1.18	0.91
3:S:4:ALA:HA	3:S:18:LYS:O	1.69	0.91
4:M:44:ASP:HB2	4:M:50:TYR:HD2	1.29	0.91
4:M:113:LYS:O	4:M:116:ASN:HB2	1.70	0.91
1:A:575:LYS:NZ	1:A:615:GLU:OE1	2.04	0.91
2:B:16:LYS:HB3	4:M:115:VAL:CG1	2.01	0.91
2:B:151:ALA:CB	2:B:188:TYR:CE2	2.54	0.91
3:S:73:ILE:CG2	3:S:88:ILE:CG2	2.49	0.91
1:A:556:VAL:HG21	1:A:603:VAL:HG22	1.53	0.91
2:B:533:LEU:O	2:B:536:ASN:N	2.04	0.91
4:M:306:LEU:CD1	4:M:317:MET:CE	2.49	0.91
2:B:223:LEU:HD11	2:B:258:GLN:HB3	0.91	0.91
2:B:560:ILE:HA	2:B:563:PHE:HB2	1.53	0.91
2:B:578:PRO:HD2	2:B:581:TYR:CZ	2.05	0.91
3:S:135:ILE:O	3:S:141:VAL:CA	2.18	0.91
1:A:257:LEU:HD22	1:A:278:ILE:HG22	1.53	0.90
2:B:12:LEU:CB	4:M:13:LYS:CG	2.40	0.90
2:B:437:SER:HB2	2:B:474:VAL:CB	2.00	0.90
2:B:513:TRP:N	2:B:551:LEU:HD13	1.86	0.90
4:M:243:ILE:O	4:M:472:TYR:HB2	1.70	0.90
1:A:578:LEU:HD23	1:A:607:LEU:HD22	1.54	0.90
2:B:25:VAL:HG23	2:B:32:GLU:HG3	1.52	0.90
2:B:106:LEU:CD2	2:B:144:ASP:OD2	2.19	0.90
3:S:53:THR:OG1	3:S:68:VAL:CA	2.18	0.90
1:A:283:GLU:OE1	1:A:318:ARG:NH2	2.03	0.90
1:A:503:ASN:HD21	4:M:60:LEU:CD2	1.85	0.90
2:B:387:ASP:HB3	2:B:388:PRO:HD2	1.52	0.90
2:B:398:ILE:O	2:B:401:THR:N	2.03	0.90
2:B:556:LEU:CD2	2:B:588:ILE:HG13	1.99	0.90
4:M:95:THR:CB	4:M:137:SER:O	2.19	0.90
4:M:293:PRO:HD2	4:M:293:PRO:O	1.68	0.90
4:M:347:PHE:CD1	4:M:350:VAL:CB	2.53	0.90
2:B:139:LEU:CG	2:B:176:ALA:HB2	1.99	0.90
2:B:396:ILE:HD13	2:B:432:ALA:CA	2.00	0.90

Continued on next page...

Continued from previous page...

Atom-1	Atom-2	Interatomic distance (Å)	Clash overlap (Å)
4:M:100:LEU:CD2	4:M:121:ILE:HG12	2.00	0.90
2:B:109:ALA:O	2:B:110:GLU:C	1.99	0.90
1:A:121:LEU:HD12	1:A:153:ILE:CG2	2.01	0.90
2:B:38:TYR:CZ	2:B:43:ASN:O	2.25	0.90
2:B:436:LEU:HD12	2:B:454:LEU:CD2	2.01	0.90
1:A:316:LEU:CD1	1:A:348:PHE:CD2	2.54	0.90
1:A:424:TYR:CE1	3:S:63:ASN:ND2	2.40	0.90
2:B:155:LEU:HD23	2:B:192:LEU:HD11	0.91	0.90
2:B:297:PRO:O	2:B:301:LEU:CD1	2.19	0.90
2:B:562:ASN:O	2:B:581:TYR:HA	1.72	0.90
4:M:317:MET:HB2	4:M:322:LEU:H	1.33	0.90
4:M:347:PHE:O	4:M:348:LYS:C	2.09	0.90
1:A:179:LYS:HE2	3:S:137:GLN:C	1.92	0.90
1:A:213:SER:HB2	3:S:142:ILE:CB	2.02	0.90
2:B:29:LYS:N	2:B:30:LEU:CA	2.34	0.90
4:M:16:PHE:HE2	4:M:125:PHE:CE2	1.90	0.90
4:M:244:VAL:CA	4:M:472:TYR:CE2	2.53	0.90
2:B:80:GLN:CG	2:B:108:PHE:HZ	1.85	0.90
2:B:278:PRO:HD3	2:B:292:GLU:HB2	1.52	0.90
2:B:279:LEU:H	2:B:288:TYR:HB2	1.12	0.90
4:M:56:VAL:H	4:M:64:LYS:HG3	1.36	0.90
4:M:242:GLY:HA2	4:M:474:THR:HG21	1.52	0.90
1:A:276:PRO:O	1:A:278:ILE:O	1.88	0.90
2:B:11:ALA:HA	2:B:40:GLN:HE21	1.32	0.90
2:B:231:ARG:CG	2:B:298:ASP:OD1	2.19	0.90
2:B:278:PRO:HD2	2:B:292:GLU:HG3	1.52	0.90
2:B:348:THR:HG21	2:B:380:LYS:CB	2.01	0.90
2:B:567:GLN:O	2:B:569:THR:CA	2.18	0.90
1:A:349:ILE:HG21	1:A:378:ILE:HG22	1.54	0.89
2:B:23:ALA:C	2:B:32:GLU:CD	2.10	0.89
2:B:25:VAL:HG22	2:B:28:SER:OG	1.70	0.89
2:B:215:TYR:HD2	2:B:233:TYR:HE2	0.91	0.89
2:B:436:LEU:CD1	2:B:454:LEU:HD21	2.02	0.89
2:B:20:ARG:HH12	4:M:118:TYR:CA	1.84	0.89
2:B:56:SER:O	2:B:57:ARG:C	2.00	0.89
2:B:300:ASP:OD2	2:B:304:GLN:NE2	2.06	0.89
4:M:222:PHE:CE1	4:M:240:ILE:CG2	2.54	0.89
2:B:403:ILE:HD12	2:B:439:CYS:O	1.68	0.89
2:B:522:GLU:O	2:B:522:GLU:HG2	1.70	0.89
2:B:597:TYR:O	2:B:601:TYR:CD2	2.25	0.89
3:S:131:VAL:CG2	3:S:153:VAL:HG22	2.02	0.89

Continued on next page...

Continued from previous page...

Atom-1	Atom-2	Interatomic distance (Å)	Clash overlap (Å)
1:A:609:LEU:HG	1:A:628:VAL:CG1	2.02	0.89
2:B:347:VAL:HG21	2:B:381:PHE:CE1	2.08	0.89
3:S:16:LEU:HD13	3:S:125:TRP:HE1	1.36	0.89
4:M:241:HIS:O	4:M:474:THR:OG1	1.90	0.89
4:M:253:ASN:HA	4:M:292:PRO:HG2	1.54	0.89
1:A:585:PHE:C	1:A:600:SER:HG	1.74	0.89
2:B:243:TRP:HZ3	4:M:98:ARG:CD	1.80	0.89
4:M:240:ILE:HG21	4:M:444:ALA:HA	1.54	0.89
2:B:216:LYS:CG	2:B:251:LEU:CD1	2.51	0.89
2:B:340:ILE:HG21	2:B:374:PHE:HA	1.55	0.89
2:B:397:GLN:HE21	2:B:431:MET:CE	1.83	0.89
4:M:48:ASP:HA	4:M:70:ASN:HD22	1.37	0.89
2:B:16:LYS:HZ1	4:M:111:ILE:HD12	1.34	0.89
2:B:38:TYR:CE2	2:B:43:ASN:CA	2.55	0.89
2:B:193:LEU:HD21	2:B:225:LEU:HB2	1.53	0.89
2:B:345:ARG:O	2:B:349:MET:HG3	1.72	0.89
2:B:396:ILE:HG21	2:B:432:ALA:HA	0.90	0.89
2:B:400:SER:HA	2:B:439:CYS:SG	2.13	0.89
4:M:247:ARG:H	4:M:470:ALA:HB2	1.35	0.89
1:A:464:ILE:O	1:A:465:SER:C	1.92	0.89
2:B:2:VAL:HG11	4:M:54:SER:HB3	0.91	0.89
2:B:17:VAL:O	2:B:21:GLU:HG2	1.73	0.89
2:B:174:ALA:O	2:B:175:LEU:C	2.02	0.89
1:A:638:LEU:CD1	2:B:557:SER:OG	2.21	0.89
2:B:72:SER:C	4:M:19:LEU:HB2	1.92	0.89
2:B:232:ARG:HG3	2:B:236:ILE:HD11	1.55	0.89
2:B:252:LEU:HB3	2:B:302:PHE:CG	2.06	0.89
2:B:378:THR:O	2:B:381:PHE:N	2.05	0.89
3:S:16:LEU:HB2	3:S:125:TRP:HE1	1.32	0.89
4:M:9:ASP:CB	4:M:111:ILE:HG22	2.03	0.89
4:M:121:ILE:O	4:M:125:PHE:CE1	2.26	0.89
1:A:410:TYR:HD1	3:S:43:ASN:ND2	1.63	0.89
1:A:503:ASN:CG	4:M:60:LEU:N	2.25	0.89
2:B:11:ALA:N	2:B:40:GLN:HE22	1.71	0.89
2:B:278:PRO:CA	2:B:288:TYR:CB	2.51	0.89
2:B:294:VAL:HG23	2:B:333:GLN:HG2	1.54	0.89
2:B:397:GLN:NE2	2:B:431:MET:HE3	1.87	0.89
3:S:5:VAL:O	3:S:17:VAL:HA	1.73	0.89
4:M:9:ASP:HB2	4:M:111:ILE:HG22	1.53	0.89
4:M:44:ASP:HB3	4:M:50:TYR:CE2	2.07	0.89
4:M:240:ILE:CG2	4:M:444:ALA:HA	2.02	0.89

Continued on next page...

Continued from previous page...

Atom-1	Atom-2	Interatomic distance (Å)	Clash overlap (Å)
4:M:260:LEU:CD2	4:M:449:VAL:HG22	2.02	0.89
4:M:437:TYR:CD1	4:M:479:PHE:CZ	2.61	0.89
2:B:13:ASP:O	2:B:17:VAL:CG2	2.20	0.88
2:B:151:ALA:HA	2:B:180:LEU:HD11	1.55	0.88
2:B:276:SER:O	2:B:295:ASN:ND2	2.06	0.88
4:M:350:VAL:HG13	4:M:442:GLN:CG	2.03	0.88
2:B:310:ILE:HD11	2:B:321:CYS:CB	2.03	0.88
2:B:437:SER:HB2	2:B:474:VAL:HG13	0.90	0.88
2:B:486:HIS:ND1	2:B:518:ILE:HD13	1.88	0.88
4:M:350:VAL:CG1	4:M:442:GLN:HB3	2.02	0.88
1:A:503:ASN:HD21	4:M:60:LEU:HG	1.39	0.88
2:B:2:VAL:HG13	4:M:54:SER:OG	1.73	0.88
2:B:11:ALA:HA	2:B:40:GLN:HE22	1.07	0.88
2:B:162:VAL:HG22	2:B:199:LEU:HD11	1.54	0.88
2:B:375:LEU:HD13	2:B:404:ASN:HD22	1.14	0.88
2:B:568:VAL:CG1	2:B:571:SER:OG	2.21	0.88
4:M:356:LEU:CD2	4:M:358:ILE:HG13	2.04	0.88
1:A:84:MET:O	1:A:85:ALA:C	2.05	0.88
2:B:70:MET:CE	2:B:107:ARG:HB3	2.03	0.88
2:B:158:VAL:HG13	2:B:173:VAL:HG12	1.53	0.88
2:B:274:PRO:CG	2:B:295:ASN:CB	2.52	0.88
4:M:219:LEU:HB3	4:M:472:TYR:O	1.71	0.88
1:A:88:ASN:O	1:A:89:PHE:C	2.04	0.88
1:A:100:LEU:O	1:A:101:GLN:C	1.98	0.88
1:A:266:VAL:O	1:A:267:GLU:CB	2.22	0.88
1:A:585:PHE:CA	1:A:600:SER:OG	2.22	0.88
2:B:14:THR:HG21	2:B:40:GLN:HG2	1.53	0.88
2:B:151:ALA:HB1	2:B:188:TYR:CE2	2.08	0.88
2:B:351:GLU:CB	4:M:476:THR:HB	1.99	0.88
4:M:219:LEU:CD2	4:M:473:LYS:HA	2.01	0.88
2:B:37:TYR:HD2	2:B:38:TYR:CD1	1.92	0.88
2:B:144:ASP:HA	2:B:179:LYS:HD3	1.54	0.88
2:B:367:SER:HB2	2:B:401:THR:HB	1.53	0.88
2:B:433:VAL:CG1	2:B:474:VAL:HG21	2.04	0.88
2:B:457:HIS:HA	2:B:461:HIS:HD2	1.37	0.88
4:M:15:ILE:HG23	4:M:115:VAL:HG23	1.54	0.88
1:A:88:ASN:HB3	1:A:120:ILE:HG23	1.56	0.88
1:A:216:SER:HB3	3:S:140:MET:SD	2.13	0.88
1:A:495:ILE:HG23	1:A:515:CYS:SG	2.13	0.88
2:B:337:THR:CG2	2:B:373:LEU:HD11	2.02	0.88
3:S:8:PHE:HB3	3:S:36:TYR:CE1	2.08	0.88

Continued on next page...

Continued from previous page...

Atom-1	Atom-2	Interatomic distance (Å)	Clash overlap (Å)
4:M:245:ASP:HB3	4:M:472:TYR:CD1	2.09	0.88
2:B:219:TYR:CE2	2:B:226:LEU:CG	2.56	0.88
2:B:280:PRO:HG3	2:B:283:TYR:CD2	2.09	0.88
2:B:367:SER:HB2	2:B:401:THR:CB	2.03	0.88
4:M:272:LEU:HD21	4:M:278:ILE:HB	1.56	0.88
4:M:288:ILE:CD1	4:M:300:LEU:HD22	2.03	0.88
1:A:621:LEU:O	1:A:622:PRO:C	1.96	0.88
2:B:38:TYR:CZ	2:B:43:ASN:N	2.31	0.88
1:A:381:GLU:HA	1:A:384:LEU:HD23	1.53	0.87
2:B:143:SER:HB3	2:B:176:ALA:HA	1.54	0.87
2:B:178:ILE:HG23	2:B:217:GLU:CB	2.03	0.87
2:B:568:VAL:HG12	2:B:571:SER:CB	2.03	0.87
4:M:221:THR:H	4:M:474:THR:HG1	0.93	0.87
1:A:411:GLU:HG3	3:S:46:PHE:HZ	1.34	0.87
1:A:536:MET:O	1:A:537:THR:C	1.89	0.87
2:B:230:PHE:CD1	2:B:298:ASP:HB3	2.08	0.87
2:B:341:GLU:HG3	2:B:377:TYR:HE1	0.77	0.87
2:B:374:PHE:HE1	2:B:381:PHE:CE2	1.90	0.87
4:M:306:LEU:HD22	4:M:317:MET:HE3	1.52	0.87
2:B:136:CYS:CB	2:B:172:GLU:HG3	2.04	0.87
4:M:347:PHE:HE1	4:M:350:VAL:HG11	1.32	0.87
4:M:383:HIS:CG	4:M:403:THR:OG1	2.26	0.87
1:A:503:ASN:CB	4:M:59:ASP:O	2.20	0.87
1:A:595:GLU:OE2	2:B:513:TRP:CH2	2.27	0.87
2:B:279:LEU:HG	2:B:288:TYR:CD2	2.09	0.87
4:M:219:LEU:HB3	4:M:472:TYR:C	1.94	0.87
4:M:240:ILE:HG22	4:M:444:ALA:CB	2.04	0.87
1:A:68:THR:H	3:S:166:LYS:HD3	1.40	0.87
2:B:106:LEU:CG	2:B:144:ASP:HB3	2.04	0.87
2:B:214:ALA:O	2:B:217:GLU:N	2.07	0.87
2:B:563:PHE:HD1	2:B:584:SER:HB3	1.39	0.87
3:S:131:VAL:HG22	3:S:153:VAL:HG22	1.57	0.87
1:A:462:GLN:C	4:M:58:ARG:CA	2.43	0.87
2:B:197:LYS:CB	2:B:229:HIS:NE2	2.36	0.87
2:B:237:ILE:CG2	2:B:238:LYS:H	1.86	0.87
2:B:367:SER:CB	2:B:401:THR:OG1	2.22	0.87
4:M:220:GLU:CG	4:M:439:TYR:HB2	2.04	0.87
1:A:488:ARG:O	1:A:491:THR:OG1	1.93	0.87
2:B:181:TYR:HE1	2:B:222:HIS:CD2	1.90	0.87
2:B:253:ILE:HG12	2:B:324:ALA:HA	1.55	0.87
4:M:92:PHE:HE2	4:M:128:CYS:O	1.58	0.87

Continued on next page...

Continued from previous page...

Atom-1	Atom-2	Interatomic distance (Å)	Clash overlap (Å)
4:M:219:LEU:CB	4:M:472:TYR:C	2.41	0.87
1:A:103:LYS:O	1:A:107:TYR:CD2	2.28	0.87
1:A:251:TRP:HE3	3:S:97:ALA:HA	1.16	0.87
2:B:553:ALA:HB2	2:B:614:ILE:HD13	1.55	0.87
2:B:569:THR:O	2:B:569:THR:CG2	2.22	0.87
4:M:74:TYR:HB3	4:M:114:ILE:CD1	2.05	0.87
4:M:223:HIS:CA	4:M:479:PHE:CE1	2.57	0.87
4:M:306:LEU:O	4:M:307:SER:C	2.03	0.87
1:A:433:ILE:HG23	1:A:476:GLN:HB2	1.57	0.87
4:M:4:SER:O	4:M:78:ALA:HA	1.72	0.87
2:B:34:SER:OG	2:B:65:ARG:CZ	2.23	0.86
2:B:69:ILE:HG23	2:B:74:ASP:HB3	1.57	0.86
2:B:386:LYS:HE3	4:M:480:GLN:HB2	1.55	0.86
2:B:224:GLU:CG	2:B:259:TYR:OH	2.23	0.86
2:B:366:LEU:O	2:B:367:SER:C	2.02	0.86
2:B:479:VAL:HG21	2:B:486:HIS:CD2	2.09	0.86
2:B:523:PHE:HE2	2:B:580:TYR:CG	1.91	0.86
2:B:556:LEU:CA	2:B:588:ILE:CD1	2.48	0.86
3:S:8:PHE:CE2	3:S:84:TYR:HB3	2.08	0.86
1:A:506:LYS:NZ	4:M:82:LYS:HD3	1.88	0.86
2:B:158:VAL:CG1	2:B:177:ILE:HG12	1.99	0.86
2:B:278:PRO:HA	2:B:288:TYR:HB3	1.55	0.86
1:A:66:SER:N	3:S:166:LYS:HE2	1.89	0.86
2:B:72:SER:O	2:B:73:ASP:HB2	1.75	0.86
2:B:107:ARG:HH12	4:M:20:LEU:CB	1.88	0.86
2:B:344:VAL:HG11	2:B:377:TYR:CB	2.05	0.86
2:B:436:LEU:HD12	2:B:454:LEU:HD21	1.57	0.86
4:M:222:PHE:CE1	4:M:240:ILE:HG23	2.10	0.86
1:A:288:THR:HG23	3:S:96:LEU:CD2	2.05	0.86
2:B:261:PRO:HD2	2:B:293:VAL:HG23	1.55	0.86
4:M:41:LEU:HD13	4:M:52:ASP:N	1.85	0.86
4:M:74:TYR:CB	4:M:114:ILE:CD1	2.52	0.86
4:M:105:ASP:O	4:M:106:LYS:HB3	1.74	0.86
4:M:306:LEU:HD13	4:M:317:MET:HE3	1.54	0.86
1:A:114:PHE:CD2	1:A:153:ILE:HG12	2.10	0.86
1:A:408:ILE:HG22	3:S:64:ASN:HB3	1.56	0.86
1:A:629:LEU:O	1:A:630:PRO:C	2.01	0.86
2:B:28:SER:C	2:B:58:GLU:CG	2.34	0.86
2:B:260:LEU:HD22	2:B:291:TYR:OH	1.75	0.86
2:B:274:PRO:CG	2:B:295:ASN:HA	2.05	0.86
1:A:462:GLN:C	4:M:58:ARG:CB	2.41	0.86

Continued on next page...

Continued from previous page...

Atom-1	Atom-2	Interatomic distance (Å)	Clash overlap (Å)
4:M:215:TYR:CG	4:M:468:LYS:HA	2.11	0.86
4:M:223:HIS:CA	4:M:479:PHE:CD1	2.55	0.86
1:A:182:ILE:HG22	1:A:221:VAL:HG21	1.58	0.86
2:B:189:HIS:NE2	2:B:222:HIS:HB3	1.91	0.86
2:B:239:GLN:O	4:M:279:ASN:HA	1.75	0.86
2:B:310:ILE:CD1	2:B:321:CYS:HB2	2.05	0.86
2:B:418:TYR:CD1	2:B:424:PHE:CE2	2.64	0.86
2:B:424:PHE:CD1	2:B:428:VAL:HG11	2.10	0.86
1:A:373:GLU:HG3	1:A:427:LYS:HE2	1.58	0.86
2:B:143:SER:C	2:B:179:LYS:CD	2.44	0.86
2:B:360:LEU:HB3	2:B:394:TRP:HB3	1.58	0.86
2:B:472:VAL:HG11	2:B:510:GLY:HA3	1.55	0.86
2:B:537:PHE:CE2	2:B:545:ARG:HB3	2.11	0.86
2:B:599:ALA:O	2:B:601:TYR:N	2.09	0.86
4:M:96:ILE:HG21	4:M:125:PHE:CE1	2.09	0.86
4:M:215:TYR:CD1	4:M:467:TYR:O	2.28	0.86
2:B:162:VAL:CG2	2:B:199:LEU:HD11	2.05	0.86
2:B:172:GLU:O	2:B:173:VAL:C	2.06	0.86
4:M:290:PHE:CZ	4:M:293:PRO:HD3	2.10	0.86
2:B:559:ASP:O	2:B:563:PHE:N	2.09	0.85
4:M:15:ILE:HG23	4:M:115:VAL:HG22	1.57	0.85
4:M:245:ASP:N	4:M:472:TYR:CG	2.29	0.85
1:A:595:GLU:OE2	2:B:513:TRP:CD2	2.29	0.85
2:B:219:TYR:CD2	2:B:226:LEU:CB	2.53	0.85
2:B:344:VAL:HG13	2:B:381:PHE:HE2	1.32	0.85
2:B:596:LEU:HD12	2:B:611:ALA:HB1	1.58	0.85
2:B:212:VAL:CG2	2:B:248:LEU:HD21	2.05	0.85
4:M:443:SER:HG	4:M:447:ILE:C	1.79	0.85
1:A:225:LEU:HB3	1:A:233:PHE:CZ	2.11	0.85
1:A:411:GLU:CG	3:S:46:PHE:CZ	2.58	0.85
1:A:506:LYS:HZ3	4:M:82:LYS:HD3	1.38	0.85
2:B:227:HIS:CA	2:B:298:ASP:OD2	2.24	0.85
2:B:256:CYS:SG	2:B:328:LEU:HD21	2.15	0.85
2:B:337:THR:CA	2:B:373:LEU:CD2	2.55	0.85
2:B:517:GLU:OE2	2:B:554:LYS:NZ	2.09	0.85
4:M:290:PHE:HB2	4:M:299:LEU:CD1	2.05	0.85
2:B:343:LEU:CD2	2:B:366:LEU:HD12	2.06	0.85
4:M:347:PHE:CE1	4:M:350:VAL:CB	2.60	0.85
2:B:5:ILE:HD11	4:M:39:PRO:CD	2.07	0.85
2:B:5:ILE:HD11	4:M:39:PRO:N	1.90	0.85
2:B:14:THR:CG2	2:B:36:THR:HG23	1.94	0.85

Continued on next page...

Continued from previous page...

Atom-1	Atom-2	Interatomic distance (Å)	Clash overlap (Å)
2:B:70:MET:SD	2:B:107:ARG:HB3	2.17	0.85
2:B:75:ASP:CG	4:M:24:ALA:HB3	1.95	0.85
2:B:293:VAL:CA	2:B:299:LEU:HG	2.06	0.85
2:B:433:VAL:CG2	2:B:471:TYR:CD2	2.58	0.85
2:B:479:VAL:HG13	2:B:486:HIS:CD2	2.10	0.85
3:S:53:THR:CB	3:S:68:VAL:C	2.44	0.85
3:S:135:ILE:HG23	3:S:141:VAL:HG13	1.58	0.85
4:M:215:TYR:CD1	4:M:468:LYS:HA	2.11	0.85
2:B:80:GLN:HG3	2:B:108:PHE:HZ	1.41	0.85
2:B:337:THR:CA	2:B:373:LEU:HD21	2.07	0.85
4:M:212:ASN:HA	4:M:249:TYR:O	1.75	0.85
2:B:50:LEU:HD23	2:B:62:ALA:HA	1.56	0.85
2:B:181:TYR:HE1	2:B:185:LYS:HG3	1.37	0.85
2:B:216:LYS:HG3	2:B:251:LEU:HD12	1.58	0.85
2:B:322:CYS:SG	2:B:362:ALA:HB1	2.16	0.85
1:A:102:GLN:NE2	3:S:166:LYS:NZ	2.25	0.85
1:A:309:PHE:CE1	1:A:348:PHE:CZ	2.64	0.85
1:A:585:PHE:CE2	1:A:603:VAL:HG12	2.11	0.85
2:B:9:ALA:C	4:M:14:LEU:CB	2.44	0.85
2:B:73:ASP:CA	4:M:19:LEU:HD23	2.00	0.85
2:B:261:PRO:CD	2:B:293:VAL:CG2	2.53	0.85
2:B:278:PRO:HA	2:B:288:TYR:CA	2.06	0.85
2:B:351:GLU:CB	4:M:476:THR:CG2	2.55	0.85
4:M:215:TYR:HD1	4:M:467:TYR:O	1.59	0.85
4:M:222:PHE:CD2	4:M:439:TYR:HE2	1.95	0.85
4:M:262:THR:C	4:M:264:GLY:H	1.80	0.85
1:A:411:GLU:CG	3:S:46:PHE:HZ	1.89	0.85
1:A:601:VAL:O	1:A:602:GLU:O	1.94	0.85
2:B:34:SER:CB	2:B:65:ARG:NH1	2.40	0.85
2:B:215:TYR:HD1	2:B:219:TYR:CE2	1.93	0.85
1:A:183:THR:O	1:A:186:PHE:HB3	1.77	0.84
1:A:244:LEU:HD11	1:A:281:LEU:CD1	2.07	0.84
1:A:410:TYR:HE1	3:S:43:ASN:ND2	1.71	0.84
1:A:503:ASN:HD21	4:M:60:LEU:CG	1.90	0.84
2:B:106:LEU:HD13	2:B:144:ASP:CA	2.06	0.84
2:B:219:TYR:CD2	2:B:226:LEU:CD1	2.53	0.84
2:B:429:VAL:HG12	2:B:467:VAL:HG13	1.57	0.84
1:A:536:MET:HG2	1:A:551:LEU:HD11	1.58	0.84
1:A:581:LEU:HD23	1:A:607:LEU:HD21	1.58	0.84
4:M:240:ILE:HG22	4:M:444:ALA:HB1	1.57	0.84
4:M:306:LEU:HD11	4:M:317:MET:HE1	1.58	0.84

Continued on next page...

Continued from previous page...

Atom-1	Atom-2	Interatomic distance (Å)	Clash overlap (Å)
4:M:376:ILE:CG2	4:M:379:LEU:CD1	2.54	0.84
2:B:274:PRO:HB2	2:B:295:ASN:OD1	1.75	0.84
4:M:215:TYR:HB2	4:M:467:TYR:C	1.95	0.84
1:A:114:PHE:O	1:A:115:TYR:O	1.95	0.84
1:A:261:THR:OG1	1:A:297:CYS:SG	2.33	0.84
1:A:402:ILE:CB	3:S:62:GLU:O	2.23	0.84
1:A:511:VAL:O	1:A:515:CYS:SG	2.36	0.84
2:B:104:TYR:O	2:B:107:ARG:N	2.09	0.84
2:B:107:ARG:NH1	4:M:20:LEU:HB3	1.92	0.84
2:B:596:LEU:HD22	2:B:615:SER:OG	1.76	0.84
4:M:306:LEU:CD1	4:M:317:MET:HE1	2.06	0.84
4:M:362:PHE:O	4:M:363:ASN:O	1.95	0.84
4:M:443:SER:HB3	4:M:447:ILE:N	1.93	0.84
1:A:204:VAL:O	1:A:205:SER:C	2.09	0.84
1:A:395:PHE:CE1	1:A:428:MET:HG3	2.12	0.84
1:A:409:VAL:HG12	3:S:42:ARG:NH1	1.92	0.84
1:A:409:VAL:HG12	3:S:42:ARG:HH12	1.42	0.84
1:A:581:LEU:HD23	1:A:607:LEU:CD1	2.07	0.84
2:B:103:LEU:HD11	4:M:127:CYS:SG	2.17	0.84
3:S:109:LEU:HD12	3:S:113:PHE:CD1	2.12	0.84
4:M:92:PHE:CZ	4:M:128:CYS:HB2	2.12	0.84
4:M:258:VAL:HG13	4:M:449:VAL:CG1	2.07	0.84
1:A:328:PRO:O	1:A:329:ASN:C	2.11	0.84
1:A:589:SER:HA	1:A:597:GLN:HG3	1.59	0.84
2:B:70:MET:HE3	2:B:107:ARG:CG	2.05	0.84
2:B:433:VAL:CG2	2:B:471:TYR:CE2	2.61	0.84
3:S:53:THR:OG1	3:S:68:VAL:N	2.11	0.84
4:M:6:TYR:HA	4:M:16:PHE:O	1.77	0.84
1:A:190:LEU:HD11	1:A:228:LYS:HE3	1.60	0.84
1:A:495:ILE:HG21	1:A:515:CYS:HB3	1.59	0.84
1:A:503:ASN:ND2	4:M:60:LEU:CD2	2.40	0.84
1:A:581:LEU:CD2	1:A:607:LEU:HD11	2.07	0.84
2:B:127:LEU:HB3	2:B:157:THR:HG23	1.59	0.84
2:B:418:TYR:C	2:B:418:TYR:HD1	1.77	0.84
2:B:430:ILE:HD11	2:B:466:SER:HB2	1.58	0.84
4:M:432:THR:HA	4:M:481:VAL:O	1.77	0.84
2:B:106:LEU:HD12	2:B:144:ASP:O	1.74	0.84
2:B:226:LEU:HD21	2:B:255:TYR:CD1	2.11	0.84
2:B:398:ILE:HG22	2:B:402:LEU:CD1	2.08	0.84
2:B:549:LEU:HD13	2:B:595:VAL:HG12	1.59	0.84
4:M:271:SER:HB3	4:M:301:GLU:HG3	1.60	0.84

Continued on next page...

Continued from previous page...

Atom-1	Atom-2	Interatomic distance (Å)	Clash overlap (Å)
4:M:339:GLU:HG3	4:M:412:ARG:HG2	1.58	0.84
1:A:581:LEU:HG	1:A:585:PHE:CE2	2.13	0.84
2:B:219:TYR:CZ	2:B:226:LEU:N	2.46	0.84
2:B:403:ILE:CG2	2:B:439:CYS:SG	2.66	0.84
4:M:336:ASP:OD1	4:M:415:ILE:O	1.96	0.84
1:A:274:LEU:O	1:A:275:LEU:C	2.13	0.83
1:A:450:TYR:HH	1:A:476:GLN:HG2	1.38	0.83
2:B:512:VAL:HG22	2:B:533:LEU:HD22	1.59	0.83
4:M:212:ASN:ND2	4:M:249:TYR:O	2.11	0.83
4:M:243:ILE:N	4:M:474:THR:HG22	1.93	0.83
4:M:364:VAL:O	4:M:367:ALA:O	1.95	0.83
4:M:378:ILE:O	4:M:413:GLY:HA3	1.77	0.83
1:A:189:PHE:CB	1:A:225:LEU:HD21	2.08	0.83
2:B:9:ALA:C	4:M:14:LEU:HB3	1.97	0.83
2:B:103:LEU:HD12	4:M:123:LEU:CD1	1.97	0.83
2:B:274:PRO:HG2	2:B:295:ASN:CA	2.08	0.83
2:B:403:ILE:CD1	2:B:439:CYS:CB	2.49	0.83
2:B:515:PHE:CD2	2:B:529:VAL:HG21	2.13	0.83
3:S:31:LEU:O	3:S:35:VAL:HG13	1.79	0.83
4:M:47:SER:HB2	4:M:50:TYR:CD1	2.12	0.83
4:M:65:TYR:CE1	4:M:86:PRO:HB3	2.13	0.83
4:M:442:GLN:HG3	4:M:443:SER:H	1.43	0.83
1:A:107:TYR:CD1	1:A:128:LEU:HD21	2.13	0.83
2:B:230:PHE:HZ	2:B:252:LEU:HD22	1.10	0.83
2:B:252:LEU:HD13	2:B:302:PHE:HD1	1.35	0.83
3:S:25:LEU:O	3:S:26:PRO:C	2.16	0.83
4:M:478:ASN:O	4:M:479:PHE:O	1.96	0.83
1:A:88:ASN:CB	1:A:120:ILE:HD12	2.09	0.83
2:B:162:VAL:HG23	2:B:173:VAL:HG11	1.58	0.83
2:B:217:GLU:OE2	4:M:133:GLU:OE1	1.97	0.83
2:B:340:ILE:CG1	2:B:373:LEU:HD23	2.08	0.83
2:B:347:VAL:O	2:B:350:THR:N	2.10	0.83
3:S:16:LEU:HD13	3:S:125:TRP:NE1	1.92	0.83
4:M:261:ASN:HB2	4:M:450:GLU:HG2	1.59	0.83
1:A:240:LEU:O	1:A:241:TYR:C	2.09	0.83
1:A:504:ILE:O	1:A:505:ASN:C	2.10	0.83
2:B:27:THR:HB	2:B:57:ARG:HD2	1.60	0.83
2:B:423:HIS:CD2	4:M:365:GLU:HB3	2.12	0.83
4:M:244:VAL:HB	4:M:300:LEU:HG	1.60	0.83
4:M:443:SER:OG	4:M:447:ILE:C	2.17	0.83
1:A:568:GLU:OE1	1:A:571:ARG:NH1	2.10	0.83

Continued on next page...

Continued from previous page...

Atom-1	Atom-2	Interatomic distance (Å)	Clash overlap (Å)
2:B:351:GLU:HB3	4:M:476:THR:CG2	2.09	0.83
3:S:130:SER:CB	3:S:156:LEU:CD1	2.57	0.83
4:M:380:ARG:O	4:M:411:LEU:HA	1.76	0.83
2:B:29:LYS:H	2:B:30:LEU:HA	1.44	0.83
2:B:378:THR:HG23	2:B:379:LYS:N	1.94	0.83
2:B:127:LEU:HD11	2:B:142:LEU:CD1	2.09	0.83
2:B:436:LEU:CD1	2:B:454:LEU:CD2	2.56	0.83
2:B:519:ALA:O	2:B:523:PHE:CD1	2.31	0.83
3:S:35:VAL:CB	3:S:77:TYR:OH	2.26	0.83
2:B:34:SER:O	2:B:37:TYR:CB	2.25	0.83
2:B:83:PHE:CD1	2:B:115:LEU:HD12	2.14	0.83
1:A:99:LYS:HG2	1:A:101:GLN:H	1.44	0.83
1:A:495:ILE:CG2	1:A:515:CYS:HB3	2.07	0.83
2:B:152:PRO:HA	2:B:188:TYR:HE1	1.44	0.83
2:B:181:TYR:HE1	2:B:222:HIS:NE2	1.76	0.83
2:B:252:LEU:CD1	2:B:302:PHE:CE1	2.62	0.83
2:B:340:ILE:HB	2:B:373:LEU:HG	1.61	0.83
2:B:403:ILE:HD12	2:B:439:CYS:C	1.99	0.83
2:B:408:VAL:HB	2:B:446:TRP:CD1	2.14	0.83
4:M:223:HIS:CG	4:M:476:THR:HG1	1.95	0.83
4:M:276:VAL:HG22	4:M:290:PHE:CD1	2.13	0.83
2:B:374:PHE:HE2	2:B:402:LEU:CD1	1.42	0.82
2:B:398:ILE:HG22	2:B:402:LEU:HD11	1.58	0.82
4:M:235:LEU:HD13	4:M:310:VAL:HG21	1.59	0.82
4:M:374:TYR:OH	4:M:394:GLN:C	2.17	0.82
2:B:90:ILE:N	2:B:101:ILE:HD13	1.94	0.82
2:B:318:ILE:HD13	2:B:346:THR:HG1	1.41	0.82
2:B:429:VAL:HG12	2:B:467:VAL:CG1	2.09	0.82
4:M:220:GLU:CG	4:M:439:TYR:CD2	2.59	0.82
2:B:100:LEU:HD21	4:M:123:LEU:CD2	2.09	0.82
2:B:106:LEU:CG	4:M:130:GLU:CB	2.40	0.82
2:B:337:THR:CB	2:B:373:LEU:HD13	2.05	0.82
2:B:415:LEU:HD12	2:B:436:LEU:CD2	2.09	0.82
4:M:245:ASP:HB3	4:M:472:TYR:HD1	1.41	0.82
4:M:327:PHE:CE1	4:M:336:ASP:HB2	2.14	0.82
2:B:72:SER:C	4:M:17:GLN:HE22	1.82	0.82
2:B:301:LEU:O	2:B:305:SER:OG	1.97	0.82
3:S:16:LEU:CD1	3:S:125:TRP:HE1	1.92	0.82
1:A:222:ILE:HG21	1:A:240:LEU:HD11	1.61	0.82
1:A:536:MET:SD	1:A:551:LEU:CD1	2.68	0.82
3:S:16:LEU:CB	3:S:125:TRP:HE1	1.91	0.82

Continued on next page...

Continued from previous page...

Atom-1	Atom-2	Interatomic distance (Å)	Clash overlap (Å)
1:A:409:VAL:CG1	3:S:42:ARG:NH1	2.42	0.82
2:B:5:ILE:HD11	4:M:39:PRO:HG3	1.60	0.82
2:B:302:PHE:CE2	2:B:328:LEU:HD11	2.13	0.82
2:B:458:MET:SD	2:B:471:TYR:HB3	2.19	0.82
2:B:580:TYR:HB3	2:B:582:ASP:CG	1.99	0.82
3:S:54:PRO:HD2	3:S:57:LEU:HB2	1.59	0.82
4:M:97:ASP:O	4:M:100:LEU:HB2	1.77	0.82
2:B:212:VAL:CG2	2:B:233:TYR:CE1	2.62	0.82
4:M:100:LEU:HD22	4:M:121:ILE:HG12	1.60	0.82
1:A:384:LEU:HD22	1:A:441:TYR:CD2	2.15	0.82
1:A:411:GLU:OE2	3:S:46:PHE:HE1	1.62	0.82
2:B:127:LEU:CD1	2:B:157:THR:HG21	2.10	0.82
2:B:139:LEU:HD11	2:B:176:ALA:CB	2.09	0.82
2:B:219:TYR:HE2	2:B:226:LEU:HD13	1.03	0.82
2:B:343:LEU:HD22	2:B:366:LEU:HD12	1.61	0.82
2:B:563:PHE:CD1	2:B:584:SER:CA	2.62	0.82
4:M:47:SER:O	4:M:75:TRP:HH2	1.61	0.82
4:M:243:ILE:N	4:M:474:THR:CG2	2.42	0.82
1:A:503:ASN:ND2	4:M:60:LEU:HG	1.95	0.82
2:B:259:TYR:C	2:B:261:PRO:N	2.23	0.82
2:B:267:ASP:H	2:B:289:PRO:CG	1.92	0.82
2:B:337:THR:C	2:B:373:LEU:HD11	1.95	0.82
1:A:606:PHE:CZ	1:A:633:PHE:CD1	2.68	0.82
2:B:136:CYS:C	2:B:172:GLU:HG3	2.00	0.82
2:B:216:LYS:NZ	4:M:136:VAL:HG21	1.95	0.82
3:S:6:LEU:HD22	3:S:32:LEU:HD22	1.62	0.82
4:M:271:SER:HB3	4:M:301:GLU:CG	2.08	0.82
1:A:213:SER:CB	3:S:142:ILE:HB	2.10	0.81
2:B:107:ARG:NH2	4:M:18:TYR:OH	2.12	0.81
2:B:109:ALA:CB	2:B:145:MET:SD	2.69	0.81
2:B:219:TYR:HB3	2:B:223:LEU:CD2	2.10	0.81
2:B:253:ILE:HG12	2:B:324:ALA:CA	2.10	0.81
2:B:379:LYS:HE2	2:B:410:GLU:CG	2.02	0.81
2:B:438:ARG:HA	2:B:441:GLN:HE21	1.45	0.81
2:B:513:TRP:O	2:B:516:GLY:N	2.12	0.81
1:A:100:LEU:O	1:A:101:GLN:O	1.96	0.81
1:A:216:SER:O	1:A:219:VAL:HB	1.80	0.81
2:B:347:VAL:CG1	2:B:381:PHE:CE1	2.63	0.81
4:M:304:VAL:HG11	4:M:445:SER:HA	1.62	0.81
1:A:504:ILE:CA	4:M:59:ASP:CG	2.46	0.81
1:A:581:LEU:HB3	1:A:607:LEU:HD13	1.61	0.81

Continued on next page...

Continued from previous page...

Atom-1	Atom-2	Interatomic distance (Å)	Clash overlap (Å)
2:B:17:VAL:HB	2:B:35:TYR:CD2	2.14	0.81
2:B:252:LEU:C	2:B:302:PHE:CE2	2.53	0.81
2:B:278:PRO:CA	2:B:288:TYR:HB2	2.10	0.81
4:M:212:ASN:HB3	4:M:250:LEU:HA	1.62	0.81
4:M:223:HIS:HA	4:M:479:PHE:CG	2.14	0.81
4:M:272:LEU:CD2	4:M:278:ILE:HB	2.08	0.81
1:A:204:VAL:HG13	1:A:239:LEU:CD1	2.09	0.81
1:A:346:THR:O	1:A:347:ASP:C	2.15	0.81
1:A:638:LEU:CD2	2:B:561:ASP:CG	2.47	0.81
2:B:67:ILE:O	4:M:18:TYR:HE1	1.62	0.81
2:B:230:PHE:HE1	2:B:234:CYS:SG	1.96	0.81
2:B:486:HIS:HE1	2:B:518:ILE:HD13	1.39	0.81
3:S:89:VAL:HG11	3:S:98:ILE:CG1	2.10	0.81
4:M:288:ILE:HD12	4:M:300:LEU:HD22	1.61	0.81
1:A:92:LEU:HD23	1:A:95:MET:HE3	1.63	0.81
1:A:121:LEU:HD13	1:A:155:THR:HG23	1.60	0.81
1:A:492:ILE:HG21	1:A:526:VAL:HG21	1.62	0.81
2:B:14:THR:CG2	2:B:40:GLN:HG2	2.09	0.81
2:B:38:TYR:CZ	2:B:46:GLN:HB2	2.16	0.81
2:B:70:MET:HE3	2:B:107:ARG:CD	2.10	0.81
2:B:348:THR:CB	4:M:305:ASP:OD2	2.28	0.81
4:M:67:SER:OG	4:M:90:PHE:HD1	1.62	0.81
4:M:293:PRO:HB2	4:M:294:ASP:O	1.79	0.81
1:A:503:ASN:ND2	4:M:60:LEU:HD23	1.94	0.81
1:A:585:PHE:HE2	1:A:603:VAL:HG11	1.33	0.81
2:B:13:ASP:O	2:B:17:VAL:HG13	1.80	0.81
2:B:106:LEU:HB3	4:M:130:GLU:CB	2.11	0.81
2:B:344:VAL:HG21	2:B:377:TYR:CG	2.15	0.81
4:M:350:VAL:CG1	4:M:442:GLN:CB	2.57	0.81
1:A:506:LYS:HE3	4:M:82:LYS:HB2	1.59	0.81
2:B:268:LYS:HA	2:B:276:SER:HB2	1.60	0.81
2:B:399:LEU:HD12	2:B:415:LEU:HD21	1.62	0.81
2:B:563:PHE:CD1	2:B:584:SER:CB	2.62	0.81
2:B:588:ILE:HG23	2:B:618:PHE:HZ	1.46	0.81
3:S:80:TYR:O	3:S:82:THR:N	2.14	0.81
3:S:89:VAL:HG11	3:S:98:ILE:CG2	2.11	0.81
4:M:218:LEU:O	4:M:441:GLY:HA2	1.81	0.81
2:B:72:SER:HA	4:M:17:GLN:HE22	0.64	0.81
2:B:261:PRO:HB2	2:B:290:SER:CB	2.10	0.81
4:M:306:LEU:HD13	4:M:317:MET:CE	2.10	0.81
4:M:327:PHE:CE1	4:M:336:ASP:OD2	2.33	0.81

Continued on next page...

Continued from previous page...

Atom-1	Atom-2	Interatomic distance (Å)	Clash overlap (Å)
4:M:354:ASP:O	4:M:438:SER:O	1.99	0.81
4:M:435:LEU:O	4:M:479:PHE:CE1	2.32	0.81
1:A:429:VAL:HG11	1:A:473:ILE:CG1	2.11	0.81
1:A:506:LYS:O	1:A:507:GLN:HB2	1.79	0.81
2:B:106:LEU:HB3	4:M:130:GLU:HB3	1.62	0.81
2:B:290:SER:O	2:B:291:TYR:C	1.88	0.81
2:B:377:TYR:O	2:B:380:LYS:HB2	1.80	0.81
3:S:3:HIS:NE2	3:S:90:ASP:OD2	2.13	0.81
2:B:70:MET:HE3	2:B:107:ARG:HD2	1.62	0.81
2:B:197:LYS:HB2	2:B:229:HIS:CD2	2.16	0.81
2:B:227:HIS:HA	2:B:298:ASP:OD2	1.81	0.81
2:B:383:VAL:O	2:B:385:PRO:CD	2.29	0.81
2:B:566:ALA:HB2	2:B:581:TYR:CD1	2.16	0.81
3:S:6:LEU:HD11	3:S:14:PRO:HB3	1.63	0.81
1:A:128:LEU:HD13	1:A:150:LEU:HG	1.62	0.80
2:B:44:PRO:CB	2:B:82:TYR:OH	2.28	0.80
2:B:106:LEU:HD21	4:M:130:GLU:CA	2.10	0.80
2:B:435:SER:O	2:B:438:ARG:N	2.13	0.80
4:M:223:HIS:CD2	4:M:478:ASN:HA	2.16	0.80
4:M:428:VAL:O	4:M:429:ASP:C	2.17	0.80
2:B:252:LEU:O	2:B:302:PHE:HE2	1.62	0.80
2:B:278:PRO:CG	2:B:292:GLU:OE1	2.28	0.80
2:B:308:CYS:O	2:B:312:SER:N	2.10	0.80
2:B:337:THR:HA	2:B:373:LEU:CD2	2.11	0.80
2:B:349:MET:HG2	4:M:305:ASP:HB2	1.63	0.80
1:A:189:PHE:HB2	1:A:225:LEU:HD21	1.61	0.80
2:B:16:LYS:NZ	4:M:111:ILE:CD1	2.44	0.80
2:B:193:LEU:HD22	2:B:225:LEU:HB3	0.81	0.80
2:B:232:ARG:O	2:B:236:ILE:HG13	1.81	0.80
2:B:243:TRP:HZ3	4:M:98:ARG:HD2	1.37	0.80
2:B:430:ILE:HG12	2:B:467:VAL:HA	1.64	0.80
2:B:599:ALA:C	2:B:601:TYR:N	2.31	0.80
3:S:80:TYR:O	3:S:81:ALA:C	2.14	0.80
4:M:120:ARG:O	4:M:124:ILE:HD12	1.81	0.80
2:B:219:TYR:CE1	2:B:226:LEU:N	2.48	0.80
2:B:267:ASP:N	2:B:289:PRO:HG3	1.96	0.80
4:M:51:LEU:HB3	4:M:68:VAL:CG2	2.09	0.80
4:M:288:ILE:HD12	4:M:300:LEU:CD2	2.11	0.80
2:B:278:PRO:HA	2:B:288:TYR:HB2	1.62	0.80
2:B:418:TYR:O	2:B:419:VAL:C	2.08	0.80
2:B:430:ILE:HD11	2:B:466:SER:CB	2.12	0.80

Continued on next page...

Continued from previous page...

Atom-1	Atom-2	Interatomic distance (Å)	Clash overlap (Å)
4:M:66:PHE:HD1	4:M:78:ALA:O	1.64	0.80
1:A:121:LEU:HD12	1:A:153:ILE:HG22	1.63	0.80
1:A:533:ILE:HG12	1:A:562:TRP:CH2	2.16	0.80
2:B:22:ALA:CB	2:B:33:SER:OG	2.27	0.80
2:B:193:LEU:HB3	2:B:225:LEU:CD1	2.11	0.80
2:B:213:LEU:CD2	4:M:136:VAL:HB	2.11	0.80
2:B:274:PRO:HG2	2:B:295:ASN:HB3	1.64	0.80
2:B:310:ILE:HA	2:B:318:ILE:HG12	1.62	0.80
2:B:343:LEU:HD22	2:B:366:LEU:CD1	2.12	0.80
3:S:55:PRO:HA	3:S:69:ASN:HB3	1.63	0.80
4:M:449:VAL:CG1	4:M:452:ILE:HG13	2.11	0.80
1:A:136:GLY:O	1:A:139:ASP:CB	2.30	0.80
1:A:411:GLU:OE2	3:S:46:PHE:CZ	2.35	0.80
1:A:536:MET:O	1:A:537:THR:O	2.00	0.80
2:B:73:ASP:N	4:M:19:LEU:HD23	1.76	0.80
2:B:396:ILE:HG21	2:B:432:ALA:N	1.96	0.80
4:M:347:PHE:CD1	4:M:350:VAL:CG1	2.65	0.80
4:M:353:VAL:HG23	4:M:438:SER:O	1.81	0.80
1:A:536:MET:SD	1:A:551:LEU:HD11	2.21	0.80
1:A:606:PHE:CZ	1:A:633:PHE:CG	2.59	0.80
2:B:243:TRP:HH2	4:M:98:ARG:HH11	1.30	0.80
2:B:389:ILE:O	2:B:390:VAL:C	2.12	0.80
2:B:69:ILE:C	2:B:71:ALA:N	2.34	0.80
2:B:243:TRP:HH2	4:M:98:ARG:NH1	1.78	0.80
3:S:53:THR:CG2	3:S:68:VAL:HA	2.11	0.80
4:M:122:SER:O	4:M:125:PHE:HB2	1.81	0.80
1:A:298:ILE:O	1:A:299:VAL:C	2.18	0.80
2:B:14:THR:CB	2:B:40:GLN:HG2	2.12	0.80
2:B:103:LEU:HD21	4:M:127:CYS:SG	2.22	0.80
2:B:144:ASP:OD1	4:M:131:ALA:CA	2.28	0.79
2:B:162:VAL:HG22	2:B:199:LEU:CG	2.12	0.79
2:B:278:PRO:HB3	2:B:288:TYR:O	1.82	0.79
2:B:318:ILE:HG21	2:B:346:THR:CB	2.12	0.79
2:B:344:VAL:HG22	2:B:374:PHE:HE1	1.43	0.79
2:B:347:VAL:CB	2:B:381:PHE:CE1	2.65	0.79
3:S:43:ASN:O	3:S:44:SER:C	2.15	0.79
4:M:16:PHE:CE2	4:M:125:PHE:CE2	2.69	0.79
4:M:222:PHE:CE2	4:M:439:TYR:HE2	1.99	0.79
1:A:536:MET:HG2	1:A:551:LEU:CD1	2.11	0.79
1:A:225:LEU:HB2	1:A:233:PHE:CZ	2.16	0.79
1:A:399:ASP:H	1:A:418:ILE:HD11	1.46	0.79

Continued on next page...

Continued from previous page...

Atom-1	Atom-2	Interatomic distance (Å)	Clash overlap (Å)
2:B:162:VAL:HG22	2:B:199:LEU:CD1	2.12	0.79
2:B:405:GLU:C	2:B:446:TRP:HE1	1.84	0.79
1:A:506:LYS:CE	4:M:82:LYS:HB3	1.98	0.79
1:A:609:LEU:HG	1:A:628:VAL:HG11	1.65	0.79
3:S:53:THR:HB	3:S:69:ASN:CB	2.13	0.79
4:M:290:PHE:HB2	4:M:299:LEU:HD11	1.63	0.79
1:A:599:ARG:NE	2:B:610:ARG:HH22	1.79	0.79
2:B:16:LYS:HZ3	4:M:111:ILE:CD1	1.95	0.79
2:B:69:ILE:HG22	2:B:77:ILE:HG13	1.62	0.79
2:B:378:THR:O	2:B:380:LYS:N	2.15	0.79
2:B:474:VAL:O	2:B:477:MET:N	2.14	0.79
2:B:580:TYR:HB3	2:B:582:ASP:OD1	1.83	0.79
4:M:343:ASN:HA	4:M:408:VAL:CG1	2.08	0.79
1:A:182:ILE:HG22	1:A:221:VAL:CG2	2.12	0.79
1:A:558:VAL:HG13	1:A:562:TRP:NE1	1.97	0.79
2:B:18:ILE:CD1	2:B:36:THR:CG2	2.49	0.79
2:B:53:SER:OG	2:B:58:GLU:OE1	2.00	0.79
2:B:108:PHE:O	2:B:111:ASN:N	2.15	0.79
2:B:199:LEU:C	2:B:201:ALA:H	1.86	0.79
2:B:215:TYR:HD1	2:B:219:TYR:HE2	1.25	0.79
2:B:277:CYS:SG	2:B:292:GLU:HG3	2.22	0.79
3:S:8:PHE:CG	3:S:36:TYR:CE1	2.71	0.79
1:A:251:TRP:CZ3	3:S:97:ALA:C	2.31	0.79
1:A:328:PRO:O	3:S:50:PHE:HZ	1.65	0.79
2:B:179:LYS:NZ	4:M:131:ALA:CA	2.46	0.79
2:B:589:SER:HA	2:B:592:TYR:HD2	1.47	0.79
4:M:65:TYR:CZ	4:M:86:PRO:CB	2.61	0.79
4:M:104:PHE:CZ	4:M:117:ASN:CB	2.65	0.79
4:M:334:ASP:O	4:M:417:TYR:N	2.15	0.79
2:B:106:LEU:CG	2:B:144:ASP:CB	2.61	0.79
2:B:107:ARG:HH12	4:M:20:LEU:CG	1.95	0.79
2:B:167:ALA:O	2:B:207:VAL:HG21	1.82	0.79
4:M:120:ARG:O	4:M:124:ILE:CD1	2.31	0.79
4:M:235:LEU:CD1	4:M:306:LEU:HB3	2.12	0.79
4:M:240:ILE:CG2	4:M:444:ALA:CA	2.61	0.79
2:B:12:LEU:CD2	4:M:13:LYS:HG3	2.13	0.79
2:B:82:TYR:HB2	2:B:104:TYR:CZ	2.17	0.79
4:M:44:ASP:CB	4:M:50:TYR:CE2	2.64	0.79
4:M:218:LEU:HG	4:M:244:VAL:HG22	1.64	0.79
1:A:558:VAL:HG12	1:A:562:TRP:CE2	2.17	0.79
2:B:158:VAL:HG13	2:B:173:VAL:CG1	2.12	0.79

Continued on next page...

Continued from previous page...

Atom-1	Atom-2	Interatomic distance (Å)	Clash overlap (Å)
4:M:217:ASP:CB	4:M:470:ALA:O	2.30	0.79
1:A:394:GLN:O	1:A:398:GLU:N	2.14	0.78
2:B:103:LEU:HD13	4:M:123:LEU:CD1	2.12	0.78
2:B:243:TRP:CH2	4:M:98:ARG:NH1	2.51	0.78
1:A:244:LEU:HG	1:A:281:LEU:HD11	1.64	0.78
1:A:638:LEU:HD11	2:B:557:SER:C	2.01	0.78
2:B:12:LEU:HB3	4:M:13:LYS:HG3	1.46	0.78
2:B:223:LEU:HD11	2:B:258:GLN:HB2	1.60	0.78
2:B:310:ILE:CG2	2:B:342:ALA:CB	2.60	0.78
3:S:53:THR:OG1	3:S:68:VAL:C	2.21	0.78
4:M:383:HIS:CB	4:M:403:THR:OG1	2.32	0.78
1:A:395:PHE:CE2	1:A:420:ILE:HD13	2.18	0.78
2:B:16:LYS:HD3	4:M:115:VAL:CG2	2.09	0.78
2:B:80:GLN:CG	2:B:108:PHE:CZ	2.66	0.78
2:B:252:LEU:HD12	2:B:302:PHE:CE1	2.18	0.78
2:B:274:PRO:HG2	2:B:275:ARG:O	1.69	0.78
1:A:179:LYS:HZ3	3:S:141:VAL:C	1.87	0.78
1:A:182:ILE:CG2	1:A:221:VAL:HG21	2.12	0.78
1:A:320:HIS:O	1:A:321:THR:C	2.19	0.78
4:M:7:ILE:CD1	4:M:121:ILE:HG21	2.12	0.78
4:M:214:LEU:O	4:M:467:TYR:N	2.16	0.78
4:M:356:LEU:CD2	4:M:358:ILE:CG1	2.62	0.78
1:A:405:THR:HA	2:B:7:ARG:HH21	0.64	0.78
4:M:317:MET:HB3	4:M:320:ILE:O	1.84	0.78
1:A:95:MET:SD	1:A:107:TYR:CD1	2.75	0.78
1:A:223:CYS:O	1:A:226:SER:OG	2.00	0.78
1:A:536:MET:CG	1:A:551:LEU:CD1	2.61	0.78
1:A:605:GLU:OE2	1:A:608:ARG:NH2	2.16	0.78
2:B:232:ARG:HG3	2:B:236:ILE:CD1	2.13	0.78
3:S:4:ALA:HB2	3:S:19:PHE:HD1	1.49	0.78
3:S:15:ARG:O	3:S:125:TRP:CZ2	2.36	0.78
1:A:332:TYR:CZ	1:A:336:ILE:HD11	2.18	0.78
1:A:384:LEU:HG	1:A:385:LYS:H	1.49	0.78
2:B:106:LEU:C	4:M:130:GLU:OE1	2.22	0.78
2:B:178:ILE:HG13	2:B:214:ALA:O	1.84	0.78
2:B:212:VAL:CG2	2:B:248:LEU:CD2	2.60	0.78
2:B:272:GLY:O	2:B:274:PRO:HD3	1.84	0.78
2:B:309:LEU:CB	2:B:317:VAL:CG1	2.57	0.78
4:M:306:LEU:CD1	4:M:317:MET:HE3	2.14	0.78
2:B:309:LEU:CB	2:B:317:VAL:HG11	2.13	0.78
2:B:310:ILE:O	2:B:311:TYR:O	2.01	0.78

Continued on next page...

Continued from previous page...

Atom-1	Atom-2	Interatomic distance (Å)	Clash overlap (Å)
2:B:313:SER:CB	4:M:269:ILE:CB	2.42	0.78
2:B:340:ILE:HG13	2:B:373:LEU:HB3	1.66	0.78
2:B:444:THR:HG22	2:B:482:ASN:OD1	1.84	0.78
3:S:35:VAL:HB	3:S:77:TYR:CZ	2.18	0.78
4:M:240:ILE:HG22	4:M:444:ALA:CA	2.14	0.78
1:A:104:ARG:HA	1:A:145:ILE:HG21	1.66	0.78
1:A:200:PHE:CZ	1:A:236:LEU:CD2	2.66	0.78
1:A:558:VAL:CG1	1:A:562:TRP:NE1	2.47	0.78
2:B:12:LEU:HD13	4:M:13:LYS:HG3	1.14	0.78
2:B:72:SER:C	4:M:19:LEU:CB	2.52	0.78
2:B:347:VAL:CG1	2:B:381:PHE:HE1	1.95	0.78
4:M:9:ASP:CB	4:M:111:ILE:HG21	2.11	0.78
4:M:78:ALA:CB	4:M:89:CYS:SG	2.72	0.78
4:M:293:PRO:O	4:M:293:PRO:CD	2.32	0.78
4:M:319:SER:HB3	4:M:343:ASN:O	1.84	0.78
2:B:16:LYS:NZ	4:M:111:ILE:CG1	2.46	0.78
2:B:162:VAL:CG2	2:B:195:ILE:HG23	2.09	0.78
2:B:178:ILE:CG1	2:B:214:ALA:CA	2.57	0.78
2:B:189:HIS:NE2	2:B:193:LEU:HD11	1.99	0.78
2:B:245:GLN:NE2	2:B:309:LEU:CD1	2.40	0.78
2:B:274:PRO:CG	2:B:295:ASN:CA	2.61	0.78
4:M:121:ILE:O	4:M:125:PHE:HD1	1.67	0.78
4:M:284:SER:O	4:M:285:PRO:C	2.11	0.78
1:A:397:ASP:O	1:A:418:ILE:HG13	1.84	0.77
2:B:140:SER:HB2	2:B:172:GLU:CD	2.05	0.77
2:B:188:TYR:O	2:B:192:LEU:HD13	1.82	0.77
2:B:199:LEU:C	2:B:201:ALA:N	2.33	0.77
2:B:215:TYR:CD1	2:B:219:TYR:HE2	2.01	0.77
2:B:534:ILE:HD13	2:B:591:MET:O	1.84	0.77
2:B:534:ILE:CD1	2:B:591:MET:O	2.32	0.77
4:M:7:ILE:HA	4:M:76:CYS:HA	1.66	0.77
4:M:131:ALA:CB	4:M:131:ALA:N	2.44	0.77
4:M:243:ILE:HD13	4:M:301:GLU:HB3	1.66	0.77
4:M:343:ASN:H	4:M:343:ASN:HD22	1.28	0.77
1:A:103:LYS:HB3	1:A:107:TYR:HE2	1.49	0.77
2:B:274:PRO:HG3	2:B:295:ASN:HA	1.65	0.77
2:B:340:ILE:HG21	2:B:374:PHE:CA	2.14	0.77
1:A:213:SER:CB	3:S:142:ILE:CA	2.62	0.77
2:B:38:TYR:OH	2:B:46:GLN:CD	2.17	0.77
2:B:102:HIS:ND1	2:B:137:PHE:HB3	1.99	0.77
4:M:350:VAL:HA	4:M:442:GLN:CB	2.13	0.77

Continued on next page...

Continued from previous page...

Atom-1	Atom-2	Interatomic distance (Å)	Clash overlap (Å)
2:B:72:SER:CA	4:M:17:GLN:NE2	2.05	0.77
2:B:565:GLN:HB2	2:B:581:TYR:CE1	2.19	0.77
4:M:265:ASN:HA	4:M:313:SER:OG	1.85	0.77
4:M:282:VAL:N	4:M:282:VAL:C	2.35	0.77
2:B:278:PRO:CD	2:B:289:PRO:O	2.32	0.77
2:B:294:VAL:C	2:B:299:LEU:HD12	2.05	0.77
2:B:437:SER:OG	2:B:477:MET:HB2	1.84	0.77
4:M:215:TYR:N	4:M:467:TYR:HB3	1.98	0.77
4:M:222:PHE:CE1	4:M:240:ILE:HG21	2.18	0.77
4:M:224:VAL:N	4:M:479:PHE:CD2	2.52	0.77
2:B:63:MET:HG2	2:B:100:LEU:HB3	1.65	0.77
2:B:219:TYR:CE1	2:B:226:LEU:CB	2.57	0.77
2:B:328:LEU:HB3	2:B:333:GLN:NE2	2.00	0.77
4:M:45:SER:HB2	4:M:75:TRP:CZ2	2.19	0.77
4:M:43:GLU:O	4:M:45:SER:N	2.17	0.77
4:M:51:LEU:HB2	4:M:68:VAL:CB	2.15	0.77
4:M:59:ASP:O	4:M:61:GLU:N	2.17	0.77
4:M:323:MET:SD	4:M:342:LEU:CA	2.73	0.77
2:B:126:SER:O	2:B:135:ARG:HG2	1.83	0.77
2:B:574:ASN:O	2:B:576:GLN:O	2.02	0.77
3:S:53:THR:HB	3:S:68:VAL:C	2.05	0.77
4:M:224:VAL:HG22	4:M:306:LEU:HD12	1.65	0.77
4:M:253:ASN:OD1	4:M:292:PRO:HD2	1.84	0.77
4:M:283:PHE:CE2	4:M:289:THR:CB	2.68	0.77
4:M:306:LEU:HD21	4:M:317:MET:CE	2.14	0.77
4:M:343:ASN:CA	4:M:408:VAL:HG13	2.06	0.77
1:A:151:SER:HB2	1:A:187:LYS:HB2	1.65	0.77
2:B:14:THR:HA	2:B:17:VAL:HG22	1.66	0.77
3:S:53:THR:HB	3:S:69:ASN:N	2.00	0.77
4:M:96:ILE:HD11	4:M:125:PHE:HA	1.67	0.77
4:M:224:VAL:O	4:M:479:PHE:HB3	1.84	0.77
4:M:262:THR:HG22	4:M:264:GLY:N	2.00	0.77
2:B:102:HIS:O	2:B:103:LEU:C	2.18	0.77
2:B:403:ILE:HG21	2:B:439:CYS:CB	2.15	0.77
2:B:433:VAL:CG1	2:B:474:VAL:CG2	2.60	0.77
2:B:438:ARG:HA	2:B:441:GLN:NE2	1.99	0.77
2:B:564:LYS:HG3	2:B:568:VAL:HG23	1.67	0.77
4:M:5:PHE:CE2	4:M:20:LEU:CD1	2.68	0.77
4:M:69:ILE:CG2	4:M:97:ASP:OD2	2.33	0.77
4:M:131:ALA:CB	4:M:131:ALA:C	2.47	0.77
1:A:300:LYS:C	1:A:302:ASN:N	2.34	0.76

Continued on next page...

Continued from previous page...

Atom-1	Atom-2	Interatomic distance (Å)	Clash overlap (Å)
1:A:348:PHE:O	1:A:352:PHE:CD2	2.38	0.76
2:B:14:THR:OG1	2:B:40:GLN:HG2	1.85	0.76
2:B:415:LEU:CD1	2:B:436:LEU:HD21	2.15	0.76
2:B:444:THR:O	2:B:445:SER:C	2.19	0.76
2:B:477:MET:O	2:B:480:GLN:HB2	1.85	0.76
2:B:16:LYS:HD2	4:M:115:VAL:HG21	1.64	0.76
3:S:35:VAL:HB	3:S:77:TYR:HE2	1.47	0.76
4:M:262:THR:CG2	4:M:265:ASN:N	2.43	0.76
1:A:349:ILE:CG2	1:A:378:ILE:HG22	2.15	0.76
1:A:426:ILE:HD13	1:A:466:ASP:OD2	1.85	0.76
1:A:462:GLN:C	4:M:58:ARG:HB3	2.04	0.76
2:B:13:ASP:C	2:B:17:VAL:HG22	2.04	0.76
2:B:38:TYR:HE2	2:B:43:ASN:C	1.81	0.76
2:B:186:ASN:C	2:B:188:TYR:N	2.37	0.76
2:B:568:VAL:O	2:B:571:SER:CB	2.32	0.76
4:M:131:ALA:C	4:M:133:GLU:H	1.85	0.76
4:M:220:GLU:N	4:M:439:TYR:O	2.13	0.76
2:B:151:ALA:CA	2:B:180:LEU:HD11	2.14	0.76
2:B:243:TRP:CH2	4:M:98:ARG:NE	2.52	0.76
2:B:433:VAL:HG13	2:B:471:TYR:HA	1.66	0.76
4:M:45:SER:HB2	4:M:75:TRP:CH2	2.21	0.76
4:M:71:LYS:HB3	4:M:74:TYR:CE1	2.20	0.76
4:M:222:PHE:C	4:M:479:PHE:CE1	2.53	0.76
1:A:121:LEU:CD1	1:A:153:ILE:HG22	2.16	0.76
1:A:281:LEU:O	1:A:282:MET:C	2.12	0.76
1:A:316:LEU:HD11	1:A:348:PHE:CD2	2.20	0.76
1:A:402:ILE:HD13	3:S:62:GLU:O	1.11	0.76
2:B:519:ALA:O	2:B:523:PHE:HD1	1.66	0.76
4:M:6:TYR:CD2	4:M:17:GLN:HG2	2.21	0.76
4:M:47:SER:O	4:M:49:ASP:N	2.19	0.76
4:M:105:ASP:O	4:M:106:LYS:HB2	1.83	0.76
4:M:317:MET:O	4:M:322:LEU:HB3	1.86	0.76
1:A:83:ASP:OD2	1:A:85:ALA:N	2.18	0.76
1:A:581:LEU:HD23	1:A:607:LEU:CD2	2.16	0.76
2:B:106:LEU:CG	4:M:130:GLU:OE1	2.32	0.76
2:B:162:VAL:CB	2:B:195:ILE:HG23	2.15	0.76
3:S:73:ILE:HG23	3:S:88:ILE:CG2	2.13	0.76
3:S:109:LEU:O	3:S:110:ASP:C	2.10	0.76
4:M:437:TYR:HD1	4:M:479:PHE:CZ	2.01	0.76
1:A:407:SER:N	3:S:64:ASN:HD21	1.84	0.76
2:B:9:ALA:CA	4:M:14:LEU:CB	2.64	0.76

Continued on next page...

Continued from previous page...

Atom-1	Atom-2	Interatomic distance (Å)	Clash overlap (Å)
2:B:23:ALA:O	2:B:32:GLU:OE2	2.01	0.76
2:B:189:HIS:CE1	2:B:193:LEU:HD11	2.20	0.76
4:M:20:LEU:HD22	4:M:129:VAL:CB	2.14	0.76
4:M:104:PHE:CE2	4:M:117:ASN:HB2	2.20	0.76
1:A:295:VAL:HG11	1:A:319:LEU:HD11	1.67	0.76
2:B:219:TYR:HD2	2:B:226:LEU:HD22	1.49	0.76
2:B:559:ASP:CB	2:B:563:PHE:CD2	2.69	0.76
3:S:71:GLU:O	3:S:73:ILE:N	2.19	0.76
2:B:268:LYS:O	2:B:273:SER:CB	2.33	0.76
2:B:347:VAL:HG22	2:B:359:LEU:HB3	0.82	0.76
3:S:8:PHE:N	3:S:8:PHE:CD1	2.51	0.76
3:S:48:SER:OG	3:S:50:PHE:N	2.18	0.76
3:S:53:THR:HB	3:S:69:ASN:CA	2.15	0.76
4:M:67:SER:HG	4:M:90:PHE:HD1	1.33	0.76
4:M:242:GLY:O	4:M:301:GLU:HA	1.86	0.76
4:M:354:ASP:HA	4:M:401:LYS:HB3	1.67	0.76
4:M:445:SER:OG	4:M:447:ILE:HG23	1.84	0.76
1:A:309:PHE:CZ	1:A:348:PHE:CE1	2.74	0.76
1:A:519:LEU:O	1:A:520:GLY:C	2.16	0.76
2:B:80:GLN:HG2	2:B:108:PHE:CZ	2.21	0.76
2:B:101:ILE:O	2:B:105:LEU:HD13	1.85	0.76
2:B:178:ILE:HD13	2:B:218:CYS:CB	2.16	0.76
3:S:53:THR:HB	3:S:69:ASN:HB2	1.66	0.76
4:M:243:ILE:H	4:M:474:THR:HG22	1.49	0.76
1:A:88:ASN:CG	1:A:120:ILE:HG21	2.07	0.75
1:A:570:LYS:O	1:A:571:ARG:HB2	1.84	0.75
2:B:223:LEU:O	2:B:224:GLU:C	2.16	0.75
2:B:261:PRO:HG2	2:B:292:GLU:HB3	1.67	0.75
4:M:6:TYR:O	4:M:77:LEU:N	2.16	0.75
4:M:217:ASP:HB2	4:M:470:ALA:O	1.86	0.75
1:A:203:PHE:CZ	1:A:221:VAL:HG11	2.22	0.75
2:B:18:ILE:HD13	2:B:36:THR:HG22	1.65	0.75
2:B:83:PHE:CE1	2:B:87:VAL:CG2	2.68	0.75
2:B:468:LEU:HD13	2:B:503:LEU:CD2	2.15	0.75
4:M:66:PHE:CD1	4:M:78:ALA:O	2.39	0.75
1:A:147:LEU:HD22	1:A:166:LEU:HD23	1.66	0.75
2:B:278:PRO:CB	2:B:288:TYR:O	2.35	0.75
2:B:344:VAL:HG21	2:B:377:TYR:HB3	1.66	0.75
2:B:418:TYR:CZ	2:B:432:ALA:HB2	2.21	0.75
2:B:513:TRP:N	2:B:551:LEU:CD1	2.49	0.75
2:B:5:ILE:CD1	4:M:39:PRO:N	2.49	0.75

Continued on next page...

Continued from previous page...

Atom-1	Atom-2	Interatomic distance (Å)	Clash overlap (Å)
2:B:106:LEU:CB	4:M:130:GLU:CB	2.65	0.75
2:B:127:LEU:HD11	2:B:142:LEU:HD12	1.69	0.75
2:B:197:LYS:HA	2:B:229:HIS:CD2	2.21	0.75
2:B:248:LEU:O	2:B:252:LEU:HG	1.86	0.75
2:B:278:PRO:CA	2:B:288:TYR:C	2.55	0.75
4:M:54:SER:HB2	4:M:66:PHE:CE2	2.21	0.75
4:M:323:MET:CE	4:M:342:LEU:HB3	2.16	0.75
1:A:101:GLN:O	1:A:104:ARG:N	2.19	0.75
2:B:82:TYR:HB2	2:B:104:TYR:HH	1.52	0.75
2:B:250:GLU:O	2:B:253:ILE:HB	1.86	0.75
2:B:280:PRO:HG3	2:B:283:TYR:CE2	2.21	0.75
2:B:515:PHE:O	2:B:516:GLY:C	2.17	0.75
4:M:15:ILE:CG2	4:M:114:ILE:HG22	2.17	0.75
4:M:41:LEU:HB3	4:M:51:LEU:HA	1.67	0.75
1:A:492:ILE:HD11	1:A:522:PHE:HB2	1.68	0.75
2:B:79:VAL:C	2:B:108:PHE:HE2	1.89	0.75
2:B:185:LYS:NZ	2:B:221:ASP:OD2	2.19	0.75
2:B:277:CYS:HA	2:B:292:GLU:HA	1.69	0.75
2:B:473:ASN:O	2:B:476:ARG:HB3	1.86	0.75
4:M:379:LEU:HD23	4:M:411:LEU:CG	2.16	0.75
1:A:369:SER:HB2	1:A:424:TYR:CE2	2.21	0.75
1:A:395:PHE:CZ	1:A:428:MET:HG3	2.22	0.75
2:B:483:PRO:HA	2:B:486:HIS:HB2	1.69	0.75
4:M:70:ASN:HA	4:M:74:TYR:O	1.85	0.75
4:M:220:GLU:HG3	4:M:439:TYR:HD2	1.36	0.75
4:M:245:ASP:CB	4:M:472:TYR:HD1	1.92	0.75
2:B:178:ILE:HD12	2:B:218:CYS:N	1.98	0.75
2:B:307:ASN:O	2:B:311:TYR:N	2.20	0.75
2:B:427:ASN:HA	2:B:430:ILE:HD12	1.67	0.75
2:B:470:ALA:O	2:B:473:ASN:N	2.20	0.75
2:B:512:VAL:HG13	2:B:533:LEU:HD11	1.62	0.75
1:A:88:ASN:HB2	1:A:120:ILE:HD12	1.69	0.75
1:A:372:ILE:CG2	1:A:431:VAL:HG21	2.17	0.75
1:A:469:LEU:O	1:A:470:GLY:C	2.21	0.75
2:B:37:TYR:HD2	2:B:38:TYR:HD1	1.31	0.75
2:B:144:ASP:CA	2:B:179:LYS:HD3	2.16	0.75
2:B:219:TYR:O	2:B:223:LEU:CD2	2.34	0.75
2:B:526:CYS:N	2:B:527:PRO:CD	2.48	0.75
4:M:65:TYR:CE2	4:M:86:PRO:HA	2.22	0.75
4:M:320:ILE:HG23	4:M:439:TYR:OH	1.86	0.75
1:A:503:ASN:ND2	4:M:60:LEU:CG	2.50	0.74

Continued on next page...

Continued from previous page...

Atom-1	Atom-2	Interatomic distance (Å)	Clash overlap (Å)
2:B:181:TYR:CE1	2:B:222:HIS:NE2	2.54	0.74
2:B:212:VAL:O	2:B:214:ALA:N	2.19	0.74
2:B:219:TYR:O	2:B:220:ALA:C	2.23	0.74
2:B:567:GLN:C	2:B:569:THR:OG1	2.24	0.74
3:S:1:MET:N	3:S:93:GLU:OE1	2.19	0.74
4:M:282:VAL:N	4:M:283:PHE:N	2.34	0.74
4:M:362:PHE:CE1	4:M:374:TYR:CZ	2.74	0.74
1:A:254:ILE:HG13	1:A:290:VAL:HG22	1.69	0.74
1:A:282:MET:O	1:A:283:GLU:C	2.22	0.74
2:B:231:ARG:O	2:B:233:TYR:N	2.20	0.74
2:B:231:ARG:HG2	2:B:297:PRO:HB2	1.69	0.74
3:S:73:ILE:HG23	3:S:88:ILE:HG23	1.69	0.74
4:M:268:GLY:H	4:M:302:TYR:HH	1.34	0.74
4:M:377:LYS:NZ	4:M:416:GLU:OE1	2.20	0.74
1:A:196:LEU:O	1:A:196:LEU:HD22	1.87	0.74
1:A:379:VAL:HG11	1:A:441:TYR:OH	1.86	0.74
1:A:429:VAL:CG2	1:A:469:LEU:HD11	2.17	0.74
2:B:549:LEU:CD2	2:B:607:ILE:O	2.34	0.74
4:M:6:TYR:CD2	4:M:17:GLN:CG	2.70	0.74
4:M:15:ILE:HG21	4:M:114:ILE:CG2	2.17	0.74
4:M:283:PHE:CE2	4:M:289:THR:OG1	2.40	0.74
4:M:327:PHE:HE1	4:M:336:ASP:HB2	1.52	0.74
4:M:380:ARG:O	4:M:410:VAL:O	2.05	0.74
1:A:258:LYS:NZ	3:S:93:GLU:C	2.40	0.74
2:B:17:VAL:HG23	2:B:35:TYR:HD2	1.43	0.74
2:B:120:ILE:CD1	2:B:150:LEU:HD13	2.17	0.74
2:B:216:LYS:HG3	2:B:251:LEU:CD1	2.16	0.74
2:B:374:PHE:CE2	2:B:398:ILE:HG21	2.20	0.74
2:B:559:ASP:HB2	2:B:563:PHE:CD2	2.22	0.74
4:M:71:LYS:HG2	4:M:74:TYR:OH	1.87	0.74
4:M:478:ASN:O	4:M:479:PHE:C	2.25	0.74
1:A:492:ILE:HG23	1:A:519:LEU:CD2	2.18	0.74
1:A:545:HIS:O	1:A:546:SER:C	2.17	0.74
2:B:5:ILE:HD11	4:M:39:PRO:CA	2.17	0.74
2:B:69:ILE:C	2:B:71:ALA:H	1.91	0.74
1:A:68:THR:CB	3:S:166:LYS:HD3	2.17	0.74
2:B:34:SER:O	2:B:37:TYR:N	2.20	0.74
2:B:347:VAL:HA	2:B:359:LEU:HG	1.68	0.74
4:M:54:SER:N	4:M:66:PHE:HD2	1.84	0.74
4:M:245:ASP:N	4:M:472:TYR:CZ	2.55	0.74
1:A:136:GLY:O	1:A:139:ASP:CA	2.35	0.74

Continued on next page...

Continued from previous page...

Atom-1	Atom-2	Interatomic distance (Å)	Clash overlap (Å)
2:B:25:VAL:HG21	2:B:31:GLY:N	2.01	0.74
2:B:216:LYS:HB2	2:B:251:LEU:HD13	0.74	0.74
2:B:422:ALA:CB	2:B:424:PHE:CE2	2.67	0.74
4:M:71:LYS:O	4:M:74:TYR:CD2	2.41	0.74
4:M:235:LEU:HD11	4:M:306:LEU:HB3	1.67	0.74
4:M:267:ILE:HD12	4:M:445:SER:OG	1.88	0.74
1:A:260:PHE:CG	1:A:274:LEU:HG	2.22	0.74
2:B:312:SER:C	4:M:269:ILE:CD1	2.50	0.74
4:M:12:ASN:CG	4:M:42:LEU:HB3	2.08	0.74
4:M:262:THR:HG22	4:M:264:GLY:CA	2.18	0.74
2:B:123:LEU:HD13	2:B:142:LEU:HG	1.68	0.74
2:B:151:ALA:HB3	2:B:188:TYR:CE2	2.23	0.74
2:B:400:SER:CB	2:B:435:SER:HB3	2.09	0.74
2:B:478:LEU:O	2:B:479:VAL:C	2.19	0.74
2:B:560:ILE:CG2	2:B:564:LYS:HB2	2.18	0.74
4:M:226:PHE:HB2	4:M:481:VAL:HG22	1.69	0.74
1:A:566:PHE:CD1	1:A:570:LYS:HA	2.23	0.74
2:B:120:ILE:HA	2:B:142:LEU:HD21	1.70	0.74
2:B:223:LEU:HD11	2:B:258:GLN:CA	2.15	0.74
3:S:75:ILE:HG22	3:S:77:TYR:CE1	2.23	0.74
4:M:70:ASN:HB2	4:M:75:TRP:CE3	2.23	0.74
1:A:186:PHE:CD2	1:A:224:GLU:CB	2.71	0.73
2:B:17:VAL:HG11	2:B:35:TYR:HE2	1.53	0.73
2:B:124:GLN:CD	2:B:153:ILE:HG23	2.09	0.73
2:B:197:LYS:CB	2:B:229:HIS:CD2	2.71	0.73
2:B:276:SER:C	2:B:295:ASN:HB2	2.06	0.73
2:B:472:VAL:HG11	2:B:510:GLY:CA	2.17	0.73
3:S:130:SER:CB	3:S:156:LEU:HD13	2.18	0.73
1:A:158:LEU:HG	1:A:162:ILE:CD1	2.18	0.73
1:A:213:SER:CB	3:S:142:ILE:HA	2.18	0.73
1:A:421:PRO:HB3	3:S:63:ASN:N	2.02	0.73
2:B:136:CYS:CA	2:B:172:GLU:HG3	2.17	0.73
2:B:139:LEU:CD1	2:B:176:ALA:HB2	2.18	0.73
2:B:140:SER:CB	2:B:172:GLU:OE1	2.36	0.73
2:B:179:LYS:CE	4:M:131:ALA:O	2.36	0.73
2:B:472:VAL:HG11	2:B:511:ILE:N	2.03	0.73
4:M:283:PHE:CE2	4:M:289:THR:HB	2.23	0.73
4:M:374:TYR:HA	4:M:417:TYR:HA	1.70	0.73
1:A:71:VAL:CG1	1:A:105:VAL:HG12	2.17	0.73
2:B:12:LEU:HD22	4:M:13:LYS:HE3	1.70	0.73
3:S:35:VAL:HB	3:S:77:TYR:OH	1.87	0.73

Continued on next page...

Continued from previous page...

Atom-1	Atom-2	Interatomic distance (Å)	Clash overlap (Å)
4:M:250:LEU:CD1	4:M:254:PRO:HG2	2.18	0.73
1:A:579:LYS:O	1:A:582:ILE:HB	1.89	0.73
2:B:253:ILE:CG1	2:B:324:ALA:CB	2.66	0.73
2:B:374:PHE:C	2:B:402:LEU:HD22	2.08	0.73
3:S:49:SER:O	3:S:77:TYR:HB2	1.89	0.73
4:M:214:LEU:C	4:M:467:TYR:H	1.91	0.73
1:A:163:ALA:O	1:A:164:ASP:C	2.24	0.73
2:B:279:LEU:HD12	2:B:285:GLU:HG2	1.69	0.73
2:B:367:SER:CB	2:B:401:THR:HG1	2.02	0.73
2:B:375:LEU:CD1	2:B:404:ASN:CB	2.56	0.73
2:B:554:LYS:O	2:B:557:SER:N	2.21	0.73
2:B:559:ASP:HA	2:B:562:ASN:HB2	1.69	0.73
3:S:135:ILE:HG23	3:S:141:VAL:CG1	2.18	0.73
4:M:42:LEU:HD23	4:M:51:LEU:HD21	1.69	0.73
1:A:215:VAL:O	1:A:216:SER:C	2.19	0.73
2:B:12:LEU:HD13	4:M:13:LYS:HB2	1.63	0.73
2:B:28:SER:CA	2:B:58:GLU:HG2	2.19	0.73
2:B:69:ILE:CG2	2:B:77:ILE:HG13	2.19	0.73
2:B:204:ASP:O	2:B:207:VAL:HB	1.89	0.73
2:B:223:LEU:HD13	2:B:259:TYR:CA	2.15	0.73
2:B:596:LEU:HD12	2:B:611:ALA:CB	2.18	0.73
4:M:19:LEU:HD21	4:M:24:ALA:CB	2.19	0.73
4:M:224:VAL:HG11	4:M:226:PHE:CZ	2.23	0.73
4:M:225:VAL:HB	4:M:237:THR:OG1	1.88	0.73
4:M:375:LYS:HE3	4:M:418:GLU:OE1	1.88	0.73
4:M:449:VAL:HG11	4:M:452:ILE:CG1	2.19	0.73
1:A:114:PHE:CG	1:A:153:ILE:HG12	2.22	0.73
2:B:1:MET:HG3	4:M:39:PRO:HG2	1.71	0.73
2:B:5:ILE:HG23	4:M:42:LEU:CD1	2.18	0.73
2:B:318:ILE:HD13	2:B:346:THR:CG2	2.18	0.73
2:B:318:ILE:HG21	2:B:346:THR:OG1	1.87	0.73
2:B:63:MET:CG	2:B:100:LEU:HB3	2.18	0.73
2:B:73:ASP:HA	4:M:19:LEU:HD22	0.88	0.73
2:B:249:ILE:HG12	2:B:306:LEU:CD2	2.19	0.73
2:B:375:LEU:HD12	2:B:404:ASN:ND2	1.82	0.73
3:S:89:VAL:HG11	3:S:98:ILE:HG13	1.68	0.73
1:A:536:MET:CB	1:A:551:LEU:HD11	2.18	0.73
2:B:215:TYR:CE2	2:B:233:TYR:CE2	2.76	0.73
2:B:259:TYR:CD1	2:B:261:PRO:CG	2.61	0.73
2:B:347:VAL:HG21	2:B:381:PHE:HE1	1.54	0.73
2:B:375:LEU:HD22	2:B:404:ASN:HB2	1.70	0.73

Continued on next page...

Continued from previous page...

Atom-1	Atom-2	Interatomic distance (Å)	Clash overlap (Å)
2:B:403:ILE:CD1	2:B:439:CYS:HB3	2.16	0.73
3:S:53:THR:O	3:S:69:ASN:CG	2.27	0.73
3:S:73:ILE:HG21	3:S:88:ILE:CG2	2.17	0.73
4:M:224:VAL:O	4:M:480:GLN:N	2.20	0.73
4:M:246:VAL:O	4:M:297:PHE:CE1	2.42	0.73
4:M:362:PHE:CE1	4:M:374:TYR:CE2	2.77	0.73
4:M:373:ALA:HB3	4:M:418:GLU:O	1.88	0.73
4:M:405:THR:C	4:M:407:THR:H	1.90	0.73
1:A:88:ASN:HB3	1:A:120:ILE:HD12	1.71	0.73
1:A:92:LEU:O	1:A:95:MET:N	2.22	0.73
1:A:420:ILE:HG23	1:A:424:TYR:HB2	1.71	0.73
1:A:595:GLU:CD	2:B:513:TRP:CH2	2.63	0.73
1:A:606:PHE:HZ	1:A:633:PHE:CD1	2.05	0.73
2:B:109:ALA:O	2:B:111:ASN:N	2.22	0.73
2:B:178:ILE:HD11	2:B:215:TYR:HA	1.71	0.73
2:B:253:ILE:HG12	2:B:324:ALA:CB	2.19	0.73
2:B:373:LEU:C	2:B:375:LEU:H	1.92	0.73
2:B:501:THR:C	2:B:508:ARG:NH2	2.41	0.73
3:S:16:LEU:CG	3:S:125:TRP:HE1	2.02	0.73
4:M:106:LYS:NZ	4:M:296:LYS:HE3	2.03	0.73
1:A:186:PHE:HB2	1:A:221:VAL:HG13	1.72	0.72
1:A:219:VAL:HG21	1:A:256:LEU:HD21	1.70	0.72
1:A:409:VAL:HG11	3:S:42:ARG:HH12	1.50	0.72
2:B:25:VAL:HG23	2:B:32:GLU:CG	2.19	0.72
2:B:35:TYR:CE1	4:M:118:TYR:OH	2.42	0.72
2:B:404:ASN:O	2:B:405:GLU:C	2.08	0.72
4:M:215:TYR:O	4:M:246:VAL:HG13	1.89	0.72
4:M:262:THR:HG22	4:M:265:ASN:N	2.04	0.72
2:B:72:SER:CB	4:M:17:GLN:CD	2.56	0.72
2:B:129:ASP:O	2:B:135:ARG:HD3	1.88	0.72
2:B:328:LEU:CB	2:B:333:GLN:NE2	2.49	0.72
1:A:488:ARG:HD2	1:A:522:PHE:CE2	2.24	0.72
2:B:208:ILE:HD13	2:B:236:ILE:CG2	2.18	0.72
2:B:249:ILE:CD1	2:B:321:CYS:SG	2.77	0.72
4:M:214:LEU:HD23	4:M:214:LEU:C	2.09	0.72
4:M:344:ILE:HG22	4:M:347:PHE:CB	1.97	0.72
4:M:347:PHE:CE1	4:M:350:VAL:HB	2.23	0.72
4:M:347:PHE:CD1	4:M:350:VAL:HG11	2.22	0.72
4:M:19:LEU:HD21	4:M:24:ALA:HB3	1.70	0.72
1:A:405:THR:O	2:B:7:ARG:CZ	2.38	0.72
2:B:196:LEU:HB3	2:B:215:TYR:OH	1.88	0.72

Continued on next page...

Continued from previous page...

Atom-1	Atom-2	Interatomic distance (Å)	Clash overlap (Å)
2:B:363:ILE:HG22	2:B:398:ILE:HG12	1.70	0.72
2:B:494:ALA:HB2	2:B:515:PHE:CZ	2.24	0.72
2:B:549:LEU:CD1	2:B:595:VAL:HG12	2.20	0.72
4:M:53:HIS:HA	4:M:66:PHE:HB2	1.70	0.72
1:A:136:GLY:O	1:A:137:ASN:C	2.18	0.72
1:A:186:PHE:CD2	1:A:224:GLU:HB3	2.23	0.72
2:B:158:VAL:HG13	2:B:177:ILE:HG13	1.71	0.72
2:B:512:VAL:HG22	2:B:533:LEU:CD2	2.19	0.72
2:B:540:GLU:OE1	2:B:548:ILE:CD1	2.37	0.72
4:M:220:GLU:OE2	4:M:439:TYR:HD2	1.65	0.72
4:M:235:LEU:HD11	4:M:306:LEU:CB	2.19	0.72
4:M:242:GLY:CA	4:M:474:THR:HG21	2.20	0.72
4:M:352:GLN:HG3	4:M:440:ILE:HB	1.70	0.72
1:A:316:LEU:HD13	1:A:348:PHE:CG	2.24	0.72
1:A:407:SER:N	3:S:64:ASN:ND2	2.38	0.72
2:B:13:ASP:OD1	4:M:15:ILE:N	2.22	0.72
2:B:25:VAL:HA	2:B:28:SER:H	1.55	0.72
2:B:260:LEU:CA	2:B:293:VAL:HG21	2.18	0.72
2:B:347:VAL:HG21	2:B:381:PHE:CZ	2.25	0.72
2:B:397:GLN:HG2	2:B:431:MET:SD	2.29	0.72
4:M:281:GLY:C	4:M:281:GLY:N	2.41	0.72
1:A:503:ASN:HB3	4:M:60:LEU:N	2.04	0.72
1:A:513:ARG:HG3	1:A:547:VAL:HG22	1.69	0.72
2:B:294:VAL:O	2:B:299:LEU:HD12	1.90	0.72
4:M:218:LEU:CA	4:M:472:TYR:CD2	2.64	0.72
4:M:302:TYR:CE1	4:M:445:SER:HB2	2.24	0.72
4:M:437:TYR:CD1	4:M:437:TYR:N	2.54	0.72
2:B:247:TYR:HD1	4:M:136:VAL:HG12	1.51	0.72
2:B:360:LEU:CD2	2:B:395:LYS:HD3	2.20	0.72
2:B:549:LEU:HD21	2:B:611:ALA:N	2.04	0.72
4:M:244:VAL:HG13	4:M:472:TYR:CZ	2.25	0.72
4:M:343:ASN:HD22	4:M:343:ASN:N	1.87	0.72
4:M:433:VAL:HG12	4:M:481:VAL:HB	1.70	0.72
1:A:114:PHE:C	1:A:115:TYR:O	2.27	0.72
1:A:322:PHE:CD1	1:A:330:LEU:HD21	2.24	0.72
1:A:535:ILE:O	1:A:535:ILE:HG22	1.89	0.72
1:A:566:PHE:C	1:A:568:GLU:H	1.90	0.72
2:B:231:ARG:O	2:B:234:CYS:N	2.23	0.72
2:B:259:TYR:O	2:B:293:VAL:CG2	2.37	0.72
2:B:360:LEU:HD21	2:B:395:LYS:HD3	1.71	0.72
2:B:367:SER:OG	2:B:401:THR:HG21	1.89	0.72

Continued on next page...

Continued from previous page...

Atom-1	Atom-2	Interatomic distance (Å)	Clash overlap (Å)
2:B:415:LEU:CD1	2:B:436:LEU:CD2	2.68	0.72
1:A:212:ILE:O	1:A:213:SER:C	2.16	0.71
1:A:585:PHE:HB3	1:A:600:SER:OG	1.89	0.71
2:B:472:VAL:O	2:B:473:ASN:C	2.18	0.71
2:B:512:VAL:CG1	2:B:533:LEU:CD1	2.36	0.71
4:M:223:HIS:CG	4:M:476:THR:OG1	2.43	0.71
1:A:213:SER:CB	3:S:142:ILE:CB	2.67	0.71
3:S:1:MET:H2	3:S:93:GLU:HB2	1.54	0.71
3:S:112:CYS:HB2	3:S:113:PHE:CD1	2.25	0.71
4:M:67:SER:O	4:M:77:LEU:HA	1.91	0.71
4:M:99:ILE:O	4:M:99:ILE:CG2	2.38	0.71
4:M:443:SER:OG	4:M:448:TYR:N	2.23	0.71
2:B:143:SER:CB	2:B:179:LYS:CD	2.67	0.71
2:B:219:TYR:O	2:B:223:LEU:HD21	1.91	0.71
4:M:279:ASN:HB2	4:M:283:PHE:CD2	2.24	0.71
4:M:449:VAL:HG11	4:M:452:ILE:HG13	1.69	0.71
1:A:405:THR:HB	2:B:7:ARG:HE	1.54	0.71
1:A:503:ASN:CB	4:M:60:LEU:N	2.52	0.71
1:A:539:ASN:O	1:A:540:ILE:C	2.18	0.71
2:B:73:ASP:H	4:M:19:LEU:CB	1.98	0.71
4:M:437:TYR:CD1	4:M:479:PHE:CE2	2.78	0.71
2:B:80:GLN:HA	2:B:108:PHE:CE2	2.26	0.71
2:B:106:LEU:HD21	4:M:130:GLU:HA	1.72	0.71
2:B:347:VAL:CG2	2:B:381:PHE:CE1	2.73	0.71
3:S:57:LEU:O	3:S:59:LEU:HA	1.90	0.71
4:M:6:TYR:CD1	4:M:77:LEU:HD23	2.26	0.71
4:M:435:LEU:HB2	4:M:437:TYR:CE1	2.25	0.71
1:A:225:LEU:HD12	1:A:233:PHE:CZ	2.26	0.71
1:A:266:VAL:O	1:A:267:GLU:HB3	1.91	0.71
1:A:292:TYR:CD1	1:A:292:TYR:O	2.44	0.71
1:A:562:TRP:O	1:A:565:ASN:N	2.23	0.71
1:A:573:GLU:O	1:A:575:LYS:N	2.23	0.71
2:B:24:ALA:HB3	2:B:32:GLU:CG	2.07	0.71
2:B:28:SER:O	2:B:58:GLU:CD	2.28	0.71
2:B:367:SER:CB	2:B:401:THR:CB	2.68	0.71
2:B:379:LYS:CE	2:B:410:GLU:HG3	2.02	0.71
3:S:4:ALA:CB	3:S:19:PHE:CD1	2.74	0.71
3:S:55:PRO:O	3:S:58:LEU:CB	2.39	0.71
4:M:244:VAL:HA	4:M:472:TYR:CG	2.18	0.71
1:A:350:SER:O	1:A:351:ARG:C	2.23	0.71
1:A:366:SER:HB3	3:S:67:GLU:OE1	1.90	0.71

Continued on next page...

Continued from previous page...

Atom-1	Atom-2	Interatomic distance (Å)	Clash overlap (Å)
1:A:421:PRO:HB3	3:S:62:GLU:C	2.11	0.71
1:A:503:ASN:HB3	4:M:59:ASP:CA	2.07	0.71
2:B:14:THR:HG21	2:B:40:GLN:CG	2.20	0.71
2:B:155:LEU:HB2	2:B:188:TYR:CD1	2.26	0.71
2:B:181:TYR:CZ	2:B:185:LYS:HE2	2.26	0.71
2:B:347:VAL:HG22	2:B:359:LEU:CG	2.20	0.71
2:B:472:VAL:CG1	2:B:510:GLY:CA	2.68	0.71
3:S:75:ILE:CG2	3:S:77:TYR:CE1	2.74	0.71
4:M:16:PHE:CZ	4:M:18:TYR:HB2	2.24	0.71
1:A:379:VAL:CG1	1:A:441:TYR:OH	2.38	0.71
1:A:638:LEU:CD1	2:B:557:SER:CA	2.68	0.71
2:B:18:ILE:CG2	2:B:36:THR:C	2.36	0.71
2:B:226:LEU:CD2	2:B:255:TYR:HD1	1.83	0.71
2:B:545:ARG:HD2	2:B:602:ASP:OD2	1.91	0.71
3:S:57:LEU:CA	3:S:58:LEU:O	2.30	0.71
4:M:78:ALA:HB1	4:M:89:CYS:SG	2.30	0.71
4:M:244:VAL:O	4:M:299:LEU:HB3	1.90	0.71
4:M:275:CYS:O	4:M:291:ILE:HG22	1.91	0.71
4:M:347:PHE:CE2	4:M:350:VAL:O	2.43	0.71
1:A:384:LEU:HG	1:A:385:LYS:N	2.05	0.71
1:A:578:LEU:HD23	1:A:607:LEU:CD2	2.19	0.71
2:B:1:MET:CG	4:M:39:PRO:CG	2.68	0.71
2:B:212:VAL:CG2	2:B:233:TYR:CD1	2.74	0.71
2:B:216:LYS:NZ	2:B:250:GLU:OE2	2.23	0.71
4:M:378:ILE:O	4:M:413:GLY:CA	2.39	0.71
2:B:73:ASP:H	4:M:19:LEU:HB3	1.55	0.71
2:B:336:ASN:HB3	2:B:339:PHE:CD2	2.25	0.71
2:B:396:ILE:HG12	2:B:418:TYR:HE2	1.56	0.71
3:S:89:VAL:HG11	3:S:98:ILE:HG21	1.71	0.71
4:M:244:VAL:CG1	4:M:472:TYR:CE2	2.74	0.71
2:B:337:THR:OG1	2:B:373:LEU:CD1	2.27	0.70
2:B:566:ALA:CA	2:B:574:ASN:HB3	2.19	0.70
4:M:74:TYR:HB2	4:M:114:ILE:CD1	2.20	0.70
1:A:71:VAL:HG12	1:A:105:VAL:HG12	1.71	0.70
1:A:206:LYS:HE2	1:A:206:LYS:HA	1.73	0.70
1:A:316:LEU:HD13	1:A:348:PHE:CD2	2.26	0.70
2:B:80:GLN:N	2:B:108:PHE:CE2	2.57	0.70
2:B:107:ARG:NH1	4:M:20:LEU:CB	2.51	0.70
2:B:382:TYR:OH	2:B:410:GLU:CB	2.38	0.70
2:B:398:ILE:O	2:B:400:SER:N	2.24	0.70
2:B:457:HIS:HA	2:B:461:HIS:CD2	2.25	0.70

Continued on next page...

Continued from previous page...

Atom-1	Atom-2	Interatomic distance (Å)	Clash overlap (Å)
2:B:475:ILE:CG2	2:B:489:ILE:CG2	2.69	0.70
2:B:505:ASP:O	2:B:506:ASN:C	2.22	0.70
2:B:512:VAL:HG13	2:B:533:LEU:HD13	0.72	0.70
2:B:513:TRP:NE1	2:B:517:GLU:CG	2.53	0.70
2:B:559:ASP:O	2:B:562:ASN:HB2	1.91	0.70
2:B:567:GLN:HA	2:B:569:THR:OG1	1.90	0.70
3:S:8:PHE:CZ	3:S:86:THR:OG1	2.42	0.70
4:M:242:GLY:CA	4:M:444:ALA:HB2	2.20	0.70
1:A:254:ILE:HD13	3:S:96:LEU:HB2	1.72	0.70
1:A:277:LYS:HG3	1:A:277:LYS:O	1.91	0.70
2:B:169:VAL:O	2:B:173:VAL:HG23	1.90	0.70
2:B:245:GLN:CD	2:B:309:LEU:HD12	1.96	0.70
4:M:356:LEU:HD21	4:M:358:ILE:CG1	2.20	0.70
1:A:121:LEU:CD1	1:A:153:ILE:CG2	2.69	0.70
1:A:508:LEU:HD12	4:M:59:ASP:CG	2.10	0.70
2:B:82:TYR:O	2:B:83:PHE:C	2.21	0.70
2:B:178:ILE:HG12	2:B:214:ALA:CB	2.11	0.70
2:B:230:PHE:CD1	2:B:298:ASP:CB	2.73	0.70
2:B:472:VAL:HG12	2:B:510:GLY:HA3	1.73	0.70
4:M:334:ASP:O	4:M:416:GLU:HA	1.91	0.70
1:A:316:LEU:CD1	1:A:348:PHE:CE2	2.74	0.70
1:A:581:LEU:CD1	1:A:585:PHE:CZ	2.73	0.70
2:B:100:LEU:CD2	4:M:123:LEU:HD22	2.19	0.70
2:B:399:LEU:HD22	2:B:411:ILE:HG23	1.74	0.70
1:A:384:LEU:CG	1:A:385:LYS:N	2.54	0.70
1:A:558:VAL:HG11	1:A:562:TRP:CZ2	2.26	0.70
1:A:585:PHE:CB	1:A:600:SER:OG	2.40	0.70
1:A:606:PHE:CE1	1:A:633:PHE:CB	2.59	0.70
2:B:53:SER:CB	2:B:58:GLU:OE1	2.40	0.70
2:B:73:ASP:N	4:M:19:LEU:CG	2.54	0.70
2:B:103:LEU:HD13	4:M:123:LEU:HD12	1.71	0.70
2:B:219:TYR:HE2	2:B:226:LEU:CD1	1.74	0.70
2:B:371:GLN:HG2	2:B:401:THR:HB	1.72	0.70
4:M:78:ALA:HB3	4:M:89:CYS:SG	2.31	0.70
4:M:243:ILE:H	4:M:474:THR:HG21	1.56	0.70
1:A:156:PRO:O	1:A:157:SER:C	2.24	0.70
1:A:462:GLN:O	1:A:463:ASP:C	2.28	0.70
1:A:585:PHE:C	1:A:600:SER:OG	2.25	0.70
1:A:595:GLU:CD	2:B:513:TRP:CE3	2.43	0.70
2:B:337:THR:HG1	2:B:373:LEU:HD13	1.54	0.70
4:M:106:LYS:O	4:M:108:LYS:N	2.24	0.70

Continued on next page...

Continued from previous page...

Atom-1	Atom-2	Interatomic distance (Å)	Clash overlap (Å)
4:M:215:TYR:HB2	4:M:467:TYR:O	1.90	0.70
1:A:370:LYS:O	1:A:374:LEU:HD13	1.91	0.70
1:A:506:LYS:O	1:A:507:GLN:CB	2.39	0.70
2:B:252:LEU:CB	2:B:302:PHE:CD1	2.74	0.70
4:M:48:ASP:CA	4:M:70:ASN:HD22	2.03	0.70
4:M:317:MET:HB2	4:M:322:LEU:N	2.07	0.70
1:A:213:SER:OG	3:S:142:ILE:HB	1.91	0.70
1:A:373:GLU:CG	1:A:427:LYS:HE2	2.21	0.70
2:B:29:LYS:HA	2:B:30:LEU:HD23	1.74	0.70
2:B:50:LEU:HD23	2:B:62:ALA:CA	2.22	0.70
2:B:245:GLN:OE1	2:B:309:LEU:CD1	2.28	0.70
2:B:337:THR:CA	2:B:373:LEU:HD13	2.08	0.70
3:S:8:PHE:CG	3:S:36:TYR:HE1	2.09	0.70
4:M:338:PHE:CD1	4:M:415:ILE:HG13	2.27	0.70
1:A:506:LYS:CE	4:M:82:LYS:CD	2.70	0.70
2:B:322:CYS:SG	2:B:366:LEU:CG	2.79	0.70
2:B:403:ILE:HG21	2:B:439:CYS:HB3	1.72	0.70
4:M:443:SER:OG	4:M:448:TYR:HA	1.92	0.70
1:A:462:GLN:OE1	4:M:60:LEU:N	2.25	0.69
2:B:73:ASP:OD1	4:M:19:LEU:HD11	1.88	0.69
3:S:65:ASN:O	3:S:67:GLU:HG3	1.91	0.69
1:A:186:PHE:CZ	1:A:224:GLU:CG	2.64	0.69
1:A:393:LYS:NZ	1:A:397:ASP:OD2	2.25	0.69
2:B:285:GLU:O	2:B:286:ILE:C	2.27	0.69
2:B:472:VAL:CG1	2:B:511:ILE:N	2.55	0.69
2:B:513:TRP:HA	2:B:551:LEU:CD1	2.22	0.69
4:M:100:LEU:HD21	4:M:121:ILE:HG12	1.73	0.69
4:M:101:LEU:C	4:M:103:TYR:O	2.30	0.69
4:M:241:HIS:O	4:M:474:THR:CG2	2.40	0.69
4:M:243:ILE:HD13	4:M:301:GLU:CB	2.21	0.69
4:M:306:LEU:HD22	4:M:317:MET:HE2	1.73	0.69
1:A:329:ASN:N	3:S:50:PHE:HE2	1.87	0.69
1:A:391:LEU:O	1:A:392:MET:O	2.08	0.69
2:B:537:PHE:CE2	2:B:545:ARG:CB	2.76	0.69
1:A:107:TYR:CE1	1:A:128:LEU:CD2	2.75	0.69
2:B:213:LEU:CD1	4:M:135:ASN:CG	2.52	0.69
1:A:67:LYS:O	1:A:71:VAL:HG23	1.92	0.69
1:A:179:LYS:HZ3	3:S:142:ILE:N	1.88	0.69
1:A:323:CYS:CB	1:A:355:LEU:HD21	2.22	0.69
1:A:402:ILE:HG12	1:A:406:GLY:HA2	1.74	0.69
2:B:102:HIS:CE1	2:B:138:ALA:HA	2.27	0.69

Continued on next page...

Continued from previous page...

Atom-1	Atom-2	Interatomic distance (Å)	Clash overlap (Å)
2:B:275:ARG:H	2:B:294:VAL:HG13	1.57	0.69
2:B:468:LEU:HD13	2:B:503:LEU:HD21	1.74	0.69
2:B:513:TRP:CD2	2:B:551:LEU:HD21	2.28	0.69
4:M:108:LYS:O	4:M:110:SER:N	2.25	0.69
4:M:114:ILE:C	4:M:116:ASN:N	2.44	0.69
4:M:350:VAL:CB	4:M:442:GLN:HG2	2.23	0.69
1:A:196:LEU:O	1:A:197:ARG:C	2.25	0.69
1:A:370:LYS:HE2	1:A:370:LYS:HA	1.74	0.69
2:B:106:LEU:HD13	2:B:144:ASP:C	2.13	0.69
2:B:479:VAL:HG22	2:B:486:HIS:CG	2.27	0.69
4:M:222:PHE:HB2	4:M:479:PHE:CZ	2.27	0.69
4:M:243:ILE:HG22	4:M:472:TYR:HB3	1.75	0.69
4:M:290:PHE:CZ	4:M:297:PHE:CZ	2.80	0.69
1:A:332:TYR:CE2	1:A:336:ILE:HD11	2.28	0.69
2:B:154:ILE:O	2:B:158:VAL:HG23	1.93	0.69
2:B:234:CYS:HB3	2:B:301:LEU:HB3	1.75	0.69
2:B:518:ILE:CD1	2:B:518:ILE:O	2.40	0.69
3:S:127:THR:CG2	3:S:153:VAL:CG1	2.70	0.69
4:M:65:TYR:CZ	4:M:66:PHE:O	2.45	0.69
4:M:223:HIS:HA	4:M:479:PHE:CZ	2.26	0.69
4:M:269:ILE:C	4:M:302:TYR:CE2	2.66	0.69
1:A:461:CYS:O	1:A:462:GLN:C	2.22	0.69
1:A:462:GLN:CG	4:M:59:ASP:CG	2.58	0.69
2:B:73:ASP:CG	4:M:19:LEU:HD22	2.12	0.69
2:B:116:THR:CG2	2:B:150:LEU:HD11	2.23	0.69
2:B:189:HIS:NE2	2:B:222:HIS:CB	2.55	0.69
2:B:375:LEU:CD1	2:B:404:ASN:CG	2.48	0.69
2:B:447:GLU:OE1	2:B:485:LYS:CG	2.40	0.69
3:S:135:ILE:HG22	3:S:141:VAL:HG22	1.75	0.69
4:M:43:GLU:C	4:M:45:SER:H	1.96	0.69
4:M:323:MET:HE3	4:M:342:LEU:HB3	1.74	0.69
4:M:327:PHE:HE1	4:M:336:ASP:OD2	1.73	0.69
4:M:379:LEU:HD13	4:M:397:TRP:HE1	1.58	0.69
1:A:121:LEU:HD13	1:A:155:THR:CG2	2.23	0.69
2:B:179:LYS:HE2	4:M:131:ALA:O	1.91	0.69
2:B:318:ILE:CD1	2:B:346:THR:CG2	2.70	0.69
2:B:545:ARG:HH11	2:B:602:ASP:CG	1.96	0.69
4:M:69:ILE:O	4:M:75:TRP:CA	2.39	0.69
4:M:99:ILE:O	4:M:99:ILE:HG22	1.92	0.69
1:A:266:VAL:O	1:A:267:GLU:HB2	1.93	0.69
2:B:243:TRP:CH2	4:M:98:ARG:CZ	2.76	0.69

Continued on next page...

Continued from previous page...

Atom-1	Atom-2	Interatomic distance (Å)	Clash overlap (Å)
2:B:278:PRO:C	2:B:288:TYR:CB	2.59	0.69
2:B:334:MET:O	2:B:373:LEU:CD2	2.41	0.69
2:B:400:SER:HB2	2:B:435:SER:HA	1.75	0.69
2:B:468:LEU:O	2:B:472:VAL:HG23	1.93	0.69
2:B:519:ALA:O	2:B:523:PHE:N	2.26	0.69
1:A:236:LEU:C	1:A:238:PRO:HD2	2.13	0.68
1:A:263:LEU:O	1:A:266:VAL:O	2.11	0.68
2:B:260:LEU:HD22	2:B:291:TYR:CZ	2.28	0.68
2:B:396:ILE:HD11	2:B:418:TYR:CZ	2.28	0.68
4:M:306:LEU:CD2	4:M:317:MET:HE2	2.23	0.68
1:A:555:LEU:HD13	1:A:581:LEU:CD1	2.22	0.68
2:B:100:LEU:CD2	4:M:123:LEU:CD2	2.71	0.68
2:B:139:LEU:CD1	2:B:176:ALA:CB	2.71	0.68
2:B:253:ILE:CG1	2:B:324:ALA:HA	2.22	0.68
2:B:260:LEU:C	2:B:261:PRO:O	2.22	0.68
2:B:346:THR:O	2:B:349:MET:N	2.25	0.68
3:S:53:THR:CB	3:S:69:ASN:HB2	2.22	0.68
4:M:275:CYS:SG	4:M:293:PRO:CB	2.76	0.68
4:M:374:TYR:HE1	4:M:393:GLY:HA2	1.58	0.68
1:A:225:LEU:HD12	1:A:233:PHE:CE1	2.29	0.68
1:A:402:ILE:HG21	3:S:62:GLU:C	2.11	0.68
2:B:25:VAL:CG2	2:B:32:GLU:HG3	2.16	0.68
2:B:97:VAL:HG12	2:B:101:ILE:CD1	2.23	0.68
2:B:147:MET:O	2:B:148:SER:C	2.30	0.68
2:B:310:ILE:CB	2:B:342:ALA:HB1	2.22	0.68
2:B:403:ILE:HD11	2:B:439:CYS:C	1.87	0.68
2:B:418:TYR:CD1	2:B:424:PHE:CD2	2.81	0.68
2:B:537:PHE:HB3	2:B:598:LEU:HD13	1.75	0.68
3:S:87:PHE:CD1	3:S:102:ILE:HG12	2.27	0.68
4:M:443:SER:HB3	4:M:447:ILE:HG12	1.74	0.68
1:A:418:ILE:HG12	1:A:418:ILE:O	1.94	0.68
2:B:17:VAL:HG23	2:B:36:THR:CA	2.23	0.68
2:B:231:ARG:CD	2:B:297:PRO:HB2	2.24	0.68
2:B:566:ALA:N	2:B:574:ASN:ND2	2.33	0.68
3:S:4:ALA:HB1	3:S:19:PHE:CD1	2.28	0.68
4:M:43:GLU:C	4:M:45:SER:N	2.44	0.68
1:A:117:ASP:OD2	1:A:120:ILE:HG12	1.92	0.68
1:A:237:SER:N	1:A:238:PRO:HD2	2.08	0.68
2:B:80:GLN:HG3	2:B:108:PHE:CZ	2.27	0.68
2:B:280:PRO:HG2	2:B:283:TYR:CG	2.28	0.68
2:B:293:VAL:HA	2:B:299:LEU:HG	1.75	0.68

Continued on next page...

Continued from previous page...

Atom-1	Atom-2	Interatomic distance (Å)	Clash overlap (Å)
2:B:340:ILE:CG2	2:B:374:PHE:HA	2.24	0.68
2:B:378:THR:HG23	2:B:379:LYS:H	1.56	0.68
2:B:550:VAL:O	2:B:553:ALA:HB3	1.93	0.68
4:M:220:GLU:OE2	4:M:350:VAL:HG11	1.93	0.68
1:A:137:ASN:C	1:A:139:ASP:H	1.79	0.68
2:B:29:LYS:HB3	2:B:58:GLU:CD	2.14	0.68
2:B:106:LEU:HD11	2:B:144:ASP:O	1.86	0.68
2:B:107:ARG:NH2	4:M:20:LEU:HD21	1.86	0.68
2:B:132:SER:HB2	2:B:169:VAL:HG23	1.74	0.68
2:B:193:LEU:O	2:B:195:ILE:N	2.26	0.68
2:B:580:TYR:HB3	2:B:582:ASP:OD2	1.93	0.68
4:M:65:TYR:CG	4:M:66:PHE:N	2.60	0.68
4:M:288:ILE:CD1	4:M:300:LEU:CD2	2.69	0.68
1:A:64:LEU:HG	1:A:102:GLN:HE22	1.56	0.68
1:A:95:MET:C	1:A:127:LEU:HD21	2.14	0.68
1:A:370:LYS:O	1:A:374:LEU:CD1	2.42	0.68
1:A:398:GLU:HA	1:A:418:ILE:HG13	1.76	0.68
2:B:73:ASP:CB	4:M:24:ALA:HB1	2.24	0.68
2:B:396:ILE:HG23	2:B:432:ALA:HA	1.66	0.68
3:S:54:PRO:CD	3:S:57:LEU:HB2	2.24	0.68
4:M:114:ILE:O	4:M:116:ASN:N	2.27	0.68
4:M:235:LEU:HD11	4:M:306:LEU:HD13	1.74	0.68
4:M:246:VAL:HB	4:M:297:PHE:CZ	2.29	0.68
1:A:101:GLN:HE22	3:S:167:ILE:HD11	1.59	0.68
1:A:450:TYR:HD2	1:A:480:LEU:HG	1.58	0.68
1:A:609:LEU:HD21	1:A:628:VAL:HG21	1.76	0.68
2:B:73:ASP:OD1	4:M:19:LEU:CD2	2.42	0.68
2:B:230:PHE:CD1	2:B:298:ASP:CA	2.76	0.68
2:B:381:PHE:O	2:B:382:TYR:C	2.26	0.68
2:B:508:ARG:O	2:B:509:ALA:C	2.21	0.68
2:B:513:TRP:CE3	2:B:551:LEU:HD21	2.29	0.68
4:M:51:LEU:CB	4:M:68:VAL:CG2	2.70	0.68
4:M:59:ASP:O	4:M:60:LEU:C	2.05	0.68
4:M:290:PHE:CZ	4:M:297:PHE:CE1	2.81	0.68
1:A:186:PHE:CE2	1:A:224:GLU:HB3	2.23	0.68
1:A:438:ALA:O	1:A:441:TYR:CD1	2.47	0.68
2:B:28:SER:CA	2:B:30:LEU:O	2.40	0.68
2:B:181:TYR:CD1	2:B:222:HIS:HD2	2.12	0.68
2:B:363:ILE:HD12	2:B:374:PHE:CE1	2.29	0.68
2:B:383:VAL:HG23	2:B:395:LYS:HG3	1.75	0.68
3:S:71:GLU:C	3:S:73:ILE:H	1.96	0.68

Continued on next page...

Continued from previous page...

Atom-1	Atom-2	Interatomic distance (Å)	Clash overlap (Å)
3:S:130:SER:HB2	3:S:156:LEU:CD1	2.23	0.68
2:B:387:ASP:HB3	2:B:388:PRO:CD	2.24	0.68
4:M:69:ILE:HG21	4:M:97:ASP:OD2	1.94	0.68
4:M:224:VAL:N	4:M:479:PHE:HA	2.09	0.68
4:M:240:ILE:HG21	4:M:444:ALA:O	1.93	0.68
1:A:515:CYS:O	1:A:519:LEU:HG	1.94	0.67
1:A:516:ILE:HG23	1:A:554:ALA:HB3	1.75	0.67
2:B:340:ILE:CB	2:B:373:LEU:HG	2.23	0.67
3:S:71:GLU:C	3:S:73:ILE:N	2.47	0.67
4:M:316:ARG:CG	4:M:322:LEU:HD13	2.24	0.67
4:M:320:ILE:HA	4:M:323:MET:HE2	1.75	0.67
1:A:429:VAL:CG1	1:A:473:ILE:HG12	2.23	0.67
1:A:488:ARG:CD	1:A:522:PHE:CE2	2.77	0.67
1:A:638:LEU:HD23	2:B:561:ASP:OD2	1.93	0.67
2:B:14:THR:HA	2:B:17:VAL:CG2	2.25	0.67
2:B:17:VAL:CG2	2:B:35:TYR:CE2	2.54	0.67
2:B:396:ILE:HD13	2:B:432:ALA:N	2.08	0.67
2:B:433:VAL:C	2:B:474:VAL:HG21	2.14	0.67
3:S:74:GLN:NE2	3:S:95:GLU:OE2	2.27	0.67
4:M:284:SER:O	4:M:286:SER:N	2.27	0.67
1:A:436:CYS:SG	1:A:450:TYR:CE2	2.85	0.67
1:A:530:ASN:O	1:A:534:LYS:O	2.12	0.67
2:B:14:THR:CA	2:B:17:VAL:HG22	2.24	0.67
2:B:34:SER:O	2:B:42:ILE:HD12	1.94	0.67
2:B:37:TYR:CD2	2:B:38:TYR:CD1	2.81	0.67
2:B:106:LEU:CD1	2:B:144:ASP:C	2.62	0.67
2:B:230:PHE:HD1	2:B:298:ASP:CA	2.08	0.67
2:B:296:ASP:OD1	2:B:297:PRO:N	2.26	0.67
4:M:43:GLU:O	4:M:44:ASP:C	2.31	0.67
4:M:220:GLU:CD	4:M:222:PHE:CE1	2.67	0.67
4:M:405:THR:O	4:M:407:THR:N	2.27	0.67
1:A:605:GLU:HG3	1:A:632:PHE:CD2	2.28	0.67
2:B:216:LYS:HZ3	4:M:136:VAL:HG21	1.59	0.67
2:B:596:LEU:CD1	2:B:611:ALA:HB1	2.24	0.67
1:A:411:GLU:CD	3:S:46:PHE:CZ	2.68	0.67
1:A:424:TYR:HD1	3:S:63:ASN:HD21	1.36	0.67
2:B:340:ILE:CG1	2:B:373:LEU:HG	2.25	0.67
4:M:331:LEU:HD12	4:M:331:LEU:O	1.93	0.67
4:M:421:GLY:O	4:M:422:PRO:C	2.22	0.67
1:A:628:VAL:O	1:A:631:SER:N	2.28	0.67
2:B:116:THR:O	2:B:120:ILE:HG12	1.93	0.67

Continued on next page...

Continued from previous page...

Atom-1	Atom-2	Interatomic distance (Å)	Clash overlap (Å)
2:B:184:GLY:O	2:B:188:TYR:CD2	2.39	0.67
2:B:200:MET:CE	2:B:229:HIS:C	2.56	0.67
2:B:311:TYR:O	2:B:312:SER:C	2.28	0.67
2:B:348:THR:HB	4:M:305:ASP:OD2	1.93	0.67
2:B:497:LEU:HD22	2:B:511:ILE:CG2	2.24	0.67
2:B:599:ALA:C	2:B:601:TYR:H	1.98	0.67
3:S:50:PHE:HA	3:S:77:TYR:HD1	1.60	0.67
4:M:126:ASN:O	4:M:129:VAL:N	2.22	0.67
4:M:374:TYR:HB3	4:M:417:TYR:HD1	1.58	0.67
1:A:88:ASN:HB3	1:A:120:ILE:CG2	2.25	0.67
1:A:207:LEU:O	1:A:243:ILE:HD11	1.93	0.67
1:A:533:ILE:CG1	1:A:562:TRP:CH2	2.78	0.67
2:B:106:LEU:HB3	4:M:130:GLU:CG	2.25	0.67
2:B:127:LEU:HD13	2:B:157:THR:CG2	2.17	0.67
2:B:193:LEU:HD21	2:B:225:LEU:CB	2.10	0.67
2:B:266:VAL:HA	2:B:289:PRO:HB2	1.75	0.67
2:B:341:GLU:HA	2:B:377:TYR:CD1	2.28	0.67
2:B:556:LEU:HB3	2:B:588:ILE:CD1	2.24	0.67
2:B:559:ASP:O	2:B:562:ASN:C	2.32	0.67
3:S:4:ALA:CB	3:S:19:PHE:HD1	2.05	0.67
1:A:279:LEU:O	1:A:280:GLU:C	2.29	0.67
1:A:397:ASP:O	1:A:418:ILE:CG1	2.43	0.67
1:A:581:LEU:CG	1:A:607:LEU:HD11	2.25	0.67
2:B:17:VAL:HG23	2:B:36:THR:N	2.10	0.67
2:B:193:LEU:C	2:B:195:ILE:H	1.98	0.67
2:B:313:SER:OG	4:M:269:ILE:C	2.33	0.67
4:M:48:ASP:HA	4:M:70:ASN:ND2	2.09	0.67
4:M:121:ILE:CG2	4:M:125:PHE:CE1	2.78	0.67
4:M:472:TYR:CD1	4:M:472:TYR:N	2.57	0.67
1:A:179:LYS:HE3	3:S:140:MET:CG	2.24	0.67
1:A:261:THR:HG1	1:A:297:CYS:HG	1.21	0.67
1:A:503:ASN:OD1	4:M:60:LEU:CB	2.42	0.67
1:A:529:GLY:HA3	1:A:562:TRP:CZ2	2.30	0.67
1:A:638:LEU:CD2	2:B:561:ASP:N	2.38	0.67
2:B:10:SER:C	2:B:40:GLN:HE22	1.98	0.67
2:B:106:LEU:CD2	4:M:131:ALA:N	2.57	0.67
2:B:226:LEU:CD2	2:B:255:TYR:CE1	2.61	0.67
2:B:513:TRP:CA	2:B:551:LEU:CD1	2.72	0.67
4:M:47:SER:HB2	4:M:50:TYR:CE1	2.29	0.67
4:M:219:LEU:HG	4:M:440:ILE:HG12	1.76	0.67
2:B:344:VAL:CG2	2:B:377:TYR:HB3	2.24	0.67

Continued on next page...

Continued from previous page...

Atom-1	Atom-2	Interatomic distance (Å)	Clash overlap (Å)
2:B:588:ILE:CG2	2:B:618:PHE:CE1	2.78	0.67
3:S:157:ASN:O	3:S:161:GLU:HG3	1.94	0.67
4:M:258:VAL:HG22	4:M:452:ILE:HG23	1.76	0.67
4:M:321:GLY:O	4:M:323:MET:N	2.29	0.67
1:A:99:LYS:HG2	1:A:101:GLN:N	2.09	0.66
1:A:395:PHE:CE2	1:A:428:MET:HE3	2.29	0.66
1:A:460:LEU:HA	4:M:58:ARG:HH21	1.60	0.66
2:B:247:TYR:CE1	4:M:136:VAL:HG12	2.30	0.66
2:B:408:VAL:CG1	2:B:412:PHE:CE2	2.78	0.66
2:B:588:ILE:CG2	2:B:618:PHE:CZ	2.77	0.66
4:M:69:ILE:CD1	4:M:94:GLU:HA	2.24	0.66
4:M:244:VAL:N	4:M:300:LEU:O	2.22	0.66
4:M:351:SER:CB	4:M:440:ILE:O	2.42	0.66
1:A:244:LEU:CD1	1:A:281:LEU:CD1	2.73	0.66
2:B:34:SER:CB	2:B:65:ARG:CZ	2.71	0.66
2:B:67:ILE:O	4:M:18:TYR:CE1	2.46	0.66
2:B:106:LEU:CD2	2:B:144:ASP:HB3	2.16	0.66
2:B:314:ASN:ND2	4:M:271:SER:OG	2.27	0.66
2:B:340:ILE:CG1	2:B:373:LEU:CG	2.74	0.66
4:M:260:LEU:HD21	4:M:449:VAL:HG22	1.75	0.66
4:M:309:GLN:O	4:M:313:SER:N	2.26	0.66
1:A:570:LYS:CD	1:A:618:THR:HG22	2.25	0.66
2:B:310:ILE:CG2	2:B:318:ILE:HG23	2.23	0.66
2:B:344:VAL:HA	2:B:381:PHE:CZ	2.31	0.66
2:B:375:LEU:CD2	2:B:404:ASN:HB2	2.25	0.66
2:B:563:PHE:HD1	2:B:584:SER:CA	2.05	0.66
4:M:376:ILE:CG2	4:M:379:LEU:HD11	2.26	0.66
4:M:376:ILE:HG21	4:M:379:LEU:CD1	2.25	0.66
1:A:147:LEU:HD22	1:A:166:LEU:CD2	2.26	0.66
1:A:233:PHE:C	1:A:235:GLN:N	2.46	0.66
1:A:638:LEU:HD23	2:B:561:ASP:OD1	1.96	0.66
3:S:16:LEU:CD1	3:S:129:GLU:HG2	2.25	0.66
3:S:55:PRO:O	3:S:58:LEU:HB3	1.94	0.66
4:M:51:LEU:HB2	4:M:68:VAL:HB	1.76	0.66
4:M:133:GLU:O	4:M:134:PRO:C	2.25	0.66
4:M:212:ASN:O	4:M:465:LYS:HB2	1.95	0.66
4:M:437:TYR:HB3	4:M:439:TYR:CE1	2.30	0.66
1:A:215:VAL:CG1	1:A:243:ILE:HG23	2.09	0.66
1:A:342:GLY:O	1:A:343:LYS:C	2.31	0.66
1:A:516:ILE:HG22	1:A:554:ALA:HB2	1.76	0.66
2:B:63:MET:HG2	2:B:100:LEU:CB	2.24	0.66

Continued on next page...

Continued from previous page...

Atom-1	Atom-2	Interatomic distance (Å)	Clash overlap (Å)
2:B:144:ASP:N	2:B:179:LYS:HD3	2.08	0.66
2:B:219:TYR:OH	2:B:226:LEU:CA	2.33	0.66
2:B:230:PHE:CZ	2:B:234:CYS:SG	2.87	0.66
2:B:392:SER:CB	2:B:424:PHE:HE1	2.08	0.66
2:B:397:GLN:CG	2:B:431:MET:SD	2.84	0.66
2:B:594:ALA:O	2:B:598:LEU:HG	1.94	0.66
3:S:8:PHE:CD2	3:S:36:TYR:CE1	2.84	0.66
3:S:135:ILE:O	3:S:141:VAL:HG22	1.95	0.66
4:M:213:GLU:O	4:M:248:SER:HA	1.95	0.66
4:M:243:ILE:HG13	4:M:473:LYS:O	1.95	0.66
4:M:247:ARG:HG3	4:M:470:ALA:HA	1.77	0.66
4:M:437:TYR:HD1	4:M:437:TYR:N	1.91	0.66
1:A:107:TYR:CE1	1:A:128:LEU:HD21	2.31	0.66
1:A:397:ASP:O	1:A:418:ILE:CD1	2.44	0.66
1:A:503:ASN:OD1	4:M:59:ASP:O	2.13	0.66
1:A:563:CYS:HA	1:A:566:PHE:HD2	1.61	0.66
2:B:17:VAL:CG1	2:B:35:TYR:CE2	2.75	0.66
2:B:278:PRO:HB3	2:B:288:TYR:C	2.14	0.66
2:B:313:SER:N	4:M:269:ILE:CD1	2.56	0.66
2:B:390:VAL:O	2:B:393:ILE:HB	1.95	0.66
2:B:396:ILE:HD11	2:B:418:TYR:OH	1.96	0.66
2:B:508:ARG:O	2:B:512:VAL:HG23	1.95	0.66
2:B:559:ASP:O	2:B:562:ASN:CA	2.44	0.66
2:B:589:SER:HA	2:B:592:TYR:CD2	2.31	0.66
4:M:224:VAL:HG22	4:M:306:LEU:CD1	2.25	0.66
4:M:257:ALA:HB3	4:M:453:ASP:OD1	1.96	0.66
4:M:379:LEU:CD2	4:M:411:LEU:HG	2.20	0.66
1:A:96:SER:N	1:A:127:LEU:HD21	2.11	0.66
1:A:225:LEU:HB2	1:A:233:PHE:CE2	2.28	0.66
1:A:273:LYS:O	1:A:276:PRO:HD2	1.96	0.66
2:B:151:ALA:HA	2:B:180:LEU:CD1	2.26	0.66
2:B:155:LEU:HG	2:B:188:TYR:HD1	1.59	0.66
2:B:278:PRO:CB	2:B:288:TYR:C	2.63	0.66
2:B:344:VAL:CG1	2:B:381:PHE:HE2	2.06	0.66
2:B:458:MET:HA	2:B:463:LEU:HD12	1.78	0.66
2:B:559:ASP:HB2	2:B:563:PHE:CE2	2.31	0.66
3:S:118:GLU:O	3:S:122:ILE:HG13	1.95	0.66
4:M:5:PHE:HE2	4:M:20:LEU:CD1	2.09	0.66
4:M:217:ASP:CG	4:M:471:LYS:HA	2.16	0.66
4:M:224:VAL:HG23	4:M:479:PHE:CD2	2.30	0.66
4:M:347:PHE:HE1	4:M:439:TYR:CG	2.12	0.66

Continued on next page...

Continued from previous page...

Atom-1	Atom-2	Interatomic distance (Å)	Clash overlap (Å)
1:A:481:MET:SD	1:A:518:CYS:SG	2.94	0.66
2:B:13:ASP:O	2:B:17:VAL:CG1	2.43	0.66
2:B:234:CYS:SG	2:B:298:ASP:O	2.49	0.66
2:B:243:TRP:CZ3	4:M:98:ARG:NE	2.63	0.66
2:B:403:ILE:HD11	2:B:439:CYS:HA	1.49	0.66
2:B:559:ASP:HB3	2:B:563:PHE:CD2	2.31	0.66
3:S:38:LEU:HB3	3:S:51:LEU:CD1	2.26	0.66
4:M:220:GLU:O	4:M:439:TYR:N	2.19	0.66
4:M:222:PHE:HB2	4:M:479:PHE:HZ	1.60	0.66
2:B:24:ALA:N	2:B:32:GLU:OE1	2.06	0.66
2:B:180:LEU:HD23	2:B:192:LEU:HD21	1.78	0.66
2:B:197:LYS:CA	2:B:229:HIS:CD2	2.77	0.66
2:B:549:LEU:CD1	2:B:595:VAL:CG1	2.74	0.66
3:S:16:LEU:HA	3:S:125:TRP:CZ2	2.31	0.66
4:M:120:ARG:O	4:M:124:ILE:CG1	2.44	0.66
2:B:142:LEU:O	2:B:143:SER:C	2.28	0.66
2:B:205:PRO:O	2:B:206:LYS:C	2.28	0.66
4:M:16:PHE:HA	4:M:118:TYR:CE2	2.31	0.66
4:M:223:HIS:CE1	4:M:476:THR:H	2.14	0.66
4:M:436:GLU:HA	4:M:479:PHE:HE1	1.57	0.66
1:A:390:THR:O	1:A:394:GLN:HG2	1.96	0.65
2:B:120:ILE:HD12	2:B:142:LEU:CD2	2.26	0.65
2:B:136:CYS:SG	2:B:168:MET:C	2.75	0.65
2:B:139:LEU:HD23	2:B:173:VAL:CA	2.24	0.65
2:B:227:HIS:O	2:B:229:HIS:N	2.29	0.65
2:B:243:TRP:HH2	4:M:98:ARG:CZ	2.08	0.65
2:B:360:LEU:HB3	2:B:394:TRP:CB	2.25	0.65
2:B:398:ILE:CG2	2:B:402:LEU:CD1	2.69	0.65
2:B:408:VAL:HG11	2:B:446:TRP:CB	2.25	0.65
2:B:433:VAL:HG22	2:B:471:TYR:CE2	2.31	0.65
2:B:456:ASP:O	2:B:460:SER:HB2	1.96	0.65
4:M:56:VAL:N	4:M:64:LYS:HG3	2.09	0.65
4:M:253:ASN:HA	4:M:292:PRO:CG	2.26	0.65
4:M:320:ILE:O	4:M:322:LEU:N	2.29	0.65
4:M:350:VAL:HG13	4:M:442:GLN:HG2	1.78	0.65
1:A:196:LEU:HD22	1:A:196:LEU:C	2.16	0.65
1:A:200:PHE:CE1	1:A:236:LEU:HD21	2.30	0.65
2:B:378:THR:CG2	2:B:379:LYS:N	2.59	0.65
2:B:549:LEU:HD12	2:B:595:VAL:HG11	1.78	0.65
3:S:89:VAL:CG1	3:S:98:ILE:HG21	2.26	0.65
4:M:247:ARG:N	4:M:470:ALA:HB2	2.10	0.65

Continued on next page...

Continued from previous page...

Atom-1	Atom-2	Interatomic distance (Å)	Clash overlap (Å)
4:M:304:VAL:CG1	4:M:445:SER:HA	2.25	0.65
4:M:351:SER:OG	4:M:441:GLY:O	2.10	0.65
2:B:567:GLN:CA	2:B:569:THR:OG1	2.43	0.65
3:S:8:PHE:HB2	3:S:36:TYR:HE1	1.61	0.65
3:S:164:ASP:HA	3:S:167:ILE:HB	1.79	0.65
4:M:120:ARG:O	4:M:124:ILE:HG13	1.96	0.65
4:M:250:LEU:HD13	4:M:254:PRO:HG2	1.79	0.65
1:A:320:HIS:HB2	1:A:352:PHE:CE1	2.31	0.65
1:A:365:VAL:O	1:A:366:SER:C	2.23	0.65
1:A:408:ILE:N	3:S:64:ASN:HD22	1.92	0.65
1:A:503:ASN:CG	4:M:59:ASP:C	2.51	0.65
1:A:585:PHE:HA	1:A:600:SER:OG	1.95	0.65
2:B:9:ALA:C	4:M:14:LEU:HB2	2.16	0.65
2:B:251:LEU:C	2:B:253:ILE:H	1.99	0.65
2:B:307:ASN:ND2	2:B:338:LYS:HB3	2.11	0.65
2:B:403:ILE:HB	2:B:408:VAL:HG22	1.79	0.65
2:B:465:ALA:HB1	2:B:504:ALA:HB2	1.77	0.65
2:B:509:ALA:HB1	2:B:547:GLN:HG3	1.78	0.65
2:B:522:GLU:O	2:B:522:GLU:CG	2.33	0.65
2:B:534:ILE:C	2:B:536:ASN:N	2.48	0.65
2:B:564:LYS:HE3	2:B:621:GLY:O	1.96	0.65
4:M:5:PHE:HA	4:M:77:LEU:O	1.97	0.65
4:M:46:SER:O	4:M:47:SER:C	2.19	0.65
4:M:219:LEU:HA	4:M:440:ILE:HA	1.77	0.65
4:M:220:GLU:CD	4:M:222:PHE:CZ	2.70	0.65
4:M:317:MET:CB	4:M:322:LEU:H	2.08	0.65
1:A:68:THR:N	3:S:166:LYS:HD3	2.11	0.65
1:A:582:ILE:HG23	1:A:604:LEU:CG	2.19	0.65
2:B:252:LEU:HD12	2:B:302:PHE:CD1	2.30	0.65
2:B:373:LEU:C	2:B:375:LEU:N	2.50	0.65
4:M:71:LYS:CB	4:M:74:TYR:CZ	2.70	0.65
4:M:220:GLU:OE2	4:M:350:VAL:CG1	2.44	0.65
4:M:278:ILE:HG23	4:M:278:ILE:O	1.95	0.65
4:M:353:VAL:HA	4:M:439:TYR:HA	1.76	0.65
1:A:170:LEU:HD22	1:A:181:ALA:HB1	1.77	0.65
1:A:219:VAL:HG11	1:A:256:LEU:HD23	1.79	0.65
1:A:257:LEU:HD22	1:A:278:ILE:HG23	1.78	0.65
1:A:516:ILE:CG2	1:A:554:ALA:CB	2.74	0.65
1:A:609:LEU:CD2	1:A:628:VAL:HG11	2.27	0.65
2:B:28:SER:HB3	2:B:61:ASP:OD2	1.96	0.65
2:B:37:TYR:CD2	2:B:38:TYR:HD1	2.13	0.65

Continued on next page...

Continued from previous page...

Atom-1	Atom-2	Interatomic distance (Å)	Clash overlap (Å)
3:S:39:ILE:HD11	3:S:77:TYR:CD2	2.32	0.65
4:M:69:ILE:CD1	4:M:94:GLU:CA	2.75	0.65
4:M:432:THR:HG23	4:M:432:THR:O	1.97	0.65
1:A:304:LEU:HD23	1:A:306:GLU:H	1.61	0.65
1:A:438:ALA:O	1:A:440:ASN:N	2.30	0.65
2:B:22:ALA:HB2	2:B:33:SER:HG	1.59	0.65
2:B:72:SER:O	2:B:73:ASP:CB	2.43	0.65
2:B:73:ASP:N	4:M:19:LEU:HB2	2.04	0.65
2:B:223:LEU:CD1	2:B:258:GLN:HB2	2.17	0.65
2:B:540:GLU:OE1	2:B:548:ILE:HD11	1.96	0.65
3:S:32:LEU:C	3:S:35:VAL:HG22	2.14	0.65
4:M:121:ILE:HG22	4:M:125:PHE:CE1	2.31	0.65
4:M:222:PHE:CB	4:M:479:PHE:CZ	2.80	0.65
4:M:353:VAL:O	4:M:401:LYS:CA	2.42	0.65
1:A:83:ASP:CG	1:A:85:ALA:H	1.99	0.65
2:B:211:ALA:O	2:B:214:ALA:HB3	1.96	0.65
2:B:456:ASP:O	2:B:460:SER:CB	2.45	0.65
2:B:513:TRP:CA	2:B:551:LEU:HD13	2.27	0.65
2:B:513:TRP:HE1	2:B:517:GLU:HG3	1.62	0.65
2:B:574:ASN:C	2:B:576:GLN:H	1.98	0.65
4:M:306:LEU:CD2	4:M:317:MET:HE3	2.13	0.65
1:A:137:ASN:HA	1:A:139:ASP:HB3	1.79	0.65
1:A:151:SER:O	1:A:152:THR:C	2.28	0.65
1:A:240:LEU:O	1:A:242:GLU:O	2.15	0.65
1:A:638:LEU:HD12	2:B:558:TYR:N	2.09	0.65
2:B:87:VAL:O	2:B:88:LYS:C	2.33	0.65
3:S:39:ILE:HG23	3:S:47:GLN:OE1	1.97	0.65
4:M:225:VAL:HG22	4:M:480:GLN:HB3	1.78	0.65
4:M:306:LEU:CD2	4:M:317:MET:HE1	2.23	0.65
4:M:433:VAL:HG13	4:M:433:VAL:O	1.97	0.65
1:A:179:LYS:HZ3	3:S:141:VAL:CA	2.05	0.65
1:A:260:PHE:CD1	1:A:274:LEU:HG	2.31	0.65
1:A:462:GLN:HG2	4:M:59:ASP:OD1	1.97	0.65
1:A:496:ILE:HD11	1:A:532:LEU:HG	1.79	0.65
2:B:106:LEU:HD13	2:B:144:ASP:O	1.91	0.65
2:B:120:ILE:HG21	2:B:150:LEU:HD22	1.79	0.65
2:B:486:HIS:CE1	2:B:518:ILE:CG1	2.79	0.65
4:M:15:ILE:HG22	4:M:114:ILE:HG22	1.79	0.65
4:M:100:LEU:O	4:M:103:TYR:O	2.14	0.65
4:M:443:SER:OG	4:M:448:TYR:CA	2.45	0.65
1:A:189:PHE:HD2	1:A:225:LEU:HD11	1.60	0.64

Continued on next page...

Continued from previous page...

Atom-1	Atom-2	Interatomic distance (Å)	Clash overlap (Å)
1:A:588:LEU:O	1:A:590:TYR:N	2.29	0.64
2:B:61:ASP:O	2:B:64:LYS:HB2	1.97	0.64
2:B:227:HIS:CE1	2:B:292:GLU:CG	2.80	0.64
2:B:290:SER:C	2:B:292:GLU:N	2.41	0.64
2:B:397:GLN:NE2	2:B:431:MET:HE1	2.11	0.64
2:B:566:ALA:CA	2:B:574:ASN:CG	2.64	0.64
3:S:75:ILE:HG21	3:S:77:TYR:CZ	2.32	0.64
4:M:443:SER:HB3	4:M:447:ILE:CA	2.27	0.64
1:A:464:ILE:HG23	1:A:465:SER:HA	1.79	0.64
1:A:496:ILE:CD1	1:A:532:LEU:HG	2.27	0.64
2:B:253:ILE:HG23	2:B:327:GLN:HB2	1.78	0.64
2:B:253:ILE:CD1	2:B:324:ALA:HA	2.28	0.64
2:B:375:LEU:HB2	2:B:376:PRO:HD3	1.79	0.64
2:B:537:PHE:C	2:B:537:PHE:CD1	2.71	0.64
3:S:89:VAL:HG11	3:S:98:ILE:CD1	2.28	0.64
1:A:143:VAL:O	1:A:147:LEU:HG	1.96	0.64
1:A:529:GLY:CA	1:A:562:TRP:CZ2	2.80	0.64
2:B:35:TYR:O	2:B:42:ILE:HD11	1.97	0.64
2:B:106:LEU:CD1	2:B:144:ASP:HB2	1.97	0.64
2:B:216:LYS:HZ1	4:M:136:VAL:CG2	2.09	0.64
2:B:231:ARG:CZ	2:B:297:PRO:HG2	2.26	0.64
2:B:278:PRO:HD3	2:B:290:SER:HA	1.79	0.64
2:B:472:VAL:CG1	2:B:510:GLY:C	2.66	0.64
4:M:7:ILE:HD12	4:M:121:ILE:HG21	1.79	0.64
4:M:7:ILE:HD11	4:M:121:ILE:HG21	1.78	0.64
4:M:230:LYS:O	4:M:232:HIS:N	2.29	0.64
4:M:363:ASN:HB2	4:M:431:GLN:HB2	1.80	0.64
1:A:244:LEU:HD11	1:A:281:LEU:HD13	1.79	0.64
2:B:249:ILE:HD13	2:B:321:CYS:SG	2.36	0.64
4:M:65:TYR:CE2	4:M:86:PRO:CA	2.80	0.64
4:M:118:TYR:HA	4:M:121:ILE:HD12	1.78	0.64
1:A:528:ASN:O	1:A:529:GLY:O	2.15	0.64
1:A:588:LEU:O	1:A:589:SER:C	2.26	0.64
2:B:143:SER:HB2	2:B:179:LYS:CG	2.27	0.64
3:S:6:LEU:HA	3:S:16:LEU:O	1.98	0.64
4:M:5:PHE:CZ	4:M:92:PHE:CD1	2.84	0.64
4:M:269:ILE:C	4:M:302:TYR:CD2	2.71	0.64
1:A:581:LEU:CG	1:A:585:PHE:CE2	2.80	0.64
2:B:73:ASP:HB3	4:M:25:PRO:C	2.17	0.64
2:B:219:TYR:CB	2:B:226:LEU:HD22	2.28	0.64
2:B:418:TYR:CD1	2:B:419:VAL:N	2.65	0.64

Continued on next page...

Continued from previous page...

Atom-1	Atom-2	Interatomic distance (Å)	Clash overlap (Å)
2:B:457:HIS:ND1	2:B:461:HIS:CD2	2.65	0.64
1:A:438:ALA:C	1:A:439:ASP:O	2.17	0.64
2:B:212:VAL:HG11	2:B:248:LEU:HD23	1.79	0.64
2:B:299:LEU:O	2:B:302:PHE:CB	2.42	0.64
2:B:340:ILE:CG1	2:B:373:LEU:CD2	2.70	0.64
2:B:429:VAL:CG1	2:B:467:VAL:HG11	2.27	0.64
2:B:430:ILE:CD1	2:B:466:SER:HB3	2.27	0.64
2:B:542:PRO:O	2:B:607:ILE:CD1	2.41	0.64
2:B:553:ALA:HB2	2:B:614:ILE:CD1	2.28	0.64
1:A:215:VAL:HG12	1:A:243:ILE:HG21	1.78	0.64
1:A:272:ALA:O	1:A:276:PRO:HD3	1.97	0.64
4:M:9:ASP:HB3	4:M:111:ILE:CG2	2.28	0.64
4:M:219:LEU:CD1	4:M:440:ILE:HG12	2.27	0.64
4:M:222:PHE:CE2	4:M:439:TYR:CE2	2.85	0.64
4:M:360:LEU:HD13	4:M:433:VAL:CG2	2.27	0.64
4:M:458:LEU:HD13	4:M:464:THR:HG21	1.80	0.64
1:A:260:PHE:O	1:A:262:ASN:N	2.29	0.64
1:A:323:CYS:SG	1:A:355:LEU:HD21	2.38	0.64
1:A:599:ARG:NE	2:B:610:ARG:NH2	2.39	0.64
2:B:43:ASN:HB3	2:B:44:PRO:HD2	1.79	0.64
2:B:73:ASP:HA	4:M:19:LEU:HD23	1.60	0.64
2:B:98:LYS:NZ	2:B:134:LEU:HD22	2.12	0.64
2:B:181:TYR:CB	2:B:218:CYS:SG	2.85	0.64
2:B:223:LEU:HD21	2:B:258:GLN:HB2	1.80	0.64
2:B:237:ILE:HG23	2:B:238:LYS:H	1.50	0.64
2:B:251:LEU:O	2:B:254:LYS:N	2.30	0.64
2:B:433:VAL:HG22	2:B:471:TYR:CZ	2.32	0.64
2:B:457:HIS:O	2:B:458:MET:C	2.30	0.64
2:B:570:GLY:C	2:B:571:SER:O	2.19	0.64
4:M:252:ASP:O	4:M:254:PRO:HD2	1.97	0.64
1:A:125:THR:OG1	1:A:158:LEU:CD1	2.37	0.64
1:A:403:LEU:O	1:A:404:GLN:C	2.27	0.64
2:B:35:TYR:O	2:B:42:ILE:CD1	2.46	0.64
2:B:37:TYR:O	2:B:40:GLN:O	2.15	0.64
2:B:80:GLN:HG2	2:B:108:PHE:HZ	1.58	0.64
2:B:351:GLU:OE1	4:M:241:HIS:HB2	1.98	0.64
2:B:452:LYS:NZ	2:B:456:ASP:OD2	2.30	0.64
2:B:545:ARG:CD	2:B:602:ASP:OD2	2.46	0.64
3:S:80:TYR:OH	3:S:110:ASP:CG	2.36	0.64
4:M:44:ASP:C	4:M:47:SER:H	2.02	0.64
1:A:117:ASP:CG	1:A:120:ILE:HG12	2.19	0.63

Continued on next page...

Continued from previous page...

Atom-1	Atom-2	Interatomic distance (Å)	Clash overlap (Å)
1:A:150:LEU:HB3	1:A:162:ILE:CD1	2.27	0.63
1:A:609:LEU:HD13	1:A:609:LEU:C	2.18	0.63
2:B:212:VAL:HG22	2:B:233:TYR:CD1	2.32	0.63
2:B:249:ILE:HD11	2:B:321:CYS:SG	2.38	0.63
2:B:398:ILE:C	2:B:400:SER:N	2.50	0.63
4:M:63:TYR:HB3	4:M:81:SER:O	1.97	0.63
4:M:221:THR:HG23	4:M:437:TYR:O	1.98	0.63
1:A:384:LEU:HD22	1:A:441:TYR:CE2	2.33	0.63
1:A:436:CYS:HB3	1:A:476:GLN:HE21	1.63	0.63
2:B:35:TYR:HE1	4:M:118:TYR:HH	1.44	0.63
2:B:178:ILE:CD1	2:B:218:CYS:H	2.07	0.63
2:B:279:LEU:H	2:B:288:TYR:CB	2.02	0.63
2:B:313:SER:HB3	4:M:269:ILE:O	1.96	0.63
2:B:386:LYS:CE	4:M:480:GLN:HB2	2.27	0.63
2:B:433:VAL:O	2:B:474:VAL:HG21	1.97	0.63
2:B:517:GLU:O	2:B:519:ALA:N	2.31	0.63
4:M:68:VAL:CA	4:M:76:CYS:O	2.44	0.63
4:M:364:VAL:C	4:M:367:ALA:O	2.36	0.63
1:A:68:THR:OG1	3:S:166:LYS:HD3	1.99	0.63
1:A:132:LEU:HD11	1:A:150:LEU:CD1	2.27	0.63
1:A:462:GLN:OE1	4:M:58:ARG:HB2	1.94	0.63
2:B:104:TYR:O	2:B:107:ARG:CA	2.45	0.63
2:B:371:GLN:C	2:B:373:LEU:H	2.01	0.63
2:B:585:GLY:O	2:B:589:SER:OG	2.17	0.63
3:S:65:ASN:O	3:S:67:GLU:N	2.32	0.63
4:M:44:ASP:OD2	4:M:50:TYR:HE2	1.80	0.63
4:M:254:PRO:HB3	4:M:454:ILE:HD11	1.79	0.63
1:A:250:ASN:OD1	1:A:285:THR:HB	1.98	0.63
1:A:289:SER:O	1:A:290:VAL:O	2.17	0.63
1:A:492:ILE:HD11	1:A:522:PHE:CB	2.29	0.63
2:B:13:ASP:O	2:B:17:VAL:CB	2.46	0.63
2:B:249:ILE:HG12	2:B:306:LEU:HD23	1.80	0.63
2:B:314:ASN:O	2:B:318:ILE:HG13	1.98	0.63
2:B:378:THR:CG2	2:B:379:LYS:H	2.11	0.63
2:B:436:LEU:HD12	2:B:454:LEU:HD23	1.79	0.63
2:B:530:LEU:CD2	2:B:591:MET:HB3	2.28	0.63
3:S:149:ILE:O	3:S:153:VAL:HG23	1.99	0.63
4:M:51:LEU:HB2	4:M:68:VAL:HG11	1.81	0.63
4:M:131:ALA:C	4:M:133:GLU:N	2.39	0.63
4:M:443:SER:CA	4:M:447:ILE:HG13	2.28	0.63
1:A:140:VAL:HG22	1:A:177:ILE:HG13	1.79	0.63

Continued on next page...

Continued from previous page...

Atom-1	Atom-2	Interatomic distance (Å)	Clash overlap (Å)
1:A:433:ILE:HD11	1:A:473:ILE:HA	1.80	0.63
1:A:435:ILE:HG23	1:A:441:TYR:CE2	2.33	0.63
1:A:495:ILE:HG21	1:A:515:CYS:CB	2.27	0.63
1:A:503:ASN:CG	4:M:59:ASP:O	2.37	0.63
1:A:506:LYS:HZ1	4:M:82:LYS:HD2	0.58	0.63
2:B:73:ASP:N	4:M:19:LEU:HB3	2.03	0.63
2:B:157:THR:O	2:B:161:LEU:HD13	1.99	0.63
2:B:186:ASN:C	2:B:188:TYR:H	2.00	0.63
2:B:497:LEU:HD22	2:B:511:ILE:HG21	1.79	0.63
2:B:589:SER:HG	2:B:618:PHE:HE2	1.44	0.63
3:S:127:THR:HG23	3:S:153:VAL:HG13	1.80	0.63
4:M:55:MET:O	4:M:56:VAL:C	2.24	0.63
4:M:374:TYR:CB	4:M:417:TYR:HD1	2.12	0.63
4:M:376:ILE:HG22	4:M:379:LEU:HD12	1.79	0.63
1:A:420:ILE:HG22	1:A:421:PRO:O	1.99	0.63
1:A:480:LEU:C	1:A:480:LEU:HD13	2.19	0.63
2:B:136:CYS:SG	2:B:169:VAL:N	2.71	0.63
2:B:212:VAL:CG1	2:B:248:LEU:HD23	2.29	0.63
2:B:237:ILE:HG22	2:B:238:LYS:H	1.64	0.63
2:B:250:GLU:HA	2:B:253:ILE:HD12	1.81	0.63
2:B:270:SER:O	2:B:273:SER:HB2	1.98	0.63
3:S:57:LEU:O	3:S:67:GLU:O	2.16	0.63
4:M:5:PHE:O	4:M:17:GLN:HA	1.97	0.63
4:M:223:HIS:CG	4:M:478:ASN:HA	2.33	0.63
4:M:271:SER:HB3	4:M:301:GLU:HG2	1.80	0.63
4:M:407:THR:O	4:M:409:PRO:HD3	1.99	0.63
1:A:111:SER:HB2	1:A:152:THR:HG1	1.63	0.63
2:B:124:GLN:OE1	2:B:153:ILE:HG23	1.99	0.63
4:M:65:TYR:CE2	4:M:86:PRO:O	2.52	0.63
4:M:265:ASN:HB3	4:M:309:GLN:CG	2.26	0.63
1:A:450:TYR:OH	1:A:476:GLN:NE2	2.31	0.63
2:B:25:VAL:HG23	2:B:32:GLU:CA	2.29	0.63
2:B:252:LEU:HD13	2:B:302:PHE:CG	2.30	0.63
2:B:295:ASN:C	2:B:300:ASP:HB2	2.01	0.63
2:B:511:ILE:O	2:B:512:VAL:C	2.35	0.63
2:B:513:TRP:HA	2:B:551:LEU:HD13	1.81	0.63
3:S:126:GLN:NE2	3:S:127:THR:OG1	2.32	0.63
4:M:356:LEU:C	4:M:356:LEU:HD23	2.19	0.63
1:A:185:LEU:CD1	1:A:203:PHE:CE1	2.82	0.63
2:B:12:LEU:HD22	4:M:13:LYS:CD	2.28	0.63
2:B:120:ILE:CD1	2:B:142:LEU:HD23	2.29	0.63

Continued on next page...

Continued from previous page...

Atom-1	Atom-2	Interatomic distance (Å)	Clash overlap (Å)
2:B:306:LEU:HD12	2:B:325:LEU:CD2	2.28	0.63
2:B:322:CYS:SG	2:B:366:LEU:HG	2.39	0.63
3:S:87:PHE:CG	3:S:102:ILE:HG12	2.34	0.63
1:A:71:VAL:HG21	1:A:94:VAL:HG11	1.81	0.62
2:B:72:SER:HB3	4:M:17:GLN:CD	2.17	0.62
2:B:136:CYS:SG	2:B:168:MET:HG2	2.39	0.62
2:B:271:GLU:O	2:B:272:GLY:C	2.29	0.62
2:B:360:LEU:HD13	2:B:391:ALA:O	1.99	0.62
2:B:418:TYR:CD1	2:B:419:VAL:HA	2.33	0.62
2:B:560:ILE:CA	2:B:563:PHE:HB2	2.27	0.62
2:B:597:TYR:O	2:B:601:TYR:CE2	2.51	0.62
4:M:220:GLU:OE1	4:M:222:PHE:CZ	2.51	0.62
1:A:211:ASP:OD1	1:A:213:SER:N	2.29	0.62
1:A:224:GLU:O	1:A:225:LEU:C	2.30	0.62
1:A:364:ASP:HB3	1:A:367:ILE:HD12	1.80	0.62
3:S:107:GLU:O	3:S:111:ARG:HG2	1.99	0.62
3:S:127:THR:HG22	3:S:153:VAL:HG13	1.77	0.62
4:M:214:LEU:N	4:M:465:LYS:O	2.31	0.62
4:M:350:VAL:CG1	4:M:442:GLN:HG2	2.30	0.62
1:A:150:LEU:O	1:A:153:ILE:N	2.32	0.62
1:A:260:PHE:CZ	1:A:274:LEU:HD11	2.34	0.62
2:B:243:TRP:HH2	4:M:98:ARG:NE	1.95	0.62
2:B:315:PRO:HG3	2:B:350:THR:HG22	1.82	0.62
2:B:566:ALA:CB	2:B:581:TYR:HB3	2.30	0.62
3:S:70:ASN:O	3:S:71:GLU:C	2.35	0.62
4:M:265:ASN:O	4:M:267:ILE:N	2.32	0.62
4:M:276:VAL:CG2	4:M:299:LEU:HD12	2.29	0.62
4:M:376:ILE:HG22	4:M:379:LEU:CD1	2.27	0.62
2:B:127:LEU:HB3	2:B:157:THR:CG2	2.30	0.62
2:B:328:LEU:HB2	2:B:333:GLN:NE2	2.14	0.62
2:B:437:SER:HB2	2:B:474:VAL:CG2	2.29	0.62
2:B:574:ASN:C	2:B:576:GLN:N	2.52	0.62
2:B:596:LEU:HD13	2:B:611:ALA:C	2.20	0.62
3:S:48:SER:HG	3:S:50:PHE:H	1.44	0.62
4:M:269:ILE:O	4:M:302:TYR:CD2	2.51	0.62
4:M:319:SER:CB	4:M:343:ASN:O	2.47	0.62
4:M:445:SER:HG	4:M:447:ILE:HG23	1.61	0.62
1:A:121:LEU:HD11	1:A:155:THR:H	1.63	0.62
1:A:289:SER:O	1:A:291:ILE:N	2.31	0.62
1:A:319:LEU:O	1:A:320:HIS:O	2.17	0.62
2:B:10:SER:N	4:M:14:LEU:HD13	2.13	0.62

Continued on next page...

Continued from previous page...

Atom-1	Atom-2	Interatomic distance (Å)	Clash overlap (Å)
2:B:68:SER:HA	4:M:18:TYR:CD1	2.34	0.62
2:B:151:ALA:CB	2:B:180:LEU:HD11	2.29	0.62
2:B:566:ALA:HA	2:B:574:ASN:CB	2.25	0.62
4:M:224:VAL:HG23	4:M:479:PHE:CE2	2.34	0.62
4:M:290:PHE:HZ	4:M:293:PRO:HD3	1.61	0.62
1:A:421:PRO:HA	3:S:62:GLU:O	2.00	0.62
1:A:429:VAL:CG1	1:A:469:LEU:HD11	2.29	0.62
1:A:536:MET:HB3	1:A:551:LEU:HD11	1.80	0.62
2:B:200:MET:HE2	2:B:229:HIS:HA	1.81	0.62
2:B:291:TYR:CE2	2:B:294:VAL:HB	2.34	0.62
4:M:15:ILE:HG21	4:M:114:ILE:HG22	1.79	0.62
4:M:290:PHE:CE1	4:M:297:PHE:CD2	2.88	0.62
4:M:300:LEU:O	4:M:300:LEU:HD12	2.00	0.62
1:A:402:ILE:HB	1:A:421:PRO:HA	1.81	0.62
1:A:517:TRP:NE1	2:B:605:PHE:CE2	2.65	0.62
2:B:310:ILE:HG21	2:B:342:ALA:CB	2.28	0.62
4:M:65:TYR:CE2	4:M:86:PRO:HB3	2.33	0.62
1:A:297:CYS:O	1:A:298:ILE:C	2.38	0.62
2:B:158:VAL:CG1	2:B:177:ILE:CD1	2.77	0.62
2:B:403:ILE:HD11	2:B:439:CYS:CA	2.01	0.62
2:B:416:LYS:HA	2:B:457:HIS:HE2	1.63	0.62
2:B:568:VAL:O	2:B:574:ASN:ND2	2.32	0.62
2:B:596:LEU:HD13	2:B:611:ALA:O	1.99	0.62
3:S:16:LEU:CB	3:S:125:TRP:NE1	2.50	0.62
4:M:19:LEU:CD2	4:M:24:ALA:CB	2.77	0.62
4:M:101:LEU:O	4:M:103:TYR:O	2.18	0.62
4:M:217:ASP:OD1	4:M:217:ASP:O	2.17	0.62
4:M:243:ILE:N	4:M:474:THR:HG21	2.14	0.62
4:M:261:ASN:CB	4:M:450:GLU:CG	2.71	0.62
4:M:306:LEU:HD21	4:M:317:MET:HE1	1.80	0.62
4:M:316:ARG:HG3	4:M:322:LEU:HD13	1.80	0.62
4:M:356:LEU:HD21	4:M:358:ILE:HG12	1.80	0.62
1:A:244:LEU:HA	1:A:256:LEU:HD13	1.80	0.62
1:A:438:ALA:O	1:A:439:ASP:HB2	1.99	0.62
1:A:495:ILE:CG2	1:A:515:CYS:SG	2.87	0.62
2:B:14:THR:CA	2:B:36:THR:HA	2.26	0.62
2:B:73:ASP:H	4:M:19:LEU:CG	2.13	0.62
2:B:107:ARG:NH2	4:M:126:ASN:HA	2.14	0.62
2:B:306:LEU:HD12	2:B:325:LEU:HD21	1.81	0.62
2:B:310:ILE:HG23	2:B:318:ILE:CG2	2.25	0.62
2:B:436:LEU:CD1	2:B:454:LEU:HD23	2.29	0.62

Continued on next page...

Continued from previous page...

Atom-1	Atom-2	Interatomic distance (Å)	Clash overlap (Å)
4:M:44:ASP:OD2	4:M:50:TYR:CE2	2.53	0.62
4:M:215:TYR:CB	4:M:467:TYR:O	2.47	0.62
4:M:373:ALA:O	4:M:418:GLU:N	2.33	0.62
4:M:442:GLN:HG3	4:M:443:SER:N	2.14	0.62
1:A:244:LEU:CG	1:A:281:LEU:HD11	2.29	0.62
1:A:609:LEU:HG	1:A:628:VAL:CB	2.30	0.62
2:B:25:VAL:HG23	2:B:32:GLU:CB	2.29	0.62
2:B:50:LEU:HG	2:B:62:ALA:HB2	1.82	0.62
2:B:220:ALA:CA	2:B:258:GLN:HG3	2.27	0.62
2:B:276:SER:O	2:B:295:ASN:CG	2.37	0.62
3:S:1:MET:H2	3:S:93:GLU:CB	2.13	0.62
4:M:69:ILE:CD1	4:M:94:GLU:N	2.63	0.62
4:M:70:ASN:CG	4:M:75:TRP:CE2	2.72	0.62
4:M:245:ASP:OD1	4:M:297:PHE:C	2.38	0.62
4:M:341:SER:HG	4:M:343:ASN:HD21	1.44	0.62
4:M:360:LEU:HD23	4:M:362:PHE:CE2	2.35	0.62
1:A:402:ILE:CG2	1:A:421:PRO:HA	2.29	0.61
1:A:433:ILE:CG2	1:A:476:GLN:HB2	2.30	0.61
1:A:495:ILE:CG2	1:A:515:CYS:CB	2.77	0.61
2:B:73:ASP:C	2:B:75:ASP:N	2.53	0.61
2:B:343:LEU:HD23	2:B:366:LEU:HD12	1.82	0.61
2:B:363:ILE:HG21	2:B:398:ILE:HD13	1.82	0.61
2:B:367:SER:HB2	2:B:401:THR:OG1	1.94	0.61
4:M:42:LEU:HD23	4:M:51:LEU:CD2	2.30	0.61
4:M:51:LEU:HB2	4:M:68:VAL:CG1	2.30	0.61
4:M:243:ILE:O	4:M:472:TYR:HD2	1.76	0.61
4:M:245:ASP:C	4:M:472:TYR:HE1	1.87	0.61
4:M:354:ASP:HB2	4:M:440:ILE:HD11	1.79	0.61
1:A:179:LYS:HD3	3:S:142:ILE:HD13	1.81	0.61
1:A:213:SER:CB	3:S:142:ILE:O	2.42	0.61
1:A:402:ILE:HG21	1:A:421:PRO:HA	1.82	0.61
2:B:90:ILE:CA	2:B:101:ILE:HD13	2.29	0.61
2:B:193:LEU:C	2:B:195:ILE:N	2.51	0.61
2:B:381:PHE:O	2:B:395:LYS:HD2	2.00	0.61
2:B:435:SER:O	2:B:437:SER:N	2.33	0.61
2:B:588:ILE:HG23	2:B:618:PHE:CE1	2.34	0.61
3:S:1:MET:H2	3:S:93:GLU:CG	2.13	0.61
3:S:29:LYS:O	3:S:32:LEU:N	2.32	0.61
3:S:136:VAL:O	3:S:140:MET:N	2.33	0.61
4:M:212:ASN:CB	4:M:250:LEU:HD23	2.30	0.61
4:M:222:PHE:CD2	4:M:439:TYR:CE2	2.85	0.61

Continued on next page...

Continued from previous page...

Atom-1	Atom-2	Interatomic distance (Å)	Clash overlap (Å)
2:B:363:ILE:HG13	2:B:381:PHE:CZ	2.35	0.61
2:B:513:TRP:NE1	2:B:517:GLU:HG3	2.15	0.61
4:M:69:ILE:HB	4:M:97:ASP:OD2	2.00	0.61
4:M:223:HIS:HB2	4:M:476:THR:OG1	1.99	0.61
4:M:223:HIS:NE2	4:M:436:GLU:HB2	2.15	0.61
1:A:158:LEU:HG	1:A:162:ILE:HD12	1.82	0.61
2:B:34:SER:HB3	2:B:65:ARG:CZ	2.30	0.61
2:B:83:PHE:CE1	2:B:87:VAL:HG21	2.35	0.61
2:B:162:VAL:HG23	2:B:173:VAL:CG1	2.30	0.61
2:B:219:TYR:CG	2:B:226:LEU:CB	2.79	0.61
2:B:382:TYR:OH	2:B:410:GLU:C	2.39	0.61
3:S:49:SER:O	3:S:77:TYR:O	2.18	0.61
4:M:88:ASP:OD2	4:M:134:PRO:HG3	2.01	0.61
4:M:224:VAL:H	4:M:479:PHE:CA	2.12	0.61
4:M:270:PRO:HB2	4:M:288:ILE:HD11	1.83	0.61
1:A:429:VAL:CG1	1:A:473:ILE:CG1	2.78	0.61
2:B:12:LEU:HD22	4:M:13:LYS:CE	2.31	0.61
2:B:216:LYS:NZ	4:M:136:VAL:CG2	2.64	0.61
2:B:231:ARG:CG	2:B:297:PRO:HB2	2.30	0.61
2:B:382:TYR:OH	2:B:411:ILE:N	2.33	0.61
2:B:429:VAL:CG1	2:B:467:VAL:CG1	2.78	0.61
4:M:45:SER:HA	4:M:75:TRP:CH2	2.35	0.61
4:M:216:VAL:O	4:M:216:VAL:HG23	1.98	0.61
4:M:222:PHE:HD1	4:M:240:ILE:HG23	1.53	0.61
4:M:250:LEU:HD11	4:M:254:PRO:HG2	1.82	0.61
1:A:204:VAL:HG13	1:A:239:LEU:HD11	1.82	0.61
1:A:217:ALA:HB2	3:S:140:MET:SD	2.40	0.61
1:A:233:PHE:O	1:A:234:ILE:O	2.15	0.61
1:A:567:GLN:O	1:A:568:GLU:C	2.35	0.61
2:B:200:MET:CG	2:B:232:ARG:HB3	2.28	0.61
2:B:260:LEU:HD23	2:B:293:VAL:HG11	1.82	0.61
2:B:292:GLU:HG3	2:B:296:ASP:HB2	1.78	0.61
2:B:325:LEU:HD13	2:B:339:PHE:CD1	2.36	0.61
2:B:347:VAL:CG2	2:B:381:PHE:HE1	2.12	0.61
4:M:114:ILE:C	4:M:116:ASN:H	2.01	0.61
1:A:251:TRP:CH2	3:S:97:ALA:C	2.74	0.61
1:A:438:ALA:O	1:A:441:TYR:CE1	2.54	0.61
1:A:441:TYR:HB3	1:A:444:VAL:HG23	1.83	0.61
1:A:453:VAL:O	1:A:457:LEU:HG	2.00	0.61
2:B:103:LEU:CD1	4:M:127:CYS:SG	2.88	0.61
2:B:237:ILE:HB	2:B:248:LEU:HD13	1.81	0.61

Continued on next page...

Continued from previous page...

Atom-1	Atom-2	Interatomic distance (Å)	Clash overlap (Å)
2:B:322:CYS:SG	2:B:343:LEU:HD22	2.41	0.61
2:B:382:TYR:OH	2:B:410:GLU:HB3	1.99	0.61
2:B:400:SER:HB3	2:B:435:SER:HA	1.65	0.61
4:M:65:TYR:CE1	4:M:66:PHE:O	2.53	0.61
4:M:212:ASN:CB	4:M:250:LEU:HA	2.29	0.61
4:M:302:TYR:HE1	4:M:304:VAL:HB	1.65	0.61
1:A:255:ARG:O	1:A:258:LYS:N	2.33	0.61
1:A:609:LEU:CG	1:A:628:VAL:HG11	2.31	0.61
2:B:9:ALA:CB	4:M:14:LEU:CB	2.53	0.61
2:B:73:ASP:O	2:B:75:ASP:N	2.34	0.61
4:M:117:ASN:O	4:M:121:ILE:HG13	2.00	0.61
4:M:405:THR:O	4:M:405:THR:HG22	2.01	0.61
4:M:443:SER:CB	4:M:447:ILE:N	2.62	0.61
1:A:154:ILE:HB	1:A:191:GLN:HG3	1.82	0.61
1:A:450:TYR:CD2	1:A:480:LEU:HG	2.36	0.61
1:A:637:GLU:OE2	2:B:554:LYS:HG3	2.00	0.61
2:B:196:LEU:CB	2:B:215:TYR:CE1	2.76	0.61
2:B:344:VAL:CG1	2:B:381:PHE:CE2	2.74	0.61
2:B:397:GLN:HG3	2:B:431:MET:CG	2.31	0.61
2:B:534:ILE:O	2:B:536:ASN:N	2.34	0.61
3:S:89:VAL:HG11	3:S:98:ILE:HD12	1.82	0.61
1:A:318:ARG:O	1:A:322:PHE:CD2	2.53	0.61
1:A:323:CYS:SG	1:A:334:SER:HB2	2.39	0.61
1:A:372:ILE:HG22	1:A:431:VAL:HG21	1.82	0.61
2:B:38:TYR:OH	2:B:43:ASN:O	2.18	0.61
2:B:178:ILE:CD1	2:B:218:CYS:CB	2.78	0.61
2:B:239:GLN:OE1	4:M:280:ASP:OD1	2.09	0.61
2:B:247:TYR:CD1	4:M:136:VAL:CG1	2.77	0.61
2:B:408:VAL:HG11	2:B:446:TRP:HB3	1.83	0.61
2:B:464:SER:OG	2:B:467:VAL:HG23	2.01	0.61
2:B:549:LEU:HD13	2:B:595:VAL:CG1	2.31	0.61
4:M:350:VAL:CA	4:M:442:GLN:HB2	2.25	0.61
1:A:103:LYS:O	1:A:104:ARG:C	2.29	0.60
2:B:216:LYS:CD	2:B:251:LEU:HA	2.30	0.60
2:B:251:LEU:C	2:B:253:ILE:N	2.54	0.60
2:B:599:ALA:O	2:B:602:ASP:N	2.30	0.60
4:M:435:LEU:H	4:M:479:PHE:HB2	1.65	0.60
1:A:132:LEU:HD11	1:A:150:LEU:HD12	1.82	0.60
1:A:185:LEU:HD13	1:A:203:PHE:CE1	2.36	0.60
1:A:231:GLN:HB2	1:A:232:PRO:HD3	1.81	0.60
1:A:422:GLU:HB2	3:S:62:GLU:CD	2.21	0.60

Continued on next page...

Continued from previous page...

Atom-1	Atom-2	Interatomic distance (Å)	Clash overlap (Å)
1:A:625:LEU:O	1:A:627:GLU:N	2.34	0.60
2:B:1:MET:HG3	4:M:39:PRO:CG	2.27	0.60
2:B:12:LEU:CD2	4:M:13:LYS:HE3	2.31	0.60
2:B:230:PHE:HD1	2:B:298:ASP:HA	1.66	0.60
3:S:137:GLN:C	3:S:140:MET:H	2.04	0.60
4:M:437:TYR:CE1	4:M:479:PHE:CE2	2.89	0.60
1:A:68:THR:OG1	3:S:166:LYS:CD	2.50	0.60
1:A:163:ALA:HB1	1:A:199:ASN:HD21	1.66	0.60
1:A:232:PRO:O	1:A:235:GLN:HB2	2.00	0.60
2:B:14:THR:CB	2:B:36:THR:HG23	2.16	0.60
2:B:252:LEU:CB	2:B:302:PHE:CG	2.83	0.60
2:B:279:LEU:HD12	2:B:285:GLU:CG	2.31	0.60
3:S:54:PRO:O	3:S:69:ASN:HB2	2.01	0.60
3:S:99:LEU:O	3:S:102:ILE:HB	2.00	0.60
4:M:7:ILE:HG12	4:M:76:CYS:SG	2.42	0.60
4:M:76:CYS:HB3	4:M:93:LEU:HD22	1.83	0.60
4:M:213:GLU:OE1	4:M:467:TYR:HD1	1.84	0.60
4:M:221:THR:N	4:M:474:THR:HG1	1.73	0.60
4:M:252:ASP:C	4:M:254:PRO:HD2	2.20	0.60
4:M:323:MET:HB3	4:M:340:LEU:CD1	2.27	0.60
1:A:200:PHE:HZ	1:A:236:LEU:HD21	1.60	0.60
1:A:318:ARG:O	1:A:322:PHE:HD2	1.84	0.60
1:A:436:CYS:HB2	1:A:450:TYR:CE1	2.36	0.60
2:B:161:LEU:CB	2:B:173:VAL:HG22	2.26	0.60
2:B:560:ILE:HG22	2:B:561:ASP:N	2.16	0.60
4:M:118:TYR:CD1	4:M:118:TYR:C	2.72	0.60
4:M:290:PHE:HZ	4:M:293:PRO:CD	2.14	0.60
4:M:360:LEU:CD2	4:M:362:PHE:CE2	2.84	0.60
1:A:64:LEU:CB	1:A:102:GLN:HE22	2.14	0.60
2:B:18:ILE:HG22	2:B:36:THR:C	1.90	0.60
2:B:34:SER:OG	2:B:35:TYR:N	2.34	0.60
2:B:106:LEU:HD21	2:B:144:ASP:CG	2.21	0.60
2:B:139:LEU:HD11	2:B:176:ALA:HB1	1.83	0.60
2:B:206:LYS:O	2:B:210:CYS:SG	2.53	0.60
2:B:374:PHE:O	2:B:374:PHE:CG	2.50	0.60
2:B:436:LEU:HB2	2:B:454:LEU:HD21	1.83	0.60
2:B:588:ILE:HG21	2:B:618:PHE:HE1	1.66	0.60
3:S:17:VAL:HG21	3:S:19:PHE:HZ	1.61	0.60
3:S:89:VAL:CG1	3:S:98:ILE:HD12	2.31	0.60
3:S:93:GLU:HA	3:S:93:GLU:OE1	2.00	0.60
4:M:344:ILE:H	4:M:408:VAL:HG22	1.67	0.60

Continued on next page...

Continued from previous page...

Atom-1	Atom-2	Interatomic distance (Å)	Clash overlap (Å)
4:M:356:LEU:HD23	4:M:358:ILE:HG13	1.82	0.60
4:M:442:GLN:CG	4:M:443:SER:H	2.12	0.60
1:A:189:PHE:CB	1:A:225:LEU:CD2	2.80	0.60
1:A:375:VAL:O	1:A:376:GLU:C	2.39	0.60
2:B:20:ARG:HD3	4:M:115:VAL:O	2.02	0.60
2:B:193:LEU:O	2:B:229:HIS:HE1	1.83	0.60
2:B:515:PHE:HD2	2:B:529:VAL:HG21	1.64	0.60
2:B:523:PHE:CD2	2:B:559:ASP:OD1	2.54	0.60
2:B:546:CYS:CB	2:B:607:ILE:HG12	2.32	0.60
4:M:219:LEU:HB2	4:M:472:TYR:C	2.16	0.60
4:M:219:LEU:H	4:M:472:TYR:HB2	1.67	0.60
4:M:225:VAL:HA	4:M:480:GLN:O	2.01	0.60
4:M:260:LEU:HD23	4:M:449:VAL:HG22	1.81	0.60
1:A:253:ILE:O	1:A:257:LEU:HG	2.02	0.60
1:A:328:PRO:O	3:S:50:PHE:CZ	2.46	0.60
1:A:537:THR:O	1:A:540:ILE:HG22	2.01	0.60
1:A:589:SER:C	1:A:597:GLN:HG3	1.87	0.60
2:B:144:ASP:OD2	4:M:131:ALA:N	2.33	0.60
2:B:399:LEU:HB3	2:B:411:ILE:CG2	2.32	0.60
1:A:178:ARG:HD3	1:A:209:ASP:OD2	2.01	0.60
1:A:609:LEU:HG	1:A:628:VAL:HB	1.84	0.60
2:B:189:HIS:CE1	2:B:222:HIS:HB3	2.36	0.60
2:B:220:ALA:O	2:B:258:GLN:OE1	2.19	0.60
3:S:53:THR:HG21	3:S:67:GLU:O	2.02	0.60
4:M:6:TYR:HA	4:M:17:GLN:HA	1.82	0.60
4:M:80:THR:HG23	4:M:89:CYS:SG	2.41	0.60
4:M:360:LEU:HD13	4:M:433:VAL:HG23	1.83	0.60
4:M:374:TYR:OH	4:M:394:GLN:CA	2.50	0.60
2:B:182:ARG:HD2	2:B:217:GLU:HB3	1.84	0.60
2:B:280:PRO:CG	2:B:283:TYR:HD2	2.07	0.60
2:B:336:ASN:O	2:B:373:LEU:CD2	2.43	0.60
2:B:418:TYR:HD1	2:B:424:PHE:CE2	2.20	0.60
2:B:497:LEU:HD13	2:B:503:LEU:HD12	1.83	0.60
2:B:500:GLN:HB3	2:B:503:LEU:HG	1.83	0.60
4:M:6:TYR:CD1	4:M:6:TYR:N	2.68	0.60
4:M:257:ALA:HB3	4:M:453:ASP:CG	2.22	0.60
1:A:441:TYR:HB3	1:A:444:VAL:CG2	2.32	0.60
1:A:480:LEU:O	1:A:483:LYS:O	2.20	0.60
1:A:632:PHE:O	1:A:635:ALA:N	2.34	0.60
2:B:216:LYS:HD3	2:B:251:LEU:HA	1.84	0.60
2:B:231:ARG:NE	2:B:297:PRO:HB2	2.17	0.60

Continued on next page...

Continued from previous page...

Atom-1	Atom-2	Interatomic distance (Å)	Clash overlap (Å)
2:B:374:PHE:CZ	2:B:381:PHE:HD2	2.06	0.60
4:M:46:SER:O	4:M:48:ASP:N	2.34	0.60
4:M:217:ASP:HB3	4:M:470:ALA:C	2.22	0.60
4:M:220:GLU:OE1	4:M:222:PHE:CE1	2.55	0.60
4:M:315:VAL:O	4:M:315:VAL:HG12	2.02	0.60
2:B:72:SER:CB	4:M:17:GLN:NE2	2.65	0.59
2:B:89:ASN:C	2:B:101:ILE:CD1	2.70	0.59
2:B:302:PHE:CD2	2:B:328:LEU:HD11	2.36	0.59
2:B:374:PHE:HE2	2:B:402:LEU:HD11	0.95	0.59
2:B:392:SER:HG	2:B:424:PHE:HE1	1.50	0.59
2:B:466:SER:O	2:B:469:ASP:HB2	2.02	0.59
2:B:513:TRP:HE1	2:B:517:GLU:CG	2.15	0.59
4:M:6:TYR:CA	4:M:16:PHE:O	2.49	0.59
4:M:65:TYR:HE2	4:M:86:PRO:O	1.85	0.59
4:M:374:TYR:CB	4:M:417:TYR:CD1	2.85	0.59
4:M:429:ASP:O	4:M:430:LEU:C	2.40	0.59
1:A:64:LEU:CG	1:A:102:GLN:HE22	2.14	0.59
1:A:460:LEU:HA	4:M:58:ARG:NH2	2.17	0.59
2:B:5:ILE:CD1	4:M:39:PRO:CD	2.80	0.59
2:B:252:LEU:CB	2:B:302:PHE:CD2	2.69	0.59
2:B:340:ILE:CD1	2:B:366:LEU:HB3	2.32	0.59
2:B:347:VAL:HB	2:B:381:PHE:CE1	2.36	0.59
2:B:495:ASP:O	2:B:499:VAL:HG23	2.02	0.59
4:M:19:LEU:HD12	4:M:20:LEU:H	1.66	0.59
4:M:226:PHE:CE2	4:M:321:GLY:O	2.55	0.59
4:M:410:VAL:HG12	4:M:412:ARG:HG3	1.83	0.59
1:A:384:LEU:HD12	1:A:385:LYS:N	2.17	0.59
1:A:397:ASP:O	1:A:418:ILE:HD12	2.03	0.59
1:A:450:TYR:HE2	1:A:480:LEU:HB2	1.66	0.59
2:B:107:ARG:HD3	4:M:130:GLU:OE2	2.03	0.59
2:B:343:LEU:O	2:B:346:THR:HB	2.01	0.59
2:B:344:VAL:HA	2:B:381:PHE:HZ	1.67	0.59
2:B:355:ASN:O	2:B:359:LEU:HD23	2.02	0.59
2:B:430:ILE:HG12	2:B:467:VAL:CA	2.31	0.59
4:M:114:ILE:O	4:M:117:ASN:N	2.35	0.59
4:M:224:VAL:N	4:M:479:PHE:CA	2.65	0.59
4:M:241:HIS:O	4:M:474:THR:HG21	2.01	0.59
4:M:269:ILE:N	4:M:302:TYR:CE2	2.70	0.59
4:M:437:TYR:HD1	4:M:437:TYR:H	1.49	0.59
1:A:402:ILE:CB	1:A:421:PRO:HA	2.32	0.59
3:S:47:GLN:O	3:S:48:SER:C	2.34	0.59

Continued on next page...

Continued from previous page...

Atom-1	Atom-2	Interatomic distance (Å)	Clash overlap (Å)
4:M:96:ILE:HD12	4:M:125:PHE:CE1	2.36	0.59
4:M:240:ILE:HG21	4:M:444:ALA:CA	2.26	0.59
4:M:290:PHE:CZ	4:M:293:PRO:CD	2.85	0.59
1:A:300:LYS:O	1:A:301:GLY:C	2.38	0.59
1:A:559:PHE:CD2	1:A:581:LEU:HD22	2.37	0.59
1:A:625:LEU:O	1:A:626:SER:C	2.32	0.59
2:B:120:ILE:HG13	2:B:150:LEU:HD22	1.84	0.59
2:B:177:ILE:HG21	2:B:196:LEU:HG	1.84	0.59
2:B:178:ILE:CD1	2:B:218:CYS:HB2	2.28	0.59
2:B:181:TYR:CZ	2:B:222:HIS:CD2	2.88	0.59
2:B:261:PRO:HG2	2:B:292:GLU:CB	2.32	0.59
2:B:436:LEU:HD13	2:B:454:LEU:CD2	2.31	0.59
4:M:20:LEU:HD13	4:M:129:VAL:HG21	1.84	0.59
4:M:42:LEU:CD2	4:M:51:LEU:HD21	2.32	0.59
1:A:395:PHE:CD2	1:A:428:MET:HE3	2.37	0.59
1:A:435:ILE:CG2	1:A:441:TYR:CE2	2.85	0.59
2:B:80:GLN:CA	2:B:108:PHE:HE2	2.16	0.59
2:B:170:ARG:HA	2:B:199:LEU:HD22	1.85	0.59
2:B:216:LYS:HZ1	4:M:136:VAL:HG21	1.66	0.59
2:B:378:THR:C	2:B:380:LYS:H	2.05	0.59
2:B:493:LEU:HD23	2:B:514:LEU:HD23	1.83	0.59
3:S:73:ILE:HG21	3:S:88:ILE:HG23	1.76	0.59
4:M:16:PHE:HB2	4:M:118:TYR:CD2	2.38	0.59
4:M:243:ILE:C	4:M:472:TYR:HB3	2.22	0.59
1:A:605:GLU:HB2	1:A:636:TYR:CE2	2.38	0.59
2:B:73:ASP:OD1	4:M:24:ALA:HB2	1.96	0.59
2:B:217:GLU:OE2	4:M:133:GLU:CD	2.40	0.59
2:B:399:LEU:HD12	2:B:415:LEU:CD2	2.33	0.59
4:M:70:ASN:HB2	4:M:75:TRP:CZ3	2.37	0.59
4:M:110:SER:O	4:M:113:LYS:N	2.27	0.59
4:M:129:VAL:O	4:M:133:GLU:O	2.21	0.59
4:M:374:TYR:HB2	4:M:417:TYR:CD1	2.38	0.59
1:A:121:LEU:CD1	1:A:155:THR:HG23	2.32	0.59
1:A:189:PHE:CD2	1:A:225:LEU:HD11	2.37	0.59
1:A:508:LEU:HD12	4:M:59:ASP:OD1	2.03	0.59
1:A:562:TRP:CE3	1:A:574:ILE:HD12	2.37	0.59
2:B:268:LYS:C	2:B:273:SER:OG	2.40	0.59
2:B:559:ASP:O	2:B:562:ASN:CB	2.50	0.59
2:B:585:GLY:O	2:B:589:SER:CB	2.50	0.59
2:B:592:TYR:C	2:B:592:TYR:CD1	2.76	0.59
4:M:224:VAL:N	4:M:479:PHE:CB	2.55	0.59

Continued on next page...

Continued from previous page...

Atom-1	Atom-2	Interatomic distance (Å)	Clash overlap (Å)
4:M:235:LEU:HD11	4:M:306:LEU:CD1	2.33	0.59
4:M:244:VAL:O	4:M:299:LEU:CB	2.51	0.59
4:M:338:PHE:CZ	4:M:379:LEU:HD21	2.37	0.59
4:M:386:PHE:CD2	4:M:386:PHE:O	2.56	0.59
1:A:185:LEU:HB3	1:A:203:PHE:HE1	1.67	0.59
2:B:13:ASP:O	2:B:17:VAL:N	2.30	0.59
2:B:90:ILE:N	2:B:101:ILE:CD1	2.65	0.59
2:B:178:ILE:CD1	2:B:214:ALA:C	2.71	0.59
3:S:89:VAL:CG1	3:S:98:ILE:CG2	2.80	0.59
4:M:217:ASP:CB	4:M:470:ALA:C	2.71	0.59
4:M:240:ILE:HB	4:M:304:VAL:HG12	1.84	0.59
4:M:271:SER:C	4:M:272:LEU:HD12	2.23	0.59
4:M:283:PHE:HE2	4:M:289:THR:HB	1.65	0.59
1:A:186:PHE:CD1	1:A:186:PHE:C	2.76	0.59
1:A:516:ILE:CG2	1:A:554:ALA:HB3	2.32	0.59
2:B:69:ILE:CG2	2:B:74:ASP:HB3	2.32	0.59
2:B:78:ASP:O	2:B:79:VAL:C	2.25	0.59
2:B:106:LEU:HD22	2:B:144:ASP:HB2	1.83	0.59
2:B:193:LEU:CB	2:B:225:LEU:CD1	2.81	0.59
2:B:299:LEU:C	2:B:299:LEU:HD13	2.24	0.59
2:B:334:MET:O	2:B:373:LEU:HD22	2.03	0.59
2:B:360:LEU:HD21	2:B:395:LYS:CE	2.33	0.59
2:B:360:LEU:HD21	2:B:395:LYS:CD	2.32	0.59
2:B:396:ILE:HD11	2:B:418:TYR:CE2	2.38	0.59
2:B:549:LEU:HD12	2:B:595:VAL:CG1	2.32	0.59
4:M:235:LEU:HD23	4:M:235:LEU:C	2.23	0.59
4:M:290:PHE:CZ	4:M:291:ILE:O	2.55	0.59
4:M:347:PHE:CE1	4:M:439:TYR:CG	2.90	0.59
1:A:125:THR:CB	1:A:158:LEU:HD13	2.33	0.58
1:A:186:PHE:HD1	1:A:187:LYS:N	2.00	0.58
1:A:225:LEU:HB3	1:A:233:PHE:HE2	1.62	0.58
1:A:383:ASN:O	1:A:387:ILE:HG12	2.03	0.58
1:A:621:LEU:HD13	1:A:621:LEU:C	2.23	0.58
2:B:211:ALA:HB3	2:B:233:TYR:CZ	2.37	0.58
2:B:223:LEU:CD1	2:B:258:GLN:CA	2.78	0.58
2:B:284:ASN:O	2:B:285:GLU:C	2.34	0.58
2:B:343:LEU:CD2	2:B:363:ILE:CD1	2.74	0.58
2:B:537:PHE:CE2	2:B:545:ARG:HG2	2.38	0.58
2:B:537:PHE:CE1	2:B:598:LEU:HB3	2.38	0.58
4:M:9:ASP:HB3	4:M:111:ILE:HG22	1.84	0.58
4:M:244:VAL:HG13	4:M:472:TYR:HE2	1.65	0.58

Continued on next page...

Continued from previous page...

Atom-1	Atom-2	Interatomic distance (Å)	Clash overlap (Å)
4:M:354:ASP:CB	4:M:440:ILE:CD1	2.77	0.58
1:A:509:PRO:O	1:A:512:LEU:HB2	2.03	0.58
1:A:555:LEU:HD13	1:A:581:LEU:HD11	1.84	0.58
2:B:37:TYR:HD2	2:B:38:TYR:CE1	2.20	0.58
2:B:200:MET:CE	2:B:229:HIS:HA	2.33	0.58
2:B:364:HIS:O	2:B:368:ILE:HG12	2.03	0.58
2:B:526:CYS:N	2:B:527:PRO:HD3	2.17	0.58
2:B:580:TYR:O	2:B:582:ASP:CG	2.41	0.58
3:S:65:ASN:O	3:S:66:ASP:C	2.39	0.58
4:M:293:PRO:CB	4:M:294:ASP:O	2.49	0.58
4:M:405:THR:O	4:M:407:THR:CG2	2.48	0.58
1:A:125:THR:CG2	1:A:158:LEU:HD13	2.34	0.58
1:A:185:LEU:CB	1:A:203:PHE:HE1	2.16	0.58
1:A:224:GLU:O	1:A:227:LYS:N	2.36	0.58
2:B:67:ILE:HG23	4:M:18:TYR:OH	2.03	0.58
2:B:80:GLN:CA	2:B:108:PHE:CE2	2.86	0.58
2:B:155:LEU:O	2:B:158:VAL:N	2.37	0.58
2:B:204:ASP:HB3	2:B:207:VAL:CG2	2.34	0.58
2:B:219:TYR:CD1	2:B:226:LEU:CB	2.82	0.58
2:B:344:VAL:CB	2:B:377:TYR:HB3	2.33	0.58
1:A:179:LYS:CE	3:S:140:MET:CB	2.66	0.58
1:A:291:ILE:HG21	1:A:322:PHE:CE2	2.37	0.58
1:A:585:PHE:HE2	1:A:603:VAL:HG12	1.49	0.58
2:B:430:ILE:CD1	2:B:466:SER:CB	2.81	0.58
3:S:15:ARG:O	3:S:125:TRP:CE2	2.57	0.58
1:A:97:SER:C	1:A:98:ASN:O	2.16	0.58
1:A:529:GLY:C	1:A:562:TRP:CZ2	2.76	0.58
2:B:143:SER:C	2:B:179:LYS:HD2	2.23	0.58
2:B:241:ASP:OD1	2:B:241:ASP:O	2.20	0.58
2:B:333:GLN:O	2:B:336:ASN:HB2	2.03	0.58
2:B:556:LEU:C	2:B:588:ILE:HD11	2.24	0.58
3:S:55:PRO:HG3	3:S:71:GLU:HG2	1.85	0.58
4:M:220:GLU:HB2	4:M:222:PHE:HE1	1.66	0.58
1:A:405:THR:CB	2:B:7:ARG:HE	2.16	0.58
1:A:562:TRP:HE3	1:A:574:ILE:HD12	1.69	0.58
2:B:34:SER:OG	2:B:65:ARG:NE	2.37	0.58
2:B:260:LEU:HD22	2:B:291:TYR:CE1	2.37	0.58
2:B:404:ASN:O	2:B:408:VAL:HG23	2.04	0.58
2:B:563:PHE:O	2:B:564:LYS:O	2.21	0.58
3:S:5:VAL:HB	3:S:132:LEU:HD21	1.84	0.58
3:S:87:PHE:CE1	3:S:102:ILE:HG12	2.39	0.58

Continued on next page...

Continued from previous page...

Atom-1	Atom-2	Interatomic distance (Å)	Clash overlap (Å)
1:A:244:LEU:CA	1:A:256:LEU:HD13	2.33	0.58
1:A:412:LYS:O	1:A:413:SER:C	2.34	0.58
1:A:462:GLN:CD	4:M:59:ASP:C	2.34	0.58
2:B:102:HIS:CE1	2:B:138:ALA:N	2.72	0.58
2:B:329:ALA:CB	2:B:334:MET:HG2	2.30	0.58
2:B:479:VAL:CG1	2:B:486:HIS:CG	2.70	0.58
2:B:519:ALA:O	2:B:523:PHE:CA	2.52	0.58
4:M:218:LEU:N	4:M:218:LEU:HD12	2.18	0.58
4:M:338:PHE:CE1	4:M:415:ILE:HG13	2.38	0.58
1:A:429:VAL:HG21	1:A:469:LEU:HD11	1.86	0.58
1:A:487:MET:O	1:A:488:ARG:C	2.38	0.58
2:B:2:VAL:CG1	4:M:54:SER:HG	2.14	0.58
2:B:120:ILE:HG13	2:B:150:LEU:CD2	2.33	0.58
2:B:127:LEU:HD11	2:B:142:LEU:HD11	1.83	0.58
2:B:139:LEU:CG	2:B:176:ALA:CB	2.80	0.58
2:B:256:CYS:O	2:B:257:LYS:C	2.38	0.58
2:B:334:MET:C	2:B:336:ASN:N	2.44	0.58
2:B:378:THR:C	2:B:380:LYS:N	2.54	0.58
2:B:479:VAL:CB	2:B:486:HIS:CD2	2.86	0.58
2:B:553:ALA:HA	2:B:556:LEU:HD12	1.86	0.58
3:S:6:LEU:HD11	3:S:14:PRO:CB	2.32	0.58
3:S:35:VAL:HG12	3:S:75:ILE:HD13	1.85	0.58
4:M:4:SER:HB3	4:M:79:SER:OG	2.03	0.58
4:M:6:TYR:CD2	4:M:17:GLN:HG3	2.38	0.58
4:M:70:ASN:HB2	4:M:75:TRP:CD2	2.39	0.58
4:M:222:PHE:CG	4:M:240:ILE:HG12	2.38	0.58
4:M:235:LEU:CD1	4:M:306:LEU:HD13	2.34	0.58
4:M:258:VAL:CG1	4:M:449:VAL:HG13	2.31	0.58
4:M:306:LEU:O	4:M:307:SER:O	2.22	0.58
1:A:436:CYS:SG	1:A:450:TYR:CZ	2.97	0.58
2:B:28:SER:CA	2:B:58:GLU:CG	2.80	0.58
2:B:100:LEU:HD23	4:M:123:LEU:CD1	2.34	0.58
2:B:226:LEU:O	2:B:226:LEU:HD12	2.04	0.58
2:B:418:TYR:HD1	2:B:418:TYR:O	1.86	0.58
2:B:493:LEU:O	2:B:496:LEU:N	2.34	0.58
2:B:534:ILE:HD11	2:B:591:MET:O	2.04	0.58
1:A:229:ASN:O	1:A:230:PRO:C	2.33	0.58
1:A:274:LEU:O	1:A:277:LYS:N	2.36	0.58
1:A:581:LEU:HD11	1:A:585:PHE:HZ	1.66	0.58
2:B:9:ALA:CB	4:M:14:LEU:HD12	2.33	0.58
2:B:28:SER:HA	2:B:58:GLU:HG3	1.86	0.58

Continued on next page...

Continued from previous page...

Atom-1	Atom-2	Interatomic distance (Å)	Clash overlap (Å)
2:B:103:LEU:CD2	4:M:127:CYS:SG	2.92	0.58
2:B:351:GLU:HB2	4:M:476:THR:HG22	1.86	0.58
2:B:546:CYS:HB2	2:B:607:ILE:HG12	1.85	0.58
4:M:241:HIS:HB2	4:M:476:THR:CG2	2.34	0.58
4:M:243:ILE:CD1	4:M:301:GLU:HB3	2.31	0.58
1:A:64:LEU:HG	1:A:102:GLN:NE2	2.18	0.57
1:A:140:VAL:O	1:A:141:VAL:C	2.38	0.57
1:A:186:PHE:CD1	1:A:187:LYS:N	2.72	0.57
1:A:278:ILE:O	1:A:280:GLU:N	2.36	0.57
1:A:293:GLU:OE1	3:S:94:SER:HB3	2.04	0.57
1:A:529:GLY:C	1:A:562:TRP:CH2	2.78	0.57
1:A:556:VAL:CG2	1:A:603:VAL:CG1	2.76	0.57
2:B:104:TYR:O	2:B:107:ARG:CG	2.51	0.57
2:B:364:HIS:CE1	2:B:368:ILE:HD11	2.38	0.57
2:B:472:VAL:HG11	2:B:511:ILE:H	1.68	0.57
2:B:513:TRP:CD1	2:B:517:GLU:HG2	2.39	0.57
2:B:519:ALA:O	2:B:523:PHE:CG	2.55	0.57
4:M:250:LEU:HD13	4:M:254:PRO:CG	2.34	0.57
4:M:479:PHE:CD1	4:M:479:PHE:N	2.57	0.57
2:B:306:LEU:CD1	2:B:325:LEU:CD2	2.83	0.57
2:B:310:ILE:HB	2:B:342:ALA:HB1	1.83	0.57
2:B:347:VAL:O	2:B:348:THR:C	2.40	0.57
2:B:396:ILE:CD1	2:B:418:TYR:OH	2.52	0.57
2:B:566:ALA:CA	2:B:574:ASN:CB	2.82	0.57
3:S:51:LEU:HB2	3:S:77:TYR:CE1	2.40	0.57
3:S:75:ILE:HG21	3:S:77:TYR:OH	2.04	0.57
3:S:98:ILE:O	3:S:102:ILE:HG13	2.04	0.57
4:M:243:ILE:HG12	4:M:474:THR:HG22	1.84	0.57
4:M:362:PHE:HE1	4:M:374:TYR:CZ	2.21	0.57
4:M:379:LEU:CA	4:M:412:ARG:O	2.49	0.57
1:A:151:SER:C	1:A:153:ILE:N	2.52	0.57
2:B:219:TYR:OH	2:B:226:LEU:N	2.37	0.57
2:B:223:LEU:CD2	2:B:258:GLN:HB2	2.33	0.57
1:A:281:LEU:O	1:A:282:MET:O	2.21	0.57
1:A:316:LEU:HD21	1:A:341:ILE:HG13	1.85	0.57
1:A:323:CYS:SG	1:A:338:PHE:HE2	2.27	0.57
1:A:462:GLN:NE2	4:M:60:LEU:O	2.37	0.57
1:A:558:VAL:CG1	1:A:562:TRP:CZ2	2.87	0.57
1:A:579:LYS:HA	1:A:582:ILE:HD12	1.85	0.57
1:A:638:LEU:HD13	2:B:557:SER:HG	1.65	0.57
2:B:245:GLN:CG	2:B:309:LEU:HD11	2.35	0.57

Continued on next page...

Continued from previous page...

Atom-1	Atom-2	Interatomic distance (Å)	Clash overlap (Å)
2:B:278:PRO:CA	2:B:288:TYR:HB3	2.26	0.57
2:B:363:ILE:CG2	2:B:398:ILE:HG12	2.34	0.57
2:B:386:LYS:NZ	4:M:479:PHE:O	2.37	0.57
2:B:437:SER:OG	2:B:474:VAL:HA	2.04	0.57
2:B:540:GLU:OE1	2:B:548:ILE:HD12	2.04	0.57
4:M:374:TYR:HH	4:M:394:GLN:C	2.08	0.57
4:M:433:VAL:O	4:M:435:LEU:HD12	2.04	0.57
1:A:92:LEU:HD13	1:A:123:LEU:CD1	2.35	0.57
2:B:20:ARG:NH2	4:M:118:TYR:CG	2.71	0.57
2:B:102:HIS:CE1	2:B:138:ALA:CA	2.87	0.57
2:B:251:LEU:O	2:B:253:ILE:N	2.38	0.57
2:B:393:ILE:HG12	2:B:428:VAL:HA	1.86	0.57
2:B:559:ASP:CB	2:B:563:PHE:CE2	2.88	0.57
3:S:17:VAL:CG2	3:S:19:PHE:CE1	2.87	0.57
4:M:104:PHE:CE2	4:M:117:ASN:CB	2.86	0.57
4:M:354:ASP:CB	4:M:440:ILE:HD11	2.33	0.57
1:A:189:PHE:HB3	1:A:225:LEU:CD2	2.35	0.57
1:A:260:PHE:CE2	1:A:274:LEU:HD11	2.40	0.57
2:B:116:THR:HG22	2:B:150:LEU:HD11	1.86	0.57
2:B:476:ARG:CB	2:B:514:LEU:HD13	2.35	0.57
2:B:588:ILE:HG21	2:B:618:PHE:CE1	2.40	0.57
4:M:66:PHE:CE1	4:M:79:SER:HB3	2.40	0.57
4:M:224:VAL:CG1	4:M:226:PHE:CE2	2.88	0.57
2:B:141:ALA:O	2:B:142:LEU:C	2.42	0.57
2:B:161:LEU:HB3	2:B:173:VAL:HG21	1.82	0.57
2:B:170:ARG:HA	2:B:199:LEU:CD2	2.35	0.57
2:B:347:VAL:CA	2:B:359:LEU:HG	2.33	0.57
3:S:64:ASN:C	3:S:66:ASP:H	2.08	0.57
3:S:167:ILE:HG22	3:S:168:GLY:HA2	1.84	0.57
4:M:41:LEU:CB	4:M:51:LEU:HA	2.34	0.57
4:M:56:VAL:H	4:M:64:LYS:CG	2.13	0.57
4:M:302:TYR:CE1	4:M:304:VAL:HB	2.40	0.57
4:M:383:HIS:HB2	4:M:403:THR:OG1	2.02	0.57
1:A:222:ILE:CG2	1:A:240:LEU:HD11	2.31	0.57
1:A:609:LEU:O	1:A:612:GLU:N	2.34	0.57
2:B:123:LEU:HD12	2:B:142:LEU:CD2	2.35	0.57
2:B:259:TYR:O	2:B:293:VAL:HG21	2.03	0.57
2:B:347:VAL:CG2	2:B:381:PHE:CZ	2.87	0.57
2:B:386:LYS:NZ	4:M:478:ASN:N	2.53	0.57
2:B:436:LEU:CB	2:B:454:LEU:HD21	2.34	0.57
2:B:451:MET:HG3	2:B:489:ILE:CG1	2.22	0.57

Continued on next page...

Continued from previous page...

Atom-1	Atom-2	Interatomic distance (Å)	Clash overlap (Å)
3:S:75:ILE:CG2	3:S:77:TYR:CZ	2.88	0.57
3:S:130:SER:HB2	3:S:156:LEU:HD13	1.83	0.57
4:M:271:SER:O	4:M:300:LEU:CA	2.48	0.57
4:M:273:HIS:CB	4:M:298:ARG:O	2.42	0.57
4:M:344:ILE:HD12	4:M:407:THR:O	2.03	0.57
1:A:216:SER:HB2	1:A:252:ILE:HG12	1.87	0.57
1:A:433:ILE:HG23	1:A:476:GLN:CB	2.33	0.57
1:A:565:ASN:O	1:A:567:GLN:N	2.38	0.57
2:B:34:SER:HB3	2:B:65:ARG:HH12	1.65	0.57
4:M:5:PHE:HE2	4:M:20:LEU:HD13	1.68	0.57
1:A:492:ILE:HD13	1:A:526:VAL:CG2	2.34	0.57
1:A:517:TRP:NE1	2:B:605:PHE:CD2	2.71	0.57
2:B:144:ASP:OD1	4:M:131:ALA:C	2.44	0.57
2:B:162:VAL:HG22	2:B:199:LEU:HG	1.86	0.57
4:M:270:PRO:O	4:M:272:LEU:CD1	2.53	0.57
4:M:358:ILE:O	4:M:397:TRP:HE3	1.88	0.57
1:A:91:ILE:HG23	1:A:106:GLY:HA2	1.86	0.56
1:A:268:PRO:HA	1:A:271:ARG:HE	1.69	0.56
1:A:291:ILE:O	1:A:295:VAL:HG23	2.05	0.56
1:A:322:PHE:HB3	1:A:330:LEU:HD21	1.87	0.56
2:B:147:MET:HB2	2:B:150:LEU:HG	1.87	0.56
2:B:227:HIS:C	2:B:229:HIS:N	2.58	0.56
2:B:256:CYS:HG	2:B:328:LEU:HD21	1.69	0.56
2:B:371:GLN:CB	2:B:401:THR:O	2.48	0.56
2:B:414:GLU:O	2:B:417:TYR:HB3	2.05	0.56
3:S:17:VAL:HG22	3:S:19:PHE:CZ	2.36	0.56
4:M:372:ILE:O	4:M:372:ILE:HG22	2.04	0.56
1:A:103:LYS:C	1:A:107:TYR:CD2	2.77	0.56
1:A:147:LEU:HD13	1:A:181:ALA:HA	1.87	0.56
1:A:215:VAL:HG13	1:A:243:ILE:CG2	2.13	0.56
1:A:257:LEU:CD2	1:A:278:ILE:HG22	2.33	0.56
2:B:340:ILE:HG13	2:B:373:LEU:CG	2.35	0.56
2:B:397:GLN:HG3	2:B:431:MET:HG2	1.85	0.56
2:B:527:PRO:HB3	2:B:587:ARG:HG3	1.85	0.56
3:S:8:PHE:CD2	3:S:36:TYR:HE1	2.22	0.56
4:M:290:PHE:CE2	4:M:297:PHE:CE1	2.93	0.56
4:M:424:PHE:CE1	4:M:428:VAL:HG22	2.40	0.56
1:A:113:SER:O	1:A:115:TYR:N	2.38	0.56
1:A:150:LEU:HD22	1:A:158:LEU:HD11	1.86	0.56
1:A:359:LEU:HB3	1:A:367:ILE:CG2	2.35	0.56
1:A:503:ASN:CA	4:M:59:ASP:C	2.55	0.56

Continued on next page...

Continued from previous page...

Atom-1	Atom-2	Interatomic distance (Å)	Clash overlap (Å)
1:A:606:PHE:HE1	1:A:633:PHE:CA	2.19	0.56
1:A:634:ASN:O	1:A:638:LEU:HB2	2.05	0.56
2:B:38:TYR:CE1	2:B:43:ASN:N	2.64	0.56
2:B:55:ASN:O	2:B:58:GLU:HB2	2.05	0.56
2:B:97:VAL:HG12	2:B:101:ILE:HD11	1.87	0.56
2:B:144:ASP:N	2:B:179:LYS:CD	2.66	0.56
2:B:260:LEU:O	2:B:261:PRO:C	2.37	0.56
2:B:293:VAL:HA	2:B:299:LEU:CB	2.35	0.56
2:B:306:LEU:HD13	2:B:325:LEU:HG	1.88	0.56
2:B:340:ILE:HG13	2:B:373:LEU:CB	2.33	0.56
2:B:440:GLY:C	2:B:442:LEU:N	2.56	0.56
2:B:513:TRP:CG	2:B:551:LEU:HD21	2.40	0.56
2:B:566:ALA:HB1	2:B:581:TYR:HB3	1.87	0.56
3:S:53:THR:CB	3:S:69:ASN:N	2.67	0.56
4:M:235:LEU:HD13	4:M:306:LEU:HB3	1.86	0.56
4:M:260:LEU:HD23	4:M:449:VAL:HA	1.85	0.56
4:M:358:ILE:O	4:M:397:TRP:CE3	2.58	0.56
4:M:405:THR:CG2	4:M:406:GLY:N	2.68	0.56
1:A:80:TYR:HB2	1:A:82:PHE:CD2	2.41	0.56
1:A:95:MET:SD	1:A:107:TYR:HD1	2.27	0.56
1:A:110:ALA:O	1:A:111:SER:C	2.34	0.56
1:A:166:LEU:HD12	1:A:185:LEU:HD23	1.87	0.56
1:A:196:LEU:O	1:A:197:ARG:O	2.22	0.56
1:A:204:VAL:HG13	1:A:239:LEU:HD13	1.85	0.56
1:A:477:PHE:CZ	1:A:491:THR:HB	2.40	0.56
2:B:73:ASP:OD1	4:M:19:LEU:HD22	2.04	0.56
2:B:212:VAL:CG2	2:B:248:LEU:HD23	2.36	0.56
2:B:329:ALA:O	2:B:330:SER:C	2.33	0.56
2:B:481:LYS:C	2:B:483:PRO:HD3	2.25	0.56
3:S:16:LEU:HD13	3:S:129:GLU:HG2	1.88	0.56
4:M:5:PHE:CE2	4:M:20:LEU:HD11	2.38	0.56
4:M:66:PHE:HB3	4:M:77:LEU:HD11	1.88	0.56
1:A:92:LEU:HD23	1:A:95:MET:CE	2.33	0.56
1:A:128:LEU:HD13	1:A:150:LEU:CG	2.35	0.56
1:A:594:PHE:CZ	2:B:477:MET:HE1	2.40	0.56
2:B:83:PHE:HE1	2:B:87:VAL:HG21	1.69	0.56
2:B:123:LEU:HD22	2:B:138:ALA:HA	1.87	0.56
2:B:132:SER:HB2	2:B:169:VAL:CG2	2.36	0.56
2:B:252:LEU:HB3	2:B:302:PHE:CD1	2.40	0.56
2:B:497:LEU:HB2	2:B:511:ILE:HG21	1.88	0.56
3:S:8:PHE:CD1	3:S:84:TYR:O	2.58	0.56

Continued on next page...

Continued from previous page...

Atom-1	Atom-2	Interatomic distance (Å)	Clash overlap (Å)
4:M:223:HIS:CD2	4:M:478:ASN:CA	2.87	0.56
4:M:242:GLY:HA3	4:M:444:ALA:HB2	1.86	0.56
1:A:63:ASP:OD1	1:A:64:LEU:N	2.39	0.56
2:B:68:SER:HA	4:M:18:TYR:CE1	2.41	0.56
2:B:72:SER:HA	4:M:17:GLN:CD	2.18	0.56
2:B:325:LEU:CD1	2:B:339:PHE:CD1	2.88	0.56
2:B:560:ILE:O	2:B:563:PHE:N	2.35	0.56
3:S:1:MET:SD	3:S:20:TYR:CD2	2.99	0.56
3:S:55:PRO:O	3:S:58:LEU:HB2	2.06	0.56
4:M:121:ILE:HG23	4:M:125:PHE:CE1	2.41	0.56
4:M:270:PRO:N	4:M:302:TYR:CD2	2.74	0.56
1:A:213:SER:HB3	3:S:142:ILE:C	2.26	0.56
1:A:481:MET:HE2	1:A:518:CYS:HB3	1.88	0.56
2:B:347:VAL:HG11	2:B:381:PHE:CD1	2.40	0.56
2:B:349:MET:CG	4:M:305:ASP:HB2	2.33	0.56
3:S:55:PRO:HB3	3:S:71:GLU:HG3	1.88	0.56
4:M:256:VAL:O	4:M:289:THR:HA	2.05	0.56
1:A:88:ASN:HB2	1:A:120:ILE:CD1	2.36	0.56
1:A:499:ILE:HG12	1:A:512:LEU:HD23	1.88	0.56
1:A:638:LEU:HD11	2:B:557:SER:O	2.05	0.56
2:B:50:LEU:CG	2:B:62:ALA:HB2	2.36	0.56
2:B:124:GLN:OE1	2:B:153:ILE:CG2	2.53	0.56
2:B:400:SER:CB	2:B:435:SER:CB	2.56	0.56
3:S:8:PHE:CE1	3:S:84:TYR:O	2.59	0.56
4:M:214:LEU:C	4:M:467:TYR:HB3	2.26	0.56
4:M:360:LEU:CD1	4:M:433:VAL:HB	2.36	0.56
1:A:140:VAL:HA	1:A:177:ILE:CG1	2.36	0.56
1:A:200:PHE:HZ	1:A:236:LEU:CD2	2.14	0.56
1:A:353:ASP:OD1	1:A:378:ILE:HD12	2.05	0.56
1:A:485:PRO:O	1:A:488:ARG:HG3	2.06	0.56
1:A:513:ARG:CG	1:A:547:VAL:HG22	2.36	0.56
1:A:638:LEU:CD2	2:B:561:ASP:CB	2.83	0.56
2:B:109:ALA:C	2:B:111:ASN:N	2.55	0.56
2:B:256:CYS:SG	2:B:299:LEU:HD21	2.45	0.56
2:B:304:GLN:O	2:B:307:ASN:HB2	2.06	0.56
2:B:475:ILE:CG2	2:B:489:ILE:HG22	2.34	0.56
3:S:53:THR:C	3:S:69:ASN:HB2	2.26	0.56
4:M:20:LEU:C	4:M:21:GLY:O	2.37	0.56
4:M:74:TYR:CG	4:M:114:ILE:HD11	2.40	0.56
4:M:347:PHE:CD2	4:M:350:VAL:HB	2.41	0.56
1:A:329:ASN:H	3:S:50:PHE:HE2	1.48	0.56

Continued on next page...

Continued from previous page...

Atom-1	Atom-2	Interatomic distance (Å)	Clash overlap (Å)
1:A:505:ASN:O	1:A:506:LYS:C	2.38	0.56
1:A:606:PHE:CE1	1:A:633:PHE:CA	2.88	0.56
2:B:73:ASP:OD1	4:M:19:LEU:CG	2.53	0.56
2:B:79:VAL:C	2:B:108:PHE:CE2	2.75	0.56
2:B:106:LEU:HG	4:M:130:GLU:CD	2.25	0.56
2:B:596:LEU:HA	2:B:611:ALA:HB1	1.88	0.56
3:S:49:SER:O	3:S:77:TYR:N	2.39	0.56
4:M:15:ILE:CG2	4:M:114:ILE:CG2	2.79	0.56
4:M:462:LYS:O	4:M:464:THR:N	2.31	0.56
1:A:220:SER:O	1:A:223:CYS:CB	2.48	0.55
1:A:566:PHE:O	1:A:569:ASP:O	2.23	0.55
1:A:582:ILE:HG12	1:A:607:LEU:HB2	1.88	0.55
2:B:162:VAL:CG2	2:B:199:LEU:CD1	2.77	0.55
2:B:189:HIS:CD2	2:B:222:HIS:CG	2.93	0.55
2:B:344:VAL:CG2	2:B:374:PHE:CE1	2.83	0.55
2:B:392:SER:HB3	2:B:424:PHE:HE1	1.71	0.55
2:B:476:ARG:HD2	2:B:513:TRP:CD1	2.41	0.55
4:M:319:SER:OG	4:M:345:GLU:N	2.39	0.55
4:M:434:SER:HB3	4:M:478:ASN:CG	2.27	0.55
1:A:84:MET:HB3	1:A:113:SER:CB	2.36	0.55
1:A:260:PHE:O	1:A:261:THR:O	2.21	0.55
1:A:594:PHE:CZ	2:B:477:MET:SD	2.93	0.55
2:B:12:LEU:HD22	4:M:13:LYS:HG3	1.88	0.55
2:B:67:ILE:CG2	4:M:18:TYR:OH	2.54	0.55
2:B:68:SER:O	4:M:18:TYR:HD1	1.88	0.55
2:B:83:PHE:CZ	2:B:105:LEU:HG	2.40	0.55
2:B:437:SER:HB2	2:B:474:VAL:HG22	1.88	0.55
2:B:534:ILE:HG21	2:B:594:ALA:CB	2.37	0.55
4:M:240:ILE:HB	4:M:304:VAL:CG1	2.35	0.55
4:M:323:MET:CB	4:M:340:LEU:HD11	2.30	0.55
1:A:403:LEU:HD23	1:A:422:GLU:HG3	1.87	0.55
1:A:581:LEU:CG	1:A:585:PHE:CZ	2.90	0.55
2:B:123:LEU:HD22	2:B:138:ALA:CA	2.36	0.55
2:B:399:LEU:HB3	2:B:411:ILE:HG23	1.88	0.55
4:M:69:ILE:CB	4:M:97:ASP:OD2	2.54	0.55
4:M:114:ILE:O	4:M:115:VAL:C	2.45	0.55
4:M:224:VAL:O	4:M:479:PHE:CB	2.54	0.55
4:M:327:PHE:HE1	4:M:336:ASP:CB	2.17	0.55
1:A:170:LEU:O	1:A:206:LYS:HD2	2.07	0.55
1:A:219:VAL:CG1	1:A:256:LEU:HD23	2.36	0.55
1:A:605:GLU:HB2	1:A:636:TYR:HE2	1.72	0.55

Continued on next page...

Continued from previous page...

Atom-1	Atom-2	Interatomic distance (Å)	Clash overlap (Å)
2:B:29:LYS:HG3	2:B:29:LYS:O	2.06	0.55
2:B:107:ARG:CZ	4:M:20:LEU:CD2	2.33	0.55
2:B:309:LEU:HD12	2:B:317:VAL:HG11	1.88	0.55
2:B:363:ILE:HD12	2:B:374:PHE:CD1	2.41	0.55
2:B:490:ILE:HG13	2:B:518:ILE:HG12	1.88	0.55
3:S:35:VAL:O	3:S:39:ILE:HG12	2.06	0.55
4:M:243:ILE:CG1	4:M:473:LYS:O	2.54	0.55
4:M:478:ASN:C	4:M:479:PHE:O	2.44	0.55
1:A:107:TYR:CE1	1:A:128:LEU:HD23	2.40	0.55
1:A:121:LEU:HD23	1:A:121:LEU:C	2.27	0.55
1:A:233:PHE:O	1:A:235:GLN:N	2.36	0.55
1:A:240:LEU:C	1:A:242:GLU:O	2.44	0.55
1:A:320:HIS:O	1:A:322:PHE:N	2.39	0.55
1:A:488:ARG:HD2	1:A:522:PHE:CD2	2.41	0.55
3:S:53:THR:CG2	3:S:67:GLU:O	2.55	0.55
4:M:45:SER:O	4:M:75:TRP:HZ2	1.89	0.55
4:M:253:ASN:N	4:M:254:PRO:CD	2.69	0.55
4:M:316:ARG:HG3	4:M:322:LEU:CD1	2.36	0.55
4:M:424:PHE:CZ	4:M:428:VAL:CG2	2.89	0.55
4:M:424:PHE:HZ	4:M:428:VAL:HG23	1.70	0.55
1:A:121:LEU:HG	1:A:158:LEU:HD22	1.87	0.55
1:A:132:LEU:O	1:A:133:LYS:C	2.43	0.55
1:A:477:PHE:CE2	1:A:491:THR:HB	2.42	0.55
1:A:625:LEU:CD1	1:A:629:LEU:HB2	2.36	0.55
2:B:12:LEU:HB3	4:M:13:LYS:CE	2.36	0.55
2:B:37:TYR:O	2:B:38:TYR:C	2.44	0.55
2:B:317:VAL:O	2:B:321:CYS:SG	2.59	0.55
2:B:377:TYR:O	2:B:380:LYS:CB	2.53	0.55
4:M:69:ILE:HD12	4:M:94:GLU:N	2.21	0.55
1:A:275:LEU:O	1:A:277:LYS:N	2.38	0.55
1:A:405:THR:O	2:B:7:ARG:NH2	2.39	0.55
1:A:405:THR:C	2:B:7:ARG:NH2	2.58	0.55
2:B:5:ILE:HD13	4:M:42:LEU:CD1	2.25	0.55
2:B:64:LYS:HZ2	4:M:120:ARG:HH12	1.45	0.55
2:B:219:TYR:CE2	2:B:226:LEU:CA	2.77	0.55
2:B:347:VAL:CB	2:B:381:PHE:HE1	2.13	0.55
2:B:390:VAL:HG12	2:B:394:TRP:CD1	2.41	0.55
2:B:418:TYR:CZ	2:B:432:ALA:CB	2.88	0.55
3:S:38:LEU:HB3	3:S:51:LEU:HD13	1.89	0.55
4:M:65:TYR:CD2	4:M:86:PRO:HA	2.42	0.55
4:M:69:ILE:HG12	4:M:90:PHE:CE1	2.42	0.55

Continued on next page...

Continued from previous page...

Atom-1	Atom-2	Interatomic distance (Å)	Clash overlap (Å)
4:M:92:PHE:CE2	4:M:128:CYS:CB	2.90	0.55
4:M:258:VAL:CG1	4:M:449:VAL:HG22	2.37	0.55
1:A:158:LEU:HG	1:A:162:ILE:HD11	1.87	0.55
1:A:180:LYS:HZ3	3:S:137:GLN:HB2	1.71	0.55
1:A:185:LEU:CB	1:A:203:PHE:CE1	2.89	0.55
1:A:203:PHE:CE2	1:A:221:VAL:HG11	2.41	0.55
1:A:244:LEU:HD13	1:A:256:LEU:HB2	1.87	0.55
2:B:185:LYS:CE	2:B:221:ASP:OD2	2.54	0.55
2:B:276:SER:C	2:B:295:ASN:CB	2.72	0.55
2:B:308:CYS:O	2:B:311:TYR:N	2.40	0.55
2:B:472:VAL:HG11	2:B:510:GLY:C	2.26	0.55
3:S:48:SER:HG	3:S:50:PHE:N	2.00	0.55
4:M:244:VAL:CB	4:M:472:TYR:CE2	2.90	0.55
4:M:305:ASP:O	4:M:307:SER:N	2.39	0.55
4:M:386:PHE:HB2	4:M:397:TRP:CD1	2.42	0.55
1:A:104:ARG:HG3	1:A:145:ILE:HG13	1.89	0.55
1:A:245:VAL:O	1:A:245:VAL:HG22	2.07	0.55
2:B:318:ILE:CD1	2:B:346:THR:HG23	2.37	0.55
2:B:418:TYR:HD1	2:B:424:PHE:CD2	2.25	0.55
2:B:453:TRP:HA	2:B:453:TRP:CE3	2.42	0.55
2:B:518:ILE:O	2:B:518:ILE:CG1	2.54	0.55
3:S:15:ARG:CZ	3:S:122:ILE:HD11	2.37	0.55
3:S:53:THR:HG1	3:S:68:VAL:C	2.09	0.55
4:M:16:PHE:HD1	4:M:118:TYR:CE2	2.25	0.55
4:M:262:THR:C	4:M:264:GLY:N	2.44	0.55
4:M:316:ARG:HG2	4:M:322:LEU:HD13	1.88	0.55
1:A:244:LEU:HD23	1:A:277:LYS:HG3	1.88	0.55
1:A:408:ILE:HA	3:S:64:ASN:HB2	1.87	0.55
1:A:581:LEU:HB3	1:A:607:LEU:CD1	2.35	0.55
2:B:120:ILE:CD1	2:B:142:LEU:CD2	2.84	0.55
2:B:291:TYR:CD2	2:B:294:VAL:HB	2.42	0.55
2:B:346:THR:O	2:B:349:MET:HB2	2.06	0.55
2:B:563:PHE:CD1	2:B:584:SER:HB3	2.31	0.55
3:S:4:ALA:CA	3:S:18:LYS:O	2.50	0.55
4:M:220:GLU:CD	4:M:439:TYR:CD2	2.57	0.55
4:M:260:LEU:CD2	4:M:449:VAL:CG2	2.81	0.55
4:M:290:PHE:HZ	4:M:293:PRO:CG	2.20	0.55
1:A:292:TYR:CD1	1:A:292:TYR:C	2.79	0.54
1:A:516:ILE:HG21	1:A:551:LEU:HA	1.88	0.54
1:A:581:LEU:CD2	1:A:607:LEU:HD21	2.32	0.54
2:B:20:ARG:NH1	4:M:118:TYR:CA	2.57	0.54

Continued on next page...

Continued from previous page...

Atom-1	Atom-2	Interatomic distance (Å)	Clash overlap (Å)
2:B:171:GLY:HA3	2:B:207:VAL:HA	1.89	0.54
2:B:398:ILE:HG23	2:B:402:LEU:HD11	1.87	0.54
3:S:7:ILE:O	3:S:15:ARG:N	2.38	0.54
4:M:54:SER:HB2	4:M:66:PHE:HE2	1.71	0.54
4:M:219:LEU:CG	4:M:440:ILE:HG12	2.36	0.54
4:M:262:THR:HG23	4:M:265:ASN:O	2.07	0.54
4:M:435:LEU:O	4:M:437:TYR:CE1	2.60	0.54
1:A:102:GLN:HE22	3:S:166:LYS:NZ	2.05	0.54
1:A:373:GLU:HG2	1:A:427:LYS:NZ	2.22	0.54
1:A:436:CYS:HB3	1:A:476:GLN:NE2	2.22	0.54
1:A:484:VAL:O	1:A:486:SER:O	2.25	0.54
1:A:584:PHE:O	1:A:587:ASN:N	2.40	0.54
2:B:17:VAL:HG23	2:B:36:THR:HA	1.88	0.54
2:B:28:SER:HA	2:B:58:GLU:CG	2.37	0.54
2:B:38:TYR:CE2	2:B:43:ASN:H	1.61	0.54
2:B:537:PHE:CD1	2:B:598:LEU:HB3	2.42	0.54
2:B:537:PHE:HE2	2:B:545:ARG:HB3	1.67	0.54
2:B:563:PHE:CE2	2:B:588:ILE:HD12	2.42	0.54
3:S:17:VAL:HG21	3:S:19:PHE:CE1	2.39	0.54
3:S:50:PHE:O	3:S:51:LEU:HD23	2.07	0.54
3:S:60:SER:O	3:S:66:ASP:HB2	2.07	0.54
4:M:7:ILE:HG13	4:M:16:PHE:HD2	1.72	0.54
4:M:242:GLY:O	4:M:302:TYR:N	2.38	0.54
4:M:469:GLY:O	4:M:470:ALA:C	2.44	0.54
1:A:150:LEU:HB3	1:A:162:ILE:HD13	1.89	0.54
1:A:438:ALA:O	1:A:441:TYR:HD1	1.91	0.54
2:B:79:VAL:CG2	2:B:108:PHE:CE2	2.81	0.54
2:B:188:TYR:HB3	2:B:192:LEU:HD13	1.87	0.54
2:B:375:LEU:CG	2:B:404:ASN:HB2	2.37	0.54
2:B:453:TRP:HA	2:B:453:TRP:HE3	1.72	0.54
3:S:53:THR:CB	3:S:68:VAL:CA	2.82	0.54
4:M:45:SER:HB2	4:M:75:TRP:CE2	2.42	0.54
1:A:288:THR:CG2	3:S:96:LEU:HD21	2.27	0.54
1:A:462:GLN:HG3	4:M:59:ASP:CG	1.94	0.54
2:B:106:LEU:HB3	4:M:130:GLU:HG3	1.88	0.54
2:B:392:SER:HB3	2:B:424:PHE:CE1	2.42	0.54
2:B:408:VAL:CG1	2:B:446:TRP:HB3	2.38	0.54
2:B:439:CYS:C	2:B:441:GLN:N	2.60	0.54
2:B:578:PRO:CD	2:B:581:TYR:CD2	2.73	0.54
4:M:259:LYS:O	4:M:450:GLU:N	2.41	0.54
4:M:352:GLN:CG	4:M:440:ILE:HB	2.36	0.54

Continued on next page...

Continued from previous page...

Atom-1	Atom-2	Interatomic distance (Å)	Clash overlap (Å)
1:A:585:PHE:CD2	1:A:603:VAL:HG12	2.40	0.54
1:A:594:PHE:CZ	2:B:477:MET:CE	2.90	0.54
2:B:230:PHE:HD1	2:B:298:ASP:CB	2.18	0.54
2:B:310:ILE:CG1	2:B:321:CYS:HB2	2.38	0.54
2:B:522:GLU:C	2:B:524:LYS:H	2.09	0.54
4:M:51:LEU:HD22	4:M:68:VAL:HG11	1.88	0.54
4:M:325:LEU:HD23	4:M:325:LEU:O	2.07	0.54
4:M:436:GLU:HA	4:M:479:PHE:CD1	2.42	0.54
1:A:71:VAL:HG11	1:A:105:VAL:HG12	1.88	0.54
1:A:244:LEU:CD1	1:A:281:LEU:HD11	2.36	0.54
1:A:532:LEU:O	1:A:533:ILE:O	2.25	0.54
2:B:24:ALA:CB	2:B:32:GLU:CG	2.72	0.54
2:B:73:ASP:HA	2:B:75:ASP:OD1	2.08	0.54
2:B:73:ASP:HA	4:M:24:ALA:HB3	1.89	0.54
2:B:83:PHE:CE2	2:B:105:LEU:HA	2.43	0.54
2:B:97:VAL:HG12	2:B:101:ILE:HD12	1.89	0.54
2:B:191:GLU:C	2:B:193:LEU:H	2.11	0.54
2:B:313:SER:HB3	4:M:269:ILE:CA	2.36	0.54
2:B:351:GLU:CB	4:M:476:THR:HG22	2.32	0.54
2:B:374:PHE:O	2:B:374:PHE:CD2	2.60	0.54
4:M:96:ILE:HG23	4:M:125:PHE:HE1	1.61	0.54
4:M:213:GLU:CB	4:M:467:TYR:HB2	2.37	0.54
4:M:319:SER:O	4:M:323:MET:SD	2.65	0.54
1:A:488:ARG:HG2	1:A:522:PHE:CE2	2.43	0.54
1:A:606:PHE:HE1	1:A:633:PHE:N	2.06	0.54
2:B:293:VAL:HA	2:B:299:LEU:HB2	1.88	0.54
2:B:343:LEU:HD21	2:B:363:ILE:N	2.23	0.54
2:B:398:ILE:HG22	2:B:402:LEU:HD12	1.89	0.54
3:S:46:PHE:O	3:S:47:GLN:C	2.24	0.54
4:M:215:TYR:O	4:M:246:VAL:CG1	2.54	0.54
4:M:253:ASN:OD1	4:M:292:PRO:CD	2.56	0.54
4:M:279:ASN:ND2	4:M:283:PHE:CE2	2.76	0.54
1:A:323:CYS:HG	1:A:338:PHE:HE2	1.54	0.54
1:A:416:ILE:O	1:A:417:PRO:C	2.41	0.54
2:B:86:VAL:CG1	2:B:101:ILE:HG23	2.31	0.54
2:B:170:ARG:HH12	2:B:198:GLU:HG2	1.73	0.54
2:B:214:ALA:O	2:B:216:LYS:N	2.41	0.54
2:B:418:TYR:CD1	2:B:419:VAL:CA	2.91	0.54
2:B:430:ILE:HD13	2:B:466:SER:HB3	1.89	0.54
3:S:112:CYS:HB2	3:S:113:PHE:CE1	2.43	0.54
4:M:19:LEU:CD2	4:M:24:ALA:HB3	2.37	0.54

Continued on next page...

Continued from previous page...

Atom-1	Atom-2	Interatomic distance (Å)	Clash overlap (Å)
4:M:47:SER:C	4:M:49:ASP:H	2.06	0.54
4:M:137:SER:O	4:M:138:ASP:C	2.45	0.54
4:M:270:PRO:CA	4:M:302:TYR:HD2	2.21	0.54
4:M:272:LEU:HD12	4:M:272:LEU:N	2.22	0.54
1:A:365:VAL:HG11	3:S:63:ASN:HB3	1.90	0.54
1:A:455:MET:HG2	1:A:498:LEU:HD11	1.90	0.54
1:A:628:VAL:O	1:A:631:SER:OG	2.07	0.54
2:B:155:LEU:HD13	2:B:155:LEU:C	2.28	0.54
2:B:422:ALA:HB3	2:B:424:PHE:CZ	2.39	0.54
2:B:429:VAL:HG11	2:B:467:VAL:HG11	1.89	0.54
2:B:522:GLU:C	2:B:524:LYS:N	2.60	0.54
2:B:566:ALA:HB2	2:B:581:TYR:CG	2.42	0.54
3:S:8:PHE:CZ	3:S:84:TYR:HB3	2.41	0.54
3:S:57:LEU:HD23	3:S:66:ASP:HA	1.90	0.54
4:M:20:LEU:CD1	4:M:129:VAL:HG21	2.37	0.54
4:M:41:LEU:CD1	4:M:52:ASP:HB2	2.38	0.54
4:M:338:PHE:CE1	4:M:415:ILE:CG1	2.90	0.54
4:M:390:ILE:O	4:M:393:GLY:N	2.41	0.54
1:A:306:GLU:O	1:A:307:ASP:C	2.38	0.54
1:A:552:ILE:O	1:A:556:VAL:HG23	2.08	0.54
2:B:87:VAL:O	2:B:90:ILE:HG22	2.07	0.54
2:B:106:LEU:HD21	4:M:131:ALA:N	2.23	0.54
2:B:120:ILE:HG23	2:B:142:LEU:HD22	1.90	0.54
2:B:285:GLU:C	2:B:286:ILE:O	2.24	0.54
2:B:292:GLU:HG3	2:B:296:ASP:CB	2.37	0.54
2:B:340:ILE:HD11	2:B:366:LEU:HB3	1.90	0.54
2:B:549:LEU:HD22	2:B:607:ILE:O	2.07	0.54
4:M:100:LEU:HD11	4:M:121:ILE:HG23	1.90	0.54
4:M:270:PRO:CB	4:M:288:ILE:HD11	2.38	0.54
1:A:155:THR:O	1:A:158:LEU:HB3	2.08	0.53
1:A:312:ALA:O	1:A:315:CYS:HB2	2.08	0.53
2:B:16:LYS:NZ	4:M:111:ILE:HG13	2.12	0.53
2:B:154:ILE:O	2:B:157:THR:HB	2.08	0.53
2:B:398:ILE:O	2:B:399:LEU:C	2.46	0.53
2:B:482:ASN:N	2:B:483:PRO:HD3	2.23	0.53
2:B:494:ALA:HB2	2:B:515:PHE:CE2	2.44	0.53
2:B:534:ILE:O	2:B:535:GLN:C	2.46	0.53
2:B:562:ASN:ND2	2:B:580:TYR:HD2	2.06	0.53
3:S:58:LEU:HD22	3:S:70:ASN:HA	1.89	0.53
4:M:104:PHE:N	4:M:104:PHE:CD1	2.64	0.53
4:M:223:HIS:NE2	4:M:436:GLU:CB	2.71	0.53

Continued on next page...

Continued from previous page...

Atom-1	Atom-2	Interatomic distance (Å)	Clash overlap (Å)
4:M:323:MET:CE	4:M:437:TYR:HE2	2.21	0.53
1:A:223:CYS:HB2	1:A:259:LEU:HD12	1.90	0.53
1:A:332:TYR:CE2	1:A:336:ILE:CD1	2.91	0.53
1:A:562:TRP:O	1:A:566:PHE:N	2.41	0.53
1:A:638:LEU:O	2:B:558:TYR:HA	2.08	0.53
2:B:191:GLU:O	2:B:193:LEU:N	2.40	0.53
2:B:234:CYS:HB3	2:B:301:LEU:CB	2.37	0.53
2:B:254:LYS:O	2:B:257:LYS:N	2.41	0.53
2:B:330:SER:O	2:B:332:LEU:N	2.41	0.53
2:B:349:MET:HG2	4:M:305:ASP:CB	2.37	0.53
2:B:472:VAL:HG12	2:B:510:GLY:CA	2.36	0.53
4:M:45:SER:O	4:M:75:TRP:CZ2	2.61	0.53
4:M:218:LEU:HD21	4:M:449:VAL:HG23	1.91	0.53
1:A:264:SER:CB	1:A:271:ARG:CG	2.87	0.53
1:A:364:ASP:OD1	3:S:65:ASN:ND2	2.41	0.53
1:A:384:LEU:CD1	1:A:385:LYS:N	2.71	0.53
1:A:395:PHE:CD2	1:A:428:MET:CE	2.92	0.53
1:A:395:PHE:O	1:A:396:VAL:C	2.40	0.53
1:A:513:ARG:HG2	1:A:547:VAL:HA	1.90	0.53
2:B:38:TYR:CD1	2:B:38:TYR:N	2.74	0.53
2:B:132:SER:O	2:B:133:GLU:C	2.34	0.53
2:B:193:LEU:O	2:B:229:HIS:CE1	2.61	0.53
2:B:367:SER:HB3	2:B:401:THR:OG1	2.08	0.53
2:B:513:TRP:CD1	2:B:517:GLU:CG	2.91	0.53
2:B:523:PHE:O	2:B:524:LYS:C	2.42	0.53
3:S:6:LEU:HD21	3:S:36:TYR:CE2	2.43	0.53
3:S:68:VAL:O	3:S:75:ILE:HD12	2.08	0.53
4:M:240:ILE:CG2	4:M:444:ALA:O	2.55	0.53
4:M:347:PHE:CZ	4:M:350:VAL:CG1	2.63	0.53
1:A:174:ARG:O	1:A:177:ILE:HB	2.09	0.53
1:A:481:MET:CE	1:A:518:CYS:HB3	2.38	0.53
2:B:191:GLU:C	2:B:193:LEU:N	2.59	0.53
2:B:196:LEU:CB	2:B:215:TYR:OH	2.57	0.53
2:B:344:VAL:HG22	2:B:374:PHE:CD1	2.42	0.53
2:B:546:CYS:HA	2:B:607:ILE:HG12	1.91	0.53
4:M:3:LEU:HA	4:M:79:SER:O	2.08	0.53
4:M:5:PHE:O	4:M:18:TYR:N	2.42	0.53
4:M:222:PHE:C	4:M:479:PHE:HZ	2.05	0.53
4:M:224:VAL:CG2	4:M:306:LEU:CD1	2.86	0.53
4:M:300:LEU:HD12	4:M:300:LEU:C	2.29	0.53
1:A:338:PHE:HE1	1:A:352:PHE:CZ	2.26	0.53

Continued on next page...

Continued from previous page...

Atom-1	Atom-2	Interatomic distance (Å)	Clash overlap (Å)
2:B:20:ARG:HH12	4:M:118:TYR:HB3	0.71	0.53
2:B:29:LYS:HB3	2:B:58:GLU:OE2	2.09	0.53
2:B:172:GLU:O	2:B:174:ALA:N	2.41	0.53
2:B:364:HIS:ND1	2:B:397:GLN:HB3	2.24	0.53
2:B:437:SER:HA	2:B:474:VAL:HG13	1.83	0.53
2:B:469:ASP:CG	2:B:506:ASN:CB	2.67	0.53
2:B:513:TRP:NE1	2:B:517:GLU:HG2	2.23	0.53
3:S:35:VAL:CG1	3:S:75:ILE:HD13	2.39	0.53
4:M:5:PHE:CE2	4:M:92:PHE:CE1	2.97	0.53
4:M:6:TYR:N	4:M:77:LEU:O	2.33	0.53
4:M:15:ILE:CG2	4:M:115:VAL:HG23	2.34	0.53
4:M:71:LYS:O	4:M:74:TYR:CE2	2.61	0.53
4:M:78:ALA:HB2	4:M:93:LEU:HG	1.90	0.53
1:A:536:MET:HB3	1:A:555:LEU:HD21	1.89	0.53
2:B:178:ILE:HA	2:B:218:CYS:HB2	1.91	0.53
2:B:374:PHE:HZ	2:B:381:PHE:CG	2.13	0.53
2:B:566:ALA:O	2:B:574:ASN:HB3	2.08	0.53
4:M:12:ASN:OD1	4:M:42:LEU:HB3	2.09	0.53
4:M:93:LEU:O	4:M:96:ILE:HB	2.08	0.53
4:M:374:TYR:O	4:M:390:ILE:CD1	2.48	0.53
4:M:424:PHE:CZ	4:M:428:VAL:HG22	2.43	0.53
4:M:443:SER:CB	4:M:447:ILE:C	2.76	0.53
1:A:301:GLY:O	1:A:302:ASN:CB	2.53	0.53
1:A:446:ASP:CG	1:A:448:GLU:O	2.47	0.53
2:B:230:PHE:HZ	2:B:302:PHE:HB2	1.72	0.53
2:B:260:LEU:CD2	2:B:293:VAL:HG11	2.39	0.53
2:B:309:LEU:O	2:B:312:SER:HB3	2.08	0.53
2:B:310:ILE:CD1	2:B:321:CYS:CB	2.78	0.53
2:B:344:VAL:HG22	2:B:381:PHE:HE2	1.74	0.53
3:S:16:LEU:HA	3:S:125:TRP:HZ2	1.70	0.53
3:S:38:LEU:HB3	3:S:51:LEU:HD11	1.89	0.53
3:S:109:LEU:CD1	3:S:113:PHE:CD1	2.88	0.53
4:M:69:ILE:HD12	4:M:94:GLU:HA	1.90	0.53
4:M:92:PHE:HZ	4:M:125:PHE:O	1.91	0.53
4:M:217:ASP:O	4:M:472:TYR:CG	2.62	0.53
4:M:221:THR:CA	4:M:474:THR:OG1	2.55	0.53
4:M:262:THR:CG2	4:M:265:ASN:O	2.57	0.53
4:M:443:SER:HA	4:M:447:ILE:HG13	1.90	0.53
1:A:114:PHE:CE2	1:A:153:ILE:HA	2.39	0.53
1:A:149:GLY:O	1:A:152:THR:N	2.42	0.53
1:A:516:ILE:CG2	1:A:554:ALA:HB2	2.39	0.53

Continued on next page...

Continued from previous page...

Atom-1	Atom-2	Interatomic distance (Å)	Clash overlap (Å)
1:A:532:LEU:O	1:A:533:ILE:C	2.30	0.53
2:B:17:VAL:CB	2:B:35:TYR:CE2	2.91	0.53
2:B:127:LEU:HD22	2:B:157:THR:HG21	1.91	0.53
2:B:136:CYS:HB3	2:B:172:GLU:HG2	1.87	0.53
2:B:266:VAL:CG1	2:B:275:ARG:HB3	2.39	0.53
2:B:534:ILE:HG21	2:B:594:ALA:HB3	1.91	0.53
2:B:546:CYS:CA	2:B:607:ILE:HG12	2.38	0.53
2:B:577:ASN:O	2:B:578:PRO:C	2.39	0.53
4:M:245:ASP:N	4:M:472:TYR:CD2	2.71	0.53
4:M:261:ASN:CB	4:M:450:GLU:HG2	2.34	0.53
1:A:379:VAL:HG11	1:A:441:TYR:HH	1.74	0.53
1:A:566:PHE:HZ	1:A:618:THR:O	1.91	0.53
2:B:105:LEU:HB3	2:B:145:MET:CE	2.39	0.53
2:B:253:ILE:HD11	2:B:324:ALA:HB2	1.90	0.53
2:B:371:GLN:OE1	2:B:442:LEU:HD11	2.08	0.53
2:B:408:VAL:HB	2:B:446:TRP:CG	2.44	0.53
2:B:435:SER:C	2:B:437:SER:N	2.60	0.53
4:M:5:PHE:CE2	4:M:20:LEU:HD13	2.43	0.53
4:M:432:THR:CB	4:M:480:GLN:HG3	2.24	0.53
1:A:185:LEU:HD13	1:A:203:PHE:HE1	1.73	0.53
2:B:14:THR:OG1	2:B:40:GLN:CG	2.57	0.53
2:B:34:SER:O	2:B:37:TYR:CA	2.57	0.53
2:B:103:LEU:CB	4:M:126:ASN:HD22	1.95	0.53
2:B:151:ALA:HB1	2:B:188:TYR:CD2	2.43	0.53
2:B:174:ALA:HB3	2:B:211:ALA:HA	1.83	0.53
2:B:177:ILE:HB	2:B:196:LEU:HD21	1.91	0.53
2:B:293:VAL:HA	2:B:299:LEU:CG	2.37	0.53
3:S:131:VAL:O	3:S:135:ILE:HG13	2.08	0.53
4:M:217:ASP:C	4:M:472:TYR:CZ	2.82	0.53
4:M:229:LYS:HG2	4:M:230:LYS:HG3	1.90	0.53
4:M:317:MET:CB	4:M:320:ILE:O	2.56	0.53
1:A:68:THR:CB	3:S:166:LYS:CB	2.71	0.52
1:A:95:MET:CB	1:A:127:LEU:HD23	2.39	0.52
1:A:219:VAL:HG13	1:A:259:LEU:HD13	1.92	0.52
1:A:399:ASP:N	1:A:418:ILE:CG1	2.72	0.52
2:B:38:TYR:C	2:B:40:GLN:N	2.62	0.52
2:B:100:LEU:HD23	4:M:123:LEU:HD11	1.91	0.52
2:B:133:GLU:HA	2:B:168:MET:SD	2.49	0.52
2:B:267:ASP:N	2:B:276:SER:HB3	2.25	0.52
2:B:393:ILE:HG23	2:B:431:MET:CB	2.39	0.52
2:B:416:LYS:HG3	2:B:457:HIS:CE1	2.43	0.52

Continued on next page...

Continued from previous page...

Atom-1	Atom-2	Interatomic distance (Å)	Clash overlap (Å)
2:B:515:PHE:HA	2:B:518:ILE:HG22	1.92	0.52
3:S:131:VAL:HG22	3:S:153:VAL:CG2	2.36	0.52
4:M:51:LEU:CB	4:M:68:VAL:CB	2.87	0.52
4:M:96:ILE:CD1	4:M:125:PHE:CD1	2.83	0.52
4:M:224:VAL:O	4:M:479:PHE:CA	2.57	0.52
4:M:347:PHE:CE1	4:M:439:TYR:CD1	2.96	0.52
4:M:373:ALA:O	4:M:417:TYR:HA	2.08	0.52
4:M:428:VAL:O	4:M:430:LEU:HA	2.08	0.52
4:M:449:VAL:CG1	4:M:452:ILE:CG1	2.82	0.52
1:A:68:THR:CB	3:S:166:LYS:CD	2.87	0.52
1:A:104:ARG:HG3	1:A:145:ILE:CG1	2.39	0.52
1:A:104:ARG:HG3	1:A:145:ILE:HD12	1.90	0.52
1:A:301:GLY:O	1:A:302:ASN:HB3	2.09	0.52
2:B:35:TYR:C	2:B:37:TYR:N	2.63	0.52
2:B:37:TYR:O	2:B:40:GLN:C	2.48	0.52
2:B:89:ASN:C	2:B:101:ILE:HD11	2.29	0.52
2:B:236:ILE:O	2:B:239:GLN:HB2	2.09	0.52
2:B:259:TYR:HD1	2:B:261:PRO:CB	2.21	0.52
2:B:303:LEU:HD22	2:B:339:PHE:HZ	1.63	0.52
2:B:307:ASN:ND2	2:B:338:LYS:CB	2.72	0.52
2:B:328:LEU:O	2:B:330:SER:N	2.42	0.52
2:B:592:TYR:CD2	2:B:618:PHE:CE2	2.97	0.52
3:S:49:SER:O	3:S:77:TYR:CA	2.57	0.52
4:M:222:PHE:HA	4:M:240:ILE:HA	1.91	0.52
4:M:277:GLU:N	4:M:289:THR:O	2.38	0.52
4:M:374:TYR:HE1	4:M:393:GLY:CA	2.21	0.52
1:A:326:GLN:O	1:A:328:PRO:HD3	2.09	0.52
1:A:409:VAL:HG22	1:A:410:TYR:N	2.25	0.52
2:B:14:THR:HB	2:B:40:GLN:HE21	1.74	0.52
2:B:143:SER:O	2:B:179:LYS:HD3	2.06	0.52
2:B:158:VAL:CG1	2:B:177:ILE:HD11	2.39	0.52
2:B:302:PHE:HE1	2:B:306:LEU:HD21	1.74	0.52
2:B:374:PHE:CZ	2:B:398:ILE:HG21	2.44	0.52
2:B:429:VAL:O	2:B:433:VAL:HG23	2.09	0.52
2:B:437:SER:CB	2:B:474:VAL:CG1	2.40	0.52
2:B:537:PHE:CZ	2:B:545:ARG:HG2	2.45	0.52
3:S:49:SER:O	3:S:77:TYR:CB	2.57	0.52
4:M:6:TYR:HD2	4:M:17:GLN:CG	2.19	0.52
4:M:48:ASP:O	4:M:70:ASN:CB	2.44	0.52
1:A:69:ASN:OD1	3:S:166:LYS:O	2.27	0.52
1:A:92:LEU:HD13	1:A:123:LEU:HD13	1.91	0.52

Continued on next page...

Continued from previous page...

Atom-1	Atom-2	Interatomic distance (Å)	Clash overlap (Å)
2:B:24:ALA:O	2:B:27:THR:OG1	2.25	0.52
2:B:25:VAL:CG2	2:B:30:LEU:O	2.33	0.52
2:B:195:ILE:C	2:B:197:LYS:N	2.60	0.52
2:B:215:TYR:HB3	2:B:226:LEU:CD1	2.40	0.52
2:B:383:VAL:O	2:B:383:VAL:HG13	2.09	0.52
2:B:527:PRO:HB3	2:B:587:ARG:CG	2.38	0.52
2:B:553:ALA:O	2:B:556:LEU:HB2	2.09	0.52
4:M:267:ILE:HG23	4:M:445:SER:OG	2.10	0.52
1:A:185:LEU:CD1	1:A:203:PHE:HE1	2.22	0.52
1:A:516:ILE:HD13	1:A:551:LEU:HB2	1.91	0.52
2:B:178:ILE:HD11	2:B:215:TYR:CA	2.37	0.52
2:B:212:VAL:C	2:B:214:ALA:N	2.57	0.52
2:B:230:PHE:CZ	2:B:302:PHE:HB2	2.45	0.52
2:B:234:CYS:CB	2:B:301:LEU:CB	2.88	0.52
2:B:239:GLN:HA	4:M:279:ASN:C	2.30	0.52
2:B:252:LEU:O	2:B:328:LEU:HD21	2.10	0.52
2:B:293:VAL:O	2:B:299:LEU:HD11	1.94	0.52
2:B:310:ILE:HG22	2:B:342:ALA:CB	2.31	0.52
2:B:333:GLN:O	2:B:336:ASN:N	2.33	0.52
4:M:245:ASP:O	4:M:472:TYR:OH	2.28	0.52
4:M:333:LYS:NZ	4:M:334:ASP:OD2	2.32	0.52
4:M:360:LEU:HD13	4:M:433:VAL:HB	1.91	0.52
4:M:435:LEU:HB2	4:M:437:TYR:HE1	1.71	0.52
1:A:64:LEU:HA	1:A:102:GLN:HE22	1.73	0.52
1:A:395:PHE:CE2	1:A:420:ILE:CD1	2.92	0.52
1:A:462:GLN:CD	4:M:60:LEU:N	2.63	0.52
2:B:35:TYR:O	2:B:37:TYR:N	2.42	0.52
2:B:176:ALA:C	2:B:178:ILE:N	2.62	0.52
2:B:253:ILE:CG1	2:B:324:ALA:CA	2.82	0.52
2:B:294:VAL:CA	2:B:299:LEU:HD12	2.39	0.52
2:B:318:ILE:HD12	2:B:346:THR:CG2	2.40	0.52
2:B:456:ASP:O	2:B:460:SER:OG	2.28	0.52
4:M:5:PHE:CD2	4:M:20:LEU:HD11	2.44	0.52
4:M:71:LYS:CG	4:M:74:TYR:OH	2.57	0.52
4:M:342:LEU:HD12	4:M:342:LEU:N	2.24	0.52
4:M:356:LEU:HD23	4:M:356:LEU:O	2.07	0.52
2:B:14:THR:CB	2:B:40:GLN:CG	2.88	0.52
2:B:16:LYS:NZ	4:M:111:ILE:HB	2.25	0.52
2:B:29:LYS:HB3	2:B:58:GLU:OE1	2.09	0.52
2:B:120:ILE:HD12	2:B:142:LEU:HD23	1.89	0.52
2:B:143:SER:O	2:B:145:MET:N	2.42	0.52

Continued on next page...

Continued from previous page...

Atom-1	Atom-2	Interatomic distance (Å)	Clash overlap (Å)
2:B:209:SER:O	2:B:212:VAL:HB	2.09	0.52
2:B:249:ILE:HG22	2:B:324:ALA:HB2	1.91	0.52
2:B:302:PHE:CE1	2:B:306:LEU:HD11	2.45	0.52
2:B:313:SER:OG	4:M:269:ILE:HG22	2.10	0.52
2:B:318:ILE:HD12	2:B:346:THR:HG23	1.91	0.52
3:S:1:MET:SD	3:S:20:TYR:CE2	3.03	0.52
4:M:288:ILE:HD12	4:M:300:LEU:HD23	1.92	0.52
4:M:334:ASP:O	4:M:416:GLU:CA	2.57	0.52
1:A:258:LYS:NZ	3:S:94:SER:N	2.58	0.52
1:A:536:MET:SD	1:A:551:LEU:HD12	2.50	0.52
1:A:566:PHE:CD1	1:A:566:PHE:C	2.83	0.52
2:B:83:PHE:HE1	2:B:87:VAL:CG2	2.20	0.52
2:B:267:ASP:HB3	2:B:289:PRO:HG3	1.90	0.52
2:B:318:ILE:HG21	2:B:346:THR:HB	1.91	0.52
2:B:454:LEU:O	2:B:457:HIS:HB2	2.09	0.52
4:M:2:TYR:CZ	4:M:64:LYS:NZ	2.75	0.52
4:M:390:ILE:C	4:M:393:GLY:H	2.13	0.52
4:M:458:LEU:HD13	4:M:464:THR:CG2	2.39	0.52
1:A:451:ASN:OD1	1:A:480:LEU:HD12	2.10	0.52
1:A:536:MET:HG2	1:A:551:LEU:HD13	1.91	0.52
1:A:563:CYS:O	1:A:566:PHE:CD2	2.62	0.52
2:B:14:THR:OG1	2:B:40:GLN:NE2	2.43	0.52
2:B:170:ARG:HG2	2:B:199:LEU:HD23	1.92	0.52
2:B:375:LEU:HB2	2:B:376:PRO:CD	2.40	0.52
2:B:566:ALA:C	2:B:574:ASN:ND2	2.63	0.52
3:S:105:PHE:CZ	3:S:128:LEU:HD11	2.45	0.52
4:M:214:LEU:O	4:M:214:LEU:CD2	2.49	0.52
4:M:248:SER:C	4:M:249:TYR:CD1	2.84	0.52
4:M:275:CYS:HG	4:M:293:PRO:HB3	1.68	0.52
4:M:353:VAL:CG2	4:M:438:SER:O	2.56	0.52
4:M:379:LEU:HD13	4:M:397:TRP:NE1	2.23	0.52
1:A:226:SER:O	1:A:227:LYS:C	2.45	0.52
1:A:384:LEU:HB2	1:A:441:TYR:HE2	1.75	0.52
1:A:441:TYR:O	1:A:442:SER:C	2.46	0.52
2:B:22:ALA:N	2:B:33:SER:N	2.56	0.52
2:B:127:LEU:HD22	2:B:157:THR:CG2	2.40	0.52
2:B:176:ALA:O	2:B:178:ILE:N	2.43	0.52
2:B:256:CYS:O	2:B:258:GLN:N	2.42	0.52
3:S:83:LEU:HD13	3:S:121:LEU:HD22	1.90	0.52
4:M:20:LEU:O	4:M:21:GLY:O	2.28	0.52
4:M:65:TYR:CE2	4:M:66:PHE:O	2.62	0.52

Continued on next page...

Continued from previous page...

Atom-1	Atom-2	Interatomic distance (Å)	Clash overlap (Å)
4:M:74:TYR:CD2	4:M:109:LEU:HB2	2.45	0.52
4:M:213:GLU:HB3	4:M:467:TYR:HB2	1.92	0.52
4:M:221:THR:HG22	4:M:223:HIS:CE1	2.45	0.52
4:M:272:LEU:HD22	4:M:278:ILE:HB	1.90	0.52
1:A:104:ARG:HG3	1:A:145:ILE:CD1	2.40	0.51
1:A:140:VAL:HA	1:A:177:ILE:HG12	1.92	0.51
1:A:406:GLY:O	3:S:64:ASN:CG	2.45	0.51
1:A:516:ILE:HG22	1:A:554:ALA:CB	2.39	0.51
1:A:533:ILE:HG12	1:A:562:TRP:HH2	1.71	0.51
1:A:544:SER:OG	1:A:547:VAL:HG23	2.10	0.51
1:A:585:PHE:CD2	1:A:603:VAL:CG1	2.92	0.51
2:B:63:MET:HB3	2:B:100:LEU:HD13	1.92	0.51
2:B:63:MET:HG3	2:B:100:LEU:HB3	1.92	0.51
2:B:107:ARG:NH2	4:M:18:TYR:CZ	2.79	0.51
2:B:215:TYR:CD1	2:B:219:TYR:CE2	2.81	0.51
2:B:215:TYR:O	2:B:219:TYR:HD2	1.93	0.51
2:B:279:LEU:HB3	2:B:280:PRO:HD2	1.91	0.51
2:B:416:LYS:HG3	2:B:457:HIS:NE2	2.25	0.51
2:B:440:GLY:C	2:B:442:LEU:H	2.12	0.51
2:B:493:LEU:CD2	2:B:514:LEU:HD23	2.39	0.51
2:B:585:GLY:O	2:B:589:SER:N	2.40	0.51
3:S:53:THR:HG22	3:S:54:PRO:O	2.10	0.51
3:S:127:THR:HG21	3:S:153:VAL:CG1	2.39	0.51
4:M:20:LEU:HD22	4:M:129:VAL:HB	1.90	0.51
4:M:250:LEU:CD1	4:M:254:PRO:CG	2.88	0.51
4:M:340:LEU:HG	4:M:342:LEU:HD11	1.91	0.51
4:M:379:LEU:HB3	4:M:411:LEU:HD11	1.91	0.51
1:A:162:ILE:O	1:A:165:ASP:N	2.44	0.51
1:A:274:LEU:O	1:A:275:LEU:O	2.28	0.51
1:A:438:ALA:O	1:A:439:ASP:CB	2.53	0.51
2:B:70:MET:HE1	2:B:107:ARG:HB2	1.88	0.51
2:B:178:ILE:HG23	2:B:218:CYS:H	1.76	0.51
2:B:227:HIS:CE1	2:B:292:GLU:HG2	2.46	0.51
2:B:253:ILE:HG12	2:B:324:ALA:HB1	1.92	0.51
2:B:563:PHE:CE1	2:B:584:SER:CA	2.93	0.51
3:S:1:MET:N	3:S:93:GLU:HB2	2.25	0.51
3:S:8:PHE:N	3:S:8:PHE:HD1	2.04	0.51
3:S:87:PHE:HB2	3:S:102:ILE:HD11	1.91	0.51
4:M:218:LEU:HG	4:M:244:VAL:CG2	2.38	0.51
1:A:496:ILE:HD11	1:A:519:LEU:CD1	2.40	0.51
2:B:38:TYR:HD1	2:B:38:TYR:N	2.09	0.51

Continued on next page...

Continued from previous page...

Atom-1	Atom-2	Interatomic distance (Å)	Clash overlap (Å)
2:B:181:TYR:CD1	2:B:222:HIS:CD2	2.89	0.51
2:B:284:ASN:O	2:B:284:ASN:CG	2.47	0.51
2:B:343:LEU:HD22	2:B:366:LEU:HD11	1.90	0.51
2:B:439:CYS:C	2:B:441:GLN:H	2.14	0.51
2:B:565:GLN:CB	2:B:581:TYR:CZ	2.93	0.51
4:M:424:PHE:HZ	4:M:428:VAL:CG2	2.23	0.51
1:A:249:ASN:HB3	1:A:252:ILE:HD12	1.91	0.51
1:A:253:ILE:HG23	1:A:281:LEU:HD13	1.92	0.51
2:B:234:CYS:CB	2:B:301:LEU:HB3	2.40	0.51
2:B:307:ASN:OD1	2:B:339:PHE:HD1	1.92	0.51
2:B:348:THR:HG23	2:B:380:LYS:HB3	1.86	0.51
2:B:404:ASN:O	2:B:405:GLU:O	2.29	0.51
2:B:437:SER:CB	2:B:474:VAL:HA	2.40	0.51
2:B:483:PRO:HA	2:B:486:HIS:CB	2.40	0.51
2:B:566:ALA:C	2:B:574:ASN:HB3	2.30	0.51
4:M:65:TYR:OH	4:M:86:PRO:HB3	2.08	0.51
4:M:95:THR:O	4:M:99:ILE:HG13	2.09	0.51
4:M:235:LEU:HD22	4:M:307:SER:HA	1.92	0.51
1:A:88:ASN:CB	1:A:120:ILE:CG2	2.89	0.51
1:A:509:PRO:HA	1:A:512:LEU:HD12	1.92	0.51
1:A:509:PRO:HB3	1:A:547:VAL:HG21	1.92	0.51
2:B:279:LEU:CD1	2:B:285:GLU:HG2	2.40	0.51
2:B:556:LEU:HA	2:B:588:ILE:HD12	1.84	0.51
2:B:563:PHE:O	2:B:566:ALA:CA	2.58	0.51
4:M:55:MET:O	4:M:56:VAL:O	2.28	0.51
4:M:80:THR:HG21	4:M:89:CYS:HB2	1.93	0.51
4:M:222:PHE:CE2	4:M:240:ILE:HD13	2.45	0.51
4:M:327:PHE:CE1	4:M:336:ASP:CB	2.89	0.51
4:M:357:LYS:O	4:M:436:GLU:HG2	2.10	0.51
1:A:91:ILE:O	1:A:94:VAL:HB	2.10	0.51
1:A:129:LYS:HG2	1:A:165:ASP:OD2	2.10	0.51
1:A:288:THR:O	1:A:291:ILE:HB	2.10	0.51
1:A:373:GLU:CG	1:A:427:LYS:CE	2.89	0.51
2:B:12:LEU:CB	4:M:13:LYS:HG2	2.21	0.51
2:B:13:ASP:OD1	4:M:14:LEU:CB	2.59	0.51
2:B:162:VAL:O	2:B:163:THR:C	2.46	0.51
2:B:170:ARG:CA	2:B:199:LEU:HD22	2.40	0.51
2:B:204:ASP:HB3	2:B:207:VAL:HG23	1.91	0.51
2:B:306:LEU:HD22	2:B:321:CYS:HA	1.91	0.51
2:B:486:HIS:NE2	2:B:518:ILE:HB	2.25	0.51
2:B:563:PHE:CE1	2:B:584:SER:HA	2.45	0.51

Continued on next page...

Continued from previous page...

Atom-1	Atom-2	Interatomic distance (Å)	Clash overlap (Å)
2:B:599:ALA:HB1	2:B:607:ILE:HG22	1.91	0.51
4:M:15:ILE:CG2	4:M:115:VAL:CG2	2.79	0.51
1:A:421:PRO:CB	3:S:63:ASN:N	2.70	0.51
1:A:504:ILE:O	1:A:505:ASN:O	2.28	0.51
1:A:514:GLU:HA	1:A:514:GLU:OE1	2.11	0.51
1:A:566:PHE:C	1:A:568:GLU:N	2.57	0.51
2:B:28:SER:CB	2:B:61:ASP:OD2	2.59	0.51
2:B:68:SER:O	4:M:18:TYR:CD1	2.63	0.51
2:B:144:ASP:CG	4:M:131:ALA:CA	2.45	0.51
2:B:217:GLU:O	2:B:218:CYS:C	2.48	0.51
2:B:340:ILE:HD13	2:B:366:LEU:HD13	1.91	0.51
2:B:344:VAL:HG21	2:B:377:TYR:HB2	1.87	0.51
2:B:424:PHE:HD1	2:B:428:VAL:HG11	1.74	0.51
2:B:474:VAL:C	2:B:476:ARG:N	2.64	0.51
4:M:18:TYR:CD1	4:M:19:LEU:N	2.78	0.51
4:M:290:PHE:CZ	4:M:297:PHE:CE2	2.99	0.51
1:A:217:ALA:CB	3:S:140:MET:SD	2.98	0.51
1:A:241:TYR:C	1:A:242:GLU:O	2.27	0.51
2:B:307:ASN:OD1	2:B:339:PHE:CD1	2.64	0.51
4:M:343:ASN:ND2	4:M:343:ASN:H	2.04	0.51
1:A:275:LEU:HA	1:A:278:ILE:CG1	2.41	0.51
1:A:316:LEU:HD11	1:A:341:ILE:HG21	1.92	0.51
1:A:398:GLU:CA	1:A:418:ILE:HG13	2.41	0.51
1:A:485:PRO:C	1:A:486:SER:O	2.42	0.51
1:A:503:ASN:OD1	4:M:60:LEU:CG	2.58	0.51
1:A:594:PHE:HZ	2:B:477:MET:HE1	1.74	0.51
2:B:38:TYR:CD1	2:B:42:ILE:CD1	2.91	0.51
2:B:517:GLU:C	2:B:519:ALA:H	2.14	0.51
1:A:158:LEU:CG	1:A:162:ILE:HD11	2.41	0.51
1:A:170:LEU:HD13	1:A:181:ALA:HB3	1.93	0.51
1:A:189:PHE:O	1:A:190:LEU:C	2.48	0.51
2:B:215:TYR:O	2:B:219:TYR:CD2	2.64	0.51
2:B:237:ILE:HD12	2:B:248:LEU:HB2	1.92	0.51
2:B:246:SER:O	2:B:249:ILE:HB	2.10	0.51
4:M:8:THR:HG22	4:M:14:LEU:HA	1.93	0.51
4:M:70:ASN:ND2	4:M:75:TRP:CZ2	2.79	0.51
4:M:212:ASN:HB3	4:M:250:LEU:HD23	1.93	0.51
4:M:276:VAL:HG21	4:M:299:LEU:HD12	1.92	0.51
4:M:280:ASP:OD2	4:M:282:VAL:HG23	2.10	0.51
1:A:450:TYR:OH	1:A:476:GLN:CD	2.48	0.50
2:B:197:LYS:CA	2:B:229:HIS:NE2	2.74	0.50

Continued on next page...

Continued from previous page...

Atom-1	Atom-2	Interatomic distance (Å)	Clash overlap (Å)
2:B:284:ASN:C	2:B:285:GLU:O	2.31	0.50
2:B:400:SER:HB2	2:B:435:SER:O	2.11	0.50
2:B:426:GLU:O	2:B:430:ILE:HG13	2.11	0.50
2:B:468:LEU:HD13	2:B:503:LEU:HD22	1.93	0.50
4:M:213:GLU:C	4:M:467:TYR:HB2	2.31	0.50
4:M:276:VAL:HG22	4:M:299:LEU:HD12	1.94	0.50
1:A:180:LYS:NZ	3:S:137:GLN:CB	2.75	0.50
1:A:251:TRP:HA	1:A:254:ILE:HD12	1.93	0.50
1:A:253:ILE:CD1	1:A:285:THR:HG21	2.41	0.50
1:A:258:LYS:HZ2	3:S:93:GLU:HA	1.63	0.50
2:B:44:PRO:HG3	2:B:77:ILE:CD1	2.42	0.50
2:B:64:LYS:HZ2	4:M:120:ARG:NH1	1.99	0.50
2:B:120:ILE:HG13	2:B:150:LEU:CD1	2.41	0.50
2:B:146:LYS:O	2:B:147:MET:SD	2.69	0.50
2:B:155:LEU:CB	2:B:188:TYR:CD1	2.93	0.50
2:B:231:ARG:C	2:B:233:TYR:N	2.62	0.50
2:B:334:MET:O	2:B:373:LEU:HD23	2.11	0.50
2:B:564:LYS:HE3	2:B:621:GLY:C	2.32	0.50
3:S:47:GLN:O	3:S:49:SER:N	2.44	0.50
3:S:109:LEU:O	3:S:111:ARG:N	2.44	0.50
4:M:323:MET:SD	4:M:342:LEU:CB	2.99	0.50
4:M:386:PHE:CB	4:M:397:TRP:CD1	2.94	0.50
1:A:64:LEU:CB	1:A:102:GLN:NE2	2.74	0.50
1:A:237:SER:N	1:A:238:PRO:CD	2.74	0.50
1:A:253:ILE:HD12	1:A:285:THR:HG21	1.93	0.50
1:A:288:THR:CG2	3:S:96:LEU:CD2	2.84	0.50
1:A:535:ILE:O	1:A:536:MET:HG3	2.11	0.50
2:B:5:ILE:CD1	4:M:39:PRO:CA	2.88	0.50
2:B:17:VAL:CG2	2:B:36:THR:HA	2.41	0.50
2:B:64:LYS:HD3	4:M:119:ASP:HB3	1.92	0.50
2:B:106:LEU:CD1	2:B:144:ASP:CA	2.78	0.50
2:B:108:PHE:HE1	2:B:112:ASP:CB	2.24	0.50
2:B:120:ILE:HD12	2:B:150:LEU:HD13	1.92	0.50
2:B:120:ILE:HD13	2:B:142:LEU:HD23	1.93	0.50
2:B:271:GLU:C	2:B:273:SER:N	2.54	0.50
2:B:336:ASN:C	2:B:373:LEU:HD22	2.24	0.50
2:B:437:SER:HB2	2:B:474:VAL:CA	2.40	0.50
2:B:580:TYR:CB	2:B:582:ASP:CG	2.75	0.50
3:S:14:PRO:HA	3:S:36:TYR:OH	2.11	0.50
3:S:125:TRP:CD1	3:S:129:GLU:HG3	2.46	0.50
4:M:278:ILE:O	4:M:278:ILE:HG12	2.12	0.50

Continued on next page...

Continued from previous page...

Atom-1	Atom-2	Interatomic distance (Å)	Clash overlap (Å)
1:A:254:ILE:CG2	3:S:94:SER:CB	2.90	0.50
1:A:429:VAL:HG11	1:A:473:ILE:HD11	1.92	0.50
1:A:582:ILE:CG1	1:A:607:LEU:HB2	2.41	0.50
1:A:589:SER:HA	1:A:597:GLN:CG	2.38	0.50
2:B:155:LEU:HB2	2:B:188:TYR:CE1	2.47	0.50
2:B:243:TRP:HH2	4:M:98:ARG:HD3	0.92	0.50
2:B:262:LYS:O	2:B:290:SER:HB2	2.12	0.50
2:B:537:PHE:CE2	2:B:545:ARG:CG	2.95	0.50
2:B:563:PHE:O	2:B:567:GLN:N	2.45	0.50
4:M:125:PHE:O	4:M:128:CYS:HB2	2.11	0.50
4:M:235:LEU:HD23	4:M:235:LEU:O	2.12	0.50
4:M:304:VAL:HB	4:M:445:SER:CB	2.42	0.50
1:A:117:ASP:OD2	1:A:120:ILE:CG1	2.59	0.50
1:A:251:TRP:HE3	3:S:97:ALA:CA	1.91	0.50
1:A:268:PRO:HG3	1:A:271:ARG:HH21	1.76	0.50
1:A:581:LEU:HG	1:A:585:PHE:CZ	2.47	0.50
2:B:352:ASN:O	2:B:355:ASN:HB2	2.12	0.50
2:B:564:LYS:HG2	2:B:565:GLN:N	2.27	0.50
3:S:8:PHE:HZ	3:S:86:THR:HG1	1.46	0.50
3:S:57:LEU:HA	3:S:58:LEU:O	2.11	0.50
3:S:83:LEU:HD11	3:S:116:VAL:CG2	2.41	0.50
3:S:100:ASP:O	3:S:104:THR:OG1	2.18	0.50
4:M:136:VAL:CG1	4:M:137:SER:N	2.74	0.50
4:M:243:ILE:CG2	4:M:472:TYR:HB3	2.41	0.50
4:M:371:GLU:O	4:M:419:ASN:OD1	2.29	0.50
1:A:128:LEU:HB2	1:A:150:LEU:HD21	1.93	0.50
1:A:384:LEU:HD12	1:A:384:LEU:C	2.30	0.50
2:B:9:ALA:HB3	4:M:14:LEU:HD12	1.94	0.50
2:B:68:SER:O	2:B:71:ALA:HB3	2.12	0.50
2:B:178:ILE:HG13	2:B:214:ALA:HA	1.82	0.50
2:B:382:TYR:OH	2:B:410:GLU:HB2	2.12	0.50
2:B:400:SER:HB2	2:B:435:SER:CA	2.32	0.50
3:S:8:PHE:HE1	3:S:86:THR:N	2.09	0.50
4:M:242:GLY:N	4:M:444:ALA:CB	2.75	0.50
4:M:447:ILE:O	4:M:448:TYR:C	2.48	0.50
1:A:182:ILE:CG2	1:A:221:VAL:CG2	2.81	0.50
1:A:341:ILE:O	1:A:344:ILE:HB	2.11	0.50
1:A:426:ILE:CD1	1:A:466:ASP:OD2	2.58	0.50
1:A:429:VAL:HG11	1:A:473:ILE:CD1	2.41	0.50
1:A:488:ARG:CG	1:A:522:PHE:CE2	2.94	0.50
1:A:638:LEU:HD12	2:B:557:SER:OG	2.11	0.50

Continued on next page...

Continued from previous page...

Atom-1	Atom-2	Interatomic distance (Å)	Clash overlap (Å)
2:B:20:ARG:NH2	4:M:118:TYR:HB3	2.23	0.50
2:B:151:ALA:HB3	2:B:188:TYR:HE2	1.73	0.50
2:B:162:VAL:HG22	2:B:199:LEU:HD21	1.93	0.50
2:B:181:TYR:CG	2:B:218:CYS:SG	2.99	0.50
2:B:185:LYS:HE2	2:B:221:ASP:OD2	2.12	0.50
2:B:513:TRP:CA	2:B:551:LEU:CD2	2.83	0.50
1:A:92:LEU:HD13	1:A:123:LEU:CB	2.42	0.50
1:A:223:CYS:HB2	1:A:259:LEU:CD1	2.41	0.50
1:A:613:ALA:HB3	1:A:621:LEU:HD23	1.93	0.50
2:B:230:PHE:HB3	2:B:298:ASP:OD2	2.12	0.50
2:B:232:ARG:CG	2:B:236:ILE:HD11	2.33	0.50
2:B:568:VAL:C	2:B:574:ASN:ND2	2.65	0.50
2:B:596:LEU:O	2:B:599:ALA:N	2.32	0.50
3:S:3:HIS:O	3:S:19:PHE:HA	2.12	0.50
4:M:220:GLU:CG	4:M:439:TYR:CB	2.85	0.50
4:M:306:LEU:CG	4:M:317:MET:CE	2.90	0.50
4:M:316:ARG:O	4:M:317:MET:C	2.45	0.50
4:M:343:ASN:HB3	4:M:345:GLU:HG3	1.94	0.50
1:A:263:LEU:O	1:A:266:VAL:C	2.51	0.50
1:A:341:ILE:O	1:A:345:ASN:N	2.45	0.50
1:A:379:VAL:CG1	1:A:441:TYR:HH	2.24	0.50
1:A:433:ILE:HD13	1:A:472:LYS:O	2.12	0.50
1:A:516:ILE:HD13	1:A:551:LEU:CA	2.42	0.50
1:A:556:VAL:HG22	1:A:603:VAL:HG13	1.90	0.50
2:B:38:TYR:O	2:B:40:GLN:N	2.45	0.50
2:B:143:SER:HB2	2:B:179:LYS:CD	2.42	0.50
2:B:189:HIS:CE1	2:B:222:HIS:CG	2.99	0.50
2:B:241:ASP:OD1	2:B:243:TRP:HB2	2.12	0.50
2:B:476:ARG:O	2:B:480:GLN:HG3	2.11	0.50
2:B:479:VAL:CG2	2:B:486:HIS:HD2	2.09	0.50
2:B:565:GLN:HB2	2:B:581:TYR:CZ	2.46	0.50
4:M:68:VAL:HG23	4:M:77:LEU:HD12	1.94	0.50
4:M:364:VAL:CA	4:M:367:ALA:O	2.60	0.50
1:A:151:SER:O	1:A:153:ILE:N	2.45	0.49
1:A:158:LEU:CD1	1:A:162:ILE:HG13	2.42	0.49
1:A:254:ILE:HG12	1:A:290:VAL:HA	1.93	0.49
1:A:508:LEU:CD1	4:M:59:ASP:OD1	2.60	0.49
2:B:131:ASN:O	2:B:135:ARG:HG3	2.12	0.49
2:B:162:VAL:HG22	2:B:199:LEU:CD2	2.42	0.49
2:B:189:HIS:NE2	2:B:222:HIS:CG	2.80	0.49
2:B:333:GLN:O	2:B:336:ASN:CB	2.60	0.49

Continued on next page...

Continued from previous page...

Atom-1	Atom-2	Interatomic distance (Å)	Clash overlap (Å)
4:M:212:ASN:CG	4:M:250:LEU:HD23	2.32	0.49
4:M:223:HIS:CA	4:M:479:PHE:CG	2.92	0.49
4:M:379:LEU:HB3	4:M:411:LEU:CD1	2.42	0.49
1:A:119:ASP:O	1:A:123:LEU:HG	2.11	0.49
2:B:17:VAL:CG1	2:B:35:TYR:CD2	2.95	0.49
2:B:97:VAL:CG1	2:B:101:ILE:HD11	2.42	0.49
2:B:260:LEU:CD2	2:B:291:TYR:OH	2.54	0.49
2:B:279:LEU:N	2:B:288:TYR:CB	2.49	0.49
2:B:390:VAL:HA	2:B:393:ILE:HD12	1.94	0.49
4:M:51:LEU:N	4:M:51:LEU:HD12	2.28	0.49
4:M:92:PHE:CE2	4:M:128:CYS:HB3	2.47	0.49
4:M:223:HIS:CB	4:M:476:THR:OG1	2.60	0.49
4:M:293:PRO:CA	4:M:294:ASP:O	2.60	0.49
1:A:460:LEU:O	1:A:463:ASP:N	2.37	0.49
1:A:496:ILE:HG13	1:A:532:LEU:HD11	1.93	0.49
2:B:37:TYR:CD2	2:B:38:TYR:CE1	3.00	0.49
2:B:83:PHE:CD1	2:B:87:VAL:HG23	2.47	0.49
2:B:162:VAL:HG11	2:B:195:ILE:HG23	1.92	0.49
2:B:195:ILE:O	2:B:197:LYS:N	2.44	0.49
2:B:215:TYR:CZ	2:B:229:HIS:HB3	2.47	0.49
2:B:223:LEU:HD22	2:B:255:TYR:CD1	2.48	0.49
2:B:335:LYS:O	2:B:373:LEU:HD22	2.12	0.49
2:B:365:PHE:O	2:B:368:ILE:HB	2.12	0.49
2:B:497:LEU:CD1	2:B:503:LEU:HD12	2.41	0.49
2:B:563:PHE:CD2	2:B:588:ILE:HD12	2.47	0.49
3:S:135:ILE:O	3:S:141:VAL:CG2	2.59	0.49
4:M:212:ASN:CA	4:M:249:TYR:O	2.55	0.49
4:M:214:LEU:N	4:M:467:TYR:H	2.11	0.49
4:M:280:ASP:OD1	4:M:280:ASP:N	2.44	0.49
4:M:290:PHE:CD2	4:M:297:PHE:CZ	2.99	0.49
4:M:291:ILE:HG12	4:M:292:PRO:CD	2.42	0.49
1:A:244:LEU:O	1:A:245:VAL:C	2.47	0.49
2:B:6:HIS:NE2	4:M:77:LEU:HD21	2.27	0.49
2:B:9:ALA:C	4:M:14:LEU:HD13	2.32	0.49
2:B:14:THR:HA	2:B:36:THR:CA	2.26	0.49
2:B:25:VAL:HA	2:B:30:LEU:O	2.12	0.49
2:B:79:VAL:HG23	2:B:108:PHE:CD2	2.43	0.49
2:B:567:GLN:O	2:B:569:THR:HB	2.00	0.49
3:S:25:LEU:O	3:S:27:LYS:N	2.45	0.49
3:S:146:VAL:O	3:S:150:VAL:HG23	2.12	0.49
4:M:270:PRO:O	4:M:272:LEU:HD12	2.11	0.49

Continued on next page...

Continued from previous page...

Atom-1	Atom-2	Interatomic distance (Å)	Clash overlap (Å)
4:M:372:ILE:CD1	4:M:428:VAL:HG22	2.42	0.49
1:A:264:SER:HB3	1:A:271:ARG:CG	2.42	0.49
1:A:329:ASN:O	1:A:333:ILE:HG13	2.13	0.49
1:A:429:VAL:HG11	1:A:473:ILE:HG13	1.91	0.49
1:A:516:ILE:HG23	1:A:554:ALA:CB	2.37	0.49
1:A:588:LEU:C	1:A:590:TYR:N	2.63	0.49
2:B:309:LEU:CG	2:B:317:VAL:HG11	2.42	0.49
2:B:512:VAL:C	2:B:551:LEU:CD1	2.67	0.49
2:B:537:PHE:CZ	2:B:545:ARG:CG	2.96	0.49
2:B:564:LYS:CE	2:B:621:GLY:C	2.79	0.49
3:S:25:LEU:HA	3:S:28:GLN:CG	2.41	0.49
4:M:276:VAL:CG2	4:M:290:PHE:HD1	2.15	0.49
4:M:362:PHE:O	4:M:364:VAL:HG13	2.12	0.49
1:A:78:GLU:O	1:A:80:TYR:O	2.30	0.49
1:A:186:PHE:HE1	1:A:187:LYS:HD3	1.78	0.49
1:A:316:LEU:O	1:A:319:LEU:N	2.44	0.49
2:B:175:LEU:O	2:B:178:ILE:HB	2.13	0.49
2:B:216:LYS:HB2	2:B:251:LEU:CG	2.37	0.49
2:B:513:TRP:CZ2	2:B:554:LYS:NZ	2.79	0.49
4:M:219:LEU:N	4:M:472:TYR:CD2	2.79	0.49
1:A:64:LEU:CA	1:A:102:GLN:HE22	2.25	0.49
1:A:134:TYR:O	1:A:135:ASP:C	2.47	0.49
1:A:170:LEU:HD12	1:A:206:LYS:HG3	1.94	0.49
1:A:179:LYS:NZ	3:S:142:ILE:H	2.08	0.49
1:A:190:LEU:HD12	1:A:228:LYS:HG3	1.94	0.49
1:A:241:TYR:C	1:A:241:TYR:CD1	2.86	0.49
1:A:293:GLU:OE1	3:S:94:SER:CB	2.59	0.49
1:A:573:GLU:O	1:A:577:VAL:HG23	2.12	0.49
1:A:638:LEU:CD1	2:B:557:SER:CB	2.91	0.49
2:B:85:ASP:O	2:B:89:ASN:ND2	2.46	0.49
2:B:120:ILE:HG13	2:B:150:LEU:HD13	1.94	0.49
2:B:231:ARG:HG2	2:B:298:ASP:OD1	2.11	0.49
2:B:294:VAL:HA	2:B:299:LEU:CD1	2.43	0.49
2:B:483:PRO:O	2:B:486:HIS:HB3	2.13	0.49
3:S:131:VAL:CG2	3:S:153:VAL:CG2	2.84	0.49
4:M:226:PHE:CE2	4:M:321:GLY:C	2.86	0.49
4:M:323:MET:HG2	4:M:437:TYR:OH	2.13	0.49
1:A:136:GLY:O	1:A:139:ASP:HA	2.12	0.49
2:B:162:VAL:CG1	2:B:195:ILE:HG23	2.42	0.49
2:B:189:HIS:CE1	2:B:222:HIS:ND1	2.81	0.49
2:B:219:TYR:CG	2:B:226:LEU:CD2	2.83	0.49

Continued on next page...

Continued from previous page...

Atom-1	Atom-2	Interatomic distance (Å)	Clash overlap (Å)
2:B:325:LEU:O	2:B:326:TYR:C	2.38	0.49
2:B:412:PHE:HB3	2:B:453:TRP:CD1	2.48	0.49
2:B:430:ILE:HG13	2:B:467:VAL:HG22	1.94	0.49
4:M:340:LEU:HG	4:M:342:LEU:CD1	2.43	0.49
4:M:480:GLN:OE1	4:M:482:ARG:NE	2.38	0.49
1:A:206:LYS:C	1:A:208:ASP:N	2.65	0.49
1:A:261:THR:O	1:A:264:SER:OG	2.18	0.49
2:B:10:SER:O	2:B:40:GLN:NE2	2.45	0.49
2:B:278:PRO:CD	2:B:292:GLU:CA	2.90	0.49
2:B:430:ILE:HG23	2:B:470:ALA:HB2	1.94	0.49
2:B:486:HIS:NE2	2:B:490:ILE:HD11	2.27	0.49
2:B:494:ALA:O	2:B:498:THR:HG23	2.12	0.49
3:S:129:GLU:O	3:S:132:LEU:N	2.45	0.49
4:M:6:TYR:O	4:M:76:CYS:HA	2.13	0.49
4:M:67:SER:O	4:M:93:LEU:HD11	2.13	0.49
4:M:379:LEU:HD22	4:M:397:TRP:CE2	2.48	0.49
1:A:264:SER:HB2	1:A:271:ARG:CG	2.43	0.49
1:A:292:TYR:O	1:A:292:TYR:HD1	1.93	0.49
1:A:486:SER:O	1:A:487:MET:HB2	2.12	0.49
1:A:610:SER:OG	1:A:625:LEU:HD13	2.13	0.49
2:B:28:SER:OG	2:B:30:LEU:O	0.49	0.49
2:B:140:SER:O	2:B:143:SER:N	2.45	0.49
2:B:214:ALA:C	2:B:216:LYS:N	2.65	0.49
4:M:69:ILE:HD12	4:M:93:LEU:C	2.33	0.49
4:M:338:PHE:HZ	4:M:379:LEU:HD21	1.78	0.49
4:M:437:TYR:CE1	4:M:479:PHE:CD2	3.01	0.49
1:A:261:THR:O	1:A:264:SER:N	2.45	0.48
1:A:638:LEU:HD13	2:B:557:SER:CB	2.42	0.48
2:B:86:VAL:HG13	2:B:101:ILE:HG12	1.93	0.48
2:B:98:LYS:HZ2	2:B:134:LEU:HD22	1.78	0.48
2:B:178:ILE:CG1	2:B:214:ALA:O	2.53	0.48
2:B:275:ARG:HG2	2:B:294:VAL:HG11	1.95	0.48
2:B:295:ASN:O	2:B:296:ASP:O	2.30	0.48
3:S:1:MET:N	3:S:93:GLU:CD	2.66	0.48
3:S:24:ASP:HB3	3:S:26:PRO:HD2	1.93	0.48
4:M:44:ASP:CG	4:M:50:TYR:CE2	2.86	0.48
1:A:169:MET:C	1:A:171:ASN:N	2.65	0.48
1:A:185:LEU:HB2	1:A:203:PHE:CZ	2.47	0.48
1:A:190:LEU:CD1	1:A:228:LYS:HE3	2.37	0.48
1:A:581:LEU:CD2	1:A:585:PHE:CE2	2.96	0.48
3:S:53:THR:CG2	3:S:69:ASN:N	2.77	0.48

Continued on next page...

Continued from previous page...

Atom-1	Atom-2	Interatomic distance (Å)	Clash overlap (Å)
3:S:64:ASN:C	3:S:66:ASP:N	2.66	0.48
1:A:76:TYR:O	1:A:80:TYR:CD2	2.67	0.48
1:A:213:SER:HB2	3:S:142:ILE:CG2	2.44	0.48
1:A:441:TYR:C	1:A:443:SER:N	2.56	0.48
1:A:498:LEU:HB3	1:A:504:ILE:HG13	1.95	0.48
2:B:98:LYS:HZ1	2:B:134:LEU:HD22	1.78	0.48
2:B:212:VAL:HG13	2:B:251:LEU:HD23	1.95	0.48
2:B:267:ASP:O	2:B:268:LYS:C	2.46	0.48
3:S:25:LEU:HA	3:S:28:GLN:HG2	1.95	0.48
3:S:33:GLU:O	3:S:36:TYR:HB2	2.12	0.48
1:A:71:VAL:CG1	1:A:105:VAL:CG1	2.89	0.48
1:A:186:PHE:CE1	1:A:187:LYS:HD3	2.48	0.48
1:A:304:LEU:HD23	1:A:304:LEU:C	2.34	0.48
2:B:249:ILE:CG2	2:B:324:ALA:HB2	2.43	0.48
2:B:297:PRO:O	2:B:301:LEU:CB	2.61	0.48
2:B:325:LEU:O	2:B:334:MET:SD	2.71	0.48
2:B:366:LEU:O	2:B:368:ILE:N	2.46	0.48
2:B:425:PRO:HD2	2:B:428:VAL:HG21	1.95	0.48
2:B:556:LEU:O	2:B:588:ILE:CD1	2.61	0.48
2:B:586:SER:O	2:B:590:GLN:HG3	2.13	0.48
2:B:599:ALA:O	2:B:600:LYS:C	2.52	0.48
3:S:49:SER:C	3:S:77:TYR:HB2	2.33	0.48
4:M:4:SER:HA	4:M:18:TYR:O	2.14	0.48
4:M:6:TYR:HD2	4:M:17:GLN:HG3	1.76	0.48
4:M:54:SER:N	4:M:66:PHE:CD2	2.68	0.48
4:M:467:TYR:CG	4:M:468:LYS:N	2.82	0.48
1:A:140:VAL:O	1:A:141:VAL:O	2.30	0.48
2:B:20:ARG:NH1	4:M:118:TYR:N	2.61	0.48
2:B:92:THR:HG22	2:B:94:ASP:H	1.78	0.48
2:B:112:ASP:C	2:B:112:ASP:OD1	2.51	0.48
2:B:219:TYR:C	2:B:221:ASP:N	2.59	0.48
2:B:227:HIS:CG	2:B:298:ASP:OD2	2.67	0.48
2:B:307:ASN:OD1	2:B:339:PHE:HA	2.13	0.48
3:S:4:ALA:HB2	3:S:19:PHE:CD1	2.36	0.48
3:S:5:VAL:HG13	3:S:87:PHE:CE2	2.48	0.48
4:M:20:LEU:O	4:M:21:GLY:C	2.44	0.48
4:M:350:VAL:HA	4:M:442:GLN:CG	2.43	0.48
1:A:207:LEU:HD23	1:A:239:LEU:HB3	1.95	0.48
1:A:536:MET:CB	1:A:555:LEU:HD21	2.44	0.48
1:A:581:LEU:HD23	1:A:607:LEU:CG	2.43	0.48
2:B:120:ILE:HD11	2:B:145:MET:HG3	1.96	0.48

Continued on next page...

Continued from previous page...

Atom-1	Atom-2	Interatomic distance (Å)	Clash overlap (Å)
2:B:426:GLU:O	2:B:467:VAL:HG22	2.14	0.48
2:B:559:ASP:O	2:B:562:ASN:N	2.46	0.48
2:B:559:ASP:CA	2:B:562:ASN:HB2	2.40	0.48
3:S:54:PRO:O	3:S:69:ASN:CB	2.62	0.48
4:M:4:SER:OG	4:M:18:TYR:O	2.15	0.48
4:M:101:LEU:HG	4:M:106:LYS:HG3	1.94	0.48
4:M:222:PHE:CD1	4:M:222:PHE:N	2.79	0.48
4:M:336:ASP:OD2	4:M:415:ILE:HB	2.14	0.48
1:A:182:ILE:HG23	1:A:203:PHE:CE2	2.49	0.48
1:A:222:ILE:HD12	1:A:240:LEU:HD21	1.96	0.48
1:A:339:TYR:HB2	1:A:374:LEU:HD11	1.95	0.48
2:B:155:LEU:CG	2:B:188:TYR:HD1	2.26	0.48
4:M:8:THR:N	4:M:75:TRP:O	2.43	0.48
4:M:133:GLU:O	4:M:135:ASN:N	2.47	0.48
4:M:379:LEU:HD22	4:M:411:LEU:HD21	1.96	0.48
1:A:99:LYS:O	1:A:103:LYS:HG3	2.14	0.48
1:A:263:LEU:O	1:A:264:SER:C	2.48	0.48
1:A:264:SER:CB	1:A:271:ARG:HG3	2.43	0.48
1:A:454:ILE:HG23	1:A:473:ILE:HG23	1.96	0.48
1:A:528:ASN:O	1:A:530:ASN:N	2.46	0.48
2:B:83:PHE:HE2	2:B:105:LEU:HA	1.79	0.48
2:B:178:ILE:HG21	2:B:217:GLU:CB	2.32	0.48
2:B:261:PRO:CB	2:B:290:SER:HB3	2.32	0.48
2:B:312:SER:CA	4:M:269:ILE:CG1	2.92	0.48
2:B:399:LEU:O	2:B:403:ILE:HG23	2.14	0.48
2:B:497:LEU:CD2	2:B:511:ILE:HG21	2.44	0.48
3:S:58:LEU:HD12	3:S:58:LEU:HA	1.83	0.48
4:M:310:VAL:HG13	4:M:315:VAL:O	2.13	0.48
4:M:376:ILE:HD12	4:M:415:ILE:HG12	1.95	0.48
1:A:121:LEU:HG	1:A:153:ILE:HG21	1.96	0.48
1:A:436:CYS:CB	1:A:450:TYR:CE1	2.97	0.48
1:A:462:GLN:CG	4:M:59:ASP:OD1	2.60	0.48
1:A:484:VAL:HG12	1:A:486:SER:O	2.13	0.48
2:B:325:LEU:HD22	2:B:339:PHE:CE1	2.49	0.48
2:B:389:ILE:HG23	2:B:428:VAL:CG2	2.43	0.48
3:S:73:ILE:HG23	3:S:88:ILE:HG22	1.93	0.48
4:M:16:PHE:CD1	4:M:118:TYR:CE2	3.01	0.48
4:M:19:LEU:HD12	4:M:20:LEU:N	2.29	0.48
4:M:126:ASN:C	4:M:128:CYS:N	2.66	0.48
4:M:275:CYS:HB2	4:M:290:PHE:HE1	1.78	0.48
4:M:290:PHE:CZ	4:M:297:PHE:CD1	3.01	0.48

Continued on next page...

Continued from previous page...

Atom-1	Atom-2	Interatomic distance (Å)	Clash overlap (Å)
1:A:341:ILE:CG2	1:A:348:PHE:HD2	2.26	0.48
1:A:531:ASP:C	1:A:534:LYS:O	2.52	0.48
2:B:83:PHE:CE2	2:B:108:PHE:HB3	2.48	0.48
2:B:253:ILE:CD1	2:B:324:ALA:CA	2.91	0.48
2:B:522:GLU:O	2:B:524:LYS:N	2.47	0.48
4:M:45:SER:CA	4:M:75:TRP:CH2	2.97	0.48
4:M:45:SER:CB	4:M:75:TRP:CH2	2.94	0.48
4:M:354:ASP:O	4:M:438:SER:HB2	2.14	0.48
4:M:376:ILE:HD13	4:M:415:ILE:HG23	1.95	0.48
4:M:405:THR:O	4:M:406:GLY:C	2.48	0.48
1:A:162:ILE:O	1:A:165:ASP:HB2	2.14	0.47
1:A:449:TRP:O	1:A:453:VAL:HG23	2.14	0.47
1:A:609:LEU:HD23	1:A:628:VAL:HG11	1.96	0.47
2:B:83:PHE:CE1	2:B:87:VAL:HG23	2.47	0.47
2:B:291:TYR:HE2	2:B:294:VAL:HB	1.79	0.47
2:B:355:ASN:O	2:B:359:LEU:CD2	2.62	0.47
2:B:374:PHE:CE2	2:B:398:ILE:HG22	2.47	0.47
2:B:437:SER:HG	2:B:477:MET:HB2	1.78	0.47
3:S:51:LEU:HD12	3:S:77:TYR:CZ	2.49	0.47
4:M:217:ASP:OD1	4:M:471:LYS:HA	2.14	0.47
1:A:114:PHE:CG	1:A:153:ILE:HG23	2.42	0.47
1:A:250:ASN:O	1:A:254:ILE:HG13	2.14	0.47
2:B:25:VAL:CG2	2:B:31:GLY:CA	2.92	0.47
2:B:367:SER:OG	2:B:401:THR:CG2	2.61	0.47
2:B:405:GLU:O	2:B:446:TRP:CD1	2.67	0.47
2:B:572:GLU:C	2:B:574:ASN:N	2.66	0.47
4:M:51:LEU:CD2	4:M:68:VAL:HG21	2.44	0.47
4:M:218:LEU:N	4:M:472:TYR:CE2	2.82	0.47
4:M:233:LEU:HD22	4:M:324:SER:HA	1.95	0.47
4:M:245:ASP:N	4:M:472:TYR:CE2	2.82	0.47
4:M:386:PHE:CB	4:M:397:TRP:HD1	2.27	0.47
1:A:84:MET:HB3	1:A:113:SER:HB2	1.95	0.47
1:A:395:PHE:CZ	1:A:428:MET:CG	2.96	0.47
1:A:504:ILE:N	4:M:59:ASP:OD2	2.47	0.47
1:A:529:GLY:O	1:A:532:LEU:HB2	2.14	0.47
1:A:581:LEU:HD21	1:A:585:PHE:CE2	2.49	0.47
2:B:161:LEU:C	2:B:173:VAL:HG21	2.34	0.47
2:B:177:ILE:HD11	2:B:195:ILE:HG21	1.95	0.47
2:B:249:ILE:HG23	2:B:306:LEU:HD21	1.94	0.47
2:B:389:ILE:O	2:B:390:VAL:O	2.32	0.47
4:M:223:HIS:CA	4:M:479:PHE:CZ	2.94	0.47

Continued on next page...

Continued from previous page...

Atom-1	Atom-2	Interatomic distance (Å)	Clash overlap (Å)
4:M:226:PHE:CE1	4:M:235:LEU:HB2	2.49	0.47
4:M:331:LEU:HA	4:M:335:SER:O	2.15	0.47
1:A:136:GLY:O	1:A:139:ASP:N	2.47	0.47
1:A:192:TYR:CE2	1:A:194:GLU:HB2	2.49	0.47
1:A:254:ILE:CG1	1:A:290:VAL:HG22	2.43	0.47
1:A:350:SER:O	1:A:352:PHE:N	2.46	0.47
2:B:100:LEU:CD2	4:M:123:LEU:CD1	2.92	0.47
2:B:162:VAL:HB	2:B:195:ILE:HG23	1.93	0.47
2:B:211:ALA:C	2:B:233:TYR:CZ	2.85	0.47
2:B:213:LEU:CD2	4:M:136:VAL:CB	2.90	0.47
2:B:215:TYR:HB3	2:B:226:LEU:HD13	1.96	0.47
2:B:415:LEU:HD13	2:B:436:LEU:HG	1.97	0.47
4:M:41:LEU:HD13	4:M:52:ASP:HB2	1.96	0.47
4:M:347:PHE:O	4:M:350:VAL:N	2.42	0.47
4:M:351:SER:HB2	4:M:441:GLY:CA	2.44	0.47
4:M:360:LEU:HD13	4:M:433:VAL:CB	2.44	0.47
1:A:187:LYS:O	1:A:190:LEU:HB3	2.14	0.47
1:A:316:LEU:O	1:A:319:LEU:HB2	2.14	0.47
2:B:16:LYS:NZ	4:M:111:ILE:CB	2.78	0.47
2:B:25:VAL:CG2	2:B:31:GLY:C	2.82	0.47
2:B:35:TYR:CZ	4:M:118:TYR:CE2	3.03	0.47
2:B:47:LEU:HD22	2:B:66:ILE:HG13	1.96	0.47
2:B:486:HIS:CD2	2:B:490:ILE:HG12	2.48	0.47
2:B:566:ALA:HB2	2:B:581:TYR:HB3	1.96	0.47
4:M:51:LEU:HD12	4:M:51:LEU:H	1.79	0.47
1:A:91:ILE:HG21	1:A:106:GLY:O	2.15	0.47
1:A:135:ASP:O	1:A:136:GLY:O	2.32	0.47
1:A:422:GLU:OE1	3:S:62:GLU:CB	2.61	0.47
1:A:423:ASN:N	3:S:62:GLU:OE1	2.48	0.47
1:A:570:LYS:O	1:A:571:ARG:CB	2.54	0.47
1:A:572:PHE:O	1:A:575:LYS:HB3	2.13	0.47
1:A:599:ARG:NH2	2:B:550:VAL:HG22	2.29	0.47
2:B:106:LEU:HG	4:M:130:GLU:HB3	1.75	0.47
2:B:270:SER:C	2:B:273:SER:HB2	2.34	0.47
2:B:360:LEU:HD22	2:B:395:LYS:HD3	1.97	0.47
2:B:494:ALA:HB2	2:B:515:PHE:HZ	1.74	0.47
2:B:563:PHE:CA	2:B:566:ALA:HB3	2.45	0.47
2:B:592:TYR:CG	2:B:593:ASN:N	2.83	0.47
4:M:76:CYS:CB	4:M:93:LEU:HD22	2.44	0.47
4:M:263:MET:HG2	4:M:263:MET:O	2.14	0.47
4:M:332:GLY:O	4:M:426:LYS:HE3	2.15	0.47

Continued on next page...

Continued from previous page...

Atom-1	Atom-2	Interatomic distance (Å)	Clash overlap (Å)
1:A:68:THR:HB	3:S:166:LYS:HD3	1.95	0.47
1:A:72:LEU:O	1:A:76:TYR:CD2	2.68	0.47
1:A:231:GLN:N	1:A:232:PRO:CD	2.78	0.47
1:A:292:TYR:O	1:A:295:VAL:HB	2.15	0.47
1:A:447:PHE:O	1:A:450:TYR:HB3	2.15	0.47
1:A:578:LEU:CD2	1:A:607:LEU:CD2	2.91	0.47
1:A:638:LEU:HA	2:B:557:SER:OG	2.14	0.47
2:B:5:ILE:CD1	4:M:39:PRO:HG3	2.40	0.47
2:B:231:ARG:HH21	2:B:279:LEU:HD21	1.64	0.47
2:B:237:ILE:HD12	2:B:248:LEU:CB	2.43	0.47
2:B:253:ILE:CG1	2:B:324:ALA:HB2	2.44	0.47
2:B:266:VAL:HG22	2:B:291:TYR:H	1.79	0.47
2:B:325:LEU:HB3	2:B:334:MET:CE	2.44	0.47
2:B:325:LEU:CB	2:B:334:MET:SD	2.95	0.47
2:B:389:ILE:HG23	2:B:428:VAL:HG23	1.97	0.47
2:B:399:LEU:CD1	2:B:415:LEU:CD2	2.93	0.47
2:B:439:CYS:O	2:B:441:GLN:N	2.48	0.47
3:S:53:THR:O	3:S:69:ASN:ND2	2.48	0.47
3:S:76:ILE:O	3:S:86:THR:HA	2.14	0.47
4:M:16:PHE:HA	4:M:118:TYR:HE2	1.78	0.47
4:M:106:LYS:O	4:M:107:ASP:O	2.31	0.47
4:M:223:HIS:CD2	4:M:478:ASN:CB	2.98	0.47
4:M:246:VAL:HA	4:M:470:ALA:HB2	1.96	0.47
4:M:257:ALA:HB3	4:M:453:ASP:OD2	2.15	0.47
4:M:290:PHE:CE1	4:M:297:PHE:CE2	3.03	0.47
1:A:84:MET:SD	1:A:112:GLN:O	2.73	0.47
1:A:134:TYR:CD2	1:A:136:GLY:N	2.81	0.47
1:A:291:ILE:HG12	1:A:322:PHE:CE2	2.50	0.47
1:A:319:LEU:O	1:A:321:THR:N	2.47	0.47
1:A:586:GLU:HB2	1:A:604:LEU:CD1	2.45	0.47
2:B:12:LEU:HD22	4:M:13:LYS:CG	2.45	0.47
2:B:219:TYR:O	2:B:223:LEU:CG	2.63	0.47
2:B:267:ASP:CB	2:B:289:PRO:HG3	2.45	0.47
2:B:313:SER:O	2:B:315:PRO:HD3	2.14	0.47
2:B:334:MET:HG3	2:B:369:LEU:HD23	1.97	0.47
3:S:38:LEU:CB	3:S:51:LEU:HD13	2.44	0.47
4:M:99:ILE:O	4:M:103:TYR:CD1	2.68	0.47
4:M:217:ASP:O	4:M:472:TYR:CD1	2.67	0.47
4:M:221:THR:CB	4:M:474:THR:OG1	2.62	0.47
4:M:347:PHE:CD2	4:M:350:VAL:O	2.68	0.47
4:M:435:LEU:CB	4:M:437:TYR:CE1	2.94	0.47

Continued on next page...

Continued from previous page...

Atom-1	Atom-2	Interatomic distance (Å)	Clash overlap (Å)
1:A:95:MET:HB2	1:A:127:LEU:HD23	1.96	0.47
1:A:166:LEU:O	1:A:170:LEU:CD2	2.63	0.47
1:A:219:VAL:CG2	1:A:256:LEU:HD21	2.40	0.47
2:B:327:GLN:O	2:B:328:LEU:C	2.51	0.47
2:B:335:LYS:C	2:B:373:LEU:HD22	2.35	0.47
2:B:403:ILE:O	2:B:403:ILE:HG13	2.15	0.47
2:B:471:TYR:O	2:B:474:VAL:HB	2.14	0.47
2:B:549:LEU:HD23	2:B:610:ARG:HB2	1.96	0.47
2:B:592:TYR:CE2	2:B:618:PHE:CE2	3.03	0.47
3:S:151:ALA:O	3:S:154:ASP:N	2.41	0.47
4:M:374:TYR:CE1	4:M:393:GLY:HA2	2.44	0.47
1:A:322:PHE:HD1	1:A:330:LEU:HD21	1.75	0.47
2:B:83:PHE:CD2	2:B:108:PHE:CB	2.97	0.47
2:B:181:TYR:CB	2:B:218:CYS:HG	2.27	0.47
2:B:230:PHE:CD1	2:B:234:CYS:SG	3.00	0.47
2:B:238:LYS:HG3	2:B:305:SER:OG	2.15	0.47
2:B:306:LEU:CD1	2:B:325:LEU:HD21	2.44	0.47
3:S:7:ILE:HD13	3:S:16:LEU:HD23	1.96	0.47
4:M:41:LEU:HD13	4:M:52:ASP:CB	2.45	0.47
4:M:100:LEU:O	4:M:109:LEU:HD21	2.15	0.47
4:M:218:LEU:HG	4:M:472:TYR:HE2	1.80	0.47
4:M:433:VAL:HG13	4:M:435:LEU:HD11	1.96	0.47
2:B:35:TYR:O	2:B:42:ILE:HG13	2.15	0.46
2:B:120:ILE:HD12	2:B:142:LEU:HD22	1.95	0.46
2:B:246:SER:HA	2:B:249:ILE:HD12	1.97	0.46
2:B:274:PRO:HG3	2:B:295:ASN:CA	2.33	0.46
2:B:278:PRO:CG	2:B:292:GLU:HB2	2.37	0.46
2:B:315:PRO:HA	2:B:318:ILE:HD12	1.97	0.46
2:B:337:THR:CA	2:B:373:LEU:CG	2.49	0.46
2:B:399:LEU:CD1	2:B:415:LEU:HD21	2.38	0.46
2:B:424:PHE:CE1	2:B:428:VAL:HG11	2.49	0.46
3:S:53:THR:HG23	3:S:57:LEU:HB3	1.97	0.46
3:S:102:ILE:O	3:S:105:PHE:HB3	2.15	0.46
4:M:2:TYR:HB3	4:M:23:THR:O	2.15	0.46
4:M:45:SER:HB2	4:M:75:TRP:CZ3	2.50	0.46
4:M:69:ILE:HD12	4:M:94:GLU:CA	2.44	0.46
4:M:222:PHE:CB	4:M:479:PHE:HZ	2.22	0.46
4:M:240:ILE:HG21	4:M:444:ALA:C	2.36	0.46
4:M:280:ASP:OD1	4:M:282:VAL:HB	2.15	0.46
4:M:311:LYS:O	4:M:312:GLN:C	2.51	0.46
4:M:338:PHE:CD1	4:M:415:ILE:CD1	2.98	0.46

Continued on next page...

Continued from previous page...

Atom-1	Atom-2	Interatomic distance (Å)	Clash overlap (Å)
1:A:294:SER:O	1:A:298:ILE:HG12	2.15	0.46
1:A:461:CYS:HB2	1:A:469:LEU:HD23	1.96	0.46
1:A:533:ILE:HD12	1:A:555:LEU:HD22	1.97	0.46
1:A:565:ASN:O	1:A:567:GLN:HG2	2.15	0.46
2:B:90:ILE:HB	2:B:101:ILE:HG21	1.96	0.46
2:B:123:LEU:HD12	2:B:142:LEU:HD23	1.95	0.46
2:B:178:ILE:HG23	2:B:218:CYS:N	2.29	0.46
2:B:219:TYR:O	2:B:223:LEU:HG	2.15	0.46
2:B:267:ASP:H	2:B:289:PRO:CB	2.28	0.46
2:B:396:ILE:HG21	2:B:431:MET:C	2.36	0.46
4:M:19:LEU:HD11	4:M:24:ALA:HB2	1.96	0.46
4:M:121:ILE:HG22	4:M:125:PHE:CZ	2.50	0.46
4:M:222:PHE:CA	4:M:479:PHE:CZ	2.97	0.46
4:M:244:VAL:O	4:M:299:LEU:CA	2.63	0.46
4:M:374:TYR:OH	4:M:395:GLY:N	2.47	0.46
1:A:219:VAL:HG22	1:A:240:LEU:HD22	1.98	0.46
1:A:300:LYS:O	1:A:302:ASN:N	2.48	0.46
1:A:355:LEU:O	1:A:359:LEU:HG	2.15	0.46
1:A:399:ASP:H	1:A:418:ILE:CD1	2.23	0.46
1:A:581:LEU:HG	1:A:607:LEU:HD11	1.95	0.46
1:A:605:GLU:CD	1:A:636:TYR:OH	2.52	0.46
1:A:625:LEU:HD11	1:A:629:LEU:HD12	1.98	0.46
2:B:106:LEU:CG	2:B:144:ASP:HB2	2.40	0.46
2:B:120:ILE:CG1	2:B:150:LEU:HD13	2.44	0.46
2:B:158:VAL:CG1	2:B:173:VAL:HG12	2.36	0.46
2:B:198:GLU:O	2:B:201:ALA:HB3	2.15	0.46
2:B:396:ILE:HD13	2:B:432:ALA:HA	1.91	0.46
2:B:450:VAL:O	2:B:453:TRP:HB2	2.16	0.46
2:B:557:SER:O	2:B:560:ILE:N	2.48	0.46
2:B:596:LEU:CD1	2:B:611:ALA:C	2.83	0.46
4:M:226:PHE:CD1	4:M:235:LEU:HA	2.50	0.46
4:M:359:ASP:HA	4:M:395:GLY:O	2.15	0.46
4:M:364:VAL:HA	4:M:367:ALA:O	2.15	0.46
1:A:128:LEU:CD1	1:A:150:LEU:CD2	2.94	0.46
1:A:215:VAL:O	1:A:219:VAL:HG23	2.16	0.46
1:A:408:ILE:CG2	3:S:41:GLN:HB3	2.09	0.46
1:A:422:GLU:H	3:S:62:GLU:HB3	1.80	0.46
1:A:462:GLN:O	1:A:464:ILE:N	2.49	0.46
2:B:185:LYS:O	2:B:186:ASN:C	2.31	0.46
2:B:253:ILE:CG1	2:B:324:ALA:HB1	2.44	0.46
2:B:316:THR:C	2:B:318:ILE:N	2.66	0.46

Continued on next page...

Continued from previous page...

Atom-1	Atom-2	Interatomic distance (Å)	Clash overlap (Å)
4:M:8:THR:HA	4:M:15:ILE:HG12	1.98	0.46
4:M:283:PHE:HE2	4:M:289:THR:CB	2.25	0.46
1:A:244:LEU:CG	1:A:281:LEU:CD1	2.94	0.46
1:A:401:VAL:HG23	1:A:418:ILE:O	2.16	0.46
1:A:461:CYS:SG	1:A:464:ILE:HG22	2.55	0.46
2:B:43:ASN:HB3	2:B:44:PRO:CD	2.45	0.46
2:B:71:ALA:CB	4:M:19:LEU:N	2.76	0.46
2:B:136:CYS:CB	2:B:172:GLU:CG	2.77	0.46
2:B:212:VAL:N	2:B:233:TYR:OH	2.45	0.46
2:B:213:LEU:HD22	4:M:136:VAL:CG2	2.46	0.46
2:B:227:HIS:C	2:B:229:HIS:H	2.19	0.46
2:B:250:GLU:OE2	4:M:136:VAL:HG22	2.15	0.46
2:B:348:THR:O	2:B:349:MET:C	2.50	0.46
3:S:53:THR:C	3:S:69:ASN:CG	2.73	0.46
4:M:223:HIS:HE1	4:M:476:THR:H	1.62	0.46
4:M:224:VAL:HG11	4:M:226:PHE:CE2	2.49	0.46
4:M:306:LEU:CG	4:M:317:MET:HE3	2.46	0.46
1:A:91:ILE:CG2	1:A:106:GLY:O	2.64	0.46
1:A:409:VAL:CG2	1:A:410:TYR:N	2.78	0.46
1:A:471:SER:HA	1:A:510:THR:HG21	1.96	0.46
2:B:321:CYS:O	2:B:325:LEU:HG	2.15	0.46
2:B:399:LEU:HD13	2:B:415:LEU:HG	1.97	0.46
2:B:477:MET:O	2:B:480:GLN:N	2.46	0.46
4:M:347:PHE:HA	4:M:350:VAL:CG2	2.46	0.46
1:A:67:LYS:HB2	3:S:166:LYS:NZ	2.31	0.46
1:A:179:LYS:NZ	3:S:141:VAL:CA	2.71	0.46
1:A:247:ILE:HG21	1:A:252:ILE:CG2	2.45	0.46
1:A:254:ILE:CD1	3:S:96:LEU:HB2	2.44	0.46
1:A:395:PHE:CZ	1:A:428:MET:HB2	2.51	0.46
1:A:554:ALA:O	1:A:557:LYS:HB2	2.15	0.46
1:A:594:PHE:HZ	2:B:477:MET:CE	2.28	0.46
2:B:27:THR:CB	2:B:57:ARG:HD2	2.38	0.46
2:B:66:ILE:HG22	2:B:104:TYR:CE1	2.51	0.46
2:B:105:LEU:HB3	2:B:145:MET:HE1	1.97	0.46
2:B:293:VAL:CA	2:B:299:LEU:CG	2.87	0.46
2:B:437:SER:C	2:B:439:CYS:N	2.69	0.46
3:S:87:PHE:CD2	3:S:102:ILE:HG12	2.51	0.46
4:M:5:PHE:CD2	4:M:20:LEU:CD1	2.98	0.46
4:M:215:TYR:HB3	4:M:470:ALA:H	1.81	0.46
4:M:222:PHE:CZ	4:M:439:TYR:CE2	3.04	0.46
4:M:222:PHE:CG	4:M:439:TYR:HE2	2.31	0.46

Continued on next page...

Continued from previous page...

Atom-1	Atom-2	Interatomic distance (Å)	Clash overlap (Å)
4:M:246:VAL:HA	4:M:470:ALA:CB	2.45	0.46
4:M:300:LEU:HD11	4:M:447:ILE:HD12	1.98	0.46
4:M:322:LEU:O	4:M:322:LEU:HD23	2.16	0.46
4:M:435:LEU:O	4:M:437:TYR:HE1	1.99	0.46
1:A:200:PHE:CZ	1:A:236:LEU:CG	2.98	0.46
1:A:204:VAL:O	1:A:206:LYS:N	2.48	0.46
1:A:280:GLU:O	1:A:283:GLU:HB3	2.15	0.46
1:A:558:VAL:HG12	1:A:562:TRP:CD2	2.50	0.46
2:B:9:ALA:HB1	4:M:14:LEU:HD12	1.97	0.46
2:B:256:CYS:HB3	2:B:328:LEU:CD2	2.35	0.46
2:B:549:LEU:CD2	2:B:610:ARG:HB2	2.46	0.46
3:S:2:ILE:CD1	3:S:101:LEU:HD22	2.45	0.46
3:S:80:TYR:HH	3:S:110:ASP:CG	2.18	0.46
4:M:121:ILE:HG23	4:M:125:PHE:HE1	1.81	0.46
4:M:376:ILE:CD1	4:M:415:ILE:HG23	2.46	0.46
4:M:435:LEU:HD12	4:M:435:LEU:N	2.31	0.46
1:A:170:LEU:HD13	1:A:170:LEU:HA	1.80	0.46
1:A:219:VAL:HG22	1:A:240:LEU:CD2	2.46	0.46
1:A:244:LEU:CB	1:A:256:LEU:HD13	2.46	0.46
1:A:275:LEU:C	1:A:275:LEU:HD23	2.36	0.46
1:A:275:LEU:HD12	1:A:303:MET:SD	2.55	0.46
1:A:405:THR:CA	2:B:7:ARG:CZ	2.89	0.46
2:B:21:GLU:HA	2:B:21:GLU:OE1	2.16	0.46
2:B:127:LEU:CG	2:B:157:THR:HG21	2.46	0.46
2:B:169:VAL:HG12	2:B:173:VAL:CG2	2.46	0.46
2:B:285:GLU:O	2:B:286:ILE:O	2.31	0.46
2:B:359:LEU:O	2:B:363:ILE:HG12	2.16	0.46
2:B:378:THR:OG1	2:B:411:ILE:HG12	2.16	0.46
2:B:399:LEU:O	2:B:400:SER:C	2.53	0.46
2:B:408:VAL:CG1	2:B:446:TRP:CB	2.93	0.46
2:B:500:GLN:CB	2:B:503:LEU:HG	2.46	0.46
2:B:591:MET:O	2:B:595:VAL:HG23	2.16	0.46
3:S:50:PHE:HA	3:S:77:TYR:CD1	2.45	0.46
4:M:51:LEU:H	4:M:51:LEU:CD1	2.28	0.46
4:M:374:TYR:CD2	4:M:376:ILE:HD11	2.51	0.46
4:M:442:GLN:CG	4:M:443:SER:N	2.76	0.46
1:A:166:LEU:O	1:A:170:LEU:HD22	2.16	0.46
1:A:197:ARG:O	1:A:198:ASP:C	2.49	0.46
1:A:244:LEU:HD22	1:A:260:PHE:HE2	1.81	0.46
1:A:316:LEU:HD11	1:A:348:PHE:CE2	2.48	0.46
1:A:436:CYS:CB	1:A:450:TYR:CZ	2.99	0.46

Continued on next page...

Continued from previous page...

Atom-1	Atom-2	Interatomic distance (Å)	Clash overlap (Å)
2:B:47:LEU:HD21	2:B:65:ARG:HB3	1.97	0.46
2:B:278:PRO:HD2	2:B:292:GLU:CD	2.33	0.46
3:S:83:LEU:HD12	3:S:116:VAL:HG11	1.97	0.46
3:S:132:LEU:O	3:S:136:VAL:HG23	2.16	0.46
4:M:9:ASP:C	4:M:9:ASP:OD1	2.54	0.46
4:M:118:TYR:O	4:M:121:ILE:HB	2.16	0.46
4:M:242:GLY:N	4:M:444:ALA:HB2	2.31	0.46
4:M:304:VAL:CB	4:M:445:SER:HA	2.46	0.46
4:M:436:GLU:HG3	4:M:436:GLU:O	2.16	0.46
4:M:437:TYR:CB	4:M:439:TYR:CZ	2.99	0.46
1:A:103:LYS:O	1:A:104:ARG:O	2.34	0.45
1:A:104:ARG:CG	1:A:145:ILE:HG13	2.46	0.45
1:A:182:ILE:HG23	1:A:221:VAL:HG21	1.93	0.45
2:B:35:TYR:CE1	4:M:118:TYR:CZ	3.04	0.45
2:B:279:LEU:O	2:B:280:PRO:C	2.52	0.45
2:B:310:ILE:HG23	2:B:318:ILE:HG12	1.98	0.45
2:B:344:VAL:HG22	2:B:381:PHE:CE2	2.51	0.45
2:B:386:LYS:CE	4:M:478:ASN:OD1	2.62	0.45
2:B:418:TYR:OH	2:B:432:ALA:HB3	2.07	0.45
2:B:418:TYR:CE1	2:B:419:VAL:HG22	2.52	0.45
2:B:447:GLU:O	2:B:450:VAL:HB	2.16	0.45
2:B:577:ASN:C	2:B:578:PRO:O	2.53	0.45
3:S:68:VAL:O	3:S:75:ILE:CD1	2.65	0.45
4:M:67:SER:OG	4:M:90:PHE:CD1	2.54	0.45
4:M:437:TYR:HD1	4:M:479:PHE:CE1	2.33	0.45
1:A:185:LEU:HB3	1:A:203:PHE:CE1	2.48	0.45
1:A:256:LEU:O	1:A:260:PHE:CD2	2.69	0.45
1:A:399:ASP:HA	1:A:420:ILE:HB	1.98	0.45
1:A:450:TYR:O	1:A:454:ILE:HG12	2.16	0.45
1:A:617:ASP:OD1	1:A:618:THR:N	2.46	0.45
1:A:638:LEU:HD21	2:B:561:ASP:CB	2.45	0.45
2:B:75:ASP:CG	4:M:24:ALA:CB	2.58	0.45
2:B:276:SER:OG	2:B:289:PRO:HG3	2.17	0.45
2:B:577:ASN:O	2:B:578:PRO:O	2.35	0.45
4:M:229:LYS:HE2	4:M:230:LYS:HE3	1.98	0.45
4:M:350:VAL:N	4:M:442:GLN:HE21	2.15	0.45
4:M:480:GLN:OE1	4:M:482:ARG:NH2	2.46	0.45
1:A:366:SER:O	1:A:370:LYS:HG2	2.17	0.45
1:A:503:ASN:CG	4:M:60:LEU:CG	2.83	0.45
2:B:106:LEU:HG	4:M:130:GLU:CB	2.41	0.45
2:B:123:LEU:CD1	2:B:142:LEU:CD2	2.94	0.45

Continued on next page...

Continued from previous page...

Atom-1	Atom-2	Interatomic distance (Å)	Clash overlap (Å)
2:B:155:LEU:HD21	2:B:192:LEU:CD1	2.10	0.45
2:B:574:ASN:O	2:B:576:GLN:N	2.49	0.45
4:M:253:ASN:O	4:M:254:PRO:C	2.53	0.45
4:M:291:ILE:HG12	4:M:292:PRO:HD2	1.98	0.45
4:M:338:PHE:CD1	4:M:415:ILE:CG1	2.97	0.45
4:M:410:VAL:HG11	4:M:412:ARG:CZ	2.47	0.45
1:A:186:PHE:CB	1:A:221:VAL:HG13	2.45	0.45
1:A:356:ILE:HD11	1:A:374:LEU:HD23	1.99	0.45
1:A:497:LYS:O	1:A:500:SER:N	2.41	0.45
1:A:604:LEU:HD23	1:A:604:LEU:C	2.37	0.45
2:B:143:SER:CA	2:B:179:LYS:HB2	2.43	0.45
2:B:261:PRO:HB2	2:B:290:SER:OG	2.17	0.45
4:M:212:ASN:CG	4:M:250:LEU:HA	2.37	0.45
4:M:220:GLU:OE1	4:M:222:PHE:HZ	1.99	0.45
4:M:290:PHE:CE2	4:M:291:ILE:O	2.70	0.45
1:A:87:CYS:O	1:A:91:ILE:HG12	2.17	0.45
1:A:213:SER:HB3	3:S:142:ILE:CA	2.46	0.45
1:A:395:PHE:CG	1:A:428:MET:HE2	2.51	0.45
1:A:476:GLN:OE1	1:A:476:GLN:HA	2.16	0.45
2:B:103:LEU:HD22	4:M:127:CYS:HA	1.97	0.45
2:B:319:LEU:HD22	2:B:358:MET:HB3	1.96	0.45
2:B:341:GLU:N	2:B:377:TYR:CE2	2.84	0.45
2:B:374:PHE:CE1	2:B:381:PHE:HE2	2.20	0.45
2:B:458:MET:SD	2:B:471:TYR:CB	2.98	0.45
2:B:474:VAL:O	2:B:476:ARG:N	2.49	0.45
2:B:560:ILE:C	2:B:563:PHE:H	2.19	0.45
2:B:592:TYR:CE2	2:B:618:PHE:CD2	3.04	0.45
3:S:30:LEU:O	3:S:34:GLN:HG3	2.16	0.45
4:M:20:LEU:CD2	4:M:129:VAL:CG2	2.71	0.45
4:M:42:LEU:HD23	4:M:51:LEU:CG	2.46	0.45
4:M:57:GLY:O	4:M:58:ARG:HG3	2.17	0.45
4:M:223:HIS:CD2	4:M:479:PHE:CE1	3.04	0.45
4:M:245:ASP:HA	4:M:297:PHE:O	2.16	0.45
4:M:261:ASN:N	4:M:448:TYR:O	2.42	0.45
4:M:374:TYR:CE2	4:M:376:ILE:HD11	2.51	0.45
1:A:258:LYS:HZ1	3:S:93:GLU:CA	1.88	0.45
1:A:364:ASP:O	1:A:367:ILE:HB	2.17	0.45
1:A:413:SER:O	1:A:415:ARG:O	2.35	0.45
1:A:492:ILE:HD13	1:A:526:VAL:HG21	1.99	0.45
1:A:566:PHE:HD1	1:A:570:LYS:HA	1.76	0.45
1:A:623:MET:O	1:A:627:GLU:HG3	2.16	0.45

Continued on next page...

Continued from previous page...

Atom-1	Atom-2	Interatomic distance (Å)	Clash overlap (Å)
2:B:5:ILE:HD11	4:M:39:PRO:CB	2.46	0.45
2:B:27:THR:HB	2:B:57:ARG:CD	2.40	0.45
2:B:100:LEU:CD2	4:M:123:LEU:HD13	2.46	0.45
2:B:120:ILE:HA	2:B:142:LEU:CD2	2.44	0.45
2:B:143:SER:CA	2:B:179:LYS:HD2	2.46	0.45
2:B:354:GLY:HA2	4:M:49:ASP:OD1	2.17	0.45
2:B:364:HIS:NE2	2:B:368:ILE:HD11	2.32	0.45
2:B:416:LYS:HA	2:B:457:HIS:NE2	2.30	0.45
2:B:549:LEU:HD21	2:B:611:ALA:H	1.80	0.45
2:B:566:ALA:C	2:B:574:ASN:CB	2.84	0.45
2:B:602:ASP:OD1	2:B:603:ASP:N	2.50	0.45
4:M:16:PHE:HA	4:M:118:TYR:CD2	2.52	0.45
4:M:215:TYR:CD2	4:M:469:GLY:N	2.80	0.45
4:M:376:ILE:HB	4:M:388:ASN:OD1	2.17	0.45
1:A:158:LEU:HD12	1:A:162:ILE:HG13	1.98	0.45
1:A:259:LEU:O	1:A:262:ASN:HB2	2.16	0.45
1:A:496:ILE:HG13	1:A:532:LEU:CD1	2.47	0.45
2:B:9:ALA:CB	4:M:14:LEU:CD1	2.94	0.45
2:B:18:ILE:O	2:B:33:SER:HA	2.16	0.45
2:B:144:ASP:OD2	4:M:131:ALA:CB	2.65	0.45
2:B:318:ILE:CG2	2:B:346:THR:OG1	2.62	0.45
2:B:327:GLN:O	2:B:329:ALA:N	2.50	0.45
2:B:396:ILE:CG1	2:B:418:TYR:HE2	2.25	0.45
2:B:408:VAL:HG11	2:B:446:TRP:HB2	1.95	0.45
3:S:53:THR:CG2	3:S:57:LEU:CB	2.95	0.45
4:M:7:ILE:HD11	4:M:121:ILE:CG2	2.45	0.45
4:M:65:TYR:CE2	4:M:86:PRO:CB	2.95	0.45
4:M:70:ASN:CG	4:M:75:TRP:CZ2	2.90	0.45
4:M:281:GLY:N	4:M:283:PHE:O	2.50	0.45
1:A:97:SER:O	1:A:98:ASN:C	2.46	0.45
1:A:333:ILE:HD11	3:S:95:GLU:CD	2.29	0.45
1:A:498:LEU:O	1:A:501:ASN:O	2.35	0.45
2:B:170:ARG:O	2:B:171:GLY:C	2.54	0.45
2:B:208:ILE:HG21	2:B:236:ILE:HG21	1.98	0.45
2:B:394:TRP:CZ3	2:B:397:GLN:OE1	2.70	0.45
2:B:418:TYR:O	2:B:419:VAL:O	2.32	0.45
2:B:564:LYS:HG2	2:B:565:GLN:H	1.82	0.45
3:S:5:VAL:O	3:S:17:VAL:HG23	2.16	0.45
4:M:372:ILE:CD1	4:M:428:VAL:HG13	2.47	0.45
1:A:128:LEU:HD13	1:A:150:LEU:CD2	2.47	0.45
1:A:206:LYS:C	1:A:208:ASP:H	2.20	0.45

Continued on next page...

Continued from previous page...

Atom-1	Atom-2	Interatomic distance (Å)	Clash overlap (Å)
1:A:215:VAL:O	1:A:216:SER:O	2.34	0.45
1:A:219:VAL:HG11	1:A:256:LEU:CD2	2.47	0.45
1:A:316:LEU:HD12	1:A:348:PHE:CE2	2.49	0.45
1:A:422:GLU:OE1	3:S:62:GLU:CD	2.55	0.45
1:A:563:CYS:CB	1:A:621:LEU:HD11	2.18	0.45
3:S:60:SER:O	3:S:66:ASP:CB	2.65	0.45
3:S:136:VAL:O	3:S:139:GLY:C	2.56	0.45
3:S:155:GLU:HA	3:S:158:LYS:HE2	1.99	0.45
4:M:372:ILE:HD12	4:M:428:VAL:CG2	2.47	0.45
4:M:453:ASP:OD1	4:M:453:ASP:O	2.35	0.45
1:A:399:ASP:OD2	1:A:403:LEU:HD12	2.17	0.45
1:A:450:TYR:CE1	1:A:454:ILE:HD11	2.52	0.45
1:A:499:ILE:HG12	1:A:512:LEU:CD2	2.47	0.45
1:A:638:LEU:HD21	2:B:561:ASP:CA	2.35	0.45
1:A:638:LEU:HD12	2:B:558:TYR:CA	2.46	0.45
2:B:17:VAL:HG23	2:B:35:TYR:CD2	2.27	0.45
2:B:87:VAL:HG11	2:B:118:LEU:HG	1.99	0.45
2:B:199:LEU:O	2:B:200:MET:C	2.50	0.45
2:B:226:LEU:HD21	2:B:255:TYR:CG	2.50	0.45
2:B:247:TYR:HD1	4:M:136:VAL:CG1	2.22	0.45
2:B:343:LEU:HG	2:B:363:ILE:CD1	2.47	0.45
2:B:344:VAL:CG2	2:B:374:PHE:CD1	3.00	0.45
2:B:396:ILE:HD11	2:B:432:ALA:HB2	1.90	0.45
2:B:433:VAL:CG2	2:B:471:TYR:CG	3.00	0.45
2:B:447:GLU:OE1	2:B:485:LYS:CB	2.64	0.45
3:S:57:LEU:N	3:S:58:LEU:O	2.47	0.45
3:S:65:ASN:O	3:S:67:GLU:CG	2.62	0.45
4:M:69:ILE:HD13	4:M:94:GLU:HA	1.95	0.45
4:M:92:PHE:HE2	4:M:128:CYS:C	2.20	0.45
4:M:242:GLY:CA	4:M:444:ALA:CB	2.93	0.45
4:M:261:ASN:CB	4:M:450:GLU:HG3	2.30	0.45
4:M:271:SER:N	4:M:301:GLU:O	2.35	0.45
1:A:166:LEU:CD1	1:A:185:LEU:HD23	2.47	0.44
1:A:216:SER:HB3	3:S:140:MET:CG	2.47	0.44
2:B:107:ARG:CZ	4:M:18:TYR:OH	2.65	0.44
2:B:116:THR:HG21	2:B:150:LEU:HD11	1.98	0.44
2:B:262:LYS:O	2:B:263:PRO:C	2.50	0.44
2:B:346:THR:O	2:B:350:THR:N	2.49	0.44
2:B:509:ALA:CB	2:B:547:GLN:HG3	2.45	0.44
2:B:546:CYS:HB2	2:B:607:ILE:CG1	2.47	0.44
2:B:565:GLN:CB	2:B:581:TYR:OH	2.65	0.44

Continued on next page...

Continued from previous page...

Atom-1	Atom-2	Interatomic distance (Å)	Clash overlap (Å)
3:S:151:ALA:O	3:S:154:ASP:HB2	2.17	0.44
4:M:270:PRO:CA	4:M:302:TYR:CD2	3.00	0.44
4:M:338:PHE:CD1	4:M:415:ILE:HD11	2.52	0.44
4:M:344:ILE:HG22	4:M:344:ILE:O	2.17	0.44
1:A:189:PHE:C	1:A:191:GLN:N	2.68	0.44
1:A:625:LEU:C	1:A:627:GLU:N	2.70	0.44
2:B:13:ASP:OD1	4:M:14:LEU:CA	2.63	0.44
2:B:14:THR:CB	2:B:40:GLN:HE21	2.30	0.44
2:B:106:LEU:HD11	2:B:144:ASP:CB	2.00	0.44
2:B:107:ARG:HA	4:M:130:GLU:CD	2.37	0.44
2:B:181:TYR:HB2	2:B:218:CYS:SG	2.57	0.44
2:B:234:CYS:CB	2:B:301:LEU:HB2	2.46	0.44
2:B:252:LEU:CA	2:B:302:PHE:CE2	3.01	0.44
2:B:270:SER:O	2:B:273:SER:CB	2.64	0.44
2:B:371:GLN:O	2:B:373:LEU:N	2.50	0.44
2:B:396:ILE:CD1	2:B:418:TYR:CE2	3.00	0.44
2:B:418:TYR:CE2	2:B:432:ALA:HB2	2.51	0.44
2:B:431:MET:O	2:B:434:LYS:N	2.50	0.44
2:B:562:ASN:HB3	2:B:580:TYR:HB2	2.00	0.44
2:B:565:GLN:HB3	2:B:581:TYR:CZ	2.51	0.44
4:M:217:ASP:O	4:M:472:TYR:CD2	2.71	0.44
4:M:434:SER:CB	4:M:479:PHE:O	2.65	0.44
1:A:92:LEU:CD1	1:A:123:LEU:CD1	2.96	0.44
1:A:180:LYS:HZ3	3:S:137:GLN:CB	2.30	0.44
1:A:290:VAL:O	1:A:293:GLU:HB3	2.16	0.44
1:A:435:ILE:HG22	1:A:441:TYR:CE2	2.53	0.44
1:A:595:GLU:OE2	2:B:513:TRP:CE2	2.70	0.44
2:B:268:LYS:HA	2:B:276:SER:CB	2.40	0.44
2:B:361:GLN:O	2:B:364:HIS:HB3	2.17	0.44
2:B:440:GLY:O	2:B:442:LEU:N	2.51	0.44
2:B:520:SER:O	2:B:523:PHE:HE1	2.01	0.44
2:B:566:ALA:O	2:B:574:ASN:CB	2.65	0.44
3:S:93:GLU:CG	3:S:98:ILE:HD11	2.48	0.44
4:M:9:ASP:OD2	4:M:13:LYS:HB3	2.17	0.44
4:M:214:LEU:CD2	4:M:466:LEU:HG	2.47	0.44
4:M:223:HIS:CD2	4:M:479:PHE:CD1	3.05	0.44
4:M:252:ASP:C	4:M:254:PRO:CD	2.85	0.44
4:M:320:ILE:HA	4:M:323:MET:CE	2.46	0.44
4:M:432:THR:O	4:M:432:THR:CG2	2.65	0.44
4:M:443:SER:CB	4:M:447:ILE:CG1	2.63	0.44
4:M:446:GLY:C	4:M:448:TYR:H	2.19	0.44

Continued on next page...

Continued from previous page...

Atom-1	Atom-2	Interatomic distance (Å)	Clash overlap (Å)
1:A:68:THR:O	1:A:72:LEU:HG	2.17	0.44
1:A:264:SER:HB2	1:A:271:ARG:HD3	2.00	0.44
2:B:141:ALA:O	2:B:145:MET:HG2	2.18	0.44
2:B:232:ARG:HG3	2:B:236:ILE:CG1	2.47	0.44
2:B:310:ILE:CG2	2:B:342:ALA:C	2.85	0.44
2:B:530:LEU:HD23	2:B:591:MET:CB	2.48	0.44
2:B:604:GLU:O	2:B:607:ILE:HB	2.18	0.44
3:S:8:PHE:HZ	3:S:86:THR:OG1	1.95	0.44
3:S:16:LEU:HB2	3:S:125:TRP:CE2	2.47	0.44
3:S:39:ILE:HG23	3:S:47:GLN:CD	2.38	0.44
4:M:134:PRO:O	4:M:136:VAL:HG23	2.17	0.44
4:M:323:MET:HE2	4:M:437:TYR:HE2	1.81	0.44
4:M:374:TYR:O	4:M:390:ILE:CG2	2.65	0.44
1:A:244:LEU:HB2	1:A:256:LEU:HD13	1.99	0.44
1:A:407:SER:CA	3:S:64:ASN:ND2	2.75	0.44
1:A:569:ASP:C	1:A:571:ARG:N	2.67	0.44
1:A:625:LEU:HD12	1:A:629:LEU:HB2	2.00	0.44
2:B:28:SER:C	2:B:58:GLU:CD	2.75	0.44
2:B:154:ILE:HB	2:B:180:LEU:HD13	1.99	0.44
2:B:174:ALA:CB	2:B:211:ALA:CA	2.80	0.44
2:B:327:GLN:C	2:B:329:ALA:N	2.70	0.44
2:B:374:PHE:CE2	2:B:398:ILE:HG23	2.48	0.44
2:B:433:VAL:C	2:B:474:VAL:CG2	2.82	0.44
2:B:545:ARG:HB2	2:B:607:ILE:HD13	2.00	0.44
3:S:16:LEU:HD11	3:S:129:GLU:HG2	1.96	0.44
4:M:90:PHE:O	4:M:93:LEU:HB2	2.16	0.44
4:M:103:TYR:CB	4:M:104:PHE:CD1	3.00	0.44
4:M:240:ILE:HG22	4:M:444:ALA:HA	1.82	0.44
1:A:68:THR:HB	3:S:166:LYS:CD	2.47	0.44
1:A:255:ARG:CZ	3:S:139:GLY:O	2.66	0.44
2:B:35:TYR:C	2:B:37:TYR:H	2.21	0.44
2:B:103:LEU:CB	4:M:126:ASN:ND2	2.60	0.44
2:B:107:ARG:NH2	4:M:18:TYR:CE2	2.85	0.44
2:B:144:ASP:OD1	4:M:132:GLY:N	2.51	0.44
2:B:200:MET:CE	2:B:229:HIS:CA	2.96	0.44
2:B:337:THR:O	2:B:373:LEU:HD11	2.16	0.44
4:M:217:ASP:C	4:M:472:TYR:CE2	2.91	0.44
4:M:217:ASP:N	4:M:472:TYR:CZ	2.86	0.44
4:M:219:LEU:HD22	4:M:474:THR:N	2.33	0.44
4:M:347:PHE:HE1	4:M:350:VAL:CG1	1.91	0.44
1:A:185:LEU:HB2	1:A:203:PHE:CE1	2.52	0.44

Continued on next page...

Continued from previous page...

Atom-1	Atom-2	Interatomic distance (Å)	Clash overlap (Å)
1:A:375:VAL:HG11	1:A:387:ILE:HG21	1.98	0.44
1:A:405:THR:OG1	1:A:406:GLY:N	2.51	0.44
2:B:83:PHE:CD1	2:B:87:VAL:CG2	3.00	0.44
2:B:188:TYR:HB3	2:B:192:LEU:CD1	2.47	0.44
2:B:216:LYS:NZ	4:M:133:GLU:OE2	2.49	0.44
2:B:266:VAL:HG12	2:B:275:ARG:HB3	1.99	0.44
2:B:303:LEU:HB3	2:B:339:PHE:CZ	2.53	0.44
2:B:572:GLU:O	2:B:575:ASN:N	2.43	0.44
3:S:8:PHE:HB3	3:S:36:TYR:HH	1.70	0.44
4:M:270:PRO:HA	4:M:302:TYR:HD2	1.83	0.44
4:M:336:ASP:CG	4:M:415:ILE:HB	2.38	0.44
4:M:374:TYR:HB3	4:M:417:TYR:CD1	2.44	0.44
1:A:153:ILE:HB	1:A:158:LEU:HD21	1.99	0.44
1:A:170:LEU:CD2	1:A:181:ALA:HB1	2.44	0.44
1:A:260:PHE:CE2	1:A:274:LEU:CD1	3.00	0.44
1:A:264:SER:HB3	1:A:271:ARG:HG2	2.00	0.44
1:A:495:ILE:HD12	1:A:515:CYS:SG	2.58	0.44
1:A:506:LYS:HB3	4:M:61:GLU:HG3	0.80	0.44
2:B:103:LEU:CD2	4:M:127:CYS:HA	2.48	0.44
2:B:123:LEU:CD1	2:B:142:LEU:HG	2.45	0.44
2:B:178:ILE:HD13	2:B:178:ILE:HA	1.90	0.44
2:B:238:LYS:HE3	2:B:305:SER:OG	2.18	0.44
2:B:389:ILE:O	2:B:393:ILE:HG13	2.17	0.44
2:B:392:SER:OG	2:B:424:PHE:HE1	2.01	0.44
2:B:430:ILE:CG1	2:B:467:VAL:HA	2.43	0.44
2:B:514:LEU:O	2:B:517:GLU:HB2	2.18	0.44
4:M:121:ILE:CG2	4:M:125:PHE:CZ	3.01	0.44
4:M:242:GLY:O	4:M:301:GLU:CA	2.60	0.44
1:A:638:LEU:HD13	2:B:557:SER:CA	2.46	0.44
2:B:47:LEU:CD2	2:B:65:ARG:HB2	2.48	0.44
2:B:123:LEU:O	2:B:127:LEU:HG	2.18	0.44
2:B:212:VAL:HG22	2:B:233:TYR:CE1	2.51	0.44
2:B:239:GLN:CA	4:M:279:ASN:O	2.57	0.44
2:B:300:ASP:C	2:B:302:PHE:N	2.69	0.44
2:B:545:ARG:HD3	2:B:602:ASP:OD2	2.18	0.44
4:M:74:TYR:HB3	4:M:114:ILE:HD12	1.94	0.44
4:M:304:VAL:O	4:M:304:VAL:HG13	2.18	0.44
4:M:357:LYS:HB2	4:M:436:GLU:OE2	2.17	0.44
4:M:437:TYR:HB2	4:M:439:TYR:CZ	2.52	0.44
1:A:114:PHE:HE1	1:A:154:ILE:HG12	1.83	0.43
1:A:314:ALA:O	1:A:318:ARG:HD3	2.18	0.43

Continued on next page...

Continued from previous page...

Atom-1	Atom-2	Interatomic distance (Å)	Clash overlap (Å)
1:A:435:ILE:O	1:A:436:CYS:C	2.56	0.43
2:B:171:GLY:CA	2:B:207:VAL:HA	2.48	0.43
2:B:224:GLU:HA	2:B:259:TYR:OH	0.58	0.43
2:B:306:LEU:HD12	2:B:325:LEU:HD23	2.00	0.43
2:B:329:ALA:O	2:B:331:PRO:HD3	2.18	0.43
2:B:371:GLN:C	2:B:373:LEU:N	2.70	0.43
2:B:469:ASP:OD1	2:B:507:ALA:CA	2.65	0.43
3:S:53:THR:HG21	3:S:69:ASN:N	2.33	0.43
4:M:222:PHE:CG	4:M:439:TYR:CE2	3.06	0.43
4:M:290:PHE:CE1	4:M:291:ILE:O	2.70	0.43
4:M:376:ILE:HG21	4:M:379:LEU:HD13	1.99	0.43
1:A:125:THR:C	1:A:127:LEU:N	2.70	0.43
2:B:20:ARG:NH1	4:M:118:TYR:H	2.16	0.43
2:B:396:ILE:C	2:B:435:SER:OG	2.56	0.43
2:B:563:PHE:C	2:B:564:LYS:O	2.57	0.43
3:S:70:ASN:HD22	3:S:73:ILE:HD12	1.83	0.43
3:S:113:PHE:CD1	3:S:113:PHE:N	2.85	0.43
4:M:212:ASN:O	4:M:465:LYS:N	2.49	0.43
4:M:219:LEU:H	4:M:472:TYR:CB	2.29	0.43
4:M:223:HIS:HB3	4:M:479:PHE:N	2.32	0.43
4:M:377:LYS:CE	4:M:416:GLU:OE1	2.67	0.43
1:A:153:ILE:HB	1:A:158:LEU:CD2	2.49	0.43
1:A:356:ILE:O	1:A:359:LEU:HB2	2.17	0.43
1:A:460:LEU:C	1:A:462:GLN:N	2.71	0.43
2:B:38:TYR:O	2:B:39:SER:C	2.55	0.43
2:B:141:ALA:O	2:B:144:ASP:N	2.50	0.43
2:B:256:CYS:HB3	2:B:328:LEU:HD22	1.91	0.43
2:B:430:ILE:O	2:B:433:VAL:HB	2.18	0.43
2:B:486:HIS:CE1	2:B:518:ILE:HB	2.53	0.43
4:M:216:VAL:O	4:M:216:VAL:CG2	2.66	0.43
4:M:245:ASP:HB2	4:M:472:TYR:CD1	2.46	0.43
4:M:273:HIS:HB3	4:M:276:VAL:HG23	2.00	0.43
4:M:291:ILE:CG1	4:M:292:PRO:CD	2.96	0.43
4:M:360:LEU:HD23	4:M:362:PHE:CZ	2.53	0.43
1:A:180:LYS:NZ	3:S:137:GLN:HB2	2.33	0.43
1:A:420:ILE:HA	1:A:421:PRO:HD3	1.83	0.43
1:A:594:PHE:O	1:A:597:GLN:HB3	2.18	0.43
2:B:14:THR:CG2	2:B:18:ILE:CG2	2.92	0.43
2:B:79:VAL:CG2	2:B:108:PHE:CD2	3.00	0.43
2:B:89:ASN:O	2:B:92:THR:HB	2.18	0.43
2:B:152:PRO:CA	2:B:188:TYR:CE1	2.82	0.43

Continued on next page...

Continued from previous page...

Atom-1	Atom-2	Interatomic distance (Å)	Clash overlap (Å)
2:B:340:ILE:HG21	2:B:374:PHE:N	2.33	0.43
2:B:515:PHE:CG	2:B:529:VAL:HG11	2.54	0.43
2:B:526:CYS:H	2:B:527:PRO:HD3	1.80	0.43
3:S:55:PRO:HB3	3:S:71:GLU:CG	2.48	0.43
4:M:220:GLU:HB2	4:M:222:PHE:CE1	2.51	0.43
4:M:331:LEU:HD12	4:M:331:LEU:C	2.38	0.43
1:A:349:ILE:O	1:A:350:SER:C	2.55	0.43
1:A:381:GLU:O	1:A:382:ASP:O	2.31	0.43
1:A:399:ASP:O	1:A:420:ILE:HB	2.18	0.43
1:A:606:PHE:CZ	1:A:633:PHE:HD1	2.32	0.43
1:A:629:LEU:O	1:A:631:SER:N	2.50	0.43
2:B:217:GLU:CD	4:M:133:GLU:OE1	2.54	0.43
2:B:292:GLU:CG	2:B:296:ASP:CB	2.96	0.43
2:B:424:PHE:HA	2:B:425:PRO:HD3	1.59	0.43
2:B:552:SER:CB	2:B:595:VAL:HG21	2.49	0.43
2:B:588:ILE:CG2	2:B:618:PHE:HZ	2.24	0.43
2:B:588:ILE:HD13	2:B:618:PHE:HE1	1.83	0.43
2:B:596:LEU:CD1	2:B:611:ALA:CB	2.89	0.43
2:B:602:ASP:O	2:B:608:ARG:NE	2.52	0.43
4:M:68:VAL:CG2	4:M:77:LEU:HD12	2.49	0.43
4:M:92:PHE:CE2	4:M:128:CYS:O	2.51	0.43
4:M:213:GLU:HA	4:M:465:LYS:O	2.18	0.43
4:M:222:PHE:HD1	4:M:222:PHE:N	2.16	0.43
4:M:262:THR:OG1	4:M:267:ILE:HG12	2.19	0.43
1:A:68:THR:OG1	3:S:166:LYS:HD2	2.19	0.43
1:A:88:ASN:CG	1:A:120:ILE:CG2	2.84	0.43
1:A:132:LEU:CD1	1:A:150:LEU:CD1	2.96	0.43
1:A:356:ILE:HG12	1:A:374:LEU:HD22	2.00	0.43
1:A:509:PRO:HB3	1:A:547:VAL:CG2	2.48	0.43
1:A:537:THR:HB	1:A:584:PHE:CE2	2.53	0.43
1:A:625:LEU:HD11	1:A:629:LEU:HB2	2.01	0.43
2:B:9:ALA:HB1	4:M:14:LEU:CD1	2.48	0.43
2:B:279:LEU:CG	2:B:288:TYR:HD2	2.13	0.43
2:B:400:SER:HB2	2:B:435:SER:C	2.39	0.43
2:B:440:GLY:HA3	2:B:478:LEU:HD22	2.00	0.43
3:S:93:GLU:HB3	3:S:98:ILE:HD11	2.00	0.43
4:M:126:ASN:C	4:M:128:CYS:H	2.22	0.43
4:M:244:VAL:HG13	4:M:472:TYR:OH	2.18	0.43
4:M:336:ASP:OD1	4:M:415:ILE:HB	2.17	0.43
1:A:427:LYS:O	1:A:430:ASN:HB2	2.19	0.43
1:A:635:ALA:C	1:A:638:LEU:H	2.22	0.43

Continued on next page...

Continued from previous page...

Atom-1	Atom-2	Interatomic distance (Å)	Clash overlap (Å)
2:B:162:VAL:O	2:B:164:ASP:N	2.52	0.43
2:B:208:ILE:O	2:B:212:VAL:HG23	2.18	0.43
2:B:223:LEU:CD1	2:B:259:TYR:H	2.05	0.43
2:B:302:PHE:CE1	2:B:306:LEU:HD21	2.53	0.43
2:B:512:VAL:CG2	2:B:533:LEU:HD22	2.39	0.43
2:B:530:LEU:HD21	2:B:591:MET:HB3	2.00	0.43
2:B:592:TYR:CD2	2:B:618:PHE:CD2	3.06	0.43
3:S:58:LEU:HD12	3:S:68:VAL:HG13	2.00	0.43
4:M:243:ILE:HG21	4:M:298:ARG:HG2	2.01	0.43
4:M:267:ILE:HG22	4:M:302:TYR:CE2	2.53	0.43
4:M:290:PHE:CZ	4:M:293:PRO:CG	3.02	0.43
4:M:290:PHE:CB	4:M:299:LEU:CD1	2.88	0.43
4:M:443:SER:OG	4:M:447:ILE:O	2.23	0.43
1:A:244:LEU:HD13	1:A:256:LEU:CB	2.49	0.43
1:A:341:ILE:HG21	1:A:348:PHE:HD2	1.84	0.43
1:A:356:ILE:HD13	1:A:374:LEU:HB3	2.00	0.43
1:A:440:ASN:C	1:A:442:SER:H	2.18	0.43
2:B:1:MET:CG	4:M:39:PRO:HG2	2.38	0.43
2:B:70:MET:SD	2:B:107:ARG:CB	3.00	0.43
2:B:124:GLN:O	2:B:127:LEU:N	2.43	0.43
2:B:310:ILE:O	4:M:269:ILE:HD13	2.09	0.43
2:B:328:LEU:O	2:B:329:ALA:C	2.54	0.43
2:B:400:SER:CB	2:B:435:SER:C	2.87	0.43
2:B:589:SER:O	2:B:592:TYR:N	2.52	0.43
4:M:44:ASP:O	4:M:46:SER:N	2.51	0.43
4:M:219:LEU:HD12	4:M:440:ILE:HG12	1.98	0.43
4:M:269:ILE:N	4:M:302:TYR:CZ	2.86	0.43
4:M:353:VAL:O	4:M:401:LYS:CB	2.67	0.43
1:A:94:VAL:O	1:A:95:MET:C	2.50	0.43
1:A:370:LYS:O	1:A:374:LEU:HD12	2.17	0.43
1:A:461:CYS:SG	1:A:469:LEU:HB3	2.58	0.43
1:A:536:MET:SD	1:A:555:LEU:HD21	2.59	0.43
2:B:44:PRO:HG3	2:B:77:ILE:HD12	2.01	0.43
2:B:256:CYS:C	2:B:258:GLN:N	2.67	0.43
2:B:397:GLN:O	2:B:401:THR:HG23	2.18	0.43
2:B:451:MET:CG	2:B:489:ILE:HG12	2.23	0.43
2:B:479:VAL:HG12	2:B:486:HIS:CE1	2.24	0.43
2:B:513:TRP:HB2	2:B:551:LEU:HD11	2.01	0.43
2:B:564:LYS:NZ	2:B:621:GLY:O	2.51	0.43
4:M:262:THR:HG23	4:M:265:ASN:H	1.68	0.43
4:M:310:VAL:O	4:M:314:GLY:N	2.51	0.43

Continued on next page...

Continued from previous page...

Atom-1	Atom-2	Interatomic distance (Å)	Clash overlap (Å)
1:A:395:PHE:CZ	1:A:420:ILE:HD13	2.54	0.43
1:A:573:GLU:O	1:A:574:ILE:O	2.29	0.43
1:A:594:PHE:CE2	2:B:477:MET:CE	3.00	0.43
2:B:17:VAL:HG23	2:B:36:THR:OG1	2.19	0.43
2:B:73:ASP:HA	4:M:24:ALA:CB	2.49	0.43
2:B:75:ASP:C	2:B:77:ILE:H	2.20	0.43
2:B:105:LEU:O	2:B:108:PHE:N	2.52	0.43
2:B:143:SER:CB	2:B:179:LYS:CB	2.67	0.43
2:B:143:SER:O	2:B:179:LYS:HB3	2.19	0.43
2:B:158:VAL:HG12	2:B:177:ILE:HD11	2.01	0.43
2:B:181:TYR:HD2	2:B:218:CYS:HA	1.84	0.43
2:B:403:ILE:HG23	2:B:439:CYS:SG	2.58	0.43
4:M:66:PHE:HB3	4:M:77:LEU:CD1	2.48	0.43
4:M:217:ASP:N	4:M:472:TYR:OH	2.52	0.43
4:M:242:GLY:HA3	4:M:444:ALA:CB	2.47	0.43
1:A:264:SER:HB2	1:A:271:ARG:HG3	2.00	0.42
1:A:356:ILE:O	1:A:359:LEU:N	2.46	0.42
1:A:500:SER:HB3	1:A:535:ILE:HD13	2.01	0.42
1:A:571:ARG:O	1:A:574:ILE:N	2.47	0.42
2:B:107:ARG:NH2	4:M:126:ASN:CA	2.79	0.42
2:B:112:ASP:OD2	2:B:115:LEU:HD23	2.18	0.42
2:B:158:VAL:CG1	2:B:173:VAL:CG1	2.92	0.42
2:B:241:ASP:C	2:B:243:TRP:H	2.21	0.42
2:B:396:ILE:HG22	2:B:435:SER:CB	2.45	0.42
2:B:537:PHE:CZ	2:B:545:ARG:HD3	2.54	0.42
2:B:564:LYS:HG3	2:B:568:VAL:CG2	2.42	0.42
3:S:16:LEU:HD12	3:S:17:VAL:N	2.34	0.42
4:M:234:ARG:O	4:M:236:LEU:N	2.51	0.42
4:M:455:VAL:HG12	4:M:455:VAL:O	2.19	0.42
1:A:140:VAL:HG22	1:A:177:ILE:CG1	2.47	0.42
1:A:192:TYR:CD2	1:A:192:TYR:O	2.72	0.42
1:A:356:ILE:CD1	1:A:374:LEU:HD23	2.49	0.42
1:A:395:PHE:CZ	1:A:428:MET:CB	3.02	0.42
1:A:398:GLU:C	1:A:418:ILE:HG13	2.39	0.42
1:A:581:LEU:CB	1:A:607:LEU:HD13	2.41	0.42
2:B:38:TYR:C	2:B:40:GLN:H	2.23	0.42
2:B:43:ASN:CB	2:B:44:PRO:CD	2.95	0.42
2:B:167:ALA:C	2:B:207:VAL:HG21	2.39	0.42
2:B:169:VAL:C	2:B:173:VAL:HG23	2.39	0.42
2:B:343:LEU:HG	2:B:363:ILE:HD11	2.00	0.42
4:M:103:TYR:HB2	4:M:104:PHE:CD1	2.54	0.42

Continued on next page...

Continued from previous page...

Atom-1	Atom-2	Interatomic distance (Å)	Clash overlap (Å)
4:M:246:VAL:HB	4:M:297:PHE:CE2	2.53	0.42
4:M:443:SER:HB3	4:M:447:ILE:H	1.80	0.42
4:M:479:PHE:HD1	4:M:479:PHE:N	1.98	0.42
1:A:192:TYR:HD2	1:A:195:ALA:H	1.68	0.42
1:A:247:ILE:HG21	1:A:252:ILE:HB	2.00	0.42
1:A:304:LEU:HD12	1:A:312:ALA:HB2	2.01	0.42
1:A:372:ILE:O	1:A:375:VAL:HB	2.19	0.42
2:B:315:PRO:HG3	2:B:350:THR:CG2	2.48	0.42
3:S:8:PHE:CZ	3:S:84:TYR:CB	2.97	0.42
4:M:219:LEU:HG	4:M:439:TYR:O	2.19	0.42
4:M:262:THR:HG22	4:M:264:GLY:H	1.81	0.42
4:M:469:GLY:C	4:M:470:ALA:O	2.56	0.42
1:A:63:ASP:O	1:A:67:LYS:HG3	2.19	0.42
1:A:213:SER:CB	3:S:142:ILE:C	2.87	0.42
1:A:327:ASP:OD1	3:S:50:PHE:HE2	2.02	0.42
1:A:406:GLY:C	3:S:64:ASN:CG	2.69	0.42
2:B:22:ALA:HA	2:B:32:GLU:HB2	1.75	0.42
2:B:195:ILE:C	2:B:197:LYS:H	2.21	0.42
2:B:343:LEU:CG	2:B:363:ILE:CD1	2.97	0.42
2:B:343:LEU:HD11	2:B:362:ALA:HB3	2.00	0.42
2:B:464:SER:O	2:B:468:LEU:HG	2.19	0.42
2:B:513:TRP:CB	2:B:551:LEU:HD21	2.50	0.42
2:B:537:PHE:HD1	2:B:538:SER:N	2.17	0.42
2:B:580:TYR:CB	2:B:582:ASP:OD2	2.64	0.42
3:S:9:ASN:ND2	3:S:118:GLU:OE1	2.53	0.42
4:M:69:ILE:HD11	4:M:94:GLU:N	2.34	0.42
4:M:74:TYR:HB2	4:M:114:ILE:HD13	2.00	0.42
4:M:213:GLU:H	4:M:249:TYR:HB2	1.84	0.42
4:M:290:PHE:CE2	4:M:297:PHE:CE2	3.04	0.42
4:M:327:PHE:HE1	4:M:336:ASP:CG	2.22	0.42
1:A:231:GLN:N	1:A:232:PRO:HD2	2.35	0.42
1:A:338:PHE:HE1	1:A:352:PHE:CE2	2.38	0.42
1:A:412:LYS:O	1:A:414:LYS:N	2.51	0.42
1:A:447:PHE:CD2	1:A:484:VAL:HG13	2.54	0.42
2:B:9:ALA:HB1	4:M:14:LEU:CG	2.45	0.42
2:B:108:PHE:HE1	2:B:112:ASP:HB3	1.84	0.42
2:B:139:LEU:HD23	2:B:172:GLU:C	2.39	0.42
2:B:253:ILE:HD11	2:B:324:ALA:CA	2.50	0.42
2:B:274:PRO:CG	2:B:295:ASN:HB3	2.37	0.42
2:B:343:LEU:CD2	2:B:366:LEU:CD1	2.81	0.42
2:B:418:TYR:CE1	2:B:419:VAL:HA	2.54	0.42

Continued on next page...

Continued from previous page...

Atom-1	Atom-2	Interatomic distance (Å)	Clash overlap (Å)
2:B:457:HIS:ND1	2:B:461:HIS:NE2	2.67	0.42
2:B:458:MET:HG3	2:B:471:TYR:CD1	2.55	0.42
4:M:74:TYR:CE2	4:M:109:LEU:HB2	2.55	0.42
4:M:323:MET:CE	4:M:342:LEU:CB	2.93	0.42
4:M:360:LEU:CD1	4:M:433:VAL:HG23	2.50	0.42
1:A:88:ASN:ND2	1:A:120:ILE:HG21	2.34	0.42
1:A:176:TYR:CD1	3:S:142:ILE:HD11	2.54	0.42
1:A:368:ARG:NH1	1:A:419:ILE:O	2.53	0.42
1:A:522:PHE:C	1:A:524:THR:N	2.72	0.42
2:B:127:LEU:HD23	2:B:135:ARG:O	2.20	0.42
2:B:374:PHE:HE2	2:B:398:ILE:CG2	2.26	0.42
2:B:405:GLU:HA	2:B:446:TRP:CD1	2.55	0.42
2:B:458:MET:HA	2:B:463:LEU:CD1	2.47	0.42
2:B:513:TRP:N	2:B:551:LEU:HD11	2.33	0.42
2:B:513:TRP:CD1	2:B:517:GLU:HG3	2.54	0.42
2:B:588:ILE:HD13	2:B:618:PHE:CE1	2.54	0.42
4:M:223:HIS:N	4:M:479:PHE:CZ	2.83	0.42
4:M:462:LYS:HD2	4:M:462:LYS:HA	2.01	0.42
1:A:71:VAL:HG11	1:A:105:VAL:CG1	2.50	0.42
1:A:353:ASP:O	1:A:356:ILE:HB	2.19	0.42
1:A:413:SER:HB3	1:A:415:ARG:O	2.19	0.42
2:B:18:ILE:HG21	2:B:36:THR:C	2.32	0.42
2:B:105:LEU:HB3	2:B:145:MET:HE3	2.02	0.42
2:B:119:SER:O	2:B:123:LEU:CG	2.56	0.42
2:B:159:LYS:HA	2:B:195:ILE:HD13	2.01	0.42
2:B:373:LEU:O	2:B:375:LEU:N	2.52	0.42
2:B:429:VAL:HG11	2:B:463:LEU:HD22	2.00	0.42
2:B:510:GLY:O	2:B:513:TRP:HB3	2.19	0.42
2:B:567:GLN:N	2:B:574:ASN:ND2	2.67	0.42
2:B:568:VAL:C	2:B:571:SER:OG	2.57	0.42
3:S:53:THR:CG2	3:S:69:ASN:HB2	2.49	0.42
3:S:58:LEU:O	3:S:59:LEU:C	2.56	0.42
3:S:89:VAL:HG21	3:S:98:ILE:HG13	2.00	0.42
4:M:110:SER:C	4:M:112:LYS:N	2.73	0.42
4:M:120:ARG:C	4:M:124:ILE:HD12	2.39	0.42
4:M:222:PHE:CB	4:M:479:PHE:CE2	3.03	0.42
4:M:241:HIS:C	4:M:474:THR:HB	2.36	0.42
4:M:435:LEU:HD22	4:M:437:TYR:OH	2.20	0.42
1:A:95:MET:HB3	1:A:127:LEU:HD23	2.01	0.42
1:A:225:LEU:CD1	1:A:233:PHE:CE1	2.92	0.42
1:A:357:ILE:O	1:A:358:ARG:C	2.57	0.42

Continued on next page...

Continued from previous page...

Atom-1	Atom-2	Interatomic distance (Å)	Clash overlap (Å)
1:A:416:ILE:HG22	1:A:417:PRO:O	2.20	0.42
1:A:561:ASN:O	1:A:562:TRP:C	2.57	0.42
2:B:13:ASP:HB3	2:B:35:TYR:HE2	1.85	0.42
2:B:158:VAL:HA	2:B:173:VAL:HG13	2.01	0.42
2:B:178:ILE:HG22	2:B:179:LYS:N	2.34	0.42
2:B:196:LEU:CA	2:B:215:TYR:OH	2.66	0.42
2:B:405:GLU:OE2	2:B:445:SER:HB2	2.20	0.42
2:B:574:ASN:O	2:B:575:ASN:C	2.50	0.42
4:M:220:GLU:OE2	4:M:350:VAL:HG13	2.19	0.42
4:M:316:ARG:O	4:M:318:ASN:N	2.53	0.42
4:M:351:SER:HB2	4:M:441:GLY:C	2.40	0.42
4:M:362:PHE:C	4:M:363:ASN:O	2.55	0.42
1:A:466:ASP:OD1	1:A:468:SER:N	2.48	0.42
1:A:546:SER:O	1:A:550:VAL:HG23	2.18	0.42
2:B:38:TYR:CZ	2:B:43:ASN:CA	2.98	0.42
2:B:40:GLN:OE1	2:B:40:GLN:HA	2.20	0.42
2:B:176:ALA:O	2:B:179:LYS:N	2.52	0.42
2:B:310:ILE:HG21	2:B:342:ALA:C	2.40	0.42
2:B:486:HIS:NE2	2:B:518:ILE:CG1	2.83	0.42
3:S:5:VAL:CG1	3:S:87:PHE:CE2	3.03	0.42
3:S:5:VAL:HG22	3:S:87:PHE:CD2	2.54	0.42
4:M:103:TYR:N	4:M:103:TYR:HD1	2.18	0.42
4:M:215:TYR:CG	4:M:467:TYR:O	2.70	0.42
4:M:245:ASP:OD1	4:M:298:ARG:N	2.53	0.42
4:M:292:PRO:HA	4:M:293:PRO:HD3	1.55	0.42
4:M:379:LEU:CD2	4:M:411:LEU:CD2	2.98	0.42
1:A:78:GLU:C	1:A:80:TYR:O	2.58	0.42
1:A:103:LYS:O	1:A:107:TYR:CG	2.71	0.42
1:A:140:VAL:HG22	1:A:177:ILE:CD1	2.50	0.42
1:A:259:LEU:HD23	1:A:259:LEU:C	2.40	0.42
1:A:512:LEU:HD13	1:A:543:TYR:CE1	2.55	0.42
2:B:31:GLY:HA2	2:B:65:ARG:HH12	1.85	0.42
2:B:83:PHE:O	2:B:86:VAL:HB	2.19	0.42
2:B:172:GLU:O	2:B:175:LEU:N	2.53	0.42
2:B:261:PRO:C	2:B:290:SER:HB3	2.41	0.42
2:B:262:LYS:O	2:B:264:THR:N	2.53	0.42
2:B:307:ASN:HD21	2:B:338:LYS:HB2	1.85	0.42
2:B:393:ILE:HG23	2:B:431:MET:HB2	2.01	0.42
2:B:403:ILE:HB	2:B:408:VAL:CG2	2.49	0.42
2:B:412:PHE:HB3	2:B:453:TRP:HD1	1.84	0.42
2:B:433:VAL:HG22	2:B:471:TYR:CE1	2.53	0.42

Continued on next page...

Continued from previous page...

Atom-1	Atom-2	Interatomic distance (Å)	Clash overlap (Å)
2:B:497:LEU:HB2	2:B:511:ILE:HD13	2.02	0.42
2:B:512:VAL:HB	2:B:551:LEU:HD12	2.02	0.42
2:B:556:LEU:CB	2:B:588:ILE:CD1	2.74	0.42
2:B:562:ASN:ND2	2:B:580:TYR:CD2	2.88	0.42
4:M:217:ASP:CG	4:M:470:ALA:O	2.58	0.42
4:M:218:LEU:HD12	4:M:218:LEU:H	1.83	0.42
4:M:290:PHE:CZ	4:M:293:PRO:HG3	2.55	0.42
4:M:323:MET:HE3	4:M:437:TYR:HE2	1.85	0.42
4:M:405:THR:HG23	4:M:406:GLY:N	2.35	0.42
1:A:264:SER:HB2	1:A:271:ARG:CD	2.49	0.41
1:A:356:ILE:HG12	1:A:374:LEU:CD2	2.50	0.41
1:A:423:ASN:CG	3:S:62:GLU:OE1	2.58	0.41
1:A:513:ARG:O	1:A:516:ILE:HB	2.19	0.41
2:B:20:ARG:CZ	4:M:118:TYR:CB	2.75	0.41
2:B:212:VAL:O	2:B:251:LEU:HD22	2.20	0.41
2:B:475:ILE:HG22	2:B:514:LEU:HD21	2.02	0.41
2:B:534:ILE:C	2:B:536:ASN:H	2.22	0.41
2:B:560:ILE:HA	2:B:563:PHE:CB	2.38	0.41
3:S:85:PHE:CZ	3:S:109:LEU:HD23	2.55	0.41
4:M:245:ASP:HB3	4:M:472:TYR:CE1	2.54	0.41
4:M:304:VAL:HG11	4:M:444:ALA:O	2.19	0.41
1:A:92:LEU:CD1	1:A:123:LEU:HD12	2.50	0.41
1:A:95:MET:SD	1:A:107:TYR:CE1	3.13	0.41
1:A:189:PHE:O	1:A:191:GLN:N	2.53	0.41
1:A:617:ASP:CG	1:A:618:THR:H	2.19	0.41
2:B:90:ILE:HA	2:B:101:ILE:CD1	2.50	0.41
2:B:108:PHE:CE1	2:B:112:ASP:HB3	2.55	0.41
2:B:151:ALA:N	2:B:152:PRO:CD	2.82	0.41
2:B:253:ILE:HG13	2:B:324:ALA:CB	2.50	0.41
2:B:268:LYS:O	2:B:273:SER:HB3	2.20	0.41
2:B:275:ARG:H	2:B:294:VAL:CG1	2.27	0.41
2:B:395:LYS:O	2:B:398:ILE:HB	2.20	0.41
3:S:53:THR:CG2	3:S:68:VAL:CA	2.90	0.41
4:M:424:PHE:CE1	4:M:427:LYS:O	2.73	0.41
1:A:304:LEU:HA	1:A:308:ASP:HB2	2.02	0.41
1:A:323:CYS:HG	1:A:355:LEU:HD21	1.83	0.41
1:A:371:ALA:O	1:A:374:LEU:HB2	2.20	0.41
1:A:429:VAL:HG11	1:A:469:LEU:HD11	2.00	0.41
2:B:10:SER:C	2:B:40:GLN:NE2	2.70	0.41
2:B:43:ASN:HA	2:B:44:PRO:HD3	1.68	0.41
2:B:72:SER:C	4:M:17:GLN:NE2	2.59	0.41

Continued on next page...

Continued from previous page...

Atom-1	Atom-2	Interatomic distance (Å)	Clash overlap (Å)
2:B:120:ILE:HG23	2:B:142:LEU:CD2	2.50	0.41
2:B:295:ASN:C	2:B:296:ASP:O	2.58	0.41
2:B:332:LEU:HG	2:B:332:LEU:O	2.19	0.41
2:B:347:VAL:CB	2:B:359:LEU:HB3	2.42	0.41
2:B:486:HIS:O	2:B:489:ILE:N	2.52	0.41
2:B:610:ARG:O	2:B:614:ILE:HG13	2.21	0.41
3:S:7:ILE:HG23	3:S:85:PHE:CD2	2.55	0.41
4:M:5:PHE:HA	4:M:78:ALA:HA	2.02	0.41
1:A:394:GLN:O	1:A:397:ASP:N	2.52	0.41
1:A:448:GLU:HB2	1:A:487:MET:SD	2.60	0.41
2:B:14:THR:O	2:B:18:ILE:HG12	2.17	0.41
2:B:90:ILE:O	2:B:98:LYS:HE2	2.21	0.41
2:B:219:TYR:CE2	2:B:226:LEU:HA	2.49	0.41
2:B:295:ASN:O	2:B:300:ASP:HB3	1.99	0.41
2:B:334:MET:O	2:B:335:LYS:C	2.56	0.41
3:S:87:PHE:CZ	3:S:102:ILE:HG12	2.55	0.41
4:M:16:PHE:HD1	4:M:118:TYR:CD2	2.37	0.41
4:M:52:ASP:O	4:M:53:HIS:HB3	2.21	0.41
4:M:422:PRO:C	4:M:424:PHE:H	2.24	0.41
1:A:460:LEU:C	1:A:462:GLN:H	2.23	0.41
1:A:555:LEU:CD1	1:A:581:LEU:HD11	2.48	0.41
1:A:556:VAL:CG2	1:A:603:VAL:HG22	2.30	0.41
2:B:513:TRP:CA	2:B:551:LEU:HD11	2.47	0.41
3:S:4:ALA:HB1	3:S:19:PHE:CE1	2.55	0.41
3:S:16:LEU:HD13	3:S:129:GLU:CG	2.50	0.41
3:S:51:LEU:HB2	3:S:77:TYR:HE1	1.84	0.41
4:M:322:LEU:HD23	4:M:322:LEU:C	2.41	0.41
4:M:323:MET:HG3	4:M:342:LEU:HG	2.01	0.41
4:M:341:SER:CB	4:M:343:ASN:HD21	2.24	0.41
4:M:374:TYR:O	4:M:390:ILE:HG23	2.19	0.41
4:M:374:TYR:H	4:M:390:ILE:HG23	1.84	0.41
4:M:434:SER:HB3	4:M:478:ASN:OD1	2.20	0.41
1:A:132:LEU:O	1:A:134:TYR:N	2.54	0.41
1:A:222:ILE:HG23	1:A:233:PHE:CD1	2.55	0.41
1:A:516:ILE:HD13	1:A:551:LEU:HA	2.02	0.41
1:A:530:ASN:O	1:A:534:LYS:N	2.54	0.41
2:B:34:SER:HG	2:B:35:TYR:N	2.19	0.41
2:B:115:LEU:HA	2:B:115:LEU:HD13	1.85	0.41
2:B:123:LEU:HB2	2:B:142:LEU:HD21	2.02	0.41
2:B:374:PHE:HB3	2:B:402:LEU:HD23	1.02	0.41
2:B:430:ILE:HG12	2:B:467:VAL:N	2.35	0.41

Continued on next page...

Continued from previous page...

Atom-1	Atom-2	Interatomic distance (Å)	Clash overlap (Å)
2:B:537:PHE:CZ	2:B:545:ARG:HB3	2.50	0.41
4:M:119:ASP:C	4:M:121:ILE:H	2.24	0.41
4:M:220:GLU:CG	4:M:222:PHE:CE1	3.04	0.41
4:M:222:PHE:CZ	4:M:240:ILE:HD13	2.56	0.41
4:M:372:ILE:HD12	4:M:428:VAL:HG22	2.03	0.41
1:A:326:GLN:HA	1:A:331:ARG:NH2	2.36	0.41
1:A:373:GLU:HG2	1:A:427:LYS:CE	2.51	0.41
1:A:381:GLU:O	1:A:383:ASN:N	2.48	0.41
1:A:451:ASN:HD21	1:A:487:MET:HB3	1.85	0.41
2:B:75:ASP:OD1	4:M:19:LEU:HD21	2.21	0.41
2:B:116:THR:HG21	2:B:147:MET:HG3	2.03	0.41
2:B:179:LYS:HE3	4:M:131:ALA:O	2.20	0.41
2:B:241:ASP:O	2:B:243:TRP:N	2.50	0.41
2:B:435:SER:C	2:B:437:SER:H	2.22	0.41
2:B:472:VAL:CG2	2:B:511:ILE:HG13	2.50	0.41
2:B:493:LEU:C	2:B:495:ASP:N	2.74	0.41
2:B:596:LEU:HD22	2:B:615:SER:CB	2.50	0.41
3:S:7:ILE:HD13	3:S:16:LEU:HB3	2.01	0.41
3:S:87:PHE:CD1	3:S:87:PHE:N	2.88	0.41
4:M:103:TYR:O	4:M:104:PHE:HB2	2.21	0.41
4:M:119:ASP:C	4:M:121:ILE:N	2.74	0.41
4:M:212:ASN:O	4:M:465:LYS:CB	2.65	0.41
1:A:182:ILE:HG22	1:A:221:VAL:HG23	2.00	0.41
1:A:393:LYS:O	1:A:394:GLN:C	2.58	0.41
1:A:451:ASN:OD1	1:A:480:LEU:CD1	2.68	0.41
1:A:529:GLY:CA	1:A:562:TRP:HZ2	2.34	0.41
2:B:136:CYS:SG	2:B:168:MET:O	2.78	0.41
2:B:208:ILE:O	2:B:209:SER:C	2.58	0.41
2:B:230:PHE:HB3	2:B:298:ASP:CG	2.41	0.41
2:B:267:ASP:CA	2:B:289:PRO:HG3	2.50	0.41
2:B:301:LEU:O	2:B:305:SER:CB	2.68	0.41
2:B:367:SER:OG	2:B:401:THR:CB	2.68	0.41
2:B:508:ARG:HD3	2:B:540:GLU:OE2	2.21	0.41
2:B:513:TRP:HA	2:B:551:LEU:CG	2.50	0.41
2:B:577:ASN:OD1	2:B:577:ASN:N	2.53	0.41
2:B:588:ILE:O	2:B:591:MET:HB2	2.21	0.41
3:S:137:GLN:O	3:S:140:MET:HB3	2.20	0.41
4:M:103:TYR:CD1	4:M:103:TYR:N	2.89	0.41
4:M:372:ILE:HD11	4:M:428:VAL:HG13	2.03	0.41
4:M:386:PHE:HB3	4:M:397:TRP:HD1	1.84	0.41
1:A:150:LEU:C	1:A:153:ILE:H	2.22	0.41

Continued on next page...

Continued from previous page...

Atom-1	Atom-2	Interatomic distance (Å)	Clash overlap (Å)
1:A:182:ILE:HD13	1:A:218:ALA:HB2	2.02	0.41
1:A:349:ILE:O	1:A:352:PHE:N	2.48	0.41
1:A:402:ILE:O	1:A:403:LEU:C	2.56	0.41
1:A:461:CYS:SG	1:A:464:ILE:CG2	3.09	0.41
1:A:516:ILE:CD1	1:A:551:LEU:HB2	2.51	0.41
1:A:528:ASN:C	1:A:530:ASN:N	2.68	0.41
1:A:530:ASN:ND2	1:A:571:ARG:NH2	2.69	0.41
2:B:56:SER:O	2:B:57:ARG:O	2.35	0.41
2:B:75:ASP:CG	4:M:24:ALA:N	2.70	0.41
2:B:83:PHE:HZ	2:B:105:LEU:HG	1.85	0.41
2:B:120:ILE:O	2:B:123:LEU:HB2	2.21	0.41
2:B:166:SER:O	2:B:170:ARG:HG3	2.21	0.41
2:B:177:ILE:HD11	2:B:195:ILE:CG2	2.51	0.41
2:B:181:TYR:HB3	2:B:218:CYS:SG	2.60	0.41
2:B:196:LEU:HD13	2:B:215:TYR:CE1	2.56	0.41
2:B:302:PHE:CZ	2:B:306:LEU:HD11	2.56	0.41
2:B:346:THR:HA	2:B:349:MET:CE	2.51	0.41
2:B:360:LEU:HD21	2:B:395:LYS:HE2	2.03	0.41
2:B:524:LYS:HG2	2:B:524:LYS:O	2.21	0.41
2:B:565:GLN:HB3	2:B:581:TYR:OH	2.21	0.41
3:S:39:ILE:HD11	3:S:77:TYR:CG	2.55	0.41
3:S:53:THR:CG2	3:S:67:GLU:C	2.89	0.41
3:S:87:PHE:CE1	3:S:102:ILE:HG23	2.55	0.41
3:S:135:ILE:O	3:S:140:MET:O	2.38	0.41
4:M:78:ALA:HB2	4:M:93:LEU:CG	2.50	0.41
4:M:96:ILE:O	4:M:99:ILE:HB	2.21	0.41
4:M:218:LEU:N	4:M:218:LEU:CD1	2.82	0.41
4:M:219:LEU:HB3	4:M:472:TYR:HB2	2.02	0.41
4:M:221:THR:CG2	4:M:223:HIS:CE1	3.04	0.41
4:M:224:VAL:O	4:M:479:PHE:HA	2.20	0.41
4:M:240:ILE:O	4:M:444:ALA:HB1	2.21	0.41
4:M:317:MET:SD	4:M:321:GLY:HA3	2.61	0.41
4:M:319:SER:OG	4:M:346:ASN:N	2.45	0.41
4:M:356:LEU:CD2	4:M:356:LEU:O	2.68	0.41
4:M:433:VAL:O	4:M:435:LEU:CD1	2.68	0.41
1:A:85:ALA:C	1:A:87:CYS:H	2.25	0.41
1:A:254:ILE:HD13	3:S:96:LEU:CB	2.46	0.41
1:A:420:ILE:HG23	1:A:424:TYR:CB	2.47	0.41
1:A:540:ILE:HG23	1:A:541:SER:N	2.35	0.41
2:B:35:TYR:O	2:B:42:ILE:CG1	2.69	0.41
2:B:100:LEU:CD2	4:M:123:LEU:HD21	2.51	0.41

Continued on next page...

Continued from previous page...

Atom-1	Atom-2	Interatomic distance (Å)	Clash overlap (Å)
2:B:107:ARG:NH1	4:M:20:LEU:CG	2.66	0.41
2:B:210:CYS:O	2:B:213:LEU:N	2.54	0.41
2:B:252:LEU:CG	2:B:302:PHE:CD1	2.99	0.41
2:B:325:LEU:HD22	2:B:339:PHE:CD1	2.56	0.41
2:B:359:LEU:HA	2:B:359:LEU:HD13	1.85	0.41
2:B:363:ILE:HB	2:B:398:ILE:HD11	2.01	0.41
2:B:375:LEU:O	2:B:376:PRO:C	2.32	0.41
3:S:135:ILE:O	3:S:141:VAL:CB	2.69	0.41
4:M:96:ILE:HD13	4:M:124:ILE:HG22	2.03	0.41
4:M:243:ILE:CB	4:M:472:TYR:HB3	2.51	0.41
4:M:270:PRO:HA	4:M:302:TYR:CD2	2.56	0.41
4:M:275:CYS:HB3	4:M:290:PHE:CE1	2.56	0.41
4:M:293:PRO:C	4:M:294:ASP:O	2.34	0.41
1:A:101:GLN:NE2	3:S:167:ILE:HD11	2.31	0.40
1:A:433:ILE:HG23	1:A:476:GLN:CG	2.50	0.40
1:A:496:ILE:O	1:A:499:ILE:HB	2.21	0.40
2:B:20:ARG:NH2	4:M:118:TYR:CD1	2.78	0.40
2:B:86:VAL:O	2:B:89:ASN:HB2	2.20	0.40
2:B:279:LEU:CG	2:B:288:TYR:CD2	2.94	0.40
2:B:396:ILE:CG2	2:B:432:ALA:CA	2.45	0.40
2:B:437:SER:C	2:B:439:CYS:H	2.24	0.40
2:B:477:MET:O	2:B:480:GLN:CB	2.63	0.40
2:B:486:HIS:CE1	2:B:518:ILE:CB	3.04	0.40
3:S:61:ASN:O	3:S:62:GLU:C	2.59	0.40
4:M:71:LYS:CG	4:M:74:TYR:CZ	3.05	0.40
4:M:223:HIS:HD2	4:M:478:ASN:HB2	1.86	0.40
4:M:280:ASP:CG	4:M:282:VAL:HG23	2.42	0.40
4:M:351:SER:HB2	4:M:441:GLY:HA3	2.01	0.40
1:A:128:LEU:HD22	1:A:146:ALA:HB1	2.02	0.40
1:A:249:ASN:OD1	1:A:251:TRP:N	2.54	0.40
2:B:204:ASP:HA	2:B:205:PRO:HD3	1.96	0.40
2:B:220:ALA:HA	2:B:258:GLN:CG	2.35	0.40
2:B:309:LEU:CB	2:B:317:VAL:HG12	2.25	0.40
2:B:347:VAL:O	2:B:349:MET:N	2.54	0.40
3:S:43:ASN:HB3	3:S:46:PHE:CD2	2.56	0.40
4:M:212:ASN:ND2	4:M:250:LEU:HA	2.36	0.40
4:M:222:PHE:CZ	4:M:439:TYR:HE2	2.35	0.40
4:M:302:TYR:HB3	4:M:447:ILE:HD13	2.03	0.40
4:M:360:LEU:CD1	4:M:433:VAL:CB	2.98	0.40
4:M:450:GLU:O	4:M:451:ALA:HB2	2.22	0.40
1:A:429:VAL:CG1	1:A:469:LEU:CD1	2.99	0.40

Continued on next page...

Continued from previous page...

Atom-1	Atom-2	Interatomic distance (Å)	Clash overlap (Å)
1:A:461:CYS:O	1:A:462:GLN:O	2.39	0.40
1:A:533:ILE:HG21	1:A:559:PHE:CZ	2.56	0.40
1:A:609:LEU:CG	1:A:628:VAL:HB	2.49	0.40
2:B:43:ASN:CB	2:B:44:PRO:HD2	2.50	0.40
2:B:127:LEU:CD2	2:B:139:LEU:HB2	2.51	0.40
2:B:143:SER:OG	2:B:179:LYS:CD	2.31	0.40
2:B:225:LEU:O	2:B:227:HIS:N	2.55	0.40
2:B:230:PHE:CG	2:B:298:ASP:HB3	2.52	0.40
2:B:326:TYR:CE2	2:B:369:LEU:HB2	2.57	0.40
2:B:356:LYS:O	2:B:360:LEU:HG	2.22	0.40
2:B:497:LEU:CD2	2:B:511:ILE:CG2	2.99	0.40
4:M:20:LEU:HD21	4:M:126:ASN:HA	2.03	0.40
4:M:100:LEU:HD21	4:M:121:ILE:HA	2.03	0.40
4:M:443:SER:CA	4:M:447:ILE:CG1	2.99	0.40
1:A:241:TYR:CE2	1:A:277:LYS:CB	3.05	0.40
1:A:244:LEU:CD2	1:A:277:LYS:HG3	2.51	0.40
1:A:580:GLU:O	1:A:583:GLU:N	2.54	0.40
1:A:614:LEU:HD21	1:A:621:LEU:HG	2.03	0.40
2:B:12:LEU:HB3	4:M:13:LYS:CD	2.46	0.40
2:B:216:LYS:HD3	2:B:251:LEU:CA	2.47	0.40
2:B:223:LEU:HD21	2:B:258:GLN:CB	2.50	0.40
2:B:309:LEU:CD1	2:B:317:VAL:HG11	2.49	0.40
2:B:392:SER:O	2:B:396:ILE:HG13	2.21	0.40
2:B:431:MET:C	2:B:433:VAL:N	2.73	0.40
2:B:537:PHE:C	2:B:539:ASN:N	2.75	0.40
3:S:37:GLU:O	3:S:40:SER:N	2.53	0.40
3:S:80:TYR:HE2	3:S:110:ASP:OD1	2.05	0.40
3:S:159:ALA:C	3:S:161:GLU:N	2.75	0.40
4:M:45:SER:CB	4:M:75:TRP:CZ2	2.95	0.40
4:M:64:LYS:HE3	4:M:79:SER:HB2	2.02	0.40
1:A:193:PRO:O	1:A:196:LEU:HB3	2.21	0.40
1:A:488:ARG:CD	1:A:522:PHE:CZ	3.04	0.40
1:A:488:ARG:HG2	1:A:522:PHE:HE2	1.85	0.40
2:B:108:PHE:HE1	2:B:112:ASP:HB2	1.87	0.40
2:B:231:ARG:C	2:B:233:TYR:H	2.25	0.40
2:B:249:ILE:O	2:B:253:ILE:HG13	2.21	0.40
2:B:478:LEU:O	2:B:480:GLN:N	2.53	0.40
2:B:549:LEU:HA	2:B:595:VAL:HG11	2.03	0.40
3:S:2:ILE:HG12	3:S:98:ILE:HD12	2.03	0.40
3:S:53:THR:O	3:S:69:ASN:OD1	2.40	0.40
3:S:80:TYR:CE2	3:S:110:ASP:OD1	2.75	0.40

Continued on next page...

Continued from previous page...

Atom-1	Atom-2	Interatomic distance (Å)	Clash overlap (Å)
3:S:110:ASP:O	3:S:114:THR:HA	2.21	0.40
4:M:47:SER:O	4:M:75:TRP:CH2	2.53	0.40
4:M:58:ARG:C	4:M:60:LEU:O	2.59	0.40
4:M:70:ASN:OD1	4:M:75:TRP:NE1	2.54	0.40
4:M:92:PHE:CZ	4:M:128:CYS:CB	2.91	0.40
4:M:389:SER:N	4:M:394:GLN:O	2.49	0.40
4:M:428:VAL:O	4:M:430:LEU:N	2.54	0.40

There are no symmetry-related clashes.

5.3 Torsion angles (i)

5.3.1 Protein backbone (i)

In the following table, the Percentiles column shows the percent Ramachandran outliers of the chain as a percentile score with respect to all PDB entries followed by that with respect to all EM entries.

The Analysed column shows the number of residues for which the backbone conformation was analysed, and the total number of residues.

Mol	Chain	Analysed	Favoured	Allowed	Outliers	Percentiles	
1	A	574/964 (60%)	529 (92%)	32 (6%)	13 (2%)	6	34
2	B	619/809 (76%)	500 (81%)	72 (12%)	47 (8%)	1	13
3	S	166/194 (86%)	160 (96%)	4 (2%)	2 (1%)	13	50
4	M	391/483 (81%)	315 (81%)	51 (13%)	25 (6%)	1	16
All	All	1750/2450 (71%)	1504 (86%)	159 (9%)	87 (5%)	4	20

All (87) Ramachandran outliers are listed below:

Mol	Chain	Res	Type
1	A	86	TRP
1	A	267	GLU
1	A	278	ILE
1	A	305	GLU
1	A	306	GLU
1	A	449	TRP
1	A	566	PHE
2	B	26	ALA

Continued on next page...

Continued from previous page...

Mol	Chain	Res	Type
2	B	70	MET
2	B	109	ALA
2	B	200	MET
2	B	242	SER
2	B	296	ASP
2	B	313	SER
2	B	436	LEU
2	B	523	PHE
2	B	558	TYR
2	B	560	ILE
2	B	561	ASP
2	B	564	LYS
2	B	565	GLN
2	B	568	VAL
3	S	72	ASP
3	S	164	ASP
4	M	51	LEU
4	M	84	LYS
4	M	104	PHE
4	M	106	LYS
4	M	280	ASP
4	M	306	LEU
4	M	405	THR
4	M	447	ILE
4	M	479	PHE
1	A	114	PHE
1	A	536	MET
2	B	182	ARG
2	B	215	TYR
2	B	228	GLY
2	B	232	ARG
2	B	259	TYR
2	B	274	PRO
2	B	347	VAL
2	B	353	GLN
2	B	372	THR
2	B	374	PHE
2	B	379	LYS
2	B	399	LEU
2	B	406	SER
2	B	600	LYS
4	M	231	SER

Continued on next page...

Continued from previous page...

Mol	Chain	Res	Type
4	M	279	ASN
4	M	316	ARG
4	M	321	GLY
4	M	429	ASP
1	A	116	LYS
1	A	174	ARG
1	A	279	LEU
2	B	36	THR
2	B	105	LEU
2	B	196	LEU
2	B	226	LEU
2	B	252	LEU
2	B	462	ASN
2	B	573	GLU
4	M	81	SER
4	M	356	LEU
4	M	363	ASN
4	M	463	ASN
4	M	470	ALA
2	B	75	ASP
2	B	177	ILE
2	B	194	ASP
2	B	199	LEU
2	B	231	ARG
2	B	535	GLN
4	M	44	ASP
4	M	263	MET
2	B	227	HIS
2	B	376	PRO
4	M	12	ASN
4	M	48	ASP
2	B	440	GLY
4	M	253	ASN
2	B	69	ILE
1	A	406	GLY
4	M	406	GLY
2	B	518	ILE

5.3.2 Protein sidechains (i)

In the following table, the Percentiles column shows the percent sidechain outliers of the chain as a percentile score with respect to all PDB entries followed by that with respect to all EM

entries.

The Analysed column shows the number of residues for which the sidechain conformation was analysed, and the total number of residues.

Mol	Chain	Analysed	Rotameric	Outliers	Percentiles	
1	A	536/898 (60%)	530 (99%)	6 (1%)	73	84
2	B	566/738 (77%)	561 (99%)	5 (1%)	78	87
3	S	159/175 (91%)	156 (98%)	3 (2%)	57	75
4	M	360/441 (82%)	351 (98%)	9 (2%)	47	68
All	All	1621/2252 (72%)	1598 (99%)	23 (1%)	68	80

All (23) residues with a non-rotameric sidechain are listed below:

Mol	Chain	Res	Type
1	A	175	PRO
1	A	186	PHE
1	A	196	LEU
1	A	241	TYR
1	A	292	TYR
1	A	418	ILE
2	B	233	TYR
2	B	259	TYR
2	B	418	TYR
2	B	453	TRP
2	B	592	TYR
3	S	8	PHE
3	S	38	LEU
3	S	113	PHE
4	M	6	TYR
4	M	118	TYR
4	M	214	LEU
4	M	250	LEU
4	M	343	ASN
4	M	379	LEU
4	M	437	TYR
4	M	472	TYR
4	M	479	PHE

Sometimes sidechains can be flipped to improve hydrogen bonding and reduce clashes. All (25) such sidechains are listed below:

Mol	Chain	Res	Type
1	A	101	GLN
1	A	102	GLN
1	A	199	ASN
1	A	634	ASN
2	B	40	GLN
2	B	41	ASN
2	B	46	GLN
2	B	222	HIS
2	B	229	HIS
2	B	258	GLN
2	B	304	GLN
2	B	333	GLN
2	B	397	GLN
2	B	404	ASN
2	B	441	GLN
2	B	461	HIS
2	B	486	HIS
2	B	574	ASN
3	S	64	ASN
3	S	65	ASN
3	S	126	GLN
4	M	70	ASN
4	M	265	ASN
4	M	343	ASN
4	M	346	ASN

5.3.3 RNA (i)

There are no RNA molecules in this entry.

5.4 Non-standard residues in protein, DNA, RNA chains (i)

There are no non-standard protein/DNA/RNA residues in this entry.

5.5 Carbohydrates (i)

There are no monosaccharides in this entry.

5.6 Ligand geometry (i)

There are no ligands in this entry.

5.7 Other polymers (i)

There are no such residues in this entry.

5.8 Polymer linkage issues (i)

The following chains have linkage breaks:

Mol	Chain	Number of breaks
4	M	33
3	S	11
1	A	9
2	B	6

All chain breaks are listed below:

Model	Chain	Residue-1	Atom-1	Residue-2	Atom-2	Distance (Å)
1	S	68:VAL	C	69:ASN	N	1.67
1	A	244:LEU	C	245:VAL	N	1.20
1	A	277:LYS	C	278:ILE	N	1.20
1	A	464:ILE	C	465:SER	N	1.20
1	B	259:TYR	C	260:LEU	N	1.20
1	S	60:SER	C	61:ASN	N	1.20
1	S	62:GLU	C	63:ASN	N	1.20
1	S	144:THR	C	145:ASN	N	1.20
1	M	6:TYR	C	7:ILE	N	1.20
1	M	252:ASP	C	253:ASN	N	1.20
1	M	280:ASP	C	281:GLY	N	1.20
1	A	378:ILE	C	379:VAL	N	1.19
1	A	439:ASP	C	440:ASN	N	1.19
1	A	535:ILE	C	536:MET	N	1.19
1	B	184:GLY	C	185:LYS	N	1.19
1	B	334:MET	C	335:LYS	N	1.19
1	S	113:PHE	C	114:THR	N	1.19
1	M	23:THR	C	24:ALA	N	1.19
1	M	82:LYS	C	83:SER	N	1.19
1	M	288:ILE	C	289:THR	N	1.19
1	M	377:LYS	C	378:ILE	N	1.19
1	A	289:SER	C	290:VAL	N	1.18
1	A	508:LEU	C	509:PRO	N	1.18
1	S	6:LEU	C	7:ILE	N	1.18
1	S	22:PRO	C	23:VAL	N	1.18
1	S	114:THR	C	115:GLU	N	1.18
1	M	56:VAL	C	57:GLY	N	1.18

Continued on next page...

Continued from previous page...

Model	Chain	Residue-1	Atom-1	Residue-2	Atom-2	Distance (Å)
1	M	64:LYS	C	65:TYR	N	1.18
1	M	136:VAL	C	137:SER	N	1.18
1	M	379:LEU	C	380:ARG	N	1.18
1	A	507:GLN	C	508:LEU	N	1.17
1	S	70:ASN	C	71:GLU	N	1.17
1	M	62:VAL	C	63:TYR	N	1.17
1	M	70:ASN	C	71:LYS	N	1.17
1	M	403:THR	C	404:ALA	N	1.17
1	M	450:GLU	C	451:ALA	N	1.17
1	S	8:PHE	C	9:ASN	N	1.16
1	M	134:PRO	C	135:ASN	N	1.16
1	M	279:ASN	C	280:ASP	N	1.16
1	M	319:SER	C	320:ILE	N	1.16
1	M	352:GLN	C	353:VAL	N	1.16
1	M	453:ASP	C	454:ILE	N	1.16
1	M	106:LYS	C	107:ASP	N	1.15
1	B	581:TYR	C	582:ASP	N	1.14
1	M	20:LEU	C	21:GLY	N	1.14
1	M	52:ASP	C	53:HIS	N	1.14
1	M	61:GLU	C	62:VAL	N	1.14
1	M	351:SER	C	352:GLN	N	1.14
1	B	284:ASN	C	285:GLU	N	1.13
1	B	293:VAL	C	294:VAL	N	1.13
1	M	21:GLY	C	22:ALA	N	1.13
1	M	63:TYR	C	64:LYS	N	1.13
1	M	130:GLU	C	131:ALA	N	1.12
1	M	80:THR	C	81:SER	N	1.11
1	M	105:ASP	C	106:LYS	N	1.10
1	M	3:LEU	C	4:SER	N	1.08
1	M	81:SER	C	82:LYS	N	1.08
1	S	48:SER	C	49:SER	N	1.03
1	M	135:ASN	C	136:VAL	N	0.93

6 Map visualisation (i)

This section contains visualisations of the EMDB entry EMD-13187. These allow visual inspection of the internal detail of the map and identification of artifacts.

No raw map or half-maps were deposited for this entry and therefore no images, graphs, etc. pertaining to the raw map can be shown.

6.1 Orthogonal projections (i)

6.1.1 Primary map

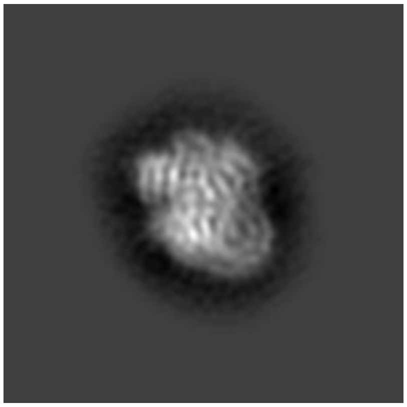

X

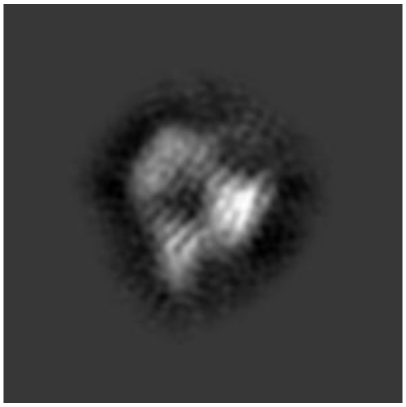

Y

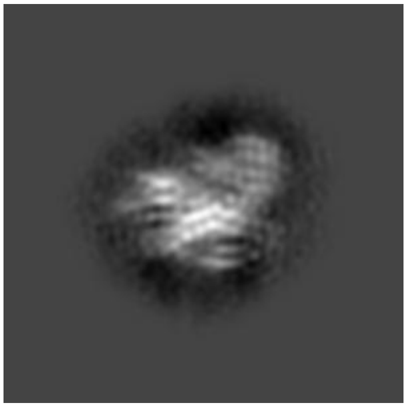

Z

The images above show the map projected in three orthogonal directions.

6.2 Central slices (i)

6.2.1 Primary map

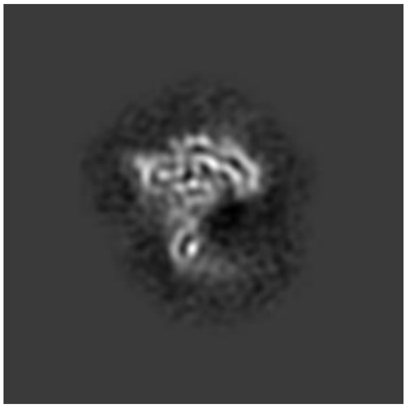

X Index: 132

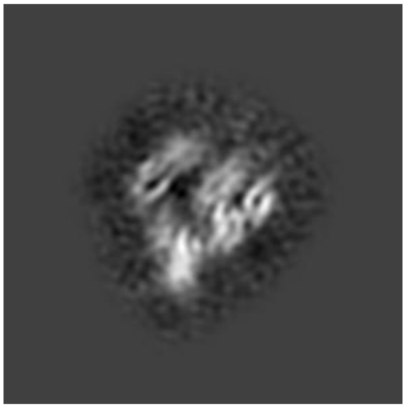

Y Index: 132

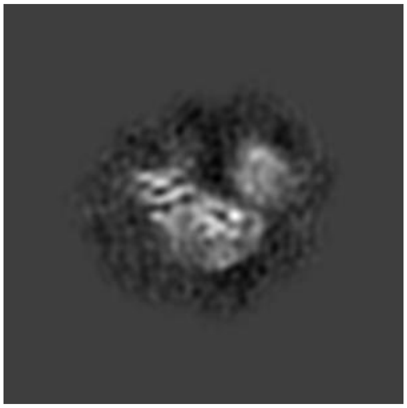

Z Index: 132

The images above show central slices of the map in three orthogonal directions.

6.3 Largest variance slices

6.3.1 Primary map

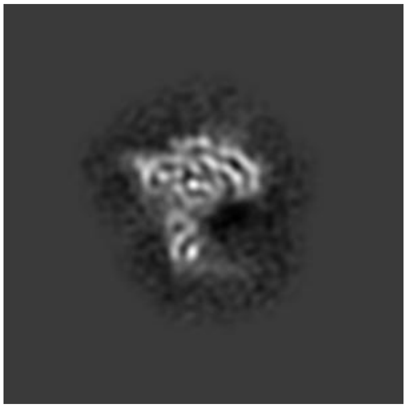
X Index: 130

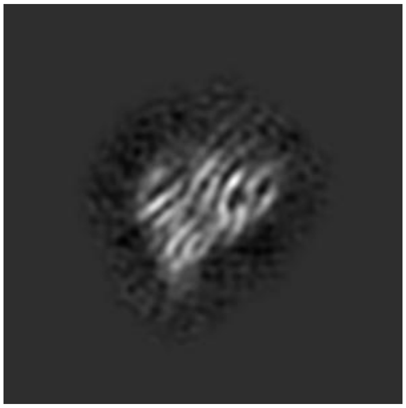
Y Index: 123

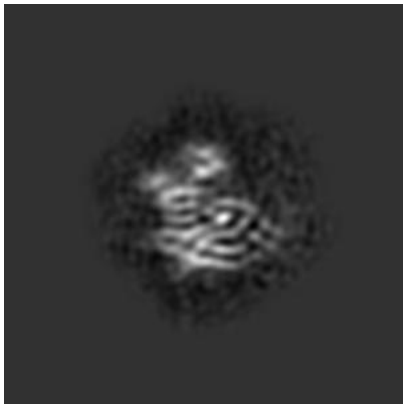
Z Index: 150

The images above show the largest variance slices of the map in three orthogonal directions.

6.4 Orthogonal surface views

6.4.1 Primary map

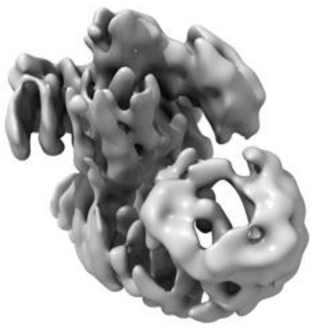
X

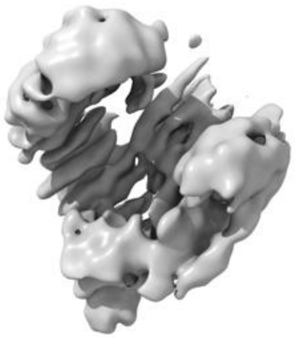
Y

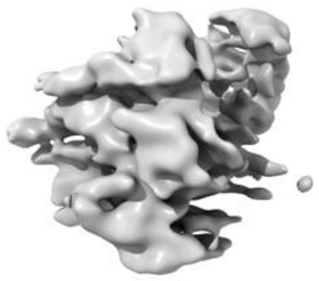
Z

The images above show the 3D surface view of the map at the recommended contour level 0.015. These images, in conjunction with the slice images, may facilitate assessment of whether an appropriate contour level has been provided.

6.5 Mask visualisation (i)

This section was not generated. No masks/segmentation were deposited.

7 Map analysis (i)

This section contains the results of statistical analysis of the map.

7.1 Map-value distribution (i)

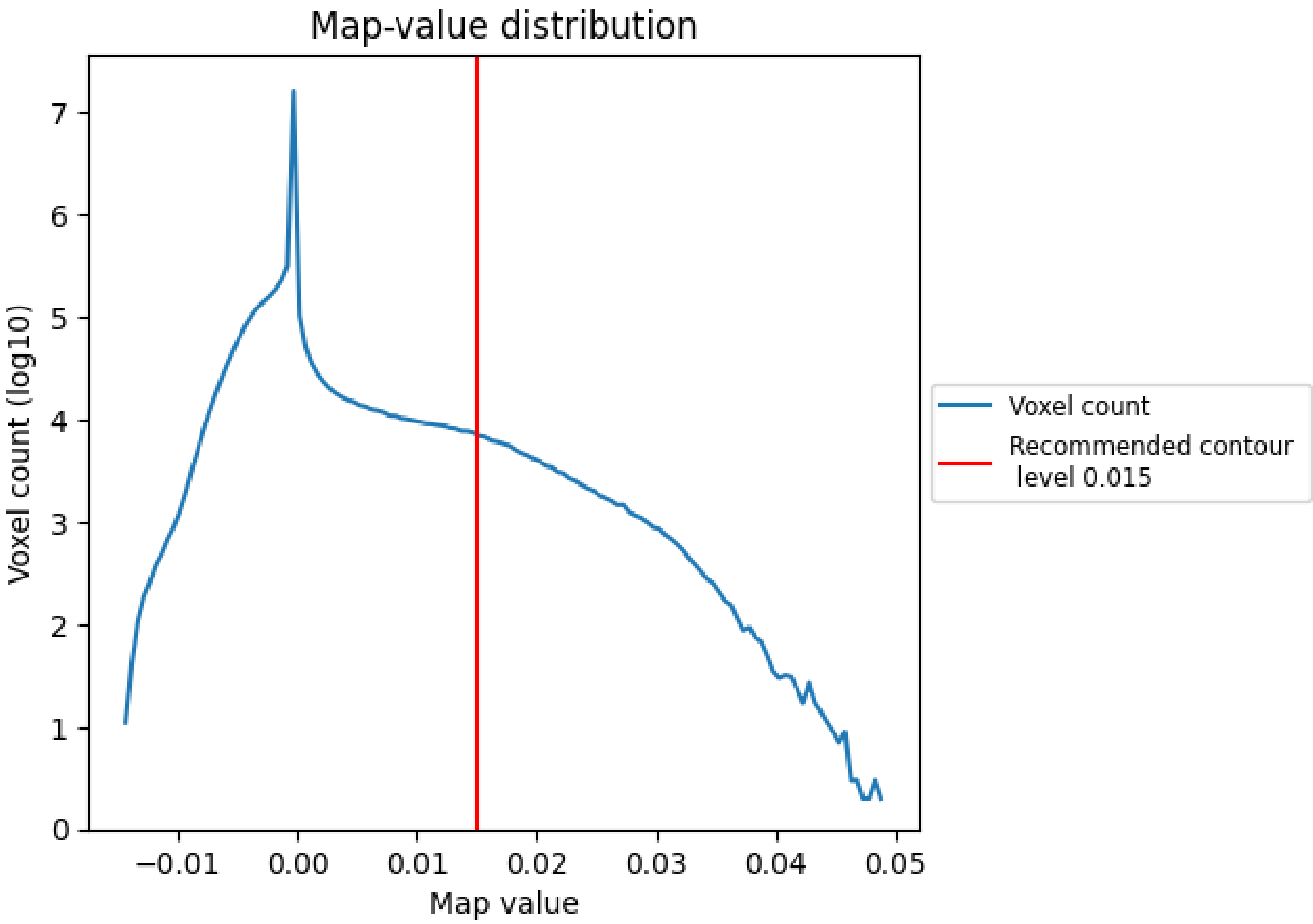

The map-value distribution is plotted in 128 intervals along the x-axis. The y-axis is logarithmic. A spike in this graph at zero usually indicates that the volume has been masked.

7.2 Volume estimate (i)

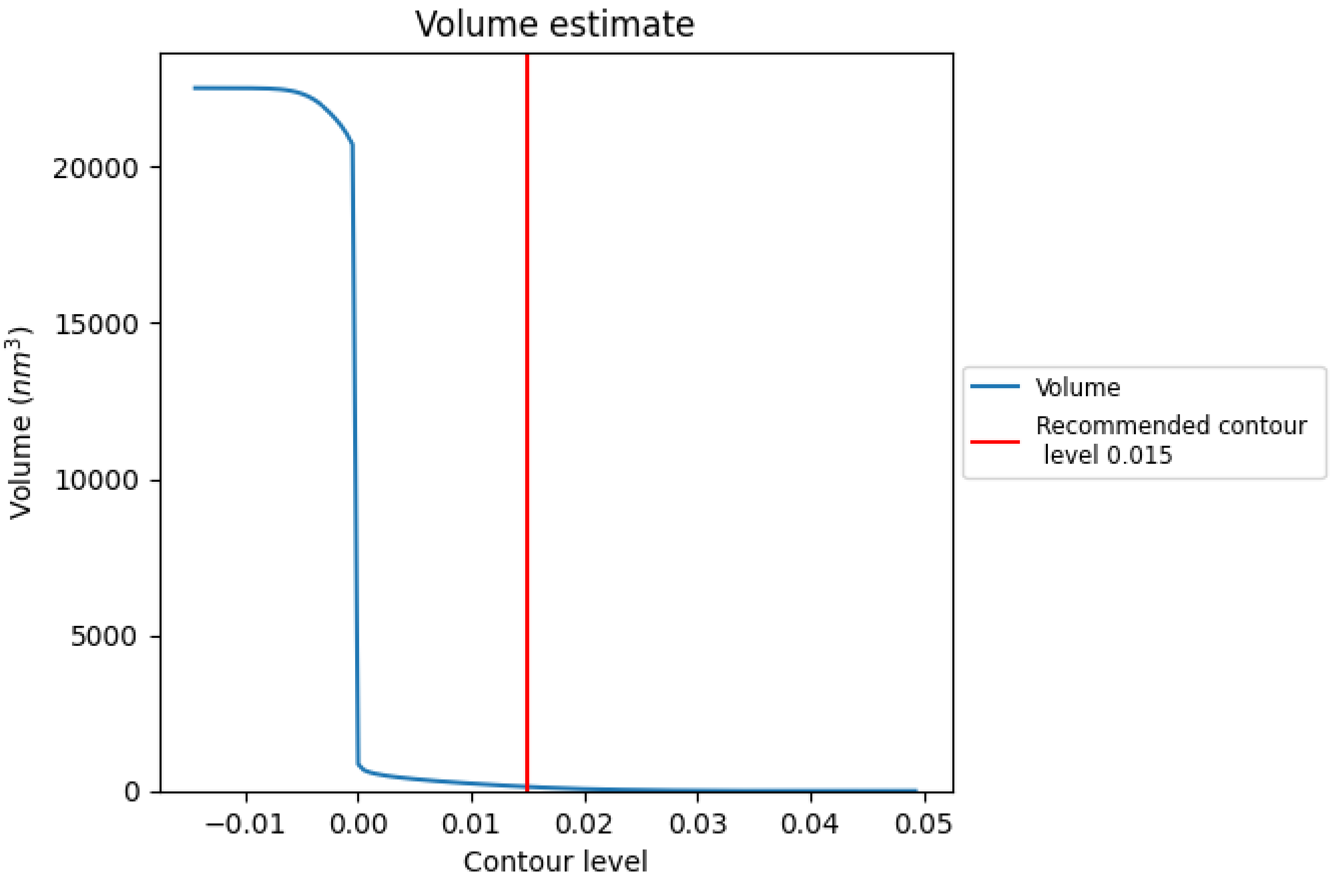

The volume at the recommended contour level is 131 nm^3; this corresponds to an approximate mass of 119 kDa.

The volume estimate graph shows how the enclosed volume varies with the contour level. The recommended contour level is shown as a vertical line and the intersection between the line and the curve gives the volume of the enclosed surface at the given level.

7.3 Rotationally averaged power spectrum ⓘ

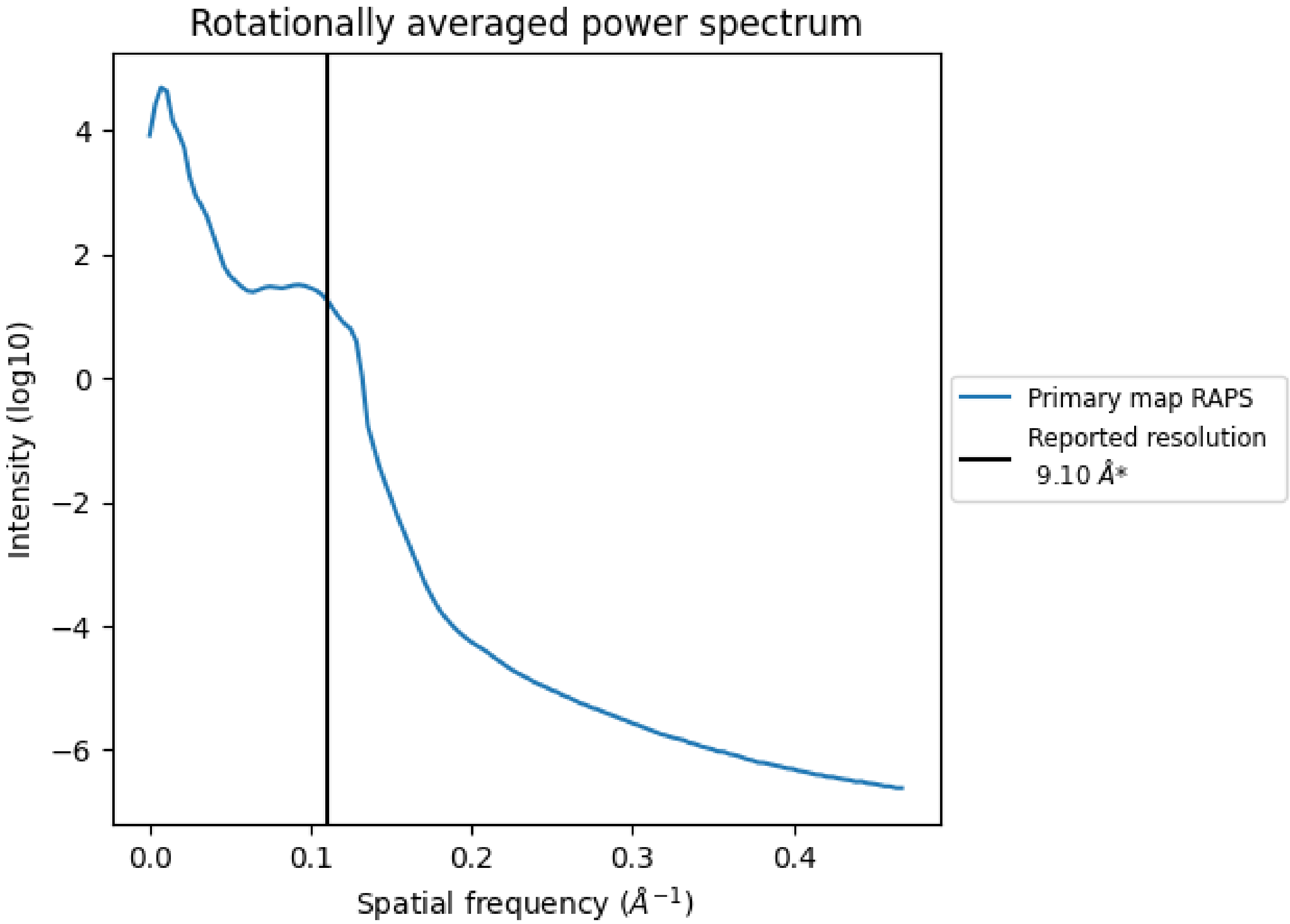

*Reported resolution corresponds to spatial frequency of 0.110 Å^{-1}

8 Fourier-Shell correlation (i)

This section was not generated. No FSC curve or half-maps provided.

9 Map-model fit (i)

This section contains information regarding the fit between EMDB map EMD-13187 and PDB model 7P3X. Per-residue inclusion information can be found in section 3 on page 5.

9.1 Map-model overlay (i)

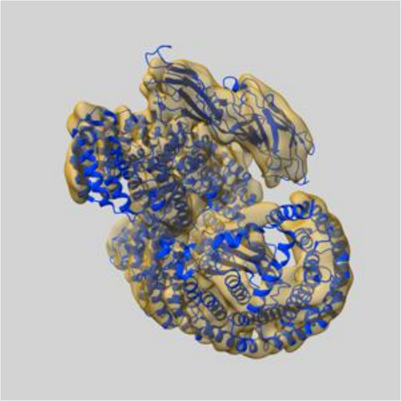

X

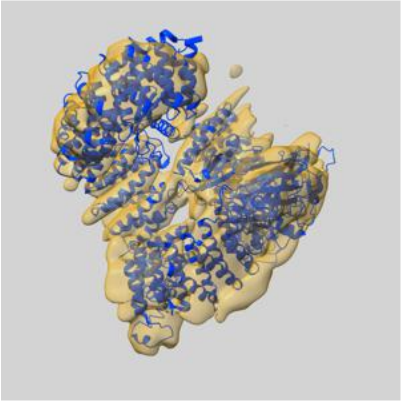

Y

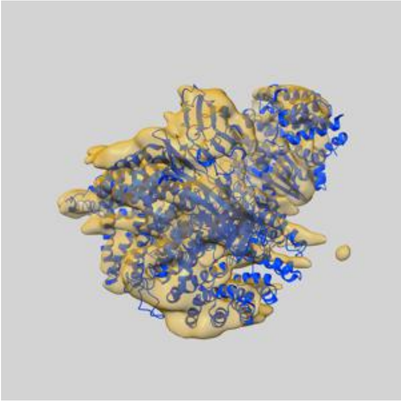

Z

The images above show the 3D surface view of the map at the recommended contour level 0.015 at 50% transparency in yellow overlaid with a ribbon representation of the model coloured in blue. These images allow for the visual assessment of the quality of fit between the atomic model and the map.

9.2 Atom inclusion (i)

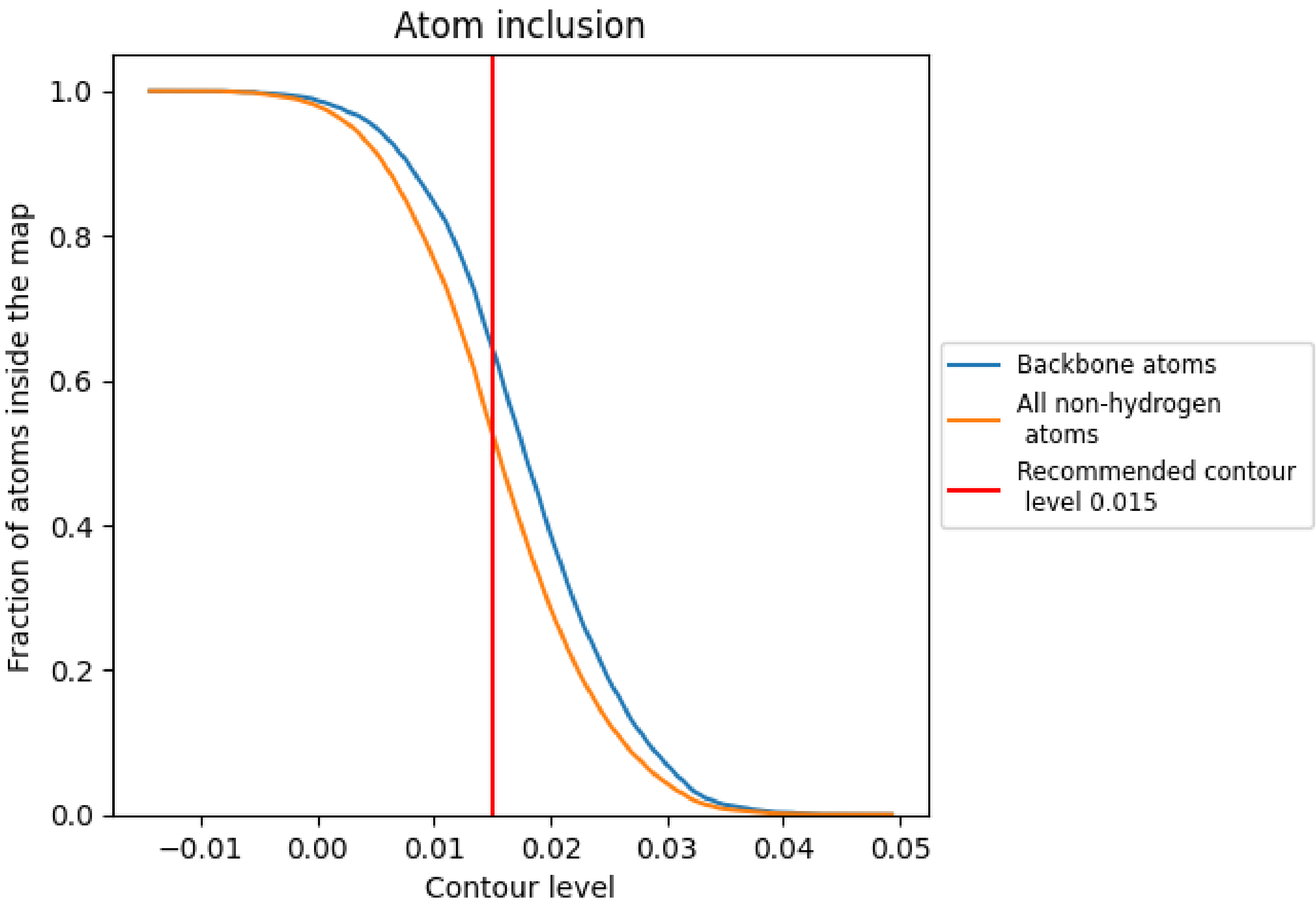

At the recommended contour level, 65% of all backbone atoms, 53% of all non-hydrogen atoms, are inside the map.
