# Supplementary material for: Flexible open conformation of the AP-3 complex explains its role in cargo recruitment at the Golgi
Source: J Biol Chem. 2021 Oct 22;297(5):101334. doi: 10.1016/j.jbc.2021.101334 (PMC8591511; doi:10.1016/j.jbc.2021.101334)
Supplement: Supporting information [file mmc7.pdf]

Full wwPDB EM Validation Report (i)

Jul 9, 2021 – 02:51 pm BST

PDB ID	:	7P3Y
EMDB ID	:	EMD-13188
Title	:	Homology model of the full-length AP-3 complex in an intermediate open conformation
Deposited on	:	2021-07-09
Resolution	:	10.10 Å(reported)

This is a Full wwPDB EM Validation Report.

This report is produced by the wwPDB biocuration pipeline after annotation of the structure.

We welcome your comments at *validation@mail.wwpdb.org*
A user guide is available at
https://www.wwpdb.org/validation/2017/EMValidationReportHelp
with specific help available everywhere you see the (i) symbol.

The following versions of software and data (see references (i)) were used in the production of this report:

EMDB validation analysis	:	0.0.0.dev84
MolProbity	:	4.02b-467
Percentile statistics	:	20191225.v01 (using entries in the PDB archive December 25th 2019)
Ideal geometry (proteins)	:	Engh & Huber (2001)
Ideal geometry (DNA, RNA)	:	Parkinson et al. (1996)
Validation Pipeline (wwPDB-VP)	:	2.22

1 Overall quality at a glance (i)

The following experimental techniques were used to determine the structure:
ELECTRON MICROSCOPY

The reported resolution of this entry is 10.10 Å.

Percentile scores (ranging between 0-100) for global validation metrics of the entry are shown in the following graphic. The table shows the number of entries on which the scores are based.

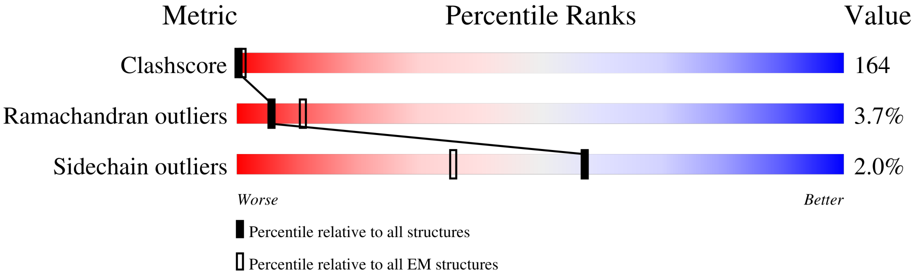

Metric	Whole archive (#Entries)	EM structures (#Entries)
Clashscore	158937	4297
Ramachandran outliers	154571	4023
Sidechain outliers	154315	3826

The table below summarises the geometric issues observed across the polymeric chains and their fit to the map. The red, orange, yellow and green segments of the bar indicate the fraction of residues that contain outliers for >=3, 2, 1 and 0 types of geometric quality criteria respectively. A grey segment represents the fraction of residues that are not modelled. The numeric value for each fraction is indicated below the corresponding segment, with a dot representing fractions <=5% The upper red bar (where present) indicates the fraction of residues that have poor fit to the EM map (all-atom inclusion < 40%). The numeric value is given above the bar.

Mol	Chain	Length	Quality of chain
1	A	964	21% / 6% 20% 28% 5% 40%
2	B	809	33% / • 26% 42% 5% 23%
3	M	483	34% / 9% 40% 28% 5% 18%
4	S	194	51% / 9% 31% 38% 9% 13%

2 Entry composition (i)

There are 4 unique types of molecules in this entry. The entry contains 14102 atoms, of which 0 are hydrogens and 0 are deuteriums.

In the tables below, the AltConf column contains the number of residues with at least one atom in alternate conformation and the Trace column contains the number of residues modelled with at most 2 atoms.

- Molecule 1 is a protein called AP-3 complex subunit delta.

Mol	Chain	Residues	Atoms					AltConf	Trace
			Total	C	N	O	S		
1	A	576	4625	2978	738	881	28	0	0

There are 32 discrepancies between the modelled and reference sequences:

Chain	Residue	Modelled	Actual	Comment	Reference
A	933	ARG	-	expression tag	UNP A0A7I9C4X2
A	934	THR	-	expression tag	UNP A0A7I9C4X2
A	935	LEU	-	expression tag	UNP A0A7I9C4X2
A	936	GLN	-	expression tag	UNP A0A7I9C4X2
A	937	VAL	-	expression tag	UNP A0A7I9C4X2
A	938	ASP	-	expression tag	UNP A0A7I9C4X2
A	939	GLY	-	expression tag	UNP A0A7I9C4X2
A	940	SER	-	expression tag	UNP A0A7I9C4X2
A	941	ASP	-	expression tag	UNP A0A7I9C4X2
A	942	TYR	-	expression tag	UNP A0A7I9C4X2
A	943	LYS	-	expression tag	UNP A0A7I9C4X2
A	944	ASP	-	expression tag	UNP A0A7I9C4X2
A	945	ASP	-	expression tag	UNP A0A7I9C4X2
A	946	ASP	-	expression tag	UNP A0A7I9C4X2
A	947	ASP	-	expression tag	UNP A0A7I9C4X2
A	948	LYS	-	expression tag	UNP A0A7I9C4X2
A	949	ASP	-	expression tag	UNP A0A7I9C4X2
A	950	TYR	-	expression tag	UNP A0A7I9C4X2
A	951	LYS	-	expression tag	UNP A0A7I9C4X2
A	952	ASP	-	expression tag	UNP A0A7I9C4X2
A	953	ASP	-	expression tag	UNP A0A7I9C4X2
A	954	ASP	-	expression tag	UNP A0A7I9C4X2
A	955	ASP	-	expression tag	UNP A0A7I9C4X2
A	956	LYS	-	expression tag	UNP A0A7I9C4X2
A	957	ASP	-	expression tag	UNP A0A7I9C4X2
A	958	TYR	-	expression tag	UNP A0A7I9C4X2
A	959	LYS	-	expression tag	UNP A0A7I9C4X2
A	960	ASP	-	expression tag	UNP A0A7I9C4X2

Continued on next page...

Continued from previous page...

Chain	Residue	Modelled	Actual	Comment	Reference
A	961	ASP	-	expression tag	UNP A0A7I9C4X2
A	962	ASP	-	expression tag	UNP A0A7I9C4X2
A	963	ASP	-	expression tag	UNP A0A7I9C4X2
A	964	LYS	-	expression tag	UNP A0A7I9C4X2

- Molecule 2 is a protein called Y55_G0035830.mRNA.1.CDS.1.

Mol	Chain	Residues	Atoms					AltConf	Trace
2	B	621	Total<br>4961	C<br>3166	N<br>830	O<br>937	S<br>28	0	0

- Molecule 3 is a protein called AP-3 complex subunit mu.

Mol	Chain	Residues	Atoms					AltConf	Trace
3	M	397	Total<br>3158	C<br>2018	N<br>516	O<br>612	S<br>12	0	0

- Molecule 4 is a protein called AP complex subunit sigma.

Mol	Chain	Residues	Atoms					AltConf	Trace
4	S	168	Total<br>1358	C<br>867	N<br>215	O<br>272	S<br>4	0	0

3 Residue-property plots

These plots are drawn for all protein, RNA, DNA and oligosaccharide chains in the entry. The first graphic for a chain summarises the proportions of the various outlier classes displayed in the second graphic. The second graphic shows the sequence view annotated by issues in geometry and atom inclusion in map density. Residues are color-coded according to the number of geometric quality criteria for which they contain at least one outlier: green = 0, yellow = 1, orange = 2 and red = 3 or more. A red diamond above a residue indicates a poor fit to the EM map for this residue (all-atom inclusion < 40%). Stretches of 2 or more consecutive residues without any outlier are shown as a green connector. Residues present in the sample, but not in the model, are shown in grey.

- Molecule 1: AP-3 complex subunit delta

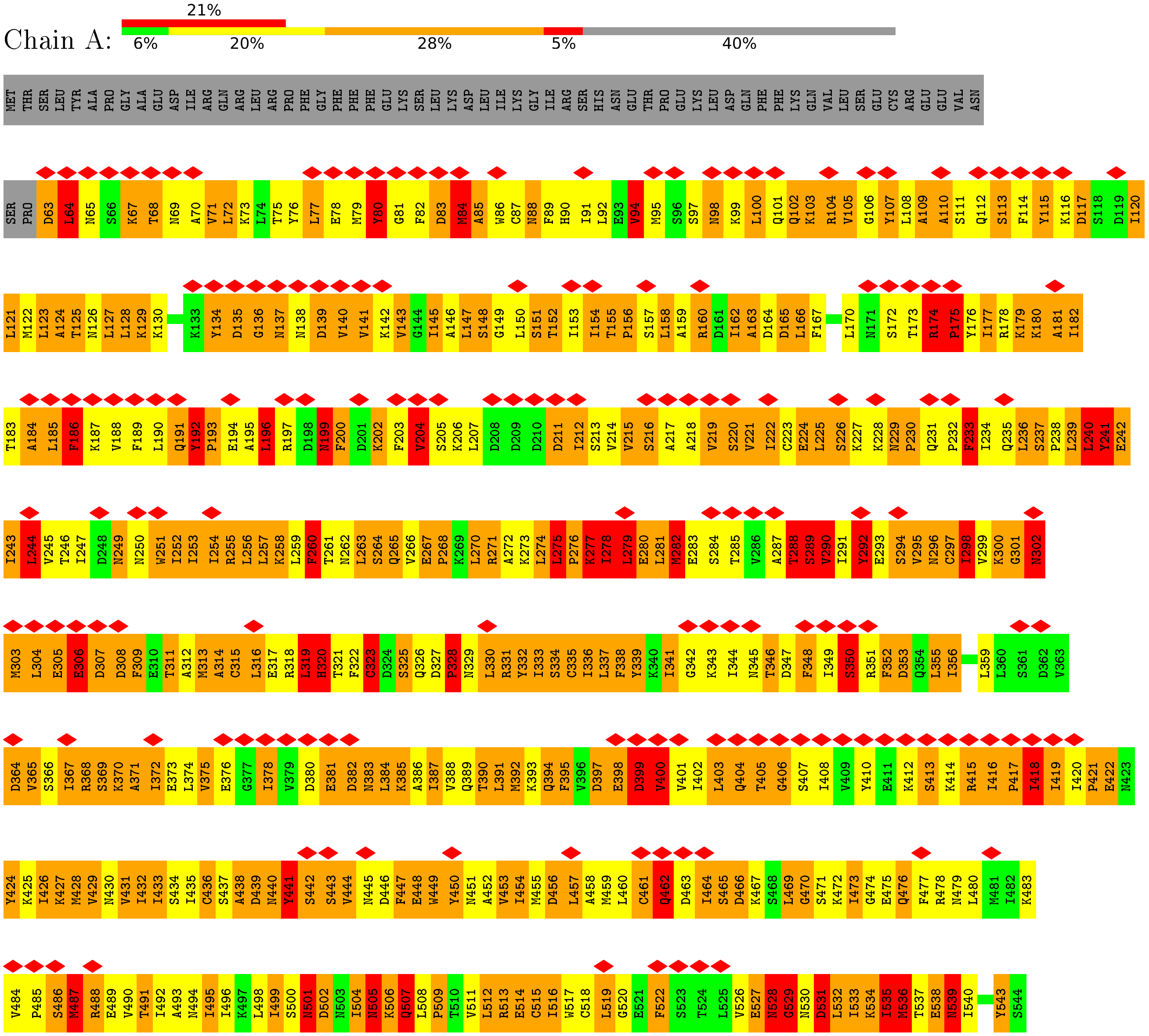

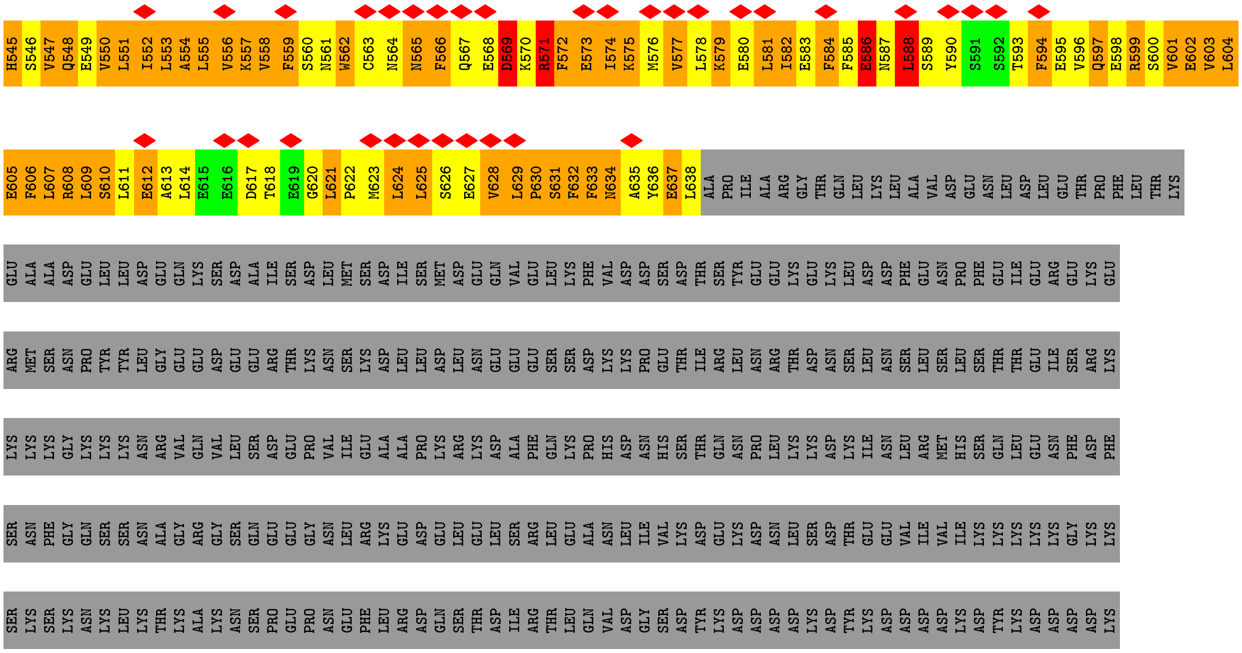

- Molecule 2: Y55_G0035830.mRNA.1.CDS.1

Chain B:

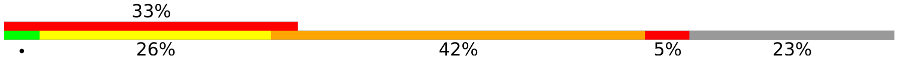

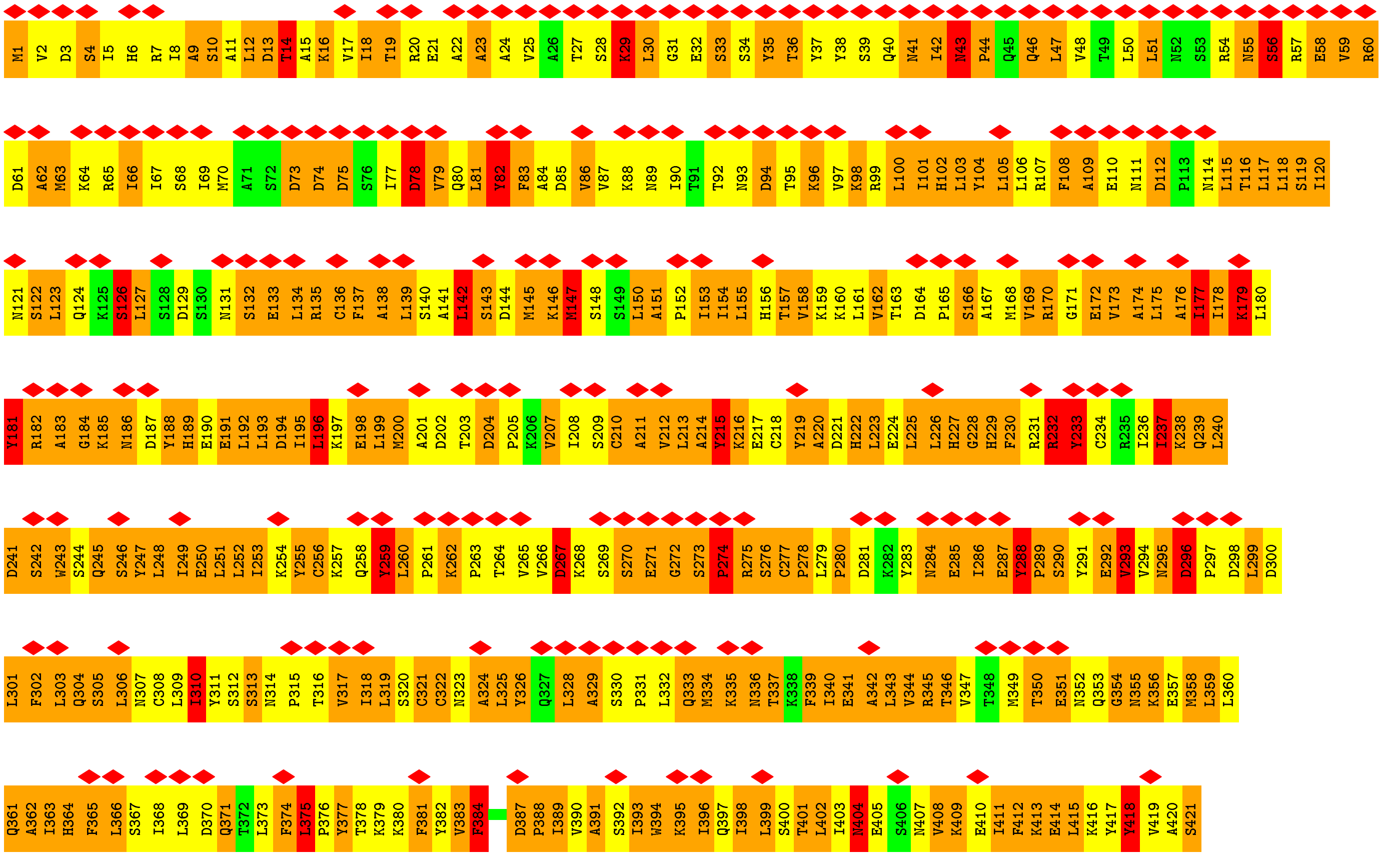

A422 H423 F424 P425 E426 N427 V428 V429 I430 M431 A432 V433 K434 S435 L436 S437 R438 C439 G440 Q441 L442 S443 T444 S445 W446 E447 S448 H449 V450 M451 K452 W453 L454 I455 D456 H457 M458 E459 S460 H461 N462 L463 S464 A465 S466 V467 L468 D469 A470 Y471 V472 N473 V474 I475 R476 M477 L478 V479 Q480 K481

N482 P483 T484 K485 H486 L487 R488 I489 I490 F491 K492 L493 A494 D495 L496 L497 T498 V499 Q500 T501 S502 L503 A504 D505 N506 A507 R508 A509 G510 I511 V512 W513 L514 F515 G516 E517 I518 A519 S520 I521 E522 F523 K524 I525 C526 P527 D528 V529 L530 R531 R532 L533 I534 Q535 N536 F537 S538 N539 E540 G541

P542 E543 T544 R545 C546 Q547 I548 L549 V550 L551 S552 A553 K554 L555 L556 S557 Y558 D559 I560 D561 N562 F563 K564 Q565 A566 Q567 V568 T569 G570 S571 E572 E573 N574 N575 Q576 N577 P578 P579 Y580 Y581 D582 F583 S584 G585 S586 R587 I588 S589 Q590 M591 Y592 N593 A594 V595 L596 Y597 L598 A599 K600 Y601

D602 D603 E604 F605 D606 I607 R608 D609 R610 A611 R612 M613 I614 S615 S616 L617 F618 D619 S620 G621 LYS TYR GLU ILE VAL SER LEU LEU LEU GLN ALA PRO LYS PRO THR ALA ARG SER ASP PHE ILE VAL SER ALA ARG LEU GLU THR HIS THR PRO GLU ILE LYS GLU PHE PHE ARG MET

LEU PRO TRP ASN THR GLU ILE THR GLU VAL GLY GLU THR GLY ASN ASP ILE ARG GLU GLY ALA GLU LEU LYS ASP TYR ASN LYS TYR LYS LYS SER PHE SER SER GLN SER PHE ILE THR ASN ASN SER ALA ARG SER PHE THR SER SER SER ASN ALA LYS LEU THR GLY ILE ASN ASP

GLY ASP SER ASN SER ILE SER GLY LYS GLY ASN VAL ASN THR PHE THR SER GLN ASN GLY LYS LYS TYR ARG LEU GLN SER LEU ASP GLU PHE PHE SER ASP ILE PRO GLU ARG LYS SER LYS PRO ARG LYS ILE ILE LYS VAL VAL GLU GLU SER SER ASP GLU ASP GLU ASP GLU SER

GLU GLU SER SER ASP ASP ASP GLU TYR SER ASP SER SER LEU GLY THR SER SER SER GLY THR SER SER SER HIS LEU GLU LEU

- Molecule 3: AP-3 complex subunit mu

Chain M: 34% | 9% 40% 28% 5% 18%

M1 Y2 L3 S4 F5 Y6 I7 T8 D9 T10 K11 N12 K13 L14 I15 F16 Q17 Y18 L19 L20 G21 A22 T23 A24 P25 SER PHE LYS HIS LEU TRP THR ARG VAL GLN SER THR CYS P39 Q40 L41 L42 E43 D44 S45 S46 S47 D48 D49 Y50 L51 D52 H53 S54 M55 V56 G57 R58 D59 L60

E61 V62 Y63 K64 Y65 F66 S67 V68 I69 N70 K71 L72 N73 Y74 W75 C76 L77 A78 S79 T80 S81 K82 S83 K84 G85 P86 L87 D88 C89 F90 T91 F92 L93 E94 T95 I96 D97 R98 I99 L100 L101 E102 Y103 F104 D105 K106 D107 K108 L109 S110 I111 K112 K113 I114 V115 N116 N117 Y118 D119 R120

I121 S122 L123 I124 F125 N126 C127 C128 V129 E130 A131 G132 E133 P134 N135 V136 S137 D138 MET LEU TYR VAL ASN LYS ILE LYS GLU ALA VAL PRO GLU ARG SER ASP LEU SER LYS PHE ILE SER SER THR ALA HIS ASN LEU GLN GLN ALA VAL GLN LEU PRO GLN GLN ARG GLN GLN GLN LEU

GLN GLN ASN GLN ILE SER ARG GLY SER ASN SER LEU ILE GLU ASN GLU GLU ILE VAL PRO TRP ARG THR SER ARG ALA SER LYS HIS GLU ASN N212 E213 L214 Y215 V216 D217 L218 L219 E220 T221 F222 H223 V224 V225 F226 E227 K228 K229 K230 S231 H232 L233 R234 L235 L236 T237 G238 S239 I240

H241 G242 I243 V244 D245 V246 R247 S248 Y249 L250 N251 D252 N253 P254 L255 V256 A257 V258 K259 L260 N261 T262 M263 G264 N265 D266 I267 G268 I269 P270 S271 L272 H273 D274 C275 V276 E277 I278 N279 D280 G281 V282 F283 S284 P285 S286 N287 I288 T289 F290 I291 P292 P293 D294 G295 K296 F297 R298 L299 L300

E301 Y302 S303 V304 D305 L306 S307 S308 Q309 V310 K311 Q312 S313 G314 V315 R316 M317 N318 S319 I320 G321 L322 M323 S324 L325 H326 F327 S330 L331 G332 K333 D334 S335 D336 E337 F338 E339 L340 S341 L342 M343 I344 E345 N346 F347 K348 K349 V350 S351 Q352 V353 D354 D355 L356 K357 I358 D359 L360 Q361

F362 N363 V364 E365 N366 A367 D368 P369 N370 E371 I372 A373 Y374 K375 I376 K377 I378 L379 R380 N381 T382 H383 G384 R385 F386 E387 N388 S389 I390 I391 M392 G393 Q394 G395 Q396 W397 I398 F399 D400 K401 S402 T403 A404 T405 G406 T407 V408 P409 V410 L411 R412 G413 C414 I415 E416 Y417 E418 N419 T420 G421

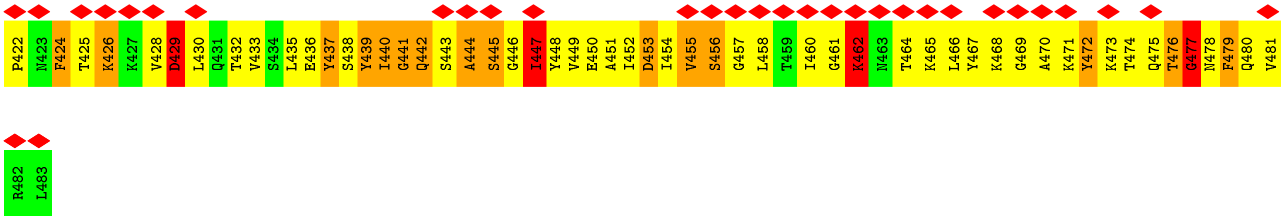

• Molecule 4: AP complex subunit sigma

Chain S:

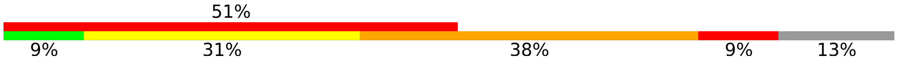

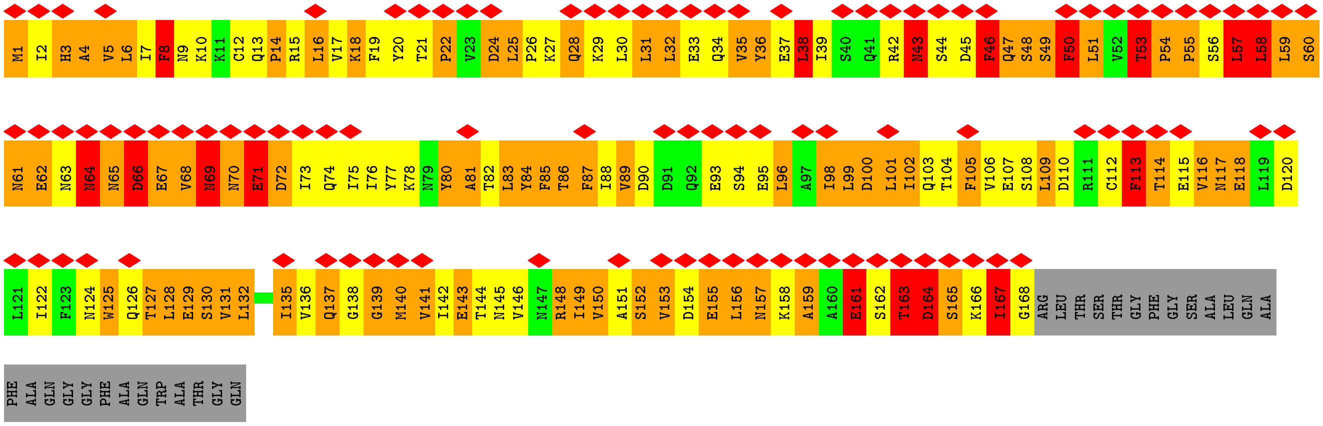

4 Experimental information (i)

Property	Value	Source
EM reconstruction method	SINGLE PARTICLE	Depositor
Imposed symmetry	POINT, Not provided	
Number of particles used	19300	Depositor
Resolution determination method	FSC 0.5 CUT-OFF	Depositor
CTF correction method	PHASE FLIPPING ONLY	Depositor
Microscope	FEI TITAN KRIOS	Depositor
Voltage (kV)	300	Depositor
Electron dose ($e^-/\AA^2$)	81	Depositor
Minimum defocus (nm)	1500	Depositor
Maximum defocus (nm)	3600	Depositor
Magnification	130000	Depositor
Image detector	GATAN K2 SUMMIT (4k x 4k)	Depositor
Maximum map value	0.051	Depositor
Minimum map value	-0.021	Depositor
Average map value	0.000	Depositor
Map value standard deviation	0.002	Depositor
Recommended contour level	0.018	Depositor
Map size (Å)	282.48, 282.48, 282.48	wwPDB
Map dimensions	264, 264, 264	wwPDB
Map angles (°)	90.0, 90.0, 90.0	wwPDB
Pixel spacing (Å)	1.07, 1.07, 1.07	Depositor

5 Model quality (i)

5.1 Standard geometry (i)

The Z score for a bond length (or angle) is the number of standard deviations the observed value is removed from the expected value. A bond length (or angle) with $|Z| > 5$ is considered an outlier worth inspection. RMSZ is the root-mean-square of all Z scores of the bond lengths (or angles).

Mol	Chain	Bond lengths		Bond angles	
		RMSZ	#|Z| >5	RMSZ	#|Z| >5
1	A	1.72	72/4699 (1.5%)	2.65	626/6358 (9.8%)
2	B	1.61	69/5055 (1.4%)	2.44	586/6852 (8.6%)
3	M	2.33	152/3217 (4.7%)	2.53	276/4346 (6.4%)
4	S	2.39	65/1379 (4.7%)	2.75	165/1874 (8.8%)
All	All	1.91	358/14350 (2.5%)	2.56	1653/19430 (8.5%)

Chiral center outliers are detected by calculating the chiral volume of a chiral center and verifying if the center is modelled as a planar moiety or with the opposite hand.A planarity outlier is detected by checking planarity of atoms in a peptide group, atoms in a mainchain group or atoms of a sidechain that are expected to be planar.

Mol	Chain	#Chirality outliers	#Planarity outliers
1	A	0	52
2	B	0	32
3	M	1	23
4	S	1	18
All	All	2	125

All (358) bond length outliers are listed below:

Mol	Chain	Res	Type	Atoms	Z	Observed(Å)	Ideal(Å)
3	M	104	PHE	N-CA	-22.87	1.00	1.46
3	M	64	LYS	N-CA	-18.24	1.09	1.46
3	M	135	ASN	C-N	-17.37	0.94	1.34
3	M	132	GLY	CA-C	-17.21	1.24	1.51
3	M	281	GLY	CA-C	16.84	1.78	1.51
1	A	438	ALA	CA-CB	-15.66	1.19	1.52
3	M	136	VAL	N-CA	-15.50	1.15	1.46
3	M	107	ASP	N-CA	-15.49	1.15	1.46
4	S	49	SER	N-CA	-15.06	1.16	1.46
4	S	53	THR	N-CA	-14.55	1.17	1.46
4	S	68	VAL	C-N	14.44	1.67	1.34
3	M	132	GLY	N-CA	-14.43	1.24	1.46

Continued on next page...

Continued from previous page...

Mol	Chain	Res	Type	Atoms	Z	Observed(Å)	Ideal(Å)
3	M	133	GLU	N-CA	-14.21	1.18	1.46
3	M	63	TYR	CA-C	-14.03	1.16	1.52
3	M	65	TYR	CA-C	-13.79	1.17	1.52
1	A	406	GLY	CA-C	13.22	1.73	1.51
4	S	48	SER	C-N	-13.05	1.04	1.34
3	M	131	ALA	CA-CB	12.62	1.78	1.52
3	M	293	PRO	N-CD	12.50	1.65	1.47
3	M	66	PHE	N-CA	-12.32	1.21	1.46
3	M	131	ALA	CA-C	-12.31	1.21	1.52
4	S	48	SER	CA-C	-11.86	1.22	1.52
3	M	3	LEU	C-N	-11.45	1.07	1.34
3	M	61	GLU	CA-C	-11.17	1.24	1.52
4	S	163	THR	CA-C	11.12	1.81	1.52
3	M	282	VAL	N-CA	11.10	1.68	1.46
4	S	46	PHE	CA-C	-11.03	1.24	1.52
3	M	63	TYR	N-CA	-10.98	1.24	1.46
3	M	81	SER	C-N	-10.94	1.08	1.34
3	M	137	SER	N-CA	-10.92	1.24	1.46
3	M	20	LEU	C-N	-10.88	1.13	1.33
4	S	55	PRO	N-CA	10.60	1.65	1.47
3	M	56	VAL	CA-C	-10.59	1.25	1.52
4	S	64	ASN	CA-C	10.50	1.80	1.52
1	A	218	ALA	CA-C	-10.29	1.26	1.52
4	S	58	LEU	CA-C	-10.28	1.26	1.52
3	M	62	VAL	N-CA	-10.18	1.25	1.46
3	M	105	ASP	C-N	-10.16	1.10	1.34
3	M	65	TYR	N-CA	-10.10	1.26	1.46
3	M	131	ALA	N-CA	-10.10	1.26	1.46
3	M	135	ASN	CA-C	-10.02	1.26	1.52
3	M	80	THR	C-N	-9.96	1.11	1.34
4	S	50	PHE	CA-C	-9.93	1.27	1.52
1	A	139	ASP	N-CA	-9.66	1.27	1.46
1	A	175	PRO	N-CD	9.66	1.61	1.47
3	M	295	GLY	CA-C	9.52	1.67	1.51
1	A	407	SER	N-CA	9.44	1.65	1.46
4	S	51	LEU	N-CA	-9.36	1.27	1.46
3	M	130	GLU	C-N	-9.34	1.12	1.34
3	M	62	VAL	CA-C	-9.28	1.28	1.52
2	B	274	PRO	N-CD	9.25	1.60	1.47
4	S	71	GLU	N-CA	-9.19	1.27	1.46
3	M	41	LEU	CA-C	-9.18	1.29	1.52
2	B	337	THR	N-CA	-9.18	1.28	1.46

Continued on next page...

Continued from previous page...

Mol	Chain	Res	Type	Atoms	Z	Observed(Å)	Ideal(Å)
3	M	64	LYS	CA-C	-9.10	1.29	1.52
2	B	284	ASN	C-N	-9.07	1.13	1.34
3	M	21	GLY	N-CA	-9.02	1.32	1.46
4	S	54	PRO	N-CA	8.98	1.62	1.47
2	B	581	TYR	N-CA	-8.97	1.28	1.46
2	B	293	VAL	C-N	-8.96	1.13	1.34
4	S	67	GLU	N-CA	-8.89	1.28	1.46
3	M	21	GLY	C-N	-8.89	1.13	1.34
3	M	52	ASP	C-N	-8.79	1.13	1.34
3	M	231	SER	N-CA	8.77	1.63	1.46
4	S	144	THR	N-CA	-8.74	1.28	1.46
3	M	351	SER	C-N	-8.64	1.14	1.34
4	S	4	ALA	CA-CB	8.64	1.70	1.52
4	S	49	SER	C-N	8.55	1.53	1.34
3	M	106	LYS	C-N	-8.52	1.14	1.34
3	M	349	LYS	N-CA	8.49	1.63	1.46
1	A	467	LYS	N-CA	-8.45	1.29	1.46
2	B	581	TYR	C-N	-8.43	1.14	1.34
2	B	289	PRO	CA-C	-8.38	1.36	1.52
3	M	406	GLY	N-CA	-8.36	1.33	1.46
3	M	281	GLY	C-N	8.34	1.53	1.34
4	S	72	ASP	N-CA	-8.34	1.29	1.46
1	A	394	GLN	CA-C	-8.31	1.31	1.52
3	M	61	GLU	C-N	-8.26	1.15	1.34
2	B	185	LYS	N-CA	-8.16	1.30	1.46
1	A	405	THR	CA-C	-8.12	1.31	1.52
3	M	225	VAL	CA-C	-8.10	1.31	1.52
3	M	335	SER	CA-C	-8.07	1.31	1.52
3	M	348	LYS	C-N	8.05	1.52	1.34
1	A	440	ASN	N-CA	-8.04	1.30	1.46
4	S	60	SER	N-CA	-7.96	1.30	1.46
2	B	260	LEU	N-CA	-7.91	1.30	1.46
4	S	83	LEU	CA-C	7.89	1.73	1.52
2	B	212	VAL	C-O	7.87	1.38	1.23
4	S	140	MET	N-CA	-7.84	1.30	1.46
3	M	279	ASN	C-N	-7.82	1.16	1.34
3	M	294	ASP	N-CA	-7.81	1.30	1.46
1	A	87	CYS	N-CA	-7.79	1.30	1.46
4	S	63	ASN	CA-C	-7.79	1.32	1.52
3	M	319	SER	C-N	-7.77	1.16	1.34
3	M	354	ASP	CA-C	7.76	1.73	1.52
3	M	134	PRO	C-N	-7.66	1.16	1.34

Continued on next page...

Continued from previous page...

Mol	Chain	Res	Type	Atoms	Z	Observed(Å)	Ideal(Å)
3	M	59	ASP	N-CA	7.63	1.61	1.46
2	B	183	ALA	CA-CB	-7.62	1.36	1.52
3	M	136	VAL	CA-C	-7.61	1.33	1.52
3	M	348	LYS	CA-C	7.60	1.72	1.52
1	A	217	ALA	C-N	7.58	1.51	1.34
4	S	55	PRO	CA-C	-7.50	1.37	1.52
3	M	62	VAL	C-N	-7.50	1.16	1.34
3	M	45	SER	CA-C	7.46	1.72	1.52
3	M	103	TYR	CA-C	7.46	1.72	1.52
3	M	352	GLN	C-N	-7.45	1.17	1.34
1	A	406	GLY	C-N	7.44	1.51	1.34
4	S	116	VAL	CA-C	-7.41	1.33	1.52
3	M	109	LEU	N-CA	-7.40	1.31	1.46
3	M	239	SER	N-CA	-7.38	1.31	1.46
2	B	334	MET	N-CA	7.37	1.61	1.46
3	M	442	GLN	N-CA	-7.37	1.31	1.46
3	M	283	PHE	N-CA	-7.36	1.31	1.46
3	M	450	GLU	C-N	-7.35	1.17	1.34
3	M	44	ASP	C-N	7.28	1.50	1.34
4	S	22	PRO	C-N	-7.27	1.17	1.34
3	M	136	VAL	C-N	-7.25	1.17	1.34
4	S	167	ILE	C-N	7.22	1.46	1.33
3	M	453	ASP	C-N	-7.17	1.17	1.34
1	A	287	ALA	CA-CB	-7.17	1.37	1.52
3	M	403	THR	C-N	-7.13	1.17	1.34
2	B	330	SER	N-CA	-7.08	1.32	1.46
4	S	6	LEU	C-N	-7.08	1.17	1.34
2	B	330	SER	C-N	-7.05	1.20	1.34
1	A	529	GLY	N-CA	-7.04	1.35	1.46
3	M	95	THR	N-CA	7.03	1.60	1.46
1	A	243	ILE	N-CA	-6.99	1.32	1.46
4	S	70	ASN	C-N	-6.98	1.18	1.34
3	M	293	PRO	CA-C	-6.96	1.39	1.52
3	M	379	LEU	C-N	-6.96	1.18	1.34
3	M	70	ASN	C-N	-6.95	1.18	1.34
3	M	441	GLY	CA-C	-6.93	1.40	1.51
1	A	218	ALA	CA-CB	-6.89	1.38	1.52
3	M	230	LYS	CA-C	6.89	1.70	1.52
2	B	582	ASP	N-CA	-6.86	1.32	1.46
3	M	440	ILE	N-CA	-6.86	1.32	1.46
1	A	301	GLY	CA-C	-6.85	1.40	1.51
3	M	441	GLY	N-CA	-6.85	1.35	1.46

Continued on next page...

Continued from previous page...

Mol	Chain	Res	Type	Atoms	Z	Observed(Å)	Ideal(Å)
1	A	230	PRO	N-CD	-6.81	1.38	1.47
2	B	285	GLU	N-CA	-6.81	1.32	1.46
1	A	289	SER	C-N	-6.81	1.18	1.34
3	M	420	THR	N-CA	-6.77	1.32	1.46
2	B	329	ALA	CA-C	-6.77	1.35	1.52
3	M	55	MET	N-CA	-6.75	1.32	1.46
3	M	55	MET	C-N	6.71	1.49	1.34
1	A	465	SER	N-CA	-6.71	1.32	1.46
3	M	81	SER	N-CA	-6.70	1.32	1.46
3	M	440	ILE	C-N	-6.69	1.21	1.33
3	M	64	LYS	C-N	-6.67	1.18	1.34
1	A	509	PRO	N-CD	6.65	1.57	1.47
3	M	280	ASP	C-N	-6.61	1.21	1.33
1	A	439	ASP	C-N	-6.59	1.18	1.34
3	M	392	MET	N-CA	-6.58	1.33	1.46
4	S	108	SER	N-CA	-6.58	1.33	1.46
4	S	143	GLU	CA-C	-6.53	1.35	1.52
3	M	322	LEU	C-O	6.51	1.35	1.23
4	S	55	PRO	N-CD	6.51	1.56	1.47
4	S	103	GLN	CA-C	-6.51	1.36	1.52
3	M	212	ASN	N-CA	-6.50	1.33	1.46
4	S	114	THR	C-N	-6.49	1.19	1.34
2	B	184	GLY	C-N	-6.44	1.19	1.34
4	S	58	LEU	C-N	6.42	1.48	1.34
3	M	320	ILE	N-CA	-6.42	1.33	1.46
2	B	259	TYR	C-N	-6.41	1.19	1.34
3	M	105	ASP	N-CA	-6.40	1.33	1.46
4	S	116	VAL	N-CA	-6.39	1.33	1.46
3	M	82	LYS	C-N	-6.36	1.19	1.34
2	B	334	MET	C-N	-6.31	1.19	1.34
4	S	138	GLY	C-N	6.31	1.44	1.33
4	S	22	PRO	N-CA	-6.29	1.36	1.47
3	M	282	VAL	CA-C	-6.29	1.36	1.52
3	M	138	ASP	N-CA	-6.28	1.33	1.46
1	A	298	ILE	C-O	6.28	1.35	1.23
4	S	62	GLU	C-N	-6.28	1.19	1.34
2	B	568	VAL	N-CA	-6.27	1.33	1.46
1	A	378	ILE	C-N	-6.27	1.19	1.34
1	A	410	TYR	N-CA	-6.26	1.33	1.46
1	A	71	VAL	CA-C	-6.25	1.36	1.52
4	S	81	ALA	CA-CB	-6.22	1.39	1.52
3	M	336	ASP	N-CA	-6.22	1.33	1.46

Continued on next page...

Continued from previous page...

Mol	Chain	Res	Type	Atoms	Z	Observed(Å)	Ideal(Å)
1	A	278	ILE	N-CA	-6.21	1.33	1.46
3	M	451	ALA	CA-CB	6.20	1.65	1.52
2	B	458	MET	C-O	6.20	1.35	1.23
1	A	566	PHE	CA-C	-6.20	1.36	1.52
4	S	124	ASN	N-CA	-6.18	1.33	1.46
1	A	394	GLN	N-CA	-6.16	1.34	1.46
3	M	60	LEU	CA-C	-6.16	1.36	1.52
2	B	329	ALA	N-CA	-6.15	1.34	1.46
2	B	585	GLY	N-CA	-6.15	1.36	1.46
1	A	464	ILE	CA-C	-6.14	1.36	1.52
3	M	6	TYR	C-N	-6.13	1.20	1.34
3	M	439	TYR	CA-C	-6.13	1.37	1.52
4	S	57	LEU	CA-C	-6.12	1.37	1.52
3	M	288	ILE	C-N	-6.12	1.20	1.34
4	S	3	HIS	N-CA	-6.11	1.34	1.46
3	M	288	ILE	N-CA	6.11	1.58	1.46
4	S	60	SER	C-N	-6.11	1.20	1.34
1	A	533	ILE	N-CA	-6.09	1.34	1.46
3	M	404	ALA	N-CA	-6.05	1.34	1.46
3	M	56	VAL	C-N	-6.04	1.22	1.33
2	B	522	GLU	CA-C	-6.02	1.37	1.52
3	M	65	TYR	C-N	-6.02	1.20	1.34
3	M	97	ASP	CA-C	-6.01	1.37	1.52
4	S	48	SER	N-CA	-6.01	1.34	1.46
1	A	221	VAL	N-CA	6.00	1.58	1.46
3	M	23	THR	C-N	-6.00	1.20	1.34
4	S	47	GLN	N-CA	-5.98	1.34	1.46
1	A	466	ASP	C-N	-5.96	1.20	1.34
1	A	534	LYS	CA-C	-5.95	1.37	1.52
2	B	293	VAL	CA-C	-5.95	1.37	1.52
3	M	288	ILE	CA-C	-5.95	1.37	1.52
1	A	444	VAL	N-CA	-5.94	1.34	1.46
1	A	281	LEU	N-CA	5.94	1.58	1.46
3	M	257	ALA	N-CA	-5.94	1.34	1.46
3	M	91	THR	CA-C	-5.93	1.37	1.52
3	M	18	TYR	N-CA	-5.93	1.34	1.46
3	M	252	ASP	C-N	-5.92	1.20	1.34
3	M	22	ALA	N-CA	-5.91	1.34	1.46
1	A	84	MET	N-CA	-5.90	1.34	1.46
3	M	25	PRO	CA-C	-5.89	1.41	1.52
1	A	277	LYS	C-N	-5.87	1.20	1.34
3	M	262	THR	CA-C	-5.87	1.37	1.52

Continued on next page...

Continued from previous page...

Mol	Chain	Res	Type	Atoms	Z	Observed(Å)	Ideal(Å)
1	A	418	ILE	C-N	5.87	1.47	1.34
4	S	139	GLY	CA-C	-5.86	1.42	1.51
1	A	302	ASN	CA-C	-5.86	1.37	1.52
2	B	259	TYR	CA-C	-5.83	1.37	1.52
3	M	263	MET	N-CA	-5.83	1.34	1.46
3	M	446	GLY	CA-C	5.83	1.61	1.51
4	S	14	PRO	C-N	-5.80	1.20	1.34
3	M	83	SER	N-CA	-5.78	1.34	1.46
3	M	377	LYS	C-N	-5.76	1.20	1.34
1	A	216	SER	C-N	5.76	1.47	1.34
2	B	567	GLN	CA-C	-5.76	1.38	1.52
2	B	579	PRO	N-CD	-5.76	1.39	1.47
1	A	303	MET	N-CA	-5.75	1.34	1.46
3	M	456	SER	C-N	5.74	1.43	1.33
1	A	508	LEU	N-CA	-5.74	1.34	1.46
2	B	187	ASP	CA-C	-5.73	1.38	1.52
3	M	278	ILE	CA-C	-5.73	1.38	1.52
4	S	64	ASN	C-N	5.72	1.47	1.34
2	B	576	GLN	C-N	-5.70	1.21	1.34
1	A	387	ILE	CA-C	-5.70	1.38	1.52
2	B	222	HIS	N-CA	5.69	1.57	1.46
1	A	263	LEU	N-CA	-5.68	1.34	1.46
1	A	406	GLY	N-CA	-5.67	1.37	1.46
4	S	59	LEU	N-CA	-5.66	1.35	1.46
4	S	115	GLU	C-N	-5.66	1.21	1.34
1	A	531	ASP	CA-C	-5.65	1.38	1.52
2	B	290	SER	N-CA	-5.65	1.35	1.46
2	B	584	SER	C-N	-5.64	1.22	1.33
2	B	526	CYS	N-CA	-5.63	1.35	1.46
2	B	82	TYR	N-CA	-5.62	1.35	1.46
2	B	184	GLY	N-CA	5.61	1.54	1.46
2	B	501	THR	N-CA	-5.60	1.35	1.46
3	M	113	LYS	CA-C	-5.60	1.38	1.52
3	M	367	ALA	CA-CB	-5.58	1.40	1.52
1	A	588	LEU	C-N	-5.58	1.21	1.34
4	S	80	TYR	N-CA	-5.57	1.35	1.46
1	A	348	PHE	CA-C	-5.56	1.38	1.52
3	M	296	LYS	CA-C	-5.55	1.38	1.52
3	M	323	MET	N-CA	-5.55	1.35	1.46
2	B	150	LEU	N-CA	-5.54	1.35	1.46
1	A	405	THR	C-N	-5.54	1.23	1.33
2	B	219	TYR	C-N	-5.54	1.21	1.34

Continued on next page...

Continued from previous page...

Mol	Chain	Res	Type	Atoms	Z	Observed(Å)	Ideal(Å)
4	S	158	LYS	CA-C	-5.52	1.38	1.52
3	M	287	ASN	CA-C	5.51	1.67	1.52
4	S	83	LEU	C-N	-5.51	1.21	1.34
4	S	144	THR	C-N	-5.51	1.21	1.34
3	M	61	GLU	N-CA	-5.50	1.35	1.46
4	S	84	TYR	C-N	-5.50	1.21	1.34
4	S	5	VAL	CA-C	5.50	1.67	1.52
2	B	485	LYS	CA-C	-5.49	1.38	1.52
3	M	68	VAL	N-CA	-5.48	1.35	1.46
3	M	457	GLY	CA-C	-5.47	1.43	1.51
3	M	96	ILE	C-N	5.46	1.46	1.34
3	M	402	SER	C-N	5.46	1.46	1.34
4	S	83	LEU	N-CA	-5.45	1.35	1.46
3	M	66	PHE	C-N	-5.43	1.21	1.34
4	S	99	LEU	CA-C	-5.43	1.38	1.52
2	B	382	TYR	N-CA	-5.43	1.35	1.46
3	M	24	ALA	N-CA	-5.43	1.35	1.46
3	M	280	ASP	CA-C	-5.42	1.38	1.52
1	A	308	ASP	N-CA	-5.42	1.35	1.46
2	B	574	ASN	N-CA	-5.42	1.35	1.46
3	M	25	PRO	N-CA	-5.42	1.38	1.47
4	S	143	GLU	C-N	-5.42	1.21	1.34
3	M	262	THR	N-CA	-5.41	1.35	1.46
3	M	284	SER	C-N	-5.41	1.24	1.34
3	M	354	ASP	C-N	-5.41	1.21	1.34
4	S	115	GLU	CA-C	-5.38	1.39	1.52
1	A	83	ASP	CA-C	-5.37	1.39	1.52
3	M	50	TYR	C-N	-5.37	1.21	1.34
2	B	336	ASN	C-N	-5.36	1.21	1.34
2	B	500	GLN	C-N	-5.36	1.21	1.34
2	B	582	ASP	C-N	-5.36	1.21	1.34
1	A	623	MET	CA-C	-5.35	1.39	1.52
3	M	75	TRP	C-N	-5.35	1.21	1.34
1	A	138	ASN	C-N	-5.33	1.21	1.34
2	B	383	VAL	CA-C	-5.33	1.39	1.52
3	M	128	CYS	C-O	5.33	1.33	1.23
1	A	445	ASN	CA-C	-5.31	1.39	1.52
2	B	290	SER	CA-C	-5.31	1.39	1.52
2	B	425	PRO	CA-C	-5.31	1.42	1.52
3	M	130	GLU	CA-C	5.31	1.66	1.52
3	M	223	HIS	C-N	-5.31	1.21	1.34
4	S	8	PHE	C-N	-5.31	1.21	1.34

Continued on next page...

Continued from previous page...

Mol	Chain	Res	Type	Atoms	Z	Observed(Å)	Ideal(Å)
2	B	83	PHE	C-N	5.30	1.46	1.34
2	B	331	PRO	N-CA	-5.29	1.38	1.47
1	A	192	TYR	CA-C	-5.28	1.39	1.52
2	B	443	SER	CA-C	-5.27	1.39	1.52
1	A	241	TYR	N-CA	-5.26	1.35	1.46
3	M	63	TYR	C-N	-5.25	1.22	1.34
2	B	276	SER	N-CA	-5.25	1.35	1.46
1	A	120	ILE	CA-C	-5.24	1.39	1.52
2	B	35	TYR	CA-C	-5.24	1.39	1.52
2	B	219	TYR	CA-C	-5.23	1.39	1.52
1	A	535	ILE	N-CA	-5.22	1.35	1.46
2	B	601	TYR	N-CA	-5.22	1.35	1.46
4	S	159	ALA	CA-CB	5.22	1.63	1.52
1	A	388	VAL	CA-C	-5.22	1.39	1.52
3	M	365	GLU	N-CA	-5.22	1.35	1.46
2	B	109	ALA	CA-CB	-5.21	1.41	1.52
2	B	384	PHE	N-CA	-5.18	1.35	1.46
1	A	288	THR	N-CA	5.17	1.56	1.46
3	M	41	LEU	C-N	5.15	1.45	1.34
3	M	476	THR	C-N	-5.15	1.23	1.33
3	M	129	VAL	C-O	-5.15	1.13	1.23
3	M	441	GLY	C-N	-5.14	1.22	1.34
1	A	85	ALA	CA-CB	-5.14	1.41	1.52
3	M	344	ILE	CA-C	-5.14	1.39	1.52
2	B	289	PRO	C-N	-5.13	1.22	1.34
3	M	228	LYS	C-N	-5.13	1.22	1.34
2	B	275	ARG	C-N	-5.12	1.22	1.34
3	M	256	VAL	C-O	5.12	1.33	1.23
3	M	444	ALA	CA-CB	5.12	1.63	1.52
3	M	253	ASN	N-CA	-5.12	1.36	1.46
1	A	507	GLN	C-N	-5.12	1.22	1.34
1	A	392	MET	N-CA	-5.11	1.36	1.46
3	M	126	ASN	CA-C	-5.11	1.39	1.52
2	B	75	ASP	C-N	-5.11	1.22	1.34
2	B	273	SER	N-CA	-5.11	1.36	1.46
2	B	190	GLU	CD-OE1	-5.10	1.20	1.25
2	B	500	GLN	CA-C	-5.10	1.39	1.52
3	M	137	SER	C-N	-5.10	1.22	1.34
2	B	424	PHE	N-CA	-5.08	1.36	1.46
2	B	172	GLU	CD-OE2	-5.08	1.20	1.25
1	A	242	GLU	C-O	-5.07	1.13	1.23
1	A	399	ASP	N-CA	-5.07	1.36	1.46

Continued on next page...

Continued from previous page...

Mol	Chain	Res	Type	Atoms	Z	Observed(Å)	Ideal(Å)
1	A	522	PHE	CA-C	-5.06	1.39	1.52
2	B	272	GLY	CA-C	-5.06	1.43	1.51
1	A	304	LEU	N-CA	-5.05	1.36	1.46
2	B	515	PHE	CA-C	5.03	1.66	1.52
1	A	302	ASN	N-CA	-5.03	1.36	1.46
2	B	407	ASN	N-CA	-5.03	1.36	1.46
4	S	29	LYS	CA-C	-5.03	1.39	1.52
1	A	464	ILE	C-N	-5.02	1.22	1.34
1	A	404	GLN	N-CA	-5.01	1.36	1.46
4	S	67	GLU	CA-C	-5.00	1.40	1.52

All (1653) bond angle outliers are listed below:

Mol	Chain	Res	Type	Atoms	Z	Observed(°)	Ideal(°)
3	M	404	ALA	CB-CA-C	25.34	148.10	110.10
4	S	69	ASN	N-CA-C	23.23	173.71	111.00
1	A	242	GLU	C-N-CA	22.78	178.65	121.70
3	M	130	GLU	N-CA-C	-19.46	58.45	111.00
1	A	265	GLN	N-CA-C	-19.25	59.03	111.00
3	M	404	ALA	N-CA-CB	-18.93	83.59	110.10
4	S	69	ASN	CA-C-N	-18.66	76.15	117.20
4	S	69	ASN	O-C-N	18.58	152.43	122.70
1	A	80	TYR	N-CA-C	-18.36	61.42	111.00
4	S	69	ASN	CA-C-O	-17.97	82.37	120.10
4	S	58	LEU	N-CA-C	-17.91	62.65	111.00
3	M	45	SER	C-N-CA	-17.45	78.07	121.70
1	A	277	LYS	C-N-CA	17.27	164.86	121.70
3	M	82	LYS	C-N-CA	17.05	164.32	121.70
4	S	69	ASN	CB-CA-C	-16.89	76.61	110.40
3	M	103	TYR	N-CA-C	-16.85	65.49	111.00
3	M	354	ASP	N-CA-C	-16.56	66.28	111.00
3	M	354	ASP	C-N-CA	16.42	162.76	121.70
1	A	444	VAL	N-CA-C	-16.35	66.84	111.00
1	A	439	ASP	N-CA-C	-16.24	67.15	111.00
3	M	403	THR	C-N-CA	16.09	161.94	121.70
3	M	373	ALA	CB-CA-C	15.36	133.15	110.10
1	A	98	ASN	C-N-CA	15.29	159.93	121.70
3	M	280	ASP	N-CA-C	15.07	151.70	111.00
2	B	584	SER	N-CA-C	15.06	151.67	111.00
2	B	580	TYR	C-N-CA	-15.06	84.05	121.70
2	B	275	ARG	N-CA-C	-14.98	70.54	111.00
1	A	304	LEU	C-N-CA	-14.94	84.36	121.70

Continued on next page...

Continued from previous page...

Mol	Chain	Res	Type	Atoms	Z	Observed(°)	Ideal(°)
3	M	21	GLY	C-N-CA	14.90	158.95	121.70
3	M	131	ALA	CA-C-N	-14.63	86.93	116.20
1	A	534	LYS	CA-C-N	14.58	149.27	117.20
2	B	290	SER	C-N-CA	-14.56	85.29	121.70
2	B	263	PRO	C-N-CA	14.55	158.09	121.70
3	M	135	ASN	N-CA-C	-14.39	72.15	111.00
3	M	103	TYR	O-C-N	-14.38	99.69	122.70
1	A	534	LYS	C-N-CA	14.13	157.03	121.70
3	M	294	ASP	N-CA-C	-13.93	73.38	111.00
3	M	103	TYR	C-N-CA	13.89	156.42	121.70
1	A	80	TYR	O-C-N	-13.47	100.31	123.20
2	B	600	LYS	C-N-CA	-13.43	88.12	121.70
1	A	532	LEU	C-N-CA	-13.41	88.17	121.70
3	M	80	THR	N-CA-C	13.40	147.18	111.00
2	B	579	PRO	C-N-CA	-13.36	88.31	121.70
2	B	78	ASP	C-N-CA	-13.24	88.60	121.70
1	A	418	ILE	C-N-CA	-13.18	88.74	121.70
1	A	98	ASN	N-CA-C	-12.81	76.40	111.00
3	M	293	PRO	CA-N-CD	-12.78	93.62	111.50
1	A	464	ILE	C-N-CA	-12.72	89.91	121.70
4	S	168	GLY	N-CA-C	12.72	144.90	113.10
3	M	59	ASP	C-N-CA	-12.70	89.95	121.70
3	M	130	GLU	C-N-CA	12.68	153.41	121.70
1	A	302	ASN	N-CA-C	-12.60	76.97	111.00
1	A	64	LEU	O-C-N	-12.58	102.57	122.70
3	M	51	LEU	N-CA-C	12.54	144.85	111.00
1	A	405	THR	C-N-CA	-12.51	96.03	122.30
1	A	260	PHE	O-C-N	-12.50	102.70	122.70
4	S	46	PHE	C-N-CA	-12.46	90.55	121.70
2	B	581	TYR	C-N-CA	12.44	152.79	121.70
3	M	133	GLU	C-N-CD	12.39	154.43	128.40
3	M	405	THR	N-CA-C	12.33	144.29	111.00
1	A	536	MET	O-C-N	-12.30	103.03	122.70
1	A	151	SER	C-N-CA	-12.25	91.08	121.70
3	M	132	GLY	CA-C-N	-12.25	90.26	117.20
3	M	54	SER	N-CA-C	12.22	144.00	111.00
3	M	61	GLU	N-CA-C	-12.19	78.10	111.00
3	M	351	SER	N-CA-C	12.14	143.77	111.00
1	A	469	LEU	C-N-CA	-12.11	96.88	122.30
3	M	22	ALA	CB-CA-C	12.07	128.21	110.10
3	M	82	LYS	N-CA-C	-12.03	78.53	111.00
1	A	289	SER	O-C-N	-12.00	103.50	122.70

Continued on next page...

Continued from previous page...

Mol	Chain	Res	Type	Atoms	Z	Observed(°)	Ideal(°)
1	A	135	ASP	C-N-CA	-11.91	97.30	122.30
3	M	50	TYR	N-CA-C	11.87	143.06	111.00
1	A	233	PHE	C-N-CA	-11.79	92.23	121.70
2	B	330	SER	N-CA-C	-11.76	79.25	111.00
1	A	323	CYS	C-N-CA	11.61	150.72	121.70
2	B	185	LYS	C-N-CA	-11.55	92.83	121.70
2	B	336	ASN	C-N-CA	11.54	150.56	121.70
1	A	265	GLN	CA-C-N	11.49	142.49	117.20
1	A	136	GLY	C-N-CA	-11.44	93.10	121.70
4	S	53	THR	C-N-CD	11.44	152.41	128.40
1	A	536	MET	C-N-CA	-11.42	93.16	121.70
2	B	286	ILE	N-CA-C	-11.36	80.32	111.00
3	M	458	LEU	O-C-N	11.34	140.85	122.70
2	B	260	LEU	N-CA-C	-11.30	80.49	111.00
2	B	404	ASN	C-N-CA	-11.29	93.48	121.70
3	M	462	LYS	N-CA-C	-11.27	80.57	111.00
2	B	329	ALA	CB-CA-C	-11.24	93.24	110.10
3	M	52	ASP	N-CA-C	11.22	141.28	111.00
1	A	465	SER	CA-C-N	-11.19	92.58	117.20
1	A	534	LYS	N-CA-C	-11.19	80.79	111.00
3	M	23	THR	N-CA-C	-11.18	80.82	111.00
1	A	242	GLU	CA-C-N	11.09	141.59	117.20
3	M	46	SER	C-N-CA	-10.93	94.37	121.70
2	B	310	ILE	C-N-CA	-10.90	94.44	121.70
4	S	43	ASN	O-C-N	-10.82	105.39	122.70
4	S	109	LEU	O-C-N	-10.80	105.41	122.70
1	A	80	TYR	CA-C-N	10.79	137.78	116.20
1	A	416	ILE	C-N-CD	10.77	151.02	128.40
4	S	163	THR	C-N-CA	10.73	148.52	121.70
1	A	287	ALA	C-N-CA	10.72	148.50	121.70
2	B	497	LEU	O-C-N	-10.68	105.61	122.70
4	S	54	PRO	CA-N-CD	-10.64	96.60	111.50
4	S	63	ASN	N-CA-C	-10.64	82.27	111.00
1	A	244	LEU	O-C-N	-10.63	105.69	122.70
1	A	204	VAL	O-C-N	-10.57	105.78	122.70
2	B	82	TYR	C-N-CA	-10.49	95.47	121.70
1	A	586	GLU	C-N-CA	-10.48	95.50	121.70
2	B	335	LYS	N-CA-C	-10.47	82.73	111.00
2	B	584	SER	C-N-CA	-10.45	100.36	122.30
2	B	287	GLU	C-N-CA	10.40	147.69	121.70
2	B	292	GLU	C-N-CA	10.37	147.62	121.70
1	A	629	LEU	C-N-CD	10.35	150.13	128.40

Continued on next page...

Continued from previous page...

Mol	Chain	Res	Type	Atoms	Z	Observed(°)	Ideal(°)
3	M	80	THR	C-N-CA	10.33	147.54	121.70
4	S	164	ASP	C-N-CA	-10.32	95.89	121.70
1	A	84	MET	C-N-CA	-10.30	95.95	121.70
1	A	519	LEU	C-N-CA	-10.29	100.69	122.30
3	M	279	ASN	N-CA-C	10.27	138.72	111.00
1	A	621	LEU	C-N-CD	10.14	149.71	128.40
2	B	571	SER	N-CA-C	-10.10	83.73	111.00
3	M	83	SER	C-N-CA	10.01	146.73	121.70
3	M	402	SER	C-N-CA	-9.96	96.79	121.70
2	B	289	PRO	C-N-CA	-9.96	96.80	121.70
1	A	84	MET	O-C-N	-9.96	106.77	122.70
1	A	569	ASP	C-N-CA	-9.91	96.92	121.70
2	B	230	PHE	C-N-CA	-9.89	96.98	121.70
2	B	162	VAL	C-N-CA	-9.88	97.00	121.70
1	A	320	HIS	O-C-N	-9.87	106.91	122.70
1	A	380	ASP	N-CA-C	-9.86	84.38	111.00
2	B	262	LYS	C-N-CD	9.82	149.03	128.40
2	B	576	GLN	N-CA-C	-9.77	84.63	111.00
4	S	57	LEU	CA-C-N	-9.76	95.72	117.20
1	A	346	THR	C-N-CA	-9.76	97.30	121.70
2	B	570	GLY	N-CA-C	-9.74	88.75	113.10
1	A	440	ASN	C-N-CA	9.70	145.94	121.70
1	A	586	GLU	O-C-N	-9.66	107.24	122.70
3	M	268	GLY	C-N-CA	-9.65	97.57	121.70
2	B	577	ASN	C-N-CD	9.63	148.62	128.40
3	M	57	GLY	C-N-CA	9.58	145.65	121.70
4	S	68	VAL	N-CA-C	9.54	136.76	111.00
1	A	319	LEU	O-C-N	-9.51	107.49	122.70
2	B	212	VAL	CA-C-O	9.50	140.05	120.10
3	M	134	PRO	N-CA-C	-9.50	87.39	112.10
1	A	305	GLU	N-CA-C	9.50	136.64	111.00
1	A	462	GLN	O-C-N	-9.49	107.51	122.70
2	B	584	SER	N-CA-CB	-9.49	96.26	110.50
1	A	504	ILE	O-C-N	-9.46	107.56	122.70
1	A	242	GLU	N-CA-C	-9.42	85.57	111.00
4	S	6	LEU	O-C-N	-9.42	107.63	122.70
1	A	212	ILE	C-N-CA	-9.41	98.18	121.70
1	A	504	ILE	C-N-CA	-9.38	98.25	121.70
1	A	569	ASP	N-CA-C	-9.38	85.68	111.00
2	B	366	LEU	O-C-N	-9.37	107.71	122.70
3	M	59	ASP	CA-C-N	9.37	137.81	117.20
3	M	252	ASP	N-CA-C	-9.37	85.72	111.00

Continued on next page...

Continued from previous page...

Mol	Chain	Res	Type	Atoms	Z	Observed(°)	Ideal(°)
1	A	282	MET	O-C-N	-9.36	107.72	122.70
3	M	279	ASN	C-N-CA	9.36	145.11	121.70
2	B	187	ASP	C-N-CA	-9.36	98.31	121.70
1	A	403	LEU	C-N-CA	-9.36	98.31	121.70
2	B	102	HIS	O-C-N	-9.35	107.75	122.70
4	S	116	VAL	CA-C-N	-9.34	96.64	117.20
3	M	379	LEU	O-C-N	-9.33	107.77	122.70
3	M	99	ILE	O-C-N	9.31	137.60	122.70
2	B	577	ASN	N-CA-C	9.30	136.11	111.00
3	M	3	LEU	O-C-N	-9.28	107.85	122.70
4	S	143	GLU	N-CA-C	-9.28	85.94	111.00
2	B	535	GLN	C-N-CA	-9.27	98.52	121.70
1	A	80	TYR	C-N-CA	9.27	141.76	122.30
2	B	505	ASP	C-N-CA	-9.27	98.53	121.70
1	A	365	VAL	C-N-CA	-9.24	98.59	121.70
2	B	560	ILE	C-N-CA	-9.23	98.62	121.70
3	M	306	LEU	O-C-N	-9.21	107.96	122.70
1	A	110	ALA	C-N-CA	-9.21	98.67	121.70
1	A	381	GLU	C-N-CA	-9.21	98.68	121.70
3	M	406	GLY	N-CA-C	-9.19	90.14	113.10
2	B	461	HIS	C-N-CA	-9.16	98.80	121.70
1	A	275	LEU	C-N-CD	9.13	147.57	128.40
2	B	205	PRO	C-N-CA	-9.10	98.94	121.70
2	B	211	ALA	O-C-N	9.09	137.25	122.70
3	M	6	TYR	O-C-N	-9.09	108.15	122.70
2	B	580	TYR	N-CA-C	-9.09	86.47	111.00
3	M	41	LEU	O-C-N	9.05	137.18	122.70
1	A	534	LYS	CA-C-O	-9.03	101.14	120.10
2	B	497	LEU	C-N-CA	-9.01	99.17	121.70
1	A	103	LYS	O-C-N	-8.99	108.31	122.70
1	A	465	SER	C-N-CA	8.99	144.18	121.70
1	A	240	LEU	C-N-CA	-8.99	99.23	121.70
1	A	163	ALA	C-N-CA	-8.98	99.24	121.70
3	M	136	VAL	CA-C-N	-8.98	97.44	117.20
1	A	86	TRP	C-N-CA	-8.96	99.31	121.70
2	B	568	VAL	CA-C-N	-8.95	97.52	117.20
3	M	351	SER	C-N-CA	8.91	143.98	121.70
3	M	477	GLY	C-N-CA	-8.90	99.46	121.70
1	A	545	HIS	C-N-CA	-8.88	99.50	121.70
1	A	288	THR	N-CA-C	8.88	134.97	111.00
2	B	569	THR	N-CA-C	8.85	134.89	111.00
1	A	154	ILE	N-CA-C	8.83	134.84	111.00

Continued on next page...

Continued from previous page...

Mol	Chain	Res	Type	Atoms	Z	Observed(°)	Ideal(°)
1	A	196	LEU	C-N-CA	-8.82	99.64	121.70
2	B	404	ASN	O-C-N	-8.82	108.59	122.70
1	A	242	GLU	O-C-N	-8.81	108.60	122.70
1	A	100	LEU	C-N-CA	-8.81	99.68	121.70
1	A	350	SER	O-C-N	-8.81	108.61	122.70
1	A	529	GLY	CA-C-O	-8.79	104.77	120.60
4	S	5	VAL	O-C-N	-8.79	108.63	122.70
2	B	559	ASP	CA-C-O	-8.78	101.66	120.10
1	A	461	CYS	O-C-N	-8.76	108.69	122.70
3	M	406	GLY	C-N-CA	-8.75	99.82	121.70
2	B	212	VAL	O-C-N	-8.73	108.73	122.70
1	A	539	ASN	O-C-N	-8.72	108.75	122.70
4	S	104	THR	O-C-N	8.72	136.65	122.70
1	A	432	ILE	O-C-N	8.71	136.63	122.70
4	S	167	ILE	C-N-CA	-8.69	104.05	122.30
1	A	88	ASN	C-N-CA	-8.69	99.97	121.70
4	S	50	PHE	C-N-CA	-8.69	99.97	121.70
4	S	58	LEU	CA-C-N	-8.69	98.08	117.20
2	B	557	SER	O-C-N	-8.67	108.82	122.70
3	M	405	THR	CA-C-N	-8.67	98.86	116.20
2	B	287	GLU	N-CA-C	-8.67	87.60	111.00
2	B	523	PHE	O-C-N	-8.62	108.90	122.70
1	A	325	SER	N-CA-C	-8.62	87.72	111.00
2	B	109	ALA	C-N-CA	-8.59	100.23	121.70
3	M	279	ASN	CA-C-N	8.58	136.08	117.20
4	S	98	ILE	O-C-N	8.54	136.37	122.70
2	B	223	LEU	C-N-CA	-8.53	100.37	121.70
1	A	367	ILE	O-C-N	8.53	136.35	122.70
4	S	81	ALA	CB-CA-C	8.52	122.89	110.10
4	S	100	ASP	O-C-N	8.52	136.33	122.70
3	M	347	PHE	N-CA-C	-8.51	88.02	111.00
1	A	391	LEU	C-N-CA	-8.51	100.42	121.70
1	A	413	SER	N-CA-C	-8.51	88.02	111.00
3	M	367	ALA	C-N-CA	8.49	142.94	121.70
1	A	233	PHE	O-C-N	-8.49	109.12	122.70
2	B	339	PHE	O-C-N	8.49	136.28	122.70
1	A	461	CYS	CA-C-O	8.46	137.87	120.10
2	B	336	ASN	CA-C-N	-8.46	98.60	117.20
3	M	420	THR	C-N-CA	-8.46	104.55	122.30
2	B	147	MET	O-C-N	-8.45	109.17	122.70
1	A	328	PRO	C-N-CA	-8.44	100.60	121.70
1	A	215	VAL	CA-C-O	8.43	137.80	120.10

Continued on next page...

Continued from previous page...

Mol	Chain	Res	Type	Atoms	Z	Observed(°)	Ideal(°)
1	A	281	LEU	CA-C-O	8.43	137.80	120.10
3	M	92	PHE	O-C-N	8.41	136.15	122.70
3	M	292	PRO	C-N-CA	8.41	157.31	122.00
2	B	146	LYS	C-N-CA	-8.40	100.70	121.70
2	B	472	VAL	O-C-N	-8.40	109.26	122.70
1	A	94	VAL	O-C-N	-8.39	109.27	122.70
1	A	508	LEU	N-CA-C	8.38	133.63	111.00
1	A	270	LEU	O-C-N	8.38	136.11	122.70
1	A	431	VAL	O-C-N	8.38	136.10	122.70
4	S	161	GLU	O-C-N	-8.38	109.30	122.70
2	B	574	ASN	C-N-CA	-8.36	100.80	121.70
4	S	103	GLN	O-C-N	8.36	136.08	122.70
3	M	81	SER	O-C-N	-8.35	109.34	122.70
3	M	53	HIS	CA-C-N	8.35	135.56	117.20
3	M	63	TYR	C-N-CA	-8.34	100.86	121.70
4	S	46	PHE	CA-C-O	-8.34	102.60	120.10
1	A	120	ILE	O-C-N	8.32	136.02	122.70
2	B	108	PHE	O-C-N	-8.32	109.39	122.70
1	A	534	LYS	O-C-N	-8.30	109.42	122.70
3	M	104	PHE	C-N-CA	8.30	142.44	121.70
2	B	444	THR	O-C-N	-8.29	109.44	122.70
4	S	66	ASP	N-CA-C	-8.29	88.63	111.00
3	M	51	LEU	C-N-CA	-8.28	100.99	121.70
3	M	457	GLY	N-CA-C	-8.28	92.40	113.10
2	B	295	ASN	C-N-CA	8.27	142.37	121.70
1	A	275	LEU	C-N-CA	-8.26	87.33	122.00
1	A	424	TYR	O-C-N	8.25	135.90	122.70
2	B	325	LEU	O-C-N	-8.25	109.50	122.70
2	B	366	LEU	C-N-CA	-8.25	101.08	121.70
2	B	205	PRO	O-C-N	-8.24	109.51	122.70
3	M	335	SER	O-C-N	8.24	135.89	122.70
3	M	91	THR	CA-C-O	-8.24	102.80	120.10
3	M	330	GLY	C-N-CA	8.23	142.28	121.70
3	M	54	SER	O-C-N	8.22	135.85	122.70
1	A	443	SER	N-CA-C	8.20	133.14	111.00
1	A	100	LEU	O-C-N	-8.19	109.59	122.70
1	A	573	GLU	O-C-N	-8.19	109.60	122.70
3	M	97	ASP	O-C-N	8.19	135.80	122.70
4	S	21	THR	C-N-CD	8.17	145.56	128.40
1	A	631	SER	O-C-N	8.16	135.76	122.70
2	B	56	SER	O-C-N	-8.16	109.65	122.70
1	A	103	LYS	CA-C-O	8.15	137.21	120.10

Continued on next page...

Continued from previous page...

Mol	Chain	Res	Type	Atoms	Z	Observed(°)	Ideal(°)
3	M	135	ASN	CA-C-N	-8.15	99.27	117.20
4	S	46	PHE	N-CA-C	-8.15	89.00	111.00
2	B	105	LEU	O-C-N	-8.14	109.68	122.70
1	A	192	TYR	CA-C-O	-8.14	103.01	120.10
3	M	105	ASP	C-N-CA	-8.14	101.36	121.70
3	M	126	ASN	O-C-N	8.13	135.70	122.70
1	A	212	ILE	O-C-N	-8.12	109.71	122.70
1	A	487	MET	O-C-N	-8.12	109.72	122.70
1	A	637	GLU	C-N-CA	-8.12	101.41	121.70
2	B	132	SER	O-C-N	-8.10	109.74	122.70
4	S	158	LYS	O-C-N	8.10	135.66	122.70
2	B	83	PHE	O-C-N	8.10	135.66	122.70
2	B	132	SER	C-N-CA	-8.09	101.48	121.70
3	M	108	LYS	C-N-CA	-8.08	101.50	121.70
1	A	415	ARG	N-CA-C	-8.08	89.19	111.00
2	B	505	ASP	O-C-N	-8.08	109.78	122.70
2	B	375	LEU	O-C-N	-8.07	105.77	121.10
3	M	104	PHE	O-C-N	-8.07	109.79	122.70
4	S	62	GLU	N-CA-C	8.06	132.77	111.00
2	B	184	GLY	CA-C-N	-8.06	99.46	117.20
1	A	138	ASN	N-CA-C	-8.05	89.25	111.00
3	M	74	TYR	CA-C-O	8.06	137.02	120.10
2	B	274	PRO	C-N-CA	-8.05	101.57	121.70
2	B	273	SER	N-CA-C	8.04	132.71	111.00
1	A	434	SER	O-C-N	8.02	135.53	122.70
1	A	450	TYR	O-C-N	8.02	135.53	122.70
4	S	74	GLN	O-C-N	-8.01	109.88	122.70
2	B	142	LEU	O-C-N	-8.00	109.90	122.70
2	B	115	LEU	O-C-N	7.99	135.48	122.70
2	B	515	PHE	C-N-CA	-7.98	105.54	122.30
1	A	547	VAL	O-C-N	7.97	135.45	122.70
2	B	566	ALA	C-N-CA	7.96	141.61	121.70
3	M	55	MET	CA-C-N	7.96	134.71	117.20
2	B	108	PHE	CA-C-O	7.94	136.78	120.10
2	B	296	ASP	CA-C-O	-7.94	103.43	120.10
1	A	264	SER	C-N-CA	-7.93	101.86	121.70
2	B	508	ARG	O-C-N	-7.93	110.01	122.70
2	B	299	LEU	O-C-N	7.92	135.38	122.70
1	A	281	LEU	C-N-CA	-7.92	101.90	121.70
2	B	35	TYR	O-C-N	7.92	135.37	122.70
4	S	108	SER	O-C-N	7.92	135.37	122.70
2	B	126	SER	O-C-N	-7.91	110.04	122.70

Continued on next page...

Continued from previous page...

Mol	Chain	Res	Type	Atoms	Z	Observed(°)	Ideal(°)
2	B	240	LEU	N-CA-C	7.91	132.35	111.00
3	M	262	THR	C-N-CA	-7.91	101.93	121.70
1	A	545	HIS	O-C-N	-7.90	110.06	122.70
2	B	325	LEU	C-N-CA	-7.89	101.97	121.70
3	M	292	PRO	N-CA-C	-7.89	91.59	112.10
1	A	588	LEU	O-C-N	-7.88	110.09	122.70
2	B	389	ILE	O-C-N	-7.87	110.11	122.70
2	B	293	VAL	C-N-CA	-7.86	102.05	121.70
4	S	113	PHE	N-CA-C	-7.86	89.78	111.00
2	B	153	ILE	O-C-N	7.84	135.24	122.70
1	A	158	LEU	O-C-N	7.82	135.21	122.70
1	A	218	ALA	O-C-N	7.82	135.20	122.70
1	A	281	LEU	O-C-N	-7.82	110.19	122.70
1	A	298	ILE	O-C-N	-7.82	110.19	122.70
1	A	448	GLU	N-CA-C	-7.80	89.93	111.00
4	S	132	LEU	O-C-N	7.80	135.19	122.70
3	M	72	LEU	C-N-CA	7.80	141.20	121.70
3	M	294	ASP	C-N-CA	7.80	138.68	122.30
1	A	156	PRO	O-C-N	-7.78	110.25	122.70
2	B	528	ASP	O-C-N	7.77	135.13	122.70
3	M	263	MET	C-N-CA	7.76	138.61	122.30
1	A	218	ALA	CB-CA-C	7.75	121.73	110.10
3	M	104	PHE	N-CA-CB	-7.75	96.65	110.60
1	A	365	VAL	O-C-N	-7.75	110.30	122.70
2	B	172	GLU	O-C-N	-7.74	110.31	122.70
2	B	147	MET	C-N-CA	-7.74	102.34	121.70
1	A	70	ALA	O-C-N	7.74	135.08	122.70
1	A	220	SER	O-C-N	7.74	135.08	122.70
3	M	237	THR	C-N-CA	-7.74	106.05	122.30
1	A	461	CYS	C-N-CA	-7.73	102.37	121.70
2	B	418	TYR	C-N-CA	-7.73	102.38	121.70
3	M	132	GLY	CA-C-O	7.73	134.51	120.60
1	A	304	LEU	O-C-N	-7.72	110.34	122.70
2	B	174	ALA	O-C-N	-7.71	110.36	122.70
1	A	394	GLN	CA-C-O	-7.71	103.91	120.10
1	A	330	LEU	O-C-N	7.70	135.02	122.70
1	A	504	ILE	CA-C-O	7.70	136.26	120.10
2	B	317	VAL	O-C-N	7.69	135.00	122.70
4	S	66	ASP	CA-C-N	-7.68	100.30	117.20
1	A	346	THR	O-C-N	-7.67	110.43	122.70
1	A	328	PRO	O-C-N	-7.66	110.45	122.70
2	B	237	ILE	O-C-N	-7.65	110.46	122.70

Continued on next page...

Continued from previous page...

Mol	Chain	Res	Type	Atoms	Z	Observed(°)	Ideal(°)
2	B	287	GLU	CA-C-N	-7.65	100.37	117.20
2	B	220	ALA	N-CA-C	-7.64	90.36	111.00
4	S	27	LYS	O-C-N	7.64	134.93	122.70
1	A	601	VAL	O-C-N	-7.64	110.48	122.70
2	B	478	LEU	O-C-N	-7.64	110.48	122.70
1	A	469	LEU	CA-C-O	7.63	136.13	120.10
4	S	60	SER	N-CA-C	-7.63	90.39	111.00
2	B	486	HIS	O-C-N	7.63	134.91	122.70
4	S	25	LEU	O-C-N	-7.63	106.60	121.10
4	S	36	TYR	O-C-N	7.63	134.91	122.70
4	S	109	LEU	CA-C-O	7.63	136.12	120.10
2	B	584	SER	O-C-N	-7.62	110.24	123.20
4	S	58	LEU	CA-C-O	7.62	136.10	120.10
4	S	139	GLY	C-N-CA	-7.62	102.65	121.70
4	S	59	LEU	CA-C-N	-7.61	100.46	117.20
1	A	527	GLU	C-N-CA	7.61	140.72	121.70
1	A	559	PHE	O-C-N	7.61	134.87	122.70
2	B	51	LEU	C-N-CA	-7.61	102.68	121.70
4	S	28	GLN	O-C-N	7.60	134.86	122.70
1	A	528	ASN	CA-C-O	7.60	136.06	120.10
3	M	230	LYS	CA-C-N	7.60	133.91	117.20
1	A	601	VAL	C-N-CA	-7.57	102.78	121.70
3	M	265	ASN	N-CA-C	-7.57	90.57	111.00
2	B	560	ILE	O-C-N	-7.57	110.59	122.70
1	A	279	LEU	O-C-N	-7.56	110.60	122.70
2	B	46	GLN	O-C-N	7.56	134.80	122.70
4	S	58	LEU	C-N-CA	-7.56	102.81	121.70
2	B	585	GLY	N-CA-C	-7.55	94.22	113.10
1	A	465	SER	N-CA-C	7.55	131.39	111.00
3	M	232	HIS	CA-C-O	7.54	135.94	120.10
3	M	279	ASN	N-CA-CB	-7.54	97.03	110.60
1	A	98	ASN	CA-C-N	7.53	133.77	117.20
2	B	416	LYS	O-C-N	7.51	134.72	122.70
3	M	75	TRP	O-C-N	-7.51	110.68	122.70
1	A	88	ASN	CA-C-O	7.51	135.87	120.10
1	A	338	PHE	O-C-N	7.49	134.69	122.70
1	A	439	ASP	CA-C-N	-7.48	100.75	117.20
2	B	277	CYS	C-N-CD	7.47	144.10	128.40
3	M	450	GLU	CA-C-O	7.47	135.79	120.10
1	A	596	VAL	O-C-N	7.46	134.64	122.70
4	S	8	PHE	O-C-N	-7.46	110.76	122.70
2	B	442	LEU	C-N-CA	-7.46	103.06	121.70

Continued on next page...

Continued from previous page...

Mol	Chain	Res	Type	Atoms	Z	Observed(°)	Ideal(°)
2	B	215	TYR	O-C-N	7.46	134.63	122.70
3	M	367	ALA	CB-CA-C	7.46	121.29	110.10
2	B	237	ILE	C-N-CA	-7.45	103.07	121.70
4	S	55	PRO	CA-N-CD	-7.45	101.07	111.50
2	B	488	ARG	O-C-N	7.45	134.62	122.70
3	M	280	ASP	CB-CA-C	-7.45	95.51	110.40
1	A	311	THR	O-C-N	7.45	134.61	122.70
1	A	465	SER	O-C-N	7.45	134.61	122.70
2	B	428	VAL	O-C-N	7.45	134.61	122.70
4	S	118	GLU	O-C-N	7.43	134.59	122.70
2	B	494	ALA	O-C-N	7.42	134.58	122.70
1	A	394	GLN	O-C-N	7.42	134.57	122.70
4	S	66	ASP	C-N-CA	7.42	140.25	121.70
4	S	25	LEU	C-N-CD	7.42	143.98	128.40
2	B	362	ALA	O-C-N	7.42	134.56	122.70
2	B	325	LEU	CA-C-O	7.40	135.64	120.10
3	M	266	ASP	CA-C-N	-7.39	100.94	117.20
1	A	606	PHE	O-C-N	7.38	134.52	122.70
3	M	407	THR	N-CA-C	-7.38	91.06	111.00
4	S	31	LEU	O-C-N	7.38	134.50	122.70
4	S	96	LEU	O-C-N	7.38	134.50	122.70
2	B	328	LEU	C-N-CA	-7.38	103.26	121.70
1	A	405	THR	N-CA-C	7.35	130.85	111.00
2	B	515	PHE	O-C-N	-7.35	110.70	123.20
1	A	519	LEU	O-C-N	-7.35	110.70	123.20
3	M	421	GLY	O-C-N	-7.35	107.14	121.10
3	M	130	GLU	CA-C-N	-7.34	101.06	117.20
2	B	223	LEU	O-C-N	-7.33	110.98	122.70
2	B	142	LEU	C-N-CA	-7.32	103.39	121.70
2	B	493	LEU	O-C-N	7.32	134.42	122.70
2	B	554	LYS	O-C-N	7.32	134.41	122.70
2	B	183	ALA	N-CA-C	7.31	130.75	111.00
4	S	54	PRO	C-N-CD	7.31	143.76	128.40
1	A	177	ILE	O-C-N	7.31	134.40	122.70
4	S	25	LEU	CA-C-O	7.31	135.44	120.10
4	S	167	ILE	N-CA-C	7.31	130.73	111.00
2	B	192	LEU	O-C-N	7.30	134.38	122.70
2	B	408	VAL	O-C-N	7.29	134.37	122.70
1	A	162	ILE	O-C-N	7.29	134.37	122.70
1	A	260	PHE	C-N-CA	-7.28	103.49	121.70
1	A	511	VAL	O-C-N	7.28	134.35	122.70
4	S	127	THR	O-C-N	7.28	134.35	122.70

Continued on next page...

Continued from previous page...

Mol	Chain	Res	Type	Atoms	Z	Observed(°)	Ideal(°)
2	B	270	SER	N-CA-C	-7.28	91.34	111.00
1	A	399	ASP	O-C-N	7.27	134.33	122.70
2	B	531	ARG	O-C-N	7.27	134.32	122.70
3	M	426	LYS	C-N-CA	7.25	139.84	121.70
3	M	460	ILE	C-N-CA	7.25	137.52	122.30
1	A	391	LEU	O-C-N	-7.25	111.11	122.70
1	A	384	LEU	O-C-N	7.24	134.28	122.70
1	A	573	GLU	C-N-CA	-7.23	103.62	121.70
2	B	243	TRP	O-C-N	7.23	134.27	122.70
1	A	265	GLN	CA-C-O	-7.22	104.93	120.10
4	S	80	TYR	CA-C-O	7.22	135.27	120.10
4	S	149	ILE	O-C-N	7.22	134.25	122.70
2	B	415	LEU	O-C-N	7.22	134.25	122.70
2	B	220	ALA	CB-CA-C	7.20	120.89	110.10
4	S	125	TRP	CA-C-O	-7.19	105.00	120.10
1	A	381	GLU	O-C-N	-7.19	111.19	122.70
3	M	53	HIS	O-C-N	-7.18	111.20	122.70
3	M	319	SER	N-CA-C	-7.18	91.61	111.00
1	A	529	GLY	N-CA-C	-7.18	95.15	113.10
1	A	256	LEU	O-C-N	7.18	134.19	122.70
3	M	129	VAL	C-N-CA	7.18	139.64	121.70
1	A	488	ARG	O-C-N	7.16	134.15	122.70
3	M	54	SER	C-N-CA	7.16	139.59	121.70
1	A	388	VAL	O-C-N	7.16	134.15	122.70
2	B	260	LEU	C-N-CD	7.15	143.42	128.40
1	A	441	TYR	CA-C-N	-7.15	101.47	117.20
4	S	47	GLN	CA-C-N	-7.15	101.47	117.20
2	B	288	TYR	N-CA-C	-7.15	91.70	111.00
4	S	83	LEU	CA-C-O	7.15	135.11	120.10
2	B	23	ALA	N-CA-CB	7.14	120.10	110.10
1	A	306	GLU	O-C-N	-7.14	111.27	122.70
2	B	150	LEU	O-C-N	7.14	134.13	122.70
1	A	300	LYS	N-CA-C	7.13	130.25	111.00
2	B	267	ASP	O-C-N	-7.12	111.30	122.70
1	A	298	ILE	CA-C-O	7.12	135.05	120.10
2	B	185	LYS	O-C-N	-7.12	111.31	122.70
4	S	84	TYR	O-C-N	-7.11	111.32	122.70
1	A	263	LEU	C-N-CA	-7.11	103.93	121.70
1	A	517	TRP	O-C-N	7.10	134.07	122.70
2	B	478	LEU	CA-C-O	7.10	135.01	120.10
3	M	96	ILE	O-C-N	7.09	134.05	122.70
3	M	61	GLU	O-C-N	7.09	134.04	122.70

Continued on next page...

Continued from previous page...

Mol	Chain	Res	Type	Atoms	Z	Observed(°)	Ideal(°)
3	M	118	TYR	CA-C-O	-7.09	105.21	120.10
1	A	539	ASN	C-N-CA	-7.08	103.99	121.70
1	A	447	PHE	O-C-N	7.08	134.03	122.70
2	B	226	LEU	O-C-N	7.08	134.03	122.70
2	B	504	ALA	O-C-N	7.08	134.03	122.70
1	A	582	ILE	O-C-N	7.07	134.01	122.70
1	A	355	LEU	O-C-N	7.06	134.00	122.70
3	M	351	SER	N-CA-CB	-7.06	99.91	110.50
2	B	555	LEU	O-C-N	7.06	134.00	122.70
2	B	526	CYS	O-C-N	7.06	134.51	121.10
2	B	611	ALA	O-C-N	7.05	133.99	122.70
1	A	225	LEU	CA-C-O	-7.05	105.29	120.10
2	B	169	VAL	O-C-N	7.05	133.98	122.70
1	A	566	PHE	N-CA-C	7.05	130.03	111.00
1	A	635	ALA	O-C-N	7.05	133.97	122.70
2	B	143	SER	C-N-CA	-7.05	104.08	121.70
2	B	422	ALA	N-CA-CB	7.04	119.96	110.10
2	B	521	ILE	C-N-CA	-7.04	104.11	121.70
3	M	458	LEU	C-N-CA	7.03	139.28	121.70
2	B	323	ASN	O-C-N	7.03	133.95	122.70
1	A	441	TYR	CA-C-O	7.03	134.86	120.10
1	A	302	ASN	CA-C-N	-7.03	101.75	117.20
2	B	389	ILE	C-N-CA	-7.03	104.14	121.70
1	A	602	GLU	O-C-N	7.02	133.93	122.70
1	A	225	LEU	O-C-N	7.02	133.93	122.70
2	B	213	LEU	O-C-N	7.02	133.93	122.70
2	B	572	GLU	O-C-N	7.02	133.93	122.70
3	M	110	SER	O-C-N	7.02	133.93	122.70
1	A	442	SER	N-CA-C	-7.01	92.06	111.00
2	B	97	VAL	O-C-N	7.01	133.91	122.70
3	M	403	THR	N-CA-C	-7.00	92.09	111.00
2	B	355	ASN	O-C-N	7.00	133.90	122.70
2	B	216	LYS	C-N-CA	-7.00	104.20	121.70
1	A	476	GLN	O-C-N	6.99	133.89	122.70
3	M	21	GLY	N-CA-C	-6.99	95.62	113.10
2	B	99	ARG	O-C-N	6.97	133.86	122.70
4	S	143	GLU	CA-C-N	-6.97	101.86	117.20
2	B	56	SER	C-N-CA	-6.97	104.27	121.70
1	A	605	GLU	O-C-N	6.97	133.85	122.70
3	M	425	THR	C-N-CA	6.97	139.12	121.70
4	S	30	LEU	O-C-N	6.97	133.85	122.70
1	A	301	GLY	N-CA-C	-6.97	95.69	113.10

Continued on next page...

Continued from previous page...

Mol	Chain	Res	Type	Atoms	Z	Observed(°)	Ideal(°)
1	A	204	VAL	C-N-CA	-6.96	104.29	121.70
3	M	105	ASP	O-C-N	-6.96	111.56	122.70
1	A	166	LEU	O-C-N	6.96	133.83	122.70
1	A	271	ARG	O-C-N	6.95	133.83	122.70
2	B	324	ALA	O-C-N	6.95	133.82	122.70
1	A	140	VAL	O-C-N	-6.95	111.58	122.70
2	B	511	ILE	O-C-N	-6.95	111.58	122.70
2	B	573	GLU	C-N-CA	-6.95	104.33	121.70
1	A	453	VAL	O-C-N	6.95	133.81	122.70
3	M	50	TYR	N-CA-CB	-6.95	98.10	110.60
3	M	106	LYS	CA-C-N	-6.94	101.92	117.20
2	B	102	HIS	CA-C-O	6.94	134.68	120.10
1	A	569	ASP	O-C-N	-6.93	111.61	122.70
2	B	252	LEU	O-C-N	6.93	133.79	122.70
1	A	383	ASN	O-C-N	6.93	133.78	122.70
1	A	138	ASN	O-C-N	6.93	133.78	122.70
1	A	608	ARG	O-C-N	6.92	133.78	122.70
1	A	623	MET	O-C-N	6.92	133.78	122.70
1	A	188	VAL	O-C-N	6.92	133.78	122.70
1	A	457	LEU	O-C-N	6.92	133.77	122.70
2	B	271	GLU	C-N-CA	-6.92	107.77	122.30
3	M	277	GLU	O-C-N	-6.92	111.64	122.70
1	A	555	LEU	O-C-N	6.91	133.76	122.70
1	A	257	LEU	O-C-N	6.90	133.75	122.70
3	M	128	CYS	C-N-CA	-6.90	104.45	121.70
1	A	279	LEU	C-N-CA	-6.90	104.46	121.70
1	A	607	LEU	O-C-N	6.90	133.73	122.70
2	B	104	TYR	O-C-N	6.89	133.73	122.70
4	S	68	VAL	C-N-CA	-6.89	104.46	121.70
2	B	568	VAL	C-N-CA	6.88	138.91	121.70
1	A	621	LEU	C-N-CA	-6.88	93.11	122.00
4	S	18	LYS	O-C-N	-6.87	111.71	122.70
4	S	157	ASN	O-C-N	6.87	133.69	122.70
3	M	317	MET	C-N-CA	-6.86	104.55	121.70
4	S	99	LEU	O-C-N	6.86	133.68	122.70
2	B	544	THR	O-C-N	6.86	133.67	122.70
4	S	24	ASP	O-C-N	6.86	133.67	122.70
2	B	457	HIS	CA-C-O	6.85	134.49	120.10
4	S	99	LEU	CA-C-O	-6.85	105.71	120.10
4	S	116	VAL	O-C-N	6.85	133.66	122.70
2	B	594	ALA	O-C-N	6.84	133.65	122.70
2	B	425	PRO	O-C-N	6.84	133.65	122.70

Continued on next page...

Continued from previous page...

Mol	Chain	Res	Type	Atoms	Z	Observed(°)	Ideal(°)
1	A	140	VAL	CA-C-O	6.84	134.46	120.10
1	A	624	LEU	O-C-N	6.84	133.64	122.70
2	B	66	ILE	O-C-N	6.84	133.64	122.70
2	B	105	LEU	C-N-CA	-6.84	104.60	121.70
2	B	559	ASP	O-C-N	6.84	133.64	122.70
1	A	348	PHE	O-C-N	6.83	133.62	122.70
1	A	102	GLN	O-C-N	6.83	133.62	122.70
4	S	148	ARG	O-C-N	6.82	133.62	122.70
4	S	8	PHE	CA-C-O	6.82	134.43	120.10
1	A	156	PRO	C-N-CA	-6.81	104.67	121.70
3	M	123	LEU	O-C-N	6.81	133.60	122.70
4	S	16	LEU	O-C-N	-6.81	111.80	122.70
2	B	134	LEU	O-C-N	6.81	133.59	122.70
2	B	78	ASP	O-C-N	-6.81	111.81	122.70
2	B	452	LYS	O-C-N	6.81	133.59	122.70
1	A	477	PHE	O-C-N	6.80	133.59	122.70
3	M	47	SER	N-CA-C	-6.80	92.64	111.00
4	S	33	GLU	O-C-N	6.80	133.58	122.70
3	M	54	SER	CB-CA-C	-6.80	97.19	110.10
3	M	54	SER	CA-C-N	-6.79	102.26	117.20
3	M	89	CYS	O-C-N	6.79	133.56	122.70
4	S	101	LEU	O-C-N	6.79	133.56	122.70
1	A	581	LEU	O-C-N	6.78	133.55	122.70
1	A	466	ASP	O-C-N	6.78	133.54	122.70
1	A	610	SER	O-C-N	6.77	133.54	122.70
3	M	63	TYR	CA-C-N	-6.77	102.30	117.20
2	B	255	TYR	O-C-N	6.77	133.53	122.70
4	S	128	LEU	O-C-N	6.77	133.53	122.70
2	B	489	ILE	O-C-N	6.77	133.53	122.70
1	A	632	PHE	O-C-N	6.76	133.52	122.70
3	M	130	GLU	N-CA-CB	6.76	122.77	110.60
1	A	515	CYS	O-C-N	6.76	133.52	122.70
2	B	174	ALA	C-N-CA	-6.76	104.80	121.70
1	A	125	THR	O-C-N	6.76	133.51	122.70
1	A	496	ILE	O-C-N	6.76	133.51	122.70
3	M	455	VAL	C-N-CA	-6.75	104.82	121.70
2	B	566	ALA	CB-CA-C	6.75	120.23	110.10
1	A	601	VAL	CA-C-O	6.75	134.27	120.10
2	B	411	ILE	O-C-N	6.75	133.50	122.70
2	B	253	ILE	O-C-N	6.75	133.49	122.70
1	A	265	GLN	N-CA-CB	6.74	122.74	110.60
1	A	196	LEU	CA-C-O	6.74	134.26	120.10

Continued on next page...

Continued from previous page...

Mol	Chain	Res	Type	Atoms	Z	Observed(°)	Ideal(°)
1	A	240	LEU	O-C-N	-6.74	111.92	122.70
1	A	418	ILE	N-CA-C	6.74	129.18	111.00
2	B	579	PRO	CA-C-N	6.73	132.01	117.20
1	A	67	LYS	O-C-N	6.73	133.47	122.70
3	M	113	LYS	O-C-N	6.73	133.47	122.70
2	B	581	TYR	N-CA-C	-6.72	92.85	111.00
2	B	361	GLN	O-C-N	6.72	133.45	122.70
3	M	85	GLY	N-CA-C	-6.72	96.31	113.10
2	B	412	PHE	O-C-N	6.71	133.44	122.70
3	M	385	ARG	O-C-N	-6.71	111.97	122.70
2	B	63	MET	O-C-N	6.70	133.42	122.70
2	B	454	LEU	O-C-N	6.70	133.42	122.70
1	A	432	ILE	CA-C-O	-6.70	106.03	120.10
2	B	100	LEU	O-C-N	6.70	133.42	122.70
3	M	425	THR	N-CA-C	-6.70	92.91	111.00
3	M	424	PHE	N-CA-C	-6.70	92.92	111.00
2	B	356	LYS	O-C-N	6.70	133.41	122.70
4	S	150	VAL	O-C-N	6.70	133.41	122.70
1	A	392	MET	O-C-N	6.69	133.41	122.70
3	M	284	SER	O-C-N	-6.69	108.39	121.10
1	A	117	ASP	O-C-N	6.69	133.40	122.70
2	B	267	ASP	C-N-CA	-6.69	104.98	121.70
1	A	87	CYS	O-C-N	6.69	133.40	122.70
1	A	236	LEU	O-C-N	6.68	133.40	122.70
2	B	521	ILE	N-CA-C	-6.67	92.99	111.00
3	M	265	ASN	CA-C-N	6.67	131.87	117.20
1	A	600	SER	O-C-N	6.67	133.37	122.70
4	S	54	PRO	N-CA-C	-6.67	94.76	112.10
2	B	273	SER	N-CA-CB	-6.67	100.50	110.50
3	M	16	PHE	CA-C-O	6.67	134.10	120.10
2	B	337	THR	CA-C-O	-6.66	106.11	120.10
2	B	592	TYR	O-C-N	6.66	133.35	122.70
1	A	556	VAL	O-C-N	6.66	133.35	122.70
1	A	128	LEU	O-C-N	6.65	133.34	122.70
1	A	522	PHE	O-C-N	6.65	133.34	122.70
3	M	319	SER	C-N-CA	6.65	138.31	121.70
2	B	154	ILE	CA-C-O	-6.64	106.15	120.10
4	S	137	GLN	CA-C-N	-6.64	102.92	116.20
2	B	139	LEU	O-C-N	6.64	133.33	122.70
2	B	460	SER	N-CA-C	6.64	128.93	111.00
2	B	285	GLU	N-CA-C	-6.64	93.08	111.00
3	M	60	LEU	O-C-N	6.63	133.31	122.70

Continued on next page...

Continued from previous page...

Mol	Chain	Res	Type	Atoms	Z	Observed(°)	Ideal(°)
1	A	307	ASP	N-CA-C	-6.63	93.09	111.00
2	B	267	ASP	CA-C-O	6.63	134.02	120.10
2	B	399	LEU	C-N-CA	-6.63	105.13	121.70
1	A	495	ILE	O-C-N	6.62	133.30	122.70
2	B	513	TRP	O-C-N	6.62	133.30	122.70
2	B	589	SER	O-C-N	6.62	133.30	122.70
1	A	265	GLN	O-C-N	-6.62	112.11	122.70
1	A	462	GLN	C-N-CA	-6.62	105.15	121.70
2	B	251	LEU	O-C-N	6.62	133.29	122.70
2	B	552	SER	O-C-N	6.62	133.29	122.70
1	A	386	ALA	O-C-N	6.61	133.28	122.70
1	A	472	LYS	O-C-N	6.61	133.28	122.70
1	A	91	ILE	O-C-N	6.61	133.27	122.70
2	B	359	LEU	O-C-N	6.61	133.27	122.70
2	B	582	ASP	CA-C-N	6.61	131.73	117.20
1	A	80	TYR	N-CA-CB	6.60	122.48	110.60
1	A	107	TYR	O-C-N	6.60	133.26	122.70
1	A	454	ILE	O-C-N	6.60	133.26	122.70
4	S	35	VAL	O-C-N	6.60	133.26	122.70
1	A	406	GLY	N-CA-C	-6.60	96.61	113.10
1	A	558	VAL	O-C-N	6.59	133.25	122.70
2	B	62	ALA	O-C-N	6.59	133.24	122.70
3	M	394	GLN	CA-C-O	6.59	133.94	120.10
1	A	436	CYS	O-C-N	-6.58	112.17	122.70
2	B	508	ARG	CA-C-O	6.58	133.93	120.10
1	A	451	ASN	O-C-N	6.58	133.22	122.70
4	S	32	LEU	O-C-N	6.58	133.22	122.70
2	B	447	GLU	O-C-N	6.58	133.22	122.70
2	B	607	ILE	O-C-N	6.57	133.22	122.70
3	M	4	SER	CA-C-O	6.57	133.90	120.10
1	A	296	ASN	O-C-N	6.57	133.21	122.70
2	B	123	LEU	O-C-N	6.57	133.21	122.70
4	S	106	VAL	O-C-N	6.57	133.21	122.70
2	B	474	VAL	O-C-N	6.57	133.21	122.70
2	B	591	MET	O-C-N	6.57	133.21	122.70
2	B	119	SER	O-C-N	6.56	133.20	122.70
2	B	346	THR	O-C-N	6.56	133.20	122.70
2	B	244	SER	O-C-N	6.55	133.19	122.70
2	B	604	GLU	O-C-N	6.55	133.19	122.70
2	B	453	TRP	O-C-N	6.55	133.18	122.70
4	S	4	ALA	CA-C-O	6.55	133.86	120.10
2	B	475	ILE	O-C-N	6.55	133.17	122.70

Continued on next page...

Continued from previous page...

Mol	Chain	Res	Type	Atoms	Z	Observed(°)	Ideal(°)
3	M	124	ILE	O-C-N	6.55	133.18	122.70
1	A	146	ALA	O-C-N	6.54	133.17	122.70
2	B	65	ARG	O-C-N	6.54	133.17	122.70
3	M	91	THR	O-C-N	6.54	133.17	122.70
1	A	332	TYR	O-C-N	6.54	133.17	122.70
2	B	388	PRO	C-N-CA	6.54	138.05	121.70
3	M	107	ASP	CA-C-N	6.54	131.59	117.20
3	M	122	SER	O-C-N	6.54	133.16	122.70
2	B	507	ALA	O-C-N	6.54	133.16	122.70
1	A	507	GLN	C-N-CA	-6.54	105.36	121.70
2	B	588	ILE	O-C-N	6.53	133.16	122.70
2	B	344	VAL	O-C-N	6.53	133.15	122.70
4	S	130	SER	O-C-N	6.53	133.15	122.70
2	B	123	LEU	CA-C-O	-6.53	106.40	120.10
1	A	145	ILE	O-C-N	6.52	133.13	122.70
1	A	611	LEU	O-C-N	6.52	133.13	122.70
4	S	124	ASN	O-C-N	6.52	133.13	122.70
1	A	492	ILE	O-C-N	6.51	133.12	122.70
1	A	139	ASP	O-C-N	6.51	133.12	122.70
2	B	467	VAL	O-C-N	6.51	133.12	122.70
2	B	363	ILE	O-C-N	6.51	133.11	122.70
2	B	301	LEU	O-C-N	6.50	133.11	122.70
2	B	146	LYS	CA-C-N	6.50	131.51	117.20
3	M	458	LEU	N-CA-C	-6.50	93.45	111.00
1	A	554	ALA	O-C-N	6.49	133.09	122.70
1	A	388	VAL	CA-C-O	-6.49	106.47	120.10
3	M	394	GLN	O-C-N	-6.49	112.17	123.20
4	S	145	ASN	O-C-N	6.49	133.08	122.70
3	M	94	GLU	O-C-N	6.48	133.07	122.70
1	A	289	SER	CA-C-O	6.48	133.71	120.10
1	A	550	VAL	O-C-N	6.48	133.06	122.70
1	A	612	GLU	O-C-N	6.48	133.06	122.70
2	B	154	ILE	O-C-N	6.47	133.04	122.70
4	S	106	VAL	CA-C-O	-6.46	106.53	120.10
1	A	516	ILE	O-C-N	6.46	133.03	122.70
2	B	145	MET	C-N-CA	-6.46	105.56	121.70
2	B	548	ILE	O-C-N	6.46	133.03	122.70
2	B	177	ILE	O-C-N	6.45	133.02	122.70
1	A	64	LEU	CA-C-O	6.45	133.65	120.10
1	A	562	TRP	O-C-N	6.44	133.01	122.70
2	B	395	LYS	O-C-N	6.44	133.01	122.70
3	M	63	TYR	O-C-N	6.44	133.01	122.70

Continued on next page...

Continued from previous page...

Mol	Chain	Res	Type	Atoms	Z	Observed(°)	Ideal(°)
3	M	389	SER	O-C-N	6.44	133.01	122.70
2	B	81	LEU	C-N-CA	-6.44	105.60	121.70
4	S	29	LYS	O-C-N	6.44	133.00	122.70
2	B	230	PHE	O-C-N	-6.43	112.41	122.70
4	S	83	LEU	O-C-N	-6.43	112.41	122.70
1	A	471	SER	O-C-N	6.43	132.99	122.70
2	B	229	HIS	C-N-CA	6.43	137.77	121.70
2	B	67	ILE	O-C-N	6.42	132.97	122.70
2	B	135	ARG	O-C-N	6.42	132.97	122.70
2	B	137	PHE	O-C-N	6.42	132.97	122.70
1	A	105	VAL	O-C-N	6.42	134.10	123.20
1	A	252	ILE	O-C-N	6.41	132.96	122.70
1	A	297	CYS	O-C-N	-6.41	112.44	122.70
3	M	457	GLY	CA-C-N	-6.41	103.09	117.20
1	A	407	SER	N-CA-C	-6.41	93.69	111.00
3	M	125	PHE	O-C-N	6.41	132.96	122.70
4	S	155	GLU	O-C-N	6.41	132.96	122.70
2	B	490	ILE	O-C-N	6.41	132.95	122.70
1	A	331	ARG	O-C-N	6.40	132.94	122.70
1	A	474	GLY	O-C-N	6.40	132.94	122.70
3	M	51	LEU	O-C-N	-6.40	112.47	122.70
1	A	467	LYS	O-C-N	6.39	132.93	122.70
4	S	156	LEU	O-C-N	6.39	132.93	122.70
1	A	185	LEU	O-C-N	6.39	132.93	122.70
1	A	415	ARG	C-N-CA	-6.39	105.72	121.70
1	A	574	ILE	O-C-N	6.39	132.93	122.70
2	B	181	TYR	O-C-N	-6.39	112.47	122.70
4	S	37	GLU	O-C-N	6.39	132.93	122.70
4	S	4	ALA	O-C-N	-6.39	112.47	122.70
3	M	281	GLY	N-CA-C	-6.39	97.12	113.10
1	A	629	LEU	C-N-CA	-6.39	95.18	122.00
2	B	170	ARG	O-C-N	-6.38	112.35	123.20
1	A	492	ILE	CA-C-O	-6.38	106.69	120.10
1	A	475	GLU	O-C-N	6.38	132.91	122.70
2	B	271	GLU	N-CA-C	6.38	128.23	111.00
2	B	302	PHE	O-C-N	6.38	132.91	122.70
1	A	313	MET	O-C-N	6.38	132.90	122.70
4	S	153	VAL	O-C-N	6.38	132.91	122.70
3	M	277	GLU	C-N-CA	-6.38	105.76	121.70
4	S	165	SER	C-N-CA	-6.37	105.77	121.70
2	B	181	TYR	C-N-CA	-6.37	105.77	121.70
2	B	612	ARG	O-C-N	6.37	132.89	122.70

Continued on next page...

Continued from previous page...

Mol	Chain	Res	Type	Atoms	Z	Observed(°)	Ideal(°)
2	B	178	ILE	O-C-N	6.37	132.89	122.70
1	A	364	ASP	O-C-N	6.37	132.89	122.70
1	A	186	PHE	O-C-N	6.36	132.88	122.70
2	B	102	HIS	C-N-CA	-6.36	105.80	121.70
2	B	341	GLU	O-C-N	6.36	132.88	122.70
2	B	414	GLU	O-C-N	6.36	132.87	122.70
1	A	155	THR	CA-C-O	-6.36	106.75	120.10
1	A	569	ASP	CA-C-O	-6.36	106.75	120.10
2	B	158	VAL	CA-C-O	-6.35	106.76	120.10
2	B	248	LEU	O-C-N	6.35	132.86	122.70
1	A	159	ALA	O-C-N	6.35	132.86	122.70
1	A	508	LEU	CA-C-O	-6.35	106.77	120.10
2	B	158	VAL	O-C-N	6.35	132.86	122.70
1	A	628	VAL	O-C-N	6.35	132.86	122.70
3	M	111	ILE	O-C-N	6.35	132.86	122.70
2	B	455	ILE	O-C-N	6.35	132.85	122.70
2	B	193	LEU	O-C-N	6.34	132.85	122.70
2	B	417	TYR	O-C-N	6.34	132.85	122.70
1	A	598	GLU	O-C-N	6.34	132.84	122.70
4	S	70	ASN	CA-C-O	6.34	133.41	120.10
3	M	65	TYR	C-N-CA	-6.34	105.86	121.70
4	S	129	GLU	O-C-N	6.34	132.84	122.70
1	A	522	PHE	CA-C-O	-6.33	106.80	120.10
2	B	305	SER	O-C-N	6.33	132.83	122.70
2	B	306	LEU	CA-C-O	-6.33	106.80	120.10
1	A	552	ILE	O-C-N	6.33	132.83	122.70
1	A	182	ILE	O-C-N	6.33	132.82	122.70
2	B	321	CYS	O-C-N	6.32	132.82	122.70
1	A	421	PRO	O-C-N	6.32	132.81	122.70
2	B	360	LEU	O-C-N	6.32	132.81	122.70
2	B	59	VAL	O-C-N	6.32	132.81	122.70
2	B	79	VAL	CA-C-O	-6.32	106.83	120.10
3	M	105	ASP	N-CA-C	6.32	128.06	111.00
4	S	1	MET	O-C-N	-6.32	112.59	122.70
3	M	290	PHE	C-N-CA	6.31	137.49	121.70
1	A	147	LEU	O-C-N	6.31	132.80	122.70
1	A	200	PHE	O-C-N	6.31	132.80	122.70
1	A	578	LEU	O-C-N	6.31	132.79	122.70
2	B	151	ALA	O-C-N	6.31	133.08	121.10
1	A	603	VAL	O-C-N	6.30	132.79	122.70
2	B	249	ILE	O-C-N	6.30	132.78	122.70
1	A	68	THR	O-C-N	6.30	132.78	122.70

Continued on next page...

Continued from previous page...

Mol	Chain	Res	Type	Atoms	Z	Observed(°)	Ideal(°)
4	S	80	TYR	O-C-N	-6.30	112.62	122.70
1	A	202	LYS	O-C-N	6.30	132.78	122.70
2	B	195	ILE	O-C-N	6.29	132.77	122.70
2	B	587	ARG	O-C-N	6.29	132.77	122.70
2	B	613	MET	O-C-N	6.29	132.77	122.70
1	A	215	VAL	O-C-N	-6.29	112.63	122.70
2	B	150	LEU	CA-C-O	-6.29	106.89	120.10
1	A	625	LEU	O-C-N	-6.29	112.64	122.70
3	M	402	SER	CA-C-N	6.29	131.03	117.20
2	B	111	ASN	N-CA-C	6.28	127.97	111.00
2	B	306	LEU	O-C-N	6.28	132.75	122.70
2	B	519	ALA	N-CA-CB	-6.28	101.30	110.10
2	B	510	GLY	CA-C-O	-6.28	109.29	120.60
2	B	409	LYS	O-C-N	6.28	132.75	122.70
2	B	429	VAL	O-C-N	6.28	132.75	122.70
1	A	429	VAL	O-C-N	6.28	132.74	122.70
2	B	105	LEU	CA-C-O	6.28	133.28	120.10
2	B	410	GLU	O-C-N	6.28	132.75	122.70
1	A	316	LEU	O-C-N	6.28	132.74	122.70
2	B	485	LYS	O-C-N	6.28	132.74	122.70
3	M	223	HIS	O-C-N	-6.28	112.66	122.70
4	S	38	LEU	O-C-N	6.28	132.74	122.70
1	A	579	LYS	O-C-N	6.27	132.74	122.70
2	B	432	ALA	O-C-N	6.27	132.74	122.70
3	M	55	MET	C-N-CA	-6.27	106.02	121.70
4	S	107	GLU	O-C-N	6.27	132.74	122.70
1	A	278	ILE	C-N-CA	6.27	137.38	121.70
3	M	135	ASN	N-CA-CB	6.27	121.88	110.60
2	B	614	ILE	O-C-N	6.27	132.73	122.70
3	M	290	PHE	N-CA-C	-6.27	94.08	111.00
1	A	385	LYS	O-C-N	6.26	132.72	122.70
2	B	610	ARG	O-C-N	6.26	132.72	122.70
1	A	613	ALA	O-C-N	6.26	132.72	122.70
2	B	151	ALA	CA-C-O	-6.26	106.95	120.10
1	A	297	CYS	CA-C-O	6.26	133.25	120.10
1	A	404	GLN	C-N-CA	6.26	137.35	121.70
1	A	302	ASN	CA-C-O	6.26	133.24	120.10
1	A	337	LEU	O-C-N	6.26	132.71	122.70
1	A	508	LEU	O-C-N	6.26	132.99	121.10
2	B	334	MET	N-CA-C	6.26	127.89	111.00
2	B	191	GLU	O-C-N	6.25	132.71	122.70
2	B	472	VAL	CA-C-O	6.25	133.23	120.10

Continued on next page...

Continued from previous page...

Mol	Chain	Res	Type	Atoms	Z	Observed(°)	Ideal(°)
3	M	86	PRO	O-C-N	6.25	132.70	122.70
1	A	315	CYS	O-C-N	6.25	132.70	122.70
2	B	214	ALA	O-C-N	6.25	132.70	122.70
2	B	43	ASN	O-C-N	6.25	132.97	121.10
2	B	155	LEU	O-C-N	6.25	132.69	122.70
3	M	107	ASP	CA-C-O	-6.25	106.98	120.10
4	S	105	PHE	O-C-N	6.25	132.70	122.70
2	B	374	PHE	O-C-N	6.25	132.69	122.70
1	A	419	ILE	N-CA-C	6.24	127.86	111.00
2	B	116	THR	O-C-N	6.24	132.69	122.70
1	A	395	PHE	C-N-CA	-6.24	106.10	121.70
1	A	83	ASP	CA-C-N	-6.24	103.48	117.20
2	B	262	LYS	C-N-CA	-6.24	95.81	122.00
1	A	533	ILE	CA-C-O	-6.23	107.01	120.10
2	B	437	SER	O-C-N	6.23	132.67	122.70
1	A	604	LEU	O-C-N	6.23	132.67	122.70
2	B	605	PHE	O-C-N	6.23	132.67	122.70
1	A	473	ILE	O-C-N	6.22	133.78	123.20
2	B	464	SER	O-C-N	6.22	132.66	122.70
1	A	124	ALA	O-C-N	6.22	132.66	122.70
1	A	143	VAL	O-C-N	6.22	133.78	123.20
2	B	246	SER	O-C-N	6.22	132.66	122.70
2	B	244	SER	CA-C-O	-6.22	107.03	120.10
4	S	85	PHE	CA-C-O	6.22	133.16	120.10
1	A	290	VAL	CA-C-O	-6.22	107.04	120.10
2	B	209	SER	O-C-N	6.22	132.65	122.70
3	M	272	LEU	C-N-CA	6.21	137.23	121.70
2	B	58	GLU	O-C-N	6.21	132.64	122.70
2	B	233	TYR	O-C-N	6.21	132.64	122.70
2	B	615	SER	O-C-N	6.21	132.64	122.70
1	A	141	VAL	O-C-N	6.21	132.63	122.70
1	A	387	ILE	O-C-N	6.21	132.63	122.70
1	A	428	MET	O-C-N	6.21	132.63	122.70
1	A	353	ASP	O-C-N	6.20	132.63	122.70
2	B	436	LEU	O-C-N	6.20	132.62	122.70
2	B	136	CYS	O-C-N	6.20	132.62	122.70
2	B	337	THR	O-C-N	6.20	132.62	122.70
2	B	450	VAL	O-C-N	6.20	132.62	122.70
3	M	126	ASN	CA-C-O	-6.20	107.08	120.10
4	S	120	ASP	O-C-N	6.20	132.62	122.70
1	A	154	ILE	C-N-CA	-6.20	106.21	121.70
2	B	157	THR	O-C-N	6.19	132.61	122.70

Continued on next page...

Continued from previous page...

Mol	Chain	Res	Type	Atoms	Z	Observed(°)	Ideal(°)
1	A	298	ILE	C-N-CA	-6.19	106.22	121.70
1	A	439	ASP	N-CA-CB	6.19	121.74	110.60
2	B	593	ASN	O-C-N	6.19	132.61	122.70
3	M	131	ALA	C-N-CA	6.19	135.30	122.30
1	A	535	ILE	CA-C-N	-6.18	103.59	117.20
1	A	421	PRO	CA-C-O	-6.18	105.36	120.20
1	A	309	PHE	O-C-N	6.17	132.58	122.70
1	A	295	VAL	CA-C-O	-6.17	107.15	120.10
2	B	296	ASP	O-C-N	6.17	132.82	121.10
1	A	323	CYS	CA-C-N	6.17	130.76	117.20
2	B	272	GLY	N-CA-C	-6.17	97.69	113.10
2	B	468	LEU	O-C-N	6.17	132.56	122.70
1	A	211	ASP	O-C-N	6.16	132.56	122.70
2	B	582	ASP	O-C-N	-6.16	112.84	122.70
2	B	595	VAL	O-C-N	6.16	132.56	122.70
2	B	433	VAL	O-C-N	6.16	132.56	122.70
3	M	78	ALA	CB-CA-C	6.16	119.34	110.10
2	B	27	THR	C-N-CA	6.16	137.09	121.70
2	B	319	LEU	O-C-N	6.16	132.55	122.70
1	A	335	CYS	O-C-N	6.16	132.55	122.70
1	A	162	ILE	CA-C-O	-6.16	107.17	120.10
2	B	47	LEU	O-C-N	6.15	132.55	122.70
2	B	550	VAL	O-C-N	6.15	132.55	122.70
3	M	131	ALA	N-CA-C	6.15	127.61	111.00
1	A	607	LEU	CA-C-O	-6.14	107.20	120.10
2	B	342	ALA	O-C-N	6.14	132.52	122.70
1	A	336	ILE	O-C-N	6.13	132.51	122.70
1	A	551	LEU	O-C-N	6.13	132.51	122.70
1	A	274	LEU	C-N-CA	-6.13	106.37	121.70
1	A	127	LEU	O-C-N	6.13	132.50	122.70
3	M	3	LEU	CA-C-O	6.12	132.96	120.10
2	B	209	SER	CA-C-O	-6.12	107.24	120.10
3	M	308	SER	O-C-N	6.12	132.49	122.70
1	A	258	LYS	O-C-N	6.12	132.49	122.70
2	B	549	LEU	O-C-N	6.11	132.48	122.70
1	A	81	GLY	C-N-CA	6.11	136.97	121.70
2	B	138	ALA	O-C-N	6.10	132.47	122.70
2	B	430	ILE	O-C-N	6.10	132.47	122.70
2	B	204	ASP	O-C-N	6.10	132.70	121.10
2	B	435	SER	O-C-N	6.10	132.47	122.70
1	A	390	THR	O-C-N	6.10	132.46	122.70
2	B	190	GLU	O-C-N	6.10	132.46	122.70

Continued on next page...

Continued from previous page...

Mol	Chain	Res	Type	Atoms	Z	Observed(°)	Ideal(°)
2	B	139	LEU	CA-C-O	-6.10	107.30	120.10
2	B	82	TYR	O-C-N	-6.10	112.95	122.70
1	A	454	ILE	CA-C-O	-6.09	107.31	120.10
2	B	343	LEU	O-C-N	6.09	132.45	122.70
3	M	90	PHE	O-C-N	6.09	132.44	122.70
2	B	98	LYS	O-C-N	6.09	132.44	122.70
2	B	166	SER	O-C-N	6.09	132.44	122.70
2	B	585	GLY	O-C-N	6.08	132.44	122.70
1	A	400	VAL	O-C-N	6.08	132.43	122.70
2	B	462	ASN	C-N-CA	-6.08	106.50	121.70
4	S	81	ALA	N-CA-C	-6.08	94.58	111.00
1	A	295	VAL	O-C-N	6.08	132.43	122.70
2	B	44	PRO	O-C-N	6.08	132.43	122.70
3	M	368	ASP	CA-C-O	-6.08	107.33	120.10
2	B	324	ALA	CA-C-O	-6.08	107.34	120.10
4	S	139	GLY	N-CA-C	-6.08	97.90	113.10
2	B	512	VAL	O-C-N	6.08	132.42	122.70
1	A	566	PHE	C-N-CA	6.07	136.88	121.70
2	B	101	ILE	O-C-N	6.07	132.42	122.70
3	M	367	ALA	N-CA-C	-6.07	94.62	111.00
2	B	522	GLU	O-C-N	6.06	132.40	122.70
1	A	633	PHE	CA-C-O	-6.06	107.38	120.10
2	B	511	ILE	CA-C-O	6.06	132.82	120.10
3	M	429	ASP	C-N-CA	-6.06	106.56	121.70
1	A	271	ARG	CA-C-O	-6.06	107.38	120.10
1	A	105	VAL	C-N-CA	6.05	135.01	122.30
1	A	372	ILE	O-C-N	6.05	132.39	122.70
2	B	320	SER	O-C-N	6.05	132.39	122.70
1	A	557	LYS	O-C-N	6.05	132.38	122.70
1	A	163	ALA	O-C-N	-6.05	113.02	122.70
1	A	465	SER	CA-C-O	6.04	132.79	120.10
1	A	339	TYR	O-C-N	6.04	132.37	122.70
2	B	579	PRO	N-CA-C	6.04	127.81	112.10
4	S	151	ALA	O-C-N	6.04	132.36	122.70
3	M	60	LEU	N-CA-C	-6.04	94.70	111.00
4	S	64	ASN	C-N-CA	-6.04	106.61	121.70
1	A	98	ASN	O-C-N	-6.04	113.04	122.70
2	B	525	ILE	O-C-N	6.04	132.36	122.70
2	B	96	LYS	O-C-N	6.03	132.35	122.70
2	B	466	SER	O-C-N	6.03	132.35	122.70
3	M	283	PHE	N-CA-C	-6.03	94.72	111.00
2	B	216	LYS	O-C-N	-6.03	113.05	122.70

Continued on next page...

Continued from previous page...

Mol	Chain	Res	Type	Atoms	Z	Observed(°)	Ideal(°)
2	B	250	GLU	O-C-N	6.02	132.34	122.70
2	B	145	MET	CA-C-N	6.02	130.44	117.20
4	S	150	VAL	CA-C-O	-6.02	107.46	120.10
1	A	325	SER	CA-C-N	-6.02	103.96	117.20
1	A	493	ALA	O-C-N	6.02	132.33	122.70
1	A	620	GLY	CA-C-O	-6.02	109.77	120.60
2	B	196	LEU	O-C-N	6.02	132.33	122.70
4	S	43	ASN	C-N-CA	-6.02	106.66	121.70
1	A	389	GLN	O-C-N	6.02	132.33	122.70
1	A	518	CYS	CA-C-O	-6.01	107.47	120.10
2	B	543	GLU	O-C-N	6.01	132.32	122.70
1	A	142	LYS	O-C-N	6.01	132.32	122.70
2	B	314	ASN	O-C-N	6.01	132.53	121.10
2	B	451	MET	O-C-N	6.01	132.32	122.70
1	A	129	LYS	O-C-N	6.01	132.32	122.70
1	A	229	ASN	C-N-CD	6.01	141.03	128.40
1	A	631	SER	CA-C-O	-6.01	107.47	120.10
2	B	74	ASP	C-N-CA	6.01	136.73	121.70
1	A	278	ILE	CA-C-O	-6.01	107.48	120.10
2	B	194	ASP	O-C-N	6.01	132.32	122.70
1	A	107	TYR	CA-C-O	-6.00	107.49	120.10
1	A	140	VAL	C-N-CA	-6.00	106.69	121.70
2	B	471	TYR	O-C-N	6.00	132.31	122.70
2	B	590	GLN	O-C-N	6.00	132.31	122.70
4	S	16	LEU	CA-C-O	6.00	132.71	120.10
1	A	237	SER	CA-C-O	-6.00	107.50	120.10
1	A	158	LEU	CA-C-O	-6.00	107.50	120.10
2	B	469	ASP	O-C-N	6.00	132.30	122.70
4	S	85	PHE	O-C-N	-6.00	113.11	122.70
3	M	381	ASN	N-CA-C	-6.00	94.81	111.00
1	A	436	CYS	C-N-CA	-5.99	106.72	121.70
3	M	65	TYR	O-C-N	5.99	132.29	122.70
1	A	121	LEU	O-C-N	5.99	132.29	122.70
1	A	425	LYS	O-C-N	5.99	132.28	122.70
2	B	103	LEU	O-C-N	5.99	132.28	122.70
1	A	553	LEU	O-C-N	5.99	132.28	122.70
1	A	130	LYS	O-C-N	5.98	132.27	122.70
1	A	214	VAL	O-C-N	5.98	132.27	122.70
1	A	513	ARG	O-C-N	5.98	132.27	122.70
2	B	112	ASP	N-CA-C	5.98	127.14	111.00
2	B	446	TRP	O-C-N	5.98	132.27	122.70
1	A	104	ARG	O-C-N	5.98	132.26	122.70

Continued on next page...

Continued from previous page...

Mol	Chain	Res	Type	Atoms	Z	Observed(°)	Ideal(°)
1	A	296	ASN	CA-C-O	-5.98	107.55	120.10
1	A	633	PHE	O-C-N	5.98	132.26	122.70
2	B	469	ASP	CA-C-O	-5.98	107.55	120.10
3	M	120	ARG	O-C-N	5.97	132.26	122.70
2	B	64	LYS	O-C-N	5.97	132.25	122.70
3	M	77	LEU	CA-C-O	5.97	132.64	120.10
3	M	232	HIS	O-C-N	-5.97	113.15	122.70
2	B	322	CYS	O-C-N	5.96	132.24	122.70
2	B	568	VAL	O-C-N	5.96	132.24	122.70
1	A	71	VAL	CA-C-O	-5.96	107.58	120.10
1	A	577	VAL	O-C-N	5.96	132.24	122.70
1	A	538	GLU	C-N-CA	-5.96	106.81	121.70
1	A	312	ALA	O-C-N	5.96	132.23	122.70
2	B	371	GLN	O-C-N	5.95	132.23	122.70
1	A	528	ASN	O-C-N	-5.95	113.09	123.20
1	A	576	MET	O-C-N	5.95	132.22	122.70
2	B	207	VAL	O-C-N	5.95	132.22	122.70
4	S	48	SER	C-N-CA	-5.94	106.84	121.70
1	A	249	ASN	O-C-N	5.94	132.20	122.70
2	B	553	ALA	O-C-N	5.94	132.20	122.70
2	B	596	LEU	O-C-N	5.94	132.21	122.70
2	B	413	LYS	O-C-N	5.94	132.20	122.70
1	A	491	THR	O-C-N	5.94	132.20	122.70
2	B	238	LYS	C-N-CA	5.93	136.54	121.70
2	B	585	GLY	CA-C-O	-5.93	109.92	120.60
1	A	625	LEU	C-N-CA	-5.93	106.88	121.70
1	A	588	LEU	CA-C-O	5.93	132.55	120.10
1	A	108	LEU	O-C-N	5.93	132.18	122.70
2	B	55	ASN	O-C-N	5.93	132.18	122.70
2	B	358	MET	O-C-N	5.92	132.18	122.70
1	A	458	ALA	O-C-N	5.92	132.17	122.70
1	A	600	SER	CA-C-O	-5.92	107.66	120.10
3	M	401	LYS	C-N-CA	-5.92	106.90	121.70
2	B	510	GLY	O-C-N	5.92	132.17	122.70
2	B	530	LEU	O-C-N	5.92	132.17	122.70
3	M	367	ALA	N-CA-CB	-5.92	101.81	110.10
2	B	225	LEU	CA-C-N	-5.91	104.19	117.20
2	B	546	CYS	O-C-N	5.91	132.16	122.70
1	A	514	GLU	O-C-N	5.91	132.15	122.70
2	B	333	GLN	N-CA-C	-5.91	95.05	111.00
4	S	58	LEU	N-CA-CB	5.91	122.22	110.40
2	B	569	THR	CA-C-N	-5.90	104.40	116.20

Continued on next page...

Continued from previous page...

Mol	Chain	Res	Type	Atoms	Z	Observed(°)	Ideal(°)
2	B	567	GLN	C-N-CA	-5.90	106.95	121.70
3	M	426	LYS	N-CA-C	-5.90	95.07	111.00
1	A	287	ALA	N-CA-C	5.89	126.91	111.00
1	A	584	PHE	O-C-N	5.89	132.13	122.70
2	B	131	ASN	O-C-N	5.89	132.13	122.70
2	B	293	VAL	O-C-N	-5.89	113.27	122.70
1	A	609	LEU	O-C-N	5.89	132.12	122.70
1	A	486	SER	N-CA-C	-5.89	95.11	111.00
2	B	184	GLY	CA-C-O	5.89	131.20	120.60
1	A	230	PRO	CA-C-O	-5.88	106.08	120.20
2	B	604	GLU	CA-C-N	-5.88	104.25	117.20
1	A	165	ASP	O-C-N	5.88	132.11	122.70
1	A	512	LEU	O-C-N	5.88	132.11	122.70
2	B	418	TYR	O-C-N	-5.88	113.29	122.70
3	M	261	ASN	C-N-CA	-5.88	107.00	121.70
3	M	121	ILE	O-C-N	5.88	132.11	122.70
3	M	321	GLY	C-N-CA	-5.88	107.01	121.70
4	S	159	ALA	O-C-N	5.88	132.10	122.70
1	A	257	LEU	CA-C-O	-5.87	107.77	120.10
2	B	396	ILE	O-C-N	5.87	132.10	122.70
2	B	526	CYS	CA-C-O	-5.87	107.77	120.10
1	A	122	MET	O-C-N	5.87	132.09	122.70
1	A	276	PRO	O-C-N	-5.87	113.31	122.70
2	B	529	VAL	O-C-N	5.87	132.09	122.70
1	A	155	THR	O-C-N	5.86	132.24	121.10
2	B	245	GLN	O-C-N	5.86	132.08	122.70
2	B	476	ARG	O-C-N	5.86	132.08	122.70
1	A	499	ILE	C-N-CA	-5.86	107.05	121.70
3	M	278	ILE	CA-C-N	-5.86	104.31	117.20
4	S	117	ASN	CA-C-O	-5.86	107.79	120.10
1	A	494	ASN	O-C-N	5.86	132.07	122.70
2	B	449	HIS	O-C-N	5.86	132.07	122.70
3	M	6	TYR	CA-C-O	5.86	132.40	120.10
1	A	369	SER	O-C-N	5.85	132.07	122.70
1	A	490	VAL	O-C-N	5.85	132.06	122.70
3	M	74	TYR	O-C-N	-5.85	113.34	122.70
4	S	117	ASN	O-C-N	5.85	132.06	122.70
1	A	479	ASN	O-C-N	5.84	132.04	122.70
2	B	212	VAL	C-N-CA	-5.84	107.10	121.70
2	B	523	PHE	C-N-CA	-5.84	107.10	121.70
2	B	551	LEU	O-C-N	5.84	132.04	122.70
2	B	555	LEU	CA-C-O	-5.84	107.84	120.10

Continued on next page...

Continued from previous page...

Mol	Chain	Res	Type	Atoms	Z	Observed(°)	Ideal(°)
1	A	417	PRO	N-CA-C	-5.83	96.93	112.10
3	M	256	VAL	O-C-N	5.83	132.03	122.70
1	A	294	SER	O-C-N	5.83	132.03	122.70
1	A	549	GLU	O-C-N	5.83	132.03	122.70
2	B	303	LEU	CA-C-O	-5.83	107.86	120.10
2	B	355	ASN	CA-C-O	-5.83	107.86	120.10
3	M	306	LEU	C-N-CA	-5.82	107.14	121.70
1	A	545	HIS	CA-C-O	5.82	132.32	120.10
2	B	43	ASN	CA-C-O	-5.82	107.88	120.10
2	B	366	LEU	CA-C-O	5.82	132.32	120.10
1	A	110	ALA	O-C-N	-5.82	113.39	122.70
2	B	444	THR	CA-C-O	5.82	132.31	120.10
4	S	1	MET	CA-C-O	5.81	132.30	120.10
2	B	232	ARG	O-C-N	5.80	131.99	122.70
2	B	394	TRP	O-C-N	5.80	131.99	122.70
1	A	64	LEU	C-N-CA	-5.80	107.19	121.70
1	A	603	VAL	CA-C-O	-5.80	107.93	120.10
2	B	48	VAL	O-C-N	5.80	131.98	122.70
2	B	79	VAL	O-C-N	5.79	131.97	122.70
4	S	32	LEU	CA-C-O	-5.79	107.93	120.10
1	A	371	ALA	O-C-N	5.79	131.97	122.70
2	B	33	SER	O-C-N	5.79	131.97	122.70
3	M	130	GLU	CB-CA-C	5.79	121.99	110.40
3	M	265	ASN	CA-C-O	-5.79	107.94	120.10
1	A	217	ALA	O-C-N	5.79	131.96	122.70
1	A	317	GLU	O-C-N	5.79	131.96	122.70
2	B	86	VAL	O-C-N	5.79	131.96	122.70
2	B	434	LYS	O-C-N	5.78	131.96	122.70
2	B	247	TYR	O-C-N	5.78	131.95	122.70
2	B	490	ILE	CA-C-O	-5.78	107.96	120.10
2	B	492	LYS	O-C-N	5.78	131.95	122.70
2	B	586	SER	O-C-N	5.78	131.95	122.70
1	A	152	THR	C-N-CA	-5.78	107.26	121.70
1	A	350	SER	C-N-CA	-5.77	107.27	121.70
2	B	457	HIS	O-C-N	-5.77	113.47	122.70
2	B	60	ARG	O-C-N	5.77	131.93	122.70
2	B	120	ILE	CA-C-O	-5.77	107.99	120.10
4	S	61	ASN	C-N-CA	-5.77	107.28	121.70
4	S	96	LEU	CA-C-O	-5.77	107.99	120.10
2	B	275	ARG	N-CA-CB	5.76	120.97	110.60
2	B	402	LEU	N-CA-C	5.76	126.56	111.00
1	A	575	LYS	O-C-N	5.76	131.92	122.70

Continued on next page...

Continued from previous page...

Mol	Chain	Res	Type	Atoms	Z	Observed(°)	Ideal(°)
2	B	241	ASP	O-C-N	5.76	131.91	122.70
1	A	356	ILE	O-C-N	5.75	131.91	122.70
3	M	421	GLY	CA-C-O	5.75	130.96	120.60
2	B	176	ALA	O-C-N	5.75	131.90	122.70
2	B	99	ARG	CA-C-O	-5.75	108.03	120.10
3	M	59	ASP	O-C-N	-5.75	113.50	122.70
1	A	216	SER	O-C-N	5.74	131.89	122.70
2	B	318	ILE	CA-C-O	-5.74	108.04	120.10
2	B	239	GLN	C-N-CA	5.74	136.05	121.70
1	A	452	ALA	O-C-N	5.74	131.88	122.70
1	A	582	ILE	CA-C-O	-5.74	108.05	120.10
2	B	189	HIS	O-C-N	5.74	131.88	122.70
3	M	133	GLU	C-N-CA	-5.74	97.90	122.00
1	A	430	ASN	O-C-N	5.74	131.88	122.70
1	A	123	LEU	O-C-N	5.73	131.87	122.70
2	B	412	PHE	CA-C-O	-5.73	108.06	120.10
2	B	448	SER	O-C-N	5.73	131.87	122.70
3	M	280	ASP	CA-C-N	-5.73	104.74	116.20
2	B	174	ALA	CA-C-O	5.73	132.13	120.10
3	M	225	VAL	O-C-N	5.73	131.86	122.70
2	B	300	ASP	O-C-N	5.72	131.86	122.70
1	A	478	ARG	O-C-N	5.72	131.86	122.70
1	A	558	VAL	CA-C-O	-5.72	108.08	120.10
2	B	438	ARG	O-C-N	5.72	131.86	122.70
2	B	532	ARG	O-C-N	5.72	131.86	122.70
2	B	341	GLU	CA-C-O	-5.72	108.09	120.10
1	A	572	PHE	C-N-CA	5.72	136.00	121.70
4	S	84	TYR	CA-C-O	5.72	132.11	120.10
2	B	545	ARG	O-C-N	5.72	131.85	122.70
1	A	109	ALA	O-C-N	5.72	131.84	122.70
2	B	393	ILE	O-C-N	5.72	131.85	122.70
2	B	426	GLU	O-C-N	5.71	131.84	122.70
2	B	318	ILE	O-C-N	5.71	131.83	122.70
3	M	237	THR	O-C-N	-5.71	113.50	123.20
1	A	413	SER	C-N-CA	-5.70	107.44	121.70
1	A	275	LEU	O-C-N	-5.70	110.27	121.10
2	B	593	ASN	CA-C-O	-5.70	108.13	120.10
4	S	154	ASP	O-C-N	5.70	131.82	122.70
3	M	306	LEU	CA-C-O	5.70	132.07	120.10
2	B	423	HIS	N-CA-C	-5.70	95.62	111.00
3	M	103	TYR	CB-CA-C	5.70	121.79	110.40
2	B	436	LEU	CA-C-O	-5.70	108.14	120.10

Continued on next page...

Continued from previous page...

Mol	Chain	Res	Type	Atoms	Z	Observed(°)	Ideal(°)
1	A	88	ASN	O-C-N	-5.69	113.59	122.70
4	S	98	ILE	CA-C-O	-5.69	108.14	120.10
2	B	133	GLU	O-C-N	5.69	131.81	122.70
3	M	398	ILE	O-C-N	-5.69	113.59	122.70
2	B	303	LEU	O-C-N	5.69	131.81	122.70
2	B	456	ASP	O-C-N	5.69	131.81	122.70
1	A	75	THR	O-C-N	5.69	131.80	122.70
1	A	265	GLN	C-N-CA	5.69	135.92	121.70
3	M	447	ILE	CA-C-O	5.69	132.04	120.10
4	S	164	ASP	O-C-N	-5.69	113.60	122.70
2	B	495	ASP	O-C-N	5.69	131.80	122.70
1	A	325	SER	C-N-CA	5.68	135.91	121.70
2	B	22	ALA	N-CA-C	-5.68	95.66	111.00
4	S	152	SER	O-C-N	5.68	131.79	122.70
2	B	211	ALA	CA-C-O	-5.68	108.18	120.10
2	B	391	ALA	O-C-N	5.67	131.78	122.70
3	M	18	TYR	CA-C-O	5.67	132.01	120.10
1	A	108	LEU	CA-C-O	-5.67	108.19	120.10
2	B	265	VAL	CA-C-N	-5.67	104.72	117.20
1	A	126	ASN	O-C-N	5.67	131.77	122.70
1	A	543	TYR	N-CA-C	5.67	126.31	111.00
1	A	422	GLU	CA-C-O	-5.67	108.19	120.10
2	B	383	VAL	CA-C-N	-5.67	104.73	117.20
2	B	122	SER	O-C-N	5.67	131.76	122.70
3	M	85	GLY	CA-C-O	-5.66	110.41	120.60
3	M	292	PRO	C-N-CD	-5.66	108.14	120.60
2	B	173	VAL	O-C-N	5.66	131.76	122.70
1	A	241	TYR	O-C-N	5.66	131.76	122.70
1	A	491	THR	CA-C-O	-5.66	108.21	120.10
2	B	322	CYS	CA-C-O	-5.66	108.21	120.10
2	B	392	SER	O-C-N	5.66	131.76	122.70
1	A	233	PHE	CA-C-O	5.66	131.98	120.10
1	A	217	ALA	CA-C-O	-5.65	108.23	120.10
1	A	575	LYS	CA-C-O	-5.65	108.23	120.10
2	B	547	GLN	O-C-N	5.65	131.74	122.70
3	M	5	PHE	O-C-N	-5.65	113.66	122.70
2	B	407	ASN	O-C-N	5.65	131.74	122.70
1	A	448	GLU	C-N-CA	5.65	135.82	121.70
2	B	172	GLU	C-N-CA	-5.65	107.58	121.70
3	M	99	ILE	CA-C-O	-5.65	108.24	120.10
3	M	385	ARG	CA-C-O	5.64	131.95	120.10
1	A	173	THR	C-N-CA	5.64	135.80	121.70

Continued on next page...

Continued from previous page...

Mol	Chain	Res	Type	Atoms	Z	Observed(°)	Ideal(°)
4	S	74	GLN	CA-C-O	5.64	131.94	120.10
3	M	284	SER	C-N-CD	5.64	140.24	128.40
4	S	158	LYS	CA-C-O	-5.64	108.26	120.10
3	M	396	GLN	O-C-N	-5.63	113.69	122.70
1	A	316	LEU	CA-C-O	-5.63	108.27	120.10
1	A	630	PRO	O-C-N	5.63	131.71	122.70
3	M	88	ASP	O-C-N	5.63	131.71	122.70
3	M	377	LYS	N-CA-C	5.63	126.20	111.00
1	A	293	GLU	O-C-N	5.63	131.70	122.70
1	A	333	ILE	O-C-N	5.63	131.70	122.70
2	B	179	LYS	O-C-N	5.63	131.70	122.70
1	A	145	ILE	CA-C-O	-5.62	108.29	120.10
2	B	204	ASP	CA-C-O	-5.62	108.29	120.10
1	A	253	ILE	O-C-N	5.62	131.70	122.70
2	B	189	HIS	CA-C-O	-5.62	108.29	120.10
2	B	496	LEU	O-C-N	5.62	131.69	122.70
3	M	87	LEU	O-C-N	5.62	131.69	122.70
2	B	605	PHE	CA-C-O	-5.62	108.31	120.10
1	A	106	GLY	CA-C-O	-5.61	110.50	120.60
1	A	635	ALA	CA-C-O	-5.61	108.32	120.10
2	B	155	LEU	CA-C-O	-5.61	108.32	120.10
2	B	415	LEU	CA-C-O	-5.61	108.32	120.10
1	A	224	GLU	C-N-CA	-5.61	107.68	121.70
3	M	135	ASN	O-C-N	5.61	131.67	122.70
4	S	137	GLN	CA-C-O	5.61	131.87	120.10
2	B	340	ILE	O-C-N	5.60	131.66	122.70
1	A	230	PRO	CA-N-CD	5.60	119.53	111.70
3	M	103	TYR	N-CA-CB	5.60	120.67	110.60
2	B	215	TYR	CA-C-O	-5.59	108.36	120.10
1	A	90	HIS	O-C-N	5.59	131.65	122.70
2	B	138	ALA	CA-C-O	-5.59	108.36	120.10
3	M	230	LYS	O-C-N	-5.59	113.75	122.70
3	M	368	ASP	O-C-N	5.59	131.71	121.10
1	A	580	GLU	O-C-N	5.58	131.64	122.70
4	S	34	GLN	O-C-N	5.58	131.64	122.70
3	M	421	GLY	C-N-CD	5.58	140.12	128.40
3	M	444	ALA	N-CA-CB	-5.58	102.28	110.10
1	A	135	ASP	CA-C-N	5.58	127.36	116.20
2	B	472	VAL	C-N-CA	-5.58	107.75	121.70
1	A	319	LEU	CA-C-O	5.58	131.82	120.10
1	A	489	GLU	O-C-N	5.57	131.61	122.70
1	A	334	SER	O-C-N	5.57	131.61	122.70

Continued on next page...

Continued from previous page...

Mol	Chain	Res	Type	Atoms	Z	Observed(°)	Ideal(°)
1	A	551	LEU	CA-C-O	-5.57	108.41	120.10
3	M	343	ASN	C-N-CA	-5.57	107.78	121.70
3	M	387	GLU	O-C-N	-5.57	113.80	122.70
1	A	456	ASP	O-C-N	5.56	131.60	122.70
3	M	117	ASN	O-C-N	5.56	131.60	122.70
2	B	522	GLU	N-CA-C	5.56	126.00	111.00
3	M	46	SER	O-C-N	-5.56	113.81	122.70
2	B	493	LEU	CA-C-O	-5.56	108.43	120.10
2	B	540	GLU	N-CA-C	5.55	126.00	111.00
1	A	387	ILE	CA-C-O	-5.55	108.44	120.10
2	B	569	THR	C-N-CA	5.55	133.96	122.30
4	S	86	THR	O-C-N	-5.55	113.82	122.70
1	A	177	ILE	CA-C-O	-5.55	108.45	120.10
2	B	302	PHE	CA-C-O	-5.55	108.44	120.10
2	B	387	ASP	N-CA-C	5.55	125.98	111.00
1	A	137	ASN	C-N-CA	-5.55	107.83	121.70
4	S	109	LEU	C-N-CA	-5.55	107.83	121.70
1	A	156	PRO	CA-C-O	5.54	133.51	120.20
2	B	117	LEU	O-C-N	5.54	131.57	122.70
2	B	245	GLN	CA-C-O	-5.54	108.46	120.10
3	M	61	GLU	CA-C-N	-5.54	105.00	117.20
1	A	578	LEU	CA-C-O	-5.54	108.46	120.10
2	B	457	HIS	C-N-CA	-5.54	107.85	121.70
1	A	501	ASN	C-N-CA	5.54	135.55	121.70
1	A	222	ILE	O-C-N	5.54	131.56	122.70
1	A	368	ARG	O-C-N	5.54	131.56	122.70
3	M	279	ASN	CA-C-O	-5.54	108.47	120.10
1	A	333	ILE	CA-C-O	-5.54	108.48	120.10
1	A	514	GLU	CA-C-O	-5.54	108.47	120.10
1	A	80	TYR	CB-CA-C	5.53	121.47	110.40
1	A	160	ARG	O-C-N	5.53	131.55	122.70
2	B	275	ARG	CA-C-N	-5.53	105.03	117.20
1	A	139	ASP	CA-C-O	-5.53	108.49	120.10
1	A	426	ILE	O-C-N	5.53	131.55	122.70
1	A	265	GLN	CB-CA-C	5.53	121.45	110.40
2	B	67	ILE	CA-C-O	-5.52	108.50	120.10
4	S	131	VAL	O-C-N	5.52	131.53	122.70
1	A	251	TRP	O-C-N	5.52	131.53	122.70
1	A	341	ILE	O-C-N	5.52	132.58	123.20
1	A	380	ASP	O-C-N	5.51	131.52	122.70
2	B	36	THR	CA-C-O	-5.51	108.52	120.10
1	A	229	ASN	C-N-CA	-5.51	98.85	122.00

Continued on next page...

Continued from previous page...

Mol	Chain	Res	Type	Atoms	Z	Observed(°)	Ideal(°)
1	A	441	TYR	N-CA-C	-5.51	96.12	111.00
3	M	136	VAL	CA-C-O	5.51	131.67	120.10
2	B	531	ARG	CA-C-O	-5.51	108.53	120.10
4	S	87	PHE	CA-C-O	5.51	131.67	120.10
1	A	184	ALA	O-C-N	5.51	131.51	122.70
1	A	254	ILE	O-C-N	5.51	131.51	122.70
1	A	292	TYR	O-C-N	5.51	131.51	122.70
1	A	63	ASP	N-CA-C	5.50	125.86	111.00
1	A	391	LEU	CA-C-O	5.50	131.66	120.10
2	B	477	MET	O-C-N	5.50	131.51	122.70
1	A	416	ILE	C-N-CA	-5.50	98.89	122.00
2	B	431	MET	O-C-N	5.50	131.50	122.70
3	M	16	PHE	O-C-N	-5.50	113.90	122.70
3	M	296	LYS	C-N-CA	-5.50	107.96	121.70
2	B	109	ALA	CB-CA-C	-5.49	101.86	110.10
2	B	458	MET	C-N-CA	-5.49	107.97	121.70
3	M	59	ASP	CA-C-O	-5.49	108.58	120.10
1	A	268	PRO	O-C-N	5.49	131.48	122.70
1	A	583	GLU	O-C-N	5.48	131.47	122.70
4	S	78	LYS	CA-C-O	5.48	131.61	120.10
1	A	352	PHE	O-C-N	5.48	131.47	122.70
2	B	475	ILE	CA-C-O	-5.48	108.59	120.10
1	A	72	LEU	O-C-N	5.48	131.47	122.70
1	A	255	ARG	O-C-N	5.48	131.47	122.70
1	A	487	MET	C-N-CA	-5.48	108.01	121.70
2	B	36	THR	O-C-N	5.47	131.46	122.70
4	S	49	SER	CA-C-N	5.47	129.25	117.20
1	A	375	VAL	O-C-N	-5.47	113.94	122.70
1	A	191	GLN	C-N-CA	5.47	135.38	121.70
2	B	290	SER	N-CA-C	5.47	125.76	111.00
1	A	188	VAL	CA-C-O	-5.47	108.62	120.10
2	B	73	ASP	C-N-CA	-5.47	108.03	121.70
3	M	267	ILE	C-N-CA	-5.47	110.82	122.30
1	A	392	MET	CA-C-O	-5.46	108.62	120.10
2	B	491	PHE	O-C-N	5.46	131.44	122.70
2	B	29	LYS	C-N-CA	-5.46	108.05	121.70
2	B	156	HIS	O-C-N	5.46	131.43	122.70
2	B	363	ILE	CA-C-O	-5.46	108.64	120.10
1	A	193	PRO	CA-C-O	-5.46	107.11	120.20
1	A	634	ASN	O-C-N	5.46	131.43	122.70
2	B	323	ASN	CA-C-O	-5.46	108.64	120.10
3	M	130	GLU	O-C-N	5.45	131.43	122.70

Continued on next page...

Continued from previous page...

Mol	Chain	Res	Type	Atoms	Z	Observed(°)	Ideal(°)
1	A	513	ARG	CA-C-O	-5.45	108.65	120.10
1	A	419	ILE	CA-C-N	-5.45	105.22	117.20
2	B	126	SER	CA-C-O	5.45	131.54	120.10
3	M	384	GLY	O-C-N	5.45	131.42	122.70
4	S	21	THR	C-N-CA	-5.45	99.13	122.00
4	S	125	TRP	O-C-N	5.44	131.41	122.70
1	A	175	PRO	O-C-N	5.44	131.40	122.70
2	B	127	LEU	C-N-CA	-5.44	108.10	121.70
2	B	586	SER	CA-C-O	-5.44	108.68	120.10
1	A	313	MET	CA-C-O	-5.44	108.69	120.10
2	B	345	ARG	O-C-N	5.43	131.39	122.70
3	M	307	SER	O-C-N	5.43	131.40	122.70
1	A	397	ASP	O-C-N	-5.43	114.01	122.70
1	A	427	LYS	O-C-N	5.43	131.39	122.70
2	B	68	SER	O-C-N	5.43	131.38	122.70
2	B	388	PRO	O-C-N	5.42	131.38	122.70
3	M	59	ASP	N-CA-C	-5.42	96.36	111.00
1	A	455	MET	O-C-N	5.42	131.37	122.70
3	M	354	ASP	N-CA-CB	5.42	120.36	110.60
1	A	136	GLY	CA-C-N	5.42	129.12	117.20
4	S	138	GLY	C-N-CA	5.42	133.67	122.30
1	A	611	LEU	CA-C-O	-5.41	108.73	120.10
2	B	430	ILE	CA-C-O	-5.41	108.74	120.10
1	A	106	GLY	O-C-N	5.41	131.35	122.70
1	A	467	LYS	C-N-CA	-5.41	108.18	121.70
3	M	98	ARG	O-C-N	5.41	131.35	122.70
1	A	244	LEU	CA-C-O	5.41	131.45	120.10
3	M	51	LEU	N-CA-CB	-5.41	99.59	110.40
1	A	308	ASP	N-CA-C	5.40	125.59	111.00
1	A	593	THR	O-C-N	5.40	131.35	122.70
1	A	91	ILE	CA-C-O	-5.40	108.75	120.10
2	B	256	CYS	O-C-N	-5.40	114.06	122.70
2	B	248	LEU	CA-C-O	-5.40	108.77	120.10
2	B	482	ASN	O-C-N	5.40	131.36	121.10
2	B	286	ILE	CA-C-N	-5.40	105.33	117.20
2	B	120	ILE	O-C-N	5.39	131.32	122.70
4	S	87	PHE	O-C-N	-5.39	114.08	122.70
2	B	61	ASP	O-C-N	5.39	131.32	122.70
2	B	365	PHE	O-C-N	5.39	131.32	122.70
1	A	566	PHE	O-C-N	5.39	131.32	122.70
1	A	604	LEU	CA-C-O	-5.39	108.79	120.10
2	B	357	GLU	O-C-N	5.39	131.32	122.70

Continued on next page...

Continued from previous page...

Mol	Chain	Res	Type	Atoms	Z	Observed(°)	Ideal(°)
2	B	364	HIS	O-C-N	5.39	131.32	122.70
2	B	609	ASP	O-C-N	5.38	131.31	122.70
1	A	305	GLU	CA-C-N	5.37	129.02	117.20
2	B	213	LEU	CA-C-O	-5.37	108.83	120.10
1	A	559	PHE	CA-C-O	-5.37	108.83	120.10
1	A	624	LEU	CA-C-O	-5.37	108.83	120.10
2	B	287	GLU	O-C-N	5.37	131.29	122.70
1	A	518	CYS	O-C-N	5.37	131.29	122.70
1	A	466	ASP	N-CA-C	-5.36	96.52	111.00
3	M	7	ILE	CA-C-O	-5.36	108.84	120.10
4	S	76	ILE	O-C-N	-5.36	114.12	122.70
2	B	351	GLU	CA-C-O	5.36	131.34	120.10
3	M	469	GLY	C-N-CA	-5.35	108.33	121.70
1	A	390	THR	CA-C-O	-5.35	108.87	120.10
4	S	49	SER	CA-C-O	-5.35	108.87	120.10
2	B	360	LEU	CA-C-O	-5.35	108.87	120.10
2	B	577	ASN	CA-C-N	5.34	132.06	117.10
1	A	216	SER	CA-C-O	-5.34	108.89	120.10
1	A	382	ASP	O-C-N	5.34	131.24	122.70
2	B	544	THR	CA-C-O	-5.34	108.89	120.10
3	M	450	GLU	O-C-N	-5.34	114.16	122.70
2	B	219	TYR	N-CA-C	5.33	125.40	111.00
3	M	441	GLY	CA-C-N	-5.33	105.47	117.20
1	A	181	ALA	O-C-N	5.33	131.23	122.70
1	A	440	ASN	N-CA-C	-5.33	96.61	111.00
2	B	178	ILE	CA-C-O	-5.33	108.91	120.10
2	B	533	LEU	O-C-N	5.33	131.23	122.70
1	A	226	SER	C-N-CA	-5.33	108.39	121.70
2	B	354	GLY	N-CA-C	-5.33	99.79	113.10
1	A	314	ALA	O-C-N	5.32	131.22	122.70
1	A	433	ILE	CA-C-O	-5.32	108.92	120.10
1	A	345	ASN	O-C-N	5.32	131.21	122.70
1	A	528	ASN	C-N-CA	-5.32	111.14	122.30
3	M	25	PRO	N-CA-C	-5.32	98.28	112.10
1	A	277	LYS	CA-C-N	5.31	128.89	117.20
3	M	1	MET	C-N-CA	-5.31	108.42	121.70
4	S	18	LYS	CA-C-O	5.31	131.25	120.10
1	A	516	ILE	CA-C-O	-5.31	108.95	120.10
2	B	94	ASP	O-C-N	5.30	131.19	122.70
2	B	439	CYS	CA-C-O	-5.30	108.96	120.10
2	B	216	LYS	CA-C-O	5.30	131.23	120.10
3	M	405	THR	N-CA-CB	-5.30	100.23	110.30

Continued on next page...

Continued from previous page...

Mol	Chain	Res	Type	Atoms	Z	Observed(°)	Ideal(°)
1	A	335	CYS	CA-C-O	-5.30	108.98	120.10
1	A	505	ASN	O-C-N	-5.30	114.22	122.70
1	A	115	TYR	C-N-CA	5.29	134.94	121.70
1	A	398	GLU	N-CA-C	5.29	125.30	111.00
3	M	55	MET	O-C-N	-5.29	114.23	122.70
1	A	312	ALA	CA-C-O	-5.29	108.99	120.10
2	B	132	SER	CA-C-O	5.29	131.22	120.10
3	M	21	GLY	CA-C-N	-5.29	105.56	117.20
1	A	85	ALA	O-C-N	5.29	131.17	122.70
2	B	595	VAL	CA-C-O	-5.29	108.99	120.10
3	M	284	SER	CA-C-O	5.29	131.21	120.10
1	A	470	GLY	O-C-N	5.29	131.16	122.70
1	A	110	ALA	CA-C-O	5.29	131.20	120.10
2	B	196	LEU	CA-C-O	-5.29	109.00	120.10
1	A	105	VAL	CA-C-O	-5.28	109.02	120.10
2	B	346	THR	CA-C-O	-5.28	109.02	120.10
2	B	467	VAL	CA-C-O	-5.28	109.02	120.10
1	A	237	SER	O-C-N	5.27	131.12	121.10
1	A	180	LYS	O-C-N	5.27	131.13	122.70
2	B	273	SER	C-N-CA	5.27	144.14	122.00
1	A	434	SER	CA-C-O	-5.27	109.04	120.10
2	B	554	LYS	CA-C-O	-5.27	109.04	120.10
1	A	125	THR	CA-C-O	-5.26	109.04	120.10
2	B	616	SER	O-C-N	5.26	131.12	122.70
1	A	548	GLN	O-C-N	5.26	131.12	122.70
3	M	24	ALA	C-N-CD	5.26	139.44	128.40
1	A	289	SER	C-N-CA	-5.26	108.56	121.70
1	A	172	SER	C-N-CA	-5.25	108.56	121.70
2	B	589	SER	CA-C-O	-5.25	109.06	120.10
3	M	379	LEU	CA-C-O	5.25	131.13	120.10
1	A	560	SER	O-C-N	5.25	131.10	122.70
2	B	222	HIS	N-CA-C	5.25	125.18	111.00
2	B	274	PRO	CA-N-CD	-5.25	104.15	111.50
2	B	451	MET	CA-C-O	-5.25	109.08	120.10
1	A	496	ILE	CA-C-O	-5.25	109.09	120.10
1	A	146	ALA	CA-C-O	-5.24	109.09	120.10
2	B	54	ARG	N-CA-C	5.24	125.16	111.00
2	B	169	VAL	CA-C-O	-5.24	109.09	120.10
1	A	370	LYS	CA-C-O	-5.24	109.10	120.10
2	B	192	LEU	CA-C-O	-5.24	109.10	120.10
3	M	309	GLN	O-C-N	5.24	131.08	122.70
1	A	163	ALA	CA-C-O	5.24	131.10	120.10

Continued on next page...

Continued from previous page...

Mol	Chain	Res	Type	Atoms	Z	Observed(°)	Ideal(°)
1	A	437	SER	N-CA-C	5.24	125.14	111.00
1	A	493	ALA	CA-C-O	-5.24	109.10	120.10
2	B	280	PRO	N-CA-C	-5.24	98.49	112.10
1	A	278	ILE	O-C-N	-5.23	114.33	122.70
1	A	327	ASP	O-C-N	5.23	131.04	121.10
2	B	489	ILE	CA-C-O	-5.23	109.11	120.10
2	B	443	SER	CA-C-N	-5.23	105.70	117.20
4	S	103	GLN	CA-C-O	-5.23	109.12	120.10
2	B	188	TYR	O-C-N	5.23	131.06	122.70
1	A	572	PHE	O-C-N	5.22	131.06	122.70
1	A	242	GLU	CA-C-O	-5.22	109.13	120.10
2	B	166	SER	C-N-CA	5.22	134.76	121.70
3	M	61	GLU	N-CA-CB	5.22	120.00	110.60
1	A	634	ASN	CA-C-O	-5.22	109.14	120.10
2	B	503	LEU	CA-C-N	-5.22	105.71	117.20
2	B	336	ASN	O-C-N	5.22	131.05	122.70
4	S	95	GLU	CA-C-O	-5.22	109.15	120.10
4	S	78	LYS	O-C-N	-5.21	114.36	122.70
1	A	526	VAL	C-N-CA	5.21	134.72	121.70
2	B	223	LEU	CA-C-O	5.21	131.04	120.10
2	B	292	GLU	CA-C-N	-5.21	105.74	117.20
3	M	60	LEU	CA-C-N	-5.21	105.75	117.20
4	S	141	VAL	N-CA-C	-5.21	96.94	111.00
1	A	439	ASP	C-N-CA	5.21	134.71	121.70
3	M	354	ASP	CB-CA-C	5.21	120.81	110.40
2	B	319	LEU	CA-C-O	-5.20	109.17	120.10
2	B	588	ILE	CA-C-O	-5.20	109.19	120.10
2	B	470	ALA	O-C-N	5.19	131.01	122.70
3	M	122	SER	CA-C-O	-5.19	109.20	120.10
3	M	90	PHE	CA-C-O	-5.19	109.20	120.10
1	A	134	TYR	C-N-CA	-5.19	108.73	121.70
1	A	562	TRP	CA-C-O	-5.19	109.20	120.10
1	A	341	ILE	CA-C-O	-5.18	109.22	120.10
1	A	239	LEU	C-N-CA	-5.18	108.75	121.70
1	A	71	VAL	O-C-N	5.18	130.99	122.70
1	A	73	LYS	O-C-N	5.18	130.99	122.70
2	B	577	ASN	N-CA-CB	-5.18	101.28	110.60
2	B	304	GLN	O-C-N	5.18	130.98	122.70
2	B	615	SER	CA-C-O	-5.18	109.23	120.10
2	B	340	ILE	CA-C-O	-5.17	109.23	120.10
2	B	551	LEU	CA-C-O	-5.17	109.23	120.10
2	B	118	LEU	O-C-N	5.17	130.98	122.70

Continued on next page...

Continued from previous page...

Mol	Chain	Res	Type	Atoms	Z	Observed(°)	Ideal(°)
2	B	494	ALA	CA-C-O	-5.17	109.24	120.10
1	A	474	GLY	CA-C-O	-5.17	111.29	120.60
2	B	63	MET	CA-C-O	-5.17	109.24	120.10
1	A	94	VAL	CA-C-O	5.17	130.96	120.10
3	M	106	LYS	C-N-CA	-5.17	108.78	121.70
3	M	331	LEU	CA-C-N	-5.17	105.87	116.20
1	A	533	ILE	O-C-N	5.16	130.96	122.70
2	B	566	ALA	N-CA-CB	-5.16	102.87	110.10
2	B	592	TYR	CA-C-O	-5.16	109.25	120.10
1	A	370	LYS	O-C-N	5.16	130.96	122.70
1	A	597	GLN	O-C-N	5.16	130.96	122.70
3	M	117	ASN	CA-C-O	-5.16	109.26	120.10
3	M	323	MET	C-N-CA	-5.16	108.80	121.70
1	A	77	LEU	O-C-N	5.16	130.95	122.70
1	A	69	ASN	O-C-N	5.16	130.95	122.70
1	A	174	ARG	O-C-N	5.16	130.90	121.10
1	A	330	LEU	CA-C-O	-5.16	109.28	120.10
2	B	608	ARG	O-C-N	5.16	130.95	122.70
2	B	401	THR	C-N-CA	5.15	134.59	121.70
1	A	193	PRO	O-C-N	5.15	130.94	122.70
1	A	380	ASP	CA-C-O	-5.15	109.28	120.10
1	A	253	ILE	CA-C-O	-5.15	109.29	120.10
2	B	175	LEU	O-C-N	5.15	130.94	122.70
2	B	399	LEU	O-C-N	-5.15	114.46	122.70
2	B	411	ILE	CA-C-O	-5.15	109.29	120.10
3	M	447	ILE	O-C-N	-5.15	114.46	122.70
2	B	350	THR	N-CA-C	5.14	124.87	111.00
4	S	154	ASP	CA-C-O	-5.14	109.31	120.10
2	B	500	GLN	N-CA-C	5.14	124.87	111.00
1	A	217	ALA	CB-CA-C	5.13	117.80	110.10
1	A	221	VAL	CA-C-O	-5.13	109.33	120.10
2	B	112	ASP	CA-C-O	-5.13	109.33	120.10
1	A	555	LEU	CA-C-O	-5.12	109.34	120.10
2	B	421	SER	N-CA-C	5.12	124.83	111.00
2	B	562	ASN	O-C-N	5.12	130.89	122.70
2	B	84	ALA	O-C-N	5.12	130.89	122.70
3	M	104	PHE	CA-C-O	5.12	130.85	120.10
1	A	103	LYS	C-N-CA	-5.11	108.91	121.70
1	A	178	ARG	O-C-N	5.11	130.88	122.70
4	S	164	ASP	CA-C-N	5.11	128.45	117.20
2	B	86	VAL	CA-C-O	-5.11	109.37	120.10
2	B	365	PHE	CA-C-O	-5.11	109.37	120.10

Continued on next page...

Continued from previous page...

Mol	Chain	Res	Type	Atoms	Z	Observed(°)	Ideal(°)
2	B	391	ALA	CA-C-O	-5.11	109.37	120.10
2	B	530	LEU	CA-C-O	-5.11	109.37	120.10
4	S	54	PRO	CA-C-N	5.11	131.41	117.10
1	A	141	VAL	CA-C-O	-5.11	109.37	120.10
4	S	89	VAL	C-N-CA	-5.10	108.95	121.70
1	A	438	ALA	N-CA-C	5.10	124.76	111.00
1	A	174	ARG	C-N-CD	-5.09	109.39	120.60
2	B	116	THR	CA-C-O	-5.09	109.41	120.10
4	S	46	PHE	CA-C-N	5.09	128.41	117.20
1	A	323	CYS	O-C-N	-5.09	114.56	122.70
1	A	512	LEU	CA-C-O	-5.09	109.42	120.10
2	B	134	LEU	CA-C-O	-5.09	109.42	120.10
2	B	230	PHE	CA-C-O	5.08	130.78	120.10
1	A	148	SER	O-C-N	5.08	131.84	123.20
1	A	602	GLU	CA-C-O	-5.08	109.43	120.10
1	A	179	LYS	O-C-N	5.08	130.82	122.70
2	B	465	ALA	O-C-N	5.08	130.82	122.70
1	A	280	GLU	O-C-N	5.07	130.81	122.70
2	B	590	GLN	CA-C-O	-5.07	109.45	120.10
1	A	252	ILE	CA-C-O	-5.07	109.46	120.10
2	B	492	LYS	CA-C-O	-5.07	109.45	120.10
4	S	60	SER	C-N-CA	5.07	134.37	121.70
1	A	69	ASN	CA-C-O	-5.06	109.47	120.10
1	A	121	LEU	CA-C-O	-5.06	109.47	120.10
1	A	338	PHE	CA-C-O	-5.06	109.47	120.10
1	A	422	GLU	O-C-N	5.06	130.80	122.70
3	M	77	LEU	O-C-N	-5.06	114.60	122.70
3	M	266	ASP	C-N-CA	5.06	134.35	121.70
2	B	398	ILE	O-C-N	5.06	130.79	122.70
1	A	628	VAL	CA-C-O	-5.06	109.48	120.10
2	B	83	PHE	CA-C-N	-5.05	106.08	117.20
4	S	132	LEU	CA-C-O	-5.05	109.48	120.10
1	A	230	PRO	O-C-N	5.05	130.78	122.70
2	B	119	SER	CA-C-O	-5.05	109.49	120.10
3	M	39	PRO	O-C-N	5.05	130.78	122.70
1	A	302	ASN	N-CA-CB	5.05	119.69	110.60
1	A	502	ASP	O-C-N	5.05	130.78	122.70
3	M	405	THR	CA-C-O	5.05	130.70	120.10
1	A	85	ALA	CA-C-N	-5.05	106.10	117.20
3	M	280	ASP	N-CA-CB	-5.05	101.52	110.60
3	M	234	ARG	O-C-N	-5.04	114.63	122.70
1	A	383	ASN	CA-C-O	-5.04	109.51	120.10

Continued on next page...

Continued from previous page...

Mol	Chain	Res	Type	Atoms	Z	Observed(°)	Ideal(°)
1	A	599	ARG	O-C-N	5.04	130.76	122.70
1	A	375	VAL	CA-C-O	5.04	130.68	120.10
2	B	482	ASN	CA-C-O	-5.04	109.52	120.10
2	B	548	ILE	CA-C-O	-5.04	109.53	120.10
1	A	199	ASN	N-CA-C	5.03	124.59	111.00
1	A	594	PHE	CA-C-O	-5.03	109.53	120.10
2	B	255	TYR	CA-C-O	-5.03	109.53	120.10
2	B	55	ASN	C-N-CA	5.03	134.28	121.70
4	S	68	VAL	N-CA-CB	-5.03	100.43	111.50
1	A	305	GLU	CA-C-O	-5.03	109.54	120.10
3	M	278	ILE	C-N-CA	-5.03	109.13	121.70
1	A	445	ASN	N-CA-C	-5.03	97.43	111.00
1	A	113	SER	N-CA-C	5.02	124.56	111.00
4	S	12	CYS	C-N-CA	-5.02	109.14	121.70
1	A	620	GLY	O-C-N	5.02	130.74	122.70
1	A	304	LEU	CA-C-N	5.02	128.24	117.20
1	A	254	ILE	CA-C-O	-5.02	109.56	120.10
2	B	210	CYS	CA-C-O	-5.02	109.56	120.10
1	A	337	LEU	CA-C-O	-5.01	109.57	120.10
4	S	42	ARG	C-N-CA	5.01	134.23	121.70
2	B	350	THR	C-N-CA	5.01	134.23	121.70
3	M	18	TYR	O-C-N	-5.01	114.68	122.70
1	A	239	LEU	O-C-N	5.01	130.72	122.70
1	A	199	ASN	O-C-N	5.01	130.71	122.70
4	S	157	ASN	CA-C-O	-5.01	109.58	120.10
2	B	60	ARG	CA-C-O	-5.00	109.59	120.10
1	A	218	ALA	CA-C-O	-5.00	109.59	120.10
2	B	253	ILE	CA-C-O	-5.00	109.59	120.10
2	B	439	CYS	O-C-N	5.00	131.70	123.20

All (2) chirality outliers are listed below:

Mol	Chain	Res	Type	Atom
3	M	22	ALA	CA
4	S	69	ASN	CA

All (125) planarity outliers are listed below:

Mol	Chain	Res	Type	Group
1	A	117	ASP	Mainchain
1	A	174	ARG	Mainchain
1	A	192	TYR	Mainchain

Continued on next page...

Continued from previous page...

Mol	Chain	Res	Type	Group
1	A	199	ASN	Mainchain
1	A	204	VAL	Mainchain
1	A	219	VAL	Mainchain
1	A	233	PHE	Mainchain
1	A	240	LEU	Mainchain
1	A	244	LEU	Mainchain
1	A	260	PHE	Mainchain
1	A	275	LEU	Mainchain
1	A	277	LYS	Mainchain
1	A	278	ILE	Mainchain
1	A	282	MET	Mainchain
1	A	288	THR	Mainchain
1	A	289	SER	Mainchain
1	A	290	VAL	Mainchain
1	A	298	ILE	Mainchain
1	A	302	ASN	Mainchain
1	A	306	GLU	Mainchain
1	A	319	LEU	Mainchain
1	A	320	HIS	Mainchain
1	A	323	CYS	Mainchain
1	A	325	SER	Mainchain
1	A	328	PRO	Mainchain
1	A	350	SER	Mainchain
1	A	399	ASP	Peptide
1	A	400	VAL	Peptide
1	A	441	TYR	Mainchain
1	A	462	GLN	Mainchain
1	A	487	MET	Mainchain
1	A	500	SER	Mainchain
1	A	501	ASN	Mainchain
1	A	505	ASN	Mainchain
1	A	506	LYS	Mainchain
1	A	527	GLU	Mainchain
1	A	528	ASN	Mainchain
1	A	529	GLY	Mainchain
1	A	531	ASP	Mainchain
1	A	535	ILE	Mainchain
1	A	536	MET	Mainchain
1	A	538	GLU	Mainchain
1	A	539	ASN	Mainchain
1	A	565	ASN	Mainchain
1	A	569	ASP	Mainchain

Continued on next page...

Continued from previous page...

Mol	Chain	Res	Type	Group
1	A	571	ARG	Mainchain
1	A	586	GLU	Mainchain
1	A	588	LEU	Mainchain
1	A	64	LEU	Mainchain
1	A	80	TYR	Mainchain
1	A	84	MET	Mainchain
1	A	94	VAL	Mainchain
2	B	126	SER	Mainchain
2	B	142	LEU	Mainchain
2	B	147	MET	Mainchain
2	B	181	TYR	Mainchain
2	B	186	ASN	Mainchain
2	B	237	ILE	Mainchain
2	B	267	ASP	Mainchain
2	B	278	PRO	Peptide
2	B	288	TYR	Mainchain
2	B	293	VAL	Mainchain
2	B	310	ILE	Mainchain
2	B	326	TYR	Mainchain
2	B	375	LEU	Mainchain
2	B	377	TYR	Mainchain
2	B	381	PHE	Mainchain
2	B	384	PHE	Mainchain
2	B	404	ASN	Mainchain
2	B	41	ASN	Peptide
2	B	444	THR	Mainchain
2	B	459	GLU	Mainchain
2	B	485	LYS	Mainchain
2	B	497	LEU	Mainchain
2	B	523	PHE	Mainchain
2	B	536	ASN	Mainchain
2	B	557	SER	Mainchain
2	B	56	SER	Mainchain
2	B	569	THR	Mainchain
2	B	573	GLU	Mainchain
2	B	581	TYR	Mainchain
2	B	584	SER	Mainchain
2	B	78	ASP	Mainchain
2	B	82	TYR	Mainchain
3	M	102	GLU	Mainchain
3	M	103	TYR	Mainchain
3	M	104	PHE	Mainchain

Continued on next page...

Continued from previous page...

Mol	Chain	Res	Type	Group
3	M	105	ASP	Peptide
3	M	131	ALA	Mainchain
3	M	134	PRO	Mainchain
3	M	265	ASN	Mainchain
3	M	284	SER	Mainchain
3	M	292	PRO	Peptide
3	M	40	GLN	Mainchain
3	M	405	THR	Mainchain
3	M	421	GLY	Mainchain
3	M	426	LYS	Peptide
3	M	445	SER	Peptide
3	M	45	SER	Peptide
3	M	456	SER	Mainchain
3	M	462	LYS	Peptide
3	M	477	GLY	Peptide
3	M	51	LEU	Peptide
3	M	57	GLY	Mainchain
3	M	79	SER	Mainchain
3	M	8	THR	Mainchain
3	M	85	GLY	Mainchain
4	S	101	LEU	Mainchain
4	S	102	ILE	Mainchain
4	S	135	ILE	Mainchain
4	S	161	GLU	Mainchain
4	S	163	THR	Peptide
4	S	167	ILE	Peptide
4	S	22	PRO	Mainchain
4	S	43	ASN	Mainchain
4	S	46	PHE	Mainchain
4	S	50	PHE	Mainchain
4	S	53	THR	Mainchain
4	S	56	SER	Mainchain
4	S	57	LEU	Mainchain
4	S	58	LEU	Peptide
4	S	64	ASN	Mainchain
4	S	66	ASP	Mainchain
4	S	69	ASN	Mainchain
4	S	71	GLU	Mainchain

5.2 Too-close contacts (i)

In the following table, the Non-H and H(model) columns list the number of non-hydrogen atoms and hydrogen atoms in the chain respectively. The H(added) column lists the number of hydrogen atoms added and optimized by MolProbity. The Clashes column lists the number of clashes within the asymmetric unit, whereas Symm-Clashes lists symmetry-related clashes.

Mol	Chain	Non-H	H(model)	H(added)	Clashes	Symm-Clashes
1	A	4625	0	4702	1245	0
2	B	4961	0	4981	2457	0
3	M	3158	0	3106	986	0
4	S	1358	0	1334	356	0
All	All	14102	0	14123	4637	0

The all-atom clashscore is defined as the number of clashes found per 1000 atoms (including hydrogen atoms). The all-atom clashscore for this structure is 164.

All (4637) close contacts within the same asymmetric unit are listed below, sorted by their clash magnitude.

Atom-1	Atom-2	Interatomic distance (Å)	Clash overlap (Å)
2:B:316:THR:HG23	3:M:90:PHE:CE2	1.28	1.66
2:B:162:VAL:HG21	2:B:195:ILE:CG2	1.27	1.65
1:A:638:LEU:HB2	2:B:558:TYR:CD1	1.25	1.63
2:B:193:LEU:HD22	2:B:225:LEU:CB	1.19	1.62
2:B:193:LEU:CG	2:B:225:LEU:HG	1.30	1.62
1:A:638:LEU:CD2	2:B:558:TYR:HB3	1.26	1.61
3:M:131:ALA:CA	3:M:131:ALA:CB	1.79	1.60
2:B:523:PHE:CZ	2:B:580:TYR:CD1	1.86	1.60
2:B:479:VAL:CG1	2:B:486:HIS:CE1	1.79	1.60
2:B:24:ALA:HB3	2:B:35:TYR:CE2	1.33	1.59
1:A:638:LEU:HG	2:B:520:SER:CB	1.32	1.58
2:B:127:LEU:HD13	2:B:157:THR:CG2	1.31	1.58
2:B:139:LEU:CD2	2:B:173:VAL:HA	1.35	1.56
2:B:252:LEU:HD13	2:B:302:PHE:CD1	1.40	1.56
1:A:630:PRO:CG	2:B:617:LEU:CD1	1.84	1.56
2:B:24:ALA:HB3	2:B:35:TYR:CZ	1.40	1.55
2:B:523:PHE:HZ	2:B:580:TYR:CD1	1.12	1.55
1:A:630:PRO:CG	2:B:617:LEU:HD13	1.08	1.55
2:B:193:LEU:HD13	2:B:225:LEU:CG	1.24	1.54
2:B:193:LEU:CD1	2:B:225:LEU:HG	1.23	1.53
4:S:16:LEU:CD1	4:S:125:TRP:HE1	1.16	1.53
2:B:70:MET:CE	2:B:107:ARG:HB2	1.33	1.52
3:M:282:VAL:N	3:M:282:VAL:CA	1.68	1.52

Continued on next page...

Continued from previous page...

Atom-1	Atom-2	Interatomic distance (Å)	Clash overlap (Å)
2:B:219:TYR:CE1	2:B:226:LEU:HD13	1.46	1.50
2:B:102:HIS:CE1	2:B:138:ALA:N	1.78	1.50
2:B:216:LYS:CB	2:B:251:LEU:HD13	1.38	1.49
4:S:64:ASN:CA	4:S:64:ASN:C	1.80	1.49
1:A:404:GLN:HA	2:B:3:ASP:CG	1.30	1.49
2:B:158:VAL:HG11	2:B:177:ILE:CG1	1.40	1.49
1:A:638:LEU:CD1	2:B:558:TYR:CD2	1.94	1.48
2:B:127:LEU:CD1	2:B:157:THR:HG21	1.41	1.48
1:A:638:LEU:HD22	2:B:558:TYR:CB	1.40	1.48
3:M:281:GLY:CA	3:M:281:GLY:C	1.78	1.48
2:B:25:VAL:HG21	2:B:36:THR:CA	1.43	1.47
2:B:316:THR:CG2	3:M:90:PHE:CE2	1.92	1.47
1:A:200:PHE:CZ	1:A:236:LEU:HD21	1.49	1.46
1:A:630:PRO:HG3	2:B:617:LEU:CD1	1.37	1.46
1:A:638:LEU:CB	2:B:558:TYR:CG	1.78	1.45
2:B:25:VAL:CG2	2:B:36:THR:N	1.79	1.44
1:A:595:GLU:CD	2:B:476:ARG:NH1	1.71	1.44
2:B:549:LEU:HD21	2:B:611:ALA:N	1.12	1.44
2:B:193:LEU:HD13	2:B:225:LEU:CD2	1.47	1.44
2:B:208:ILE:HD13	2:B:236:ILE:CG2	1.47	1.44
4:S:163:THR:CA	4:S:163:THR:C	1.81	1.44
2:B:546:CYS:CB	2:B:607:ILE:HG12	1.44	1.43
1:A:65:ASN:HA	4:S:166:LYS:CA	1.39	1.43
1:A:179:LYS:HB3	4:S:142:ILE:CD1	1.34	1.43
1:A:638:LEU:CG	2:B:520:SER:HB3	1.48	1.43
1:A:403:LEU:O	2:B:3:ASP:CB	1.65	1.43
2:B:534:ILE:HG21	2:B:594:ALA:CB	1.44	1.43
2:B:102:HIS:CE1	2:B:138:ALA:HA	1.54	1.42
2:B:102:HIS:NE2	2:B:138:ALA:HA	1.25	1.42
2:B:513:TRP:N	2:B:551:LEU:HD13	1.33	1.42
2:B:25:VAL:HG22	2:B:36:THR:N	1.14	1.42
2:B:215:TYR:CD1	2:B:233:TYR:HE1	1.37	1.42
2:B:486:HIS:ND1	2:B:518:ILE:HD13	1.34	1.42
2:B:29:LYS:HE2	2:B:30:LEU:N	1.26	1.41
2:B:102:HIS:CE1	2:B:138:ALA:CA	2.00	1.41
2:B:226:LEU:HD23	2:B:255:TYR:CD1	1.52	1.41
2:B:316:THR:CG2	3:M:90:PHE:HE2	1.29	1.41
2:B:178:ILE:HG13	2:B:214:ALA:C	1.41	1.41
2:B:316:THR:HG23	3:M:90:PHE:CD2	1.55	1.40
2:B:70:MET:HE1	2:B:107:ARG:CB	1.45	1.40
2:B:479:VAL:HG13	2:B:486:HIS:ND1	1.31	1.40

Continued on next page...

Continued from previous page...

Atom-1	Atom-2	Interatomic distance (Å)	Clash overlap (Å)
4:S:16:LEU:HD13	4:S:125:TRP:NE1	1.10	1.40
2:B:24:ALA:CB	2:B:35:TYR:CE1	2.04	1.40
1:A:638:LEU:HD13	2:B:558:TYR:CB	1.50	1.39
2:B:70:MET:CE	2:B:107:ARG:CB	1.98	1.39
2:B:24:ALA:CB	2:B:35:TYR:CZ	2.05	1.39
2:B:155:LEU:HD23	2:B:192:LEU:CD1	1.51	1.39
1:A:404:GLN:C	2:B:3:ASP:HB3	1.37	1.39
2:B:243:TRP:CZ3	3:M:94:GLU:HB3	1.57	1.38
2:B:193:LEU:CD2	2:B:225:LEU:CB	2.01	1.38
2:B:278:PRO:HD2	2:B:292:GLU:CB	1.50	1.38
3:M:41:LEU:CD1	3:M:52:ASP:H	1.37	1.38
1:A:638:LEU:HD22	2:B:558:TYR:CA	1.54	1.35
2:B:29:LYS:CE	2:B:30:LEU:H	1.35	1.35
2:B:513:TRP:HA	2:B:551:LEU:CD2	1.55	1.35
1:A:404:GLN:O	2:B:3:ASP:HB3	1.17	1.34
1:A:638:LEU:CG	2:B:558:TYR:HB3	1.57	1.34
2:B:24:ALA:HB3	2:B:35:TYR:CD2	1.62	1.34
2:B:479:VAL:HG11	2:B:486:HIS:CE1	1.47	1.33
2:B:181:TYR:O	2:B:183:ALA:N	1.61	1.33
2:B:219:TYR:CE1	2:B:226:LEU:CD1	2.11	1.33
2:B:21:GLU:OE1	2:B:39:SER:CB	1.75	1.32
2:B:513:TRP:N	2:B:551:LEU:CD1	1.90	1.32
1:A:111:SER:HB2	1:A:152:THR:OG1	1.29	1.32
2:B:193:LEU:CD1	2:B:225:LEU:CG	1.90	1.32
2:B:243:TRP:CH2	3:M:94:GLU:HG2	1.63	1.32
2:B:178:ILE:HG13	2:B:214:ALA:CA	1.58	1.31
2:B:83:PHE:CZ	2:B:119:SER:HB3	1.64	1.31
1:A:599:ARG:NH1	2:B:513:TRP:HZ3	1.24	1.31
2:B:178:ILE:CG2	2:B:217:GLU:HB2	1.59	1.31
1:A:257:LEU:HD22	1:A:278:ILE:CG2	1.60	1.31
2:B:162:VAL:CG2	2:B:195:ILE:CG2	2.09	1.31
1:A:404:GLN:HA	2:B:3:ASP:CB	1.60	1.30
1:A:638:LEU:HB2	2:B:558:TYR:CG	1.14	1.30
1:A:258:LYS:NZ	4:S:94:SER:HB3	1.47	1.30
1:A:125:THR:OG1	1:A:158:LEU:HD13	1.13	1.30
2:B:230:PHE:CE2	2:B:298:ASP:O	1.84	1.30
2:B:230:PHE:CZ	2:B:252:LEU:HD22	1.66	1.30
2:B:252:LEU:HB2	2:B:302:PHE:CZ	1.66	1.29
2:B:556:LEU:CA	2:B:588:ILE:HD11	1.61	1.29
3:M:221:THR:N	3:M:474:THR:OG1	1.64	1.29
2:B:193:LEU:HD22	2:B:225:LEU:CG	1.60	1.29

Continued on next page...

Continued from previous page...

Atom-1	Atom-2	Interatomic distance (Å)	Clash overlap (Å)
2:B:534:ILE:CD1	2:B:591:MET:O	1.80	1.29
2:B:223:LEU:CB	2:B:259:TYR:CD1	1.78	1.29
1:A:179:LYS:CB	4:S:142:ILE:CD1	2.03	1.28
2:B:223:LEU:HD13	2:B:259:TYR:N	1.48	1.28
2:B:247:TYR:OH	3:M:137:SER:HB3	1.33	1.28
2:B:546:CYS:HB2	2:B:607:ILE:CG1	1.64	1.28
2:B:243:TRP:HE1	3:M:98:ARG:CD	1.45	1.28
2:B:584:SER:O	2:B:588:ILE:HG22	1.30	1.28
2:B:278:PRO:CD	2:B:292:GLU:HB2	1.62	1.27
1:A:369:SER:HB2	1:A:424:TYR:CE2	1.68	1.27
1:A:404:GLN:HA	2:B:3:ASP:OD2	1.19	1.27
2:B:193:LEU:CD2	2:B:225:LEU:HG	1.63	1.27
1:A:186:PHE:CE1	1:A:224:GLU:CB	2.17	1.27
2:B:243:TRP:HH2	3:M:94:GLU:C	1.35	1.27
2:B:102:HIS:HE1	2:B:138:ALA:N	1.17	1.27
2:B:479:VAL:HG11	2:B:486:HIS:NE2	1.49	1.27
2:B:184:GLY:O	2:B:188:TYR:HD1	1.14	1.26
2:B:223:LEU:HD11	2:B:258:GLN:CB	1.63	1.26
2:B:316:THR:CB	3:M:90:PHE:HE2	1.46	1.26
1:A:638:LEU:CD1	2:B:558:TYR:CB	2.12	1.26
2:B:279:LEU:N	2:B:288:TYR:HB2	1.47	1.26
1:A:88:ASN:ND2	1:A:120:ILE:HG21	1.48	1.26
2:B:231:ARG:HG3	2:B:298:ASP:OD1	1.35	1.26
1:A:65:ASN:HA	4:S:166:LYS:CB	1.49	1.25
2:B:98:LYS:NZ	2:B:134:LEU:HB3	1.50	1.25
2:B:162:VAL:CG2	2:B:195:ILE:HG23	1.65	1.25
3:M:432:THR:OG1	3:M:480:GLN:HG3	1.33	1.25
2:B:20:ARG:HD2	2:B:35:TYR:OH	1.35	1.25
2:B:239:GLN:OE1	3:M:279:ASN:HA	1.26	1.25
2:B:353:GLN:CB	3:M:50:TYR:HD1	1.46	1.25
2:B:230:PHE:HE2	2:B:298:ASP:O	1.04	1.25
2:B:136:CYS:SG	2:B:169:VAL:HA	1.77	1.25
2:B:167:ALA:HA	2:B:202:ASP:OD1	1.33	1.25
2:B:230:PHE:CE1	2:B:252:LEU:HD23	1.71	1.25
2:B:472:VAL:CG1	2:B:510:GLY:HA3	1.65	1.25
2:B:215:TYR:CD1	2:B:233:TYR:CE1	2.24	1.24
1:A:65:ASN:CA	4:S:166:LYS:HA	1.46	1.24
1:A:84:MET:SD	1:A:113:SER:HA	1.78	1.24
2:B:216:LYS:HA	2:B:251:LEU:CD1	1.64	1.24
2:B:219:TYR:CZ	2:B:226:LEU:CA	2.19	1.24
2:B:422:ALA:HB3	2:B:424:PHE:CD1	1.71	1.24

Continued on next page...

Continued from previous page...

Atom-1	Atom-2	Interatomic distance (Å)	Clash overlap (Å)
2:B:241:ASP:CB	3:M:274:ASP:OD1	1.84	1.24
3:M:16:PHE:HA	3:M:118:TYR:CE1	1.73	1.24
2:B:24:ALA:HB1	2:B:35:TYR:CD1	1.71	1.24
2:B:219:TYR:CE1	2:B:226:LEU:HB2	1.73	1.24
2:B:231:ARG:CG	2:B:298:ASP:OD1	1.85	1.24
1:A:200:PHE:CZ	1:A:236:LEU:CD2	2.20	1.23
1:A:289:SER:CB	4:S:96:LEU:HD13	1.66	1.23
2:B:345:ARG:NH1	3:M:305:ASP:OD2	1.68	1.23
1:A:404:GLN:CA	2:B:3:ASP:HB3	1.67	1.23
2:B:21:GLU:OE2	2:B:35:TYR:CG	1.91	1.23
2:B:422:ALA:HB3	2:B:424:PHE:CE1	1.71	1.23
2:B:523:PHE:CZ	2:B:580:TYR:CG	2.25	1.23
2:B:519:ALA:O	2:B:523:PHE:HB3	1.30	1.23
3:M:222:PHE:O	3:M:479:PHE:CE2	1.92	1.23
2:B:252:LEU:CD1	2:B:302:PHE:CD1	2.21	1.23
2:B:278:PRO:HA	2:B:288:TYR:C	1.59	1.23
2:B:278:PRO:HA	2:B:288:TYR:CB	1.68	1.23
2:B:21:GLU:OE2	2:B:35:TYR:CD1	1.92	1.23
2:B:24:ALA:CB	2:B:35:TYR:CD1	2.22	1.23
2:B:223:LEU:HB3	2:B:259:TYR:CD1	1.06	1.23
1:A:638:LEU:HD13	2:B:558:TYR:CG	1.73	1.22
2:B:25:VAL:HG11	2:B:36:THR:OG1	1.39	1.22
2:B:512:VAL:C	2:B:551:LEU:HD13	1.56	1.22
1:A:638:LEU:CG	2:B:520:SER:CB	2.07	1.22
2:B:245:GLN:CD	2:B:309:LEU:CD1	2.08	1.22
2:B:290:SER:O	2:B:292:GLU:N	1.71	1.22
1:A:563:CYS:HB3	1:A:621:LEU:CD1	1.70	1.22
2:B:352:ASN:ND2	3:M:70:ASN:HB3	1.54	1.22
2:B:479:VAL:HG13	2:B:486:HIS:CE1	1.55	1.22
2:B:546:CYS:CA	2:B:607:ILE:HG12	1.69	1.22
2:B:280:PRO:HG2	2:B:283:TYR:CD1	1.74	1.21
2:B:337:THR:CA	2:B:373:LEU:HD21	1.69	1.21
2:B:219:TYR:CD1	2:B:226:LEU:HD13	1.75	1.21
2:B:353:GLN:HB3	3:M:50:TYR:CD1	1.75	1.21
2:B:486:HIS:CE1	2:B:518:ILE:HD13	1.75	1.21
1:A:88:ASN:CG	1:A:120:ILE:HG21	1.61	1.21
1:A:275:LEU:O	1:A:276:PRO:C	1.73	1.21
1:A:638:LEU:CD1	2:B:558:TYR:HD2	1.39	1.21
2:B:158:VAL:CG1	2:B:177:ILE:CG1	2.19	1.21
3:M:244:VAL:HA	3:M:472:TYR:CD2	1.75	1.21
2:B:193:LEU:CD1	2:B:225:LEU:CD2	2.12	1.20

Continued on next page...

Continued from previous page...

Atom-1	Atom-2	Interatomic distance (Å)	Clash overlap (Å)
2:B:243:TRP:NE1	3:M:98:ARG:HD2	1.54	1.20
2:B:513:TRP:CA	2:B:551:LEU:CD1	2.18	1.20
1:A:404:GLN:CA	2:B:3:ASP:OD2	1.90	1.20
2:B:42:ILE:O	2:B:46:GLN:OE1	1.60	1.20
2:B:245:GLN:CD	2:B:309:LEU:HD11	1.61	1.20
1:A:275:LEU:HD21	1:A:311:THR:OG1	1.39	1.20
3:M:344:ILE:CG2	3:M:347:PHE:HB3	1.73	1.20
2:B:193:LEU:CD2	2:B:225:LEU:CG	2.15	1.19
2:B:293:VAL:O	2:B:299:LEU:CG	1.89	1.19
2:B:374:PHE:CE2	2:B:402:LEU:HD11	1.76	1.19
2:B:567:GLN:O	2:B:569:THR:N	1.75	1.19
1:A:186:PHE:CE1	1:A:224:GLU:HB2	1.72	1.19
2:B:16:LYS:HD2	3:M:118:TYR:CE2	1.76	1.19
2:B:277:CYS:O	2:B:288:TYR:CB	1.88	1.19
1:A:595:GLU:OE2	2:B:476:ARG:NH1	1.76	1.19
2:B:181:TYR:O	2:B:182:ARG:C	1.75	1.19
2:B:219:TYR:CZ	2:B:226:LEU:HB2	1.76	1.19
2:B:309:LEU:HB3	2:B:317:VAL:CG1	1.71	1.19
2:B:556:LEU:HA	2:B:588:ILE:CD1	1.72	1.19
2:B:243:TRP:CH2	3:M:94:GLU:CG	2.24	1.18
2:B:567:GLN:O	2:B:569:THR:OG1	1.61	1.18
2:B:140:SER:HB2	2:B:172:GLU:OE1	1.42	1.18
2:B:155:LEU:CD2	2:B:192:LEU:CD1	2.19	1.18
1:A:638:LEU:CG	2:B:558:TYR:CG	2.26	1.18
2:B:16:LYS:HD3	3:M:119:ASP:OD1	1.37	1.18
2:B:219:TYR:CD1	2:B:226:LEU:HB2	1.77	1.18
2:B:549:LEU:HD21	2:B:611:ALA:CA	1.74	1.18
2:B:83:PHE:CE2	2:B:119:SER:HB3	1.78	1.18
2:B:226:LEU:CD2	2:B:255:TYR:CD1	2.25	1.18
2:B:567:GLN:O	2:B:569:THR:CB	1.92	1.18
1:A:408:ILE:HG22	4:S:64:ASN:C	1.56	1.18
1:A:464:ILE:HA	2:B:1:MET:SD	1.84	1.18
2:B:219:TYR:CE1	2:B:226:LEU:CB	2.26	1.18
2:B:243:TRP:CH2	3:M:94:GLU:C	2.17	1.18
2:B:337:THR:HA	2:B:373:LEU:HD21	1.25	1.18
1:A:599:ARG:NH1	2:B:513:TRP:CZ3	2.12	1.17
2:B:241:ASP:HB2	3:M:274:ASP:CG	1.64	1.17
2:B:549:LEU:CD2	2:B:611:ALA:N	2.07	1.17
2:B:513:TRP:HA	2:B:551:LEU:CD1	1.74	1.17
1:A:563:CYS:HB3	1:A:621:LEU:HD11	1.26	1.17
2:B:139:LEU:HD23	2:B:173:VAL:CA	1.73	1.17

Continued on next page...

Continued from previous page...

Atom-1	Atom-2	Interatomic distance (Å)	Clash overlap (Å)
1:A:404:GLN:CA	2:B:3:ASP:CB	2.21	1.17
2:B:197:LYS:HB2	2:B:229:HIS:NE2	1.58	1.17
2:B:247:TYR:OH	3:M:137:SER:CB	1.92	1.17
1:A:404:GLN:O	2:B:3:ASP:CB	1.93	1.17
1:A:595:GLU:OE2	2:B:476:ARG:CZ	1.92	1.17
2:B:216:LYS:CA	2:B:251:LEU:HD13	1.72	1.17
1:A:403:LEU:O	2:B:3:ASP:HB2	1.00	1.16
2:B:25:VAL:CB	2:B:36:THR:OG1	1.91	1.16
3:M:435:LEU:O	3:M:479:PHE:CD2	1.97	1.16
1:A:225:LEU:HB3	1:A:233:PHE:CE1	1.81	1.16
1:A:630:PRO:HG3	2:B:617:LEU:HD11	1.25	1.16
2:B:143:SER:CB	2:B:179:LYS:HD2	1.74	1.16
2:B:534:ILE:CG2	2:B:594:ALA:HB1	1.73	1.16
3:M:355:ASP:OD2	3:M:357:LYS:NZ	1.77	1.16
2:B:87:VAL:HG13	2:B:122:SER:CB	1.75	1.16
2:B:534:ILE:HD13	2:B:591:MET:O	1.40	1.16
2:B:24:ALA:HB3	2:B:35:TYR:CE1	1.74	1.16
2:B:219:TYR:CD1	2:B:226:LEU:HD22	1.81	1.16
1:A:638:LEU:CD1	2:B:558:TYR:HB3	1.72	1.16
2:B:472:VAL:HG11	2:B:510:GLY:CA	1.76	1.16
2:B:25:VAL:CG1	2:B:36:THR:OG1	1.93	1.15
1:A:595:GLU:CA	2:B:476:ARG:HH22	1.58	1.15
2:B:102:HIS:CE1	2:B:137:PHE:C	2.17	1.15
2:B:245:GLN:CG	2:B:309:LEU:HD11	1.75	1.15
2:B:534:ILE:HG21	2:B:594:ALA:HB3	1.23	1.15
2:B:278:PRO:HD2	2:B:292:GLU:CG	1.75	1.15
3:M:219:LEU:HD13	3:M:472:TYR:O	1.42	1.15
1:A:404:GLN:HB3	2:B:7:ARG:NH2	1.60	1.15
2:B:519:ALA:O	2:B:523:PHE:CB	1.95	1.15
3:M:379:LEU:HA	3:M:412:ARG:O	1.44	1.15
1:A:594:PHE:CD2	2:B:477:MET:HG3	1.81	1.15
2:B:243:TRP:CH2	3:M:94:GLU:CB	2.30	1.14
1:A:179:LYS:HB3	4:S:142:ILE:HD12	1.29	1.14
1:A:595:GLU:OE1	2:B:476:ARG:NH1	1.79	1.14
2:B:25:VAL:HG21	2:B:36:THR:CB	1.76	1.14
2:B:230:PHE:CZ	2:B:252:LEU:CD2	2.31	1.14
1:A:114:PHE:CZ	1:A:152:THR:O	2.01	1.14
2:B:25:VAL:CG2	2:B:36:THR:CA	2.16	1.14
2:B:293:VAL:O	2:B:299:LEU:HG	1.43	1.14
2:B:178:ILE:CG1	2:B:214:ALA:HB1	1.76	1.14
2:B:208:ILE:CD1	2:B:236:ILE:HG21	1.78	1.14

Continued on next page...

Continued from previous page...

Atom-1	Atom-2	Interatomic distance (Å)	Clash overlap (Å)
2:B:534:ILE:HG21	2:B:594:ALA:HB1	1.15	1.14
1:A:638:LEU:HG	2:B:520:SER:HB2	1.21	1.13
1:A:638:LEU:CG	2:B:558:TYR:CB	2.23	1.13
2:B:234:CYS:O	2:B:237:ILE:HG22	1.43	1.13
3:M:268:GLY:N	3:M:302:TYR:OH	1.77	1.13
1:A:225:LEU:HD13	1:A:233:PHE:HZ	1.04	1.13
1:A:332:TYR:CZ	1:A:336:ILE:HD11	1.83	1.13
2:B:353:GLN:CG	3:M:50:TYR:HD1	1.60	1.13
3:M:59:ASP:O	3:M:61:GLU:N	1.81	1.13
1:A:638:LEU:CD1	2:B:558:TYR:CG	2.25	1.13
1:A:102:GLN:HE22	4:S:166:LYS:NZ	1.47	1.12
2:B:18:ILE:HB	2:B:23:ALA:HB2	1.24	1.12
2:B:21:GLU:OE1	2:B:39:SER:HB2	1.39	1.12
2:B:80:GLN:HG2	2:B:115:LEU:HD21	1.25	1.12
2:B:87:VAL:CG1	2:B:122:SER:HB3	1.79	1.12
2:B:151:ALA:HA	2:B:180:LEU:HD11	1.29	1.13
2:B:123:LEU:HD13	2:B:142:LEU:HG	1.15	1.12
2:B:230:PHE:CE1	2:B:252:LEU:CD2	2.31	1.12
2:B:523:PHE:HZ	2:B:580:TYR:CE1	1.67	1.12
2:B:553:ALA:HB2	2:B:614:ILE:CD1	1.79	1.12
1:A:260:PHE:O	1:A:261:THR:C	1.77	1.12
1:A:295:VAL:HG22	1:A:315:CYS:HB3	1.27	1.12
2:B:280:PRO:CG	2:B:283:TYR:CD1	2.33	1.12
1:A:179:LYS:CB	4:S:142:ILE:HD13	1.66	1.12
2:B:374:PHE:HD2	2:B:402:LEU:CD2	1.62	1.12
2:B:545:ARG:HD3	2:B:602:ASP:HB2	1.25	1.12
2:B:559:ASP:CB	2:B:563:PHE:CD1	2.32	1.12
1:A:594:PHE:CE2	2:B:477:MET:HG3	1.84	1.11
2:B:486:HIS:NE2	2:B:518:ILE:HB	1.65	1.11
2:B:139:LEU:HG	2:B:176:ALA:HB2	1.28	1.11
2:B:437:SER:HB2	2:B:474:VAL:HG13	1.18	1.11
4:S:135:ILE:O	4:S:141:VAL:HA	1.49	1.11
1:A:121:LEU:HD21	1:A:158:LEU:HB2	1.11	1.11
1:A:630:PRO:HG2	2:B:617:LEU:CD1	1.64	1.11
2:B:38:TYR:HA	2:B:42:ILE:N	1.65	1.11
2:B:143:SER:HB2	2:B:179:LYS:HB2	1.33	1.11
2:B:559:ASP:O	2:B:563:PHE:N	1.82	1.11
1:A:179:LYS:HE2	4:S:140:MET:O	1.48	1.11
2:B:181:TYR:CE1	2:B:222:HIS:CD2	2.37	1.11
2:B:216:LYS:HB2	2:B:251:LEU:HD13	1.19	1.11
2:B:219:TYR:CZ	2:B:226:LEU:CB	2.32	1.11

Continued on next page...

Continued from previous page...

Atom-1	Atom-2	Interatomic distance (Å)	Clash overlap (Å)
2:B:252:LEU:CD1	2:B:302:PHE:CE1	2.32	1.11
3:M:347:PHE:CE1	3:M:350:VAL:CG1	2.33	1.11
1:A:156:PRO:HB3	1:A:192:TYR:CE1	1.86	1.10
2:B:155:LEU:CD2	2:B:192:LEU:HD12	1.79	1.10
2:B:243:TRP:NE1	3:M:98:ARG:CD	2.09	1.10
3:M:41:LEU:HD13	3:M:52:ASP:N	1.63	1.10
3:M:222:PHE:O	3:M:479:PHE:CZ	2.04	1.10
2:B:24:ALA:CB	2:B:35:TYR:CE2	2.26	1.10
2:B:219:TYR:CE2	2:B:226:LEU:HB2	1.86	1.10
2:B:433:VAL:HG12	2:B:474:VAL:CG2	1.81	1.10
1:A:257:LEU:HD22	1:A:278:ILE:HG22	1.16	1.10
1:A:589:SER:O	1:A:597:GLN:HG3	1.49	1.10
2:B:559:ASP:HB2	2:B:563:PHE:CD1	1.86	1.10
2:B:44:PRO:HB3	2:B:82:TYR:OH	1.49	1.10
2:B:136:CYS:C	2:B:172:GLU:HG3	1.72	1.10
2:B:479:VAL:HG13	2:B:486:HIS:CG	1.84	1.10
1:A:289:SER:HB3	4:S:96:LEU:CD1	1.67	1.10
2:B:219:TYR:CG	2:B:226:LEU:HB2	1.86	1.10
2:B:243:TRP:CE2	3:M:98:ARG:HD2	1.87	1.10
1:A:101:GLN:OE1	4:S:167:ILE:HD11	1.51	1.09
1:A:638:LEU:HD12	2:B:558:TYR:HD2	0.95	1.09
2:B:277:CYS:O	2:B:288:TYR:HB3	0.93	1.09
1:A:609:LEU:CD2	1:A:628:VAL:HG11	1.81	1.09
2:B:374:PHE:CD2	2:B:402:LEU:HD21	1.87	1.09
1:A:65:ASN:CA	4:S:166:LYS:CA	2.15	1.09
1:A:114:PHE:CD1	1:A:153:ILE:HG23	1.88	1.09
1:A:225:LEU:CD1	1:A:233:PHE:CZ	2.35	1.09
1:A:638:LEU:CB	2:B:558:TYR:CD1	2.17	1.09
2:B:86:VAL:HG12	2:B:101:ILE:HG23	1.35	1.09
2:B:216:LYS:CA	2:B:251:LEU:CD1	2.28	1.09
2:B:239:GLN:OE1	3:M:279:ASN:CA	1.89	1.09
2:B:241:ASP:HB2	3:M:274:ASP:OD1	0.93	1.09
2:B:243:TRP:CH2	3:M:95:THR:N	2.20	1.09
2:B:513:TRP:HA	2:B:551:LEU:HD22	1.35	1.09
2:B:549:LEU:HD11	2:B:611:ALA:HA	1.19	1.09
2:B:578:PRO:HD2	2:B:581:TYR:CE1	1.87	1.09
1:A:151:SER:HB2	1:A:187:LYS:HB2	1.34	1.09
1:A:179:LYS:HZ1	4:S:141:VAL:HA	1.18	1.09
1:A:429:VAL:CB	1:A:469:LEU:HD11	1.82	1.09
2:B:136:CYS:SG	2:B:169:VAL:CA	2.40	1.09
3:M:106:LYS:HB3	3:M:296:LYS:HZ3	1.11	1.09

Continued on next page...

Continued from previous page...

Atom-1	Atom-2	Interatomic distance (Å)	Clash overlap (Å)
3:M:265:ASN:OD1	3:M:313:SER:OG	1.67	1.09
1:A:638:LEU:HD12	2:B:558:TYR:CD2	1.72	1.09
2:B:151:ALA:CA	2:B:180:LEU:HD11	1.83	1.09
2:B:337:THR:HA	2:B:373:LEU:CD2	1.82	1.09
2:B:353:GLN:CG	3:M:50:TYR:CD1	2.35	1.09
2:B:537:PHE:CD2	2:B:598:LEU:HB3	1.87	1.09
1:A:429:VAL:HB	1:A:469:LEU:HD11	1.32	1.08
2:B:25:VAL:HG23	2:B:35:TYR:HD2	1.14	1.08
2:B:178:ILE:HG12	2:B:214:ALA:HB1	1.25	1.08
2:B:527:PRO:HB3	2:B:587:ARG:HG3	1.22	1.08
2:B:87:VAL:HG13	2:B:122:SER:HB3	1.27	1.08
2:B:139:LEU:CD2	2:B:173:VAL:CA	2.31	1.08
2:B:215:TYR:HD1	2:B:233:TYR:CE1	1.65	1.08
2:B:278:PRO:HG2	2:B:292:GLU:OE1	1.52	1.08
2:B:184:GLY:O	2:B:188:TYR:CD1	2.07	1.08
2:B:226:LEU:HD23	2:B:255:TYR:CE1	1.89	1.08
2:B:261:PRO:HB2	2:B:290:SER:HB3	1.32	1.08
3:M:41:LEU:CD1	3:M:52:ASP:N	2.14	1.08
3:M:343:ASN:HA	3:M:408:VAL:HG13	1.18	1.08
1:A:102:GLN:NE2	4:S:166:LYS:HD3	1.69	1.07
1:A:189:PHE:HB2	1:A:225:LEU:HD21	1.31	1.07
2:B:120:ILE:HG13	2:B:150:LEU:HD22	1.23	1.07
2:B:123:LEU:HD12	2:B:142:LEU:CD2	1.83	1.07
2:B:162:VAL:CB	2:B:195:ILE:HG23	1.84	1.07
2:B:216:LYS:HA	2:B:251:LEU:HD11	1.31	1.07
2:B:549:LEU:CD2	2:B:607:ILE:O	2.02	1.07
3:M:96:ILE:HG21	3:M:125:PHE:CZ	1.86	1.07
1:A:258:LYS:NZ	4:S:94:SER:CB	2.18	1.07
2:B:278:PRO:HA	2:B:288:TYR:CA	1.84	1.07
2:B:537:PHE:HB3	2:B:598:LEU:HD13	1.07	1.07
1:A:244:LEU:HD11	1:A:281:LEU:CD1	1.85	1.07
2:B:41:ASN:HB3	2:B:43:ASN:OD1	1.52	1.07
2:B:196:LEU:O	2:B:215:TYR:OH	1.71	1.07
2:B:212:VAL:HG21	2:B:248:LEU:HD21	1.30	1.07
2:B:252:LEU:CB	2:B:302:PHE:CZ	2.37	1.07
2:B:523:PHE:CZ	2:B:580:TYR:CE1	2.42	1.07
1:A:638:LEU:CD1	2:B:520:SER:CA	2.31	1.07
2:B:21:GLU:OE1	2:B:39:SER:HB3	1.49	1.07
2:B:199:LEU:O	2:B:201:ALA:N	1.86	1.07
2:B:38:TYR:HA	2:B:42:ILE:H	1.14	1.07
1:A:125:THR:OG1	1:A:158:LEU:CD1	2.02	1.06

Continued on next page...

Continued from previous page...

Atom-1	Atom-2	Interatomic distance (Å)	Clash overlap (Å)
2:B:354:GLY:O	2:B:358:MET:HG2	1.55	1.06
2:B:523:PHE:CD1	2:B:582:ASP:OD2	2.07	1.06
1:A:638:LEU:CD2	2:B:558:TYR:CB	2.04	1.06
2:B:78:ASP:O	2:B:79:VAL:C	1.72	1.06
2:B:105:LEU:HB3	2:B:145:MET:HE3	1.37	1.06
2:B:247:TYR:CE1	3:M:137:SER:OG	2.07	1.06
2:B:279:LEU:H	2:B:288:TYR:HB2	1.01	1.06
2:B:523:PHE:CE1	2:B:580:TYR:CG	2.42	1.06
2:B:534:ILE:CG2	2:B:594:ALA:CB	2.33	1.06
3:M:245:ASP:HB3	3:M:472:TYR:CD1	1.89	1.06
1:A:189:PHE:CB	1:A:225:LEU:HD21	1.86	1.06
1:A:215:VAL:CG1	1:A:243:ILE:CG2	2.34	1.06
1:A:408:ILE:CG2	4:S:64:ASN:C	2.21	1.06
2:B:208:ILE:HG12	2:B:236:ILE:HD13	1.33	1.06
2:B:219:TYR:HB3	2:B:223:LEU:HD23	1.32	1.06
2:B:236:ILE:HG22	2:B:240:LEU:HD11	1.33	1.06
2:B:523:PHE:CE1	2:B:580:TYR:CB	2.36	1.06
2:B:25:VAL:HG21	2:B:36:THR:HA	1.30	1.06
2:B:25:VAL:HG22	2:B:35:TYR:C	1.74	1.06
2:B:549:LEU:HD22	2:B:611:ALA:HB2	1.35	1.06
4:S:17:VAL:HG21	4:S:19:PHE:CZ	1.90	1.06
1:A:464:ILE:CA	2:B:1:MET:SD	2.43	1.06
2:B:171:GLY:CA	2:B:207:VAL:HG13	1.85	1.06
2:B:181:TYR:CE1	2:B:185:LYS:HG3	1.91	1.06
2:B:243:TRP:CZ3	3:M:94:GLU:CB	2.38	1.06
2:B:337:THR:HG23	2:B:373:LEU:HD11	1.28	1.06
2:B:490:ILE:HG13	2:B:518:ILE:HG21	1.35	1.06
3:M:245:ASP:O	3:M:472:TYR:CE1	2.07	1.06
1:A:176:TYR:OH	4:S:148:ARG:HB3	1.54	1.05
1:A:464:ILE:C	2:B:1:MET:SD	2.35	1.05
2:B:316:THR:CG2	3:M:90:PHE:CD2	2.25	1.05
2:B:517:GLU:OE2	2:B:554:LYS:NZ	1.89	1.05
1:A:207:LEU:O	1:A:243:ILE:HD11	1.55	1.05
1:A:225:LEU:HD13	1:A:233:PHE:CZ	1.89	1.05
2:B:167:ALA:CA	2:B:202:ASP:OD1	2.05	1.05
2:B:553:ALA:HB2	2:B:614:ILE:HD13	1.30	1.05
2:B:25:VAL:HG23	2:B:35:TYR:CD2	1.90	1.05
2:B:178:ILE:CG1	2:B:214:ALA:C	2.25	1.05
2:B:193:LEU:CD2	2:B:225:LEU:HB2	1.85	1.05
2:B:158:VAL:HG11	2:B:177:ILE:HG13	1.33	1.05
2:B:193:LEU:HD13	2:B:225:LEU:HD21	1.36	1.05

Continued on next page...

Continued from previous page...

Atom-1	Atom-2	Interatomic distance (Å)	Clash overlap (Å)
2:B:219:TYR:CZ	2:B:226:LEU:HA	1.87	1.05
2:B:219:TYR:CD2	2:B:226:LEU:HB2	1.90	1.05
3:M:105:ASP:O	3:M:106:LYS:CB	2.03	1.05
1:A:186:PHE:HE1	1:A:224:GLU:HB2	0.96	1.04
2:B:24:ALA:CB	2:B:35:TYR:CG	2.40	1.04
2:B:170:ARG:HH12	2:B:198:GLU:HG2	1.15	1.04
2:B:513:TRP:CA	2:B:551:LEU:HD13	1.83	1.04
1:A:436:CYS:SG	1:A:450:TYR:CZ	2.50	1.04
1:A:586:GLU:O	1:A:587:ASN:C	1.77	1.04
2:B:47:LEU:HD22	2:B:66:ILE:HG13	1.39	1.04
2:B:216:LYS:CB	2:B:251:LEU:CD1	2.35	1.04
2:B:371:GLN:CD	2:B:401:THR:O	1.95	1.04
2:B:374:PHE:CD2	2:B:402:LEU:CD2	2.40	1.04
3:M:218:LEU:HA	3:M:472:TYR:CE2	1.93	1.04
1:A:163:ALA:HB1	1:A:199:ASN:HD21	0.94	1.04
1:A:180:LYS:NZ	4:S:137:GLN:HB3	1.72	1.04
2:B:24:ALA:HB3	2:B:35:TYR:CG	1.92	1.04
2:B:155:LEU:CD2	2:B:192:LEU:HD11	1.83	1.04
2:B:178:ILE:HD12	2:B:218:CYS:H	1.16	1.04
2:B:123:LEU:CD1	2:B:142:LEU:HG	1.88	1.04
2:B:158:VAL:CG1	2:B:177:ILE:HG13	1.86	1.04
2:B:223:LEU:HD13	2:B:258:GLN:C	1.77	1.04
2:B:461:HIS:O	2:B:462:ASN:C	1.84	1.04
1:A:200:PHE:CE2	1:A:236:LEU:HD21	1.91	1.03
1:A:405:THR:N	2:B:7:ARG:CZ	2.10	1.03
2:B:162:VAL:HG21	2:B:195:ILE:HG22	1.08	1.03
2:B:162:VAL:HG21	2:B:195:ILE:HG23	1.13	1.03
3:M:379:LEU:HD23	3:M:411:LEU:HG	1.38	1.03
1:A:186:PHE:CZ	1:A:224:GLU:HG2	1.92	1.03
1:A:215:VAL:CG1	1:A:243:ILE:HG21	1.88	1.03
1:A:637:GLU:HA	2:B:517:GLU:OE1	1.57	1.03
2:B:70:MET:HE3	2:B:107:ARG:CB	1.87	1.03
3:M:219:LEU:CD1	3:M:472:TYR:O	2.06	1.03
2:B:542:PRO:HA	2:B:602:ASP:OD2	1.56	1.03
3:M:350:VAL:HG22	3:M:442:GLN:HG2	1.40	1.03
2:B:98:LYS:HZ1	2:B:134:LEU:HB3	1.03	1.03
2:B:174:ALA:CB	2:B:211:ALA:HA	1.89	1.03
2:B:197:LYS:HA	2:B:229:HIS:CD2	1.92	1.03
2:B:278:PRO:CB	2:B:288:TYR:O	2.07	1.03
2:B:556:LEU:HD22	2:B:588:ILE:HG12	1.38	1.03
1:A:186:PHE:CE1	1:A:224:GLU:CG	2.42	1.03

Continued on next page...

Continued from previous page...

Atom-1	Atom-2	Interatomic distance (Å)	Clash overlap (Å)
1:A:225:LEU:CB	1:A:233:PHE:CZ	2.41	1.03
4:S:131:VAL:HG22	4:S:153:VAL:HG22	1.41	1.03
1:A:180:LYS:NZ	4:S:137:GLN:CB	2.20	1.02
1:A:222:ILE:HG21	1:A:240:LEU:HD11	1.41	1.02
2:B:278:PRO:HD2	2:B:292:GLU:HB2	1.16	1.02
1:A:176:TYR:CE2	4:S:143:GLU:OE2	2.11	1.02
1:A:219:VAL:HG21	1:A:256:LEU:HD21	1.41	1.02
1:A:638:LEU:CD1	2:B:520:SER:N	2.21	1.02
2:B:247:TYR:CZ	3:M:137:SER:OG	2.10	1.02
2:B:347:VAL:HG22	2:B:359:LEU:HB3	1.41	1.02
2:B:437:SER:CB	2:B:474:VAL:HG13	1.89	1.02
3:M:347:PHE:CD1	3:M:350:VAL:HB	1.95	1.02
2:B:230:PHE:CE2	2:B:234:CYS:SG	2.52	1.02
2:B:278:PRO:HD3	2:B:289:PRO:O	1.59	1.02
1:A:163:ALA:HB1	1:A:199:ASN:ND2	1.74	1.02
1:A:605:GLU:HG3	1:A:632:PHE:CD2	1.95	1.02
1:A:638:LEU:CB	2:B:520:SER:HB3	1.89	1.02
2:B:260:LEU:HA	2:B:293:VAL:HG21	1.38	1.02
1:A:114:PHE:CZ	1:A:153:ILE:HA	1.95	1.02
2:B:102:HIS:NE2	2:B:138:ALA:CA	2.12	1.02
2:B:523:PHE:CD1	2:B:559:ASP:OD1	2.13	1.02
2:B:546:CYS:CB	2:B:607:ILE:CG1	2.27	1.02
4:S:53:THR:HG21	4:S:68:VAL:HA	1.39	1.02
1:A:154:ILE:CG2	1:A:191:GLN:HG3	1.90	1.01
2:B:25:VAL:CG2	2:B:36:THR:OG1	2.08	1.01
2:B:223:LEU:CD1	2:B:258:GLN:C	2.28	1.01
2:B:566:ALA:O	2:B:574:ASN:CB	2.07	1.01
2:B:567:GLN:C	2:B:569:THR:N	2.12	1.01
3:M:344:ILE:HG22	3:M:347:PHE:HB3	1.40	1.01
1:A:349:ILE:HG21	1:A:378:ILE:HG22	1.40	1.01
2:B:252:LEU:HB2	2:B:302:PHE:CE1	1.94	1.01
4:S:8:PHE:CZ	4:S:86:THR:OG1	2.11	1.01
2:B:139:LEU:CG	2:B:176:ALA:HB2	1.90	1.01
2:B:223:LEU:HB3	2:B:259:TYR:CG	1.96	1.01
2:B:230:PHE:HZ	2:B:252:LEU:HD22	1.20	1.01
2:B:497:LEU:HD23	2:B:533:LEU:HD21	1.40	1.01
3:M:432:THR:OG1	3:M:480:GLN:CG	2.07	1.01
3:M:443:SER:HB3	3:M:447:ILE:HG13	1.43	1.01
1:A:154:ILE:HB	1:A:191:GLN:HG3	1.41	1.01
1:A:258:LYS:HZ1	4:S:94:SER:CB	1.71	1.01
1:A:638:LEU:HD13	2:B:558:TYR:CD2	1.72	1.01

Continued on next page...

Continued from previous page...

Atom-1	Atom-2	Interatomic distance (Å)	Clash overlap (Å)
1:A:638:LEU:HD13	2:B:558:TYR:HB2	1.36	1.01
3:M:95:THR:OG1	3:M:137:SER:HB2	1.59	1.01
2:B:24:ALA:CB	2:B:35:TYR:CD2	2.42	1.00
2:B:337:THR:CG2	2:B:373:LEU:HD11	1.91	1.00
2:B:374:PHE:CD2	2:B:402:LEU:HD11	1.96	1.00
2:B:433:VAL:HG12	2:B:474:VAL:HG21	1.02	1.00
2:B:458:MET:SD	2:B:471:TYR:HB3	2.02	1.00
2:B:534:ILE:HD11	2:B:591:MET:O	1.60	1.00
1:A:595:GLU:N	2:B:476:ARG:HH22	1.60	1.00
2:B:143:SER:OG	2:B:179:LYS:HD2	1.61	1.00
2:B:261:PRO:HB2	2:B:290:SER:CB	1.91	1.00
2:B:309:LEU:HB3	2:B:317:VAL:HG11	1.40	1.00
2:B:523:PHE:HB2	2:B:559:ASP:OD2	1.61	1.00
1:A:533:ILE:HG12	1:A:562:TRP:CH2	1.97	1.00
2:B:25:VAL:HG21	2:B:36:THR:OG1	1.59	1.00
2:B:297:PRO:O	2:B:301:LEU:HG	1.61	1.00
2:B:509:ALA:HB1	2:B:547:GLN:HG3	1.41	1.00
2:B:578:PRO:HD2	2:B:581:TYR:CD1	1.95	1.00
1:A:225:LEU:CD1	1:A:233:PHE:HZ	1.72	1.00
1:A:239:LEU:O	1:A:242:GLU:O	1.78	1.00
1:A:262:ASN:O	1:A:265:GLN:O	1.77	1.00
2:B:158:VAL:HG11	2:B:177:ILE:HG12	1.01	1.00
2:B:178:ILE:HG23	2:B:217:GLU:CB	1.91	1.00
2:B:252:LEU:HD13	2:B:302:PHE:HD1	1.20	1.00
2:B:259:TYR:HD2	2:B:261:PRO:HG3	1.27	1.00
2:B:259:TYR:CD2	2:B:261:PRO:HG3	1.97	1.00
2:B:353:GLN:HB3	3:M:50:TYR:HD1	1.05	1.00
2:B:374:PHE:HZ	2:B:381:PHE:CD1	1.80	1.00
1:A:404:GLN:C	2:B:3:ASP:CB	2.30	0.99
1:A:609:LEU:HG	1:A:628:VAL:CG1	1.92	0.99
2:B:181:TYR:CE2	2:B:218:CYS:O	2.14	0.99
2:B:212:VAL:O	2:B:214:ALA:N	1.95	0.99
2:B:546:CYS:HB2	2:B:607:ILE:HG12	1.19	0.99
2:B:549:LEU:CD2	2:B:611:ALA:HB2	1.91	0.99
1:A:101:GLN:OE1	4:S:167:ILE:CD1	2.11	0.99
1:A:450:TYR:OH	1:A:476:GLN:CG	2.10	0.99
2:B:422:ALA:CB	2:B:424:PHE:CE1	2.46	0.99
1:A:225:LEU:CB	1:A:233:PHE:CE1	2.46	0.99
1:A:637:GLU:OE2	2:B:517:GLU:HG2	1.57	0.99
2:B:243:TRP:HE1	3:M:98:ARG:HD2	1.15	0.99
2:B:513:TRP:HA	2:B:551:LEU:CG	1.91	0.99

Continued on next page...

Continued from previous page...

Atom-1	Atom-2	Interatomic distance (Å)	Clash overlap (Å)
1:A:260:PHE:CD2	1:A:274:LEU:HG	1.96	0.99
2:B:278:PRO:CA	2:B:288:TYR:C	2.30	0.99
2:B:337:THR:HA	2:B:373:LEU:HD11	1.42	0.99
3:M:245:ASP:O	3:M:472:TYR:CZ	2.16	0.99
4:S:35:VAL:HG12	4:S:75:ILE:HD13	1.41	0.99
2:B:252:LEU:HD12	2:B:302:PHE:CE1	1.98	0.99
3:M:245:ASP:CB	3:M:472:TYR:CD1	2.46	0.99
1:A:65:ASN:CA	4:S:166:LYS:CB	2.39	0.99
1:A:289:SER:HB3	4:S:96:LEU:HD13	1.00	0.99
1:A:323:CYS:SG	1:A:334:SER:HB3	2.03	0.99
2:B:193:LEU:HD22	2:B:225:LEU:HB3	0.99	0.99
2:B:512:VAL:HG11	2:B:548:ILE:HA	1.41	0.99
3:M:293:PRO:HD2	3:M:293:PRO:O	1.62	0.99
2:B:200:MET:HG2	2:B:232:ARG:HB3	1.43	0.99
2:B:212:VAL:O	2:B:213:LEU:C	1.91	0.99
2:B:155:LEU:HD21	2:B:192:LEU:HD12	1.40	0.98
2:B:352:ASN:O	2:B:355:ASN:HB2	1.63	0.98
2:B:479:VAL:CG2	2:B:486:HIS:CD2	2.45	0.98
2:B:42:ILE:O	2:B:46:GLN:CD	2.00	0.98
2:B:106:LEU:HD22	2:B:144:ASP:CB	1.92	0.98
2:B:374:PHE:CD2	2:B:402:LEU:CD1	2.47	0.98
3:M:106:LYS:HB3	3:M:296:LYS:NZ	1.75	0.98
2:B:404:ASN:O	2:B:405:GLU:C	1.83	0.98
2:B:433:VAL:CG1	2:B:474:VAL:HG21	1.93	0.98
2:B:25:VAL:HG11	2:B:36:THR:CB	1.93	0.98
2:B:197:LYS:CB	2:B:229:HIS:NE2	2.26	0.98
2:B:223:LEU:CD1	2:B:258:GLN:HB3	1.93	0.98
2:B:353:GLN:CB	3:M:50:TYR:CD1	2.37	0.98
3:M:350:VAL:HG13	3:M:442:GLN:CB	1.94	0.98
1:A:258:LYS:CE	4:S:94:SER:HB3	1.92	0.98
3:M:364:VAL:O	3:M:367:ALA:O	1.80	0.98
3:M:380:ARG:O	3:M:410:VAL:O	1.82	0.98
2:B:256:CYS:SG	2:B:328:LEU:CD2	2.52	0.98
2:B:563:PHE:HD2	2:B:584:SER:CB	1.76	0.98
3:M:223:HIS:CD2	3:M:478:ASN:HA	1.99	0.98
2:B:227:HIS:O	2:B:229:HIS:N	1.97	0.98
2:B:267:ASP:O	2:B:276:SER:HB2	1.63	0.98
3:M:222:PHE:CD1	3:M:240:ILE:HG23	1.98	0.98
3:M:244:VAL:O	3:M:299:LEU:N	1.96	0.98
1:A:289:SER:O	1:A:290:VAL:C	1.87	0.98
1:A:581:LEU:HD23	1:A:607:LEU:CD1	1.94	0.98

Continued on next page...

Continued from previous page...

Atom-1	Atom-2	Interatomic distance (Å)	Clash overlap (Å)
2:B:588:ILE:HG23	2:B:618:PHE:CZ	1.97	0.98
2:B:105:LEU:O	2:B:106:LEU:C	1.96	0.97
2:B:132:SER:HB2	2:B:169:VAL:HG23	1.45	0.97
2:B:550:VAL:HG22	2:B:610:ARG:HD3	1.45	0.97
3:M:223:HIS:HA	3:M:479:PHE:CD2	1.98	0.97
2:B:181:TYR:CD2	2:B:218:CYS:O	2.17	0.97
3:M:378:ILE:O	3:M:413:GLY:HA3	1.64	0.97
2:B:219:TYR:OH	2:B:226:LEU:HA	1.62	0.97
2:B:261:PRO:CD	2:B:293:VAL:HG23	1.94	0.97
1:A:233:PHE:O	1:A:234:ILE:C	1.77	0.97
2:B:523:PHE:HD1	2:B:582:ASP:OD2	1.43	0.97
3:M:350:VAL:HG13	3:M:442:GLN:HB3	1.46	0.97
3:M:443:SER:HB3	3:M:447:ILE:CG1	1.93	0.97
1:A:88:ASN:CG	1:A:120:ILE:CG2	2.33	0.97
1:A:323:CYS:SG	1:A:334:SER:CB	2.52	0.97
1:A:638:LEU:HD12	2:B:520:SER:N	1.77	0.97
2:B:143:SER:C	2:B:179:LYS:HD3	1.85	0.97
1:A:594:PHE:CD2	2:B:477:MET:CG	2.47	0.97
1:A:638:LEU:CD1	2:B:520:SER:HB3	1.94	0.97
2:B:196:LEU:HB3	2:B:215:TYR:CE2	2.00	0.97
2:B:501:THR:HA	2:B:508:ARG:HH22	1.29	0.97
1:A:102:GLN:NE2	4:S:166:LYS:CD	2.27	0.97
1:A:555:LEU:HD13	1:A:581:LEU:CD1	1.95	0.97
2:B:230:PHE:CD2	2:B:298:ASP:HB3	1.99	0.97
2:B:559:ASP:HB2	2:B:563:PHE:CE1	1.99	0.97
1:A:102:GLN:HE21	4:S:166:LYS:CD	1.78	0.96
1:A:154:ILE:CB	1:A:191:GLN:HG3	1.95	0.96
1:A:219:VAL:HG11	1:A:256:LEU:HD23	1.46	0.96
2:B:178:ILE:HG13	2:B:214:ALA:CB	1.94	0.96
2:B:316:THR:OG1	3:M:90:PHE:CE2	2.16	0.96
1:A:257:LEU:CD2	1:A:278:ILE:HG22	1.94	0.96
1:A:260:PHE:CZ	1:A:274:LEU:HD11	2.00	0.96
2:B:42:ILE:C	2:B:46:GLN:OE1	2.01	0.96
2:B:151:ALA:HA	2:B:180:LEU:CD1	1.95	0.96
2:B:337:THR:HA	2:B:373:LEU:CD1	1.95	0.96
2:B:403:ILE:HB	2:B:408:VAL:HG22	1.45	0.96
2:B:105:LEU:HB3	2:B:145:MET:CE	1.94	0.96
2:B:139:LEU:HD11	2:B:176:ALA:CB	1.95	0.96
1:A:637:GLU:OE2	2:B:517:GLU:CG	2.09	0.96
3:M:218:LEU:HA	3:M:472:TYR:CD2	1.99	0.96
2:B:337:THR:N	2:B:373:LEU:HD21	1.80	0.96

Continued on next page...

Continued from previous page...

Atom-1	Atom-2	Interatomic distance (Å)	Clash overlap (Å)
2:B:537:PHE:HB3	2:B:598:LEU:CD1	1.94	0.96
3:M:351:SER:HB2	3:M:440:ILE:O	1.66	0.96
1:A:381:GLU:O	1:A:382:ASP:C	1.93	0.96
2:B:508:ARG:O	2:B:512:VAL:HG23	1.66	0.96
1:A:404:GLN:CA	2:B:3:ASP:CG	2.26	0.95
1:A:595:GLU:HA	2:B:476:ARG:NH2	1.79	0.95
2:B:297:PRO:O	2:B:301:LEU:CG	2.14	0.95
3:M:272:LEU:CD2	3:M:278:ILE:HB	1.96	0.95
4:S:17:VAL:CG2	4:S:19:PHE:CZ	2.49	0.95
2:B:133:GLU:HA	2:B:168:MET:SD	2.06	0.95
2:B:490:ILE:CG1	2:B:518:ILE:HG21	1.95	0.95
3:M:105:ASP:O	3:M:106:LYS:HB3	1.65	0.95
1:A:200:PHE:HZ	1:A:236:LEU:CD2	1.75	0.95
1:A:488:ARG:O	1:A:491:THR:OG1	1.84	0.95
2:B:106:LEU:CD2	2:B:144:ASP:HB3	1.96	0.95
2:B:174:ALA:HB1	2:B:211:ALA:HA	1.46	0.95
1:A:114:PHE:CE2	1:A:152:THR:O	2.19	0.95
1:A:403:LEU:HD23	1:A:422:GLU:HG3	1.46	0.95
2:B:136:CYS:SG	2:B:168:MET:C	2.45	0.95
2:B:189:HIS:NE2	2:B:193:LEU:HD11	1.80	0.95
2:B:513:TRP:CA	2:B:551:LEU:CD2	2.44	0.95
4:S:1:MET:N	4:S:93:GLU:OE1	1.99	0.95
1:A:186:PHE:CE1	1:A:224:GLU:HG2	2.00	0.95
2:B:374:PHE:CZ	2:B:381:PHE:CD1	2.54	0.95
2:B:155:LEU:HD23	2:B:192:LEU:HD11	0.97	0.95
2:B:278:PRO:HB3	2:B:288:TYR:O	1.65	0.95
2:B:418:TYR:CD1	2:B:418:TYR:C	2.33	0.95
2:B:479:VAL:HG22	2:B:486:HIS:CD2	2.01	0.95
2:B:566:ALA:C	2:B:574:ASN:ND2	2.19	0.95
1:A:559:PHE:CE1	1:A:581:LEU:HD22	2.02	0.95
2:B:278:PRO:CA	2:B:288:TYR:CB	2.44	0.95
2:B:559:ASP:HB3	2:B:563:PHE:CD1	1.98	0.95
3:M:223:HIS:HD1	3:M:476:THR:HG1	1.01	0.95
2:B:231:ARG:HG2	2:B:298:ASP:OD1	1.65	0.95
3:M:339:GLU:HG3	3:M:412:ARG:HG2	1.45	0.95
2:B:219:TYR:CD1	2:B:226:LEU:CD2	2.49	0.95
1:A:215:VAL:HG13	1:A:243:ILE:HG21	1.45	0.95
2:B:278:PRO:C	2:B:288:TYR:HB2	1.85	0.95
2:B:566:ALA:HA	2:B:574:ASN:HB3	1.48	0.95
3:M:265:ASN:OD1	3:M:313:SER:CB	2.15	0.95
1:A:65:ASN:HA	4:S:166:LYS:HA	1.07	0.94

Continued on next page...

Continued from previous page...

Atom-1	Atom-2	Interatomic distance (Å)	Clash overlap (Å)
2:B:136:CYS:SG	2:B:169:VAL:N	2.39	0.94
2:B:178:ILE:CG1	2:B:214:ALA:CB	2.45	0.94
2:B:486:HIS:CE1	2:B:518:ILE:HB	2.02	0.94
2:B:278:PRO:CD	2:B:292:GLU:CB	2.30	0.94
2:B:316:THR:CB	3:M:90:PHE:CE2	2.39	0.94
1:A:179:LYS:CE	4:S:140:MET:O	2.15	0.94
2:B:212:VAL:CG2	2:B:248:LEU:HD21	1.97	0.94
2:B:223:LEU:HD11	2:B:258:GLN:HB3	0.97	0.94
2:B:25:VAL:HG13	2:B:33:SER:HA	1.50	0.94
2:B:83:PHE:CZ	2:B:119:SER:CB	2.50	0.94
2:B:143:SER:O	2:B:179:LYS:HD3	1.67	0.94
2:B:219:TYR:CD1	2:B:226:LEU:CG	2.50	0.94
2:B:343:LEU:HD23	2:B:363:ILE:HD13	1.48	0.94
3:M:96:ILE:HG23	3:M:125:PHE:CE1	2.02	0.94
4:S:80:TYR:O	4:S:82:THR:N	2.00	0.94
2:B:232:ARG:O	2:B:236:ILE:HG13	1.68	0.94
2:B:245:GLN:OE1	2:B:309:LEU:CD1	2.14	0.94
2:B:513:TRP:CA	2:B:551:LEU:HD11	1.95	0.94
1:A:176:TYR:CZ	4:S:143:GLU:OE2	2.21	0.94
1:A:225:LEU:HB3	1:A:233:PHE:CZ	2.03	0.94
2:B:219:TYR:CD1	2:B:226:LEU:CB	2.47	0.94
2:B:223:LEU:CD1	2:B:258:GLN:CB	2.46	0.94
2:B:267:ASP:H	2:B:289:PRO:CB	1.80	0.94
2:B:527:PRO:CB	2:B:587:ARG:HG3	1.98	0.94
2:B:566:ALA:CA	2:B:574:ASN:CG	2.34	0.94
3:M:41:LEU:HD13	3:M:52:ASP:H	0.79	0.94
2:B:139:LEU:HD21	2:B:173:VAL:HA	1.47	0.94
2:B:208:ILE:CG1	2:B:236:ILE:HD13	1.98	0.94
2:B:237:ILE:O	2:B:238:LYS:C	1.99	0.94
2:B:158:VAL:HG13	2:B:173:VAL:HG12	1.48	0.93
2:B:167:ALA:O	2:B:207:VAL:HG21	1.65	0.93
2:B:216:LYS:CG	2:B:251:LEU:HD13	1.99	0.93
2:B:215:TYR:HD2	2:B:219:TYR:CE1	1.86	0.93
2:B:340:ILE:HG12	2:B:373:LEU:HD23	1.49	0.93
1:A:289:SER:CA	4:S:96:LEU:HD13	1.98	0.93
2:B:143:SER:C	2:B:179:LYS:CD	2.36	0.93
2:B:549:LEU:HD21	2:B:611:ALA:H	1.17	0.93
1:A:595:GLU:HA	2:B:476:ARG:HH22	1.30	0.93
2:B:38:TYR:CA	2:B:42:ILE:H	1.80	0.93
2:B:90:ILE:O	2:B:98:LYS:HE2	1.67	0.93
2:B:178:ILE:HG23	2:B:217:GLU:HB2	0.95	0.93

Continued on next page...

Continued from previous page...

Atom-1	Atom-2	Interatomic distance (Å)	Clash overlap (Å)
2:B:476:ARG:HA	2:B:514:LEU:HD13	1.48	0.93
2:B:564:LYS:HD2	2:B:621:GLY:O	1.67	0.93
1:A:215:VAL:HG11	1:A:243:ILE:HG23	1.48	0.93
2:B:230:PHE:HE1	2:B:252:LEU:HD23	1.24	0.93
1:A:244:LEU:HG	1:A:281:LEU:HD11	1.49	0.93
2:B:566:ALA:HA	2:B:574:ASN:CB	1.98	0.93
3:M:220:GLU:CG	3:M:439:TYR:HD1	1.82	0.93
4:S:89:VAL:HG11	4:S:98:ILE:HG13	1.48	0.93
1:A:637:GLU:OE1	2:B:554:LYS:NZ	2.02	0.93
2:B:556:LEU:HA	2:B:588:ILE:HD11	0.93	0.93
2:B:70:MET:CE	2:B:107:ARG:CG	2.47	0.92
4:S:8:PHE:CD1	4:S:84:TYR:HB2	2.03	0.92
1:A:179:LYS:HB3	4:S:142:ILE:HD13	0.93	0.92
1:A:319:LEU:O	1:A:320:HIS:C	2.05	0.92
1:A:638:LEU:HG	2:B:520:SER:CA	2.00	0.92
2:B:171:GLY:N	2:B:207:VAL:HG13	1.83	0.92
2:B:241:ASP:CB	3:M:274:ASP:HA	2.00	0.92
2:B:252:LEU:CB	2:B:302:PHE:CE1	2.52	0.92
2:B:527:PRO:HB3	2:B:587:ARG:CG	1.99	0.92
2:B:513:TRP:N	2:B:551:LEU:HD11	1.82	0.92
3:M:336:ASP:OD1	3:M:415:ILE:O	1.86	0.92
1:A:77:LEU:O	1:A:80:TYR:O	1.87	0.92
1:A:513:ARG:HD2	1:A:550:VAL:HG21	1.52	0.92
2:B:42:ILE:CA	2:B:46:GLN:OE1	2.17	0.92
3:M:235:LEU:HD11	3:M:306:LEU:HB3	1.52	0.92
2:B:219:TYR:CZ	2:B:226:LEU:N	2.37	0.92
2:B:568:VAL:O	2:B:571:SER:HB2	1.69	0.92
2:B:248:LEU:O	2:B:252:LEU:HG	1.68	0.92
1:A:595:GLU:CA	2:B:476:ARG:NH2	2.33	0.92
1:A:260:PHE:CG	1:A:274:LEU:HG	2.05	0.92
2:B:143:SER:HB2	2:B:179:LYS:CB	2.00	0.92
2:B:241:ASP:HB3	3:M:274:ASP:HA	1.50	0.92
2:B:479:VAL:CG1	2:B:486:HIS:NE2	2.12	0.92
3:M:96:ILE:CG2	3:M:125:PHE:CE1	2.53	0.92
1:A:121:LEU:HD13	1:A:155:THR:HG23	1.52	0.92
1:A:219:VAL:CG1	1:A:256:LEU:HD23	2.00	0.92
1:A:102:GLN:HE21	4:S:166:LYS:HD3	1.27	0.91
1:A:156:PRO:HB3	1:A:192:TYR:HE1	1.23	0.91
1:A:180:LYS:HZ2	4:S:137:GLN:HB3	1.36	0.91
1:A:461:CYS:O	1:A:462:GLN:C	1.94	0.91
2:B:41:ASN:CB	2:B:43:ASN:OD1	2.18	0.91

Continued on next page...

Continued from previous page...

Atom-1	Atom-2	Interatomic distance (Å)	Clash overlap (Å)
1:A:289:SER:CB	4:S:96:LEU:CD1	2.36	0.91
1:A:349:ILE:CG2	1:A:378:ILE:HG22	2.00	0.91
2:B:393:ILE:HG23	2:B:431:MET:HG2	1.52	0.91
2:B:518:ILE:O	2:B:518:ILE:HD12	1.70	0.91
2:B:549:LEU:CD1	2:B:611:ALA:HA	1.98	0.91
2:B:211:ALA:O	2:B:214:ALA:HB3	1.67	0.91
1:A:502:ASP:OD2	1:A:506:LYS:NZ	2.03	0.91
1:A:609:LEU:HD23	1:A:628:VAL:HG11	1.51	0.91
2:B:437:SER:HB2	2:B:474:VAL:CG1	2.00	0.91
2:B:546:CYS:HA	2:B:607:ILE:HG12	1.48	0.91
3:M:16:PHE:HA	3:M:118:TYR:CD1	2.05	0.91
3:M:344:ILE:CG2	3:M:347:PHE:CB	2.48	0.91
2:B:219:TYR:CE2	2:B:226:LEU:N	2.39	0.91
3:M:220:GLU:HG3	3:M:439:TYR:HD1	1.36	0.91
1:A:189:PHE:CB	1:A:225:LEU:CD2	2.48	0.91
2:B:374:PHE:CE2	2:B:402:LEU:CD1	2.53	0.91
2:B:566:ALA:HA	2:B:574:ASN:CG	1.90	0.91
1:A:244:LEU:CD1	1:A:281:LEU:CD1	2.47	0.91
2:B:239:GLN:OE1	3:M:279:ASN:C	2.08	0.91
2:B:307:ASN:OD1	2:B:339:PHE:CD2	2.22	0.91
2:B:374:PHE:HE2	2:B:402:LEU:HD11	1.36	0.91
2:B:567:GLN:HA	2:B:569:THR:OG1	1.70	0.91
3:M:71:LYS:HB3	3:M:74:TYR:CZ	2.06	0.91
1:A:180:LYS:HZ2	4:S:137:GLN:CB	1.83	0.91
1:A:204:VAL:HG13	1:A:239:LEU:CD1	2.00	0.91
2:B:105:LEU:HG	2:B:119:SER:HB2	1.53	0.91
2:B:124:GLN:HA	2:B:127:LEU:HD12	1.53	0.91
2:B:252:LEU:HB3	2:B:302:PHE:CE2	2.06	0.91
1:A:220:SER:O	1:A:223:CYS:HB3	1.71	0.90
1:A:630:PRO:CB	2:B:617:LEU:HD13	2.00	0.90
1:A:638:LEU:HD11	2:B:520:SER:HA	1.53	0.90
2:B:123:LEU:HD13	2:B:142:LEU:CG	2.01	0.90
2:B:193:LEU:HD13	2:B:225:LEU:CD1	1.99	0.90
2:B:219:TYR:CD1	2:B:226:LEU:CD1	2.44	0.90
2:B:256:CYS:SG	2:B:328:LEU:HD22	2.11	0.90
2:B:337:THR:HG23	2:B:373:LEU:CD1	2.01	0.90
2:B:486:HIS:ND1	2:B:518:ILE:CD1	2.29	0.90
2:B:584:SER:O	2:B:588:ILE:CG2	2.18	0.90
3:M:347:PHE:CE1	3:M:350:VAL:HG12	2.04	0.90
3:M:362:PHE:O	3:M:363:ASN:C	2.07	0.90
1:A:190:LEU:HD12	1:A:228:LYS:HG3	1.52	0.90

Continued on next page...

Continued from previous page...

Atom-1	Atom-2	Interatomic distance (Å)	Clash overlap (Å)
1:A:404:GLN:O	2:B:3:ASP:C	2.10	0.90
2:B:189:HIS:CE1	2:B:193:LEU:HD11	2.06	0.90
2:B:230:PHE:O	2:B:231:ARG:O	1.88	0.90
2:B:29:LYS:CE	2:B:30:LEU:N	2.07	0.90
2:B:231:ARG:NH2	2:B:279:LEU:HD21	1.85	0.90
2:B:566:ALA:O	2:B:574:ASN:HB3	1.72	0.90
1:A:114:PHE:CG	1:A:153:ILE:HG23	2.05	0.90
1:A:370:LYS:O	1:A:374:LEU:HD13	1.72	0.90
1:A:563:CYS:HB3	1:A:621:LEU:HD12	1.51	0.90
2:B:24:ALA:HB2	2:B:35:TYR:CZ	2.04	0.90
2:B:219:TYR:CG	2:B:226:LEU:HD22	2.06	0.90
1:A:516:ILE:HG22	1:A:554:ALA:CB	2.02	0.90
1:A:638:LEU:HD11	2:B:520:SER:CA	2.00	0.90
2:B:20:ARG:CD	2:B:21:GLU:HB2	2.00	0.90
2:B:486:HIS:CE1	2:B:518:ILE:CD1	2.54	0.90
1:A:599:ARG:CZ	2:B:513:TRP:CZ3	2.54	0.90
2:B:181:TYR:CE1	2:B:222:HIS:HD2	1.88	0.90
2:B:353:GLN:HG2	3:M:50:TYR:CD1	2.04	0.90
2:B:523:PHE:CE1	2:B:580:TYR:CD1	2.56	0.90
3:M:59:ASP:O	3:M:60:LEU:C	1.83	0.90
3:M:219:LEU:HD22	3:M:473:LYS:HA	1.53	0.90
3:M:240:ILE:HG21	3:M:444:ALA:O	1.72	0.90
1:A:111:SER:HB2	1:A:152:THR:HG1	1.36	0.90
2:B:106:LEU:CD2	2:B:144:ASP:CB	2.50	0.90
2:B:208:ILE:CD1	2:B:236:ILE:CG2	2.42	0.90
2:B:261:PRO:HD3	2:B:293:VAL:HG23	1.53	0.90
2:B:299:LEU:O	2:B:302:PHE:HB3	1.72	0.90
1:A:595:GLU:CD	2:B:476:ARG:CZ	2.38	0.90
2:B:29:LYS:CD	2:B:30:LEU:H	1.84	0.90
2:B:226:LEU:HD23	2:B:255:TYR:HD1	1.28	0.90
1:A:176:TYR:CD2	4:S:143:GLU:OE2	2.25	0.90
1:A:563:CYS:CB	1:A:621:LEU:HD11	2.02	0.90
2:B:25:VAL:HG22	2:B:36:THR:H	1.25	0.90
2:B:553:ALA:HB2	2:B:614:ILE:CG1	2.02	0.90
2:B:170:ARG:HA	2:B:199:LEU:HD22	1.53	0.89
1:A:381:GLU:HA	1:A:384:LEU:HD23	1.51	0.89
1:A:638:LEU:CA	2:B:520:SER:HB3	1.97	0.89
2:B:374:PHE:HB3	2:B:402:LEU:CD2	2.01	0.89
4:S:80:TYR:O	4:S:81:ALA:C	2.07	0.89
2:B:123:LEU:O	2:B:127:LEU:HG	1.70	0.89
2:B:193:LEU:CD2	2:B:225:LEU:HB3	1.87	0.89

Continued on next page...

Continued from previous page...

Atom-1	Atom-2	Interatomic distance (Å)	Clash overlap (Å)
2:B:375:LEU:HD21	2:B:402:LEU:O	1.71	0.89
3:M:334:ASP:O	3:M:417:TYR:N	2.05	0.89
2:B:243:TRP:CZ2	3:M:98:ARG:HD2	2.07	0.89
2:B:267:ASP:O	2:B:276:SER:CB	2.21	0.89
2:B:352:ASN:ND2	3:M:70:ASN:CB	2.35	0.89
2:B:500:GLN:HB3	2:B:503:LEU:HG	1.54	0.89
3:M:327:PHE:HE1	3:M:336:ASP:HB2	1.38	0.89
3:M:443:SER:CB	3:M:447:ILE:HG13	2.02	0.89
1:A:179:LYS:CB	4:S:142:ILE:HD12	1.84	0.89
1:A:366:SER:O	1:A:370:LYS:HG2	1.71	0.89
2:B:139:LEU:CD1	2:B:176:ALA:CB	2.50	0.89
2:B:309:LEU:HB3	2:B:317:VAL:HG12	1.54	0.89
2:B:549:LEU:HD23	2:B:607:ILE:O	1.71	0.89
3:M:65:TYR:CZ	3:M:86:PRO:HB3	2.08	0.89
3:M:302:TYR:CD2	3:M:445:SER:HB3	2.07	0.89
1:A:257:LEU:HD22	1:A:278:ILE:HG23	1.55	0.89
1:A:369:SER:HB2	1:A:424:TYR:HE2	1.38	0.89
2:B:83:PHE:HZ	2:B:119:SER:HB3	1.25	0.89
2:B:178:ILE:HD13	2:B:218:CYS:HB2	1.53	0.89
2:B:252:LEU:HB3	2:B:302:PHE:CD2	2.08	0.89
2:B:472:VAL:CG1	2:B:510:GLY:CA	2.44	0.89
1:A:516:ILE:CG2	1:A:554:ALA:HB3	2.03	0.89
2:B:472:VAL:HG11	2:B:510:GLY:HA3	0.89	0.89
3:M:347:PHE:O	3:M:348:LYS:C	2.11	0.89
1:A:88:ASN:ND2	1:A:120:ILE:CG2	2.35	0.88
1:A:638:LEU:HD22	2:B:558:TYR:C	1.92	0.88
2:B:127:LEU:HB3	2:B:157:THR:HG23	1.54	0.88
2:B:193:LEU:CG	2:B:225:LEU:CG	2.26	0.88
1:A:102:GLN:NE2	4:S:166:LYS:NZ	2.20	0.88
2:B:20:ARG:CZ	2:B:21:GLU:HG3	2.03	0.88
2:B:193:LEU:HB3	2:B:225:LEU:CD1	2.03	0.88
2:B:223:LEU:HB2	2:B:259:TYR:CD1	2.08	0.88
3:M:235:LEU:HD13	3:M:310:VAL:HG21	1.55	0.88
2:B:103:LEU:CD2	3:M:131:ALA:HA	2.02	0.88
3:M:306:LEU:HD11	3:M:317:MET:HE1	1.53	0.88
1:A:101:GLN:CD	4:S:167:ILE:HD11	1.93	0.88
2:B:127:LEU:HD13	2:B:157:THR:CB	2.03	0.88
2:B:549:LEU:CD2	2:B:611:ALA:CB	2.51	0.88
2:B:552:SER:O	2:B:556:LEU:HG	1.72	0.88
3:M:223:HIS:HA	3:M:479:PHE:CE2	2.09	0.88
2:B:219:TYR:CE1	2:B:226:LEU:CG	2.57	0.88

Continued on next page...

Continued from previous page...

Atom-1	Atom-2	Interatomic distance (Å)	Clash overlap (Å)
3:M:222:PHE:C	3:M:479:PHE:CZ	2.47	0.88
3:M:272:LEU:HD21	3:M:278:ILE:HB	1.54	0.88
2:B:193:LEU:HB3	2:B:225:LEU:HD12	1.54	0.88
2:B:549:LEU:HD11	2:B:611:ALA:CA	2.03	0.88
1:A:609:LEU:HD21	1:A:628:VAL:HG21	1.54	0.88
1:A:638:LEU:CD1	2:B:520:SER:CB	2.50	0.88
2:B:534:ILE:HD13	2:B:594:ALA:HB3	1.55	0.88
3:M:343:ASN:CA	3:M:408:VAL:HG13	2.02	0.88
3:M:347:PHE:CE1	3:M:350:VAL:HG11	2.09	0.88
2:B:219:TYR:HE1	2:B:226:LEU:HD13	1.11	0.88
3:M:378:ILE:O	3:M:413:GLY:CA	2.22	0.88
2:B:139:LEU:HD23	2:B:173:VAL:HA	0.89	0.88
4:S:54:PRO:HD2	4:S:57:LEU:HB2	1.56	0.88
1:A:289:SER:C	4:S:96:LEU:HD13	1.94	0.87
1:A:516:ILE:CG2	1:A:554:ALA:CB	2.52	0.87
2:B:21:GLU:CD	2:B:35:TYR:CD2	2.46	0.87
3:M:327:PHE:CE1	3:M:336:ASP:HB2	2.09	0.87
2:B:42:ILE:HA	2:B:46:GLN:OE1	1.75	0.87
2:B:215:TYR:CD2	2:B:219:TYR:HE1	1.92	0.87
3:M:220:GLU:HG3	3:M:439:TYR:CD1	2.09	0.87
3:M:290:PHE:CE2	3:M:297:PHE:CZ	2.62	0.87
3:M:306:LEU:O	3:M:307:SER:C	2.08	0.87
2:B:208:ILE:HG21	2:B:236:ILE:HG21	1.56	0.87
2:B:497:LEU:O	2:B:498:THR:C	2.00	0.87
3:M:435:LEU:O	3:M:479:PHE:CG	2.27	0.87
4:S:75:ILE:HG22	4:S:77:TYR:CE1	2.09	0.87
2:B:82:TYR:O	2:B:83:PHE:C	2.01	0.87
2:B:569:THR:HG22	2:B:569:THR:O	1.73	0.87
1:A:174:ARG:NH2	4:S:148:ARG:HH22	1.73	0.87
1:A:450:TYR:OH	1:A:476:GLN:HG3	1.75	0.87
1:A:573:GLU:O	1:A:574:ILE:C	2.01	0.87
2:B:25:VAL:N	2:B:35:TYR:CD2	2.43	0.87
2:B:86:VAL:CG1	2:B:101:ILE:HG23	2.05	0.87
2:B:120:ILE:CD1	2:B:150:LEU:HD13	2.05	0.87
2:B:243:TRP:NE1	3:M:98:ARG:NE	2.22	0.87
2:B:243:TRP:HD1	3:M:274:ASP:OD2	1.56	0.87
2:B:340:ILE:CG1	2:B:373:LEU:HD23	2.04	0.87
1:A:370:LYS:O	1:A:374:LEU:CD1	2.23	0.87
1:A:633:PHE:CE2	2:B:554:LYS:HB2	2.10	0.87
2:B:290:SER:O	2:B:291:TYR:C	1.93	0.87
2:B:418:TYR:O	2:B:419:VAL:C	2.00	0.87

Continued on next page...

Continued from previous page...

Atom-1	Atom-2	Interatomic distance (Å)	Clash overlap (Å)
2:B:486:HIS:CG	2:B:518:ILE:HD13	2.08	0.87
2:B:553:ALA:CB	2:B:614:ILE:HG12	2.05	0.87
1:A:254:ILE:HG13	1:A:290:VAL:HG22	1.55	0.87
2:B:208:ILE:HD13	2:B:236:ILE:HG21	0.89	0.87
1:A:88:ASN:HB3	1:A:120:ILE:HG23	1.56	0.86
2:B:139:LEU:CD1	2:B:176:ALA:HB2	2.04	0.86
2:B:243:TRP:HZ3	3:M:94:GLU:HB3	1.05	0.86
2:B:328:LEU:CB	2:B:333:GLN:HE22	1.88	0.86
2:B:523:PHE:HE1	2:B:580:TYR:HB2	1.37	0.86
3:M:479:PHE:H	3:M:479:PHE:HD2	1.17	0.86
4:S:35:VAL:HB	4:S:77:TYR:OH	1.73	0.86
1:A:339:TYR:HB2	1:A:374:LEU:HD11	1.57	0.86
2:B:352:ASN:HD21	3:M:70:ASN:HB3	1.36	0.86
3:M:323:MET:SD	3:M:342:LEU:HA	2.14	0.86
2:B:120:ILE:HG13	2:B:150:LEU:CD2	2.04	0.86
2:B:537:PHE:CE1	2:B:545:ARG:HG2	2.08	0.86
2:B:549:LEU:CD2	2:B:611:ALA:CA	2.46	0.86
3:M:235:LEU:HD11	3:M:306:LEU:CB	2.05	0.86
1:A:179:LYS:NZ	4:S:141:VAL:CA	2.09	0.86
1:A:384:LEU:HD22	1:A:441:TYR:CD2	2.10	0.86
1:A:420:ILE:HG23	1:A:424:TYR:HB2	1.57	0.86
2:B:197:LYS:CA	2:B:229:HIS:NE2	2.38	0.86
2:B:293:VAL:O	2:B:299:LEU:CD1	2.23	0.86
2:B:375:LEU:O	2:B:377:TYR:N	2.09	0.86
2:B:549:LEU:HD22	2:B:607:ILE:O	1.74	0.86
1:A:368:ARG:NH1	1:A:419:ILE:O	2.09	0.86
1:A:595:GLU:N	2:B:476:ARG:NH2	2.23	0.86
2:B:162:VAL:HG22	2:B:199:LEU:CG	2.04	0.86
2:B:208:ILE:HD13	2:B:236:ILE:HG23	1.53	0.86
2:B:563:PHE:CE1	2:B:588:ILE:HD12	2.09	0.86
2:B:77:ILE:O	2:B:79:VAL:N	2.08	0.86
2:B:127:LEU:CD2	2:B:161:LEU:HD21	2.06	0.86
2:B:214:ALA:O	2:B:217:GLU:N	2.08	0.86
1:A:436:CYS:SG	1:A:450:TYR:CE2	2.68	0.86
1:A:536:MET:O	1:A:537:THR:C	1.94	0.86
2:B:135:ARG:NH2	2:B:164:ASP:OD1	2.08	0.86
2:B:243:TRP:CZ2	3:M:98:ARG:CD	2.59	0.86
2:B:527:PRO:CB	2:B:587:ARG:CG	2.53	0.86
1:A:129:LYS:HG2	1:A:165:ASP:OD2	1.73	0.86
1:A:183:THR:O	1:A:186:PHE:HB3	1.76	0.86
1:A:609:LEU:HG	1:A:628:VAL:CB	2.05	0.86

Continued on next page...

Continued from previous page...

Atom-1	Atom-2	Interatomic distance (Å)	Clash overlap (Å)
2:B:277:CYS:C	2:B:288:TYR:HB3	1.96	0.86
2:B:546:CYS:HB2	2:B:607:ILE:CD1	2.05	0.86
2:B:559:ASP:CB	2:B:563:PHE:CE1	2.56	0.86
2:B:351:GLU:O	3:M:49:ASP:OD2	1.94	0.86
3:M:15:ILE:O	3:M:118:TYR:HD1	1.59	0.86
1:A:114:PHE:CD2	1:A:153:ILE:HG12	2.11	0.86
2:B:38:TYR:HA	2:B:42:ILE:CA	1.91	0.86
1:A:316:LEU:CD1	1:A:348:PHE:CD2	2.58	0.85
3:M:101:LEU:HD11	3:M:106:LYS:O	1.75	0.85
4:S:109:LEU:HD12	4:S:113:PHE:CD1	2.11	0.85
1:A:609:LEU:HG	1:A:628:VAL:HB	1.57	0.85
2:B:25:VAL:HA	2:B:35:TYR:HB3	1.56	0.85
2:B:107:ARG:O	2:B:110:GLU:N	2.10	0.85
2:B:178:ILE:CG1	2:B:214:ALA:CA	2.51	0.85
2:B:268:LYS:HA	2:B:276:SER:HB2	1.58	0.85
1:A:464:ILE:O	1:A:465:SER:C	1.98	0.85
2:B:178:ILE:CG2	2:B:217:GLU:CB	2.52	0.85
2:B:556:LEU:CB	2:B:588:ILE:HD11	2.06	0.85
2:B:174:ALA:O	2:B:175:LEU:C	2.05	0.85
2:B:243:TRP:CE2	3:M:98:ARG:CD	2.55	0.85
3:M:350:VAL:HG13	3:M:442:GLN:CG	2.07	0.85
1:A:189:PHE:HD2	1:A:225:LEU:HD11	1.40	0.85
1:A:609:LEU:CG	1:A:628:VAL:HG11	2.05	0.85
2:B:83:PHE:HZ	2:B:119:SER:CB	1.87	0.85
2:B:102:HIS:ND1	2:B:137:PHE:C	2.30	0.85
2:B:161:LEU:HB3	2:B:173:VAL:CG2	2.05	0.85
2:B:352:ASN:HD21	3:M:70:ASN:CB	1.90	0.85
2:B:493:LEU:HG	2:B:511:ILE:HG23	1.58	0.85
2:B:545:ARG:HD2	2:B:602:ASP:CG	1.97	0.85
3:M:432:THR:HG1	3:M:480:GLN:HG3	1.36	0.85
2:B:24:ALA:HB3	2:B:35:TYR:CD1	1.98	0.85
2:B:161:LEU:HB3	2:B:173:VAL:HG22	1.56	0.85
2:B:256:CYS:SG	2:B:328:LEU:HD21	2.16	0.85
3:M:235:LEU:CD1	3:M:306:LEU:HB3	2.06	0.85
4:S:4:ALA:HA	4:S:18:LYS:O	1.77	0.85
2:B:151:ALA:CB	2:B:180:LEU:HD11	2.07	0.85
4:S:8:PHE:HB3	4:S:36:TYR:OH	1.77	0.85
4:S:89:VAL:HG11	4:S:98:ILE:CG1	2.06	0.85
1:A:121:LEU:HD13	1:A:155:THR:CG2	2.06	0.84
1:A:128:LEU:HD13	1:A:150:LEU:HG	1.59	0.84
1:A:189:PHE:HB3	1:A:225:LEU:CD2	2.07	0.84

Continued on next page...

Continued from previous page...

Atom-1	Atom-2	Interatomic distance (Å)	Clash overlap (Å)
2:B:103:LEU:HD21	3:M:131:ALA:O	1.77	0.84
2:B:336:ASN:HB3	2:B:339:PHE:CE1	2.12	0.84
2:B:497:LEU:CD2	2:B:533:LEU:HD21	2.07	0.84
3:M:96:ILE:CG2	3:M:125:PHE:CZ	2.60	0.84
2:B:123:LEU:HD12	2:B:142:LEU:HD23	1.58	0.84
3:M:218:LEU:O	3:M:441:GLY:N	2.10	0.84
1:A:84:MET:O	1:A:85:ALA:C	2.08	0.84
2:B:13:ASP:OD2	3:M:17:GLN:OE1	1.96	0.84
2:B:193:LEU:HD21	2:B:225:LEU:HB2	1.58	0.84
1:A:266:VAL:O	1:A:267:GLU:CB	2.26	0.84
2:B:243:TRP:CZ2	3:M:94:GLU:HG2	2.13	0.84
2:B:501:THR:C	2:B:508:ARG:NH2	2.31	0.84
2:B:136:CYS:HB3	2:B:172:GLU:CG	2.07	0.84
2:B:278:PRO:HD2	2:B:292:GLU:HG3	1.59	0.84
2:B:537:PHE:CB	2:B:598:LEU:HD13	2.02	0.84
2:B:546:CYS:HA	2:B:607:ILE:HG23	1.58	0.84
3:M:246:VAL:HB	3:M:297:PHE:CZ	2.11	0.84
4:S:46:PHE:O	4:S:48:SER:N	2.10	0.84
1:A:289:SER:CA	4:S:96:LEU:CD1	2.55	0.84
2:B:78:ASP:O	2:B:80:GLN:N	2.10	0.84
2:B:127:LEU:CB	2:B:157:THR:HG23	2.07	0.84
2:B:245:GLN:CB	2:B:309:LEU:HD11	2.07	0.84
2:B:563:PHE:O	2:B:566:ALA:HB3	1.78	0.84
3:M:245:ASP:N	3:M:472:TYR:CD1	2.45	0.84
1:A:436:CYS:SG	1:A:450:TYR:CE1	2.70	0.84
2:B:102:HIS:HE2	2:B:138:ALA:HA	1.40	0.84
1:A:174:ARG:CZ	4:S:148:ARG:HH22	1.91	0.84
2:B:143:SER:CB	2:B:179:LYS:HB2	2.08	0.84
2:B:167:ALA:O	2:B:207:VAL:CG2	2.24	0.84
2:B:276:SER:O	2:B:295:ASN:ND2	2.10	0.84
3:M:41:LEU:HD12	3:M:52:ASP:N	1.93	0.84
3:M:362:PHE:O	3:M:364:VAL:HG13	1.77	0.84
2:B:252:LEU:CB	2:B:302:PHE:CE2	2.61	0.84
2:B:418:TYR:OH	2:B:432:ALA:HB2	1.75	0.84
2:B:556:LEU:CD2	2:B:588:ILE:CG1	2.56	0.84
1:A:88:ASN:CB	1:A:120:ILE:HG23	2.08	0.83
1:A:429:VAL:HB	1:A:469:LEU:CD1	2.08	0.83
1:A:621:LEU:O	1:A:622:PRO:C	1.97	0.83
2:B:243:TRP:CH2	3:M:94:GLU:HB3	1.97	0.83
2:B:375:LEU:CD2	2:B:402:LEU:O	2.25	0.83
3:M:284:SER:O	3:M:285:PRO:C	2.09	0.83

Continued on next page...

Continued from previous page...

Atom-1	Atom-2	Interatomic distance (Å)	Clash overlap (Å)
2:B:193:LEU:HD11	2:B:225:LEU:CD2	2.04	0.83
2:B:337:THR:CB	2:B:373:LEU:HD11	2.07	0.83
2:B:549:LEU:HD22	2:B:611:ALA:CB	2.08	0.83
3:M:223:HIS:ND1	3:M:476:THR:OG1	2.01	0.83
3:M:344:ILE:HG23	3:M:347:PHE:CB	2.08	0.83
1:A:295:VAL:HG11	1:A:319:LEU:HD11	1.58	0.83
2:B:108:PHE:CZ	2:B:115:LEU:HG	2.13	0.83
2:B:151:ALA:CB	2:B:188:TYR:CE1	2.61	0.83
3:M:405:THR:C	3:M:407:THR:N	2.26	0.83
2:B:193:LEU:C	2:B:195:ILE:H	1.82	0.83
3:M:246:VAL:O	3:M:297:PHE:CE2	2.31	0.83
1:A:244:LEU:HD11	1:A:281:LEU:HD13	1.60	0.83
1:A:519:LEU:O	1:A:520:GLY:C	2.12	0.83
1:A:589:SER:O	1:A:597:GLN:NE2	2.11	0.83
2:B:20:ARG:CD	2:B:35:TYR:OH	2.21	0.83
2:B:243:TRP:HH2	3:M:94:GLU:CA	1.91	0.83
2:B:278:PRO:HA	2:B:288:TYR:HB2	1.57	0.83
1:A:203:PHE:CZ	1:A:221:VAL:HG11	2.13	0.83
1:A:215:VAL:HG11	1:A:243:ILE:CG2	2.06	0.83
1:A:346:THR:O	1:A:347:ASP:C	2.10	0.83
1:A:629:LEU:O	1:A:630:PRO:C	2.00	0.83
2:B:106:LEU:HD22	2:B:144:ASP:HB2	1.57	0.83
2:B:143:SER:HB2	2:B:179:LYS:HD2	1.60	0.83
2:B:212:VAL:HG21	2:B:248:LEU:CD2	2.08	0.83
2:B:337:THR:HA	2:B:373:LEU:CG	2.09	0.83
2:B:44:PRO:CB	2:B:82:TYR:OH	2.26	0.83
2:B:170:ARG:HA	2:B:199:LEU:CD2	2.09	0.83
1:A:533:ILE:CG1	1:A:562:TRP:CH2	2.60	0.83
2:B:501:THR:C	2:B:508:ARG:HH21	1.82	0.83
4:S:25:LEU:O	4:S:26:PRO:C	2.15	0.83
1:A:64:LEU:O	1:A:65:ASN:C	2.14	0.83
1:A:100:LEU:O	1:A:101:GLN:C	2.04	0.83
1:A:638:LEU:HD11	2:B:520:SER:N	1.91	0.83
2:B:106:LEU:HD13	2:B:144:ASP:CB	2.07	0.83
2:B:337:THR:CA	2:B:373:LEU:HD11	2.09	0.83
2:B:418:TYR:C	2:B:418:TYR:HD1	1.77	0.83
2:B:568:VAL:O	2:B:571:SER:CB	2.26	0.83
1:A:101:GLN:CD	4:S:167:ILE:CD1	2.48	0.83
2:B:158:VAL:HG13	2:B:173:VAL:CG1	2.08	0.83
2:B:245:GLN:HB3	2:B:309:LEU:CD1	2.08	0.83
2:B:279:LEU:HG	2:B:288:TYR:HD1	1.42	0.83

Continued on next page...

Continued from previous page...

Atom-1	Atom-2	Interatomic distance (Å)	Clash overlap (Å)
3:M:326:HIS:O	3:M:338:PHE:HA	1.79	0.83
1:A:298:ILE:HD11	1:A:311:THR:HG21	1.59	0.82
2:B:193:LEU:O	2:B:195:ILE:N	2.11	0.82
3:M:245:ASP:HB3	3:M:472:TYR:HD1	1.41	0.82
4:S:43:ASN:O	4:S:44:SER:C	2.15	0.82
2:B:216:LYS:HB2	2:B:251:LEU:CD1	2.05	0.82
3:M:16:PHE:HE1	3:M:122:SER:HB2	1.44	0.82
1:A:250:ASN:OD1	1:A:285:THR:HB	1.79	0.82
2:B:20:ARG:NE	2:B:21:GLU:HB2	1.94	0.82
2:B:560:ILE:HG23	2:B:564:LYS:HB2	1.62	0.82
2:B:139:LEU:HG	2:B:176:ALA:CB	2.09	0.82
3:M:338:PHE:CD2	3:M:415:ILE:HG13	2.14	0.82
2:B:132:SER:HA	2:B:169:VAL:CG2	2.09	0.82
2:B:519:ALA:O	2:B:523:PHE:CD2	2.32	0.82
2:B:556:LEU:CD2	2:B:588:ILE:HG12	2.09	0.82
3:M:244:VAL:HA	3:M:472:TYR:CE2	2.14	0.82
4:S:16:LEU:HD13	4:S:125:TRP:CD1	2.11	0.82
1:A:638:LEU:CG	2:B:520:SER:CA	2.55	0.82
2:B:24:ALA:HB2	2:B:35:TYR:CE1	2.15	0.82
2:B:136:CYS:SG	2:B:168:MET:HG2	2.19	0.82
1:A:225:LEU:HB2	1:A:233:PHE:CZ	2.14	0.82
1:A:465:SER:N	2:B:1:MET:HE1	1.93	0.82
2:B:545:ARG:HD2	2:B:602:ASP:OD2	1.80	0.82
2:B:567:GLN:O	2:B:569:THR:CA	2.27	0.82
3:M:323:MET:SD	3:M:342:LEU:HG	2.19	0.82
4:S:48:SER:OG	4:S:50:PHE:N	2.12	0.82
1:A:204:VAL:O	1:A:205:SER:C	2.09	0.82
1:A:581:LEU:HD23	1:A:607:LEU:CD2	2.09	0.82
2:B:185:LYS:NZ	2:B:221:ASP:OD2	2.13	0.82
2:B:366:LEU:O	2:B:367:SER:C	1.98	0.82
2:B:486:HIS:CE1	2:B:518:ILE:CB	2.63	0.82
2:B:568:VAL:HG12	2:B:571:SER:OG	1.79	0.82
4:S:75:ILE:CG2	4:S:77:TYR:CE1	2.63	0.82
1:A:179:LYS:HZ1	4:S:141:VAL:CA	1.78	0.82
2:B:70:MET:HE1	2:B:104:TYR:O	1.79	0.82
2:B:79:VAL:HG23	2:B:108:PHE:CZ	2.15	0.82
2:B:219:TYR:CE2	2:B:226:LEU:CB	2.59	0.82
2:B:227:HIS:C	2:B:229:HIS:H	1.83	0.82
2:B:227:HIS:CE1	2:B:292:GLU:CG	2.62	0.82
2:B:297:PRO:O	2:B:301:LEU:CD1	2.28	0.82
3:M:99:ILE:HG21	3:M:124:ILE:CG2	2.10	0.82

Continued on next page...

Continued from previous page...

Atom-1	Atom-2	Interatomic distance (Å)	Clash overlap (Å)
1:A:219:VAL:CG2	1:A:256:LEU:HD21	2.09	0.82
2:B:127:LEU:HD22	2:B:161:LEU:CD2	2.09	0.82
3:M:258:VAL:HG13	3:M:449:VAL:HG13	1.62	0.82
3:M:293:PRO:O	3:M:293:PRO:CD	2.28	0.82
1:A:589:SER:O	1:A:597:GLN:CG	2.28	0.81
2:B:79:VAL:HG23	2:B:108:PHE:CE1	2.15	0.81
2:B:127:LEU:CD1	2:B:157:THR:CG2	2.20	0.81
2:B:193:LEU:CD1	2:B:225:LEU:HD21	1.97	0.81
2:B:123:LEU:CD1	2:B:142:LEU:CD2	2.57	0.81
2:B:219:TYR:HB3	2:B:223:LEU:CD2	2.10	0.81
2:B:247:TYR:OH	3:M:137:SER:OG	1.95	0.81
2:B:278:PRO:CA	2:B:288:TYR:HB2	2.09	0.81
2:B:545:ARG:HD3	2:B:602:ASP:CB	2.07	0.81
3:M:68:VAL:HA	3:M:76:CYS:O	1.79	0.81
3:M:131:ALA:CB	3:M:131:ALA:C	2.46	0.81
1:A:163:ALA:CB	1:A:199:ASN:HD21	1.86	0.81
1:A:219:VAL:HG11	1:A:256:LEU:CD2	2.10	0.81
1:A:258:LYS:HZ3	4:S:94:SER:HB3	1.42	0.81
1:A:264:SER:HB2	1:A:271:ARG:CD	2.10	0.81
1:A:273:LYS:O	1:A:276:PRO:HD2	1.79	0.81
1:A:316:LEU:HD11	1:A:341:ILE:HG21	1.61	0.81
2:B:36:THR:CG2	2:B:40:GLN:HG3	2.10	0.81
2:B:98:LYS:HZ1	2:B:134:LEU:CB	1.90	0.81
2:B:124:GLN:OE1	2:B:153:ILE:HG23	1.80	0.81
2:B:181:TYR:HE1	2:B:185:LYS:HG3	1.41	0.81
3:M:16:PHE:HA	3:M:118:TYR:HE1	1.36	0.81
4:S:10:LYS:HA	4:S:84:TYR:CE1	2.15	0.81
1:A:605:GLU:OE1	1:A:636:TYR:OH	1.97	0.81
2:B:140:SER:HB2	2:B:172:GLU:CD	2.01	0.81
2:B:279:LEU:HD12	2:B:285:GLU:HG2	1.60	0.81
2:B:367:SER:OG	2:B:401:THR:CB	2.29	0.81
2:B:542:PRO:O	2:B:607:ILE:CD1	2.29	0.81
2:B:563:PHE:O	2:B:567:GLN:N	2.12	0.81
1:A:391:LEU:O	1:A:392:MET:C	2.00	0.81
2:B:158:VAL:CG1	2:B:177:ILE:HG12	1.93	0.81
1:A:633:PHE:HZ	2:B:550:VAL:HG12	1.43	0.81
2:B:236:ILE:HG22	2:B:240:LEU:CD1	2.09	0.81
1:A:204:VAL:HG13	1:A:239:LEU:HD13	1.63	0.81
2:B:47:LEU:HD22	2:B:66:ILE:CG1	2.10	0.81
2:B:120:ILE:HD11	2:B:150:LEU:HD13	1.60	0.81
2:B:245:GLN:OE1	2:B:309:LEU:HD12	1.81	0.81

Continued on next page...

Continued from previous page...

Atom-1	Atom-2	Interatomic distance (Å)	Clash overlap (Å)
2:B:479:VAL:CG1	2:B:486:HIS:CD2	2.64	0.81
2:B:494:ALA:HB2	2:B:515:PHE:CE2	2.16	0.81
1:A:282:MET:O	1:A:283:GLU:C	2.18	0.81
2:B:232:ARG:HG3	2:B:236:ILE:HD11	1.62	0.81
2:B:278:PRO:CD	2:B:289:PRO:O	2.28	0.81
2:B:155:LEU:HB2	2:B:188:TYR:CD2	2.15	0.81
2:B:162:VAL:HG23	2:B:173:VAL:HG11	1.61	0.81
2:B:347:VAL:HG22	2:B:359:LEU:CB	2.10	0.81
2:B:566:ALA:O	2:B:574:ASN:HB2	1.81	0.81
3:M:100:LEU:O	3:M:103:TYR:O	1.98	0.81
1:A:216:SER:HB2	1:A:252:ILE:HG12	1.61	0.80
1:A:244:LEU:CG	1:A:281:LEU:HD11	2.09	0.80
1:A:332:TYR:CD1	1:A:366:SER:OG	2.33	0.80
1:A:412:LYS:O	1:A:413:SER:C	2.19	0.80
3:M:131:ALA:CB	3:M:131:ALA:N	2.42	0.80
3:M:215:TYR:CG	3:M:468:LYS:HA	2.16	0.80
1:A:125:THR:CB	1:A:158:LEU:HD13	2.11	0.80
2:B:268:LYS:O	2:B:273:SER:OG	2.00	0.80
1:A:630:PRO:HG2	2:B:617:LEU:HD13	0.80	0.80
1:A:633:PHE:CZ	2:B:550:VAL:CG1	2.64	0.80
2:B:42:ILE:O	2:B:43:ASN:HB2	1.82	0.80
3:M:48:ASP:C	3:M:75:TRP:CH2	2.54	0.80
3:M:405:THR:O	3:M:407:THR:HG23	1.82	0.80
1:A:189:PHE:HB2	1:A:225:LEU:CD2	2.08	0.80
1:A:264:SER:CB	1:A:271:ARG:CG	2.60	0.80
1:A:276:PRO:O	1:A:278:ILE:O	1.99	0.80
1:A:528:ASN:O	1:A:529:GLY:C	2.10	0.80
2:B:62:ALA:O	2:B:66:ILE:HG13	1.80	0.80
2:B:120:ILE:HA	2:B:142:LEU:HD21	1.61	0.80
3:M:48:ASP:HA	3:M:75:TRP:HZ2	1.45	0.80
1:A:84:MET:SD	1:A:113:SER:CA	2.66	0.80
2:B:567:GLN:C	2:B:569:THR:OG1	2.20	0.80
3:M:282:VAL:N	3:M:282:VAL:C	2.34	0.80
1:A:125:THR:HG1	1:A:158:LEU:HD13	0.99	0.80
1:A:275:LEU:CD2	1:A:311:THR:OG1	2.27	0.80
1:A:581:LEU:HD23	1:A:607:LEU:HD11	1.63	0.80
4:S:131:VAL:CG2	4:S:153:VAL:HG22	2.11	0.80
1:A:258:LYS:NZ	4:S:94:SER:N	2.29	0.80
1:A:504:ILE:O	1:A:505:ASN:C	2.06	0.80
1:A:609:LEU:HG	1:A:628:VAL:HG11	1.64	0.80
2:B:280:PRO:HG3	2:B:283:TYR:CE1	2.15	0.80

Continued on next page...

Continued from previous page...

Atom-1	Atom-2	Interatomic distance (Å)	Clash overlap (Å)
2:B:377:TYR:O	2:B:380:LYS:HB2	1.81	0.80
2:B:549:LEU:CD1	2:B:611:ALA:CB	2.59	0.80
3:M:99:ILE:HG21	3:M:124:ILE:HG23	1.62	0.80
3:M:242:GLY:CA	3:M:444:ALA:HB2	2.11	0.80
3:M:244:VAL:HB	3:M:300:LEU:HG	1.63	0.80
1:A:323:CYS:CB	1:A:355:LEU:HD21	2.12	0.80
2:B:36:THR:O	2:B:40:GLN:N	2.14	0.80
2:B:387:ASP:HB3	2:B:391:ALA:HB3	1.62	0.80
2:B:193:LEU:CB	2:B:225:LEU:HG	2.12	0.80
2:B:140:SER:CB	2:B:172:GLU:OE1	2.27	0.79
2:B:188:TYR:O	2:B:192:LEU:HD13	1.79	0.79
2:B:522:GLU:O	2:B:522:GLU:HG2	1.79	0.79
3:M:351:SER:OG	3:M:441:GLY:O	2.01	0.79
2:B:227:HIS:CE1	2:B:292:GLU:HG2	2.16	0.79
2:B:451:MET:SD	2:B:489:ILE:HG12	2.22	0.79
3:M:383:HIS:CG	3:M:403:THR:OG1	2.34	0.79
1:A:179:LYS:HB2	4:S:142:ILE:HB	1.64	0.79
2:B:285:GLU:O	2:B:286:ILE:C	2.20	0.79
1:A:200:PHE:CZ	1:A:236:LEU:CG	2.64	0.79
1:A:215:VAL:CG1	1:A:243:ILE:HG23	2.07	0.79
1:A:552:ILE:O	1:A:556:VAL:HG23	1.83	0.79
2:B:20:ARG:NH1	2:B:21:GLU:HG2	1.97	0.79
2:B:588:ILE:HG23	2:B:618:PHE:HZ	1.47	0.79
3:M:306:LEU:CD1	3:M:317:MET:HE1	2.12	0.79
3:M:317:MET:HB3	3:M:320:ILE:O	1.83	0.79
3:M:350:VAL:CG2	3:M:442:GLN:HG2	2.11	0.79
1:A:95:MET:SD	1:A:107:TYR:CD2	2.76	0.79
1:A:353:ASP:OD1	1:A:378:ILE:HD12	1.82	0.79
1:A:516:ILE:HG22	1:A:554:ALA:HB2	1.64	0.79
2:B:70:MET:HE1	2:B:107:ARG:CG	2.08	0.79
2:B:116:THR:O	2:B:120:ILE:HG12	1.83	0.79
2:B:120:ILE:CG1	2:B:150:LEU:HD22	2.10	0.79
2:B:328:LEU:HB2	2:B:333:GLN:HE22	1.46	0.79
2:B:567:GLN:CA	2:B:569:THR:OG1	2.30	0.79
3:M:245:ASP:N	3:M:472:TYR:CG	2.49	0.79
1:A:401:VAL:HG23	1:A:418:ILE:O	1.81	0.79
2:B:170:ARG:NH1	2:B:198:GLU:HG2	1.96	0.79
2:B:566:ALA:C	2:B:574:ASN:HD22	1.85	0.79
1:A:180:LYS:HZ3	4:S:137:GLN:CB	1.90	0.79
1:A:244:LEU:CD1	1:A:281:LEU:HD11	2.12	0.79
1:A:404:GLN:HB3	2:B:7:ARG:HH21	1.41	0.79

Continued on next page...

Continued from previous page...

Atom-1	Atom-2	Interatomic distance (Å)	Clash overlap (Å)
1:A:520:GLY:HA2	1:A:558:VAL:HG22	1.64	0.79
2:B:197:LYS:CA	2:B:229:HIS:CD2	2.65	0.79
2:B:396:ILE:HG21	2:B:432:ALA:HA	1.62	0.79
1:A:240:LEU:O	1:A:241:TYR:C	2.07	0.79
2:B:247:TYR:HH	3:M:137:SER:HB3	1.48	0.79
2:B:513:TRP:O	2:B:551:LEU:HD22	1.83	0.79
2:B:534:ILE:O	2:B:598:LEU:HD11	1.83	0.79
3:M:220:GLU:OE2	3:M:439:TYR:CD1	2.36	0.79
2:B:267:ASP:H	2:B:289:PRO:CG	1.95	0.79
2:B:37:TYR:HD2	2:B:38:TYR:CD1	2.00	0.79
2:B:178:ILE:CD1	2:B:218:CYS:H	1.95	0.79
2:B:490:ILE:HG13	2:B:518:ILE:CG2	2.12	0.79
1:A:332:TYR:CE1	1:A:366:SER:HB2	2.18	0.78
2:B:566:ALA:HB2	2:B:581:TYR:CD2	2.17	0.78
3:M:380:ARG:O	3:M:411:LEU:HA	1.81	0.78
1:A:581:LEU:CD2	1:A:607:LEU:HD11	2.13	0.78
2:B:280:PRO:HG3	2:B:283:TYR:CD1	2.17	0.78
1:A:114:PHE:HE1	1:A:154:ILE:HG12	1.46	0.78
1:A:264:SER:HB2	1:A:271:ARG:HD3	1.63	0.78
1:A:320:HIS:O	1:A:321:THR:C	2.18	0.78
2:B:103:LEU:HD22	3:M:131:ALA:HA	1.65	0.78
2:B:534:ILE:HD11	2:B:595:VAL:HG23	1.63	0.78
3:M:338:PHE:CE2	3:M:415:ILE:HG13	2.18	0.78
1:A:186:PHE:CZ	1:A:224:GLU:CG	2.63	0.78
1:A:298:ILE:O	1:A:299:VAL:C	2.19	0.78
2:B:63:MET:HE2	2:B:104:TYR:HB2	1.66	0.78
2:B:123:LEU:CD1	2:B:142:LEU:CG	2.58	0.78
2:B:337:THR:CA	2:B:373:LEU:CD2	2.50	0.78
4:S:164:ASP:HA	4:S:167:ILE:HB	1.66	0.78
1:A:450:TYR:OH	1:A:476:GLN:NE2	2.16	0.78
2:B:25:VAL:HB	2:B:36:THR:OG1	1.83	0.78
2:B:139:LEU:CG	2:B:176:ALA:CB	2.61	0.78
3:M:360:LEU:HD23	3:M:362:PHE:CZ	2.18	0.78
3:M:383:HIS:CB	3:M:403:THR:OG1	2.31	0.78
2:B:63:MET:HG3	2:B:100:LEU:HB3	1.64	0.78
2:B:196:LEU:HB3	2:B:215:TYR:CZ	2.18	0.78
2:B:458:MET:O	2:B:459:GLU:C	2.22	0.78
2:B:478:LEU:O	2:B:479:VAL:C	2.12	0.78
1:A:121:LEU:HD11	1:A:155:THR:H	1.49	0.78
1:A:189:PHE:HB3	1:A:225:LEU:HD22	1.65	0.78
1:A:222:ILE:CG2	1:A:240:LEU:HD11	2.13	0.78

Continued on next page...

Continued from previous page...

Atom-1	Atom-2	Interatomic distance (Å)	Clash overlap (Å)
2:B:136:CYS:HB3	2:B:172:GLU:HG3	1.66	0.78
2:B:143:SER:CB	2:B:179:LYS:CD	2.60	0.78
2:B:374:PHE:HD2	2:B:402:LEU:CD1	1.91	0.78
2:B:537:PHE:CZ	2:B:545:ARG:HG2	2.18	0.78
1:A:114:PHE:HZ	1:A:152:THR:O	1.67	0.78
2:B:106:LEU:CD1	2:B:144:ASP:HB3	2.14	0.78
1:A:114:PHE:CE2	1:A:153:ILE:HA	2.18	0.78
1:A:373:GLU:CG	1:A:427:LYS:HE2	2.13	0.78
2:B:393:ILE:CG2	2:B:431:MET:HG2	2.13	0.78
2:B:162:VAL:CG2	2:B:199:LEU:HD11	2.13	0.78
3:M:247:ARG:H	3:M:470:ALA:HB2	1.49	0.78
3:M:271:SER:HB3	3:M:301:GLU:HG3	1.64	0.78
1:A:176:TYR:HE1	4:S:148:ARG:NH2	1.81	0.77
2:B:21:GLU:HA	2:B:24:ALA:CB	2.14	0.77
2:B:79:VAL:HB	2:B:108:PHE:CE1	2.19	0.77
2:B:293:VAL:O	2:B:299:LEU:CB	2.32	0.77
2:B:546:CYS:CA	2:B:607:ILE:CG1	2.55	0.77
2:B:553:ALA:HB1	2:B:614:ILE:HG12	1.65	0.77
4:S:5:VAL:O	4:S:17:VAL:HA	1.83	0.77
1:A:185:LEU:HD13	1:A:203:PHE:CE1	2.19	0.77
2:B:215:TYR:HD1	2:B:233:TYR:HE1	0.90	0.77
4:S:3:HIS:NE2	4:S:90:ASP:OD2	2.18	0.77
1:A:225:LEU:HD12	1:A:233:PHE:CE2	2.19	0.77
2:B:70:MET:CE	2:B:107:ARG:HB3	2.14	0.77
2:B:497:LEU:O	2:B:499:VAL:N	2.17	0.77
2:B:523:PHE:CG	2:B:559:ASP:OD1	2.37	0.77
3:M:323:MET:HB3	3:M:340:LEU:HD11	1.65	0.77
4:S:28:GLN:O	4:S:32:LEU:HG	1.85	0.77
4:S:64:ASN:C	4:S:66:ASP:H	1.86	0.77
2:B:20:ARG:NH1	2:B:21:GLU:CG	2.47	0.77
2:B:127:LEU:HB3	2:B:161:LEU:HD11	1.64	0.77
2:B:316:THR:OG1	3:M:90:PHE:HE2	1.58	0.77
3:M:375:LYS:N	3:M:416:GLU:O	2.16	0.77
4:S:8:PHE:CD2	4:S:8:PHE:N	2.50	0.77
1:A:605:GLU:OE2	1:A:608:ARG:NH2	2.17	0.77
2:B:66:ILE:HG22	2:B:104:TYR:CE1	2.19	0.77
2:B:451:MET:HG3	2:B:489:ILE:CD1	2.15	0.77
2:B:486:HIS:NE2	2:B:518:ILE:CB	2.46	0.77
4:S:39:ILE:HG23	4:S:47:GLN:OE1	1.85	0.77
1:A:638:LEU:HB2	2:B:558:TYR:CE1	2.10	0.77
2:B:20:ARG:CZ	2:B:21:GLU:CG	2.62	0.77

Continued on next page...

Continued from previous page...

Atom-1	Atom-2	Interatomic distance (Å)	Clash overlap (Å)
2:B:172:GLU:O	2:B:173:VAL:C	2.09	0.77
2:B:227:HIS:C	2:B:229:HIS:N	2.36	0.77
2:B:256:CYS:CB	2:B:328:LEU:CD2	2.62	0.77
2:B:261:PRO:HD2	2:B:293:VAL:HG23	1.65	0.77
1:A:436:CYS:CB	1:A:450:TYR:CE1	2.67	0.77
2:B:35:TYR:O	2:B:39:SER:HB3	1.84	0.77
2:B:310:ILE:HG12	2:B:318:ILE:HA	1.65	0.77
2:B:490:ILE:CD1	2:B:518:ILE:HG21	2.14	0.77
2:B:523:PHE:HE1	2:B:580:TYR:CB	1.85	0.77
2:B:556:LEU:HD22	2:B:588:ILE:CG1	2.13	0.77
3:M:323:MET:CE	3:M:342:LEU:HB3	2.15	0.77
2:B:36:THR:HG23	2:B:40:GLN:HG3	1.66	0.77
2:B:174:ALA:HB3	2:B:211:ALA:HA	1.67	0.77
2:B:534:ILE:CD1	2:B:595:VAL:HG23	2.15	0.77
1:A:84:MET:HB3	1:A:113:SER:HB2	1.66	0.77
1:A:320:HIS:HB2	1:A:352:PHE:CE2	2.20	0.77
1:A:384:LEU:HD22	1:A:441:TYR:CE2	2.20	0.77
1:A:403:LEU:O	2:B:3:ASP:CG	2.16	0.77
1:A:545:HIS:O	1:A:546:SER:C	2.15	0.77
2:B:21:GLU:OE2	2:B:35:TYR:CD2	2.37	0.77
2:B:79:VAL:CG2	2:B:108:PHE:CE1	2.67	0.77
2:B:316:THR:HG21	3:M:90:PHE:CE2	2.18	0.77
3:M:374:TYR:O	3:M:390:ILE:HD12	1.85	0.77
1:A:566:PHE:HZ	1:A:618:THR:O	1.67	0.77
2:B:37:TYR:CD2	2:B:42:ILE:HA	2.20	0.77
2:B:186:ASN:C	2:B:188:TYR:N	2.36	0.77
2:B:293:VAL:C	2:B:299:LEU:HG	2.05	0.77
1:A:215:VAL:HG13	1:A:243:ILE:CG2	2.08	0.76
2:B:70:MET:HE3	2:B:107:ARG:CG	2.13	0.76
2:B:123:LEU:HD12	2:B:142:LEU:HD21	1.66	0.76
2:B:127:LEU:HD23	2:B:161:LEU:HD21	1.66	0.76
2:B:345:ARG:HH12	3:M:305:ASP:CG	1.88	0.76
2:B:28:SER:H	2:B:32:GLU:HB3	1.49	0.76
2:B:28:SER:N	2:B:32:GLU:HB3	1.82	0.76
4:S:130:SER:OG	4:S:156:LEU:CD1	2.33	0.76
1:A:404:GLN:CB	2:B:7:ARG:NH2	2.44	0.76
1:A:595:GLU:CG	2:B:473:ASN:OD1	2.33	0.76
2:B:307:ASN:OD1	2:B:339:PHE:HD2	1.67	0.76
2:B:374:PHE:CE1	2:B:381:PHE:CE1	2.73	0.76
3:M:48:ASP:O	3:M:75:TRP:CH2	2.39	0.76
1:A:332:TYR:HE1	1:A:366:SER:HB2	1.50	0.76

Continued on next page...

Continued from previous page...

Atom-1	Atom-2	Interatomic distance (Å)	Clash overlap (Å)
1:A:429:VAL:CG1	1:A:469:LEU:HD11	2.14	0.76
2:B:106:LEU:CD1	2:B:144:ASP:CB	2.63	0.76
2:B:505:ASP:HA	2:B:544:THR:OG1	1.86	0.76
2:B:513:TRP:HA	2:B:551:LEU:HD21	1.61	0.76
3:M:241:HIS:HB2	3:M:476:THR:CG2	2.16	0.76
3:M:243:ILE:H	3:M:474:THR:HG22	1.51	0.76
2:B:143:SER:HB2	2:B:179:LYS:CG	2.15	0.76
2:B:245:GLN:CB	2:B:309:LEU:CD1	2.64	0.76
2:B:278:PRO:HD3	2:B:292:GLU:HB2	1.67	0.76
1:A:174:ARG:NH2	4:S:148:ARG:NH2	2.34	0.76
1:A:539:ASN:O	1:A:540:ILE:C	2.18	0.76
1:A:585:PHE:CE2	1:A:603:VAL:CG1	2.68	0.76
2:B:178:ILE:HD12	2:B:218:CYS:N	1.96	0.76
2:B:208:ILE:CD1	2:B:236:ILE:HD13	2.15	0.76
3:M:225:VAL:HB	3:M:237:THR:OG1	1.85	0.76
3:M:306:LEU:CD1	3:M:317:MET:CE	2.63	0.76
4:S:8:PHE:CE1	4:S:84:TYR:CB	2.69	0.76
1:A:638:LEU:HD21	2:B:558:TYR:HB3	1.62	0.76
2:B:16:LYS:CD	3:M:119:ASP:OD1	2.27	0.76
2:B:20:ARG:NH1	2:B:35:TYR:HE1	1.84	0.76
2:B:523:PHE:CE1	2:B:580:TYR:HB3	2.19	0.76
3:M:350:VAL:HG13	3:M:442:GLN:HG2	1.67	0.76
1:A:189:PHE:CD2	1:A:225:LEU:HD11	2.20	0.76
1:A:408:ILE:HG22	4:S:65:ASN:N	2.01	0.76
2:B:29:LYS:HE2	2:B:30:LEU:CA	2.14	0.76
3:M:131:ALA:C	3:M:133:GLU:H	1.89	0.76
4:S:53:THR:HB	4:S:69:ASN:CB	2.16	0.76
2:B:178:ILE:HD11	2:B:215:TYR:HA	1.68	0.76
2:B:500:GLN:CB	2:B:503:LEU:HG	2.16	0.76
2:B:560:ILE:HA	2:B:563:PHE:HB2	1.67	0.76
3:M:70:ASN:HA	3:M:74:TYR:O	1.86	0.76
3:M:245:ASP:C	3:M:472:TYR:CE1	2.59	0.76
2:B:120:ILE:HG23	2:B:142:LEU:HD22	1.68	0.76
2:B:219:TYR:CE1	2:B:226:LEU:CA	2.66	0.76
2:B:475:ILE:HG23	2:B:489:ILE:HG21	1.68	0.76
1:A:163:ALA:O	1:A:164:ASP:C	2.17	0.75
1:A:264:SER:HB2	1:A:271:ARG:CG	2.16	0.75
1:A:322:PHE:CD2	1:A:330:LEU:HD21	2.22	0.75
2:B:170:ARG:HG2	2:B:199:LEU:HD23	1.66	0.75
2:B:243:TRP:CE2	3:M:98:ARG:NE	2.54	0.75
2:B:261:PRO:CB	2:B:290:SER:HB3	2.15	0.75

Continued on next page...

Continued from previous page...

Atom-1	Atom-2	Interatomic distance (Å)	Clash overlap (Å)
2:B:515:PHE:CD2	2:B:529:VAL:HG11	2.21	0.75
4:S:38:LEU:HD23	4:S:68:VAL:HG21	1.68	0.75
2:B:212:VAL:HG23	2:B:233:TYR:CE2	2.21	0.75
2:B:487:LEU:CD2	2:B:522:GLU:HB3	2.17	0.75
4:S:10:LYS:HA	4:S:84:TYR:HE1	1.49	0.75
1:A:180:LYS:HZ3	4:S:137:GLN:HB3	1.48	0.75
1:A:182:ILE:HG22	1:A:221:VAL:HG21	1.68	0.75
1:A:263:LEU:O	1:A:266:VAL:O	2.04	0.75
2:B:63:MET:CG	2:B:100:LEU:HB3	2.16	0.75
2:B:143:SER:C	2:B:179:LYS:HD2	2.05	0.75
2:B:267:ASP:HB3	2:B:289:PRO:HD3	1.67	0.75
2:B:278:PRO:CG	2:B:292:GLU:OE1	2.33	0.75
2:B:476:ARG:HA	2:B:514:LEU:CD1	2.15	0.75
3:M:16:PHE:CA	3:M:118:TYR:CE1	2.64	0.75
3:M:437:TYR:N	3:M:437:TYR:CD1	2.53	0.75
1:A:373:GLU:HG3	1:A:427:LYS:HE2	1.66	0.75
2:B:109:ALA:O	2:B:110:GLU:C	2.17	0.75
3:M:6:TYR:HA	3:M:16:PHE:O	1.86	0.75
2:B:70:MET:CE	2:B:107:ARG:HG3	2.14	0.75
2:B:352:ASN:CG	3:M:49:ASP:HA	2.07	0.75
3:M:317:MET:O	3:M:322:LEU:HB3	1.86	0.75
4:S:32:LEU:O	4:S:35:VAL:HG22	1.87	0.75
4:S:71:GLU:O	4:S:73:ILE:N	2.20	0.75
1:A:200:PHE:CE1	1:A:236:LEU:HG	2.21	0.75
2:B:280:PRO:HG2	2:B:283:TYR:CG	2.21	0.75
2:B:297:PRO:O	2:B:301:LEU:HD12	1.86	0.75
2:B:549:LEU:HD13	2:B:611:ALA:HB2	1.69	0.75
2:B:563:PHE:CD2	2:B:584:SER:CB	2.67	0.75
3:M:421:GLY:O	3:M:422:PRO:C	2.22	0.75
1:A:225:LEU:HD12	1:A:233:PHE:CZ	2.22	0.75
2:B:296:ASP:OD1	2:B:297:PRO:HD2	1.86	0.75
2:B:418:TYR:CD1	2:B:424:PHE:CD1	2.74	0.75
3:M:290:PHE:CZ	3:M:293:PRO:HD3	2.21	0.75
2:B:315:PRO:HA	2:B:318:ILE:HD12	1.69	0.75
2:B:487:LEU:HD22	2:B:522:GLU:HB3	1.68	0.75
2:B:556:LEU:HD23	2:B:588:ILE:CG1	2.17	0.75
2:B:132:SER:HB2	2:B:169:VAL:CG2	2.17	0.75
2:B:167:ALA:CB	2:B:202:ASP:OD1	2.35	0.75
2:B:236:ILE:CG2	2:B:240:LEU:HD11	2.13	0.75
2:B:344:VAL:HG13	2:B:381:PHE:CE1	2.20	0.74
2:B:513:TRP:CG	2:B:551:LEU:HD21	2.21	0.74

Continued on next page...

Continued from previous page...

Atom-1	Atom-2	Interatomic distance (Å)	Clash overlap (Å)
2:B:531:ARG:HA	2:B:591:MET:SD	2.27	0.74
3:M:55:MET:O	3:M:56:VAL:C	2.19	0.74
1:A:186:PHE:CD1	1:A:224:GLU:HB3	2.22	0.74
1:A:462:GLN:O	1:A:463:ASP:C	2.25	0.74
2:B:530:LEU:HG	2:B:591:MET:HB3	1.67	0.74
2:B:278:PRO:C	2:B:288:TYR:CB	2.55	0.74
2:B:310:ILE:CG2	2:B:342:ALA:HB1	2.18	0.74
2:B:351:GLU:OE2	3:M:476:THR:O	2.05	0.74
1:A:107:TYR:CD2	1:A:128:LEU:HD21	2.23	0.74
1:A:215:VAL:O	1:A:216:SER:C	2.22	0.74
3:M:223:HIS:CG	3:M:478:ASN:HA	2.21	0.74
4:S:1:MET:H2	4:S:93:GLU:HB2	1.51	0.74
1:A:304:LEU:HA	1:A:308:ASP:HB2	1.70	0.74
2:B:29:LYS:HE2	2:B:30:LEU:H	0.72	0.74
2:B:219:TYR:O	2:B:220:ALA:C	2.23	0.74
2:B:317:VAL:O	2:B:321:CYS:SG	2.46	0.74
2:B:549:LEU:CD1	2:B:611:ALA:HB2	2.16	0.74
2:B:556:LEU:HD23	2:B:588:ILE:HG13	1.69	0.74
2:B:566:ALA:CA	2:B:574:ASN:ND2	2.51	0.74
4:S:98:ILE:O	4:S:102:ILE:HG13	1.87	0.74
1:A:158:LEU:HG	1:A:162:ILE:CD1	2.17	0.74
1:A:101:GLN:HE22	4:S:167:ILE:HD12	1.52	0.74
1:A:121:LEU:HD21	1:A:158:LEU:CB	2.06	0.74
1:A:196:LEU:O	1:A:197:ARG:C	2.18	0.74
1:A:294:SER:O	1:A:298:ILE:HG12	1.88	0.74
1:A:585:PHE:CE2	1:A:603:VAL:HG11	2.23	0.74
1:A:264:SER:HB3	1:A:271:ARG:CG	2.17	0.74
1:A:328:PRO:O	1:A:329:ASN:C	2.16	0.74
2:B:108:PHE:CE2	2:B:112:ASP:HB3	2.23	0.74
2:B:212:VAL:CG2	2:B:248:LEU:CD2	2.65	0.74
2:B:400:SER:HB3	2:B:435:SER:HB3	1.68	0.74
3:M:260:LEU:CD2	3:M:449:VAL:HG22	2.18	0.74
3:M:267:ILE:HD12	3:M:445:SER:OG	1.88	0.74
3:M:281:GLY:C	3:M:281:GLY:N	2.41	0.74
1:A:196:LEU:O	1:A:196:LEU:HD22	1.87	0.74
1:A:316:LEU:HD13	1:A:348:PHE:CG	2.23	0.74
2:B:279:LEU:H	2:B:288:TYR:CB	1.91	0.74
2:B:393:ILE:HG23	2:B:431:MET:CG	2.17	0.74
1:A:88:ASN:O	1:A:89:PHE:C	2.14	0.74
1:A:154:ILE:CG2	1:A:191:GLN:CG	2.65	0.74
1:A:281:LEU:O	1:A:282:MET:C	2.17	0.74

Continued on next page...

Continued from previous page...

Atom-1	Atom-2	Interatomic distance (Å)	Clash overlap (Å)
1:A:436:CYS:HB2	1:A:450:TYR:CE1	2.23	0.74
2:B:237:ILE:HB	2:B:248:LEU:HD13	1.70	0.74
2:B:526:CYS:N	2:B:527:PRO:CD	2.49	0.74
2:B:563:PHE:HD2	2:B:584:SER:HB2	1.52	0.74
3:M:347:PHE:CD1	3:M:350:VAL:CB	2.70	0.74
4:S:8:PHE:HE2	4:S:86:THR:N	1.85	0.74
1:A:638:LEU:CD1	2:B:520:SER:HA	2.09	0.73
2:B:70:MET:HE3	2:B:107:ARG:HG3	1.69	0.73
2:B:316:THR:HG21	3:M:90:PHE:CD2	2.21	0.73
3:M:121:ILE:O	3:M:125:PHE:CD1	2.41	0.73
3:M:379:LEU:HD23	3:M:411:LEU:CG	2.16	0.73
4:S:53:THR:OG1	4:S:68:VAL:CA	2.35	0.73
1:A:393:LYS:NZ	1:A:397:ASP:OD2	2.21	0.73
1:A:399:ASP:O	1:A:420:ILE:HB	1.88	0.73
2:B:162:VAL:HG22	2:B:199:LEU:HG	1.69	0.73
2:B:268:LYS:O	2:B:273:SER:CB	2.36	0.73
2:B:274:PRO:HG2	2:B:295:ASN:HB3	1.67	0.73
2:B:274:PRO:CG	2:B:295:ASN:CB	2.63	0.73
2:B:433:VAL:HG11	2:B:471:TYR:HA	1.69	0.73
3:M:121:ILE:HG22	3:M:125:PHE:CE1	2.22	0.73
3:M:437:TYR:CD1	3:M:479:PHE:CZ	2.76	0.73
1:A:274:LEU:O	1:A:275:LEU:C	2.21	0.73
2:B:135:ARG:HH22	2:B:164:ASP:CG	1.90	0.73
2:B:279:LEU:N	2:B:288:TYR:CB	2.41	0.73
2:B:545:ARG:CD	2:B:602:ASP:HB2	2.12	0.73
4:S:55:PRO:HA	4:S:69:ASN:HB3	1.69	0.73
1:A:348:PHE:O	1:A:352:PHE:CD1	2.42	0.73
2:B:98:LYS:HZ2	2:B:134:LEU:HB3	1.50	0.73
2:B:106:LEU:HD21	2:B:144:ASP:HB3	1.68	0.73
2:B:127:LEU:CG	2:B:157:THR:HG21	2.18	0.73
2:B:274:PRO:HG2	2:B:295:ASN:CB	1.90	0.73
2:B:418:TYR:CD1	2:B:424:PHE:CE1	2.77	0.73
3:M:214:LEU:HD23	3:M:214:LEU:C	2.08	0.73
1:A:103:LYS:O	1:A:104:ARG:C	2.24	0.73
2:B:215:TYR:CE1	2:B:233:TYR:CE1	2.76	0.73
2:B:501:THR:O	2:B:508:ARG:NH2	2.21	0.73
2:B:549:LEU:HD13	2:B:611:ALA:CB	2.18	0.73
2:B:559:ASP:HA	2:B:562:ASN:HB2	1.68	0.73
1:A:101:GLN:NE2	4:S:167:ILE:CD1	2.51	0.73
1:A:185:LEU:CD1	1:A:203:PHE:CE1	2.72	0.73
1:A:186:PHE:CE1	1:A:224:GLU:HB3	2.20	0.73

Continued on next page...

Continued from previous page...

Atom-1	Atom-2	Interatomic distance (Å)	Clash overlap (Å)
2:B:178:ILE:HG13	2:B:214:ALA:HA	1.69	0.73
2:B:181:TYR:CZ	2:B:185:LYS:HE2	2.24	0.73
2:B:230:PHE:O	2:B:231:ARG:C	2.07	0.73
2:B:234:CYS:HB3	2:B:301:LEU:HB3	1.71	0.73
2:B:293:VAL:O	2:B:299:LEU:HD12	1.88	0.73
3:M:133:GLU:O	3:M:135:ASN:N	2.22	0.73
3:M:331:LEU:HD12	3:M:331:LEU:O	1.89	0.73
4:S:53:THR:HB	4:S:69:ASN:HB2	1.69	0.73
1:A:186:PHE:HB2	1:A:221:VAL:HG13	1.71	0.73
1:A:498:LEU:O	1:A:501:ASN:O	2.05	0.73
2:B:3:ASP:O	2:B:7:ARG:HG3	1.88	0.73
2:B:21:GLU:CD	2:B:39:SER:HB3	2.08	0.73
2:B:25:VAL:CA	2:B:35:TYR:HB3	2.18	0.73
2:B:158:VAL:CG1	2:B:177:ILE:CD1	2.67	0.73
3:M:243:ILE:H	3:M:474:THR:CG2	2.01	0.73
3:M:245:ASP:N	3:M:472:TYR:CE1	2.56	0.73
3:M:320:ILE:HG23	3:M:439:TYR:OH	1.89	0.73
2:B:170:ARG:CA	2:B:199:LEU:HD22	2.18	0.73
1:A:185:LEU:HB3	1:A:203:PHE:HE1	1.54	0.73
1:A:260:PHE:CE1	1:A:274:LEU:CD1	2.72	0.73
1:A:404:GLN:O	2:B:3:ASP:CA	2.37	0.73
2:B:108:PHE:O	2:B:109:ALA:C	2.18	0.73
2:B:318:ILE:HD13	2:B:346:THR:OG1	1.88	0.73
3:M:290:PHE:HB2	3:M:299:LEU:HD11	1.70	0.73
1:A:176:TYR:CE1	4:S:143:GLU:OE2	2.42	0.73
2:B:162:VAL:HG22	2:B:199:LEU:HD11	1.71	0.73
2:B:566:ALA:C	2:B:574:ASN:CB	2.57	0.73
4:S:6:LEU:HD22	4:S:32:LEU:HD22	1.70	0.73
4:S:55:PRO:O	4:S:58:LEU:HB3	1.88	0.73
1:A:232:PRO:O	1:A:235:GLN:HB2	1.88	0.72
1:A:450:TYR:HH	1:A:476:GLN:HG2	1.53	0.72
3:M:243:ILE:O	3:M:472:TYR:HD2	1.71	0.72
4:S:75:ILE:HG21	4:S:77:TYR:CZ	2.22	0.72
1:A:110:ALA:O	1:A:111:SER:C	2.18	0.72
1:A:121:LEU:CD1	1:A:155:THR:HG23	2.18	0.72
2:B:231:ARG:HH22	2:B:279:LEU:HD21	1.53	0.72
3:M:338:PHE:CE2	3:M:415:ILE:CG1	2.72	0.72
3:M:343:ASN:HD22	3:M:343:ASN:N	1.87	0.72
4:S:53:THR:OG1	4:S:68:VAL:N	2.23	0.72
1:A:532:LEU:O	1:A:533:ILE:C	2.13	0.72
1:A:567:GLN:O	1:A:568:GLU:C	2.26	0.72

Continued on next page...

Continued from previous page...

Atom-1	Atom-2	Interatomic distance (Å)	Clash overlap (Å)
1:A:638:LEU:HD12	2:B:520:SER:HB3	1.71	0.72
2:B:41:ASN:ND2	2:B:43:ASN:OD1	2.22	0.72
1:A:143:VAL:O	1:A:147:LEU:HG	1.89	0.72
1:A:394:GLN:O	1:A:398:GLU:N	2.22	0.72
1:A:638:LEU:HD22	2:B:558:TYR:HA	1.64	0.72
2:B:63:MET:CE	2:B:104:TYR:HB2	2.19	0.72
2:B:389:ILE:O	2:B:390:VAL:C	2.23	0.72
3:M:243:ILE:O	3:M:472:TYR:CD2	2.41	0.72
3:M:336:ASP:OD2	3:M:415:ILE:HB	1.90	0.72
3:M:347:PHE:CG	3:M:350:VAL:HB	2.23	0.72
3:M:374:TYR:HE2	3:M:393:GLY:HA2	1.53	0.72
2:B:479:VAL:HG21	2:B:486:HIS:CD2	2.23	0.72
3:M:223:HIS:CE1	3:M:476:THR:H	2.08	0.72
4:S:39:ILE:HD11	4:S:77:TYR:CD2	2.24	0.72
1:A:289:SER:C	4:S:96:LEU:CD1	2.58	0.72
1:A:332:TYR:CZ	1:A:336:ILE:CD1	2.69	0.72
1:A:332:TYR:CE2	1:A:336:ILE:HD11	2.24	0.72
2:B:513:TRP:O	2:B:516:GLY:N	2.23	0.72
3:M:5:PHE:O	3:M:17:GLN:HA	1.90	0.72
3:M:48:ASP:HA	3:M:75:TRP:CZ2	2.25	0.72
3:M:219:LEU:O	3:M:474:THR:HG21	1.89	0.72
2:B:20:ARG:NH1	2:B:35:TYR:CE1	2.57	0.72
2:B:151:ALA:HB3	2:B:188:TYR:CE1	2.25	0.72
2:B:212:VAL:C	2:B:214:ALA:N	2.41	0.72
4:S:35:VAL:CB	4:S:77:TYR:OH	2.37	0.72
2:B:515:PHE:O	2:B:516:GLY:C	2.26	0.72
3:M:65:TYR:O	3:M:79:SER:HA	1.90	0.72
3:M:224:VAL:HG22	3:M:306:LEU:HD12	1.71	0.72
3:M:240:ILE:HG22	3:M:444:ALA:HB1	1.72	0.72
4:S:53:THR:CB	4:S:68:VAL:C	2.58	0.72
4:S:136:VAL:O	4:S:140:MET:N	2.22	0.72
1:A:251:TRP:CH2	4:S:100:ASP:N	2.45	0.72
2:B:341:GLU:HB2	2:B:377:TYR:OH	1.90	0.72
2:B:399:LEU:HB3	2:B:411:ILE:CG2	2.20	0.72
2:B:399:LEU:HB3	2:B:411:ILE:HG23	1.70	0.72
1:A:306:GLU:O	1:A:307:ASP:C	2.22	0.72
3:M:306:LEU:HD22	3:M:317:MET:HE3	1.71	0.72
1:A:298:ILE:CD1	1:A:311:THR:HG21	2.20	0.71
2:B:374:PHE:CG	2:B:402:LEU:HD21	2.25	0.71
4:S:65:ASN:O	4:S:67:GLU:HG3	1.90	0.71
1:A:182:ILE:CG2	1:A:221:VAL:HG21	2.20	0.71

Continued on next page...

Continued from previous page...

Atom-1	Atom-2	Interatomic distance (Å)	Clash overlap (Å)
1:A:633:PHE:CZ	2:B:554:LYS:HE3	2.24	0.71
2:B:219:TYR:CG	2:B:226:LEU:CB	2.72	0.71
2:B:519:ALA:O	2:B:523:PHE:CG	2.43	0.71
3:M:244:VAL:HG13	3:M:472:TYR:CE2	2.25	0.71
1:A:263:LEU:O	1:A:266:VAL:N	2.24	0.71
1:A:420:ILE:HG22	1:A:421:PRO:O	1.91	0.71
1:A:633:PHE:HZ	2:B:550:VAL:CG1	2.00	0.71
2:B:102:HIS:HE1	2:B:138:ALA:H	1.33	0.71
2:B:519:ALA:HB1	2:B:555:LEU:CD1	2.20	0.71
3:M:245:ASP:OD1	3:M:297:PHE:C	2.29	0.71
3:M:306:LEU:CD2	3:M:317:MET:CE	2.69	0.71
1:A:332:TYR:CE2	1:A:336:ILE:CD1	2.73	0.71
2:B:537:PHE:CZ	2:B:545:ARG:CG	2.73	0.71
3:M:16:PHE:CZ	3:M:18:TYR:HB2	2.25	0.71
3:M:95:THR:HG21	3:M:137:SER:HA	1.69	0.71
3:M:96:ILE:O	3:M:100:LEU:HG	1.91	0.71
4:S:8:PHE:CE1	4:S:84:TYR:HB2	2.25	0.71
4:S:94:SER:O	4:S:98:ILE:HG12	1.90	0.71
1:A:509:PRO:HB3	1:A:547:VAL:CG2	2.20	0.71
1:A:625:LEU:O	1:A:626:SER:C	2.26	0.71
2:B:20:ARG:HG3	2:B:21:GLU:H	1.55	0.71
2:B:215:TYR:CD2	2:B:219:TYR:CE1	2.71	0.71
2:B:436:LEU:HB3	2:B:450:VAL:HG13	1.72	0.71
2:B:580:TYR:HB3	2:B:582:ASP:CG	2.11	0.71
1:A:279:LEU:O	1:A:280:GLU:C	2.23	0.71
1:A:638:LEU:HB2	2:B:558:TYR:CB	2.15	0.71
2:B:530:LEU:CD2	2:B:591:MET:HB3	2.20	0.71
3:M:310:VAL:HG13	3:M:315:VAL:O	1.91	0.71
3:M:479:PHE:CD2	3:M:479:PHE:N	2.59	0.71
1:A:182:ILE:HG22	1:A:221:VAL:CG2	2.21	0.71
2:B:127:LEU:HD22	2:B:161:LEU:HD22	1.71	0.71
2:B:162:VAL:HG22	2:B:199:LEU:CD1	2.20	0.71
2:B:429:VAL:O	2:B:433:VAL:HG23	1.90	0.71
3:M:273:HIS:HB2	3:M:298:ARG:O	1.91	0.71
3:M:282:VAL:N	3:M:283:PHE:N	2.38	0.71
1:A:125:THR:HG1	1:A:158:LEU:CD1	1.91	0.71
1:A:566:PHE:C	1:A:568:GLU:H	1.90	0.71
1:A:601:VAL:O	1:A:602:GLU:C	2.12	0.71
2:B:37:TYR:HD2	2:B:38:TYR:HD1	1.37	0.71
3:M:67:SER:O	3:M:77:LEU:HA	1.91	0.71
3:M:233:LEU:HD22	3:M:324:SER:HA	1.73	0.71

Continued on next page...

Continued from previous page...

Atom-1	Atom-2	Interatomic distance (Å)	Clash overlap (Å)
3:M:379:LEU:CA	3:M:412:ARG:O	2.33	0.71
4:S:8:PHE:CE1	4:S:84:TYR:HB3	2.26	0.71
4:S:75:ILE:HG21	4:S:77:TYR:OH	1.91	0.71
1:A:555:LEU:CD1	1:A:581:LEU:HD11	2.21	0.71
2:B:25:VAL:CG1	2:B:33:SER:HA	2.21	0.71
2:B:30:LEU:HD12	2:B:30:LEU:O	1.91	0.71
2:B:199:LEU:C	2:B:201:ALA:N	2.35	0.71
2:B:549:LEU:CD1	2:B:611:ALA:CA	2.67	0.71
1:A:266:VAL:O	1:A:267:GLU:HB3	1.89	0.71
1:A:602:GLU:OE2	1:A:633:PHE:CE1	2.44	0.71
2:B:102:HIS:O	2:B:103:LEU:C	2.21	0.71
2:B:259:TYR:HD2	2:B:261:PRO:CG	2.00	0.71
2:B:387:ASP:HB3	2:B:391:ALA:CB	2.21	0.71
3:M:224:VAL:HG11	3:M:226:PHE:CZ	2.26	0.71
3:M:323:MET:HE3	3:M:342:LEU:HD23	1.71	0.71
3:M:350:VAL:HA	3:M:442:GLN:HB2	1.71	0.71
1:A:179:LYS:HB2	4:S:142:ILE:HD12	1.71	0.70
1:A:222:ILE:HD12	1:A:240:LEU:HD21	1.72	0.70
1:A:277:LYS:HG3	1:A:277:LYS:O	1.91	0.70
1:A:350:SER:O	1:A:351:ARG:C	2.29	0.70
1:A:461:CYS:O	1:A:462:GLN:O	2.09	0.70
2:B:151:ALA:HB1	2:B:188:TYR:CE1	2.26	0.70
2:B:302:PHE:CE2	2:B:328:LEU:HD11	2.26	0.70
2:B:353:GLN:CD	3:M:50:TYR:CD1	2.64	0.70
2:B:550:VAL:CG2	2:B:610:ARG:HD3	2.21	0.70
3:M:376:ILE:CG2	3:M:379:LEU:CD1	2.69	0.70
4:S:38:LEU:HG	4:S:68:VAL:HG23	1.72	0.70
4:S:55:PRO:O	4:S:58:LEU:CB	2.39	0.70
1:A:114:PHE:O	1:A:115:TYR:C	2.25	0.70
1:A:275:LEU:HD13	1:A:308:ASP:OD1	1.90	0.70
1:A:555:LEU:HD13	1:A:581:LEU:HD11	1.72	0.70
2:B:24:ALA:HB1	2:B:35:TYR:CE1	1.94	0.70
2:B:508:ARG:HB2	2:B:544:THR:HG23	1.72	0.70
3:M:405:THR:O	3:M:405:THR:HG22	1.92	0.70
4:S:87:PHE:CE1	4:S:102:ILE:HG12	2.26	0.70
1:A:64:LEU:HG	1:A:102:GLN:HE22	1.53	0.70
1:A:206:LYS:HE2	1:A:206:LYS:HA	1.73	0.70
2:B:223:LEU:O	2:B:224:GLU:C	2.20	0.70
2:B:267:ASP:H	2:B:289:PRO:HG3	1.56	0.70
2:B:347:VAL:HA	2:B:359:LEU:HG	1.73	0.70
2:B:497:LEU:CD2	2:B:533:LEU:CD2	2.69	0.70

Continued on next page...

Continued from previous page...

Atom-1	Atom-2	Interatomic distance (Å)	Clash overlap (Å)
2:B:549:LEU:HD21	2:B:610:ARG:C	2.06	0.70
3:M:78:ALA:CB	3:M:89:CYS:SG	2.80	0.70
1:A:179:LYS:CB	4:S:142:ILE:HB	2.16	0.70
1:A:264:SER:HB3	1:A:271:ARG:HG2	1.73	0.70
2:B:70:MET:HE3	2:B:107:ARG:HB3	1.72	0.70
2:B:83:PHE:CE2	2:B:119:SER:CB	2.68	0.70
2:B:219:TYR:HE1	2:B:226:LEU:CD1	1.73	0.70
2:B:477:MET:O	2:B:480:GLN:HB2	1.92	0.70
3:M:272:LEU:HD22	3:M:278:ILE:HB	1.72	0.70
3:M:436:GLU:HA	3:M:479:PHE:CE2	2.26	0.70
1:A:487:MET:O	1:A:488:ARG:C	2.22	0.70
1:A:537:THR:HB	1:A:584:PHE:CE1	2.26	0.70
2:B:63:MET:HG2	2:B:100:LEU:HB2	1.73	0.70
2:B:143:SER:HB2	2:B:179:LYS:CD	2.18	0.70
2:B:325:LEU:HD13	2:B:339:PHE:CD2	2.25	0.70
2:B:390:VAL:O	2:B:393:ILE:HB	1.91	0.70
2:B:592:TYR:CD2	2:B:618:PHE:CD2	2.80	0.70
3:M:242:GLY:HA3	3:M:444:ALA:CB	2.22	0.70
3:M:344:ILE:HG23	3:M:347:PHE:HB2	1.73	0.70
1:A:176:TYR:HE1	4:S:148:ARG:HH21	1.36	0.70
2:B:63:MET:HG2	2:B:100:LEU:CB	2.22	0.70
2:B:86:VAL:HG13	2:B:101:ILE:HG12	1.72	0.70
2:B:278:PRO:CB	2:B:288:TYR:C	2.56	0.70
2:B:316:THR:CG2	3:M:90:PHE:HD2	1.98	0.70
2:B:494:ALA:HB2	2:B:515:PHE:CZ	2.27	0.70
2:B:25:VAL:HG11	2:B:36:THR:HB	1.74	0.70
2:B:267:ASP:N	2:B:289:PRO:HG3	2.07	0.70
2:B:334:MET:C	2:B:336:ASN:N	2.34	0.70
2:B:444:THR:O	2:B:445:SER:C	2.23	0.70
2:B:531:ARG:CA	2:B:591:MET:SD	2.79	0.70
2:B:592:TYR:CD2	2:B:618:PHE:CE2	2.80	0.70
1:A:186:PHE:CD1	1:A:224:GLU:CB	2.73	0.70
1:A:326:GLN:HA	1:A:331:ARG:NH2	2.05	0.70
1:A:365:VAL:O	1:A:366:SER:C	2.22	0.70
2:B:241:ASP:CB	3:M:274:ASP:CG	2.47	0.70
2:B:243:TRP:HZ2	3:M:98:ARG:HG3	1.56	0.70
2:B:513:TRP:CA	2:B:551:LEU:HD22	2.13	0.70
2:B:588:ILE:HG23	2:B:618:PHE:CE1	2.27	0.70
2:B:343:LEU:HD21	2:B:362:ALA:HB3	1.74	0.70
1:A:628:VAL:O	1:A:631:SER:OG	2.05	0.70
2:B:162:VAL:HB	2:B:195:ILE:HG23	1.70	0.70

Continued on next page...

Continued from previous page...

Atom-1	Atom-2	Interatomic distance (Å)	Clash overlap (Å)
2:B:458:MET:O	2:B:460:SER:N	2.25	0.70
3:M:12:ASN:OD1	3:M:45:SER:OG	2.09	0.70
3:M:242:GLY:CA	3:M:444:ALA:CB	2.69	0.70
3:M:257:ALA:HB3	3:M:453:ASP:OD1	1.92	0.70
1:A:421:PRO:HG2	1:A:424:TYR:CE1	2.26	0.69
2:B:340:ILE:HG12	2:B:373:LEU:CD2	2.21	0.69
2:B:508:ARG:CB	2:B:544:THR:HG23	2.22	0.69
1:A:395:PHE:CE1	1:A:428:MET:HG3	2.27	0.69
2:B:90:ILE:O	2:B:98:LYS:CE	2.39	0.69
2:B:193:LEU:C	2:B:195:ILE:N	2.42	0.69
2:B:223:LEU:CB	2:B:259:TYR:HD1	1.54	0.69
2:B:243:TRP:HE1	3:M:98:ARG:HD3	1.52	0.69
2:B:345:ARG:NH1	3:M:305:ASP:CG	2.41	0.69
2:B:371:GLN:CG	2:B:401:THR:O	2.40	0.69
2:B:400:SER:HA	2:B:439:CYS:SG	2.31	0.69
2:B:418:TYR:CD1	2:B:419:VAL:N	2.60	0.69
2:B:487:LEU:HD21	2:B:522:GLU:CB	2.22	0.69
2:B:508:ARG:HB2	2:B:544:THR:CG2	2.22	0.69
3:M:214:LEU:O	3:M:467:TYR:N	2.20	0.69
3:M:247:ARG:N	3:M:470:ALA:HB2	2.06	0.69
3:M:96:ILE:HD12	3:M:125:PHE:CD1	2.27	0.69
4:S:130:SER:OG	4:S:156:LEU:HD12	1.92	0.69
4:S:135:ILE:HG22	4:S:141:VAL:HG22	1.72	0.69
1:A:136:GLY:O	1:A:137:ASN:C	2.15	0.69
2:B:20:ARG:HG3	2:B:21:GLU:N	2.06	0.69
2:B:25:VAL:CG2	2:B:35:TYR:HD2	1.99	0.69
2:B:132:SER:CA	2:B:169:VAL:CG2	2.69	0.69
2:B:472:VAL:O	2:B:473:ASN:C	2.28	0.69
2:B:534:ILE:HG12	2:B:595:VAL:HG22	1.75	0.69
3:M:114:ILE:O	3:M:118:TYR:N	2.26	0.69
3:M:128:CYS:O	3:M:134:PRO:HA	1.92	0.69
3:M:472:TYR:CD1	3:M:472:TYR:N	2.58	0.69
2:B:178:ILE:HD13	2:B:218:CYS:CB	2.21	0.69
2:B:267:ASP:O	2:B:268:LYS:C	2.27	0.69
3:M:276:VAL:HG22	3:M:290:PHE:HD1	1.56	0.69
3:M:290:PHE:HB2	3:M:299:LEU:CD1	2.21	0.69
3:M:437:TYR:N	3:M:437:TYR:HD1	1.91	0.69
4:S:109:LEU:O	4:S:110:ASP:C	2.20	0.69
1:A:88:ASN:CB	1:A:120:ILE:CG2	2.67	0.69
1:A:260:PHE:CE1	1:A:274:LEU:HD11	2.27	0.69
2:B:310:ILE:O	2:B:311:TYR:C	2.25	0.69

Continued on next page...

Continued from previous page...

Atom-1	Atom-2	Interatomic distance (Å)	Clash overlap (Å)
3:M:15:ILE:HG22	3:M:114:ILE:HG22	1.75	0.69
3:M:443:SER:OG	3:M:447:ILE:C	2.30	0.69
1:A:101:GLN:NE2	4:S:167:ILE:HD12	2.06	0.69
1:A:244:LEU:HD23	1:A:277:LYS:O	1.92	0.69
2:B:29:LYS:C	2:B:32:GLU:HG3	1.86	0.69
2:B:374:PHE:HE1	2:B:381:PHE:CE1	2.10	0.69
2:B:494:ALA:N	2:B:515:PHE:HZ	1.90	0.69
3:M:219:LEU:HG	3:M:439:TYR:O	1.93	0.69
3:M:242:GLY:O	3:M:301:GLU:HA	1.92	0.69
3:M:265:ASN:OD1	3:M:313:SER:HB3	1.91	0.69
1:A:103:LYS:HB3	1:A:107:TYR:CE1	2.28	0.69
1:A:638:LEU:HD12	2:B:520:SER:H	1.53	0.69
2:B:44:PRO:HB3	2:B:82:TYR:HH	1.57	0.69
2:B:83:PHE:HE2	2:B:119:SER:HB3	1.54	0.69
2:B:243:TRP:HH2	3:M:94:GLU:CB	1.85	0.69
2:B:278:PRO:HB3	2:B:288:TYR:C	2.13	0.69
2:B:553:ALA:CB	2:B:614:ILE:CG1	2.67	0.69
3:M:74:TYR:HB3	3:M:114:ILE:HD11	1.74	0.69
3:M:428:VAL:O	3:M:429:ASP:C	2.29	0.69
1:A:634:ASN:ND2	2:B:554:LYS:HA	2.08	0.69
2:B:136:CYS:CA	2:B:172:GLU:HG3	2.22	0.69
2:B:193:LEU:HD11	2:B:225:LEU:HD23	1.75	0.69
3:M:121:ILE:CG2	3:M:125:PHE:CE1	2.76	0.69
4:S:35:VAL:CG1	4:S:75:ILE:HD13	2.21	0.69
4:S:112:CYS:HB2	4:S:113:PHE:CD1	2.26	0.69
1:A:140:VAL:O	1:A:141:VAL:C	2.25	0.69
1:A:200:PHE:CE1	1:A:236:LEU:CG	2.76	0.69
2:B:139:LEU:HD11	2:B:176:ALA:HB1	1.73	0.69
2:B:374:PHE:HD2	2:B:402:LEU:HD22	1.54	0.69
2:B:515:PHE:CE2	2:B:529:VAL:HG21	2.27	0.69
3:M:454:ILE:HD13	3:M:464:THR:HG21	1.75	0.69
1:A:156:PRO:CB	1:A:192:TYR:CE1	2.71	0.68
1:A:637:GLU:HA	2:B:517:GLU:CD	2.14	0.68
2:B:38:TYR:CA	2:B:42:ILE:N	2.47	0.68
2:B:103:LEU:HD21	3:M:131:ALA:HA	1.74	0.68
2:B:186:ASN:C	2:B:188:TYR:H	1.96	0.68
2:B:512:VAL:C	2:B:551:LEU:CD1	2.45	0.68
3:M:44:ASP:O	3:M:46:SER:N	2.26	0.68
3:M:224:VAL:H	3:M:479:PHE:HA	1.58	0.68
1:A:92:LEU:O	1:A:127:LEU:CD2	2.42	0.68
1:A:137:ASN:O	1:A:139:ASP:N	2.25	0.68

Continued on next page...

Continued from previous page...

Atom-1	Atom-2	Interatomic distance (Å)	Clash overlap (Å)
1:A:369:SER:HB2	1:A:424:TYR:CZ	2.28	0.68
2:B:135:ARG:NH2	2:B:164:ASP:CG	2.46	0.68
2:B:328:LEU:HB3	2:B:333:GLN:HE22	1.58	0.68
3:M:222:PHE:O	3:M:479:PHE:HE2	1.71	0.68
1:A:258:LYS:HZ3	4:S:94:SER:N	1.90	0.68
2:B:468:LEU:O	2:B:472:VAL:HG23	1.94	0.68
2:B:566:ALA:CA	2:B:574:ASN:HB3	2.20	0.68
3:M:443:SER:HB3	3:M:447:ILE:N	2.08	0.68
4:S:53:THR:CG2	4:S:68:VAL:HA	2.20	0.68
1:A:104:ARG:HA	1:A:145:ILE:HG21	1.74	0.68
1:A:399:ASP:H	1:A:418:ILE:HD11	1.57	0.68
1:A:556:VAL:CG2	1:A:603:VAL:CG1	2.72	0.68
3:M:245:ASP:CA	3:M:472:TYR:CE1	2.76	0.68
3:M:306:LEU:HD13	3:M:317:MET:HE3	1.75	0.68
3:M:360:LEU:CD2	3:M:362:PHE:CE2	2.76	0.68
1:A:176:TYR:CG	4:S:143:GLU:OE2	2.47	0.68
1:A:404:GLN:CB	2:B:7:ARG:HH21	2.05	0.68
1:A:594:PHE:C	2:B:476:ARG:NH2	2.47	0.68
1:A:605:GLU:CG	1:A:632:PHE:CD2	2.73	0.68
2:B:542:PRO:O	2:B:607:ILE:HD13	1.93	0.68
2:B:546:CYS:HA	2:B:607:ILE:CG2	2.24	0.68
2:B:589:SER:HA	2:B:592:TYR:HD2	1.58	0.68
1:A:67:LYS:O	1:A:71:VAL:HG23	1.94	0.68
1:A:585:PHE:CE2	1:A:607:LEU:HD11	2.28	0.68
2:B:144:ASP:HA	2:B:179:LYS:HD3	1.74	0.68
2:B:158:VAL:HG13	2:B:177:ILE:HG13	1.73	0.68
2:B:230:PHE:HD2	2:B:298:ASP:HB3	1.59	0.68
2:B:418:TYR:O	2:B:419:VAL:O	2.11	0.68
2:B:451:MET:HG3	2:B:489:ILE:HG12	1.74	0.68
2:B:588:ILE:CG2	2:B:618:PHE:CE1	2.76	0.68
1:A:88:ASN:HD22	1:A:120:ILE:HG13	1.58	0.68
1:A:253:ILE:HD12	1:A:285:THR:HG21	1.75	0.68
1:A:375:VAL:O	1:A:376:GLU:C	2.26	0.68
1:A:403:LEU:C	2:B:3:ASP:OD2	2.31	0.68
1:A:450:TYR:HH	1:A:476:GLN:CG	2.05	0.68
2:B:136:CYS:CB	2:B:172:GLU:HG3	2.24	0.68
2:B:219:TYR:O	2:B:223:LEU:CD2	2.41	0.68
2:B:525:ILE:C	2:B:527:PRO:HD2	2.14	0.68
1:A:128:LEU:HD13	1:A:150:LEU:CG	2.24	0.68
2:B:21:GLU:CD	2:B:35:TYR:CG	2.65	0.68
3:M:220:GLU:CD	3:M:439:TYR:HD1	1.97	0.68

Continued on next page...

Continued from previous page...

Atom-1	Atom-2	Interatomic distance (Å)	Clash overlap (Å)
2:B:16:LYS:CD	3:M:118:TYR:CE2	2.50	0.68
2:B:79:VAL:C	2:B:108:PHE:HE1	1.98	0.68
2:B:178:ILE:O	2:B:180:LEU:N	2.27	0.68
2:B:181:TYR:C	2:B:183:ALA:N	2.46	0.68
2:B:534:ILE:HG12	2:B:595:VAL:CG2	2.24	0.68
3:M:136:VAL:C	3:M:138:ASP:H	1.96	0.68
1:A:240:LEU:O	1:A:242:GLU:O	2.11	0.68
2:B:199:LEU:C	2:B:201:ALA:H	1.95	0.68
2:B:215:TYR:HE2	2:B:229:HIS:HD1	1.41	0.68
2:B:537:PHE:CE1	2:B:545:ARG:CG	2.77	0.68
1:A:114:PHE:CE1	1:A:154:ILE:HG12	2.29	0.67
1:A:237:SER:N	1:A:238:PRO:HD2	2.09	0.67
1:A:506:LYS:O	1:A:507:GLN:HB2	1.94	0.67
2:B:181:TYR:HE1	2:B:222:HIS:CD2	2.08	0.67
2:B:212:VAL:CG1	2:B:248:LEU:HD23	2.24	0.67
2:B:501:THR:CA	2:B:508:ARG:HH22	2.03	0.67
3:M:212:ASN:O	3:M:465:LYS:HB2	1.93	0.67
3:M:302:TYR:HE2	3:M:304:VAL:HB	1.59	0.67
4:S:75:ILE:CG2	4:S:77:TYR:CZ	2.77	0.67
1:A:102:GLN:HE22	4:S:166:LYS:CE	2.07	0.67
1:A:333:ILE:O	1:A:337:LEU:HG	1.95	0.67
1:A:384:LEU:HG	1:A:385:LYS:H	1.60	0.67
1:A:599:ARG:HH11	2:B:513:TRP:HZ3	1.34	0.67
2:B:171:GLY:HA2	2:B:207:VAL:HG13	1.75	0.67
2:B:226:LEU:HD21	2:B:255:TYR:CD1	2.25	0.67
2:B:412:PHE:HE2	2:B:446:TRP:HB3	1.60	0.67
2:B:530:LEU:CG	2:B:591:MET:HB3	2.24	0.67
2:B:578:PRO:CD	2:B:581:TYR:CE1	2.72	0.67
4:S:1:MET:H2	4:S:93:GLU:CB	2.08	0.67
1:A:103:LYS:O	1:A:107:TYR:CD1	2.48	0.67
1:A:323:CYS:SG	1:A:334:SER:HB2	2.34	0.67
2:B:77:ILE:CG2	2:B:82:TYR:CE1	2.78	0.67
3:M:131:ALA:C	3:M:133:GLU:N	2.40	0.67
3:M:253:ASN:OD1	3:M:292:PRO:HG2	1.94	0.67
4:S:17:VAL:HG22	4:S:19:PHE:CZ	2.28	0.67
1:A:95:MET:SD	1:A:107:TYR:HD2	2.15	0.67
1:A:185:LEU:CB	1:A:203:PHE:HE1	2.07	0.67
1:A:585:PHE:CE2	1:A:603:VAL:HG12	2.30	0.67
2:B:176:ALA:O	2:B:178:ILE:N	2.28	0.67
2:B:239:GLN:CD	3:M:279:ASN:HA	2.13	0.67
2:B:252:LEU:HD12	2:B:302:PHE:HE1	1.58	0.67

Continued on next page...

Continued from previous page...

Atom-1	Atom-2	Interatomic distance (Å)	Clash overlap (Å)
2:B:278:PRO:CA	2:B:288:TYR:O	2.40	0.67
3:M:215:TYR:CD1	3:M:468:LYS:HA	2.28	0.67
4:S:2:ILE:HG12	4:S:98:ILE:HD12	1.76	0.67
1:A:216:SER:CB	1:A:252:ILE:HG12	2.25	0.67
1:A:275:LEU:HD21	1:A:311:THR:HG1	1.57	0.67
2:B:87:VAL:CG1	2:B:122:SER:CB	2.52	0.67
2:B:158:VAL:CG1	2:B:177:ILE:HD11	2.25	0.67
2:B:337:THR:N	2:B:373:LEU:CD2	2.56	0.67
2:B:398:ILE:O	2:B:401:THR:N	2.21	0.67
3:M:241:HIS:O	3:M:474:THR:HB	1.94	0.67
4:S:71:GLU:C	4:S:73:ILE:H	1.98	0.67
1:A:166:LEU:O	1:A:170:LEU:CD2	2.42	0.67
2:B:24:ALA:C	2:B:35:TYR:CD2	2.67	0.67
2:B:79:VAL:CB	2:B:108:PHE:CE1	2.78	0.67
2:B:243:TRP:CZ3	3:M:94:GLU:CG	2.73	0.67
1:A:332:TYR:CE1	1:A:366:SER:CB	2.77	0.67
2:B:6:HIS:CG	3:M:25:PRO:C	2.46	0.67
2:B:353:GLN:OE1	3:M:50:TYR:HB2	1.94	0.67
2:B:537:PHE:CE1	2:B:545:ARG:HB3	2.29	0.67
3:M:219:LEU:HD22	3:M:473:LYS:CA	2.24	0.67
1:A:196:LEU:HD22	1:A:196:LEU:C	2.15	0.67
1:A:634:ASN:HB3	2:B:558:TYR:N	2.10	0.67
2:B:70:MET:SD	2:B:107:ARG:HB2	2.34	0.67
2:B:154:ILE:O	2:B:158:VAL:HG23	1.95	0.67
2:B:178:ILE:CD1	2:B:214:ALA:C	2.63	0.67
1:A:225:LEU:HB3	1:A:233:PHE:HE1	1.58	0.67
1:A:581:LEU:CD2	1:A:607:LEU:CD1	2.69	0.67
2:B:261:PRO:HD3	2:B:293:VAL:CG2	2.23	0.67
2:B:267:ASP:C	2:B:276:SER:CB	2.63	0.67
2:B:549:LEU:HG	2:B:614:ILE:HD12	1.77	0.67
3:M:78:ALA:HB1	3:M:89:CYS:SG	2.35	0.67
3:M:428:VAL:O	3:M:430:LEU:N	2.27	0.67
4:S:93:GLU:CG	4:S:98:ILE:HD11	2.25	0.67
1:A:151:SER:HB2	1:A:187:LYS:CB	2.19	0.67
2:B:78:ASP:OD2	2:B:81:LEU:HG	1.94	0.67
2:B:303:LEU:HD11	2:B:333:GLN:CG	2.24	0.67
2:B:375:LEU:O	2:B:376:PRO:C	2.33	0.67
2:B:501:THR:HA	2:B:508:ARG:NH2	2.06	0.67
2:B:553:ALA:HA	2:B:614:ILE:CG2	2.25	0.67
2:B:597:TYR:O	2:B:601:TYR:CE1	2.48	0.67
2:B:599:ALA:C	2:B:601:TYR:N	2.44	0.67

Continued on next page...

Continued from previous page...

Atom-1	Atom-2	Interatomic distance (Å)	Clash overlap (Å)
3:M:99:ILE:CG2	3:M:124:ILE:HG21	2.25	0.67
1:A:153:ILE:HB	1:A:158:LEU:CD2	2.25	0.66
1:A:249:ASN:HB3	1:A:252:ILE:HD12	1.77	0.66
2:B:279:LEU:HG	2:B:288:TYR:CD1	2.29	0.66
2:B:592:TYR:OH	2:B:619:ASP:OD1	2.13	0.66
3:M:442:GLN:HG3	3:M:443:SER:H	1.59	0.66
1:A:64:LEU:HG	1:A:102:GLN:NE2	2.06	0.66
2:B:93:ASN:HA	2:B:134:LEU:HD11	1.76	0.66
2:B:116:THR:CG2	2:B:150:LEU:HD11	2.26	0.66
2:B:310:ILE:HG22	2:B:342:ALA:HB1	1.75	0.66
2:B:311:TYR:CE2	2:B:342:ALA:HB2	2.29	0.66
2:B:580:TYR:HB3	2:B:582:ASP:OD1	1.96	0.66
4:S:57:LEU:O	4:S:67:GLU:O	2.12	0.66
1:A:465:SER:N	2:B:1:MET:CE	2.58	0.66
2:B:309:LEU:CB	2:B:317:VAL:HG11	2.23	0.66
3:M:278:ILE:HG23	3:M:278:ILE:O	1.94	0.66
1:A:107:TYR:CE2	1:A:128:LEU:HD23	2.31	0.66
2:B:116:THR:HG22	2:B:150:LEU:HD11	1.78	0.66
2:B:123:LEU:HD22	2:B:138:ALA:O	1.96	0.66
2:B:274:PRO:CG	2:B:295:ASN:HB3	2.26	0.66
2:B:490:ILE:HG22	2:B:515:PHE:CE2	2.30	0.66
2:B:545:ARG:CD	2:B:602:ASP:CG	2.64	0.66
3:M:323:MET:SD	3:M:342:LEU:CB	2.84	0.66
4:S:54:PRO:CD	4:S:57:LEU:HB2	2.24	0.66
1:A:216:SER:O	1:A:219:VAL:HB	1.96	0.66
1:A:465:SER:N	2:B:1:MET:SD	2.68	0.66
2:B:42:ILE:O	2:B:43:ASN:CB	2.42	0.66
2:B:77:ILE:HG22	2:B:82:TYR:CE1	2.31	0.66
2:B:136:CYS:C	2:B:172:GLU:CG	2.57	0.66
2:B:216:LYS:CA	2:B:251:LEU:HD11	2.10	0.66
2:B:340:ILE:CG1	2:B:373:LEU:CD2	2.73	0.66
2:B:353:GLN:HG2	3:M:50:TYR:CE1	2.30	0.66
2:B:451:MET:CG	2:B:489:ILE:HG12	2.25	0.66
2:B:483:PRO:HA	2:B:486:HIS:HB2	1.76	0.66
2:B:546:CYS:SG	2:B:607:ILE:HG12	2.35	0.66
2:B:566:ALA:C	2:B:574:ASN:CG	2.54	0.66
3:M:262:THR:CG2	3:M:265:ASN:H	2.08	0.66
1:A:137:ASN:C	1:A:139:ASP:N	2.43	0.66
1:A:426:ILE:HD13	1:A:466:ASP:OD2	1.96	0.66
2:B:102:HIS:ND1	2:B:137:PHE:HB3	2.11	0.66
2:B:108:PHE:CE2	2:B:115:LEU:HG	2.30	0.66

Continued on next page...

Continued from previous page...

Atom-1	Atom-2	Interatomic distance (Å)	Clash overlap (Å)
2:B:133:GLU:CA	2:B:168:MET:SD	2.82	0.66
2:B:374:PHE:HB3	2:B:402:LEU:HD21	1.73	0.66
2:B:378:THR:HG23	2:B:379:LYS:N	2.10	0.66
2:B:418:TYR:CD1	2:B:419:VAL:HA	2.31	0.66
2:B:490:ILE:CG2	2:B:515:PHE:CE2	2.78	0.66
3:M:45:SER:CA	3:M:47:SER:N	2.59	0.66
3:M:66:PHE:HB3	3:M:77:LEU:HD11	1.77	0.66
2:B:232:ARG:HG3	2:B:236:ILE:CD1	2.25	0.66
2:B:277:CYS:HA	2:B:292:GLU:HA	1.76	0.66
2:B:343:LEU:CD2	2:B:366:LEU:HD12	2.25	0.66
2:B:540:GLU:OE1	2:B:548:ILE:CD1	2.44	0.66
3:M:271:SER:HB3	3:M:301:GLU:CG	2.25	0.66
1:A:94:VAL:O	1:A:95:MET:C	2.26	0.66
1:A:153:ILE:HB	1:A:158:LEU:HD21	1.78	0.66
1:A:185:LEU:CB	1:A:203:PHE:CE1	2.78	0.66
2:B:169:VAL:O	2:B:173:VAL:HG23	1.96	0.66
2:B:189:HIS:NE2	2:B:222:HIS:HB3	2.10	0.66
2:B:216:LYS:HG3	2:B:251:LEU:CD1	2.25	0.66
2:B:230:PHE:CD2	2:B:298:ASP:CB	2.77	0.66
2:B:241:ASP:HB2	3:M:274:ASP:CB	2.26	0.66
2:B:268:LYS:O	2:B:273:SER:HB3	1.95	0.66
2:B:487:LEU:CD2	2:B:522:GLU:CB	2.74	0.66
2:B:549:LEU:CD2	2:B:611:ALA:H	1.85	0.66
2:B:567:GLN:O	2:B:569:THR:HB	1.93	0.66
3:M:372:ILE:CD1	3:M:428:VAL:HG22	2.26	0.66
1:A:264:SER:CB	1:A:271:ARG:HG3	2.24	0.66
1:A:272:ALA:O	1:A:276:PRO:HD3	1.95	0.66
2:B:120:ILE:HA	2:B:142:LEU:CD2	2.25	0.66
2:B:243:TRP:CZ3	3:M:95:THR:N	2.63	0.66
2:B:343:LEU:HD22	2:B:366:LEU:CD1	2.25	0.66
3:M:220:GLU:OE2	3:M:439:TYR:HD1	1.77	0.66
1:A:212:ILE:O	1:A:213:SER:C	2.26	0.66
1:A:384:LEU:HG	1:A:385:LYS:N	2.11	0.66
1:A:469:LEU:O	1:A:470:GLY:C	2.28	0.66
1:A:555:LEU:CD1	1:A:581:LEU:CD1	2.73	0.66
2:B:116:THR:HG21	2:B:147:MET:HG3	1.77	0.66
2:B:549:LEU:HG	2:B:614:ILE:CD1	2.25	0.66
2:B:566:ALA:CA	2:B:574:ASN:CB	2.70	0.66
2:B:602:ASP:OD1	2:B:603:ASP:N	2.28	0.66
3:M:224:VAL:N	3:M:479:PHE:HA	2.10	0.66
3:M:245:ASP:CA	3:M:472:TYR:CD1	2.78	0.66

Continued on next page...

Continued from previous page...

Atom-1	Atom-2	Interatomic distance (Å)	Clash overlap (Å)
3:M:323:MET:SD	3:M:342:LEU:CG	2.84	0.66
1:A:586:GLU:O	1:A:587:ASN:O	2.13	0.65
1:A:595:GLU:HA	2:B:476:ARG:CZ	2.26	0.65
2:B:21:GLU:OE2	2:B:35:TYR:CE1	2.38	0.65
2:B:219:TYR:OH	2:B:226:LEU:CA	2.32	0.65
2:B:458:MET:SD	2:B:471:TYR:CB	2.83	0.65
3:M:222:PHE:CD1	3:M:240:ILE:CG2	2.78	0.65
3:M:245:ASP:HA	3:M:297:PHE:O	1.96	0.65
3:M:347:PHE:CZ	3:M:350:VAL:HG12	2.31	0.65
4:S:99:LEU:O	4:S:102:ILE:HB	1.96	0.65
1:A:92:LEU:CD2	1:A:124:ALA:HA	2.26	0.65
2:B:475:ILE:HG23	2:B:489:ILE:CG2	2.25	0.65
3:M:319:SER:OG	3:M:345:GLU:N	2.29	0.65
1:A:370:LYS:HE2	1:A:370:LYS:HA	1.76	0.65
2:B:108:PHE:CE2	2:B:115:LEU:CG	2.79	0.65
2:B:243:TRP:HZ2	3:M:98:ARG:CG	2.09	0.65
2:B:537:PHE:CE1	2:B:545:ARG:CB	2.79	0.65
1:A:323:CYS:HG	1:A:338:PHE:HE1	1.44	0.65
1:A:404:GLN:CB	2:B:3:ASP:OD2	2.43	0.65
1:A:438:ALA:O	1:A:441:TYR:CD1	2.50	0.65
2:B:178:ILE:HG13	2:B:214:ALA:O	1.92	0.65
2:B:212:VAL:C	2:B:214:ALA:H	1.99	0.65
2:B:219:TYR:CB	2:B:226:LEU:HD22	2.25	0.65
2:B:234:CYS:CB	2:B:301:LEU:HB3	2.26	0.65
2:B:245:GLN:CD	2:B:309:LEU:HD13	2.11	0.65
2:B:602:ASP:O	2:B:608:ARG:NE	2.29	0.65
4:S:89:VAL:HG11	4:S:98:ILE:CD1	2.27	0.65
1:A:121:LEU:CD1	1:A:153:ILE:HG22	2.26	0.65
1:A:185:LEU:HB3	1:A:203:PHE:CE1	2.31	0.65
1:A:231:GLN:HB2	1:A:232:PRO:HD3	1.78	0.65
1:A:292:TYR:CD1	1:A:292:TYR:O	2.50	0.65
1:A:408:ILE:HG21	4:S:64:ASN:C	2.13	0.65
1:A:460:LEU:O	1:A:463:ASP:HB2	1.97	0.65
1:A:637:GLU:CG	2:B:517:GLU:HA	2.23	0.65
2:B:276:SER:O	2:B:291:TYR:O	2.14	0.65
2:B:278:PRO:HD2	2:B:292:GLU:CA	2.25	0.65
2:B:508:ARG:CB	2:B:544:THR:CG2	2.75	0.65
3:M:218:LEU:CA	3:M:472:TYR:CE2	2.78	0.65
1:A:158:LEU:HG	1:A:162:ILE:HD12	1.78	0.65
1:A:170:LEU:HD22	1:A:181:ALA:HB1	1.78	0.65
1:A:254:ILE:CG1	1:A:290:VAL:HG22	2.26	0.65

Continued on next page...

Continued from previous page...

Atom-1	Atom-2	Interatomic distance (Å)	Clash overlap (Å)
1:A:586:GLU:HB2	1:A:604:LEU:CD1	2.26	0.65
2:B:62:ALA:O	2:B:66:ILE:CG1	2.44	0.65
2:B:104:TYR:O	2:B:107:ARG:HB2	1.95	0.65
2:B:108:PHE:CE2	2:B:115:LEU:HB2	2.31	0.65
2:B:167:ALA:C	2:B:207:VAL:HG21	2.17	0.65
2:B:243:TRP:CH2	3:M:94:GLU:CA	2.74	0.65
2:B:393:ILE:HG23	2:B:431:MET:CB	2.27	0.65
3:M:217:ASP:N	3:M:472:TYR:OH	2.30	0.65
1:A:192:TYR:HD2	1:A:195:ALA:H	1.43	0.65
1:A:244:LEU:CG	1:A:281:LEU:CD1	2.74	0.65
1:A:258:LYS:HZ3	4:S:94:SER:CB	2.02	0.65
1:A:275:LEU:O	1:A:277:LYS:N	2.29	0.65
1:A:279:LEU:HD13	1:A:314:ALA:HB3	1.79	0.65
1:A:531:ASP:C	1:A:534:LYS:O	2.35	0.65
2:B:25:VAL:CG2	2:B:35:TYR:CD2	2.74	0.65
2:B:29:LYS:O	2:B:32:GLU:HG3	1.96	0.65
2:B:129:ASP:O	2:B:135:ARG:HD3	1.96	0.65
2:B:252:LEU:HB3	2:B:302:PHE:CG	2.32	0.65
2:B:267:ASP:C	2:B:276:SER:HB2	2.16	0.65
3:M:226:PHE:CE1	3:M:321:GLY:O	2.50	0.65
4:S:1:MET:N	4:S:93:GLU:CD	2.49	0.65
2:B:181:TYR:HE1	2:B:222:HIS:NE2	1.95	0.65
2:B:513:TRP:HA	2:B:551:LEU:HD13	1.57	0.65
4:S:8:PHE:HZ	4:S:86:THR:OG1	1.72	0.65
1:A:556:VAL:CG2	1:A:603:VAL:HG13	2.27	0.65
1:A:556:VAL:HG22	1:A:603:VAL:CG1	2.26	0.65
2:B:245:GLN:HB3	2:B:309:LEU:HD11	1.71	0.65
2:B:513:TRP:CB	2:B:551:LEU:HD11	2.27	0.65
2:B:546:CYS:HB2	2:B:607:ILE:HD11	1.79	0.65
3:M:347:PHE:CE1	3:M:350:VAL:CB	2.79	0.65
3:M:347:PHE:CE1	3:M:350:VAL:HB	2.31	0.65
4:S:53:THR:OG1	4:S:68:VAL:C	2.35	0.65
4:S:130:SER:CB	4:S:156:LEU:HD13	2.27	0.65
1:A:128:LEU:CD1	1:A:150:LEU:CD2	2.75	0.65
1:A:258:LYS:HZ3	4:S:94:SER:CA	2.10	0.65
2:B:267:ASP:HB3	2:B:289:PRO:CG	2.27	0.65
2:B:352:ASN:HD22	3:M:70:ASN:HB3	1.54	0.65
1:A:95:MET:HB2	1:A:127:LEU:HD23	1.77	0.64
1:A:383:ASN:O	1:A:387:ILE:HG12	1.96	0.64
1:A:460:LEU:O	1:A:463:ASP:N	2.28	0.64
2:B:74:ASP:O	2:B:77:ILE:HB	1.97	0.64

Continued on next page...

Continued from previous page...

Atom-1	Atom-2	Interatomic distance (Å)	Clash overlap (Å)
2:B:343:LEU:HD22	2:B:366:LEU:HD12	1.78	0.64
2:B:347:VAL:HG22	2:B:359:LEU:CG	2.27	0.64
2:B:399:LEU:O	2:B:400:SER:C	2.34	0.64
2:B:479:VAL:CG1	2:B:486:HIS:CG	2.66	0.64
1:A:536:MET:O	1:A:537:THR:O	2.15	0.64
1:A:633:PHE:CZ	2:B:550:VAL:HG12	2.27	0.64
2:B:374:PHE:HD2	2:B:402:LEU:CG	2.11	0.64
2:B:394:TRP:CZ3	2:B:397:GLN:OE1	2.49	0.64
2:B:447:GLU:HG3	2:B:482:ASN:ND2	2.12	0.64
3:M:69:ILE:O	3:M:75:TRP:HA	1.97	0.64
3:M:214:LEU:HD23	3:M:214:LEU:O	1.96	0.64
3:M:221:THR:N	3:M:474:THR:HG1	1.94	0.64
3:M:245:ASP:N	3:M:472:TYR:CD2	2.64	0.64
1:A:349:ILE:HG21	1:A:378:ILE:CG2	2.24	0.64
2:B:208:ILE:HG21	2:B:236:ILE:CG2	2.26	0.64
2:B:267:ASP:HB3	2:B:289:PRO:CD	2.28	0.64
2:B:351:GLU:OE2	3:M:476:THR:C	2.35	0.64
2:B:518:ILE:O	2:B:518:ILE:CD1	2.46	0.64
2:B:545:ARG:HB2	2:B:602:ASP:OD2	1.97	0.64
2:B:41:ASN:CG	2:B:43:ASN:OD1	2.36	0.64
3:M:212:ASN:HB3	3:M:250:LEU:HD23	1.78	0.64
3:M:306:LEU:HD13	3:M:317:MET:CE	2.26	0.64
2:B:226:LEU:CD2	2:B:255:TYR:CE1	2.70	0.64
3:M:220:GLU:CG	3:M:439:TYR:CD1	2.71	0.64
3:M:223:HIS:CG	3:M:476:THR:HG1	2.12	0.64
3:M:323:MET:SD	3:M:342:LEU:CA	2.85	0.64
3:M:327:PHE:HE1	3:M:336:ASP:CB	2.09	0.64
1:A:185:LEU:HD13	1:A:203:PHE:HE1	1.63	0.64
2:B:25:VAL:CG2	2:B:35:TYR:C	2.49	0.64
2:B:127:LEU:HB3	2:B:161:LEU:CD1	2.27	0.64
2:B:178:ILE:C	2:B:180:LEU:H	2.00	0.64
2:B:219:TYR:OH	2:B:226:LEU:N	2.30	0.64
2:B:537:PHE:CE2	2:B:598:LEU:O	2.51	0.64
2:B:546:CYS:HA	2:B:607:ILE:CG1	2.21	0.64
3:M:105:ASP:O	3:M:106:LYS:HB2	1.95	0.64
1:A:136:GLY:O	1:A:139:ASP:HB3	1.97	0.64
1:A:236:LEU:C	1:A:238:PRO:HD2	2.18	0.64
1:A:608:ARG:NH1	1:A:612:GLU:OE2	2.30	0.64
2:B:132:SER:CB	2:B:169:VAL:CG2	2.76	0.64
2:B:208:ILE:HG12	2:B:236:ILE:CD1	2.20	0.64
4:S:73:ILE:CG2	4:S:88:ILE:HG23	2.26	0.64

Continued on next page...

Continued from previous page...

Atom-1	Atom-2	Interatomic distance (Å)	Clash overlap (Å)
1:A:166:LEU:O	1:A:170:LEU:HD22	1.98	0.64
1:A:586:GLU:HB2	1:A:604:LEU:HD11	1.79	0.64
2:B:25:VAL:CG2	2:B:36:THR:CB	2.57	0.64
2:B:239:GLN:CD	3:M:279:ASN:C	2.56	0.64
2:B:566:ALA:N	2:B:574:ASN:ND2	2.42	0.64
3:M:245:ASP:O	3:M:472:TYR:OH	2.14	0.64
4:S:16:LEU:CD2	4:S:128:LEU:HD23	2.28	0.64
4:S:17:VAL:CG2	4:S:19:PHE:CE2	2.81	0.64
1:A:207:LEU:O	1:A:243:ILE:CD1	2.38	0.64
1:A:581:LEU:HG	1:A:585:PHE:CE2	2.33	0.64
2:B:25:VAL:HG22	2:B:35:TYR:CA	2.27	0.64
2:B:143:SER:HB3	2:B:176:ALA:HA	1.79	0.64
2:B:534:ILE:O	2:B:598:LEU:CD1	2.45	0.64
2:B:597:TYR:O	2:B:601:TYR:CD1	2.51	0.64
3:M:217:ASP:O	3:M:472:TYR:CG	2.51	0.64
4:S:53:THR:HB	4:S:69:ASN:CA	2.28	0.64
1:A:279:LEU:CD1	1:A:314:ALA:CB	2.75	0.64
1:A:342:GLY:O	1:A:343:LYS:C	2.34	0.64
2:B:223:LEU:CD1	2:B:259:TYR:N	2.40	0.64
2:B:303:LEU:CD1	2:B:333:GLN:HB3	2.27	0.64
2:B:537:PHE:CD2	2:B:537:PHE:C	2.71	0.64
3:M:262:THR:HG22	3:M:264:GLY:N	2.13	0.64
3:M:347:PHE:CE2	3:M:350:VAL:O	2.51	0.64
2:B:501:THR:CA	2:B:508:ARG:NH2	2.60	0.63
3:M:356:LEU:C	3:M:356:LEU:HD23	2.19	0.63
4:S:47:GLN:O	4:S:48:SER:C	2.30	0.63
1:A:102:GLN:NE2	4:S:166:LYS:CE	2.61	0.63
1:A:463:ASP:HA	3:M:58:ARG:NH2	2.14	0.63
1:A:637:GLU:O	2:B:517:GLU:O	2.17	0.63
2:B:80:GLN:HG2	2:B:115:LEU:CD2	2.15	0.63
2:B:196:LEU:C	2:B:215:TYR:OH	2.35	0.63
2:B:253:ILE:HD11	2:B:324:ALA:HB2	1.80	0.63
2:B:566:ALA:C	2:B:574:ASN:HB3	2.16	0.63
3:M:74:TYR:HB3	3:M:114:ILE:CD1	2.28	0.63
3:M:223:HIS:CD2	3:M:478:ASN:CA	2.77	0.63
4:S:6:LEU:HA	4:S:16:LEU:O	1.99	0.63
4:S:70:ASN:O	4:S:71:GLU:C	2.30	0.63
4:S:71:GLU:C	4:S:73:ILE:N	2.50	0.63
1:A:588:LEU:O	1:A:589:SER:C	2.36	0.63
1:A:595:GLU:HG2	2:B:473:ASN:OD1	1.99	0.63
2:B:214:ALA:O	2:B:216:LYS:N	2.32	0.63

Continued on next page...

Continued from previous page...

Atom-1	Atom-2	Interatomic distance (Å)	Clash overlap (Å)
2:B:226:LEU:O	2:B:229:HIS:HB2	1.99	0.63
2:B:276:SER:O	2:B:295:ASN:HB2	1.98	0.63
1:A:92:LEU:O	1:A:127:LEU:HD21	1.98	0.63
1:A:237:SER:HB2	1:A:270:LEU:CD1	2.28	0.63
1:A:438:ALA:O	1:A:441:TYR:CE1	2.51	0.63
1:A:581:LEU:HD11	1:A:585:PHE:CZ	2.33	0.63
1:A:626:SER:HB2	2:B:617:LEU:HD21	1.80	0.63
2:B:230:PHE:HE2	2:B:234:CYS:SG	2.20	0.63
2:B:243:TRP:HZ2	3:M:98:ARG:CD	2.09	0.63
2:B:367:SER:OG	2:B:401:THR:HB	1.96	0.63
3:M:373:ALA:O	3:M:418:GLU:N	2.32	0.63
4:S:1:MET:H2	4:S:93:GLU:CG	2.11	0.63
1:A:241:TYR:C	1:A:242:GLU:O	2.22	0.63
1:A:594:PHE:CG	2:B:477:MET:SD	2.92	0.63
2:B:87:VAL:O	2:B:90:ILE:HG22	1.98	0.63
2:B:132:SER:CB	2:B:169:VAL:HG23	2.24	0.63
2:B:486:HIS:CE1	2:B:518:ILE:CG1	2.82	0.63
2:B:512:VAL:HG21	2:B:548:ILE:HG12	1.80	0.63
2:B:563:PHE:C	2:B:566:ALA:HB3	2.17	0.63
3:M:242:GLY:HA3	3:M:444:ALA:HB2	1.80	0.63
3:M:244:VAL:N	3:M:300:LEU:O	2.31	0.63
3:M:244:VAL:CA	3:M:472:TYR:CD2	2.68	0.63
3:M:306:LEU:CD2	3:M:317:MET:HE3	2.28	0.63
1:A:128:LEU:HD13	1:A:150:LEU:CD2	2.29	0.63
1:A:320:HIS:O	1:A:322:PHE:N	2.30	0.63
1:A:402:ILE:HB	1:A:421:PRO:HA	1.80	0.63
1:A:480:LEU:C	1:A:480:LEU:HD13	2.18	0.63
2:B:25:VAL:CG2	2:B:36:THR:HA	2.04	0.63
2:B:132:SER:HA	2:B:169:VAL:HG21	1.80	0.63
2:B:162:VAL:CG2	2:B:195:ILE:HG22	2.01	0.63
2:B:216:LYS:CG	2:B:251:LEU:CD1	2.73	0.63
2:B:249:ILE:HG12	2:B:306:LEU:CD2	2.28	0.63
2:B:347:VAL:CG2	2:B:359:LEU:HB3	2.22	0.63
2:B:447:GLU:OE1	2:B:485:LYS:HG3	1.98	0.63
3:M:217:ASP:O	3:M:472:TYR:CD2	2.52	0.63
1:A:149:GLY:O	1:A:152:THR:N	2.27	0.63
1:A:174:ARG:NH1	4:S:148:ARG:HH22	1.95	0.63
1:A:186:PHE:HD2	1:A:187:LYS:N	1.97	0.63
1:A:319:LEU:O	1:A:320:HIS:O	2.16	0.63
1:A:573:GLU:O	1:A:577:VAL:HG23	1.98	0.63
1:A:625:LEU:O	1:A:627:GLU:N	2.31	0.63

Continued on next page...

Continued from previous page...

Atom-1	Atom-2	Interatomic distance (Å)	Clash overlap (Å)
2:B:24:ALA:HB1	2:B:35:TYR:CG	2.19	0.63
2:B:513:TRP:CA	2:B:551:LEU:HD21	2.22	0.63
2:B:520:SER:O	2:B:523:PHE:HE2	1.81	0.63
2:B:531:ARG:HB2	2:B:591:MET:SD	2.39	0.63
3:M:217:ASP:CG	3:M:471:LYS:HA	2.19	0.63
1:A:225:LEU:HB2	1:A:233:PHE:CE1	2.28	0.63
1:A:637:GLU:HG3	2:B:517:GLU:HA	1.80	0.63
1:A:638:LEU:CG	2:B:520:SER:HA	2.27	0.63
2:B:104:TYR:O	2:B:107:ARG:N	2.31	0.63
2:B:127:LEU:CG	2:B:157:THR:CG2	2.74	0.63
2:B:347:VAL:HG11	2:B:381:PHE:HE2	1.63	0.63
2:B:431:MET:O	2:B:434:LYS:N	2.32	0.63
2:B:486:HIS:CG	2:B:518:ILE:CD1	2.76	0.63
2:B:500:GLN:CD	2:B:503:LEU:HD21	2.19	0.63
1:A:200:PHE:CZ	1:A:236:LEU:HG	2.33	0.63
1:A:219:VAL:CG1	1:A:256:LEU:CD2	2.74	0.63
1:A:323:CYS:SG	1:A:338:PHE:HE1	2.22	0.63
2:B:256:CYS:O	2:B:257:LYS:C	2.35	0.63
2:B:542:PRO:O	2:B:607:ILE:HD11	1.98	0.63
3:M:45:SER:CA	3:M:47:SER:H	2.12	0.63
1:A:99:LYS:HG2	1:A:101:GLN:H	1.63	0.62
1:A:581:LEU:HD23	1:A:607:LEU:HD13	1.81	0.62
2:B:25:VAL:HG11	2:B:36:THR:HG1	1.60	0.62
2:B:30:LEU:HD12	2:B:30:LEU:C	2.18	0.62
2:B:44:PRO:HG3	2:B:77:ILE:HD12	1.81	0.62
2:B:143:SER:CA	2:B:179:LYS:HD2	2.29	0.62
2:B:167:ALA:HA	2:B:202:ASP:CG	2.17	0.62
2:B:408:VAL:CG1	2:B:412:PHE:CE2	2.83	0.62
2:B:430:ILE:O	2:B:433:VAL:HB	1.98	0.62
2:B:553:ALA:HA	2:B:614:ILE:HG21	1.81	0.62
3:M:44:ASP:O	3:M:47:SER:N	2.32	0.62
3:M:94:GLU:O	3:M:97:ASP:HB2	1.99	0.62
1:A:258:LYS:NZ	4:S:94:SER:H	1.96	0.62
1:A:384:LEU:CG	1:A:385:LYS:N	2.62	0.62
1:A:585:PHE:CD2	1:A:603:VAL:HG12	2.35	0.62
2:B:36:THR:HG22	2:B:40:GLN:HG3	1.81	0.62
2:B:106:LEU:HD13	2:B:144:ASP:HB2	1.80	0.62
2:B:239:GLN:CD	3:M:279:ASN:O	2.37	0.62
2:B:267:ASP:HB3	2:B:289:PRO:HG3	1.79	0.62
2:B:371:GLN:NE2	2:B:401:THR:O	2.32	0.62
3:M:219:LEU:HB2	3:M:472:TYR:O	1.99	0.62

Continued on next page...

Continued from previous page...

Atom-1	Atom-2	Interatomic distance (Å)	Clash overlap (Å)
1:A:516:ILE:HG23	1:A:554:ALA:HB3	1.79	0.62
3:M:437:TYR:CD1	3:M:479:PHE:CE1	2.87	0.62
2:B:505:ASP:O	2:B:506:ASN:C	2.24	0.62
2:B:512:VAL:HB	2:B:551:LEU:HD12	1.81	0.62
3:M:316:ARG:HG2	3:M:322:LEU:HD13	1.80	0.62
3:M:467:TYR:CG	3:M:468:LYS:N	2.68	0.62
1:A:533:ILE:HG12	1:A:562:TRP:HH2	1.62	0.62
2:B:25:VAL:N	2:B:35:TYR:HD2	1.97	0.62
2:B:302:PHE:CD2	2:B:328:LEU:HD11	2.34	0.62
2:B:311:TYR:O	2:B:312:SER:C	2.37	0.62
2:B:487:LEU:HD21	2:B:522:GLU:HB2	1.81	0.62
2:B:580:TYR:HB3	2:B:582:ASP:OD2	1.98	0.62
1:A:289:SER:O	1:A:290:VAL:O	2.17	0.62
2:B:127:LEU:CD2	2:B:161:LEU:CD2	2.73	0.62
2:B:177:ILE:HG21	2:B:196:LEU:HG	1.81	0.62
2:B:486:HIS:CD2	2:B:518:ILE:HG12	2.35	0.62
2:B:486:HIS:HD1	2:B:518:ILE:HD13	1.56	0.62
3:M:224:VAL:H	3:M:479:PHE:CB	2.12	0.62
3:M:327:PHE:CE1	3:M:336:ASP:OD2	2.53	0.62
1:A:68:THR:HB	4:S:166:LYS:O	2.00	0.62
1:A:260:PHE:CZ	1:A:274:LEU:CD1	2.81	0.62
1:A:279:LEU:HD11	1:A:314:ALA:CB	2.29	0.62
2:B:328:LEU:HB2	2:B:333:GLN:NE2	2.14	0.62
2:B:519:ALA:O	2:B:523:PHE:HD2	1.77	0.62
3:M:78:ALA:HB3	3:M:89:CYS:SG	2.39	0.62
3:M:350:VAL:CG1	3:M:442:GLN:HG2	2.30	0.62
4:S:38:LEU:HB3	4:S:51:LEU:CD1	2.30	0.62
4:S:53:THR:CB	4:S:69:ASN:HB2	2.28	0.62
1:A:186:PHE:HA	1:A:221:VAL:HG13	1.81	0.62
1:A:349:ILE:O	1:A:352:PHE:N	2.30	0.62
2:B:219:TYR:O	2:B:223:LEU:HD21	1.99	0.62
2:B:520:SER:O	2:B:523:PHE:CE2	2.53	0.62
3:M:215:TYR:O	3:M:246:VAL:HG13	2.00	0.62
1:A:364:ASP:HB3	1:A:367:ILE:HD12	1.82	0.62
2:B:311:TYR:HE2	2:B:342:ALA:HB2	1.65	0.62
3:M:265:ASN:HA	3:M:313:SER:OG	2.00	0.62
3:M:360:LEU:HD23	3:M:362:PHE:CE2	2.35	0.62
4:S:5:VAL:HG22	4:S:87:PHE:CD2	2.34	0.62
1:A:638:LEU:CB	2:B:558:TYR:CE1	2.69	0.62
2:B:141:ALA:O	2:B:142:LEU:C	2.35	0.62
2:B:185:LYS:CE	2:B:221:ASP:OD2	2.47	0.62

Continued on next page...

Continued from previous page...

Atom-1	Atom-2	Interatomic distance (Å)	Clash overlap (Å)
2:B:592:TYR:CE2	2:B:618:PHE:CD2	2.87	0.62
3:M:65:TYR:CG	3:M:66:PHE:N	2.66	0.62
3:M:107:ASP:OD1	3:M:296:LYS:NZ	2.33	0.62
4:S:132:LEU:O	4:S:136:VAL:HG23	2.00	0.62
1:A:121:LEU:HD12	1:A:153:ILE:CG2	2.29	0.61
1:A:404:GLN:CG	2:B:7:ARG:HH21	2.13	0.61
2:B:25:VAL:CG1	2:B:36:THR:CB	2.69	0.61
2:B:63:MET:CG	2:B:100:LEU:CB	2.78	0.61
2:B:260:LEU:CA	2:B:293:VAL:HG21	2.23	0.61
4:S:49:SER:O	4:S:77:TYR:HB2	2.00	0.61
1:A:88:ASN:HB2	1:A:120:ILE:HD12	1.82	0.61
1:A:223:CYS:HB2	1:A:259:LEU:HD12	1.81	0.61
2:B:178:ILE:HD11	2:B:215:TYR:CA	2.29	0.61
2:B:243:TRP:CH2	3:M:95:THR:CA	2.83	0.61
2:B:280:PRO:CG	2:B:283:TYR:HD1	2.10	0.61
2:B:451:MET:HG3	2:B:489:ILE:CG1	2.30	0.61
2:B:530:LEU:HD23	2:B:591:MET:CB	2.30	0.61
3:M:320:ILE:O	3:M:322:LEU:N	2.32	0.61
4:S:64:ASN:C	4:S:66:ASP:N	2.47	0.61
4:S:65:ASN:O	4:S:67:GLU:N	2.33	0.61
2:B:20:ARG:CG	2:B:21:GLU:N	2.63	0.61
2:B:132:SER:O	2:B:133:GLU:C	2.27	0.61
2:B:155:LEU:CB	2:B:188:TYR:CD2	2.83	0.61
2:B:285:GLU:C	2:B:286:ILE:O	2.21	0.61
2:B:563:PHE:CD2	2:B:584:SER:CA	2.83	0.61
3:M:224:VAL:H	3:M:479:PHE:CA	2.12	0.61
4:S:53:THR:HG21	4:S:67:GLU:O	2.00	0.61
4:S:87:PHE:CD1	4:S:102:ILE:HG12	2.36	0.61
1:A:461:CYS:C	1:A:463:ASP:N	2.46	0.61
2:B:25:VAL:HG22	2:B:35:TYR:CB	2.31	0.61
2:B:95:THR:CG2	2:B:137:PHE:HE2	2.13	0.61
2:B:523:PHE:CE1	2:B:582:ASP:OD2	2.52	0.61
2:B:563:PHE:HD2	2:B:584:SER:HB3	1.63	0.61
3:M:15:ILE:O	3:M:118:TYR:CD1	2.46	0.61
3:M:219:LEU:CB	3:M:472:TYR:O	2.48	0.61
1:A:438:ALA:O	1:A:439:ASP:HB2	1.99	0.61
2:B:293:VAL:HA	2:B:299:LEU:HB2	1.81	0.61
2:B:381:PHE:O	2:B:395:LYS:HD2	2.00	0.61
2:B:424:PHE:CD2	2:B:428:VAL:HG11	2.35	0.61
2:B:474:VAL:O	2:B:477:MET:N	2.33	0.61
2:B:559:ASP:O	2:B:562:ASN:C	2.38	0.61

Continued on next page...

Continued from previous page...

Atom-1	Atom-2	Interatomic distance (Å)	Clash overlap (Å)
3:M:219:LEU:O	3:M:474:THR:CG2	2.48	0.61
1:A:316:LEU:HD13	1:A:348:PHE:CD2	2.31	0.61
1:A:495:ILE:HG23	1:A:515:CYS:SG	2.40	0.61
2:B:120:ILE:HD11	2:B:145:MET:HG3	1.83	0.61
2:B:167:ALA:O	2:B:207:VAL:HG11	2.00	0.61
2:B:231:ARG:O	2:B:234:CYS:N	2.33	0.61
2:B:261:PRO:HG2	2:B:292:GLU:HB3	1.82	0.61
2:B:279:LEU:HD12	2:B:285:GLU:CG	2.28	0.61
2:B:336:ASN:HB3	2:B:339:PHE:CD1	2.35	0.61
2:B:336:ASN:C	2:B:373:LEU:HD21	2.20	0.61
2:B:562:ASN:O	2:B:581:TYR:HD2	1.83	0.61
3:M:134:PRO:O	3:M:136:VAL:N	2.24	0.61
3:M:212:ASN:CB	3:M:250:LEU:HD23	2.30	0.61
1:A:186:PHE:CD2	1:A:187:LYS:N	2.69	0.61
1:A:211:ASP:OD1	1:A:213:SER:N	2.32	0.61
2:B:237:ILE:HG21	2:B:305:SER:HB3	1.82	0.61
2:B:604:GLU:HB3	2:B:607:ILE:HD12	1.81	0.61
3:M:106:LYS:CB	3:M:296:LYS:NZ	2.32	0.61
3:M:133:GLU:O	3:M:134:PRO:C	2.24	0.61
3:M:253:ASN:HA	3:M:292:PRO:HG2	1.82	0.61
3:M:351:SER:CB	3:M:440:ILE:O	2.43	0.61
3:M:445:SER:OG	3:M:447:ILE:HG23	2.01	0.61
4:S:135:ILE:HG23	4:S:141:VAL:HG13	1.83	0.61
1:A:258:LYS:NZ	4:S:94:SER:CA	2.63	0.61
2:B:243:TRP:CD1	3:M:274:ASP:OD2	2.47	0.61
2:B:319:LEU:HD13	2:B:358:MET:HB3	1.83	0.61
3:M:134:PRO:O	3:M:136:VAL:CG2	2.49	0.61
1:A:264:SER:HB2	1:A:271:ARG:HG3	1.83	0.61
1:A:405:THR:H	2:B:7:ARG:CZ	2.10	0.61
2:B:307:ASN:O	2:B:310:ILE:N	2.33	0.61
2:B:351:GLU:O	3:M:49:ASP:CG	2.38	0.61
2:B:475:ILE:HG22	2:B:514:LEU:HD21	1.82	0.61
2:B:497:LEU:HD23	2:B:533:LEU:CD2	2.24	0.61
2:B:513:TRP:CD2	2:B:551:LEU:HD21	2.36	0.61
2:B:589:SER:HG	2:B:618:PHE:HE2	1.37	0.61
3:M:218:LEU:O	3:M:441:GLY:CA	2.49	0.61
3:M:360:LEU:HD13	3:M:433:VAL:HB	1.82	0.61
3:M:433:VAL:HG13	3:M:433:VAL:O	2.01	0.61
1:A:244:LEU:HD13	1:A:256:LEU:HB2	1.82	0.61
1:A:562:TRP:O	1:A:565:ASN:N	2.24	0.61
1:A:582:ILE:HG12	1:A:607:LEU:HB2	1.83	0.61

Continued on next page...

Continued from previous page...

Atom-1	Atom-2	Interatomic distance (Å)	Clash overlap (Å)
2:B:102:HIS:CD2	2:B:138:ALA:HA	2.26	0.61
2:B:151:ALA:O	2:B:188:TYR:CE2	2.54	0.61
2:B:568:VAL:O	2:B:571:SER:OG	2.18	0.61
4:S:53:THR:HB	4:S:68:VAL:C	2.21	0.61
4:S:135:ILE:O	4:S:141:VAL:CA	2.38	0.61
1:A:586:GLU:O	1:A:588:LEU:N	2.34	0.60
1:A:633:PHE:CZ	2:B:550:VAL:HG13	2.34	0.60
1:A:638:LEU:CD2	2:B:558:TYR:C	2.67	0.60
2:B:29:LYS:O	2:B:30:LEU:C	2.37	0.60
2:B:291:TYR:CE2	2:B:294:VAL:HB	2.36	0.60
2:B:292:GLU:O	2:B:296:ASP:CB	2.49	0.60
2:B:412:PHE:CZ	2:B:450:VAL:CG2	2.83	0.60
3:M:74:TYR:CB	3:M:114:ILE:HD11	2.30	0.60
3:M:350:VAL:CG1	3:M:442:GLN:HB3	2.28	0.60
3:M:433:VAL:HG12	3:M:481:VAL:HB	1.82	0.60
3:M:443:SER:OG	3:M:448:TYR:HA	2.00	0.60
1:A:316:LEU:HD11	1:A:348:PHE:CD2	2.35	0.60
2:B:230:PHE:CD2	2:B:298:ASP:O	2.50	0.60
2:B:345:ARG:NH1	3:M:305:ASP:CB	2.63	0.60
2:B:513:TRP:HB2	2:B:551:LEU:HD11	1.83	0.60
3:M:223:HIS:HA	3:M:479:PHE:CG	2.35	0.60
3:M:265:ASN:CG	3:M:313:SER:HG	2.02	0.60
1:A:484:VAL:O	1:A:486:SER:O	2.19	0.60
1:A:581:LEU:HD23	1:A:607:LEU:HD21	1.81	0.60
2:B:334:MET:HG3	2:B:369:LEU:HD23	1.82	0.60
2:B:335:LYS:HA	2:B:370:ASP:OD2	2.01	0.60
2:B:588:ILE:HG21	2:B:618:PHE:HE1	1.66	0.60
1:A:233:PHE:C	1:A:235:GLN:N	2.50	0.60
2:B:189:HIS:NE2	2:B:193:LEU:CD1	2.60	0.60
2:B:278:PRO:CG	2:B:292:GLU:HB2	2.30	0.60
3:M:6:TYR:N	3:M:6:TYR:CD1	2.69	0.60
3:M:235:LEU:HD23	3:M:235:LEU:C	2.22	0.60
3:M:258:VAL:HG13	3:M:449:VAL:CG1	2.30	0.60
3:M:284:SER:O	3:M:286:SER:N	2.33	0.60
3:M:372:ILE:O	3:M:372:ILE:HG22	2.02	0.60
1:A:244:LEU:HA	1:A:256:LEU:HD13	1.82	0.60
2:B:20:ARG:HD2	2:B:21:GLU:HB2	1.73	0.60
2:B:396:ILE:HD11	2:B:418:TYR:CE2	2.37	0.60
3:M:118:TYR:C	3:M:118:TYR:CD2	2.73	0.60
1:A:552:ILE:HG12	1:A:585:PHE:CE1	2.37	0.60
3:M:222:PHE:CE1	3:M:240:ILE:CG2	2.84	0.60

Continued on next page...

Continued from previous page...

Atom-1	Atom-2	Interatomic distance (Å)	Clash overlap (Å)
3:M:383:HIS:HB3	3:M:403:THR:OG1	2.01	0.60
1:A:100:LEU:O	1:A:101:GLN:O	2.20	0.60
1:A:349:ILE:CG2	1:A:378:ILE:CG2	2.77	0.60
1:A:398:GLU:HA	1:A:418:ILE:HG13	1.84	0.60
1:A:535:ILE:O	1:A:535:ILE:HG22	2.01	0.60
2:B:296:ASP:OD1	2:B:297:PRO:CD	2.49	0.60
4:S:70:ASN:HD22	4:S:73:ILE:HD12	1.66	0.60
1:A:200:PHE:CE1	1:A:236:LEU:HD11	2.37	0.60
1:A:251:TRP:CZ3	4:S:96:LEU:O	2.54	0.60
1:A:563:CYS:O	1:A:566:PHE:CD2	2.55	0.60
1:A:569:ASP:C	1:A:571:ARG:N	2.54	0.60
1:A:602:GLU:OE2	1:A:633:PHE:HE1	1.82	0.60
2:B:21:GLU:OE2	2:B:35:TYR:O	2.19	0.60
2:B:452:LYS:NZ	2:B:456:ASP:OD2	2.34	0.60
2:B:497:LEU:HD21	2:B:533:LEU:CD2	2.32	0.60
2:B:563:PHE:CD2	2:B:584:SER:HB2	2.36	0.60
3:M:44:ASP:CB	3:M:50:TYR:CD2	2.85	0.60
3:M:217:ASP:C	3:M:472:TYR:CZ	2.75	0.60
3:M:243:ILE:HB	3:M:473:LYS:O	2.01	0.60
3:M:288:ILE:CD1	3:M:300:LEU:HD22	2.32	0.60
1:A:605:GLU:OE2	1:A:632:PHE:CE2	2.54	0.60
1:A:621:LEU:HD13	1:A:621:LEU:C	2.22	0.60
2:B:266:VAL:HG11	2:B:275:ARG:HB3	1.83	0.60
2:B:519:ALA:O	2:B:523:PHE:CA	2.49	0.60
2:B:580:TYR:O	2:B:582:ASP:CG	2.39	0.60
2:B:602:ASP:O	2:B:608:ARG:NH2	2.35	0.60
3:M:243:ILE:HD13	3:M:301:GLU:HB3	1.84	0.60
2:B:24:ALA:C	2:B:35:TYR:CG	2.75	0.60
2:B:83:PHE:CZ	2:B:105:LEU:HG	2.37	0.60
2:B:108:PHE:CE2	2:B:115:LEU:CB	2.85	0.60
2:B:219:TYR:HD1	2:B:226:LEU:HD22	1.58	0.60
2:B:340:ILE:HG13	2:B:373:LEU:HD23	1.82	0.60
2:B:346:THR:O	2:B:349:MET:N	2.34	0.60
2:B:394:TRP:HZ3	2:B:397:GLN:OE1	1.83	0.60
2:B:463:LEU:HD13	2:B:467:VAL:HG11	1.83	0.60
2:B:577:ASN:O	2:B:578:PRO:O	2.19	0.60
2:B:105:LEU:HG	2:B:119:SER:CB	2.29	0.59
2:B:136:CYS:HB3	2:B:172:GLU:HG2	1.82	0.59
2:B:176:ALA:C	2:B:178:ILE:N	2.55	0.59
2:B:197:LYS:CB	2:B:229:HIS:CD2	2.85	0.59
2:B:223:LEU:HD11	2:B:258:GLN:CA	2.31	0.59

Continued on next page...

Continued from previous page...

Atom-1	Atom-2	Interatomic distance (Å)	Clash overlap (Å)
2:B:241:ASP:CB	3:M:274:ASP:CA	2.77	0.59
2:B:243:TRP:CZ2	3:M:95:THR:HA	2.37	0.59
2:B:602:ASP:O	2:B:608:ARG:CZ	2.50	0.59
1:A:266:VAL:O	1:A:267:GLU:HB2	2.02	0.59
2:B:136:CYS:HA	2:B:172:GLU:HB2	1.83	0.59
2:B:165:PRO:HA	2:B:170:ARG:HH21	1.67	0.59
3:M:316:ARG:CG	3:M:322:LEU:HD13	2.31	0.59
3:M:319:SER:HB3	3:M:343:ASN:O	2.02	0.59
4:S:6:LEU:HD21	4:S:36:TYR:CE1	2.37	0.59
1:A:244:LEU:CD2	1:A:277:LYS:O	2.49	0.59
1:A:513:ARG:CD	1:A:550:VAL:HG21	2.28	0.59
2:B:261:PRO:HG2	2:B:292:GLU:CB	2.31	0.59
2:B:479:VAL:HG13	2:B:486:HIS:CD2	2.24	0.59
2:B:508:ARG:O	2:B:509:ALA:C	2.30	0.59
3:M:99:ILE:CG2	3:M:124:ILE:CG2	2.81	0.59
3:M:262:THR:HG22	3:M:265:ASN:H	1.67	0.59
2:B:2:VAL:CG1	2:B:6:HIS:NE2	2.66	0.59
2:B:24:ALA:CA	2:B:35:TYR:CD2	2.86	0.59
2:B:25:VAL:HG22	2:B:35:TYR:HB3	1.85	0.59
2:B:37:TYR:CE2	2:B:42:ILE:HA	2.37	0.59
2:B:70:MET:HE1	2:B:107:ARG:HB2	0.63	0.59
2:B:305:SER:O	2:B:309:LEU:HD23	2.02	0.59
2:B:374:PHE:CB	2:B:402:LEU:HD21	2.32	0.59
2:B:418:TYR:HD1	2:B:424:PHE:CD1	2.19	0.59
2:B:525:ILE:C	2:B:527:PRO:CD	2.71	0.59
2:B:560:ILE:CA	2:B:563:PHE:HB2	2.32	0.59
2:B:584:SER:O	2:B:585:GLY:C	2.25	0.59
3:M:71:LYS:O	3:M:74:TYR:CD2	2.55	0.59
2:B:172:GLU:O	2:B:174:ALA:N	2.36	0.59
2:B:261:PRO:CD	2:B:293:VAL:CG2	2.75	0.59
2:B:461:HIS:O	2:B:462:ASN:O	2.21	0.59
2:B:490:ILE:HG13	2:B:518:ILE:HG12	1.84	0.59
2:B:523:PHE:CD1	2:B:559:ASP:CG	2.75	0.59
3:M:100:LEU:HB3	3:M:109:LEU:CD2	2.32	0.59
3:M:128:CYS:C	3:M:130:GLU:O	2.41	0.59
4:S:5:VAL:HG13	4:S:87:PHE:CE2	2.37	0.59
4:S:87:PHE:CZ	4:S:102:ILE:HG12	2.37	0.59
4:S:126:GLN:NE2	4:S:127:THR:OG1	2.36	0.59
1:A:186:PHE:CD2	1:A:186:PHE:C	2.75	0.59
1:A:582:ILE:HG23	1:A:604:LEU:HG	1.84	0.59
2:B:167:ALA:O	2:B:207:VAL:CG1	2.51	0.59

Continued on next page...

Continued from previous page...

Atom-1	Atom-2	Interatomic distance (Å)	Clash overlap (Å)
2:B:303:LEU:HD11	2:B:333:GLN:HB3	1.84	0.59
2:B:519:ALA:HB1	2:B:555:LEU:HD12	1.83	0.59
1:A:289:SER:HB3	4:S:96:LEU:HB3	1.85	0.59
1:A:599:ARG:CZ	2:B:513:TRP:CE3	2.86	0.59
2:B:37:TYR:O	2:B:38:TYR:C	2.40	0.59
2:B:231:ARG:O	2:B:233:TYR:N	2.36	0.59
2:B:473:ASN:O	2:B:476:ARG:HB3	2.02	0.59
2:B:592:TYR:C	2:B:592:TYR:CD1	2.76	0.59
2:B:599:ALA:O	2:B:601:TYR:N	2.36	0.59
3:M:306:LEU:CD1	3:M:317:MET:HE3	2.32	0.59
4:S:17:VAL:HG21	4:S:19:PHE:CE2	2.38	0.59
1:A:150:LEU:HB3	1:A:162:ILE:CD1	2.33	0.59
1:A:553:LEU:HD22	2:B:610:ARG:HH21	1.67	0.59
2:B:291:TYR:HE2	2:B:294:VAL:HB	1.68	0.59
3:M:16:PHE:CE1	3:M:122:SER:HB2	2.32	0.59
3:M:245:ASP:N	3:M:472:TYR:CZ	2.71	0.59
3:M:338:PHE:CD2	3:M:415:ILE:CG1	2.85	0.59
2:B:37:TYR:CD2	2:B:38:TYR:HD1	2.20	0.59
2:B:256:CYS:SG	2:B:299:LEU:HD23	2.43	0.59
3:M:58:ARG:C	3:M:60:LEU:O	2.41	0.59
3:M:66:PHE:HA	3:M:78:ALA:O	2.03	0.59
3:M:95:THR:OG1	3:M:137:SER:CB	2.43	0.59
4:S:31:LEU:O	4:S:35:VAL:HG13	2.03	0.59
1:A:461:CYS:SG	1:A:469:LEU:HB3	2.42	0.59
2:B:2:VAL:HA	2:B:5:ILE:HD12	1.84	0.59
2:B:549:LEU:HD23	2:B:610:ARG:HB2	1.85	0.59
3:M:217:ASP:HB2	3:M:470:ALA:O	2.03	0.59
1:A:156:PRO:O	1:A:157:SER:C	2.31	0.58
1:A:203:PHE:CE2	1:A:221:VAL:HG11	2.38	0.58
1:A:464:ILE:HG23	1:A:465:SER:HA	1.85	0.58
1:A:570:LYS:O	1:A:571:ARG:HB2	2.03	0.58
2:B:230:PHE:HD2	2:B:298:ASP:CB	2.12	0.58
2:B:267:ASP:O	2:B:269:SER:N	2.36	0.58
3:M:214:LEU:HD11	3:M:452:ILE:HG21	1.85	0.58
3:M:316:ARG:O	3:M:317:MET:C	2.41	0.58
3:M:376:ILE:HG21	3:M:379:LEU:CD1	2.32	0.58
3:M:379:LEU:HB3	3:M:411:LEU:HD11	1.84	0.58
4:S:17:VAL:HG21	4:S:19:PHE:HZ	1.63	0.58
1:A:279:LEU:HD11	1:A:314:ALA:HB1	1.85	0.58
1:A:429:VAL:HG11	1:A:473:ILE:HD11	1.85	0.58
1:A:609:LEU:CD2	1:A:628:VAL:CG1	2.71	0.58

Continued on next page...

Continued from previous page...

Atom-1	Atom-2	Interatomic distance (Å)	Clash overlap (Å)
2:B:219:TYR:CD2	2:B:226:LEU:CB	2.77	0.58
2:B:352:ASN:ND2	3:M:48:ASP:O	2.36	0.58
3:M:245:ASP:O	3:M:246:VAL:HG22	2.03	0.58
3:M:302:TYR:CE2	3:M:445:SER:CB	2.86	0.58
3:M:317:MET:HB2	3:M:322:LEU:H	1.67	0.58
3:M:374:TYR:HB3	3:M:417:TYR:HD2	1.68	0.58
3:M:403:THR:CG2	3:M:407:THR:OG1	2.52	0.58
1:A:107:TYR:CD2	1:A:128:LEU:CD2	2.86	0.58
2:B:321:CYS:O	2:B:325:LEU:HG	2.03	0.58
2:B:353:GLN:C	3:M:49:ASP:HB3	1.62	0.58
2:B:374:PHE:CZ	2:B:381:PHE:CE1	2.92	0.58
3:M:215:TYR:HB2	3:M:467:TYR:C	2.24	0.58
1:A:170:LEU:HB3	1:A:206:LYS:HD2	1.85	0.58
1:A:291:ILE:HG21	1:A:322:PHE:CE1	2.39	0.58
1:A:303:MET:HG2	1:A:308:ASP:OD2	2.03	0.58
1:A:556:VAL:HG22	1:A:603:VAL:HG13	1.85	0.58
2:B:123:LEU:O	2:B:127:LEU:CG	2.48	0.58
2:B:334:MET:O	2:B:335:LYS:C	2.38	0.58
2:B:479:VAL:HG22	2:B:486:HIS:CG	2.37	0.58
2:B:545:ARG:CD	2:B:602:ASP:OD2	2.52	0.58
2:B:563:PHE:CD1	2:B:588:ILE:HD12	2.38	0.58
3:M:244:VAL:O	3:M:299:LEU:HB3	2.04	0.58
3:M:341:SER:OG	3:M:343:ASN:ND2	2.35	0.58
4:S:39:ILE:HG23	4:S:47:GLN:CD	2.23	0.58
1:A:260:PHE:CE1	1:A:274:LEU:HD12	2.39	0.58
1:A:512:LEU:HD13	1:A:543:TYR:CE1	2.39	0.58
1:A:516:ILE:HD13	1:A:551:LEU:HA	1.84	0.58
2:B:261:PRO:C	2:B:290:SER:HB3	2.24	0.58
2:B:325:LEU:HB3	2:B:334:MET:SD	2.43	0.58
2:B:475:ILE:HG22	2:B:514:LEU:CD2	2.33	0.58
2:B:486:HIS:NE2	2:B:490:ILE:HD11	2.17	0.58
3:M:216:VAL:O	3:M:216:VAL:HG23	2.03	0.58
3:M:217:ASP:O	3:M:472:TYR:CD1	2.57	0.58
3:M:257:ALA:HB3	3:M:453:ASP:CG	2.23	0.58
3:M:290:PHE:CE2	3:M:297:PHE:CE1	2.91	0.58
3:M:437:TYR:CE1	3:M:479:PHE:CE1	2.91	0.58
4:S:8:PHE:CE2	4:S:86:THR:OG1	2.45	0.58
4:S:16:LEU:HD23	4:S:128:LEU:CD2	2.33	0.58
1:A:71:VAL:CG1	1:A:105:VAL:HG12	2.33	0.58
1:A:505:ASN:O	1:A:506:LYS:C	2.38	0.58
1:A:531:ASP:O	1:A:534:LYS:O	2.20	0.58

Continued on next page...

Continued from previous page...

Atom-1	Atom-2	Interatomic distance (Å)	Clash overlap (Å)
1:A:556:VAL:CG2	1:A:603:VAL:HG11	2.33	0.58
2:B:396:ILE:CD1	2:B:418:TYR:CE2	2.86	0.58
3:M:48:ASP:O	3:M:75:TRP:CZ2	2.57	0.58
3:M:245:ASP:HB3	3:M:472:TYR:CE1	2.35	0.58
3:M:276:VAL:CG2	3:M:299:LEU:HD12	2.33	0.58
3:M:433:VAL:CG1	3:M:481:VAL:HB	2.33	0.58
4:S:47:GLN:HG2	4:S:84:TYR:CE2	2.39	0.58
1:A:240:LEU:C	1:A:242:GLU:O	2.42	0.58
1:A:429:VAL:HG11	1:A:469:LEU:HD11	1.83	0.58
2:B:37:TYR:O	2:B:40:GLN:C	2.41	0.58
2:B:141:ALA:O	2:B:143:SER:N	2.37	0.58
2:B:162:VAL:CG2	2:B:199:LEU:CD1	2.81	0.58
2:B:178:ILE:HG22	2:B:179:LYS:N	2.17	0.58
2:B:374:PHE:HE2	2:B:402:LEU:CD1	2.05	0.58
3:M:16:PHE:CA	3:M:118:TYR:CD1	2.84	0.58
3:M:99:ILE:CD1	3:M:128:CYS:SG	2.92	0.58
3:M:315:VAL:O	3:M:315:VAL:HG12	2.03	0.58
1:A:85:ALA:O	1:A:88:ASN:HB2	2.03	0.58
1:A:176:TYR:CD1	4:S:143:GLU:OE2	2.55	0.58
2:B:136:CYS:SG	2:B:168:MET:O	2.62	0.58
2:B:278:PRO:CA	2:B:288:TYR:HB3	2.20	0.58
4:S:105:PHE:CZ	4:S:128:LEU:HD11	2.39	0.58
1:A:80:TYR:HB2	1:A:82:PHE:CD2	2.39	0.58
1:A:186:PHE:CA	1:A:221:VAL:HG13	2.34	0.58
1:A:520:GLY:HA2	1:A:558:VAL:CG2	2.34	0.58
1:A:630:PRO:CG	2:B:617:LEU:HD11	1.98	0.58
2:B:415:LEU:O	2:B:418:TYR:N	2.33	0.58
2:B:418:TYR:OH	2:B:432:ALA:CB	2.51	0.58
2:B:553:ALA:CB	2:B:614:ILE:CD1	2.70	0.58
3:M:100:LEU:HD11	3:M:121:ILE:HG23	1.86	0.58
3:M:218:LEU:N	3:M:218:LEU:HD12	2.18	0.58
3:M:223:HIS:HE1	3:M:476:THR:H	1.52	0.58
4:S:14:PRO:HA	4:S:36:TYR:OH	2.03	0.58
4:S:93:GLU:OE1	4:S:93:GLU:HA	2.04	0.58
1:A:204:VAL:HG13	1:A:239:LEU:HD11	1.81	0.58
1:A:260:PHE:CD2	1:A:274:LEU:CG	2.81	0.58
1:A:274:LEU:O	1:A:277:LYS:N	2.36	0.58
1:A:609:LEU:CG	1:A:628:VAL:HB	2.31	0.58
2:B:197:LYS:N	2:B:229:HIS:CE1	2.72	0.58
2:B:469:ASP:OD1	2:B:506:ASN:HB2	2.04	0.58
2:B:563:PHE:O	2:B:566:ALA:CB	2.49	0.58

Continued on next page...

Continued from previous page...

Atom-1	Atom-2	Interatomic distance (Å)	Clash overlap (Å)
3:M:265:ASN:HA	3:M:313:SER:HG	1.67	0.58
3:M:379:LEU:HD22	3:M:397:TRP:CE2	2.39	0.58
1:A:78:GLU:O	1:A:80:TYR:O	2.21	0.57
1:A:114:PHE:C	1:A:115:TYR:O	2.37	0.57
1:A:244:LEU:HD11	1:A:281:LEU:HD12	1.84	0.57
1:A:323:CYS:SG	1:A:355:LEU:HD21	2.43	0.57
2:B:90:ILE:HD11	2:B:102:HIS:CD2	2.38	0.57
2:B:137:PHE:O	2:B:140:SER:HB3	2.04	0.57
2:B:253:ILE:HG12	2:B:324:ALA:CB	2.34	0.57
2:B:299:LEU:C	2:B:299:LEU:HD13	2.24	0.57
3:M:134:PRO:O	3:M:136:VAL:CA	2.52	0.57
3:M:214:LEU:C	3:M:467:TYR:H	2.04	0.57
4:S:157:ASN:O	4:S:161:GLU:HG3	2.04	0.57
1:A:71:VAL:HG12	1:A:105:VAL:HG12	1.84	0.57
1:A:147:LEU:HD22	1:A:166:LEU:HD23	1.84	0.57
1:A:247:ILE:HG21	1:A:252:ILE:CG2	2.33	0.57
1:A:391:LEU:O	1:A:392:MET:O	2.22	0.57
1:A:516:ILE:HD13	1:A:551:LEU:CA	2.34	0.57
2:B:161:LEU:HB3	2:B:173:VAL:HG21	1.83	0.57
2:B:513:TRP:CB	2:B:551:LEU:HD21	2.34	0.57
3:M:293:PRO:HB2	3:M:294:ASP:O	2.03	0.57
3:M:374:TYR:CB	3:M:417:TYR:CD2	2.87	0.57
2:B:177:ILE:HB	2:B:196:LEU:HD21	1.87	0.57
2:B:344:VAL:HG13	2:B:381:PHE:CZ	2.39	0.57
2:B:526:CYS:N	2:B:527:PRO:HD3	2.17	0.57
2:B:545:ARG:CD	2:B:602:ASP:CB	2.76	0.57
4:S:53:THR:CG2	4:S:67:GLU:O	2.52	0.57
4:S:109:LEU:CD1	4:S:113:PHE:CD1	2.87	0.57
1:A:190:LEU:HD11	1:A:228:LYS:HE3	1.86	0.57
1:A:555:LEU:HD13	1:A:581:LEU:HD13	1.81	0.57
2:B:268:LYS:CA	2:B:276:SER:HB2	2.33	0.57
2:B:310:ILE:HG23	2:B:318:ILE:HG23	1.87	0.57
2:B:353:GLN:C	2:B:355:ASN:N	2.48	0.57
2:B:546:CYS:N	2:B:607:ILE:HD13	2.19	0.57
3:M:364:VAL:C	3:M:367:ALA:O	2.43	0.57
1:A:480:LEU:O	1:A:483:LYS:O	2.22	0.57
2:B:178:ILE:C	2:B:180:LEU:N	2.58	0.57
2:B:243:TRP:CZ2	3:M:98:ARG:NE	2.71	0.57
2:B:267:ASP:H	2:B:289:PRO:HB3	1.68	0.57
2:B:277:CYS:SG	2:B:292:GLU:HG3	2.45	0.57
2:B:472:VAL:CG1	2:B:510:GLY:C	2.73	0.57

Continued on next page...

Continued from previous page...

Atom-1	Atom-2	Interatomic distance (Å)	Clash overlap (Å)
3:M:338:PHE:CD2	3:M:415:ILE:CD1	2.87	0.57
1:A:326:GLN:HA	1:A:331:ARG:HH21	1.68	0.57
1:A:371:ALA:O	1:A:374:LEU:HB2	2.04	0.57
1:A:464:ILE:O	2:B:1:MET:SD	2.62	0.57
2:B:140:SER:O	2:B:143:SER:N	2.35	0.57
2:B:141:ALA:C	2:B:143:SER:N	2.55	0.57
2:B:185:LYS:HE2	2:B:221:ASP:OD2	2.04	0.57
2:B:219:TYR:CE1	2:B:226:LEU:HA	2.33	0.57
2:B:512:VAL:HG13	2:B:533:LEU:HD13	1.86	0.57
2:B:560:ILE:CG2	2:B:564:LYS:HB2	2.32	0.57
4:S:60:SER:O	4:S:66:ASP:HB2	2.03	0.57
1:A:297:CYS:O	1:A:298:ILE:C	2.40	0.57
1:A:373:GLU:HG2	1:A:427:LYS:HE2	1.86	0.57
1:A:638:LEU:CD2	2:B:558:TYR:CA	2.50	0.57
2:B:37:TYR:CD2	2:B:38:TYR:CD1	2.90	0.57
2:B:166:SER:O	2:B:170:ARG:HG3	2.04	0.57
2:B:188:TYR:HB3	2:B:192:LEU:HD13	1.86	0.57
2:B:269:SER:C	2:B:270:SER:O	2.39	0.57
2:B:409:LYS:O	2:B:413:LYS:HG3	2.04	0.57
2:B:431:MET:O	2:B:433:VAL:N	2.38	0.57
2:B:482:ASN:N	2:B:483:PRO:HD3	2.20	0.57
3:M:97:ASP:O	3:M:100:LEU:HB2	2.04	0.57
3:M:374:TYR:CE1	3:M:376:ILE:HD11	2.40	0.57
1:A:170:LEU:O	1:A:206:LYS:HD2	2.04	0.57
1:A:185:LEU:HB2	1:A:203:PHE:CZ	2.40	0.57
1:A:244:LEU:HD23	1:A:277:LYS:HG3	1.86	0.57
1:A:298:ILE:CD1	1:A:311:THR:CG2	2.83	0.57
1:A:429:VAL:HG21	1:A:469:LEU:HD21	1.87	0.57
2:B:293:VAL:O	2:B:299:LEU:HB3	2.04	0.57
2:B:519:ALA:CB	2:B:555:LEU:HD13	2.35	0.57
2:B:567:GLN:N	2:B:574:ASN:ND2	2.52	0.57
3:M:319:SER:CB	3:M:343:ASN:O	2.53	0.57
3:M:419:ASN:OD1	3:M:424:PHE:CD2	2.57	0.57
1:A:121:LEU:HD23	1:A:121:LEU:C	2.24	0.57
1:A:154:ILE:HG21	1:A:191:GLN:CG	2.34	0.57
1:A:323:CYS:HB2	1:A:355:LEU:HD21	1.87	0.57
2:B:285:GLU:O	2:B:286:ILE:O	2.21	0.57
2:B:418:TYR:CD1	2:B:419:VAL:CA	2.88	0.57
2:B:520:SER:HB3	2:B:558:TYR:CD2	2.40	0.57
2:B:588:ILE:CG2	2:B:618:PHE:CZ	2.81	0.57
3:M:354:ASP:O	3:M:438:SER:O	2.22	0.57

Continued on next page...

Continued from previous page...

Atom-1	Atom-2	Interatomic distance (Å)	Clash overlap (Å)
4:S:131:VAL:HG22	4:S:153:VAL:CG2	2.25	0.57
1:A:114:PHE:O	1:A:115:TYR:O	2.22	0.57
1:A:253:ILE:CD1	1:A:285:THR:HG21	2.34	0.57
2:B:103:LEU:CD2	3:M:131:ALA:O	2.51	0.57
2:B:307:ASN:OD1	2:B:339:PHE:CE2	2.57	0.57
2:B:361:GLN:O	2:B:364:HIS:HB3	2.05	0.57
3:M:4:SER:HB3	3:M:79:SER:HG	1.68	0.57
3:M:224:VAL:HG22	3:M:306:LEU:CD1	2.35	0.57
3:M:323:MET:HE1	3:M:342:LEU:HB3	1.87	0.57
4:S:110:ASP:O	4:S:114:THR:HA	2.05	0.57
1:A:224:GLU:O	1:A:225:LEU:C	2.38	0.56
1:A:322:PHE:HB3	1:A:330:LEU:HD21	1.86	0.56
1:A:429:VAL:CG2	1:A:469:LEU:HD11	2.33	0.56
2:B:185:LYS:O	2:B:186:ASN:C	2.23	0.56
2:B:234:CYS:CB	2:B:301:LEU:CB	2.82	0.56
2:B:343:LEU:HD21	2:B:362:ALA:CB	2.35	0.56
3:M:69:ILE:HG21	3:M:97:ASP:OD2	2.05	0.56
3:M:375:LYS:HE3	3:M:418:GLU:OE1	2.05	0.56
3:M:378:ILE:O	3:M:413:GLY:HA2	2.03	0.56
1:A:260:PHE:O	1:A:262:ASN:N	2.35	0.56
1:A:268:PRO:HG3	1:A:271:ARG:HH21	1.69	0.56
1:A:300:LYS:C	1:A:302:ASN:N	2.49	0.56
1:A:402:ILE:HG12	1:A:406:GLY:HA2	1.86	0.56
1:A:498:LEU:HB3	1:A:504:ILE:HG13	1.85	0.56
1:A:563:CYS:HA	1:A:566:PHE:HD2	1.68	0.56
2:B:18:ILE:H	2:B:18:ILE:HD13	1.70	0.56
2:B:42:ILE:HG22	2:B:43:ASN:O	2.05	0.56
2:B:227:HIS:O	2:B:298:ASP:OD2	2.22	0.56
2:B:355:ASN:C	2:B:359:LEU:HD23	2.25	0.56
3:M:220:GLU:OE1	3:M:443:SER:O	2.23	0.56
3:M:302:TYR:CE2	3:M:445:SER:HB2	2.40	0.56
1:A:114:PHE:CE1	1:A:153:ILE:HA	2.38	0.56
1:A:229:ASN:O	1:A:230:PRO:C	2.35	0.56
1:A:441:TYR:C	1:A:443:SER:N	2.56	0.56
1:A:450:TYR:CE1	1:A:454:ILE:HD11	2.41	0.56
1:A:609:LEU:CG	1:A:628:VAL:CB	2.82	0.56
2:B:108:PHE:HE2	2:B:112:ASP:HB3	1.69	0.56
2:B:127:LEU:HD23	2:B:135:ARG:O	2.05	0.56
2:B:247:TYR:HE1	3:M:137:SER:OG	1.82	0.56
2:B:454:LEU:O	2:B:457:HIS:HB2	2.05	0.56
3:M:217:ASP:N	3:M:472:TYR:CZ	2.73	0.56

Continued on next page...

Continued from previous page...

Atom-1	Atom-2	Interatomic distance (Å)	Clash overlap (Å)
3:M:217:ASP:O	3:M:472:TYR:CE2	2.58	0.56
3:M:235:LEU:HD11	3:M:306:LEU:HD13	1.87	0.56
3:M:386:PHE:HB2	3:M:397:TRP:CD1	2.40	0.56
4:S:16:LEU:HD22	4:S:128:LEU:HD23	1.85	0.56
4:S:164:ASP:O	4:S:165:SER:C	2.29	0.56
1:A:101:GLN:OE1	4:S:167:ILE:HD12	2.01	0.56
1:A:318:ARG:O	1:A:322:PHE:HD1	1.88	0.56
1:A:515:CYS:O	1:A:519:LEU:HG	2.05	0.56
1:A:556:VAL:HG21	1:A:603:VAL:CG1	2.34	0.56
2:B:162:VAL:CG1	2:B:195:ILE:HG23	2.36	0.56
2:B:200:MET:CG	2:B:232:ARG:HB3	2.27	0.56
2:B:241:ASP:HB2	3:M:274:ASP:CA	2.35	0.56
2:B:267:ASP:CA	2:B:289:PRO:HG3	2.35	0.56
2:B:453:TRP:HE3	2:B:453:TRP:HA	1.71	0.56
3:M:7:ILE:HA	3:M:75:TRP:O	2.05	0.56
3:M:222:PHE:HB2	3:M:479:PHE:CZ	2.39	0.56
3:M:224:VAL:O	3:M:480:GLN:N	2.36	0.56
3:M:372:ILE:HD12	3:M:428:VAL:HG22	1.85	0.56
1:A:125:THR:CG2	1:A:158:LEU:CD1	2.84	0.56
1:A:154:ILE:HG22	1:A:191:GLN:HG3	1.84	0.56
1:A:202:LYS:O	1:A:205:SER:N	2.39	0.56
1:A:295:VAL:HG11	1:A:337:LEU:HD13	1.86	0.56
1:A:332:TYR:OH	1:A:336:ILE:HD11	2.03	0.56
2:B:69:ILE:O	2:B:70:MET:C	2.39	0.56
2:B:171:GLY:HA3	2:B:207:VAL:HA	1.86	0.56
3:M:350:VAL:CB	3:M:442:GLN:HG2	2.35	0.56
4:S:9:ASN:ND2	4:S:118:GLU:OE1	2.39	0.56
2:B:103:LEU:HD21	3:M:131:ALA:C	2.25	0.56
2:B:151:ALA:CB	2:B:180:LEU:CD1	2.81	0.56
2:B:182:ARG:CG	2:B:217:GLU:HB3	2.35	0.56
2:B:215:TYR:HB3	2:B:226:LEU:CD1	2.36	0.56
2:B:252:LEU:CB	2:B:302:PHE:CD1	2.89	0.56
2:B:311:TYR:HB2	3:M:269:ILE:HD13	1.87	0.56
2:B:347:VAL:HG22	2:B:359:LEU:HD12	1.87	0.56
1:A:254:ILE:HG12	1:A:290:VAL:HA	1.86	0.56
1:A:292:TYR:CD1	1:A:292:TYR:C	2.78	0.56
1:A:633:PHE:HE2	2:B:554:LYS:HB2	1.65	0.56
2:B:171:GLY:CA	2:B:207:VAL:CG1	2.73	0.56
2:B:292:GLU:HG3	2:B:296:ASP:HB2	1.88	0.56
2:B:453:TRP:HA	2:B:453:TRP:CE3	2.41	0.56
2:B:550:VAL:CG2	2:B:610:ARG:CD	2.83	0.56

Continued on next page...

Continued from previous page...

Atom-1	Atom-2	Interatomic distance (Å)	Clash overlap (Å)
4:S:8:PHE:N	4:S:8:PHE:HD2	2.03	0.56
1:A:121:LEU:CD1	1:A:153:ILE:CG2	2.83	0.56
1:A:128:LEU:CD1	1:A:150:LEU:HD23	2.36	0.56
1:A:532:LEU:O	1:A:533:ILE:O	2.24	0.56
2:B:90:ILE:HD11	2:B:123:LEU:CD2	2.36	0.56
2:B:139:LEU:HD23	2:B:172:GLU:C	2.25	0.56
2:B:276:SER:OG	2:B:289:PRO:CG	2.53	0.56
2:B:355:ASN:O	2:B:358:MET:N	2.38	0.56
3:M:217:ASP:CB	3:M:470:ALA:O	2.54	0.56
3:M:443:SER:HB3	3:M:447:ILE:HG12	1.80	0.56
3:M:461:GLY:C	3:M:462:LYS:O	2.27	0.56
4:S:8:PHE:CD2	4:S:84:TYR:O	2.58	0.56
1:A:190:LEU:CD1	1:A:228:LYS:HG3	2.31	0.56
1:A:384:LEU:HD12	1:A:385:LYS:N	2.20	0.56
2:B:57:ARG:O	2:B:60:ARG:HB3	2.05	0.56
2:B:127:LEU:CB	2:B:157:THR:CG2	2.81	0.56
2:B:502:SER:O	2:B:503:LEU:C	2.39	0.56
2:B:523:PHE:CE2	2:B:580:TYR:CE1	2.94	0.56
2:B:589:SER:HG	2:B:618:PHE:HZ	1.45	0.56
3:M:319:SER:HA	3:M:343:ASN:O	2.05	0.56
3:M:376:ILE:HG22	3:M:379:LEU:HD12	1.88	0.56
4:S:38:LEU:HB3	4:S:51:LEU:HD13	1.88	0.56
1:A:420:ILE:HG23	1:A:424:TYR:CB	2.33	0.56
1:A:606:PHE:CD2	1:A:629:LEU:HD11	2.41	0.56
2:B:42:ILE:HG22	2:B:43:ASN:N	2.20	0.56
2:B:175:LEU:O	2:B:178:ILE:HB	2.06	0.56
2:B:197:LYS:O	2:B:199:LEU:N	2.39	0.56
2:B:247:TYR:CE1	3:M:136:VAL:HG13	2.41	0.56
2:B:404:ASN:O	2:B:408:VAL:HG23	2.06	0.56
2:B:523:PHE:CE1	2:B:580:TYR:HB2	2.17	0.56
3:M:244:VAL:CA	3:M:472:TYR:CE2	2.88	0.56
3:M:478:ASN:O	3:M:479:PHE:C	2.42	0.56
1:A:186:PHE:CB	1:A:221:VAL:HG13	2.35	0.55
1:A:244:LEU:CA	1:A:256:LEU:HD13	2.35	0.55
1:A:292:TYR:CE1	1:A:296:ASN:HB2	2.42	0.55
1:A:441:TYR:O	1:A:442:SER:C	2.43	0.55
2:B:50:LEU:HG	2:B:58:GLU:O	2.06	0.55
2:B:167:ALA:HB1	2:B:202:ASP:OD2	2.05	0.55
2:B:212:VAL:HG11	2:B:248:LEU:HD23	1.87	0.55
2:B:340:ILE:HG13	2:B:373:LEU:CG	2.37	0.55
3:M:253:ASN:OD1	3:M:292:PRO:CG	2.54	0.55

Continued on next page...

Continued from previous page...

Atom-1	Atom-2	Interatomic distance (Å)	Clash overlap (Å)
1:A:225:LEU:CG	1:A:233:PHE:CZ	2.90	0.55
2:B:219:TYR:CG	2:B:226:LEU:CD2	2.83	0.55
2:B:306:LEU:HD12	2:B:325:LEU:CD2	2.37	0.55
2:B:604:GLU:O	2:B:607:ILE:HB	2.06	0.55
3:M:41:LEU:HB3	3:M:50:TYR:O	2.06	0.55
1:A:64:LEU:HA	1:A:102:GLN:HE22	1.70	0.55
1:A:128:LEU:HB2	1:A:150:LEU:HD21	1.87	0.55
1:A:332:TYR:CE2	1:A:336:ILE:HD12	2.39	0.55
1:A:438:ALA:O	1:A:439:ASP:CB	2.54	0.55
2:B:50:LEU:HD23	2:B:62:ALA:HB2	1.89	0.55
2:B:139:LEU:HD11	2:B:158:VAL:HG22	1.88	0.55
2:B:141:ALA:O	2:B:144:ASP:N	2.40	0.55
2:B:155:LEU:HG	2:B:188:TYR:HD2	1.70	0.55
2:B:278:PRO:HA	2:B:288:TYR:O	2.00	0.55
2:B:431:MET:C	2:B:433:VAL:H	2.10	0.55
2:B:523:PHE:O	2:B:524:LYS:C	2.36	0.55
2:B:559:ASP:HB2	2:B:563:PHE:HD1	1.58	0.55
3:M:220:GLU:HG2	3:M:439:TYR:HB2	1.88	0.55
3:M:245:ASP:N	3:M:472:TYR:CE2	2.75	0.55
3:M:437:TYR:HD1	3:M:479:PHE:CZ	2.21	0.55
1:A:260:PHE:CD1	1:A:274:LEU:HD12	2.42	0.55
1:A:332:TYR:CE1	1:A:366:SER:OG	2.55	0.55
1:A:558:VAL:O	1:A:561:ASN:N	2.37	0.55
1:A:603:VAL:O	1:A:606:PHE:HB2	2.07	0.55
2:B:127:LEU:HD13	2:B:157:THR:HG21	0.59	0.55
2:B:230:PHE:CE2	2:B:298:ASP:C	2.76	0.55
2:B:231:ARG:NH2	2:B:297:PRO:HD2	2.21	0.55
2:B:537:PHE:CE2	2:B:598:LEU:HB3	2.37	0.55
2:B:572:GLU:O	2:B:575:ASN:N	2.34	0.55
3:M:9:ASP:HB3	3:M:15:ILE:HD11	1.88	0.55
3:M:215:TYR:CD1	3:M:467:TYR:O	2.59	0.55
3:M:215:TYR:HD1	3:M:467:TYR:O	1.89	0.55
3:M:347:PHE:CD1	3:M:350:VAL:CG1	2.88	0.55
4:S:55:PRO:HB3	4:S:71:GLU:CG	2.37	0.55
1:A:185:LEU:CD1	1:A:203:PHE:HE1	2.16	0.55
1:A:609:LEU:HD13	1:A:609:LEU:C	2.26	0.55
1:A:634:ASN:HB3	2:B:558:TYR:HD1	1.70	0.55
2:B:199:LEU:O	2:B:200:MET:C	2.44	0.55
3:M:219:LEU:HD22	3:M:474:THR:N	2.22	0.55
4:S:53:THR:O	4:S:69:ASN:CG	2.44	0.55
4:S:55:PRO:HG3	4:S:71:GLU:HG2	1.89	0.55

Continued on next page...

Continued from previous page...

Atom-1	Atom-2	Interatomic distance (Å)	Clash overlap (Å)
1:A:158:LEU:HG	1:A:162:ILE:HD11	1.89	0.55
2:B:237:ILE:HG13	2:B:248:LEU:HD12	1.87	0.55
2:B:262:LYS:O	2:B:264:THR:N	2.40	0.55
2:B:299:LEU:O	2:B:302:PHE:CB	2.52	0.55
4:S:38:LEU:CD2	4:S:68:VAL:CG2	2.85	0.55
1:A:102:GLN:HE21	4:S:166:LYS:HD2	1.69	0.55
1:A:370:LYS:O	1:A:374:LEU:HD12	2.05	0.55
2:B:87:VAL:HG13	2:B:122:SER:OG	2.07	0.55
2:B:249:ILE:CD1	2:B:321:CYS:SG	2.95	0.55
2:B:276:SER:C	2:B:295:ASN:ND2	2.49	0.55
3:M:377:LYS:NZ	3:M:416:GLU:OE1	2.34	0.55
4:S:43:ASN:HB3	4:S:46:PHE:CD1	2.42	0.55
1:A:219:VAL:HG13	1:A:256:LEU:HD23	1.85	0.55
2:B:252:LEU:HB3	2:B:302:PHE:CZ	2.27	0.55
2:B:531:ARG:CB	2:B:591:MET:SD	2.95	0.55
3:M:100:LEU:HD22	3:M:121:ILE:HG12	1.89	0.55
3:M:136:VAL:C	3:M:138:ASP:N	2.57	0.55
3:M:271:SER:N	3:M:301:GLU:O	2.31	0.55
3:M:288:ILE:HD12	3:M:300:LEU:CD2	2.37	0.55
4:S:4:ALA:HB2	4:S:19:PHE:HD2	1.72	0.55
1:A:84:MET:CE	1:A:113:SER:O	2.55	0.55
1:A:318:ARG:O	1:A:322:PHE:CD1	2.60	0.55
1:A:435:ILE:HG23	1:A:441:TYR:CE2	2.42	0.55
1:A:570:LYS:HD2	1:A:614:LEU:HB3	1.89	0.55
1:A:626:SER:O	1:A:630:PRO:CD	2.55	0.55
2:B:249:ILE:HG12	2:B:306:LEU:HD23	1.88	0.55
2:B:341:GLU:HG2	2:B:345:ARG:HE	1.72	0.55
2:B:515:PHE:HA	2:B:518:ILE:HG22	1.89	0.55
2:B:599:ALA:O	2:B:602:ASP:N	2.29	0.55
1:A:264:SER:CB	1:A:271:ARG:HD3	2.33	0.55
1:A:440:ASN:C	1:A:442:SER:H	2.09	0.55
2:B:162:VAL:O	2:B:163:THR:C	2.40	0.55
2:B:374:PHE:CD2	2:B:402:LEU:HD13	2.40	0.55
2:B:550:VAL:HG22	2:B:610:ARG:CD	2.26	0.55
1:A:401:VAL:CG2	1:A:418:ILE:N	2.70	0.54
1:A:465:SER:H	2:B:1:MET:HE1	1.70	0.54
2:B:103:LEU:HD21	3:M:131:ALA:CA	2.38	0.54
2:B:189:HIS:NE2	2:B:222:HIS:CB	2.69	0.54
2:B:223:LEU:HB3	2:B:259:TYR:HD1	0.75	0.54
2:B:332:LEU:HG	2:B:332:LEU:O	2.07	0.54
2:B:508:ARG:O	2:B:509:ALA:O	2.25	0.54

Continued on next page...

Continued from previous page...

Atom-1	Atom-2	Interatomic distance (Å)	Clash overlap (Å)
2:B:577:ASN:O	2:B:578:PRO:C	2.39	0.54
3:M:290:PHE:CZ	3:M:297:PHE:CE1	2.96	0.54
1:A:125:THR:CG2	1:A:158:LEU:HD13	2.36	0.54
1:A:174:ARG:HH22	4:S:148:ARG:NH2	2.04	0.54
2:B:136:CYS:O	2:B:172:GLU:HB3	2.07	0.54
2:B:226:LEU:O	2:B:226:LEU:HD12	2.07	0.54
2:B:596:LEU:HD13	2:B:611:ALA:O	2.06	0.54
3:M:56:VAL:N	3:M:64:LYS:HB2	2.22	0.54
3:M:403:THR:HG22	3:M:407:THR:OG1	2.06	0.54
1:A:375:VAL:HA	1:A:378:ILE:HG12	1.88	0.54
1:A:404:GLN:O	2:B:4:SER:N	2.40	0.54
2:B:162:VAL:HG22	2:B:199:LEU:CD2	2.37	0.54
2:B:493:LEU:HD21	2:B:511:ILE:HA	1.88	0.54
3:M:99:ILE:HG22	3:M:124:ILE:HD13	1.88	0.54
3:M:338:PHE:HD2	3:M:415:ILE:HG13	1.70	0.54
1:A:418:ILE:O	1:A:418:ILE:HG12	2.07	0.54
1:A:485:PRO:O	1:A:488:ARG:HG3	2.08	0.54
2:B:292:GLU:O	2:B:296:ASP:HB3	2.07	0.54
2:B:337:THR:CB	2:B:373:LEU:CD1	2.83	0.54
2:B:490:ILE:CD1	2:B:514:LEU:HG	2.38	0.54
2:B:519:ALA:HB1	2:B:555:LEU:HD13	1.88	0.54
2:B:530:LEU:HD23	2:B:591:MET:HB3	1.87	0.54
3:M:48:ASP:C	3:M:75:TRP:HH2	2.08	0.54
3:M:222:PHE:CE1	3:M:240:ILE:HG23	2.38	0.54
3:M:272:LEU:N	3:M:272:LEU:HD12	2.23	0.54
1:A:166:LEU:O	1:A:170:LEU:HD23	2.08	0.54
1:A:301:GLY:O	1:A:302:ASN:CB	2.54	0.54
2:B:18:ILE:O	2:B:23:ALA:CB	2.55	0.54
2:B:29:LYS:CD	2:B:30:LEU:N	2.59	0.54
2:B:267:ASP:O	2:B:276:SER:OG	2.25	0.54
2:B:436:LEU:HB3	2:B:450:VAL:CG1	2.38	0.54
3:M:44:ASP:HB3	3:M:50:TYR:CD2	2.43	0.54
3:M:115:VAL:O	3:M:116:ASN:C	2.44	0.54
3:M:376:ILE:HD12	3:M:415:ILE:HG12	1.88	0.54
4:S:53:THR:HB	4:S:69:ASN:N	2.22	0.54
1:A:220:SER:O	1:A:223:CYS:CB	2.52	0.54
1:A:279:LEU:HD13	1:A:314:ALA:CB	2.38	0.54
1:A:281:LEU:O	1:A:282:MET:O	2.26	0.54
1:A:461:CYS:O	1:A:463:ASP:N	2.39	0.54
1:A:627:GLU:O	1:A:630:PRO:HD2	2.08	0.54
2:B:278:PRO:CD	2:B:292:GLU:CA	2.86	0.54

Continued on next page...

Continued from previous page...

Atom-1	Atom-2	Interatomic distance (Å)	Clash overlap (Å)
3:M:16:PHE:HB2	3:M:118:TYR:CD1	2.42	0.54
3:M:74:TYR:CB	3:M:114:ILE:CD1	2.85	0.54
3:M:136:VAL:O	3:M:138:ASP:N	2.41	0.54
3:M:362:PHE:O	3:M:363:ASN:O	2.26	0.54
3:M:374:TYR:HB3	3:M:417:TYR:CD2	2.42	0.54
1:A:147:LEU:HD13	1:A:181:ALA:HA	1.89	0.54
1:A:309:PHE:CZ	1:A:348:PHE:CZ	2.95	0.54
1:A:453:VAL:O	1:A:457:LEU:HG	2.06	0.54
1:A:502:ASP:O	3:M:59:ASP:OD2	2.26	0.54
2:B:223:LEU:HD13	2:B:259:TYR:H	1.62	0.54
2:B:418:TYR:O	2:B:418:TYR:HD1	1.89	0.54
2:B:476:ARG:CA	2:B:514:LEU:HD13	2.31	0.54
2:B:534:ILE:CG2	2:B:594:ALA:HB3	2.15	0.54
2:B:537:PHE:CZ	2:B:545:ARG:HD3	2.42	0.54
3:M:44:ASP:C	3:M:46:SER:N	2.60	0.54
3:M:476:THR:HG1	3:M:477:GLY:N	2.05	0.54
1:A:554:ALA:O	1:A:557:LYS:HB2	2.08	0.54
2:B:241:ASP:CB	3:M:274:ASP:CB	2.85	0.54
2:B:435:SER:O	2:B:438:ARG:N	2.39	0.54
2:B:557:SER:O	2:B:560:ILE:N	2.40	0.54
2:B:589:SER:OG	2:B:618:PHE:CZ	2.52	0.54
3:M:15:ILE:CG2	3:M:114:ILE:HG22	2.37	0.54
3:M:327:PHE:HE1	3:M:336:ASP:OD2	1.89	0.54
1:A:134:TYR:O	1:A:135:ASP:C	2.42	0.54
1:A:158:LEU:CD1	1:A:162:ILE:HG13	2.37	0.54
1:A:488:ARG:HD2	1:A:522:PHE:CE2	2.43	0.54
1:A:589:SER:HB2	1:A:601:VAL:CG2	2.38	0.54
2:B:21:GLU:CD	2:B:39:SER:CB	2.66	0.54
2:B:105:LEU:HB3	2:B:145:MET:HE1	1.82	0.54
2:B:177:ILE:HD11	2:B:195:ILE:HG21	1.88	0.54
2:B:310:ILE:HG21	2:B:342:ALA:HB1	1.90	0.54
2:B:374:PHE:CD2	2:B:402:LEU:HD22	2.34	0.54
3:M:48:ASP:C	3:M:75:TRP:CZ2	2.81	0.54
3:M:296:LYS:HG3	3:M:296:LYS:O	2.06	0.54
4:S:49:SER:C	4:S:77:TYR:HB2	2.27	0.54
1:A:114:PHE:CE2	1:A:152:THR:C	2.82	0.54
1:A:233:PHE:O	1:A:234:ILE:O	2.22	0.54
3:M:7:ILE:CD1	3:M:121:ILE:HG21	2.38	0.54
3:M:220:GLU:CD	3:M:222:PHE:CE1	2.81	0.54
3:M:233:LEU:CD2	3:M:323:MET:O	2.55	0.54
3:M:336:ASP:O	3:M:414:CYS:SG	2.54	0.54

Continued on next page...

Continued from previous page...

Atom-1	Atom-2	Interatomic distance (Å)	Clash overlap (Å)
3:M:360:LEU:CD2	3:M:362:PHE:CZ	2.91	0.54
4:S:50:PHE:HA	4:S:77:TYR:HD1	1.73	0.54
4:S:83:LEU:HD11	4:S:116:VAL:CG1	2.38	0.54
1:A:153:ILE:CG2	1:A:158:LEU:HD23	2.37	0.53
1:A:245:VAL:O	1:A:245:VAL:HG22	2.08	0.53
1:A:552:ILE:HG22	1:A:603:VAL:HG21	1.90	0.53
1:A:638:LEU:HD12	2:B:520:SER:CB	2.26	0.53
2:B:79:VAL:HB	2:B:108:PHE:HE1	1.69	0.53
2:B:490:ILE:HG13	2:B:518:ILE:CG1	2.36	0.53
2:B:575:ASN:C	2:B:576:GLN:O	2.31	0.53
3:M:131:ALA:HB3	3:M:135:ASN:HB2	1.90	0.53
3:M:219:LEU:HD13	3:M:473:LYS:HA	1.90	0.53
3:M:302:TYR:CE2	3:M:304:VAL:HB	2.40	0.53
4:S:1:MET:SD	4:S:20:TYR:CE2	3.02	0.53
1:A:395:PHE:CZ	1:A:428:MET:HG3	2.44	0.53
2:B:155:LEU:CB	2:B:188:TYR:HD2	2.20	0.53
2:B:367:SER:OG	2:B:401:THR:OG1	2.25	0.53
2:B:515:PHE:HE2	2:B:529:VAL:HG21	1.72	0.53
2:B:556:LEU:HD12	2:B:614:ILE:CG2	2.38	0.53
3:M:269:ILE:C	3:M:302:TYR:CE1	2.82	0.53
3:M:317:MET:HB2	3:M:322:LEU:N	2.24	0.53
4:S:5:VAL:CG1	4:S:87:PHE:CE2	2.91	0.53
1:A:121:LEU:HD12	1:A:153:ILE:HG22	1.89	0.53
1:A:223:CYS:HB2	1:A:259:LEU:CD1	2.38	0.53
1:A:375:VAL:HG11	1:A:387:ILE:HG21	1.90	0.53
2:B:50:LEU:HB3	2:B:62:ALA:HB2	1.89	0.53
2:B:80:GLN:CG	2:B:115:LEU:HD21	2.18	0.53
2:B:105:LEU:CG	2:B:119:SER:HB2	2.34	0.53
2:B:174:ALA:CB	2:B:211:ALA:CA	2.75	0.53
2:B:234:CYS:SG	2:B:298:ASP:O	2.67	0.53
2:B:310:ILE:HG23	2:B:318:ILE:HG12	1.91	0.53
2:B:310:ILE:HD11	2:B:321:CYS:HB2	1.89	0.53
2:B:549:LEU:CG	2:B:611:ALA:HB2	2.38	0.53
3:M:338:PHE:CE2	3:M:415:ILE:HD11	2.43	0.53
3:M:371:GLU:O	3:M:419:ASN:OD1	2.26	0.53
4:S:83:LEU:HD12	4:S:116:VAL:HG11	1.90	0.53
2:B:36:THR:HA	2:B:39:SER:OG	2.09	0.53
2:B:237:ILE:HD12	2:B:248:LEU:CB	2.39	0.53
2:B:378:THR:CG2	2:B:379:LYS:N	2.71	0.53
2:B:519:ALA:O	2:B:523:PHE:N	2.41	0.53
3:M:96:ILE:HD11	3:M:125:PHE:HA	1.91	0.53

Continued on next page...

Continued from previous page...

Atom-1	Atom-2	Interatomic distance (Å)	Clash overlap (Å)
3:M:121:ILE:O	3:M:125:PHE:HD1	1.90	0.53
3:M:350:VAL:HA	3:M:442:GLN:CB	2.38	0.53
3:M:437:TYR:HB3	3:M:439:TYR:CE2	2.43	0.53
4:S:32:LEU:O	4:S:35:VAL:CG2	2.55	0.53
4:S:130:SER:CB	4:S:156:LEU:CD1	2.86	0.53
1:A:95:MET:HB2	1:A:127:LEU:CD2	2.38	0.53
1:A:244:LEU:O	1:A:245:VAL:C	2.46	0.53
2:B:2:VAL:HG13	2:B:6:HIS:NE2	2.24	0.53
2:B:8:ILE:HG22	2:B:12:LEU:HD11	1.90	0.53
2:B:237:ILE:CG2	2:B:238:LYS:N	2.71	0.53
2:B:476:ARG:O	2:B:480:GLN:HG3	2.08	0.53
2:B:560:ILE:O	2:B:563:PHE:N	2.41	0.53
2:B:566:ALA:HB2	2:B:581:TYR:HD2	1.71	0.53
3:M:221:THR:CB	3:M:474:THR:OG1	2.57	0.53
3:M:276:VAL:HG22	3:M:290:PHE:CD1	2.40	0.53
3:M:375:LYS:CE	3:M:418:GLU:OE1	2.57	0.53
3:M:442:GLN:HG3	3:M:443:SER:N	2.23	0.53
4:S:47:GLN:HG2	4:S:84:TYR:HE2	1.74	0.53
2:B:29:LYS:HD3	2:B:30:LEU:H	1.72	0.53
2:B:81:LEU:C	2:B:83:PHE:N	2.55	0.53
2:B:117:LEU:HD23	2:B:150:LEU:CD2	2.39	0.53
2:B:181:TYR:HD2	2:B:218:CYS:HA	1.73	0.53
2:B:189:HIS:CE1	2:B:222:HIS:ND1	2.76	0.53
2:B:335:LYS:O	2:B:373:LEU:HD22	2.09	0.53
3:M:442:GLN:CG	3:M:443:SER:H	2.21	0.53
1:A:446:ASP:O	1:A:448:GLU:O	2.27	0.53
2:B:108:PHE:CD2	2:B:115:LEU:HB2	2.44	0.53
2:B:151:ALA:CA	2:B:180:LEU:CD1	2.67	0.53
2:B:151:ALA:HB1	2:B:188:TYR:CD1	2.44	0.53
2:B:455:ILE:O	2:B:459:GLU:N	2.38	0.53
3:M:250:LEU:HD13	3:M:254:PRO:HG2	1.89	0.53
4:S:130:SER:HB2	4:S:156:LEU:HD13	1.89	0.53
1:A:151:SER:O	1:A:152:THR:C	2.41	0.53
1:A:207:LEU:HD23	1:A:239:LEU:HB3	1.91	0.53
2:B:17:VAL:O	2:B:18:ILE:O	2.26	0.53
2:B:50:LEU:CG	2:B:62:ALA:HB2	2.39	0.53
2:B:126:SER:O	2:B:135:ARG:HG2	2.09	0.53
2:B:217:GLU:O	2:B:218:CYS:C	2.47	0.53
3:M:262:THR:O	3:M:264:GLY:N	2.36	0.53
1:A:83:ASP:CG	1:A:85:ALA:H	2.12	0.53
1:A:153:ILE:HG22	1:A:158:LEU:HD23	1.91	0.53

Continued on next page...

Continued from previous page...

Atom-1	Atom-2	Interatomic distance (Å)	Clash overlap (Å)
1:A:174:ARG:CZ	4:S:148:ARG:NH2	2.69	0.53
1:A:322:PHE:HD2	1:A:330:LEU:HD21	1.69	0.53
1:A:529:GLY:HA3	1:A:562:TRP:CZ2	2.44	0.53
2:B:13:ASP:O	2:B:14:THR:C	2.47	0.53
2:B:325:LEU:HD13	2:B:339:PHE:CG	2.43	0.53
2:B:577:ASN:C	2:B:578:PRO:O	2.48	0.53
3:M:223:HIS:HB3	3:M:477:GLY:O	2.09	0.53
3:M:306:LEU:O	3:M:307:SER:O	2.27	0.53
3:M:306:LEU:HD22	3:M:317:MET:CE	2.33	0.53
1:A:187:LYS:O	1:A:190:LEU:HB3	2.08	0.53
1:A:289:SER:O	1:A:291:ILE:N	2.39	0.53
1:A:402:ILE:CB	1:A:421:PRO:HA	2.39	0.53
1:A:509:PRO:HB3	1:A:547:VAL:HG21	1.90	0.53
2:B:390:VAL:HA	2:B:393:ILE:HD12	1.91	0.53
2:B:397:GLN:O	2:B:400:SER:OG	2.17	0.53
2:B:592:TYR:CE2	2:B:618:PHE:CE2	2.97	0.53
3:M:290:PHE:CE1	3:M:297:PHE:CD1	2.97	0.53
3:M:443:SER:OG	3:M:447:ILE:O	2.27	0.53
2:B:147:MET:O	2:B:148:SER:C	2.41	0.52
2:B:246:SER:O	2:B:249:ILE:HB	2.08	0.52
3:M:306:LEU:HD21	3:M:317:MET:CE	2.39	0.52
4:S:1:MET:H2	4:S:93:GLU:CD	2.11	0.52
1:A:196:LEU:O	1:A:197:ARG:O	2.27	0.52
1:A:435:ILE:CG2	1:A:441:TYR:CE2	2.93	0.52
1:A:533:ILE:HG12	1:A:562:TRP:CZ2	2.43	0.52
2:B:25:VAL:CG2	2:B:35:TYR:HB3	2.39	0.52
2:B:132:SER:HB2	2:B:166:SER:OG	2.09	0.52
2:B:151:ALA:C	2:B:188:TYR:CE2	2.66	0.52
2:B:245:GLN:NE2	2:B:309:LEU:CD1	2.69	0.52
2:B:483:PRO:O	2:B:486:HIS:HB3	2.09	0.52
2:B:490:ILE:O	2:B:515:PHE:CZ	2.62	0.52
2:B:537:PHE:CZ	2:B:545:ARG:CD	2.92	0.52
2:B:556:LEU:HB3	2:B:588:ILE:HD11	1.89	0.52
2:B:563:PHE:CZ	2:B:588:ILE:HB	2.44	0.52
2:B:564:LYS:CD	2:B:621:GLY:O	2.50	0.52
3:M:243:ILE:HD13	3:M:301:GLU:CB	2.39	0.52
1:A:207:LEU:CD1	1:A:243:ILE:HG13	2.39	0.52
1:A:237:SER:HB2	1:A:270:LEU:HD13	1.91	0.52
1:A:506:LYS:O	1:A:507:GLN:CB	2.56	0.52
2:B:154:ILE:HB	2:B:180:LEU:HD13	1.91	0.52
2:B:271:GLU:C	2:B:273:SER:N	2.54	0.52

Continued on next page...

Continued from previous page...

Atom-1	Atom-2	Interatomic distance (Å)	Clash overlap (Å)
2:B:458:MET:SD	2:B:475:ILE:HD11	2.49	0.52
2:B:549:LEU:CG	2:B:614:ILE:HD12	2.38	0.52
2:B:564:LYS:O	2:B:567:GLN:N	2.42	0.52
3:M:84:LYS:O	3:M:88:ASP:HB3	2.08	0.52
3:M:224:VAL:CG1	3:M:226:PHE:CE1	2.92	0.52
3:M:235:LEU:HD11	3:M:306:LEU:CD1	2.40	0.52
3:M:253:ASN:N	3:M:254:PRO:CD	2.72	0.52
3:M:347:PHE:HE1	3:M:350:VAL:HG11	1.65	0.52
3:M:374:TYR:HE1	3:M:376:ILE:CG1	2.23	0.52
4:S:57:LEU:O	4:S:59:LEU:HA	2.09	0.52
1:A:183:THR:OG1	4:S:137:GLN:O	2.28	0.52
2:B:29:LYS:O	2:B:32:GLU:CG	2.56	0.52
2:B:83:PHE:O	2:B:87:VAL:HG23	2.10	0.52
2:B:214:ALA:C	2:B:216:LYS:N	2.61	0.52
3:M:276:VAL:HG21	3:M:299:LEU:HD12	1.90	0.52
3:M:283:PHE:CE2	3:M:289:THR:HB	2.44	0.52
3:M:327:PHE:CE1	3:M:336:ASP:CB	2.89	0.52
3:M:343:ASN:HD22	3:M:343:ASN:H	1.56	0.52
4:S:3:HIS:O	4:S:19:PHE:HA	2.10	0.52
1:A:341:ILE:O	1:A:344:ILE:HB	2.10	0.52
1:A:609:LEU:CG	1:A:628:VAL:CG1	2.66	0.52
2:B:25:VAL:CG2	2:B:36:THR:H	1.91	0.52
2:B:90:ILE:CD1	2:B:123:LEU:HD23	2.40	0.52
2:B:197:LYS:N	2:B:229:HIS:NE2	2.57	0.52
2:B:216:LYS:HG3	2:B:251:LEU:HD12	1.90	0.52
2:B:340:ILE:HD13	2:B:366:LEU:HD13	1.92	0.52
2:B:470:ALA:O	2:B:473:ASN:N	2.42	0.52
3:M:243:ILE:N	3:M:474:THR:HG22	2.21	0.52
3:M:290:PHE:CZ	3:M:297:PHE:CZ	2.98	0.52
3:M:405:THR:C	3:M:407:THR:H	2.12	0.52
1:A:595:GLU:HG2	2:B:473:ASN:CG	2.30	0.52
2:B:132:SER:HA	2:B:169:VAL:HG22	1.90	0.52
2:B:146:LYS:O	2:B:147:MET:SD	2.67	0.52
2:B:208:ILE:CD1	2:B:236:ILE:HG23	2.28	0.52
2:B:237:ILE:HD12	2:B:248:LEU:HB2	1.92	0.52
2:B:241:ASP:HB3	3:M:274:ASP:CA	2.32	0.52
2:B:351:GLU:C	3:M:49:ASP:OD2	2.47	0.52
2:B:452:LYS:NZ	2:B:456:ASP:OD1	2.42	0.52
2:B:560:ILE:HG22	2:B:561:ASP:N	2.25	0.52
4:S:146:VAL:O	4:S:150:VAL:HG23	2.10	0.52
2:B:102:HIS:ND1	2:B:137:PHE:O	2.41	0.52

Continued on next page...

Continued from previous page...

Atom-1	Atom-2	Interatomic distance (Å)	Clash overlap (Å)
2:B:180:LEU:HD23	2:B:192:LEU:HD21	1.90	0.52
2:B:215:TYR:HB3	2:B:226:LEU:HD13	1.91	0.52
2:B:270:SER:O	2:B:273:SER:HB2	2.09	0.52
2:B:284:ASN:O	2:B:284:ASN:CG	2.45	0.52
2:B:311:TYR:CD1	3:M:269:ILE:HD11	2.44	0.52
2:B:545:ARG:NH1	2:B:602:ASP:HA	2.24	0.52
2:B:596:LEU:HD22	2:B:615:SER:OG	2.09	0.52
3:M:217:ASP:O	3:M:472:TYR:CE1	2.63	0.52
3:M:240:ILE:CG2	3:M:444:ALA:O	2.52	0.52
3:M:374:TYR:HA	3:M:417:TYR:HA	1.92	0.52
4:S:55:PRO:HB3	4:S:71:GLU:HG3	1.91	0.52
1:A:128:LEU:HD12	1:A:150:LEU:CD2	2.40	0.52
2:B:208:ILE:CG2	2:B:236:ILE:HG21	2.35	0.52
2:B:267:ASP:CB	2:B:289:PRO:HG3	2.40	0.52
2:B:340:ILE:CD1	2:B:366:LEU:HB3	2.40	0.52
3:M:228:LYS:NZ	3:M:326:HIS:HA	2.25	0.52
4:S:10:LYS:CA	4:S:84:TYR:HE1	2.22	0.52
4:S:149:ILE:O	4:S:153:VAL:HG23	2.10	0.52
1:A:250:ASN:OD1	1:A:285:THR:CB	2.54	0.52
2:B:73:ASP:O	2:B:75:ASP:N	2.43	0.52
2:B:120:ILE:CG1	2:B:150:LEU:HD13	2.39	0.52
2:B:139:LEU:HD23	2:B:173:VAL:N	2.23	0.52
2:B:155:LEU:HB2	2:B:188:TYR:HD2	1.70	0.52
2:B:433:VAL:HG22	2:B:471:TYR:CZ	2.45	0.52
3:M:217:ASP:O	3:M:472:TYR:CZ	2.63	0.52
3:M:300:LEU:C	3:M:300:LEU:HD12	2.31	0.52
3:M:342:LEU:O	3:M:409:PRO:HD2	2.10	0.52
3:M:350:VAL:HG22	3:M:442:GLN:CG	2.28	0.52
4:S:57:LEU:HD23	4:S:66:ASP:HA	1.92	0.52
1:A:244:LEU:HG	1:A:281:LEU:CD1	2.29	0.52
1:A:301:GLY:O	1:A:302:ASN:HB3	2.10	0.52
1:A:599:ARG:CZ	2:B:547:GLN:HE22	2.22	0.52
2:B:311:TYR:CB	3:M:269:ILE:CD1	2.87	0.52
2:B:374:PHE:CB	2:B:402:LEU:CD2	2.81	0.52
2:B:490:ILE:HD12	2:B:518:ILE:HG21	1.89	0.52
3:M:58:ARG:O	3:M:60:LEU:O	2.27	0.52
3:M:218:LEU:HG	3:M:244:VAL:HG22	1.92	0.52
1:A:395:PHE:CZ	1:A:428:MET:HB2	2.45	0.51
2:B:87:VAL:O	2:B:88:LYS:C	2.44	0.51
3:M:302:TYR:CE2	3:M:445:SER:HB3	2.44	0.51
3:M:344:ILE:H	3:M:408:VAL:HG22	1.75	0.51

Continued on next page...

Continued from previous page...

Atom-1	Atom-2	Interatomic distance (Å)	Clash overlap (Å)
3:M:350:VAL:CG1	3:M:442:GLN:CB	2.80	0.51
3:M:374:TYR:CB	3:M:417:TYR:HD2	2.23	0.51
1:A:121:LEU:HG	1:A:153:ILE:HG21	1.91	0.51
1:A:237:SER:HB2	1:A:270:LEU:HD11	1.92	0.51
1:A:289:SER:CA	4:S:96:LEU:HD11	2.39	0.51
2:B:189:HIS:CD2	2:B:222:HIS:CG	2.98	0.51
2:B:253:ILE:CG1	2:B:324:ALA:HB2	2.40	0.51
3:M:64:LYS:HE3	3:M:66:PHE:CZ	2.44	0.51
3:M:114:ILE:HG23	3:M:121:ILE:CD1	2.41	0.51
3:M:443:SER:OG	3:M:448:TYR:N	2.43	0.51
1:A:404:GLN:C	2:B:7:ARG:CZ	2.76	0.51
1:A:637:GLU:HG3	2:B:516:GLY:C	2.30	0.51
2:B:18:ILE:O	2:B:23:ALA:HB2	2.10	0.51
2:B:37:TYR:O	2:B:40:GLN:N	2.43	0.51
2:B:188:TYR:HB3	2:B:192:LEU:CD1	2.41	0.51
2:B:223:LEU:CD1	2:B:258:GLN:CA	2.87	0.51
2:B:574:ASN:O	2:B:576:GLN:O	2.28	0.51
3:M:52:ASP:O	3:M:53:HIS:HB3	2.11	0.51
3:M:220:GLU:C	3:M:474:THR:OG1	2.43	0.51
1:A:288:THR:O	1:A:291:ILE:HB	2.10	0.51
1:A:373:GLU:HG2	1:A:427:LYS:CE	2.40	0.51
1:A:397:ASP:O	1:A:418:ILE:HG13	2.11	0.51
1:A:416:ILE:O	1:A:417:PRO:C	2.40	0.51
1:A:566:PHE:C	1:A:568:GLU:N	2.55	0.51
2:B:203:THR:HG22	2:B:232:ARG:HH22	1.74	0.51
2:B:204:ASP:O	2:B:207:VAL:HB	2.10	0.51
2:B:589:SER:HA	2:B:592:TYR:CD2	2.43	0.51
3:M:241:HIS:CB	3:M:476:THR:CG2	2.86	0.51
3:M:242:GLY:HA3	3:M:444:ALA:HB3	1.91	0.51
4:S:137:GLN:C	4:S:140:MET:H	2.13	0.51
1:A:200:PHE:CE1	1:A:236:LEU:CD1	2.93	0.51
1:A:257:LEU:CD2	1:A:278:ILE:CG2	2.55	0.51
1:A:291:ILE:O	1:A:295:VAL:HG23	2.11	0.51
2:B:98:LYS:HB2	2:B:134:LEU:HD22	1.92	0.51
2:B:162:VAL:HG23	2:B:173:VAL:CG1	2.37	0.51
3:M:342:LEU:HD12	3:M:342:LEU:N	2.25	0.51
4:S:24:ASP:HB3	4:S:26:PRO:HD2	1.91	0.51
1:A:176:TYR:CE1	4:S:148:ARG:NH2	2.69	0.51
2:B:195:ILE:C	2:B:197:LYS:N	2.62	0.51
2:B:219:TYR:O	2:B:223:LEU:HG	2.10	0.51
2:B:322:CYS:SG	2:B:362:ALA:HB1	2.51	0.51

Continued on next page...

Continued from previous page...

Atom-1	Atom-2	Interatomic distance (Å)	Clash overlap (Å)
2:B:353:GLN:C	2:B:355:ASN:H	2.08	0.51
2:B:433:VAL:CG1	2:B:474:VAL:CG2	2.69	0.51
2:B:486:HIS:NE2	2:B:518:ILE:CG1	2.73	0.51
2:B:519:ALA:HA	2:B:526:CYS:SG	2.51	0.51
2:B:563:PHE:O	2:B:566:ALA:CA	2.58	0.51
3:M:44:ASP:HB2	3:M:50:TYR:CD2	2.45	0.51
3:M:54:SER:HB2	3:M:66:PHE:CE1	2.46	0.51
3:M:103:TYR:O	3:M:104:PHE:HB2	2.11	0.51
1:A:79:MET:HB3	4:S:25:LEU:HD11	1.92	0.51
1:A:167:PHE:CE1	1:A:202:LYS:HD3	2.46	0.51
2:B:55:ASN:O	2:B:59:VAL:HG23	2.11	0.51
2:B:215:TYR:CZ	2:B:229:HIS:HB3	2.46	0.51
2:B:223:LEU:HD22	2:B:255:TYR:CD1	2.46	0.51
2:B:337:THR:CA	2:B:373:LEU:CD1	2.78	0.51
2:B:340:ILE:HB	2:B:373:LEU:HG	1.93	0.51
2:B:431:MET:C	2:B:433:VAL:N	2.62	0.51
2:B:567:GLN:N	2:B:574:ASN:HD22	2.06	0.51
3:M:235:LEU:CD1	3:M:306:LEU:HD13	2.40	0.51
3:M:270:PRO:CA	3:M:302:TYR:CD1	2.94	0.51
3:M:270:PRO:CA	3:M:302:TYR:HD1	2.24	0.51
3:M:270:PRO:HA	3:M:302:TYR:CD1	2.46	0.51
1:A:103:LYS:HB3	1:A:107:TYR:HE1	1.76	0.51
2:B:44:PRO:HG3	2:B:77:ILE:CD1	2.41	0.51
2:B:77:ILE:HG23	2:B:82:TYR:CE1	2.45	0.51
2:B:162:VAL:O	2:B:164:ASP:N	2.43	0.51
2:B:177:ILE:HD11	2:B:195:ILE:CG2	2.41	0.51
2:B:303:LEU:HD11	2:B:333:GLN:CD	2.30	0.51
2:B:367:SER:OG	2:B:401:THR:HG21	2.10	0.51
2:B:411:ILE:O	2:B:414:GLU:N	2.40	0.51
2:B:450:VAL:O	2:B:453:TRP:HB2	2.11	0.51
3:M:51:LEU:N	3:M:51:LEU:HD12	2.26	0.51
3:M:213:GLU:CB	3:M:467:TYR:HB2	2.41	0.51
3:M:233:LEU:CD2	3:M:324:SER:HA	2.39	0.51
3:M:272:LEU:HD22	3:M:278:ILE:CB	2.40	0.51
3:M:372:ILE:HD12	3:M:428:VAL:CG2	2.41	0.51
1:A:68:THR:OG1	4:S:166:LYS:HB3	2.11	0.51
1:A:88:ASN:ND2	1:A:120:ILE:HG13	2.25	0.51
1:A:121:LEU:HD13	1:A:155:THR:OG1	2.11	0.51
2:B:155:LEU:HD13	2:B:155:LEU:C	2.30	0.51
2:B:181:TYR:CZ	2:B:222:HIS:CD2	2.96	0.51
2:B:303:LEU:HD11	2:B:333:GLN:CB	2.41	0.51

Continued on next page...

Continued from previous page...

Atom-1	Atom-2	Interatomic distance (Å)	Clash overlap (Å)
2:B:311:TYR:HB3	3:M:269:ILE:HD12	1.92	0.51
2:B:556:LEU:CA	2:B:588:ILE:CD1	2.53	0.51
3:M:56:VAL:H	3:M:64:LYS:HB2	1.76	0.51
3:M:99:ILE:O	3:M:103:TYR:CD1	2.64	0.51
3:M:429:ASP:O	3:M:430:LEU:C	2.45	0.51
3:M:443:SER:OG	3:M:448:TYR:CA	2.58	0.51
4:S:73:ILE:CG2	4:S:88:ILE:CG2	2.88	0.51
1:A:128:LEU:HD12	1:A:150:LEU:HD23	1.91	0.51
1:A:260:PHE:CD1	1:A:274:LEU:CD1	2.94	0.51
1:A:638:LEU:HD11	2:B:519:ALA:C	2.31	0.51
2:B:30:LEU:C	2:B:32:GLU:H	2.14	0.51
2:B:112:ASP:C	2:B:112:ASP:OD1	2.49	0.51
2:B:193:LEU:CB	2:B:225:LEU:CD1	2.84	0.51
2:B:196:LEU:HB3	2:B:215:TYR:OH	2.10	0.51
2:B:308:CYS:O	2:B:311:TYR:N	2.44	0.51
2:B:508:ARG:HB3	2:B:544:THR:CG2	2.40	0.51
3:M:224:VAL:HB	3:M:226:PHE:CE1	2.46	0.51
4:S:38:LEU:HD23	4:S:68:VAL:CG2	2.39	0.51
4:S:49:SER:O	4:S:77:TYR:O	2.29	0.51
1:A:223:CYS:CB	1:A:259:LEU:HG	2.41	0.50
1:A:233:PHE:O	1:A:235:GLN:N	2.42	0.50
1:A:253:ILE:O	1:A:257:LEU:HG	2.11	0.50
1:A:436:CYS:CB	1:A:450:TYR:CZ	2.94	0.50
1:A:566:PHE:C	1:A:566:PHE:CD1	2.83	0.50
2:B:120:ILE:HD12	2:B:142:LEU:CD2	2.41	0.50
2:B:513:TRP:HE1	2:B:517:GLU:HG3	1.76	0.50
2:B:534:ILE:CG1	2:B:595:VAL:CG2	2.89	0.50
2:B:592:TYR:CZ	2:B:619:ASP:OD1	2.64	0.50
3:M:260:LEU:HD21	3:M:449:VAL:HG22	1.91	0.50
1:A:107:TYR:CE2	1:A:128:LEU:CD2	2.94	0.50
1:A:150:LEU:HB3	1:A:162:ILE:HD13	1.93	0.50
1:A:402:ILE:CG2	1:A:421:PRO:HA	2.41	0.50
2:B:181:TYR:CE1	2:B:222:HIS:NE2	2.70	0.50
2:B:249:ILE:HD13	2:B:321:CYS:SG	2.52	0.50
2:B:286:ILE:CG2	2:B:288:TYR:CE2	2.94	0.50
2:B:353:GLN:HB3	3:M:50:TYR:CG	2.38	0.50
2:B:449:HIS:O	2:B:453:TRP:CD1	2.64	0.50
2:B:486:HIS:HA	2:B:489:ILE:HD12	1.92	0.50
3:M:4:SER:HB3	3:M:79:SER:OG	2.11	0.50
3:M:226:PHE:N	3:M:480:GLN:O	2.41	0.50
3:M:374:TYR:HB2	3:M:417:TYR:CD2	2.46	0.50

Continued on next page...

Continued from previous page...

Atom-1	Atom-2	Interatomic distance (Å)	Clash overlap (Å)
4:S:47:GLN:O	4:S:49:SER:N	2.44	0.50
1:A:512:LEU:HD13	1:A:543:TYR:CZ	2.46	0.50
2:B:20:ARG:NH1	2:B:21:GLU:HG3	2.19	0.50
2:B:51:LEU:HD11	2:B:66:ILE:HD13	1.93	0.50
2:B:162:VAL:HG22	2:B:199:LEU:HD21	1.93	0.50
2:B:237:ILE:O	2:B:239:GLN:N	2.44	0.50
2:B:396:ILE:CD1	2:B:418:TYR:HE2	2.23	0.50
3:M:6:TYR:CD1	3:M:77:LEU:HD23	2.47	0.50
3:M:235:LEU:HD22	3:M:307:SER:HA	1.93	0.50
3:M:241:HIS:O	3:M:474:THR:CB	2.59	0.50
4:S:83:LEU:CD1	4:S:116:VAL:HG11	2.41	0.50
1:A:109:ALA:O	1:A:112:GLN:N	2.45	0.50
1:A:298:ILE:HD11	1:A:311:THR:CG2	2.34	0.50
2:B:77:ILE:HG22	2:B:82:TYR:HE1	1.74	0.50
2:B:157:THR:O	2:B:160:LYS:N	2.44	0.50
2:B:172:GLU:OE1	2:B:175:LEU:HD12	2.12	0.50
2:B:193:LEU:CB	2:B:225:LEU:CG	2.83	0.50
2:B:219:TYR:CE1	2:B:226:LEU:HD12	2.32	0.50
2:B:343:LEU:CD2	2:B:363:ILE:HD13	2.33	0.50
2:B:523:PHE:HB2	2:B:559:ASP:CG	2.30	0.50
4:S:38:LEU:CD2	4:S:68:VAL:HG21	2.39	0.50
1:A:271:ARG:NH1	1:A:302:ASN:O	2.44	0.50
2:B:38:TYR:CD1	2:B:38:TYR:N	2.76	0.50
2:B:79:VAL:CB	2:B:108:PHE:HE1	2.20	0.50
2:B:120:ILE:CD1	2:B:142:LEU:HD23	2.42	0.50
2:B:553:ALA:CA	2:B:614:ILE:HG21	2.41	0.50
3:M:19:LEU:CD2	3:M:24:ALA:HB3	2.42	0.50
3:M:260:LEU:HD23	3:M:449:VAL:HG22	1.92	0.50
1:A:349:ILE:O	1:A:350:SER:C	2.49	0.50
2:B:114:ASN:O	2:B:117:LEU:HB2	2.12	0.50
2:B:139:LEU:HD21	2:B:173:VAL:O	2.12	0.50
2:B:161:LEU:CB	2:B:173:VAL:HG22	2.35	0.50
2:B:247:TYR:HE1	3:M:136:VAL:HG13	1.76	0.50
2:B:276:SER:OG	2:B:289:PRO:HG3	2.12	0.50
2:B:311:TYR:CD1	3:M:269:ILE:CD1	2.94	0.50
2:B:578:PRO:CD	2:B:581:TYR:CD1	2.84	0.50
3:M:4:SER:HA	3:M:18:TYR:O	2.11	0.50
4:S:46:PHE:O	4:S:47:GLN:C	2.29	0.50
4:S:89:VAL:HG11	4:S:98:ILE:HD12	1.93	0.50
1:A:260:PHE:CE2	1:A:274:LEU:HD11	2.44	0.50
1:A:556:VAL:HG22	1:A:603:VAL:HG11	1.93	0.50

Continued on next page...

Continued from previous page...

Atom-1	Atom-2	Interatomic distance (Å)	Clash overlap (Å)
2:B:62:ALA:O	2:B:66:ILE:CD1	2.60	0.50
2:B:346:THR:O	2:B:350:THR:N	2.44	0.50
3:M:356:LEU:CD2	3:M:358:ILE:HG13	2.41	0.50
1:A:71:VAL:HG21	1:A:94:VAL:HG11	1.93	0.50
1:A:147:LEU:HD22	1:A:166:LEU:CD2	2.41	0.50
1:A:158:LEU:CG	1:A:162:ILE:HD11	2.41	0.50
1:A:279:LEU:CD1	1:A:314:ALA:HB1	2.41	0.50
1:A:401:VAL:HG23	1:A:418:ILE:N	2.27	0.50
1:A:581:LEU:CD2	1:A:607:LEU:HD21	2.42	0.50
2:B:171:GLY:HA2	2:B:207:VAL:CG1	2.40	0.50
2:B:196:LEU:HA	2:B:199:LEU:HD12	1.93	0.50
2:B:242:SER:OG	3:M:98:ARG:NH2	2.45	0.50
2:B:251:LEU:O	2:B:254:LYS:N	2.45	0.50
2:B:292:GLU:CG	2:B:296:ASP:HB2	2.41	0.50
2:B:292:GLU:HG2	2:B:296:ASP:CB	2.41	0.50
2:B:352:ASN:HD22	3:M:70:ASN:HD22	1.59	0.50
3:M:235:LEU:HD23	3:M:235:LEU:O	2.10	0.50
3:M:374:TYR:CZ	3:M:390:ILE:HD13	2.47	0.50
4:S:8:PHE:CE2	4:S:86:THR:N	2.74	0.50
1:A:428:MET:O	1:A:431:VAL:HB	2.12	0.50
2:B:245:GLN:HG2	2:B:309:LEU:HD11	1.84	0.50
2:B:469:ASP:OD1	2:B:506:ASN:CB	2.60	0.50
3:M:44:ASP:HB2	3:M:50:TYR:HD2	1.77	0.50
4:S:15:ARG:NH1	4:S:122:ILE:HD11	2.27	0.50
1:A:186:PHE:HE2	1:A:187:LYS:HD3	1.77	0.49
1:A:401:VAL:HG23	1:A:418:ILE:CA	2.42	0.49
2:B:147:MET:HB2	2:B:150:LEU:HG	1.92	0.49
2:B:212:VAL:CG2	2:B:233:TYR:CE2	2.94	0.49
2:B:397:GLN:HE21	2:B:431:MET:HE3	1.76	0.49
2:B:511:ILE:O	2:B:512:VAL:C	2.48	0.49
2:B:563:PHE:CA	2:B:566:ALA:HB3	2.42	0.49
3:M:233:LEU:HD22	3:M:323:MET:O	2.12	0.49
3:M:245:ASP:O	3:M:246:VAL:CG2	2.60	0.49
3:M:262:THR:C	3:M:264:GLY:H	2.14	0.49
4:S:93:GLU:HG2	4:S:98:ILE:HD11	1.94	0.49
4:S:116:VAL:HG22	4:S:117:ASN:O	2.11	0.49
1:A:64:LEU:CB	1:A:102:GLN:NE2	2.74	0.49
1:A:92:LEU:HD21	1:A:124:ALA:HA	1.93	0.49
1:A:395:PHE:CE2	1:A:420:ILE:HD13	2.48	0.49
1:A:450:TYR:OH	1:A:476:GLN:CD	2.50	0.49
2:B:151:ALA:N	2:B:152:PRO:CD	2.75	0.49

Continued on next page...

Continued from previous page...

Atom-1	Atom-2	Interatomic distance (Å)	Clash overlap (Å)
2:B:189:HIS:CE1	2:B:222:HIS:CG	3.01	0.49
2:B:519:ALA:C	2:B:523:PHE:HB3	2.23	0.49
2:B:523:PHE:CZ	2:B:580:TYR:HD1	2.05	0.49
3:M:288:ILE:CD1	3:M:300:LEU:CD2	2.90	0.49
1:A:80:TYR:HB2	1:A:82:PHE:CE2	2.47	0.49
1:A:179:LYS:NZ	4:S:141:VAL:HA	1.86	0.49
1:A:253:ILE:HG23	1:A:281:LEU:HD13	1.93	0.49
1:A:292:TYR:O	1:A:295:VAL:HB	2.13	0.49
1:A:486:SER:O	1:A:487:MET:HB2	2.11	0.49
1:A:571:ARG:NH2	1:A:573:GLU:OE1	2.37	0.49
2:B:253:ILE:CD1	2:B:324:ALA:HB2	2.43	0.49
2:B:389:ILE:O	2:B:393:ILE:HG13	2.13	0.49
2:B:599:ALA:C	2:B:601:TYR:H	2.16	0.49
3:M:290:PHE:CZ	3:M:297:PHE:CD1	3.00	0.49
3:M:443:SER:CB	3:M:447:ILE:N	2.75	0.49
4:S:102:ILE:O	4:S:105:PHE:HB3	2.13	0.49
1:A:289:SER:HA	4:S:96:LEU:HD11	1.94	0.49
1:A:316:LEU:CD1	1:A:341:ILE:HG21	2.40	0.49
1:A:446:ASP:CG	1:A:448:GLU:O	2.50	0.49
1:A:529:GLY:C	1:A:562:TRP:CZ2	2.86	0.49
1:A:581:LEU:HB3	1:A:607:LEU:HD13	1.95	0.49
1:A:602:GLU:CD	1:A:633:PHE:HE1	2.15	0.49
2:B:38:TYR:HD1	2:B:38:TYR:N	2.10	0.49
2:B:133:GLU:O	2:B:168:MET:CE	2.60	0.49
2:B:234:CYS:HB3	2:B:301:LEU:CB	2.39	0.49
2:B:589:SER:OG	2:B:618:PHE:CE2	2.53	0.49
3:M:16:PHE:HE1	3:M:122:SER:CB	2.20	0.49
3:M:74:TYR:OH	3:M:97:ASP:HB3	2.12	0.49
2:B:139:LEU:HD21	2:B:173:VAL:CA	2.25	0.49
2:B:168:MET:HA	2:B:207:VAL:HG22	1.94	0.49
2:B:364:HIS:O	2:B:368:ILE:HG12	2.12	0.49
2:B:537:PHE:CG	2:B:598:LEU:HB3	2.42	0.49
3:M:222:PHE:CE1	3:M:240:ILE:HG21	2.47	0.49
3:M:248:SER:C	3:M:249:TYR:CD1	2.86	0.49
4:S:35:VAL:HB	4:S:77:TYR:CZ	2.46	0.49
1:A:225:LEU:CD1	1:A:233:PHE:CE2	2.80	0.49
1:A:371:ALA:O	1:A:374:LEU:N	2.45	0.49
1:A:529:GLY:CA	1:A:562:TRP:CZ2	2.96	0.49
2:B:20:ARG:HH11	2:B:35:TYR:HE1	1.55	0.49
2:B:29:LYS:HE2	2:B:30:LEU:CB	2.42	0.49
2:B:118:LEU:O	2:B:121:ASN:N	2.44	0.49

Continued on next page...

Continued from previous page...

Atom-1	Atom-2	Interatomic distance (Å)	Clash overlap (Å)
2:B:121:ASN:O	2:B:124:GLN:HB3	2.12	0.49
2:B:178:ILE:CD1	2:B:218:CYS:CB	2.89	0.49
2:B:195:ILE:O	2:B:197:LYS:N	2.46	0.49
2:B:267:ASP:N	2:B:289:PRO:CB	2.63	0.49
2:B:374:PHE:HB3	2:B:402:LEU:HD22	1.87	0.49
2:B:433:VAL:HG21	2:B:471:TYR:CE1	2.47	0.49
2:B:461:HIS:O	2:B:462:ASN:HB3	2.12	0.49
2:B:572:GLU:C	2:B:574:ASN:N	2.66	0.49
2:B:585:GLY:O	2:B:589:SER:OG	2.30	0.49
3:M:223:HIS:CD2	3:M:479:PHE:CD2	3.01	0.49
3:M:250:LEU:HD13	3:M:254:PRO:CG	2.43	0.49
3:M:280:ASP:N	3:M:280:ASP:OD1	2.45	0.49
3:M:476:THR:OG1	3:M:477:GLY:N	2.46	0.49
4:S:83:LEU:CD1	4:S:116:VAL:CG1	2.90	0.49
1:A:535:ILE:O	1:A:536:MET:HG3	2.12	0.49
1:A:566:PHE:CD1	1:A:567:GLN:N	2.81	0.49
2:B:305:SER:O	2:B:309:LEU:CD2	2.61	0.49
2:B:316:THR:OG1	3:M:90:PHE:CZ	2.51	0.49
2:B:367:SER:OG	2:B:401:THR:CG2	2.60	0.49
2:B:371:GLN:HG2	2:B:401:THR:O	2.10	0.49
3:M:69:ILE:HD12	3:M:93:LEU:HB3	1.95	0.49
3:M:113:LYS:O	3:M:117:ASN:ND2	2.38	0.49
3:M:217:ASP:C	3:M:472:TYR:CE2	2.86	0.49
3:M:221:THR:CA	3:M:474:THR:OG1	2.53	0.49
3:M:235:LEU:CD2	3:M:307:SER:HA	2.42	0.49
3:M:316:ARG:HG3	3:M:322:LEU:CD1	2.42	0.49
3:M:338:PHE:CD2	3:M:415:ILE:HD11	2.48	0.49
3:M:437:TYR:CE1	3:M:479:PHE:CD1	3.01	0.49
4:S:53:THR:HG22	4:S:54:PRO:O	2.11	0.49
1:A:179:LYS:HE3	4:S:140:MET:HG2	1.95	0.49
1:A:183:THR:OG1	4:S:137:GLN:C	2.50	0.49
2:B:78:ASP:OD1	2:B:80:GLN:HB2	2.13	0.49
2:B:90:ILE:N	2:B:101:ILE:HD13	2.28	0.49
2:B:215:TYR:HE2	2:B:229:HIS:ND1	2.10	0.49
2:B:260:LEU:O	2:B:261:PRO:C	2.39	0.49
2:B:366:LEU:O	2:B:368:ILE:N	2.45	0.49
2:B:427:ASN:HA	2:B:430:ILE:HD12	1.95	0.49
2:B:479:VAL:CG1	2:B:486:HIS:ND1	2.15	0.49
2:B:500:GLN:NE2	2:B:503:LEU:HD21	2.27	0.49
2:B:534:ILE:CG1	2:B:595:VAL:HG23	2.42	0.49
2:B:596:LEU:HD22	2:B:615:SER:CB	2.43	0.49

Continued on next page...

Continued from previous page...

Atom-1	Atom-2	Interatomic distance (Å)	Clash overlap (Å)
3:M:7:ILE:HG13	3:M:16:PHE:HD2	1.78	0.49
3:M:15:ILE:CG2	3:M:114:ILE:CG2	2.91	0.49
3:M:245:ASP:C	3:M:246:VAL:CG2	2.81	0.49
3:M:300:LEU:O	3:M:300:LEU:HD12	2.13	0.49
4:S:17:VAL:HG13	4:S:17:VAL:O	2.13	0.49
1:A:97:SER:C	1:A:98:ASN:O	2.32	0.49
1:A:99:LYS:HG2	1:A:101:GLN:N	2.27	0.49
1:A:400:VAL:O	1:A:403:LEU:HB2	2.13	0.49
2:B:83:PHE:CE1	2:B:105:LEU:HA	2.48	0.49
2:B:133:GLU:CB	2:B:168:MET:SD	3.01	0.49
2:B:230:PHE:HB3	2:B:298:ASP:OD2	2.12	0.49
2:B:237:ILE:CG2	2:B:305:SER:HB3	2.42	0.49
2:B:336:ASN:C	2:B:373:LEU:CD2	2.81	0.49
2:B:530:LEU:CD2	2:B:591:MET:CB	2.89	0.49
3:M:76:CYS:HB3	3:M:93:LEU:HD22	1.94	0.49
3:M:219:LEU:CD2	3:M:473:LYS:HA	2.34	0.49
4:S:136:VAL:O	4:S:139:GLY:C	2.50	0.49
1:A:320:HIS:HB2	1:A:352:PHE:HE2	1.74	0.49
1:A:488:ARG:CD	1:A:522:PHE:CE2	2.96	0.49
2:B:106:LEU:HD11	2:B:144:ASP:HB3	1.89	0.49
2:B:182:ARG:HD2	2:B:217:GLU:CG	2.42	0.49
2:B:261:PRO:HB2	2:B:290:SER:OG	2.13	0.49
2:B:276:SER:CB	2:B:289:PRO:HG3	2.43	0.49
2:B:398:ILE:CG2	2:B:402:LEU:HD11	2.43	0.49
2:B:400:SER:CA	2:B:439:CYS:SG	3.01	0.49
2:B:463:LEU:HD13	2:B:467:VAL:CG1	2.43	0.49
2:B:546:CYS:N	2:B:607:ILE:CD1	2.76	0.49
3:M:56:VAL:HB	3:M:64:LYS:HG3	1.93	0.49
2:B:102:HIS:HE2	2:B:138:ALA:CB	2.25	0.48
2:B:256:CYS:HB3	2:B:328:LEU:CD2	2.42	0.48
2:B:513:TRP:NE1	2:B:517:GLU:CG	2.76	0.48
3:M:256:VAL:CG2	3:M:452:ILE:CG2	2.91	0.48
1:A:304:LEU:C	1:A:304:LEU:HD23	2.33	0.48
1:A:309:PHE:CE1	1:A:348:PHE:CE2	3.00	0.48
1:A:435:ILE:O	1:A:441:TYR:CE1	2.66	0.48
2:B:37:TYR:HD2	2:B:38:TYR:CE1	2.29	0.48
2:B:403:ILE:CB	2:B:408:VAL:HG22	2.31	0.48
2:B:448:SER:OG	2:B:485:LYS:HE3	2.14	0.48
2:B:490:ILE:CG2	2:B:515:PHE:CD2	2.96	0.48
3:M:222:PHE:CD1	3:M:222:PHE:N	2.81	0.48
3:M:223:HIS:CD2	3:M:479:PHE:CE2	3.01	0.48

Continued on next page...

Continued from previous page...

Atom-1	Atom-2	Interatomic distance (Å)	Clash overlap (Å)
3:M:252:ASP:O	3:M:254:PRO:HD2	2.14	0.48
3:M:265:ASN:O	3:M:266:ASP:C	2.47	0.48
4:S:112:CYS:HB2	4:S:113:PHE:CE1	2.48	0.48
1:A:176:TYR:OH	4:S:148:ARG:CB	2.45	0.48
1:A:179:LYS:CE	4:S:140:MET:C	2.81	0.48
1:A:237:SER:N	1:A:238:PRO:CD	2.75	0.48
1:A:356:ILE:HD13	1:A:374:LEU:HB3	1.94	0.48
2:B:133:GLU:HG2	2:B:168:MET:SD	2.53	0.48
2:B:337:THR:OG1	2:B:373:LEU:HD13	2.13	0.48
2:B:550:VAL:O	2:B:553:ALA:HB3	2.12	0.48
3:M:54:SER:HB2	3:M:66:PHE:CD1	2.48	0.48
3:M:218:LEU:HD21	3:M:449:VAL:HG23	1.95	0.48
3:M:220:GLU:CD	3:M:222:PHE:CZ	2.87	0.48
3:M:252:ASP:C	3:M:254:PRO:HD2	2.33	0.48
3:M:290:PHE:CZ	3:M:291:ILE:O	2.66	0.48
1:A:226:SER:HB2	1:A:263:LEU:HD23	1.96	0.48
1:A:384:LEU:HD12	1:A:384:LEU:C	2.34	0.48
1:A:433:ILE:HD11	1:A:473:ILE:HA	1.95	0.48
2:B:136:CYS:CB	2:B:172:GLU:CG	2.84	0.48
2:B:181:TYR:CD2	2:B:218:CYS:HA	2.48	0.48
2:B:243:TRP:CH2	3:M:95:THR:HA	2.47	0.48
2:B:280:PRO:HG2	2:B:283:TYR:HD1	1.63	0.48
2:B:383:VAL:CG2	2:B:384:PHE:N	2.75	0.48
3:M:56:VAL:H	3:M:64:LYS:CB	2.26	0.48
3:M:100:LEU:HD23	3:M:124:ILE:CD1	2.44	0.48
4:S:53:THR:HG21	4:S:68:VAL:CA	2.28	0.48
1:A:403:LEU:C	2:B:3:ASP:CB	2.67	0.48
1:A:509:PRO:HB3	1:A:547:VAL:HG23	1.93	0.48
2:B:220:ALA:HA	2:B:258:GLN:HG3	1.95	0.48
2:B:343:LEU:HD11	2:B:359:LEU:HD13	1.95	0.48
2:B:343:LEU:HD12	2:B:359:LEU:CD1	2.43	0.48
2:B:352:ASN:OD1	3:M:49:ASP:HA	2.12	0.48
3:M:84:LYS:O	3:M:88:ASP:CB	2.61	0.48
3:M:213:GLU:C	3:M:467:TYR:HB2	2.34	0.48
3:M:244:VAL:O	3:M:299:LEU:CB	2.61	0.48
4:S:65:ASN:O	4:S:66:ASP:C	2.46	0.48
4:S:89:VAL:CG1	4:S:98:ILE:HD12	2.43	0.48
1:A:158:LEU:HD12	1:A:162:ILE:HG13	1.95	0.48
2:B:530:LEU:HD11	2:B:595:VAL:HG21	1.95	0.48
2:B:540:GLU:OE1	2:B:548:ILE:HD11	2.13	0.48
3:M:95:THR:CG2	3:M:137:SER:HA	2.43	0.48

Continued on next page...

Continued from previous page...

Atom-1	Atom-2	Interatomic distance (Å)	Clash overlap (Å)
3:M:99:ILE:CG2	3:M:99:ILE:O	2.62	0.48
3:M:379:LEU:HB3	3:M:411:LEU:CD1	2.44	0.48
1:A:174:ARG:HH22	4:S:148:ARG:HH22	1.51	0.48
1:A:186:PHE:CE2	1:A:187:LYS:HD3	2.48	0.48
2:B:51:LEU:HD11	2:B:66:ILE:CD1	2.44	0.48
2:B:126:SER:O	2:B:127:LEU:C	2.47	0.48
2:B:155:LEU:O	2:B:158:VAL:N	2.46	0.48
2:B:175:LEU:HG	2:B:210:CYS:HB3	1.93	0.48
2:B:176:ALA:C	2:B:178:ILE:H	2.15	0.48
2:B:193:LEU:HB3	2:B:225:LEU:CG	2.43	0.48
2:B:461:HIS:HB2	2:B:463:LEU:CD2	2.44	0.48
2:B:467:VAL:HG12	2:B:471:TYR:CD1	2.49	0.48
2:B:537:PHE:CZ	2:B:545:ARG:HB3	2.48	0.48
3:M:41:LEU:HB3	3:M:51:LEU:HA	1.96	0.48
1:A:375:VAL:CG1	1:A:387:ILE:HG21	2.44	0.48
1:A:384:LEU:CD1	1:A:385:LYS:N	2.77	0.48
1:A:579:LYS:O	1:A:582:ILE:HB	2.14	0.48
1:A:581:LEU:HD23	1:A:607:LEU:CG	2.43	0.48
2:B:13:ASP:O	2:B:16:LYS:N	2.47	0.48
2:B:21:GLU:HA	2:B:24:ALA:HB2	1.86	0.48
2:B:538:SER:HB3	2:B:598:LEU:HD22	1.94	0.48
3:M:104:PHE:CZ	3:M:120:ARG:HB2	2.49	0.48
3:M:214:LEU:C	3:M:467:TYR:HB3	2.33	0.48
3:M:240:ILE:HG22	3:M:444:ALA:CB	2.41	0.48
3:M:242:GLY:N	3:M:444:ALA:CB	2.76	0.48
3:M:290:PHE:HZ	3:M:293:PRO:CD	2.27	0.48
2:B:219:TYR:CE2	2:B:226:LEU:CA	2.79	0.48
2:B:219:TYR:C	2:B:221:ASP:N	2.58	0.48
2:B:393:ILE:CG2	2:B:431:MET:HE3	2.43	0.48
2:B:483:PRO:HA	2:B:486:HIS:CB	2.43	0.48
2:B:588:ILE:O	2:B:591:MET:HB2	2.14	0.48
3:M:65:TYR:CE1	3:M:66:PHE:O	2.67	0.48
3:M:245:ASP:CB	3:M:472:TYR:CE1	2.95	0.48
3:M:356:LEU:HD11	3:M:437:TYR:CE2	2.49	0.48
3:M:386:PHE:CB	3:M:397:TRP:CD1	2.97	0.48
1:A:76:TYR:O	1:A:80:TYR:CD1	2.67	0.48
1:A:121:LEU:HG	1:A:158:LEU:HD22	1.94	0.48
1:A:516:ILE:HG12	1:A:551:LEU:HD13	1.96	0.48
1:A:617:ASP:OD1	1:A:618:THR:N	2.45	0.48
2:B:306:LEU:CD1	2:B:325:LEU:CD2	2.91	0.48
2:B:334:MET:O	2:B:336:ASN:C	2.51	0.48

Continued on next page...

Continued from previous page...

Atom-1	Atom-2	Interatomic distance (Å)	Clash overlap (Å)
2:B:341:GLU:CB	2:B:377:TYR:OH	2.60	0.48
2:B:508:ARG:HB3	2:B:544:THR:HG23	1.95	0.48
2:B:587:ARG:O	2:B:591:MET:HG2	2.14	0.48
3:M:212:ASN:HB3	3:M:250:LEU:HA	1.96	0.48
3:M:435:LEU:O	3:M:479:PHE:CE2	2.60	0.48
4:S:1:MET:SD	4:S:20:TYR:CD2	3.06	0.48
1:A:156:PRO:HA	1:A:192:TYR:CD1	2.49	0.47
1:A:438:ALA:O	1:A:441:TYR:HD1	1.96	0.47
1:A:461:CYS:SG	1:A:464:ILE:HG22	2.54	0.47
1:A:533:ILE:HG13	1:A:562:TRP:CH2	2.44	0.47
1:A:637:GLU:CG	2:B:516:GLY:C	2.83	0.47
2:B:108:PHE:CD2	2:B:112:ASP:HB3	2.49	0.47
2:B:230:PHE:CZ	2:B:234:CYS:SG	3.06	0.47
2:B:231:ARG:NH2	2:B:279:LEU:CD2	2.70	0.47
2:B:237:ILE:HG21	2:B:305:SER:CB	2.43	0.47
2:B:311:TYR:HD1	3:M:269:ILE:HD11	1.79	0.47
2:B:367:SER:CB	2:B:401:THR:OG1	2.62	0.47
2:B:578:PRO:CB	2:B:579:PRO:CD	2.92	0.47
2:B:586:SER:O	2:B:590:GLN:HG3	2.13	0.47
2:B:592:TYR:CG	2:B:593:ASN:N	2.82	0.47
3:M:45:SER:HA	3:M:47:SER:N	2.28	0.47
3:M:104:PHE:N	3:M:104:PHE:CD1	2.66	0.47
3:M:290:PHE:HZ	3:M:293:PRO:CG	2.26	0.47
4:S:16:LEU:HD12	4:S:17:VAL:N	2.29	0.47
1:A:289:SER:HA	4:S:96:LEU:CD1	2.42	0.47
1:A:412:LYS:O	1:A:414:LYS:N	2.47	0.47
2:B:486:HIS:CD2	2:B:518:ILE:CG1	2.97	0.47
2:B:494:ALA:N	2:B:515:PHE:CZ	2.76	0.47
2:B:500:GLN:OE1	2:B:503:LEU:HD21	2.14	0.47
2:B:546:CYS:HB2	2:B:607:ILE:HG13	1.80	0.47
2:B:592:TYR:HH	2:B:619:ASP:CG	2.16	0.47
3:M:309:GLN:O	3:M:313:SER:N	2.40	0.47
3:M:386:PHE:CB	3:M:397:TRP:HD1	2.27	0.47
4:S:58:LEU:HD13	4:S:69:ASN:N	2.28	0.47
1:A:504:ILE:O	1:A:505:ASN:O	2.33	0.47
1:A:624:LEU:O	1:A:627:GLU:HB2	2.15	0.47
2:B:74:ASP:O	2:B:77:ILE:CG1	2.62	0.47
2:B:85:ASP:O	2:B:89:ASN:ND2	2.47	0.47
2:B:120:ILE:HG13	2:B:150:LEU:HD13	1.96	0.47
2:B:279:LEU:O	2:B:280:PRO:C	2.52	0.47
2:B:475:ILE:CG2	2:B:489:ILE:CG2	2.90	0.47

Continued on next page...

Continued from previous page...

Atom-1	Atom-2	Interatomic distance (Å)	Clash overlap (Å)
2:B:490:ILE:HG23	2:B:515:PHE:CE2	2.47	0.47
2:B:553:ALA:CB	2:B:614:ILE:CG2	2.92	0.47
2:B:570:GLY:C	2:B:571:SER:O	2.35	0.47
3:M:44:ASP:C	3:M:47:SER:H	2.18	0.47
3:M:56:VAL:H	3:M:64:LYS:HG3	1.77	0.47
3:M:121:ILE:HG23	3:M:125:PHE:CE1	2.49	0.47
3:M:302:TYR:CD2	3:M:445:SER:CB	2.88	0.47
3:M:320:ILE:CG2	3:M:439:TYR:OH	2.62	0.47
3:M:410:VAL:HG11	3:M:412:ARG:CZ	2.45	0.47
1:A:436:CYS:SG	1:A:450:TYR:CD2	3.03	0.47
1:A:626:SER:O	1:A:630:PRO:HD2	2.15	0.47
2:B:189:HIS:NE2	2:B:222:HIS:CG	2.82	0.47
2:B:200:MET:HE1	2:B:228:GLY:O	2.14	0.47
2:B:215:TYR:HD1	2:B:233:TYR:CZ	2.26	0.47
2:B:490:ILE:CD1	2:B:518:ILE:CG2	2.87	0.47
3:M:265:ASN:O	3:M:267:ILE:N	2.47	0.47
3:M:317:MET:CB	3:M:320:ILE:O	2.60	0.47
3:M:373:ALA:HB3	3:M:418:GLU:O	2.15	0.47
4:S:8:PHE:CE2	4:S:84:TYR:O	2.67	0.47
1:A:275:LEU:HA	1:A:278:ILE:CG1	2.45	0.47
1:A:316:LEU:CD1	1:A:348:PHE:CE2	2.97	0.47
1:A:356:ILE:HD11	1:A:374:LEU:HD23	1.95	0.47
1:A:438:ALA:HB3	1:A:441:TYR:CE1	2.48	0.47
1:A:595:GLU:HA	2:B:476:ARG:NH1	2.30	0.47
2:B:95:THR:HG23	2:B:137:PHE:CE2	2.50	0.47
2:B:196:LEU:HB2	2:B:229:HIS:CE1	2.48	0.47
2:B:352:ASN:HD21	3:M:70:ASN:HB2	1.74	0.47
2:B:546:CYS:O	2:B:549:LEU:HB3	2.14	0.47
2:B:553:ALA:HA	2:B:614:ILE:HG23	1.96	0.47
2:B:563:PHE:O	2:B:564:LYS:O	2.32	0.47
3:M:214:LEU:H	3:M:466:LEU:HA	1.79	0.47
3:M:325:LEU:HA	3:M:339:GLU:O	2.14	0.47
3:M:376:ILE:HG22	3:M:379:LEU:CD1	2.45	0.47
3:M:443:SER:HB3	3:M:447:ILE:CA	2.45	0.47
1:A:92:LEU:HD13	1:A:123:LEU:HB3	1.96	0.47
1:A:316:LEU:O	1:A:319:LEU:HB2	2.14	0.47
2:B:14:THR:O	2:B:15:ALA:C	2.53	0.47
2:B:41:ASN:HB3	2:B:43:ASN:CG	2.32	0.47
2:B:267:ASP:OD1	2:B:269:SER:HB2	2.14	0.47
2:B:302:PHE:CE1	2:B:306:LEU:HD21	2.49	0.47
2:B:343:LEU:HD23	2:B:363:ILE:CD1	2.32	0.47

Continued on next page...

Continued from previous page...

Atom-1	Atom-2	Interatomic distance (Å)	Clash overlap (Å)
2:B:588:ILE:HG21	2:B:618:PHE:CE1	2.44	0.47
3:M:62:VAL:HG12	3:M:64:LYS:HD3	1.97	0.47
3:M:99:ILE:HD13	3:M:128:CYS:SG	2.55	0.47
3:M:220:GLU:OE1	3:M:222:PHE:CZ	2.68	0.47
1:A:140:VAL:O	1:A:141:VAL:O	2.32	0.47
1:A:166:LEU:HD12	1:A:185:LEU:HD23	1.96	0.47
1:A:170:LEU:HD13	1:A:181:ALA:HB3	1.96	0.47
1:A:222:ILE:HG23	1:A:233:PHE:CD2	2.49	0.47
1:A:253:ILE:HG12	1:A:281:LEU:HD13	1.97	0.47
1:A:263:LEU:O	1:A:266:VAL:C	2.52	0.47
1:A:268:PRO:HA	1:A:271:ARG:HE	1.79	0.47
1:A:397:ASP:O	1:A:418:ILE:CD1	2.63	0.47
1:A:400:VAL:H	1:A:418:ILE:HG12	1.79	0.47
1:A:429:VAL:CG1	1:A:469:LEU:CD1	2.91	0.47
1:A:433:ILE:HG23	1:A:476:GLN:HB2	1.96	0.47
1:A:438:ALA:O	1:A:440:ASN:N	2.35	0.47
1:A:566:PHE:CZ	1:A:618:THR:O	2.57	0.47
2:B:39:SER:HG	2:B:40:GLN:N	2.13	0.47
2:B:60:ARG:O	2:B:63:MET:N	2.45	0.47
2:B:101:ILE:O	2:B:104:TYR:HB3	2.14	0.47
2:B:155:LEU:CG	2:B:188:TYR:HD2	2.27	0.47
2:B:260:LEU:HD22	2:B:291:TYR:OH	2.15	0.47
2:B:343:LEU:CD1	2:B:359:LEU:CD1	2.92	0.47
2:B:347:VAL:HG22	2:B:359:LEU:CD1	2.44	0.47
2:B:497:LEU:HB2	2:B:511:ILE:HG21	1.95	0.47
3:M:48:ASP:CA	3:M:75:TRP:CZ2	2.95	0.47
3:M:111:ILE:HG13	3:M:112:LYS:N	2.29	0.47
3:M:362:PHE:O	3:M:364:VAL:N	2.48	0.47
4:S:83:LEU:HD11	4:S:116:VAL:HG13	1.95	0.47
1:A:136:GLY:O	1:A:139:ASP:CA	2.62	0.47
1:A:223:CYS:SG	1:A:259:LEU:HA	2.55	0.47
1:A:244:LEU:HD13	1:A:256:LEU:CB	2.44	0.47
1:A:421:PRO:HG2	1:A:424:TYR:CD1	2.49	0.47
1:A:429:VAL:HG11	1:A:473:ILE:CG1	2.45	0.47
1:A:637:GLU:HG3	2:B:516:GLY:O	2.14	0.47
2:B:70:MET:HE2	2:B:104:TYR:CD1	2.50	0.47
2:B:303:LEU:HD13	2:B:333:GLN:HB3	1.95	0.47
2:B:340:ILE:HG13	2:B:373:LEU:HB3	1.96	0.47
2:B:418:TYR:CE1	2:B:419:VAL:HA	2.49	0.47
2:B:592:TYR:HD2	2:B:618:PHE:CE2	2.29	0.47
3:M:220:GLU:OE1	3:M:222:PHE:CE1	2.68	0.47

Continued on next page...

Continued from previous page...

Atom-1	Atom-2	Interatomic distance (Å)	Clash overlap (Å)
3:M:419:ASN:ND2	3:M:424:PHE:HD2	2.12	0.47
1:A:180:LYS:HD3	4:S:137:GLN:HB2	1.97	0.47
1:A:313:MET:HB2	1:A:348:PHE:CZ	2.50	0.47
1:A:555:LEU:O	1:A:559:PHE:HD1	1.97	0.47
1:A:638:LEU:HA	2:B:520:SER:HB3	1.89	0.47
2:B:2:VAL:HG12	2:B:6:HIS:CE1	2.49	0.47
2:B:18:ILE:HB	2:B:23:ALA:CB	2.18	0.47
2:B:278:PRO:CD	2:B:292:GLU:CG	2.69	0.47
2:B:374:PHE:CD2	2:B:402:LEU:CG	2.93	0.47
2:B:490:ILE:HG23	2:B:515:PHE:CD2	2.50	0.47
2:B:534:ILE:O	2:B:535:GLN:C	2.54	0.47
2:B:610:ARG:O	2:B:614:ILE:HG13	2.15	0.47
3:M:64:LYS:HZ2	3:M:79:SER:HB2	1.80	0.47
3:M:242:GLY:HA2	3:M:444:ALA:HB2	1.94	0.47
3:M:466:LEU:HD23	3:M:467:TYR:O	2.15	0.47
1:A:150:LEU:O	1:A:153:ILE:N	2.47	0.47
1:A:192:TYR:CE2	1:A:194:GLU:HB2	2.50	0.47
1:A:275:LEU:C	1:A:275:LEU:HD23	2.35	0.47
1:A:441:TYR:HB3	1:A:444:VAL:HG23	1.96	0.47
1:A:446:ASP:CB	1:A:448:GLU:O	2.63	0.47
1:A:569:ASP:C	1:A:571:ARG:H	2.18	0.47
1:A:605:GLU:OE2	1:A:632:PHE:CZ	2.68	0.47
3:M:273:HIS:CB	3:M:298:ARG:O	2.59	0.47
3:M:376:ILE:CG2	3:M:379:LEU:HD11	2.45	0.47
3:M:435:LEU:H	3:M:479:PHE:HB2	1.78	0.47
4:S:38:LEU:CB	4:S:51:LEU:HD13	2.44	0.47
1:A:278:ILE:O	1:A:280:GLU:N	2.44	0.46
1:A:292:TYR:O	1:A:292:TYR:HD1	1.98	0.46
1:A:436:CYS:SG	1:A:450:TYR:CD1	3.05	0.46
1:A:449:TRP:O	1:A:453:VAL:HG23	2.15	0.46
2:B:21:GLU:O	2:B:25:VAL:HG23	2.13	0.46
2:B:127:LEU:HD22	2:B:157:THR:CG2	2.44	0.46
2:B:127:LEU:O	2:B:135:ARG:HD3	2.15	0.46
2:B:340:ILE:HD11	2:B:366:LEU:HB3	1.97	0.46
2:B:440:GLY:C	2:B:442:LEU:N	2.66	0.46
2:B:494:ALA:CA	2:B:515:PHE:CZ	2.98	0.46
3:M:64:LYS:HB3	3:M:66:PHE:CE1	2.51	0.46
3:M:224:VAL:CG1	3:M:226:PHE:CZ	2.96	0.46
3:M:319:SER:CA	3:M:343:ASN:O	2.63	0.46
3:M:338:PHE:CE2	3:M:415:ILE:CD1	2.98	0.46
4:S:8:PHE:HB3	4:S:36:TYR:CZ	2.50	0.46

Continued on next page...

Continued from previous page...

Atom-1	Atom-2	Interatomic distance (Å)	Clash overlap (Å)
4:S:8:PHE:HA	4:S:13:GLN:O	2.16	0.46
1:A:406:GLY:N	2:B:7:ARG:NH1	2.55	0.46
1:A:435:ILE:O	1:A:441:TYR:CZ	2.68	0.46
1:A:476:GLN:OE1	1:A:476:GLN:HA	2.15	0.46
1:A:485:PRO:C	1:A:486:SER:O	2.48	0.46
2:B:66:ILE:CG2	2:B:104:TYR:CE1	2.95	0.46
2:B:215:TYR:CD1	2:B:233:TYR:CZ	2.98	0.46
2:B:279:LEU:CG	2:B:288:TYR:HD1	2.22	0.46
2:B:325:LEU:O	2:B:326:TYR:C	2.39	0.46
2:B:375:LEU:HB2	2:B:376:PRO:HD3	1.97	0.46
2:B:397:GLN:HE21	2:B:431:MET:CE	2.27	0.46
2:B:537:PHE:HE1	2:B:545:ARG:HB3	1.78	0.46
1:A:381:GLU:O	1:A:383:ASN:N	2.46	0.46
1:A:401:VAL:HG23	1:A:418:ILE:C	2.35	0.46
1:A:559:PHE:CD1	1:A:581:LEU:HD22	2.47	0.46
2:B:108:PHE:CD2	2:B:115:LEU:CB	2.98	0.46
2:B:347:VAL:CG2	2:B:359:LEU:HD12	2.46	0.46
2:B:578:PRO:CD	2:B:581:TYR:HE1	2.26	0.46
3:M:244:VAL:HG13	3:M:472:TYR:HE2	1.78	0.46
3:M:253:ASN:O	3:M:254:PRO:C	2.53	0.46
4:S:38:LEU:HB3	4:S:51:LEU:HD11	1.97	0.46
1:A:77:LEU:O	1:A:82:PHE:CD2	2.69	0.46
1:A:193:PRO:O	1:A:196:LEU:HB3	2.16	0.46
1:A:429:VAL:HG11	1:A:469:LEU:CD1	2.44	0.46
1:A:461:CYS:HB2	1:A:469:LEU:HD23	1.96	0.46
2:B:44:PRO:O	2:B:47:LEU:HB2	2.14	0.46
2:B:197:LYS:C	2:B:199:LEU:N	2.67	0.46
2:B:227:HIS:HE1	2:B:292:GLU:HG2	1.73	0.46
2:B:252:LEU:HB3	2:B:302:PHE:CD1	2.49	0.46
2:B:306:LEU:HD12	2:B:325:LEU:HD21	1.98	0.46
2:B:383:VAL:HG22	2:B:384:PHE:N	2.31	0.46
2:B:383:VAL:HA	2:B:395:LYS:HE3	1.97	0.46
2:B:481:LYS:C	2:B:483:PRO:HD3	2.35	0.46
2:B:570:GLY:O	2:B:571:SER:C	2.50	0.46
3:M:134:PRO:O	3:M:136:VAL:HG23	2.14	0.46
1:A:399:ASP:N	1:A:418:ILE:HD11	2.27	0.46
1:A:595:GLU:CG	2:B:476:ARG:HH22	2.29	0.46
2:B:191:GLU:O	2:B:195:ILE:HG13	2.15	0.46
2:B:227:HIS:C	2:B:298:ASP:OD2	2.54	0.46
2:B:276:SER:HB3	2:B:289:PRO:HG3	1.96	0.46
2:B:334:MET:CE	2:B:339:PHE:CE1	2.98	0.46

Continued on next page...

Continued from previous page...

Atom-1	Atom-2	Interatomic distance (Å)	Clash overlap (Å)
3:M:347:PHE:HE1	3:M:350:VAL:CG1	2.14	0.46
3:M:347:PHE:CD2	3:M:350:VAL:O	2.68	0.46
3:M:353:VAL:O	3:M:401:LYS:HB2	2.15	0.46
1:A:182:ILE:HG23	1:A:221:VAL:HG21	1.97	0.46
1:A:219:VAL:HG13	1:A:259:LEU:HD13	1.97	0.46
1:A:241:TYR:C	1:A:241:TYR:CD2	2.88	0.46
2:B:17:VAL:O	2:B:18:ILE:C	2.54	0.46
2:B:18:ILE:CB	2:B:23:ALA:HB2	2.18	0.46
2:B:212:VAL:CG2	2:B:233:TYR:CD2	2.99	0.46
2:B:231:ARG:C	2:B:233:TYR:N	2.65	0.46
2:B:249:ILE:HD11	2:B:321:CYS:SG	2.55	0.46
3:M:235:LEU:HD13	3:M:306:LEU:HB3	1.91	0.46
3:M:270:PRO:HA	3:M:302:TYR:HD1	1.79	0.46
3:M:435:LEU:HD12	3:M:435:LEU:N	2.31	0.46
4:S:61:ASN:O	4:S:62:GLU:C	2.53	0.46
1:A:244:LEU:CB	1:A:256:LEU:HD13	2.46	0.46
2:B:115:LEU:HD13	2:B:115:LEU:HA	1.83	0.46
2:B:231:ARG:CD	2:B:297:PRO:HB2	2.45	0.46
2:B:356:LYS:HE3	3:M:49:ASP:OD2	2.16	0.46
2:B:508:ARG:HB2	2:B:544:THR:HG21	1.97	0.46
2:B:594:ALA:O	2:B:598:LEU:HG	2.15	0.46
3:M:66:PHE:HB3	3:M:77:LEU:CD1	2.42	0.46
3:M:256:VAL:O	3:M:289:THR:HA	2.16	0.46
4:S:35:VAL:CG1	4:S:77:TYR:OH	2.63	0.46
4:S:50:PHE:O	4:S:51:LEU:HD23	2.15	0.46
1:A:151:SER:HB3	1:A:184:ALA:HA	1.98	0.46
1:A:189:PHE:O	1:A:190:LEU:C	2.51	0.46
1:A:200:PHE:HZ	1:A:236:LEU:HD23	1.70	0.46
1:A:280:GLU:O	1:A:283:GLU:HB3	2.16	0.46
1:A:369:SER:CB	1:A:424:TYR:CE2	2.64	0.46
2:B:20:ARG:HD2	2:B:21:GLU:CB	2.30	0.46
2:B:245:GLN:NE2	2:B:309:LEU:HD13	2.31	0.46
3:M:376:ILE:CD1	3:M:415:ILE:HG12	2.45	0.46
4:S:65:ASN:O	4:S:67:GLU:CG	2.61	0.46
1:A:381:GLU:CA	1:A:384:LEU:HD23	2.36	0.46
2:B:108:PHE:C	2:B:110:GLU:N	2.64	0.46
2:B:155:LEU:HB2	2:B:188:TYR:CE2	2.51	0.46
2:B:252:LEU:CG	2:B:302:PHE:CE1	2.95	0.46
2:B:310:ILE:HA	2:B:318:ILE:HG12	1.98	0.46
2:B:340:ILE:HG13	2:B:373:LEU:CD2	2.43	0.46
2:B:400:SER:HB2	2:B:439:CYS:SG	2.55	0.46

Continued on next page...

Continued from previous page...

Atom-1	Atom-2	Interatomic distance (Å)	Clash overlap (Å)
2:B:527:PRO:HB2	2:B:587:ARG:HD2	1.98	0.46
3:M:6:TYR:O	3:M:77:LEU:N	2.32	0.46
3:M:51:LEU:HB3	3:M:68:VAL:HG21	1.98	0.46
3:M:217:ASP:O	3:M:217:ASP:OD1	2.34	0.46
3:M:379:LEU:HD22	3:M:397:TRP:NE1	2.31	0.46
1:A:163:ALA:CB	1:A:199:ASN:ND2	2.60	0.46
1:A:604:LEU:HD23	1:A:604:LEU:C	2.35	0.46
2:B:90:ILE:HD11	2:B:123:LEU:HD21	1.97	0.46
2:B:182:ARG:HD2	2:B:217:GLU:HB3	1.98	0.46
2:B:237:ILE:HG23	2:B:238:LYS:N	2.30	0.46
2:B:245:GLN:CB	2:B:309:LEU:HD12	2.45	0.46
2:B:313:SER:O	2:B:315:PRO:HD3	2.15	0.46
2:B:430:ILE:HG23	2:B:470:ALA:HB2	1.98	0.46
3:M:9:ASP:C	3:M:9:ASP:OD1	2.54	0.46
3:M:249:TYR:CE1	3:M:467:TYR:CZ	3.04	0.46
3:M:271:SER:C	3:M:272:LEU:HD12	2.36	0.46
3:M:338:PHE:HE2	3:M:415:ILE:CG1	2.27	0.46
3:M:340:LEU:HG	3:M:342:LEU:HD11	1.96	0.46
3:M:379:LEU:HD23	3:M:411:LEU:CD2	2.45	0.46
1:A:121:LEU:HD11	1:A:153:ILE:HG22	1.96	0.45
1:A:154:ILE:HG21	1:A:191:GLN:HG3	1.85	0.45
1:A:216:SER:HB2	1:A:252:ILE:CG1	2.39	0.45
1:A:364:ASP:O	1:A:367:ILE:HB	2.16	0.45
1:A:420:ILE:HA	1:A:421:PRO:HD3	1.80	0.45
1:A:516:ILE:HG21	1:A:551:LEU:HA	1.97	0.45
2:B:193:LEU:HD13	2:B:225:LEU:HD11	1.91	0.45
2:B:250:GLU:O	2:B:253:ILE:HB	2.16	0.45
2:B:396:ILE:HG22	2:B:435:SER:OG	2.15	0.45
2:B:467:VAL:HG12	2:B:471:TYR:HD1	1.81	0.45
2:B:475:ILE:CG2	2:B:514:LEU:HD21	2.46	0.45
2:B:478:LEU:O	2:B:480:GLN:N	2.49	0.45
3:M:262:THR:HG22	3:M:264:GLY:CA	2.46	0.45
4:S:8:PHE:HE2	4:S:86:THR:H	1.63	0.45
4:S:73:ILE:HG23	4:S:88:ILE:CG2	2.46	0.45
4:S:113:PHE:CD1	4:S:113:PHE:N	2.84	0.45
1:A:329:ASN:O	1:A:333:ILE:HG13	2.17	0.45
1:A:404:GLN:N	2:B:3:ASP:OD2	2.42	0.45
1:A:588:LEU:O	1:A:590:TYR:N	2.49	0.45
1:A:601:VAL:O	1:A:602:GLU:O	2.34	0.45
1:A:625:LEU:C	1:A:627:GLU:N	2.69	0.45
2:B:9:ALA:HA	2:B:12:LEU:HD12	1.99	0.45

Continued on next page...

Continued from previous page...

Atom-1	Atom-2	Interatomic distance (Å)	Clash overlap (Å)
2:B:102:HIS:HB3	2:B:141:ALA:HB2	1.98	0.45
2:B:226:LEU:CD2	2:B:255:TYR:HD1	1.99	0.45
2:B:280:PRO:CG	2:B:283:TYR:CE1	2.81	0.45
2:B:452:LYS:NZ	2:B:456:ASP:CG	2.69	0.45
2:B:559:ASP:C	2:B:563:PHE:HB2	2.36	0.45
1:A:203:PHE:HZ	1:A:221:VAL:HG11	1.78	0.45
1:A:226:SER:O	1:A:227:LYS:C	2.53	0.45
1:A:403:LEU:O	2:B:3:ASP:OD2	2.26	0.45
2:B:208:ILE:HD13	2:B:236:ILE:HD13	1.97	0.45
3:M:16:PHE:HE2	3:M:125:PHE:CE2	2.34	0.45
3:M:317:MET:SD	3:M:321:GLY:HA3	2.56	0.45
3:M:449:VAL:CG1	3:M:452:ILE:HG13	2.46	0.45
1:A:528:ASN:O	1:A:529:GLY:O	2.34	0.45
1:A:581:LEU:CG	1:A:607:LEU:HD11	2.46	0.45
1:A:638:LEU:CD2	2:B:558:TYR:O	2.65	0.45
2:B:28:SER:N	2:B:32:GLU:CD	2.70	0.45
2:B:158:VAL:HG13	2:B:177:ILE:CG1	2.33	0.45
2:B:189:HIS:CD2	2:B:193:LEU:HG	2.52	0.45
2:B:219:TYR:HD1	2:B:226:LEU:HD13	1.65	0.45
2:B:306:LEU:HD12	2:B:325:LEU:HD23	1.98	0.45
2:B:328:LEU:O	2:B:329:ALA:C	2.47	0.45
2:B:520:SER:C	2:B:523:PHE:CD2	2.90	0.45
2:B:553:ALA:CA	2:B:614:ILE:CG2	2.94	0.45
2:B:592:TYR:OH	2:B:619:ASP:CG	2.55	0.45
3:M:243:ILE:HG21	3:M:298:ARG:HG2	1.98	0.45
3:M:374:TYR:CE1	3:M:390:ILE:HD13	2.51	0.45
1:A:97:SER:O	1:A:103:LYS:HE2	2.16	0.45
1:A:313:MET:HB2	1:A:348:PHE:HZ	1.80	0.45
1:A:373:GLU:CG	1:A:427:LYS:CE	2.90	0.45
2:B:103:LEU:CD2	3:M:131:ALA:C	2.85	0.45
2:B:527:PRO:CB	2:B:587:ARG:HG2	2.40	0.45
3:M:15:ILE:HG23	3:M:115:VAL:CG2	2.46	0.45
3:M:45:SER:HA	3:M:47:SER:O	2.17	0.45
3:M:242:GLY:O	3:M:302:TYR:N	2.47	0.45
3:M:353:VAL:O	3:M:401:LYS:CB	2.65	0.45
1:A:158:LEU:CG	1:A:162:ILE:CD1	2.92	0.45
1:A:244:LEU:CD2	1:A:277:LYS:HG3	2.47	0.45
1:A:244:LEU:HB2	1:A:256:LEU:HD13	1.98	0.45
1:A:495:ILE:CG2	1:A:515:CYS:HB3	2.46	0.45
1:A:556:VAL:HG21	1:A:603:VAL:HG13	1.96	0.45
2:B:74:ASP:O	2:B:77:ILE:CB	2.63	0.45

Continued on next page...

Continued from previous page...

Atom-1	Atom-2	Interatomic distance (Å)	Clash overlap (Å)
2:B:102:HIS:ND1	2:B:137:PHE:CB	2.79	0.45
2:B:143:SER:O	2:B:145:MET:N	2.50	0.45
2:B:403:ILE:O	2:B:403:ILE:HG13	2.16	0.45
2:B:526:CYS:HB2	2:B:555:LEU:HD11	1.97	0.45
2:B:559:ASP:O	2:B:562:ASN:CA	2.64	0.45
3:M:6:TYR:CD2	3:M:17:GLN:CG	3.00	0.45
4:S:7:ILE:HD13	4:S:16:LEU:HD23	1.99	0.45
4:S:55:PRO:O	4:S:58:LEU:HB2	2.14	0.45
1:A:114:PHE:CG	1:A:153:ILE:HG12	2.52	0.45
1:A:241:TYR:CE1	1:A:277:LYS:CB	2.99	0.45
1:A:289:SER:HB3	4:S:96:LEU:CB	2.28	0.45
1:A:421:PRO:HD2	1:A:424:TYR:CG	2.51	0.45
1:A:499:ILE:HD11	1:A:515:CYS:CB	2.46	0.45
1:A:605:GLU:CG	1:A:632:PHE:CG	3.00	0.45
2:B:2:VAL:CG1	2:B:6:HIS:CE1	3.00	0.45
2:B:60:ARG:HD2	2:B:96:LYS:HG2	1.99	0.45
2:B:139:LEU:HD23	2:B:172:GLU:O	2.17	0.45
2:B:246:SER:HA	2:B:249:ILE:HD12	1.98	0.45
2:B:292:GLU:CG	2:B:296:ASP:OD2	2.54	0.45
3:M:64:LYS:NZ	3:M:79:SER:HB2	2.32	0.45
3:M:103:TYR:O	3:M:104:PHE:CB	2.63	0.45
3:M:212:ASN:CG	3:M:250:LEU:HD23	2.37	0.45
1:A:258:LYS:HZ1	4:S:94:SER:HB3	1.31	0.45
1:A:595:GLU:CD	2:B:473:ASN:OD1	2.55	0.45
2:B:90:ILE:HD11	2:B:123:LEU:HD23	1.97	0.45
2:B:191:GLU:C	2:B:193:LEU:N	2.67	0.45
2:B:256:CYS:CB	2:B:328:LEU:HD23	2.46	0.45
2:B:256:CYS:O	2:B:258:GLN:N	2.50	0.45
2:B:266:VAL:HG13	2:B:291:TYR:H	1.82	0.45
2:B:472:VAL:HG11	2:B:510:GLY:C	2.34	0.45
3:M:100:LEU:HD21	3:M:121:ILE:HG23	1.99	0.45
3:M:128:CYS:O	3:M:130:GLU:O	2.35	0.45
3:M:331:LEU:HD12	3:M:331:LEU:C	2.36	0.45
3:M:360:LEU:HD21	3:M:362:PHE:CE2	2.49	0.45
3:M:455:VAL:HG12	3:M:455:VAL:O	2.16	0.45
4:S:35:VAL:O	4:S:39:ILE:HG12	2.16	0.45
4:S:54:PRO:O	4:S:69:ASN:HB2	2.17	0.45
1:A:226:SER:O	1:A:230:PRO:HG3	2.17	0.45
1:A:264:SER:HB3	1:A:271:ARG:HG3	1.92	0.45
1:A:413:SER:HB3	1:A:415:ARG:O	2.17	0.45
1:A:446:ASP:O	1:A:447:PHE:C	2.51	0.45

Continued on next page...

Continued from previous page...

Atom-1	Atom-2	Interatomic distance (Å)	Clash overlap (Å)
2:B:120:ILE:HG22	2:B:153:ILE:HG21	1.99	0.45
2:B:167:ALA:CA	2:B:202:ASP:CG	2.83	0.45
2:B:311:TYR:CB	3:M:269:ILE:HD13	2.47	0.45
2:B:430:ILE:HG13	2:B:467:VAL:HG22	1.99	0.45
2:B:483:PRO:HB3	2:B:521:ILE:HG13	1.97	0.45
2:B:494:ALA:CB	2:B:515:PHE:CZ	3.00	0.45
2:B:500:GLN:OE1	2:B:503:LEU:HD11	2.17	0.45
2:B:519:ALA:CB	2:B:555:LEU:CD1	2.92	0.45
2:B:527:PRO:HG2	2:B:587:ARG:HG2	1.99	0.45
2:B:544:THR:O	2:B:548:ILE:HG13	2.17	0.45
3:M:134:PRO:O	3:M:136:VAL:HA	2.17	0.45
3:M:256:VAL:CG2	3:M:452:ILE:HG22	2.47	0.45
3:M:293:PRO:C	3:M:294:ASP:O	2.39	0.45
3:M:296:LYS:O	3:M:296:LYS:CG	2.65	0.45
3:M:374:TYR:CE1	3:M:376:ILE:HG12	2.51	0.45
1:A:271:ARG:HH11	1:A:303:MET:HA	1.81	0.45
1:A:502:ASP:O	1:A:505:ASN:N	2.50	0.45
2:B:6:HIS:CB	3:M:25:PRO:C	2.85	0.45
2:B:24:ALA:CA	2:B:35:TYR:CE2	2.98	0.45
2:B:37:TYR:O	2:B:42:ILE:N	2.50	0.45
2:B:508:ARG:CB	2:B:544:THR:HG21	2.47	0.45
3:M:290:PHE:CZ	3:M:297:PHE:CE2	3.04	0.45
3:M:323:MET:CG	3:M:342:LEU:HG	2.46	0.45
3:M:435:LEU:HB2	3:M:437:TYR:CE1	2.52	0.45
1:A:121:LEU:CD1	1:A:155:THR:OG1	2.65	0.44
1:A:179:LYS:HB2	4:S:142:ILE:CD1	2.23	0.44
1:A:215:VAL:O	1:A:216:SER:O	2.35	0.44
2:B:25:VAL:HG13	2:B:33:SER:CA	2.32	0.44
2:B:142:LEU:O	2:B:143:SER:C	2.47	0.44
2:B:174:ALA:HB1	2:B:211:ALA:CA	2.33	0.44
2:B:279:LEU:HB3	2:B:280:PRO:HD2	1.98	0.44
2:B:291:TYR:CZ	2:B:293:VAL:CG1	3.00	0.44
2:B:515:PHE:CD2	2:B:529:VAL:HG21	2.51	0.44
4:S:98:ILE:HG22	4:S:102:ILE:HD11	1.98	0.44
1:A:71:VAL:CG2	1:A:94:VAL:HG11	2.47	0.44
1:A:85:ALA:HA	1:A:88:ASN:ND2	2.32	0.44
1:A:261:THR:O	1:A:264:SER:OG	2.23	0.44
1:A:338:PHE:HE2	1:A:352:PHE:CZ	2.36	0.44
1:A:548:GLN:CD	1:A:588:LEU:HD11	2.38	0.44
2:B:296:ASP:OD1	2:B:297:PRO:N	2.49	0.44
2:B:534:ILE:HG12	2:B:595:VAL:HG23	1.98	0.44

Continued on next page...

Continued from previous page...

Atom-1	Atom-2	Interatomic distance (Å)	Clash overlap (Å)
3:M:243:ILE:H	3:M:474:THR:HG21	1.81	0.44
3:M:276:VAL:HG22	3:M:299:LEU:HD12	2.00	0.44
3:M:316:ARG:O	3:M:318:ASN:N	2.51	0.44
4:S:53:THR:CG2	4:S:67:GLU:C	2.85	0.44
4:S:58:LEU:HD12	4:S:58:LEU:HA	1.81	0.44
2:B:2:VAL:HG21	3:M:58:ARG:HD2	1.99	0.44
2:B:120:ILE:CD1	2:B:142:LEU:CD2	2.95	0.44
2:B:162:VAL:C	2:B:164:ASP:N	2.70	0.44
2:B:512:VAL:HB	2:B:551:LEU:CD1	2.48	0.44
3:M:287:ASN:O	3:M:288:ILE:HG13	2.18	0.44
3:M:473:LYS:O	3:M:474:THR:HG22	2.17	0.44
4:S:4:ALA:CA	4:S:18:LYS:O	2.56	0.44
1:A:174:ARG:O	1:A:177:ILE:HB	2.18	0.44
1:A:441:TYR:HB3	1:A:444:VAL:CG2	2.47	0.44
1:A:563:CYS:CB	1:A:621:LEU:CD1	2.63	0.44
1:A:586:GLU:N	1:A:604:LEU:HD12	2.32	0.44
2:B:120:ILE:HG23	2:B:142:LEU:CD2	2.44	0.44
2:B:374:PHE:CZ	2:B:381:PHE:HD1	2.29	0.44
2:B:511:ILE:O	2:B:515:PHE:HD1	2.00	0.44
2:B:591:MET:O	2:B:595:VAL:HG23	2.17	0.44
2:B:592:TYR:OH	2:B:619:ASP:OD2	2.35	0.44
3:M:56:VAL:N	3:M:64:LYS:HG3	2.32	0.44
3:M:101:LEU:CD1	3:M:106:LYS:O	2.58	0.44
3:M:213:GLU:HB3	3:M:467:TYR:CA	2.47	0.44
3:M:304:VAL:HG21	3:M:309:GLN:NE2	2.32	0.44
4:S:16:LEU:CD2	4:S:128:LEU:CD2	2.93	0.44
4:S:17:VAL:HG22	4:S:19:PHE:CE2	2.51	0.44
1:A:136:GLY:O	1:A:139:ASP:CB	2.65	0.44
1:A:273:LYS:O	1:A:276:PRO:CD	2.60	0.44
2:B:10:SER:O	2:B:11:ALA:C	2.56	0.44
2:B:90:ILE:CD1	2:B:123:LEU:CD2	2.95	0.44
2:B:177:ILE:HG21	2:B:196:LEU:CG	2.47	0.44
2:B:208:ILE:O	2:B:212:VAL:HG23	2.17	0.44
2:B:334:MET:O	2:B:336:ASN:N	2.49	0.44
2:B:347:VAL:HG11	2:B:381:PHE:CE2	2.49	0.44
2:B:545:ARG:HH11	2:B:602:ASP:CG	2.21	0.44
3:M:218:LEU:O	3:M:441:GLY:HA2	2.18	0.44
4:S:135:ILE:O	4:S:140:MET:O	2.35	0.44
1:A:190:LEU:HD13	1:A:224:GLU:OE2	2.18	0.44
2:B:150:LEU:O	2:B:154:ILE:HG13	2.18	0.44
2:B:181:TYR:CB	2:B:218:CYS:SG	3.05	0.44

Continued on next page...

Continued from previous page...

Atom-1	Atom-2	Interatomic distance (Å)	Clash overlap (Å)
2:B:223:LEU:CD1	2:B:258:GLN:HB2	2.39	0.44
2:B:513:TRP:C	2:B:551:LEU:HD22	2.38	0.44
3:M:220:GLU:HB2	3:M:222:PHE:HE1	1.82	0.44
3:M:304:VAL:HG21	3:M:309:GLN:CD	2.38	0.44
3:M:419:ASN:ND2	3:M:424:PHE:CD2	2.85	0.44
1:A:114:PHE:CD1	1:A:114:PHE:C	2.90	0.44
1:A:313:MET:HG3	1:A:348:PHE:CE1	2.53	0.44
1:A:585:PHE:HE2	1:A:603:VAL:CG1	2.29	0.44
2:B:108:PHE:CE2	2:B:115:LEU:HD23	2.53	0.44
2:B:136:CYS:O	2:B:172:GLU:CB	2.64	0.44
2:B:143:SER:CA	2:B:179:LYS:HB2	2.47	0.44
2:B:171:GLY:HA2	2:B:207:VAL:O	2.17	0.44
2:B:212:VAL:HG13	2:B:248:LEU:HD23	1.99	0.44
2:B:260:LEU:CD2	2:B:293:VAL:HG11	2.46	0.44
2:B:278:PRO:HD3	2:B:290:SER:HA	1.98	0.44
2:B:464:SER:O	2:B:468:LEU:HG	2.17	0.44
2:B:512:VAL:HG21	2:B:548:ILE:CG1	2.48	0.44
4:S:5:VAL:HG13	4:S:87:PHE:CD2	2.52	0.44
4:S:53:THR:CG2	4:S:68:VAL:CA	2.94	0.44
4:S:159:ALA:O	4:S:162:SER:N	2.46	0.44
1:A:247:ILE:HG21	1:A:252:ILE:HB	2.00	0.44
1:A:397:ASP:O	1:A:418:ILE:HD12	2.18	0.44
1:A:572:PHE:O	1:A:575:LYS:HB3	2.17	0.44
2:B:261:PRO:HG2	2:B:292:GLU:HB2	1.99	0.44
2:B:365:PHE:O	2:B:368:ILE:HB	2.18	0.44
2:B:424:PHE:HA	2:B:425:PRO:HD3	1.58	0.44
3:M:121:ILE:HG23	3:M:125:PHE:HE1	1.83	0.44
3:M:219:LEU:CB	3:M:472:TYR:C	2.86	0.44
3:M:262:THR:CG2	3:M:265:ASN:N	2.80	0.44
1:A:529:GLY:C	1:A:562:TRP:CH2	2.91	0.44
2:B:2:VAL:HG12	2:B:6:HIS:NE2	2.32	0.44
2:B:136:CYS:CA	2:B:172:GLU:CG	2.94	0.44
2:B:219:TYR:O	2:B:223:LEU:CG	2.65	0.44
2:B:334:MET:O	2:B:336:ASN:O	2.36	0.44
2:B:404:ASN:O	2:B:405:GLU:O	2.32	0.44
3:M:100:LEU:C	3:M:103:TYR:O	2.56	0.44
3:M:222:PHE:C	3:M:479:PHE:CE2	2.78	0.44
3:M:223:HIS:CB	3:M:477:GLY:O	2.66	0.44
3:M:288:ILE:HD12	3:M:300:LEU:HD22	1.98	0.44
3:M:290:PHE:CE2	3:M:297:PHE:CE2	3.05	0.44
4:S:59:LEU:HD12	4:S:60:SER:O	2.18	0.44

Continued on next page...

Continued from previous page...

Atom-1	Atom-2	Interatomic distance (Å)	Clash overlap (Å)
4:S:112:CYS:HB2	4:S:113:PHE:HD1	1.80	0.44
4:S:131:VAL:CG2	4:S:153:VAL:CG2	2.89	0.44
1:A:101:GLN:CD	4:S:167:ILE:HD12	2.29	0.43
1:A:302:ASN:O	1:A:303:MET:C	2.55	0.43
1:A:395:PHE:CD2	1:A:428:MET:HE3	2.53	0.43
1:A:399:ASP:OD2	1:A:403:LEU:HD12	2.18	0.43
1:A:502:ASP:OD2	3:M:59:ASP:OD2	2.36	0.43
1:A:610:SER:OG	1:A:625:LEU:HD13	2.18	0.43
2:B:20:ARG:NH2	2:B:21:GLU:HG3	2.32	0.43
2:B:134:LEU:C	2:B:136:CYS:N	2.72	0.43
2:B:512:VAL:O	2:B:515:PHE:HB2	2.18	0.43
2:B:588:ILE:HG23	2:B:589:SER:N	2.33	0.43
3:M:19:LEU:CD2	3:M:24:ALA:CB	2.95	0.43
3:M:65:TYR:CZ	3:M:66:PHE:O	2.71	0.43
3:M:224:VAL:CG2	3:M:306:LEU:CD1	2.95	0.43
3:M:401:LYS:O	3:M:402:SER:C	2.54	0.43
1:A:170:LEU:CD2	1:A:181:ALA:HB1	2.47	0.43
1:A:394:GLN:O	1:A:397:ASP:N	2.52	0.43
1:A:464:ILE:O	1:A:464:ILE:HG12	2.18	0.43
1:A:480:LEU:HD13	1:A:480:LEU:O	2.18	0.43
1:A:594:PHE:HB3	2:B:476:ARG:HH21	1.83	0.43
1:A:630:PRO:HG2	2:B:617:LEU:CG	2.42	0.43
2:B:136:CYS:HA	2:B:172:GLU:CB	2.47	0.43
2:B:493:LEU:CG	2:B:511:ILE:HG23	2.40	0.43
2:B:596:LEU:HD13	2:B:611:ALA:C	2.38	0.43
3:M:16:PHE:CB	3:M:118:TYR:CD1	3.01	0.43
3:M:270:PRO:N	3:M:302:TYR:CD1	2.85	0.43
3:M:290:PHE:HZ	3:M:293:PRO:HD3	1.75	0.43
3:M:317:MET:HB2	3:M:322:LEU:HB2	2.01	0.43
1:A:72:LEU:O	1:A:76:TYR:CD1	2.72	0.43
1:A:79:MET:O	4:S:25:LEU:CD1	2.66	0.43
1:A:244:LEU:HD22	1:A:260:PHE:HE1	1.83	0.43
1:A:261:THR:O	1:A:264:SER:N	2.50	0.43
1:A:460:LEU:O	1:A:463:ASP:CB	2.65	0.43
1:A:566:PHE:O	1:A:568:GLU:N	2.51	0.43
1:A:595:GLU:CD	2:B:476:ARG:NH2	2.71	0.43
2:B:109:ALA:HA	2:B:116:THR:OG1	2.17	0.43
2:B:178:ILE:CG1	2:B:214:ALA:O	2.58	0.43
2:B:223:LEU:HD13	2:B:259:TYR:CA	2.41	0.43
2:B:234:CYS:HB2	2:B:301:LEU:CB	2.48	0.43
2:B:253:ILE:CG1	2:B:324:ALA:CB	2.96	0.43

Continued on next page...

Continued from previous page...

Atom-1	Atom-2	Interatomic distance (Å)	Clash overlap (Å)
2:B:430:ILE:HG12	2:B:467:VAL:HA	2.01	0.43
2:B:487:LEU:HD22	2:B:522:GLU:CB	2.42	0.43
3:M:66:PHE:CD2	3:M:77:LEU:HD11	2.53	0.43
3:M:215:TYR:HD2	3:M:470:ALA:N	2.16	0.43
3:M:374:TYR:CD1	3:M:376:ILE:HD11	2.52	0.43
1:A:194:GLU:O	1:A:197:ARG:N	2.48	0.43
1:A:282:MET:O	1:A:284:SER:N	2.52	0.43
1:A:402:ILE:HG21	1:A:421:PRO:HA	1.99	0.43
1:A:447:PHE:O	1:A:450:TYR:HB3	2.18	0.43
1:A:495:ILE:HG21	1:A:515:CYS:HB3	1.99	0.43
2:B:20:ARG:HD2	2:B:21:GLU:N	2.33	0.43
2:B:38:TYR:C	2:B:42:ILE:H	2.19	0.43
2:B:174:ALA:HB3	2:B:211:ALA:CA	2.44	0.43
2:B:398:ILE:HG22	2:B:402:LEU:CD1	2.48	0.43
2:B:554:LYS:O	2:B:557:SER:N	2.51	0.43
3:M:63:TYR:CG	3:M:64:LYS:N	2.85	0.43
3:M:374:TYR:CE1	3:M:390:ILE:CD1	3.01	0.43
1:A:153:ILE:HB	1:A:158:LEU:HD23	2.01	0.43
1:A:157:SER:O	1:A:160:ARG:HB2	2.18	0.43
1:A:350:SER:O	1:A:352:PHE:N	2.51	0.43
1:A:432:ILE:O	1:A:435:ILE:HB	2.18	0.43
2:B:16:LYS:HA	2:B:17:VAL:HA	1.83	0.43
2:B:34:SER:O	2:B:37:TYR:HB3	2.18	0.43
2:B:281:ASP:OD1	2:B:287:GLU:CG	2.66	0.43
2:B:534:ILE:C	2:B:536:ASN:N	2.71	0.43
3:M:51:LEU:CB	3:M:68:VAL:HG21	2.48	0.43
3:M:217:ASP:CB	3:M:470:ALA:C	2.87	0.43
3:M:219:LEU:HD22	3:M:473:LYS:C	2.39	0.43
3:M:243:ILE:CD1	3:M:301:GLU:HB3	2.47	0.43
3:M:350:VAL:HA	3:M:442:GLN:CG	2.48	0.43
4:S:1:MET:H1	4:S:93:GLU:CD	2.11	0.43
4:S:14:PRO:CA	4:S:36:TYR:OH	2.65	0.43
1:A:185:LEU:HB2	1:A:203:PHE:CE1	2.51	0.43
1:A:241:TYR:CE1	1:A:277:LYS:HB2	2.54	0.43
1:A:300:LYS:O	1:A:301:GLY:C	2.55	0.43
1:A:335:CYS:O	1:A:338:PHE:HB2	2.19	0.43
1:A:384:LEU:HB2	1:A:441:TYR:HE2	1.83	0.43
2:B:77:ILE:O	2:B:78:ASP:C	2.55	0.43
2:B:77:ILE:CG2	2:B:82:TYR:HE1	2.26	0.43
2:B:90:ILE:HG13	2:B:98:LYS:HD3	2.01	0.43
2:B:105:LEU:O	2:B:106:LEU:O	2.32	0.43

Continued on next page...

Continued from previous page...

Atom-1	Atom-2	Interatomic distance (Å)	Clash overlap (Å)
2:B:353:GLN:O	2:B:354:GLY:C	2.56	0.43
2:B:418:TYR:OH	2:B:428:VAL:HG12	2.19	0.43
2:B:522:GLU:O	2:B:522:GLU:CG	2.40	0.43
2:B:549:LEU:CD1	2:B:614:ILE:HD12	2.49	0.43
3:M:410:VAL:HG12	3:M:412:ARG:HG3	2.01	0.43
4:S:25:LEU:O	4:S:28:GLN:HG3	2.18	0.43
4:S:130:SER:OG	4:S:156:LEU:HD13	2.12	0.43
1:A:295:VAL:HG12	1:A:337:LEU:HD22	2.01	0.43
1:A:405:THR:N	2:B:7:ARG:NE	2.49	0.43
2:B:36:THR:O	2:B:40:GLN:CB	2.66	0.43
2:B:237:ILE:HG12	2:B:309:LEU:HD21	2.01	0.43
2:B:337:THR:OG1	2:B:373:LEU:CD1	2.66	0.43
2:B:475:ILE:CG2	2:B:514:LEU:CD2	2.97	0.43
2:B:560:ILE:HA	2:B:564:LYS:H	1.82	0.43
3:M:45:SER:HA	3:M:47:SER:H	1.83	0.43
3:M:221:THR:HG22	3:M:223:HIS:CE1	2.54	0.43
3:M:290:PHE:CB	3:M:299:LEU:CD1	2.95	0.43
4:S:16:LEU:CD1	4:S:125:TRP:NE1	2.02	0.43
4:S:53:THR:CB	4:S:68:VAL:CA	2.96	0.43
1:A:256:LEU:O	1:A:260:PHE:CD1	2.71	0.43
1:A:545:HIS:O	1:A:546:SER:O	2.37	0.43
1:A:581:LEU:CG	1:A:607:LEU:CD1	2.97	0.43
2:B:63:MET:HE1	2:B:104:TYR:CG	2.54	0.43
2:B:291:TYR:HD2	2:B:294:VAL:HG12	1.84	0.43
2:B:292:GLU:CG	2:B:296:ASP:CB	2.96	0.43
2:B:334:MET:HE2	2:B:339:PHE:CE1	2.54	0.43
2:B:343:LEU:HD23	2:B:366:LEU:HD12	2.00	0.43
2:B:389:ILE:HG12	2:B:425:PRO:HG2	2.01	0.43
2:B:418:TYR:CZ	2:B:432:ALA:HB2	2.53	0.43
2:B:564:LYS:HG3	2:B:568:VAL:HG23	2.01	0.43
2:B:585:GLY:O	2:B:589:SER:CB	2.66	0.43
2:B:592:TYR:CE2	2:B:618:PHE:HD2	2.32	0.43
3:M:20:LEU:C	3:M:21:GLY:O	2.50	0.43
3:M:271:SER:O	3:M:300:LEU:HA	2.19	0.43
3:M:278:ILE:O	3:M:278:ILE:HG12	2.18	0.43
1:A:78:GLU:C	1:A:80:TYR:O	2.57	0.43
1:A:251:TRP:CH2	4:S:96:LEU:O	2.72	0.43
2:B:50:LEU:CD2	2:B:62:ALA:HB2	2.49	0.43
2:B:127:LEU:CD2	2:B:157:THR:HG21	2.48	0.43
2:B:148:SER:CB	2:B:183:ALA:HB1	2.48	0.43
2:B:354:GLY:O	2:B:358:MET:CG	2.46	0.43

Continued on next page...

Continued from previous page...

Atom-1	Atom-2	Interatomic distance (Å)	Clash overlap (Å)
2:B:461:HIS:HB3	2:B:463:LEU:HD23	2.01	0.43
3:M:63:TYR:N	3:M:81:SER:O	2.52	0.43
3:M:100:LEU:HB3	3:M:109:LEU:HD22	2.01	0.43
3:M:220:GLU:HA	3:M:474:THR:HG21	2.01	0.43
3:M:246:VAL:HB	3:M:297:PHE:CE1	2.49	0.43
3:M:253:ASN:OD1	3:M:292:PRO:HB2	2.19	0.43
3:M:324:SER:O	3:M:340:LEU:HA	2.19	0.43
3:M:343:ASN:N	3:M:343:ASN:ND2	2.56	0.43
3:M:364:VAL:HG12	3:M:372:ILE:CB	2.49	0.43
3:M:407:THR:O	3:M:409:PRO:HD3	2.19	0.43
1:A:239:LEU:O	1:A:242:GLU:C	2.51	0.43
1:A:270:LEU:CD1	1:A:274:LEU:HD23	2.49	0.43
2:B:25:VAL:CG2	2:B:35:TYR:CB	2.96	0.43
2:B:181:TYR:CD1	2:B:222:HIS:HD2	2.32	0.43
2:B:196:LEU:O	2:B:199:LEU:HB2	2.19	0.43
2:B:518:ILE:O	2:B:518:ILE:CG1	2.67	0.43
2:B:542:PRO:O	2:B:545:ARG:HB2	2.19	0.43
3:M:96:ILE:CD1	3:M:125:PHE:HA	2.48	0.43
3:M:317:MET:CB	3:M:322:LEU:H	2.31	0.43
3:M:323:MET:CE	3:M:437:TYR:HE2	2.32	0.43
3:M:327:PHE:CD2	3:M:430:LEU:HD22	2.54	0.43
3:M:347:PHE:O	3:M:349:LYS:N	2.52	0.43
2:B:120:ILE:HD13	2:B:142:LEU:HD23	2.00	0.42
2:B:296:ASP:C	2:B:298:ASP:N	2.71	0.42
2:B:310:ILE:HD11	2:B:321:CYS:CB	2.49	0.42
2:B:345:ARG:HH12	3:M:305:ASP:CB	2.31	0.42
2:B:398:ILE:HG22	2:B:402:LEU:HD11	2.01	0.42
3:M:6:TYR:HA	3:M:17:GLN:HA	2.00	0.42
3:M:241:HIS:HB2	3:M:476:THR:HG22	1.95	0.42
3:M:258:VAL:CG1	3:M:449:VAL:HG22	2.49	0.42
3:M:280:ASP:OD1	3:M:282:VAL:HG23	2.19	0.42
4:S:53:THR:HG21	4:S:69:ASN:N	2.34	0.42
1:A:274:LEU:O	1:A:275:LEU:O	2.38	0.42
2:B:56:SER:O	2:B:57:ARG:C	2.43	0.42
2:B:367:SER:CB	2:B:401:THR:HB	2.48	0.42
2:B:426:GLU:O	2:B:429:VAL:HB	2.20	0.42
2:B:512:VAL:CB	2:B:551:LEU:HD12	2.47	0.42
3:M:293:PRO:CA	3:M:294:ASP:O	2.66	0.42
3:M:374:TYR:CE2	3:M:393:GLY:HA2	2.44	0.42
4:S:53:THR:CG2	4:S:69:ASN:HB2	2.49	0.42
4:S:57:LEU:N	4:S:58:LEU:O	2.46	0.42

Continued on next page...

Continued from previous page...

Atom-1	Atom-2	Interatomic distance (Å)	Clash overlap (Å)
4:S:89:VAL:CG1	4:S:98:ILE:CD1	2.97	0.42
1:A:92:LEU:HD21	1:A:124:ALA:CA	2.49	0.42
1:A:154:ILE:HG22	1:A:191:GLN:CG	2.45	0.42
1:A:189:PHE:CD2	1:A:225:LEU:HD21	2.55	0.42
1:A:250:ASN:OD1	1:A:285:THR:CG2	2.68	0.42
1:A:251:TRP:HA	1:A:254:ILE:HD12	2.02	0.42
1:A:427:LYS:O	1:A:431:VAL:HG23	2.18	0.42
2:B:13:ASP:O	2:B:16:LYS:O	2.38	0.42
2:B:123:LEU:CD1	2:B:142:LEU:HD23	2.35	0.42
2:B:340:ILE:CB	2:B:373:LEU:HG	2.49	0.42
2:B:537:PHE:CZ	2:B:599:ALA:HA	2.54	0.42
3:M:368:ASP:O	3:M:371:GLU:HB2	2.19	0.42
4:S:89:VAL:HG11	4:S:98:ILE:CG2	2.50	0.42
1:A:186:PHE:HD1	1:A:221:VAL:HA	1.84	0.42
1:A:204:VAL:O	1:A:206:LYS:N	2.51	0.42
1:A:275:LEU:CD1	1:A:308:ASP:CG	2.88	0.42
1:A:356:ILE:CD1	1:A:374:LEU:HD23	2.49	0.42
1:A:366:SER:O	1:A:370:LYS:CG	2.56	0.42
1:A:634:ASN:ND2	2:B:554:LYS:O	2.52	0.42
1:A:634:ASN:HB3	2:B:558:TYR:CD1	2.53	0.42
2:B:158:VAL:HG12	2:B:177:ILE:HD11	2.00	0.42
2:B:219:TYR:CD2	2:B:222:HIS:O	2.72	0.42
2:B:256:CYS:CB	2:B:328:LEU:HD21	2.43	0.42
2:B:434:LYS:O	2:B:437:SER:HB3	2.19	0.42
3:M:117:ASN:O	3:M:120:ARG:N	2.52	0.42
3:M:226:PHE:CD2	3:M:235:LEU:HA	2.55	0.42
3:M:288:ILE:HD12	3:M:300:LEU:HD23	2.02	0.42
3:M:419:ASN:CG	3:M:424:PHE:CD2	2.93	0.42
4:S:55:PRO:CG	4:S:71:GLU:HG2	2.49	0.42
1:A:121:LEU:HD13	1:A:155:THR:CB	2.49	0.42
1:A:186:PHE:HB2	1:A:221:VAL:HG22	2.01	0.42
1:A:192:TYR:CD2	1:A:192:TYR:O	2.72	0.42
1:A:260:PHE:O	1:A:261:THR:O	2.29	0.42
1:A:372:ILE:O	1:A:375:VAL:HB	2.20	0.42
1:A:403:LEU:CD2	1:A:422:GLU:HG3	2.34	0.42
1:A:536:MET:HB3	1:A:555:LEU:HD21	2.01	0.42
1:A:554:ALA:O	1:A:557:LYS:N	2.49	0.42
2:B:527:PRO:CG	2:B:587:ARG:HG2	2.50	0.42
3:M:57:GLY:O	3:M:58:ARG:HG3	2.20	0.42
3:M:114:ILE:O	3:M:115:VAL:C	2.57	0.42
1:A:83:ASP:OD2	1:A:85:ALA:N	2.52	0.42

Continued on next page...

Continued from previous page...

Atom-1	Atom-2	Interatomic distance (Å)	Clash overlap (Å)
1:A:148:SER:O	1:A:151:SER:OG	2.25	0.42
2:B:101:ILE:O	2:B:105:LEU:HD13	2.20	0.42
2:B:102:HIS:CB	2:B:141:ALA:HB2	2.50	0.42
2:B:231:ARG:C	2:B:233:TYR:H	2.22	0.42
2:B:279:LEU:CD1	2:B:285:GLU:HG2	2.41	0.42
2:B:343:LEU:HD22	2:B:366:LEU:HD11	1.99	0.42
2:B:534:ILE:HD11	2:B:591:MET:C	2.34	0.42
3:M:347:PHE:O	3:M:350:VAL:N	2.49	0.42
4:S:152:SER:O	4:S:155:GLU:HB2	2.20	0.42
1:A:84:MET:HE3	1:A:113:SER:O	2.19	0.42
1:A:250:ASN:O	1:A:254:ILE:HG13	2.19	0.42
1:A:537:THR:O	1:A:540:ILE:HG22	2.20	0.42
1:A:556:VAL:HG21	1:A:603:VAL:CG2	2.49	0.42
1:A:582:ILE:CD1	1:A:608:ARG:HA	2.50	0.42
1:A:589:SER:O	1:A:597:GLN:CD	2.57	0.42
2:B:18:ILE:HD13	2:B:18:ILE:N	2.32	0.42
2:B:63:MET:SD	2:B:101:ILE:HA	2.59	0.42
2:B:106:LEU:CG	2:B:144:ASP:HB3	2.49	0.42
2:B:162:VAL:CG2	2:B:199:LEU:HG	2.45	0.42
2:B:302:PHE:HE1	2:B:306:LEU:HD21	1.84	0.42
2:B:304:GLN:O	2:B:307:ASN:HB2	2.20	0.42
2:B:367:SER:CB	2:B:401:THR:CB	2.98	0.42
2:B:538:SER:HB3	2:B:598:LEU:CD2	2.50	0.42
2:B:566:ALA:HB1	2:B:581:TYR:HB3	2.00	0.42
3:M:223:HIS:HB2	3:M:476:THR:OG1	2.19	0.42
3:M:290:PHE:CZ	3:M:297:PHE:CG	3.08	0.42
3:M:473:LYS:C	3:M:474:THR:CG2	2.87	0.42
1:A:151:SER:C	1:A:153:ILE:N	2.68	0.42
1:A:154:ILE:HB	1:A:191:GLN:CG	2.31	0.42
1:A:224:GLU:O	1:A:227:LYS:N	2.48	0.42
1:A:589:SER:HB2	1:A:601:VAL:HG22	2.02	0.42
1:A:606:PHE:CD1	1:A:629:LEU:HG	2.54	0.42
1:A:637:GLU:O	2:B:517:GLU:HA	2.19	0.42
2:B:178:ILE:HD11	2:B:215:TYR:N	2.35	0.42
2:B:227:HIS:O	2:B:230:PHE:N	2.51	0.42
2:B:345:ARG:O	2:B:349:MET:HG3	2.20	0.42
2:B:374:PHE:HA	2:B:377:TYR:HD1	1.85	0.42
2:B:400:SER:CB	2:B:439:CYS:SG	3.08	0.42
2:B:494:ALA:HB2	2:B:515:PHE:HE2	1.79	0.42
3:M:19:LEU:HD11	3:M:24:ALA:HB2	2.01	0.42
3:M:65:TYR:CE1	3:M:86:PRO:HB3	2.50	0.42

Continued on next page...

Continued from previous page...

Atom-1	Atom-2	Interatomic distance (Å)	Clash overlap (Å)
3:M:111:ILE:CG1	3:M:112:LYS:N	2.82	0.42
3:M:223:HIS:CD2	3:M:478:ASN:CB	3.03	0.42
3:M:245:ASP:O	3:M:472:TYR:HE1	1.90	0.42
4:S:43:ASN:O	4:S:45:ASP:N	2.51	0.42
1:A:153:ILE:CG2	1:A:158:LEU:CD2	2.97	0.42
1:A:298:ILE:HG13	1:A:311:THR:HG22	2.01	0.42
1:A:359:LEU:HB3	1:A:367:ILE:CG2	2.49	0.42
1:A:369:SER:CB	1:A:424:TYR:HE2	2.21	0.42
1:A:404:GLN:C	2:B:7:ARG:NE	2.73	0.42
1:A:555:LEU:HD12	1:A:581:LEU:HD11	2.00	0.42
1:A:566:PHE:CD1	1:A:570:LYS:HA	2.55	0.42
1:A:605:GLU:CD	1:A:636:TYR:OH	2.57	0.42
2:B:98:LYS:HD2	2:B:102:HIS:NE2	2.35	0.42
2:B:215:TYR:CE2	2:B:229:HIS:ND1	2.86	0.42
2:B:230:PHE:HD2	2:B:298:ASP:CA	2.32	0.42
2:B:493:LEU:HD11	2:B:511:ILE:HG12	2.01	0.42
2:B:533:LEU:O	2:B:536:ASN:N	2.46	0.42
3:M:54:SER:H	3:M:66:PHE:HD1	1.67	0.42
3:M:222:PHE:HB2	3:M:479:PHE:CE1	2.54	0.42
3:M:364:VAL:HG12	3:M:372:ILE:HG13	2.01	0.42
3:M:445:SER:HG	3:M:447:ILE:HG23	1.83	0.42
4:S:135:ILE:CG2	4:S:141:VAL:HG22	2.44	0.42
1:A:121:LEU:CD1	1:A:155:THR:H	2.26	0.42
1:A:395:PHE:CE2	1:A:428:MET:HE3	2.54	0.42
1:A:584:PHE:O	1:A:587:ASN:N	2.53	0.42
2:B:9:ALA:O	2:B:10:SER:C	2.58	0.42
2:B:50:LEU:HD23	2:B:62:ALA:CA	2.50	0.42
2:B:82:TYR:HB2	2:B:104:TYR:OH	2.19	0.42
2:B:94:ASP:OD1	2:B:95:THR:N	2.53	0.42
2:B:127:LEU:CD1	2:B:157:THR:OG1	2.68	0.42
2:B:181:TYR:C	2:B:183:ALA:H	2.20	0.42
2:B:408:VAL:HG11	2:B:446:TRP:CG	2.55	0.42
3:M:217:ASP:HB3	3:M:470:ALA:C	2.41	0.42
3:M:323:MET:HG2	3:M:437:TYR:OH	2.20	0.42
3:M:443:SER:CB	3:M:447:ILE:C	2.88	0.42
4:S:58:LEU:HD12	4:S:68:VAL:HG13	2.02	0.42
1:A:390:THR:O	1:A:394:GLN:HG2	2.20	0.41
1:A:456:ASP:O	1:A:459:MET:N	2.49	0.41
1:A:570:LYS:HB3	1:A:618:THR:HG22	2.02	0.41
2:B:104:TYR:O	2:B:107:ARG:CB	2.65	0.41
2:B:159:LYS:HA	2:B:195:ILE:HD13	2.02	0.41

Continued on next page...

Continued from previous page...

Atom-1	Atom-2	Interatomic distance (Å)	Clash overlap (Å)
2:B:234:CYS:CB	2:B:301:LEU:HB2	2.50	0.41
2:B:340:ILE:CG1	2:B:373:LEU:CG	2.97	0.41
2:B:435:SER:C	2:B:437:SER:N	2.73	0.41
2:B:513:TRP:HE1	2:B:517:GLU:CG	2.33	0.41
2:B:563:PHE:CE2	2:B:584:SER:CA	3.03	0.41
3:M:64:LYS:CE	3:M:79:SER:HB2	2.50	0.41
3:M:262:THR:HG22	3:M:264:GLY:H	1.81	0.41
3:M:263:MET:HG2	3:M:263:MET:O	2.20	0.41
4:S:7:ILE:HG23	4:S:85:PHE:CD2	2.54	0.41
4:S:55:PRO:HB3	4:S:71:GLU:HG2	2.02	0.41
4:S:58:LEU:HD22	4:S:70:ASN:HA	2.02	0.41
4:S:130:SER:HB2	4:S:156:LEU:CD1	2.50	0.41
1:A:223:CYS:HB2	1:A:259:LEU:CG	2.50	0.41
1:A:316:LEU:O	1:A:319:LEU:N	2.53	0.41
1:A:438:ALA:HB3	1:A:441:TYR:HE1	1.86	0.41
1:A:528:ASN:C	1:A:530:ASN:N	2.71	0.41
2:B:117:LEU:HD23	2:B:150:LEU:HD23	2.02	0.41
2:B:137:PHE:N	2:B:172:GLU:HG3	2.31	0.41
2:B:170:ARG:O	2:B:171:GLY:C	2.55	0.41
2:B:191:GLU:C	2:B:193:LEU:H	2.24	0.41
2:B:329:ALA:HB2	2:B:334:MET:HG2	2.02	0.41
2:B:451:MET:HG3	2:B:489:ILE:HD13	2.01	0.41
2:B:486:HIS:CD2	2:B:490:ILE:HG12	2.55	0.41
2:B:490:ILE:CG1	2:B:518:ILE:HG12	2.47	0.41
2:B:534:ILE:CD1	2:B:591:MET:C	2.77	0.41
3:M:15:ILE:HG21	3:M:114:ILE:CG2	2.50	0.41
3:M:104:PHE:CD2	3:M:109:LEU:HD21	2.56	0.41
3:M:432:THR:O	3:M:432:THR:HG23	2.19	0.41
1:A:322:PHE:CB	1:A:330:LEU:HD21	2.49	0.41
1:A:514:GLU:HA	1:A:514:GLU:OE1	2.20	0.41
1:A:548:GLN:NE2	1:A:588:LEU:HD11	2.36	0.41
2:B:95:THR:HG22	2:B:137:PHE:HE2	1.84	0.41
2:B:117:LEU:O	2:B:120:ILE:HB	2.20	0.41
2:B:234:CYS:HB2	2:B:301:LEU:HB3	2.02	0.41
2:B:396:ILE:HD13	2:B:432:ALA:HB2	2.02	0.41
3:M:6:TYR:CA	3:M:16:PHE:O	2.63	0.41
3:M:65:TYR:CE2	3:M:86:PRO:HA	2.55	0.41
3:M:72:LEU:O	3:M:109:LEU:O	2.38	0.41
3:M:221:THR:HG21	3:M:475:GLN:HA	2.02	0.41
3:M:374:TYR:HE1	3:M:376:ILE:HG12	1.85	0.41
3:M:405:THR:CG2	3:M:406:GLY:N	2.77	0.41

Continued on next page...

Continued from previous page...

Atom-1	Atom-2	Interatomic distance (Å)	Clash overlap (Å)
3:M:443:SER:CA	3:M:447:ILE:HG13	2.50	0.41
4:S:87:PHE:CD1	4:S:87:PHE:N	2.89	0.41
4:S:135:ILE:HG23	4:S:141:VAL:CG1	2.49	0.41
1:A:166:LEU:CD2	1:A:184:ALA:HB3	2.50	0.41
1:A:408:ILE:HG21	4:S:64:ASN:O	2.19	0.41
1:A:609:LEU:HD21	1:A:628:VAL:CG2	2.37	0.41
2:B:108:PHE:HE2	2:B:112:ASP:CB	2.32	0.41
2:B:277:CYS:HB2	2:B:296:ASP:H	1.84	0.41
2:B:421:SER:C	2:B:422:ALA:O	2.50	0.41
2:B:464:SER:OG	2:B:467:VAL:HG23	2.20	0.41
2:B:508:ARG:O	2:B:512:VAL:CG2	2.54	0.41
2:B:513:TRP:NE1	2:B:517:GLU:HG3	2.35	0.41
2:B:569:THR:HA	2:B:571:SER:H	1.85	0.41
3:M:19:LEU:HD21	3:M:24:ALA:HB3	2.02	0.41
3:M:221:THR:HG23	3:M:437:TYR:O	2.20	0.41
3:M:304:VAL:HG11	3:M:445:SER:HA	2.02	0.41
4:S:64:ASN:O	4:S:66:ASP:N	2.53	0.41
4:S:87:PHE:CE2	4:S:102:ILE:HG12	2.56	0.41
1:A:275:LEU:HD11	1:A:311:THR:OG1	2.21	0.41
2:B:124:GLN:CA	2:B:127:LEU:HD12	2.36	0.41
2:B:136:CYS:CA	2:B:172:GLU:CB	2.99	0.41
2:B:276:SER:OG	2:B:289:PRO:CD	2.69	0.41
2:B:293:VAL:HA	2:B:299:LEU:CB	2.48	0.41
2:B:296:ASP:O	2:B:298:ASP:N	2.53	0.41
2:B:534:ILE:HG23	2:B:598:LEU:HD12	2.02	0.41
2:B:568:VAL:C	2:B:571:SER:OG	2.59	0.41
3:M:18:TYR:CD1	3:M:19:LEU:N	2.89	0.41
4:S:161:GLU:O	4:S:162:SER:C	2.56	0.41
1:A:79:MET:HG2	1:A:112:GLN:OE1	2.21	0.41
1:A:222:ILE:HG21	1:A:240:LEU:CD1	2.30	0.41
1:A:244:LEU:CD1	1:A:281:LEU:HD13	2.34	0.41
1:A:450:TYR:O	1:A:454:ILE:HG12	2.21	0.41
1:A:637:GLU:HG2	2:B:554:LYS:HB3	1.95	0.41
2:B:74:ASP:O	2:B:77:ILE:HG12	2.21	0.41
2:B:246:SER:OG	2:B:317:VAL:HG22	2.20	0.41
2:B:296:ASP:C	2:B:298:ASP:H	2.22	0.41
2:B:374:PHE:CG	2:B:374:PHE:O	2.74	0.41
2:B:433:VAL:CG2	2:B:471:TYR:CZ	3.03	0.41
2:B:436:LEU:HD12	2:B:454:LEU:HD21	2.02	0.41
2:B:458:MET:C	2:B:460:SER:N	2.73	0.41
3:M:7:ILE:HD11	3:M:121:ILE:HG21	2.02	0.41

Continued on next page...

Continued from previous page...

Atom-1	Atom-2	Interatomic distance (Å)	Clash overlap (Å)
3:M:442:GLN:CG	3:M:443:SER:N	2.82	0.41
1:A:231:GLN:N	1:A:232:PRO:CD	2.84	0.41
1:A:330:LEU:O	1:A:330:LEU:HD12	2.21	0.41
1:A:356:ILE:O	1:A:359:LEU:N	2.52	0.41
1:A:401:VAL:O	1:A:404:GLN:N	2.38	0.41
1:A:464:ILE:O	1:A:464:ILE:HG23	2.20	0.41
1:A:564:ASN:OD1	1:A:622:PRO:HB3	2.20	0.41
1:A:582:ILE:HD11	1:A:608:ARG:HA	2.02	0.41
2:B:100:LEU:O	2:B:103:LEU:HB2	2.21	0.41
2:B:267:ASP:N	2:B:289:PRO:HB3	2.33	0.41
2:B:387:ASP:CB	2:B:388:PRO:HD2	2.50	0.41
2:B:505:ASP:CA	2:B:544:THR:OG1	2.63	0.41
3:M:45:SER:HB3	3:M:51:LEU:HD12	2.01	0.41
3:M:117:ASN:O	3:M:120:ARG:HB2	2.21	0.41
3:M:240:ILE:HB	3:M:304:VAL:CG1	2.51	0.41
3:M:270:PRO:N	3:M:302:TYR:CE1	2.89	0.41
1:A:63:ASP:C	1:A:63:ASP:OD1	2.58	0.41
1:A:219:VAL:O	1:A:259:LEU:CD1	2.69	0.41
1:A:244:LEU:O	1:A:246:THR:N	2.53	0.41
1:A:279:LEU:HA	1:A:282:MET:HG2	2.03	0.41
1:A:289:SER:CB	4:S:96:LEU:HB3	2.51	0.41
1:A:426:ILE:CD1	1:A:466:ASP:OD2	2.67	0.41
2:B:102:HIS:CD2	2:B:123:LEU:HD21	2.56	0.41
2:B:175:LEU:HA	2:B:214:ALA:HB2	2.02	0.41
2:B:256:CYS:C	2:B:258:GLN:N	2.73	0.41
2:B:346:THR:HG22	2:B:350:THR:HG23	2.02	0.41
2:B:422:ALA:HB2	2:B:424:PHE:CE1	2.44	0.41
2:B:433:VAL:HG21	2:B:471:TYR:CD1	2.56	0.41
2:B:436:LEU:O	2:B:450:VAL:HG11	2.21	0.41
3:M:222:PHE:HB2	3:M:479:PHE:HZ	1.85	0.41
3:M:245:ASP:C	3:M:246:VAL:HG23	2.40	0.41
1:A:64:LEU:HA	1:A:102:GLN:NE2	2.33	0.41
1:A:401:VAL:HG21	1:A:419:ILE:HB	2.03	0.41
1:A:480:LEU:C	1:A:480:LEU:CD1	2.88	0.41
1:A:581:LEU:CD1	1:A:585:PHE:CZ	3.03	0.41
1:A:581:LEU:CG	1:A:585:PHE:CE2	3.02	0.41
1:A:595:GLU:OE2	2:B:473:ASN:OD1	2.39	0.41
2:B:28:SER:OG	2:B:31:GLY:O	2.35	0.41
2:B:42:ILE:O	2:B:46:GLN:CG	2.68	0.41
2:B:63:MET:HE1	2:B:104:TYR:CB	2.50	0.41
2:B:102:HIS:CE1	2:B:137:PHE:CB	3.04	0.41

Continued on next page...

Continued from previous page...

Atom-1	Atom-2	Interatomic distance (Å)	Clash overlap (Å)
2:B:142:LEU:HB3	2:B:154:ILE:HG12	2.02	0.41
2:B:162:VAL:HG11	2:B:195:ILE:HG23	2.02	0.41
2:B:178:ILE:CD1	2:B:214:ALA:O	2.68	0.41
2:B:178:ILE:HG21	2:B:217:GLU:HB2	1.82	0.41
2:B:271:GLU:O	2:B:272:GLY:C	2.52	0.41
2:B:277:CYS:HB2	2:B:295:ASN:HB2	2.02	0.41
2:B:278:PRO:CD	2:B:292:GLU:HG3	2.42	0.41
2:B:279:LEU:HD12	2:B:285:GLU:CB	2.51	0.41
2:B:355:ASN:O	2:B:359:LEU:CD2	2.69	0.41
2:B:408:VAL:HB	2:B:446:TRP:CD1	2.55	0.41
2:B:419:VAL:O	2:B:420:ALA:C	2.58	0.41
2:B:549:LEU:CG	2:B:611:ALA:HA	2.50	0.41
2:B:556:LEU:HD11	2:B:592:TYR:HB2	2.02	0.41
2:B:569:THR:C	2:B:571:SER:N	2.57	0.41
3:M:6:TYR:CD2	3:M:17:GLN:HG2	2.56	0.41
3:M:134:PRO:O	3:M:136:VAL:HG22	2.20	0.41
3:M:222:PHE:N	3:M:222:PHE:HD1	2.19	0.41
3:M:356:LEU:HD12	3:M:437:TYR:CD2	2.56	0.41
4:S:15:ARG:CZ	4:S:122:ILE:HD11	2.50	0.41
4:S:125:TRP:O	4:S:129:GLU:HG3	2.21	0.41
1:A:253:ILE:CD1	1:A:285:THR:CG2	2.99	0.41
1:A:313:MET:HG3	1:A:348:PHE:HE1	1.86	0.41
1:A:585:PHE:CD2	1:A:603:VAL:CG1	3.01	0.41
2:B:4:SER:O	2:B:8:ILE:HD12	2.21	0.41
2:B:92:THR:HG22	2:B:93:ASN:N	2.35	0.41
2:B:172:GLU:O	2:B:175:LEU:N	2.54	0.41
2:B:230:PHE:CD2	2:B:298:ASP:CA	3.04	0.41
3:M:99:ILE:HG21	3:M:124:ILE:HG21	1.85	0.41
3:M:213:GLU:HB3	3:M:467:TYR:HA	2.03	0.41
3:M:229:LYS:HG2	3:M:230:LYS:HG3	2.02	0.41
3:M:355:ASP:OD2	3:M:357:LYS:CE	2.65	0.41
1:A:104:ARG:HG3	1:A:145:ILE:HD12	2.03	0.40
1:A:190:LEU:HD11	1:A:228:LYS:CE	2.48	0.40
1:A:259:LEU:HD23	1:A:259:LEU:C	2.41	0.40
1:A:446:ASP:C	1:A:448:GLU:O	2.59	0.40
2:B:62:ALA:O	2:B:66:ILE:HD12	2.21	0.40
2:B:252:LEU:C	2:B:302:PHE:CE2	2.95	0.40
2:B:563:PHE:O	2:B:566:ALA:N	2.54	0.40
3:M:304:VAL:O	3:M:304:VAL:HG13	2.21	0.40
3:M:353:VAL:HG22	3:M:354:ASP:O	2.21	0.40
4:S:4:ALA:CB	4:S:19:PHE:CD2	3.03	0.40

Continued on next page...

Continued from previous page...

Atom-1	Atom-2	Interatomic distance (Å)	Clash overlap (Å)
1:A:320:HIS:ND1	1:A:352:PHE:HD2	2.18	0.40
2:B:105:LEU:O	2:B:107:ARG:N	2.54	0.40
2:B:119:SER:O	2:B:123:LEU:HG	2.21	0.40
2:B:127:LEU:CB	2:B:161:LEU:HD11	2.43	0.40
2:B:127:LEU:O	2:B:135:ARG:CD	2.69	0.40
2:B:182:ARG:HD2	2:B:217:GLU:HG3	2.03	0.40
2:B:325:LEU:CD1	2:B:339:PHE:CD2	2.99	0.40
2:B:490:ILE:HG13	2:B:518:ILE:CB	2.50	0.40
2:B:522:GLU:C	2:B:524:LYS:N	2.72	0.40
2:B:523:PHE:CZ	2:B:580:TYR:CD2	3.01	0.40
2:B:556:LEU:CD2	2:B:588:ILE:HG13	2.34	0.40
2:B:589:SER:HA	2:B:618:PHE:CE2	2.55	0.40
3:M:226:PHE:HB2	3:M:481:VAL:HG22	2.03	0.40
3:M:322:LEU:HD23	3:M:322:LEU:C	2.42	0.40
3:M:386:PHE:HB3	3:M:397:TRP:HD1	1.83	0.40
4:S:3:HIS:CE1	4:S:90:ASP:HB3	2.56	0.40
4:S:58:LEU:O	4:S:59:LEU:C	2.59	0.40
1:A:170:LEU:HD13	1:A:170:LEU:HA	1.82	0.40
1:A:292:TYR:HE1	1:A:296:ASN:HB2	1.83	0.40
1:A:319:LEU:HD13	1:A:337:LEU:HB2	2.03	0.40
1:A:429:VAL:HG11	1:A:473:ILE:CD1	2.49	0.40
1:A:634:ASN:HD22	2:B:554:LYS:HA	1.82	0.40
2:B:25:VAL:O	2:B:32:GLU:O	2.40	0.40
2:B:136:CYS:SG	2:B:168:MET:CG	3.01	0.40
2:B:136:CYS:CA	2:B:172:GLU:HB2	2.51	0.40
2:B:527:PRO:CG	2:B:587:ARG:CG	2.98	0.40
2:B:546:CYS:HA	2:B:607:ILE:CB	2.51	0.40
3:M:216:VAL:O	3:M:216:VAL:CG2	2.69	0.40
3:M:234:ARG:O	3:M:236:LEU:N	2.54	0.40
3:M:319:SER:HB3	3:M:344:ILE:HA	2.03	0.40
1:A:174:ARG:HA	1:A:175:PRO:HD2	1.67	0.40
1:A:182:ILE:CG2	1:A:221:VAL:CG2	2.88	0.40
1:A:255:ARG:O	1:A:258:LYS:N	2.55	0.40
2:B:90:ILE:HD13	2:B:123:LEU:HD23	2.03	0.40
2:B:196:LEU:HB3	2:B:215:TYR:HE2	1.70	0.40
2:B:378:THR:CG2	2:B:379:LYS:H	2.34	0.40
2:B:397:GLN:NE2	2:B:431:MET:CE	2.84	0.40
2:B:433:VAL:CG1	2:B:471:TYR:HA	2.43	0.40
2:B:513:TRP:C	2:B:513:TRP:CD1	2.95	0.40
3:M:71:LYS:O	3:M:72:LEU:C	2.60	0.40
3:M:222:PHE:CB	3:M:479:PHE:CZ	3.04	0.40

Continued on next page...

Continued from previous page...

Atom-1	Atom-2	Interatomic distance (Å)	Clash overlap (Å)
3:M:262:THR:HG23	3:M:265:ASN:O	2.21	0.40
3:M:293:PRO:CB	3:M:294:ASP:O	2.68	0.40
4:S:35:VAL:HG12	4:S:77:TYR:OH	2.20	0.40
1:A:373:GLU:HG2	1:A:427:LYS:NZ	2.36	0.40
1:A:563:CYS:O	1:A:566:PHE:CE2	2.74	0.40
1:A:629:LEU:C	1:A:629:LEU:HD23	2.42	0.40
2:B:148:SER:HB3	2:B:183:ALA:HB1	2.03	0.40
2:B:158:VAL:HA	2:B:173:VAL:HG13	2.02	0.40
2:B:181:TYR:O	2:B:183:ALA:CA	2.61	0.40
2:B:343:LEU:O	2:B:347:VAL:HG23	2.21	0.40
2:B:447:GLU:O	2:B:450:VAL:HB	2.21	0.40
2:B:467:VAL:CG1	2:B:471:TYR:CE1	3.04	0.40
2:B:477:MET:O	2:B:480:GLN:N	2.54	0.40
2:B:537:PHE:C	2:B:539:ASN:N	2.75	0.40
2:B:549:LEU:O	2:B:552:SER:N	2.54	0.40
2:B:617:LEU:O	2:B:618:PHE:C	2.60	0.40
3:M:64:LYS:HE3	3:M:79:SER:HB2	2.04	0.40
3:M:122:SER:O	3:M:125:PHE:HB2	2.21	0.40
3:M:136:VAL:CG1	3:M:137:SER:N	2.83	0.40
3:M:250:LEU:CD1	3:M:254:PRO:HG2	2.52	0.40
3:M:443:SER:HG	3:M:448:TYR:HA	1.85	0.40
4:S:60:SER:O	4:S:66:ASP:CB	2.70	0.40

There are no symmetry-related clashes.

5.3 Torsion angles (i)

5.3.1 Protein backbone (i)

In the following table, the Percentiles column shows the percent Ramachandran outliers of the chain as a percentile score with respect to all PDB entries followed by that with respect to all EM entries.

The Analysed column shows the number of residues for which the backbone conformation was analysed, and the total number of residues.

Mol	Chain	Analysed	Favoured	Allowed	Outliers	Percentiles	
1	A	574/964 (60%)	547 (95%)	16 (3%)	11 (2%)	8	38
2	B	619/809 (76%)	521 (84%)	64 (10%)	34 (6%)	2	19
3	M	391/483 (81%)	331 (85%)	44 (11%)	16 (4%)	3	23

Continued on next page...

Continued from previous page...

Mol	Chain	Analysed	Favoured	Allowed	Outliers	Percentiles	
4	S	166/194 (86%)	157 (95%)	6 (4%)	3 (2%)	8	40
All	All	1750/2450 (71%)	1556 (89%)	130 (7%)	64 (4%)	6	24

All (64) Ramachandran outliers are listed below:

Mol	Chain	Res	Type
1	A	267	GLU
1	A	278	ILE
1	A	305	GLU
1	A	449	TRP
2	B	13	ASP
2	B	18	ILE
2	B	19	THR
2	B	200	MET
2	B	228	GLY
2	B	558	TYR
2	B	560	ILE
2	B	564	LYS
2	B	565	GLN
3	M	104	PHE
3	M	106	LYS
3	M	280	ASP
3	M	429	ASP
3	M	447	ILE
4	S	72	ASP
1	A	174	ARG
1	A	536	MET
1	A	571	ARG
2	B	29	LYS
2	B	78	ASP
2	B	177	ILE
2	B	179	LYS
2	B	194	ASP
2	B	215	TYR
2	B	313	SER
2	B	432	ALA
2	B	561	ASP
2	B	568	VAL
3	M	355	ASP
3	M	405	THR
4	S	65	ASN
1	A	507	GLN

Continued on next page...

Continued from previous page...

Mol	Chain	Res	Type
2	B	14	THR
2	B	42	ILE
2	B	43	ASN
2	B	196	LEU
2	B	199	LEU
2	B	227	HIS
2	B	232	ARG
2	B	242	SER
2	B	573	GLU
3	M	316	ARG
3	M	352	GLN
2	B	274	PRO
2	B	578	PRO
3	M	45	SER
3	M	254	PRO
3	M	363	ASN
4	S	164	ASP
1	A	279	LEU
1	A	306	GLU
2	B	9	ALA
2	B	182	ARG
2	B	198	GLU
2	B	296	ASP
3	M	81	SER
3	M	84	LYS
3	M	346	ASN
1	A	116	LYS
3	M	321	GLY

5.3.2 Protein sidechains ⓘ

In the following table, the Percentiles column shows the percent sidechain outliers of the chain as a percentile score with respect to all PDB entries followed by that with respect to all EM entries.

The Analysed column shows the number of residues for which the sidechain conformation was analysed, and the total number of residues.

Mol	Chain	Analysed	Rotameric	Outliers	Percentiles	
1	A	536/898 (60%)	530 (99%)	6 (1%)	73	84
2	B	565/738 (77%)	551 (98%)	14 (2%)	47	68
3	M	360/441 (82%)	351 (98%)	9 (2%)	47	68

Continued on next page...

Continued from previous page...

Mol	Chain	Analysed	Rotameric	Outliers	Percentiles	
4	S	159/175 (91%)	156 (98%)	3 (2%)	57	75
All	All	1620/2252 (72%)	1588 (98%)	32 (2%)	57	74

All (32) residues with a non-rotameric sidechain are listed below:

Mol	Chain	Res	Type
1	A	175	PRO
1	A	186	PHE
1	A	196	LEU
1	A	241	TYR
1	A	292	TYR
1	A	418	ILE
2	B	1	MET
2	B	4	SER
2	B	10	SER
2	B	12	LEU
2	B	14	THR
2	B	16	LYS
2	B	19	THR
2	B	29	LYS
2	B	30	LEU
2	B	233	TYR
2	B	259	TYR
2	B	418	TYR
2	B	453	TRP
2	B	592	TYR
3	M	6	TYR
3	M	118	TYR
3	M	214	LEU
3	M	293	PRO
3	M	343	ASN
3	M	379	LEU
3	M	437	TYR
3	M	472	TYR
3	M	479	PHE
4	S	8	PHE
4	S	38	LEU
4	S	113	PHE

Sometimes sidechains can be flipped to improve hydrogen bonding and reduce clashes. All (22) such sidechains are listed below:

Mol	Chain	Res	Type
1	A	88	ASN
1	A	102	GLN
1	A	199	ASN
1	A	476	GLN
1	A	634	ASN
2	B	41	ASN
2	B	222	HIS
2	B	258	GLN
2	B	333	GLN
2	B	352	ASN
2	B	371	GLN
2	B	397	GLN
2	B	441	GLN
2	B	486	HIS
2	B	574	ASN
3	M	17	GLN
3	M	70	ASN
3	M	309	GLN
3	M	343	ASN
3	M	346	ASN
4	S	70	ASN
4	S	126	GLN

5.3.3 RNA (i)

There are no RNA molecules in this entry.

5.4 Non-standard residues in protein, DNA, RNA chains (i)

There are no non-standard protein/DNA/RNA residues in this entry.

5.5 Carbohydrates (i)

There are no monosaccharides in this entry.

5.6 Ligand geometry (i)

There are no ligands in this entry.

5.7 Other polymers (i)

There are no such residues in this entry.

5.8 Polymer linkage issues (i)

The following chains have linkage breaks:

Mol	Chain	Number of breaks
3	M	30
4	S	8
2	B	6
1	A	4

All chain breaks are listed below:

Model	Chain	Residue-1	Atom-1	Residue-2	Atom-2	Distance (Å)
1	S	68:VAL	C	69:ASN	N	1.67
1	A	466:ASP	C	467:LYS	N	1.20
1	M	23:THR	C	24:ALA	N	1.20
1	M	65:TYR	C	66:PHE	N	1.20
1	M	252:ASP	C	253:ASN	N	1.20
1	M	288:ILE	C	289:THR	N	1.20
1	S	60:SER	C	61:ASN	N	1.20
1	A	378:ILE	C	379:VAL	N	1.19
1	B	184:GLY	C	185:LYS	N	1.19
1	B	259:TYR	C	260:LEU	N	1.19
1	B	334:MET	C	335:LYS	N	1.19
1	M	6:TYR	C	7:ILE	N	1.19
1	M	82:LYS	C	83:SER	N	1.19
1	S	62:GLU	C	63:ASN	N	1.19
1	S	114:THR	C	115:GLU	N	1.19
1	A	289:SER	C	290:VAL	N	1.18
1	A	439:ASP	C	440:ASN	N	1.18
1	M	64:LYS	C	65:TYR	N	1.18
1	M	70:ASN	C	71:LYS	N	1.18
1	M	379:LEU	C	380:ARG	N	1.18
1	S	70:ASN	C	71:GLU	N	1.18
1	M	136:VAL	C	137:SER	N	1.17
1	M	403:THR	C	404:ALA	N	1.17
1	M	450:GLU	C	451:ALA	N	1.17
1	M	453:ASP	C	454:ILE	N	1.17
1	S	6:LEU	C	7:ILE	N	1.17
1	S	22:PRO	C	23:VAL	N	1.17

Continued on next page...

Continued from previous page...

Model	Chain	Residue-1	Atom-1	Residue-2	Atom-2	Distance (Å)
1	M	62:VAL	C	63:TYR	N	1.16
1	M	134:PRO	C	135:ASN	N	1.16
1	M	279:ASN	C	280:ASP	N	1.16
1	M	319:SER	C	320:ILE	N	1.16
1	M	352:GLN	C	353:VAL	N	1.16
1	M	61:GLU	C	62:VAL	N	1.15
1	B	581:TYR	C	582:ASP	N	1.14
1	M	106:LYS	C	107:ASP	N	1.14
1	M	351:SER	C	352:GLN	N	1.14
1	B	284:ASN	C	285:GLU	N	1.13
1	B	293:VAL	C	294:VAL	N	1.13
1	M	20:LEU	C	21:GLY	N	1.13
1	M	21:GLY	C	22:ALA	N	1.13
1	M	52:ASP	C	53:HIS	N	1.13
1	M	130:GLU	C	131:ALA	N	1.12
1	M	80:THR	C	81:SER	N	1.11
1	M	105:ASP	C	106:LYS	N	1.10
1	M	81:SER	C	82:LYS	N	1.08
1	M	3:LEU	C	4:SER	N	1.07
1	S	48:SER	C	49:SER	N	1.04
1	M	135:ASN	C	136:VAL	N	0.94

6 Map visualisation (i)

This section contains visualisations of the EMDB entry EMD-13188. These allow visual inspection of the internal detail of the map and identification of artifacts.

No raw map or half-maps were deposited for this entry and therefore no images, graphs, etc. pertaining to the raw map can be shown.

6.1 Orthogonal projections (i)

6.1.1 Primary map

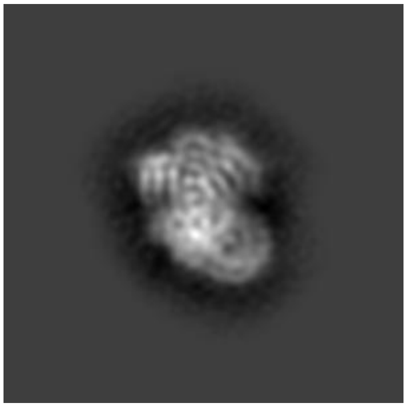

X

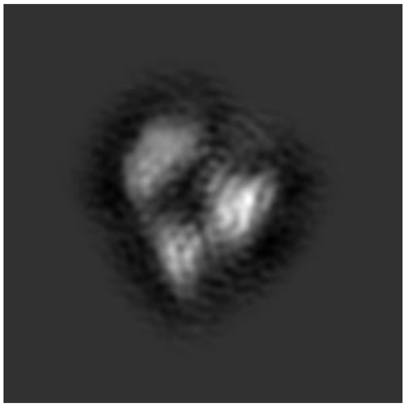

Y

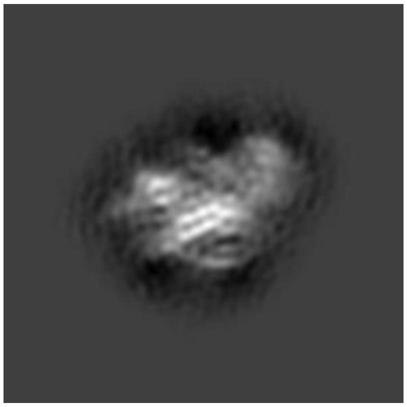

Z

The images above show the map projected in three orthogonal directions.

6.2 Central slices (i)

6.2.1 Primary map

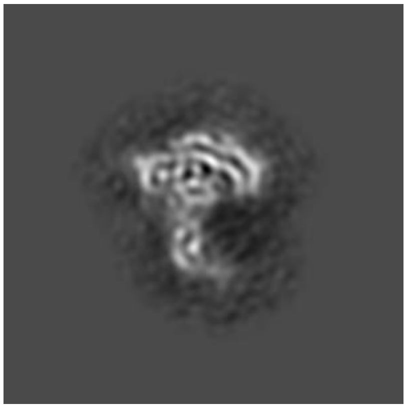

X Index: 132

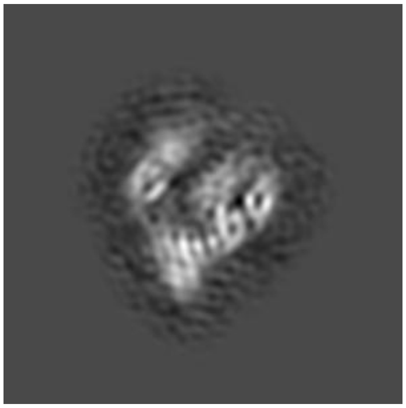

Y Index: 132

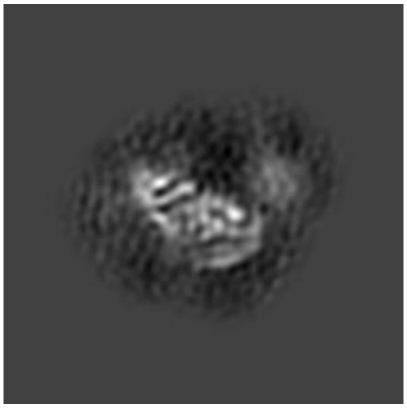

Z Index: 132

The images above show central slices of the map in three orthogonal directions.

6.3 Largest variance slices (i)

6.3.1 Primary map

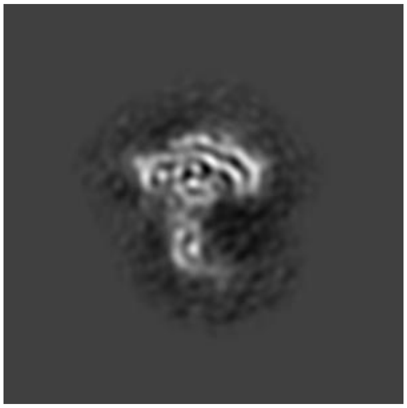

X Index: 131

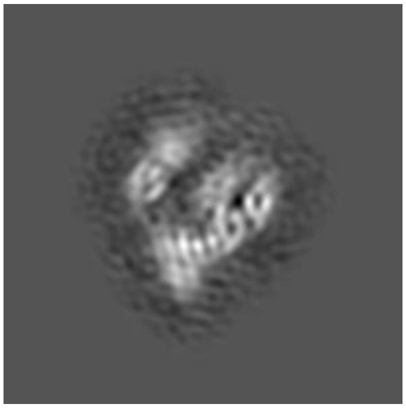

Y Index: 133

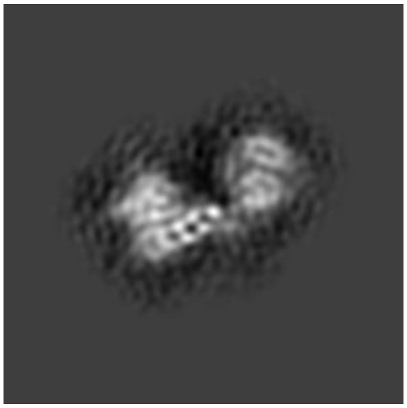

Z Index: 111

The images above show the largest variance slices of the map in three orthogonal directions.

6.4 Orthogonal surface views (i)

6.4.1 Primary map

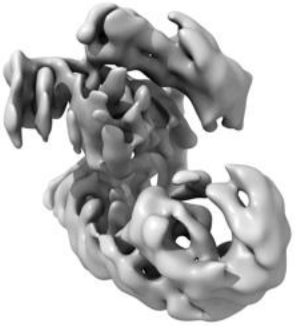

X

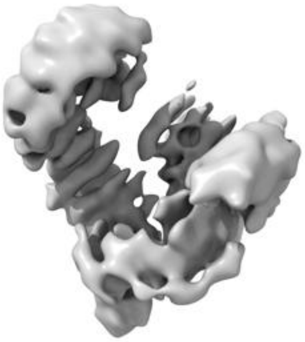

Y

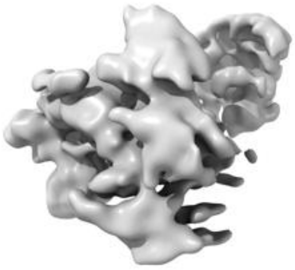

Z

The images above show the 3D surface view of the map at the recommended contour level 0.018. These images, in conjunction with the slice images, may facilitate assessment of whether an appropriate contour level has been provided.

6.5 Mask visualisation (i)

This section was not generated. No masks/segmentation were deposited.

7 Map analysis (i)

This section contains the results of statistical analysis of the map.

7.1 Map-value distribution (i)

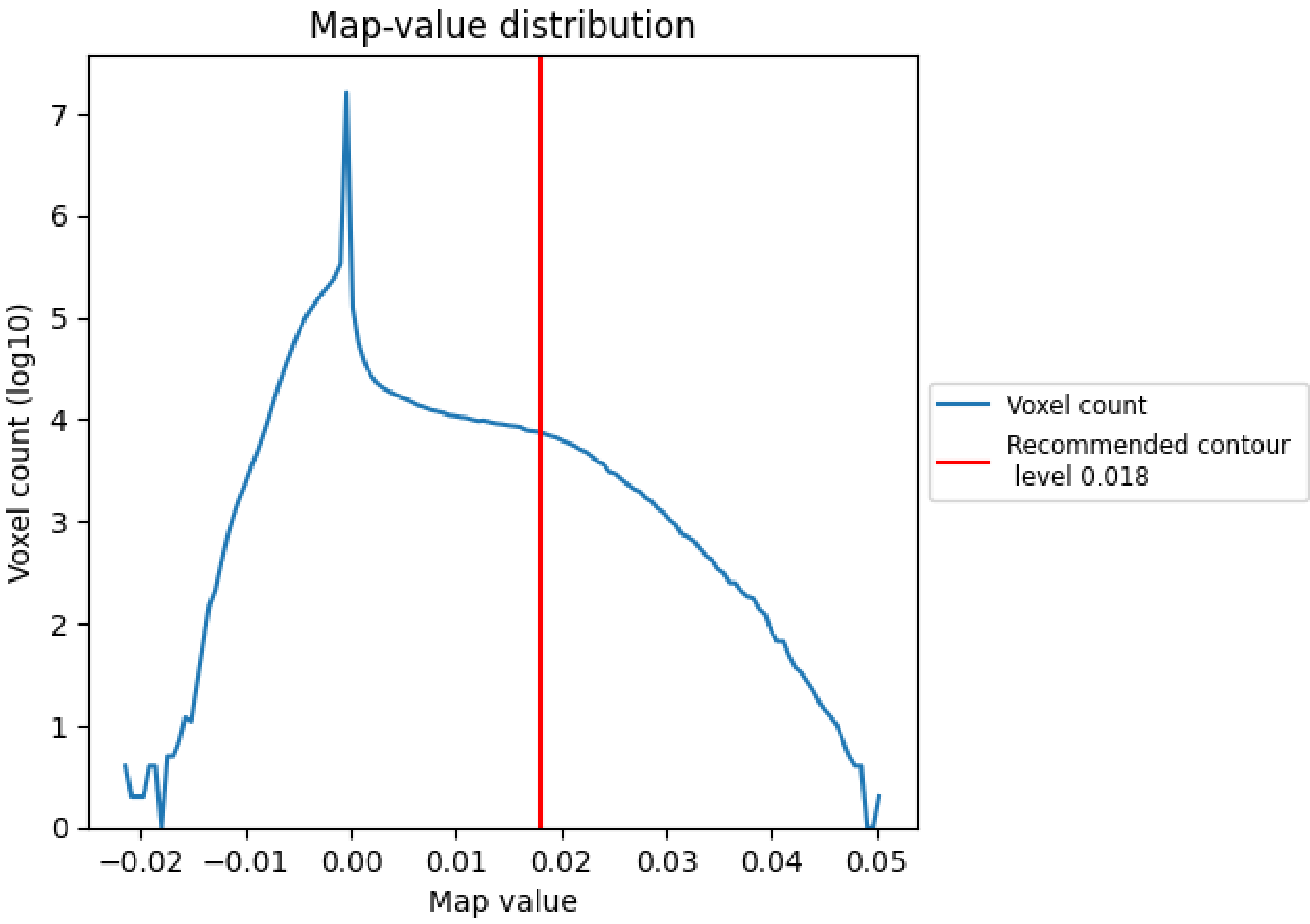

The map-value distribution is plotted in 128 intervals along the x-axis. The y-axis is logarithmic. A spike in this graph at zero usually indicates that the volume has been masked.

7.2 Volume estimate (i)

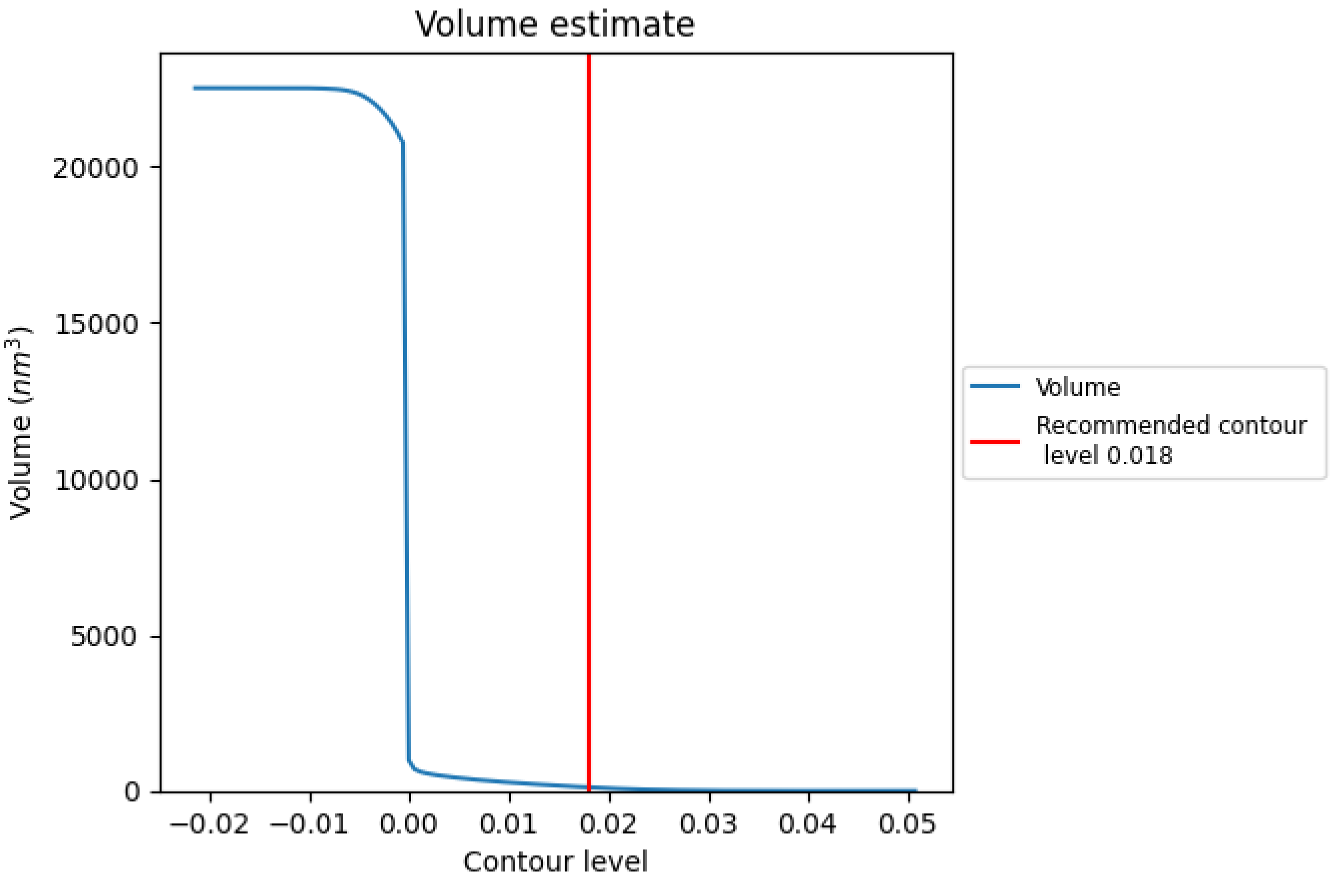

The volume at the recommended contour level is 115 nm^3; this corresponds to an approximate mass of 104 kDa.

The volume estimate graph shows how the enclosed volume varies with the contour level. The recommended contour level is shown as a vertical line and the intersection between the line and the curve gives the volume of the enclosed surface at the given level.

7.3 Rotationally averaged power spectrum ⓘ

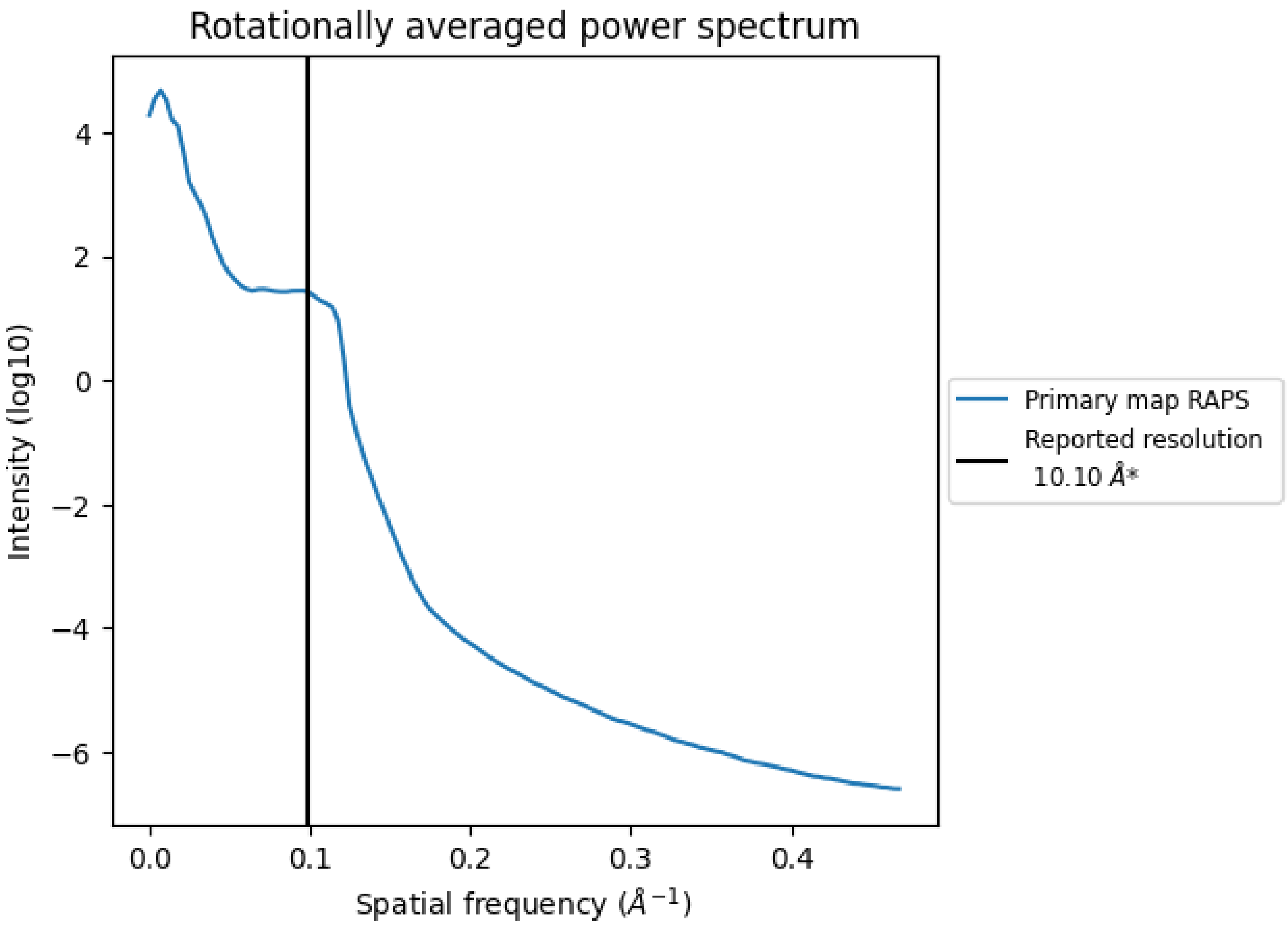

*Reported resolution corresponds to spatial frequency of 0.099 Å^{-1}

8 Fourier-Shell correlation (i)

This section was not generated. No FSC curve or half-maps provided.

9 Map-model fit (i)

This section contains information regarding the fit between EMDB map EMD-13188 and PDB model 7P3Y. Per-residue inclusion information can be found in section 3 on page 5.

9.1 Map-model overlay (i)

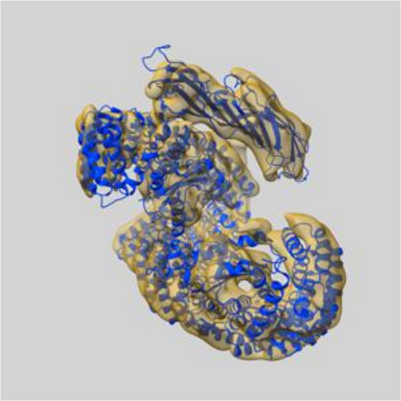

X

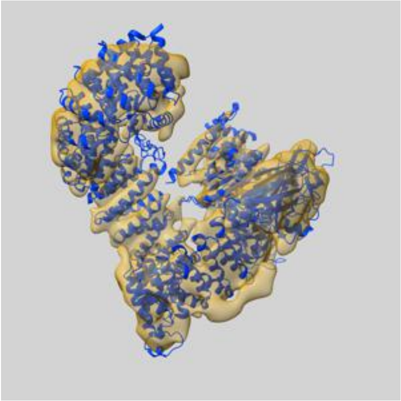

Y

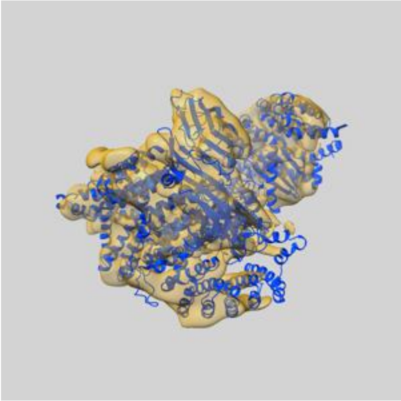

Z

The images above show the 3D surface view of the map at the recommended contour level 0.018 at 50% transparency in yellow overlaid with a ribbon representation of the model coloured in blue. These images allow for the visual assessment of the quality of fit between the atomic model and the map.

9.2 Atom inclusion (i)

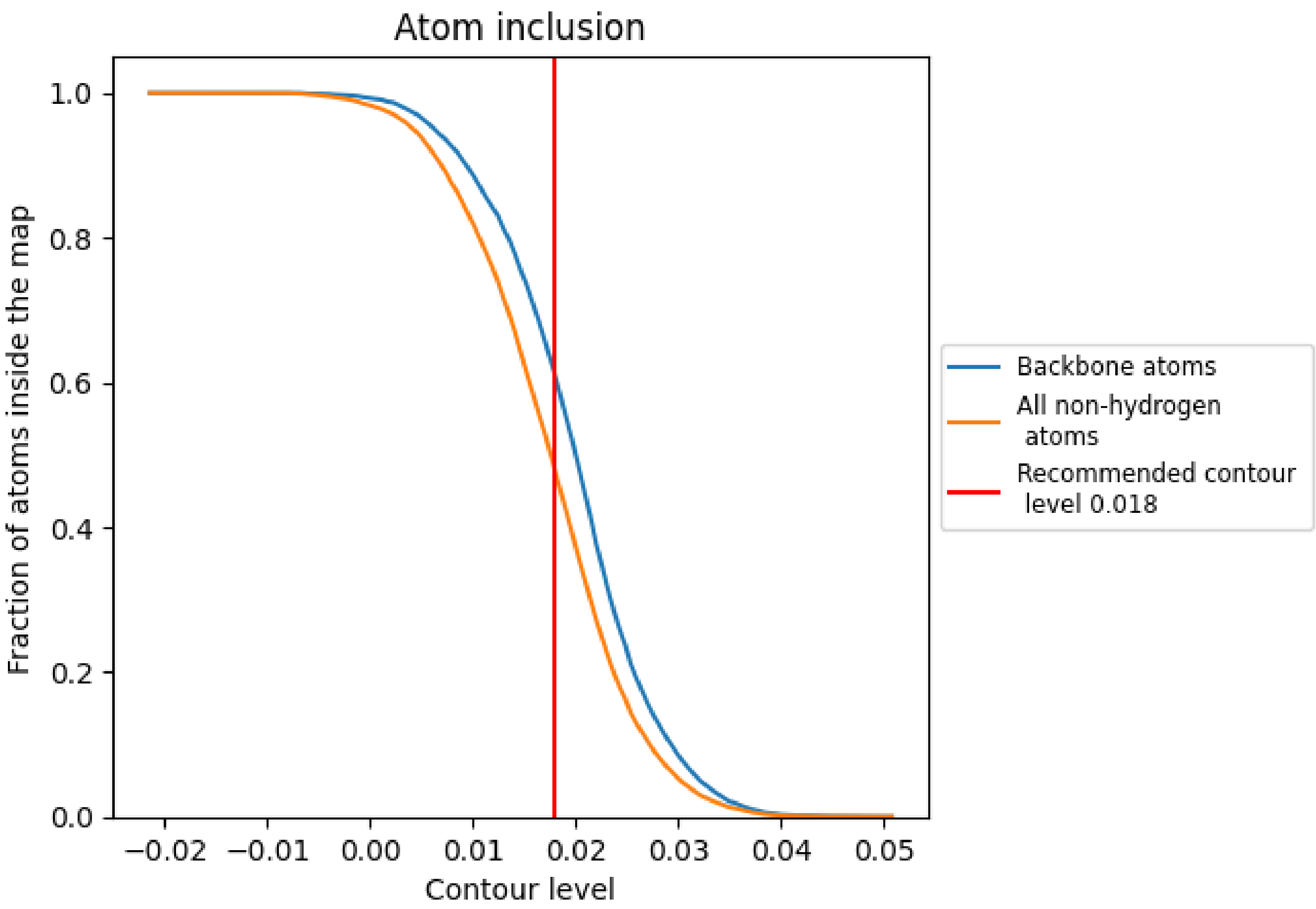

At the recommended contour level, 61% of all backbone atoms, 48% of all non-hydrogen atoms, are inside the map.
