# Supplementary material for: Flexible open conformation of the AP-3 complex explains its role in cargo recruitment at the Golgi
Source: J Biol Chem. 2021 Oct 22;297(5):101334. doi: 10.1016/j.jbc.2021.101334 (PMC8591511; doi:10.1016/j.jbc.2021.101334)
Supplement: Supporting information [file mmc8.pdf]

Full wwPDB EM Validation Report (i)

Jul 22, 2021 – 03:22 pm BST

PDB ID	:	7P3Z
EMDB ID	:	EMD-13189
Title	:	Homology model of the full-length AP-3 complex in a stretched open conformation
Deposited on	:	2021-07-09
Resolution	:	10.50 Å(reported)

This is a Full wwPDB EM Validation Report.

This report is produced by the wwPDB biocuration pipeline after annotation of the structure.

We welcome your comments at *validation@mail.wwpdb.org*
A user guide is available at
https://www.wwpdb.org/validation/2017/EMValidationReportHelp
with specific help available everywhere you see the (i) symbol.

The following versions of software and data (see references (i)) were used in the production of this report:

EMDB validation analysis	:	0.0.0.dev84
MolProbity	:	4.02b-467
Percentile statistics	:	20191225.v01 (using entries in the PDB archive December 25th 2019)
Ideal geometry (proteins)	:	Engh & Huber (2001)
Ideal geometry (DNA, RNA)	:	Parkinson et al. (1996)
Validation Pipeline (wwPDB-VP)	:	2.22

1 Overall quality at a glance (i)

The following experimental techniques were used to determine the structure:
ELECTRON MICROSCOPY

The reported resolution of this entry is 10.50 Å.

Percentile scores (ranging between 0-100) for global validation metrics of the entry are shown in the following graphic. The table shows the number of entries on which the scores are based.

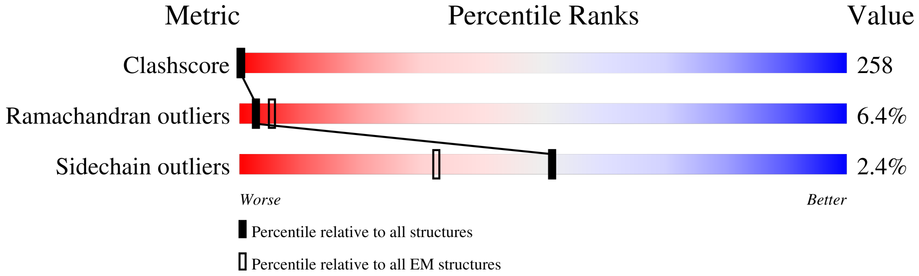

Metric	Whole archive (#Entries)	EM structures (#Entries)
Clashscore	158937	4297
Ramachandran outliers	154571	4023
Sidechain outliers	154315	3826

The table below summarises the geometric issues observed across the polymeric chains and their fit to the map. The red, orange, yellow and green segments of the bar indicate the fraction of residues that contain outliers for >=3, 2, 1 and 0 types of geometric quality criteria respectively. A grey segment represents the fraction of residues that are not modelled. The numeric value for each fraction is indicated below the corresponding segment, with a dot representing fractions <=5% The upper red bar (where present) indicates the fraction of residues that have poor fit to the EM map (all-atom inclusion < 40%). The numeric value is given above the bar.

Mol	Chain	Length	Quality of chain
1	A	964	17% / • 20% 31% 6% 40%
2	B	809	30% / • 25% 42% 7% 23%
3	M	483	27% / 10% 48% 20% • 19%
4	S	194	32% / 7% 38% 37% • 13%

2 Entry composition

There are 4 unique types of molecules in this entry. The entry contains 14041 atoms, of which 0 are hydrogens and 0 are deuteriums.

In the tables below, the AltConf column contains the number of residues with at least one atom in alternate conformation and the Trace column contains the number of residues modelled with at most 2 atoms.

- Molecule 1 is a protein called AP-3 complex subunit delta.

Mol	Chain	Residues	Atoms					AltConf	Trace
			Total	C	N	O	S		
1	A	576	4625	2978	738	881	28	0	0

There are 32 discrepancies between the modelled and reference sequences:

Chain	Residue	Modelled	Actual	Comment	Reference
A	933	ARG	-	expression tag	UNP A0A7I9C4X2
A	934	THR	-	expression tag	UNP A0A7I9C4X2
A	935	LEU	-	expression tag	UNP A0A7I9C4X2
A	936	GLN	-	expression tag	UNP A0A7I9C4X2
A	937	VAL	-	expression tag	UNP A0A7I9C4X2
A	938	ASP	-	expression tag	UNP A0A7I9C4X2
A	939	GLY	-	expression tag	UNP A0A7I9C4X2
A	940	SER	-	expression tag	UNP A0A7I9C4X2
A	941	ASP	-	expression tag	UNP A0A7I9C4X2
A	942	TYR	-	expression tag	UNP A0A7I9C4X2
A	943	LYS	-	expression tag	UNP A0A7I9C4X2
A	944	ASP	-	expression tag	UNP A0A7I9C4X2
A	945	ASP	-	expression tag	UNP A0A7I9C4X2
A	946	ASP	-	expression tag	UNP A0A7I9C4X2
A	947	ASP	-	expression tag	UNP A0A7I9C4X2
A	948	LYS	-	expression tag	UNP A0A7I9C4X2
A	949	ASP	-	expression tag	UNP A0A7I9C4X2
A	950	TYR	-	expression tag	UNP A0A7I9C4X2
A	951	LYS	-	expression tag	UNP A0A7I9C4X2
A	952	ASP	-	expression tag	UNP A0A7I9C4X2
A	953	ASP	-	expression tag	UNP A0A7I9C4X2
A	954	ASP	-	expression tag	UNP A0A7I9C4X2
A	955	ASP	-	expression tag	UNP A0A7I9C4X2
A	956	LYS	-	expression tag	UNP A0A7I9C4X2
A	957	ASP	-	expression tag	UNP A0A7I9C4X2
A	958	TYR	-	expression tag	UNP A0A7I9C4X2
A	959	LYS	-	expression tag	UNP A0A7I9C4X2
A	960	ASP	-	expression tag	UNP A0A7I9C4X2

Continued on next page...

Continued from previous page...

Chain	Residue	Modelled	Actual	Comment	Reference
A	961	ASP	-	expression tag	UNP A0A7I9C4X2
A	962	ASP	-	expression tag	UNP A0A7I9C4X2
A	963	ASP	-	expression tag	UNP A0A7I9C4X2
A	964	LYS	-	expression tag	UNP A0A7I9C4X2

- Molecule 2 is a protein called Y55_G0035830.mRNA.1.CDS.1.

Mol	Chain	Residues	Atoms					AltConf	Trace
2	B	621	Total<br>4954	C<br>3160	N<br>830	O<br>936	S<br>28	0	0

- Molecule 3 is a protein called AP-3 complex subunit mu.

Mol	Chain	Residues	Atoms					AltConf	Trace
3	M	391	Total<br>3106	C<br>1986	N<br>509	O<br>599	S<br>12	0	0

- Molecule 4 is a protein called AP complex subunit sigma.

Mol	Chain	Residues	Atoms					AltConf	Trace
4	S	168	Total<br>1356	C<br>867	N<br>215	O<br>270	S<br>4	0	0

3 Residue-property plots (i)

These plots are drawn for all protein, RNA, DNA and oligosaccharide chains in the entry. The first graphic for a chain summarises the proportions of the various outlier classes displayed in the second graphic. The second graphic shows the sequence view annotated by issues in geometry and atom inclusion in map density. Residues are color-coded according to the number of geometric quality criteria for which they contain at least one outlier: green = 0, yellow = 1, orange = 2 and red = 3 or more. A red diamond above a residue indicates a poor fit to the EM map for this residue (all-atom inclusion < 40%). Stretches of 2 or more consecutive residues without any outlier are shown as a green connector. Residues present in the sample, but not in the model, are shown in grey.

- Molecule 1: AP-3 complex subunit delta

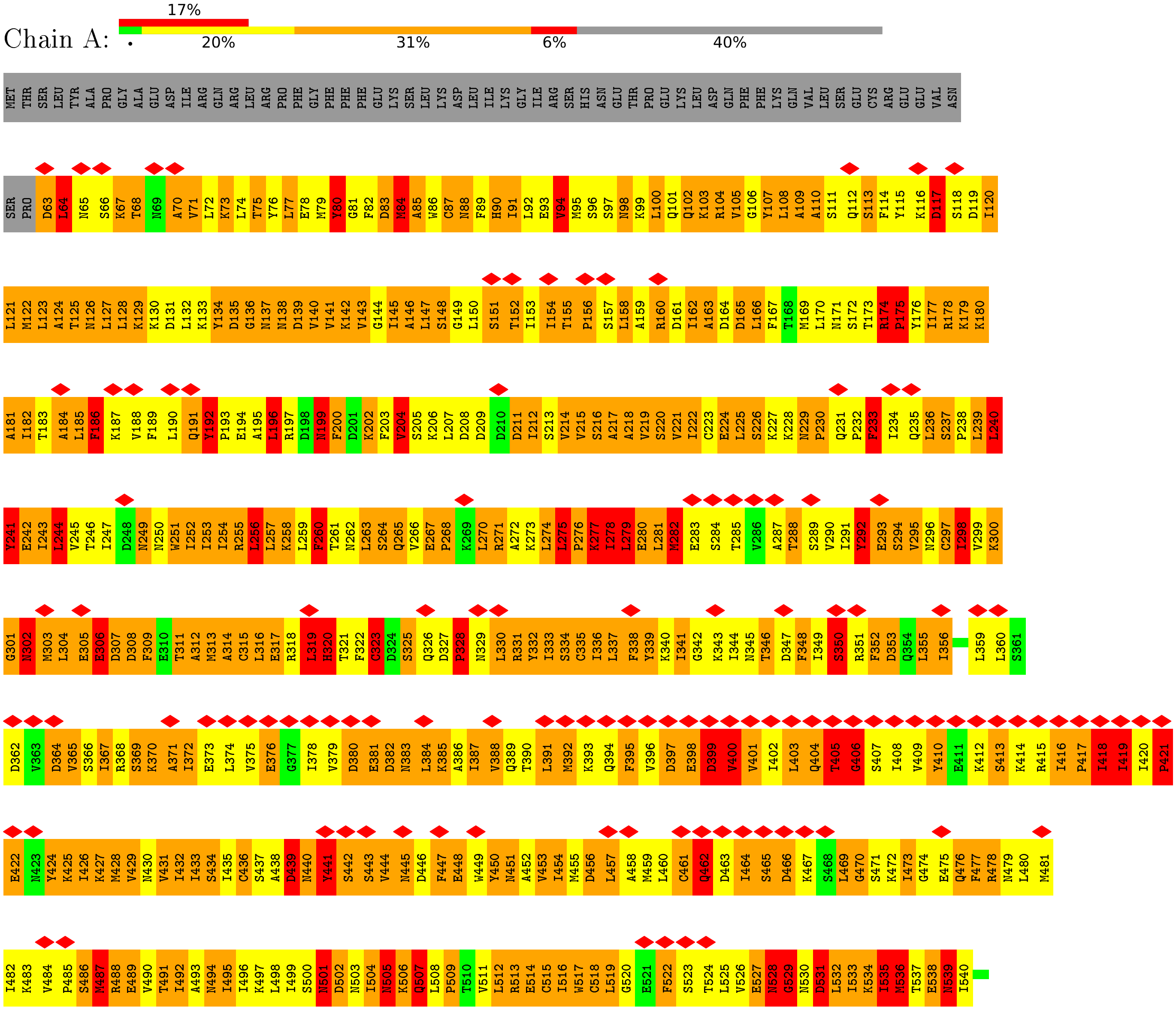

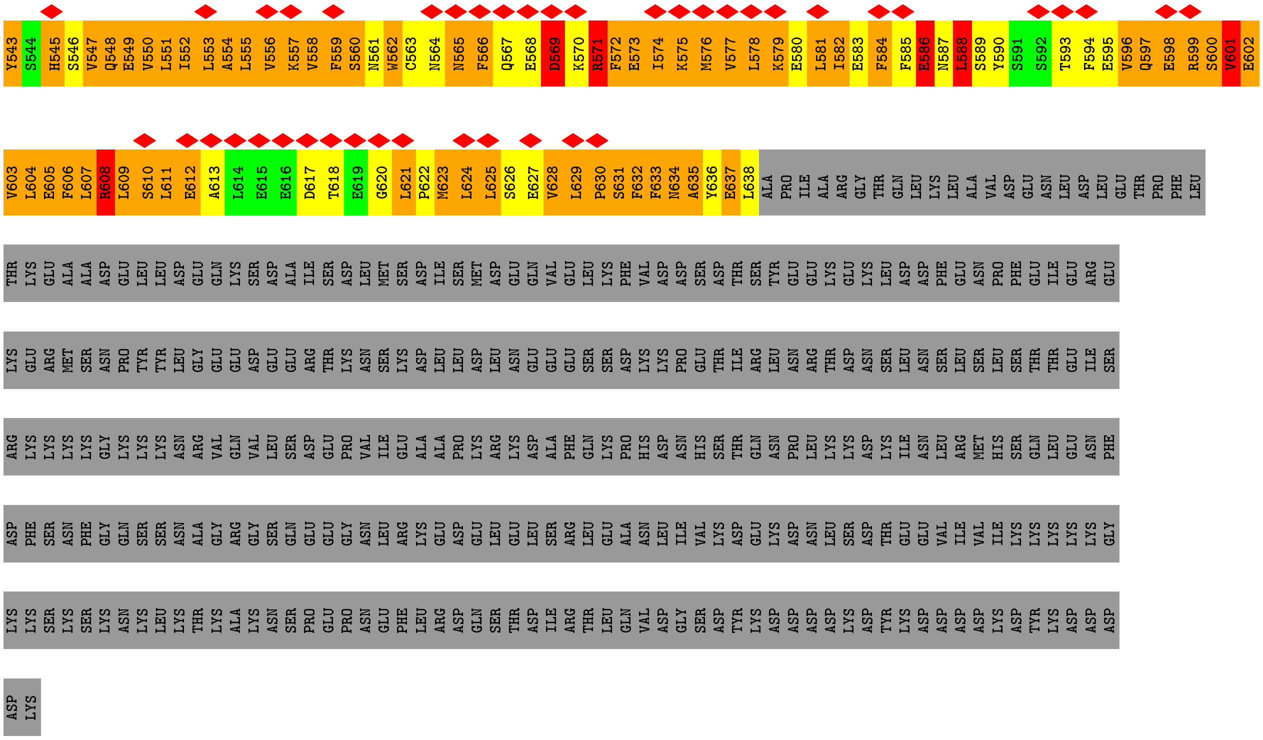

- Molecule 2: Y55_G0035830.mRNA.1.CDS.1

Chain B:

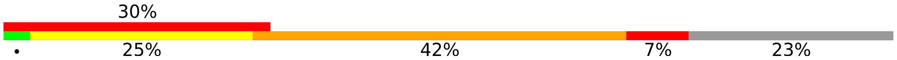

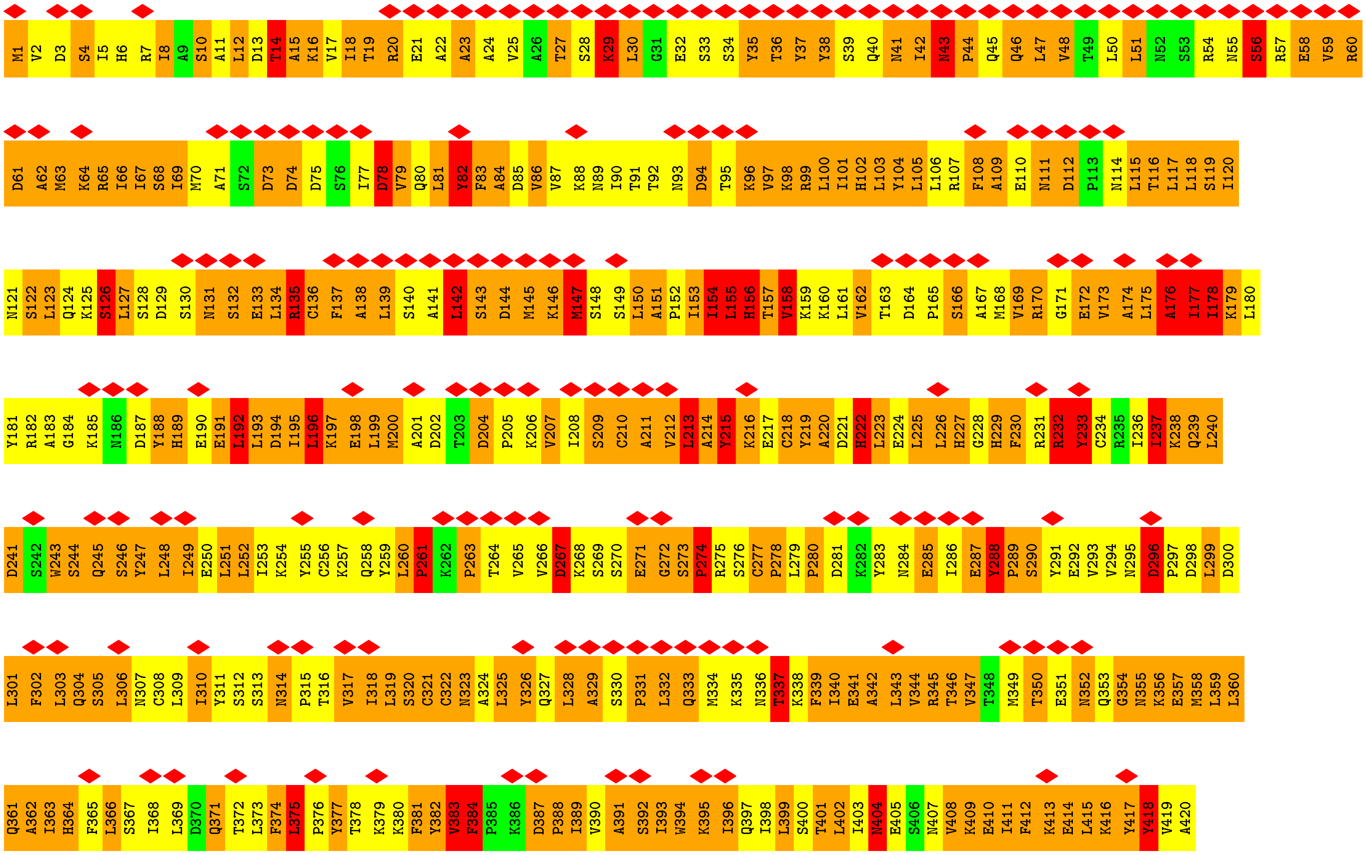

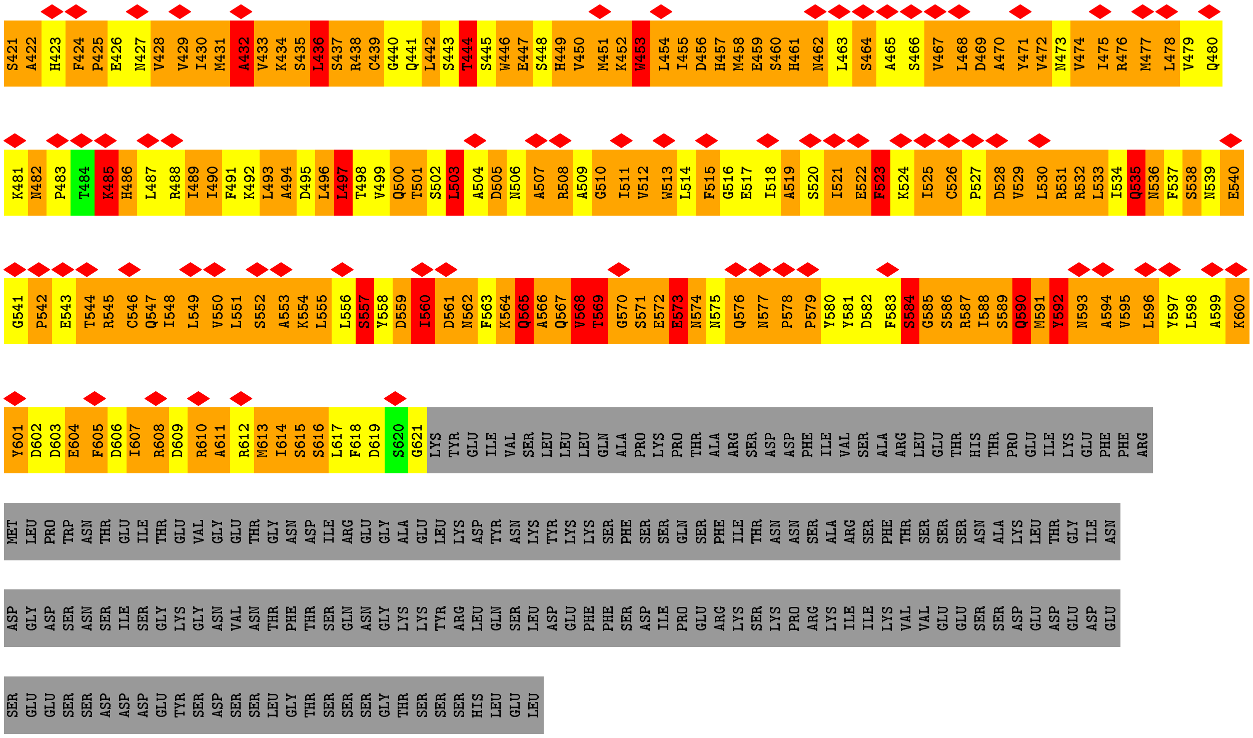

- Molecule 3: AP-3 complex subunit mu

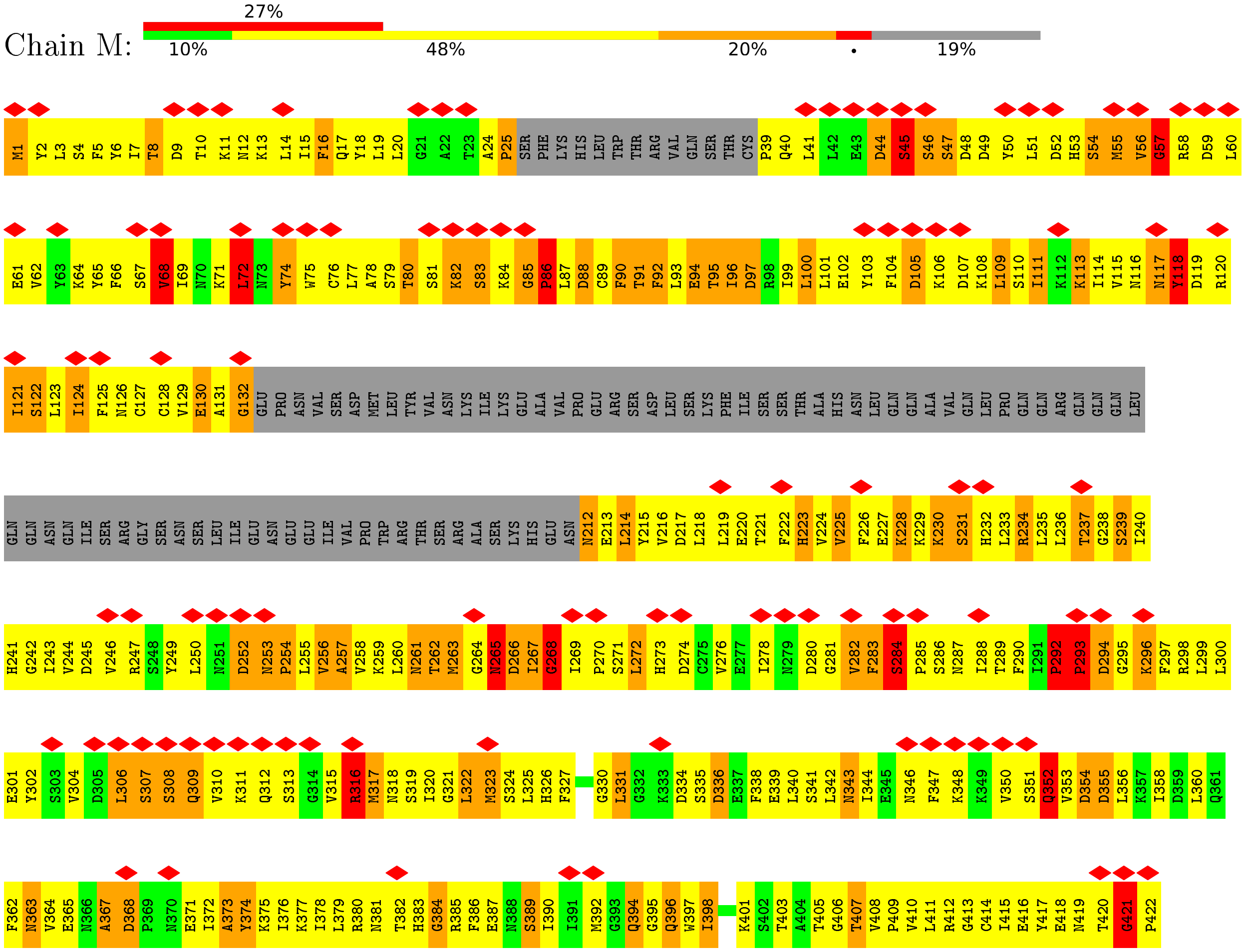

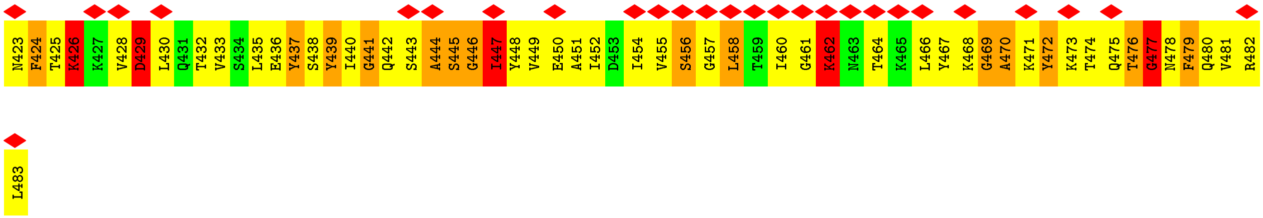

• Molecule 4: AP complex subunit sigma

Chain S:

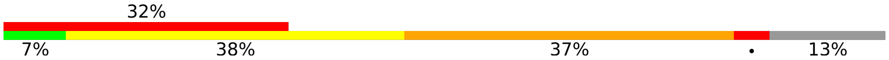

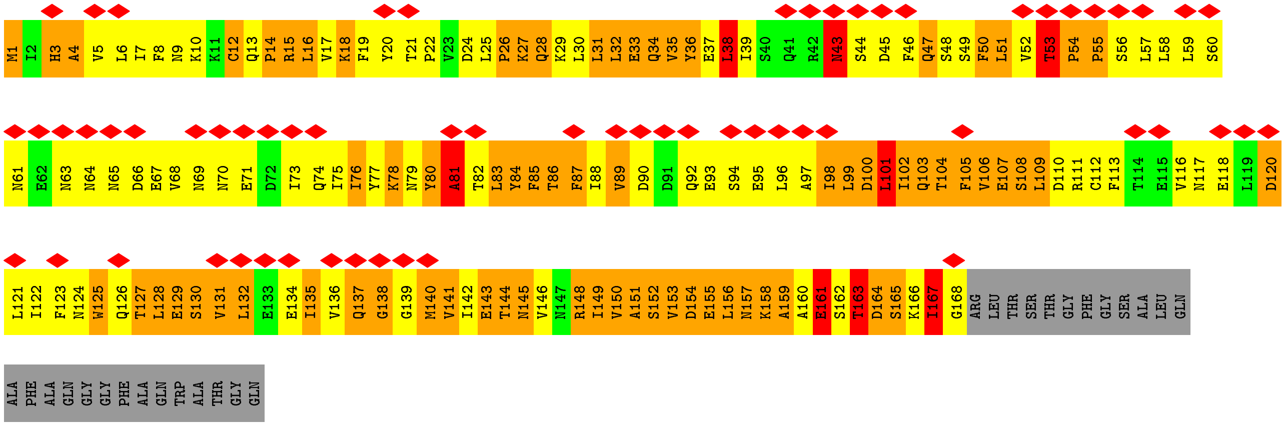

4 Experimental information (i)

Property	Value	Source
EM reconstruction method	SINGLE PARTICLE	Depositor
Imposed symmetry	POINT, C1	Depositor
Number of particles used	20312	Depositor
Resolution determination method	FSC 0.5 CUT-OFF	Depositor
CTF correction method	PHASE FLIPPING ONLY	Depositor
Microscope	FEI TITAN KRIOS	Depositor
Voltage (kV)	300	Depositor
Electron dose $(e^-/\text{Å}^2)$	81	Depositor
Minimum defocus (nm)	1500	Depositor
Maximum defocus (nm)	3600	Depositor
Magnification	130000	Depositor
Image detector	GATAN K2 SUMMIT (4k x 4k)	Depositor
Maximum map value	0.046	Depositor
Minimum map value	-0.015	Depositor
Average map value	0.000	Depositor
Map value standard deviation	0.002	Depositor
Recommended contour level	0.0155	Depositor
Map size (Å)	282.48, 282.48, 282.48	wwPDB
Map dimensions	264, 264, 264	wwPDB
Map angles (°)	90.0, 90.0, 90.0	wwPDB
Pixel spacing (Å)	1.07, 1.07, 1.07	Depositor

5 Model quality (i)

5.1 Standard geometry (i)

The Z score for a bond length (or angle) is the number of standard deviations the observed value is removed from the expected value. A bond length (or angle) with $|Z| > 5$ is considered an outlier worth inspection. RMSZ is the root-mean-square of all Z scores of the bond lengths (or angles).

Mol	Chain	Bond lengths		Bond angles	
		RMSZ	$\#\|Z\| > 5$	RMSZ	$\#\|Z\| > 5$
1	A	1.68	70/4699 (1.5%)	2.61	607/6358 (9.5%)
2	B	1.52	52/5047 (1.0%)	2.32	540/6841 (7.9%)
3	M	1.59	61/3163 (1.9%)	1.85	139/4271 (3.3%)
4	S	1.84	33/1377 (2.4%)	2.17	116/1872 (6.2%)
All	All	1.62	216/14286 (1.5%)	2.31	1402/19342 (7.2%)

Chiral center outliers are detected by calculating the chiral volume of a chiral center and verifying if the center is modelled as a planar moiety or with the opposite hand.A planarity outlier is detected by checking planarity of atoms in a peptide group, atoms in a mainchain group or atoms of a sidechain that are expected to be planar.

Mol	Chain	#Chirality outliers	#Planarity outliers
1	A	0	49
2	B	0	28
3	M	0	14
4	S	0	8
All	All	0	99

All (216) bond length outliers are listed below:

Mol	Chain	Res	Type	Atoms	Z	Observed(Å)	Ideal(Å)
3	M	132	GLY	CA-C	-17.19	1.24	1.51
4	S	53	THR	N-CA	-14.56	1.17	1.46
3	M	132	GLY	N-CA	-14.40	1.24	1.46
1	A	406	GLY	CA-C	13.34	1.73	1.51
3	M	293	PRO	N-CD	12.51	1.65	1.47
4	S	163	THR	CA-C	11.13	1.81	1.52
4	S	46	PHE	CA-C	-11.05	1.24	1.52
4	S	55	PRO	N-CA	10.71	1.65	1.47
3	M	56	VAL	CA-C	-10.60	1.25	1.52
1	A	218	ALA	CA-C	-10.18	1.26	1.52
1	A	139	ASP	N-CA	-9.65	1.27	1.46
1	A	407	SER	N-CA	9.46	1.65	1.46

Continued on next page...

Continued from previous page...

Mol	Chain	Res	Type	Atoms	Z	Observed(Å)	Ideal(Å)
4	S	51	LEU	N-CA	-9.35	1.27	1.46
1	A	175	PRO	N-CD	9.26	1.60	1.47
3	M	41	LEU	CA-C	-9.12	1.29	1.52
2	B	337	THR	N-CA	-9.11	1.28	1.46
3	M	295	GLY	CA-C	9.02	1.66	1.51
4	S	54	PRO	N-CA	8.98	1.62	1.47
4	S	144	THR	N-CA	-8.82	1.28	1.46
3	M	231	SER	N-CA	8.77	1.63	1.46
4	S	4	ALA	CA-CB	8.56	1.70	1.52
1	A	467	LYS	N-CA	-8.49	1.29	1.46
2	B	289	PRO	CA-C	-8.40	1.36	1.52
1	A	394	GLN	CA-C	-8.30	1.31	1.52
1	A	405	THR	CA-C	-8.16	1.31	1.52
3	M	225	VAL	CA-C	-8.04	1.32	1.52
3	M	335	SER	CA-C	-8.04	1.32	1.52
2	B	212	VAL	C-O	8.02	1.38	1.23
4	S	83	LEU	CA-C	7.92	1.73	1.52
3	M	354	ASP	CA-C	7.83	1.73	1.52
4	S	140	MET	N-CA	-7.76	1.30	1.46
1	A	87	CYS	N-CA	-7.71	1.30	1.46
3	M	294	ASP	N-CA	-7.71	1.30	1.46
3	M	283	PHE	N-CA	-7.54	1.31	1.46
4	S	55	PRO	CA-C	-7.50	1.37	1.52
3	M	45	SER	CA-C	7.49	1.72	1.52
1	A	406	GLY	C-N	7.45	1.51	1.34
1	A	217	ALA	C-N	7.39	1.51	1.34
3	M	239	SER	N-CA	-7.35	1.31	1.46
3	M	109	LEU	N-CA	-7.32	1.31	1.46
3	M	44	ASP	C-N	7.23	1.50	1.34
4	S	167	ILE	C-N	7.19	1.46	1.33
3	M	293	PRO	CA-C	-7.19	1.38	1.52
3	M	441	GLY	CA-C	-7.16	1.40	1.51
3	M	95	THR	N-CA	7.10	1.60	1.46
1	A	529	GLY	N-CA	-7.03	1.35	1.46
2	B	330	SER	N-CA	-6.99	1.32	1.46
2	B	330	SER	C-N	-6.96	1.21	1.34
1	A	243	ILE	N-CA	-6.96	1.32	1.46
1	A	301	GLY	CA-C	-6.95	1.40	1.51
2	B	329	ALA	CA-C	-6.86	1.35	1.52
3	M	441	GLY	N-CA	-6.81	1.35	1.46
1	A	218	ALA	CA-CB	-6.77	1.38	1.52
1	A	230	PRO	N-CD	-6.74	1.38	1.47

Continued on next page...

Continued from previous page...

Mol	Chain	Res	Type	Atoms	Z	Observed(Å)	Ideal(Å)
3	M	55	MET	N-CA	-6.73	1.32	1.46
3	M	420	THR	N-CA	-6.69	1.32	1.46
3	M	55	MET	C-N	6.68	1.49	1.34
1	A	465	SER	N-CA	-6.67	1.33	1.46
3	M	230	LYS	CA-C	6.67	1.70	1.52
1	A	509	PRO	N-CD	6.65	1.57	1.47
3	M	322	LEU	C-O	6.61	1.35	1.23
3	M	392	MET	N-CA	-6.59	1.33	1.46
4	S	55	PRO	N-CD	6.53	1.56	1.47
4	S	108	SER	N-CA	-6.50	1.33	1.46
3	M	212	ASN	N-CA	-6.48	1.33	1.46
4	S	143	GLU	CA-C	-6.48	1.36	1.52
1	A	533	ILE	N-CA	-6.46	1.33	1.46
4	S	138	GLY	C-N	6.46	1.44	1.33
4	S	103	GLN	CA-C	-6.43	1.36	1.52
3	M	336	ASP	N-CA	-6.34	1.33	1.46
1	A	298	ILE	C-O	6.30	1.35	1.23
4	S	3	HIS	N-CA	-6.26	1.33	1.46
2	B	458	MET	C-O	6.25	1.35	1.23
2	B	329	ALA	N-CA	-6.23	1.33	1.46
1	A	410	TYR	N-CA	-6.21	1.33	1.46
4	S	81	ALA	CA-CB	-6.21	1.39	1.52
1	A	71	VAL	CA-C	-6.20	1.36	1.52
4	S	124	ASN	N-CA	-6.18	1.33	1.46
2	B	568	VAL	N-CA	-6.18	1.33	1.46
1	A	221	VAL	N-CA	6.16	1.58	1.46
1	A	566	PHE	CA-C	-6.15	1.36	1.52
1	A	394	GLN	N-CA	-6.14	1.34	1.46
2	B	585	GLY	N-CA	-6.11	1.36	1.46
1	A	278	ILE	N-CA	-6.10	1.34	1.46
1	A	464	ILE	CA-C	-6.10	1.37	1.52
2	B	172	GLU	CD-OE2	-6.02	1.19	1.25
3	M	56	VAL	C-N	-6.00	1.22	1.33
2	B	522	GLU	CA-C	-5.98	1.37	1.52
3	M	91	THR	CA-C	-5.95	1.37	1.52
1	A	418	ILE	C-N	5.93	1.47	1.34
1	A	281	LEU	N-CA	5.92	1.58	1.46
1	A	444	VAL	N-CA	-5.92	1.34	1.46
1	A	466	ASP	C-N	-5.91	1.20	1.34
1	A	534	LYS	CA-C	-5.91	1.37	1.52
1	A	277	LYS	C-N	-5.91	1.20	1.34
3	M	97	ASP	CA-C	-5.90	1.37	1.52

Continued on next page...

Continued from previous page...

Mol	Chain	Res	Type	Atoms	Z	Observed(Å)	Ideal(Å)
3	M	252	ASP	C-N	-5.90	1.20	1.34
3	M	257	ALA	N-CA	-5.90	1.34	1.46
3	M	262	THR	CA-C	-5.90	1.37	1.52
4	S	14	PRO	C-N	-5.87	1.20	1.34
3	M	446	GLY	CA-C	5.86	1.61	1.51
3	M	25	PRO	CA-C	-5.85	1.41	1.52
1	A	84	MET	N-CA	-5.84	1.34	1.46
3	M	263	MET	N-CA	-5.78	1.34	1.46
3	M	456	SER	C-N	5.78	1.43	1.33
1	A	302	ASN	CA-C	-5.77	1.38	1.52
3	M	83	SER	N-CA	-5.76	1.34	1.46
2	B	573	GLU	CD-OE2	-5.75	1.19	1.25
4	S	139	GLY	CA-C	-5.75	1.42	1.51
2	B	150	LEU	N-CA	-5.75	1.34	1.46
2	B	567	GLN	CA-C	-5.75	1.38	1.52
1	A	588	LEU	C-N	-5.73	1.20	1.34
2	B	576	GLN	C-N	-5.73	1.20	1.34
1	A	303	MET	N-CA	-5.72	1.34	1.46
1	A	241	TYR	N-CA	-5.71	1.34	1.46
1	A	508	LEU	N-CA	-5.70	1.34	1.46
4	S	95	GLU	CD-OE2	-5.70	1.19	1.25
2	B	290	SER	N-CA	-5.69	1.34	1.46
2	B	187	ASP	CA-C	-5.68	1.38	1.52
1	A	406	GLY	N-CA	-5.68	1.37	1.46
2	B	579	PRO	N-CD	-5.67	1.40	1.47
2	B	222	HIS	N-CA	5.65	1.57	1.46
3	M	96	ILE	C-N	5.61	1.47	1.34
3	M	113	LYS	CA-C	-5.61	1.38	1.52
1	A	387	ILE	CA-C	-5.61	1.38	1.52
2	B	501	THR	N-CA	-5.60	1.35	1.46
1	A	531	ASP	CA-C	-5.60	1.38	1.52
1	A	348	PHE	CA-C	-5.58	1.38	1.52
2	B	82	TYR	N-CA	-5.58	1.35	1.46
4	S	83	LEU	C-N	-5.58	1.21	1.34
1	A	405	THR	C-N	-5.57	1.23	1.33
2	B	526	CYS	N-CA	-5.57	1.35	1.46
4	S	158	LYS	CA-C	-5.55	1.38	1.52
3	M	296	LYS	CA-C	-5.54	1.38	1.52
2	B	219	TYR	C-N	-5.52	1.21	1.34
2	B	485	LYS	CA-C	-5.51	1.38	1.52
4	S	99	LEU	CA-C	-5.51	1.38	1.52
1	A	263	LEU	N-CA	-5.51	1.35	1.46

Continued on next page...

Continued from previous page...

Mol	Chain	Res	Type	Atoms	Z	Observed(Å)	Ideal(Å)
3	M	323	MET	N-CA	-5.50	1.35	1.46
4	S	80	TYR	N-CA	-5.50	1.35	1.46
4	S	83	LEU	N-CA	-5.50	1.35	1.46
2	B	584	SER	C-N	-5.49	1.23	1.33
4	S	84	TYR	C-N	-5.48	1.21	1.34
3	M	367	ALA	CA-CB	-5.47	1.41	1.52
3	M	25	PRO	N-CA	-5.44	1.38	1.47
4	S	144	THR	C-N	-5.43	1.21	1.34
1	A	373	GLU	CD-OE2	-5.42	1.19	1.25
3	M	284	SER	C-N	-5.42	1.24	1.34
3	M	457	GLY	CA-C	-5.40	1.43	1.51
2	B	574	ASN	N-CA	-5.39	1.35	1.46
2	B	601	TYR	N-CA	-5.39	1.35	1.46
3	M	354	ASP	C-N	-5.39	1.21	1.34
1	A	308	ASP	N-CA	-5.39	1.35	1.46
2	B	382	TYR	N-CA	-5.38	1.35	1.46
1	A	216	SER	C-N	5.38	1.46	1.34
1	A	83	ASP	CA-C	-5.37	1.39	1.52
4	S	143	GLU	C-N	-5.36	1.21	1.34
3	M	262	THR	N-CA	-5.34	1.35	1.46
1	A	224	GLU	CD-OE2	-5.33	1.19	1.25
1	A	392	MET	N-CA	-5.33	1.35	1.46
2	B	83	PHE	C-N	5.33	1.46	1.34
1	A	623	MET	CA-C	-5.33	1.39	1.52
3	M	223	HIS	C-N	-5.33	1.21	1.34
1	A	404	GLN	N-CA	-5.33	1.35	1.46
2	B	290	SER	CA-C	-5.32	1.39	1.52
2	B	383	VAL	CA-C	-5.32	1.39	1.52
1	A	192	TYR	CA-C	-5.32	1.39	1.52
1	A	535	ILE	N-CA	-5.30	1.35	1.46
2	B	190	GLU	CD-OE1	-5.30	1.19	1.25
2	B	500	GLN	C-N	-5.30	1.21	1.34
1	A	138	ASN	C-N	-5.29	1.21	1.34
2	B	331	PRO	N-CA	-5.28	1.38	1.47
2	B	425	PRO	CA-C	-5.28	1.42	1.52
1	A	464	ILE	C-N	-5.27	1.22	1.34
2	B	75	ASP	C-N	-5.27	1.22	1.34
2	B	219	TYR	CA-C	-5.26	1.39	1.52
2	B	407	ASN	N-CA	-5.25	1.35	1.46
2	B	443	SER	CA-C	-5.25	1.39	1.52
2	B	35	TYR	CA-C	-5.25	1.39	1.52
2	B	226	LEU	CA-C	-5.24	1.39	1.52

Continued on next page...

Continued from previous page...

Mol	Chain	Res	Type	Atoms	Z	Observed(Å)	Ideal(Å)
2	B	273	SER	N-CA	-5.24	1.35	1.46
1	A	388	VAL	CA-C	-5.23	1.39	1.52
2	B	133	GLU	CD-OE2	-5.22	1.20	1.25
2	B	384	PHE	N-CA	-5.21	1.35	1.46
3	M	444	ALA	CA-CB	5.21	1.63	1.52
2	B	424	PHE	N-CA	-5.21	1.35	1.46
2	B	109	ALA	CA-CB	-5.20	1.41	1.52
3	M	256	VAL	C-O	5.20	1.33	1.23
4	S	159	ALA	CA-CB	5.18	1.63	1.52
2	B	190	GLU	CD-OE2	-5.17	1.20	1.25
1	A	120	ILE	CA-C	-5.17	1.39	1.52
3	M	253	ASN	N-CA	-5.17	1.36	1.46
1	A	445	ASN	CA-C	-5.17	1.39	1.52
3	M	374	TYR	N-CA	-5.15	1.36	1.46
3	M	365	GLU	N-CA	-5.13	1.36	1.46
2	B	573	GLU	CD-OE1	-5.13	1.20	1.25
3	M	476	THR	C-N	-5.13	1.23	1.33
1	A	399	ASP	N-CA	-5.12	1.36	1.46
1	A	507	GLN	C-N	-5.11	1.22	1.34
4	S	29	LYS	CA-C	-5.11	1.39	1.52
3	M	41	LEU	C-N	5.10	1.45	1.34
1	A	224	GLU	CD-OE1	-5.10	1.20	1.25
3	M	228	LYS	C-N	-5.10	1.22	1.34
2	B	289	PRO	C-N	-5.10	1.22	1.34
3	M	238	GLY	CA-C	-5.10	1.43	1.51
2	B	500	GLN	CA-C	-5.09	1.39	1.52
3	M	373	ALA	C-N	-5.08	1.22	1.34
2	B	515	PHE	CA-C	5.06	1.66	1.52
3	M	227	GLU	CD-OE2	-5.06	1.20	1.25
1	A	85	ALA	CA-CB	-5.05	1.41	1.52
1	A	302	ASN	N-CA	-5.05	1.36	1.46
1	A	514	GLU	CD-OE1	-5.05	1.20	1.25
1	A	466	ASP	CA-C	-5.04	1.39	1.52
1	A	304	LEU	N-CA	-5.04	1.36	1.46
1	A	522	PHE	CA-C	-5.02	1.39	1.52
1	A	242	GLU	C-O	-5.02	1.13	1.23

All (1402) bond angle outliers are listed below:

Mol	Chain	Res	Type	Atoms	Z	Observed(°)	Ideal(°)
1	A	242	GLU	C-N-CA	22.76	178.60	121.70
1	A	265	GLN	N-CA-C	-19.23	59.09	111.00

Continued on next page...

Continued from previous page...

Mol	Chain	Res	Type	Atoms	Z	Observed(°)	Ideal(°)
1	A	80	TYR	N-CA-C	-18.37	61.39	111.00
3	M	45	SER	C-N-CA	-17.46	78.06	121.70
1	A	277	LYS	C-N-CA	17.23	164.78	121.70
3	M	354	ASP	N-CA-C	-16.54	66.34	111.00
3	M	354	ASP	C-N-CA	16.54	163.04	121.70
1	A	444	VAL	N-CA-C	-16.42	66.65	111.00
3	M	373	ALA	CB-CA-C	15.46	133.29	110.10
1	A	98	ASN	C-N-CA	15.35	160.08	121.70
2	B	584	SER	N-CA-C	15.03	151.59	111.00
1	A	304	LEU	C-N-CA	-14.99	84.22	121.70
2	B	263	PRO	C-N-CA	14.60	158.20	121.70
1	A	534	LYS	CA-C-N	14.58	149.28	117.20
2	B	290	SER	C-N-CA	-14.53	85.37	121.70
1	A	534	LYS	C-N-CA	14.19	157.16	121.70
3	M	294	ASP	N-CA-C	-13.94	73.35	111.00
2	B	600	LYS	C-N-CA	-13.51	87.92	121.70
1	A	532	LEU	C-N-CA	-13.43	88.14	121.70
1	A	80	TYR	O-C-N	-13.25	100.68	123.20
1	A	418	ILE	C-N-CA	-13.24	88.60	121.70
2	B	78	ASP	C-N-CA	-13.24	88.60	121.70
3	M	293	PRO	CA-N-CD	-12.75	93.65	111.50
1	A	98	ASN	N-CA-C	-12.73	76.62	111.00
1	A	464	ILE	C-N-CA	-12.72	89.90	121.70
4	S	168	GLY	N-CA-C	12.69	144.83	113.10
1	A	64	LEU	O-C-N	-12.60	102.54	122.70
1	A	302	ASN	N-CA-C	-12.60	76.99	111.00
1	A	260	PHE	O-C-N	-12.55	102.61	122.70
1	A	405	THR	C-N-CA	-12.50	96.04	122.30
1	A	536	MET	O-C-N	-12.30	103.02	122.70
1	A	151	SER	C-N-CA	-12.26	91.06	121.70
3	M	54	SER	N-CA-C	12.18	143.88	111.00
1	A	469	LEU	C-N-CA	-12.12	96.86	122.30
1	A	135	ASP	C-N-CA	-11.93	97.25	122.30
2	B	330	SER	N-CA-C	-11.78	79.19	111.00
1	A	233	PHE	C-N-CA	-11.76	92.31	121.70
1	A	323	CYS	C-N-CA	11.63	150.78	121.70
1	A	136	GLY	C-N-CA	-11.47	93.02	121.70
1	A	265	GLN	CA-C-N	11.46	142.42	117.20
1	A	536	MET	C-N-CA	-11.42	93.16	121.70
4	S	53	THR	C-N-CD	11.41	152.36	128.40
3	M	458	LEU	O-C-N	11.37	140.89	122.70
2	B	329	ALA	CB-CA-C	-11.32	93.12	110.10

Continued on next page...

Continued from previous page...

Mol	Chain	Res	Type	Atoms	Z	Observed(°)	Ideal(°)
2	B	404	ASN	C-N-CA	-11.29	93.48	121.70
3	M	462	LYS	N-CA-C	-11.24	80.64	111.00
1	A	465	SER	CA-C-N	-11.18	92.60	117.20
1	A	242	GLU	CA-C-N	11.16	141.76	117.20
1	A	534	LYS	N-CA-C	-11.16	80.85	111.00
3	M	46	SER	C-N-CA	-10.93	94.37	121.70
2	B	310	ILE	C-N-CA	-10.93	94.38	121.70
4	S	109	LEU	O-C-N	-10.91	105.24	122.70
1	A	416	ILE	C-N-CD	10.83	151.15	128.40
4	S	43	ASN	O-C-N	-10.74	105.51	122.70
2	B	497	LEU	O-C-N	-10.67	105.62	122.70
1	A	80	TYR	CA-C-N	10.67	137.54	116.20
4	S	163	THR	C-N-CA	10.67	148.37	121.70
4	S	54	PRO	CA-N-CD	-10.63	96.61	111.50
1	A	204	VAL	O-C-N	-10.58	105.78	122.70
1	A	586	GLU	C-N-CA	-10.53	95.37	121.70
2	B	82	TYR	C-N-CA	-10.53	95.37	121.70
1	A	244	LEU	O-C-N	-10.47	105.95	122.70
2	B	584	SER	C-N-CA	-10.44	100.38	122.30
1	A	629	LEU	C-N-CD	10.32	150.08	128.40
1	A	84	MET	C-N-CA	-10.31	95.92	121.70
4	S	164	ASP	C-N-CA	-10.30	95.94	121.70
1	A	519	LEU	C-N-CA	-10.28	100.72	122.30
1	A	621	LEU	C-N-CD	10.16	149.74	128.40
2	B	571	SER	N-CA-C	-10.09	83.77	111.00
1	A	569	ASP	C-N-CA	-10.04	96.61	121.70
3	M	83	SER	C-N-CA	10.02	146.75	121.70
2	B	289	PRO	C-N-CA	-9.94	96.84	121.70
2	B	162	VAL	C-N-CA	-9.91	96.93	121.70
1	A	84	MET	O-C-N	-9.90	106.86	122.70
1	A	320	HIS	O-C-N	-9.90	106.86	122.70
1	A	380	ASP	N-CA-C	-9.90	84.27	111.00
2	B	230	PHE	C-N-CA	-9.86	97.05	121.70
2	B	570	GLY	N-CA-C	-9.73	88.77	113.10
1	A	346	THR	C-N-CA	-9.72	97.39	121.70
1	A	586	GLU	O-C-N	-9.70	107.18	122.70
2	B	576	GLN	N-CA-C	-9.66	84.92	111.00
3	M	268	GLY	C-N-CA	-9.63	97.62	121.70
1	A	504	ILE	O-C-N	-9.63	107.30	122.70
3	M	57	GLY	C-N-CA	9.58	145.66	121.70
2	B	577	ASN	C-N-CD	9.56	148.48	128.40
1	A	462	GLN	O-C-N	-9.55	107.41	122.70

Continued on next page...

Continued from previous page...

Mol	Chain	Res	Type	Atoms	Z	Observed(°)	Ideal(°)
1	A	319	LEU	O-C-N	-9.53	107.45	122.70
2	B	212	VAL	CA-C-O	9.50	140.05	120.10
2	B	584	SER	N-CA-CB	-9.47	96.30	110.50
1	A	212	ILE	C-N-CA	-9.45	98.06	121.70
1	A	305	GLU	N-CA-C	9.45	136.51	111.00
1	A	242	GLU	N-CA-C	-9.43	85.53	111.00
2	B	102	HIS	O-C-N	-9.39	107.68	122.70
1	A	569	ASP	N-CA-C	-9.37	85.69	111.00
2	B	187	ASP	C-N-CA	-9.37	98.27	121.70
1	A	403	LEU	C-N-CA	-9.36	98.30	121.70
3	M	252	ASP	N-CA-C	-9.34	85.78	111.00
2	B	366	LEU	O-C-N	-9.33	107.77	122.70
2	B	577	ASN	N-CA-C	9.33	136.19	111.00
1	A	282	MET	O-C-N	-9.32	107.78	122.70
1	A	381	GLU	C-N-CA	-9.30	98.44	121.70
2	B	535	GLN	C-N-CA	-9.29	98.48	121.70
4	S	143	GLU	N-CA-C	-9.29	85.93	111.00
1	A	80	TYR	C-N-CA	9.28	141.79	122.30
2	B	560	ILE	C-N-CA	-9.24	98.59	121.70
3	M	306	LEU	O-C-N	-9.24	107.91	122.70
2	B	461	HIS	C-N-CA	-9.23	98.62	121.70
2	B	505	ASP	C-N-CA	-9.19	98.72	121.70
1	A	504	ILE	C-N-CA	-9.18	98.76	121.70
1	A	110	ALA	C-N-CA	-9.16	98.79	121.70
2	B	205	PRO	C-N-CA	-9.13	98.89	121.70
1	A	275	LEU	C-N-CD	9.12	147.55	128.40
1	A	365	VAL	C-N-CA	-9.11	98.92	121.70
1	A	163	ALA	C-N-CA	-9.08	99.00	121.70
1	A	534	LYS	CA-C-O	-9.07	101.04	120.10
3	M	41	LEU	O-C-N	9.07	137.21	122.70
2	B	211	ALA	O-C-N	9.05	137.18	122.70
1	A	465	SER	C-N-CA	9.01	144.21	121.70
1	A	103	LYS	O-C-N	-8.99	108.31	122.70
3	M	80	THR	N-CA-C	8.99	135.26	111.00
2	B	568	VAL	CA-C-N	-8.98	97.44	117.20
2	B	497	LEU	C-N-CA	-8.98	99.25	121.70
1	A	539	ASN	O-C-N	-8.88	108.49	122.70
1	A	242	GLU	O-C-N	-8.87	108.50	122.70
1	A	86	TRP	C-N-CA	-8.86	99.55	121.70
1	A	100	LEU	C-N-CA	-8.85	99.59	121.70
2	B	569	THR	N-CA-C	8.83	134.84	111.00
3	M	477	GLY	C-N-CA	-8.83	99.62	121.70

Continued on next page...

Continued from previous page...

Mol	Chain	Res	Type	Atoms	Z	Observed(°)	Ideal(°)
1	A	196	LEU	C-N-CA	-8.82	99.64	121.70
1	A	240	LEU	C-N-CA	-8.82	99.64	121.70
1	A	529	GLY	CA-C-O	-8.81	104.74	120.60
1	A	461	CYS	O-C-N	-8.81	108.61	122.70
2	B	559	ASP	CA-C-O	-8.80	101.62	120.10
1	A	350	SER	O-C-N	-8.79	108.64	122.70
2	B	212	VAL	O-C-N	-8.78	108.65	122.70
2	B	404	ASN	O-C-N	-8.78	108.66	122.70
1	A	545	HIS	C-N-CA	-8.77	99.79	121.70
4	S	104	THR	O-C-N	8.72	136.65	122.70
1	A	88	ASN	C-N-CA	-8.71	99.92	121.70
2	B	557	SER	O-C-N	-8.71	108.77	122.70
4	S	167	ILE	C-N-CA	-8.68	104.08	122.30
2	B	523	PHE	O-C-N	-8.62	108.91	122.70
1	A	154	ILE	N-CA-C	8.61	134.25	111.00
4	S	98	ILE	O-C-N	8.61	136.47	122.70
2	B	109	ALA	C-N-CA	-8.60	100.21	121.70
1	A	325	SER	N-CA-C	-8.56	87.87	111.00
2	B	223	LEU	C-N-CA	-8.53	100.39	121.70
3	M	92	PHE	O-C-N	8.52	136.34	122.70
1	A	413	SER	N-CA-C	-8.52	88.01	111.00
3	M	367	ALA	C-N-CA	8.51	142.97	121.70
4	S	81	ALA	CB-CA-C	8.51	122.86	110.10
4	S	100	ASP	O-C-N	8.51	136.31	122.70
1	A	233	PHE	O-C-N	-8.49	109.11	122.70
3	M	420	THR	C-N-CA	-8.48	104.49	122.30
1	A	367	ILE	O-C-N	8.47	136.25	122.70
2	B	339	PHE	O-C-N	8.44	136.21	122.70
1	A	461	CYS	CA-C-O	8.44	137.83	120.10
3	M	292	PRO	C-N-CA	8.44	157.45	122.00
1	A	432	ILE	O-C-N	8.43	136.19	122.70
1	A	328	PRO	C-N-CA	-8.43	100.64	121.70
1	A	281	LEU	CA-C-O	8.42	137.79	120.10
1	A	391	LEU	C-N-CA	-8.42	100.66	121.70
1	A	508	LEU	N-CA-C	8.42	133.72	111.00
2	B	147	MET	O-C-N	-8.41	109.24	122.70
2	B	146	LYS	C-N-CA	-8.41	100.67	121.70
1	A	450	TYR	O-C-N	8.39	136.13	122.70
4	S	103	GLN	O-C-N	8.38	136.10	122.70
2	B	574	ASN	C-N-CA	-8.36	100.79	121.70
1	A	215	VAL	CA-C-O	8.36	137.65	120.10
2	B	472	VAL	O-C-N	-8.33	109.38	122.70

Continued on next page...

Continued from previous page...

Mol	Chain	Res	Type	Atoms	Z	Observed(°)	Ideal(°)
1	A	120	ILE	O-C-N	8.32	136.02	122.70
4	S	46	PHE	CA-C-O	-8.31	102.64	120.10
1	A	424	TYR	O-C-N	8.31	135.99	122.70
1	A	94	VAL	O-C-N	-8.31	109.41	122.70
3	M	457	GLY	N-CA-C	-8.31	92.33	113.10
4	S	161	GLU	O-C-N	-8.30	109.42	122.70
2	B	108	PHE	O-C-N	-8.30	109.42	122.70
1	A	270	LEU	O-C-N	8.27	135.93	122.70
3	M	68	VAL	N-CA-C	8.27	133.34	111.00
3	M	335	SER	O-C-N	8.27	135.94	122.70
2	B	325	LEU	O-C-N	-8.27	109.47	122.70
3	M	97	ASP	O-C-N	8.27	135.92	122.70
2	B	444	THR	O-C-N	-8.26	109.48	122.70
2	B	366	LEU	C-N-CA	-8.25	101.08	121.70
1	A	275	LEU	C-N-CA	-8.24	87.37	122.00
1	A	534	LYS	O-C-N	-8.24	109.52	122.70
2	B	56	SER	O-C-N	-8.24	109.52	122.70
3	M	54	SER	O-C-N	8.22	135.86	122.70
1	A	573	GLU	O-C-N	-8.22	109.55	122.70
2	B	205	PRO	O-C-N	-8.19	109.59	122.70
3	M	330	GLY	C-N-CA	8.19	142.17	121.70
1	A	192	TYR	CA-C-O	-8.19	102.91	120.10
3	M	91	THR	CA-C-O	-8.17	102.94	120.10
1	A	631	SER	O-C-N	8.16	135.76	122.70
4	S	158	LYS	O-C-N	8.16	135.76	122.70
1	A	443	SER	N-CA-C	8.15	133.01	111.00
2	B	83	PHE	O-C-N	8.14	135.72	122.70
4	S	46	PHE	N-CA-C	-8.13	89.05	111.00
1	A	431	VAL	O-C-N	8.12	135.70	122.70
2	B	105	LEU	O-C-N	-8.12	109.71	122.70
2	B	505	ASP	O-C-N	-8.12	109.71	122.70
2	B	132	SER	C-N-CA	-8.11	101.42	121.70
1	A	415	ARG	N-CA-C	-8.11	89.10	111.00
1	A	487	MET	O-C-N	-8.11	109.72	122.70
1	A	100	LEU	O-C-N	-8.11	109.73	122.70
1	A	637	GLU	C-N-CA	-8.10	101.44	121.70
1	A	103	LYS	CA-C-O	8.09	137.10	120.10
1	A	434	SER	O-C-N	8.09	135.65	122.70
3	M	74	TYR	CA-C-O	8.08	137.07	120.10
2	B	132	SER	O-C-N	-8.08	109.77	122.70
4	S	74	GLN	O-C-N	-8.07	109.79	122.70
1	A	138	ASN	N-CA-C	-8.05	89.26	111.00

Continued on next page...

Continued from previous page...

Mol	Chain	Res	Type	Atoms	Z	Observed(°)	Ideal(°)
2	B	142	LEU	O-C-N	-8.04	109.84	122.70
2	B	115	LEU	O-C-N	8.02	135.53	122.70
2	B	375	LEU	O-C-N	-8.02	105.86	121.10
2	B	515	PHE	C-N-CA	-8.02	105.45	122.30
2	B	296	ASP	CA-C-O	-8.01	103.28	120.10
1	A	547	VAL	O-C-N	8.01	135.51	122.70
2	B	273	SER	N-CA-C	8.01	132.62	111.00
1	A	220	SER	O-C-N	7.99	135.49	122.70
2	B	240	LEU	N-CA-C	7.98	132.55	111.00
2	B	35	TYR	O-C-N	7.97	135.46	122.70
3	M	55	MET	CA-C-N	7.97	134.74	117.20
3	M	294	ASP	C-N-CA	7.97	139.03	122.30
1	A	264	SER	C-N-CA	-7.96	101.79	121.70
2	B	389	ILE	O-C-N	-7.94	109.99	122.70
1	A	545	HIS	O-C-N	-7.94	110.00	122.70
2	B	108	PHE	CA-C-O	7.94	136.77	120.10
2	B	566	ALA	C-N-CA	7.94	141.54	121.70
3	M	262	THR	C-N-CA	-7.92	101.90	121.70
2	B	299	LEU	O-C-N	7.91	135.36	122.70
1	A	281	LEU	C-N-CA	-7.91	101.93	121.70
2	B	126	SER	O-C-N	-7.91	110.05	122.70
3	M	292	PRO	N-CA-C	-7.90	91.57	112.10
1	A	218	ALA	O-C-N	7.89	135.33	122.70
2	B	508	ARG	O-C-N	-7.89	110.08	122.70
2	B	325	LEU	C-N-CA	-7.88	101.99	121.70
1	A	212	ILE	O-C-N	-7.86	110.13	122.70
1	A	281	LEU	O-C-N	-7.86	110.13	122.70
1	A	448	GLU	N-CA-C	-7.84	89.82	111.00
2	B	261	PRO	CA-N-CD	-7.84	100.52	111.50
1	A	365	VAL	O-C-N	-7.84	110.16	122.70
1	A	158	LEU	O-C-N	7.84	135.24	122.70
4	S	108	SER	O-C-N	7.84	135.24	122.70
2	B	153	ILE	O-C-N	7.83	135.23	122.70
3	M	72	LEU	C-N-CA	7.82	141.24	121.70
2	B	317	VAL	O-C-N	7.80	135.18	122.70
1	A	70	ALA	O-C-N	7.80	135.18	122.70
2	B	172	GLU	O-C-N	-7.80	110.22	122.70
3	M	263	MET	C-N-CA	7.80	138.68	122.30
1	A	298	ILE	O-C-N	-7.78	110.25	122.70
4	S	132	LEU	O-C-N	7.77	135.13	122.70
2	B	418	TYR	C-N-CA	-7.76	102.29	121.70
2	B	528	ASP	O-C-N	7.76	135.12	122.70

Continued on next page...

Continued from previous page...

Mol	Chain	Res	Type	Atoms	Z	Observed(°)	Ideal(°)
1	A	588	LEU	O-C-N	-7.75	110.29	122.70
1	A	218	ALA	CB-CA-C	7.75	121.72	110.10
2	B	147	MET	C-N-CA	-7.74	102.34	121.70
3	M	237	THR	C-N-CA	-7.74	106.05	122.30
1	A	328	PRO	O-C-N	-7.71	110.36	122.70
1	A	330	LEU	O-C-N	7.71	135.04	122.70
1	A	394	GLN	CA-C-O	-7.71	103.92	120.10
4	S	109	LEU	CA-C-O	7.70	136.28	120.10
2	B	478	LEU	O-C-N	-7.70	110.39	122.70
3	M	132	GLY	CA-C-O	7.69	134.45	120.60
1	A	461	CYS	C-N-CA	-7.69	102.47	121.70
2	B	584	SER	O-C-N	-7.68	110.14	123.20
1	A	304	LEU	O-C-N	-7.68	110.42	122.70
2	B	174	ALA	O-C-N	-7.68	110.42	122.70
1	A	346	THR	O-C-N	-7.67	110.43	122.70
2	B	560	ILE	O-C-N	-7.66	110.44	122.70
1	A	527	GLU	C-N-CA	7.66	140.84	121.70
2	B	237	ILE	O-C-N	-7.65	110.46	122.70
1	A	559	PHE	O-C-N	7.65	134.93	122.70
4	S	36	TYR	O-C-N	7.64	134.93	122.70
3	M	232	HIS	CA-C-O	7.64	136.15	120.10
1	A	156	PRO	O-C-N	-7.63	110.49	122.70
2	B	220	ALA	N-CA-C	-7.63	90.40	111.00
1	A	601	VAL	O-C-N	-7.62	110.50	122.70
4	S	139	GLY	C-N-CA	-7.62	102.64	121.70
1	A	469	LEU	CA-C-O	7.61	136.07	120.10
2	B	51	LEU	C-N-CA	-7.61	102.68	121.70
4	S	28	GLN	O-C-N	7.60	134.86	122.70
1	A	601	VAL	C-N-CA	-7.59	102.72	121.70
2	B	486	HIS	O-C-N	7.59	134.85	122.70
2	B	585	GLY	N-CA-C	-7.58	94.14	113.10
4	S	27	LYS	O-C-N	7.57	134.81	122.70
1	A	279	LEU	O-C-N	-7.57	110.60	122.70
1	A	504	ILE	CA-C-O	7.55	135.96	120.10
1	A	465	SER	N-CA-C	7.55	131.38	111.00
1	A	98	ASN	CA-C-N	7.54	133.80	117.20
2	B	237	ILE	C-N-CA	-7.54	102.86	121.70
1	A	528	ASN	CA-C-O	7.54	135.93	120.10
3	M	265	ASN	N-CA-C	-7.53	90.66	111.00
2	B	488	ARG	O-C-N	7.53	134.75	122.70
1	A	88	ASN	CA-C-O	7.53	135.91	120.10
2	B	277	CYS	C-N-CD	7.53	144.20	128.40

Continued on next page...

Continued from previous page...

Mol	Chain	Res	Type	Atoms	Z	Observed(°)	Ideal(°)
1	A	338	PHE	O-C-N	7.52	134.73	122.70
3	M	230	LYS	CA-C-N	7.51	133.73	117.20
1	A	596	VAL	O-C-N	7.50	134.70	122.70
2	B	46	GLN	O-C-N	7.50	134.70	122.70
4	S	55	PRO	CA-N-CD	-7.50	101.00	111.50
2	B	416	LYS	O-C-N	7.48	134.66	122.70
2	B	215	TYR	O-C-N	7.47	134.65	122.70
1	A	394	GLN	O-C-N	7.46	134.64	122.70
2	B	442	LEU	C-N-CA	-7.45	103.07	121.70
3	M	266	ASP	CA-C-N	-7.44	100.83	117.20
3	M	367	ALA	CB-CA-C	7.44	121.26	110.10
4	S	96	LEU	O-C-N	7.43	134.59	122.70
2	B	325	LEU	CA-C-O	7.43	135.70	120.10
1	A	465	SER	O-C-N	7.42	134.58	122.70
2	B	328	LEU	C-N-CA	-7.42	103.14	121.70
1	A	606	PHE	O-C-N	7.42	134.57	122.70
2	B	494	ALA	O-C-N	7.42	134.56	122.70
1	A	519	LEU	O-C-N	-7.41	110.61	123.20
2	B	428	VAL	O-C-N	7.41	134.55	122.70
2	B	362	ALA	O-C-N	7.37	134.49	122.70
1	A	311	THR	O-C-N	7.37	134.49	122.70
3	M	407	THR	N-CA-C	-7.36	91.12	111.00
1	A	391	LEU	O-C-N	-7.35	110.94	122.70
2	B	142	LEU	C-N-CA	-7.34	103.35	121.70
3	M	421	GLY	O-C-N	-7.34	107.16	121.10
4	S	54	PRO	C-N-CD	7.33	143.79	128.40
1	A	405	THR	N-CA-C	7.33	130.78	111.00
2	B	493	LEU	O-C-N	7.32	134.42	122.70
1	A	177	ILE	O-C-N	7.32	134.41	122.70
1	A	256	LEU	O-C-N	7.32	134.41	122.70
4	S	31	LEU	O-C-N	7.31	134.40	122.70
2	B	223	LEU	O-C-N	-7.30	111.01	122.70
4	S	149	ILE	O-C-N	7.30	134.39	122.70
2	B	192	LEU	O-C-N	7.30	134.38	122.70
2	B	288	TYR	N-CA-C	-7.30	91.29	111.00
1	A	260	PHE	C-N-CA	-7.30	103.46	121.70
1	A	162	ILE	O-C-N	7.29	134.37	122.70
2	B	515	PHE	O-C-N	-7.29	110.80	123.20
4	S	167	ILE	N-CA-C	7.29	130.68	111.00
1	A	511	VAL	O-C-N	7.29	134.36	122.70
4	S	80	TYR	CA-C-O	7.28	135.40	120.10
2	B	408	VAL	O-C-N	7.27	134.34	122.70

Continued on next page...

Continued from previous page...

Mol	Chain	Res	Type	Atoms	Z	Observed(°)	Ideal(°)
2	B	220	ALA	CB-CA-C	7.27	121.00	110.10
2	B	270	SER	N-CA-C	-7.27	91.37	111.00
2	B	531	ARG	O-C-N	7.27	134.33	122.70
1	A	399	ASP	O-C-N	7.26	134.32	122.70
4	S	127	THR	O-C-N	7.26	134.32	122.70
1	A	384	LEU	O-C-N	7.26	134.31	122.70
2	B	267	ASP	O-C-N	-7.26	111.09	122.70
2	B	554	LYS	O-C-N	7.25	134.30	122.70
3	M	426	LYS	C-N-CA	7.25	139.82	121.70
3	M	460	ILE	C-N-CA	7.23	137.49	122.30
1	A	573	GLU	C-N-CA	-7.23	103.64	121.70
1	A	529	GLY	N-CA-C	-7.21	95.08	113.10
1	A	265	GLN	CA-C-O	-7.17	105.04	120.10
4	S	83	LEU	CA-C-O	7.17	135.16	120.10
1	A	306	GLU	O-C-N	-7.17	111.23	122.70
4	S	125	TRP	CA-C-O	-7.16	105.06	120.10
3	M	54	SER	C-N-CA	7.16	139.60	121.70
1	A	298	ILE	CA-C-O	7.16	135.14	120.10
2	B	150	LEU	O-C-N	7.15	134.14	122.70
2	B	243	TRP	O-C-N	7.15	134.14	122.70
1	A	441	TYR	CA-C-N	-7.15	101.48	117.20
2	B	478	LEU	CA-C-O	7.14	135.10	120.10
1	A	300	LYS	N-CA-C	7.14	130.28	111.00
2	B	415	LEU	O-C-N	7.14	134.12	122.70
1	A	263	LEU	C-N-CA	-7.13	103.87	121.70
3	M	96	ILE	O-C-N	7.13	134.10	122.70
1	A	388	VAL	O-C-N	7.12	134.09	122.70
1	A	447	PHE	O-C-N	7.12	134.09	122.70
3	M	118	TYR	CA-C-O	-7.11	105.17	120.10
1	A	517	TRP	O-C-N	7.11	134.08	122.70
1	A	488	ARG	O-C-N	7.11	134.07	122.70
1	A	355	LEU	O-C-N	7.10	134.06	122.70
2	B	23	ALA	N-CA-CB	7.09	120.03	110.10
1	A	582	ILE	O-C-N	7.09	134.04	122.70
1	A	381	GLU	O-C-N	-7.08	111.36	122.70
4	S	84	TYR	O-C-N	-7.08	111.37	122.70
2	B	611	ALA	O-C-N	7.08	134.02	122.70
1	A	539	ASN	C-N-CA	-7.08	104.01	121.70
2	B	226	LEU	O-C-N	7.08	134.02	122.70
1	A	453	VAL	O-C-N	7.07	134.01	122.70
2	B	521	ILE	C-N-CA	-7.07	104.03	121.70
2	B	213	LEU	O-C-N	7.05	133.99	122.70

Continued on next page...

Continued from previous page...

Mol	Chain	Res	Type	Atoms	Z	Observed(°)	Ideal(°)
4	S	99	LEU	CA-C-O	-7.05	105.29	120.10
3	M	110	SER	O-C-N	7.05	133.98	122.70
2	B	555	LEU	O-C-N	7.05	133.97	122.70
1	A	566	PHE	N-CA-C	7.04	130.02	111.00
2	B	504	ALA	O-C-N	7.04	133.97	122.70
2	B	526	CYS	O-C-N	7.04	134.48	121.10
1	A	166	LEU	O-C-N	7.03	133.95	122.70
1	A	441	TYR	CA-C-O	7.03	134.86	120.10
1	A	302	ASN	CA-C-N	-7.03	101.74	117.20
4	S	30	LEU	O-C-N	7.03	133.94	122.70
1	A	442	SER	N-CA-C	-7.02	92.04	111.00
1	A	225	LEU	CA-C-O	-7.02	105.36	120.10
1	A	635	ALA	O-C-N	7.02	133.93	122.70
2	B	389	ILE	C-N-CA	-7.01	104.17	121.70
2	B	143	SER	C-N-CA	-7.01	104.17	121.70
3	M	458	LEU	C-N-CA	7.00	139.21	121.70
3	M	425	THR	C-N-CA	7.00	139.19	121.70
1	A	271	ARG	O-C-N	6.99	133.88	122.70
2	B	97	VAL	O-C-N	6.99	133.88	122.70
1	A	225	LEU	O-C-N	6.99	133.88	122.70
2	B	422	ALA	N-CA-CB	6.99	119.88	110.10
1	A	301	GLY	N-CA-C	-6.98	95.64	113.10
2	B	572	GLU	O-C-N	6.98	133.87	122.70
1	A	605	GLU	O-C-N	6.98	133.87	122.70
4	S	143	GLU	CA-C-N	-6.98	101.84	117.20
1	A	476	GLN	O-C-N	6.98	133.87	122.70
2	B	216	LYS	C-N-CA	-6.96	104.29	121.70
1	A	140	VAL	O-C-N	-6.96	111.56	122.70
1	A	257	LEU	O-C-N	6.96	133.83	122.70
2	B	56	SER	C-N-CA	-6.96	104.31	121.70
1	A	156	PRO	C-N-CA	-6.96	104.31	121.70
2	B	102	HIS	CA-C-O	6.95	134.70	120.10
2	B	355	ASN	O-C-N	6.95	133.82	122.70
1	A	138	ASN	O-C-N	6.95	133.82	122.70
2	B	324	ALA	O-C-N	6.95	133.82	122.70
2	B	425	PRO	O-C-N	6.95	133.82	122.70
2	B	104	TYR	O-C-N	6.94	133.80	122.70
2	B	559	ASP	O-C-N	6.93	133.79	122.70
2	B	252	LEU	O-C-N	6.92	133.78	122.70
1	A	204	VAL	C-N-CA	-6.92	104.40	121.70
1	A	607	LEU	O-C-N	6.91	133.75	122.70
2	B	99	ARG	O-C-N	6.90	133.74	122.70

Continued on next page...

Continued from previous page...

Mol	Chain	Res	Type	Atoms	Z	Observed(°)	Ideal(°)
1	A	240	LEU	O-C-N	-6.90	111.66	122.70
1	A	348	PHE	O-C-N	6.90	133.73	122.70
2	B	271	GLU	C-N-CA	-6.89	107.83	122.30
2	B	511	ILE	O-C-N	-6.89	111.68	122.70
1	A	383	ASN	O-C-N	6.89	133.72	122.70
1	A	621	LEU	C-N-CA	-6.88	93.09	122.00
2	B	169	VAL	O-C-N	6.88	133.72	122.70
1	A	279	LEU	C-N-CA	-6.88	104.50	121.70
1	A	457	LEU	O-C-N	6.88	133.71	122.70
1	A	608	ARG	O-C-N	6.88	133.70	122.70
4	S	18	LYS	O-C-N	-6.88	111.69	122.70
2	B	573	GLU	C-N-CA	-6.87	104.52	121.70
1	A	632	PHE	O-C-N	6.87	133.69	122.70
2	B	323	ASN	O-C-N	6.87	133.69	122.70
4	S	157	ASN	O-C-N	6.87	133.69	122.70
2	B	134	LEU	O-C-N	6.86	133.68	122.70
4	S	99	LEU	O-C-N	6.86	133.67	122.70
1	A	140	VAL	CA-C-O	6.86	134.50	120.10
2	B	105	LEU	C-N-CA	-6.85	104.58	121.70
4	S	33	GLU	O-C-N	6.85	133.65	122.70
3	M	89	CYS	O-C-N	6.84	133.65	122.70
2	B	544	THR	O-C-N	6.84	133.65	122.70
1	A	102	GLN	O-C-N	6.84	133.64	122.70
1	A	602	GLU	O-C-N	6.83	133.63	122.70
1	A	555	LEU	O-C-N	6.83	133.63	122.70
2	B	63	MET	O-C-N	6.83	133.63	122.70
1	A	610	SER	O-C-N	6.82	133.61	122.70
2	B	568	VAL	C-N-CA	6.82	138.74	121.70
2	B	594	ALA	O-C-N	6.81	133.60	122.70
3	M	123	LEU	O-C-N	6.81	133.60	122.70
1	A	623	MET	O-C-N	6.81	133.60	122.70
2	B	66	ILE	O-C-N	6.81	133.60	122.70
1	A	477	PHE	O-C-N	6.81	133.59	122.70
2	B	457	HIS	CA-C-O	6.81	134.39	120.10
1	A	236	LEU	O-C-N	6.81	133.59	122.70
1	A	188	VAL	O-C-N	6.80	133.59	122.70
4	S	148	ARG	O-C-N	6.80	133.58	122.70
1	A	496	ILE	O-C-N	6.79	133.56	122.70
1	A	624	LEU	O-C-N	6.79	133.56	122.70
2	B	454	LEU	O-C-N	6.79	133.56	122.70
3	M	54	SER	CB-CA-C	-6.78	97.21	110.10
2	B	174	ALA	C-N-CA	-6.78	104.76	121.70

Continued on next page...

Continued from previous page...

Mol	Chain	Res	Type	Atoms	Z	Observed(°)	Ideal(°)
3	M	54	SER	CA-C-N	-6.78	102.29	117.20
4	S	101	LEU	O-C-N	6.78	133.54	122.70
2	B	489	ILE	O-C-N	6.78	133.54	122.70
1	A	466	ASP	O-C-N	6.77	133.54	122.70
2	B	100	LEU	O-C-N	6.76	133.52	122.70
3	M	47	SER	N-CA-C	-6.76	92.74	111.00
2	B	411	ILE	O-C-N	6.75	133.51	122.70
3	M	385	ARG	O-C-N	-6.75	111.89	122.70
4	S	16	LEU	O-C-N	-6.75	111.89	122.70
1	A	581	LEU	O-C-N	6.75	133.50	122.70
1	A	601	VAL	CA-C-O	6.75	134.28	120.10
2	B	78	ASP	O-C-N	-6.75	111.90	122.70
1	A	67	LYS	O-C-N	6.75	133.49	122.70
1	A	418	ILE	N-CA-C	6.73	129.18	111.00
2	B	253	ILE	O-C-N	6.73	133.47	122.70
1	A	125	THR	O-C-N	6.72	133.46	122.70
3	M	424	PHE	N-CA-C	-6.72	92.85	111.00
2	B	452	LYS	O-C-N	6.72	133.45	122.70
3	M	425	THR	N-CA-C	-6.72	92.86	111.00
1	A	91	ILE	O-C-N	6.71	133.44	122.70
1	A	265	GLN	N-CA-CB	6.71	122.69	110.60
4	S	128	LEU	O-C-N	6.71	133.44	122.70
2	B	566	ALA	CB-CA-C	6.71	120.17	110.10
3	M	85	GLY	N-CA-C	-6.71	96.33	113.10
2	B	592	TYR	O-C-N	6.70	133.42	122.70
1	A	432	ILE	CA-C-O	-6.69	106.05	120.10
1	A	87	CYS	O-C-N	6.69	133.40	122.70
1	A	117	ASP	O-C-N	6.69	133.40	122.70
1	A	307	ASP	N-CA-C	-6.68	92.95	111.00
3	M	265	ASN	CA-C-N	6.68	131.91	117.20
3	M	284	SER	O-C-N	-6.68	108.41	121.10
4	S	137	GLN	CA-C-N	-6.68	102.84	116.20
1	A	128	LEU	O-C-N	6.68	133.38	122.70
1	A	472	LYS	O-C-N	6.68	133.38	122.70
1	A	196	LEU	CA-C-O	6.68	134.12	120.10
1	A	569	ASP	O-C-N	-6.68	112.02	122.70
3	M	113	LYS	O-C-N	6.68	133.38	122.70
2	B	356	LYS	O-C-N	6.67	133.38	122.70
2	B	154	ILE	CA-C-O	-6.67	106.09	120.10
3	M	16	PHE	CA-C-O	6.67	134.10	120.10
2	B	139	LEU	O-C-N	6.67	133.37	122.70
4	S	150	VAL	O-C-N	6.67	133.36	122.70

Continued on next page...

Continued from previous page...

Mol	Chain	Res	Type	Atoms	Z	Observed(°)	Ideal(°)
2	B	361	GLN	O-C-N	6.66	133.36	122.70
1	A	392	MET	O-C-N	6.66	133.35	122.70
2	B	460	SER	N-CA-C	6.66	128.97	111.00
2	B	607	ILE	O-C-N	6.66	133.35	122.70
4	S	54	PRO	N-CA-C	-6.66	94.79	112.10
2	B	244	SER	O-C-N	6.65	133.34	122.70
2	B	589	SER	O-C-N	6.65	133.34	122.70
2	B	337	THR	CA-C-O	-6.65	106.14	120.10
2	B	399	LEU	C-N-CA	-6.65	105.08	121.70
2	B	412	PHE	O-C-N	6.65	133.34	122.70
1	A	107	TYR	O-C-N	6.64	133.33	122.70
2	B	273	SER	N-CA-CB	-6.64	100.53	110.50
1	A	265	GLN	O-C-N	-6.64	112.07	122.70
2	B	65	ARG	O-C-N	6.64	133.32	122.70
1	A	522	PHE	O-C-N	6.63	133.31	122.70
1	A	600	SER	O-C-N	6.63	133.31	122.70
2	B	447	GLU	O-C-N	6.63	133.31	122.70
1	A	462	GLN	C-N-CA	-6.63	105.13	121.70
1	A	296	ASN	O-C-N	6.63	133.30	122.70
3	M	394	GLN	CA-C-O	6.63	134.02	120.10
2	B	552	SER	O-C-N	6.62	133.30	122.70
4	S	106	VAL	O-C-N	6.62	133.30	122.70
2	B	521	ILE	N-CA-C	-6.62	93.12	111.00
1	A	515	CYS	O-C-N	6.62	133.29	122.70
1	A	556	VAL	O-C-N	6.60	133.26	122.70
1	A	436	CYS	O-C-N	-6.59	112.15	122.70
4	S	35	VAL	O-C-N	6.59	133.25	122.70
1	A	80	TYR	N-CA-CB	6.59	122.46	110.60
2	B	123	LEU	CA-C-O	-6.59	106.27	120.10
2	B	267	ASP	C-N-CA	-6.58	105.24	121.70
1	A	139	ASP	O-C-N	6.58	133.23	122.70
1	A	558	VAL	O-C-N	6.58	133.23	122.70
2	B	267	ASP	CA-C-O	6.58	133.93	120.10
2	B	591	MET	O-C-N	6.58	133.23	122.70
1	A	554	ALA	O-C-N	6.58	133.23	122.70
1	A	611	LEU	O-C-N	6.58	133.23	122.70
4	S	32	LEU	O-C-N	6.58	133.23	122.70
2	B	123	LEU	O-C-N	6.58	133.22	122.70
2	B	251	LEU	O-C-N	6.58	133.22	122.70
1	A	495	ILE	O-C-N	6.57	133.21	122.70
1	A	332	TYR	O-C-N	6.57	133.21	122.70
2	B	475	ILE	O-C-N	6.57	133.21	122.70

Continued on next page...

Continued from previous page...

Mol	Chain	Res	Type	Atoms	Z	Observed(°)	Ideal(°)
1	A	406	GLY	N-CA-C	-6.57	96.69	113.10
2	B	62	ALA	O-C-N	6.57	133.21	122.70
2	B	474	VAL	O-C-N	6.57	133.20	122.70
1	A	454	ILE	O-C-N	6.56	133.19	122.70
4	S	29	LYS	O-C-N	6.56	133.19	122.70
2	B	359	LEU	O-C-N	6.55	133.19	122.70
1	A	507	GLN	C-N-CA	-6.55	105.33	121.70
2	B	513	TRP	O-C-N	6.54	133.17	122.70
2	B	508	ARG	CA-C-O	6.54	133.83	120.10
1	A	562	TRP	O-C-N	6.54	133.16	122.70
1	A	145	ILE	O-C-N	6.53	133.15	122.70
1	A	451	ASN	O-C-N	6.53	133.15	122.70
2	B	346	THR	O-C-N	6.53	133.15	122.70
1	A	386	ALA	O-C-N	6.53	133.15	122.70
2	B	604	GLU	O-C-N	6.53	133.15	122.70
2	B	301	LEU	O-C-N	6.53	133.14	122.70
1	A	492	ILE	O-C-N	6.52	133.14	122.70
2	B	588	ILE	O-C-N	6.52	133.13	122.70
2	B	453	TRP	O-C-N	6.51	133.12	122.70
3	M	458	LEU	N-CA-C	-6.51	93.42	111.00
3	M	394	GLN	O-C-N	-6.51	112.14	123.20
2	B	467	VAL	O-C-N	6.51	133.11	122.70
2	B	154	ILE	O-C-N	6.50	133.11	122.70
4	S	129	GLU	O-C-N	6.50	133.10	122.70
2	B	388	PRO	C-N-CA	6.50	137.94	121.70
2	B	177	ILE	O-C-N	6.50	133.09	122.70
1	A	612	GLU	O-C-N	6.50	133.09	122.70
2	B	344	VAL	O-C-N	6.49	133.09	122.70
4	S	83	LEU	O-C-N	-6.49	112.31	122.70
2	B	137	PHE	O-C-N	6.49	133.09	122.70
2	B	146	LYS	CA-C-N	6.49	131.48	117.20
4	S	4	ALA	CA-C-O	6.49	133.73	120.10
2	B	507	ALA	O-C-N	6.49	133.08	122.70
1	A	569	ASP	CA-C-O	-6.49	106.48	120.10
4	S	124	ASN	O-C-N	6.49	133.08	122.70
2	B	119	SER	O-C-N	6.48	133.07	122.70
3	M	91	THR	O-C-N	6.48	133.07	122.70
3	M	124	ILE	O-C-N	6.48	133.07	122.70
4	S	106	VAL	CA-C-O	-6.48	106.49	120.10
4	S	130	SER	O-C-N	6.48	133.07	122.70
2	B	548	ILE	O-C-N	6.48	133.06	122.70
3	M	122	SER	O-C-N	6.48	133.06	122.70

Continued on next page...

Continued from previous page...

Mol	Chain	Res	Type	Atoms	Z	Observed(°)	Ideal(°)
2	B	145	MET	C-N-CA	-6.46	105.56	121.70
2	B	230	PHE	O-C-N	-6.46	112.37	122.70
1	A	146	ALA	O-C-N	6.45	133.02	122.70
4	S	145	ASN	O-C-N	6.45	133.02	122.70
1	A	105	VAL	O-C-N	6.45	134.16	123.20
3	M	94	GLU	O-C-N	6.45	133.01	122.70
3	M	389	SER	O-C-N	6.44	133.01	122.70
1	A	388	VAL	CA-C-O	-6.44	106.57	120.10
2	B	229	HIS	C-N-CA	6.44	137.80	121.70
1	A	64	LEU	CA-C-O	6.44	133.62	120.10
1	A	185	LEU	O-C-N	6.43	132.99	122.70
2	B	81	LEU	C-N-CA	-6.43	105.62	121.70
2	B	363	ILE	O-C-N	6.43	132.99	122.70
2	B	587	ARG	O-C-N	6.42	132.96	122.70
3	M	457	GLY	CA-C-N	-6.41	103.10	117.20
2	B	490	ILE	O-C-N	6.41	132.95	122.70
2	B	306	LEU	CA-C-O	-6.40	106.65	120.10
1	A	252	ILE	O-C-N	6.40	132.94	122.70
1	A	313	MET	O-C-N	6.40	132.94	122.70
1	A	550	VAL	O-C-N	6.40	132.94	122.70
2	B	414	GLU	O-C-N	6.40	132.93	122.70
1	A	159	ALA	O-C-N	6.39	132.93	122.70
1	A	407	SER	N-CA-C	-6.39	93.74	111.00
1	A	467	LYS	O-C-N	6.39	132.92	122.70
2	B	135	ARG	O-C-N	6.39	132.92	122.70
1	A	331	ARG	O-C-N	6.39	132.92	122.70
1	A	364	ASP	O-C-N	6.38	132.92	122.70
2	B	193	LEU	O-C-N	6.38	132.92	122.70
1	A	492	ILE	CA-C-O	-6.38	106.70	120.10
1	A	516	ILE	O-C-N	6.38	132.91	122.70
4	S	155	GLU	O-C-N	6.38	132.91	122.70
1	A	598	GLU	O-C-N	6.38	132.90	122.70
1	A	629	LEU	C-N-CA	-6.38	95.22	122.00
2	B	102	HIS	C-N-CA	-6.38	105.76	121.70
1	A	415	ARG	C-N-CA	-6.37	105.78	121.70
1	A	552	ILE	O-C-N	6.37	132.89	122.70
1	A	215	VAL	O-C-N	-6.37	112.52	122.70
2	B	178	ILE	O-C-N	6.36	132.88	122.70
2	B	271	GLU	N-CA-C	6.36	128.18	111.00
4	S	165	SER	C-N-CA	-6.36	105.79	121.70
2	B	170	ARG	O-C-N	-6.36	112.38	123.20
1	A	474	GLY	O-C-N	6.36	132.88	122.70

Continued on next page...

Continued from previous page...

Mol	Chain	Res	Type	Atoms	Z	Observed(°)	Ideal(°)
1	A	186	PHE	O-C-N	6.36	132.87	122.70
1	A	604	LEU	O-C-N	6.36	132.87	122.70
4	S	153	VAL	O-C-N	6.36	132.87	122.70
3	M	111	ILE	O-C-N	6.35	132.87	122.70
2	B	395	LYS	O-C-N	6.35	132.86	122.70
4	S	156	LEU	O-C-N	6.35	132.87	122.70
2	B	151	ALA	O-C-N	6.35	133.17	121.10
1	A	471	SER	O-C-N	6.35	132.86	122.70
2	B	248	LEU	O-C-N	6.35	132.85	122.70
2	B	510	GLY	CA-C-O	-6.35	109.18	120.60
1	A	475	GLU	O-C-N	6.34	132.85	122.70
3	M	272	LEU	C-N-CA	6.34	137.55	121.70
2	B	79	VAL	CA-C-O	-6.34	106.79	120.10
1	A	603	VAL	O-C-N	6.33	132.84	122.70
1	A	297	CYS	O-C-N	-6.33	112.57	122.70
1	A	147	LEU	O-C-N	6.33	132.83	122.70
2	B	519	ALA	N-CA-CB	-6.33	101.24	110.10
2	B	67	ILE	O-C-N	6.33	132.82	122.70
2	B	417	TYR	O-C-N	6.33	132.82	122.70
2	B	432	ALA	O-C-N	6.33	132.82	122.70
1	A	628	VAL	O-C-N	6.32	132.82	122.70
1	A	387	ILE	O-C-N	6.32	132.81	122.70
4	S	37	GLU	O-C-N	6.32	132.81	122.70
2	B	150	LEU	CA-C-O	-6.32	106.83	120.10
2	B	204	ASP	O-C-N	6.32	133.10	121.10
1	A	202	LYS	O-C-N	6.31	132.80	122.70
2	B	59	VAL	O-C-N	6.31	132.80	122.70
2	B	455	ILE	O-C-N	6.31	132.80	122.70
2	B	158	VAL	CA-C-O	-6.31	106.85	120.10
2	B	305	SER	O-C-N	6.31	132.79	122.70
2	B	306	LEU	O-C-N	6.31	132.79	122.70
2	B	612	ARG	O-C-N	6.31	132.80	122.70
1	A	578	LEU	O-C-N	6.31	132.79	122.70
2	B	209	SER	O-C-N	6.31	132.79	122.70
2	B	341	GLU	O-C-N	6.30	132.79	122.70
2	B	302	PHE	O-C-N	6.30	132.78	122.70
3	M	86	PRO	O-C-N	6.30	132.78	122.70
1	A	182	ILE	O-C-N	6.30	132.78	122.70
2	B	429	VAL	O-C-N	6.30	132.78	122.70
2	B	610	ARG	O-C-N	6.30	132.78	122.70
4	S	38	LEU	O-C-N	6.30	132.78	122.70
4	S	80	TYR	O-C-N	-6.30	112.62	122.70

Continued on next page...

Continued from previous page...

Mol	Chain	Res	Type	Atoms	Z	Observed(°)	Ideal(°)
1	A	429	VAL	O-C-N	6.30	132.77	122.70
1	A	508	LEU	CA-C-O	-6.30	106.88	120.10
2	B	613	MET	O-C-N	6.30	132.77	122.70
4	S	105	PHE	O-C-N	6.30	132.77	122.70
1	A	574	ILE	O-C-N	6.29	132.77	122.70
2	B	111	ASN	N-CA-C	6.29	127.99	111.00
1	A	337	LEU	O-C-N	6.29	132.76	122.70
1	A	613	ALA	O-C-N	6.29	132.76	122.70
1	A	421	PRO	O-C-N	6.29	132.76	122.70
4	S	120	ASP	O-C-N	6.29	132.76	122.70
4	S	107	GLU	O-C-N	6.28	132.75	122.70
2	B	360	LEU	O-C-N	6.28	132.75	122.70
2	B	437	SER	O-C-N	6.28	132.75	122.70
4	S	1	MET	O-C-N	-6.28	112.65	122.70
1	A	522	PHE	CA-C-O	-6.28	106.92	120.10
2	B	116	THR	O-C-N	6.28	132.74	122.70
1	A	302	ASN	CA-C-O	6.27	133.27	120.10
1	A	508	LEU	O-C-N	6.27	133.02	121.10
3	M	55	MET	C-N-CA	-6.27	106.02	121.70
2	B	43	ASN	O-C-N	6.27	133.01	121.10
1	A	249	ASN	O-C-N	6.27	132.73	122.70
2	B	249	ILE	O-C-N	6.27	132.73	122.70
2	B	158	VAL	O-C-N	6.27	132.73	122.70
1	A	68	THR	O-C-N	6.26	132.72	122.70
2	B	244	SER	CA-C-O	-6.26	106.95	120.10
2	B	472	VAL	CA-C-O	6.26	133.25	120.10
2	B	485	LYS	O-C-N	6.26	132.71	122.70
2	B	595	VAL	O-C-N	6.26	132.71	122.70
1	A	395	PHE	C-N-CA	-6.25	106.06	121.70
1	A	385	LYS	O-C-N	6.25	132.70	122.70
2	B	374	PHE	O-C-N	6.25	132.70	122.70
2	B	605	PHE	O-C-N	6.25	132.70	122.70
1	A	278	ILE	C-N-CA	6.25	137.32	121.70
2	B	321	CYS	O-C-N	6.25	132.69	122.70
2	B	410	GLU	O-C-N	6.25	132.69	122.70
1	A	83	ASP	CA-C-N	-6.24	103.46	117.20
1	A	315	CYS	O-C-N	6.24	132.69	122.70
1	A	428	MET	O-C-N	6.24	132.69	122.70
2	B	191	GLU	O-C-N	6.24	132.69	122.70
1	A	625	LEU	O-C-N	-6.24	112.72	122.70
2	B	58	GLU	O-C-N	6.24	132.68	122.70
1	A	353	ASP	O-C-N	6.23	132.67	122.70

Continued on next page...

Continued from previous page...

Mol	Chain	Res	Type	Atoms	Z	Observed(°)	Ideal(°)
1	A	419	ILE	N-CA-C	6.23	127.83	111.00
2	B	214	ALA	O-C-N	6.23	132.67	122.70
2	B	436	LEU	O-C-N	6.23	132.67	122.70
1	A	124	ALA	O-C-N	6.23	132.66	122.70
1	A	404	GLN	C-N-CA	6.22	137.26	121.70
4	S	85	PHE	CA-C-O	6.22	133.17	120.10
1	A	473	ILE	O-C-N	6.22	133.78	123.20
2	B	151	ALA	CA-C-O	-6.22	107.03	120.10
2	B	105	LEU	CA-C-O	6.22	133.16	120.10
1	A	579	LYS	O-C-N	6.22	132.65	122.70
2	B	155	LEU	O-C-N	6.22	132.65	122.70
2	B	615	SER	O-C-N	6.22	132.65	122.70
1	A	200	PHE	O-C-N	6.21	132.64	122.70
2	B	409	LYS	O-C-N	6.21	132.64	122.70
2	B	157	THR	O-C-N	6.21	132.64	122.70
2	B	246	SER	O-C-N	6.21	132.63	122.70
3	M	223	HIS	O-C-N	-6.21	112.77	122.70
1	A	298	ILE	C-N-CA	-6.21	106.19	121.70
1	A	421	PRO	CA-C-O	-6.20	105.31	120.20
1	A	309	PHE	O-C-N	6.20	132.62	122.70
2	B	593	ASN	O-C-N	6.20	132.62	122.70
1	A	141	VAL	O-C-N	6.20	132.61	122.70
2	B	136	CYS	O-C-N	6.19	132.61	122.70
2	B	614	ILE	O-C-N	6.19	132.60	122.70
2	B	27	THR	C-N-CA	6.19	137.17	121.70
2	B	433	VAL	O-C-N	6.19	132.60	122.70
1	A	535	ILE	CA-C-N	-6.18	103.60	117.20
1	A	297	CYS	CA-C-O	6.18	133.08	120.10
1	A	316	LEU	O-C-N	6.18	132.59	122.70
1	A	551	LEU	O-C-N	6.18	132.59	122.70
2	B	233	TYR	O-C-N	6.18	132.59	122.70
2	B	337	THR	O-C-N	6.18	132.58	122.70
2	B	468	LEU	O-C-N	6.17	132.58	122.70
2	B	342	ALA	O-C-N	6.17	132.57	122.70
1	A	162	ILE	CA-C-O	-6.17	107.15	120.10
2	B	550	VAL	O-C-N	6.16	132.56	122.70
2	B	209	SER	CA-C-O	-6.16	107.17	120.10
1	A	533	ILE	CA-C-O	-6.16	107.17	120.10
1	A	81	GLY	C-N-CA	6.15	137.08	121.70
1	A	607	LEU	CA-C-O	-6.15	107.18	120.10
1	A	274	LEU	C-N-CA	-6.15	106.33	121.70
2	B	430	ILE	O-C-N	6.14	132.53	122.70

Continued on next page...

Continued from previous page...

Mol	Chain	Res	Type	Atoms	Z	Observed(°)	Ideal(°)
1	A	127	LEU	O-C-N	6.14	132.52	122.70
2	B	138	ALA	O-C-N	6.14	132.52	122.70
2	B	296	ASP	O-C-N	6.14	132.76	121.10
2	B	319	LEU	O-C-N	6.14	132.52	122.70
1	A	143	VAL	O-C-N	6.14	133.63	123.20
1	A	323	CYS	CA-C-N	6.14	130.70	117.20
2	B	272	GLY	N-CA-C	-6.14	97.76	113.10
2	B	343	LEU	O-C-N	6.13	132.52	122.70
1	A	295	VAL	CA-C-O	-6.13	107.22	120.10
1	A	335	CYS	O-C-N	6.13	132.51	122.70
2	B	512	VAL	O-C-N	6.13	132.51	122.70
1	A	295	VAL	O-C-N	6.13	132.50	122.70
2	B	195	ILE	O-C-N	6.13	132.50	122.70
2	B	139	LEU	CA-C-O	-6.12	107.24	120.10
2	B	324	ALA	CA-C-O	-6.12	107.24	120.10
1	A	155	THR	CA-C-O	-6.12	107.25	120.10
2	B	98	LYS	O-C-N	6.12	132.49	122.70
1	A	454	ILE	CA-C-O	-6.12	107.25	120.10
2	B	464	SER	O-C-N	6.12	132.49	122.70
2	B	549	LEU	O-C-N	6.11	132.47	122.70
2	B	196	LEU	O-C-N	6.11	132.47	122.70
1	A	633	PHE	CA-C-O	-6.10	107.30	120.10
1	A	390	THR	O-C-N	6.10	132.45	122.70
2	B	585	GLY	O-C-N	6.09	132.45	122.70
1	A	105	VAL	C-N-CA	6.09	135.09	122.30
4	S	81	ALA	N-CA-C	-6.09	94.56	111.00
2	B	82	TYR	O-C-N	-6.09	112.96	122.70
1	A	557	LYS	O-C-N	6.08	132.44	122.70
2	B	190	GLU	O-C-N	6.08	132.43	122.70
1	A	336	ILE	O-C-N	6.08	132.43	122.70
2	B	44	PRO	O-C-N	6.08	132.43	122.70
2	B	250	GLU	O-C-N	6.08	132.43	122.70
3	M	308	SER	O-C-N	6.08	132.43	122.70
1	A	271	ARG	CA-C-O	-6.08	107.34	120.10
1	A	584	PHE	O-C-N	6.07	132.42	122.70
2	B	166	SER	O-C-N	6.07	132.42	122.70
3	M	368	ASP	CA-C-O	-6.07	107.35	120.10
2	B	47	LEU	O-C-N	6.07	132.41	122.70
3	M	367	ALA	N-CA-C	-6.07	94.61	111.00
2	B	546	CYS	O-C-N	6.06	132.40	122.70
2	B	568	VAL	O-C-N	6.06	132.40	122.70
2	B	462	ASN	C-N-CA	-6.06	106.55	121.70

Continued on next page...

Continued from previous page...

Mol	Chain	Res	Type	Atoms	Z	Observed(°)	Ideal(°)
3	M	232	HIS	O-C-N	-6.06	113.00	122.70
2	B	451	MET	O-C-N	6.06	132.40	122.70
2	B	466	SER	O-C-N	6.06	132.40	122.70
2	B	216	LYS	O-C-N	-6.06	113.01	122.70
2	B	522	GLU	O-C-N	6.06	132.39	122.70
2	B	103	LEU	O-C-N	6.06	132.39	122.70
4	S	139	GLY	N-CA-C	-6.05	97.96	113.10
1	A	211	ASP	O-C-N	6.05	132.38	122.70
1	A	400	VAL	O-C-N	6.05	132.38	122.70
3	M	429	ASP	C-N-CA	-6.05	106.58	121.70
1	A	465	SER	CA-C-O	6.05	132.80	120.10
1	A	142	LYS	O-C-N	6.05	132.38	122.70
1	A	278	ILE	CA-C-O	-6.05	107.40	120.10
1	A	566	PHE	C-N-CA	6.05	136.82	121.70
2	B	596	LEU	O-C-N	6.05	132.38	122.70
2	B	579	PRO	N-CA-C	6.04	127.82	112.10
4	S	16	LEU	CA-C-O	6.04	132.79	120.10
1	A	369	SER	O-C-N	6.04	132.36	122.70
4	S	150	VAL	CA-C-O	-6.04	107.42	120.10
2	B	435	SER	O-C-N	6.04	132.36	122.70
1	A	107	TYR	CA-C-O	-6.04	107.43	120.10
2	B	101	ILE	O-C-N	6.04	132.35	122.70
1	A	339	TYR	O-C-N	6.03	132.35	122.70
1	A	389	GLN	O-C-N	6.03	132.35	122.70
3	M	90	PHE	O-C-N	6.03	132.35	122.70
1	A	158	LEU	CA-C-O	-6.03	107.43	120.10
1	A	372	ILE	O-C-N	6.03	132.35	122.70
1	A	163	ALA	O-C-N	-6.03	113.06	122.70
1	A	258	LYS	O-C-N	6.03	132.35	122.70
1	A	631	SER	CA-C-O	-6.03	107.44	120.10
2	B	525	ILE	O-C-N	6.03	132.34	122.70
3	M	283	PHE	N-CA-C	-6.03	94.73	111.00
1	A	518	CYS	CA-C-O	-6.02	107.45	120.10
2	B	511	ILE	CA-C-O	6.02	132.75	120.10
1	A	325	SER	CA-C-N	-6.02	103.96	117.20
1	A	620	GLY	CA-C-O	-6.02	109.77	120.60
2	B	64	LYS	O-C-N	6.02	132.33	122.70
2	B	543	GLU	O-C-N	6.02	132.33	122.70
2	B	590	GLN	O-C-N	6.02	132.33	122.70
4	S	151	ALA	O-C-N	6.01	132.32	122.70
1	A	436	CYS	C-N-CA	-6.01	106.67	121.70
2	B	450	VAL	O-C-N	6.01	132.32	122.70

Continued on next page...

Continued from previous page...

Mol	Chain	Res	Type	Atoms	Z	Observed(°)	Ideal(°)
1	A	312	ALA	O-C-N	6.01	132.31	122.70
3	M	120	ARG	O-C-N	6.01	132.31	122.70
2	B	320	SER	O-C-N	6.01	132.31	122.70
2	B	145	MET	CA-C-N	6.00	130.41	117.20
2	B	446	TRP	O-C-N	6.00	132.31	122.70
3	M	367	ALA	N-CA-CB	-6.00	101.70	110.10
1	A	140	VAL	C-N-CA	-6.00	106.70	121.70
2	B	74	ASP	C-N-CA	6.00	136.70	121.70
2	B	510	GLY	O-C-N	6.00	132.30	122.70
1	A	493	ALA	O-C-N	6.00	132.29	122.70
2	B	238	LYS	C-N-CA	5.99	136.68	121.70
2	B	232	ARG	O-C-N	5.99	132.29	122.70
2	B	529	VAL	O-C-N	5.99	132.29	122.70
1	A	129	LYS	O-C-N	5.98	132.27	122.70
3	M	130	GLU	N-CA-C	-5.98	94.84	111.00
4	S	43	ASN	C-N-CA	-5.98	106.74	121.70
1	A	576	MET	O-C-N	5.98	132.27	122.70
2	B	469	ASP	CA-C-O	-5.98	107.55	120.10
1	A	71	VAL	CA-C-O	-5.98	107.55	120.10
2	B	207	VAL	O-C-N	5.98	132.26	122.70
1	A	229	ASN	C-N-CD	5.97	140.94	128.40
1	A	577	VAL	O-C-N	5.97	132.25	122.70
1	A	121	LEU	O-C-N	5.97	132.25	122.70
2	B	569	THR	CA-C-N	-5.97	104.26	116.20
1	A	104	ARG	O-C-N	5.96	132.24	122.70
1	A	296	ASN	CA-C-O	-5.96	107.57	120.10
3	M	121	ILE	O-C-N	5.96	132.24	122.70
1	A	633	PHE	O-C-N	5.96	132.24	122.70
2	B	55	ASN	O-C-N	5.96	132.24	122.70
2	B	112	ASP	N-CA-C	5.96	127.09	111.00
2	B	225	LEU	CA-C-N	-5.96	104.08	117.20
1	A	130	LYS	O-C-N	5.96	132.23	122.70
1	A	553	LEU	O-C-N	5.96	132.23	122.70
1	A	108	LEU	O-C-N	5.96	132.23	122.70
2	B	471	TYR	O-C-N	5.96	132.23	122.70
1	A	486	SER	N-CA-C	-5.95	94.92	111.00
2	B	322	CYS	O-C-N	5.95	132.22	122.70
1	A	490	VAL	O-C-N	5.95	132.22	122.70
2	B	585	GLY	CA-C-O	-5.95	109.89	120.60
1	A	237	SER	CA-C-O	-5.95	107.61	120.10
1	A	425	LYS	O-C-N	5.95	132.22	122.70
1	A	491	THR	O-C-N	5.95	132.22	122.70

Continued on next page...

Continued from previous page...

Mol	Chain	Res	Type	Atoms	Z	Observed(°)	Ideal(°)
2	B	96	LYS	O-C-N	5.95	132.22	122.70
1	A	513	ARG	O-C-N	5.95	132.22	122.70
1	A	538	GLU	C-N-CA	-5.95	106.83	121.70
2	B	333	GLN	N-CA-C	-5.95	94.95	111.00
1	A	214	VAL	O-C-N	5.94	132.21	122.70
2	B	553	ALA	O-C-N	5.94	132.21	122.70
4	S	85	PHE	O-C-N	-5.94	113.19	122.70
2	B	358	MET	O-C-N	5.94	132.20	122.70
1	A	165	ASP	O-C-N	5.93	132.19	122.70
2	B	314	ASN	O-C-N	5.93	132.38	121.10
2	B	131	ASN	O-C-N	5.93	132.19	122.70
1	A	528	ASN	O-C-N	-5.93	113.12	123.20
1	A	625	LEU	C-N-CA	-5.93	106.88	121.70
1	A	154	ILE	C-N-CA	-5.92	106.90	121.70
2	B	530	LEU	O-C-N	5.92	132.17	122.70
2	B	567	GLN	C-N-CA	-5.92	106.91	121.70
2	B	469	ASP	O-C-N	5.91	132.16	122.70
1	A	98	ASN	O-C-N	-5.91	113.24	122.70
3	M	426	LYS	N-CA-C	-5.91	95.04	111.00
1	A	458	ALA	O-C-N	5.90	132.15	122.70
1	A	230	PRO	CA-C-O	-5.90	106.03	120.20
2	B	396	ILE	O-C-N	5.90	132.14	122.70
4	S	159	ALA	O-C-N	5.90	132.13	122.70
1	A	588	LEU	CA-C-O	5.89	132.47	120.10
2	B	371	GLN	O-C-N	5.89	132.12	122.70
2	B	476	ARG	O-C-N	5.89	132.12	122.70
2	B	604	GLU	CA-C-N	-5.89	104.25	117.20
1	A	499	ILE	C-N-CA	-5.88	106.99	121.70
2	B	418	TYR	O-C-N	-5.88	113.29	122.70
2	B	413	LYS	O-C-N	5.88	132.11	122.70
3	M	261	ASN	C-N-CA	-5.88	107.00	121.70
1	A	110	ALA	O-C-N	-5.88	113.30	122.70
1	A	257	LEU	CA-C-O	-5.88	107.75	120.10
1	A	371	ALA	O-C-N	5.88	132.10	122.70
1	A	609	LEU	O-C-N	5.88	132.10	122.70
2	B	247	TYR	O-C-N	5.88	132.10	122.70
1	A	575	LYS	O-C-N	5.87	132.10	122.70
2	B	526	CYS	CA-C-O	-5.87	107.77	120.10
3	M	265	ASN	CA-C-O	-5.87	107.77	120.10
1	A	217	ALA	O-C-N	5.86	132.08	122.70
2	B	33	SER	O-C-N	5.86	132.08	122.70
1	A	600	SER	CA-C-O	-5.86	107.79	120.10

Continued on next page...

Continued from previous page...

Mol	Chain	Res	Type	Atoms	Z	Observed(°)	Ideal(°)
2	B	449	HIS	O-C-N	5.86	132.07	122.70
1	A	276	PRO	O-C-N	-5.86	113.33	122.70
2	B	48	VAL	O-C-N	5.86	132.07	122.70
1	A	512	LEU	O-C-N	5.85	132.07	122.70
2	B	523	PHE	C-N-CA	-5.85	107.07	121.70
2	B	43	ASN	CA-C-O	-5.85	107.82	120.10
3	M	256	VAL	O-C-N	5.84	132.05	122.70
2	B	245	GLN	O-C-N	5.83	132.04	122.70
4	S	98	ILE	CA-C-O	-5.83	107.86	120.10
2	B	60	ARG	O-C-N	5.82	132.02	122.70
1	A	122	MET	O-C-N	5.82	132.01	122.70
2	B	212	VAL	C-N-CA	-5.82	107.15	121.70
2	B	366	LEU	CA-C-O	5.82	132.33	120.10
1	A	494	ASN	O-C-N	5.82	132.01	122.70
1	A	603	VAL	CA-C-O	-5.82	107.88	120.10
4	S	1	MET	CA-C-O	5.82	132.32	120.10
1	A	317	GLU	O-C-N	5.81	132.00	122.70
2	B	176	ALA	O-C-N	5.81	132.00	122.70
2	B	355	ASN	CA-C-O	-5.81	107.89	120.10
1	A	514	GLU	O-C-N	5.81	132.00	122.70
2	B	194	ASP	O-C-N	5.80	131.99	122.70
2	B	444	THR	CA-C-O	5.80	132.29	120.10
2	B	490	ILE	CA-C-O	-5.80	107.92	120.10
2	B	551	LEU	O-C-N	5.80	131.98	122.70
2	B	555	LEU	CA-C-O	-5.80	107.92	120.10
2	B	303	LEU	CA-C-O	-5.80	107.93	120.10
4	S	32	LEU	CA-C-O	-5.79	107.93	120.10
1	A	549	GLU	O-C-N	5.79	131.96	122.70
1	A	64	LEU	C-N-CA	-5.79	107.23	121.70
3	M	306	LEU	C-N-CA	-5.79	107.23	121.70
1	A	545	HIS	CA-C-O	5.79	132.25	120.10
1	A	417	PRO	N-CA-C	-5.78	97.06	112.10
2	B	133	GLU	O-C-N	5.78	131.95	122.70
2	B	189	HIS	O-C-N	5.78	131.95	122.70
1	A	294	SER	O-C-N	5.78	131.95	122.70
2	B	492	LYS	O-C-N	5.78	131.94	122.70
2	B	586	SER	O-C-N	5.78	131.95	122.70
2	B	402	LEU	N-CA-C	5.77	126.58	111.00
1	A	356	ILE	O-C-N	5.77	131.93	122.70
2	B	79	VAL	O-C-N	5.77	131.93	122.70
2	B	412	PHE	CA-C-O	-5.77	107.98	120.10
1	A	152	THR	C-N-CA	-5.77	107.28	121.70

Continued on next page...

Continued from previous page...

Mol	Chain	Res	Type	Atoms	Z	Observed(°)	Ideal(°)
2	B	495	ASP	O-C-N	5.77	131.93	122.70
1	A	268	PRO	O-C-N	5.76	131.92	122.70
3	M	421	GLY	CA-C-O	5.76	130.97	120.60
1	A	275	LEU	O-C-N	-5.75	110.17	121.10
2	B	457	HIS	O-C-N	-5.75	113.50	122.70
1	A	350	SER	C-N-CA	-5.75	107.32	121.70
2	B	86	VAL	O-C-N	5.75	131.90	122.70
3	M	225	VAL	O-C-N	5.75	131.90	122.70
1	A	582	ILE	CA-C-O	-5.75	108.03	120.10
2	B	394	TRP	O-C-N	5.75	131.89	122.70
3	M	292	PRO	C-N-CD	-5.74	107.97	120.60
2	B	99	ARG	CA-C-O	-5.74	108.04	120.10
2	B	120	ILE	CA-C-O	-5.74	108.05	120.10
2	B	532	ARG	O-C-N	5.74	131.88	122.70
1	A	430	ASN	O-C-N	5.74	131.88	122.70
1	A	109	ALA	O-C-N	5.74	131.88	122.70
1	A	217	ALA	CA-C-O	-5.74	108.06	120.10
4	S	96	LEU	CA-C-O	-5.73	108.06	120.10
1	A	173	THR	C-N-CA	5.73	136.02	121.70
2	B	423	HIS	N-CA-C	-5.73	95.54	111.00
1	A	543	TYR	N-CA-C	5.73	126.46	111.00
1	A	126	ASN	O-C-N	5.72	131.85	122.70
2	B	241	ASP	O-C-N	5.72	131.84	122.70
3	M	85	GLY	CA-C-O	-5.72	110.31	120.60
3	M	447	ILE	CA-C-O	5.72	132.10	120.10
1	A	413	SER	C-N-CA	-5.71	107.41	121.70
2	B	174	ALA	CA-C-O	5.71	132.10	120.10
2	B	204	ASP	CA-C-O	-5.71	108.10	120.10
2	B	122	SER	O-C-N	5.71	131.84	122.70
1	A	422	GLU	CA-C-O	-5.71	108.11	120.10
2	B	434	LYS	O-C-N	5.71	131.83	122.70
4	S	152	SER	O-C-N	5.70	131.83	122.70
2	B	393	ILE	O-C-N	5.70	131.82	122.70
4	S	74	GLN	CA-C-O	5.70	132.07	120.10
1	A	123	LEU	O-C-N	5.70	131.81	122.70
1	A	108	LEU	CA-C-O	-5.69	108.14	120.10
2	B	318	ILE	CA-C-O	-5.69	108.15	120.10
2	B	341	GLU	CA-C-O	-5.69	108.15	120.10
2	B	392	SER	O-C-N	5.69	131.81	122.70
2	B	265	VAL	CA-C-N	-5.69	104.68	117.20
4	S	164	ASP	O-C-N	-5.69	113.60	122.70
1	A	325	SER	C-N-CA	5.69	135.92	121.70

Continued on next page...

Continued from previous page...

Mol	Chain	Res	Type	Atoms	Z	Observed(°)	Ideal(°)
2	B	300	ASP	O-C-N	5.69	131.80	122.70
2	B	593	ASN	CA-C-O	-5.69	108.16	120.10
1	A	75	THR	O-C-N	5.69	131.80	122.70
2	B	211	ALA	CA-C-O	-5.68	108.17	120.10
4	S	158	LYS	CA-C-O	-5.68	108.17	120.10
2	B	173	VAL	O-C-N	5.68	131.79	122.70
1	A	106	GLY	CA-C-O	-5.68	110.38	120.60
1	A	491	THR	CA-C-O	-5.68	108.18	120.10
1	A	558	VAL	CA-C-O	-5.67	108.18	120.10
1	A	514	GLU	CA-C-O	-5.67	108.19	120.10
2	B	438	ARG	O-C-N	5.67	131.78	122.70
2	B	436	LEU	CA-C-O	-5.67	108.19	120.10
3	M	88	ASP	O-C-N	5.67	131.77	122.70
1	A	456	ASP	O-C-N	5.67	131.77	122.70
2	B	448	SER	O-C-N	5.67	131.77	122.70
2	B	322	CYS	CA-C-O	-5.67	108.20	120.10
3	M	306	LEU	CA-C-O	5.67	132.00	120.10
1	A	155	THR	O-C-N	5.67	131.86	121.10
2	B	22	ALA	N-CA-C	-5.67	95.70	111.00
4	S	84	TYR	CA-C-O	5.67	132.00	120.10
1	A	478	ARG	O-C-N	5.66	131.76	122.70
3	M	237	THR	O-C-N	-5.66	113.58	123.20
3	M	398	ILE	O-C-N	-5.66	113.64	122.70
1	A	293	GLU	O-C-N	5.66	131.75	122.70
2	B	545	ARG	O-C-N	5.66	131.75	122.70
2	B	318	ILE	O-C-N	5.66	131.75	122.70
3	M	396	GLN	O-C-N	-5.66	113.65	122.70
1	A	253	ILE	O-C-N	5.65	131.75	122.70
2	B	189	HIS	CA-C-O	-5.65	108.23	120.10
1	A	452	ALA	O-C-N	5.65	131.74	122.70
2	B	383	VAL	CA-C-N	-5.65	104.77	117.20
2	B	456	ASP	O-C-N	5.65	131.74	122.70
3	M	284	SER	C-N-CD	5.65	140.27	128.40
4	S	154	ASP	O-C-N	5.65	131.74	122.70
1	A	448	GLU	C-N-CA	5.65	135.82	121.70
1	A	479	ASN	O-C-N	5.65	131.74	122.70
1	A	572	PHE	C-N-CA	5.65	135.82	121.70
4	S	137	GLN	CA-C-O	5.65	131.96	120.10
3	M	87	LEU	O-C-N	5.64	131.73	122.70
2	B	274	PRO	N-CA-C	-5.64	97.43	112.10
2	B	391	ALA	O-C-N	5.64	131.73	122.70
1	A	233	PHE	CA-C-O	5.64	131.95	120.10

Continued on next page...

Continued from previous page...

Mol	Chain	Res	Type	Atoms	Z	Observed(°)	Ideal(°)
3	M	421	GLY	C-N-CD	5.64	140.25	128.40
1	A	230	PRO	CA-N-CD	5.63	119.59	111.70
2	B	239	GLN	C-N-CA	5.63	135.78	121.70
1	A	88	ASN	O-C-N	-5.63	113.69	122.70
3	M	385	ARG	CA-C-O	5.63	131.93	120.10
1	A	241	TYR	O-C-N	5.63	131.71	122.70
2	B	407	ASN	O-C-N	5.63	131.71	122.70
3	M	230	LYS	O-C-N	-5.63	113.70	122.70
2	B	472	VAL	C-N-CA	-5.62	107.64	121.70
2	B	172	GLU	C-N-CA	-5.62	107.64	121.70
1	A	224	GLU	C-N-CA	-5.62	107.65	121.70
1	A	145	ILE	CA-C-O	-5.62	108.30	120.10
1	A	265	GLN	C-N-CA	5.62	135.75	121.70
2	B	117	LEU	O-C-N	5.62	131.69	122.70
3	M	444	ALA	N-CA-CB	-5.62	102.24	110.10
1	A	575	LYS	CA-C-O	-5.60	108.33	120.10
2	B	303	LEU	O-C-N	5.60	131.66	122.70
1	A	90	HIS	O-C-N	5.59	131.65	122.70
1	A	551	LEU	CA-C-O	-5.59	108.35	120.10
1	A	387	ILE	CA-C-O	-5.59	108.36	120.10
4	S	34	GLN	O-C-N	5.59	131.65	122.70
2	B	426	GLU	O-C-N	5.59	131.64	122.70
2	B	215	TYR	CA-C-O	-5.58	108.37	120.10
2	B	496	LEU	O-C-N	5.58	131.64	122.70
1	A	139	ASP	CA-C-O	-5.58	108.38	120.10
1	A	630	PRO	O-C-N	5.58	131.63	122.70
1	A	184	ALA	O-C-N	5.58	131.62	122.70
2	B	138	ALA	CA-C-O	-5.58	108.38	120.10
2	B	302	PHE	CA-C-O	-5.58	108.39	120.10
2	B	387	ASP	N-CA-C	5.58	126.06	111.00
3	M	384	GLY	O-C-N	5.57	131.62	122.70
2	B	155	LEU	CA-C-O	-5.57	108.41	120.10
1	A	334	SER	O-C-N	5.57	131.61	122.70
2	B	605	PHE	CA-C-O	-5.57	108.41	120.10
1	A	380	ASP	O-C-N	5.56	131.60	122.70
1	A	635	ALA	CA-C-O	-5.56	108.42	120.10
3	M	368	ASP	O-C-N	5.56	131.67	121.10
1	A	489	GLU	O-C-N	5.56	131.59	122.70
1	A	135	ASP	CA-C-N	5.55	127.31	116.20
1	A	137	ASN	C-N-CA	-5.55	107.81	121.70
1	A	319	LEU	CA-C-O	5.55	131.76	120.10
1	A	177	ILE	CA-C-O	-5.55	108.44	120.10

Continued on next page...

Continued from previous page...

Mol	Chain	Res	Type	Atoms	Z	Observed(°)	Ideal(°)
1	A	352	PHE	O-C-N	5.55	131.58	122.70
2	B	493	LEU	CA-C-O	-5.55	108.45	120.10
2	B	540	GLU	N-CA-C	5.55	125.98	111.00
1	A	193	PRO	CA-C-O	-5.54	106.89	120.20
2	B	569	THR	C-N-CA	5.54	133.94	122.30
1	A	222	ILE	O-C-N	5.54	131.57	122.70
1	A	156	PRO	CA-C-O	5.54	133.49	120.20
3	M	387	GLU	O-C-N	-5.54	113.84	122.70
4	S	86	THR	O-C-N	-5.54	113.84	122.70
1	A	578	LEU	CA-C-O	-5.54	108.47	120.10
1	A	416	ILE	C-N-CA	-5.53	98.76	122.00
2	B	415	LEU	CA-C-O	-5.53	108.48	120.10
4	S	109	LEU	C-N-CA	-5.53	107.87	121.70
2	B	522	GLU	N-CA-C	5.53	125.93	111.00
1	A	216	SER	O-C-N	5.53	131.54	122.70
1	A	254	ILE	O-C-N	5.53	131.54	122.70
3	M	46	SER	O-C-N	-5.53	113.86	122.70
2	B	477	MET	O-C-N	5.53	131.54	122.70
4	S	87	PHE	O-C-N	-5.53	113.86	122.70
1	A	80	TYR	CB-CA-C	5.52	121.45	110.40
1	A	487	MET	C-N-CA	-5.52	107.89	121.70
1	A	513	ARG	CA-C-O	-5.52	108.50	120.10
4	S	87	PHE	CA-C-O	5.52	131.70	120.10
1	A	583	GLU	O-C-N	5.52	131.53	122.70
1	A	72	LEU	O-C-N	5.52	131.53	122.70
1	A	341	ILE	O-C-N	5.52	132.58	123.20
1	A	251	TRP	O-C-N	5.52	131.53	122.70
2	B	457	HIS	C-N-CA	-5.51	107.91	121.70
1	A	333	ILE	CA-C-O	-5.51	108.52	120.10
1	A	419	ILE	CA-C-N	-5.51	105.07	117.20
1	A	229	ASN	C-N-CA	-5.51	98.87	122.00
2	B	73	ASP	C-N-CA	-5.51	107.94	121.70
1	A	501	ASN	C-N-CA	5.50	135.46	121.70
2	B	156	HIS	O-C-N	5.50	131.51	122.70
2	B	68	SER	O-C-N	5.50	131.51	122.70
1	A	265	GLN	CB-CA-C	5.50	121.40	110.40
1	A	63	ASP	N-CA-C	5.50	125.85	111.00
1	A	426	ILE	O-C-N	5.50	131.50	122.70
2	B	340	ILE	O-C-N	5.49	131.49	122.70
3	M	117	ASN	O-C-N	5.49	131.49	122.70
4	S	131	VAL	O-C-N	5.49	131.49	122.70
1	A	292	TYR	O-C-N	5.49	131.49	122.70

Continued on next page...

Continued from previous page...

Mol	Chain	Res	Type	Atoms	Z	Observed(°)	Ideal(°)
2	B	290	SER	N-CA-C	5.49	125.81	111.00
2	B	345	ARG	O-C-N	5.48	131.47	122.70
1	A	191	GLN	C-N-CA	5.48	135.40	121.70
1	A	333	ILE	O-C-N	5.48	131.47	122.70
1	A	580	GLU	O-C-N	5.48	131.47	122.70
2	B	245	GLN	CA-C-O	-5.48	108.59	120.10
2	B	458	MET	C-N-CA	-5.48	108.00	121.70
4	S	78	LYS	CA-C-O	5.48	131.61	120.10
2	B	36	THR	CA-C-O	-5.48	108.59	120.10
1	A	188	VAL	CA-C-O	-5.48	108.60	120.10
2	B	547	GLN	O-C-N	5.48	131.46	122.70
3	M	74	TYR	CA-C-N	-5.48	105.15	117.20
1	A	441	TYR	N-CA-C	-5.47	96.22	111.00
2	B	109	ALA	CB-CA-C	-5.47	101.89	110.10
2	B	531	ARG	CA-C-O	-5.47	108.61	120.10
4	S	138	GLY	C-N-CA	5.47	133.78	122.30
3	M	296	LYS	C-N-CA	-5.47	108.03	121.70
2	B	475	ILE	CA-C-O	-5.47	108.62	120.10
2	B	431	MET	O-C-N	5.46	131.44	122.70
2	B	126	SER	CA-C-O	5.46	131.57	120.10
2	B	365	PHE	O-C-N	5.46	131.43	122.70
2	B	36	THR	O-C-N	5.45	131.42	122.70
3	M	267	ILE	C-N-CA	-5.45	110.86	122.30
1	A	175	PRO	O-C-N	5.45	131.41	122.70
2	B	491	PHE	O-C-N	5.45	131.41	122.70
2	B	127	LEU	C-N-CA	-5.44	108.09	121.70
1	A	106	GLY	O-C-N	5.44	131.41	122.70
1	A	392	MET	CA-C-O	-5.44	108.67	120.10
1	A	255	ARG	O-C-N	5.44	131.41	122.70
1	A	634	ASN	O-C-N	5.44	131.40	122.70
2	B	388	PRO	O-C-N	5.44	131.40	122.70
3	M	469	GLY	C-N-CA	-5.44	108.10	121.70
1	A	91	ILE	CA-C-O	-5.44	108.68	120.10
1	A	427	LYS	O-C-N	5.44	131.40	122.70
2	B	29	LYS	C-N-CA	-5.43	108.11	121.70
1	A	71	VAL	O-C-N	5.43	131.39	122.70
2	B	586	SER	CA-C-O	-5.43	108.70	120.10
1	A	316	LEU	CA-C-O	-5.42	108.71	120.10
2	B	67	ILE	CA-C-O	-5.42	108.71	120.10
1	A	305	GLU	CA-C-N	5.42	129.13	117.20
1	A	611	LEU	CA-C-O	-5.42	108.72	120.10
3	M	354	ASP	N-CA-CB	5.42	120.35	110.60

Continued on next page...

Continued from previous page...

Mol	Chain	Res	Type	Atoms	Z	Observed(°)	Ideal(°)
1	A	313	MET	CA-C-O	-5.42	108.72	120.10
2	B	248	LEU	CA-C-O	-5.42	108.72	120.10
1	A	160	ARG	O-C-N	5.42	131.36	122.70
1	A	433	ILE	CA-C-O	-5.41	108.74	120.10
2	B	482	ASN	O-C-N	5.41	131.38	121.10
1	A	308	ASP	N-CA-C	5.41	125.60	111.00
3	M	307	SER	O-C-N	5.41	131.35	122.70
1	A	397	ASP	O-C-N	-5.41	114.05	122.70
1	A	593	THR	O-C-N	5.41	131.35	122.70
2	B	94	ASP	O-C-N	5.41	131.35	122.70
2	B	430	ILE	CA-C-O	-5.41	108.75	120.10
1	A	467	LYS	C-N-CA	-5.40	108.20	121.70
3	M	16	PHE	O-C-N	-5.40	114.06	122.70
2	B	609	ASP	O-C-N	5.40	131.34	122.70
2	B	439	CYS	CA-C-O	-5.40	108.77	120.10
1	A	136	GLY	CA-C-N	5.39	129.06	117.20
2	B	213	LEU	CA-C-O	-5.39	108.78	120.10
2	B	360	LEU	CA-C-O	-5.39	108.78	120.10
2	B	120	ILE	O-C-N	5.39	131.32	122.70
1	A	455	MET	O-C-N	5.39	131.32	122.70
2	B	363	ILE	CA-C-O	-5.39	108.79	120.10
1	A	368	ARG	O-C-N	5.38	131.31	122.70
1	A	572	PHE	O-C-N	5.38	131.30	122.70
1	A	604	LEU	CA-C-O	-5.37	108.82	120.10
1	A	566	PHE	O-C-N	5.37	131.30	122.70
1	A	226	SER	C-N-CA	-5.37	108.28	121.70
1	A	390	THR	CA-C-O	-5.37	108.83	120.10
1	A	624	LEU	CA-C-O	-5.37	108.83	120.10
2	B	544	THR	CA-C-O	-5.37	108.83	120.10
4	S	76	ILE	O-C-N	-5.37	114.11	122.70
4	S	125	TRP	O-C-N	5.37	131.28	122.70
2	B	577	ASN	CA-C-N	5.36	132.11	117.10
1	A	105	VAL	CA-C-O	-5.36	108.85	120.10
1	A	216	SER	CA-C-O	-5.36	108.85	120.10
1	A	391	LEU	CA-C-O	5.35	131.34	120.10
1	A	466	ASP	N-CA-C	-5.35	96.55	111.00
1	A	180	LYS	O-C-N	5.35	131.26	122.70
1	A	181	ALA	O-C-N	5.35	131.25	122.70
2	B	219	TYR	N-CA-C	5.34	125.43	111.00
1	A	559	PHE	CA-C-O	-5.34	108.88	120.10
1	A	518	CYS	O-C-N	5.34	131.24	122.70
1	A	172	SER	C-N-CA	-5.33	108.37	121.70

Continued on next page...

Continued from previous page...

Mol	Chain	Res	Type	Atoms	Z	Observed(°)	Ideal(°)
1	A	277	LYS	CA-C-N	5.33	128.93	117.20
2	B	364	HIS	O-C-N	5.33	131.23	122.70
3	M	25	PRO	N-CA-C	-5.33	98.25	112.10
2	B	61	ASP	O-C-N	5.33	131.22	122.70
2	B	178	ILE	CA-C-O	-5.33	108.91	120.10
1	A	312	ALA	CA-C-O	-5.32	108.92	120.10
1	A	434	SER	CA-C-O	-5.32	108.94	120.10
1	A	493	ALA	CA-C-O	-5.32	108.94	120.10
2	B	132	SER	CA-C-O	5.32	131.26	120.10
2	B	595	VAL	CA-C-O	-5.32	108.94	120.10
1	A	345	ASN	O-C-N	5.31	131.20	122.70
1	A	314	ALA	O-C-N	5.31	131.20	122.70
2	B	354	GLY	N-CA-C	-5.31	99.83	113.10
2	B	169	VAL	CA-C-O	-5.30	108.97	120.10
2	B	196	LEU	CA-C-O	-5.30	108.97	120.10
4	S	18	LYS	CA-C-O	5.30	131.22	120.10
1	A	110	ALA	CA-C-O	5.29	131.22	120.10
1	A	115	TYR	C-N-CA	5.29	134.93	121.70
4	S	95	GLU	CA-C-O	-5.29	108.99	120.10
2	B	616	SER	O-C-N	5.29	131.16	122.70
3	M	55	MET	O-C-N	-5.29	114.24	122.70
1	A	237	SER	O-C-N	5.28	131.14	121.10
1	A	335	CYS	CA-C-O	-5.28	109.01	120.10
2	B	357	GLU	O-C-N	5.28	131.15	122.70
2	B	467	VAL	CA-C-O	-5.28	109.00	120.10
1	A	244	LEU	CA-C-O	5.28	131.19	120.10
1	A	470	GLY	O-C-N	5.28	131.15	122.70
3	M	309	GLN	O-C-N	5.28	131.15	122.70
1	A	505	ASN	O-C-N	-5.28	114.25	122.70
3	M	284	SER	CA-C-O	5.28	131.18	120.10
1	A	382	ASP	O-C-N	5.28	131.14	122.70
1	A	398	GLU	N-CA-C	5.28	125.24	111.00
2	B	451	MET	CA-C-O	-5.27	109.03	120.10
2	B	188	TYR	O-C-N	5.27	131.13	122.70
3	M	1	MET	C-N-CA	-5.27	108.53	121.70
2	B	216	LYS	CA-C-O	5.27	131.16	120.10
1	A	125	THR	CA-C-O	-5.26	109.04	120.10
2	B	54	ARG	N-CA-C	5.26	125.21	111.00
2	B	533	LEU	O-C-N	5.26	131.12	122.70
2	B	222	HIS	N-CA-C	5.26	125.21	111.00
2	B	443	SER	CA-C-N	-5.26	105.63	117.20
2	B	323	ASN	CA-C-O	-5.26	109.06	120.10

Continued on next page...

Continued from previous page...

Mol	Chain	Res	Type	Atoms	Z	Observed(°)	Ideal(°)
1	A	437	SER	N-CA-C	5.25	125.19	111.00
2	B	588	ILE	CA-C-O	-5.25	109.07	120.10
1	A	370	LYS	CA-C-O	-5.25	109.07	120.10
1	A	528	ASN	C-N-CA	-5.25	111.27	122.30
2	B	346	THR	CA-C-O	-5.25	109.07	120.10
1	A	242	GLU	CA-C-O	-5.24	109.09	120.10
1	A	496	ILE	CA-C-O	-5.24	109.09	120.10
1	A	163	ALA	CA-C-O	5.24	131.10	120.10
2	B	554	LYS	CA-C-O	-5.24	109.10	120.10
2	B	589	SER	CA-C-O	-5.24	109.09	120.10
2	B	319	LEU	CA-C-O	-5.24	109.10	120.10
2	B	439	CYS	O-C-N	5.24	132.11	123.20
2	B	192	LEU	CA-C-O	-5.24	109.11	120.10
4	S	78	LYS	O-C-N	-5.24	114.32	122.70
1	A	516	ILE	CA-C-O	-5.23	109.11	120.10
2	B	63	MET	CA-C-O	-5.22	109.13	120.10
3	M	331	LEU	CA-C-N	-5.22	105.75	116.20
1	A	526	VAL	C-N-CA	5.22	134.75	121.70
1	A	327	ASP	O-C-N	5.22	131.01	121.10
1	A	85	ALA	O-C-N	5.21	131.03	122.70
2	B	118	LEU	O-C-N	5.21	131.03	122.70
2	B	489	ILE	CA-C-O	-5.21	109.16	120.10
1	A	341	ILE	CA-C-O	-5.21	109.17	120.10
1	A	562	TRP	CA-C-O	-5.20	109.17	120.10
3	M	90	PHE	CA-C-O	-5.20	109.19	120.10
3	M	323	MET	C-N-CA	-5.19	108.71	121.70
1	A	548	GLN	O-C-N	5.19	131.01	122.70
1	A	634	ASN	CA-C-O	-5.19	109.19	120.10
2	B	494	ALA	CA-C-O	-5.19	109.20	120.10
2	B	411	ILE	CA-C-O	-5.19	109.20	120.10
1	A	330	LEU	CA-C-O	-5.19	109.21	120.10
2	B	503	LEU	CA-C-N	-5.19	105.79	117.20
2	B	223	LEU	CA-C-O	5.18	130.99	120.10
1	A	146	ALA	CA-C-O	-5.18	109.22	120.10
1	A	278	ILE	O-C-N	-5.18	114.42	122.70
3	M	122	SER	CA-C-O	-5.17	109.24	120.10
1	A	370	LYS	O-C-N	5.17	130.98	122.70
4	S	141	VAL	N-CA-C	-5.17	97.03	111.00
1	A	178	ARG	O-C-N	5.17	130.97	122.70
1	A	253	ILE	CA-C-O	-5.17	109.25	120.10
3	M	354	ASP	CB-CA-C	5.17	120.73	110.40
2	B	399	LEU	O-C-N	-5.16	114.44	122.70

Continued on next page...

Continued from previous page...

Mol	Chain	Res	Type	Atoms	Z	Observed(°)	Ideal(°)
2	B	551	LEU	CA-C-O	-5.16	109.26	120.10
2	B	112	ASP	CA-C-O	-5.16	109.26	120.10
2	B	175	LEU	O-C-N	5.16	130.96	122.70
3	M	117	ASN	CA-C-O	-5.16	109.26	120.10
1	A	134	TYR	C-N-CA	-5.16	108.80	121.70
1	A	221	VAL	CA-C-O	-5.16	109.27	120.10
4	S	103	GLN	CA-C-O	-5.15	109.28	120.10
1	A	474	GLY	CA-C-O	-5.15	111.32	120.60
1	A	533	ILE	O-C-N	5.15	130.94	122.70
2	B	166	SER	C-N-CA	5.15	134.58	121.70
2	B	304	GLN	O-C-N	5.15	130.94	122.70
2	B	350	THR	N-CA-C	5.15	124.91	111.00
1	A	94	VAL	CA-C-O	5.15	130.91	120.10
1	A	174	ARG	C-N-CD	-5.15	109.28	120.60
1	A	239	LEU	C-N-CA	-5.15	108.83	121.70
2	B	566	ALA	N-CA-CB	-5.15	102.89	110.10
1	A	141	VAL	CA-C-O	-5.14	109.30	120.10
2	B	577	ASN	N-CA-CB	-5.14	101.34	110.60
1	A	597	GLN	O-C-N	5.14	130.92	122.70
2	B	470	ALA	O-C-N	5.14	130.92	122.70
2	B	280	PRO	N-CA-C	-5.13	98.75	112.10
1	A	380	ASP	CA-C-O	-5.13	109.32	120.10
2	B	608	ARG	O-C-N	5.13	130.91	122.70
2	B	84	ALA	O-C-N	5.13	130.91	122.70
4	S	154	ASP	CA-C-O	-5.13	109.33	120.10
1	A	555	LEU	CA-C-O	-5.13	109.33	120.10
2	B	134	LEU	CA-C-O	-5.13	109.33	120.10
2	B	401	THR	C-N-CA	5.13	134.52	121.70
2	B	530	LEU	CA-C-O	-5.12	109.34	120.10
2	B	592	TYR	CA-C-O	-5.12	109.34	120.10
4	S	164	ASP	CA-C-N	5.12	128.47	117.20
2	B	391	ALA	CA-C-O	-5.12	109.36	120.10
2	B	500	GLN	N-CA-C	5.12	124.81	111.00
1	A	174	ARG	O-C-N	5.11	130.82	121.10
2	B	421	SER	N-CA-C	5.11	124.81	111.00
1	A	230	PRO	O-C-N	5.11	130.87	122.70
2	B	615	SER	CA-C-O	-5.11	109.38	120.10
4	S	89	VAL	C-N-CA	-5.11	108.93	121.70
4	S	54	PRO	CA-C-N	5.10	131.39	117.10
2	B	116	THR	CA-C-O	-5.10	109.38	120.10
2	B	230	PHE	CA-C-O	5.10	130.80	120.10
2	B	492	LYS	CA-C-O	-5.09	109.40	120.10

Continued on next page...

Continued from previous page...

Mol	Chain	Res	Type	Atoms	Z	Observed(°)	Ideal(°)
1	A	302	ASN	N-CA-CB	5.09	119.77	110.60
2	B	365	PHE	CA-C-O	-5.09	109.41	120.10
1	A	121	LEU	CA-C-O	-5.08	109.43	120.10
2	B	590	GLN	CA-C-O	-5.08	109.43	120.10
1	A	148	SER	O-C-N	5.08	131.83	123.20
1	A	103	LYS	C-N-CA	-5.07	109.02	121.70
1	A	280	GLU	O-C-N	5.07	130.82	122.70
1	A	445	ASN	N-CA-C	-5.07	97.30	111.00
1	A	599	ARG	O-C-N	5.07	130.82	122.70
3	M	39	PRO	O-C-N	5.07	130.82	122.70
1	A	305	GLU	CA-C-O	-5.07	109.45	120.10
1	A	628	VAL	CA-C-O	-5.07	109.45	120.10
1	A	193	PRO	O-C-N	5.07	130.81	122.70
1	A	254	ILE	CA-C-O	-5.07	109.46	120.10
1	A	512	LEU	CA-C-O	-5.07	109.46	120.10
2	B	340	ILE	CA-C-O	-5.07	109.46	120.10
2	B	562	ASN	O-C-N	5.07	130.80	122.70
1	A	179	LYS	O-C-N	5.06	130.80	122.70
2	B	465	ALA	O-C-N	5.06	130.79	122.70
1	A	218	ALA	CA-C-O	-5.06	109.48	120.10
1	A	338	PHE	CA-C-O	-5.06	109.48	120.10
1	A	199	ASN	N-CA-C	5.05	124.64	111.00
1	A	337	LEU	CA-C-O	-5.05	109.49	120.10
1	A	422	GLU	O-C-N	5.05	130.78	122.70
1	A	502	ASP	O-C-N	5.05	130.77	122.70
2	B	426	GLU	CA-C-O	-5.05	109.50	120.10
2	B	548	ILE	CA-C-O	-5.04	109.51	120.10
1	A	113	SER	N-CA-C	5.04	124.60	111.00
1	A	252	ILE	CA-C-O	-5.04	109.52	120.10
1	A	602	GLU	CA-C-O	-5.04	109.52	120.10
3	M	316	ARG	C-N-CA	-5.04	109.10	121.70
1	A	73	LYS	O-C-N	5.04	130.76	122.70
1	A	383	ASN	CA-C-O	-5.04	109.53	120.10
2	B	86	VAL	CA-C-O	-5.04	109.53	120.10
3	M	57	GLY	O-C-N	-5.03	114.65	122.70
2	B	119	SER	CA-C-O	-5.03	109.54	120.10
3	M	234	ARG	O-C-N	-5.03	114.66	122.70
1	A	620	GLY	O-C-N	5.02	130.74	122.70
1	A	77	LEU	O-C-N	5.02	130.73	122.70
2	B	210	CYS	CA-C-O	-5.02	109.56	120.10
2	B	69	ILE	C-N-CA	-5.02	109.16	121.70
4	S	36	TYR	CA-C-O	-5.02	109.57	120.10

Continued on next page...

Continued from previous page...

Mol	Chain	Res	Type	Atoms	Z	Observed(^{o})	Ideal(^{o})
1	A	90	HIS	CA-C-O	-5.01	109.57	120.10
2	B	34	SER	O-C-N	5.01	130.72	122.70
2	B	60	ARG	CA-C-O	-5.01	109.58	120.10
2	B	157	THR	CA-C-O	-5.01	109.58	120.10
4	S	12	CYS	C-N-CA	-5.01	109.18	121.70
1	A	323	CYS	O-C-N	-5.01	114.69	122.70
4	S	132	LEU	CA-C-O	-5.01	109.58	120.10
1	A	185	LEU	CA-C-O	-5.00	109.59	120.10
1	A	469	LEU	CA-C-N	-5.00	106.19	116.20
4	S	157	ASN	CA-C-O	-5.00	109.59	120.10
1	A	85	ALA	CA-C-N	-5.00	106.19	117.20
2	B	482	ASN	CA-C-O	-5.00	109.59	120.10
1	A	431	VAL	CA-C-O	-5.00	109.60	120.10
1	A	560	SER	O-C-N	5.00	130.70	122.70

There are no chirality outliers.

All (99) planarity outliers are listed below:

Mol	Chain	Res	Type	Group
1	A	117	ASP	Mainchain
1	A	174	ARG	Mainchain
1	A	192	TYR	Mainchain
1	A	199	ASN	Mainchain
1	A	204	VAL	Mainchain
1	A	219	VAL	Mainchain
1	A	233	PHE	Mainchain
1	A	240	LEU	Mainchain
1	A	244	LEU	Mainchain
1	A	260	PHE	Mainchain
1	A	275	LEU	Mainchain
1	A	277	LYS	Mainchain
1	A	278	ILE	Mainchain
1	A	282	MET	Mainchain
1	A	298	ILE	Mainchain
1	A	302	ASN	Mainchain
1	A	306	GLU	Mainchain
1	A	319	LEU	Mainchain
1	A	320	HIS	Mainchain
1	A	323	CYS	Mainchain
1	A	325	SER	Mainchain
1	A	328	PRO	Mainchain
1	A	350	SER	Mainchain

Continued on next page...

Continued from previous page...

Mol	Chain	Res	Type	Group
1	A	399	ASP	Peptide
1	A	400	VAL	Peptide
1	A	441	TYR	Mainchain
1	A	462	GLN	Mainchain
1	A	487	MET	Mainchain
1	A	500	SER	Mainchain
1	A	501	ASN	Mainchain
1	A	505	ASN	Mainchain
1	A	506	LYS	Mainchain
1	A	527	GLU	Mainchain
1	A	528	ASN	Mainchain
1	A	529	GLY	Mainchain
1	A	531	ASP	Mainchain
1	A	535	ILE	Mainchain
1	A	536	MET	Mainchain
1	A	538	GLU	Mainchain
1	A	539	ASN	Mainchain
1	A	565	ASN	Mainchain
1	A	569	ASP	Mainchain
1	A	571	ARG	Mainchain
1	A	586	GLU	Mainchain
1	A	588	LEU	Mainchain
1	A	64	LEU	Mainchain
1	A	80	TYR	Mainchain
1	A	84	MET	Mainchain
1	A	94	VAL	Mainchain
2	B	126	SER	Mainchain
2	B	142	LEU	Mainchain
2	B	147	MET	Mainchain
2	B	237	ILE	Mainchain
2	B	267	ASP	Mainchain
2	B	278	PRO	Peptide
2	B	288	TYR	Mainchain
2	B	326	TYR	Mainchain
2	B	375	LEU	Mainchain
2	B	377	TYR	Mainchain
2	B	381	PHE	Mainchain
2	B	384	PHE	Mainchain
2	B	404	ASN	Mainchain
2	B	41	ASN	Peptide
2	B	444	THR	Mainchain
2	B	459	GLU	Mainchain

Continued on next page...

Continued from previous page...

Mol	Chain	Res	Type	Group
2	B	485	LYS	Mainchain
2	B	497	LEU	Mainchain
2	B	523	PHE	Mainchain
2	B	536	ASN	Mainchain
2	B	557	SER	Mainchain
2	B	56	SER	Mainchain
2	B	565	GLN	Mainchain
2	B	569	THR	Mainchain
2	B	573	GLU	Mainchain
2	B	584	SER	Mainchain
2	B	78	ASP	Mainchain
2	B	82	TYR	Mainchain
3	M	265	ASN	Mainchain
3	M	284	SER	Mainchain
3	M	292	PRO	Peptide
3	M	40	GLN	Mainchain
3	M	421	GLY	Mainchain
3	M	426	LYS	Peptide
3	M	445	SER	Peptide
3	M	45	SER	Peptide
3	M	456	SER	Mainchain
3	M	462	LYS	Peptide
3	M	477	GLY	Peptide
3	M	57	GLY	Mainchain
3	M	8	THR	Mainchain
3	M	85	GLY	Mainchain
4	S	101	LEU	Mainchain
4	S	102	ILE	Mainchain
4	S	135	ILE	Mainchain
4	S	161	GLU	Mainchain
4	S	163	THR	Peptide
4	S	167	ILE	Peptide
4	S	43	ASN	Mainchain
4	S	53	THR	Mainchain

5.2 Too-close contacts (i)

In the following table, the Non-H and H(model) columns list the number of non-hydrogen atoms and hydrogen atoms in the chain respectively. The H(added) column lists the number of hydrogen atoms added and optimized by MolProbity. The Clashes column lists the number of clashes within the asymmetric unit, whereas Symm-Clashes lists symmetry-related clashes.

Mol	Chain	Non-H	H(model)	H(added)	Clashes	Symm-Clashes
1	A	4625	0	4688	2296	0
2	B	4954	0	4969	3353	0
3	M	3106	0	3081	1519	0
4	S	1356	0	1329	910	0
All	All	14041	0	14067	7266	0

The all-atom clashscore is defined as the number of clashes found per 1000 atoms (including hydrogen atoms). The all-atom clashscore for this structure is 258.

All (7266) close contacts within the same asymmetric unit are listed below, sorted by their clash magnitude.

Atom-1	Atom-2	Interatomic distance (Å)	Clash overlap (Å)
4:S:8:PHE:CE1	4:S:84:TYR:HB2	1.15	1.68
2:B:171:GLY:CA	2:B:207:VAL:HG13	1.22	1.64
1:A:140:VAL:HA	1:A:177:ILE:CG1	1.17	1.64
1:A:102:GLN:CG	4:S:166:LYS:H	1.11	1.63
1:A:633:PHE:CE1	2:B:513:TRP:CE3	1.74	1.63
2:B:127:LEU:HD13	2:B:157:THR:CG2	1.14	1.62
2:B:219:TYR:HB3	2:B:223:LEU:CD2	1.19	1.61
1:A:217:ALA:CA	4:S:142:ILE:HB	1.14	1.61
3:M:104:PHE:CE1	3:M:113:LYS:HE2	1.25	1.61
2:B:123:LEU:CD1	2:B:142:LEU:HG	1.29	1.61
2:B:252:LEU:HD13	2:B:302:PHE:CD1	1.15	1.61
4:S:5:VAL:HG21	4:S:132:LEU:CD2	1.26	1.61
1:A:288:THR:HG21	1:A:322:PHE:CZ	1.25	1.60
2:B:216:LYS:CB	2:B:251:LEU:HD13	1.19	1.60
4:S:8:PHE:CE1	4:S:84:TYR:CB	1.77	1.60
2:B:83:PHE:CZ	2:B:119:SER:HB3	1.14	1.60
2:B:260:LEU:HB3	2:B:291:TYR:CE1	1.14	1.60
3:M:449:VAL:CG1	3:M:452:ILE:HD11	1.26	1.60
2:B:556:LEU:HA	2:B:588:ILE:CD1	1.27	1.59
2:B:219:TYR:CE1	2:B:226:LEU:CB	1.80	1.59
2:B:260:LEU:CB	2:B:291:TYR:HE1	1.14	1.59
3:M:104:PHE:CE1	3:M:113:LYS:CE	1.74	1.59
2:B:83:PHE:CE2	2:B:119:SER:HB3	1.09	1.59
2:B:139:LEU:HD23	2:B:173:VAL:CA	1.23	1.59
1:A:557:LYS:CE	2:B:606:ASP:H	1.06	1.58
2:B:433:VAL:HG12	2:B:474:VAL:CG2	1.24	1.58
3:M:215:TYR:CD1	3:M:468:LYS:HA	1.35	1.58
1:A:105:VAL:HG23	4:S:167:ILE:CD1	1.11	1.58
1:A:105:VAL:H	4:S:167:ILE:CD1	1.13	1.58
3:M:69:ILE:CD1	3:M:90:PHE:HZ	1.10	1.57

Continued on next page...

Continued from previous page...

Atom-1	Atom-2	Interatomic distance (Å)	Clash overlap (Å)
3:M:244:VAL:HA	3:M:472:TYR:CD2	1.13	1.57
2:B:316:THR:HG21	3:M:90:PHE:CE2	1.38	1.57
2:B:546:CYS:HB2	2:B:607:ILE:CG1	1.20	1.57
3:M:104:PHE:CZ	3:M:113:LYS:CE	1.80	1.57
3:M:379:LEU:HD22	3:M:386:PHE:CD1	1.36	1.57
2:B:2:VAL:CG1	2:B:6:HIS:NE2	1.67	1.57
2:B:319:LEU:CD1	2:B:358:MET:CG	1.80	1.57
2:B:523:PHE:HZ	2:B:580:TYR:CD2	1.21	1.57
1:A:605:GLU:HG3	1:A:632:PHE:CD2	1.37	1.57
2:B:291:TYR:HD2	2:B:294:VAL:CG1	1.18	1.57
2:B:549:LEU:HD21	2:B:611:ALA:CA	1.29	1.56
2:B:275:ARG:HB2	2:B:294:VAL:CG1	1.32	1.56
1:A:630:PRO:CG	2:B:614:ILE:HG12	1.18	1.56
2:B:87:VAL:HG13	2:B:122:SER:CB	1.17	1.56
1:A:384:LEU:HD22	1:A:441:TYR:CE2	1.41	1.55
2:B:472:VAL:HG11	2:B:510:GLY:CA	1.35	1.55
2:B:523:PHE:CZ	2:B:580:TYR:HD2	1.21	1.55
2:B:275:ARG:CG	2:B:294:VAL:HG11	1.24	1.55
2:B:70:MET:CE	2:B:107:ARG:HB2	1.11	1.55
2:B:162:VAL:CG2	2:B:195:ILE:HG23	1.29	1.55
3:M:223:HIS:HA	3:M:479:PHE:CD2	1.38	1.55
4:S:73:ILE:CG2	4:S:88:ILE:HG23	1.37	1.55
2:B:24:ALA:HB3	2:B:35:TYR:CE2	1.41	1.54
1:A:633:PHE:CG	2:B:550:VAL:HG12	1.03	1.54
2:B:216:LYS:HA	2:B:251:LEU:CD1	1.32	1.54
2:B:513:TRP:HA	2:B:551:LEU:CD2	1.37	1.54
2:B:267:ASP:H	2:B:289:PRO:CB	1.15	1.54
3:M:69:ILE:CD1	3:M:90:PHE:CZ	1.87	1.54
1:A:217:ALA:HA	4:S:142:ILE:CB	1.06	1.54
1:A:595:GLU:HG3	2:B:469:ASP:CB	1.18	1.54
1:A:217:ALA:HB1	4:S:142:ILE:CD1	1.34	1.53
2:B:556:LEU:CA	2:B:588:ILE:HD11	1.34	1.53
2:B:127:LEU:CB	2:B:157:THR:HG23	1.35	1.53
2:B:162:VAL:CG2	2:B:195:ILE:CG2	1.85	1.53
2:B:219:TYR:CZ	2:B:226:LEU:CA	1.92	1.53
2:B:127:LEU:CD2	2:B:161:LEU:HD21	1.27	1.53
3:M:101:LEU:HG	3:M:106:LYS:CA	1.30	1.52
2:B:83:PHE:CE2	2:B:119:SER:CB	1.91	1.52
2:B:158:VAL:HG11	2:B:177:ILE:CG1	1.11	1.52
1:A:630:PRO:HG3	2:B:614:ILE:CG1	1.07	1.51
4:S:5:VAL:CG2	4:S:132:LEU:HD21	1.05	1.51

Continued on next page...

Continued from previous page...

Atom-1	Atom-2	Interatomic distance (Å)	Clash overlap (Å)
2:B:219:TYR:CE1	2:B:226:LEU:HB2	1.34	1.51
2:B:537:PHE:HB3	2:B:598:LEU:CD1	1.35	1.51
2:B:353:GLN:HG3	3:M:47:SER:C	1.20	1.51
2:B:549:LEU:CD2	2:B:611:ALA:N	1.72	1.50
2:B:139:LEU:CD2	2:B:173:VAL:HA	1.07	1.50
2:B:291:TYR:CD2	2:B:294:VAL:CG1	1.93	1.50
2:B:513:TRP:N	2:B:551:LEU:CD1	1.71	1.50
2:B:537:PHE:CD2	2:B:598:LEU:HB3	1.47	1.50
1:A:128:LEU:HD13	1:A:150:LEU:CD2	1.38	1.50
2:B:47:LEU:HD22	2:B:66:ILE:CG1	1.06	1.50
2:B:219:TYR:CB	2:B:223:LEU:HD23	1.42	1.50
2:B:47:LEU:CD2	2:B:66:ILE:HG13	1.36	1.49
2:B:208:ILE:CD1	2:B:236:ILE:HG21	1.42	1.49
2:B:219:TYR:CD1	2:B:226:LEU:HB2	1.46	1.49
3:M:283:PHE:CZ	3:M:289:THR:OG1	1.65	1.49
2:B:215:TYR:CD1	2:B:233:TYR:HE1	1.29	1.49
2:B:275:ARG:CB	2:B:294:VAL:HG11	1.39	1.49
2:B:278:PRO:HA	2:B:288:TYR:C	1.21	1.49
1:A:220:SER:HB3	4:S:142:ILE:CG2	1.36	1.49
2:B:127:LEU:HD22	2:B:161:LEU:CD2	1.38	1.49
4:S:163:THR:CA	4:S:163:THR:C	1.81	1.49
1:A:179:LYS:CE	4:S:143:GLU:HB2	1.40	1.48
2:B:223:LEU:CD1	2:B:259:TYR:CA	1.90	1.48
1:A:128:LEU:CD1	1:A:150:LEU:HD21	1.44	1.48
1:A:204:VAL:HG22	1:A:236:LEU:CD2	1.43	1.48
2:B:178:ILE:HG13	2:B:214:ALA:C	1.17	1.48
2:B:208:ILE:HD13	2:B:236:ILE:CG2	1.01	1.48
2:B:219:TYR:CZ	2:B:226:LEU:HB2	1.49	1.48
2:B:559:ASP:HB2	2:B:563:PHE:CD1	1.46	1.48
1:A:408:ILE:CG2	4:S:64:ASN:C	1.81	1.48
2:B:537:PHE:CB	2:B:598:LEU:HD13	1.43	1.48
1:A:408:ILE:HG22	4:S:65:ASN:N	1.20	1.47
2:B:275:ARG:CB	2:B:294:VAL:CG1	1.90	1.47
1:A:128:LEU:CD1	1:A:150:LEU:CD2	1.91	1.47
1:A:212:ILE:CD1	4:S:145:ASN:ND2	1.73	1.47
2:B:303:LEU:CD1	2:B:333:GLN:HB3	1.41	1.47
2:B:337:THR:HA	2:B:373:LEU:CD2	1.44	1.47
2:B:433:VAL:CG1	2:B:474:VAL:HG21	1.43	1.47
2:B:70:MET:HE1	2:B:107:ARG:CB	1.43	1.47
2:B:523:PHE:CZ	2:B:580:TYR:CD2	1.96	1.47
2:B:252:LEU:CD1	2:B:302:PHE:CD1	1.97	1.46

Continued on next page...

Continued from previous page...

Atom-1	Atom-2	Interatomic distance (Å)	Clash overlap (Å)
1:A:100:LEU:H	4:S:162:SER:CB	1.25	1.46
1:A:105:VAL:CG2	4:S:167:ILE:CD1	1.91	1.46
2:B:70:MET:CE	2:B:107:ARG:CB	1.93	1.46
2:B:230:PHE:CE1	2:B:252:LEU:HD23	1.50	1.46
2:B:20:ARG:NH1	2:B:21:GLU:HG3	1.23	1.46
2:B:260:LEU:CB	2:B:291:TYR:CE1	1.88	1.46
1:A:633:PHE:CG	2:B:550:VAL:CG1	1.97	1.46
2:B:83:PHE:CZ	2:B:119:SER:CB	1.91	1.46
2:B:219:TYR:CG	2:B:255:TYR:HE1	1.33	1.46
3:M:215:TYR:CG	3:M:468:LYS:HA	1.51	1.46
2:B:106:LEU:HD13	2:B:144:ASP:CB	1.45	1.45
1:A:211:ASP:OD1	4:S:148:ARG:CD	1.65	1.45
1:A:408:ILE:HG23	4:S:64:ASN:CB	1.01	1.45
4:S:53:THR:C	4:S:69:ASN:HB2	1.34	1.45
2:B:127:LEU:CD1	2:B:157:THR:HG21	0.98	1.45
3:M:101:LEU:CG	3:M:106:LYS:HA	1.47	1.45
2:B:230:PHE:CZ	2:B:252:LEU:CD2	1.97	1.45
1:A:147:LEU:HD22	1:A:166:LEU:CD2	1.46	1.44
1:A:408:ILE:CG2	4:S:64:ASN:CB	1.96	1.44
2:B:247:TYR:CE2	3:M:91:THR:HG21	1.50	1.44
2:B:311:TYR:HE2	2:B:342:ALA:CB	1.30	1.44
3:M:104:PHE:CE2	3:M:113:LYS:NZ	1.83	1.44
2:B:527:PRO:CB	2:B:587:ARG:HG3	1.47	1.44
2:B:216:LYS:CA	2:B:251:LEU:HD13	1.44	1.44
2:B:549:LEU:CD2	2:B:611:ALA:CA	1.96	1.44
2:B:219:TYR:CE1	2:B:226:LEU:CD1	1.99	1.43
4:S:109:LEU:HD12	4:S:113:PHE:CD1	1.48	1.43
1:A:96:SER:N	1:A:127:LEU:CD2	1.78	1.43
1:A:101:GLN:HG3	4:S:160:ALA:CB	1.44	1.43
2:B:178:ILE:HG13	2:B:215:TYR:N	1.30	1.43
2:B:566:ALA:O	2:B:574:ASN:CB	1.65	1.43
1:A:100:LEU:N	4:S:162:SER:HB2	1.24	1.43
1:A:107:TYR:CE2	1:A:128:LEU:HD21	1.49	1.43
4:S:109:LEU:CD1	4:S:113:PHE:CD1	2.00	1.43
1:A:185:LEU:CD1	1:A:203:PHE:HE1	1.30	1.42
2:B:219:TYR:CE1	2:B:226:LEU:HD13	1.53	1.42
2:B:260:LEU:CD2	2:B:291:TYR:OH	1.66	1.42
2:B:279:LEU:N	2:B:288:TYR:HB2	1.10	1.42
2:B:549:LEU:CD2	2:B:611:ALA:CB	1.96	1.42
2:B:87:VAL:CG1	2:B:122:SER:CB	1.79	1.42
2:B:106:LEU:CD1	2:B:144:ASP:CB	1.95	1.42

Continued on next page...

Continued from previous page...

Atom-1	Atom-2	Interatomic distance (Å)	Clash overlap (Å)
2:B:219:TYR:CD1	2:B:226:LEU:HD22	1.52	1.42
1:A:212:ILE:CD1	4:S:145:ASN:HD21	1.25	1.42
2:B:20:ARG:CD	2:B:21:GLU:HB2	1.49	1.42
1:A:629:LEU:CD1	2:B:610:ARG:NH1	1.80	1.42
2:B:311:TYR:CE2	2:B:342:ALA:HB2	1.54	1.42
2:B:546:CYS:CB	2:B:607:ILE:HG12	0.94	1.42
3:M:245:ASP:N	3:M:472:TYR:CD1	1.87	1.42
2:B:309:LEU:HB3	2:B:317:VAL:CG1	1.48	1.41
1:A:594:PHE:HB3	2:B:473:ASN:CB	1.49	1.41
1:A:631:SER:CB	2:B:557:SER:OG	1.66	1.41
2:B:20:ARG:NH1	2:B:21:GLU:CG	1.83	1.41
2:B:422:ALA:HB3	2:B:424:PHE:CE1	1.53	1.41
3:M:69:ILE:HD12	3:M:90:PHE:CZ	1.49	1.41
1:A:408:ILE:HG22	4:S:64:ASN:C	1.37	1.41
2:B:108:PHE:CZ	2:B:115:LEU:HG	1.52	1.41
2:B:546:CYS:HB2	2:B:607:ILE:CD1	1.48	1.41
2:B:546:CYS:CB	2:B:607:ILE:CG1	1.83	1.41
2:B:29:LYS:HE2	2:B:30:LEU:N	1.32	1.41
3:M:65:TYR:CZ	3:M:86:PRO:HB3	1.52	1.41
1:A:250:ASN:OD1	1:A:285:THR:CG2	1.67	1.40
2:B:319:LEU:CD1	2:B:358:MET:HG3	0.93	1.40
1:A:215:VAL:CG2	1:A:243:ILE:HD12	1.49	1.40
2:B:549:LEU:CD2	2:B:611:ALA:HB2	1.49	1.40
4:S:73:ILE:CG2	4:S:88:ILE:CG2	1.98	1.40
1:A:111:SER:CB	1:A:152:THR:OG1	1.67	1.40
4:S:17:VAL:HG21	4:S:19:PHE:CZ	1.54	1.40
1:A:102:GLN:HG2	4:S:166:LYS:N	1.13	1.40
2:B:352:ASN:CB	3:M:49:ASP:OD2	1.69	1.40
2:B:422:ALA:HB3	2:B:424:PHE:CD1	1.57	1.40
3:M:319:SER:CB	3:M:346:ASN:HB2	1.48	1.40
1:A:450:TYR:OH	1:A:476:GLN:CG	1.66	1.39
2:B:158:VAL:CG1	2:B:177:ILE:CG1	2.01	1.39
3:M:260:LEU:CD2	3:M:449:VAL:HG22	1.50	1.39
1:A:633:PHE:CB	2:B:550:VAL:HG12	1.52	1.39
3:M:222:PHE:O	3:M:479:PHE:CE2	1.75	1.39
2:B:215:TYR:CD1	2:B:233:TYR:CE1	2.11	1.39
4:S:109:LEU:CD1	4:S:113:PHE:CE1	2.04	1.39
1:A:217:ALA:C	4:S:142:ILE:HB	1.43	1.38
2:B:87:VAL:CG1	2:B:122:SER:HB3	0.92	1.38
2:B:170:ARG:HA	2:B:199:LEU:CD2	1.53	1.38
3:M:281:GLY:O	3:M:282:VAL:CG2	1.72	1.38

Continued on next page...

Continued from previous page...

Atom-1	Atom-2	Interatomic distance (Å)	Clash overlap (Å)
1:A:288:THR:CG2	1:A:322:PHE:CZ	2.06	1.38
1:A:633:PHE:CD2	2:B:550:VAL:C	1.97	1.38
2:B:62:ALA:O	2:B:66:ILE:CG1	1.70	1.38
2:B:171:GLY:CA	2:B:207:VAL:CG1	2.01	1.38
2:B:549:LEU:HD22	2:B:611:ALA:CB	1.50	1.38
2:B:223:LEU:HD13	2:B:259:TYR:CB	1.53	1.37
1:A:142:LYS:N	4:S:159:ALA:HB2	1.33	1.37
2:B:215:TYR:HD1	2:B:233:TYR:CE1	1.43	1.37
2:B:343:LEU:HD12	2:B:359:LEU:CD1	1.55	1.37
2:B:261:PRO:HG2	2:B:292:GLU:N	1.08	1.37
3:M:290:PHE:CE1	3:M:297:PHE:CD1	2.13	1.37
1:A:595:GLU:CG	2:B:469:ASP:HB3	1.53	1.37
1:A:629:LEU:HD11	2:B:610:ARG:NH1	1.09	1.36
2:B:123:LEU:HD12	2:B:142:LEU:CD2	1.52	1.36
2:B:230:PHE:CZ	2:B:252:LEU:HD22	1.57	1.36
3:M:443:SER:HB3	3:M:447:ILE:CG1	1.55	1.36
2:B:106:LEU:CD1	2:B:144:ASP:HB3	1.54	1.36
2:B:353:GLN:NE2	3:M:47:SER:CB	1.86	1.36
3:M:223:HIS:HA	3:M:479:PHE:CE2	1.58	1.36
4:S:8:PHE:CZ	4:S:84:TYR:HB3	1.58	1.36
2:B:261:PRO:CG	2:B:292:GLU:H	1.37	1.36
1:A:96:SER:CB	1:A:127:LEU:HD11	1.54	1.35
1:A:288:THR:HG21	1:A:322:PHE:CE2	1.57	1.35
4:S:17:VAL:CG2	4:S:19:PHE:CZ	2.09	1.35
3:M:339:GLU:CD	3:M:412:ARG:HE	1.24	1.35
1:A:182:ILE:HG22	1:A:221:VAL:CG2	1.54	1.35
1:A:102:GLN:CA	4:S:167:ILE:HG12	1.57	1.35
2:B:181:TYR:CD2	2:B:218:CYS:O	1.79	1.35
3:M:215:TYR:CD2	3:M:469:GLY:N	1.91	1.35
1:A:107:TYR:CE2	1:A:128:LEU:CD2	2.07	1.34
1:A:136:GLY:O	1:A:139:ASP:HB3	1.24	1.34
2:B:208:ILE:CD1	2:B:236:ILE:CG2	1.94	1.34
2:B:393:ILE:CG2	2:B:431:MET:HG2	1.54	1.34
1:A:219:VAL:O	1:A:259:LEU:HD11	1.26	1.34
1:A:224:GLU:CB	4:S:138:GLY:O	1.73	1.34
2:B:219:TYR:CD1	2:B:226:LEU:CG	2.09	1.34
4:S:53:THR:HB	4:S:69:ASN:N	1.02	1.34
2:B:223:LEU:HD13	2:B:259:TYR:CA	1.53	1.34
3:M:245:ASP:O	3:M:472:TYR:CE1	1.80	1.34
1:A:291:ILE:CG2	1:A:318:ARG:HB3	1.57	1.33
1:A:403:LEU:HD21	1:A:421:PRO:C	1.48	1.33

Continued on next page...

Continued from previous page...

Atom-1	Atom-2	Interatomic distance (Å)	Clash overlap (Å)
2:B:197:LYS:HA	2:B:229:HIS:CD2	1.61	1.33
4:S:8:PHE:CB	4:S:36:TYR:HE2	1.36	1.33
1:A:105:VAL:CG2	4:S:167:ILE:HD12	1.47	1.33
1:A:185:LEU:CD1	1:A:203:PHE:CE1	2.09	1.33
1:A:255:ARG:NH2	4:S:135:ILE:CG2	1.92	1.33
3:M:101:LEU:O	3:M:106:LYS:N	1.58	1.33
2:B:140:SER:HB2	2:B:172:GLU:CD	1.46	1.33
2:B:252:LEU:CD1	2:B:302:PHE:CE1	2.10	1.33
2:B:566:ALA:HA	2:B:574:ASN:CB	1.55	1.33
1:A:520:GLY:CA	1:A:558:VAL:HG22	1.56	1.33
2:B:353:GLN:NE2	3:M:47:SER:HB3	1.00	1.33
2:B:559:ASP:CB	2:B:563:PHE:CD1	2.08	1.33
1:A:463:ASP:O	2:B:1:MET:HG3	1.15	1.33
2:B:546:CYS:HA	2:B:607:ILE:CG2	1.58	1.33
4:S:53:THR:CB	4:S:69:ASN:N	1.90	1.33
1:A:141:VAL:HG12	4:S:159:ALA:CB	1.59	1.32
2:B:106:LEU:CD1	2:B:144:ASP:O	1.73	1.32
2:B:519:ALA:O	2:B:523:PHE:CB	1.74	1.32
2:B:2:VAL:HG12	2:B:6:HIS:NE2	1.21	1.32
2:B:41:ASN:HB3	2:B:43:ASN:OD1	1.23	1.32
2:B:219:TYR:CD1	2:B:226:LEU:CD2	2.11	1.32
2:B:393:ILE:HG23	2:B:431:MET:CB	1.56	1.32
2:B:230:PHE:CE2	2:B:298:ASP:O	1.80	1.32
2:B:556:LEU:CD2	2:B:588:ILE:HG12	1.57	1.32
2:B:193:LEU:O	2:B:195:ILE:N	1.60	1.32
2:B:219:TYR:CG	2:B:255:TYR:CE1	2.17	1.32
3:M:244:VAL:CA	3:M:472:TYR:CD2	2.09	1.32
1:A:103:LYS:HE3	1:A:131:ASP:OD1	1.19	1.32
2:B:352:ASN:CA	3:M:49:ASP:OD2	1.76	1.32
2:B:143:SER:C	2:B:179:LYS:CD	1.97	1.31
2:B:519:ALA:O	2:B:523:PHE:HB3	1.16	1.31
3:M:67:SER:OG	3:M:90:PHE:CD1	1.71	1.31
1:A:138:ASN:OD1	4:S:158:LYS:CE	1.77	1.31
1:A:255:ARG:NH2	4:S:135:ILE:HG23	1.41	1.31
3:M:104:PHE:CE1	3:M:113:LYS:NZ	1.84	1.31
3:M:125:PHE:O	3:M:129:VAL:CG2	1.77	1.31
2:B:62:ALA:O	2:B:66:ILE:CD1	1.79	1.31
2:B:274:PRO:HG2	2:B:295:ASN:CG	1.45	1.31
2:B:343:LEU:CD2	2:B:362:ALA:HB3	1.60	1.31
3:M:454:ILE:CG2	3:M:464:THR:HG21	1.61	1.31
4:S:53:THR:CA	4:S:69:ASN:HB2	1.60	1.31

Continued on next page...

Continued from previous page...

Atom-1	Atom-2	Interatomic distance (Å)	Clash overlap (Å)
1:A:105:VAL:CB	4:S:167:ILE:HD13	1.59	1.31
1:A:140:VAL:CA	1:A:177:ILE:CG1	2.07	1.31
1:A:170:LEU:O	1:A:206:LYS:HD2	1.26	1.31
1:A:251:TRP:CH2	4:S:103:GLN:HB2	1.66	1.31
3:M:283:PHE:CE2	3:M:289:THR:OG1	1.81	1.31
1:A:105:VAL:N	4:S:167:ILE:CD1	1.92	1.31
1:A:176:TYR:CB	4:S:155:GLU:HG3	1.61	1.31
1:A:332:TYR:CD1	1:A:366:SER:OG	1.77	1.31
1:A:631:SER:CB	2:B:557:SER:CB	2.07	1.31
2:B:117:LEU:HD21	2:B:149:SER:OG	1.25	1.31
2:B:230:PHE:HE2	2:B:298:ASP:O	1.06	1.31
1:A:429:VAL:CB	1:A:469:LEU:HD11	1.59	1.30
1:A:633:PHE:CD1	2:B:513:TRP:CZ3	2.19	1.30
2:B:123:LEU:CD1	2:B:142:LEU:CG	2.08	1.30
2:B:278:PRO:CA	2:B:288:TYR:C	1.99	1.30
3:M:65:TYR:CE1	3:M:86:PRO:HB3	1.63	1.30
1:A:252:ILE:HA	4:S:144:THR:OG1	1.13	1.30
2:B:158:VAL:HG11	2:B:177:ILE:CD1	1.61	1.30
2:B:181:TYR:HD2	2:B:218:CYS:C	1.32	1.30
2:B:216:LYS:CA	2:B:251:LEU:CD1	2.01	1.30
3:M:218:LEU:HA	3:M:472:TYR:CE2	1.67	1.30
4:S:8:PHE:CB	4:S:36:TYR:CE2	2.13	1.30
4:S:8:PHE:HB2	4:S:36:TYR:CE2	1.66	1.30
2:B:140:SER:CB	2:B:172:GLU:OE1	1.80	1.30
2:B:559:ASP:O	2:B:563:PHE:N	1.64	1.30
3:M:60:LEU:HD22	3:M:62:VAL:CG2	1.60	1.30
3:M:317:MET:HB3	3:M:320:ILE:O	1.13	1.30
1:A:384:LEU:CD2	1:A:441:TYR:CE2	2.14	1.29
2:B:274:PRO:C	2:B:295:ASN:HD21	1.35	1.29
3:M:217:ASP:CB	3:M:470:ALA:C	2.00	1.29
2:B:170:ARG:NH1	2:B:198:GLU:HG2	1.45	1.29
2:B:307:ASN:HD21	2:B:336:ASN:ND2	1.27	1.29
1:A:275:LEU:HD13	1:A:308:ASP:OD1	1.23	1.29
1:A:633:PHE:CD2	2:B:551:LEU:N	2.01	1.29
1:A:633:PHE:CD1	2:B:550:VAL:HG12	1.68	1.29
2:B:513:TRP:N	2:B:551:LEU:HD13	0.97	1.29
2:B:566:ALA:CA	2:B:574:ASN:HB3	1.62	1.29
1:A:128:LEU:HD13	1:A:150:LEU:CG	1.62	1.28
2:B:37:TYR:OH	2:B:46:GLN:CD	1.71	1.28
3:M:221:THR:CB	3:M:474:THR:O	1.81	1.28
4:S:39:ILE:HD11	4:S:77:TYR:CD2	1.68	1.28

Continued on next page...

Continued from previous page...

Atom-1	Atom-2	Interatomic distance (Å)	Clash overlap (Å)
1:A:147:LEU:CD1	1:A:181:ALA:HA	1.62	1.28
2:B:24:ALA:HB3	2:B:35:TYR:CZ	1.66	1.28
2:B:279:LEU:H	2:B:288:TYR:CB	1.44	1.28
2:B:418:TYR:OH	2:B:432:ALA:HB2	1.18	1.28
1:A:633:PHE:CD1	2:B:550:VAL:CG1	2.14	1.28
2:B:77:ILE:O	2:B:79:VAL:N	1.64	1.28
2:B:181:TYR:HD2	2:B:218:CYS:O	0.95	1.28
2:B:227:HIS:O	2:B:229:HIS:N	1.64	1.28
2:B:546:CYS:SG	2:B:607:ILE:HG12	1.71	1.28
3:M:241:HIS:O	3:M:474:THR:HB	1.29	1.28
3:M:319:SER:HB3	3:M:346:ASN:N	1.48	1.28
4:S:9:ASN:OD1	4:S:13:GLN:N	1.63	1.28
1:A:403:LEU:HD22	1:A:422:GLU:CG	1.64	1.28
2:B:116:THR:CG2	2:B:150:LEU:HD11	1.62	1.28
3:M:379:LEU:HD22	3:M:386:PHE:CG	1.65	1.28
1:A:633:PHE:CE2	2:B:551:LEU:N	1.98	1.28
2:B:226:LEU:HB3	2:B:255:TYR:OH	1.21	1.28
2:B:563:PHE:HD2	2:B:584:SER:CB	1.45	1.28
2:B:274:PRO:HG2	2:B:295:ASN:OD1	1.13	1.27
1:A:463:ASP:O	2:B:1:MET:CG	1.80	1.27
2:B:315:PRO:HB3	2:B:352:ASN:OD1	1.34	1.27
3:M:449:VAL:HG11	3:M:452:ILE:CD1	1.63	1.27
2:B:140:SER:HB2	2:B:172:GLU:OE1	1.10	1.27
2:B:225:LEU:HD12	2:B:283:TYR:OH	1.21	1.27
3:M:339:GLU:HG3	3:M:412:ARG:CG	1.63	1.27
1:A:516:ILE:HG22	1:A:554:ALA:CB	1.63	1.27
2:B:549:LEU:CD1	2:B:611:ALA:HA	1.63	1.27
2:B:127:LEU:CD1	2:B:157:THR:CG2	1.84	1.27
2:B:353:GLN:HG3	3:M:47:SER:O	1.26	1.27
2:B:512:VAL:C	2:B:551:LEU:HD13	1.55	1.27
2:B:513:TRP:CA	2:B:551:LEU:CD1	2.10	1.27
3:M:66:PHE:CA	3:M:77:LEU:HD11	1.63	1.27
3:M:443:SER:CB	3:M:447:ILE:HG13	1.65	1.27
2:B:162:VAL:HG22	2:B:199:LEU:CG	1.66	1.26
2:B:316:THR:CG2	3:M:90:PHE:CE2	2.15	1.26
2:B:252:LEU:HD12	2:B:302:PHE:CE1	1.65	1.26
2:B:278:PRO:CG	2:B:292:GLU:OE1	1.83	1.26
2:B:337:THR:CA	2:B:373:LEU:HD21	1.64	1.26
2:B:21:GLU:HA	2:B:24:ALA:CB	1.66	1.26
2:B:29:LYS:CE	2:B:30:LEU:H	1.48	1.26
2:B:123:LEU:HD13	2:B:142:LEU:CG	1.64	1.26

Continued on next page...

Continued from previous page...

Atom-1	Atom-2	Interatomic distance (Å)	Clash overlap (Å)
2:B:219:TYR:CB	2:B:255:TYR:CE1	2.16	1.26
3:M:268:GLY:N	3:M:302:TYR:OH	1.69	1.26
2:B:143:SER:C	2:B:179:LYS:HD2	1.50	1.25
2:B:566:ALA:C	2:B:574:ASN:CB	2.04	1.25
4:S:14:PRO:HA	4:S:36:TYR:OH	1.33	1.25
1:A:250:ASN:OD1	1:A:285:THR:HG22	1.20	1.25
1:A:633:PHE:CE1	2:B:513:TRP:CZ3	2.24	1.25
1:A:96:SER:CA	1:A:127:LEU:HD21	1.65	1.25
1:A:204:VAL:CG2	1:A:236:LEU:HD21	1.64	1.25
3:M:342:LEU:HD11	3:M:411:LEU:CD2	1.65	1.25
1:A:141:VAL:CG1	4:S:159:ALA:HB3	1.67	1.25
2:B:219:TYR:CG	2:B:226:LEU:HB2	1.72	1.25
2:B:245:GLN:CD	2:B:309:LEU:CD1	2.05	1.25
3:M:101:LEU:HA	3:M:109:LEU:CD1	1.65	1.25
3:M:224:VAL:H	3:M:479:PHE:CB	1.50	1.25
1:A:138:ASN:OD1	4:S:158:LYS:HE3	1.08	1.25
2:B:553:ALA:HB2	2:B:614:ILE:CD1	1.63	1.25
2:B:556:LEU:HD23	2:B:588:ILE:CG1	1.66	1.25
3:M:217:ASP:HB2	3:M:470:ALA:O	1.33	1.25
3:M:218:LEU:HA	3:M:472:TYR:CD2	1.72	1.25
3:M:241:HIS:O	3:M:474:THR:CB	1.83	1.25
3:M:260:LEU:HD23	3:M:449:VAL:CG2	1.67	1.25
1:A:102:GLN:HB2	4:S:163:THR:CB	1.65	1.25
1:A:399:ASP:O	1:A:420:ILE:O	1.55	1.25
1:A:633:PHE:CD2	2:B:551:LEU:CA	2.07	1.25
2:B:25:VAL:HG11	2:B:36:THR:OG1	1.34	1.25
2:B:225:LEU:HD13	2:B:283:TYR:CE1	1.70	1.25
2:B:291:TYR:CE2	2:B:294:VAL:HB	1.69	1.25
3:M:254:PRO:HB3	3:M:454:ILE:CD1	1.65	1.25
1:A:103:LYS:H	4:S:163:THR:CB	1.49	1.24
2:B:219:TYR:CZ	2:B:226:LEU:N	2.03	1.24
4:S:53:THR:CG2	4:S:67:GLU:O	1.85	1.24
1:A:96:SER:CA	1:A:127:LEU:HD11	1.66	1.24
1:A:220:SER:CB	4:S:142:ILE:HG22	1.65	1.24
2:B:158:VAL:CG1	2:B:177:ILE:HD11	1.66	1.24
2:B:159:LYS:HA	2:B:195:ILE:CD1	1.67	1.24
2:B:353:GLN:CG	3:M:47:SER:C	2.06	1.24
3:M:272:LEU:CD2	3:M:278:ILE:HB	1.67	1.24
2:B:143:SER:O	2:B:179:LYS:CD	1.85	1.24
2:B:178:ILE:CG1	2:B:214:ALA:C	2.05	1.24
4:S:56:SER:O	4:S:60:SER:CB	1.86	1.24

Continued on next page...

Continued from previous page...

Atom-1	Atom-2	Interatomic distance (Å)	Clash overlap (Å)
3:M:219:LEU:CB	3:M:472:TYR:O	1.85	1.24
3:M:244:VAL:HG13	3:M:472:TYR:CE2	1.72	1.24
3:M:449:VAL:CG1	3:M:452:ILE:CD1	2.14	1.24
1:A:101:GLN:CG	4:S:160:ALA:HB1	1.56	1.24
1:A:513:ARG:CD	1:A:550:VAL:HG21	1.65	1.24
1:A:637:GLU:CG	2:B:516:GLY:H	1.49	1.24
4:S:48:SER:CB	4:S:77:TYR:C	2.06	1.24
1:A:516:ILE:CG2	1:A:554:ALA:HB3	1.67	1.23
1:A:631:SER:HB2	2:B:557:SER:OG	1.23	1.23
2:B:24:ALA:CB	2:B:35:TYR:CZ	2.21	1.23
2:B:37:TYR:HD2	2:B:38:TYR:CD1	1.55	1.23
2:B:162:VAL:HG23	2:B:195:ILE:CG2	1.51	1.23
2:B:199:LEU:O	2:B:201:ALA:N	1.71	1.23
3:M:432:THR:OG1	3:M:480:GLN:CG	1.86	1.23
4:S:53:THR:HB	4:S:69:ASN:CA	1.66	1.23
2:B:86:VAL:CG1	2:B:101:ILE:HG23	1.66	1.23
2:B:223:LEU:CD1	2:B:259:TYR:N	2.01	1.23
2:B:584:SER:O	2:B:588:ILE:HG22	1.08	1.23
3:M:217:ASP:CB	3:M:470:ALA:O	1.85	1.23
4:S:8:PHE:CD1	4:S:84:TYR:HB2	1.71	1.23
1:A:147:LEU:HB3	1:A:184:ALA:CB	1.69	1.23
1:A:326:GLN:HA	1:A:331:ARG:NH2	1.54	1.23
1:A:557:LYS:HE2	2:B:606:ASP:N	0.92	1.23
2:B:143:SER:O	2:B:179:LYS:HD3	1.09	1.23
2:B:219:TYR:CZ	2:B:226:LEU:CB	2.01	1.23
2:B:278:PRO:CB	2:B:288:TYR:O	1.85	1.23
3:M:378:ILE:O	3:M:413:GLY:HA3	1.12	1.23
4:S:8:PHE:CZ	4:S:84:TYR:CB	2.16	1.23
1:A:217:ALA:CB	4:S:142:ILE:HD12	1.68	1.23
2:B:261:PRO:CG	2:B:292:GLU:N	1.98	1.23
2:B:277:CYS:O	2:B:288:TYR:HB3	1.06	1.23
3:M:66:PHE:CB	3:M:77:LEU:HD11	1.43	1.23
2:B:212:VAL:CG2	2:B:248:LEU:HD21	1.69	1.22
4:S:47:GLN:NE2	4:S:79:ASN:N	1.86	1.22
1:A:88:ASN:CB	1:A:120:ILE:HD12	1.68	1.22
1:A:212:ILE:HD12	4:S:145:ASN:ND2	0.91	1.22
1:A:291:ILE:HG23	1:A:318:ARG:CB	1.69	1.22
1:A:609:LEU:HG	1:A:628:VAL:CB	1.67	1.22
2:B:216:LYS:CB	2:B:251:LEU:CD1	2.16	1.22
2:B:472:VAL:CG1	2:B:510:GLY:HA3	1.68	1.22
3:M:214:LEU:C	3:M:467:TYR:HB3	1.57	1.22

Continued on next page...

Continued from previous page...

Atom-1	Atom-2	Interatomic distance (Å)	Clash overlap (Å)
1:A:563:CYS:CB	1:A:621:LEU:HD12	1.68	1.22
2:B:20:ARG:CZ	2:B:21:GLU:HG3	1.68	1.22
2:B:219:TYR:CD1	2:B:226:LEU:HD13	1.74	1.22
2:B:275:ARG:N	2:B:295:ASN:ND2	1.87	1.22
2:B:479:VAL:HG22	2:B:486:HIS:CD2	1.73	1.22
3:M:221:THR:OG1	3:M:474:THR:O	1.56	1.22
1:A:92:LEU:HD21	1:A:120:ILE:O	1.38	1.22
2:B:135:ARG:NH2	2:B:164:ASP:OD1	1.72	1.22
2:B:219:TYR:CB	2:B:255:TYR:HE1	1.52	1.22
2:B:542:PRO:HA	2:B:602:ASP:OD2	1.29	1.22
4:S:48:SER:CB	4:S:77:TYR:CB	2.18	1.22
4:S:73:ILE:HG21	4:S:88:ILE:CG2	1.65	1.22
1:A:96:SER:CB	1:A:127:LEU:CD1	2.16	1.21
2:B:171:GLY:N	2:B:207:VAL:HG13	1.53	1.21
2:B:223:LEU:CD2	2:B:255:TYR:CE1	2.23	1.21
4:S:131:VAL:CG2	4:S:153:VAL:HG22	1.69	1.21
1:A:638:LEU:HB2	2:B:516:GLY:O	1.36	1.21
2:B:20:ARG:NE	2:B:21:GLU:HB2	1.55	1.21
1:A:217:ALA:CB	4:S:142:ILE:CG1	2.19	1.21
1:A:219:VAL:CG1	1:A:259:LEU:HD13	1.70	1.21
2:B:231:ARG:HG3	2:B:298:ASP:OD1	1.39	1.21
2:B:277:CYS:O	2:B:288:TYR:CB	1.88	1.21
2:B:352:ASN:HA	3:M:49:ASP:CG	1.39	1.21
3:M:2:TYR:O	3:M:81:SER:HB2	1.38	1.21
1:A:105:VAL:CG2	4:S:167:ILE:HG23	1.69	1.21
1:A:213:SER:O	4:S:143:GLU:CG	1.87	1.21
1:A:219:VAL:O	1:A:259:LEU:CD1	1.87	1.21
1:A:557:LYS:NZ	2:B:604:GLU:CG	1.70	1.21
2:B:87:VAL:HG13	2:B:122:SER:OG	1.39	1.21
4:S:34:GLN:OE1	4:S:58:LEU:HD21	1.32	1.21
1:A:101:GLN:HE21	4:S:167:ILE:CG2	1.53	1.21
2:B:47:LEU:CD2	2:B:66:ILE:CG1	2.02	1.21
2:B:79:VAL:HB	2:B:108:PHE:CE1	1.74	1.21
2:B:105:LEU:HB3	2:B:145:MET:CE	1.71	1.21
2:B:219:TYR:CD1	2:B:226:LEU:CB	2.07	1.21
2:B:290:SER:O	2:B:292:GLU:N	1.67	1.21
2:B:219:TYR:HB3	2:B:255:TYR:CE1	1.76	1.20
4:S:15:ARG:NH1	4:S:122:ILE:HD11	1.54	1.20
1:A:101:GLN:C	4:S:167:ILE:HG13	1.59	1.20
1:A:140:VAL:CA	1:A:177:ILE:HG12	1.70	1.20
1:A:211:ASP:CG	4:S:148:ARG:HD3	1.60	1.20

Continued on next page...

Continued from previous page...

Atom-1	Atom-2	Interatomic distance (Å)	Clash overlap (Å)
1:A:557:LYS:HB3	2:B:605:PHE:CD2	1.76	1.20
2:B:124:GLN:HA	2:B:157:THR:OG1	1.38	1.20
2:B:344:VAL:HG13	2:B:381:PHE:CZ	1.76	1.20
2:B:531:ARG:HA	2:B:591:MET:SD	1.80	1.20
3:M:319:SER:CB	3:M:346:ASN:CB	2.18	1.20
1:A:125:THR:OG1	1:A:158:LEU:HD13	1.38	1.20
1:A:388:VAL:HG13	1:A:432:ILE:CD1	1.70	1.20
2:B:37:TYR:CD2	2:B:38:TYR:CD1	2.28	1.20
2:B:127:LEU:CB	2:B:157:THR:CG2	2.19	1.20
4:S:34:GLN:OE1	4:S:58:LEU:HD11	1.35	1.20
2:B:219:TYR:CE1	2:B:226:LEU:CG	2.22	1.20
2:B:278:PRO:HA	2:B:288:TYR:CA	1.69	1.20
3:M:222:PHE:CD1	3:M:240:ILE:HG23	1.75	1.20
1:A:96:SER:N	1:A:127:LEU:HD21	0.89	1.20
2:B:162:VAL:CB	2:B:195:ILE:HG23	1.72	1.20
2:B:245:GLN:CD	2:B:309:LEU:HD11	1.56	1.20
2:B:261:PRO:CA	2:B:290:SER:HB3	1.49	1.20
1:A:211:ASP:OD1	4:S:148:ARG:HD3	1.02	1.19
1:A:316:LEU:CD1	1:A:348:PHE:CD2	2.26	1.19
1:A:594:PHE:CB	2:B:473:ASN:HB2	1.72	1.19
2:B:62:ALA:O	2:B:66:ILE:HG13	1.21	1.19
2:B:219:TYR:CE2	2:B:226:LEU:HB2	1.75	1.19
2:B:513:TRP:CA	2:B:551:LEU:CD2	2.20	1.19
3:M:243:ILE:H	3:M:474:THR:CG2	1.53	1.19
4:S:47:GLN:NE2	4:S:79:ASN:H	1.38	1.19
1:A:559:PHE:CE1	1:A:581:LEU:HD22	1.76	1.19
1:A:631:SER:HA	2:B:557:SER:CB	1.71	1.19
2:B:25:VAL:HG23	2:B:35:TYR:CD2	1.77	1.19
2:B:267:ASP:N	2:B:289:PRO:CB	2.00	1.19
2:B:278:PRO:HG2	2:B:292:GLU:OE1	1.41	1.19
2:B:291:TYR:CD2	2:B:294:VAL:HG12	1.61	1.19
3:M:9:ASP:C	3:M:75:TRP:CD1	2.16	1.19
3:M:340:LEU:O	3:M:411:LEU:HB3	1.39	1.19
1:A:102:GLN:CB	4:S:166:LYS:HB2	1.71	1.19
2:B:197:LYS:O	2:B:199:LEU:N	1.74	1.19
2:B:279:LEU:N	2:B:288:TYR:CB	2.02	1.19
2:B:513:TRP:CA	2:B:551:LEU:HD13	1.66	1.19
1:A:217:ALA:HB2	4:S:143:GLU:HG3	1.23	1.19
2:B:291:TYR:CD2	2:B:294:VAL:HG11	1.68	1.19
2:B:311:TYR:CE2	2:B:342:ALA:CB	2.17	1.19
2:B:393:ILE:HG23	2:B:431:MET:CG	1.71	1.19

Continued on next page...

Continued from previous page...

Atom-1	Atom-2	Interatomic distance (Å)	Clash overlap (Å)
2:B:546:CYS:CA	2:B:607:ILE:HG23	1.72	1.19
3:M:246:VAL:HB	3:M:297:PHE:CZ	1.75	1.19
1:A:185:LEU:HD12	1:A:203:PHE:CE1	1.74	1.19
1:A:605:GLU:CG	1:A:632:PHE:CD2	2.26	1.19
2:B:108:PHE:CE2	2:B:115:LEU:HB2	1.78	1.19
2:B:151:ALA:O	2:B:188:TYR:CE2	1.85	1.19
2:B:223:LEU:HD22	2:B:255:TYR:CE1	1.78	1.19
2:B:582:ASP:O	2:B:584:SER:HB3	1.43	1.19
1:A:101:GLN:CG	4:S:167:ILE:HG21	1.72	1.18
1:A:179:LYS:HE3	4:S:143:GLU:CB	1.73	1.18
2:B:223:LEU:CD1	2:B:259:TYR:HA	1.57	1.18
1:A:502:ASP:OD2	1:A:506:LYS:NZ	1.72	1.18
1:A:275:LEU:HD21	1:A:311:THR:OG1	1.41	1.18
2:B:25:VAL:CG1	2:B:36:THR:OG1	1.92	1.18
2:B:225:LEU:CD1	2:B:283:TYR:OH	1.91	1.18
2:B:267:ASP:N	2:B:289:PRO:HB3	1.53	1.18
2:B:277:CYS:SG	2:B:292:GLU:HG3	1.83	1.18
2:B:303:LEU:CD1	2:B:333:GLN:CB	2.22	1.18
2:B:531:ARG:CA	2:B:591:MET:SD	2.31	1.18
3:M:66:PHE:CB	3:M:77:LEU:CD1	2.21	1.18
3:M:319:SER:OG	3:M:346:ASN:CB	1.92	1.18
4:S:135:ILE:O	4:S:141:VAL:HA	1.40	1.18
2:B:422:ALA:CB	2:B:424:PHE:CE1	2.26	1.18
3:M:338:PHE:CD2	3:M:415:ILE:HG13	1.78	1.18
1:A:103:LYS:O	1:A:107:TYR:CD1	1.97	1.18
2:B:98:LYS:NZ	2:B:134:LEU:HB3	1.58	1.18
4:S:130:SER:OG	4:S:156:LEU:CD1	1.89	1.18
2:B:12:LEU:O	2:B:16:LYS:HB2	1.42	1.17
2:B:56:SER:CB	2:B:92:THR:HG21	1.74	1.17
2:B:315:PRO:HB3	2:B:355:ASN:ND2	1.58	1.17
2:B:567:GLN:O	2:B:569:THR:OG1	1.54	1.17
2:B:47:LEU:HD22	2:B:66:ILE:HG12	1.20	1.17
2:B:170:ARG:HH12	2:B:198:GLU:CG	1.57	1.17
3:M:244:VAL:HA	3:M:472:TYR:CE2	1.77	1.17
1:A:102:GLN:HB3	4:S:166:LYS:CB	1.74	1.17
1:A:166:LEU:CD1	1:A:185:LEU:HD23	1.73	1.17
1:A:631:SER:CA	2:B:557:SER:CB	2.21	1.17
2:B:352:ASN:CA	3:M:49:ASP:CG	2.10	1.17
3:M:222:PHE:C	3:M:479:PHE:CZ	2.18	1.17
1:A:253:ILE:HD11	1:A:281:LEU:HD22	1.20	1.17
1:A:399:ASP:CA	1:A:420:ILE:HB	1.74	1.17

Continued on next page...

Continued from previous page...

Atom-1	Atom-2	Interatomic distance (Å)	Clash overlap (Å)
1:A:595:GLU:CG	2:B:469:ASP:CB	2.14	1.17
2:B:171:GLY:HA3	2:B:207:VAL:CG1	1.70	1.17
2:B:252:LEU:HB2	2:B:302:PHE:CZ	1.78	1.17
2:B:360:LEU:CD1	2:B:391:ALA:HA	1.73	1.17
1:A:71:VAL:CG1	1:A:105:VAL:HG12	1.71	1.17
2:B:278:PRO:CD	2:B:292:GLU:OE1	1.91	1.17
2:B:523:PHE:HZ	2:B:580:TYR:CE2	1.63	1.17
2:B:545:ARG:HD3	2:B:602:ASP:HB2	1.25	1.17
3:M:226:PHE:HB2	3:M:481:VAL:HG22	1.17	1.17
3:M:336:ASP:OD1	3:M:415:ILE:O	1.62	1.17
1:A:105:VAL:N	4:S:167:ILE:HD11	1.53	1.16
1:A:185:LEU:HD13	1:A:203:PHE:CE1	1.71	1.16
1:A:557:LYS:HD3	2:B:605:PHE:HB3	1.24	1.16
1:A:631:SER:HA	2:B:557:SER:HB3	1.24	1.16
3:M:260:LEU:CD2	3:M:449:VAL:CG2	2.20	1.16
1:A:99:LYS:HD3	4:S:164:ASP:H	1.06	1.16
1:A:107:TYR:CD2	1:A:128:LEU:HD21	1.80	1.16
2:B:479:VAL:CG2	2:B:486:HIS:CD2	2.29	1.16
3:M:339:GLU:CD	3:M:412:ARG:NE	1.98	1.16
2:B:83:PHE:HE2	2:B:119:SER:CA	1.58	1.16
2:B:216:LYS:CG	2:B:251:LEU:HD13	1.76	1.16
2:B:497:LEU:HD23	2:B:533:LEU:CD2	1.75	1.16
3:M:215:TYR:CD1	3:M:468:LYS:CA	2.27	1.16
3:M:339:GLU:HG3	3:M:412:ARG:CD	1.74	1.16
4:S:47:GLN:CD	4:S:79:ASN:N	1.97	1.16
1:A:147:LEU:CD2	1:A:166:LEU:CD2	2.23	1.16
1:A:219:VAL:HG21	1:A:256:LEU:HD21	1.22	1.16
2:B:105:LEU:HB3	2:B:145:MET:HE3	1.22	1.16
3:M:219:LEU:HD13	3:M:472:TYR:O	1.45	1.16
4:S:80:TYR:O	4:S:82:THR:N	1.78	1.16
1:A:103:LYS:CE	1:A:131:ASP:OD1	1.94	1.16
1:A:213:SER:O	4:S:143:GLU:HG2	0.99	1.16
1:A:216:SER:HB3	4:S:143:GLU:HA	1.18	1.16
2:B:175:LEU:CG	2:B:210:CYS:HB3	1.75	1.16
2:B:275:ARG:CG	2:B:294:VAL:CG1	2.16	1.16
2:B:275:ARG:N	2:B:295:ASN:HD21	1.41	1.16
3:M:219:LEU:CD1	3:M:472:TYR:O	1.94	1.16
1:A:217:ALA:O	4:S:142:ILE:HB	1.43	1.15
1:A:421:PRO:HG2	1:A:424:TYR:CE1	1.81	1.15
3:M:92:PHE:CZ	3:M:129:VAL:HG22	1.79	1.15
3:M:379:LEU:CD2	3:M:386:PHE:CD1	2.28	1.15

Continued on next page...

Continued from previous page...

Atom-1	Atom-2	Interatomic distance (Å)	Clash overlap (Å)
1:A:219:VAL:C	1:A:259:LEU:CD1	2.13	1.15
2:B:219:TYR:CZ	2:B:226:LEU:HA	1.78	1.15
2:B:556:LEU:CD2	2:B:588:ILE:CG1	2.24	1.15
4:S:53:THR:HG21	4:S:67:GLU:O	1.44	1.15
1:A:103:LYS:H	4:S:163:THR:CG2	1.57	1.15
1:A:104:ARG:HA	1:A:145:ILE:HG21	1.22	1.15
1:A:147:LEU:CD2	1:A:166:LEU:HD23	1.77	1.15
2:B:80:GLN:HG2	2:B:115:LEU:HD11	1.18	1.15
2:B:151:ALA:HA	2:B:180:LEU:CD1	1.74	1.15
2:B:275:ARG:HG3	2:B:291:TYR:CD2	1.79	1.15
2:B:393:ILE:CG2	2:B:431:MET:CG	2.23	1.15
3:M:319:SER:HB3	3:M:346:ASN:CB	1.74	1.15
3:M:338:PHE:CE2	3:M:415:ILE:HG13	1.81	1.15
4:S:34:GLN:OE1	4:S:58:LEU:CD2	1.93	1.15
1:A:163:ALA:CB	1:A:195:ALA:HB1	1.76	1.15
1:A:630:PRO:CB	2:B:614:ILE:HG12	1.75	1.15
2:B:162:VAL:CG2	2:B:195:ILE:HG22	1.76	1.15
2:B:223:LEU:HD12	2:B:259:TYR:CA	1.64	1.15
2:B:230:PHE:CD2	2:B:298:ASP:HB3	1.81	1.15
3:M:443:SER:OG	3:M:447:ILE:C	1.84	1.15
1:A:102:GLN:HB2	4:S:163:THR:CA	1.77	1.15
1:A:147:LEU:HD12	1:A:181:ALA:HA	1.29	1.15
1:A:219:VAL:HG13	1:A:259:LEU:HD13	1.24	1.15
1:A:450:TYR:OH	1:A:476:GLN:HG2	1.34	1.15
1:A:450:TYR:OH	1:A:476:GLN:HG3	1.42	1.15
2:B:132:SER:HA	2:B:169:VAL:HG21	1.20	1.15
1:A:630:PRO:HG2	2:B:614:ILE:HA	1.22	1.14
1:A:634:ASN:O	2:B:516:GLY:C	1.86	1.14
2:B:158:VAL:CG1	2:B:177:ILE:CD1	2.17	1.14
2:B:353:GLN:CG	3:M:49:ASP:H	1.59	1.14
2:B:513:TRP:HA	2:B:551:LEU:CG	1.76	1.14
1:A:92:LEU:HD11	1:A:120:ILE:HA	1.20	1.14
1:A:140:VAL:N	1:A:177:ILE:HD11	1.61	1.14
1:A:288:THR:OG1	1:A:291:ILE:HB	1.43	1.14
1:A:609:LEU:CG	1:A:628:VAL:HB	1.78	1.14
3:M:217:ASP:CG	3:M:471:LYS:HA	1.67	1.14
3:M:340:LEU:O	3:M:411:LEU:CB	1.92	1.14
1:A:101:GLN:HE21	4:S:167:ILE:HG22	1.10	1.14
1:A:629:LEU:HD11	2:B:610:ARG:CZ	1.77	1.14
2:B:2:VAL:HG12	2:B:6:HIS:CE1	1.82	1.14
2:B:154:ILE:HD12	2:B:180:LEU:CD1	1.78	1.14

Continued on next page...

Continued from previous page...

Atom-1	Atom-2	Interatomic distance (Å)	Clash overlap (Å)
2:B:523:PHE:CE1	2:B:580:TYR:HD2	1.66	1.14
1:A:251:TRP:HH2	4:S:103:GLN:OE1	1.27	1.14
1:A:275:LEU:O	1:A:276:PRO:C	1.73	1.14
1:A:555:LEU:HD13	1:A:581:LEU:HD11	1.21	1.14
1:A:609:LEU:CD2	1:A:628:VAL:HG21	1.78	1.14
2:B:127:LEU:CD2	2:B:161:LEU:CD2	2.06	1.14
2:B:127:LEU:HD22	2:B:161:LEU:HD22	1.24	1.14
1:A:67:LYS:H	4:S:165:SER:HB2	1.00	1.14
1:A:176:TYR:HB3	4:S:155:GLU:HG3	1.28	1.14
1:A:638:LEU:HD12	2:B:518:ILE:CG2	1.78	1.14
2:B:260:LEU:CA	2:B:291:TYR:CE1	2.21	1.14
2:B:556:LEU:HD22	2:B:588:ILE:HG12	1.16	1.14
3:M:343:ASN:HA	3:M:408:VAL:HG13	1.21	1.14
1:A:178:ARG:HD3	1:A:209:ASP:OD2	1.48	1.13
1:A:217:ALA:CB	4:S:142:ILE:CD1	2.24	1.13
2:B:79:VAL:HG23	2:B:108:PHE:CZ	1.82	1.13
2:B:196:LEU:HB2	2:B:229:HIS:CE1	1.83	1.13
4:S:7:ILE:HG21	4:S:121:LEU:HD21	1.26	1.13
1:A:262:ASN:O	1:A:265:GLN:O	1.65	1.13
1:A:399:ASP:HA	1:A:420:ILE:CB	1.77	1.13
1:A:631:SER:O	2:B:554:LYS:HG3	1.49	1.13
2:B:219:TYR:CD1	2:B:226:LEU:CD1	2.27	1.13
2:B:275:ARG:HB3	2:B:291:TYR:HB3	1.19	1.13
2:B:400:SER:HB2	2:B:439:CYS:SG	1.87	1.13
3:M:283:PHE:CE2	3:M:289:THR:CB	2.31	1.13
3:M:319:SER:OG	3:M:346:ASN:HB2	0.99	1.13
1:A:637:GLU:HG2	2:B:516:GLY:H	1.11	1.13
2:B:197:LYS:CA	2:B:229:HIS:NE2	2.11	1.13
2:B:260:LEU:HD22	2:B:291:TYR:CZ	1.83	1.13
2:B:563:PHE:CD2	2:B:584:SER:CB	2.32	1.13
3:M:125:PHE:O	3:M:129:VAL:HG23	0.98	1.13
3:M:224:VAL:N	3:M:479:PHE:CG	2.15	1.13
3:M:323:MET:SD	3:M:342:LEU:HG	1.88	1.13
4:S:5:VAL:CG2	4:S:132:LEU:CD2	2.00	1.13
4:S:34:GLN:OE1	4:S:58:LEU:CD1	1.95	1.13
1:A:253:ILE:HD13	1:A:281:LEU:HB3	1.25	1.13
1:A:595:GLU:HG3	2:B:469:ASP:CA	1.61	1.13
2:B:108:PHE:CE2	2:B:115:LEU:CB	2.30	1.13
2:B:513:TRP:CA	2:B:551:LEU:HD22	1.77	1.13
2:B:123:LEU:O	2:B:127:LEU:HG	1.48	1.12
2:B:566:ALA:CA	2:B:574:ASN:CB	2.21	1.13

Continued on next page...

Continued from previous page...

Atom-1	Atom-2	Interatomic distance (Å)	Clash overlap (Å)
2:B:584:SER:O	2:B:588:ILE:CG2	1.97	1.13
2:B:139:LEU:CD2	2:B:173:VAL:CA	1.95	1.12
2:B:200:MET:HG2	2:B:232:ARG:HB3	1.23	1.12
2:B:527:PRO:CB	2:B:587:ARG:CG	2.27	1.12
2:B:559:ASP:HB2	2:B:563:PHE:CE1	1.83	1.12
1:A:244:LEU:HD13	1:A:256:LEU:CD1	1.79	1.12
1:A:252:ILE:CA	4:S:144:THR:OG1	1.96	1.12
2:B:174:ALA:CB	2:B:211:ALA:HA	1.79	1.12
2:B:178:ILE:O	2:B:180:LEU:N	1.83	1.12
2:B:181:TYR:CD2	2:B:218:CYS:C	2.15	1.12
2:B:212:VAL:O	2:B:214:ALA:N	1.82	1.12
2:B:461:HIS:O	2:B:462:ASN:C	1.74	1.12
2:B:537:PHE:CZ	2:B:545:ARG:HG2	1.84	1.12
3:M:18:TYR:CD1	3:M:122:SER:CA	2.30	1.12
4:S:48:SER:CB	4:S:77:TYR:O	1.96	1.12
1:A:74:LEU:HD22	1:A:87:CYS:SG	1.90	1.12
1:A:219:VAL:HG11	1:A:256:LEU:HD23	1.31	1.12
1:A:225:LEU:HD13	1:A:233:PHE:CZ	1.83	1.12
1:A:255:ARG:HH22	4:S:135:ILE:CG2	1.58	1.12
2:B:418:TYR:OH	2:B:432:ALA:CB	1.96	1.12
2:B:483:PRO:HB3	2:B:521:ILE:CG2	1.79	1.12
3:M:342:LEU:CD1	3:M:411:LEU:HB2	1.78	1.12
1:A:141:VAL:C	4:S:159:ALA:HB2	1.70	1.12
1:A:215:VAL:HG22	1:A:243:ILE:HD12	1.23	1.12
1:A:225:LEU:CD1	1:A:233:PHE:CZ	2.31	1.12
2:B:230:PHE:CE1	2:B:252:LEU:CD2	2.24	1.12
2:B:393:ILE:HG22	2:B:431:MET:HG2	1.23	1.12
2:B:563:PHE:HD2	2:B:584:SER:HB2	1.05	1.12
1:A:182:ILE:CG2	1:A:221:VAL:HG21	1.79	1.12
1:A:637:GLU:CG	2:B:516:GLY:N	2.12	1.12
2:B:25:VAL:CG2	2:B:35:TYR:HD2	1.61	1.12
2:B:336:ASN:O	2:B:337:THR:HB	1.50	1.12
2:B:353:GLN:CD	3:M:47:SER:CB	2.18	1.12
2:B:549:LEU:HD11	2:B:611:ALA:CA	1.78	1.12
3:M:243:ILE:N	3:M:474:THR:HG22	1.64	1.12
3:M:272:LEU:HD22	3:M:278:ILE:HB	1.20	1.12
1:A:128:LEU:HD12	1:A:150:LEU:HD21	1.21	1.11
1:A:251:TRP:CZ2	4:S:103:GLN:CB	2.32	1.11
2:B:127:LEU:CG	2:B:157:THR:HG21	1.78	1.11
2:B:178:ILE:CG1	2:B:215:TYR:N	2.13	1.11
2:B:353:GLN:HG2	3:M:49:ASP:N	1.64	1.11

Continued on next page...

Continued from previous page...

Atom-1	Atom-2	Interatomic distance (Å)	Clash overlap (Å)
2:B:437:SER:HA	2:B:478:LEU:HD21	1.14	1.11
2:B:549:LEU:CD1	2:B:611:ALA:CB	2.27	1.11
3:M:223:HIS:CA	3:M:479:PHE:CD2	2.33	1.11
3:M:265:ASN:OD1	3:M:313:SER:OG	1.67	1.11
3:M:432:THR:OG1	3:M:480:GLN:HG3	0.94	1.11
4:S:53:THR:O	4:S:69:ASN:CB	1.97	1.11
1:A:99:LYS:NZ	4:S:164:ASP:HB2	1.65	1.11
2:B:144:ASP:HA	2:B:179:LYS:NZ	1.63	1.11
2:B:219:TYR:CD2	2:B:226:LEU:HB2	1.84	1.11
2:B:234:CYS:O	2:B:237:ILE:HG22	1.49	1.11
3:M:45:SER:HB2	3:M:51:LEU:HD11	1.18	1.11
1:A:102:GLN:HB2	4:S:163:THR:HB	1.15	1.11
1:A:217:ALA:HB1	4:S:142:ILE:CG1	1.81	1.11
1:A:403:LEU:HD22	1:A:422:GLU:HG3	1.16	1.11
2:B:352:ASN:HB3	3:M:49:ASP:OD2	1.42	1.11
2:B:472:VAL:CG1	2:B:510:GLY:CA	2.25	1.11
2:B:483:PRO:CB	2:B:521:ILE:HG21	1.81	1.11
3:M:18:TYR:CD1	3:M:122:SER:HA	1.66	1.11
4:S:73:ILE:HG23	4:S:88:ILE:CG2	1.77	1.11
2:B:219:TYR:CG	2:B:226:LEU:HD22	1.83	1.11
2:B:319:LEU:HD11	2:B:358:MET:CG	1.70	1.11
3:M:219:LEU:HD22	3:M:473:LYS:HA	1.13	1.11
1:A:103:LYS:CB	4:S:163:THR:HG21	1.80	1.11
1:A:204:VAL:HG22	1:A:236:LEU:HD21	1.18	1.11
2:B:24:ALA:CB	2:B:35:TYR:CE2	2.32	1.11
2:B:106:LEU:HD12	2:B:144:ASP:O	1.43	1.11
2:B:307:ASN:ND2	2:B:336:ASN:ND2	1.98	1.11
2:B:334:MET:HA	2:B:334:MET:HE2	1.30	1.11
1:A:408:ILE:CG2	4:S:64:ASN:CA	2.28	1.10
2:B:37:TYR:HH	2:B:46:GLN:CD	1.47	1.10
2:B:121:ASN:OD1	2:B:153:ILE:CD1	1.97	1.10
2:B:127:LEU:HB3	2:B:161:LEU:CD1	1.80	1.10
2:B:343:LEU:HD22	2:B:362:ALA:CB	1.80	1.10
3:M:319:SER:HB3	3:M:346:ASN:CA	1.79	1.10
4:S:53:THR:C	4:S:69:ASN:CB	2.18	1.10
4:S:53:THR:CB	4:S:68:VAL:C	2.18	1.10
1:A:163:ALA:HB2	1:A:195:ALA:CB	1.80	1.10
2:B:25:VAL:HG23	2:B:35:TYR:HD2	0.94	1.10
2:B:37:TYR:CD2	2:B:38:TYR:HD1	1.65	1.10
2:B:212:VAL:HG22	2:B:248:LEU:HD21	1.30	1.10
3:M:221:THR:HB	3:M:474:THR:O	1.50	1.10

Continued on next page...

Continued from previous page...

Atom-1	Atom-2	Interatomic distance (Å)	Clash overlap (Å)
1:A:101:GLN:HG2	4:S:167:ILE:HG21	1.32	1.10
1:A:102:GLN:CB	4:S:163:THR:HB	1.72	1.10
2:B:231:ARG:CG	2:B:298:ASP:OD1	1.98	1.10
2:B:267:ASP:HB3	2:B:289:PRO:HD3	1.31	1.10
2:B:497:LEU:HD11	2:B:508:ARG:NH1	1.67	1.10
2:B:549:LEU:HD21	2:B:611:ALA:N	0.78	1.10
1:A:631:SER:HB3	2:B:557:SER:OG	1.45	1.10
2:B:227:HIS:CD2	2:B:292:GLU:OE2	2.05	1.10
3:M:317:MET:CB	3:M:320:ILE:O	1.98	1.10
3:M:339:GLU:HG3	3:M:412:ARG:HG2	1.16	1.10
4:S:5:VAL:CB	4:S:132:LEU:CD2	2.29	1.10
4:S:9:ASN:HD21	4:S:13:GLN:CG	1.64	1.10
1:A:204:VAL:CG2	1:A:236:LEU:CD2	2.26	1.10
1:A:408:ILE:HG23	4:S:64:ASN:CA	1.82	1.10
2:B:2:VAL:HG12	2:B:6:HIS:CD2	1.86	1.10
2:B:25:VAL:HG21	2:B:36:THR:OG1	1.51	1.10
2:B:127:LEU:CG	2:B:157:THR:CG2	2.27	1.10
2:B:139:LEU:HD21	2:B:173:VAL:O	1.49	1.10
2:B:162:VAL:HG21	2:B:195:ILE:CG2	1.62	1.10
2:B:512:VAL:HG11	2:B:548:ILE:HA	1.17	1.10
3:M:217:ASP:HB3	3:M:470:ALA:C	1.67	1.10
1:A:253:ILE:HG12	1:A:281:LEU:CD1	1.80	1.09
1:A:520:GLY:HA3	1:A:558:VAL:CG2	1.81	1.09
2:B:212:VAL:O	2:B:213:LEU:C	1.83	1.09
3:M:101:LEU:HA	3:M:109:LEU:HD11	1.23	1.09
1:A:488:ARG:HG2	1:A:522:PHE:CE2	1.88	1.09
1:A:637:GLU:OE1	2:B:513:TRP:CD1	2.06	1.09
2:B:98:LYS:HZ1	2:B:134:LEU:CB	1.63	1.09
2:B:108:PHE:CZ	2:B:115:LEU:CG	2.34	1.09
2:B:170:ARG:CA	2:B:199:LEU:HD22	1.82	1.09
2:B:178:ILE:HA	2:B:218:CYS:HB2	1.28	1.09
2:B:208:ILE:HD13	2:B:236:ILE:HG23	1.17	1.09
2:B:343:LEU:CD1	2:B:359:LEU:HD13	1.80	1.09
2:B:353:GLN:HG2	3:M:49:ASP:H	1.06	1.09
3:M:92:PHE:HZ	3:M:129:VAL:HG22	0.98	1.09
1:A:142:LYS:N	4:S:159:ALA:CB	2.16	1.09
1:A:254:ILE:HG23	1:A:293:GLU:HG2	1.32	1.09
2:B:20:ARG:HD2	2:B:21:GLU:CB	1.83	1.09
2:B:79:VAL:CG2	2:B:108:PHE:CE1	2.36	1.09
2:B:151:ALA:HA	2:B:180:LEU:HD11	1.16	1.09
2:B:553:ALA:HB2	2:B:614:ILE:HD13	1.11	1.09

Continued on next page...

Continued from previous page...

Atom-1	Atom-2	Interatomic distance (Å)	Clash overlap (Å)
3:M:342:LEU:HD11	3:M:411:LEU:HD22	1.18	1.09
4:S:109:LEU:HD11	4:S:113:PHE:CD1	1.85	1.09
1:A:103:LYS:HG3	4:S:163:THR:CG2	1.82	1.09
1:A:586:GLU:O	1:A:587:ASN:C	1.83	1.09
2:B:219:TYR:OH	2:B:226:LEU:HA	1.50	1.09
2:B:515:PHE:HE2	2:B:529:VAL:HG21	1.15	1.09
2:B:530:LEU:HG	2:B:591:MET:HB3	1.32	1.09
2:B:566:ALA:C	2:B:574:ASN:HD22	1.56	1.09
2:B:568:VAL:O	2:B:571:SER:HB2	1.49	1.09
3:M:443:SER:HB3	3:M:447:ILE:HG13	1.17	1.09
3:M:451:ALA:C	3:M:452:ILE:HD12	1.72	1.09
4:S:5:VAL:CB	4:S:132:LEU:HD21	1.81	1.09
1:A:182:ILE:HD13	1:A:218:ALA:HB2	1.34	1.09
1:A:403:LEU:HD22	1:A:422:GLU:CD	1.70	1.09
2:B:225:LEU:HD13	2:B:283:TYR:HE1	1.05	1.09
2:B:292:GLU:CG	2:B:296:ASP:HB2	1.82	1.09
3:M:267:ILE:HD12	3:M:445:SER:OG	1.52	1.09
1:A:67:LYS:N	4:S:165:SER:HB2	1.68	1.08
1:A:105:VAL:HB	4:S:167:ILE:HD13	1.34	1.08
1:A:141:VAL:HG21	4:S:158:LYS:H	1.03	1.08
1:A:513:ARG:HD2	1:A:550:VAL:CG2	1.81	1.08
2:B:197:LYS:N	2:B:229:HIS:NE2	2.01	1.08
3:M:217:ASP:H	3:M:470:ALA:HB3	1.07	1.08
1:A:132:LEU:HD22	1:A:169:MET:CG	1.82	1.08
1:A:140:VAL:HA	1:A:177:ILE:HG13	1.22	1.08
1:A:215:VAL:HG22	1:A:243:ILE:CD1	1.82	1.08
1:A:298:ILE:HD11	1:A:311:THR:HG21	1.33	1.08
1:A:408:ILE:CG2	4:S:64:ASN:HB3	1.70	1.08
1:A:429:VAL:HB	1:A:469:LEU:CD1	1.82	1.08
1:A:448:GLU:HB2	1:A:487:MET:SD	1.93	1.08
2:B:343:LEU:HD12	2:B:359:LEU:HD13	1.23	1.08
3:M:65:TYR:CE2	3:M:86:PRO:HB3	1.88	1.08
3:M:323:MET:HB3	3:M:340:LEU:HD11	1.21	1.08
3:M:435:LEU:O	3:M:479:PHE:CD2	2.06	1.08
4:S:5:VAL:CG1	4:S:132:LEU:HD22	1.83	1.08
1:A:132:LEU:O	1:A:169:MET:CE	2.00	1.08
1:A:217:ALA:HA	4:S:142:ILE:CA	1.63	1.08
1:A:224:GLU:HB2	4:S:138:GLY:O	0.92	1.08
2:B:107:ARG:O	2:B:110:GLU:N	1.87	1.08
2:B:347:VAL:HG22	2:B:359:LEU:HB3	1.23	1.08
2:B:534:ILE:HD13	2:B:594:ALA:HB3	1.33	1.08

Continued on next page...

Continued from previous page...

Atom-1	Atom-2	Interatomic distance (Å)	Clash overlap (Å)
2:B:566:ALA:O	2:B:574:ASN:HB2	0.91	1.08
3:M:245:ASP:N	3:M:472:TYR:CE1	2.20	1.08
1:A:103:LYS:N	4:S:163:THR:CB	2.16	1.08
2:B:56:SER:HB3	2:B:92:THR:HG21	1.31	1.08
2:B:175:LEU:HG	2:B:210:CYS:HB3	1.11	1.08
2:B:215:TYR:HB3	2:B:226:LEU:HD11	1.29	1.08
2:B:400:SER:CB	2:B:439:CYS:SG	2.42	1.08
2:B:497:LEU:HD23	2:B:533:LEU:HD21	1.09	1.08
2:B:567:GLN:HG3	2:B:569:THR:OG1	1.53	1.08
3:M:336:ASP:O	3:M:414:CYS:SG	2.10	1.08
1:A:68:THR:HA	4:S:167:ILE:N	1.67	1.08
1:A:132:LEU:O	1:A:169:MET:HE1	1.52	1.08
1:A:170:LEU:O	1:A:206:LYS:CD	2.00	1.08
1:A:219:VAL:HG21	1:A:256:LEU:CD2	1.82	1.08
1:A:609:LEU:HD21	1:A:628:VAL:CG2	1.83	1.08
2:B:175:LEU:HD21	2:B:210:CYS:HA	1.32	1.08
2:B:216:LYS:HB2	2:B:251:LEU:HD13	1.31	1.08
2:B:307:ASN:ND2	2:B:336:ASN:HD21	1.48	1.08
2:B:319:LEU:HD11	2:B:358:MET:HG3	1.29	1.08
3:M:454:ILE:HG21	3:M:464:THR:CG2	1.84	1.08
4:S:15:ARG:CZ	4:S:122:ILE:HD11	1.84	1.08
1:A:132:LEU:HD22	1:A:169:MET:HG3	1.13	1.07
1:A:182:ILE:HG22	1:A:221:VAL:HG21	1.20	1.07
2:B:21:GLU:HA	2:B:24:ALA:HB2	1.35	1.07
2:B:159:LYS:CA	2:B:195:ILE:HD11	1.84	1.07
2:B:175:LEU:HD11	2:B:210:CYS:SG	1.94	1.07
2:B:279:LEU:HG	2:B:288:TYR:HD1	1.12	1.07
2:B:292:GLU:OE2	2:B:296:ASP:OD2	1.71	1.07
2:B:352:ASN:HA	3:M:49:ASP:OD2	1.37	1.07
2:B:396:ILE:HD13	2:B:432:ALA:HB2	1.36	1.07
2:B:433:VAL:HG12	2:B:474:VAL:CB	1.84	1.07
3:M:254:PRO:HB3	3:M:454:ILE:HD12	1.26	1.07
4:S:53:THR:HG21	4:S:68:VAL:CA	1.83	1.07
2:B:197:LYS:CA	2:B:229:HIS:CD2	2.36	1.07
2:B:208:ILE:HG12	2:B:236:ILE:HD13	1.31	1.07
2:B:247:TYR:CE2	3:M:91:THR:CG2	2.36	1.07
2:B:247:TYR:CZ	3:M:91:THR:HG21	1.87	1.07
2:B:316:THR:OG1	3:M:90:PHE:HZ	1.37	1.07
2:B:458:MET:SD	2:B:471:TYR:HB3	1.94	1.07
3:M:222:PHE:C	3:M:479:PHE:CE2	2.28	1.07
1:A:251:TRP:CZ2	4:S:103:GLN:HB2	1.90	1.07

Continued on next page...

Continued from previous page...

Atom-1	Atom-2	Interatomic distance (Å)	Clash overlap (Å)
1:A:322:PHE:CD2	1:A:330:LEU:HD21	1.88	1.07
1:A:509:PRO:HB3	1:A:547:VAL:CG2	1.84	1.07
1:A:555:LEU:HD13	1:A:581:LEU:CD1	1.84	1.07
1:A:633:PHE:CB	2:B:550:VAL:CG1	2.22	1.07
1:A:633:PHE:HB2	2:B:550:VAL:CG1	1.80	1.07
2:B:123:LEU:HD12	2:B:142:LEU:CG	1.79	1.07
2:B:278:PRO:HD3	2:B:289:PRO:O	1.52	1.07
2:B:353:GLN:CB	3:M:49:ASP:N	2.09	1.07
3:M:217:ASP:HB2	3:M:470:ALA:C	1.67	1.07
3:M:217:ASP:N	3:M:470:ALA:HB3	1.70	1.07
3:M:219:LEU:HB2	3:M:472:TYR:O	1.54	1.07
4:S:53:THR:O	4:S:69:ASN:HB2	1.50	1.07
4:S:73:ILE:HG21	4:S:88:ILE:HG21	1.33	1.07
2:B:278:PRO:CA	2:B:288:TYR:O	2.00	1.07
4:S:5:VAL:HG11	4:S:132:LEU:HD22	1.09	1.07
1:A:166:LEU:HD13	1:A:185:LEU:CD2	1.85	1.07
1:A:384:LEU:HD22	1:A:441:TYR:CZ	1.88	1.07
1:A:384:LEU:HD13	1:A:435:ILE:CG2	1.85	1.07
2:B:154:ILE:HD12	2:B:180:LEU:HD13	1.12	1.07
2:B:274:PRO:C	2:B:295:ASN:ND2	2.08	1.07
2:B:290:SER:O	2:B:291:TYR:C	1.75	1.07
2:B:367:SER:OG	2:B:401:THR:HG21	1.55	1.07
2:B:549:LEU:HD23	2:B:607:ILE:O	1.54	1.07
2:B:560:ILE:HG23	2:B:564:LYS:HB2	1.35	1.07
1:A:207:LEU:HD23	1:A:239:LEU:HB2	1.29	1.06
1:A:237:SER:HB2	1:A:270:LEU:HD13	1.37	1.06
1:A:594:PHE:HB3	2:B:473:ASN:HB3	1.32	1.06
2:B:77:ILE:HG22	2:B:82:TYR:HE1	1.18	1.06
2:B:120:ILE:HA	2:B:142:LEU:HD21	1.34	1.06
2:B:132:SER:HA	2:B:169:VAL:CG2	1.85	1.06
2:B:158:VAL:HG11	2:B:177:ILE:HG13	1.34	1.06
2:B:219:TYR:CE1	2:B:226:LEU:CA	2.27	1.06
2:B:515:PHE:CE2	2:B:529:VAL:HG21	1.89	1.06
2:B:537:PHE:CE1	2:B:545:ARG:HG2	1.91	1.06
3:M:45:SER:CB	3:M:51:LEU:HD11	1.85	1.06
4:S:53:THR:HG21	4:S:68:VAL:HA	1.36	1.06
2:B:175:LEU:HD23	2:B:210:CYS:O	1.53	1.06
2:B:291:TYR:HE2	2:B:294:VAL:HB	0.90	1.06
2:B:343:LEU:HD11	2:B:359:LEU:HA	1.06	1.06
2:B:353:GLN:CD	3:M:47:SER:HB2	1.76	1.06
3:M:215:TYR:CG	3:M:468:LYS:CA	2.38	1.06

Continued on next page...

Continued from previous page...

Atom-1	Atom-2	Interatomic distance (Å)	Clash overlap (Å)
1:A:67:LYS:HB3	1:A:94:VAL:HG22	1.32	1.06
1:A:80:TYR:HB2	1:A:82:PHE:CD2	1.90	1.06
1:A:204:VAL:HG22	1:A:236:LEU:HD22	1.21	1.06
1:A:211:ASP:OD2	4:S:148:ARG:NH1	1.89	1.06
2:B:225:LEU:CD1	2:B:283:TYR:CE1	2.36	1.06
2:B:303:LEU:HD11	2:B:333:GLN:HB3	1.31	1.06
2:B:527:PRO:CG	2:B:587:ARG:HG3	1.85	1.06
3:M:374:TYR:O	3:M:390:ILE:HD12	1.51	1.06
1:A:295:VAL:HG22	1:A:315:CYS:HB3	1.25	1.06
2:B:29:LYS:CE	2:B:30:LEU:N	2.11	1.06
2:B:127:LEU:HD23	2:B:161:LEU:HD21	1.07	1.06
2:B:178:ILE:CG2	2:B:217:GLU:HB2	1.85	1.06
2:B:274:PRO:CG	2:B:295:ASN:OD1	2.03	1.06
2:B:353:GLN:CG	3:M:47:SER:O	2.02	1.06
3:M:302:TYR:CD2	3:M:445:SER:HB3	1.90	1.06
3:M:338:PHE:CE2	3:M:415:ILE:CG1	2.39	1.06
1:A:207:LEU:CD2	1:A:239:LEU:HB2	1.85	1.06
1:A:215:VAL:HG21	1:A:243:ILE:HD12	1.31	1.06
1:A:224:GLU:HB2	4:S:138:GLY:C	1.75	1.06
1:A:258:LYS:HZ1	4:S:97:ALA:HB2	1.20	1.06
2:B:136:CYS:C	2:B:172:GLU:HG3	1.75	1.06
2:B:177:ILE:HB	2:B:196:LEU:HD21	1.37	1.06
2:B:256:CYS:SG	2:B:299:LEU:HD23	1.96	1.06
2:B:293:VAL:O	2:B:299:LEU:CB	2.04	1.06
2:B:319:LEU:HD13	2:B:358:MET:HG3	1.09	1.06
2:B:437:SER:CA	2:B:478:LEU:HD21	1.86	1.06
2:B:508:ARG:O	2:B:512:VAL:HG23	1.54	1.06
2:B:527:PRO:HB3	2:B:587:ARG:HG3	1.32	1.06
1:A:533:ILE:HG12	1:A:562:TRP:CH2	1.91	1.05
2:B:25:VAL:CG2	2:B:36:THR:OG1	2.04	1.05
2:B:219:TYR:OH	2:B:226:LEU:N	1.87	1.05
2:B:433:VAL:CG1	2:B:474:VAL:CG2	2.12	1.05
2:B:546:CYS:CA	2:B:607:ILE:HG12	1.85	1.05
3:M:226:PHE:N	3:M:480:GLN:O	1.89	1.05
3:M:360:LEU:HD23	3:M:362:PHE:CZ	1.90	1.05
1:A:225:LEU:HD13	1:A:233:PHE:HZ	0.99	1.05
1:A:630:PRO:O	2:B:554:LYS:CA	2.05	1.05
2:B:79:VAL:CB	2:B:108:PHE:CE1	2.38	1.05
2:B:116:THR:HG22	2:B:150:LEU:HD11	1.35	1.05
2:B:549:LEU:CD1	2:B:611:ALA:CA	2.31	1.05
3:M:60:LEU:CD2	3:M:62:VAL:HG23	1.86	1.05

Continued on next page...

Continued from previous page...

Atom-1	Atom-2	Interatomic distance (Å)	Clash overlap (Å)
3:M:66:PHE:HA	3:M:77:LEU:HD11	1.38	1.05
3:M:244:VAL:O	3:M:299:LEU:N	1.88	1.05
1:A:67:LYS:H	4:S:165:SER:CB	1.68	1.05
1:A:166:LEU:CD1	1:A:185:LEU:CD2	2.33	1.05
1:A:200:PHE:CE1	1:A:232:PRO:O	2.09	1.05
1:A:260:PHE:CD2	1:A:274:LEU:HG	1.91	1.05
1:A:629:LEU:CG	2:B:610:ARG:NH1	2.18	1.05
1:A:630:PRO:HG3	2:B:614:ILE:HG13	1.27	1.05
1:A:633:PHE:CD1	2:B:513:TRP:CE3	2.40	1.05
2:B:77:ILE:HG22	2:B:82:TYR:CE1	1.92	1.05
2:B:566:ALA:CA	2:B:574:ASN:CG	2.25	1.05
3:M:348:LYS:HG3	3:M:405:THR:HG22	1.38	1.05
1:A:557:LYS:HE3	2:B:606:ASP:HB2	1.38	1.05
1:A:631:SER:CA	2:B:557:SER:HB3	1.82	1.05
2:B:286:ILE:O	2:B:287:GLU:O	1.73	1.05
2:B:315:PRO:CB	2:B:352:ASN:OD1	2.04	1.05
3:M:341:SER:OG	3:M:343:ASN:ND2	1.88	1.05
3:M:342:LEU:HD13	3:M:411:LEU:HB2	1.38	1.05
1:A:101:GLN:HG2	4:S:167:ILE:CG2	1.86	1.05
1:A:176:TYR:HB2	4:S:155:GLU:HG3	1.33	1.05
2:B:20:ARG:HD2	2:B:21:GLU:HB2	1.15	1.05
2:B:245:GLN:CG	2:B:309:LEU:HD11	1.85	1.05
2:B:309:LEU:HB3	2:B:317:VAL:HG12	1.07	1.05
2:B:545:ARG:CD	2:B:602:ASP:HB2	1.86	1.05
2:B:566:ALA:C	2:B:574:ASN:ND2	2.11	1.05
3:M:64:LYS:NZ	3:M:79:SER:O	1.90	1.05
3:M:244:VAL:HB	3:M:300:LEU:HG	1.39	1.05
4:S:8:PHE:HB2	4:S:36:TYR:HE2	0.90	1.05
1:A:182:ILE:HG22	1:A:221:VAL:HG23	1.33	1.04
1:A:260:PHE:O	1:A:261:THR:C	1.78	1.04
1:A:440:ASN:CG	1:A:442:SER:HB3	1.77	1.04
2:B:2:VAL:CG1	2:B:6:HIS:CE1	2.36	1.04
2:B:20:ARG:HD2	2:B:35:TYR:OH	1.56	1.04
2:B:154:ILE:HD13	2:B:180:LEU:HB2	1.37	1.04
2:B:162:VAL:HG22	2:B:199:LEU:HG	1.11	1.04
2:B:219:TYR:HE1	2:B:226:LEU:CD1	1.51	1.04
2:B:223:LEU:HD11	2:B:258:GLN:C	1.77	1.04
2:B:563:PHE:O	2:B:567:GLN:N	1.88	1.04
3:M:49:ASP:HA	3:M:75:TRP:CH2	1.91	1.04
1:A:67:LYS:O	1:A:71:VAL:HG23	1.58	1.04
1:A:182:ILE:CG2	1:A:221:VAL:CG2	2.33	1.04

Continued on next page...

Continued from previous page...

Atom-1	Atom-2	Interatomic distance (Å)	Clash overlap (Å)
1:A:630:PRO:HG2	2:B:614:ILE:CA	1.87	1.04
1:A:633:PHE:CD2	2:B:551:LEU:HA	1.54	1.04
2:B:158:VAL:HG13	2:B:173:VAL:HG12	1.37	1.04
2:B:162:VAL:HG21	2:B:195:ILE:HG22	1.34	1.04
2:B:178:ILE:HG23	2:B:217:GLU:HB2	1.38	1.04
2:B:215:TYR:HB3	2:B:226:LEU:CD1	1.87	1.04
2:B:278:PRO:HA	2:B:288:TYR:CB	1.87	1.04
2:B:396:ILE:HG12	2:B:418:TYR:CE2	1.93	1.04
3:M:327:PHE:HE1	3:M:336:ASP:HB2	1.19	1.04
4:S:53:THR:HB	4:S:68:VAL:C	1.77	1.04
1:A:71:VAL:HG11	1:A:105:VAL:HG12	1.36	1.04
1:A:147:LEU:HD22	1:A:166:LEU:HD21	1.07	1.04
2:B:162:VAL:HG22	2:B:199:LEU:CD1	1.87	1.04
2:B:537:PHE:CE2	2:B:598:LEU:O	2.11	1.04
2:B:567:GLN:CG	2:B:569:THR:OG1	2.05	1.04
3:M:215:TYR:HD1	3:M:467:TYR:O	1.39	1.04
3:M:245:ASP:N	3:M:472:TYR:CG	2.09	1.04
1:A:150:LEU:HB3	1:A:162:ILE:HD13	1.37	1.04
1:A:557:LYS:HE2	2:B:606:ASP:CA	1.86	1.04
1:A:557:LYS:HE3	2:B:606:ASP:CB	1.86	1.04
1:A:594:PHE:CB	2:B:473:ASN:CB	2.34	1.04
2:B:248:LEU:O	2:B:252:LEU:HG	1.58	1.04
2:B:340:ILE:HD11	2:B:366:LEU:HB3	1.38	1.04
2:B:542:PRO:HA	2:B:602:ASP:CG	1.76	1.04
3:M:244:VAL:HG13	3:M:472:TYR:CZ	1.92	1.04
1:A:96:SER:HB3	1:A:127:LEU:CD1	1.86	1.04
1:A:251:TRP:CH2	4:S:103:GLN:CB	2.41	1.04
1:A:636:TYR:HB2	2:B:554:LYS:HZ2	1.19	1.04
2:B:41:ASN:CB	2:B:43:ASN:OD1	2.04	1.04
2:B:261:PRO:CA	2:B:290:SER:CB	2.35	1.04
2:B:303:LEU:HD11	2:B:333:GLN:CB	1.84	1.04
2:B:309:LEU:CB	2:B:317:VAL:CG1	2.35	1.04
2:B:519:ALA:O	2:B:523:PHE:CD2	2.11	1.04
3:M:222:PHE:HB2	3:M:479:PHE:CZ	1.92	1.04
3:M:223:HIS:CA	3:M:479:PHE:CE2	2.41	1.04
3:M:226:PHE:HZ	3:M:321:GLY:O	1.41	1.04
1:A:103:LYS:H	4:S:163:THR:HG21	1.23	1.03
1:A:104:ARG:CA	1:A:145:ILE:HG21	1.88	1.03
1:A:105:VAL:HG21	4:S:167:ILE:HG23	1.40	1.03
2:B:178:ILE:HD11	2:B:215:TYR:HA	1.07	1.03
2:B:267:ASP:C	2:B:276:SER:HB2	1.79	1.03

Continued on next page...

Continued from previous page...

Atom-1	Atom-2	Interatomic distance (Å)	Clash overlap (Å)
2:B:340:ILE:HD13	2:B:366:LEU:HD13	1.32	1.03
2:B:343:LEU:HD21	2:B:362:ALA:HB3	1.35	1.03
2:B:537:PHE:CG	2:B:598:LEU:HB3	1.92	1.03
3:M:214:LEU:O	3:M:467:TYR:N	1.89	1.03
1:A:251:TRP:CZ2	4:S:103:GLN:HB3	1.93	1.03
2:B:158:VAL:CG1	2:B:177:ILE:HG13	1.86	1.03
2:B:175:LEU:HD21	2:B:210:CYS:CA	1.88	1.03
2:B:343:LEU:HD12	2:B:359:LEU:HD12	1.33	1.03
2:B:493:LEU:HG	2:B:511:ILE:HG23	1.40	1.03
2:B:530:LEU:CG	2:B:591:MET:HB3	1.88	1.03
3:M:319:SER:HB3	3:M:346:ASN:H	0.98	1.03
3:M:449:VAL:HG12	3:M:452:ILE:HD11	1.36	1.03
1:A:323:CYS:SG	1:A:334:SER:HB3	1.97	1.03
1:A:397:ASP:O	1:A:418:ILE:HG13	1.59	1.03
2:B:154:ILE:CD1	2:B:180:LEU:HB2	1.87	1.03
2:B:175:LEU:HA	2:B:214:ALA:HB2	1.35	1.03
2:B:508:ARG:CB	2:B:544:THR:HG23	1.87	1.03
3:M:60:LEU:HD23	3:M:61:GLU:N	1.71	1.03
3:M:243:ILE:O	3:M:472:TYR:CD2	2.12	1.03
3:M:290:PHE:CZ	3:M:297:PHE:CG	2.47	1.03
3:M:319:SER:CB	3:M:346:ASN:H	1.70	1.03
1:A:68:THR:HA	4:S:166:LYS:C	1.76	1.03
1:A:140:VAL:CG2	1:A:177:ILE:HG13	1.87	1.03
2:B:424:PHE:CD2	2:B:428:VAL:HG11	1.93	1.03
2:B:542:PRO:CA	2:B:602:ASP:OD2	2.07	1.03
3:M:2:TYR:N	3:M:81:SER:CB	2.22	1.03
3:M:224:VAL:O	3:M:480:GLN:N	1.91	1.03
3:M:243:ILE:H	3:M:474:THR:HG22	0.87	1.03
1:A:132:LEU:HD13	1:A:165:ASP:HB3	1.40	1.03
1:A:176:TYR:CB	4:S:155:GLU:CG	2.35	1.03
1:A:183:THR:O	1:A:186:PHE:HB3	1.59	1.03
1:A:398:GLU:O	1:A:420:ILE:N	1.90	1.03
1:A:594:PHE:HB3	2:B:473:ASN:HB2	1.09	1.03
1:A:605:GLU:HG3	1:A:632:PHE:CE2	1.93	1.03
2:B:120:ILE:HA	2:B:142:LEU:CD2	1.88	1.03
2:B:227:HIS:C	2:B:229:HIS:H	1.59	1.03
2:B:371:GLN:NE2	2:B:401:THR:O	1.90	1.03
1:A:96:SER:HA	1:A:127:LEU:HD11	1.39	1.02
1:A:140:VAL:HG22	1:A:177:ILE:HG13	1.33	1.02
1:A:225:LEU:HB3	1:A:233:PHE:CE1	1.94	1.02
1:A:323:CYS:SG	1:A:334:SER:CB	2.47	1.02

Continued on next page...

Continued from previous page...

Atom-1	Atom-2	Interatomic distance (Å)	Clash overlap (Å)
2:B:135:ARG:NH2	2:B:164:ASP:CG	2.12	1.02
2:B:215:TYR:CZ	2:B:229:HIS:HB3	1.94	1.02
2:B:567:GLN:N	2:B:574:ASN:HD22	1.55	1.02
3:M:214:LEU:HD11	3:M:256:VAL:HG21	1.41	1.02
3:M:242:GLY:HA2	3:M:474:THR:HG21	1.40	1.02
1:A:96:SER:HB2	1:A:127:LEU:HD11	1.39	1.02
1:A:111:SER:HB2	1:A:152:THR:OG1	0.86	1.02
1:A:219:VAL:HG22	1:A:240:LEU:HD22	1.42	1.02
1:A:463:ASP:O	2:B:1:MET:SD	2.16	1.02
1:A:563:CYS:HA	1:A:566:PHE:HD2	1.23	1.02
1:A:630:PRO:CG	2:B:614:ILE:CG1	1.92	1.02
2:B:243:TRP:CZ3	3:M:91:THR:HA	1.94	1.02
2:B:553:ALA:HB2	2:B:614:ILE:CG1	1.88	1.02
2:B:564:LYS:HD2	2:B:621:GLY:O	1.58	1.02
2:B:588:ILE:HG23	2:B:618:PHE:CZ	1.93	1.02
3:M:243:ILE:HB	3:M:473:LYS:O	1.55	1.02
4:S:8:PHE:HB3	4:S:36:TYR:CE2	1.92	1.02
4:S:17:VAL:HG22	4:S:19:PHE:CZ	1.94	1.02
1:A:102:GLN:HA	4:S:167:ILE:CG1	1.88	1.02
1:A:102:GLN:OE1	4:S:166:LYS:HG3	1.58	1.02
1:A:217:ALA:O	4:S:142:ILE:CG2	2.06	1.02
1:A:251:TRP:CH2	4:S:103:GLN:OE1	2.11	1.02
2:B:106:LEU:CD1	2:B:144:ASP:HB2	1.74	1.02
2:B:418:TYR:O	2:B:419:VAL:C	1.88	1.02
3:M:7:ILE:HA	3:M:76:CYS:HA	1.40	1.02
3:M:65:TYR:CE1	3:M:86:PRO:CB	2.43	1.02
1:A:141:VAL:HB	4:S:159:ALA:N	1.73	1.02
2:B:127:LEU:HB2	2:B:157:THR:HG23	1.39	1.02
2:B:143:SER:C	2:B:179:LYS:HD3	1.70	1.02
2:B:219:TYR:CB	2:B:223:LEU:CD2	2.14	1.02
2:B:267:ASP:H	2:B:289:PRO:HB3	0.88	1.02
2:B:343:LEU:HD22	2:B:362:ALA:HB3	1.33	1.02
4:S:53:THR:CG2	4:S:67:GLU:C	2.27	1.02
1:A:67:LYS:HB2	4:S:165:SER:OG	1.59	1.02
1:A:96:SER:CA	1:A:127:LEU:CD1	2.38	1.02
1:A:200:PHE:HE1	1:A:232:PRO:O	1.39	1.02
1:A:223:CYS:HB2	1:A:259:LEU:HG	1.42	1.02
2:B:38:TYR:HA	2:B:42:ILE:H	1.23	1.02
2:B:70:MET:HE3	2:B:107:ARG:CB	1.82	1.02
2:B:79:VAL:CG2	2:B:108:PHE:CZ	2.43	1.02
2:B:278:PRO:C	2:B:288:TYR:HB2	1.78	1.02

Continued on next page...

Continued from previous page...

Atom-1	Atom-2	Interatomic distance (Å)	Clash overlap (Å)
2:B:537:PHE:CD2	2:B:598:LEU:CB	2.43	1.02
4:S:48:SER:CB	4:S:77:TYR:HB2	1.86	1.02
2:B:56:SER:HB3	2:B:92:THR:CG2	1.89	1.01
2:B:62:ALA:C	2:B:66:ILE:HD12	1.80	1.01
2:B:155:LEU:HB2	2:B:188:TYR:HD2	1.22	1.01
2:B:261:PRO:HA	2:B:290:SER:HB3	1.03	1.01
2:B:415:LEU:HD13	2:B:436:LEU:HD21	1.39	1.01
2:B:550:VAL:HG22	2:B:610:ARG:HD3	1.37	1.01
3:M:344:ILE:HG23	3:M:347:PHE:CB	1.89	1.01
1:A:101:GLN:HG2	4:S:167:ILE:CB	1.90	1.01
1:A:104:ARG:HG3	1:A:145:ILE:CG1	1.89	1.01
1:A:217:ALA:O	4:S:142:ILE:CB	2.00	1.01
1:A:253:ILE:CG1	1:A:281:LEU:HD13	1.90	1.01
2:B:70:MET:HE1	2:B:104:TYR:O	1.59	1.01
2:B:178:ILE:HG21	2:B:214:ALA:O	1.60	1.01
2:B:219:TYR:O	2:B:223:LEU:CD2	2.08	1.01
2:B:293:VAL:O	2:B:299:LEU:HB2	1.58	1.01
2:B:513:TRP:HA	2:B:551:LEU:HD22	1.02	1.01
3:M:101:LEU:CG	3:M:106:LYS:CA	2.17	1.01
3:M:212:ASN:CB	3:M:250:LEU:HD23	1.90	1.01
3:M:256:VAL:HG12	3:M:290:PHE:HB3	1.39	1.01
3:M:342:LEU:CD1	3:M:411:LEU:CB	2.37	1.01
4:S:56:SER:O	4:S:60:SER:HB2	1.61	1.01
1:A:253:ILE:CD1	1:A:281:LEU:HD22	1.88	1.01
2:B:171:GLY:HA3	2:B:207:VAL:HG13	1.04	1.01
2:B:182:ARG:HD2	2:B:217:GLU:OE1	1.61	1.01
2:B:549:LEU:HD13	2:B:611:ALA:CB	1.89	1.01
2:B:562:ASN:HB3	2:B:580:TYR:HB3	1.39	1.01
3:M:443:SER:OG	3:M:447:ILE:O	1.77	1.01
1:A:147:LEU:HD22	1:A:166:LEU:HD23	1.35	1.01
1:A:295:VAL:HG11	1:A:319:LEU:HD11	1.42	1.01
1:A:398:GLU:O	1:A:420:ILE:HG13	1.61	1.01
1:A:426:ILE:HG13	1:A:464:ILE:HD13	1.40	1.01
2:B:120:ILE:CG2	2:B:154:ILE:HG13	1.90	1.01
2:B:174:ALA:HB1	2:B:211:ALA:HA	1.40	1.01
2:B:291:TYR:CE2	2:B:294:VAL:CB	2.43	1.01
2:B:513:TRP:CG	2:B:551:LEU:HD21	1.96	1.01
2:B:559:ASP:HB3	2:B:563:PHE:CD1	1.93	1.01
3:M:257:ALA:O	3:M:452:ILE:HG23	1.58	1.01
3:M:378:ILE:O	3:M:413:GLY:CA	2.07	1.01
1:A:105:VAL:HG23	4:S:167:ILE:CG1	1.90	1.01

Continued on next page...

Continued from previous page...

Atom-1	Atom-2	Interatomic distance (Å)	Clash overlap (Å)
1:A:136:GLY:O	1:A:139:ASP:CB	2.09	1.01
1:A:516:ILE:HD13	1:A:551:LEU:HA	1.42	1.01
2:B:178:ILE:CD1	2:B:215:TYR:HA	1.89	1.01
3:M:16:PHE:CZ	3:M:18:TYR:HB2	1.96	1.01
3:M:339:GLU:CG	3:M:412:ARG:HG2	1.90	1.01
3:M:379:LEU:CD2	3:M:386:PHE:HB2	1.90	1.01
4:S:48:SER:CA	4:S:77:TYR:HB2	1.91	1.01
2:B:77:ILE:CG2	2:B:82:TYR:HE1	1.73	1.00
2:B:127:LEU:HB3	2:B:157:THR:HG23	1.04	1.00
2:B:178:ILE:HD11	2:B:215:TYR:CA	1.91	1.00
2:B:230:PHE:O	2:B:231:ARG:O	1.79	1.00
2:B:303:LEU:HD11	2:B:333:GLN:CG	1.91	1.00
2:B:393:ILE:CG2	2:B:431:MET:CB	2.38	1.00
2:B:497:LEU:CD2	2:B:533:LEU:CD2	2.38	1.00
2:B:508:ARG:HB2	2:B:544:THR:CG2	1.91	1.00
2:B:508:ARG:HB2	2:B:544:THR:HG23	1.37	1.00
4:S:53:THR:HG22	4:S:57:LEU:HB2	1.39	1.00
1:A:288:THR:CG2	1:A:322:PHE:HZ	1.71	1.00
1:A:429:VAL:CG1	1:A:469:LEU:HD11	1.90	1.00
2:B:77:ILE:CG2	2:B:82:TYR:CE1	2.44	1.00
2:B:127:LEU:HB3	2:B:161:LEU:HD11	1.39	1.00
2:B:223:LEU:HD23	2:B:255:TYR:CE1	1.93	1.00
2:B:226:LEU:HB3	2:B:255:TYR:CZ	1.85	1.00
3:M:344:ILE:CG2	3:M:347:PHE:HB3	1.90	1.00
4:S:73:ILE:HG22	4:S:88:ILE:HG23	1.41	1.00
4:S:80:TYR:O	4:S:81:ALA:C	1.98	1.00
1:A:178:ARG:CD	1:A:209:ASP:OD2	2.08	1.00
1:A:254:ILE:CG1	1:A:290:VAL:HG22	1.91	1.00
2:B:108:PHE:CE2	2:B:115:LEU:CG	2.43	1.00
2:B:185:LYS:HG3	2:B:222:HIS:CE1	1.96	1.00
2:B:189:HIS:NE2	2:B:193:LEU:HD11	1.75	1.00
2:B:393:ILE:HG23	2:B:431:MET:HB3	1.42	1.00
2:B:404:ASN:O	2:B:405:GLU:C	1.80	1.00
2:B:447:GLU:OE1	2:B:485:LYS:HG3	1.61	1.00
3:M:327:PHE:CE1	3:M:336:ASP:HB2	1.97	1.00
3:M:339:GLU:CG	3:M:412:ARG:NE	2.23	1.00
1:A:104:ARG:HG3	1:A:145:ILE:HG13	1.41	1.00
1:A:104:ARG:CG	1:A:145:ILE:HG13	1.91	1.00
1:A:141:VAL:HG21	4:S:158:LYS:N	1.77	1.00
1:A:244:LEU:CD2	1:A:277:LYS:O	2.10	1.00
1:A:408:ILE:HG21	4:S:64:ASN:C	1.82	1.00

Continued on next page...

Continued from previous page...

Atom-1	Atom-2	Interatomic distance (Å)	Clash overlap (Å)
2:B:546:CYS:SG	2:B:607:ILE:CG1	2.40	1.00
3:M:290:PHE:CZ	3:M:297:PHE:CD1	2.49	1.00
1:A:217:ALA:HB2	4:S:142:ILE:HG13	1.43	1.00
1:A:408:ILE:HG23	4:S:64:ASN:HB3	1.01	1.00
2:B:158:VAL:HG12	2:B:195:ILE:HG21	1.40	1.00
3:M:217:ASP:OD1	3:M:471:LYS:HA	1.58	1.00
3:M:353:VAL:HG23	3:M:438:SER:O	1.61	1.00
1:A:166:LEU:HD12	1:A:185:LEU:HD23	1.41	1.00
1:A:220:SER:CB	4:S:142:ILE:CG2	2.32	1.00
1:A:528:ASN:O	1:A:529:GLY:C	1.92	1.00
1:A:633:PHE:HE1	2:B:513:TRP:CE3	1.33	1.00
2:B:380:LYS:NZ	3:M:236:LEU:HG	1.76	1.00
2:B:487:LEU:HD21	2:B:522:GLU:HB3	1.43	1.00
3:M:244:VAL:HA	3:M:472:TYR:HD2	1.26	1.00
3:M:379:LEU:HD23	3:M:386:PHE:HB2	1.41	1.00
2:B:182:ARG:HD2	2:B:217:GLU:HB3	1.41	1.00
3:M:65:TYR:CZ	3:M:86:PRO:CB	2.44	1.00
3:M:69:ILE:HG13	3:M:90:PHE:CE1	1.97	1.00
1:A:429:VAL:HB	1:A:469:LEU:HD11	1.03	0.99
1:A:629:LEU:CG	2:B:610:ARG:HH11	1.74	0.99
2:B:120:ILE:HG23	2:B:154:ILE:HG13	1.43	0.99
3:M:326:HIS:O	3:M:338:PHE:HA	1.61	0.99
4:S:131:VAL:HG22	4:S:153:VAL:HG22	1.40	0.99
2:B:80:GLN:HG2	2:B:115:LEU:CD1	1.91	0.99
2:B:303:LEU:HD13	2:B:333:GLN:HB3	1.05	0.99
2:B:307:ASN:OD1	2:B:339:PHE:CE2	2.15	0.99
2:B:513:TRP:HA	2:B:551:LEU:CD1	1.83	0.99
1:A:275:LEU:CD1	1:A:308:ASP:CG	2.31	0.99
1:A:388:VAL:HG13	1:A:432:ILE:HD12	1.03	0.99
2:B:106:LEU:HD11	2:B:144:ASP:HB3	1.02	0.99
3:M:101:LEU:HD21	3:M:106:LYS:O	1.62	0.99
2:B:63:MET:HG3	2:B:100:LEU:HB3	1.44	0.99
2:B:219:TYR:OH	2:B:226:LEU:CA	2.05	0.99
3:M:339:GLU:CG	3:M:412:ARG:HE	1.75	0.99
1:A:101:GLN:NE2	4:S:167:ILE:CG2	2.25	0.99
2:B:37:TYR:CE2	2:B:38:TYR:CE1	2.50	0.99
2:B:162:VAL:CG2	2:B:199:LEU:CD1	2.40	0.99
2:B:523:PHE:CD1	2:B:559:ASP:OD1	2.16	0.99
2:B:549:LEU:CD2	2:B:607:ILE:O	2.10	0.99
3:M:245:ASP:CA	3:M:472:TYR:CD1	2.45	0.99
3:M:290:PHE:CE1	3:M:297:PHE:CE1	2.50	0.99

Continued on next page...

Continued from previous page...

Atom-1	Atom-2	Interatomic distance (Å)	Clash overlap (Å)
4:S:34:GLN:OE1	4:S:58:LEU:CG	2.09	0.99
1:A:88:ASN:O	1:A:89:PHE:C	1.91	0.99
1:A:128:LEU:CD1	1:A:150:LEU:HD23	1.87	0.99
1:A:638:LEU:HD12	2:B:518:ILE:HG23	1.42	0.99
2:B:121:ASN:OD1	2:B:153:ILE:HD11	1.59	0.99
2:B:159:LYS:HA	2:B:195:ILE:HD11	1.00	0.99
2:B:490:ILE:CG2	2:B:515:PHE:CE2	2.44	0.99
2:B:519:ALA:O	2:B:523:PHE:CG	2.15	0.99
4:S:6:LEU:HD22	4:S:32:LEU:HD22	1.42	0.99
1:A:189:PHE:CD2	1:A:225:LEU:HD21	1.96	0.99
2:B:225:LEU:CD1	2:B:283:TYR:CZ	2.45	0.99
2:B:277:CYS:SG	2:B:292:GLU:CG	2.50	0.99
2:B:592:TYR:OH	2:B:619:ASP:OD2	1.81	0.99
1:A:264:SER:HB2	1:A:271:ARG:CD	1.93	0.99
1:A:275:LEU:CD1	1:A:308:ASP:OD1	2.11	0.99
1:A:287:ALA:HB3	1:A:289:SER:N	1.76	0.99
1:A:563:CYS:HB3	1:A:621:LEU:HD12	1.41	0.99
2:B:279:LEU:HG	2:B:288:TYR:CD1	1.96	0.99
1:A:384:LEU:HD22	1:A:441:TYR:HE2	1.27	0.99
2:B:83:PHE:HZ	2:B:119:SER:CB	1.51	0.99
2:B:86:VAL:HG12	2:B:101:ILE:HG23	1.01	0.99
2:B:243:TRP:HZ3	3:M:91:THR:HA	1.24	0.99
2:B:530:LEU:HD11	2:B:595:VAL:CG2	1.92	0.99
3:M:69:ILE:HD11	3:M:90:PHE:CZ	1.66	0.99
1:A:179:LYS:CE	4:S:143:GLU:CB	2.36	0.99
1:A:217:ALA:CB	4:S:142:ILE:HG13	1.90	0.99
2:B:303:LEU:HD11	2:B:333:GLN:NE2	1.77	0.99
2:B:309:LEU:HB3	2:B:317:VAL:HG11	1.41	0.99
3:M:224:VAL:HG23	3:M:479:PHE:CD1	1.98	0.99
4:S:8:PHE:HB3	4:S:36:TYR:CZ	1.98	0.99
4:S:54:PRO:CG	4:S:57:LEU:HD11	1.89	0.99
1:A:128:LEU:HD13	1:A:150:LEU:HG	1.39	0.98
2:B:60:ARG:HD2	2:B:96:LYS:HG2	1.41	0.98
2:B:274:PRO:CG	2:B:295:ASN:CG	2.32	0.98
2:B:459:GLU:HA	2:B:496:LEU:HD22	1.44	0.98
3:M:66:PHE:HB3	3:M:77:LEU:CD1	1.90	0.98
1:A:183:THR:OG1	4:S:142:ILE:CD1	2.11	0.98
2:B:50:LEU:HB3	2:B:62:ALA:HB2	1.43	0.98
2:B:178:ILE:HG22	2:B:179:LYS:N	1.76	0.98
2:B:350:THR:HB	2:B:352:ASN:ND2	1.78	0.98
3:M:293:PRO:HD2	3:M:293:PRO:O	1.61	0.98

Continued on next page...

Continued from previous page...

Atom-1	Atom-2	Interatomic distance (Å)	Clash overlap (Å)
1:A:388:VAL:CG1	1:A:432:ILE:HD12	1.92	0.98
2:B:127:LEU:CA	2:B:161:LEU:HD11	1.93	0.98
2:B:307:ASN:OD1	2:B:339:PHE:CD2	2.15	0.98
2:B:490:ILE:HG22	2:B:515:PHE:CE2	1.97	0.98
1:A:84:MET:O	1:A:85:ALA:C	1.93	0.98
2:B:78:ASP:OD2	2:B:81:LEU:HG	1.62	0.98
2:B:86:VAL:HG13	2:B:101:ILE:HG12	1.45	0.98
2:B:230:PHE:CZ	2:B:252:LEU:HD23	1.80	0.98
2:B:261:PRO:HA	2:B:290:SER:CB	1.93	0.98
2:B:530:LEU:HD11	2:B:595:VAL:HG21	1.45	0.98
2:B:549:LEU:HD11	2:B:611:ALA:HA	0.99	0.98
3:M:67:SER:OG	3:M:90:PHE:CG	2.16	0.98
1:A:132:LEU:CD2	1:A:169:MET:HG3	1.93	0.98
1:A:215:VAL:CG2	1:A:243:ILE:CD1	2.36	0.98
1:A:408:ILE:CG2	4:S:65:ASN:N	2.14	0.98
2:B:105:LEU:HG	2:B:119:SER:HB2	1.44	0.98
2:B:296:ASP:OD1	2:B:297:PRO:HD2	1.63	0.98
1:A:88:ASN:HB3	1:A:120:ILE:HD12	0.99	0.98
1:A:589:SER:O	1:A:597:GLN:HG3	1.61	0.98
2:B:268:LYS:O	2:B:273:SER:CB	2.12	0.98
2:B:527:PRO:HB2	2:B:587:ARG:CG	1.93	0.98
4:S:130:SER:OG	4:S:156:LEU:HD12	1.59	0.98
1:A:320:HIS:HB2	1:A:352:PHE:CE2	1.99	0.98
1:A:332:TYR:HD1	1:A:366:SER:OG	1.21	0.98
1:A:408:ILE:HG23	4:S:64:ASN:HB2	0.99	0.98
2:B:2:VAL:HG13	2:B:6:HIS:NE2	1.79	0.98
2:B:243:TRP:CZ3	3:M:91:THR:O	2.16	0.98
2:B:275:ARG:CG	2:B:291:TYR:CD2	2.47	0.98
2:B:508:ARG:CB	2:B:544:THR:CG2	2.42	0.98
2:B:549:LEU:CG	2:B:611:ALA:HB2	1.93	0.98
3:M:66:PHE:HA	3:M:77:LEU:CD1	1.94	0.98
1:A:101:GLN:C	4:S:167:ILE:CG1	2.30	0.98
1:A:516:ILE:CG2	1:A:554:ALA:CB	2.34	0.98
2:B:25:VAL:HG11	2:B:36:THR:HG1	1.26	0.98
2:B:140:SER:CA	2:B:172:GLU:OE1	2.11	0.98
2:B:275:ARG:HG3	2:B:294:VAL:HG11	1.02	0.98
2:B:353:GLN:CG	3:M:49:ASP:N	2.19	0.98
1:A:142:LYS:HA	4:S:159:ALA:HB1	1.42	0.98
1:A:288:THR:CG2	1:A:322:PHE:CE2	2.39	0.98
2:B:223:LEU:CD1	2:B:259:TYR:CB	2.31	0.98
2:B:517:GLU:OE2	2:B:554:LYS:NZ	1.96	0.98

Continued on next page...

Continued from previous page...

Atom-1	Atom-2	Interatomic distance (Å)	Clash overlap (Å)
1:A:211:ASP:OD1	4:S:148:ARG:NE	1.96	0.98
1:A:537:THR:HB	1:A:584:PHE:CE1	1.99	0.98
2:B:37:TYR:CZ	2:B:46:GLN:NE2	1.78	0.98
2:B:212:VAL:CG2	2:B:248:LEU:CD2	2.42	0.98
2:B:275:ARG:CB	2:B:291:TYR:HD2	1.76	0.98
2:B:582:ASP:O	2:B:584:SER:N	1.96	0.98
1:A:105:VAL:CG2	4:S:167:ILE:HD13	1.75	0.97
1:A:219:VAL:C	1:A:259:LEU:HD12	1.81	0.97
1:A:275:LEU:HD13	1:A:308:ASP:CG	1.83	0.97
2:B:566:ALA:HA	2:B:574:ASN:CG	1.83	0.97
3:M:281:GLY:O	3:M:282:VAL:HG23	0.80	0.97
4:S:109:LEU:HD11	4:S:113:PHE:CE1	1.92	0.97
1:A:96:SER:N	4:S:166:LYS:NZ	2.10	0.97
1:A:96:SER:HA	1:A:127:LEU:CD1	1.94	0.97
1:A:217:ALA:CA	4:S:142:ILE:CB	1.92	0.97
2:B:86:VAL:HG12	2:B:101:ILE:CG2	1.93	0.97
2:B:90:ILE:HD13	2:B:123:LEU:HD23	1.46	0.97
2:B:279:LEU:CG	2:B:288:TYR:HD1	1.76	0.97
2:B:377:TYR:O	2:B:380:LYS:HB2	1.62	0.97
2:B:418:TYR:CD1	2:B:418:TYR:C	2.33	0.97
2:B:553:ALA:CB	2:B:614:ILE:HG12	1.93	0.97
3:M:241:HIS:HB2	3:M:476:THR:CG2	1.93	0.97
1:A:107:TYR:CE2	1:A:128:LEU:HD23	1.96	0.97
3:M:217:ASP:HB3	3:M:471:LYS:N	1.78	0.97
2:B:37:TYR:CD2	2:B:38:TYR:CE1	2.52	0.97
2:B:191:GLU:O	2:B:193:LEU:N	1.97	0.97
2:B:196:LEU:O	2:B:215:TYR:OH	1.82	0.97
2:B:212:VAL:HG21	2:B:248:LEU:HD21	1.46	0.97
3:M:65:TYR:CD1	3:M:86:PRO:HG3	2.00	0.97
4:S:5:VAL:HG11	4:S:132:LEU:CD2	1.94	0.97
4:S:8:PHE:HE1	4:S:84:TYR:CB	1.70	0.97
1:A:323:CYS:SG	1:A:338:PHE:HE1	1.86	0.97
1:A:627:GLU:CB	2:B:617:LEU:HB3	1.91	0.97
1:A:633:PHE:CD1	2:B:550:VAL:HG11	1.94	0.97
2:B:70:MET:CE	2:B:107:ARG:CG	2.41	0.97
2:B:208:ILE:CG1	2:B:236:ILE:HG21	1.92	0.97
2:B:223:LEU:HD13	2:B:259:TYR:N	1.68	0.97
3:M:347:PHE:HE1	3:M:439:TYR:CD2	1.83	0.97
4:S:53:THR:HG21	4:S:67:GLU:C	1.83	0.97
2:B:102:HIS:HD1	2:B:137:PHE:HB3	1.26	0.97
2:B:219:TYR:CB	2:B:255:TYR:CD1	2.46	0.97

Continued on next page...

Continued from previous page...

Atom-1	Atom-2	Interatomic distance (Å)	Clash overlap (Å)
2:B:343:LEU:CD1	2:B:359:LEU:CD1	2.38	0.97
2:B:346:THR:CG2	2:B:350:THR:HG23	1.94	0.97
3:M:273:HIS:HB2	3:M:298:ARG:O	1.62	0.97
3:M:360:LEU:CD2	3:M:362:PHE:CE2	2.47	0.97
1:A:92:LEU:CD2	1:A:120:ILE:O	2.12	0.97
1:A:212:ILE:HB	1:A:247:ILE:CD1	1.95	0.97
1:A:399:ASP:O	1:A:420:ILE:C	2.02	0.97
2:B:116:THR:HG22	2:B:150:LEU:CD1	1.94	0.97
2:B:127:LEU:CB	2:B:161:LEU:HD11	1.94	0.97
2:B:181:TYR:CE2	2:B:222:HIS:N	2.32	0.97
2:B:344:VAL:HG22	2:B:363:ILE:HD11	1.46	0.97
3:M:319:SER:CB	3:M:346:ASN:N	2.25	0.97
1:A:630:PRO:O	2:B:554:LYS:HA	1.62	0.97
2:B:38:TYR:CD2	2:B:65:ARG:HD3	1.96	0.97
3:M:95:THR:O	3:M:99:ILE:CD1	2.13	0.97
3:M:246:VAL:HA	3:M:470:ALA:CB	1.93	0.97
2:B:87:VAL:HG12	2:B:122:SER:HB3	0.97	0.97
2:B:219:TYR:O	2:B:223:LEU:HG	1.65	0.97
3:M:8:THR:N	3:M:75:TRP:O	1.96	0.97
1:A:88:ASN:HB3	1:A:120:ILE:CD1	1.94	0.97
1:A:557:LYS:NZ	2:B:604:GLU:HG3	1.78	0.97
1:A:584:PHE:O	1:A:587:ASN:N	1.97	0.97
1:A:636:TYR:HB2	2:B:554:LYS:NZ	1.78	0.97
3:M:261:ASN:N	3:M:448:TYR:O	1.97	0.97
1:A:166:LEU:HD13	1:A:185:LEU:HD23	1.45	0.96
1:A:185:LEU:HD13	1:A:203:PHE:HE1	0.82	0.96
1:A:255:ARG:HH22	4:S:135:ILE:HG22	1.29	0.96
2:B:20:ARG:CD	2:B:21:GLU:CB	2.42	0.96
2:B:79:VAL:CB	2:B:108:PHE:HE1	1.74	0.96
2:B:219:TYR:HD1	2:B:226:LEU:HD22	1.21	0.96
2:B:437:SER:HA	2:B:478:LEU:CD2	1.94	0.96
2:B:549:LEU:CD2	2:B:611:ALA:H	1.51	0.96
1:A:77:LEU:O	1:A:80:TYR:O	1.81	0.96
2:B:38:TYR:HA	2:B:42:ILE:N	1.81	0.96
2:B:291:TYR:HD2	2:B:294:VAL:HG11	0.82	0.96
2:B:340:ILE:HG13	2:B:373:LEU:HD23	1.45	0.96
2:B:394:TRP:HZ3	2:B:397:GLN:OE1	1.47	0.96
3:M:224:VAL:N	3:M:479:PHE:HA	1.80	0.96
3:M:302:TYR:CE2	3:M:445:SER:HB3	2.00	0.96
1:A:151:SER:HB2	1:A:187:LYS:CB	1.94	0.96
1:A:260:PHE:CZ	1:A:274:LEU:HD11	2.00	0.96

Continued on next page...

Continued from previous page...

Atom-1	Atom-2	Interatomic distance (Å)	Clash overlap (Å)
1:A:630:PRO:CG	2:B:614:ILE:CB	2.43	0.96
2:B:274:PRO:HG2	2:B:295:ASN:ND2	1.80	0.96
2:B:343:LEU:HD11	2:B:359:LEU:CA	1.94	0.96
2:B:559:ASP:HB2	2:B:563:PHE:HD1	1.20	0.96
3:M:244:VAL:CG1	3:M:472:TYR:CE2	2.47	0.96
4:S:83:LEU:HD11	4:S:116:VAL:HG21	1.44	0.96
1:A:176:TYR:HB3	4:S:155:GLU:CG	1.95	0.96
2:B:278:PRO:CD	2:B:289:PRO:O	2.13	0.96
3:M:4:SER:O	3:M:78:ALA:CB	1.90	0.96
3:M:8:THR:C	3:M:75:TRP:HB2	1.86	0.96
1:A:244:LEU:HD11	1:A:281:LEU:CD1	1.94	0.96
1:A:637:GLU:HB3	2:B:516:GLY:CA	1.95	0.96
2:B:44:PRO:O	2:B:47:LEU:HB2	1.65	0.96
3:M:101:LEU:HA	3:M:109:LEU:HD13	1.46	0.96
3:M:319:SER:CB	3:M:346:ASN:CA	2.42	0.96
1:A:103:LYS:HG3	4:S:163:THR:HG22	1.44	0.96
1:A:540:ILE:HG13	1:A:551:LEU:HD23	1.46	0.96
2:B:197:LYS:HB2	2:B:229:HIS:NE2	1.80	0.96
2:B:216:LYS:HA	2:B:251:LEU:HD11	0.97	0.96
1:A:93:GLU:O	4:S:166:LYS:NZ	1.89	0.96
2:B:139:LEU:HD22	2:B:173:VAL:HA	1.48	0.96
2:B:159:LYS:HE3	2:B:191:GLU:OE1	1.64	0.96
3:M:222:PHE:O	3:M:479:PHE:HE2	1.23	0.96
3:M:226:PHE:CZ	3:M:321:GLY:O	2.18	0.96
2:B:212:VAL:HG22	2:B:248:LEU:CD2	1.95	0.96
2:B:343:LEU:CD2	2:B:362:ALA:CB	2.38	0.96
2:B:343:LEU:CD1	2:B:359:LEU:HA	1.94	0.96
1:A:65:ASN:C	4:S:165:SER:CB	2.34	0.96
1:A:506:LYS:HE2	3:M:58:ARG:HA	1.46	0.96
2:B:151:ALA:CA	2:B:180:LEU:HD11	1.70	0.96
2:B:286:ILE:HG23	2:B:288:TYR:CE2	2.00	0.96
2:B:389:ILE:CG2	2:B:427:ASN:HB2	1.96	0.96
3:M:283:PHE:CE2	3:M:289:THR:HB	1.98	0.96
2:B:366:LEU:O	2:B:367:SER:C	1.85	0.95
3:M:101:LEU:HD11	3:M:106:LYS:C	1.85	0.95
4:S:109:LEU:CD1	4:S:113:PHE:HE1	1.78	0.95
2:B:48:VAL:HG23	2:B:82:TYR:HE2	1.30	0.95
2:B:117:LEU:HD21	2:B:149:SER:HG	1.18	0.95
1:A:101:GLN:HG3	4:S:160:ALA:HB3	1.46	0.95
1:A:316:LEU:HD13	1:A:348:PHE:CG	2.01	0.95
2:B:80:GLN:CG	2:B:115:LEU:HD11	1.94	0.95

Continued on next page...

Continued from previous page...

Atom-1	Atom-2	Interatomic distance (Å)	Clash overlap (Å)
2:B:363:ILE:HG21	2:B:398:ILE:CD1	1.96	0.95
3:M:6:TYR:CD2	3:M:17:GLN:HA	2.00	0.95
3:M:339:GLU:CG	3:M:412:ARG:CD	2.44	0.95
3:M:433:VAL:HG12	3:M:481:VAL:HB	1.47	0.95
4:S:135:ILE:O	4:S:141:VAL:CA	2.13	0.95
1:A:186:PHE:CE1	1:A:224:GLU:CG	2.50	0.95
2:B:230:PHE:HE1	2:B:252:LEU:HD23	1.15	0.95
3:M:243:ILE:O	3:M:472:TYR:CB	2.14	0.95
1:A:64:LEU:CG	1:A:102:GLN:HE22	1.79	0.95
1:A:253:ILE:HG12	1:A:281:LEU:HD13	0.96	0.95
2:B:79:VAL:HB	2:B:108:PHE:HE1	1.17	0.95
2:B:559:ASP:CB	2:B:563:PHE:CE1	2.45	0.95
3:M:101:LEU:CD1	3:M:106:LYS:C	2.34	0.95
4:S:25:LEU:HB2	4:S:26:PRO:HD3	1.49	0.95
2:B:37:TYR:CE2	2:B:38:TYR:HE1	1.84	0.95
2:B:211:ALA:O	2:B:214:ALA:HB3	1.65	0.95
3:M:71:LYS:O	3:M:72:LEU:HB2	1.65	0.95
3:M:244:VAL:HA	3:M:472:TYR:CG	2.02	0.95
4:S:89:VAL:HG11	4:S:98:ILE:HG21	1.45	0.95
1:A:64:LEU:CB	1:A:102:GLN:HE22	1.78	0.95
1:A:140:VAL:CA	1:A:177:ILE:HG13	1.86	0.95
1:A:258:LYS:NZ	4:S:97:ALA:HB2	1.80	0.95
2:B:127:LEU:HD13	2:B:157:THR:CB	1.97	0.95
2:B:185:LYS:HG3	2:B:222:HIS:NE2	1.82	0.95
3:M:243:ILE:O	3:M:472:TYR:HD2	1.49	0.95
3:M:323:MET:HE3	3:M:342:LEU:HD23	1.49	0.95
1:A:186:PHE:CE1	1:A:224:GLU:HB2	2.02	0.95
1:A:403:LEU:CD2	1:A:422:GLU:HG3	1.97	0.95
2:B:196:LEU:HB3	2:B:215:TYR:CE2	2.01	0.95
2:B:268:LYS:O	2:B:273:SER:HB3	1.65	0.95
3:M:51:LEU:HD13	3:M:75:TRP:CZ3	2.01	0.95
4:S:89:VAL:HG11	4:S:98:ILE:CG2	1.95	0.95
1:A:163:ALA:HB2	1:A:195:ALA:HB1	0.96	0.95
1:A:251:TRP:HZ2	4:S:103:GLN:HB3	1.26	0.95
2:B:42:ILE:CD1	2:B:65:ARG:HD3	1.95	0.95
2:B:174:ALA:CB	2:B:211:ALA:CA	2.44	0.95
2:B:174:ALA:CB	2:B:211:ALA:CB	2.44	0.95
2:B:178:ILE:HG13	2:B:214:ALA:CA	1.97	0.95
2:B:437:SER:O	2:B:478:LEU:CD2	2.14	0.95
3:M:18:TYR:CD1	3:M:122:SER:OG	2.20	0.95
3:M:218:LEU:O	3:M:441:GLY:N	2.00	0.95

Continued on next page...

Continued from previous page...

Atom-1	Atom-2	Interatomic distance (Å)	Clash overlap (Å)
3:M:222:PHE:CB	3:M:479:PHE:CZ	2.49	0.95
1:A:145:ILE:CD1	4:S:156:LEU:HD22	1.97	0.95
1:A:186:PHE:CE1	1:A:224:GLU:HG2	2.02	0.95
1:A:610:SER:OG	1:A:625:LEU:HD13	1.67	0.95
1:A:147:LEU:HD13	1:A:166:LEU:HD22	1.47	0.94
1:A:217:ALA:HA	4:S:142:ILE:CG1	1.95	0.94
2:B:117:LEU:CD2	2:B:149:SER:OG	2.14	0.94
2:B:158:VAL:HG11	2:B:177:ILE:HG12	0.96	0.94
2:B:178:ILE:CG1	2:B:214:ALA:HB1	1.97	0.94
3:M:7:ILE:HG12	3:M:76:CYS:SG	2.07	0.94
3:M:347:PHE:CE1	3:M:439:TYR:HD2	1.84	0.94
1:A:64:LEU:CB	1:A:102:GLN:NE2	2.30	0.94
1:A:101:GLN:HG3	4:S:160:ALA:HB1	0.95	0.94
1:A:581:LEU:HD23	1:A:607:LEU:CD2	1.96	0.94
2:B:12:LEU:O	2:B:16:LYS:CB	2.13	0.94
2:B:175:LEU:CD2	2:B:210:CYS:O	2.14	0.94
2:B:267:ASP:H	2:B:289:PRO:HB2	1.32	0.94
3:M:67:SER:CB	3:M:90:PHE:HD1	1.78	0.94
1:A:102:GLN:CD	4:S:166:LYS:H	1.70	0.94
1:A:366:SER:O	1:A:370:LYS:HG2	1.66	0.94
1:A:520:GLY:CA	1:A:558:VAL:CG2	2.42	0.94
2:B:123:LEU:CD1	2:B:142:LEU:CD2	2.39	0.94
2:B:394:TRP:CZ3	2:B:397:GLN:OE1	2.20	0.94
1:A:264:SER:HB2	1:A:271:ARG:HD3	1.49	0.94
1:A:408:ILE:HD12	1:A:410:TYR:CE1	2.03	0.94
2:B:143:SER:O	2:B:145:MET:N	1.99	0.94
2:B:226:LEU:CB	2:B:255:TYR:OH	2.13	0.94
2:B:566:ALA:HA	2:B:574:ASN:HB3	0.96	0.94
3:M:60:LEU:HD22	3:M:62:VAL:HG23	0.95	0.94
3:M:222:PHE:HB2	3:M:479:PHE:HZ	1.28	0.94
3:M:343:ASN:CA	3:M:408:VAL:HG13	1.97	0.94
4:S:131:VAL:HG21	4:S:153:VAL:HG22	1.48	0.94
1:A:244:LEU:HD13	1:A:256:LEU:HD13	1.45	0.94
1:A:485:PRO:O	1:A:488:ARG:HG3	1.67	0.94
1:A:555:LEU:CD1	1:A:581:LEU:HD11	1.98	0.94
2:B:219:TYR:O	2:B:223:LEU:CG	2.15	0.94
4:S:17:VAL:CG2	4:S:19:PHE:CE2	2.51	0.94
4:S:83:LEU:HD11	4:S:116:VAL:HG11	1.47	0.94
1:A:107:TYR:CZ	1:A:128:LEU:HD23	2.02	0.94
2:B:24:ALA:CB	2:B:35:TYR:CE1	2.50	0.94
2:B:62:ALA:C	2:B:66:ILE:CD1	2.35	0.94

Continued on next page...

Continued from previous page...

Atom-1	Atom-2	Interatomic distance (Å)	Clash overlap (Å)
2:B:171:GLY:HA2	2:B:207:VAL:CG1	1.94	0.94
2:B:451:MET:SD	2:B:489:ILE:HG12	2.08	0.94
2:B:476:ARG:HA	2:B:514:LEU:CD1	1.96	0.94
4:S:53:THR:OG1	4:S:68:VAL:C	2.06	0.94
1:A:399:ASP:O	1:A:420:ILE:CA	2.14	0.94
2:B:48:VAL:HG23	2:B:82:TYR:CE2	2.02	0.94
2:B:123:LEU:HD12	2:B:142:LEU:HD21	1.48	0.94
2:B:123:LEU:O	2:B:127:LEU:CG	2.15	0.94
2:B:267:ASP:HB3	2:B:289:PRO:CD	1.98	0.94
2:B:523:PHE:CZ	2:B:580:TYR:CE2	2.44	0.94
3:M:215:TYR:CB	3:M:467:TYR:HD2	1.80	0.94
3:M:443:SER:HB3	3:M:447:ILE:HG12	1.47	0.94
1:A:101:GLN:NE2	4:S:167:ILE:HG22	1.83	0.94
1:A:150:LEU:HB3	1:A:162:ILE:CD1	1.97	0.94
1:A:212:ILE:CG1	4:S:145:ASN:ND2	2.31	0.94
1:A:316:LEU:HD13	1:A:348:PHE:CD2	1.99	0.94
1:A:557:LYS:CE	2:B:606:ASP:N	1.85	0.94
2:B:117:LEU:HD23	2:B:150:LEU:HD23	1.45	0.94
2:B:135:ARG:HB3	2:B:161:LEU:HG	1.48	0.94
2:B:162:VAL:HG21	2:B:195:ILE:C	1.88	0.94
2:B:239:GLN:OE1	3:M:278:ILE:HG23	1.66	0.94
2:B:303:LEU:HD11	2:B:333:GLN:CD	1.88	0.94
2:B:374:PHE:HE1	2:B:381:PHE:CE1	1.86	0.94
3:M:218:LEU:CA	3:M:472:TYR:CE2	2.50	0.94
3:M:432:THR:HG1	3:M:480:GLN:HG3	1.14	0.94
4:S:8:PHE:CE1	4:S:84:TYR:CG	2.56	0.94
1:A:509:PRO:HB3	1:A:547:VAL:HG23	1.50	0.94
2:B:215:TYR:HD2	2:B:219:TYR:HE1	1.04	0.94
2:B:363:ILE:HG22	2:B:398:ILE:HG12	1.48	0.94
3:M:302:TYR:CE2	3:M:445:SER:CB	2.51	0.94
1:A:557:LYS:CE	2:B:606:ASP:CB	2.45	0.94
2:B:103:LEU:HD13	3:M:132:GLY:HA3	1.50	0.94
2:B:556:LEU:HD23	2:B:588:ILE:HG13	1.49	0.94
3:M:272:LEU:HD22	3:M:278:ILE:CB	1.98	0.94
3:M:364:VAL:O	3:M:367:ALA:O	1.85	0.94
1:A:332:TYR:CE1	1:A:366:SER:OG	2.12	0.93
2:B:162:VAL:CG2	2:B:199:LEU:HD11	1.97	0.93
2:B:352:ASN:CB	3:M:49:ASP:CG	2.35	0.93
2:B:353:GLN:CD	3:M:47:SER:HB3	1.83	0.93
2:B:490:ILE:HG13	2:B:518:ILE:HG21	1.51	0.93
2:B:546:CYS:CA	2:B:607:ILE:CG2	2.39	0.93

Continued on next page...

Continued from previous page...

Atom-1	Atom-2	Interatomic distance (Å)	Clash overlap (Å)
2:B:582:ASP:O	2:B:584:SER:CB	2.16	0.93
3:M:319:SER:HG	3:M:346:ASN:HB2	1.19	0.93
1:A:244:LEU:HD23	1:A:277:LYS:O	1.66	0.93
3:M:222:PHE:O	3:M:479:PHE:CZ	2.20	0.93
3:M:443:SER:HG	3:M:447:ILE:C	1.67	0.93
2:B:117:LEU:HD21	2:B:149:SER:CB	1.99	0.93
2:B:223:LEU:HD22	2:B:255:TYR:CD1	2.01	0.93
2:B:256:CYS:HA	2:B:293:VAL:HG21	1.47	0.93
2:B:396:ILE:HD11	2:B:418:TYR:CZ	2.04	0.93
2:B:553:ALA:HB1	2:B:614:ILE:HG12	1.48	0.93
2:B:563:PHE:CD2	2:B:584:SER:HB2	1.95	0.93
3:M:100:LEU:HD22	3:M:101:LEU:N	1.81	0.93
1:A:178:ARG:NH1	1:A:209:ASP:HB3	1.83	0.93
1:A:182:ILE:C	1:A:221:VAL:HG21	1.89	0.93
1:A:207:LEU:HD23	1:A:239:LEU:CB	1.97	0.93
2:B:29:LYS:HE2	2:B:30:LEU:H	0.87	0.93
2:B:106:LEU:HD13	2:B:144:ASP:HB2	0.95	0.93
2:B:216:LYS:HG3	2:B:251:LEU:CD1	1.99	0.93
3:M:215:TYR:HB2	3:M:467:TYR:CD2	2.02	0.93
3:M:219:LEU:HB3	3:M:472:TYR:C	1.87	0.93
3:M:290:PHE:CZ	3:M:297:PHE:CD2	2.57	0.93
1:A:71:VAL:HG12	1:A:105:VAL:HG12	1.47	0.93
1:A:282:MET:O	1:A:283:GLU:C	2.01	0.93
2:B:51:LEU:CD2	2:B:59:VAL:HG13	1.98	0.93
2:B:159:LYS:HD2	2:B:191:GLU:HG3	1.49	0.93
2:B:552:SER:O	2:B:556:LEU:HG	1.68	0.93
3:M:244:VAL:CA	3:M:472:TYR:CE2	2.43	0.93
1:A:180:LYS:HE3	4:S:156:LEU:HD21	1.51	0.93
2:B:158:VAL:HG13	2:B:173:VAL:CG1	1.98	0.93
2:B:267:ASP:C	2:B:276:SER:CB	2.36	0.93
3:M:2:TYR:O	3:M:81:SER:CB	2.16	0.93
3:M:224:VAL:N	3:M:479:PHE:CA	2.31	0.93
3:M:317:MET:HB3	3:M:320:ILE:C	1.87	0.93
3:M:327:PHE:HE1	3:M:336:ASP:CB	1.81	0.93
4:S:63:ASN:O	4:S:66:ASP:OD1	1.85	0.93
1:A:213:SER:OG	4:S:143:GLU:OE1	1.86	0.93
2:B:24:ALA:HB3	2:B:35:TYR:CD2	2.04	0.93
2:B:123:LEU:HD22	2:B:138:ALA:O	1.67	0.93
2:B:311:TYR:HE2	2:B:342:ALA:HB2	0.80	0.93
2:B:549:LEU:CG	2:B:611:ALA:HA	1.98	0.93
3:M:104:PHE:CZ	3:M:113:LYS:NZ	0.73	0.93

Continued on next page...

Continued from previous page...

Atom-1	Atom-2	Interatomic distance (Å)	Clash overlap (Å)
1:A:102:GLN:HB3	4:S:166:LYS:HB2	0.96	0.93
1:A:103:LYS:CG	4:S:163:THR:HG21	1.98	0.93
1:A:401:VAL:HB	1:A:419:ILE:HD12	1.49	0.93
2:B:24:ALA:HB1	2:B:35:TYR:CE1	2.03	0.93
2:B:275:ARG:HG3	2:B:294:VAL:CG1	1.85	0.93
3:M:69:ILE:HD11	3:M:90:PHE:HZ	0.77	0.93
4:S:4:ALA:HA	4:S:18:LYS:O	1.69	0.93
1:A:606:PHE:HZ	2:B:550:VAL:HG11	1.34	0.93
1:A:629:LEU:HD11	2:B:610:ARG:HH11	1.28	0.93
2:B:132:SER:CA	2:B:169:VAL:CG2	2.47	0.93
2:B:252:LEU:HD12	2:B:302:PHE:HE1	1.34	0.93
2:B:549:LEU:HG	2:B:614:ILE:HD12	1.50	0.93
1:A:132:LEU:CD1	1:A:165:ASP:HB3	1.99	0.93
1:A:316:LEU:CD1	1:A:348:PHE:CE2	2.52	0.93
1:A:332:TYR:CZ	1:A:336:ILE:HD11	2.03	0.93
1:A:523:SER:CB	1:A:562:TRP:HE1	1.82	0.93
2:B:67:ILE:CD1	2:B:103:LEU:HB2	1.99	0.93
2:B:219:TYR:O	2:B:223:LEU:HD21	1.66	0.93
2:B:553:ALA:HA	2:B:614:ILE:CG2	1.99	0.93
2:B:559:ASP:O	2:B:562:ASN:C	2.08	0.93
1:A:189:PHE:HD2	1:A:225:LEU:CD2	1.81	0.92
1:A:605:GLU:OE2	1:A:632:PHE:CZ	2.22	0.92
1:A:633:PHE:HD1	2:B:513:TRP:CZ3	1.76	0.92
2:B:106:LEU:HD11	2:B:144:ASP:O	1.56	0.92
2:B:197:LYS:CB	2:B:229:HIS:NE2	2.32	0.92
4:S:87:PHE:CD1	4:S:102:ILE:HG12	2.04	0.92
1:A:99:LYS:NZ	4:S:164:ASP:CB	2.32	0.92
1:A:563:CYS:HA	1:A:566:PHE:CD2	2.03	0.92
2:B:197:LYS:N	2:B:229:HIS:CE1	2.37	0.92
2:B:277:CYS:CA	2:B:292:GLU:HG3	1.99	0.92
2:B:396:ILE:CD1	2:B:418:TYR:CE2	2.52	0.92
2:B:497:LEU:CD2	2:B:533:LEU:HD21	1.97	0.92
3:M:4:SER:O	3:M:78:ALA:HB1	1.17	0.92
3:M:69:ILE:HG13	3:M:90:PHE:HE1	1.32	0.92
4:S:54:PRO:C	4:S:57:LEU:HD13	1.90	0.92
1:A:104:ARG:HA	1:A:145:ILE:CG2	1.97	0.92
1:A:138:ASN:OD1	4:S:158:LYS:CD	2.13	0.92
1:A:141:VAL:CG1	4:S:159:ALA:CB	2.35	0.92
1:A:264:SER:HB3	1:A:271:ARG:CG	1.99	0.92
1:A:319:LEU:O	1:A:320:HIS:C	1.98	0.92
1:A:557:LYS:CB	2:B:605:PHE:HD2	1.80	0.92

Continued on next page...

Continued from previous page...

Atom-1	Atom-2	Interatomic distance (Å)	Clash overlap (Å)
1:A:609:LEU:HD21	1:A:628:VAL:HG21	0.92	0.92
2:B:20:ARG:HG3	2:B:21:GLU:H	1.31	0.92
2:B:182:ARG:CD	2:B:217:GLU:HB3	2.00	0.92
2:B:286:ILE:CG2	2:B:288:TYR:CZ	2.52	0.92
2:B:566:ALA:C	2:B:574:ASN:HB2	1.78	0.92
3:M:52:ASP:HA	3:M:67:SER:C	1.90	0.92
3:M:435:LEU:O	3:M:479:PHE:CG	2.22	0.92
2:B:195:ILE:O	2:B:197:LYS:N	2.02	0.92
2:B:478:LEU:O	2:B:479:VAL:C	2.00	0.92
3:M:81:SER:O	3:M:82:LYS:HB3	1.69	0.92
4:S:15:ARG:HD2	4:S:122:ILE:CG1	2.00	0.92
1:A:216:SER:CB	4:S:143:GLU:HA	1.99	0.92
1:A:609:LEU:HD23	1:A:628:VAL:HG11	1.51	0.92
2:B:176:ALA:O	2:B:178:ILE:N	2.02	0.92
3:M:215:TYR:CE1	3:M:468:LYS:HG2	2.04	0.92
3:M:224:VAL:N	3:M:479:PHE:CB	2.31	0.92
1:A:253:ILE:CD1	1:A:281:LEU:HB3	1.99	0.92
1:A:516:ILE:HG21	1:A:554:ALA:HB3	1.51	0.92
2:B:174:ALA:HB1	2:B:211:ALA:CA	2.00	0.92
2:B:351:GLU:N	2:B:351:GLU:OE2	2.01	0.92
3:M:66:PHE:CA	3:M:77:LEU:CD1	2.41	0.92
3:M:245:ASP:O	3:M:472:TYR:HE1	1.39	0.92
4:S:48:SER:CB	4:S:77:TYR:HB3	1.99	0.92
1:A:244:LEU:HD13	1:A:256:LEU:HD12	1.51	0.92
2:B:62:ALA:O	2:B:66:ILE:HD12	1.66	0.92
2:B:132:SER:CA	2:B:169:VAL:HG21	1.99	0.92
3:M:281:GLY:C	3:M:282:VAL:HG23	1.90	0.92
4:S:53:THR:HG23	4:S:67:GLU:O	1.67	0.92
2:B:102:HIS:O	2:B:103:LEU:C	1.98	0.92
2:B:144:ASP:CA	2:B:179:LYS:NZ	2.27	0.92
2:B:174:ALA:O	2:B:175:LEU:C	1.99	0.92
2:B:219:TYR:CE2	2:B:226:LEU:CB	2.44	0.92
2:B:278:PRO:HA	2:B:288:TYR:O	1.63	0.92
3:M:4:SER:OG	3:M:6:TYR:CE1	2.23	0.92
3:M:256:VAL:CG1	3:M:290:PHE:HB3	1.99	0.92
3:M:347:PHE:CE1	3:M:439:TYR:CD2	2.58	0.92
4:S:5:VAL:O	4:S:17:VAL:HA	1.68	0.92
4:S:7:ILE:HG21	4:S:121:LEU:CD2	2.00	0.92
1:A:103:LYS:HB3	1:A:107:TYR:CE1	2.04	0.92
1:A:105:VAL:CG2	4:S:167:ILE:CG2	2.46	0.92
1:A:170:LEU:HD12	1:A:206:LYS:HG3	1.48	0.92

Continued on next page...

Continued from previous page...

Atom-1	Atom-2	Interatomic distance (Å)	Clash overlap (Å)
2:B:292:GLU:HG2	2:B:296:ASP:HB2	1.52	0.92
2:B:578:PRO:CB	2:B:579:PRO:CD	2.48	0.92
3:M:245:ASP:CB	3:M:472:TYR:CD1	2.52	0.92
1:A:64:LEU:HG	1:A:102:GLN:HE22	1.35	0.92
1:A:144:GLY:HA2	1:A:180:LYS:CB	2.00	0.92
2:B:158:VAL:C	2:B:195:ILE:HD13	1.90	0.92
2:B:483:PRO:HB3	2:B:521:ILE:HG21	0.95	0.92
2:B:578:PRO:HB2	2:B:579:PRO:HD2	1.49	0.92
3:M:254:PRO:HB3	3:M:454:ILE:HD11	1.51	0.92
4:S:8:PHE:HB3	4:S:36:TYR:OH	1.69	0.92
4:S:130:SER:CB	4:S:156:LEU:HD13	1.99	0.92
1:A:189:PHE:HD2	1:A:225:LEU:HD21	1.29	0.91
2:B:151:ALA:HA	2:B:180:LEU:CG	2.00	0.91
2:B:181:TYR:HE2	2:B:222:HIS:N	1.64	0.91
2:B:193:LEU:C	2:B:195:ILE:N	2.20	0.91
2:B:215:TYR:HD2	2:B:219:TYR:CE1	1.87	0.91
2:B:230:PHE:HZ	2:B:252:LEU:HD22	0.87	0.91
4:S:21:THR:HB	4:S:22:PRO:HD2	1.50	0.91
2:B:412:PHE:HE2	2:B:446:TRP:HB3	1.32	0.91
3:M:433:VAL:CG1	3:M:481:VAL:HB	2.00	0.91
1:A:186:PHE:CE1	1:A:224:GLU:CB	2.53	0.91
1:A:291:ILE:HG23	1:A:318:ARG:HB3	0.93	0.91
2:B:93:ASN:HA	2:B:134:LEU:HD11	1.51	0.91
2:B:162:VAL:HG23	2:B:195:ILE:HG23	1.09	0.91
2:B:170:ARG:HA	2:B:199:LEU:HD22	0.92	0.91
2:B:340:ILE:CG1	2:B:373:LEU:HD23	2.01	0.91
4:S:109:LEU:HD13	4:S:113:PHE:CE1	2.03	0.91
1:A:147:LEU:CD2	1:A:166:LEU:HD21	1.92	0.91
1:A:633:PHE:CD2	2:B:550:VAL:HG12	2.02	0.91
2:B:116:THR:CG2	2:B:150:LEU:CD1	2.48	0.91
2:B:154:ILE:CD1	2:B:180:LEU:HD13	2.00	0.91
2:B:252:LEU:HB3	2:B:302:PHE:CD2	2.05	0.91
2:B:274:PRO:CG	2:B:295:ASN:ND2	2.33	0.91
2:B:319:LEU:HD11	2:B:358:MET:SD	2.10	0.91
3:M:256:VAL:O	3:M:289:THR:HA	1.69	0.91
1:A:179:LYS:HE3	4:S:143:GLU:HB2	0.94	0.91
1:A:629:LEU:CD2	2:B:610:ARG:HH11	1.82	0.91
1:A:637:GLU:HG3	2:B:516:GLY:N	1.84	0.91
2:B:286:ILE:HG23	2:B:288:TYR:CZ	2.06	0.91
3:M:19:LEU:HD13	3:M:24:ALA:HB3	1.51	0.91
4:S:16:LEU:HD23	4:S:128:LEU:HD23	1.50	0.91

Continued on next page...

Continued from previous page...

Atom-1	Atom-2	Interatomic distance (Å)	Clash overlap (Å)
1:A:103:LYS:HD3	1:A:131:ASP:CG	1.91	0.91
2:B:87:VAL:HG11	2:B:122:SER:HB3	1.48	0.91
2:B:237:ILE:HG21	2:B:305:SER:HB3	1.50	0.91
2:B:319:LEU:HD13	2:B:354:GLY:O	1.69	0.91
2:B:518:ILE:O	2:B:518:ILE:HD12	1.70	0.91
3:M:2:TYR:N	3:M:81:SER:OG	2.04	0.91
1:A:99:LYS:HZ3	4:S:164:ASP:HB2	1.28	0.91
1:A:143:VAL:O	1:A:147:LEU:HG	1.71	0.91
1:A:147:LEU:HD13	1:A:181:ALA:HA	1.49	0.91
2:B:307:ASN:HD21	2:B:336:ASN:HD22	1.13	0.91
2:B:549:LEU:CG	2:B:611:ALA:CA	2.49	0.91
3:M:101:LEU:HG	3:M:106:LYS:C	1.89	0.91
3:M:362:PHE:O	3:M:363:ASN:C	2.07	0.91
1:A:102:GLN:CB	4:S:163:THR:CB	2.27	0.91
2:B:143:SER:CB	2:B:179:LYS:HD2	2.01	0.91
2:B:219:TYR:CE2	2:B:226:LEU:N	2.28	0.91
3:M:45:SER:CA	3:M:47:SER:H	1.83	0.91
1:A:220:SER:HB3	4:S:142:ILE:HG23	1.51	0.91
1:A:250:ASN:OD1	1:A:285:THR:HG21	1.69	0.91
1:A:403:LEU:CD2	1:A:422:GLU:CD	2.38	0.91
1:A:573:GLU:O	1:A:574:ILE:C	1.97	0.91
1:A:631:SER:CA	2:B:557:SER:HB2	1.98	0.91
2:B:351:GLU:O	3:M:48:ASP:HB2	1.70	0.91
2:B:486:HIS:CD2	2:B:518:ILE:HG12	2.06	0.91
2:B:596:LEU:HD22	2:B:615:SER:CB	2.00	0.91
3:M:245:ASP:C	3:M:472:TYR:CE1	2.44	0.91
3:M:262:THR:HG22	3:M:264:GLY:N	1.86	0.91
4:S:53:THR:O	4:S:69:ASN:ND2	2.04	0.91
4:S:53:THR:N	4:S:69:ASN:HB2	1.85	0.91
1:A:100:LEU:O	4:S:160:ALA:HA	1.30	0.91
1:A:101:GLN:CG	4:S:160:ALA:CB	2.17	0.91
1:A:102:GLN:HA	4:S:167:ILE:HG12	0.92	0.91
1:A:148:SER:O	1:A:151:SER:OG	1.87	0.91
2:B:70:MET:HE3	2:B:107:ARG:CG	2.01	0.91
3:M:344:ILE:HB	3:M:407:THR:O	1.70	0.91
4:S:16:LEU:HD12	4:S:17:VAL:H	1.35	0.91
1:A:65:ASN:C	4:S:165:SER:HB2	1.91	0.90
1:A:80:TYR:CB	1:A:82:PHE:CE2	2.54	0.90
1:A:623:MET:O	2:B:617:LEU:HD21	1.71	0.90
2:B:588:ILE:HG23	2:B:618:PHE:HZ	1.31	0.90
3:M:45:SER:HA	3:M:47:SER:H	1.36	0.90

Continued on next page...

Continued from previous page...

Atom-1	Atom-2	Interatomic distance (Å)	Clash overlap (Å)
3:M:65:TYR:CD1	3:M:86:PRO:HB3	2.05	0.90
1:A:92:LEU:HD13	1:A:123:LEU:HD12	1.50	0.90
1:A:179:LYS:CD	4:S:143:GLU:HB2	1.99	0.90
1:A:381:GLU:O	1:A:382:ASP:C	1.97	0.90
2:B:346:THR:HG22	2:B:350:THR:CG2	2.00	0.90
3:M:92:PHE:HZ	3:M:129:VAL:CG2	1.84	0.90
1:A:105:VAL:HG21	4:S:167:ILE:CA	2.01	0.90
1:A:609:LEU:HG	1:A:628:VAL:HB	0.90	0.90
1:A:638:LEU:CD1	2:B:518:ILE:HG23	2.00	0.90
2:B:20:ARG:NH1	2:B:35:TYR:OH	2.03	0.90
2:B:29:LYS:CD	2:B:30:LEU:H	1.83	0.90
2:B:441:GLN:HG2	2:B:481:LYS:HG3	1.53	0.90
4:S:8:PHE:HE1	4:S:84:TYR:CG	1.89	0.90
1:A:634:ASN:O	2:B:517:GLU:N	2.02	0.90
2:B:353:GLN:HB2	3:M:49:ASP:N	1.84	0.90
3:M:317:MET:HB2	3:M:322:LEU:N	1.84	0.90
1:A:67:LYS:C	4:S:166:LYS:HA	1.91	0.90
1:A:107:TYR:CZ	1:A:128:LEU:CD2	2.54	0.90
1:A:326:GLN:HA	1:A:331:ARG:HH21	1.33	0.90
2:B:123:LEU:HD12	2:B:142:LEU:HD23	1.50	0.90
2:B:354:GLY:O	2:B:358:MET:HG2	1.71	0.90
2:B:513:TRP:N	2:B:551:LEU:HD11	1.85	0.90
2:B:537:PHE:CZ	2:B:545:ARG:CG	2.54	0.90
3:M:375:LYS:N	3:M:416:GLU:O	2.03	0.90
3:M:379:LEU:HD22	3:M:386:PHE:HD1	1.34	0.90
4:S:56:SER:O	4:S:60:SER:OG	1.88	0.90
1:A:102:GLN:HE21	4:S:165:SER:H	1.18	0.90
1:A:224:GLU:OE1	4:S:138:GLY:O	1.90	0.90
2:B:20:ARG:HH12	2:B:21:GLU:HG3	1.34	0.90
2:B:83:PHE:CZ	2:B:119:SER:HB2	2.05	0.90
2:B:108:PHE:CE2	2:B:115:LEU:HG	2.05	0.90
3:M:223:HIS:HB3	3:M:478:ASN:HA	1.54	0.90
4:S:135:ILE:HG22	4:S:141:VAL:HG22	1.51	0.90
1:A:403:LEU:HD21	1:A:422:GLU:N	1.86	0.90
1:A:454:ILE:HG23	1:A:473:ILE:HG23	1.51	0.90
1:A:498:LEU:O	1:A:501:ASN:O	1.88	0.90
2:B:63:MET:CE	2:B:104:TYR:HB2	2.02	0.90
2:B:70:MET:SD	2:B:107:ARG:HB2	2.11	0.90
2:B:527:PRO:HB2	2:B:587:ARG:HG3	1.52	0.90
2:B:553:ALA:CB	2:B:614:ILE:CG1	2.48	0.90
4:S:15:ARG:NH1	4:S:122:ILE:CD1	2.35	0.90

Continued on next page...

Continued from previous page...

Atom-1	Atom-2	Interatomic distance (Å)	Clash overlap (Å)
4:S:17:VAL:HG21	4:S:19:PHE:HZ	1.22	0.90
1:A:96:SER:HA	1:A:127:LEU:CG	2.00	0.90
2:B:135:ARG:HH22	2:B:164:ASP:CG	1.73	0.90
2:B:278:PRO:HB3	2:B:288:TYR:O	1.69	0.90
2:B:319:LEU:HD12	2:B:358:MET:HG3	0.91	0.90
2:B:418:TYR:CD1	2:B:424:PHE:CE1	2.59	0.90
2:B:525:ILE:C	2:B:527:PRO:HD2	1.92	0.90
1:A:634:ASN:O	2:B:517:GLU:HA	1.71	0.90
2:B:67:ILE:HD11	2:B:103:LEU:HB2	1.51	0.90
2:B:219:TYR:CD1	2:B:255:TYR:CE1	2.49	0.90
2:B:360:LEU:HD13	2:B:391:ALA:HA	1.51	0.90
2:B:549:LEU:HD22	2:B:611:ALA:HB2	0.91	0.90
2:B:602:ASP:O	2:B:608:ARG:NH2	2.04	0.90
3:M:104:PHE:HE1	3:M:113:LYS:CE	1.42	0.90
3:M:219:LEU:CB	3:M:472:TYR:C	2.39	0.90
1:A:186:PHE:CD1	1:A:224:GLU:CB	2.54	0.90
1:A:631:SER:HA	2:B:557:SER:HB2	1.53	0.90
1:A:637:GLU:OE1	2:B:513:TRP:HD1	1.50	0.90
2:B:155:LEU:HB2	2:B:188:TYR:CD2	2.07	0.90
2:B:311:TYR:CE2	2:B:342:ALA:HB1	2.07	0.90
2:B:319:LEU:HD13	2:B:358:MET:CG	1.72	0.90
3:M:222:PHE:CD1	3:M:240:ILE:CG2	2.55	0.90
3:M:379:LEU:CD2	3:M:386:PHE:CG	2.51	0.90
4:S:39:ILE:CD1	4:S:77:TYR:CD2	2.54	0.90
1:A:105:VAL:HG21	4:S:167:ILE:CG2	2.01	0.89
2:B:20:ARG:CZ	2:B:21:GLU:CG	2.37	0.89
2:B:563:PHE:CE2	2:B:584:SER:CA	2.55	0.89
3:M:101:LEU:HG	3:M:106:LYS:CB	2.02	0.89
3:M:265:ASN:OD1	3:M:313:SER:CB	2.18	0.89
1:A:102:GLN:CB	4:S:163:THR:CA	2.48	0.89
1:A:533:ILE:CG1	1:A:562:TRP:CH2	2.56	0.89
1:A:531:ASP:C	1:A:534:LYS:O	2.10	0.89
2:B:237:ILE:O	2:B:238:LYS:C	2.04	0.89
2:B:286:ILE:HG21	2:B:288:TYR:OH	1.73	0.89
3:M:101:LEU:CG	3:M:106:LYS:C	2.41	0.89
3:M:215:TYR:CB	3:M:467:TYR:CD2	2.54	0.89
1:A:219:VAL:CG1	1:A:259:LEU:CD1	2.51	0.89
2:B:346:THR:HG22	2:B:350:THR:HG23	1.53	0.89
3:M:219:LEU:HD22	3:M:473:LYS:CA	2.00	0.89
1:A:99:LYS:HD3	4:S:164:ASP:N	1.86	0.89
1:A:557:LYS:CE	2:B:606:ASP:HB2	2.03	0.89

Continued on next page...

Continued from previous page...

Atom-1	Atom-2	Interatomic distance (Å)	Clash overlap (Å)
1:A:605:GLU:HG3	1:A:632:PHE:CG	2.07	0.89
2:B:24:ALA:HB1	2:B:35:TYR:CD1	2.07	0.89
2:B:120:ILE:HD13	2:B:142:LEU:HD23	1.54	0.89
2:B:275:ARG:HB2	2:B:294:VAL:HG13	0.90	0.89
2:B:563:PHE:CD2	2:B:584:SER:CA	2.55	0.89
3:M:224:VAL:H	3:M:479:PHE:CA	1.85	0.89
3:M:246:VAL:HA	3:M:470:ALA:HB2	1.52	0.89
1:A:531:ASP:O	1:A:534:LYS:O	1.90	0.89
2:B:378:THR:HG23	2:B:379:LYS:N	1.86	0.89
2:B:562:ASN:HB3	2:B:580:TYR:CB	2.02	0.89
3:M:243:ILE:O	3:M:472:TYR:HB2	1.72	0.89
3:M:338:PHE:CD2	3:M:415:ILE:CG1	2.56	0.89
1:A:214:VAL:CG2	4:S:148:ARG:NH2	2.35	0.89
1:A:225:LEU:CD1	1:A:233:PHE:HZ	1.72	0.89
1:A:372:ILE:HG21	1:A:427:LYS:HE3	1.53	0.89
1:A:586:GLU:HB2	1:A:604:LEU:HD11	1.54	0.89
1:A:631:SER:HB3	2:B:557:SER:CB	1.93	0.89
2:B:140:SER:HB2	2:B:172:GLU:OE2	1.71	0.89
2:B:177:ILE:HD13	2:B:196:LEU:HG	1.54	0.89
2:B:216:LYS:CA	2:B:251:LEU:HD11	1.86	0.89
2:B:243:TRP:CZ2	3:M:95:THR:N	2.40	0.89
2:B:353:GLN:HG3	3:M:47:SER:CA	2.02	0.89
2:B:474:VAL:O	2:B:477:MET:N	2.06	0.89
3:M:436:GLU:HA	3:M:479:PHE:CE2	2.08	0.89
4:S:109:LEU:HD13	4:S:113:PHE:HE1	1.36	0.89
1:A:254:ILE:HG12	1:A:290:VAL:HG22	1.52	0.89
1:A:392:MET:SD	1:A:457:LEU:CD2	2.61	0.89
2:B:20:ARG:CZ	2:B:21:GLU:CB	2.51	0.89
2:B:226:LEU:CB	2:B:255:TYR:CZ	2.42	0.89
2:B:291:TYR:CE2	2:B:294:VAL:CG1	2.55	0.89
2:B:475:ILE:HG22	2:B:514:LEU:HD21	1.54	0.89
3:M:479:PHE:H	3:M:479:PHE:HD2	1.17	0.89
1:A:147:LEU:HB3	1:A:184:ALA:HB2	1.55	0.89
1:A:288:THR:HG1	1:A:291:ILE:HB	1.38	0.89
2:B:346:THR:CG2	2:B:350:THR:CG2	2.50	0.89
2:B:415:LEU:HD13	2:B:436:LEU:CD2	2.02	0.89
2:B:553:ALA:CB	2:B:614:ILE:CD1	2.51	0.89
3:M:218:LEU:HG	3:M:244:VAL:HG22	1.54	0.89
3:M:255:LEU:O	3:M:454:ILE:HA	1.71	0.89
4:S:53:THR:HB	4:S:69:ASN:H	1.33	0.89
1:A:128:LEU:HD12	1:A:150:LEU:CD2	1.77	0.89

Continued on next page...

Continued from previous page...

Atom-1	Atom-2	Interatomic distance (Å)	Clash overlap (Å)
1:A:147:LEU:HD13	1:A:166:LEU:CD2	2.02	0.89
1:A:150:LEU:HD13	1:A:162:ILE:HG12	1.52	0.89
1:A:605:GLU:CG	1:A:632:PHE:CG	2.55	0.89
2:B:161:LEU:HB3	2:B:173:VAL:HG22	1.55	0.89
2:B:219:TYR:C	2:B:223:LEU:HD21	1.93	0.89
2:B:219:TYR:HB2	2:B:255:TYR:CD1	2.07	0.89
4:S:54:PRO:HG2	4:S:57:LEU:HD11	1.55	0.89
1:A:95:MET:SD	1:A:107:TYR:CD2	2.65	0.88
2:B:278:PRO:CA	2:B:288:TYR:CB	2.50	0.88
3:M:219:LEU:CG	3:M:472:TYR:O	2.21	0.88
1:A:105:VAL:CB	4:S:167:ILE:CD1	2.33	0.88
1:A:516:ILE:HG22	1:A:554:ALA:HB1	1.54	0.88
1:A:630:PRO:CG	2:B:614:ILE:HA	2.02	0.88
2:B:56:SER:HB2	2:B:92:THR:HG21	1.55	0.88
2:B:79:VAL:HG23	2:B:108:PHE:CE1	2.06	0.88
2:B:208:ILE:HD13	2:B:236:ILE:HG22	1.54	0.88
2:B:275:ARG:CG	2:B:291:TYR:HD2	1.83	0.88
3:M:242:GLY:CA	3:M:444:ALA:HB2	2.03	0.88
1:A:101:GLN:O	4:S:167:ILE:HG13	1.73	0.88
1:A:213:SER:C	4:S:143:GLU:OE1	2.12	0.88
1:A:217:ALA:CB	4:S:143:GLU:HG3	2.02	0.88
1:A:316:LEU:HD12	1:A:348:PHE:CE2	2.07	0.88
2:B:98:LYS:HD2	2:B:138:ALA:HB2	1.55	0.88
2:B:219:TYR:HB3	2:B:223:LEU:HD21	1.50	0.88
2:B:274:PRO:CD	2:B:295:ASN:ND2	2.36	0.88
2:B:337:THR:HG23	2:B:373:LEU:HD11	1.55	0.88
2:B:550:VAL:HG22	2:B:610:ARG:CD	2.02	0.88
1:A:96:SER:CA	1:A:127:LEU:CD2	2.37	0.88
1:A:102:GLN:CA	4:S:167:ILE:CG1	2.49	0.88
1:A:216:SER:O	1:A:219:VAL:HB	1.71	0.88
1:A:320:HIS:HB2	1:A:352:PHE:HE2	1.33	0.88
1:A:326:GLN:HA	1:A:331:ARG:CZ	2.02	0.88
2:B:20:ARG:CZ	2:B:21:GLU:HB2	2.03	0.88
2:B:227:HIS:NE2	2:B:292:GLU:OE2	2.06	0.88
2:B:268:LYS:O	2:B:273:SER:OG	1.91	0.88
2:B:303:LEU:HD13	2:B:333:GLN:CB	1.97	0.88
3:M:283:PHE:HE2	3:M:289:THR:HB	1.37	0.88
1:A:225:LEU:CB	1:A:233:PHE:CE1	2.55	0.88
2:B:309:LEU:CB	2:B:317:VAL:HG12	1.99	0.88
3:M:10:THR:HA	3:M:75:TRP:NE1	1.88	0.88
3:M:374:TYR:HB3	3:M:417:TYR:HD2	1.38	0.88

Continued on next page...

Continued from previous page...

Atom-1	Atom-2	Interatomic distance (Å)	Clash overlap (Å)
1:A:64:LEU:HG	1:A:102:GLN:NE2	1.87	0.88
1:A:103:LYS:N	4:S:163:THR:HG21	1.89	0.88
1:A:141:VAL:C	4:S:159:ALA:CB	2.41	0.88
1:A:513:ARG:HD2	1:A:550:VAL:HG21	0.88	0.88
2:B:105:LEU:O	2:B:106:LEU:C	2.00	0.88
2:B:592:TYR:OH	2:B:619:ASP:CG	2.11	0.88
1:A:151:SER:HB2	1:A:187:LYS:HB2	1.55	0.88
2:B:78:ASP:OD1	2:B:80:GLN:HB2	1.73	0.88
2:B:116:THR:HG21	2:B:150:LEU:HD11	1.55	0.88
3:M:220:GLU:HG3	3:M:439:TYR:HD1	1.36	0.88
3:M:272:LEU:HD21	3:M:278:ILE:HB	1.54	0.88
4:S:53:THR:CG2	4:S:57:LEU:HB2	2.04	0.88
1:A:96:SER:CA	1:A:127:LEU:CG	2.52	0.88
1:A:154:ILE:HG22	1:A:191:GLN:HG3	1.54	0.88
1:A:264:SER:CB	1:A:271:ARG:HD3	2.03	0.88
1:A:595:GLU:OE2	2:B:472:VAL:HB	1.73	0.88
2:B:120:ILE:HG13	2:B:150:LEU:HB3	1.54	0.88
2:B:158:VAL:CG1	2:B:177:ILE:HG12	1.84	0.88
2:B:260:LEU:HD22	2:B:291:TYR:OH	0.70	0.88
3:M:214:LEU:CD1	3:M:256:VAL:HG21	2.04	0.88
3:M:220:GLU:HG3	3:M:439:TYR:CD1	2.08	0.88
3:M:334:ASP:O	3:M:417:TYR:N	2.06	0.88
1:A:67:LYS:CB	4:S:165:SER:OG	2.22	0.88
1:A:100:LEU:HG	4:S:162:SER:H	1.38	0.88
1:A:537:THR:HB	1:A:584:PHE:CD1	2.08	0.88
2:B:162:VAL:CG2	2:B:199:LEU:HG	2.03	0.88
2:B:353:GLN:CG	3:M:48:ASP:N	2.36	0.88
2:B:513:TRP:H	2:B:551:LEU:CD1	1.83	0.88
1:A:536:MET:O	1:A:537:THR:C	1.98	0.88
2:B:178:ILE:HD13	2:B:218:CYS:HB3	1.55	0.88
2:B:527:PRO:HG2	2:B:587:ARG:CG	2.02	0.88
2:B:531:ARG:N	2:B:591:MET:SD	2.46	0.88
3:M:226:PHE:HB2	3:M:481:VAL:CG2	2.04	0.88
3:M:317:MET:HB2	3:M:321:GLY:C	1.94	0.88
4:S:34:GLN:CD	4:S:58:LEU:HD11	1.95	0.88
4:S:73:ILE:HG23	4:S:88:ILE:HG23	1.37	0.88
2:B:360:LEU:CD1	2:B:391:ALA:CA	2.52	0.87
2:B:577:ASN:O	2:B:578:PRO:O	1.93	0.87
3:M:4:SER:O	3:M:78:ALA:CA	2.21	0.87
4:S:17:VAL:HG21	4:S:19:PHE:CE2	2.09	0.87
1:A:178:ARG:NE	1:A:209:ASP:OD2	2.07	0.87

Continued on next page...

Continued from previous page...

Atom-1	Atom-2	Interatomic distance (Å)	Clash overlap (Å)
1:A:464:ILE:O	1:A:465:SER:C	1.96	0.87
2:B:215:TYR:HE2	2:B:229:HIS:HD1	1.22	0.87
2:B:317:VAL:O	2:B:321:CYS:SG	2.32	0.87
3:M:243:ILE:CB	3:M:473:LYS:O	2.21	0.87
3:M:245:ASP:HB3	3:M:472:TYR:HD1	1.39	0.87
3:M:336:ASP:OD2	3:M:415:ILE:HB	1.74	0.87
4:S:130:SER:OG	4:S:156:LEU:HD13	1.70	0.87
1:A:64:LEU:CA	1:A:102:GLN:HE22	1.88	0.87
1:A:212:ILE:HD12	4:S:145:ASN:CG	1.95	0.87
1:A:629:LEU:HD21	2:B:610:ARG:HH11	1.38	0.87
2:B:127:LEU:HB3	2:B:157:THR:CG2	1.95	0.87
2:B:154:ILE:HD12	2:B:180:LEU:CG	2.04	0.87
2:B:247:TYR:HE2	3:M:91:THR:HG21	1.09	0.87
2:B:278:PRO:C	2:B:288:TYR:CB	2.38	0.87
2:B:393:ILE:HG23	2:B:431:MET:HG2	1.35	0.87
2:B:451:MET:HG3	2:B:489:ILE:CD1	2.04	0.87
3:M:215:TYR:N	3:M:467:TYR:HB3	1.88	0.87
4:S:54:PRO:O	4:S:57:LEU:HD13	1.72	0.87
1:A:140:VAL:CB	1:A:177:ILE:HG13	2.02	0.87
1:A:633:PHE:HD2	2:B:550:VAL:C	1.74	0.87
2:B:215:TYR:CD2	2:B:219:TYR:HE1	1.92	0.87
2:B:344:VAL:CG1	2:B:381:PHE:CZ	2.58	0.87
2:B:374:PHE:CE1	2:B:381:PHE:CE1	2.63	0.87
3:M:215:TYR:HB3	3:M:467:TYR:HD2	1.39	0.87
3:M:454:ILE:HG21	3:M:464:THR:HG21	0.88	0.87
1:A:557:LYS:HB3	2:B:605:PHE:HD2	1.24	0.87
2:B:121:ASN:OD1	2:B:153:ILE:HD13	1.73	0.87
2:B:174:ALA:HB3	2:B:211:ALA:HA	1.56	0.87
2:B:321:CYS:O	2:B:325:LEU:HG	1.73	0.87
2:B:592:TYR:OH	2:B:619:ASP:OD1	1.93	0.87
3:M:340:LEU:HB3	3:M:411:LEU:HG	1.54	0.87
3:M:374:TYR:OH	3:M:394:GLN:C	2.13	0.87
1:A:105:VAL:H	4:S:167:ILE:HD11	0.72	0.87
1:A:461:CYS:SG	1:A:469:LEU:HD23	2.14	0.87
2:B:20:ARG:NH1	2:B:21:GLU:HG2	1.85	0.87
2:B:143:SER:HA	2:B:179:LYS:HB2	1.56	0.87
2:B:178:ILE:HG21	2:B:214:ALA:HA	1.55	0.87
2:B:297:PRO:O	2:B:301:LEU:HG	1.73	0.87
2:B:513:TRP:CB	2:B:551:LEU:HD21	2.02	0.87
3:M:3:LEU:HB2	3:M:20:LEU:HD12	1.55	0.87
3:M:18:TYR:HD1	3:M:122:SER:HA	1.04	0.87

Continued on next page...

Continued from previous page...

Atom-1	Atom-2	Interatomic distance (Å)	Clash overlap (Å)
3:M:217:ASP:CG	3:M:470:ALA:O	2.13	0.87
3:M:259:LYS:O	3:M:449:VAL:HA	1.73	0.87
1:A:557:LYS:CB	2:B:605:PHE:CD2	2.55	0.87
2:B:83:PHE:HE2	2:B:119:SER:CB	1.51	0.87
2:B:214:ALA:O	2:B:216:LYS:N	2.07	0.87
3:M:214:LEU:C	3:M:467:TYR:CB	2.43	0.87
1:A:294:SER:O	1:A:298:ILE:HG12	1.75	0.87
2:B:153:ILE:O	2:B:155:LEU:N	2.08	0.87
2:B:223:LEU:CD2	2:B:255:TYR:CD1	2.56	0.87
2:B:236:ILE:HG22	2:B:240:LEU:HD11	1.54	0.87
2:B:408:VAL:CG1	2:B:412:PHE:CE2	2.58	0.87
2:B:497:LEU:O	2:B:498:THR:C	2.03	0.87
2:B:559:ASP:HA	2:B:562:ASN:HB2	1.53	0.87
3:M:44:ASP:O	3:M:46:SER:N	2.07	0.87
3:M:212:ASN:HB3	3:M:250:LEU:HD23	1.56	0.87
3:M:219:LEU:O	3:M:474:THR:CG2	2.23	0.87
3:M:306:LEU:O	3:M:307:SER:C	2.04	0.87
4:S:105:PHE:CZ	4:S:128:LEU:HD11	2.10	0.87
1:A:213:SER:CB	4:S:143:GLU:OE1	2.23	0.87
1:A:503:ASN:OD1	3:M:59:ASP:OD2	1.93	0.87
2:B:106:LEU:CD1	2:B:144:ASP:C	2.43	0.87
2:B:215:TYR:CD2	2:B:226:LEU:CD1	2.58	0.87
2:B:219:TYR:CB	2:B:226:LEU:HD22	2.02	0.87
2:B:452:LYS:NZ	2:B:456:ASP:OD1	2.08	0.87
3:M:219:LEU:CD2	3:M:473:LYS:HA	2.04	0.87
4:S:83:LEU:HD11	4:S:116:VAL:CG2	2.05	0.87
1:A:67:LYS:HG3	4:S:165:SER:HG	1.40	0.86
1:A:125:THR:HG1	1:A:158:LEU:HD13	1.40	0.86
1:A:633:PHE:CE1	2:B:513:TRP:HE3	1.39	0.86
2:B:196:LEU:HB2	2:B:229:HIS:ND1	1.90	0.86
2:B:208:ILE:CD1	2:B:236:ILE:HG23	1.80	0.86
2:B:267:ASP:O	2:B:276:SER:OG	1.93	0.86
2:B:378:THR:HG23	2:B:379:LYS:H	1.38	0.86
3:M:223:HIS:HD2	3:M:478:ASN:HB2	1.40	0.86
3:M:379:LEU:CD2	3:M:386:PHE:CB	2.53	0.86
4:S:50:PHE:HB2	4:S:76:ILE:HD13	1.56	0.86
1:A:76:TYR:HH	4:S:125:TRP:HZ3	1.20	0.86
1:A:264:SER:CB	1:A:271:ARG:CG	2.54	0.86
1:A:633:PHE:HD2	2:B:551:LEU:HA	1.09	0.86
2:B:165:PRO:HA	2:B:170:ARG:HH21	1.38	0.86
2:B:245:GLN:OE1	2:B:309:LEU:HD12	1.75	0.86

Continued on next page...

Continued from previous page...

Atom-1	Atom-2	Interatomic distance (Å)	Clash overlap (Å)
2:B:252:LEU:HD13	2:B:302:PHE:HD1	1.04	0.86
2:B:267:ASP:H	2:B:289:PRO:CG	1.87	0.86
1:A:64:LEU:HA	1:A:102:GLN:NE2	1.90	0.86
2:B:232:ARG:O	2:B:236:ILE:HG13	1.74	0.86
4:S:111:ARG:HB2	4:S:150:VAL:HG22	1.58	0.86
2:B:219:TYR:CD2	2:B:223:LEU:HA	2.11	0.86
2:B:243:TRP:CH2	3:M:91:THR:O	2.27	0.86
2:B:353:GLN:HB2	3:M:50:TYR:N	1.87	0.86
3:M:223:HIS:CG	3:M:478:ASN:HA	2.11	0.86
1:A:426:ILE:HG13	1:A:464:ILE:CD1	2.04	0.86
1:A:637:GLU:O	2:B:518:ILE:N	1.86	0.86
2:B:161:LEU:HB3	2:B:173:VAL:CG2	2.05	0.86
2:B:232:ARG:HG3	2:B:236:ILE:CD1	2.06	0.86
2:B:252:LEU:CB	2:B:302:PHE:CZ	2.59	0.86
2:B:275:ARG:HB3	2:B:291:TYR:CB	2.02	0.86
2:B:143:SER:CA	2:B:179:LYS:HD2	2.05	0.86
3:M:45:SER:HB2	3:M:51:LEU:CD1	2.05	0.86
3:M:242:GLY:HA3	3:M:444:ALA:HB2	1.58	0.86
1:A:76:TYR:OH	4:S:125:TRP:HZ3	1.57	0.86
1:A:316:LEU:HD11	1:A:348:PHE:CD2	2.07	0.86
1:A:421:PRO:HG2	1:A:424:TYR:CD1	2.11	0.86
1:A:630:PRO:O	2:B:554:LYS:N	2.07	0.86
2:B:232:ARG:HG3	2:B:236:ILE:HD11	1.58	0.86
2:B:389:ILE:HG21	2:B:427:ASN:HB2	1.56	0.86
4:S:8:PHE:CZ	4:S:84:TYR:HB2	1.96	0.86
4:S:135:ILE:HG23	4:S:141:VAL:CG1	2.04	0.86
1:A:141:VAL:CB	4:S:159:ALA:N	2.38	0.86
2:B:178:ILE:CD1	2:B:215:TYR:CA	2.51	0.86
2:B:215:TYR:CD2	2:B:226:LEU:HD12	2.10	0.86
2:B:534:ILE:O	2:B:598:LEU:HD11	1.76	0.86
2:B:546:CYS:HB2	2:B:607:ILE:HD11	1.55	0.86
2:B:566:ALA:CA	2:B:574:ASN:ND2	2.38	0.86
3:M:6:TYR:CD2	3:M:17:GLN:CA	2.47	0.86
3:M:323:MET:HE1	3:M:342:LEU:HB3	1.56	0.86
4:S:48:SER:CA	4:S:77:TYR:CB	2.51	0.86
1:A:220:SER:HB2	4:S:141:VAL:O	1.76	0.86
2:B:79:VAL:HG23	2:B:108:PHE:HZ	1.40	0.86
2:B:513:TRP:CD2	2:B:551:LEU:HD21	2.11	0.86
4:S:39:ILE:HD11	4:S:77:TYR:CG	2.09	0.86
1:A:132:LEU:HD13	1:A:165:ASP:CB	2.05	0.86
2:B:267:ASP:C	2:B:289:PRO:HG3	1.95	0.86

Continued on next page...

Continued from previous page...

Atom-1	Atom-2	Interatomic distance (Å)	Clash overlap (Å)
2:B:553:ALA:CB	2:B:614:ILE:HD13	2.02	0.86
2:B:553:ALA:HA	2:B:614:ILE:HG21	1.56	0.86
3:M:258:VAL:HG22	3:M:452:ILE:CG2	2.06	0.86
4:S:54:PRO:CG	4:S:57:LEU:CD1	2.46	0.86
1:A:103:LYS:N	4:S:163:THR:HB	1.91	0.85
2:B:223:LEU:CD1	2:B:258:GLN:C	2.38	0.85
3:M:18:TYR:CE1	3:M:122:SER:OG	2.29	0.85
3:M:253:ASN:OD1	3:M:292:PRO:HG2	1.75	0.85
3:M:290:PHE:CZ	3:M:297:PHE:CE1	2.64	0.85
3:M:405:THR:HG22	3:M:406:GLY:H	1.40	0.85
4:S:14:PRO:CA	4:S:36:TYR:OH	2.22	0.85
2:B:2:VAL:HG11	2:B:6:HIS:NE2	1.90	0.85
2:B:127:LEU:CG	2:B:157:THR:HG23	2.00	0.85
2:B:151:ALA:O	2:B:188:TYR:CD2	2.29	0.85
2:B:196:LEU:CB	2:B:229:HIS:ND1	2.39	0.85
2:B:280:PRO:O	2:B:283:TYR:HB2	1.77	0.85
3:M:2:TYR:C	3:M:81:SER:HB2	1.95	0.85
3:M:323:MET:SD	3:M:342:LEU:CG	2.64	0.85
4:S:135:ILE:HG23	4:S:141:VAL:HG13	1.56	0.85
3:M:2:TYR:N	3:M:81:SER:HB2	1.88	0.85
3:M:222:PHE:CG	3:M:439:TYR:HE1	1.94	0.85
3:M:360:LEU:HD23	3:M:362:PHE:CE2	2.08	0.85
1:A:147:LEU:CB	1:A:184:ALA:CB	2.54	0.85
1:A:332:TYR:CE1	1:A:366:SER:CB	2.58	0.85
2:B:56:SER:CB	2:B:92:THR:CG2	2.52	0.85
2:B:191:GLU:C	2:B:193:LEU:H	1.78	0.85
2:B:355:ASN:C	2:B:359:LEU:HD23	1.96	0.85
3:M:215:TYR:HD2	3:M:470:ALA:N	1.74	0.85
1:A:64:LEU:CA	1:A:102:GLN:NE2	2.39	0.85
1:A:240:LEU:O	1:A:241:TYR:C	2.00	0.85
1:A:252:ILE:CG1	4:S:145:ASN:N	2.36	0.85
1:A:516:ILE:HD13	1:A:551:LEU:CA	2.05	0.85
2:B:70:MET:CE	2:B:104:TYR:O	2.24	0.85
2:B:196:LEU:CB	2:B:229:HIS:CE1	2.58	0.85
2:B:316:THR:CG2	3:M:90:PHE:CZ	2.59	0.85
4:S:135:ILE:O	4:S:141:VAL:HG22	1.77	0.85
1:A:217:ALA:CB	4:S:142:ILE:HB	2.05	0.85
2:B:374:PHE:CZ	2:B:381:PHE:CD1	2.65	0.85
2:B:396:ILE:HG12	2:B:418:TYR:HE2	1.37	0.85
2:B:566:ALA:N	2:B:574:ASN:ND2	2.24	0.85
1:A:95:MET:C	1:A:127:LEU:HD21	1.94	0.85

Continued on next page...

Continued from previous page...

Atom-1	Atom-2	Interatomic distance (Å)	Clash overlap (Å)
1:A:381:GLU:HA	1:A:384:LEU:HD23	1.59	0.85
1:A:384:LEU:CD2	1:A:441:TYR:HE2	1.81	0.85
2:B:162:VAL:HB	2:B:195:ILE:HG23	1.57	0.85
2:B:177:ILE:HG21	2:B:196:LEU:HG	1.56	0.85
2:B:404:ASN:O	2:B:408:VAL:HG23	1.76	0.85
3:M:6:TYR:O	3:M:77:LEU:N	2.10	0.85
3:M:7:ILE:CA	3:M:75:TRP:O	2.24	0.85
4:S:28:GLN:O	4:S:32:LEU:HG	1.75	0.85
1:A:178:ARG:HH11	1:A:209:ASP:HB3	1.40	0.85
1:A:196:LEU:O	1:A:196:LEU:HD22	1.75	0.85
2:B:21:GLU:CA	2:B:24:ALA:HB3	2.07	0.85
2:B:352:ASN:HB2	3:M:49:ASP:CB	2.06	0.85
2:B:396:ILE:CG1	2:B:418:TYR:CE2	2.59	0.85
2:B:519:ALA:C	2:B:523:PHE:HB3	1.96	0.85
2:B:592:TYR:CD2	2:B:618:PHE:CE2	2.64	0.85
3:M:258:VAL:HA	3:M:452:ILE:HG13	1.56	0.85
1:A:225:LEU:CB	1:A:233:PHE:CZ	2.60	0.85
1:A:288:THR:CB	1:A:322:PHE:CZ	2.59	0.85
1:A:298:ILE:HD11	1:A:311:THR:CG2	2.07	0.85
2:B:20:ARG:NE	2:B:21:GLU:CB	2.39	0.85
3:M:121:ILE:HG22	3:M:125:PHE:CE1	2.12	0.85
3:M:215:TYR:CD1	3:M:467:TYR:O	2.27	0.85
3:M:226:PHE:CB	3:M:481:VAL:HG22	2.04	0.85
1:A:95:MET:C	1:A:127:LEU:CD2	2.45	0.85
1:A:608:ARG:NH2	1:A:632:PHE:HZ	1.74	0.85
1:A:633:PHE:HB2	2:B:550:VAL:HG13	1.56	0.85
2:B:143:SER:OG	2:B:175:LEU:HB3	1.75	0.85
2:B:230:PHE:HZ	2:B:252:LEU:CD2	1.58	0.85
2:B:562:ASN:CG	2:B:580:TYR:HB2	1.97	0.85
2:B:562:ASN:CG	2:B:580:TYR:CB	2.45	0.85
3:M:342:LEU:CD1	3:M:411:LEU:HD22	2.05	0.85
4:S:57:LEU:HB3	4:S:67:GLU:O	1.76	0.85
1:A:101:GLN:HG2	4:S:167:ILE:CG1	2.07	0.84
1:A:147:LEU:CD1	1:A:181:ALA:CA	2.53	0.84
1:A:482:ILE:HG12	1:A:517:TRP:CH2	2.12	0.84
1:A:557:LYS:CG	2:B:605:PHE:HD2	1.90	0.84
1:A:563:CYS:HB2	1:A:621:LEU:HD12	1.58	0.84
1:A:606:PHE:HZ	2:B:550:VAL:CG1	1.90	0.84
1:A:636:TYR:CB	2:B:554:LYS:HZ2	1.89	0.84
2:B:268:LYS:HA	2:B:276:SER:HB2	1.58	0.84
1:A:251:TRP:CD2	4:S:104:THR:OG1	2.30	0.84

Continued on next page...

Continued from previous page...

Atom-1	Atom-2	Interatomic distance (Å)	Clash overlap (Å)
1:A:636:TYR:CB	2:B:554:LYS:NZ	2.39	0.84
2:B:82:TYR:O	2:B:83:PHE:C	2.06	0.84
2:B:260:LEU:HA	2:B:291:TYR:CE1	2.11	0.84
1:A:88:ASN:CB	1:A:120:ILE:CD1	2.51	0.84
1:A:132:LEU:O	1:A:169:MET:HE3	1.77	0.84
1:A:638:LEU:CB	2:B:516:GLY:O	2.23	0.84
2:B:380:LYS:HZ3	3:M:236:LEU:HG	1.36	0.84
3:M:6:TYR:HA	3:M:16:PHE:O	1.77	0.84
1:A:260:PHE:CZ	1:A:274:LEU:CD1	2.59	0.84
1:A:266:VAL:O	1:A:267:GLU:CB	2.25	0.84
1:A:557:LYS:O	2:B:605:PHE:HE2	1.57	0.84
2:B:223:LEU:HD12	2:B:259:TYR:HA	0.87	0.84
2:B:549:LEU:CG	2:B:611:ALA:CB	2.54	0.84
3:M:224:VAL:HG23	3:M:479:PHE:CE1	2.13	0.84
3:M:290:PHE:CE2	3:M:297:PHE:CZ	2.64	0.84
3:M:347:PHE:CD2	3:M:350:VAL:HB	2.13	0.84
4:S:48:SER:CB	4:S:77:TYR:CA	2.56	0.84
1:A:103:LYS:HG3	4:S:163:THR:HG21	1.55	0.84
1:A:401:VAL:HG23	1:A:418:ILE:O	1.76	0.84
1:A:408:ILE:CG2	4:S:64:ASN:HB2	1.82	0.84
2:B:162:VAL:O	2:B:164:ASP:N	2.10	0.84
2:B:245:GLN:OE1	2:B:309:LEU:CD1	2.24	0.84
2:B:418:TYR:C	2:B:418:TYR:HD1	1.77	0.84
2:B:569:THR:C	2:B:571:SER:H	1.76	0.84
1:A:96:SER:HB3	1:A:127:LEU:CD2	2.07	0.84
1:A:504:ILE:O	1:A:505:ASN:C	2.06	0.84
1:A:589:SER:HB2	1:A:601:VAL:CG2	2.08	0.84
2:B:42:ILE:CG1	2:B:65:ARG:HD3	2.06	0.84
3:M:323:MET:HB3	3:M:340:LEU:CD1	2.06	0.84
4:S:83:LEU:CD1	4:S:116:VAL:HG21	2.07	0.84
1:A:103:LYS:HB2	4:S:163:THR:HG21	1.57	0.84
1:A:125:THR:OG1	1:A:158:LEU:CD1	2.23	0.84
1:A:259:LEU:O	1:A:262:ASN:N	2.10	0.84
1:A:533:ILE:HG12	1:A:562:TRP:HH2	1.41	0.84
1:A:595:GLU:CB	2:B:469:ASP:HB3	2.07	0.84
1:A:605:GLU:OE2	1:A:632:PHE:CE2	2.31	0.84
2:B:418:TYR:O	2:B:419:VAL:O	1.93	0.84
3:M:19:LEU:CD1	3:M:24:ALA:HB3	2.07	0.84
3:M:82:LYS:O	3:M:84:LYS:HG2	1.78	0.84
3:M:212:ASN:CG	3:M:250:LEU:HD23	1.97	0.84
1:A:103:LYS:CD	1:A:131:ASP:CG	2.46	0.84

Continued on next page...

Continued from previous page...

Atom-1	Atom-2	Interatomic distance (Å)	Clash overlap (Å)
1:A:629:LEU:O	1:A:630:PRO:C	2.00	0.84
2:B:490:ILE:O	2:B:515:PHE:CZ	2.30	0.84
3:M:254:PRO:CB	3:M:454:ILE:HD12	2.06	0.84
4:S:83:LEU:CD1	4:S:116:VAL:HG11	2.07	0.84
1:A:64:LEU:HA	1:A:102:GLN:HE22	1.40	0.84
1:A:179:LYS:HG3	1:A:217:ALA:HB2	1.58	0.84
2:B:48:VAL:CG2	2:B:82:TYR:CE2	2.60	0.84
3:M:233:LEU:HD22	3:M:324:SER:HA	1.59	0.84
1:A:225:LEU:HD12	1:A:233:PHE:CZ	2.13	0.84
2:B:182:ARG:CD	2:B:217:GLU:OE1	2.25	0.84
2:B:353:GLN:HE21	3:M:47:SER:HB3	1.04	0.84
2:B:396:ILE:HD13	2:B:432:ALA:CB	2.08	0.84
3:M:293:PRO:O	3:M:293:PRO:CD	2.26	0.84
4:S:15:ARG:HD2	4:S:122:ILE:HG12	1.58	0.84
1:A:67:LYS:HB2	4:S:165:SER:C	1.97	0.83
1:A:101:GLN:HG2	4:S:167:ILE:HG13	1.59	0.83
1:A:253:ILE:HD13	1:A:281:LEU:CB	2.07	0.83
2:B:264:THR:O	2:B:266:VAL:HG23	1.78	0.83
2:B:534:ILE:CD1	2:B:594:ALA:HB3	1.99	0.83
3:M:215:TYR:CG	3:M:469:GLY:N	2.25	0.83
3:M:223:HIS:CD2	3:M:478:ASN:CB	2.61	0.83
4:S:5:VAL:HG21	4:S:132:LEU:CG	2.08	0.83
1:A:66:SER:N	4:S:165:SER:HB2	1.93	0.83
2:B:70:MET:HE1	2:B:107:ARG:CG	2.07	0.83
2:B:274:PRO:CA	2:B:295:ASN:HD21	1.90	0.83
2:B:316:THR:OG1	3:M:90:PHE:CZ	2.19	0.83
2:B:336:ASN:O	2:B:337:THR:CB	2.20	0.83
2:B:344:VAL:HG22	2:B:363:ILE:CD1	2.06	0.83
3:M:101:LEU:O	3:M:106:LYS:CA	2.26	0.83
1:A:101:GLN:HE21	4:S:167:ILE:HG21	1.40	0.83
1:A:103:LYS:N	4:S:163:THR:CG2	2.40	0.83
1:A:211:ASP:CG	4:S:148:ARG:CD	2.31	0.83
1:A:320:HIS:O	1:A:321:THR:C	2.14	0.83
1:A:581:LEU:HD23	1:A:607:LEU:CD1	2.06	0.83
1:A:586:GLU:HB2	1:A:604:LEU:CD1	2.07	0.83
1:A:630:PRO:HG2	2:B:614:ILE:CB	2.05	0.83
1:A:634:ASN:O	2:B:517:GLU:CA	2.27	0.83
2:B:25:VAL:CG2	2:B:35:TYR:CD2	2.49	0.83
2:B:102:HIS:ND1	2:B:137:PHE:HB3	1.90	0.83
2:B:275:ARG:CB	2:B:291:TYR:CD2	2.61	0.83
2:B:512:VAL:HG21	2:B:548:ILE:HG12	1.58	0.83

Continued on next page...

Continued from previous page...

Atom-1	Atom-2	Interatomic distance (Å)	Clash overlap (Å)
2:B:531:ARG:CB	2:B:591:MET:SD	2.66	0.83
2:B:549:LEU:HD13	2:B:611:ALA:HB1	1.56	0.83
3:M:403:THR:CG2	3:M:407:THR:OG1	2.26	0.83
1:A:253:ILE:O	1:A:257:LEU:HG	1.78	0.83
1:A:384:LEU:HD13	1:A:441:TYR:HE2	1.44	0.83
1:A:585:PHE:CE2	1:A:607:LEU:HD11	2.14	0.83
2:B:177:ILE:HD12	2:B:196:LEU:HD23	1.59	0.83
2:B:232:ARG:HG3	2:B:236:ILE:CG1	2.06	0.83
2:B:513:TRP:CA	2:B:551:LEU:HD11	2.07	0.83
4:S:61:ASN:OD1	4:S:66:ASP:OD2	1.95	0.83
1:A:255:ARG:HH21	4:S:135:ILE:HG23	1.43	0.83
1:A:331:ARG:NH1	1:A:362:ASP:OD2	2.12	0.83
2:B:159:LYS:CA	2:B:195:ILE:CD1	2.50	0.83
4:S:53:THR:CA	4:S:69:ASN:CB	2.53	0.83
1:A:140:VAL:HA	1:A:177:ILE:CD1	2.08	0.83
1:A:306:GLU:O	1:A:307:ASP:C	2.12	0.83
1:A:332:TYR:HE1	1:A:366:SER:CB	1.92	0.83
2:B:173:VAL:HB	2:B:199:LEU:CD1	2.09	0.83
2:B:177:ILE:O	2:B:181:TYR:HB2	1.78	0.83
2:B:292:GLU:HG2	2:B:296:ASP:CB	2.09	0.83
3:M:121:ILE:O	3:M:125:PHE:CD1	2.31	0.83
3:M:261:ASN:O	3:M:446:GLY:O	1.97	0.83
4:S:5:VAL:CG1	4:S:132:LEU:CD2	2.49	0.83
1:A:403:LEU:HD11	1:A:421:PRO:O	1.79	0.83
1:A:429:VAL:HG21	1:A:469:LEU:HD21	1.60	0.83
2:B:178:ILE:CG2	2:B:214:ALA:O	2.26	0.83
3:M:220:GLU:CG	3:M:439:TYR:HB2	2.07	0.83
3:M:262:THR:HG22	3:M:264:GLY:CA	2.09	0.83
1:A:95:MET:SD	1:A:107:TYR:HD2	2.00	0.83
1:A:163:ALA:O	1:A:164:ASP:C	2.08	0.83
3:M:218:LEU:HG	3:M:472:TYR:HE2	1.43	0.83
3:M:224:VAL:O	3:M:479:PHE:HB3	1.79	0.83
2:B:20:ARG:HH11	2:B:21:GLU:CG	1.91	0.83
2:B:155:LEU:CB	2:B:188:TYR:HD2	1.91	0.83
2:B:243:TRP:HZ2	3:M:94:GLU:C	1.82	0.83
1:A:182:ILE:CD1	1:A:218:ALA:HB2	2.07	0.83
1:A:275:LEU:HD21	1:A:311:THR:HG1	1.44	0.83
1:A:403:LEU:CD2	1:A:422:GLU:CG	2.52	0.83
1:A:637:GLU:HG3	2:B:515:PHE:N	1.68	0.83
4:S:9:ASN:ND2	4:S:13:GLN:HB2	1.93	0.83
4:S:53:THR:O	4:S:69:ASN:CG	2.17	0.83

Continued on next page...

Continued from previous page...

Atom-1	Atom-2	Interatomic distance (Å)	Clash overlap (Å)
1:A:559:PHE:HE1	1:A:581:LEU:HD22	1.43	0.82
1:A:633:PHE:CD2	2:B:550:VAL:O	2.31	0.82
2:B:50:LEU:HG	2:B:58:GLU:O	1.79	0.82
2:B:231:ARG:HH21	2:B:297:PRO:HD2	1.44	0.82
2:B:281:ASP:OD1	2:B:287:GLU:OE2	1.97	0.82
2:B:566:ALA:C	2:B:574:ASN:CG	2.38	0.82
3:M:219:LEU:HB3	3:M:472:TYR:O	1.79	0.82
1:A:65:ASN:O	4:S:165:SER:HA	1.79	0.82
1:A:140:VAL:N	1:A:177:ILE:CD1	2.41	0.82
1:A:151:SER:HB2	1:A:187:LYS:HB3	1.59	0.82
1:A:348:PHE:O	1:A:352:PHE:CD1	2.32	0.82
1:A:566:PHE:CE1	1:A:618:THR:HB	2.14	0.82
2:B:393:ILE:HG12	2:B:428:VAL:HA	1.62	0.82
1:A:103:LYS:CD	1:A:131:ASP:OD1	2.28	0.82
1:A:163:ALA:HB1	1:A:199:ASN:ND2	1.92	0.82
1:A:621:LEU:O	1:A:622:PRO:C	1.97	0.82
1:A:634:ASN:O	2:B:516:GLY:O	1.95	0.82
2:B:42:ILE:HG12	2:B:65:ARG:HD3	1.60	0.82
2:B:171:GLY:HA2	2:B:207:VAL:HG12	1.61	0.82
2:B:505:ASP:HA	2:B:544:THR:OG1	1.79	0.82
2:B:522:GLU:O	2:B:522:GLU:HG2	1.79	0.82
3:M:262:THR:HG22	3:M:264:GLY:H	1.43	0.82
4:S:47:GLN:HE22	4:S:78:LYS:HA	1.45	0.82
1:A:260:PHE:CE2	1:A:274:LEU:HG	2.14	0.82
1:A:564:ASN:OD1	1:A:622:PRO:HG3	1.78	0.82
2:B:127:LEU:HB3	2:B:161:LEU:HD13	1.61	0.82
2:B:158:VAL:CG1	2:B:195:ILE:HG21	2.08	0.82
2:B:197:LYS:HD2	2:B:283:TYR:CE2	2.14	0.82
2:B:310:ILE:HA	2:B:318:ILE:HG12	1.61	0.82
2:B:527:PRO:HB2	2:B:587:ARG:CD	2.09	0.82
2:B:562:ASN:CB	2:B:580:TYR:HB3	2.08	0.82
4:S:83:LEU:HD11	4:S:116:VAL:CG1	2.07	0.82
1:A:114:PHE:CG	1:A:153:ILE:HG23	2.13	0.82
1:A:140:VAL:CG1	1:A:176:TYR:HB2	2.10	0.82
1:A:144:GLY:HA2	1:A:180:LYS:HB2	1.61	0.82
2:B:70:MET:HE3	2:B:107:ARG:HG3	1.59	0.82
2:B:178:ILE:C	2:B:180:LEU:H	1.82	0.82
2:B:311:TYR:HE2	2:B:342:ALA:HB1	1.40	0.82
2:B:418:TYR:CD1	2:B:419:VAL:N	2.47	0.82
2:B:493:LEU:CG	2:B:511:ILE:HG23	2.09	0.82
2:B:542:PRO:O	2:B:602:ASP:OD2	1.95	0.82

Continued on next page...

Continued from previous page...

Atom-1	Atom-2	Interatomic distance (Å)	Clash overlap (Å)
2:B:592:TYR:CD2	2:B:618:PHE:CD2	2.67	0.82
3:M:231:SER:O	3:M:326:HIS:HE1	1.62	0.82
1:A:166:LEU:HD13	1:A:185:LEU:CG	2.09	0.82
1:A:432:ILE:HG21	1:A:457:LEU:HD11	1.59	0.82
2:B:42:ILE:O	2:B:46:GLN:OE1	1.96	0.82
2:B:309:LEU:HD12	2:B:317:VAL:HG11	1.62	0.82
2:B:344:VAL:HG13	2:B:381:PHE:HZ	1.42	0.82
2:B:396:ILE:CD1	2:B:418:TYR:CZ	2.62	0.82
2:B:512:VAL:HB	2:B:551:LEU:HD12	1.62	0.82
1:A:217:ALA:O	4:S:142:ILE:HG22	1.77	0.82
1:A:563:CYS:CB	1:A:621:LEU:CD1	2.55	0.82
1:A:563:CYS:SG	1:A:621:LEU:CD1	2.68	0.82
2:B:182:ARG:CZ	2:B:217:GLU:OE1	2.27	0.82
2:B:431:MET:O	2:B:434:LYS:N	2.12	0.82
3:M:3:LEU:HG	3:M:80:THR:O	1.79	0.82
3:M:101:LEU:CA	3:M:109:LEU:HD13	2.09	0.82
3:M:243:ILE:CG1	3:M:473:LYS:O	2.27	0.82
1:A:140:VAL:CG1	1:A:176:TYR:CB	2.52	0.82
1:A:255:ARG:HD3	4:S:141:VAL:O	1.80	0.82
1:A:258:LYS:HZ1	4:S:97:ALA:CB	1.92	0.82
1:A:258:LYS:NZ	4:S:97:ALA:CB	2.42	0.82
2:B:230:PHE:HB3	2:B:298:ASP:OD2	1.80	0.82
2:B:177:ILE:HD11	2:B:195:ILE:CG2	2.10	0.82
2:B:197:LYS:HD2	2:B:283:TYR:CD2	2.15	0.82
2:B:252:LEU:HB2	2:B:302:PHE:CE1	2.15	0.82
2:B:568:VAL:O	2:B:571:SER:CB	2.28	0.82
2:B:569:THR:C	2:B:571:SER:N	2.28	0.82
1:A:288:THR:CB	1:A:322:PHE:HZ	1.93	0.82
1:A:520:GLY:HA3	1:A:558:VAL:HG22	0.86	0.82
2:B:353:GLN:CB	3:M:50:TYR:N	2.39	0.82
2:B:360:LEU:HD12	2:B:391:ALA:HA	1.60	0.82
2:B:437:SER:HB2	2:B:474:VAL:HG13	1.60	0.82
3:M:290:PHE:CE1	3:M:297:PHE:CG	2.66	0.82
3:M:353:VAL:HG22	3:M:354:ASP:O	1.80	0.82
1:A:71:VAL:CG1	1:A:105:VAL:CG1	2.56	0.81
1:A:219:VAL:HG12	1:A:259:LEU:CD1	2.08	0.81
2:B:120:ILE:HG23	2:B:154:ILE:CG1	2.10	0.81
2:B:199:LEU:C	2:B:201:ALA:N	2.30	0.81
2:B:578:PRO:HB2	2:B:579:PRO:CD	2.07	0.81
3:M:241:HIS:O	3:M:474:THR:OG1	1.97	0.81
4:S:54:PRO:CB	4:S:57:LEU:HD11	2.10	0.81

Continued on next page...

Continued from previous page...

Atom-1	Atom-2	Interatomic distance (Å)	Clash overlap (Å)
1:A:129:LYS:HD2	1:A:161:ASP:CG	2.00	0.81
1:A:163:ALA:HB1	1:A:199:ASN:HD21	1.43	0.81
1:A:189:PHE:CD2	1:A:225:LEU:CD2	2.60	0.81
1:A:219:VAL:CG1	1:A:256:LEU:HD23	2.09	0.81
1:A:263:LEU:O	1:A:266:VAL:N	2.12	0.81
1:A:298:ILE:O	1:A:299:VAL:C	2.16	0.81
2:B:170:ARG:HG2	2:B:199:LEU:HD23	1.61	0.81
2:B:340:ILE:CD1	2:B:366:LEU:HB3	2.09	0.81
3:M:7:ILE:HA	3:M:75:TRP:O	1.80	0.81
3:M:101:LEU:CD2	3:M:106:LYS:O	2.28	0.81
3:M:344:ILE:HG23	3:M:347:PHE:HB3	0.93	0.81
1:A:147:LEU:CB	1:A:184:ALA:HB2	2.10	0.81
1:A:178:ARG:NH1	1:A:209:ASP:CG	2.33	0.81
1:A:566:PHE:HE1	1:A:618:THR:HB	1.43	0.81
2:B:90:ILE:CD1	2:B:123:LEU:CD2	2.58	0.81
2:B:116:THR:HG22	2:B:150:LEU:HD21	1.60	0.81
2:B:171:GLY:N	2:B:207:VAL:CG1	2.34	0.81
2:B:239:GLN:OE1	3:M:278:ILE:O	1.98	0.81
2:B:374:PHE:CD2	2:B:402:LEU:HD11	2.15	0.81
2:B:490:ILE:HG23	2:B:515:PHE:CE2	2.16	0.81
2:B:527:PRO:HG2	2:B:587:ARG:HG2	1.61	0.81
2:B:556:LEU:CB	2:B:588:ILE:HD11	2.10	0.81
3:M:245:ASP:CA	3:M:472:TYR:CE1	2.63	0.81
3:M:257:ALA:HB2	3:M:455:VAL:HG21	1.60	0.81
3:M:374:TYR:HA	3:M:417:TYR:HA	1.63	0.81
1:A:298:ILE:CD1	1:A:311:THR:HG21	2.10	0.81
3:M:339:GLU:OE2	3:M:412:ARG:NE	2.07	0.81
1:A:96:SER:HB3	1:A:127:LEU:HD13	1.60	0.81
1:A:103:LYS:CE	1:A:131:ASP:CG	2.49	0.81
1:A:104:ARG:HE	4:S:160:ALA:HB2	1.45	0.81
1:A:408:ILE:HG21	4:S:64:ASN:O	1.79	0.81
2:B:120:ILE:HG23	2:B:142:LEU:HD22	1.63	0.81
2:B:182:ARG:HD2	2:B:217:GLU:CB	2.10	0.81
2:B:182:ARG:CG	2:B:217:GLU:HB3	2.10	0.81
2:B:553:ALA:CA	2:B:614:ILE:HG21	2.11	0.81
3:M:458:LEU:HD22	3:M:462:LYS:HG2	1.62	0.81
1:A:211:ASP:OD1	1:A:213:SER:N	2.14	0.81
1:A:634:ASN:HD22	2:B:554:LYS:HB3	1.46	0.81
2:B:109:ALA:O	2:B:110:GLU:C	2.06	0.81
2:B:123:LEU:CB	2:B:142:LEU:HD11	2.09	0.81
2:B:158:VAL:HG13	2:B:177:ILE:HD11	1.61	0.81

Continued on next page...

Continued from previous page...

Atom-1	Atom-2	Interatomic distance (Å)	Clash overlap (Å)
2:B:223:LEU:O	2:B:224:GLU:C	2.11	0.81
2:B:277:CYS:HA	2:B:292:GLU:HG3	1.61	0.81
2:B:303:LEU:CD1	2:B:333:GLN:CG	2.55	0.81
3:M:245:ASP:HB3	3:M:472:TYR:CD1	2.16	0.81
4:S:48:SER:C	4:S:77:TYR:HB2	2.00	0.81
1:A:71:VAL:HG11	1:A:105:VAL:CG1	2.11	0.81
1:A:260:PHE:CE1	1:A:274:LEU:HD12	2.15	0.81
1:A:567:GLN:O	1:A:568:GLU:C	2.17	0.81
1:A:586:GLU:N	1:A:604:LEU:HD12	1.94	0.81
2:B:90:ILE:HD13	2:B:123:LEU:CD2	2.10	0.81
3:M:49:ASP:HA	3:M:75:TRP:HH2	1.40	0.81
3:M:101:LEU:HG	3:M:106:LYS:HA	0.81	0.81
1:A:96:SER:H	1:A:127:LEU:HD21	0.98	0.81
1:A:102:GLN:NE2	4:S:165:SER:H	1.79	0.81
1:A:225:LEU:HD12	1:A:233:PHE:CE2	2.16	0.81
1:A:346:THR:O	1:A:347:ASP:C	2.10	0.81
1:A:532:LEU:O	1:A:533:ILE:C	2.00	0.81
2:B:151:ALA:CA	2:B:180:LEU:CD1	2.44	0.81
2:B:396:ILE:CD1	2:B:418:TYR:OH	2.29	0.81
2:B:563:PHE:C	2:B:566:ALA:HB3	2.02	0.81
4:S:9:ASN:ND2	4:S:13:GLN:CG	2.44	0.81
1:A:99:LYS:CE	4:S:164:ASP:HB2	2.11	0.81
1:A:140:VAL:HA	1:A:177:ILE:HG12	0.81	0.81
1:A:140:VAL:CA	1:A:177:ILE:CD1	2.59	0.81
1:A:163:ALA:CB	1:A:199:ASN:HD21	1.94	0.81
1:A:237:SER:HB2	1:A:270:LEU:CD1	2.11	0.81
2:B:47:LEU:HD22	2:B:62:ALA:O	1.80	0.81
2:B:214:ALA:O	2:B:217:GLU:N	2.13	0.81
2:B:219:TYR:O	2:B:220:ALA:C	2.16	0.81
2:B:353:GLN:CB	3:M:49:ASP:H	1.78	0.81
2:B:433:VAL:CG1	2:B:474:VAL:CB	2.51	0.81
2:B:477:MET:O	2:B:480:GLN:HB2	1.80	0.81
2:B:527:PRO:CG	2:B:587:ARG:CG	2.51	0.81
3:M:69:ILE:CD1	3:M:90:PHE:CE1	2.64	0.81
3:M:101:LEU:HD23	3:M:106:LYS:HG3	1.62	0.81
3:M:212:ASN:HB3	3:M:250:LEU:HA	1.63	0.81
1:A:103:LYS:HD3	1:A:131:ASP:OD2	1.81	0.81
1:A:140:VAL:CG1	1:A:176:TYR:HB3	2.09	0.81
1:A:182:ILE:O	1:A:221:VAL:HG21	1.81	0.81
1:A:202:LYS:O	1:A:205:SER:N	2.13	0.81
1:A:631:SER:HB2	2:B:557:SER:CB	1.92	0.81

Continued on next page...

Continued from previous page...

Atom-1	Atom-2	Interatomic distance (Å)	Clash overlap (Å)
2:B:77:ILE:O	2:B:78:ASP:C	2.17	0.81
2:B:87:VAL:HG12	2:B:122:SER:CB	1.77	0.81
2:B:343:LEU:HD22	2:B:362:ALA:HB1	1.62	0.81
2:B:545:ARG:HH11	2:B:602:ASP:HA	1.45	0.81
3:M:224:VAL:HG11	3:M:226:PHE:CZ	2.16	0.81
3:M:310:VAL:HG13	3:M:315:VAL:O	1.81	0.81
3:M:342:LEU:CD1	3:M:411:LEU:CD2	2.56	0.81
1:A:68:THR:CA	4:S:167:ILE:N	2.44	0.80
1:A:105:VAL:CA	4:S:167:ILE:CD1	2.59	0.80
1:A:215:VAL:HG22	1:A:243:ILE:CG1	2.10	0.80
1:A:463:ASP:C	2:B:1:MET:SD	2.59	0.80
1:A:637:GLU:HB3	2:B:516:GLY:N	1.96	0.80
2:B:136:CYS:O	2:B:172:GLU:HB3	1.80	0.80
2:B:260:LEU:CD2	2:B:291:TYR:CZ	2.53	0.80
2:B:380:LYS:HZ3	3:M:236:LEU:CG	1.94	0.80
2:B:472:VAL:HG11	2:B:510:GLY:C	2.01	0.80
2:B:560:ILE:HA	2:B:564:LYS:H	1.46	0.80
3:M:7:ILE:CG2	3:M:75:TRP:O	2.29	0.80
1:A:488:ARG:O	1:A:491:THR:OG1	1.99	0.80
2:B:296:ASP:OD1	2:B:297:PRO:CD	2.29	0.80
2:B:337:THR:CA	2:B:373:LEU:CD2	2.40	0.80
2:B:425:PRO:HD2	2:B:428:VAL:HG21	1.61	0.80
2:B:508:ARG:HB3	2:B:544:THR:CG2	2.12	0.80
3:M:223:HIS:HA	3:M:479:PHE:CG	2.13	0.80
3:M:340:LEU:HG	3:M:411:LEU:CB	2.09	0.80
4:S:108:SER:CB	4:S:149:ILE:HG21	2.10	0.80
1:A:244:LEU:CD1	1:A:256:LEU:HD13	2.11	0.80
1:A:264:SER:CB	1:A:271:ARG:CD	2.59	0.80
1:A:408:ILE:HD12	1:A:410:TYR:CD1	2.15	0.80
1:A:609:LEU:CG	1:A:628:VAL:CB	2.48	0.80
1:A:637:GLU:CB	2:B:516:GLY:N	2.44	0.80
2:B:90:ILE:CD1	2:B:123:LEU:HD23	2.11	0.80
2:B:135:ARG:NH1	2:B:164:ASP:HB2	1.97	0.80
2:B:177:ILE:HG21	2:B:196:LEU:CG	2.10	0.80
2:B:178:ILE:HG22	2:B:179:LYS:H	1.44	0.80
2:B:252:LEU:CB	2:B:302:PHE:CE2	2.65	0.80
2:B:256:CYS:HA	2:B:293:VAL:CG2	2.05	0.80
2:B:563:PHE:O	2:B:566:ALA:HB3	1.80	0.80
3:M:215:TYR:CD2	3:M:469:GLY:CA	2.65	0.80
4:S:7:ILE:CG2	4:S:121:LEU:HD21	2.09	0.80
1:A:64:LEU:O	1:A:65:ASN:C	2.19	0.80

Continued on next page...

Continued from previous page...

Atom-1	Atom-2	Interatomic distance (Å)	Clash overlap (Å)
2:B:433:VAL:HG11	2:B:471:TYR:HA	1.61	0.80
1:A:96:SER:CB	1:A:127:LEU:CD2	2.60	0.80
1:A:222:ILE:HG23	1:A:233:PHE:HB3	1.64	0.80
2:B:315:PRO:CG	2:B:352:ASN:OD1	2.28	0.80
2:B:546:CYS:HA	2:B:607:ILE:HG23	0.82	0.80
3:M:71:LYS:O	3:M:74:TYR:CE2	2.34	0.80
1:A:101:GLN:NE2	4:S:167:ILE:HG21	1.93	0.80
1:A:207:LEU:HD11	1:A:240:LEU:HD23	1.62	0.80
1:A:253:ILE:HD11	1:A:281:LEU:CD2	2.09	0.80
1:A:279:LEU:O	1:A:280:GLU:C	2.11	0.80
1:A:465:SER:H	2:B:1:MET:HG2	1.47	0.80
1:A:557:LYS:HB3	2:B:605:PHE:CE2	2.15	0.80
1:A:608:ARG:NH2	1:A:632:PHE:CZ	2.49	0.80
2:B:230:PHE:CD2	2:B:298:ASP:CB	2.63	0.80
2:B:486:HIS:NE2	2:B:518:ILE:HB	1.95	0.80
2:B:560:ILE:CG2	2:B:564:LYS:HB2	2.11	0.80
3:M:18:TYR:CD1	3:M:122:SER:CB	2.64	0.80
1:A:170:LEU:O	1:A:206:LYS:CE	2.28	0.80
1:A:213:SER:C	4:S:143:GLU:HG2	2.01	0.80
1:A:384:LEU:CD1	1:A:441:TYR:HE2	1.94	0.80
1:A:399:ASP:HA	1:A:420:ILE:HB	0.87	0.80
1:A:545:HIS:O	1:A:546:SER:C	2.07	0.80
1:A:582:ILE:HD11	1:A:608:ARG:HA	1.64	0.80
2:B:230:PHE:O	2:B:231:ARG:C	1.96	0.80
2:B:266:VAL:HG13	2:B:291:TYR:H	1.46	0.80
2:B:344:VAL:CG2	2:B:363:ILE:HD11	2.12	0.80
2:B:371:GLN:CD	2:B:401:THR:O	2.20	0.80
2:B:525:ILE:C	2:B:527:PRO:CD	2.50	0.80
4:S:14:PRO:HB3	4:S:36:TYR:HE1	1.47	0.80
4:S:108:SER:HB3	4:S:149:ILE:CG2	2.12	0.80
1:A:224:GLU:CG	4:S:138:GLY:O	2.30	0.80
1:A:392:MET:SD	1:A:457:LEU:HD23	2.22	0.80
1:A:537:THR:CB	1:A:584:PHE:CE1	2.64	0.80
2:B:200:MET:HE3	2:B:232:ARG:H	1.47	0.80
3:M:66:PHE:HB3	3:M:77:LEU:CD2	2.10	0.80
4:S:87:PHE:CG	4:S:102:ILE:HG12	2.15	0.80
4:S:109:LEU:O	4:S:110:ASP:C	2.13	0.80
1:A:426:ILE:CG1	1:A:464:ILE:HD13	2.12	0.80
1:A:436:CYS:SG	1:A:450:TYR:CZ	2.75	0.80
2:B:527:PRO:HB2	2:B:587:ARG:HD2	1.62	0.80
2:B:550:VAL:CG2	2:B:610:ARG:CD	2.60	0.80

Continued on next page...

Continued from previous page...

Atom-1	Atom-2	Interatomic distance (Å)	Clash overlap (Å)
3:M:71:LYS:O	3:M:72:LEU:CB	2.30	0.80
1:A:151:SER:CB	1:A:187:LYS:HB2	2.12	0.80
2:B:62:ALA:HB1	2:B:66:ILE:HD11	1.64	0.80
2:B:337:THR:HG23	2:B:373:LEU:CD1	2.12	0.80
3:M:245:ASP:O	3:M:472:TYR:CZ	2.34	0.80
1:A:178:ARG:NH1	1:A:209:ASP:CB	2.44	0.79
1:A:291:ILE:HG21	1:A:322:PHE:HE1	1.44	0.79
2:B:47:LEU:CD2	2:B:62:ALA:O	2.30	0.79
2:B:219:TYR:HE1	2:B:226:LEU:HD12	1.47	0.79
2:B:352:ASN:O	2:B:355:ASN:HB2	1.81	0.79
2:B:519:ALA:O	2:B:523:PHE:HD2	1.65	0.79
3:M:223:HIS:CB	3:M:478:ASN:HA	2.12	0.79
1:A:125:THR:CB	1:A:158:LEU:HD13	2.12	0.79
1:A:145:ILE:HG12	4:S:156:LEU:HD22	1.62	0.79
2:B:170:ARG:NH1	2:B:198:GLU:O	2.15	0.79
2:B:189:HIS:CD2	2:B:222:HIS:HB3	2.17	0.79
2:B:341:GLU:HG2	2:B:345:ARG:HE	1.46	0.79
2:B:389:ILE:O	2:B:390:VAL:C	2.17	0.79
2:B:458:MET:O	2:B:459:GLU:C	2.19	0.79
1:A:185:LEU:HD22	1:A:189:PHE:CZ	2.16	0.79
2:B:50:LEU:CB	2:B:62:ALA:HB2	2.13	0.79
2:B:63:MET:CG	2:B:100:LEU:HB3	2.12	0.79
2:B:178:ILE:HG21	2:B:214:ALA:CA	2.12	0.79
2:B:217:GLU:O	2:B:219:TYR:N	2.15	0.79
2:B:245:GLN:NE2	2:B:309:LEU:CD1	2.45	0.79
2:B:374:PHE:CE2	2:B:398:ILE:CG2	2.66	0.79
3:M:379:LEU:HD23	3:M:397:TRP:CD1	2.17	0.79
1:A:96:SER:CB	1:A:127:LEU:HD21	2.11	0.79
1:A:137:ASN:C	1:A:139:ASP:N	2.28	0.79
1:A:182:ILE:HD13	1:A:218:ALA:CB	2.12	0.79
1:A:291:ILE:HG21	1:A:322:PHE:CE1	2.17	0.79
2:B:63:MET:SD	2:B:101:ILE:HA	2.22	0.79
2:B:139:LEU:CD2	2:B:173:VAL:C	2.50	0.79
2:B:162:VAL:HG23	2:B:199:LEU:HD11	1.61	0.79
2:B:174:ALA:HB3	2:B:211:ALA:CA	2.09	0.79
2:B:178:ILE:O	2:B:181:TYR:N	2.16	0.79
3:M:65:TYR:CD1	3:M:86:PRO:CB	2.65	0.79
3:M:69:ILE:CG1	3:M:90:PHE:CE1	2.66	0.79
1:A:102:GLN:N	4:S:167:ILE:HG12	1.97	0.79
1:A:170:LEU:C	1:A:206:LYS:HD2	2.02	0.79
1:A:461:CYS:O	1:A:462:GLN:C	2.08	0.79

Continued on next page...

Continued from previous page...

Atom-1	Atom-2	Interatomic distance (Å)	Clash overlap (Å)
2:B:116:THR:HG22	2:B:150:LEU:CD2	2.12	0.79
2:B:353:GLN:HG2	3:M:48:ASP:N	1.97	0.79
2:B:490:ILE:CG2	2:B:515:PHE:CD2	2.66	0.79
3:M:65:TYR:CD1	3:M:86:PRO:CG	2.65	0.79
1:A:217:ALA:CB	4:S:142:ILE:CB	2.52	0.79
1:A:260:PHE:CE1	1:A:274:LEU:CD1	2.65	0.79
1:A:605:GLU:HG2	1:A:632:PHE:CG	2.17	0.79
1:A:636:TYR:H	2:B:554:LYS:HD3	1.47	0.79
2:B:42:ILE:HD13	2:B:65:ARG:HD3	1.61	0.79
1:A:137:ASN:C	1:A:139:ASP:H	1.80	0.79
1:A:220:SER:HB2	4:S:141:VAL:C	2.02	0.79
1:A:326:GLN:CA	1:A:331:ARG:NH2	2.42	0.79
1:A:393:LYS:NZ	1:A:397:ASP:OD2	2.16	0.79
2:B:42:ILE:O	2:B:43:ASN:HB2	1.83	0.79
2:B:216:LYS:HG3	2:B:251:LEU:HD12	1.63	0.79
2:B:236:ILE:HG22	2:B:240:LEU:CD1	2.12	0.79
2:B:367:SER:OG	2:B:401:THR:CG2	2.30	0.79
2:B:556:LEU:HB3	2:B:618:PHE:CD1	2.18	0.79
1:A:142:LYS:CA	4:S:159:ALA:CB	2.61	0.79
1:A:143:VAL:HG11	1:A:169:MET:HB3	1.65	0.79
1:A:189:PHE:CD2	1:A:225:LEU:HD11	2.17	0.79
1:A:244:LEU:CD1	1:A:256:LEU:CD1	2.59	0.79
1:A:254:ILE:CG2	1:A:293:GLU:HG2	2.10	0.79
2:B:136:CYS:C	2:B:172:GLU:CG	2.50	0.79
2:B:170:ARG:CA	2:B:199:LEU:CD2	2.48	0.79
3:M:262:THR:C	3:M:264:GLY:H	1.86	0.79
3:M:374:TYR:CB	3:M:417:TYR:HD2	1.95	0.79
1:A:557:LYS:HD3	2:B:605:PHE:CB	2.10	0.79
1:A:628:VAL:O	1:A:631:SER:OG	2.00	0.79
2:B:137:PHE:N	2:B:172:GLU:HG3	1.98	0.79
4:S:53:THR:HB	4:S:69:ASN:CB	2.13	0.79
1:A:371:ALA:O	1:A:374:LEU:N	2.16	0.79
1:A:461:CYS:SG	1:A:469:LEU:HB3	2.23	0.79
2:B:123:LEU:O	2:B:127:LEU:CD1	2.30	0.79
2:B:151:ALA:HB3	2:B:184:GLY:HA3	1.64	0.79
2:B:155:LEU:O	2:B:157:THR:N	2.16	0.79
2:B:309:LEU:CB	2:B:317:VAL:HG11	2.06	0.79
2:B:567:GLN:HG3	2:B:585:GLY:HA3	1.62	0.79
3:M:356:LEU:CD2	3:M:358:ILE:HG13	2.13	0.79
3:M:374:TYR:O	3:M:390:ILE:CD1	2.31	0.79
4:S:3:HIS:O	4:S:19:PHE:HA	1.82	0.79

Continued on next page...

Continued from previous page...

Atom-1	Atom-2	Interatomic distance (Å)	Clash overlap (Å)
4:S:6:LEU:HD13	4:S:32:LEU:HD13	1.65	0.79
1:A:145:ILE:CG1	4:S:156:LEU:HD22	2.13	0.78
2:B:25:VAL:CB	2:B:36:THR:OG1	2.31	0.78
2:B:37:TYR:O	2:B:40:GLN:N	2.16	0.78
2:B:165:PRO:HA	2:B:170:ARG:NH2	1.96	0.78
2:B:178:ILE:HB	2:B:214:ALA:HB1	1.65	0.78
4:S:9:ASN:HD21	4:S:13:GLN:HG3	1.46	0.78
1:A:74:LEU:CD2	1:A:87:CYS:SG	2.70	0.78
1:A:102:GLN:CG	4:S:166:LYS:N	1.94	0.78
1:A:440:ASN:OD1	1:A:442:SER:HB3	1.83	0.78
2:B:123:LEU:HB2	2:B:142:LEU:HD11	1.66	0.78
2:B:151:ALA:CB	2:B:184:GLY:HA3	2.13	0.78
2:B:219:TYR:CE1	2:B:226:LEU:HA	2.06	0.78
2:B:219:TYR:HB2	2:B:255:TYR:HD1	1.45	0.78
2:B:267:ASP:O	2:B:276:SER:CB	2.31	0.78
2:B:311:TYR:CZ	2:B:342:ALA:HB2	2.19	0.78
2:B:339:PHE:O	2:B:343:LEU:HB2	1.81	0.78
2:B:531:ARG:HB2	2:B:591:MET:SD	2.23	0.78
3:M:9:ASP:C	3:M:75:TRP:HD1	1.85	0.78
3:M:16:PHE:HA	3:M:118:TYR:CD1	2.18	0.78
3:M:320:ILE:HG21	3:M:439:TYR:CE2	2.18	0.78
3:M:342:LEU:HD12	3:M:411:LEU:CB	2.10	0.78
1:A:80:TYR:CB	1:A:82:PHE:CD2	2.65	0.78
1:A:519:LEU:O	1:A:520:GLY:C	2.13	0.78
2:B:120:ILE:CD1	2:B:142:LEU:HD23	2.13	0.78
2:B:267:ASP:CA	2:B:289:PRO:HG3	2.14	0.78
2:B:537:PHE:CE2	2:B:598:LEU:HB3	2.13	0.78
3:M:101:LEU:CB	3:M:106:LYS:HA	2.14	0.78
3:M:104:PHE:HZ	3:M:113:LYS:CE	1.54	0.78
1:A:67:LYS:CB	1:A:94:VAL:HG22	2.12	0.78
1:A:114:PHE:CD1	1:A:153:ILE:HG23	2.18	0.78
2:B:83:PHE:CE2	2:B:119:SER:CA	2.47	0.78
2:B:148:SER:O	2:B:183:ALA:HB3	1.70	0.78
2:B:501:THR:HA	2:B:508:ARG:HH22	1.47	0.78
3:M:95:THR:O	3:M:99:ILE:HD13	1.82	0.78
3:M:233:LEU:HD22	3:M:323:MET:O	1.82	0.78
3:M:338:PHE:HD2	3:M:415:ILE:HG13	1.48	0.78
3:M:362:PHE:O	3:M:363:ASN:O	2.00	0.78
3:M:449:VAL:HG12	3:M:452:ILE:CD1	1.96	0.78
4:S:8:PHE:HZ	4:S:84:TYR:HB3	1.45	0.78
1:A:241:TYR:C	1:A:242:GLU:O	2.02	0.78

Continued on next page...

Continued from previous page...

Atom-1	Atom-2	Interatomic distance (Å)	Clash overlap (Å)
1:A:629:LEU:HD21	2:B:610:ARG:HD3	1.65	0.78
1:A:637:GLU:CG	2:B:515:PHE:N	2.36	0.78
3:M:67:SER:OG	3:M:90:PHE:HB2	1.84	0.78
1:A:147:LEU:HD12	1:A:181:ALA:CA	2.10	0.78
1:A:384:LEU:HD13	1:A:441:TYR:CE2	2.19	0.78
2:B:291:TYR:CD2	2:B:294:VAL:CB	2.66	0.78
2:B:350:THR:HB	2:B:352:ASN:HD21	1.45	0.78
2:B:515:PHE:CE2	2:B:529:VAL:HG11	2.19	0.78
3:M:244:VAL:CB	3:M:472:TYR:CE2	2.66	0.78
3:M:354:ASP:HB2	3:M:440:ILE:HD12	1.63	0.78
1:A:141:VAL:CG1	4:S:159:ALA:N	2.47	0.78
2:B:20:ARG:NH1	2:B:35:TYR:CE1	2.51	0.78
2:B:396:ILE:CD1	2:B:432:ALA:HB2	2.14	0.78
2:B:409:LYS:O	2:B:413:LYS:HG3	1.83	0.78
2:B:566:ALA:C	2:B:574:ASN:HB3	1.86	0.78
3:M:9:ASP:O	3:M:75:TRP:CD1	2.37	0.78
3:M:228:LYS:NZ	3:M:326:HIS:HA	1.98	0.78
3:M:403:THR:HG23	3:M:407:THR:OG1	1.82	0.78
1:A:429:VAL:HG11	1:A:473:ILE:HD11	1.64	0.78
1:A:634:ASN:OD1	2:B:556:LEU:N	2.10	0.78
2:B:104:TYR:O	2:B:107:ARG:N	2.16	0.78
2:B:144:ASP:HA	2:B:179:LYS:CE	2.13	0.78
2:B:146:LYS:O	2:B:147:MET:SD	2.42	0.78
2:B:174:ALA:O	2:B:214:ALA:CB	2.32	0.78
2:B:219:TYR:CG	2:B:226:LEU:CB	2.48	0.78
2:B:336:ASN:ND2	2:B:338:LYS:HB2	1.98	0.78
2:B:374:PHE:CE2	2:B:398:ILE:HG21	2.18	0.78
2:B:437:SER:O	2:B:478:LEU:HD23	1.83	0.78
2:B:509:ALA:HB1	2:B:547:GLN:HG3	1.47	0.78
3:M:45:SER:CA	3:M:47:SER:N	2.47	0.78
3:M:360:LEU:CD2	3:M:362:PHE:CZ	2.66	0.78
4:S:8:PHE:CG	4:S:36:TYR:HE2	2.01	0.78
1:A:182:ILE:HG21	1:A:218:ALA:HA	1.66	0.78
1:A:244:LEU:HD21	1:A:277:LYS:O	1.83	0.78
1:A:333:ILE:O	1:A:337:LEU:HG	1.84	0.78
2:B:37:TYR:HE2	2:B:38:TYR:CE1	1.97	0.78
2:B:178:ILE:HG21	2:B:214:ALA:C	2.04	0.78
2:B:275:ARG:HG3	2:B:291:TYR:HD2	1.37	0.78
3:M:379:LEU:HD22	3:M:386:PHE:CB	2.13	0.78
4:S:34:GLN:CB	4:S:58:LEU:HD11	2.14	0.78
1:A:90:HIS:O	1:A:94:VAL:HG23	1.83	0.78

Continued on next page...

Continued from previous page...

Atom-1	Atom-2	Interatomic distance (Å)	Clash overlap (Å)
1:A:408:ILE:HG22	4:S:65:ASN:CA	2.13	0.78
1:A:631:SER:CB	2:B:557:SER:HB2	2.10	0.78
2:B:472:VAL:CG1	2:B:510:GLY:C	2.51	0.78
2:B:500:GLN:OE1	2:B:503:LEU:HD21	1.83	0.78
3:M:101:LEU:CA	3:M:109:LEU:CD1	2.55	0.78
2:B:178:ILE:CG1	2:B:214:ALA:CB	2.61	0.77
2:B:223:LEU:HD23	2:B:255:TYR:HE1	1.47	0.77
2:B:230:PHE:HD2	2:B:298:ASP:HB3	1.48	0.77
2:B:475:ILE:CG2	2:B:514:LEU:HD21	2.13	0.77
3:M:283:PHE:CZ	3:M:289:THR:CB	2.62	0.77
2:B:20:ARG:HG3	2:B:21:GLU:N	1.99	0.77
2:B:170:ARG:HA	2:B:199:LEU:HD23	1.64	0.77
2:B:252:LEU:HB2	2:B:302:PHE:CE2	2.19	0.77
2:B:278:PRO:CB	2:B:288:TYR:C	2.44	0.77
2:B:331:PRO:HB3	2:B:369:LEU:HD11	1.64	0.77
2:B:513:TRP:O	2:B:551:LEU:HD22	1.83	0.77
3:M:224:VAL:N	3:M:479:PHE:CD1	2.53	0.77
3:M:380:ARG:HH11	3:M:410:VAL:HG11	1.47	0.77
4:S:16:LEU:CD2	4:S:128:LEU:HD23	2.14	0.77
1:A:139:ASP:C	1:A:177:ILE:HD11	2.04	0.77
1:A:140:VAL:HG22	1:A:177:ILE:CG1	2.14	0.77
1:A:323:CYS:SG	1:A:334:SER:HB2	2.25	0.77
1:A:401:VAL:CG2	1:A:418:ILE:N	2.47	0.77
2:B:353:GLN:HE22	3:M:47:SER:HB3	1.42	0.77
2:B:380:LYS:NZ	3:M:236:LEU:CG	2.47	0.77
2:B:537:PHE:HE2	2:B:598:LEU:O	1.68	0.77
3:M:290:PHE:CZ	3:M:297:PHE:CE2	2.71	0.77
4:S:109:LEU:HD12	4:S:113:PHE:HD1	0.92	0.77
1:A:256:LEU:O	1:A:260:PHE:CD1	2.37	0.77
1:A:498:LEU:HB3	1:A:504:ILE:HG13	1.66	0.77
2:B:170:ARG:HH12	2:B:198:GLU:HG2	0.69	0.77
2:B:364:HIS:ND1	2:B:397:GLN:HB3	2.00	0.77
3:M:228:LYS:HZ2	3:M:326:HIS:HA	1.50	0.77
3:M:360:LEU:HD13	3:M:433:VAL:HB	1.66	0.77
4:S:53:THR:CB	4:S:69:ASN:CA	2.54	0.77
4:S:108:SER:OG	4:S:149:ILE:HG21	1.83	0.77
1:A:147:LEU:CD1	1:A:166:LEU:CD2	2.62	0.77
1:A:176:TYR:HB2	4:S:155:GLU:CG	2.09	0.77
1:A:275:LEU:CD2	1:A:311:THR:OG1	2.28	0.77
1:A:304:LEU:HD22	1:A:344:ILE:HG21	1.66	0.77
2:B:123:LEU:HD22	2:B:138:ALA:C	2.04	0.77

Continued on next page...

Continued from previous page...

Atom-1	Atom-2	Interatomic distance (Å)	Clash overlap (Å)
2:B:219:TYR:HB3	2:B:255:TYR:CD1	2.17	0.77
2:B:340:ILE:HG13	2:B:373:LEU:CD2	2.13	0.77
2:B:479:VAL:HG21	2:B:486:HIS:CD2	2.20	0.77
2:B:530:LEU:CD2	2:B:591:MET:HB3	2.14	0.77
2:B:562:ASN:O	2:B:580:TYR:O	2.03	0.77
3:M:101:LEU:CD1	3:M:106:LYS:HA	2.14	0.77
3:M:304:VAL:HG11	3:M:445:SER:HA	1.65	0.77
3:M:428:VAL:O	3:M:429:ASP:C	2.19	0.77
1:A:142:LYS:HA	4:S:159:ALA:CB	2.13	0.77
1:A:401:VAL:CB	1:A:419:ILE:HD12	2.14	0.77
2:B:63:MET:HE1	2:B:104:TYR:HB2	1.66	0.77
2:B:127:LEU:CD1	2:B:157:THR:CB	2.58	0.77
2:B:284:ASN:CG	2:B:285:GLU:HG3	2.03	0.77
2:B:437:SER:O	2:B:478:LEU:HD21	1.81	0.77
2:B:545:ARG:HB2	2:B:602:ASP:OD2	1.84	0.77
2:B:562:ASN:CB	2:B:580:TYR:CB	2.62	0.77
3:M:69:ILE:CG1	3:M:90:PHE:CZ	2.67	0.77
3:M:99:ILE:H	3:M:99:ILE:HD12	1.48	0.77
3:M:284:SER:O	3:M:285:PRO:C	2.09	0.77
3:M:302:TYR:CE2	3:M:445:SER:HB2	2.19	0.77
1:A:401:VAL:HB	1:A:419:ILE:CD1	2.14	0.77
1:A:460:LEU:O	1:A:463:ASP:N	2.16	0.77
2:B:2:VAL:HG11	2:B:6:HIS:CE1	2.17	0.77
2:B:106:LEU:HD13	2:B:144:ASP:CA	2.14	0.77
3:M:243:ILE:N	3:M:474:THR:CG2	2.35	0.77
3:M:256:VAL:HG22	3:M:452:ILE:CG2	2.15	0.77
3:M:257:ALA:HB1	3:M:287:ASN:HD21	1.50	0.77
4:S:75:ILE:HG22	4:S:86:THR:HG23	1.67	0.77
1:A:244:LEU:HG	1:A:277:LYS:HG3	1.65	0.77
1:A:384:LEU:HD13	1:A:435:ILE:HG22	1.67	0.77
2:B:139:LEU:HD23	2:B:173:VAL:N	2.00	0.77
2:B:513:TRP:HB2	2:B:551:LEU:HD11	1.65	0.77
2:B:563:PHE:CE1	2:B:588:ILE:HD12	2.19	0.77
3:M:235:LEU:CD1	3:M:306:LEU:HB3	2.14	0.77
3:M:348:LYS:HG3	3:M:405:THR:CG2	2.13	0.77
1:A:178:ARG:HB3	1:A:214:VAL:HG13	1.67	0.77
1:A:353:ASP:OD1	1:A:378:ILE:HD13	1.84	0.77
1:A:636:TYR:H	2:B:554:LYS:CD	1.97	0.77
2:B:191:GLU:C	2:B:193:LEU:N	2.37	0.77
2:B:292:GLU:HG3	2:B:296:ASP:HB2	1.46	0.77
3:M:223:HIS:CE1	3:M:476:THR:H	2.03	0.77

Continued on next page...

Continued from previous page...

Atom-1	Atom-2	Interatomic distance (Å)	Clash overlap (Å)
3:M:235:LEU:HD11	3:M:306:LEU:CB	2.14	0.77
3:M:340:LEU:HG	3:M:411:LEU:HB3	1.66	0.77
3:M:379:LEU:CD2	3:M:397:TRP:HE1	1.98	0.77
2:B:86:VAL:CG1	2:B:101:ILE:CG2	2.57	0.77
2:B:105:LEU:HB3	2:B:145:MET:HE1	1.65	0.77
2:B:124:GLN:HB2	2:B:153:ILE:HG23	1.67	0.77
2:B:424:PHE:HD2	2:B:428:VAL:HG11	1.48	0.77
3:M:4:SER:O	3:M:78:ALA:C	2.23	0.77
4:S:57:LEU:HA	4:S:60:SER:HB2	1.67	0.77
2:B:120:ILE:CD1	2:B:142:LEU:CD2	2.63	0.76
3:M:3:LEU:HB2	3:M:20:LEU:CD1	2.13	0.76
3:M:246:VAL:O	3:M:297:PHE:CE2	2.37	0.76
4:S:47:GLN:OE1	4:S:79:ASN:N	2.18	0.76
4:S:87:PHE:CE1	4:S:102:ILE:HG23	2.20	0.76
4:S:89:VAL:CG1	4:S:98:ILE:HG21	2.15	0.76
4:S:112:CYS:SG	4:S:153:VAL:HG21	2.25	0.76
1:A:79:MET:HG2	1:A:112:GLN:OE1	1.84	0.76
1:A:255:ARG:O	1:A:258:LYS:N	2.18	0.76
1:A:288:THR:OG1	1:A:291:ILE:CB	2.28	0.76
1:A:316:LEU:HD11	1:A:341:ILE:HG21	1.67	0.76
2:B:144:ASP:HA	2:B:179:LYS:CD	2.16	0.76
2:B:334:MET:HE2	2:B:339:PHE:CE1	2.20	0.76
3:M:215:TYR:CE1	3:M:468:LYS:HA	2.18	0.76
3:M:253:ASN:OD1	3:M:292:PRO:CG	2.33	0.76
1:A:252:ILE:HA	4:S:144:THR:HG1	1.49	0.76
2:B:90:ILE:HD11	2:B:102:HIS:CD2	2.21	0.76
2:B:184:GLY:O	2:B:188:TYR:HB2	1.84	0.76
2:B:197:LYS:C	2:B:199:LEU:N	2.36	0.76
2:B:227:HIS:C	2:B:229:HIS:N	2.21	0.76
2:B:243:TRP:CZ2	3:M:94:GLU:C	2.55	0.76
2:B:279:LEU:CG	2:B:288:TYR:CD1	2.64	0.76
2:B:526:CYS:N	2:B:527:PRO:CD	2.48	0.76
2:B:549:LEU:HD21	2:B:610:ARG:C	2.01	0.76
4:S:75:ILE:HG22	4:S:77:TYR:CE1	2.21	0.76
1:A:102:GLN:HE21	4:S:165:SER:N	1.83	0.76
1:A:594:PHE:CE2	2:B:474:VAL:HG22	2.20	0.76
2:B:29:LYS:HE2	2:B:30:LEU:CA	2.16	0.76
2:B:196:LEU:HB3	2:B:215:TYR:CZ	2.20	0.76
2:B:458:MET:SD	2:B:471:TYR:CB	2.72	0.76
4:S:53:THR:HG23	4:S:67:GLU:C	1.98	0.76
1:A:63:ASP:O	4:S:165:SER:OG	2.01	0.76

Continued on next page...

Continued from previous page...

Atom-1	Atom-2	Interatomic distance (Å)	Clash overlap (Å)
1:A:67:LYS:HB3	1:A:94:VAL:CG2	2.14	0.76
1:A:103:LYS:HB3	1:A:107:TYR:HE1	1.46	0.76
1:A:105:VAL:CA	4:S:167:ILE:HD13	2.16	0.76
1:A:183:THR:OG1	4:S:142:ILE:HD11	1.83	0.76
1:A:215:VAL:O	1:A:216:SER:C	2.18	0.76
2:B:5:ILE:HA	2:B:8:ILE:HD12	1.66	0.76
2:B:6:HIS:ND1	3:M:25:PRO:HB3	2.00	0.76
2:B:93:ASN:HA	2:B:134:LEU:CD1	2.15	0.76
2:B:136:CYS:HA	2:B:172:GLU:HB2	1.67	0.76
2:B:182:ARG:NH1	2:B:217:GLU:OE1	2.18	0.76
2:B:232:ARG:HG3	2:B:236:ILE:HG13	1.65	0.76
2:B:261:PRO:HG2	2:B:292:GLU:CA	2.15	0.76
2:B:334:MET:HA	2:B:334:MET:CE	2.14	0.76
4:S:17:VAL:HG13	4:S:19:PHE:CE1	2.21	0.76
4:S:53:THR:HG21	4:S:68:VAL:N	2.00	0.76
1:A:140:VAL:C	4:S:155:GLU:HB3	2.05	0.76
1:A:516:ILE:CD1	1:A:551:LEU:HD13	2.15	0.76
2:B:105:LEU:CB	2:B:145:MET:HE3	2.11	0.76
2:B:215:TYR:CE2	2:B:229:HIS:CB	2.69	0.76
3:M:348:LYS:O	3:M:405:THR:HG23	1.85	0.76
4:S:131:VAL:HG21	4:S:153:VAL:CG2	2.14	0.76
1:A:101:GLN:O	4:S:167:ILE:CG1	2.31	0.76
1:A:370:LYS:O	1:A:374:LEU:HD13	1.86	0.76
2:B:47:LEU:HB3	2:B:66:ILE:HG12	1.67	0.76
2:B:172:GLU:O	2:B:173:VAL:C	2.15	0.76
2:B:212:VAL:C	2:B:214:ALA:N	2.35	0.76
2:B:260:LEU:HB3	2:B:291:TYR:CZ	2.12	0.76
2:B:479:VAL:CG2	2:B:486:HIS:NE2	2.48	0.76
3:M:65:TYR:CD2	3:M:86:PRO:HA	2.20	0.76
3:M:373:ALA:HB3	3:M:418:GLU:O	1.86	0.76
3:M:443:SER:HB3	3:M:447:ILE:N	2.00	0.76
4:S:53:THR:CB	4:S:69:ASN:HB2	2.16	0.76
4:S:69:ASN:O	4:S:73:ILE:O	2.04	0.76
4:S:75:ILE:CG2	4:S:86:THR:CG2	2.64	0.76
1:A:99:LYS:HZ3	4:S:164:ASP:CB	1.97	0.76
1:A:606:PHE:CG	1:A:629:LEU:HG	2.20	0.76
2:B:83:PHE:O	2:B:87:VAL:HG23	1.85	0.76
2:B:178:ILE:HG23	2:B:217:GLU:CB	2.15	0.76
2:B:334:MET:HB2	2:B:369:LEU:HD23	1.67	0.76
3:M:214:LEU:O	3:M:467:TYR:HB3	1.85	0.76
3:M:273:HIS:CB	3:M:298:ARG:O	2.32	0.76

Continued on next page...

Continued from previous page...

Atom-1	Atom-2	Interatomic distance (Å)	Clash overlap (Å)
1:A:101:GLN:HG2	4:S:160:ALA:HB1	1.66	0.76
1:A:207:LEU:O	1:A:243:ILE:HD11	1.86	0.76
2:B:36:THR:HA	2:B:39:SER:OG	1.84	0.76
2:B:396:ILE:CG1	2:B:418:TYR:HE2	1.98	0.76
3:M:101:LEU:C	3:M:106:LYS:HA	2.06	0.76
3:M:223:HIS:CD2	3:M:478:ASN:HB2	2.20	0.76
3:M:224:VAL:O	3:M:479:PHE:CA	2.34	0.76
4:S:85:PHE:CE2	4:S:109:LEU:HD23	2.21	0.76
1:A:207:LEU:CD2	1:A:239:LEU:CB	2.58	0.76
1:A:244:LEU:HD11	1:A:281:LEU:HD11	1.68	0.76
1:A:623:MET:O	2:B:617:LEU:CD2	2.34	0.76
1:A:627:GLU:HG2	2:B:617:LEU:HA	1.67	0.76
2:B:549:LEU:HD21	2:B:611:ALA:H	0.94	0.76
2:B:559:ASP:O	2:B:562:ASN:CA	2.33	0.76
2:B:563:PHE:O	2:B:564:LYS:O	2.03	0.76
3:M:217:ASP:OD1	3:M:440:ILE:HG23	1.85	0.76
3:M:342:LEU:HD12	3:M:411:LEU:HB3	1.68	0.76
1:A:74:LEU:HD22	1:A:87:CYS:CB	2.14	0.75
1:A:101:GLN:N	4:S:162:SER:N	2.34	0.75
1:A:179:LYS:NZ	4:S:143:GLU:HB2	2.00	0.75
1:A:254:ILE:HG13	1:A:290:VAL:HG22	1.68	0.75
1:A:264:SER:HB3	1:A:271:ARG:HG3	1.68	0.75
1:A:421:PRO:CG	1:A:424:TYR:CE1	2.66	0.75
1:A:481:MET:HE2	1:A:518:CYS:HB3	1.68	0.75
1:A:595:GLU:HG3	2:B:469:ASP:HB3	0.75	0.75
2:B:67:ILE:HD13	2:B:103:LEU:CB	2.15	0.75
2:B:178:ILE:HD13	2:B:218:CYS:CB	2.15	0.75
2:B:433:VAL:CB	2:B:474:VAL:HG21	2.15	0.75
2:B:500:GLN:HB3	2:B:503:LEU:HG	1.68	0.75
2:B:589:SER:OG	2:B:618:PHE:CE2	2.39	0.75
3:M:60:LEU:HD22	3:M:62:VAL:HG22	1.67	0.75
3:M:99:ILE:HG12	3:M:128:CYS:SG	2.27	0.75
3:M:222:PHE:CB	3:M:479:PHE:HZ	1.93	0.75
3:M:224:VAL:C	3:M:479:PHE:HA	2.06	0.75
3:M:245:ASP:CB	3:M:472:TYR:HD1	1.95	0.75
3:M:306:LEU:HD22	3:M:317:MET:HE3	1.68	0.75
3:M:383:HIS:HB2	3:M:403:THR:OG1	1.85	0.75
1:A:103:LYS:CG	4:S:163:THR:CG2	2.57	0.75
1:A:461:CYS:CB	1:A:469:LEU:HD23	2.17	0.75
2:B:29:LYS:O	2:B:32:GLU:HG3	1.78	0.75
2:B:154:ILE:CD1	2:B:180:LEU:CB	2.64	0.75

Continued on next page...

Continued from previous page...

Atom-1	Atom-2	Interatomic distance (Å)	Clash overlap (Å)
2:B:178:ILE:CB	2:B:214:ALA:HB1	2.16	0.75
2:B:231:ARG:HG2	2:B:298:ASP:OD1	1.84	0.75
2:B:556:LEU:HD23	2:B:588:ILE:CD1	2.17	0.75
2:B:596:LEU:HD22	2:B:615:SER:OG	1.84	0.75
3:M:101:LEU:CG	3:M:106:LYS:O	2.35	0.75
3:M:104:PHE:HZ	3:M:113:LYS:HZ2	0.77	0.75
3:M:320:ILE:HG13	3:M:347:PHE:CD1	2.20	0.75
3:M:379:LEU:HD23	3:M:397:TRP:NE1	2.01	0.75
4:S:54:PRO:CB	4:S:57:LEU:CD1	2.64	0.75
1:A:166:LEU:HD12	1:A:185:LEU:CD2	2.04	0.75
1:A:178:ARG:CZ	1:A:209:ASP:CG	2.55	0.75
1:A:320:HIS:O	1:A:322:PHE:N	2.19	0.75
2:B:346:THR:O	2:B:350:THR:N	2.18	0.75
2:B:390:VAL:O	2:B:393:ILE:HB	1.86	0.75
2:B:563:PHE:O	2:B:566:ALA:CA	2.35	0.75
2:B:592:TYR:CE2	2:B:618:PHE:CD2	2.74	0.75
3:M:437:TYR:HB2	3:M:439:TYR:CZ	2.21	0.75
4:S:9:ASN:HD21	4:S:13:GLN:HG2	1.51	0.75
4:S:47:GLN:HE22	4:S:79:ASN:N	1.84	0.75
4:S:73:ILE:HG23	4:S:88:ILE:HG22	1.65	0.75
1:A:178:ARG:CZ	1:A:209:ASP:OD2	2.35	0.75
1:A:264:SER:CB	1:A:271:ARG:HG3	2.15	0.75
2:B:108:PHE:CD2	2:B:115:LEU:HB2	2.22	0.75
2:B:307:ASN:O	2:B:310:ILE:N	2.19	0.75
2:B:343:LEU:HD21	2:B:359:LEU:O	1.86	0.75
2:B:363:ILE:HG21	2:B:398:ILE:HD13	1.66	0.75
2:B:469:ASP:OD1	2:B:506:ASN:HB3	1.86	0.75
3:M:6:TYR:OH	3:M:17:GLN:NE2	2.18	0.75
3:M:16:PHE:HA	3:M:118:TYR:CE1	2.22	0.75
3:M:18:TYR:HE2	3:M:20:LEU:CD2	1.98	0.75
4:S:131:VAL:CG2	4:S:153:VAL:CG2	2.58	0.75
1:A:117:ASP:CG	1:A:120:ILE:HG12	2.07	0.75
1:A:260:PHE:CG	1:A:274:LEU:HG	2.22	0.75
2:B:106:LEU:HD11	2:B:144:ASP:CB	1.80	0.75
2:B:267:ASP:N	2:B:289:PRO:CG	2.49	0.75
3:M:374:TYR:CB	3:M:417:TYR:CD2	2.69	0.75
1:A:104:ARG:HG3	1:A:145:ILE:CD1	2.15	0.75
1:A:403:LEU:CD2	1:A:421:PRO:C	2.44	0.75
1:A:638:LEU:O	2:B:486:HIS:HE1	1.70	0.75
2:B:177:ILE:CG2	2:B:196:LEU:HD11	2.16	0.75
2:B:277:CYS:C	2:B:288:TYR:HB3	2.04	0.75

Continued on next page...

Continued from previous page...

Atom-1	Atom-2	Interatomic distance (Å)	Clash overlap (Å)
2:B:334:MET:CE	2:B:339:PHE:CE1	2.70	0.75
3:M:221:THR:HB	3:M:474:THR:C	2.06	0.75
3:M:290:PHE:CD1	3:M:297:PHE:CE1	2.75	0.75
3:M:350:VAL:HG22	3:M:442:GLN:CD	2.06	0.75
3:M:421:GLY:O	3:M:422:PRO:C	2.22	0.75
4:S:8:PHE:CB	4:S:36:TYR:CZ	2.62	0.75
1:A:322:PHE:CG	1:A:330:LEU:HD21	2.22	0.75
1:A:557:LYS:CD	2:B:605:PHE:HB3	2.12	0.75
2:B:118:LEU:O	2:B:121:ASN:N	2.20	0.75
2:B:286:ILE:CG2	2:B:288:TYR:OH	2.34	0.75
2:B:473:ASN:O	2:B:476:ARG:HB3	1.86	0.75
2:B:550:VAL:CG2	2:B:610:ARG:HD3	2.13	0.75
3:M:121:ILE:CG2	3:M:125:PHE:CE1	2.70	0.75
3:M:219:LEU:O	3:M:474:THR:HG23	1.86	0.75
3:M:222:PHE:CD1	3:M:439:TYR:HE1	2.04	0.75
3:M:241:HIS:HB2	3:M:476:THR:HG23	1.67	0.75
3:M:454:ILE:CG2	3:M:464:THR:CG2	2.53	0.75
4:S:16:LEU:HD23	4:S:128:LEU:CD2	2.16	0.75
1:A:304:LEU:HD12	1:A:312:ALA:HB2	1.68	0.75
2:B:602:ASP:OD1	2:B:603:ASP:N	2.19	0.75
3:M:258:VAL:HG13	3:M:452:ILE:CG1	2.16	0.75
3:M:290:PHE:CZ	3:M:297:PHE:CZ	2.74	0.75
3:M:437:TYR:N	3:M:437:TYR:CD1	2.53	0.75
1:A:581:LEU:HD23	1:A:607:LEU:HD21	1.69	0.75
1:A:633:PHE:HD2	2:B:551:LEU:CA	1.71	0.75
1:A:636:TYR:CG	2:B:554:LYS:NZ	2.55	0.75
2:B:60:ARG:HD2	2:B:96:LYS:CG	2.16	0.75
2:B:171:GLY:HA3	2:B:207:VAL:CA	2.17	0.75
2:B:189:HIS:NE2	2:B:225:LEU:HD23	2.00	0.75
2:B:200:MET:CG	2:B:232:ARG:HB3	2.13	0.75
2:B:215:TYR:CB	2:B:226:LEU:CD1	2.63	0.75
2:B:310:ILE:HG12	2:B:318:ILE:HA	1.69	0.75
2:B:319:LEU:HD12	2:B:358:MET:CG	1.79	0.75
2:B:328:LEU:HB2	2:B:333:GLN:HE22	1.52	0.75
2:B:346:THR:HG23	2:B:350:THR:HG23	1.66	0.75
2:B:444:THR:O	2:B:445:SER:C	2.15	0.75
2:B:597:TYR:O	2:B:601:TYR:CE1	2.39	0.75
3:M:52:ASP:HA	3:M:67:SER:CA	2.17	0.75
1:A:129:LYS:HA	1:A:165:ASP:OD2	1.86	0.74
2:B:219:TYR:CE1	2:B:226:LEU:HD12	2.19	0.74
2:B:225:LEU:HD13	2:B:283:TYR:CZ	2.15	0.74

Continued on next page...

Continued from previous page...

Atom-1	Atom-2	Interatomic distance (Å)	Clash overlap (Å)
2:B:596:LEU:HD13	2:B:611:ALA:O	1.87	0.74
3:M:67:SER:CB	3:M:90:PHE:CD1	2.61	0.74
3:M:101:LEU:CD1	3:M:109:LEU:CD1	2.65	0.74
3:M:342:LEU:HD11	3:M:411:LEU:HD23	1.65	0.74
1:A:291:ILE:HG21	1:A:318:ARG:HB3	1.63	0.74
1:A:372:ILE:CG2	1:A:427:LYS:HE3	2.17	0.74
1:A:556:VAL:HG11	2:B:610:ARG:NH2	2.02	0.74
2:B:219:TYR:CG	2:B:223:LEU:HD23	2.20	0.74
2:B:299:LEU:O	2:B:302:PHE:HB3	1.87	0.74
2:B:337:THR:HA	2:B:373:LEU:HD22	1.63	0.74
2:B:500:GLN:CB	2:B:503:LEU:HG	2.17	0.74
4:S:54:PRO:HB2	4:S:57:LEU:HD11	1.70	0.74
1:A:99:LYS:CD	4:S:164:ASP:H	1.94	0.74
1:A:186:PHE:CD1	1:A:224:GLU:HB3	2.22	0.74
1:A:609:LEU:CD2	1:A:628:VAL:HG11	2.17	0.74
2:B:37:TYR:CE2	2:B:42:ILE:HA	2.22	0.74
2:B:37:TYR:O	2:B:39:SER:N	2.20	0.74
2:B:63:MET:HE2	2:B:104:TYR:HB2	1.69	0.74
2:B:278:PRO:N	2:B:292:GLU:CD	2.37	0.74
2:B:546:CYS:CB	2:B:607:ILE:CD1	2.42	0.74
3:M:18:TYR:HD1	3:M:122:SER:CA	1.80	0.74
3:M:449:VAL:HG11	3:M:452:ILE:HD11	0.75	0.74
1:A:163:ALA:CA	1:A:199:ASN:HD21	2.00	0.74
1:A:287:ALA:HB3	1:A:288:THR:C	2.06	0.74
2:B:38:TYR:CD2	2:B:65:ARG:CD	2.33	0.74
2:B:70:MET:HB2	2:B:104:TYR:CE1	2.22	0.74
2:B:143:SER:CA	2:B:179:LYS:HB2	2.16	0.74
2:B:599:ALA:C	2:B:601:TYR:N	2.39	0.74
3:M:443:SER:CA	3:M:447:ILE:HG13	2.18	0.74
1:A:397:ASP:O	1:A:418:ILE:CG1	2.35	0.74
2:B:310:ILE:O	2:B:311:TYR:C	2.13	0.74
4:S:17:VAL:HG22	4:S:19:PHE:CE2	2.21	0.74
4:S:85:PHE:CD1	4:S:106:VAL:HG22	2.21	0.74
1:A:183:THR:OG1	4:S:142:ILE:HD13	1.86	0.74
1:A:220:SER:HB3	4:S:142:ILE:HG22	0.75	0.74
2:B:378:THR:CG2	2:B:379:LYS:H	1.99	0.74
2:B:390:VAL:HA	2:B:393:ILE:HD12	1.69	0.74
2:B:602:ASP:O	2:B:608:ARG:CZ	2.36	0.74
3:M:67:SER:OG	3:M:90:PHE:CB	2.35	0.74
3:M:95:THR:O	3:M:99:ILE:HD12	1.87	0.74
1:A:80:TYR:HB2	1:A:82:PHE:CE2	2.22	0.74

Continued on next page...

Continued from previous page...

Atom-1	Atom-2	Interatomic distance (Å)	Clash overlap (Å)
1:A:110:ALA:O	1:A:111:SER:C	2.14	0.74
1:A:142:LYS:CA	4:S:159:ALA:HB1	2.17	0.74
1:A:179:LYS:NZ	4:S:149:ILE:HG12	2.01	0.74
1:A:212:ILE:CG2	1:A:247:ILE:HD13	2.17	0.74
1:A:398:GLU:O	1:A:420:ILE:CG1	2.34	0.74
2:B:178:ILE:HG13	2:B:214:ALA:CB	2.18	0.74
2:B:197:LYS:C	2:B:199:LEU:H	1.91	0.74
3:M:222:PHE:CE1	3:M:240:ILE:CG2	2.70	0.74
3:M:360:LEU:HD21	3:M:362:PHE:CE2	2.23	0.74
1:A:304:LEU:HA	1:A:308:ASP:HB2	1.67	0.74
1:A:540:ILE:CG1	1:A:551:LEU:HD23	2.16	0.74
2:B:20:ARG:CG	2:B:21:GLU:N	2.49	0.74
2:B:225:LEU:CD1	2:B:283:TYR:HE1	1.83	0.74
1:A:105:VAL:CG2	4:S:167:ILE:CB	2.66	0.74
1:A:175:PRO:HB3	1:A:211:ASP:CG	2.07	0.74
1:A:506:LYS:HE2	3:M:58:ARG:CA	2.18	0.74
1:A:634:ASN:ND2	2:B:554:LYS:HB3	1.79	0.74
2:B:175:LEU:CD2	2:B:210:CYS:CA	2.65	0.74
2:B:277:CYS:CB	2:B:292:GLU:HG3	2.16	0.74
3:M:17:GLN:O	3:M:118:TYR:OH	2.02	0.74
3:M:241:HIS:C	3:M:474:THR:HB	2.06	0.74
1:A:67:LYS:HB3	4:S:166:LYS:HA	1.70	0.74
1:A:67:LYS:CG	4:S:165:SER:HG	2.01	0.74
1:A:391:LEU:O	1:A:392:MET:C	2.17	0.74
1:A:436:CYS:HB2	1:A:450:TYR:CE1	2.23	0.74
1:A:606:PHE:CZ	2:B:550:VAL:HG11	2.21	0.74
1:A:624:LEU:O	2:B:617:LEU:HD11	1.88	0.74
2:B:490:ILE:HG23	2:B:515:PHE:CD2	2.23	0.74
3:M:5:PHE:CD2	3:M:78:ALA:HB3	2.16	0.74
3:M:64:LYS:HG2	3:M:79:SER:C	2.07	0.74
3:M:101:LEU:CD1	3:M:106:LYS:CA	2.65	0.74
1:A:213:SER:H	4:S:148:ARG:HD3	1.52	0.73
1:A:383:ASN:O	1:A:387:ILE:HG12	1.88	0.73
2:B:90:ILE:HG12	2:B:98:LYS:HE2	1.69	0.73
2:B:139:LEU:HD21	2:B:173:VAL:C	2.08	0.73
2:B:162:VAL:HG11	2:B:195:ILE:HA	1.67	0.73
2:B:171:GLY:HA3	2:B:207:VAL:CB	2.18	0.73
2:B:199:LEU:C	2:B:201:ALA:H	1.89	0.73
2:B:497:LEU:CD2	2:B:533:LEU:HD22	2.18	0.73
3:M:10:THR:HG23	3:M:11:LYS:HG3	1.70	0.73
3:M:94:GLU:O	3:M:97:ASP:HB2	1.88	0.73

Continued on next page...

Continued from previous page...

Atom-1	Atom-2	Interatomic distance (Å)	Clash overlap (Å)
1:A:132:LEU:CD1	1:A:165:ASP:CB	2.65	0.73
1:A:384:LEU:CD1	1:A:441:TYR:CE2	2.71	0.73
1:A:450:TYR:O	1:A:454:ILE:HG12	1.88	0.73
1:A:513:ARG:CD	1:A:550:VAL:CG2	2.53	0.73
1:A:633:PHE:CZ	2:B:513:TRP:CE3	2.70	0.73
2:B:114:ASN:O	2:B:117:LEU:HB2	1.87	0.73
2:B:132:SER:HB2	2:B:166:SER:HB3	1.70	0.73
2:B:430:ILE:HG23	2:B:470:ALA:CB	2.18	0.73
2:B:478:LEU:O	2:B:480:GLN:N	2.21	0.73
2:B:553:ALA:CB	2:B:614:ILE:CG2	2.66	0.73
2:B:83:PHE:HE2	2:B:119:SER:HA	1.53	0.73
2:B:141:ALA:O	2:B:143:SER:N	2.21	0.73
2:B:175:LEU:CG	2:B:210:CYS:CB	2.63	0.73
3:M:214:LEU:C	3:M:214:LEU:HD23	2.08	0.73
3:M:217:ASP:H	3:M:470:ALA:CB	1.93	0.73
3:M:222:PHE:CG	3:M:439:TYR:CE1	2.76	0.73
4:S:109:LEU:CD1	4:S:113:PHE:HD1	1.70	0.73
1:A:378:ILE:O	1:A:378:ILE:HG13	1.87	0.73
2:B:479:VAL:HG22	2:B:486:HIS:CG	2.22	0.73
2:B:513:TRP:CB	2:B:551:LEU:HD11	2.18	0.73
3:M:45:SER:CB	3:M:51:LEU:CD1	2.62	0.73
4:S:4:ALA:CA	4:S:18:LYS:O	2.35	0.73
1:A:186:PHE:HE2	1:A:187:LYS:HD3	1.53	0.73
1:A:254:ILE:HG23	1:A:293:GLU:CG	2.16	0.73
2:B:108:PHE:HE2	2:B:115:LEU:HB2	1.50	0.73
3:M:247:ARG:H	3:M:470:ALA:HB2	1.53	0.73
3:M:443:SER:CB	3:M:447:ILE:N	2.51	0.73
1:A:102:GLN:NE2	4:S:165:SER:N	2.37	0.73
1:A:233:PHE:O	1:A:234:ILE:C	2.05	0.73
1:A:319:LEU:O	1:A:320:HIS:O	2.07	0.73
1:A:322:PHE:CD2	1:A:330:LEU:CD2	2.71	0.73
1:A:516:ILE:HG12	1:A:551:LEU:HD13	1.68	0.73
1:A:633:PHE:HE1	2:B:513:TRP:CZ3	1.88	0.73
2:B:212:VAL:HG21	2:B:248:LEU:CD2	2.14	0.73
2:B:343:LEU:O	2:B:347:VAL:HG23	1.88	0.73
2:B:438:ARG:HA	2:B:441:GLN:HE21	1.52	0.73
2:B:476:ARG:HA	2:B:514:LEU:HD13	1.69	0.73
2:B:567:GLN:N	2:B:574:ASN:ND2	2.33	0.73
3:M:101:LEU:HD12	3:M:109:LEU:CD1	2.18	0.73
3:M:437:TYR:CD1	3:M:479:PHE:CE1	2.76	0.73
4:S:43:ASN:O	4:S:44:SER:C	2.20	0.73

Continued on next page...

Continued from previous page...

Atom-1	Atom-2	Interatomic distance (Å)	Clash overlap (Å)
1:A:211:ASP:CG	4:S:148:ARG:CZ	2.57	0.73
2:B:219:TYR:C	2:B:223:LEU:CD2	2.52	0.73
2:B:567:GLN:C	2:B:569:THR:OG1	2.26	0.73
3:M:96:ILE:HG21	3:M:125:PHE:CZ	2.24	0.73
3:M:262:THR:O	3:M:264:GLY:N	2.20	0.73
3:M:338:PHE:HE2	3:M:415:ILE:CG1	2.02	0.73
4:S:17:VAL:CG1	4:S:19:PHE:CE1	2.71	0.73
1:A:630:PRO:CB	2:B:614:ILE:HG23	2.19	0.73
2:B:208:ILE:HD13	2:B:236:ILE:HG21	0.73	0.73
2:B:374:PHE:CZ	2:B:398:ILE:HG21	2.24	0.73
2:B:508:ARG:HD2	2:B:544:THR:HG21	1.70	0.73
4:S:146:VAL:O	4:S:150:VAL:HG23	1.88	0.73
1:A:185:LEU:HD12	1:A:203:PHE:CZ	2.23	0.73
1:A:495:ILE:HD12	1:A:515:CYS:SG	2.28	0.73
2:B:237:ILE:HG12	2:B:309:LEU:HD21	1.70	0.73
2:B:374:PHE:CE1	2:B:381:PHE:CD1	2.77	0.73
2:B:403:ILE:HD11	2:B:442:LEU:HD12	1.70	0.73
2:B:418:TYR:CD1	2:B:424:PHE:CD1	2.75	0.73
2:B:508:ARG:CD	2:B:544:THR:HG21	2.18	0.73
2:B:515:PHE:CD2	2:B:529:VAL:HG11	2.23	0.73
4:S:109:LEU:HD12	4:S:113:PHE:CE1	1.91	0.73
1:A:609:LEU:HG	1:A:628:VAL:CG1	2.19	0.73
2:B:154:ILE:HG23	2:B:176:ALA:HB1	1.71	0.73
2:B:174:ALA:O	2:B:214:ALA:HB1	1.88	0.73
2:B:177:ILE:HG21	2:B:196:LEU:CD1	2.18	0.73
2:B:275:ARG:CB	2:B:294:VAL:HG13	1.82	0.73
2:B:393:ILE:CG2	2:B:431:MET:HB2	2.18	0.73
3:M:65:TYR:CE2	3:M:86:PRO:CB	2.68	0.73
3:M:290:PHE:HZ	3:M:297:PHE:CD2	2.07	0.73
3:M:343:ASN:HD22	3:M:343:ASN:N	1.87	0.73
4:S:10:LYS:HB3	4:S:82:THR:O	1.89	0.73
1:A:100:LEU:N	4:S:162:SER:CA	2.48	0.72
1:A:252:ILE:HG12	4:S:145:ASN:N	2.04	0.72
2:B:154:ILE:HD12	2:B:180:LEU:CB	2.18	0.72
2:B:271:GLU:O	2:B:272:GLY:C	2.20	0.72
3:M:126:ASN:O	3:M:130:GLU:HG2	1.88	0.72
3:M:257:ALA:CB	3:M:455:VAL:HG21	2.19	0.72
4:S:50:PHE:CB	4:S:76:ILE:HD13	2.19	0.72
4:S:53:THR:CG2	4:S:68:VAL:CA	2.63	0.72
1:A:223:CYS:O	1:A:226:SER:OG	2.03	0.72
1:A:263:LEU:O	1:A:266:VAL:O	2.07	0.72

Continued on next page...

Continued from previous page...

Atom-1	Atom-2	Interatomic distance (Å)	Clash overlap (Å)
1:A:408:ILE:HD12	1:A:410:TYR:HE1	1.51	0.72
1:A:539:ASN:O	1:A:540:ILE:C	2.23	0.72
1:A:557:LYS:CE	2:B:606:ASP:CA	2.57	0.72
1:A:633:PHE:HB3	2:B:550:VAL:O	1.88	0.72
3:M:7:ILE:HG23	3:M:75:TRP:O	1.89	0.72
3:M:316:ARG:O	3:M:318:ASN:N	2.21	0.72
1:A:147:LEU:HD21	1:A:166:LEU:HD23	1.66	0.72
1:A:402:ILE:O	1:A:402:ILE:CG2	2.36	0.72
1:A:636:TYR:N	2:B:554:LYS:HD3	1.95	0.72
2:B:63:MET:CG	2:B:100:LEU:CB	2.67	0.72
2:B:267:ASP:C	2:B:276:SER:OG	2.27	0.72
2:B:352:ASN:H	2:B:352:ASN:HD22	1.37	0.72
2:B:553:ALA:CA	2:B:614:ILE:CG2	2.66	0.72
2:B:159:LYS:HD2	2:B:191:GLU:CG	2.18	0.72
2:B:166:SER:O	2:B:170:ARG:HG3	1.89	0.72
2:B:212:VAL:C	2:B:214:ALA:H	1.93	0.72
2:B:490:ILE:O	2:B:515:PHE:HZ	1.71	0.72
4:S:135:ILE:CG2	4:S:141:VAL:HG22	2.20	0.72
1:A:461:CYS:HB2	1:A:469:LEU:HD23	1.71	0.72
1:A:599:ARG:HD2	2:B:547:GLN:NE2	2.04	0.72
2:B:64:LYS:HA	2:B:100:LEU:HD22	1.71	0.72
2:B:106:LEU:CD1	2:B:144:ASP:CA	2.68	0.72
2:B:162:VAL:HG21	2:B:195:ILE:CA	2.20	0.72
2:B:252:LEU:CB	2:B:302:PHE:CE1	2.73	0.72
2:B:278:PRO:N	2:B:292:GLU:OE1	2.22	0.72
2:B:368:ILE:CD1	2:B:401:THR:HG22	2.19	0.72
2:B:416:LYS:HD2	2:B:453:TRP:CE2	2.25	0.72
3:M:353:VAL:CG2	3:M:438:SER:O	2.37	0.72
3:M:379:LEU:HD23	3:M:386:PHE:CB	2.15	0.72
1:A:564:ASN:OD1	1:A:622:PRO:CG	2.38	0.72
1:A:606:PHE:CZ	2:B:550:VAL:CG1	2.72	0.72
1:A:637:GLU:HB2	2:B:551:LEU:HD22	1.69	0.72
2:B:277:CYS:O	2:B:288:TYR:CA	2.37	0.72
2:B:398:ILE:O	2:B:401:THR:N	2.18	0.72
2:B:472:VAL:HG11	2:B:510:GLY:HA3	0.73	0.72
4:S:44:SER:O	4:S:47:GLN:HB2	1.89	0.72
4:S:108:SER:HB3	4:S:149:ILE:HG22	1.70	0.72
1:A:63:ASP:O	1:A:67:LYS:HG3	1.90	0.72
1:A:94:VAL:HG13	1:A:102:GLN:HB3	1.64	0.72
1:A:223:CYS:CB	1:A:259:LEU:HG	2.17	0.72
1:A:291:ILE:HG13	1:A:318:ARG:HG2	1.70	0.72

Continued on next page...

Continued from previous page...

Atom-1	Atom-2	Interatomic distance (Å)	Clash overlap (Å)
2:B:133:GLU:HA	2:B:168:MET:SD	2.28	0.72
2:B:170:ARG:CZ	2:B:198:GLU:HG2	2.20	0.72
2:B:554:LYS:O	2:B:557:SER:N	2.21	0.72
3:M:6:TYR:CD2	3:M:14:LEU:HD11	2.24	0.72
3:M:225:VAL:HA	3:M:480:GLN:O	1.90	0.72
3:M:243:ILE:HG22	3:M:472:TYR:HB3	1.72	0.72
1:A:100:LEU:O	4:S:160:ALA:CA	1.85	0.72
1:A:350:SER:O	1:A:351:ARG:C	2.26	0.72
1:A:523:SER:OG	1:A:562:TRP:NE1	1.85	0.72
1:A:557:LYS:HG3	2:B:606:ASP:HB2	1.71	0.72
2:B:20:ARG:HD2	2:B:21:GLU:CA	2.18	0.72
2:B:98:LYS:HZ1	2:B:134:LEU:HB3	0.70	0.72
2:B:284:ASN:CB	2:B:285:GLU:HG3	2.20	0.72
2:B:505:ASP:O	2:B:506:ASN:C	2.17	0.72
2:B:564:LYS:CD	2:B:621:GLY:O	2.37	0.72
3:M:250:LEU:HD13	3:M:254:PRO:HG2	1.71	0.72
3:M:381:ASN:OD1	3:M:382:THR:N	2.23	0.72
4:S:75:ILE:CG2	4:S:77:TYR:CE1	2.72	0.72
1:A:105:VAL:CG2	4:S:167:ILE:CA	2.67	0.72
1:A:166:LEU:HD13	1:A:185:LEU:HG	1.70	0.72
1:A:213:SER:H	4:S:148:ARG:CD	1.90	0.72
1:A:226:SER:O	1:A:230:PRO:HG3	1.89	0.72
1:A:295:VAL:CG2	1:A:315:CYS:HB3	2.14	0.72
2:B:51:LEU:HD23	2:B:59:VAL:HG13	1.69	0.72
2:B:158:VAL:HG12	2:B:177:ILE:HD11	1.67	0.72
1:A:121:LEU:CD1	1:A:155:THR:HG23	2.20	0.72
1:A:214:VAL:HG22	4:S:148:ARG:NH2	2.03	0.72
1:A:252:ILE:HG12	4:S:144:THR:OG1	1.89	0.72
2:B:177:ILE:HD11	2:B:195:ILE:HG21	1.72	0.72
2:B:227:HIS:CG	2:B:292:GLU:OE2	2.43	0.72
2:B:493:LEU:HG	2:B:511:ILE:CG2	2.18	0.72
3:M:290:PHE:HE1	3:M:297:PHE:CD1	2.05	0.72
1:A:441:TYR:HB3	1:A:444:VAL:CG2	2.20	0.71
2:B:63:MET:HG3	2:B:100:LEU:CB	2.19	0.71
2:B:363:ILE:CG2	2:B:398:ILE:HG12	2.19	0.71
2:B:374:PHE:CE2	2:B:402:LEU:HD11	2.25	0.71
3:M:96:ILE:O	3:M:100:LEU:HD12	1.90	0.71
3:M:219:LEU:HG	3:M:440:ILE:HG12	1.72	0.71
4:S:55:PRO:C	4:S:57:LEU:H	1.91	0.71
1:A:95:MET:C	4:S:166:LYS:NZ	2.43	0.71
1:A:149:GLY:O	1:A:152:THR:N	2.15	0.71

Continued on next page...

Continued from previous page...

Atom-1	Atom-2	Interatomic distance (Å)	Clash overlap (Å)
1:A:204:VAL:O	1:A:205:SER:C	2.25	0.71
1:A:268:PRO:HG3	1:A:271:ARG:HH21	1.55	0.71
1:A:554:ALA:O	1:A:557:LYS:N	2.23	0.71
1:A:603:VAL:HG22	2:B:610:ARG:HH21	1.55	0.71
2:B:578:PRO:CB	2:B:579:PRO:HD2	2.18	0.71
3:M:243:ILE:O	3:M:472:TYR:CG	2.42	0.71
3:M:258:VAL:HG13	3:M:449:VAL:HG11	1.71	0.71
4:S:14:PRO:O	4:S:15:ARG:HD3	1.90	0.71
4:S:57:LEU:CB	4:S:67:GLU:O	2.38	0.71
1:A:80:TYR:CG	1:A:82:PHE:CE2	2.78	0.71
1:A:170:LEU:CD1	1:A:206:LYS:HG3	2.21	0.71
1:A:212:ILE:CB	1:A:247:ILE:CD1	2.68	0.71
1:A:353:ASP:OD1	1:A:378:ILE:CD1	2.39	0.71
1:A:603:VAL:O	1:A:606:PHE:HB2	1.89	0.71
2:B:403:ILE:HB	2:B:408:VAL:HG22	1.72	0.71
2:B:545:ARG:CD	2:B:602:ASP:CB	2.65	0.71
2:B:546:CYS:HA	2:B:607:ILE:CB	2.20	0.71
2:B:550:VAL:O	2:B:553:ALA:HB3	1.89	0.71
3:M:225:VAL:HG22	3:M:480:GLN:HB3	1.71	0.71
1:A:100:LEU:HD22	1:A:138:ASN:HB3	1.72	0.71
1:A:101:GLN:N	4:S:163:THR:H	1.88	0.71
1:A:103:LYS:CA	4:S:163:THR:HG21	2.20	0.71
1:A:121:LEU:HD11	1:A:155:THR:HG23	1.73	0.71
2:B:154:ILE:HD12	2:B:180:LEU:HB2	1.70	0.71
2:B:215:TYR:CE2	2:B:229:HIS:HB3	2.25	0.71
1:A:141:VAL:CB	4:S:159:ALA:CB	2.67	0.71
1:A:189:PHE:HB2	1:A:225:LEU:HD21	1.72	0.71
1:A:275:LEU:O	1:A:277:LYS:N	2.23	0.71
1:A:585:PHE:CE2	1:A:603:VAL:HG12	2.25	0.71
2:B:2:VAL:O	2:B:6:HIS:CD2	2.43	0.71
2:B:93:ASN:CA	2:B:134:LEU:HD11	2.21	0.71
2:B:431:MET:O	2:B:433:VAL:N	2.23	0.71
3:M:18:TYR:HE2	3:M:20:LEU:HD23	1.54	0.71
3:M:99:ILE:HD12	3:M:99:ILE:N	2.04	0.71
3:M:220:GLU:N	3:M:439:TYR:O	2.23	0.71
3:M:287:ASN:OD1	3:M:288:ILE:N	2.22	0.71
3:M:341:SER:HG	3:M:343:ASN:HD21	1.38	0.71
3:M:347:PHE:CZ	3:M:350:VAL:HG11	2.26	0.71
3:M:374:TYR:HB3	3:M:417:TYR:CD2	2.23	0.71
3:M:376:ILE:HD12	3:M:415:ILE:HG12	1.71	0.71
4:S:53:THR:H	4:S:69:ASN:CB	2.04	0.71

Continued on next page...

Continued from previous page...

Atom-1	Atom-2	Interatomic distance (Å)	Clash overlap (Å)
4:S:135:ILE:O	4:S:141:VAL:CG2	2.38	0.71
1:A:179:LYS:CD	4:S:143:GLU:CB	2.68	0.71
1:A:212:ILE:HB	1:A:247:ILE:HD12	1.73	0.71
1:A:329:ASN:OD1	4:S:50:PHE:HZ	1.72	0.71
1:A:626:SER:C	2:B:617:LEU:HG	2.10	0.71
2:B:108:PHE:CD2	2:B:115:LEU:CB	2.73	0.71
2:B:120:ILE:CG1	2:B:150:LEU:HD22	2.21	0.71
2:B:231:ARG:NH2	2:B:297:PRO:HD2	2.04	0.71
2:B:247:TYR:HE2	3:M:91:THR:CG2	1.88	0.71
2:B:337:THR:HA	2:B:373:LEU:HD21	0.75	0.71
2:B:403:ILE:HG22	2:B:411:ILE:HD12	1.71	0.71
3:M:52:ASP:HA	3:M:68:VAL:N	2.04	0.71
3:M:101:LEU:CB	3:M:109:LEU:HD13	2.20	0.71
3:M:258:VAL:HG22	3:M:452:ILE:CG1	2.20	0.71
3:M:290:PHE:CE2	3:M:297:PHE:CE2	2.77	0.71
3:M:343:ASN:HA	3:M:408:VAL:CG1	2.11	0.71
3:M:405:THR:HG22	3:M:406:GLY:N	2.04	0.71
3:M:448:TYR:HE2	3:M:450:GLU:OE1	1.74	0.71
1:A:266:VAL:O	1:A:267:GLU:HB3	1.90	0.71
1:A:332:TYR:HE1	1:A:366:SER:HB2	1.56	0.71
1:A:436:CYS:CB	1:A:450:TYR:CE1	2.73	0.71
2:B:20:ARG:NH1	2:B:35:TYR:CZ	2.48	0.71
2:B:120:ILE:HG13	2:B:150:LEU:HD22	1.73	0.71
2:B:169:VAL:O	2:B:173:VAL:HG23	1.91	0.71
2:B:278:PRO:HB2	2:B:288:TYR:O	1.87	0.71
2:B:396:ILE:HD13	2:B:418:TYR:OH	1.89	0.71
3:M:81:SER:O	3:M:82:LYS:CB	2.38	0.71
3:M:256:VAL:CG2	3:M:452:ILE:HG22	2.20	0.71
4:S:98:ILE:O	4:S:102:ILE:HG13	1.91	0.71
1:A:638:LEU:HD11	2:B:526:CYS:SG	2.31	0.71
2:B:129:ASP:O	2:B:135:ARG:HD3	1.91	0.71
2:B:137:PHE:O	2:B:140:SER:HB3	1.91	0.71
2:B:267:ASP:HB3	2:B:289:PRO:CG	2.21	0.71
2:B:563:PHE:CA	2:B:566:ALA:HB3	2.19	0.71
3:M:65:TYR:CE1	3:M:86:PRO:CG	2.74	0.71
3:M:293:PRO:HB2	3:M:294:ASP:O	1.91	0.71
4:S:105:PHE:CZ	4:S:128:LEU:CD1	2.74	0.71
1:A:147:LEU:HB3	1:A:184:ALA:HB1	1.70	0.71
1:A:400:VAL:O	1:A:403:LEU:HB2	1.90	0.71
1:A:520:GLY:HA2	1:A:558:VAL:HG22	1.67	0.71
1:A:594:PHE:CD2	2:B:474:VAL:HG22	2.25	0.71

Continued on next page...

Continued from previous page...

Atom-1	Atom-2	Interatomic distance (Å)	Clash overlap (Å)
2:B:169:VAL:HG12	2:B:173:VAL:CG2	2.20	0.71
2:B:303:LEU:HD23	2:B:325:LEU:HD23	1.73	0.71
2:B:549:LEU:HG	2:B:614:ILE:CD1	2.20	0.71
3:M:340:LEU:C	3:M:411:LEU:HB3	2.12	0.71
4:S:7:ILE:HD12	4:S:16:LEU:HD23	1.71	0.71
1:A:179:LYS:O	4:S:142:ILE:HD11	1.91	0.71
1:A:244:LEU:O	1:A:245:VAL:C	2.28	0.71
2:B:6:HIS:CG	3:M:25:PRO:HB3	2.26	0.71
2:B:47:LEU:CD2	2:B:66:ILE:HG12	1.93	0.71
2:B:50:LEU:HD23	2:B:62:ALA:HB2	1.73	0.71
2:B:537:PHE:CE2	2:B:598:LEU:C	2.63	0.71
3:M:341:SER:HA	3:M:411:LEU:H	1.56	0.71
3:M:360:LEU:HD23	3:M:362:PHE:HZ	1.55	0.71
3:M:372:ILE:CD1	3:M:428:VAL:HG22	2.21	0.71
2:B:153:ILE:C	2:B:155:LEU:N	2.44	0.70
2:B:252:LEU:HB3	2:B:302:PHE:CE2	2.25	0.70
2:B:275:ARG:HB3	2:B:291:TYR:CD2	2.25	0.70
2:B:368:ILE:HD13	2:B:401:THR:HG22	1.72	0.70
2:B:378:THR:CG2	2:B:379:LYS:N	2.54	0.70
2:B:418:TYR:CD1	2:B:419:VAL:HA	2.26	0.70
3:M:219:LEU:O	3:M:474:THR:HG21	1.91	0.70
3:M:323:MET:CE	3:M:342:LEU:HB3	2.21	0.70
3:M:373:ALA:O	3:M:418:GLU:N	2.24	0.70
4:S:50:PHE:CB	4:S:76:ILE:HA	2.20	0.70
1:A:528:ASN:O	1:A:530:ASN:N	2.24	0.70
2:B:375:LEU:O	2:B:377:TYR:N	2.23	0.70
2:B:458:MET:O	2:B:460:SER:N	2.25	0.70
2:B:530:LEU:HD23	2:B:591:MET:CB	2.21	0.70
3:M:224:VAL:CG2	3:M:479:PHE:CD1	2.74	0.70
3:M:343:ASN:HD22	3:M:343:ASN:H	1.39	0.70
3:M:443:SER:CB	3:M:447:ILE:C	2.59	0.70
4:S:135:ILE:HG22	4:S:141:VAL:CG2	2.20	0.70
1:A:103:LYS:N	4:S:163:THR:OG1	2.14	0.70
1:A:179:LYS:O	4:S:142:ILE:CD1	2.40	0.70
1:A:462:GLN:O	1:A:463:ASP:C	2.26	0.70
2:B:127:LEU:HA	2:B:161:LEU:HD11	1.72	0.70
2:B:245:GLN:NE2	2:B:309:LEU:HD11	2.05	0.70
2:B:285:GLU:O	2:B:286:ILE:HG22	1.91	0.70
2:B:549:LEU:CD1	2:B:611:ALA:HB2	2.07	0.70
3:M:215:TYR:HB3	3:M:467:TYR:CD2	2.21	0.70
3:M:240:ILE:HD11	3:M:306:LEU:HD11	1.72	0.70

Continued on next page...

Continued from previous page...

Atom-1	Atom-2	Interatomic distance (Å)	Clash overlap (Å)
1:A:129:LYS:HD2	1:A:161:ASP:HB3	1.73	0.70
1:A:222:ILE:HD12	1:A:240:LEU:HD21	1.71	0.70
2:B:212:VAL:HG13	2:B:248:LEU:HD23	1.71	0.70
2:B:267:ASP:O	2:B:269:SER:N	2.25	0.70
2:B:515:PHE:CE2	2:B:529:VAL:CG2	2.73	0.70
2:B:546:CYS:CA	2:B:607:ILE:CG1	2.54	0.70
3:M:306:LEU:O	3:M:307:SER:O	2.10	0.70
3:M:331:LEU:HD12	3:M:331:LEU:O	1.91	0.70
1:A:105:VAL:HG21	4:S:167:ILE:HA	1.73	0.70
1:A:150:LEU:HD22	1:A:162:ILE:HG12	1.73	0.70
1:A:163:ALA:CB	1:A:195:ALA:CB	2.55	0.70
1:A:403:LEU:HD21	1:A:421:PRO:O	1.91	0.70
2:B:215:TYR:CE1	2:B:233:TYR:CE1	2.77	0.70
2:B:292:GLU:CG	2:B:296:ASP:CB	2.65	0.70
2:B:563:PHE:CD2	2:B:584:SER:HB3	2.24	0.70
3:M:65:TYR:CD2	3:M:86:PRO:HB3	2.26	0.70
3:M:244:VAL:CB	3:M:300:LEU:HG	2.20	0.70
3:M:258:VAL:HG22	3:M:452:ILE:HG21	1.72	0.70
3:M:283:PHE:CE1	3:M:289:THR:OG1	2.39	0.70
3:M:378:ILE:C	3:M:379:LEU:HD12	2.11	0.70
3:M:452:ILE:HD12	3:M:452:ILE:N	2.06	0.70
4:S:69:ASN:OD1	4:S:73:ILE:O	2.09	0.70
1:A:67:LYS:CB	4:S:165:SER:C	2.59	0.70
1:A:399:ASP:C	1:A:420:ILE:H	1.95	0.70
2:B:120:ILE:HD12	2:B:142:LEU:HD22	1.72	0.70
2:B:306:LEU:HD22	2:B:321:CYS:HA	1.72	0.70
3:M:215:TYR:O	3:M:246:VAL:HG13	1.90	0.70
3:M:256:VAL:CG2	3:M:452:ILE:CG2	2.70	0.70
2:B:159:LYS:CE	2:B:191:GLU:OE1	2.39	0.70
2:B:252:LEU:HB3	2:B:302:PHE:CG	2.27	0.70
2:B:584:SER:O	2:B:585:GLY:C	2.20	0.70
3:M:217:ASP:OD1	3:M:217:ASP:O	2.10	0.70
3:M:374:TYR:H	3:M:390:ILE:HG23	1.56	0.70
1:A:129:LYS:HD2	1:A:161:ASP:CB	2.22	0.70
1:A:182:ILE:HG21	1:A:218:ALA:CA	2.22	0.70
1:A:421:PRO:HB2	1:A:424:TYR:CD1	2.27	0.70
2:B:139:LEU:HD23	2:B:172:GLU:O	1.91	0.70
2:B:217:GLU:O	2:B:218:CYS:C	2.28	0.70
2:B:278:PRO:CA	2:B:292:GLU:OE1	2.40	0.70
2:B:475:ILE:HG22	2:B:514:LEU:CD2	2.22	0.70
2:B:515:PHE:HE2	2:B:529:VAL:CG2	2.01	0.70

Continued on next page...

Continued from previous page...

Atom-1	Atom-2	Interatomic distance (Å)	Clash overlap (Å)
2:B:589:SER:HG	2:B:618:PHE:HZ	1.35	0.70
3:M:246:VAL:HB	3:M:297:PHE:CE1	2.26	0.70
4:S:16:LEU:HD12	4:S:17:VAL:N	2.07	0.70
1:A:67:LYS:CG	4:S:165:SER:OG	2.40	0.70
1:A:609:LEU:CD2	1:A:628:VAL:CG2	2.56	0.70
2:B:230:PHE:HD2	2:B:298:ASP:CB	2.03	0.70
3:M:276:VAL:CG2	3:M:299:LEU:HD12	2.21	0.70
1:A:237:SER:N	1:A:238:PRO:HD2	2.07	0.70
1:A:582:ILE:CD1	1:A:608:ARG:HA	2.22	0.70
1:A:603:VAL:HG22	2:B:610:ARG:NH2	2.07	0.70
2:B:20:ARG:CZ	2:B:35:TYR:OH	2.38	0.70
2:B:216:LYS:HB2	2:B:251:LEU:CD1	2.04	0.70
2:B:422:ALA:HB2	2:B:424:PHE:CE1	2.27	0.70
3:M:217:ASP:OD2	3:M:470:ALA:O	2.09	0.70
3:M:226:PHE:O	3:M:481:VAL:HA	1.91	0.70
3:M:350:VAL:HG13	3:M:442:GLN:HB2	1.72	0.70
4:S:5:VAL:HB	4:S:132:LEU:CD2	2.21	0.70
4:S:75:ILE:CG2	4:S:77:TYR:CZ	2.75	0.70
4:S:75:ILE:HG21	4:S:77:TYR:CZ	2.26	0.70
1:A:450:TYR:CE1	1:A:454:ILE:HD11	2.25	0.69
1:A:488:ARG:CG	1:A:522:PHE:CE2	2.71	0.69
2:B:42:ILE:O	2:B:43:ASN:CB	2.40	0.69
2:B:408:VAL:HG11	2:B:412:PHE:CE2	2.27	0.69
2:B:546:CYS:SG	2:B:607:ILE:HG13	2.32	0.69
2:B:574:ASN:O	2:B:576:GLN:O	2.10	0.69
2:B:592:TYR:CZ	2:B:619:ASP:OD1	2.44	0.69
2:B:599:ALA:O	2:B:601:TYR:N	2.25	0.69
3:M:9:ASP:CA	3:M:75:TRP:HD1	2.04	0.69
1:A:107:TYR:HB3	1:A:149:GLY:HA3	1.74	0.69
1:A:211:ASP:CG	4:S:148:ARG:NE	2.46	0.69
1:A:217:ALA:HB1	4:S:142:ILE:HD12	0.73	0.69
2:B:36:THR:O	2:B:40:GLN:HB2	1.93	0.69
2:B:120:ILE:HG22	2:B:153:ILE:HB	1.74	0.69
3:M:53:HIS:HB3	3:M:65:TYR:CE1	2.28	0.69
3:M:323:MET:SD	3:M:342:LEU:HA	2.32	0.69
1:A:252:ILE:CG1	4:S:144:THR:OG1	2.41	0.69
1:A:282:MET:HE3	1:A:294:SER:HB2	1.75	0.69
1:A:398:GLU:O	1:A:419:ILE:C	2.30	0.69
1:A:421:PRO:HG2	1:A:424:TYR:CZ	2.27	0.69
2:B:68:SER:O	2:B:71:ALA:N	2.24	0.69
2:B:70:MET:HE1	2:B:107:ARG:HB2	0.70	0.69

Continued on next page...

Continued from previous page...

Atom-1	Atom-2	Interatomic distance (Å)	Clash overlap (Å)
2:B:319:LEU:HD13	2:B:358:MET:HG2	1.69	0.69
2:B:563:PHE:CE2	2:B:584:SER:HA	2.25	0.69
2:B:565:GLN:C	2:B:574:ASN:ND2	2.38	0.69
1:A:215:VAL:HG21	1:A:243:ILE:CD1	2.15	0.69
2:B:155:LEU:HD13	2:B:156:HIS:N	2.07	0.69
2:B:435:SER:O	2:B:437:SER:N	2.26	0.69
2:B:508:ARG:HB3	2:B:544:THR:HG23	1.68	0.69
2:B:550:VAL:CG2	2:B:610:ARG:HD2	2.22	0.69
3:M:101:LEU:HD11	3:M:106:LYS:O	1.91	0.69
3:M:231:SER:O	3:M:326:HIS:CE1	2.46	0.69
1:A:140:VAL:HG13	1:A:176:TYR:CB	2.21	0.69
1:A:147:LEU:HB3	1:A:184:ALA:HB3	1.67	0.69
1:A:219:VAL:C	4:S:140:MET:SD	2.49	0.69
1:A:225:LEU:HB3	1:A:233:PHE:CZ	2.26	0.69
2:B:67:ILE:CD1	2:B:103:LEU:CB	2.71	0.69
2:B:132:SER:O	2:B:133:GLU:C	2.24	0.69
2:B:162:VAL:HG23	2:B:195:ILE:HG22	1.54	0.69
2:B:174:ALA:HB1	2:B:211:ALA:CB	2.22	0.69
2:B:178:ILE:HA	2:B:218:CYS:CB	2.14	0.69
2:B:215:TYR:HD2	2:B:226:LEU:CD1	2.04	0.69
2:B:247:TYR:CZ	3:M:91:THR:CG2	2.68	0.69
2:B:306:LEU:HD12	2:B:325:LEU:HD23	1.73	0.69
2:B:353:GLN:CG	3:M:47:SER:CB	2.70	0.69
2:B:493:LEU:CD2	2:B:511:ILE:HG23	2.22	0.69
3:M:220:GLU:CD	3:M:442:GLN:HB3	2.12	0.69
4:S:14:PRO:HA	4:S:36:TYR:HH	1.56	0.69
1:A:250:ASN:OD1	1:A:285:THR:CB	2.38	0.69
1:A:408:ILE:N	4:S:64:ASN:HB2	2.07	0.69
2:B:219:TYR:CD2	2:B:226:LEU:CB	2.64	0.69
2:B:322:CYS:HA	2:B:325:LEU:HD12	1.73	0.69
2:B:500:GLN:CD	2:B:503:LEU:HD21	2.12	0.69
2:B:613:MET:HE3	2:B:617:LEU:HD11	1.74	0.69
1:A:97:SER:C	1:A:98:ASN:O	2.05	0.69
1:A:323:CYS:HG	1:A:338:PHE:HE1	0.74	0.69
2:B:64:LYS:HG3	2:B:100:LEU:HD11	1.73	0.69
2:B:206:LYS:O	2:B:210:CYS:SG	2.42	0.69
2:B:433:VAL:CG1	2:B:474:VAL:HB	2.22	0.69
2:B:569:THR:HG22	2:B:569:THR:O	1.92	0.69
4:S:9:ASN:ND2	4:S:13:GLN:CB	2.55	0.69
1:A:104:ARG:HE	4:S:160:ALA:CB	2.06	0.69
1:A:140:VAL:HG11	1:A:176:TYR:HB2	1.64	0.69

Continued on next page...

Continued from previous page...

Atom-1	Atom-2	Interatomic distance (Å)	Clash overlap (Å)
1:A:186:PHE:CE2	1:A:187:LYS:HD3	2.28	0.69
1:A:206:LYS:HE2	1:A:206:LYS:HA	1.74	0.69
1:A:207:LEU:HD21	1:A:239:LEU:HB2	1.75	0.69
1:A:288:THR:HA	1:A:291:ILE:HD13	1.73	0.69
1:A:477:PHE:CE2	1:A:481:MET:SD	2.86	0.69
1:A:594:PHE:CD1	2:B:434:LYS:HE2	2.27	0.69
2:B:37:TYR:CE2	2:B:38:TYR:CD1	2.77	0.69
2:B:121:ASN:O	2:B:124:GLN:HB3	1.93	0.69
2:B:177:ILE:CB	2:B:196:LEU:HD21	2.18	0.69
2:B:178:ILE:HG12	2:B:214:ALA:HB1	1.74	0.69
2:B:182:ARG:NE	2:B:217:GLU:OE1	2.26	0.69
2:B:230:PHE:CE2	2:B:234:CYS:SG	2.86	0.69
2:B:236:ILE:CG2	2:B:240:LEU:HD11	2.21	0.69
2:B:508:ARG:CB	2:B:544:THR:HG21	2.22	0.69
2:B:513:TRP:CB	2:B:551:LEU:CD2	2.67	0.69
2:B:519:ALA:O	2:B:523:PHE:CA	2.41	0.69
2:B:525:ILE:O	2:B:527:PRO:HD2	1.93	0.69
3:M:16:PHE:HZ	3:M:18:TYR:HB2	1.52	0.69
3:M:214:LEU:O	3:M:467:TYR:CA	2.41	0.69
3:M:215:TYR:O	3:M:470:ALA:HB2	1.92	0.69
3:M:224:VAL:O	3:M:479:PHE:CB	2.40	0.69
3:M:233:LEU:HD21	3:M:325:LEU:H	1.56	0.69
3:M:235:LEU:HD11	3:M:306:LEU:HB3	1.71	0.69
3:M:246:VAL:HA	3:M:470:ALA:HB1	1.74	0.69
1:A:189:PHE:CB	1:A:225:LEU:CD2	2.71	0.69
1:A:211:ASP:CG	4:S:148:ARG:NH1	2.46	0.69
1:A:252:ILE:HG13	4:S:145:ASN:N	2.07	0.69
1:A:272:ALA:O	1:A:276:PRO:HD3	1.93	0.69
1:A:298:ILE:CD1	1:A:311:THR:CG2	2.69	0.69
1:A:629:LEU:CD1	2:B:610:ARG:HH11	1.74	0.69
2:B:116:THR:O	2:B:120:ILE:HG12	1.93	0.69
2:B:215:TYR:CZ	2:B:229:HIS:CB	2.76	0.69
2:B:267:ASP:O	2:B:276:SER:HB2	1.93	0.69
2:B:275:ARG:HG3	2:B:291:TYR:CE2	2.28	0.69
2:B:357:GLU:HG2	2:B:361:GLN:NE2	2.06	0.69
2:B:374:PHE:HZ	2:B:381:PHE:CD1	2.09	0.69
3:M:55:MET:O	3:M:56:VAL:C	2.17	0.69
3:M:260:LEU:HD23	3:M:449:VAL:HG22	0.73	0.69
3:M:374:TYR:HB2	3:M:417:TYR:CD2	2.28	0.69
3:M:378:ILE:HB	3:M:413:GLY:HA2	1.73	0.69
4:S:7:ILE:CG2	4:S:121:LEU:CD2	2.70	0.69

Continued on next page...

Continued from previous page...

Atom-1	Atom-2	Interatomic distance (Å)	Clash overlap (Å)
1:A:231:GLN:HB2	1:A:232:PRO:HD3	1.74	0.69
1:A:322:PHE:HD2	1:A:330:LEU:HD21	1.53	0.69
1:A:332:TYR:CZ	1:A:336:ILE:CD1	2.75	0.69
1:A:482:ILE:HG12	1:A:517:TRP:CZ3	2.27	0.69
1:A:582:ILE:HG12	1:A:607:LEU:HB2	1.74	0.69
2:B:87:VAL:O	2:B:90:ILE:HG22	1.93	0.69
2:B:171:GLY:HA3	2:B:207:VAL:HA	1.75	0.69
2:B:332:LEU:HG	2:B:332:LEU:O	1.93	0.69
2:B:523:PHE:HB2	2:B:559:ASP:OD2	1.93	0.69
3:M:472:TYR:CD1	3:M:472:TYR:N	2.58	0.69
4:S:15:ARG:HH21	4:S:118:GLU:CD	1.96	0.69
4:S:53:THR:OG1	4:S:68:VAL:O	2.10	0.69
4:S:75:ILE:HG21	4:S:77:TYR:OH	1.92	0.69
1:A:64:LEU:CG	1:A:102:GLN:NE2	2.44	0.68
1:A:101:GLN:CD	4:S:167:ILE:HG21	2.12	0.68
1:A:186:PHE:HD2	1:A:187:LYS:N	1.91	0.68
1:A:211:ASP:OD2	4:S:148:ARG:HD3	1.93	0.68
1:A:223:CYS:HB2	1:A:259:LEU:CG	2.19	0.68
1:A:488:ARG:HG2	1:A:522:PHE:CD2	2.28	0.68
1:A:552:ILE:HG22	1:A:603:VAL:HG21	1.74	0.68
2:B:8:ILE:O	2:B:12:LEU:HD12	1.92	0.68
2:B:81:LEU:C	2:B:83:PHE:N	2.33	0.68
2:B:135:ARG:CZ	2:B:164:ASP:HB2	2.24	0.68
2:B:267:ASP:O	2:B:268:LYS:C	2.29	0.68
2:B:306:LEU:HB2	2:B:325:LEU:HD21	1.74	0.68
2:B:360:LEU:HD11	2:B:391:ALA:CB	2.22	0.68
2:B:542:PRO:O	2:B:545:ARG:N	2.26	0.68
2:B:592:TYR:HD2	2:B:618:PHE:CE2	2.09	0.68
3:M:339:GLU:HG3	3:M:412:ARG:HD3	1.75	0.68
3:M:410:VAL:HG13	3:M:412:ARG:NH1	2.08	0.68
4:S:87:PHE:CE1	4:S:102:ILE:HG12	2.27	0.68
1:A:244:LEU:HB2	1:A:256:LEU:HD13	1.74	0.68
1:A:566:PHE:C	1:A:568:GLU:H	1.96	0.68
1:A:630:PRO:CG	2:B:614:ILE:CA	2.60	0.68
2:B:48:VAL:O	2:B:51:LEU:N	2.22	0.68
2:B:307:ASN:OD1	2:B:339:PHE:HD2	1.76	0.68
2:B:340:ILE:CG1	2:B:373:LEU:CD2	2.70	0.68
2:B:352:ASN:CB	3:M:49:ASP:CB	2.48	0.68
3:M:245:ASP:OD1	3:M:297:PHE:C	2.32	0.68
1:A:636:TYR:H	2:B:554:LYS:NZ	1.90	0.68
2:B:542:PRO:C	2:B:602:ASP:OD2	2.31	0.68

Continued on next page...

Continued from previous page...

Atom-1	Atom-2	Interatomic distance (Å)	Clash overlap (Å)
3:M:66:PHE:HE2	3:M:77:LEU:O	1.77	0.68
3:M:67:SER:HG	3:M:90:PHE:HD1	0.70	0.68
3:M:101:LEU:CD1	3:M:109:LEU:HD13	2.22	0.68
3:M:222:PHE:CD1	3:M:439:TYR:CE1	2.81	0.68
3:M:244:VAL:N	3:M:300:LEU:O	2.27	0.68
3:M:260:LEU:HA	3:M:448:TYR:O	1.93	0.68
4:S:85:PHE:CZ	4:S:109:LEU:HD23	2.28	0.68
4:S:111:ARG:CB	4:S:150:VAL:HG22	2.22	0.68
1:A:96:SER:HA	1:A:127:LEU:HG	1.76	0.68
1:A:170:LEU:HB3	1:A:206:LYS:CG	2.23	0.68
1:A:566:PHE:CD1	1:A:570:LYS:HA	2.28	0.68
2:B:47:LEU:HD22	2:B:66:ILE:CB	2.15	0.68
2:B:176:ALA:C	2:B:178:ILE:N	2.44	0.68
2:B:177:ILE:HD12	2:B:196:LEU:CD2	2.24	0.68
2:B:219:TYR:HB2	2:B:226:LEU:HD22	1.73	0.68
2:B:352:ASN:HB2	3:M:49:ASP:CG	2.12	0.68
3:M:223:HIS:CD2	3:M:478:ASN:HA	2.27	0.68
3:M:242:GLY:HA2	3:M:474:THR:CG2	2.22	0.68
3:M:319:SER:O	3:M:343:ASN:O	2.12	0.68
3:M:443:SER:HB3	3:M:447:ILE:CA	2.23	0.68
4:S:50:PHE:HA	4:S:77:TYR:HD1	1.58	0.68
1:A:79:MET:CG	1:A:112:GLN:OE1	2.40	0.68
1:A:277:LYS:O	1:A:277:LYS:HG3	1.94	0.68
1:A:303:MET:HG2	1:A:308:ASP:OD2	1.92	0.68
1:A:441:TYR:HB3	1:A:444:VAL:HG23	1.75	0.68
1:A:509:PRO:CB	1:A:547:VAL:CG2	2.67	0.68
1:A:589:SER:CB	1:A:601:VAL:CG2	2.70	0.68
2:B:2:VAL:HG21	3:M:58:ARG:HD2	1.75	0.68
2:B:102:HIS:ND1	2:B:137:PHE:CB	2.52	0.68
2:B:563:PHE:O	2:B:566:ALA:CB	2.42	0.68
3:M:65:TYR:CG	3:M:86:PRO:HA	2.29	0.68
3:M:224:VAL:HG22	3:M:306:LEU:HD12	1.73	0.68
3:M:225:VAL:HB	3:M:237:THR:OG1	1.93	0.68
1:A:105:VAL:HG21	4:S:167:ILE:CB	2.23	0.68
1:A:178:ARG:HB3	1:A:214:VAL:CG1	2.23	0.68
1:A:189:PHE:CD2	1:A:225:LEU:CD1	2.76	0.68
1:A:192:TYR:CE2	1:A:194:GLU:HB2	2.28	0.68
1:A:271:ARG:NH1	1:A:302:ASN:O	2.26	0.68
1:A:461:CYS:HB2	1:A:469:LEU:CD2	2.23	0.68
2:B:24:ALA:CB	2:B:35:TYR:CD2	2.68	0.68
2:B:146:LYS:O	2:B:147:MET:CG	2.42	0.68

Continued on next page...

Continued from previous page...

Atom-1	Atom-2	Interatomic distance (Å)	Clash overlap (Å)
2:B:412:PHE:CE2	2:B:446:TRP:HB3	2.22	0.68
2:B:422:ALA:CB	2:B:424:PHE:CD1	2.54	0.68
2:B:512:VAL:HG21	2:B:548:ILE:CG1	2.22	0.68
3:M:222:PHE:HB2	3:M:479:PHE:CE1	2.29	0.68
4:S:105:PHE:HZ	4:S:128:LEU:HD11	1.58	0.68
1:A:214:VAL:HG23	4:S:148:ARG:NH2	2.08	0.68
1:A:240:LEU:O	1:A:241:TYR:O	2.11	0.68
1:A:244:LEU:CD1	1:A:281:LEU:HD11	2.24	0.68
1:A:482:ILE:HG12	1:A:517:TRP:HH2	1.58	0.68
2:B:37:TYR:O	2:B:38:TYR:C	2.31	0.68
2:B:90:ILE:HG13	2:B:98:LYS:HD3	1.74	0.68
2:B:247:TYR:OH	3:M:91:THR:HG21	1.94	0.68
2:B:334:MET:HE2	2:B:334:MET:CA	2.17	0.68
2:B:361:GLN:HG2	2:B:394:TRP:CZ2	2.29	0.68
2:B:433:VAL:O	2:B:474:VAL:HG11	1.93	0.68
2:B:589:SER:HA	2:B:592:TYR:HD2	1.58	0.68
3:M:306:LEU:HD22	3:M:317:MET:CE	2.23	0.68
3:M:317:MET:CB	3:M:321:GLY:C	2.57	0.68
1:A:605:GLU:CG	1:A:632:PHE:CE2	2.69	0.68
2:B:37:TYR:CE2	2:B:46:GLN:NE2	2.48	0.68
2:B:47:LEU:CD1	2:B:66:ILE:HA	2.23	0.68
2:B:216:LYS:CG	2:B:251:LEU:CD1	2.47	0.68
3:M:65:TYR:CD2	3:M:86:PRO:CA	2.76	0.68
3:M:244:VAL:CG1	3:M:472:TYR:CZ	2.75	0.68
3:M:246:VAL:HB	3:M:297:PHE:HZ	1.51	0.68
3:M:339:GLU:CD	3:M:412:ARG:CD	2.60	0.68
3:M:360:LEU:CD1	3:M:433:VAL:HB	2.23	0.68
4:S:9:ASN:ND2	4:S:118:GLU:OE1	2.27	0.68
1:A:102:GLN:N	4:S:167:ILE:CG1	2.55	0.68
1:A:558:VAL:O	1:A:561:ASN:N	2.15	0.68
2:B:20:ARG:CG	2:B:21:GLU:H	2.06	0.68
2:B:42:ILE:O	2:B:46:GLN:CD	2.31	0.68
2:B:140:SER:O	2:B:143:SER:N	2.21	0.68
2:B:523:PHE:CD1	2:B:559:ASP:CG	2.67	0.68
2:B:549:LEU:HD13	2:B:611:ALA:HB2	1.71	0.68
3:M:10:THR:OG1	3:M:48:ASP:OD1	2.09	0.68
3:M:101:LEU:CD2	3:M:106:LYS:HG3	2.24	0.68
3:M:300:LEU:HD11	3:M:447:ILE:HD12	1.76	0.68
4:S:8:PHE:CB	4:S:36:TYR:OH	2.41	0.68
1:A:100:LEU:CD2	1:A:138:ASN:HB3	2.23	0.68
1:A:189:PHE:CE2	1:A:225:LEU:HD11	2.28	0.68

Continued on next page...

Continued from previous page...

Atom-1	Atom-2	Interatomic distance (Å)	Clash overlap (Å)
1:A:401:VAL:CG1	1:A:419:ILE:HD12	2.23	0.68
1:A:594:PHE:CD2	2:B:474:VAL:CG2	2.77	0.68
2:B:144:ASP:HA	2:B:179:LYS:HD3	1.72	0.68
2:B:380:LYS:HZ1	3:M:236:LEU:CD1	2.07	0.68
1:A:102:GLN:HB3	4:S:166:LYS:CG	2.23	0.67
1:A:212:ILE:HG22	1:A:247:ILE:HD13	1.75	0.67
1:A:635:ALA:C	2:B:517:GLU:HA	2.15	0.67
2:B:124:GLN:CA	2:B:157:THR:OG1	2.31	0.67
2:B:148:SER:O	2:B:183:ALA:CB	2.36	0.67
3:M:223:HIS:HB3	3:M:478:ASN:CA	2.24	0.67
3:M:354:ASP:O	3:M:438:SER:O	2.12	0.67
3:M:371:GLU:HB3	3:M:424:PHE:HD1	1.60	0.67
4:S:14:PRO:O	4:S:15:ARG:CG	2.43	0.67
4:S:130:SER:CB	4:S:156:LEU:CD1	2.65	0.67
1:A:186:PHE:CD1	1:A:224:GLU:HB2	2.27	0.67
1:A:186:PHE:CZ	4:S:138:GLY:N	2.29	0.67
1:A:190:LEU:CD1	1:A:228:LYS:HE3	2.23	0.67
1:A:318:ARG:O	1:A:322:PHE:HD1	1.76	0.67
1:A:384:LEU:CG	1:A:441:TYR:HE2	2.06	0.67
1:A:446:ASP:O	1:A:448:GLU:O	2.11	0.67
2:B:67:ILE:HD13	2:B:103:LEU:HB2	1.76	0.67
2:B:204:ASP:O	2:B:207:VAL:HB	1.94	0.67
2:B:293:VAL:O	2:B:299:LEU:CG	2.42	0.67
2:B:493:LEU:HD21	2:B:511:ILE:HG12	1.74	0.67
3:M:269:ILE:O	3:M:302:TYR:CD1	2.47	0.67
3:M:281:GLY:O	3:M:282:VAL:CB	2.42	0.67
3:M:304:VAL:HG21	3:M:309:GLN:NE2	2.09	0.67
4:S:94:SER:O	4:S:98:ILE:HG12	1.94	0.67
1:A:70:ALA:O	1:A:74:LEU:HG	1.94	0.67
1:A:137:ASN:O	1:A:139:ASP:N	2.26	0.67
1:A:292:TYR:CD1	1:A:292:TYR:O	2.48	0.67
1:A:409:VAL:O	1:A:409:VAL:HG13	1.93	0.67
1:A:572:PHE:O	1:A:575:LYS:HB3	1.93	0.67
1:A:585:PHE:CE2	1:A:603:VAL:CG1	2.77	0.67
2:B:94:ASP:O	2:B:134:LEU:HD21	1.94	0.67
3:M:66:PHE:CB	3:M:77:LEU:CD2	2.64	0.67
3:M:215:TYR:HB2	3:M:468:LYS:N	2.09	0.67
3:M:222:PHE:CA	3:M:479:PHE:CZ	2.76	0.67
4:S:9:ASN:CG	4:S:13:GLN:HB2	2.14	0.67
4:S:47:GLN:OE1	4:S:78:LYS:C	2.32	0.67
1:A:189:PHE:HD2	1:A:225:LEU:CD1	2.06	0.67

Continued on next page...

Continued from previous page...

Atom-1	Atom-2	Interatomic distance (Å)	Clash overlap (Å)
1:A:249:ASN:HD21	4:S:146:VAL:HG23	1.58	0.67
1:A:282:MET:O	1:A:284:SER:N	2.28	0.67
1:A:429:VAL:HG11	1:A:469:LEU:HD11	1.77	0.67
1:A:465:SER:N	2:B:1:MET:HG2	2.09	0.67
2:B:92:THR:O	2:B:134:LEU:HD13	1.93	0.67
2:B:173:VAL:HB	2:B:199:LEU:HD13	1.75	0.67
2:B:215:TYR:HE2	2:B:229:HIS:ND1	1.90	0.67
2:B:216:LYS:HD3	2:B:251:LEU:HB2	1.77	0.67
3:M:96:ILE:HG23	3:M:125:PHE:CE1	2.30	0.67
3:M:245:ASP:HA	3:M:297:PHE:O	1.94	0.67
1:A:448:GLU:CB	1:A:487:MET:SD	2.78	0.67
2:B:226:LEU:O	2:B:229:HIS:HB2	1.95	0.67
2:B:260:LEU:CB	2:B:291:TYR:CZ	2.73	0.67
2:B:309:LEU:CD1	2:B:317:VAL:HG11	2.24	0.67
2:B:380:LYS:NZ	3:M:236:LEU:CD1	2.58	0.67
3:M:253:ASN:HA	3:M:292:PRO:HG2	1.75	0.67
1:A:99:LYS:HZ2	4:S:164:ASP:CB	2.05	0.67
1:A:103:LYS:NZ	1:A:131:ASP:HB2	2.08	0.67
1:A:105:VAL:HG23	4:S:167:ILE:HD12	0.68	0.67
1:A:189:PHE:CB	1:A:225:LEU:HD21	2.24	0.67
1:A:212:ILE:O	1:A:213:SER:C	2.21	0.67
1:A:392:MET:SD	1:A:457:LEU:HD21	2.34	0.67
2:B:4:SER:O	2:B:8:ILE:CD1	2.43	0.67
2:B:123:LEU:HB3	2:B:142:LEU:HD11	1.76	0.67
3:M:375:LYS:HG3	3:M:418:GLU:OE1	1.95	0.67
1:A:100:LEU:H	4:S:162:SER:CA	2.00	0.67
1:A:104:ARG:CD	1:A:145:ILE:HG13	2.25	0.67
1:A:140:VAL:CA	1:A:177:ILE:HD11	2.22	0.67
1:A:145:ILE:HD11	4:S:156:LEU:HD13	1.77	0.67
1:A:594:PHE:HB2	2:B:473:ASN:HB2	1.71	0.67
2:B:47:LEU:CG	2:B:66:ILE:HG12	2.25	0.67
3:M:327:PHE:CE1	3:M:336:ASP:CB	2.69	0.67
1:A:125:THR:O	1:A:127:LEU:N	2.27	0.67
1:A:320:HIS:CB	1:A:352:PHE:HE2	2.05	0.67
1:A:401:VAL:HB	1:A:419:ILE:CG1	2.24	0.67
1:A:552:ILE:HD13	1:A:600:SER:CB	2.25	0.67
1:A:625:LEU:CG	2:B:613:MET:SD	2.79	0.67
2:B:175:LEU:CA	2:B:214:ALA:HB2	2.19	0.67
2:B:208:ILE:HG21	2:B:236:ILE:HG21	1.77	0.67
2:B:315:PRO:HA	2:B:318:ILE:HD12	1.76	0.67
2:B:613:MET:CE	2:B:617:LEU:HD11	2.25	0.67

Continued on next page...

Continued from previous page...

Atom-1	Atom-2	Interatomic distance (Å)	Clash overlap (Å)
3:M:2:TYR:CE1	3:M:64:LYS:NZ	2.57	0.67
3:M:2:TYR:CA	3:M:81:SER:HB2	2.25	0.67
1:A:100:LEU:O	1:A:101:GLN:O	2.13	0.67
1:A:264:SER:HB3	1:A:271:ARG:HG2	1.75	0.67
2:B:37:TYR:HD2	2:B:38:TYR:CE1	2.01	0.67
2:B:120:ILE:HD12	2:B:142:LEU:CD2	2.24	0.67
2:B:139:LEU:HD23	2:B:172:GLU:C	2.15	0.67
2:B:560:ILE:HG22	2:B:561:ASP:N	2.10	0.67
3:M:7:ILE:C	3:M:75:TRP:O	2.32	0.67
4:S:53:THR:CB	4:S:69:ASN:CB	2.73	0.67
1:A:109:ALA:O	1:A:112:GLN:N	2.27	0.67
1:A:140:VAL:CG2	1:A:177:ILE:CG1	2.68	0.67
1:A:182:ILE:CG2	1:A:221:VAL:HG23	2.17	0.67
1:A:370:LYS:O	1:A:374:LEU:CD1	2.42	0.67
1:A:421:PRO:CG	1:A:424:TYR:CD1	2.76	0.67
1:A:478:ARG:HD3	1:A:514:GLU:OE1	1.95	0.67
1:A:556:VAL:CG2	1:A:603:VAL:CG1	2.73	0.67
1:A:637:GLU:CB	2:B:551:LEU:HD22	2.25	0.67
2:B:219:TYR:CA	2:B:223:LEU:HD23	2.24	0.67
2:B:340:ILE:CD1	2:B:366:LEU:CB	2.73	0.67
3:M:54:SER:HB2	3:M:66:PHE:CD1	2.30	0.67
3:M:222:PHE:CD2	3:M:439:TYR:CE1	2.82	0.67
3:M:233:LEU:CD2	3:M:323:MET:O	2.42	0.67
3:M:338:PHE:CD2	3:M:415:ILE:CD1	2.78	0.67
1:A:186:PHE:CD2	1:A:187:LYS:N	2.63	0.66
2:B:20:ARG:HD2	2:B:21:GLU:N	2.09	0.66
2:B:80:GLN:CG	2:B:115:LEU:HD21	2.25	0.66
2:B:195:ILE:C	2:B:197:LYS:N	2.43	0.66
2:B:277:CYS:HA	2:B:292:GLU:CG	2.25	0.66
2:B:303:LEU:HD11	2:B:333:GLN:HE21	1.58	0.66
2:B:347:VAL:HG22	2:B:359:LEU:CB	2.13	0.66
2:B:381:PHE:O	2:B:395:LYS:HD2	1.95	0.66
2:B:537:PHE:CE1	2:B:545:ARG:CG	2.75	0.66
3:M:3:LEU:HD12	3:M:3:LEU:N	2.10	0.66
3:M:215:TYR:HB3	3:M:470:ALA:H	1.60	0.66
4:S:9:ASN:HD21	4:S:13:GLN:CB	2.09	0.66
4:S:99:LEU:O	4:S:102:ILE:HB	1.95	0.66
1:A:104:ARG:HG3	1:A:145:ILE:CG2	2.25	0.66
1:A:214:VAL:HG23	4:S:148:ARG:CZ	2.26	0.66
1:A:557:LYS:HE3	2:B:606:ASP:HB3	1.78	0.66
1:A:566:PHE:HZ	1:A:618:THR:C	1.99	0.66

Continued on next page...

Continued from previous page...

Atom-1	Atom-2	Interatomic distance (Å)	Clash overlap (Å)
1:A:605:GLU:OE2	1:A:608:ARG:NH2	2.25	0.66
2:B:13:ASP:OD2	3:M:17:GLN:OE1	2.14	0.66
2:B:120:ILE:HD13	2:B:142:LEU:CD2	2.22	0.66
2:B:175:LEU:CD1	2:B:210:CYS:HB3	2.25	0.66
2:B:249:ILE:HG21	2:B:320:SER:HB3	1.76	0.66
2:B:363:ILE:CG2	2:B:398:ILE:CD1	2.73	0.66
2:B:364:HIS:O	2:B:367:SER:N	2.28	0.66
2:B:375:LEU:HD23	2:B:402:LEU:HD22	1.78	0.66
3:M:269:ILE:C	3:M:302:TYR:CE1	2.68	0.66
3:M:324:SER:O	3:M:339:GLU:O	2.13	0.66
3:M:340:LEU:N	3:M:411:LEU:O	2.28	0.66
3:M:443:SER:CB	3:M:447:ILE:CG1	2.37	0.66
4:S:14:PRO:O	4:S:15:ARG:HG2	1.95	0.66
1:A:429:VAL:CG1	1:A:469:LEU:CD1	2.69	0.66
2:B:343:LEU:CD1	2:B:359:LEU:HD12	2.18	0.66
2:B:363:ILE:HG21	2:B:398:ILE:HD11	1.77	0.66
2:B:534:ILE:HD13	2:B:594:ALA:CB	2.20	0.66
3:M:410:VAL:CG1	3:M:412:ARG:HH11	2.08	0.66
3:M:437:TYR:CD1	3:M:479:PHE:CZ	2.83	0.66
1:A:103:LYS:O	1:A:107:TYR:HD1	1.74	0.66
1:A:219:VAL:O	4:S:140:MET:SD	2.52	0.66
1:A:244:LEU:CD1	1:A:256:LEU:HD12	2.25	0.66
1:A:625:LEU:HG	2:B:613:MET:SD	2.35	0.66
2:B:77:ILE:O	2:B:79:VAL:CA	2.43	0.66
2:B:124:GLN:HB2	2:B:153:ILE:CG2	2.24	0.66
2:B:127:LEU:HD12	2:B:157:THR:OG1	1.96	0.66
2:B:472:VAL:O	2:B:473:ASN:C	2.33	0.66
2:B:512:VAL:CG1	2:B:548:ILE:HA	2.10	0.66
3:M:396:GLN:HG2	3:M:398:ILE:HD11	1.77	0.66
4:S:85:PHE:HE2	4:S:109:LEU:CD2	2.08	0.66
1:A:64:LEU:HB3	1:A:102:GLN:NE2	2.10	0.66
1:A:370:LYS:HE2	1:A:370:LYS:HA	1.76	0.66
1:A:581:LEU:HB3	1:A:607:LEU:HD13	1.77	0.66
1:A:631:SER:O	2:B:554:LYS:CG	2.35	0.66
2:B:85:ASP:O	2:B:89:ASN:ND2	2.28	0.66
2:B:173:VAL:HB	2:B:199:LEU:HD11	1.76	0.66
2:B:174:ALA:HB3	2:B:211:ALA:CB	2.22	0.66
2:B:204:ASP:HB3	2:B:207:VAL:CG2	2.25	0.66
2:B:208:ILE:HG21	2:B:236:ILE:CG2	2.25	0.66
2:B:344:VAL:HG22	2:B:381:PHE:HZ	1.61	0.66
2:B:553:ALA:HA	2:B:614:ILE:HG23	1.78	0.66

Continued on next page...

Continued from previous page...

Atom-1	Atom-2	Interatomic distance (Å)	Clash overlap (Å)
3:M:8:THR:HA	3:M:13:LYS:O	1.95	0.66
3:M:220:GLU:HB2	3:M:222:PHE:HE1	1.60	0.66
4:S:130:SER:HB2	4:S:156:LEU:HD13	1.76	0.66
1:A:516:ILE:CG1	1:A:551:LEU:HD13	2.26	0.66
1:A:557:LYS:CD	2:B:606:ASP:N	2.56	0.66
1:A:629:LEU:HG	2:B:610:ARG:NH1	2.05	0.66
2:B:90:ILE:CD1	2:B:123:LEU:HD21	2.24	0.66
2:B:94:ASP:C	2:B:134:LEU:HD21	2.16	0.66
2:B:139:LEU:CD2	2:B:173:VAL:O	2.32	0.66
2:B:315:PRO:HG3	2:B:352:ASN:OD1	1.94	0.66
3:M:437:TYR:N	3:M:437:TYR:HD1	1.91	0.66
4:S:110:ASP:O	4:S:113:PHE:O	2.14	0.66
1:A:221:VAL:HG23	4:S:142:ILE:HG21	1.77	0.66
1:A:241:TYR:CD2	1:A:242:GLU:N	2.63	0.66
1:A:399:ASP:O	1:A:420:ILE:CB	2.43	0.66
1:A:454:ILE:HG23	1:A:473:ILE:CG2	2.24	0.66
1:A:626:SER:C	2:B:617:LEU:CG	2.35	0.66
2:B:123:LEU:CD2	2:B:138:ALA:O	2.42	0.66
2:B:230:PHE:CD2	2:B:298:ASP:O	2.48	0.66
2:B:232:ARG:CG	2:B:236:ILE:HD11	2.26	0.66
2:B:234:CYS:O	2:B:237:ILE:CG2	2.38	0.66
2:B:437:SER:C	2:B:478:LEU:HD21	2.15	0.66
3:M:51:LEU:N	3:M:51:LEU:HD12	2.10	0.66
3:M:379:LEU:HD23	3:M:397:TRP:HE1	1.58	0.66
1:A:92:LEU:HD11	1:A:120:ILE:CA	2.13	0.66
1:A:401:VAL:CG2	1:A:419:ILE:HB	2.26	0.66
1:A:509:PRO:HB3	1:A:547:VAL:HG21	1.75	0.66
1:A:581:LEU:CD2	1:A:607:LEU:HD11	2.26	0.66
2:B:353:GLN:HG2	3:M:48:ASP:CA	2.26	0.66
2:B:375:LEU:O	2:B:376:PRO:C	2.33	0.66
2:B:403:ILE:HA	2:B:411:ILE:HD12	1.77	0.66
2:B:537:PHE:CG	2:B:598:LEU:CB	2.74	0.66
2:B:556:LEU:CA	2:B:588:ILE:CD1	2.21	0.66
2:B:563:PHE:HE2	2:B:584:SER:CA	2.06	0.66
3:M:259:LYS:O	3:M:449:VAL:HG13	1.96	0.66
4:S:48:SER:HA	4:S:77:TYR:CB	2.25	0.66
4:S:54:PRO:HB2	4:S:57:LEU:CD1	2.26	0.66
4:S:137:GLN:O	4:S:140:MET:HB3	1.96	0.66
1:A:92:LEU:O	1:A:95:MET:N	2.27	0.66
1:A:100:LEU:O	1:A:101:GLN:C	2.16	0.66
1:A:102:GLN:CA	4:S:166:LYS:HB2	2.26	0.66

Continued on next page...

Continued from previous page...

Atom-1	Atom-2	Interatomic distance (Å)	Clash overlap (Å)
1:A:150:LEU:HD22	1:A:162:ILE:CG1	2.26	0.66
1:A:288:THR:HB	1:A:322:PHE:HZ	1.59	0.66
1:A:318:ARG:O	1:A:322:PHE:CD1	2.49	0.66
1:A:625:LEU:O	1:A:627:GLU:N	2.28	0.66
2:B:215:TYR:HD2	2:B:226:LEU:HD13	1.60	0.66
2:B:400:SER:HB3	2:B:439:CYS:SG	2.35	0.66
3:M:215:TYR:HD2	3:M:469:GLY:H	1.29	0.66
3:M:265:ASN:HB3	3:M:309:GLN:HG3	1.77	0.66
1:A:92:LEU:HD22	1:A:123:LEU:HB2	1.78	0.66
1:A:215:VAL:O	1:A:219:VAL:HG23	1.95	0.66
2:B:141:ALA:O	2:B:142:LEU:C	2.31	0.66
2:B:390:VAL:HG12	2:B:394:TRP:CD1	2.31	0.66
2:B:418:TYR:CD1	2:B:419:VAL:CA	2.79	0.66
2:B:497:LEU:HD21	2:B:533:LEU:HD22	1.77	0.66
2:B:592:TYR:CE2	2:B:618:PHE:HD2	2.13	0.66
3:M:2:TYR:H	3:M:81:SER:CB	2.03	0.66
3:M:100:LEU:CD1	3:M:100:LEU:H	2.09	0.66
3:M:258:VAL:HG13	3:M:452:ILE:HG13	1.77	0.66
3:M:272:LEU:HD21	3:M:288:ILE:HD13	1.76	0.66
3:M:435:LEU:O	3:M:479:PHE:CE2	2.49	0.66
4:S:57:LEU:N	4:S:57:LEU:HD12	2.11	0.66
1:A:323:CYS:CB	1:A:355:LEU:HD21	2.26	0.65
2:B:123:LEU:HD13	2:B:142:LEU:HG	0.67	0.65
2:B:530:LEU:HD23	2:B:591:MET:HB2	1.78	0.65
3:M:213:GLU:C	3:M:467:TYR:HB2	2.16	0.65
3:M:360:LEU:HD13	3:M:433:VAL:CG2	2.26	0.65
1:A:520:GLY:HA2	1:A:558:VAL:CG2	2.23	0.65
2:B:67:ILE:HD11	2:B:100:LEU:O	1.95	0.65
2:B:103:LEU:CD1	3:M:132:GLY:HA3	2.24	0.65
2:B:120:ILE:HD11	2:B:150:LEU:HD13	1.77	0.65
2:B:175:LEU:CD2	2:B:210:CYS:C	2.64	0.65
2:B:336:ASN:HB3	2:B:339:PHE:CD1	2.32	0.65
3:M:2:TYR:CE2	3:M:62:VAL:HG13	2.31	0.65
3:M:302:TYR:HE2	3:M:304:VAL:HB	1.61	0.65
3:M:403:THR:HG23	3:M:407:THR:HG1	1.62	0.65
3:M:437:TYR:CE1	3:M:479:PHE:CE1	2.85	0.65
1:A:225:LEU:HB2	1:A:233:PHE:CZ	2.31	0.65
1:A:253:ILE:HG21	1:A:281:LEU:HB3	1.76	0.65
1:A:264:SER:HB2	1:A:271:ARG:CG	2.24	0.65
1:A:557:LYS:O	2:B:605:PHE:CE2	2.45	0.65
1:A:557:LYS:HG2	2:B:606:ASP:HA	1.76	0.65

Continued on next page...

Continued from previous page...

Atom-1	Atom-2	Interatomic distance (Å)	Clash overlap (Å)
1:A:609:LEU:O	1:A:612:GLU:N	2.29	0.65
2:B:123:LEU:HD22	2:B:138:ALA:CA	2.27	0.65
2:B:208:ILE:CG1	2:B:236:ILE:HD13	2.19	0.65
2:B:212:VAL:CG1	2:B:248:LEU:HD23	2.26	0.65
2:B:252:LEU:HD13	2:B:302:PHE:CG	2.14	0.65
2:B:331:PRO:HB3	2:B:369:LEU:CD1	2.27	0.65
2:B:334:MET:HE2	2:B:339:PHE:CD1	2.31	0.65
2:B:346:THR:O	2:B:349:MET:N	2.29	0.65
2:B:353:GLN:HG2	3:M:48:ASP:C	2.17	0.65
2:B:447:GLU:OE1	2:B:485:LYS:CG	2.41	0.65
2:B:545:ARG:HD2	2:B:602:ASP:CG	2.17	0.65
2:B:549:LEU:HD22	2:B:607:ILE:O	1.96	0.65
3:M:55:MET:O	3:M:57:GLY:N	2.30	0.65
3:M:229:LYS:HG2	3:M:230:LYS:HG3	1.79	0.65
3:M:379:LEU:HD12	3:M:379:LEU:N	2.10	0.65
4:S:108:SER:CB	4:S:149:ILE:CG2	2.73	0.65
1:A:132:LEU:CD2	1:A:169:MET:CG	2.62	0.65
1:A:170:LEU:HB3	1:A:206:LYS:HG3	1.77	0.65
1:A:557:LYS:CG	2:B:606:ASP:HB2	2.25	0.65
2:B:211:ALA:C	2:B:233:TYR:CZ	2.52	0.65
2:B:223:LEU:HD11	2:B:259:TYR:N	1.90	0.65
2:B:340:ILE:HD13	2:B:366:LEU:CD1	2.18	0.65
2:B:438:ARG:HA	2:B:441:GLN:NE2	2.11	0.65
3:M:214:LEU:CD1	3:M:256:VAL:CG2	2.74	0.65
3:M:241:HIS:HD2	3:M:475:GLN:H	1.42	0.65
3:M:340:LEU:CB	3:M:411:LEU:HG	2.14	0.65
4:S:24:ASP:OD2	4:S:27:LYS:HD2	1.96	0.65
1:A:128:LEU:HD13	1:A:150:LEU:HD21	1.22	0.65
1:A:189:PHE:HB2	1:A:225:LEU:CD2	2.26	0.65
1:A:485:PRO:O	1:A:488:ARG:CG	2.43	0.65
1:A:638:LEU:O	2:B:486:HIS:CE1	2.50	0.65
2:B:90:ILE:CG1	2:B:98:LYS:HD3	2.27	0.65
2:B:178:ILE:CG2	2:B:214:ALA:C	2.64	0.65
2:B:274:PRO:N	2:B:295:ASN:ND2	2.44	0.65
3:M:66:PHE:CB	3:M:77:LEU:HD21	2.03	0.65
3:M:242:GLY:HA3	3:M:444:ALA:CB	2.25	0.65
1:A:67:LYS:CB	4:S:166:LYS:HA	2.27	0.65
1:A:78:GLU:O	1:A:80:TYR:O	2.13	0.65
1:A:88:ASN:HB2	1:A:120:ILE:HD12	1.73	0.65
1:A:105:VAL:HG23	4:S:167:ILE:CB	2.26	0.65
1:A:211:ASP:OD2	4:S:148:ARG:CD	2.43	0.65

Continued on next page...

Continued from previous page...

Atom-1	Atom-2	Interatomic distance (Å)	Clash overlap (Å)
1:A:416:ILE:HG22	1:A:417:PRO:O	1.96	0.65
1:A:420:ILE:HG23	1:A:421:PRO:HD2	1.77	0.65
1:A:638:LEU:O	2:B:518:ILE:CD1	2.44	0.65
2:B:5:ILE:CA	2:B:8:ILE:HD12	2.26	0.65
2:B:67:ILE:HD13	2:B:103:LEU:HB3	1.77	0.65
2:B:159:LYS:HE3	2:B:191:GLU:CD	2.16	0.65
2:B:178:ILE:CG2	2:B:214:ALA:HA	2.24	0.65
2:B:479:VAL:HG21	2:B:486:HIS:NE2	2.11	0.65
2:B:490:ILE:CG1	2:B:518:ILE:HG21	2.23	0.65
4:S:105:PHE:HZ	4:S:128:LEU:CD1	2.08	0.65
1:A:103:LYS:HE3	1:A:131:ASP:CG	2.05	0.65
1:A:412:LYS:O	1:A:413:SER:C	2.31	0.65
1:A:530:ASN:OD1	1:A:577:VAL:HG21	1.96	0.65
2:B:177:ILE:HG22	2:B:196:LEU:HD11	1.79	0.65
2:B:277:CYS:HA	2:B:292:GLU:HA	1.77	0.65
2:B:351:GLU:O	3:M:48:ASP:CB	2.42	0.65
2:B:430:ILE:HG23	2:B:470:ALA:HB2	1.78	0.65
3:M:5:PHE:HB2	3:M:125:PHE:CE2	2.31	0.65
3:M:216:VAL:O	3:M:216:VAL:HG23	1.96	0.65
3:M:222:PHE:CE1	3:M:240:ILE:HG21	2.32	0.65
3:M:327:PHE:CE1	3:M:336:ASP:OD2	2.49	0.65
3:M:433:VAL:HG13	3:M:433:VAL:O	1.97	0.65
2:B:105:LEU:CB	2:B:145:MET:CE	2.65	0.65
2:B:178:ILE:CG2	2:B:179:LYS:N	2.51	0.65
2:B:435:SER:O	2:B:438:ARG:N	2.19	0.65
2:B:575:ASN:C	2:B:576:GLN:O	2.26	0.65
1:A:196:LEU:HD22	1:A:196:LEU:C	2.15	0.65
1:A:220:SER:HB2	4:S:142:ILE:HA	1.79	0.65
1:A:291:ILE:N	1:A:291:ILE:HD12	2.12	0.65
1:A:364:ASP:HB3	1:A:367:ILE:HD12	1.79	0.65
1:A:384:LEU:HD21	1:A:441:TYR:CE2	2.28	0.65
1:A:480:LEU:C	1:A:480:LEU:HD13	2.17	0.65
1:A:570:LYS:O	1:A:571:ARG:HB2	1.97	0.65
2:B:37:TYR:HH	2:B:46:GLN:HE21	0.83	0.65
2:B:158:VAL:HG13	2:B:177:ILE:CD1	2.19	0.65
2:B:196:LEU:HB3	2:B:229:HIS:ND1	2.10	0.65
2:B:567:GLN:HG2	2:B:569:THR:OG1	1.97	0.65
3:M:54:SER:HB2	3:M:66:PHE:HD1	1.62	0.65
3:M:101:LEU:HG	3:M:106:LYS:CG	2.26	0.65
3:M:271:SER:HB3	3:M:301:GLU:HG3	1.79	0.65
3:M:316:ARG:O	3:M:317:MET:C	2.34	0.65

Continued on next page...

Continued from previous page...

Atom-1	Atom-2	Interatomic distance (Å)	Clash overlap (Å)
3:M:319:SER:HB2	3:M:347:PHE:N	2.11	0.65
1:A:97:SER:H	4:S:166:LYS:HZ1	1.45	0.65
1:A:104:ARG:NE	4:S:160:ALA:HB2	2.11	0.65
1:A:136:GLY:O	1:A:137:ASN:C	2.27	0.65
2:B:50:LEU:HB3	2:B:62:ALA:CB	2.23	0.65
2:B:50:LEU:CG	2:B:62:ALA:HB2	2.27	0.65
2:B:141:ALA:C	2:B:143:SER:N	2.46	0.65
2:B:350:THR:CB	2:B:352:ASN:HD21	2.10	0.65
3:M:224:VAL:HB	3:M:226:PHE:CE1	2.32	0.65
3:M:244:VAL:HG13	3:M:472:TYR:HE2	1.50	0.65
1:A:185:LEU:HB3	1:A:189:PHE:CE2	2.32	0.64
1:A:638:LEU:HD13	2:B:515:PHE:O	1.98	0.64
2:B:73:ASP:OD1	2:B:111:ASN:ND2	2.28	0.64
2:B:157:THR:O	2:B:159:LYS:N	2.30	0.64
2:B:227:HIS:CE1	2:B:292:GLU:HG2	2.32	0.64
2:B:306:LEU:HB3	2:B:321:CYS:HB3	1.78	0.64
2:B:578:PRO:HB3	2:B:579:PRO:HD3	1.80	0.64
3:M:371:GLU:O	3:M:419:ASN:OD1	2.14	0.64
4:S:3:HIS:NE2	4:S:90:ASP:OD2	2.30	0.64
1:A:145:ILE:HD13	4:S:156:LEU:HD22	1.78	0.64
1:A:150:LEU:HD22	1:A:158:LEU:HD11	1.79	0.64
2:B:196:LEU:C	2:B:229:HIS:CE1	2.69	0.64
2:B:212:VAL:HG23	2:B:233:TYR:CE2	2.33	0.64
2:B:344:VAL:HG22	2:B:381:PHE:CZ	2.32	0.64
2:B:519:ALA:HB1	2:B:555:LEU:HD12	1.80	0.64
2:B:549:LEU:CG	2:B:614:ILE:HD12	2.27	0.64
3:M:3:LEU:HA	3:M:80:THR:HA	1.79	0.64
3:M:101:LEU:C	3:M:106:LYS:CA	2.64	0.64
1:A:323:CYS:SG	1:A:338:PHE:CE1	2.74	0.64
2:B:90:ILE:HD11	2:B:123:LEU:CD2	2.27	0.64
2:B:344:VAL:CG2	2:B:363:ILE:CD1	2.74	0.64
2:B:396:ILE:HG21	2:B:432:ALA:HA	1.78	0.64
2:B:513:TRP:CG	2:B:551:LEU:CD2	2.76	0.64
3:M:255:LEU:O	3:M:454:ILE:HG13	1.97	0.64
3:M:256:VAL:HG12	3:M:290:PHE:CB	2.23	0.64
3:M:352:GLN:HB3	3:M:403:THR:O	1.97	0.64
4:S:69:ASN:ND2	4:S:71:GLU:O	2.31	0.64
4:S:135:ILE:C	4:S:141:VAL:HG22	2.18	0.64
1:A:552:ILE:O	1:A:556:VAL:HG23	1.97	0.64
1:A:585:PHE:CD2	1:A:603:VAL:HG12	2.32	0.64
1:A:634:ASN:ND2	2:B:554:LYS:N	2.37	0.64

Continued on next page...

Continued from previous page...

Atom-1	Atom-2	Interatomic distance (Å)	Clash overlap (Å)
2:B:158:VAL:CG2	2:B:177:ILE:HG13	2.27	0.64
2:B:275:ARG:HB3	2:B:294:VAL:CG1	2.19	0.64
3:M:49:ASP:HA	3:M:75:TRP:CZ3	2.32	0.64
3:M:250:LEU:HD13	3:M:254:PRO:CG	2.27	0.64
3:M:350:VAL:CG1	3:M:442:GLN:HB2	2.26	0.64
1:A:67:LYS:HB2	4:S:166:LYS:N	2.11	0.64
1:A:96:SER:HB2	1:A:127:LEU:CD1	2.08	0.64
1:A:239:LEU:O	1:A:242:GLU:O	2.14	0.64
2:B:20:ARG:HH11	2:B:21:GLU:HG2	1.57	0.64
2:B:79:VAL:HG21	2:B:108:PHE:CZ	2.33	0.64
2:B:123:LEU:CD2	2:B:138:ALA:HB1	2.27	0.64
2:B:214:ALA:C	2:B:216:LYS:N	2.48	0.64
2:B:316:THR:CG2	3:M:90:PHE:HE2	1.75	0.64
2:B:346:THR:HG22	2:B:350:THR:HG21	1.78	0.64
3:M:74:TYR:OH	3:M:97:ASP:HB3	1.98	0.64
3:M:214:LEU:O	3:M:214:LEU:HD23	1.97	0.64
3:M:257:ALA:HB2	3:M:455:VAL:CG2	2.28	0.64
4:S:8:PHE:HE2	4:S:86:THR:OG1	1.81	0.64
1:A:91:ILE:HG21	1:A:110:ALA:HB2	1.80	0.64
1:A:103:LYS:C	1:A:107:TYR:CD1	2.70	0.64
2:B:87:VAL:HG13	2:B:122:SER:HG	1.57	0.64
2:B:266:VAL:HA	2:B:289:PRO:HB2	1.79	0.64
2:B:513:TRP:C	2:B:551:LEU:HD22	2.17	0.64
2:B:559:ASP:HB3	2:B:563:PHE:CG	2.33	0.64
3:M:96:ILE:CG2	3:M:125:PHE:CE1	2.81	0.64
3:M:260:LEU:HD22	3:M:449:VAL:CG2	2.24	0.64
1:A:119:ASP:O	1:A:123:LEU:HG	1.97	0.64
1:A:513:ARG:HB3	1:A:550:VAL:HG11	1.78	0.64
1:A:575:LYS:HG3	1:A:611:LEU:HG	1.77	0.64
2:B:29:LYS:HD3	2:B:30:LEU:H	1.62	0.64
2:B:30:LEU:C	2:B:30:LEU:HD12	2.18	0.64
2:B:219:TYR:HD1	2:B:226:LEU:CD2	1.83	0.64
2:B:306:LEU:HD12	2:B:325:LEU:CD2	2.28	0.64
3:M:217:ASP:CB	3:M:471:LYS:HA	2.27	0.64
1:A:255:ARG:O	1:A:257:LEU:N	2.31	0.64
1:A:405:THR:OG1	1:A:406:GLY:N	2.27	0.64
1:A:557:LYS:HG2	2:B:606:ASP:CA	2.28	0.64
1:A:581:LEU:HD23	1:A:607:LEU:HD22	1.80	0.64
1:A:631:SER:HB3	2:B:557:SER:HB2	1.74	0.64
2:B:132:SER:HB2	2:B:166:SER:CB	2.27	0.64
2:B:396:ILE:HD11	2:B:418:TYR:OH	1.92	0.64

Continued on next page...

Continued from previous page...

Atom-1	Atom-2	Interatomic distance (Å)	Clash overlap (Å)
3:M:214:LEU:O	3:M:467:TYR:CB	2.44	0.64
3:M:356:LEU:HD21	3:M:358:ILE:CG1	2.27	0.64
1:A:125:THR:C	1:A:127:LEU:N	2.50	0.64
1:A:436:CYS:SG	1:A:450:TYR:CE2	2.87	0.64
1:A:609:LEU:CD2	1:A:628:VAL:CB	2.76	0.64
2:B:79:VAL:HB	2:B:108:PHE:CD1	2.33	0.64
2:B:277:CYS:HA	2:B:292:GLU:CB	2.26	0.64
2:B:461:HIS:O	2:B:462:ASN:O	2.16	0.64
2:B:508:ARG:O	2:B:509:ALA:O	2.16	0.64
2:B:534:ILE:HD11	2:B:595:VAL:HG23	1.80	0.64
2:B:566:ALA:O	2:B:574:ASN:HB3	1.78	0.64
3:M:16:PHE:HE2	3:M:125:PHE:CE2	2.16	0.64
3:M:316:ARG:O	3:M:318:ASN:OD1	2.15	0.64
4:S:53:THR:CG2	4:S:69:ASN:N	2.61	0.64
4:S:157:ASN:O	4:S:161:GLU:HG3	1.97	0.64
1:A:67:LYS:HB3	4:S:166:LYS:CA	2.28	0.64
1:A:219:VAL:CG2	1:A:256:LEU:CD2	2.69	0.64
2:B:2:VAL:CG1	2:B:6:HIS:CD2	2.61	0.64
2:B:8:ILE:O	2:B:12:LEU:CD1	2.45	0.64
2:B:47:LEU:CB	2:B:66:ILE:HG12	2.28	0.64
2:B:50:LEU:HD23	2:B:62:ALA:N	2.13	0.64
2:B:136:CYS:O	2:B:172:GLU:CB	2.45	0.64
2:B:309:LEU:CG	2:B:317:VAL:HG11	2.27	0.64
3:M:316:ARG:HG3	3:M:322:LEU:HD13	1.80	0.64
3:M:375:LYS:HE3	3:M:418:GLU:OE1	1.97	0.64
4:S:8:PHE:CE2	4:S:86:THR:OG1	2.50	0.64
1:A:105:VAL:CG2	4:S:167:ILE:HA	2.28	0.63
1:A:484:VAL:O	1:A:486:SER:O	2.16	0.63
1:A:598:GLU:O	1:A:602:GLU:HG3	1.99	0.63
2:B:45:GLN:C	2:B:47:LEU:H	2.01	0.63
2:B:93:ASN:C	2:B:134:LEU:HD11	2.18	0.63
2:B:275:ARG:NE	2:B:275:ARG:HA	2.13	0.63
2:B:449:HIS:O	2:B:453:TRP:CD1	2.51	0.63
2:B:520:SER:O	2:B:523:PHE:HE2	1.81	0.63
3:M:45:SER:HA	3:M:47:SER:N	2.07	0.63
3:M:100:LEU:HD13	3:M:100:LEU:N	2.13	0.63
1:A:104:ARG:NH1	4:S:126:GLN:C	2.52	0.63
1:A:121:LEU:CD1	1:A:155:THR:CG2	2.77	0.63
1:A:196:LEU:O	1:A:197:ARG:C	2.31	0.63
1:A:297:CYS:O	1:A:298:ILE:C	2.32	0.63
1:A:451:ASN:OD1	1:A:480:LEU:HD12	1.98	0.63

Continued on next page...

Continued from previous page...

Atom-1	Atom-2	Interatomic distance (Å)	Clash overlap (Å)
2:B:63:MET:HA	2:B:66:ILE:HD12	1.78	0.63
2:B:83:PHE:HZ	2:B:119:SER:HB2	1.53	0.63
2:B:98:LYS:HB2	2:B:134:LEU:HD22	1.78	0.63
2:B:215:TYR:CD2	2:B:226:LEU:HD13	2.31	0.63
2:B:487:LEU:HD21	2:B:522:GLU:CB	2.25	0.63
2:B:545:ARG:HD2	2:B:602:ASP:HB2	1.79	0.63
3:M:100:LEU:H	3:M:100:LEU:HD13	1.61	0.63
3:M:215:TYR:HB2	3:M:467:TYR:C	2.18	0.63
3:M:241:HIS:CB	3:M:476:THR:HG23	2.29	0.63
1:A:67:LYS:C	4:S:166:LYS:CA	2.66	0.63
1:A:103:LYS:HZ2	1:A:131:ASP:HB2	1.63	0.63
1:A:322:PHE:HB3	1:A:330:LEU:HD21	1.79	0.63
2:B:157:THR:C	2:B:159:LYS:N	2.51	0.63
2:B:167:ALA:O	2:B:207:VAL:HG11	1.99	0.63
2:B:245:GLN:NE2	2:B:309:LEU:HD13	2.14	0.63
2:B:287:GLU:O	2:B:288:TYR:CD2	2.50	0.63
2:B:364:HIS:CE1	2:B:397:GLN:HB3	2.33	0.63
2:B:537:PHE:CD2	2:B:537:PHE:C	2.71	0.63
1:A:100:LEU:HD11	4:S:157:ASN:C	2.17	0.63
2:B:36:THR:O	2:B:40:GLN:CB	2.46	0.63
3:M:10:THR:HA	3:M:75:TRP:HE1	1.62	0.63
3:M:96:ILE:CG2	3:M:125:PHE:CZ	2.81	0.63
3:M:258:VAL:CA	3:M:452:ILE:HG13	2.28	0.63
3:M:265:ASN:HB3	3:M:309:GLN:CG	2.29	0.63
3:M:320:ILE:HG13	3:M:347:PHE:HD1	1.59	0.63
4:S:164:ASP:O	4:S:165:SER:C	2.20	0.63
1:A:104:ARG:HG2	4:S:126:GLN:OE1	1.97	0.63
1:A:139:ASP:OD1	1:A:177:ILE:HD11	1.98	0.63
1:A:156:PRO:O	1:A:160:ARG:HG3	1.98	0.63
1:A:175:PRO:HA	1:A:214:VAL:CG2	2.29	0.63
2:B:178:ILE:HG22	2:B:217:GLU:HB2	1.74	0.63
2:B:243:TRP:CZ3	3:M:91:THR:CA	2.75	0.63
2:B:274:PRO:CG	2:B:295:ASN:HD21	2.05	0.63
2:B:319:LEU:O	2:B:322:CYS:N	2.31	0.63
2:B:360:LEU:HD11	2:B:391:ALA:HB1	1.81	0.63
2:B:475:ILE:CG2	2:B:514:LEU:CD2	2.76	0.63
3:M:125:PHE:O	3:M:129:VAL:HG21	1.93	0.63
3:M:347:PHE:CE2	3:M:350:VAL:HB	2.34	0.63
1:A:68:THR:HA	4:S:167:ILE:CA	2.28	0.63
1:A:185:LEU:O	1:A:189:PHE:CG	2.51	0.63
1:A:571:ARG:NH2	1:A:573:GLU:OE1	2.30	0.63

Continued on next page...

Continued from previous page...

Atom-1	Atom-2	Interatomic distance (Å)	Clash overlap (Å)
2:B:90:ILE:HD11	2:B:123:LEU:HD21	1.80	0.63
2:B:295:ASN:O	2:B:296:ASP:HB2	1.99	0.63
2:B:334:MET:HB2	2:B:369:LEU:CD2	2.27	0.63
2:B:396:ILE:HD11	2:B:418:TYR:CE2	2.25	0.63
2:B:452:LYS:HZ3	2:B:456:ASP:CG	2.01	0.63
2:B:515:PHE:O	2:B:516:GLY:C	2.33	0.63
2:B:526:CYS:N	2:B:527:PRO:HD3	2.14	0.63
3:M:354:ASP:CB	3:M:440:ILE:HD12	2.28	0.63
3:M:360:LEU:HD13	3:M:433:VAL:CB	2.27	0.63
4:S:6:LEU:CD2	4:S:32:LEU:HD22	2.24	0.63
1:A:141:VAL:HG12	4:S:159:ALA:HB3	0.73	0.63
1:A:179:LYS:CD	4:S:143:GLU:CG	2.74	0.63
1:A:313:MET:HA	1:A:348:PHE:CZ	2.33	0.63
1:A:563:CYS:SG	1:A:621:LEU:HD11	2.39	0.63
1:A:575:LYS:HE3	1:A:611:LEU:HD21	1.80	0.63
1:A:581:LEU:CD2	1:A:607:LEU:CD1	2.77	0.63
2:B:362:ALA:O	2:B:366:LEU:HG	1.99	0.63
2:B:592:TYR:CE2	2:B:619:ASP:OD1	2.52	0.63
3:M:10:THR:HG23	3:M:11:LYS:N	2.13	0.63
3:M:215:TYR:CD2	3:M:470:ALA:N	2.64	0.63
1:A:96:SER:H	4:S:166:LYS:NZ	1.96	0.63
1:A:141:VAL:HG11	4:S:159:ALA:N	2.12	0.63
1:A:178:ARG:HB2	1:A:214:VAL:HG22	1.78	0.63
1:A:253:ILE:CD1	1:A:281:LEU:CD2	2.70	0.63
1:A:369:SER:HB2	1:A:424:TYR:CE2	2.32	0.63
1:A:566:PHE:HZ	1:A:618:THR:O	1.82	0.63
1:A:607:LEU:O	1:A:609:LEU:N	2.32	0.63
2:B:268:LYS:CA	2:B:276:SER:HB2	2.29	0.63
2:B:293:VAL:O	2:B:299:LEU:HG	1.99	0.63
2:B:481:LYS:C	2:B:483:PRO:HD3	2.18	0.63
3:M:306:LEU:CD2	3:M:317:MET:CE	2.77	0.63
4:S:7:ILE:CD1	4:S:16:LEU:HD23	2.28	0.63
4:S:20:TYR:HD2	4:S:21:THR:HG23	1.62	0.63
4:S:80:TYR:HB2	4:S:106:VAL:HG11	1.80	0.63
1:A:100:LEU:N	4:S:162:SER:CB	2.04	0.63
1:A:102:GLN:CD	4:S:166:LYS:N	2.42	0.63
1:A:225:LEU:HB2	1:A:233:PHE:CE1	2.33	0.63
1:A:563:CYS:SG	1:A:621:LEU:HD12	2.37	0.63
1:A:581:LEU:HD23	1:A:607:LEU:HD11	1.81	0.63
2:B:4:SER:O	2:B:8:ILE:HD12	1.99	0.63
2:B:136:CYS:C	2:B:172:GLU:CB	2.67	0.63

Continued on next page...

Continued from previous page...

Atom-1	Atom-2	Interatomic distance (Å)	Clash overlap (Å)
2:B:431:MET:C	2:B:433:VAL:H	2.03	0.63
2:B:518:ILE:O	2:B:518:ILE:CD1	2.47	0.63
2:B:545:ARG:HD2	2:B:602:ASP:CB	2.28	0.63
3:M:99:ILE:CD1	3:M:99:ILE:H	2.12	0.63
3:M:245:ASP:N	3:M:472:TYR:CZ	2.66	0.63
3:M:377:LYS:HE3	3:M:416:GLU:CB	2.28	0.63
3:M:437:TYR:HD1	3:M:479:PHE:CZ	2.17	0.63
1:A:144:GLY:HA2	1:A:180:LYS:HB3	1.78	0.62
2:B:90:ILE:HD11	2:B:102:HIS:NE2	2.14	0.62
2:B:124:GLN:HA	2:B:157:THR:HG1	1.63	0.62
2:B:154:ILE:CD1	2:B:180:LEU:CG	2.77	0.62
2:B:154:ILE:O	2:B:158:VAL:HG23	1.98	0.62
2:B:353:GLN:HG3	3:M:47:SER:CB	2.29	0.62
2:B:353:GLN:CG	3:M:47:SER:HB2	2.29	0.62
3:M:320:ILE:HG22	3:M:321:GLY:N	2.14	0.62
4:S:8:PHE:CG	4:S:36:TYR:CE2	2.83	0.62
4:S:47:GLN:HE22	4:S:78:LYS:CA	2.11	0.62
4:S:137:GLN:C	4:S:140:MET:H	2.02	0.62
1:A:100:LEU:CA	4:S:162:SER:HB2	2.22	0.62
1:A:117:ASP:OD2	1:A:120:ILE:HG12	2.00	0.62
1:A:142:LYS:H	4:S:159:ALA:HB2	1.53	0.62
1:A:237:SER:CB	1:A:270:LEU:HD13	2.23	0.62
1:A:342:GLY:O	1:A:343:LYS:C	2.35	0.62
2:B:63:MET:HE1	2:B:104:TYR:CB	2.29	0.62
2:B:435:SER:C	2:B:437:SER:N	2.53	0.62
2:B:512:VAL:HG12	2:B:551:LEU:HB2	1.82	0.62
2:B:567:GLN:CA	2:B:569:THR:OG1	2.47	0.62
3:M:66:PHE:CE2	3:M:77:LEU:O	2.53	0.62
3:M:104:PHE:HE1	3:M:113:LYS:HE2	0.77	0.62
3:M:272:LEU:CD2	3:M:288:ILE:HD13	2.30	0.62
1:A:80:TYR:CD1	1:A:82:PHE:CE2	2.88	0.62
1:A:180:LYS:HE3	4:S:156:LEU:HD11	1.81	0.62
1:A:240:LEU:O	1:A:242:GLU:O	2.18	0.62
2:B:157:THR:O	2:B:160:LYS:N	2.32	0.62
2:B:182:ARG:HD2	2:B:217:GLU:CG	2.29	0.62
2:B:307:ASN:OD1	2:B:339:PHE:HE2	1.77	0.62
2:B:416:LYS:HD2	2:B:453:TRP:CD2	2.34	0.62
2:B:500:GLN:O	2:B:508:ARG:NH2	2.32	0.62
3:M:10:THR:CA	3:M:75:TRP:NE1	2.62	0.62
1:A:76:TYR:OH	4:S:125:TRP:CZ3	2.39	0.62
1:A:96:SER:N	4:S:166:LYS:HZ1	1.97	0.62

Continued on next page...

Continued from previous page...

Atom-1	Atom-2	Interatomic distance (Å)	Clash overlap (Å)
1:A:101:GLN:N	4:S:162:SER:H	1.96	0.62
1:A:176:TYR:CG	4:S:155:GLU:HG2	2.34	0.62
1:A:212:ILE:HB	4:S:145:ASN:HD22	1.63	0.62
1:A:255:ARG:HH21	4:S:135:ILE:CG2	1.99	0.62
1:A:403:LEU:HD21	1:A:421:PRO:CA	2.28	0.62
1:A:513:ARG:CG	1:A:550:VAL:HG21	2.29	0.62
2:B:136:CYS:CA	2:B:172:GLU:HB2	2.28	0.62
2:B:153:ILE:C	2:B:155:LEU:H	2.03	0.62
3:M:242:GLY:CA	3:M:444:ALA:CB	2.77	0.62
4:S:48:SER:HA	4:S:77:TYR:HB3	1.81	0.62
4:S:164:ASP:HA	4:S:167:ILE:HB	1.79	0.62
1:A:141:VAL:CB	4:S:159:ALA:HB2	2.29	0.62
2:B:135:ARG:NH2	2:B:164:ASP:CB	2.62	0.62
2:B:196:LEU:O	2:B:199:LEU:HB2	1.99	0.62
2:B:196:LEU:CB	2:B:229:HIS:HD1	2.13	0.62
2:B:316:THR:HG23	3:M:90:PHE:CE2	2.31	0.62
2:B:319:LEU:CD1	2:B:358:MET:SD	2.78	0.62
2:B:472:VAL:HG11	2:B:510:GLY:HA2	1.68	0.62
3:M:407:THR:O	3:M:409:PRO:HD3	1.98	0.62
1:A:175:PRO:O	1:A:214:VAL:HG22	2.00	0.62
3:M:62:VAL:O	3:M:62:VAL:HG12	2.00	0.62
3:M:224:VAL:CA	3:M:479:PHE:HA	2.29	0.62
3:M:356:LEU:C	3:M:356:LEU:HD23	2.19	0.62
4:S:85:PHE:CE1	4:S:106:VAL:HG22	2.34	0.62
1:A:80:TYR:HB3	1:A:82:PHE:CZ	2.34	0.62
1:A:99:LYS:HZ2	4:S:164:ASP:HB3	1.65	0.62
1:A:212:ILE:HB	1:A:247:ILE:HD13	1.78	0.62
1:A:480:LEU:O	1:A:483:LYS:O	2.18	0.62
1:A:623:MET:C	2:B:617:LEU:HD21	2.20	0.62
2:B:106:LEU:CD2	2:B:144:ASP:CB	2.76	0.62
2:B:177:ILE:HG21	2:B:196:LEU:HD11	1.79	0.62
2:B:309:LEU:CG	2:B:317:VAL:CG1	2.77	0.62
2:B:511:ILE:O	2:B:515:PHE:HD1	1.83	0.62
2:B:588:ILE:HG23	2:B:618:PHE:CE1	2.34	0.62
3:M:216:VAL:HG11	3:M:452:ILE:HG12	1.81	0.62
3:M:225:VAL:HA	3:M:480:GLN:H	1.65	0.62
3:M:362:PHE:O	3:M:364:VAL:HG13	2.00	0.62
4:S:75:ILE:CG2	4:S:86:THR:HG23	2.26	0.62
1:A:101:GLN:O	1:A:104:ARG:N	2.29	0.62
1:A:589:SER:O	1:A:597:GLN:NE2	2.32	0.62
2:B:47:LEU:HD13	2:B:66:ILE:HA	1.81	0.62

Continued on next page...

Continued from previous page...

Atom-1	Atom-2	Interatomic distance (Å)	Clash overlap (Å)
2:B:143:SER:HB3	2:B:175:LEU:C	2.18	0.62
2:B:486:HIS:HA	2:B:489:ILE:HD12	1.82	0.62
1:A:67:LYS:HB3	4:S:166:LYS:CG	2.29	0.62
1:A:91:ILE:CG2	1:A:110:ALA:HB2	2.30	0.62
1:A:182:ILE:CG2	1:A:218:ALA:HA	2.29	0.62
1:A:341:ILE:O	1:A:344:ILE:HB	1.99	0.62
1:A:408:ILE:CG2	4:S:64:ASN:O	2.34	0.62
2:B:360:LEU:HD13	2:B:391:ALA:O	2.00	0.62
2:B:476:ARG:CA	2:B:514:LEU:HD13	2.30	0.62
2:B:501:THR:C	2:B:508:ARG:NH2	2.53	0.62
3:M:4:SER:OG	3:M:6:TYR:CZ	2.51	0.62
3:M:24:ALA:O	3:M:25:PRO:C	2.34	0.62
3:M:222:PHE:CB	3:M:479:PHE:CE1	2.82	0.62
3:M:320:ILE:HG21	3:M:439:TYR:CZ	2.35	0.62
4:S:32:LEU:O	4:S:36:TYR:CD1	2.53	0.62
4:S:47:GLN:OE1	4:S:79:ASN:CA	2.42	0.62
1:A:80:TYR:HB3	1:A:82:PHE:CE2	2.33	0.62
1:A:326:GLN:CA	1:A:331:ARG:HH21	2.11	0.62
1:A:556:VAL:CG2	1:A:603:VAL:HG11	2.29	0.62
2:B:70:MET:HE2	2:B:104:TYR:HA	1.80	0.62
2:B:545:ARG:HD3	2:B:602:ASP:CB	2.17	0.62
2:B:545:ARG:NH1	2:B:602:ASP:HA	2.14	0.62
3:M:1:MET:CA	3:M:81:SER:OG	2.48	0.62
3:M:215:TYR:CD1	3:M:468:LYS:HG2	2.35	0.62
3:M:223:HIS:CD2	3:M:478:ASN:CA	2.82	0.62
3:M:449:VAL:HG11	3:M:452:ILE:CG1	2.30	0.62
4:S:9:ASN:OD1	4:S:13:GLN:CA	2.46	0.62
1:A:212:ILE:CB	1:A:247:ILE:HD13	2.30	0.61
1:A:399:ASP:O	1:A:420:ILE:N	2.33	0.61
2:B:216:LYS:HB2	2:B:251:LEU:CG	2.30	0.61
2:B:302:PHE:CD2	2:B:328:LEU:HD11	2.35	0.61
2:B:336:ASN:CG	2:B:338:LYS:HB2	2.21	0.61
2:B:512:VAL:HG11	2:B:548:ILE:CA	2.11	0.61
2:B:362:ALA:O	2:B:366:LEU:N	2.28	0.61
3:M:214:LEU:N	3:M:467:TYR:HB2	2.15	0.61
3:M:215:TYR:CE1	3:M:468:LYS:CG	2.82	0.61
3:M:386:PHE:CD1	3:M:386:PHE:O	2.54	0.61
3:M:469:GLY:C	3:M:470:ALA:O	2.38	0.61
1:A:182:ILE:O	1:A:221:VAL:CG2	2.47	0.61
1:A:183:THR:C	4:S:137:GLN:OE1	2.39	0.61
1:A:630:PRO:CG	2:B:614:ILE:HG23	2.30	0.61

Continued on next page...

Continued from previous page...

Atom-1	Atom-2	Interatomic distance (Å)	Clash overlap (Å)
2:B:21:GLU:O	2:B:24:ALA:HB3	2.01	0.61
2:B:162:VAL:C	2:B:164:ASP:N	2.53	0.61
2:B:463:LEU:O	2:B:468:LEU:HD11	2.00	0.61
2:B:508:ARG:HB2	2:B:544:THR:HG21	1.78	0.61
2:B:520:SER:O	2:B:523:PHE:CE2	2.53	0.61
3:M:233:LEU:CD2	3:M:325:LEU:H	2.13	0.61
3:M:258:VAL:HG22	3:M:452:ILE:HG12	1.81	0.61
3:M:267:ILE:HG23	3:M:445:SER:OG	1.99	0.61
3:M:338:PHE:HE2	3:M:415:ILE:HG13	1.55	0.61
4:S:3:HIS:O	4:S:20:TYR:N	2.31	0.61
4:S:17:VAL:HG22	4:S:19:PHE:CE1	2.34	0.61
1:A:259:LEU:CD1	4:S:140:MET:HE1	2.31	0.61
1:A:312:ALA:O	1:A:315:CYS:HB2	2.00	0.61
1:A:556:VAL:HG22	1:A:603:VAL:CG1	2.30	0.61
1:A:589:SER:CB	1:A:601:VAL:HG22	2.30	0.61
2:B:276:SER:C	2:B:289:PRO:HG2	2.14	0.61
2:B:311:TYR:O	2:B:312:SER:C	2.38	0.61
3:M:44:ASP:C	3:M:46:SER:N	2.53	0.61
3:M:69:ILE:HD12	3:M:90:PHE:CE2	2.25	0.61
3:M:224:VAL:CG1	3:M:226:PHE:CE1	2.84	0.61
3:M:242:GLY:N	3:M:444:ALA:CB	2.64	0.61
3:M:323:MET:CE	3:M:342:LEU:HD23	2.27	0.61
3:M:356:LEU:CD2	3:M:358:ILE:CG1	2.77	0.61
3:M:478:ASN:O	3:M:479:PHE:C	2.37	0.61
1:A:273:LYS:O	1:A:276:PRO:HD2	2.01	0.61
1:A:630:PRO:CG	2:B:614:ILE:CG2	2.78	0.61
2:B:133:GLU:O	2:B:168:MET:HE1	2.00	0.61
2:B:534:ILE:C	2:B:536:ASN:N	2.53	0.61
3:M:121:ILE:O	3:M:125:PHE:HD1	1.81	0.61
3:M:265:ASN:HA	3:M:313:SER:OG	2.00	0.61
4:S:53:THR:CG2	4:S:57:LEU:CB	2.77	0.61
1:A:328:PRO:O	1:A:329:ASN:C	2.25	0.61
1:A:439:ASP:O	1:A:440:ASN:HB2	2.00	0.61
2:B:44:PRO:HB3	2:B:82:TYR:OH	2.00	0.61
2:B:178:ILE:HB	2:B:214:ALA:CB	2.29	0.61
2:B:340:ILE:HG12	2:B:373:LEU:HD23	1.82	0.61
2:B:355:ASN:O	2:B:359:LEU:CD2	2.48	0.61
2:B:498:THR:HG21	2:B:532:ARG:HG3	1.82	0.61
2:B:556:LEU:HD11	2:B:592:TYR:HB2	1.82	0.61
3:M:244:VAL:HG13	3:M:472:TYR:OH	1.99	0.61
3:M:443:SER:CB	3:M:447:ILE:CA	2.79	0.61

Continued on next page...

Continued from previous page...

Atom-1	Atom-2	Interatomic distance (Å)	Clash overlap (Å)
1:A:104:ARG:HG3	1:A:145:ILE:HD12	1.81	0.61
1:A:224:GLU:CD	4:S:138:GLY:O	2.39	0.61
1:A:266:VAL:O	1:A:267:GLU:HB2	2.01	0.61
1:A:384:LEU:HG	1:A:385:LYS:N	2.15	0.61
1:A:573:GLU:O	1:A:577:VAL:HG23	2.00	0.61
2:B:101:ILE:O	2:B:104:TYR:HB3	2.00	0.61
2:B:158:VAL:O	2:B:195:ILE:HD13	1.99	0.61
2:B:162:VAL:C	2:B:164:ASP:H	2.04	0.61
2:B:237:ILE:CG1	2:B:309:LEU:HD21	2.31	0.61
2:B:267:ASP:N	2:B:289:PRO:HG3	2.14	0.61
2:B:353:GLN:CB	3:M:47:SER:O	2.48	0.61
3:M:451:ALA:O	3:M:452:ILE:HD12	2.00	0.61
4:S:108:SER:O	4:S:112:CYS:SG	2.58	0.61
1:A:369:SER:HB2	1:A:424:TYR:HE2	1.66	0.61
1:A:376:GLU:H	1:A:376:GLU:CD	2.03	0.61
1:A:634:ASN:HA	2:B:516:GLY:HA3	1.83	0.61
2:B:127:LEU:HD23	2:B:135:ARG:O	2.01	0.61
2:B:274:PRO:HD2	2:B:295:ASN:ND2	2.13	0.61
2:B:498:THR:HG22	2:B:532:ARG:CB	2.31	0.61
3:M:118:TYR:C	3:M:118:TYR:CD2	2.73	0.61
3:M:215:TYR:HE1	3:M:468:LYS:HG2	1.60	0.61
3:M:461:GLY:C	3:M:462:LYS:O	2.23	0.61
1:A:100:LEU:CA	4:S:163:THR:HG23	2.27	0.61
1:A:166:LEU:O	1:A:170:LEU:CD2	2.48	0.61
1:A:180:LYS:HE3	4:S:156:LEU:CD2	2.29	0.61
1:A:186:PHE:CD1	1:A:224:GLU:HG2	2.35	0.61
1:A:244:LEU:O	1:A:246:THR:N	2.34	0.61
2:B:204:ASP:HB3	2:B:207:VAL:HG23	1.82	0.61
2:B:271:GLU:C	2:B:273:SER:N	2.44	0.61
2:B:339:PHE:O	2:B:343:LEU:CB	2.49	0.61
2:B:356:LYS:O	2:B:359:LEU:HB2	2.00	0.61
3:M:6:TYR:CA	3:M:16:PHE:O	2.49	0.61
3:M:217:ASP:HB3	3:M:471:LYS:CA	2.31	0.61
3:M:244:VAL:HG22	3:M:472:TYR:CE2	2.36	0.61
3:M:435:LEU:H	3:M:479:PHE:HB2	1.66	0.61
2:B:38:TYR:CD1	2:B:42:ILE:HA	2.35	0.61
2:B:127:LEU:HB2	2:B:157:THR:CG2	2.11	0.61
2:B:216:LYS:HB2	2:B:251:LEU:HD22	1.82	0.61
2:B:216:LYS:HG3	2:B:251:LEU:HA	1.83	0.61
2:B:362:ALA:HB1	2:B:366:LEU:HD11	1.81	0.61
2:B:512:VAL:C	2:B:551:LEU:CD1	2.46	0.61

Continued on next page...

Continued from previous page...

Atom-1	Atom-2	Interatomic distance (Å)	Clash overlap (Å)
3:M:9:ASP:CA	3:M:75:TRP:CD1	2.83	0.61
3:M:9:ASP:OD2	3:M:13:LYS:HB3	2.01	0.61
3:M:220:GLU:HG3	3:M:439:TYR:HB2	1.82	0.61
3:M:220:GLU:HG2	3:M:439:TYR:O	2.01	0.61
3:M:228:LYS:NZ	3:M:327:PHE:H	1.98	0.61
3:M:479:PHE:CD2	3:M:479:PHE:N	2.59	0.61
4:S:53:THR:CG2	4:S:68:VAL:N	2.62	0.61
1:A:582:ILE:HD11	1:A:608:ARG:CA	2.30	0.60
1:A:627:GLU:HG2	2:B:617:LEU:CA	1.96	0.60
2:B:486:HIS:NE2	2:B:518:ILE:CB	2.48	0.60
2:B:497:LEU:O	2:B:499:VAL:N	2.34	0.60
2:B:563:PHE:O	2:B:566:ALA:C	2.38	0.60
3:M:84:LYS:O	3:M:88:ASP:HB3	2.01	0.60
4:S:14:PRO:O	4:S:15:ARG:CD	2.49	0.60
4:S:25:LEU:CB	4:S:26:PRO:HD3	2.24	0.60
2:B:77:ILE:C	2:B:79:VAL:N	2.48	0.60
2:B:124:GLN:HA	2:B:127:LEU:HD12	1.82	0.60
2:B:178:ILE:CG1	2:B:214:ALA:CA	2.70	0.60
2:B:278:PRO:C	2:B:288:TYR:HB3	2.19	0.60
2:B:343:LEU:HD21	2:B:362:ALA:CB	2.18	0.60
2:B:354:GLY:O	2:B:358:MET:CG	2.47	0.60
2:B:435:SER:C	2:B:437:SER:H	2.04	0.60
2:B:602:ASP:O	2:B:608:ARG:NE	2.33	0.60
3:M:380:ARG:CZ	3:M:412:ARG:HD2	2.31	0.60
4:S:47:GLN:OE1	4:S:84:TYR:HD2	1.84	0.60
1:A:111:SER:HB3	1:A:152:THR:OG1	1.91	0.60
1:A:288:THR:CA	1:A:291:ILE:HD13	2.30	0.60
1:A:309:PHE:CE1	1:A:348:PHE:CZ	2.89	0.60
2:B:132:SER:HB2	2:B:169:VAL:HG23	1.82	0.60
2:B:279:LEU:HD12	2:B:288:TYR:CE1	2.35	0.60
2:B:393:ILE:HG23	2:B:431:MET:HB2	1.66	0.60
3:M:60:LEU:CD2	3:M:62:VAL:CG2	2.56	0.60
3:M:220:GLU:HG3	3:M:439:TYR:CB	2.31	0.60
4:S:6:LEU:CD1	4:S:32:LEU:HD13	2.32	0.60
1:A:186:PHE:CD2	1:A:186:PHE:C	2.75	0.60
1:A:270:LEU:HD12	1:A:274:LEU:HD23	1.84	0.60
1:A:436:CYS:SG	1:A:450:TYR:CE1	2.94	0.60
1:A:631:SER:CB	2:B:557:SER:HB3	2.12	0.60
2:B:47:LEU:HD22	2:B:66:ILE:HG13	0.63	0.60
2:B:57:ARG:O	2:B:60:ARG:HB3	2.01	0.60
2:B:90:ILE:HG12	2:B:98:LYS:CD	2.31	0.60

Continued on next page...

Continued from previous page...

Atom-1	Atom-2	Interatomic distance (Å)	Clash overlap (Å)
2:B:103:LEU:HD13	3:M:132:GLY:CA	2.28	0.60
2:B:178:ILE:HG23	2:B:218:CYS:H	1.63	0.60
2:B:231:ARG:O	2:B:233:TYR:N	2.34	0.60
2:B:303:LEU:CD2	2:B:325:LEU:HD23	2.31	0.60
3:M:65:TYR:CG	3:M:86:PRO:HB3	2.35	0.60
3:M:68:VAL:O	3:M:68:VAL:HG12	2.01	0.60
3:M:364:VAL:C	3:M:367:ALA:O	2.39	0.60
4:S:10:LYS:HA	4:S:84:TYR:CE1	2.36	0.60
1:A:156:PRO:O	1:A:157:SER:C	2.30	0.60
1:A:399:ASP:O	1:A:420:ILE:HB	2.01	0.60
1:A:624:LEU:HA	2:B:617:LEU:HD21	1.84	0.60
1:A:633:PHE:HE1	2:B:513:TRP:HE3	0.80	0.60
2:B:136:CYS:SG	2:B:168:MET:HG2	2.41	0.60
2:B:154:ILE:CG2	2:B:176:ALA:HB1	2.31	0.60
2:B:197:LYS:HA	2:B:229:HIS:HD2	1.53	0.60
2:B:219:TYR:HB3	2:B:223:LEU:HD23	0.61	0.60
2:B:319:LEU:CD1	2:B:358:MET:HG2	2.12	0.60
2:B:341:GLU:CG	2:B:345:ARG:HE	2.13	0.60
2:B:546:CYS:SG	2:B:607:ILE:HA	2.42	0.60
2:B:553:ALA:CB	2:B:614:ILE:HG21	2.28	0.60
2:B:589:SER:HG	2:B:618:PHE:HE2	1.43	0.60
2:B:592:TYR:CE2	2:B:618:PHE:CE2	2.89	0.60
1:A:121:LEU:HD11	1:A:155:THR:CG2	2.30	0.60
1:A:360:LEU:HD21	1:A:375:VAL:HG21	1.83	0.60
1:A:403:LEU:HD22	1:A:422:GLU:OE2	2.01	0.60
1:A:586:GLU:O	1:A:587:ASN:O	2.17	0.60
2:B:25:VAL:N	2:B:35:TYR:CD2	2.70	0.60
2:B:178:ILE:C	2:B:180:LEU:N	2.45	0.60
2:B:195:ILE:C	2:B:197:LYS:H	2.03	0.60
2:B:351:GLU:H	2:B:351:GLU:CD	2.04	0.60
2:B:374:PHE:HD2	2:B:402:LEU:HD11	1.63	0.60
2:B:417:TYR:O	2:B:421:SER:HB2	2.01	0.60
3:M:104:PHE:CE1	3:M:113:LYS:HE3	2.18	0.60
3:M:223:HIS:N	3:M:479:PHE:CE2	2.69	0.60
3:M:250:LEU:CD1	3:M:254:PRO:HG2	2.30	0.60
4:S:135:ILE:CG2	4:S:141:VAL:CG2	2.78	0.60
1:A:78:GLU:CD	1:A:113:SER:HB3	2.20	0.60
1:A:416:ILE:O	1:A:418:ILE:HG22	2.02	0.60
2:B:196:LEU:HB3	2:B:215:TYR:OH	2.02	0.60
2:B:208:ILE:CG2	2:B:236:ILE:HG21	2.31	0.60
2:B:431:MET:C	2:B:433:VAL:N	2.55	0.60

Continued on next page...

Continued from previous page...

Atom-1	Atom-2	Interatomic distance (Å)	Clash overlap (Å)
2:B:451:MET:HG3	2:B:489:ILE:HD11	1.83	0.60
2:B:582:ASP:O	2:B:584:SER:CA	2.49	0.60
3:M:235:LEU:HD23	3:M:235:LEU:C	2.22	0.60
1:A:88:ASN:O	1:A:90:HIS:N	2.33	0.60
1:A:96:SER:HB3	1:A:127:LEU:HD22	1.83	0.60
1:A:104:ARG:HG3	1:A:145:ILE:HG21	1.83	0.60
2:B:98:LYS:HD2	2:B:102:HIS:NE2	2.15	0.60
2:B:196:LEU:HA	2:B:199:LEU:HD12	1.83	0.60
2:B:493:LEU:HD21	2:B:511:ILE:HA	1.83	0.60
2:B:530:LEU:CD2	2:B:591:MET:CB	2.79	0.60
3:M:121:ILE:CG2	3:M:125:PHE:HE1	2.15	0.60
3:M:124:ILE:CG2	3:M:128:CYS:SG	2.90	0.60
3:M:271:SER:O	3:M:300:LEU:HA	2.01	0.60
3:M:454:ILE:HG22	3:M:464:THR:HG21	1.77	0.60
1:A:67:LYS:HD2	1:A:102:GLN:OE1	2.02	0.60
1:A:150:LEU:CD1	1:A:162:ILE:HG12	2.27	0.60
1:A:166:LEU:CD1	1:A:185:LEU:HD21	2.30	0.60
1:A:212:ILE:CD1	4:S:145:ASN:CG	2.62	0.60
1:A:292:TYR:O	1:A:295:VAL:HB	2.01	0.60
1:A:633:PHE:CG	2:B:550:VAL:CB	2.83	0.60
2:B:174:ALA:CB	2:B:211:ALA:HB2	2.30	0.60
2:B:274:PRO:N	2:B:295:ASN:HD21	1.99	0.60
2:B:286:ILE:O	2:B:286:ILE:HG23	2.00	0.60
2:B:562:ASN:HB3	2:B:580:TYR:O	2.02	0.60
3:M:99:ILE:HG22	3:M:103:TYR:CD1	2.37	0.60
3:M:247:ARG:N	3:M:470:ALA:HB2	2.17	0.60
1:A:139:ASP:C	1:A:177:ILE:CD1	2.69	0.60
1:A:251:TRP:HH2	4:S:103:GLN:CD	2.03	0.60
1:A:392:MET:HE3	1:A:428:MET:HE2	1.84	0.60
2:B:237:ILE:O	2:B:239:GLN:N	2.35	0.60
2:B:357:GLU:O	2:B:361:GLN:HG3	2.01	0.60
2:B:482:ASN:N	2:B:483:PRO:HD3	2.16	0.60
2:B:502:SER:O	2:B:503:LEU:C	2.35	0.60
2:B:549:LEU:CD2	2:B:611:ALA:HA	2.10	0.60
2:B:596:LEU:HD22	2:B:615:SER:HB2	1.83	0.60
3:M:225:VAL:HG13	3:M:480:GLN:HB3	1.83	0.60
3:M:226:PHE:O	3:M:482:ARG:N	2.29	0.60
1:A:170:LEU:CA	1:A:206:LYS:HD2	2.32	0.59
2:B:98:LYS:HD2	2:B:138:ALA:CB	2.29	0.59
2:B:146:LYS:C	2:B:147:MET:HG2	2.23	0.59
2:B:182:ARG:HG3	2:B:217:GLU:HB3	1.83	0.59

Continued on next page...

Continued from previous page...

Atom-1	Atom-2	Interatomic distance (Å)	Clash overlap (Å)
2:B:254:LYS:O	2:B:258:GLN:HG2	2.02	0.59
2:B:353:GLN:HE22	2:B:356:LYS:HD2	1.67	0.59
2:B:360:LEU:HB3	2:B:394:TRP:CB	2.31	0.59
2:B:394:TRP:CZ3	2:B:397:GLN:CD	2.75	0.59
2:B:544:THR:O	2:B:548:ILE:HG13	2.01	0.59
3:M:53:HIS:HB3	3:M:65:TYR:CZ	2.37	0.59
3:M:386:PHE:HB2	3:M:397:TRP:CD1	2.38	0.59
4:S:48:SER:CA	4:S:77:TYR:HB3	2.27	0.59
1:A:88:ASN:HB2	1:A:120:ILE:CD1	2.29	0.59
1:A:147:LEU:CG	1:A:166:LEU:CD2	2.80	0.59
1:A:630:PRO:C	2:B:554:LYS:HA	2.22	0.59
2:B:37:TYR:OH	2:B:46:GLN:NE2	0.65	0.59
2:B:80:GLN:CB	2:B:115:LEU:HD11	2.32	0.59
2:B:144:ASP:CA	2:B:179:LYS:HD3	2.32	0.59
2:B:215:TYR:O	2:B:219:TYR:HD1	1.85	0.59
2:B:412:PHE:HE2	2:B:446:TRP:CB	2.13	0.59
3:M:217:ASP:CG	3:M:471:LYS:CA	2.58	0.59
1:A:331:ARG:O	1:A:334:SER:N	2.35	0.59
1:A:533:ILE:HG13	1:A:562:TRP:CH2	2.36	0.59
2:B:143:SER:HB2	2:B:179:LYS:HD2	1.81	0.59
2:B:237:ILE:CG1	2:B:245:GLN:HG2	2.33	0.59
2:B:316:THR:HG21	3:M:90:PHE:HE2	0.87	0.59
2:B:563:PHE:O	2:B:566:ALA:N	2.35	0.59
3:M:212:ASN:CB	3:M:250:LEU:CD2	2.73	0.59
3:M:220:GLU:OE2	3:M:442:GLN:HB3	2.02	0.59
3:M:222:PHE:HD1	3:M:240:ILE:HG23	1.57	0.59
3:M:257:ALA:O	3:M:452:ILE:CG2	2.41	0.59
3:M:344:ILE:O	3:M:344:ILE:HG22	2.02	0.59
3:M:437:TYR:CB	3:M:439:TYR:CZ	2.84	0.59
1:A:134:TYR:O	1:A:135:ASP:C	2.36	0.59
1:A:145:ILE:O	1:A:148:SER:N	2.33	0.59
1:A:213:SER:C	4:S:143:GLU:CG	2.67	0.59
1:A:224:GLU:O	1:A:225:LEU:C	2.38	0.59
1:A:253:ILE:CG2	1:A:281:LEU:HB3	2.32	0.59
1:A:314:ALA:O	1:A:318:ARG:HD3	2.01	0.59
1:A:447:PHE:O	1:A:450:TYR:HB3	2.03	0.59
1:A:606:PHE:CD1	1:A:629:LEU:HG	2.37	0.59
1:A:607:LEU:O	1:A:610:SER:N	2.35	0.59
1:A:633:PHE:HD2	2:B:550:VAL:O	1.79	0.59
2:B:176:ALA:C	2:B:178:ILE:H	2.04	0.59
2:B:178:ILE:CB	2:B:214:ALA:C	2.71	0.59

Continued on next page...

Continued from previous page...

Atom-1	Atom-2	Interatomic distance (Å)	Clash overlap (Å)
2:B:297:PRO:O	2:B:301:LEU:CG	2.49	0.59
2:B:352:ASN:HB2	3:M:49:ASP:HB3	1.81	0.59
2:B:563:PHE:HE2	2:B:584:SER:HA	1.64	0.59
3:M:354:ASP:HA	3:M:401:LYS:HB3	1.83	0.59
3:M:374:TYR:OH	3:M:395:GLY:N	2.35	0.59
3:M:378:ILE:C	3:M:413:GLY:HA3	2.13	0.59
3:M:443:SER:HB3	3:M:447:ILE:CB	2.29	0.59
1:A:68:THR:OG1	4:S:165:SER:C	2.38	0.59
1:A:153:ILE:HB	1:A:158:LEU:HD21	1.85	0.59
1:A:219:VAL:HG12	1:A:259:LEU:HD12	1.84	0.59
1:A:584:PHE:C	1:A:587:ASN:H	2.02	0.59
1:A:630:PRO:HG2	2:B:614:ILE:CG2	2.33	0.59
2:B:64:LYS:HG3	2:B:100:LEU:CD1	2.33	0.59
2:B:161:LEU:HB3	2:B:173:VAL:HG21	1.84	0.59
2:B:215:TYR:CD1	2:B:233:TYR:CZ	2.87	0.59
2:B:215:TYR:HD1	2:B:233:TYR:CZ	2.12	0.59
3:M:99:ILE:CG2	3:M:103:TYR:HE1	2.15	0.59
3:M:225:VAL:CA	3:M:480:GLN:O	2.51	0.59
3:M:278:ILE:HG23	3:M:278:ILE:O	2.02	0.59
3:M:323:MET:SD	3:M:342:LEU:CB	2.91	0.59
3:M:338:PHE:CE2	3:M:415:ILE:HG12	2.33	0.59
1:A:68:THR:OG1	4:S:167:ILE:N	2.36	0.59
1:A:125:THR:C	1:A:127:LEU:H	2.04	0.59
1:A:179:LYS:HD2	4:S:143:GLU:CB	2.30	0.59
1:A:244:LEU:CB	1:A:256:LEU:HD13	2.32	0.59
1:A:561:ASN:O	1:A:564:ASN:N	2.35	0.59
2:B:38:TYR:CZ	2:B:42:ILE:HG12	2.21	0.59
2:B:537:PHE:CZ	2:B:545:ARG:CD	2.85	0.59
3:M:220:GLU:CG	3:M:439:TYR:CD1	2.85	0.59
3:M:290:PHE:CD1	3:M:299:LEU:HD13	2.37	0.59
1:A:170:LEU:O	1:A:206:LYS:NZ	2.35	0.59
1:A:322:PHE:HD2	1:A:330:LEU:CD2	2.14	0.59
1:A:375:VAL:O	1:A:378:ILE:O	2.21	0.59
1:A:634:ASN:ND2	2:B:554:LYS:CB	2.49	0.59
2:B:117:LEU:HA	2:B:150:LEU:CD2	2.33	0.59
2:B:135:ARG:HH12	2:B:164:ASP:HB2	1.66	0.59
2:B:597:TYR:O	2:B:601:TYR:CD1	2.55	0.59
1:A:107:TYR:HE2	1:A:128:LEU:CD2	2.03	0.59
1:A:153:ILE:HB	1:A:158:LEU:CD2	2.33	0.59
1:A:291:ILE:HD12	1:A:291:ILE:H	1.67	0.59
1:A:404:GLN:OE1	2:B:7:ARG:NH2	2.35	0.59

Continued on next page...

Continued from previous page...

Atom-1	Atom-2	Interatomic distance (Å)	Clash overlap (Å)
1:A:556:VAL:CG2	1:A:603:VAL:HG13	2.33	0.59
1:A:621:LEU:HD13	1:A:621:LEU:C	2.23	0.59
2:B:24:ALA:HB1	2:B:35:TYR:CG	2.37	0.59
2:B:91:THR:HA	2:B:126:SER:HB2	1.85	0.59
2:B:197:LYS:CD	2:B:283:TYR:CE2	2.86	0.59
2:B:334:MET:C	2:B:336:ASN:H	2.06	0.59
3:M:3:LEU:CG	3:M:80:THR:O	2.49	0.59
3:M:104:PHE:CD1	3:M:117:ASN:ND2	2.71	0.59
3:M:240:ILE:HG22	3:M:444:ALA:HB1	1.85	0.59
3:M:467:TYR:CG	3:M:468:LYS:N	2.71	0.59
1:A:67:LYS:HB2	4:S:165:SER:CB	2.33	0.59
1:A:140:VAL:O	1:A:141:VAL:C	2.34	0.59
2:B:37:TYR:C	2:B:39:SER:N	2.55	0.59
2:B:50:LEU:HD23	2:B:62:ALA:CA	2.33	0.59
2:B:142:LEU:HD13	2:B:154:ILE:HG12	1.85	0.59
2:B:155:LEU:HD21	2:B:192:LEU:N	2.17	0.59
2:B:178:ILE:CB	2:B:214:ALA:CA	2.81	0.59
2:B:215:TYR:CE2	2:B:229:HIS:ND1	2.70	0.59
2:B:275:ARG:HG2	2:B:294:VAL:HG21	1.83	0.59
2:B:451:MET:CG	2:B:489:ILE:HG12	2.33	0.59
2:B:461:HIS:HB3	2:B:463:LEU:HD23	1.84	0.59
2:B:556:LEU:CD2	2:B:588:ILE:CD1	2.79	0.59
3:M:243:ILE:HG21	3:M:298:ARG:HG2	1.83	0.59
1:A:182:ILE:C	1:A:221:VAL:CG2	2.67	0.59
1:A:402:ILE:O	1:A:402:ILE:HG22	2.02	0.59
1:A:483:LYS:C	1:A:484:VAL:HG23	2.23	0.59
2:B:387:ASP:HB3	2:B:391:ALA:CB	2.32	0.59
2:B:389:ILE:HG23	2:B:427:ASN:HB2	1.82	0.59
2:B:592:TYR:C	2:B:592:TYR:CD1	2.76	0.59
3:M:244:VAL:HG22	3:M:472:TYR:HE2	1.66	0.59
4:S:38:LEU:HB3	4:S:51:LEU:HD13	1.83	0.59
1:A:260:PHE:CE2	1:A:274:LEU:CG	2.84	0.58
1:A:516:ILE:HD13	1:A:551:LEU:CB	2.32	0.58
2:B:90:ILE:HG12	2:B:98:LYS:CE	2.32	0.58
2:B:182:ARG:HD2	2:B:217:GLU:CD	2.22	0.58
2:B:257:LYS:HA	2:B:260:LEU:CG	2.33	0.58
2:B:360:LEU:O	2:B:363:ILE:HB	2.03	0.58
3:M:99:ILE:CG2	3:M:103:TYR:CE1	2.86	0.58
3:M:218:LEU:HG	3:M:244:VAL:CG2	2.29	0.58
3:M:225:VAL:HA	3:M:480:GLN:N	2.18	0.58
3:M:347:PHE:CZ	3:M:350:VAL:CG1	2.85	0.58

Continued on next page...

Continued from previous page...

Atom-1	Atom-2	Interatomic distance (Å)	Clash overlap (Å)
1:A:97:SER:H	4:S:166:LYS:NZ	2.02	0.58
1:A:176:TYR:CB	4:S:155:GLU:HG2	2.31	0.58
1:A:270:LEU:CD1	1:A:274:LEU:HD23	2.32	0.58
1:A:278:ILE:O	1:A:280:GLU:N	2.36	0.58
1:A:607:LEU:C	1:A:609:LEU:N	2.55	0.58
2:B:175:LEU:O	2:B:178:ILE:HB	2.03	0.58
2:B:263:PRO:HA	2:B:266:VAL:CG2	2.33	0.58
2:B:355:ASN:O	2:B:359:LEU:HD23	2.03	0.58
2:B:549:LEU:HD22	2:B:611:ALA:HB3	1.72	0.58
2:B:562:ASN:CB	2:B:580:TYR:HB2	2.30	0.58
3:M:6:TYR:CD2	3:M:16:PHE:O	2.56	0.58
3:M:245:ASP:O	3:M:246:VAL:HG22	2.03	0.58
3:M:290:PHE:HZ	3:M:297:PHE:CG	2.11	0.58
3:M:410:VAL:HG13	3:M:412:ARG:HH11	1.66	0.58
4:S:8:PHE:CE1	4:S:84:TYR:HB3	1.80	0.58
1:A:180:LYS:CE	4:S:156:LEU:HD11	2.33	0.58
1:A:223:CYS:SG	1:A:262:ASN:ND2	2.66	0.58
1:A:638:LEU:HD12	2:B:518:ILE:HG22	1.77	0.58
2:B:87:VAL:CG2	2:B:119:SER:HA	2.33	0.58
2:B:120:ILE:C	2:B:153:ILE:HG21	2.23	0.58
2:B:177:ILE:CD1	2:B:196:LEU:CD2	2.82	0.58
2:B:278:PRO:HB3	2:B:288:TYR:C	2.16	0.58
2:B:534:ILE:O	2:B:535:GLN:C	2.38	0.58
2:B:537:PHE:CB	2:B:598:LEU:CD1	2.30	0.58
2:B:563:PHE:HA	2:B:566:ALA:HB3	1.84	0.58
2:B:569:THR:CA	2:B:571:SER:H	2.16	0.58
2:B:589:SER:OG	2:B:618:PHE:CZ	2.46	0.58
3:M:56:VAL:O	3:M:56:VAL:CG1	2.50	0.58
3:M:217:ASP:HB2	3:M:470:ALA:CA	2.33	0.58
3:M:235:LEU:HD13	3:M:306:LEU:HB3	1.85	0.58
3:M:245:ASP:N	3:M:472:TYR:CD2	2.57	0.58
3:M:443:SER:OG	3:M:448:TYR:N	2.35	0.58
1:A:229:ASN:O	1:A:230:PRO:C	2.36	0.58
1:A:533:ILE:HG12	1:A:562:TRP:CZ2	2.39	0.58
1:A:566:PHE:HD1	1:A:570:LYS:HA	1.68	0.58
2:B:120:ILE:HG22	2:B:153:ILE:CG2	2.33	0.58
2:B:181:TYR:CD2	2:B:218:CYS:CA	2.85	0.58
2:B:237:ILE:CD1	2:B:309:LEU:HD21	2.34	0.58
2:B:308:CYS:O	2:B:311:TYR:N	2.37	0.58
2:B:588:ILE:CG2	2:B:618:PHE:CE1	2.86	0.58
2:B:588:ILE:CG2	2:B:618:PHE:CZ	2.78	0.58

Continued on next page...

Continued from previous page...

Atom-1	Atom-2	Interatomic distance (Å)	Clash overlap (Å)
3:M:9:ASP:HA	3:M:75:TRP:HD1	1.67	0.58
3:M:19:LEU:CD1	3:M:24:ALA:CB	2.80	0.58
3:M:442:GLN:OE1	3:M:442:GLN:HA	2.02	0.58
4:S:53:THR:N	4:S:69:ASN:CB	2.58	0.58
2:B:51:LEU:HD22	2:B:59:VAL:HG13	1.84	0.58
2:B:497:LEU:HD11	2:B:508:ARG:CZ	2.31	0.58
2:B:501:THR:C	2:B:508:ARG:HH21	2.06	0.58
3:M:215:TYR:CB	3:M:469:GLY:H	2.13	0.58
3:M:219:LEU:HA	3:M:440:ILE:HA	1.85	0.58
1:A:190:LEU:HD11	1:A:228:LYS:HE3	1.85	0.58
1:A:338:PHE:HE2	1:A:352:PHE:CZ	2.21	0.58
1:A:634:ASN:OD1	2:B:553:ALA:C	2.41	0.58
2:B:120:ILE:HG21	2:B:154:ILE:HG13	1.82	0.58
2:B:177:ILE:HD11	2:B:195:ILE:HG22	1.84	0.58
2:B:299:LEU:C	2:B:299:LEU:HD13	2.24	0.58
2:B:452:LYS:NZ	2:B:456:ASP:CG	2.55	0.58
3:M:317:MET:CG	3:M:320:ILE:O	2.51	0.58
3:M:354:ASP:HB2	3:M:440:ILE:CD1	2.33	0.58
3:M:360:LEU:CD2	3:M:362:PHE:HE2	2.14	0.58
1:A:581:LEU:HG	1:A:607:LEU:HD11	1.86	0.58
2:B:29:LYS:O	2:B:32:GLU:CG	2.50	0.58
2:B:50:LEU:CD2	2:B:62:ALA:HB2	2.34	0.58
2:B:133:GLU:O	2:B:168:MET:CE	2.51	0.58
2:B:174:ALA:CB	2:B:211:ALA:HB1	2.31	0.58
2:B:188:TYR:O	2:B:192:LEU:HD13	2.02	0.58
2:B:316:THR:HG23	3:M:90:PHE:CZ	2.39	0.58
2:B:346:THR:CG2	2:B:350:THR:HG21	2.29	0.58
3:M:380:ARG:NH1	3:M:412:ARG:HD2	2.19	0.58
1:A:488:ARG:CG	1:A:522:PHE:CD2	2.87	0.58
2:B:29:LYS:O	2:B:30:LEU:C	2.40	0.58
2:B:37:TYR:HH	2:B:46:GLN:NE2	0.27	0.58
3:M:1:MET:HA	3:M:81:SER:OG	2.04	0.58
3:M:437:TYR:HB2	3:M:439:TYR:CE1	2.38	0.58
4:S:55:PRO:HB3	4:S:71:GLU:HA	1.86	0.58
4:S:117:ASN:HB2	4:S:120:ASP:OD2	2.04	0.58
1:A:402:ILE:C	1:A:404:GLN:N	2.56	0.58
2:B:30:LEU:HD12	2:B:30:LEU:O	2.04	0.58
2:B:37:TYR:CE2	2:B:46:GLN:OE1	2.56	0.58
2:B:83:PHE:CZ	2:B:105:LEU:HG	2.39	0.58
2:B:90:ILE:O	2:B:98:LYS:HE2	2.04	0.58
2:B:159:LYS:HG3	2:B:191:GLU:OE1	2.03	0.58

Continued on next page...

Continued from previous page...

Atom-1	Atom-2	Interatomic distance (Å)	Clash overlap (Å)
2:B:267:ASP:N	2:B:289:PRO:HB2	2.03	0.58
3:M:101:LEU:CD2	3:M:106:LYS:CG	2.81	0.58
3:M:218:LEU:N	3:M:218:LEU:HD12	2.18	0.58
3:M:242:GLY:O	3:M:301:GLU:HA	2.04	0.58
3:M:347:PHE:HE2	3:M:352:GLN:N	2.02	0.58
1:A:68:THR:CB	4:S:167:ILE:N	2.66	0.58
1:A:101:GLN:CG	4:S:167:ILE:HG13	2.31	0.58
1:A:102:GLN:HG2	4:S:166:LYS:CA	2.22	0.58
1:A:104:ARG:CG	1:A:145:ILE:HG21	2.34	0.58
1:A:322:PHE:CB	1:A:330:LEU:HD21	2.32	0.58
1:A:555:LEU:HD12	1:A:585:PHE:CE1	2.39	0.58
1:A:581:LEU:CD2	1:A:607:LEU:HD21	2.33	0.58
1:A:596:VAL:O	1:A:599:ARG:HB2	2.03	0.58
2:B:87:VAL:O	2:B:88:LYS:C	2.39	0.58
2:B:334:MET:CB	2:B:369:LEU:HD23	2.34	0.58
2:B:367:SER:OG	2:B:401:THR:CB	2.51	0.58
2:B:400:SER:CA	2:B:439:CYS:SG	2.91	0.58
2:B:403:ILE:HG22	2:B:411:ILE:CD1	2.33	0.58
1:A:189:PHE:HD2	1:A:225:LEU:HD11	1.62	0.57
1:A:204:VAL:HG13	1:A:239:LEU:HD11	1.86	0.57
1:A:384:LEU:HD13	1:A:435:ILE:HG23	1.80	0.57
1:A:438:ALA:O	1:A:441:TYR:CD1	2.56	0.57
1:A:464:ILE:HG23	1:A:465:SER:HA	1.86	0.57
1:A:516:ILE:HG12	1:A:551:LEU:CD1	2.34	0.57
1:A:625:LEU:O	1:A:626:SER:C	2.36	0.57
2:B:42:ILE:C	2:B:46:GLN:OE1	2.43	0.57
2:B:178:ILE:CD1	2:B:215:TYR:N	2.67	0.57
2:B:189:HIS:CE1	2:B:193:LEU:HD11	2.38	0.57
2:B:223:LEU:HD22	2:B:255:TYR:CZ	2.34	0.57
2:B:278:PRO:CG	2:B:289:PRO:O	2.51	0.57
2:B:350:THR:HB	2:B:352:ASN:HD22	1.69	0.57
2:B:470:ALA:O	2:B:473:ASN:N	2.35	0.57
2:B:508:ARG:O	2:B:509:ALA:C	2.30	0.57
2:B:519:ALA:HB2	2:B:555:LEU:HD13	1.86	0.57
2:B:534:ILE:O	2:B:536:ASN:N	2.37	0.57
3:M:435:LEU:HB2	3:M:437:TYR:CE1	2.39	0.57
1:A:241:TYR:HD2	1:A:242:GLU:N	2.01	0.57
1:A:355:LEU:O	1:A:359:LEU:HG	2.04	0.57
1:A:603:VAL:O	1:A:606:PHE:N	2.36	0.57
1:A:637:GLU:HG3	2:B:515:PHE:C	2.24	0.57
2:B:123:LEU:HD22	2:B:138:ALA:HB1	1.86	0.57

Continued on next page...

Continued from previous page...

Atom-1	Atom-2	Interatomic distance (Å)	Clash overlap (Å)
2:B:175:LEU:CD2	2:B:210:CYS:HB3	2.34	0.57
2:B:200:MET:CE	2:B:232:ARG:H	2.15	0.57
2:B:219:TYR:CA	2:B:223:LEU:CD2	2.82	0.57
2:B:247:TYR:OH	3:M:91:THR:CB	2.51	0.57
2:B:278:PRO:HD3	2:B:290:SER:HA	1.85	0.57
2:B:310:ILE:O	2:B:311:TYR:O	2.21	0.57
2:B:433:VAL:HG12	2:B:474:VAL:HG21	0.60	0.57
2:B:537:PHE:CZ	2:B:599:ALA:HA	2.39	0.57
3:M:52:ASP:CA	3:M:67:SER:CA	2.82	0.57
3:M:218:LEU:HG	3:M:472:TYR:CE2	2.32	0.57
3:M:380:ARG:N	3:M:412:ARG:O	2.37	0.57
3:M:440:ILE:O	3:M:440:ILE:HG22	2.03	0.57
4:S:34:GLN:CD	4:S:58:LEU:HD21	2.17	0.57
4:S:107:GLU:HG2	4:S:146:VAL:HG21	1.85	0.57
1:A:92:LEU:HD13	1:A:123:LEU:CD1	2.27	0.57
1:A:101:GLN:O	4:S:167:ILE:CD1	2.53	0.57
1:A:189:PHE:CG	1:A:225:LEU:HD21	2.37	0.57
1:A:637:GLU:HB3	2:B:516:GLY:HA3	1.80	0.57
2:B:44:PRO:O	2:B:47:LEU:CB	2.48	0.57
2:B:178:ILE:CG2	2:B:214:ALA:CA	2.83	0.57
2:B:178:ILE:CA	2:B:218:CYS:HB2	2.18	0.57
2:B:189:HIS:NE2	2:B:222:HIS:HB3	2.20	0.57
2:B:306:LEU:HD22	2:B:321:CYS:SG	2.45	0.57
2:B:472:VAL:HG13	2:B:510:GLY:C	2.24	0.57
2:B:508:ARG:O	2:B:512:VAL:CG2	2.43	0.57
2:B:542:PRO:CA	2:B:602:ASP:CG	2.60	0.57
3:M:2:TYR:C	3:M:3:LEU:HD12	2.24	0.57
4:S:80:TYR:CE2	4:S:110:ASP:HB2	2.39	0.57
1:A:132:LEU:HD23	1:A:143:VAL:HG22	1.86	0.57
1:A:485:PRO:C	1:A:486:SER:O	2.39	0.57
1:A:492:ILE:HD11	1:A:522:PHE:HB3	1.85	0.57
1:A:627:GLU:O	1:A:630:PRO:HD2	2.05	0.57
2:B:64:LYS:CG	2:B:100:LEU:HD11	2.34	0.57
2:B:559:ASP:OD2	2:B:563:PHE:CE1	2.57	0.57
3:M:17:GLN:H	3:M:118:TYR:HE1	1.51	0.57
3:M:74:TYR:HD2	3:M:109:LEU:O	1.88	0.57
3:M:223:HIS:HB3	3:M:478:ASN:C	2.24	0.57
4:S:15:ARG:HD2	4:S:122:ILE:HG13	1.84	0.57
1:A:234:ILE:HG23	1:A:267:GLU:HG2	1.86	0.57
1:A:253:ILE:HG12	1:A:281:LEU:CG	2.35	0.57
1:A:594:PHE:HD2	2:B:474:VAL:HG23	1.70	0.57

Continued on next page...

Continued from previous page...

Atom-1	Atom-2	Interatomic distance (Å)	Clash overlap (Å)
2:B:134:LEU:O	2:B:136:CYS:N	2.37	0.57
2:B:158:VAL:HG21	2:B:177:ILE:HG13	1.86	0.57
2:B:162:VAL:HG21	2:B:195:ILE:CB	2.31	0.57
2:B:313:SER:O	2:B:315:PRO:HD3	2.04	0.57
2:B:396:ILE:CD1	2:B:418:TYR:HE2	2.11	0.57
3:M:374:TYR:CZ	3:M:390:ILE:HD13	2.40	0.57
4:S:8:PHE:HA	4:S:13:GLN:O	2.04	0.57
4:S:35:VAL:HB	4:S:77:TYR:OH	2.05	0.57
1:A:183:THR:HG21	4:S:134:GLU:HA	1.87	0.57
1:A:253:ILE:CG1	1:A:281:LEU:HD22	2.35	0.57
1:A:637:GLU:HB2	2:B:551:LEU:CD2	2.35	0.57
2:B:17:VAL:O	2:B:18:ILE:O	2.21	0.57
2:B:108:PHE:CZ	2:B:115:LEU:CD2	2.88	0.57
2:B:251:LEU:O	2:B:254:LYS:N	2.36	0.57
3:M:114:ILE:O	3:M:118:TYR:N	2.38	0.57
3:M:121:ILE:HG23	3:M:125:PHE:HE1	1.69	0.57
3:M:317:MET:HB2	3:M:322:LEU:CB	2.35	0.57
4:S:14:PRO:HB3	4:S:36:TYR:CE1	2.33	0.57
4:S:111:ARG:HB2	4:S:150:VAL:CG2	2.32	0.57
1:A:105:VAL:HG23	4:S:167:ILE:CG2	2.27	0.57
1:A:600:SER:C	1:A:602:GLU:N	2.56	0.57
1:A:630:PRO:HG2	2:B:614:ILE:HG23	1.86	0.57
1:A:637:GLU:HG2	2:B:516:GLY:N	1.95	0.57
2:B:21:GLU:CA	2:B:24:ALA:CB	2.58	0.57
2:B:64:LYS:N	2:B:100:LEU:HD13	2.19	0.57
2:B:70:MET:HE1	2:B:107:ARG:CA	2.31	0.57
2:B:155:LEU:C	2:B:157:THR:N	2.55	0.57
2:B:231:ARG:O	2:B:234:CYS:N	2.38	0.57
2:B:277:CYS:SG	2:B:292:GLU:OE2	2.62	0.57
2:B:360:LEU:HD13	2:B:391:ALA:CA	2.25	0.57
1:A:63:ASP:O	4:S:165:SER:CB	2.51	0.57
1:A:114:PHE:CD2	1:A:153:ILE:HG23	2.40	0.57
1:A:163:ALA:O	1:A:165:ASP:N	2.37	0.57
1:A:225:LEU:CD1	1:A:233:PHE:CE2	2.81	0.57
1:A:316:LEU:O	1:A:319:LEU:N	2.35	0.57
1:A:535:ILE:O	1:A:535:ILE:HG22	2.04	0.57
2:B:257:LYS:HA	2:B:260:LEU:HD21	1.86	0.57
2:B:275:ARG:HE	2:B:275:ARG:CA	2.17	0.57
2:B:340:ILE:HG21	2:B:373:LEU:HG	1.87	0.57
2:B:451:MET:CE	2:B:489:ILE:HG12	2.34	0.57
2:B:574:ASN:C	2:B:576:GLN:H	2.08	0.57

Continued on next page...

Continued from previous page...

Atom-1	Atom-2	Interatomic distance (Å)	Clash overlap (Å)
3:M:96:ILE:HD12	3:M:125:PHE:HA	1.86	0.57
3:M:104:PHE:HD1	3:M:117:ASN:ND2	2.02	0.57
3:M:235:LEU:HD22	3:M:307:SER:HA	1.86	0.57
4:S:15:ARG:HH11	4:S:122:ILE:HD11	1.57	0.57
4:S:55:PRO:HD3	4:S:71:GLU:HA	1.87	0.57
1:A:255:ARG:C	1:A:257:LEU:N	2.56	0.57
1:A:292:TYR:CD1	1:A:292:TYR:C	2.78	0.57
2:B:50:LEU:HD21	2:B:61:ASP:HB2	1.86	0.57
2:B:151:ALA:HA	2:B:180:LEU:HG	1.82	0.57
2:B:208:ILE:HG21	2:B:240:LEU:HD11	1.87	0.57
2:B:336:ASN:HB2	2:B:339:PHE:CE1	2.39	0.57
2:B:453:TRP:HE3	2:B:453:TRP:HA	1.70	0.57
2:B:519:ALA:HB1	2:B:555:LEU:CD1	2.35	0.57
3:M:308:SER:O	3:M:312:GLN:N	2.33	0.57
3:M:327:PHE:HE1	3:M:336:ASP:CG	2.07	0.57
1:A:94:VAL:O	1:A:95:MET:C	2.39	0.57
1:A:170:LEU:HB2	1:A:202:LYS:HG2	1.86	0.57
1:A:421:PRO:CB	1:A:424:TYR:CD1	2.88	0.57
1:A:465:SER:H	2:B:1:MET:CG	2.15	0.57
1:A:563:CYS:HB3	1:A:621:LEU:CD1	2.23	0.57
2:B:20:ARG:CD	2:B:21:GLU:N	2.68	0.57
2:B:60:ARG:CD	2:B:96:LYS:HG2	2.25	0.57
2:B:245:GLN:CB	2:B:309:LEU:HD11	2.34	0.57
2:B:310:ILE:HG23	2:B:318:ILE:HG23	1.87	0.57
2:B:399:LEU:O	2:B:400:SER:C	2.38	0.57
3:M:60:LEU:HD23	3:M:60:LEU:C	2.22	0.57
3:M:249:TYR:CE1	3:M:467:TYR:CZ	2.93	0.57
4:S:111:ARG:CB	4:S:150:VAL:CG2	2.83	0.57
1:A:67:LYS:N	4:S:165:SER:CB	2.44	0.56
1:A:253:ILE:CG1	1:A:281:LEU:HB3	2.34	0.56
2:B:80:GLN:HG3	2:B:115:LEU:HD21	1.87	0.56
2:B:143:SER:CB	2:B:179:LYS:HB2	2.35	0.56
2:B:197:LYS:CD	2:B:283:TYR:CD2	2.88	0.56
2:B:387:ASP:HB3	2:B:391:ALA:HB3	1.86	0.56
3:M:241:HIS:HB2	3:M:476:THR:HG22	1.84	0.56
3:M:302:TYR:CE2	3:M:304:VAL:HB	2.40	0.56
3:M:327:PHE:HE1	3:M:336:ASP:OD2	1.88	0.56
4:S:50:PHE:HB3	4:S:76:ILE:HA	1.86	0.56
4:S:93:GLU:HA	4:S:93:GLU:OE1	2.04	0.56
2:B:24:ALA:CB	2:B:35:TYR:CD1	2.84	0.56
2:B:275:ARG:N	2:B:295:ASN:CG	2.54	0.56

Continued on next page...

Continued from previous page...

Atom-1	Atom-2	Interatomic distance (Å)	Clash overlap (Å)
2:B:278:PRO:CB	2:B:292:GLU:OE1	2.52	0.56
2:B:355:ASN:C	2:B:359:LEU:CD2	2.73	0.56
2:B:498:THR:HG22	2:B:532:ARG:HB2	1.86	0.56
3:M:432:THR:HG23	3:M:432:THR:O	2.03	0.56
1:A:158:LEU:HG	1:A:162:ILE:HD11	1.86	0.56
1:A:166:LEU:O	1:A:170:LEU:HD22	2.05	0.56
1:A:186:PHE:CD1	1:A:224:GLU:CG	2.88	0.56
1:A:384:LEU:HG	1:A:385:LYS:H	1.70	0.56
1:A:404:GLN:OE1	2:B:7:ARG:CZ	2.53	0.56
2:B:41:ASN:CG	2:B:43:ASN:OD1	2.44	0.56
2:B:86:VAL:HG13	2:B:101:ILE:CG1	2.29	0.56
2:B:123:LEU:O	2:B:127:LEU:HD12	2.04	0.56
2:B:140:SER:N	2:B:172:GLU:OE1	2.37	0.56
2:B:181:TYR:CE2	2:B:218:CYS:O	2.52	0.56
2:B:199:LEU:O	2:B:200:MET:C	2.40	0.56
2:B:220:ALA:HA	2:B:258:GLN:HB3	1.88	0.56
2:B:436:LEU:HD12	2:B:454:LEU:HD21	1.87	0.56
2:B:461:HIS:O	2:B:463:LEU:N	2.35	0.56
2:B:468:LEU:O	2:B:472:VAL:HG23	2.06	0.56
3:M:66:PHE:HB3	3:M:77:LEU:HD13	1.83	0.56
3:M:96:ILE:CD1	3:M:125:PHE:HA	2.35	0.56
3:M:222:PHE:CE1	3:M:439:TYR:CE1	2.93	0.56
3:M:373:ALA:O	3:M:418:GLU:O	2.24	0.56
3:M:403:THR:CG2	3:M:407:THR:HG1	2.16	0.56
4:S:58:LEU:O	4:S:59:LEU:HB3	2.04	0.56
1:A:97:SER:O	1:A:98:ASN:C	2.37	0.56
1:A:99:LYS:HB3	4:S:163:THR:C	2.25	0.56
1:A:170:LEU:CB	1:A:206:LYS:HG3	2.34	0.56
1:A:214:VAL:N	4:S:143:GLU:OE1	2.39	0.56
1:A:477:PHE:HE2	1:A:481:MET:SD	2.29	0.56
2:B:21:GLU:C	2:B:24:ALA:HB3	2.25	0.56
2:B:37:TYR:HE2	2:B:38:TYR:HE1	1.35	0.56
2:B:278:PRO:CA	2:B:288:TYR:HB3	2.25	0.56
2:B:352:ASN:ND2	2:B:352:ASN:H	2.02	0.56
2:B:523:PHE:HD1	2:B:559:ASP:CG	2.09	0.56
2:B:556:LEU:HD23	2:B:588:ILE:HD11	1.88	0.56
2:B:559:ASP:C	2:B:563:PHE:HB2	2.26	0.56
2:B:599:ALA:C	2:B:601:TYR:H	2.08	0.56
3:M:222:PHE:CE1	3:M:240:ILE:HG23	2.28	0.56
3:M:428:VAL:O	3:M:430:LEU:N	2.39	0.56
4:S:17:VAL:CG2	4:S:19:PHE:CE1	2.81	0.56

Continued on next page...

Continued from previous page...

Atom-1	Atom-2	Interatomic distance (Å)	Clash overlap (Å)
1:A:141:VAL:CA	4:S:159:ALA:HB2	2.35	0.56
1:A:274:LEU:O	1:A:275:LEU:C	2.37	0.56
2:B:50:LEU:HD23	2:B:62:ALA:CB	2.35	0.56
2:B:98:LYS:CD	2:B:138:ALA:HB2	2.31	0.56
2:B:139:LEU:HD23	2:B:173:VAL:C	2.11	0.56
2:B:247:TYR:OH	3:M:91:THR:CG2	2.53	0.56
3:M:360:LEU:HD21	3:M:362:PHE:HE2	1.66	0.56
3:M:372:ILE:HD13	3:M:424:PHE:CE1	2.40	0.56
1:A:67:LYS:CB	4:S:166:LYS:N	2.69	0.56
1:A:101:GLN:CG	4:S:167:ILE:CG2	2.53	0.56
1:A:179:LYS:HE3	4:S:143:GLU:CA	2.33	0.56
1:A:397:ASP:O	1:A:418:ILE:CD1	2.53	0.56
3:M:5:PHE:HB2	3:M:125:PHE:CD2	2.40	0.56
1:A:208:ASP:OD1	1:A:239:LEU:HD22	2.05	0.56
1:A:275:LEU:HD12	1:A:308:ASP:CG	2.22	0.56
1:A:481:MET:SD	1:A:518:CYS:SG	3.04	0.56
1:A:556:VAL:HG21	1:A:603:VAL:CG1	2.36	0.56
2:B:102:HIS:O	2:B:103:LEU:O	2.24	0.56
2:B:108:PHE:CE2	2:B:112:ASP:HB3	2.41	0.56
2:B:134:LEU:C	2:B:136:CYS:N	2.57	0.56
2:B:175:LEU:HD21	2:B:210:CYS:CB	2.35	0.56
2:B:215:TYR:CG	2:B:226:LEU:CD1	2.88	0.56
2:B:277:CYS:HA	2:B:292:GLU:CA	2.34	0.56
2:B:560:ILE:CA	2:B:564:LYS:H	2.17	0.56
2:B:585:GLY:O	2:B:589:SER:N	2.29	0.56
3:M:67:SER:CB	3:M:90:PHE:HB2	2.34	0.56
3:M:219:LEU:HB2	3:M:472:TYR:C	2.14	0.56
3:M:348:LYS:CG	3:M:405:THR:HG22	2.23	0.56
1:A:121:LEU:HD23	1:A:121:LEU:C	2.24	0.56
1:A:186:PHE:CZ	1:A:224:GLU:HG2	2.40	0.56
1:A:215:VAL:HG22	1:A:243:ILE:HG13	1.87	0.56
1:A:401:VAL:HG23	1:A:418:ILE:CA	2.36	0.56
2:B:173:VAL:CG1	2:B:199:LEU:HD11	2.36	0.56
2:B:275:ARG:HA	2:B:275:ARG:HE	1.70	0.56
2:B:388:PRO:O	2:B:391:ALA:HB3	2.05	0.56
2:B:560:ILE:HA	2:B:563:PHE:HB2	1.88	0.56
3:M:49:ASP:CA	3:M:75:TRP:HH2	1.96	0.56
3:M:338:PHE:CE2	3:M:415:ILE:CD1	2.89	0.56
4:S:53:THR:CB	4:S:68:VAL:CA	2.84	0.56
1:A:179:LYS:HZ1	4:S:149:ILE:HG12	1.70	0.56
1:A:225:LEU:HB3	1:A:233:PHE:HE1	1.66	0.56

Continued on next page...

Continued from previous page...

Atom-1	Atom-2	Interatomic distance (Å)	Clash overlap (Å)
1:A:384:LEU:CG	1:A:385:LYS:N	2.68	0.56
2:B:81:LEU:O	2:B:82:TYR:C	2.35	0.56
2:B:178:ILE:CG1	2:B:215:TYR:CA	2.78	0.56
2:B:189:HIS:NE2	2:B:193:LEU:CD1	2.61	0.56
2:B:189:HIS:CD2	2:B:222:HIS:CB	2.87	0.56
2:B:226:LEU:HG	2:B:255:TYR:CE2	2.40	0.56
2:B:245:GLN:HG2	2:B:309:LEU:HD11	1.84	0.56
2:B:350:THR:CG2	2:B:352:ASN:HD21	2.18	0.56
2:B:374:PHE:HD2	2:B:402:LEU:HD21	1.71	0.56
2:B:578:PRO:HB3	2:B:579:PRO:CD	2.33	0.56
3:M:4:SER:O	3:M:79:SER:N	2.39	0.56
3:M:18:TYR:CE2	3:M:20:LEU:CD2	2.86	0.56
3:M:105:ASP:O	3:M:106:LYS:HB3	2.04	0.56
3:M:212:ASN:HB3	3:M:250:LEU:CD2	2.31	0.56
4:S:50:PHE:HA	4:S:75:ILE:O	2.06	0.56
4:S:68:VAL:O	4:S:75:ILE:HD12	2.06	0.56
1:A:88:ASN:HB3	1:A:120:ILE:HG23	1.87	0.56
1:A:107:TYR:CD1	1:A:146:ALA:HA	2.41	0.56
1:A:162:ILE:O	1:A:165:ASP:HB2	2.06	0.56
1:A:167:PHE:CD1	1:A:202:LYS:HB2	2.41	0.56
1:A:178:ARG:HB2	1:A:214:VAL:CG2	2.35	0.56
2:B:214:ALA:C	2:B:216:LYS:H	2.09	0.56
2:B:483:PRO:HA	2:B:486:HIS:HB2	1.88	0.56
2:B:519:ALA:O	2:B:523:PHE:N	2.39	0.56
3:M:223:HIS:HB3	3:M:479:PHE:N	2.21	0.56
1:A:155:THR:O	1:A:158:LEU:HB3	2.06	0.55
1:A:255:ARG:HD3	4:S:141:VAL:C	2.25	0.55
1:A:295:VAL:HG11	1:A:337:LEU:HD13	1.88	0.55
2:B:172:GLU:O	2:B:174:ALA:N	2.38	0.55
2:B:174:ALA:HB3	2:B:211:ALA:HB2	1.88	0.55
2:B:215:TYR:CD2	2:B:219:TYR:CE1	2.79	0.55
2:B:307:ASN:HD22	2:B:336:ASN:HD21	1.43	0.55
2:B:340:ILE:HG13	2:B:373:LEU:CG	2.36	0.55
3:M:101:LEU:HD13	3:M:109:LEU:HD13	1.88	0.55
3:M:262:THR:HG23	3:M:267:ILE:HG12	1.87	0.55
3:M:437:TYR:HD1	3:M:437:TYR:H	1.51	0.55
2:B:69:ILE:O	2:B:70:MET:C	2.36	0.55
2:B:512:VAL:HB	2:B:551:LEU:CD1	2.34	0.55
2:B:596:LEU:HD13	2:B:611:ALA:C	2.27	0.55
3:M:16:PHE:CE2	3:M:125:PHE:CE2	2.94	0.55
3:M:18:TYR:OH	3:M:126:ASN:HB2	2.05	0.55

Continued on next page...

Continued from previous page...

Atom-1	Atom-2	Interatomic distance (Å)	Clash overlap (Å)
3:M:216:VAL:HB	3:M:472:TYR:OH	2.06	0.55
3:M:224:VAL:O	3:M:479:PHE:HA	2.04	0.55
3:M:244:VAL:O	3:M:299:LEU:HB3	2.06	0.55
3:M:272:LEU:HD22	3:M:278:ILE:CG2	2.35	0.55
1:A:99:LYS:HG2	1:A:101:GLN:N	2.21	0.55
1:A:283:GLU:OE1	1:A:318:ARG:NH2	2.38	0.55
1:A:631:SER:C	2:B:554:LYS:HG3	2.24	0.55
2:B:10:SER:O	2:B:13:ASP:N	2.38	0.55
2:B:50:LEU:CG	2:B:58:GLU:O	2.51	0.55
2:B:139:LEU:HD22	2:B:173:VAL:HG13	1.89	0.55
2:B:267:ASP:CA	2:B:289:PRO:CG	2.84	0.55
2:B:537:PHE:C	2:B:539:ASN:H	2.09	0.55
3:M:300:LEU:O	3:M:300:LEU:HD12	2.07	0.55
4:S:15:ARG:NH2	4:S:118:GLU:CD	2.59	0.55
1:A:114:PHE:CD2	1:A:153:ILE:HG12	2.42	0.55
1:A:215:VAL:CG2	1:A:243:ILE:CG1	2.79	0.55
1:A:516:ILE:HG21	1:A:551:LEU:HA	1.88	0.55
2:B:108:PHE:CE2	2:B:115:LEU:CD2	2.90	0.55
2:B:116:THR:HG22	2:B:150:LEU:CG	2.36	0.55
2:B:120:ILE:HG13	2:B:150:LEU:CB	2.32	0.55
2:B:430:ILE:O	2:B:433:VAL:HB	2.07	0.55
3:M:253:ASN:N	3:M:254:PRO:CD	2.70	0.55
4:S:89:VAL:CG1	4:S:98:ILE:CG2	2.76	0.55
1:A:185:LEU:O	1:A:189:PHE:CD2	2.59	0.55
1:A:279:LEU:HA	1:A:282:MET:HG2	1.89	0.55
1:A:301:GLY:O	1:A:302:ASN:CB	2.52	0.55
1:A:441:TYR:O	1:A:442:SER:C	2.44	0.55
1:A:441:TYR:C	1:A:443:SER:N	2.58	0.55
1:A:606:PHE:CD2	1:A:629:LEU:HG	2.41	0.55
2:B:159:LYS:CD	2:B:191:GLU:OE1	2.55	0.55
2:B:225:LEU:HD11	2:B:283:TYR:CE1	2.39	0.55
3:M:65:TYR:CG	3:M:86:PRO:CB	2.89	0.55
3:M:215:TYR:O	3:M:246:VAL:CG1	2.55	0.55
3:M:443:SER:OG	3:M:447:ILE:HG13	2.05	0.55
1:A:96:SER:HB3	1:A:127:LEU:CG	2.37	0.55
1:A:136:GLY:O	1:A:139:ASP:CA	2.55	0.55
1:A:606:PHE:HB3	1:A:629:LEU:HD12	1.88	0.55
2:B:177:ILE:HD13	2:B:196:LEU:CG	2.34	0.55
2:B:453:TRP:HA	2:B:453:TRP:CE3	2.40	0.55
2:B:559:ASP:C	2:B:563:PHE:H	2.05	0.55
3:M:101:LEU:HD12	3:M:109:LEU:HD12	1.88	0.55

Continued on next page...

Continued from previous page...

Atom-1	Atom-2	Interatomic distance (Å)	Clash overlap (Å)
3:M:217:ASP:CB	3:M:471:LYS:CA	2.85	0.55
3:M:262:THR:HG22	3:M:264:GLY:HA2	1.88	0.55
3:M:262:THR:C	3:M:264:GLY:N	2.55	0.55
3:M:327:PHE:CD2	3:M:430:LEU:HD22	2.41	0.55
3:M:358:ILE:HB	3:M:397:TRP:HE3	1.70	0.55
4:S:75:ILE:HG23	4:S:86:THR:CG2	2.36	0.55
1:A:528:ASN:C	1:A:530:ASN:N	2.52	0.55
1:A:575:LYS:HE3	1:A:611:LEU:CD2	2.35	0.55
1:A:586:GLU:CB	1:A:604:LEU:CD1	2.84	0.55
1:A:609:LEU:HD13	1:A:609:LEU:C	2.26	0.55
2:B:175:LEU:HD11	2:B:210:CYS:CB	2.37	0.55
3:M:124:ILE:HG23	3:M:128:CYS:SG	2.47	0.55
3:M:131:ALA:O	3:M:132:GLY:C	2.42	0.55
3:M:376:ILE:CD1	3:M:415:ILE:HG12	2.37	0.55
4:S:21:THR:HB	4:S:22:PRO:CD	2.33	0.55
1:A:118:SER:O	1:A:122:MET:HG2	2.07	0.55
1:A:182:ILE:HD13	1:A:218:ALA:CA	2.36	0.55
1:A:398:GLU:O	1:A:420:ILE:CB	2.55	0.55
1:A:429:VAL:HG11	1:A:469:LEU:CD1	2.36	0.55
1:A:487:MET:O	1:A:488:ARG:C	2.43	0.55
1:A:624:LEU:O	2:B:617:LEU:CD1	2.54	0.55
2:B:60:ARG:O	2:B:63:MET:N	2.34	0.55
2:B:83:PHE:HE2	2:B:119:SER:N	2.03	0.55
2:B:158:VAL:HG12	2:B:195:ILE:HD13	1.87	0.55
2:B:159:LYS:N	2:B:195:ILE:HD13	2.22	0.55
2:B:286:ILE:O	2:B:287:GLU:C	2.45	0.55
2:B:323:ASN:O	2:B:327:GLN:HG2	2.07	0.55
3:M:2:TYR:OH	3:M:62:VAL:CG1	2.55	0.55
3:M:16:PHE:CE2	3:M:18:TYR:HB2	2.41	0.55
3:M:293:PRO:C	3:M:294:ASP:O	2.30	0.55
3:M:331:LEU:HD22	3:M:426:LYS:NZ	2.22	0.55
4:S:9:ASN:ND2	4:S:13:GLN:HG2	2.17	0.55
1:A:396:VAL:O	1:A:396:VAL:HG12	2.05	0.55
1:A:451:ASN:OD1	1:A:480:LEU:CD1	2.55	0.55
2:B:108:PHE:O	2:B:109:ALA:C	2.31	0.55
2:B:132:SER:C	2:B:169:VAL:CG2	2.76	0.55
2:B:450:VAL:O	2:B:453:TRP:HB2	2.07	0.55
2:B:546:CYS:N	2:B:607:ILE:CG2	2.70	0.55
3:M:100:LEU:HD22	3:M:101:LEU:H	1.71	0.55
3:M:222:PHE:CE2	3:M:439:TYR:CE1	2.94	0.55
3:M:222:PHE:HA	3:M:240:ILE:HA	1.88	0.55

Continued on next page...

Continued from previous page...

Atom-1	Atom-2	Interatomic distance (Å)	Clash overlap (Å)
3:M:223:HIS:N	3:M:479:PHE:CZ	2.74	0.55
3:M:242:GLY:CA	3:M:474:THR:HG21	2.27	0.55
3:M:269:ILE:C	3:M:302:TYR:CD1	2.80	0.55
4:S:50:PHE:CA	4:S:75:ILE:O	2.55	0.55
1:A:105:VAL:HG21	4:S:167:ILE:O	2.07	0.55
1:A:236:LEU:C	1:A:238:PRO:HD2	2.27	0.55
1:A:401:VAL:HG21	1:A:418:ILE:N	2.21	0.55
1:A:495:ILE:CG2	1:A:515:CYS:HB3	2.37	0.55
1:A:537:THR:HB	1:A:584:PHE:CZ	2.41	0.55
1:A:582:ILE:CG1	1:A:607:LEU:HB2	2.37	0.55
1:A:606:PHE:CZ	2:B:550:VAL:HG13	2.40	0.55
2:B:45:GLN:C	2:B:47:LEU:N	2.59	0.55
2:B:196:LEU:C	2:B:215:TYR:OH	2.46	0.55
2:B:226:LEU:HD12	2:B:226:LEU:O	2.07	0.55
2:B:277:CYS:CA	2:B:292:GLU:CG	2.80	0.55
2:B:392:SER:HB3	2:B:424:PHE:HE2	1.71	0.55
3:M:101:LEU:HD13	3:M:109:LEU:CD1	2.36	0.55
3:M:443:SER:HA	3:M:447:ILE:HG13	1.89	0.55
4:S:54:PRO:CA	4:S:57:LEU:HD13	2.37	0.55
1:A:461:CYS:C	1:A:463:ASP:N	2.51	0.54
2:B:70:MET:HB2	2:B:104:TYR:HE1	1.69	0.54
2:B:120:ILE:CD1	2:B:142:LEU:HD22	2.34	0.54
2:B:132:SER:CB	2:B:169:VAL:CG2	2.85	0.54
2:B:177:ILE:CD1	2:B:196:LEU:HG	2.33	0.54
2:B:193:LEU:O	2:B:195:ILE:CA	2.49	0.54
2:B:249:ILE:HD13	2:B:320:SER:HB2	1.87	0.54
2:B:306:LEU:CD1	2:B:325:LEU:CD2	2.85	0.54
2:B:418:TYR:HD1	2:B:424:PHE:CD1	2.22	0.54
2:B:534:ILE:CG1	2:B:595:VAL:HG23	2.37	0.54
4:S:134:GLU:O	4:S:137:GLN:HG2	2.07	0.54
1:A:105:VAL:CB	4:S:167:ILE:HA	2.37	0.54
1:A:151:SER:CB	1:A:187:LYS:CB	2.74	0.54
1:A:332:TYR:CE1	1:A:336:ILE:HD11	2.40	0.54
1:A:506:LYS:O	1:A:507:GLN:HB2	2.07	0.54
1:A:594:PHE:HD2	2:B:474:VAL:CG2	2.20	0.54
1:A:637:GLU:HG3	2:B:515:PHE:CA	2.37	0.54
2:B:90:ILE:CG1	2:B:98:LYS:CD	2.85	0.54
2:B:316:THR:CB	3:M:90:PHE:CZ	2.90	0.54
2:B:340:ILE:HG21	2:B:373:LEU:O	2.07	0.54
2:B:564:LYS:O	2:B:567:GLN:N	2.40	0.54
2:B:589:SER:HA	2:B:618:PHE:CE2	2.42	0.54

Continued on next page...

Continued from previous page...

Atom-1	Atom-2	Interatomic distance (Å)	Clash overlap (Å)
3:M:52:ASP:CA	3:M:67:SER:C	2.69	0.54
4:S:89:VAL:HG11	4:S:98:ILE:HG23	1.87	0.54
4:S:131:VAL:HG22	4:S:153:VAL:CG2	2.26	0.54
1:A:92:LEU:HD22	1:A:124:ALA:N	2.23	0.54
1:A:163:ALA:HA	1:A:199:ASN:HD21	1.72	0.54
1:A:185:LEU:HD22	1:A:189:PHE:CE1	2.43	0.54
2:B:248:LEU:O	2:B:252:LEU:CG	2.45	0.54
2:B:449:HIS:O	2:B:453:TRP:CG	2.60	0.54
3:M:101:LEU:CD1	3:M:109:LEU:HD12	2.36	0.54
1:A:101:GLN:O	4:S:167:ILE:HD11	2.08	0.54
1:A:320:HIS:ND1	1:A:352:PHE:CD2	2.76	0.54
1:A:606:PHE:O	1:A:609:LEU:HB3	2.07	0.54
2:B:18:ILE:H	2:B:18:ILE:HD13	1.70	0.54
2:B:28:SER:N	2:B:32:GLU:OE1	2.41	0.54
2:B:155:LEU:CD1	2:B:156:HIS:N	2.70	0.54
2:B:174:ALA:O	2:B:175:LEU:O	2.26	0.54
2:B:247:TYR:OH	3:M:91:THR:HB	2.08	0.54
2:B:415:LEU:O	2:B:418:TYR:N	2.33	0.54
2:B:563:PHE:CD2	2:B:584:SER:C	2.81	0.54
2:B:592:TYR:CG	2:B:593:ASN:N	2.75	0.54
3:M:250:LEU:CD1	3:M:254:PRO:HB2	2.38	0.54
3:M:380:ARG:NH1	3:M:412:ARG:HH11	2.05	0.54
4:S:25:LEU:O	4:S:28:GLN:HG3	2.08	0.54
1:A:170:LEU:HD12	1:A:206:LYS:CG	2.29	0.54
1:A:178:ARG:HH11	1:A:209:ASP:CB	2.10	0.54
1:A:182:ILE:HG21	1:A:218:ALA:N	2.22	0.54
1:A:418:ILE:O	1:A:418:ILE:HG12	2.05	0.54
1:A:505:ASN:O	1:A:506:LYS:C	2.43	0.54
2:B:28:SER:N	2:B:32:GLU:CD	2.55	0.54
2:B:127:LEU:CD2	2:B:157:THR:CG2	2.86	0.54
2:B:362:ALA:HB1	2:B:366:LEU:CD1	2.38	0.54
2:B:418:TYR:CE1	2:B:419:VAL:HA	2.41	0.54
2:B:505:ASP:OD1	2:B:541:GLY:HA3	2.08	0.54
3:M:218:LEU:CG	3:M:472:TYR:HE2	2.16	0.54
4:S:54:PRO:CB	4:S:57:LEU:HD13	2.37	0.54
1:A:129:LYS:CD	1:A:161:ASP:HB3	2.38	0.54
1:A:513:ARG:CB	1:A:550:VAL:HG11	2.37	0.54
2:B:41:ASN:HB3	2:B:43:ASN:CG	2.19	0.54
2:B:105:LEU:O	2:B:106:LEU:O	2.26	0.54
2:B:134:LEU:C	2:B:136:CYS:H	2.11	0.54
2:B:143:SER:HA	2:B:179:LYS:CB	2.32	0.54

Continued on next page...

Continued from previous page...

Atom-1	Atom-2	Interatomic distance (Å)	Clash overlap (Å)
2:B:144:ASP:CA	2:B:179:LYS:CD	2.84	0.54
2:B:451:MET:HG3	2:B:489:ILE:HG12	1.90	0.54
2:B:483:PRO:HA	2:B:486:HIS:CB	2.38	0.54
2:B:534:ILE:CD1	2:B:595:VAL:HG23	2.38	0.54
2:B:562:ASN:OD1	2:B:580:TYR:HB2	2.06	0.54
3:M:16:PHE:HE1	3:M:122:SER:HB2	1.71	0.54
3:M:65:TYR:CD2	3:M:86:PRO:CB	2.88	0.54
3:M:476:THR:OG1	3:M:477:GLY:N	2.34	0.54
1:A:100:LEU:HG	4:S:162:SER:N	2.18	0.54
1:A:150:LEU:CD2	1:A:158:LEU:HD11	2.37	0.54
1:A:428:MET:O	1:A:431:VAL:HB	2.07	0.54
1:A:495:ILE:HG23	1:A:515:CYS:HB3	1.89	0.54
1:A:532:LEU:O	1:A:533:ILE:O	2.26	0.54
2:B:159:LYS:CE	2:B:191:GLU:CD	2.75	0.54
2:B:170:ARG:NH1	2:B:198:GLU:C	2.61	0.54
2:B:178:ILE:CB	2:B:214:ALA:CB	2.84	0.54
2:B:197:LYS:O	2:B:198:GLU:C	2.42	0.54
2:B:451:MET:HG3	2:B:489:ILE:CG1	2.37	0.54
3:M:225:VAL:HG22	3:M:480:GLN:CB	2.38	0.54
3:M:265:ASN:O	3:M:266:ASP:C	2.44	0.54
1:A:132:LEU:HD23	1:A:169:MET:SD	2.47	0.54
1:A:255:ARG:C	1:A:257:LEU:H	2.11	0.54
1:A:524:THR:HG21	1:A:565:ASN:ND2	2.22	0.54
1:A:586:GLU:CA	1:A:604:LEU:HD12	2.38	0.54
1:A:602:GLU:CD	1:A:633:PHE:HE1	1.97	0.54
1:A:636:TYR:N	2:B:554:LYS:CD	2.63	0.54
1:A:638:LEU:O	2:B:518:ILE:HD12	2.08	0.54
2:B:117:LEU:CD2	2:B:149:SER:HG	2.07	0.54
2:B:121:ASN:O	2:B:124:GLN:CB	2.56	0.54
2:B:237:ILE:HG13	2:B:245:GLN:HG2	1.90	0.54
2:B:366:LEU:O	2:B:367:SER:O	2.25	0.54
2:B:397:GLN:NE2	2:B:431:MET:SD	2.81	0.54
2:B:418:TYR:CZ	2:B:432:ALA:HB2	2.30	0.54
2:B:455:ILE:O	2:B:459:GLU:N	2.31	0.54
3:M:10:THR:CA	3:M:75:TRP:HE1	2.20	0.54
3:M:101:LEU:HD11	3:M:108:LYS:N	2.23	0.54
4:S:56:SER:O	4:S:60:SER:HB3	1.98	0.54
1:A:102:GLN:O	4:S:167:ILE:CD1	2.56	0.54
1:A:105:VAL:HG22	4:S:167:ILE:HG23	1.81	0.54
1:A:143:VAL:CG1	1:A:169:MET:HB3	2.36	0.54
1:A:440:ASN:CB	1:A:442:SER:HB3	2.37	0.54

Continued on next page...

Continued from previous page...

Atom-1	Atom-2	Interatomic distance (Å)	Clash overlap (Å)
1:A:609:LEU:CG	1:A:628:VAL:CG1	2.85	0.54
2:B:79:VAL:C	2:B:108:PHE:HE1	2.12	0.54
2:B:120:ILE:HG22	2:B:153:ILE:CB	2.38	0.54
2:B:335:LYS:O	2:B:335:LYS:HG2	2.08	0.54
2:B:553:ALA:HB2	2:B:614:ILE:CG2	2.38	0.54
3:M:252:ASP:C	3:M:254:PRO:HD2	2.28	0.54
3:M:257:ALA:CB	3:M:455:VAL:CG2	2.85	0.54
3:M:271:SER:HB3	3:M:301:GLU:CG	2.37	0.54
3:M:293:PRO:CA	3:M:294:ASP:O	2.55	0.54
3:M:316:ARG:CG	3:M:322:LEU:HD13	2.37	0.54
3:M:317:MET:HB2	3:M:322:LEU:HB2	1.89	0.54
4:S:12:CYS:HB3	4:S:36:TYR:HB3	1.89	0.54
4:S:14:PRO:HA	4:S:36:TYR:CZ	2.36	0.54
4:S:136:VAL:O	4:S:140:MET:N	2.41	0.54
1:A:64:LEU:HB3	1:A:102:GLN:HE21	1.71	0.54
1:A:103:LYS:O	1:A:104:ARG:C	2.38	0.54
1:A:104:ARG:CG	4:S:126:GLN:OE1	2.47	0.54
1:A:288:THR:CB	1:A:291:ILE:HD13	2.37	0.54
1:A:322:PHE:HB3	1:A:330:LEU:CD2	2.38	0.54
2:B:18:ILE:O	2:B:23:ALA:HB3	2.07	0.54
2:B:155:LEU:CB	2:B:188:TYR:CD2	2.79	0.54
2:B:275:ARG:CB	2:B:291:TYR:HB3	2.13	0.54
2:B:498:THR:CG2	2:B:532:ARG:CB	2.86	0.54
3:M:243:ILE:HG13	3:M:473:LYS:O	2.07	0.54
3:M:372:ILE:O	3:M:372:ILE:HG22	2.07	0.54
1:A:254:ILE:O	1:A:257:LEU:HB2	2.09	0.53
1:A:384:LEU:CD1	1:A:435:ILE:CG2	2.74	0.53
1:A:384:LEU:CG	1:A:441:TYR:CE2	2.85	0.53
1:A:488:ARG:HD2	1:A:522:PHE:CG	2.43	0.53
2:B:124:GLN:OE1	2:B:153:ILE:HG23	2.07	0.53
2:B:158:VAL:HG12	2:B:195:ILE:CG2	2.27	0.53
2:B:275:ARG:HG3	2:B:294:VAL:CB	2.37	0.53
2:B:302:PHE:CE2	2:B:328:LEU:HD11	2.44	0.53
2:B:559:ASP:O	2:B:562:ASN:N	2.41	0.53
2:B:562:ASN:ND2	2:B:580:TYR:CG	2.77	0.53
3:M:215:TYR:CG	3:M:468:LYS:C	2.81	0.53
3:M:272:LEU:N	3:M:272:LEU:HD12	2.23	0.53
3:M:353:VAL:HG13	3:M:353:VAL:O	2.08	0.53
4:S:53:THR:OG1	4:S:68:VAL:N	2.40	0.53
1:A:220:SER:CB	4:S:141:VAL:C	2.75	0.53
1:A:304:LEU:HD22	1:A:344:ILE:CG2	2.36	0.53

Continued on next page...

Continued from previous page...

Atom-1	Atom-2	Interatomic distance (Å)	Clash overlap (Å)
1:A:356:ILE:HD13	1:A:374:LEU:HB3	1.89	0.53
1:A:356:ILE:O	1:A:359:LEU:N	2.37	0.53
2:B:185:LYS:HA	2:B:222:HIS:NE2	2.23	0.53
2:B:243:TRP:CH2	3:M:95:THR:N	2.76	0.53
2:B:302:PHE:O	2:B:306:LEU:HG	2.08	0.53
2:B:433:VAL:HG22	2:B:471:TYR:CE2	2.42	0.53
2:B:505:ASP:CA	2:B:544:THR:OG1	2.55	0.53
1:A:401:VAL:HG23	1:A:418:ILE:C	2.28	0.53
1:A:494:ASN:O	1:A:498:LEU:HG	2.08	0.53
1:A:522:PHE:C	1:A:524:THR:H	2.11	0.53
1:A:601:VAL:O	1:A:602:GLU:C	2.31	0.53
2:B:95:THR:OG1	2:B:133:GLU:OE1	2.24	0.53
2:B:189:HIS:HB2	2:B:222:HIS:CE1	2.43	0.53
2:B:237:ILE:CG2	2:B:305:SER:HB3	2.31	0.53
2:B:408:VAL:CG1	2:B:412:PHE:CD2	2.91	0.53
2:B:512:VAL:CB	2:B:551:LEU:HD12	2.36	0.53
2:B:596:LEU:HB2	2:B:615:SER:OG	2.08	0.53
3:M:215:TYR:CE2	3:M:469:GLY:N	2.67	0.53
1:A:561:ASN:O	1:A:562:TRP:C	2.44	0.53
2:B:209:SER:HB2	2:B:244:SER:OG	2.08	0.53
2:B:352:ASN:HD22	2:B:352:ASN:N	1.99	0.53
2:B:363:ILE:CG2	2:B:398:ILE:CG1	2.85	0.53
3:M:67:SER:OG	3:M:90:PHE:HD1	1.31	0.53
3:M:74:TYR:CD1	3:M:76:CYS:SG	3.02	0.53
3:M:217:ASP:O	3:M:472:TYR:CD1	2.62	0.53
3:M:220:GLU:HG2	3:M:439:TYR:HB2	1.89	0.53
3:M:374:TYR:HH	3:M:394:GLN:C	2.09	0.53
4:S:33:GLU:HA	4:S:36:TYR:HD1	1.73	0.53
1:A:80:TYR:CD1	1:A:82:PHE:HE2	2.26	0.53
1:A:182:ILE:HG23	1:A:203:PHE:HE2	1.72	0.53
1:A:498:LEU:HB3	1:A:504:ILE:CG1	2.37	0.53
1:A:610:SER:HG	1:A:625:LEU:HD13	1.72	0.53
2:B:2:VAL:HA	2:B:5:ILE:HD12	1.90	0.53
2:B:296:ASP:OD1	2:B:297:PRO:N	2.41	0.53
2:B:578:PRO:CB	2:B:579:PRO:HD3	2.29	0.53
2:B:586:SER:O	2:B:590:GLN:HG3	2.09	0.53
3:M:5:PHE:CB	3:M:125:PHE:HE2	2.21	0.53
3:M:276:VAL:HG22	3:M:299:LEU:HD12	1.90	0.53
3:M:354:ASP:CB	3:M:440:ILE:CD1	2.85	0.53
1:A:263:LEU:O	1:A:266:VAL:C	2.46	0.53
1:A:338:PHE:HE2	1:A:352:PHE:CE2	2.27	0.53

Continued on next page...

Continued from previous page...

Atom-1	Atom-2	Interatomic distance (Å)	Clash overlap (Å)
1:A:589:SER:HB3	1:A:601:VAL:HG22	1.91	0.53
2:B:182:ARG:N	2:B:218:CYS:HA	2.23	0.53
2:B:350:THR:CB	2:B:352:ASN:ND2	2.61	0.53
1:A:163:ALA:CB	1:A:199:ASN:ND2	2.62	0.53
1:A:171:ASN:OD1	1:A:202:LYS:NZ	2.38	0.53
1:A:252:ILE:CB	4:S:144:THR:OG1	2.54	0.53
1:A:408:ILE:CD1	1:A:410:TYR:CD1	2.90	0.53
1:A:585:PHE:CE2	1:A:603:VAL:HG11	2.44	0.53
2:B:83:PHE:CE1	2:B:105:LEU:HA	2.43	0.53
2:B:120:ILE:HG12	2:B:150:LEU:HD22	1.89	0.53
2:B:133:GLU:CA	2:B:168:MET:SD	2.96	0.53
2:B:136:CYS:CA	2:B:172:GLU:CB	2.86	0.53
2:B:184:GLY:O	2:B:188:TYR:CD1	2.62	0.53
2:B:523:PHE:CE1	2:B:580:TYR:CD2	2.58	0.53
3:M:99:ILE:HG22	3:M:103:TYR:CE1	2.44	0.53
3:M:276:VAL:HG21	3:M:299:LEU:HA	1.91	0.53
3:M:306:LEU:CD2	3:M:317:MET:HE3	2.38	0.53
1:A:104:ARG:CB	1:A:145:ILE:HG13	2.39	0.53
1:A:104:ARG:HD3	1:A:145:ILE:HG13	1.91	0.53
1:A:105:VAL:HG21	4:S:167:ILE:C	2.30	0.53
1:A:245:VAL:HG13	1:A:246:THR:HG23	1.90	0.53
1:A:609:LEU:CD2	1:A:628:VAL:CG1	2.85	0.53
1:A:637:GLU:HB2	2:B:513:TRP:CD1	2.43	0.53
2:B:108:PHE:CE2	2:B:115:LEU:HD23	2.44	0.53
2:B:136:CYS:O	2:B:139:LEU:N	2.40	0.53
2:B:151:ALA:N	2:B:152:PRO:CD	2.71	0.53
2:B:167:ALA:O	2:B:207:VAL:HG21	2.09	0.53
2:B:523:PHE:CE1	2:B:582:ASP:OD1	2.62	0.53
2:B:594:ALA:O	2:B:598:LEU:HG	2.07	0.53
3:M:74:TYR:CB	3:M:114:ILE:HD11	2.37	0.53
3:M:258:VAL:HG13	3:M:449:VAL:CG1	2.39	0.53
3:M:293:PRO:CB	3:M:294:ASP:O	2.55	0.53
4:S:45:ASP:C	4:S:47:GLN:H	2.12	0.53
1:A:88:ASN:CG	1:A:120:ILE:HG21	2.29	0.53
1:A:204:VAL:HG13	1:A:239:LEU:CD1	2.39	0.53
1:A:435:ILE:HG23	1:A:441:TYR:CE2	2.44	0.53
1:A:481:MET:CE	1:A:518:CYS:HB3	2.36	0.53
2:B:38:TYR:CD1	2:B:38:TYR:N	2.76	0.53
2:B:189:HIS:HB2	2:B:222:HIS:CD2	2.43	0.53
2:B:189:HIS:CG	2:B:222:HIS:CG	2.96	0.53
2:B:277:CYS:HB2	2:B:295:ASN:C	2.28	0.53

Continued on next page...

Continued from previous page...

Atom-1	Atom-2	Interatomic distance (Å)	Clash overlap (Å)
2:B:292:GLU:OE2	2:B:296:ASP:CG	2.46	0.53
2:B:464:SER:OG	2:B:467:VAL:HG23	2.08	0.53
3:M:7:ILE:CG2	3:M:74:TYR:HB2	2.39	0.53
3:M:220:GLU:HA	3:M:474:THR:HG21	1.91	0.53
3:M:263:MET:HG2	3:M:263:MET:O	2.09	0.53
1:A:101:GLN:HG3	4:S:167:ILE:HG21	1.80	0.53
1:A:125:THR:HG21	1:A:158:LEU:HA	1.90	0.53
1:A:463:ASP:CA	2:B:1:MET:SD	2.96	0.53
1:A:581:LEU:CG	1:A:607:LEU:HD11	2.38	0.53
2:B:80:GLN:HG2	2:B:115:LEU:HD21	1.90	0.53
2:B:306:LEU:CB	2:B:325:LEU:HD21	2.38	0.53
2:B:560:ILE:N	2:B:563:PHE:HB2	2.24	0.53
3:M:215:TYR:HB2	3:M:467:TYR:CG	2.42	0.53
3:M:242:GLY:N	3:M:444:ALA:HB2	2.23	0.53
4:S:1:MET:N	4:S:93:GLU:OE1	2.41	0.53
4:S:16:LEU:HD11	4:S:129:GLU:HG2	1.91	0.53
4:S:39:ILE:HD12	4:S:48:SER:HA	1.90	0.53
1:A:366:SER:O	1:A:370:LYS:CG	2.49	0.52
1:A:512:LEU:HD13	1:A:543:TYR:CE1	2.44	0.52
2:B:65:ARG:O	2:B:68:SER:N	2.34	0.52
2:B:87:VAL:HG22	2:B:119:SER:HA	1.89	0.52
2:B:100:LEU:O	2:B:103:LEU:HB2	2.10	0.52
2:B:132:SER:CA	2:B:169:VAL:HG23	2.38	0.52
2:B:155:LEU:HD21	2:B:192:LEU:H	1.75	0.52
2:B:196:LEU:HB3	2:B:215:TYR:HE2	1.62	0.52
2:B:400:SER:HA	2:B:439:CYS:SG	2.48	0.52
2:B:493:LEU:HD11	2:B:511:ILE:HG12	1.90	0.52
3:M:372:ILE:HD12	3:M:428:VAL:HG22	1.89	0.52
3:M:437:TYR:CE1	3:M:479:PHE:CD1	2.97	0.52
4:S:6:LEU:HD22	4:S:32:LEU:CD2	2.29	0.52
4:S:34:GLN:HB3	4:S:58:LEU:HD11	1.89	0.52
1:A:103:LYS:CB	1:A:107:TYR:CE1	2.87	0.52
1:A:557:LYS:HD3	2:B:605:PHE:CD2	2.44	0.52
1:A:588:LEU:O	1:A:589:SER:C	2.42	0.52
2:B:42:ILE:HG22	2:B:43:ASN:N	2.24	0.52
2:B:157:THR:HG22	2:B:158:VAL:N	2.24	0.52
4:S:76:ILE:O	4:S:86:THR:HA	2.09	0.52
1:A:140:VAL:HG13	1:A:176:TYR:HB3	1.86	0.52
1:A:204:VAL:HG23	1:A:236:LEU:HD21	1.80	0.52
1:A:309:PHE:CE1	1:A:348:PHE:CE2	2.97	0.52
1:A:400:VAL:HA	1:A:403:LEU:HD12	1.90	0.52

Continued on next page...

Continued from previous page...

Atom-1	Atom-2	Interatomic distance (Å)	Clash overlap (Å)
1:A:420:ILE:C	1:A:421:PRO:O	2.48	0.52
1:A:536:MET:O	1:A:537:THR:O	2.24	0.52
1:A:600:SER:C	1:A:602:GLU:H	2.11	0.52
2:B:36:THR:O	2:B:40:GLN:HG3	2.08	0.52
2:B:36:THR:CA	2:B:39:SER:OG	2.57	0.52
2:B:136:CYS:HB3	2:B:172:GLU:CG	2.39	0.52
2:B:181:TYR:C	2:B:218:CYS:HA	2.30	0.52
2:B:352:ASN:ND2	2:B:352:ASN:O	2.43	0.52
2:B:461:HIS:CB	2:B:463:LEU:HD23	2.39	0.52
2:B:519:ALA:CB	2:B:555:LEU:CD1	2.88	0.52
2:B:546:CYS:O	2:B:549:LEU:HB3	2.09	0.52
3:M:267:ILE:CD1	3:M:445:SER:O	2.57	0.52
4:S:9:ASN:OD1	4:S:13:GLN:CB	2.57	0.52
1:A:104:ARG:CB	1:A:145:ILE:HG21	2.37	0.52
1:A:244:LEU:HD11	1:A:281:LEU:HD13	1.88	0.52
1:A:401:VAL:HG23	1:A:418:ILE:N	2.24	0.52
2:B:123:LEU:HD23	2:B:138:ALA:HB1	1.91	0.52
2:B:132:SER:HB2	2:B:169:VAL:CG2	2.38	0.52
2:B:143:SER:HB2	2:B:179:LYS:HB2	1.91	0.52
2:B:170:ARG:CG	2:B:199:LEU:HD23	2.34	0.52
2:B:184:GLY:O	2:B:188:TYR:HD1	1.92	0.52
2:B:197:LYS:O	2:B:199:LEU:CA	2.56	0.52
2:B:256:CYS:O	2:B:260:LEU:HG	2.10	0.52
2:B:513:TRP:HA	2:B:551:LEU:HD13	1.60	0.52
2:B:542:PRO:HA	2:B:602:ASP:OD1	2.08	0.52
3:M:104:PHE:HD2	3:M:105:ASP:OD2	1.93	0.52
1:A:117:ASP:O	1:A:120:ILE:HB	2.09	0.52
1:A:251:TRP:CG	4:S:104:THR:OG1	2.53	0.52
1:A:581:LEU:HD23	1:A:607:LEU:HD13	1.92	0.52
1:A:589:SER:O	1:A:597:GLN:CG	2.45	0.52
2:B:94:ASP:O	2:B:134:LEU:CD2	2.57	0.52
2:B:98:LYS:HG3	2:B:102:HIS:CE1	2.44	0.52
2:B:175:LEU:CD2	2:B:210:CYS:CB	2.87	0.52
2:B:219:TYR:HD1	2:B:226:LEU:HD13	1.54	0.52
2:B:275:ARG:NE	2:B:275:ARG:CA	2.73	0.52
2:B:404:ASN:O	2:B:405:GLU:O	2.24	0.52
2:B:542:PRO:CB	2:B:602:ASP:OD1	2.58	0.52
2:B:566:ALA:CB	2:B:581:TYR:HA	2.39	0.52
3:M:7:ILE:O	3:M:15:ILE:N	2.41	0.52
3:M:53:HIS:CB	3:M:65:TYR:CE1	2.93	0.52
1:A:100:LEU:H	4:S:162:SER:HB2	0.43	0.52

Continued on next page...

Continued from previous page...

Atom-1	Atom-2	Interatomic distance (Å)	Clash overlap (Å)
1:A:212:ILE:CG1	4:S:145:ASN:HD22	2.21	0.52
1:A:431:VAL:O	1:A:434:SER:HB2	2.09	0.52
1:A:463:ASP:HA	2:B:1:MET:SD	2.49	0.52
1:A:528:ASN:O	1:A:529:GLY:O	2.27	0.52
1:A:554:ALA:O	1:A:557:LYS:HB2	2.10	0.52
2:B:21:GLU:HA	2:B:24:ALA:HB3	1.39	0.52
2:B:62:ALA:CB	2:B:66:ILE:HD11	2.37	0.52
2:B:92:THR:O	2:B:134:LEU:CD1	2.58	0.52
2:B:476:ARG:O	2:B:480:GLN:HG3	2.10	0.52
2:B:584:SER:O	2:B:585:GLY:O	2.27	0.52
3:M:7:ILE:CG1	3:M:76:CYS:SG	2.91	0.52
3:M:80:THR:O	3:M:81:SER:HB3	2.10	0.52
3:M:455:VAL:HG12	3:M:455:VAL:O	2.09	0.52
4:S:10:LYS:HD3	4:S:82:THR:HB	1.92	0.52
4:S:126:GLN:NE2	4:S:127:THR:OG1	2.42	0.52
1:A:348:PHE:HB3	1:A:352:PHE:HE1	1.74	0.52
1:A:426:ILE:HD13	1:A:466:ASP:OD2	2.09	0.52
1:A:630:PRO:O	2:B:554:LYS:CB	2.56	0.52
2:B:63:MET:CG	2:B:100:LEU:HB2	2.39	0.52
2:B:189:HIS:CD2	2:B:189:HIS:O	2.63	0.52
2:B:234:CYS:CB	2:B:301:LEU:HB3	2.40	0.52
2:B:243:TRP:HZ2	3:M:95:THR:N	1.90	0.52
2:B:295:ASN:O	2:B:296:ASP:CB	2.57	0.52
2:B:368:ILE:HD11	2:B:401:THR:HG22	1.91	0.52
2:B:596:LEU:O	2:B:599:ALA:N	2.42	0.52
3:M:5:PHE:CB	3:M:125:PHE:CE2	2.93	0.52
3:M:74:TYR:CD2	3:M:109:LEU:O	2.63	0.52
3:M:104:PHE:HZ	3:M:113:LYS:NZ	0.39	0.52
3:M:347:PHE:HE2	3:M:352:GLN:CA	2.22	0.52
4:S:4:ALA:CB	4:S:18:LYS:O	2.58	0.52
1:A:249:ASN:ND2	4:S:146:VAL:H	2.08	0.52
1:A:301:GLY:O	1:A:302:ASN:HB3	2.09	0.52
1:A:585:PHE:CD2	1:A:607:LEU:HD11	2.44	0.52
2:B:4:SER:O	2:B:8:ILE:HG13	2.09	0.52
2:B:123:LEU:CB	2:B:142:LEU:CD1	2.84	0.52
2:B:174:ALA:C	2:B:214:ALA:CB	2.77	0.52
2:B:189:HIS:CG	2:B:189:HIS:O	2.62	0.52
2:B:196:LEU:HB2	2:B:229:HIS:HE1	1.61	0.52
2:B:208:ILE:HD11	2:B:236:ILE:HG23	1.83	0.52
2:B:223:LEU:CD1	2:B:258:GLN:O	2.58	0.52
2:B:501:THR:HA	2:B:508:ARG:NH2	2.20	0.52

Continued on next page...

Continued from previous page...

Atom-1	Atom-2	Interatomic distance (Å)	Clash overlap (Å)
2:B:537:PHE:CZ	2:B:598:LEU:O	2.62	0.52
3:M:82:LYS:O	3:M:83:SER:C	2.48	0.52
3:M:270:PRO:HA	3:M:302:TYR:HD1	1.74	0.52
4:S:50:PHE:HA	4:S:76:ILE:HA	1.91	0.52
1:A:92:LEU:CD1	1:A:123:LEU:HD12	2.32	0.52
1:A:260:PHE:O	1:A:262:ASN:N	2.40	0.52
1:A:566:PHE:CZ	1:A:618:THR:O	2.63	0.52
1:A:637:GLU:CB	2:B:516:GLY:H	2.05	0.52
2:B:98:LYS:HG3	2:B:137:PHE:HB2	1.91	0.52
2:B:140:SER:HA	2:B:172:GLU:OE1	2.05	0.52
2:B:227:HIS:CE1	2:B:292:GLU:OE2	2.45	0.52
2:B:451:MET:HG3	2:B:489:ILE:HD13	1.88	0.52
2:B:500:GLN:HB2	2:B:503:LEU:HG	1.89	0.52
2:B:559:ASP:OD2	2:B:582:ASP:OD2	2.28	0.52
3:M:10:THR:CG2	3:M:11:LYS:N	2.73	0.52
3:M:44:ASP:C	3:M:46:SER:H	2.13	0.52
3:M:102:GLU:HA	3:M:106:LYS:HB2	1.91	0.52
3:M:443:SER:HB3	3:M:447:ILE:H	1.74	0.52
1:A:100:LEU:HB3	1:A:142:LYS:HG3	1.91	0.52
1:A:384:LEU:CD2	1:A:441:TYR:CD2	2.86	0.52
1:A:441:TYR:HD1	1:A:441:TYR:H	1.58	0.52
1:A:516:ILE:CD1	1:A:551:LEU:CD1	2.86	0.52
2:B:36:THR:O	2:B:40:GLN:N	2.43	0.52
2:B:139:LEU:CD2	2:B:172:GLU:O	2.55	0.52
2:B:216:LYS:HB2	2:B:251:LEU:CD2	2.40	0.52
2:B:307:ASN:CG	2:B:339:PHE:CE2	2.82	0.52
2:B:550:VAL:HA	2:B:614:ILE:HD11	1.91	0.52
3:M:222:PHE:CA	3:M:479:PHE:HZ	2.17	0.52
3:M:258:VAL:HG22	3:M:452:ILE:HG23	1.90	0.52
3:M:280:ASP:OD1	3:M:281:GLY:N	2.43	0.52
3:M:317:MET:O	3:M:322:LEU:HB3	2.10	0.52
3:M:368:ASP:O	3:M:371:GLU:HB2	2.10	0.52
4:S:83:LEU:HD11	4:S:116:VAL:CB	2.39	0.52
1:A:67:LYS:CB	4:S:166:LYS:CA	2.87	0.51
1:A:91:ILE:O	1:A:94:VAL:HB	2.10	0.51
1:A:102:GLN:CB	4:S:166:LYS:CB	2.56	0.51
1:A:320:HIS:CA	1:A:338:PHE:HZ	2.22	0.51
1:A:566:PHE:HZ	1:A:618:THR:CA	2.22	0.51
1:A:624:LEU:CA	2:B:617:LEU:HD21	2.40	0.51
2:B:70:MET:CE	2:B:107:ARG:HG3	2.23	0.51
2:B:196:LEU:HB2	2:B:229:HIS:HD1	1.73	0.51

Continued on next page...

Continued from previous page...

Atom-1	Atom-2	Interatomic distance (Å)	Clash overlap (Å)
2:B:344:VAL:CG1	2:B:381:PHE:CE1	2.93	0.51
2:B:403:ILE:CG2	2:B:411:ILE:HD12	2.39	0.51
2:B:537:PHE:C	2:B:539:ASN:N	2.62	0.51
3:M:100:LEU:C	3:M:109:LEU:HD21	2.30	0.51
3:M:379:LEU:N	3:M:379:LEU:CD1	2.73	0.51
4:S:57:LEU:CD1	4:S:57:LEU:N	2.73	0.51
1:A:140:VAL:CG2	1:A:177:ILE:CD1	2.88	0.51
1:A:187:LYS:O	1:A:190:LEU:HB3	2.09	0.51
1:A:190:LEU:HD12	1:A:228:LYS:HE3	1.91	0.51
1:A:224:GLU:HG2	4:S:138:GLY:HA2	1.88	0.51
1:A:298:ILE:HG13	1:A:311:THR:HG22	1.91	0.51
1:A:403:LEU:CD1	1:A:421:PRO:O	2.55	0.51
1:A:425:LYS:HB3	1:A:464:ILE:HD12	1.93	0.51
1:A:626:SER:O	2:B:614:ILE:HA	2.10	0.51
2:B:60:ARG:CD	2:B:96:LYS:CG	2.87	0.51
2:B:123:LEU:C	2:B:127:LEU:HD12	2.30	0.51
2:B:165:PRO:C	2:B:170:ARG:HE	2.12	0.51
2:B:173:VAL:CB	2:B:199:LEU:HD11	2.40	0.51
3:M:343:ASN:CB	3:M:408:VAL:HG13	2.40	0.51
4:S:47:GLN:OE1	4:S:84:TYR:CD2	2.63	0.51
1:A:71:VAL:HG12	1:A:105:VAL:CG1	2.31	0.51
1:A:185:LEU:HB3	1:A:189:PHE:CZ	2.46	0.51
2:B:47:LEU:HD23	2:B:62:ALA:O	2.11	0.51
2:B:81:LEU:C	2:B:83:PHE:H	2.13	0.51
2:B:108:PHE:CE1	2:B:115:LEU:HG	2.31	0.51
2:B:112:ASP:C	2:B:112:ASP:OD1	2.49	0.51
2:B:123:LEU:HB3	2:B:142:LEU:CD1	2.39	0.51
2:B:208:ILE:O	2:B:209:SER:C	2.48	0.51
2:B:246:SER:O	2:B:249:ILE:HB	2.09	0.51
2:B:393:ILE:HG21	2:B:431:MET:CE	2.41	0.51
3:M:6:TYR:HB3	3:M:14:LEU:HG	1.92	0.51
3:M:18:TYR:HE2	3:M:20:LEU:HD21	1.73	0.51
3:M:379:LEU:CD2	3:M:386:PHE:HD1	1.99	0.51
1:A:261:THR:O	1:A:264:SER:OG	2.17	0.51
1:A:624:LEU:O	1:A:627:GLU:HB2	2.11	0.51
2:B:24:ALA:CB	2:B:35:TYR:CG	2.92	0.51
2:B:132:SER:CB	2:B:166:SER:HB3	2.39	0.51
2:B:135:ARG:NH2	2:B:164:ASP:HB2	2.24	0.51
2:B:162:VAL:CG1	2:B:195:ILE:HA	2.37	0.51
2:B:313:SER:OG	3:M:301:GLU:OE2	2.20	0.51
2:B:344:VAL:CG1	2:B:381:PHE:HZ	2.12	0.51

Continued on next page...

Continued from previous page...

Atom-1	Atom-2	Interatomic distance (Å)	Clash overlap (Å)
2:B:553:ALA:HB2	2:B:614:ILE:HG21	1.91	0.51
4:S:87:PHE:HB2	4:S:102:ILE:HD11	1.92	0.51
1:A:144:GLY:CA	1:A:180:LYS:CB	2.82	0.51
1:A:418:ILE:O	1:A:419:ILE:HB	2.09	0.51
1:A:557:LYS:CG	2:B:605:PHE:CD2	2.81	0.51
1:A:579:LYS:O	1:A:582:ILE:HB	2.09	0.51
1:A:633:PHE:CD1	2:B:513:TRP:HZ3	2.16	0.51
1:A:638:LEU:HD11	2:B:518:ILE:HG23	1.88	0.51
2:B:15:ALA:O	2:B:17:VAL:HA	2.10	0.51
2:B:189:HIS:HB2	2:B:222:HIS:NE2	2.26	0.51
2:B:341:GLU:HG2	2:B:345:ARG:NE	2.22	0.51
2:B:360:LEU:CD1	2:B:391:ALA:CB	2.87	0.51
2:B:501:THR:CA	2:B:508:ARG:HH22	2.19	0.51
3:M:72:LEU:CD1	3:M:101:LEU:HD22	2.41	0.51
3:M:101:LEU:CD1	3:M:106:LYS:O	2.50	0.51
3:M:380:ARG:NH1	3:M:410:VAL:HG11	2.07	0.51
3:M:443:SER:HG	3:M:447:ILE:HG13	1.75	0.51
4:S:6:LEU:HD12	4:S:16:LEU:O	2.10	0.51
1:A:129:LYS:HD2	1:A:161:ASP:OD2	2.09	0.51
1:A:141:VAL:N	4:S:155:GLU:HB3	2.24	0.51
1:A:158:LEU:HG	1:A:162:ILE:CD1	2.40	0.51
1:A:399:ASP:C	1:A:420:ILE:HB	2.26	0.51
1:A:412:LYS:O	1:A:414:LYS:N	2.44	0.51
1:A:425:LYS:HD2	1:A:428:MET:HE3	1.92	0.51
1:A:563:CYS:CA	1:A:566:PHE:CD2	2.86	0.51
1:A:566:PHE:C	1:A:568:GLU:N	2.59	0.51
2:B:144:ASP:N	2:B:179:LYS:CD	2.71	0.51
2:B:216:LYS:CD	2:B:251:LEU:HA	2.41	0.51
2:B:227:HIS:CG	2:B:292:GLU:CD	2.84	0.51
2:B:408:VAL:HG12	2:B:412:PHE:CD2	2.45	0.51
2:B:522:GLU:O	2:B:522:GLU:CG	2.40	0.51
3:M:2:TYR:CZ	3:M:62:VAL:HG13	2.46	0.51
3:M:60:LEU:HD23	3:M:61:GLU:H	1.68	0.51
3:M:64:LYS:HG2	3:M:79:SER:O	2.10	0.51
3:M:235:LEU:CD2	3:M:307:SER:HA	2.41	0.51
3:M:478:ASN:O	3:M:479:PHE:O	2.29	0.51
4:S:87:PHE:CD2	4:S:102:ILE:HG12	2.45	0.51
1:A:105:VAL:HB	4:S:167:ILE:HA	1.93	0.51
1:A:195:ALA:O	1:A:199:ASN:ND2	2.44	0.51
1:A:295:VAL:CG1	1:A:319:LEU:HD11	2.28	0.51
1:A:450:TYR:CZ	1:A:476:GLN:HG3	2.39	0.51

Continued on next page...

Continued from previous page...

Atom-1	Atom-2	Interatomic distance (Å)	Clash overlap (Å)
1:A:522:PHE:C	1:A:524:THR:N	2.60	0.51
1:A:530:ASN:HD21	1:A:573:GLU:HB3	1.75	0.51
1:A:545:HIS:O	1:A:546:SER:O	2.28	0.51
2:B:189:HIS:CG	2:B:222:HIS:ND1	2.79	0.51
2:B:252:LEU:CG	2:B:302:PHE:CD1	2.88	0.51
2:B:530:LEU:HD23	2:B:591:MET:HB3	1.86	0.51
3:M:10:THR:N	3:M:75:TRP:CD1	2.76	0.51
3:M:101:LEU:CA	3:M:106:LYS:HA	2.40	0.51
3:M:218:LEU:CG	3:M:472:TYR:CE2	2.94	0.51
1:A:176:TYR:CG	4:S:155:GLU:CG	2.91	0.51
1:A:253:ILE:CD1	1:A:281:LEU:CB	2.79	0.51
1:A:537:THR:OG1	1:A:584:PHE:CE1	2.63	0.51
2:B:278:PRO:C	2:B:292:GLU:OE1	2.29	0.51
2:B:374:PHE:HA	2:B:377:TYR:HD1	1.75	0.51
2:B:454:LEU:O	2:B:457:HIS:HB2	2.09	0.51
2:B:463:LEU:O	2:B:468:LEU:CD1	2.58	0.51
3:M:3:LEU:N	3:M:3:LEU:CD1	2.73	0.51
3:M:220:GLU:CD	3:M:222:PHE:CE1	2.85	0.51
3:M:342:LEU:HD12	3:M:342:LEU:N	2.25	0.51
1:A:129:LYS:O	1:A:132:LEU:N	2.44	0.51
1:A:638:LEU:HD21	2:B:523:PHE:HB3	1.93	0.51
2:B:59:VAL:HG11	2:B:89:ASN:HB3	1.91	0.51
2:B:275:ARG:HB3	2:B:291:TYR:CG	2.45	0.51
2:B:560:ILE:CA	2:B:563:PHE:HB2	2.41	0.51
2:B:574:ASN:C	2:B:576:GLN:N	2.57	0.51
3:M:220:GLU:CG	3:M:439:TYR:HD1	2.15	0.51
3:M:224:VAL:C	3:M:480:GLN:H	2.07	0.51
3:M:340:LEU:HG	3:M:342:LEU:HD11	1.91	0.51
1:A:65:ASN:O	4:S:165:SER:CA	2.57	0.51
1:A:101:GLN:CD	4:S:167:ILE:CG2	2.77	0.51
1:A:582:ILE:CD1	1:A:608:ARG:CA	2.88	0.51
2:B:38:TYR:HA	2:B:42:ILE:CA	2.41	0.51
2:B:82:TYR:HB2	2:B:104:TYR:OH	2.10	0.51
2:B:196:LEU:HB3	2:B:229:HIS:HD1	1.75	0.51
2:B:294:VAL:HG13	2:B:295:ASN:N	2.26	0.51
2:B:336:ASN:CB	2:B:339:PHE:CE1	2.94	0.51
3:M:101:LEU:HD23	3:M:106:LYS:CG	2.35	0.51
3:M:245:ASP:O	3:M:246:VAL:CG2	2.59	0.51
3:M:350:VAL:HG22	3:M:442:GLN:OE1	2.11	0.51
4:S:10:LYS:HA	4:S:84:TYR:HE1	1.76	0.51
4:S:55:PRO:C	4:S:57:LEU:N	2.59	0.51

Continued on next page...

Continued from previous page...

Atom-1	Atom-2	Interatomic distance (Å)	Clash overlap (Å)
1:A:166:LEU:CB	1:A:185:LEU:HD21	2.42	0.50
1:A:219:VAL:C	1:A:259:LEU:HD13	2.23	0.50
1:A:300:LYS:C	1:A:302:ASN:N	2.57	0.50
1:A:438:ALA:O	1:A:441:TYR:CE1	2.64	0.50
1:A:606:PHE:CD2	1:A:629:LEU:CD1	2.78	0.50
2:B:63:MET:HG2	2:B:100:LEU:HB2	1.93	0.50
2:B:63:MET:CA	2:B:66:ILE:HD12	2.41	0.50
2:B:185:LYS:CG	2:B:222:HIS:NE2	2.66	0.50
2:B:219:TYR:HE1	2:B:226:LEU:HD13	1.12	0.50
2:B:389:ILE:O	2:B:393:ILE:HG13	2.10	0.50
3:M:258:VAL:CG2	3:M:452:ILE:CG2	2.86	0.50
3:M:348:LYS:CG	3:M:405:THR:CG2	2.85	0.50
3:M:376:ILE:HD13	3:M:415:ILE:HG23	1.93	0.50
1:A:83:ASP:CG	1:A:85:ALA:H	2.14	0.50
1:A:107:TYR:OH	1:A:128:LEU:HD23	2.11	0.50
1:A:137:ASN:HA	1:A:139:ASP:HB3	1.93	0.50
1:A:237:SER:N	1:A:238:PRO:CD	2.73	0.50
1:A:309:PHE:CZ	1:A:348:PHE:CZ	2.99	0.50
1:A:461:CYS:SG	1:A:469:LEU:CD2	2.95	0.50
2:B:29:LYS:CD	2:B:30:LEU:N	2.58	0.50
2:B:108:PHE:CD2	2:B:115:LEU:HB3	2.46	0.50
2:B:155:LEU:HD13	2:B:155:LEU:C	2.31	0.50
2:B:159:LYS:HA	2:B:195:ILE:HD13	1.82	0.50
2:B:159:LYS:N	2:B:195:ILE:CD1	2.73	0.50
2:B:281:ASP:C	2:B:283:TYR:N	2.64	0.50
2:B:343:LEU:HD11	2:B:359:LEU:HD13	1.86	0.50
2:B:562:ASN:O	2:B:581:TYR:HA	2.10	0.50
3:M:56:VAL:O	3:M:56:VAL:HG12	2.10	0.50
3:M:101:LEU:CG	3:M:106:LYS:HG3	2.42	0.50
3:M:222:PHE:CZ	3:M:439:TYR:CE1	3.00	0.50
3:M:300:LEU:HD12	3:M:300:LEU:C	2.31	0.50
3:M:383:HIS:CB	3:M:403:THR:OG1	2.56	0.50
4:S:16:LEU:HD22	4:S:125:TRP:HD1	1.76	0.50
1:A:68:THR:CA	4:S:166:LYS:C	2.65	0.50
1:A:103:LYS:C	1:A:107:TYR:CE1	2.85	0.50
1:A:147:LEU:HB2	1:A:184:ALA:HB2	1.92	0.50
1:A:260:PHE:O	1:A:261:THR:O	2.26	0.50
1:A:566:PHE:C	1:A:566:PHE:CD1	2.83	0.50
1:A:595:GLU:O	1:A:599:ARG:HG2	2.11	0.50
2:B:62:ALA:O	2:B:66:ILE:HD11	1.95	0.50
2:B:137:PHE:O	2:B:140:SER:CB	2.59	0.50

Continued on next page...

Continued from previous page...

Atom-1	Atom-2	Interatomic distance (Å)	Clash overlap (Å)
2:B:215:TYR:CB	2:B:226:LEU:HD13	2.39	0.50
2:B:219:TYR:OH	2:B:225:LEU:C	2.47	0.50
2:B:344:VAL:CG2	2:B:381:PHE:HZ	2.24	0.50
2:B:501:THR:CA	2:B:508:ARG:NH2	2.75	0.50
2:B:599:ALA:O	2:B:602:ASP:N	2.27	0.50
3:M:1:MET:C	3:M:81:SER:OG	2.50	0.50
3:M:254:PRO:CB	3:M:454:ILE:CD1	2.60	0.50
3:M:265:ASN:HB3	3:M:309:GLN:NE2	2.26	0.50
3:M:348:LYS:HA	3:M:405:THR:C	2.32	0.50
3:M:353:VAL:CG2	3:M:354:ASP:O	2.55	0.50
1:A:125:THR:HG23	1:A:158:LEU:HD12	1.94	0.50
1:A:178:ARG:CB	1:A:214:VAL:HG13	2.39	0.50
1:A:557:LYS:CD	2:B:605:PHE:HD2	2.24	0.50
1:A:559:PHE:CD2	1:A:578:LEU:CD2	2.94	0.50
1:A:581:LEU:CG	1:A:607:LEU:CD1	2.89	0.50
1:A:633:PHE:CD1	2:B:550:VAL:CB	2.93	0.50
2:B:127:LEU:C	2:B:161:LEU:HD11	2.31	0.50
2:B:136:CYS:CB	2:B:168:MET:HG2	2.42	0.50
2:B:175:LEU:CD1	2:B:210:CYS:SG	2.84	0.50
2:B:281:ASP:O	2:B:283:TYR:O	2.29	0.50
2:B:306:LEU:CD2	2:B:321:CYS:SG	3.00	0.50
2:B:337:THR:O	2:B:373:LEU:HD21	2.10	0.50
2:B:352:ASN:HB2	3:M:49:ASP:OD2	1.92	0.50
2:B:371:GLN:C	2:B:373:LEU:H	2.15	0.50
3:M:45:SER:HB3	3:M:51:LEU:CD1	2.40	0.50
3:M:51:LEU:HD13	3:M:75:TRP:CE3	2.43	0.50
3:M:100:LEU:CD1	3:M:100:LEU:N	2.73	0.50
3:M:246:VAL:CA	3:M:470:ALA:HB2	2.34	0.50
3:M:259:LYS:O	3:M:449:VAL:CA	2.53	0.50
3:M:339:GLU:CG	3:M:412:ARG:CG	2.53	0.50
4:S:34:GLN:HB2	4:S:58:LEU:HD11	1.94	0.50
4:S:97:ALA:O	4:S:101:LEU:HB2	2.12	0.50
1:A:371:ALA:O	1:A:374:LEU:HB2	2.12	0.50
1:A:488:ARG:CD	1:A:522:PHE:CD2	2.95	0.50
1:A:547:VAL:O	1:A:550:VAL:HB	2.12	0.50
1:A:581:LEU:CB	1:A:607:LEU:HD13	2.42	0.50
1:A:636:TYR:HB2	2:B:513:TRP:CZ2	2.46	0.50
2:B:25:VAL:HG22	2:B:35:TYR:CD2	2.44	0.50
2:B:68:SER:O	2:B:71:ALA:HB3	2.12	0.50
2:B:132:SER:CB	2:B:166:SER:CB	2.89	0.50
2:B:223:LEU:CB	2:B:259:TYR:CB	2.89	0.50

Continued on next page...

Continued from previous page...

Atom-1	Atom-2	Interatomic distance (Å)	Clash overlap (Å)
2:B:316:THR:CB	3:M:90:PHE:CE2	2.91	0.50
2:B:331:PRO:C	2:B:333:GLN:H	2.14	0.50
2:B:553:ALA:CB	2:B:614:ILE:HG23	2.42	0.50
2:B:560:ILE:HD11	2:B:618:PHE:HA	1.92	0.50
2:B:610:ARG:O	2:B:614:ILE:HG13	2.11	0.50
3:M:52:ASP:CB	3:M:67:SER:HA	2.42	0.50
3:M:52:ASP:HB3	3:M:67:SER:HA	1.93	0.50
1:A:96:SER:CB	1:A:127:LEU:HD13	2.18	0.50
1:A:254:ILE:HG21	4:S:100:ASP:OD2	2.12	0.50
1:A:488:ARG:HD2	1:A:522:PHE:CD2	2.47	0.50
1:A:492:ILE:HD11	1:A:522:PHE:CB	2.42	0.50
2:B:21:GLU:OE2	2:B:35:TYR:O	2.30	0.50
2:B:47:LEU:HD23	2:B:66:ILE:HG13	1.69	0.50
2:B:120:ILE:HG21	2:B:150:LEU:O	2.11	0.50
2:B:127:LEU:HA	2:B:135:ARG:HG2	1.93	0.50
2:B:245:GLN:HB3	2:B:309:LEU:CD1	2.41	0.50
2:B:355:ASN:O	2:B:359:LEU:HD22	2.12	0.50
2:B:411:ILE:O	2:B:414:GLU:N	2.23	0.50
2:B:475:ILE:HG23	2:B:489:ILE:HG21	1.93	0.50
3:M:18:TYR:CE2	3:M:20:LEU:HD21	2.47	0.50
3:M:51:LEU:CD1	3:M:51:LEU:N	2.73	0.50
3:M:338:PHE:HE2	3:M:415:ILE:HG12	1.75	0.50
3:M:374:TYR:H	3:M:390:ILE:CG2	2.25	0.50
4:S:70:ASN:CG	4:S:73:ILE:HD12	2.31	0.50
4:S:75:ILE:HG22	4:S:77:TYR:CZ	2.45	0.50
1:A:95:MET:HB2	1:A:127:LEU:HD23	1.92	0.50
1:A:96:SER:N	1:A:127:LEU:CG	2.66	0.50
1:A:101:GLN:HG2	4:S:167:ILE:HB	1.84	0.50
2:B:44:PRO:O	2:B:47:LEU:N	2.45	0.50
2:B:60:ARG:O	2:B:100:LEU:CD1	2.60	0.50
2:B:162:VAL:O	2:B:163:THR:C	2.35	0.50
2:B:267:ASP:CB	2:B:289:PRO:CG	2.90	0.50
2:B:461:HIS:HB2	2:B:463:LEU:CD2	2.42	0.50
2:B:577:ASN:C	2:B:578:PRO:O	2.49	0.50
3:M:215:TYR:CE1	3:M:468:LYS:CB	2.95	0.50
3:M:220:GLU:CD	3:M:222:PHE:CZ	2.85	0.50
3:M:224:VAL:O	3:M:479:PHE:C	2.49	0.50
4:S:16:LEU:HB2	4:S:125:TRP:CD1	2.47	0.50
4:S:17:VAL:CG1	4:S:19:PHE:CZ	2.95	0.50
4:S:75:ILE:HB	4:S:77:TYR:HE1	1.76	0.50
1:A:91:ILE:HG22	1:A:95:MET:HE2	1.93	0.50

Continued on next page...

Continued from previous page...

Atom-1	Atom-2	Interatomic distance (Å)	Clash overlap (Å)
1:A:92:LEU:HD13	1:A:123:LEU:HB2	1.93	0.50
1:A:141:VAL:CG1	4:S:159:ALA:CA	2.89	0.50
1:A:150:LEU:CD2	1:A:162:ILE:HG12	2.41	0.50
1:A:251:TRP:CE3	1:A:254:ILE:HD12	2.47	0.50
1:A:432:ILE:O	1:A:435:ILE:HB	2.11	0.50
2:B:162:VAL:HG23	2:B:173:VAL:HG11	1.94	0.50
2:B:252:LEU:CB	2:B:302:PHE:CD1	2.93	0.50
2:B:325:LEU:HB3	2:B:334:MET:HE1	1.93	0.50
2:B:512:VAL:O	2:B:515:PHE:HB2	2.12	0.50
3:M:380:ARG:NH1	3:M:412:ARG:NH1	2.60	0.50
1:A:78:GLU:C	1:A:80:TYR:O	2.50	0.50
1:A:145:ILE:HG12	4:S:156:LEU:CD2	2.37	0.50
1:A:200:PHE:CZ	1:A:232:PRO:O	2.62	0.50
1:A:497:LYS:O	1:A:501:ASN:N	2.44	0.50
1:A:556:VAL:HG22	1:A:603:VAL:HG13	1.93	0.50
1:A:573:GLU:O	1:A:575:LYS:N	2.45	0.50
2:B:143:SER:HB2	2:B:179:LYS:CG	2.42	0.50
2:B:144:ASP:N	2:B:179:LYS:HD2	2.22	0.50
2:B:197:LYS:CB	2:B:229:HIS:CD2	2.89	0.50
2:B:452:LYS:NZ	2:B:456:ASP:OD2	2.44	0.50
2:B:553:ALA:CA	2:B:614:ILE:HG23	2.39	0.50
3:M:115:VAL:O	3:M:116:ASN:C	2.46	0.50
3:M:375:LYS:CE	3:M:418:GLU:OE1	2.60	0.50
4:S:78:LYS:O	4:S:84:TYR:HA	2.12	0.50
1:A:166:LEU:O	1:A:170:LEU:HD23	2.12	0.49
1:A:212:ILE:CB	4:S:145:ASN:HD22	2.25	0.49
2:B:256:CYS:CA	2:B:293:VAL:HG21	2.31	0.49
2:B:560:ILE:HA	2:B:564:LYS:N	2.22	0.49
3:M:6:TYR:CG	3:M:14:LEU:HD11	2.46	0.49
3:M:235:LEU:HD23	3:M:235:LEU:O	2.11	0.49
3:M:256:VAL:HG22	3:M:452:ILE:HG23	1.92	0.49
3:M:373:ALA:CB	3:M:418:GLU:O	2.57	0.49
3:M:396:GLN:HG2	3:M:398:ILE:CD1	2.40	0.49
3:M:424:PHE:HZ	3:M:428:VAL:CG2	2.25	0.49
3:M:452:ILE:CD1	3:M:452:ILE:N	2.73	0.49
4:S:4:ALA:HB2	4:S:19:PHE:HD2	1.77	0.49
1:A:104:ARG:HB3	4:S:160:ALA:HB2	1.95	0.49
1:A:133:LYS:HE2	1:A:165:ASP:OD1	2.11	0.49
1:A:139:ASP:OD1	1:A:177:ILE:CD1	2.60	0.49
1:A:186:PHE:HD1	1:A:224:GLU:HB3	1.76	0.49
1:A:349:ILE:O	1:A:352:PHE:N	2.42	0.49

Continued on next page...

Continued from previous page...

Atom-1	Atom-2	Interatomic distance (Å)	Clash overlap (Å)
1:A:395:PHE:CD2	1:A:420:ILE:HD13	2.47	0.49
1:A:420:ILE:O	1:A:421:PRO:O	2.30	0.49
1:A:433:ILE:HD11	1:A:473:ILE:HA	1.94	0.49
1:A:461:CYS:O	1:A:462:GLN:O	2.30	0.49
1:A:549:GLU:O	1:A:553:LEU:HG	2.12	0.49
2:B:81:LEU:O	2:B:84:ALA:N	2.45	0.49
2:B:109:ALA:HB2	2:B:145:MET:SD	2.53	0.49
2:B:332:LEU:O	2:B:332:LEU:CG	2.60	0.49
2:B:349:MET:O	2:B:351:GLU:OE2	2.30	0.49
2:B:418:TYR:CG	2:B:424:PHE:CE1	3.00	0.49
2:B:494:ALA:HB2	2:B:529:VAL:HG22	1.94	0.49
2:B:534:ILE:HG12	2:B:595:VAL:HG23	1.93	0.49
3:M:260:LEU:CA	3:M:448:TYR:O	2.59	0.49
3:M:288:ILE:HG22	3:M:289:THR:N	2.27	0.49
3:M:323:MET:CE	3:M:342:LEU:CB	2.89	0.49
3:M:379:LEU:CD2	3:M:397:TRP:NE1	2.66	0.49
1:A:100:LEU:HD11	4:S:157:ASN:O	2.12	0.49
1:A:217:ALA:CA	4:S:142:ILE:CA	2.53	0.49
1:A:456:ASP:O	1:A:459:MET:N	2.37	0.49
1:A:602:GLU:OE1	1:A:633:PHE:HE1	1.95	0.49
1:A:623:MET:O	2:B:617:LEU:CG	2.60	0.49
2:B:343:LEU:HD23	2:B:363:ILE:HD13	1.93	0.49
2:B:421:SER:C	2:B:422:ALA:O	2.42	0.49
3:M:244:VAL:CG2	3:M:472:TYR:CE2	2.94	0.49
3:M:296:LYS:HG3	3:M:296:LYS:O	2.12	0.49
3:M:320:ILE:CG2	3:M:321:GLY:N	2.75	0.49
3:M:371:GLU:HB3	3:M:424:PHE:CD1	2.45	0.49
1:A:179:LYS:HG3	1:A:217:ALA:CB	2.35	0.49
1:A:244:LEU:CG	1:A:277:LYS:HG3	2.39	0.49
1:A:441:TYR:HB3	1:A:444:VAL:HG22	1.91	0.49
1:A:630:PRO:CB	2:B:614:ILE:CG2	2.88	0.49
2:B:125:LYS:O	2:B:128:SER:N	2.45	0.49
2:B:132:SER:CB	2:B:169:VAL:HG21	2.41	0.49
2:B:267:ASP:CA	2:B:289:PRO:HB3	2.39	0.49
2:B:284:ASN:HB3	2:B:285:GLU:HG3	1.91	0.49
2:B:334:MET:CE	2:B:339:PHE:CD1	2.94	0.49
3:M:74:TYR:HB3	3:M:114:ILE:HD11	1.95	0.49
3:M:222:PHE:CD1	3:M:222:PHE:N	2.80	0.49
3:M:317:MET:CB	3:M:322:LEU:N	2.65	0.49
4:S:14:PRO:CA	4:S:36:TYR:CZ	2.95	0.49
4:S:17:VAL:HG13	4:S:17:VAL:O	2.12	0.49

Continued on next page...

Continued from previous page...

Atom-1	Atom-2	Interatomic distance (Å)	Clash overlap (Å)
4:S:51:LEU:HB2	4:S:77:TYR:CE1	2.47	0.49
1:A:153:ILE:CG2	1:A:158:LEU:HD23	2.42	0.49
1:A:403:LEU:HG	1:A:420:ILE:O	2.12	0.49
1:A:440:ASN:OD1	1:A:442:SER:CB	2.58	0.49
1:A:552:ILE:HD13	1:A:600:SER:OG	2.12	0.49
2:B:143:SER:C	2:B:145:MET:H	2.14	0.49
2:B:223:LEU:HD11	2:B:258:GLN:O	2.08	0.49
2:B:267:ASP:OD1	2:B:269:SER:HB2	2.12	0.49
2:B:393:ILE:HG21	2:B:431:MET:HB2	1.95	0.49
3:M:244:VAL:C	3:M:472:TYR:CD2	2.81	0.49
3:M:350:VAL:HG22	3:M:442:GLN:NE2	2.28	0.49
1:A:317:GLU:CG	1:A:351:ARG:HH12	2.26	0.49
1:A:421:PRO:HB2	1:A:424:TYR:HD1	1.78	0.49
2:B:132:SER:O	2:B:136:CYS:SG	2.70	0.49
2:B:221:ASP:O	2:B:223:LEU:HG	2.11	0.49
2:B:257:LYS:HA	2:B:260:LEU:CD2	2.42	0.49
2:B:299:LEU:O	2:B:299:LEU:HD13	2.13	0.49
2:B:315:PRO:CB	2:B:355:ASN:ND2	2.51	0.49
2:B:340:ILE:O	2:B:343:LEU:HB3	2.12	0.49
2:B:424:PHE:CE2	2:B:428:VAL:HG11	2.45	0.49
2:B:427:ASN:HA	2:B:430:ILE:HD12	1.94	0.49
2:B:436:LEU:HB3	2:B:450:VAL:HG13	1.93	0.49
4:S:69:ASN:CG	4:S:71:GLU:O	2.51	0.49
1:A:65:ASN:C	4:S:165:SER:CA	2.81	0.49
1:A:123:LEU:O	1:A:127:LEU:HB2	2.13	0.49
1:A:170:LEU:CG	1:A:206:LYS:HG3	2.42	0.49
1:A:224:GLU:H	4:S:140:MET:HE3	1.78	0.49
1:A:557:LYS:CG	2:B:606:ASP:CA	2.89	0.49
2:B:120:ILE:CG2	2:B:153:ILE:HB	2.41	0.49
2:B:132:SER:O	2:B:169:VAL:CG2	2.60	0.49
2:B:227:HIS:O	2:B:230:PHE:N	2.44	0.49
2:B:292:GLU:CG	2:B:295:ASN:O	2.60	0.49
2:B:362:ALA:O	2:B:366:LEU:CG	2.58	0.49
2:B:455:ILE:O	2:B:458:MET:N	2.46	0.49
2:B:494:ALA:CB	2:B:529:VAL:HG13	2.43	0.49
2:B:513:TRP:HE1	2:B:517:GLU:HG3	1.77	0.49
3:M:103:TYR:CG	3:M:124:ILE:HD11	2.48	0.49
3:M:106:LYS:O	3:M:106:LYS:HG2	2.13	0.49
3:M:218:LEU:HB3	3:M:442:GLN:O	2.11	0.49
3:M:315:VAL:O	3:M:315:VAL:HG12	2.11	0.49
3:M:443:SER:HB2	3:M:447:ILE:N	2.27	0.49

Continued on next page...

Continued from previous page...

Atom-1	Atom-2	Interatomic distance (Å)	Clash overlap (Å)
4:S:9:ASN:CG	4:S:13:GLN:CB	2.79	0.49
1:A:65:ASN:C	4:S:165:SER:HA	2.33	0.49
1:A:67:LYS:CD	1:A:102:GLN:OE1	2.60	0.49
1:A:589:SER:HB2	1:A:601:VAL:HG23	1.92	0.49
1:A:624:LEU:O	2:B:613:MET:CE	2.50	0.49
2:B:106:LEU:HD22	2:B:144:ASP:OD2	2.13	0.49
2:B:123:LEU:HD22	2:B:138:ALA:CB	2.43	0.49
2:B:237:ILE:HG21	2:B:305:SER:CB	2.35	0.49
2:B:275:ARG:CG	2:B:294:VAL:HG21	2.42	0.49
2:B:343:LEU:HD21	2:B:363:ILE:N	2.27	0.49
2:B:346:THR:HG23	2:B:350:THR:CG2	2.30	0.49
2:B:389:ILE:CG2	2:B:427:ASN:CB	2.81	0.49
3:M:225:VAL:C	3:M:480:GLN:O	2.50	0.49
3:M:265:ASN:O	3:M:267:ILE:N	2.45	0.49
1:A:84:MET:SD	1:A:113:SER:HA	2.52	0.49
2:B:150:LEU:O	2:B:154:ILE:HD12	2.08	0.49
2:B:178:ILE:HG23	2:B:217:GLU:CA	2.43	0.49
2:B:200:MET:O	2:B:202:ASP:N	2.45	0.49
2:B:394:TRP:CE3	2:B:397:GLN:OE1	2.64	0.49
2:B:513:TRP:CE3	2:B:551:LEU:HD21	2.40	0.49
2:B:572:GLU:O	2:B:575:ASN:N	2.28	0.49
3:M:101:LEU:HB2	3:M:109:LEU:HD13	1.94	0.49
3:M:276:VAL:HG21	3:M:299:LEU:HD12	1.93	0.49
3:M:331:LEU:HD22	3:M:426:LYS:HZ2	1.77	0.49
4:S:33:GLU:O	4:S:36:TYR:HB2	2.12	0.49
1:A:275:LEU:HA	1:A:278:ILE:CG1	2.43	0.49
1:A:398:GLU:O	1:A:419:ILE:CA	2.61	0.49
1:A:627:GLU:HB3	2:B:617:LEU:HB3	1.90	0.49
1:A:633:PHE:CB	2:B:550:VAL:O	2.58	0.49
2:B:80:GLN:HA	2:B:115:LEU:HD11	1.95	0.49
2:B:151:ALA:HB2	2:B:180:LEU:O	2.13	0.49
2:B:559:ASP:O	2:B:562:ASN:CB	2.61	0.49
3:M:1:MET:HB3	3:M:81:SER:HB3	1.95	0.49
3:M:235:LEU:HD11	3:M:306:LEU:CD1	2.42	0.49
1:A:105:VAL:O	1:A:108:LEU:HB2	2.13	0.48
1:A:252:ILE:HA	4:S:144:THR:CB	2.30	0.48
1:A:319:LEU:O	1:A:321:THR:N	2.46	0.48
1:A:582:ILE:HD11	1:A:608:ARG:N	2.28	0.48
1:A:589:SER:CB	1:A:601:VAL:HG23	2.42	0.48
2:B:38:TYR:HD1	2:B:38:TYR:N	2.10	0.48
2:B:77:ILE:HG23	2:B:82:TYR:CE1	2.42	0.48

Continued on next page...

Continued from previous page...

Atom-1	Atom-2	Interatomic distance (Å)	Clash overlap (Å)
2:B:92:THR:HG22	2:B:93:ASN:N	2.27	0.48
2:B:143:SER:C	2:B:145:MET:N	2.66	0.48
2:B:231:ARG:C	2:B:233:TYR:N	2.65	0.48
2:B:353:GLN:HB2	3:M:50:TYR:H	1.71	0.48
2:B:520:SER:C	2:B:523:PHE:CD2	2.86	0.48
3:M:3:LEU:CD1	3:M:80:THR:O	2.61	0.48
3:M:101:LEU:CG	3:M:106:LYS:CG	2.91	0.48
3:M:224:VAL:CG1	3:M:226:PHE:CZ	2.89	0.48
3:M:226:PHE:CD2	3:M:235:LEU:HA	2.48	0.48
3:M:245:ASP:C	3:M:246:VAL:CG2	2.81	0.48
3:M:389:SER:N	3:M:394:GLN:O	2.33	0.48
1:A:179:LYS:HD3	4:S:152:SER:OG	2.13	0.48
1:A:408:ILE:HG21	4:S:64:ASN:HB3	1.82	0.48
1:A:512:LEU:HD13	1:A:543:TYR:CZ	2.48	0.48
2:B:146:LYS:C	2:B:147:MET:CG	2.80	0.48
2:B:174:ALA:O	2:B:214:ALA:HB2	2.07	0.48
2:B:219:TYR:HD1	2:B:226:LEU:CD1	2.06	0.48
2:B:237:ILE:CG2	2:B:238:LYS:N	2.76	0.48
3:M:93:LEU:O	3:M:96:ILE:HB	2.14	0.48
3:M:215:TYR:CD1	3:M:468:LYS:CB	2.94	0.48
3:M:222:PHE:CG	3:M:240:ILE:HG12	2.48	0.48
3:M:235:LEU:HD13	3:M:310:VAL:HG21	1.95	0.48
3:M:386:PHE:CB	3:M:397:TRP:HD1	2.26	0.48
3:M:435:LEU:O	3:M:437:TYR:CE1	2.66	0.48
4:S:53:THR:CG2	4:S:68:VAL:C	2.80	0.48
1:A:102:GLN:O	4:S:167:ILE:HD11	2.13	0.48
2:B:5:ILE:HA	2:B:8:ILE:CD1	2.38	0.48
2:B:56:SER:O	2:B:97:VAL:CG2	2.61	0.48
2:B:293:VAL:O	2:B:299:LEU:HB3	2.04	0.48
2:B:418:TYR:HD1	2:B:424:PHE:CE1	2.26	0.48
2:B:447:GLU:O	2:B:450:VAL:HB	2.13	0.48
2:B:580:TYR:O	2:B:582:ASP:N	2.43	0.48
3:M:7:ILE:HA	3:M:76:CYS:CA	2.28	0.48
3:M:226:PHE:CD1	3:M:481:VAL:HG22	2.49	0.48
3:M:235:LEU:HD11	3:M:306:LEU:HD13	1.94	0.48
4:S:80:TYR:CG	4:S:106:VAL:CG1	2.96	0.48
1:A:316:LEU:HD12	1:A:348:PHE:CZ	2.45	0.48
2:B:98:LYS:HD2	2:B:102:HIS:CE1	2.48	0.48
2:B:230:PHE:CE2	2:B:298:ASP:C	2.77	0.48
2:B:302:PHE:CE1	2:B:306:LEU:HD21	2.48	0.48
2:B:389:ILE:HG21	2:B:427:ASN:CB	2.35	0.48

Continued on next page...

Continued from previous page...

Atom-1	Atom-2	Interatomic distance (Å)	Clash overlap (Å)
2:B:513:TRP:NE1	2:B:517:GLU:CG	2.76	0.48
2:B:538:SER:CA	2:B:598:LEU:HD22	2.43	0.48
2:B:542:PRO:O	2:B:545:ARG:HB2	2.14	0.48
2:B:589:SER:OG	2:B:618:PHE:HE2	1.90	0.48
3:M:224:VAL:HG22	3:M:306:LEU:CD1	2.41	0.48
3:M:228:LYS:HZ2	3:M:327:PHE:H	1.58	0.48
3:M:249:TYR:CE1	3:M:467:TYR:CE1	3.02	0.48
3:M:323:MET:SD	3:M:342:LEU:CA	3.01	0.48
3:M:338:PHE:CE2	3:M:415:ILE:HD11	2.48	0.48
3:M:347:PHE:HA	3:M:350:VAL:HG23	1.96	0.48
1:A:207:LEU:CD1	1:A:240:LEU:HD23	2.38	0.48
1:A:304:LEU:C	1:A:304:LEU:HD23	2.34	0.48
1:A:384:LEU:C	1:A:384:LEU:HD12	2.34	0.48
1:A:478:ARG:O	1:A:482:ILE:HG13	2.13	0.48
2:B:80:GLN:HG2	2:B:115:LEU:CD2	2.43	0.48
2:B:139:LEU:CD2	2:B:173:VAL:CB	2.87	0.48
2:B:277:CYS:HA	2:B:295:ASN:O	2.13	0.48
2:B:360:LEU:HB3	2:B:394:TRP:HB2	1.95	0.48
2:B:393:ILE:CG1	2:B:428:VAL:HA	2.40	0.48
2:B:541:GLY:O	2:B:544:THR:HB	2.14	0.48
3:M:65:TYR:CG	3:M:86:PRO:CA	2.93	0.48
3:M:240:ILE:HG22	3:M:444:ALA:CB	2.42	0.48
3:M:319:SER:CB	3:M:346:ASN:C	2.81	0.48
3:M:380:ARG:HH11	3:M:412:ARG:HH11	1.59	0.48
1:A:67:LYS:HB3	4:S:166:LYS:HG2	1.86	0.48
1:A:78:GLU:O	1:A:80:TYR:C	2.52	0.48
1:A:121:LEU:HD13	1:A:155:THR:CG2	2.44	0.48
1:A:153:ILE:HG22	1:A:158:LEU:HD23	1.94	0.48
1:A:350:SER:O	1:A:352:PHE:N	2.46	0.48
1:A:384:LEU:HD13	1:A:435:ILE:HG21	1.86	0.48
1:A:509:PRO:CB	1:A:547:VAL:HG21	2.38	0.48
1:A:594:PHE:CG	2:B:434:LYS:HE2	2.48	0.48
2:B:27:THR:HG22	2:B:27:THR:O	2.14	0.48
2:B:56:SER:HB2	2:B:92:THR:CG2	2.30	0.48
2:B:126:SER:O	2:B:127:LEU:C	2.49	0.48
2:B:429:VAL:O	2:B:433:VAL:HG23	2.12	0.48
3:M:241:HIS:CD2	3:M:475:GLN:H	2.29	0.48
4:S:57:LEU:CA	4:S:60:SER:HB2	2.41	0.48
1:A:132:LEU:CB	1:A:165:ASP:HB3	2.44	0.48
1:A:196:LEU:O	1:A:197:ARG:O	2.31	0.48
1:A:320:HIS:ND1	1:A:352:PHE:HD2	2.12	0.48

Continued on next page...

Continued from previous page...

Atom-1	Atom-2	Interatomic distance (Å)	Clash overlap (Å)
1:A:402:ILE:O	1:A:402:ILE:HG23	2.12	0.48
1:A:516:ILE:HD13	1:A:551:LEU:CD1	2.43	0.48
1:A:556:VAL:HG22	1:A:603:VAL:HG11	1.94	0.48
1:A:556:VAL:HG21	1:A:603:VAL:HG13	1.96	0.48
2:B:83:PHE:CE2	2:B:119:SER:HA	2.38	0.48
2:B:279:LEU:CB	2:B:288:TYR:HD1	2.23	0.48
2:B:377:TYR:O	2:B:380:LYS:CB	2.49	0.48
2:B:394:TRP:CZ3	2:B:397:GLN:NE2	2.82	0.48
2:B:396:ILE:HD13	2:B:418:TYR:CE2	2.43	0.48
2:B:515:PHE:CD2	2:B:529:VAL:HG21	2.44	0.48
2:B:542:PRO:CA	2:B:602:ASP:OD1	2.61	0.48
1:A:154:ILE:O	1:A:155:THR:C	2.49	0.48
1:A:316:LEU:O	1:A:319:LEU:HB2	2.13	0.48
1:A:416:ILE:O	1:A:417:PRO:C	2.40	0.48
2:B:106:LEU:CG	2:B:144:ASP:CB	2.86	0.48
2:B:227:HIS:CE1	2:B:292:GLU:CG	2.96	0.48
2:B:237:ILE:HG12	2:B:245:GLN:HG2	1.95	0.48
2:B:433:VAL:C	2:B:474:VAL:HG21	2.33	0.48
2:B:440:GLY:C	2:B:442:LEU:N	2.61	0.48
2:B:515:PHE:CE2	2:B:529:VAL:CG1	2.95	0.48
2:B:519:ALA:CB	2:B:555:LEU:HD13	2.44	0.48
2:B:538:SER:HB3	2:B:598:LEU:CD2	2.44	0.48
2:B:589:SER:C	2:B:591:MET:N	2.65	0.48
3:M:6:TYR:CB	3:M:16:PHE:O	2.62	0.48
3:M:217:ASP:C	3:M:472:TYR:CZ	2.87	0.48
3:M:221:THR:HA	3:M:437:TYR:O	2.13	0.48
3:M:234:ARG:O	3:M:235:LEU:C	2.51	0.48
3:M:243:ILE:CG2	3:M:298:ARG:HG2	2.43	0.48
3:M:319:SER:HG	3:M:346:ASN:CB	2.05	0.48
4:S:5:VAL:O	4:S:18:LYS:N	2.44	0.48
4:S:17:VAL:HG13	4:S:19:PHE:HE1	1.74	0.48
1:A:144:GLY:HA3	1:A:180:LYS:HG3	1.96	0.48
1:A:215:VAL:O	1:A:216:SER:O	2.31	0.48
1:A:381:GLU:CA	1:A:384:LEU:HD23	2.39	0.48
1:A:454:ILE:CG2	1:A:473:ILE:CG2	2.92	0.48
1:A:488:ARG:CD	1:A:522:PHE:CE2	2.96	0.48
2:B:80:GLN:CA	2:B:115:LEU:HD11	2.43	0.48
2:B:417:TYR:O	2:B:421:SER:CB	2.62	0.48
3:M:7:ILE:HG21	3:M:74:TYR:HB2	1.96	0.48
3:M:215:TYR:CD1	3:M:468:LYS:CG	2.97	0.48
3:M:234:ARG:O	3:M:236:LEU:N	2.47	0.48

Continued on next page...

Continued from previous page...

Atom-1	Atom-2	Interatomic distance (Å)	Clash overlap (Å)
1:A:125:THR:CG2	1:A:158:LEU:HA	2.43	0.48
1:A:306:GLU:O	1:A:308:ASP:N	2.47	0.48
1:A:446:ASP:C	1:A:448:GLU:O	2.52	0.48
2:B:44:PRO:HA	2:B:47:LEU:HD12	1.96	0.48
2:B:155:LEU:O	2:B:158:VAL:N	2.47	0.48
2:B:340:ILE:CB	2:B:373:LEU:HG	2.44	0.48
2:B:380:LYS:NZ	3:M:236:LEU:HD11	2.28	0.48
2:B:522:GLU:C	2:B:524:LYS:N	2.67	0.48
3:M:10:THR:HA	3:M:75:TRP:CE2	2.47	0.48
3:M:10:THR:N	3:M:75:TRP:NE1	2.62	0.48
3:M:260:LEU:O	3:M:286:SER:HB3	2.14	0.48
3:M:300:LEU:HD11	3:M:447:ILE:CD1	2.41	0.48
1:A:100:LEU:CB	1:A:142:LYS:HG3	2.44	0.47
1:A:408:ILE:CD1	1:A:410:TYR:HD1	2.27	0.47
1:A:610:SER:OG	1:A:625:LEU:CD1	2.52	0.47
2:B:136:CYS:HA	2:B:172:GLU:CB	2.41	0.47
2:B:540:GLU:OE1	2:B:548:ILE:CD1	2.62	0.47
2:B:542:PRO:HB3	2:B:602:ASP:OD1	2.14	0.47
3:M:9:ASP:C	3:M:75:TRP:NE1	2.64	0.47
3:M:16:PHE:HZ	3:M:18:TYR:CD1	2.32	0.47
3:M:244:VAL:CA	3:M:472:TYR:CG	2.76	0.47
3:M:324:SER:O	3:M:340:LEU:HA	2.14	0.47
4:S:47:GLN:NE2	4:S:78:LYS:C	2.63	0.47
1:A:79:MET:HG3	1:A:112:GLN:OE1	2.14	0.47
1:A:216:SER:HB3	4:S:143:GLU:CA	2.13	0.47
1:A:384:LEU:HD12	1:A:385:LYS:N	2.28	0.47
1:A:429:VAL:CG1	1:A:473:ILE:HD11	2.40	0.47
1:A:563:CYS:SG	1:A:621:LEU:HG	2.54	0.47
1:A:625:LEU:C	1:A:627:GLU:N	2.68	0.47
1:A:633:PHE:CA	2:B:554:LYS:HB2	2.15	0.47
2:B:117:LEU:HD21	2:B:149:SER:HB2	1.92	0.47
2:B:207:VAL:O	2:B:210:CYS:HB2	2.14	0.47
2:B:278:PRO:CA	2:B:288:TYR:HB2	2.29	0.47
2:B:304:GLN:O	2:B:307:ASN:HB2	2.14	0.47
2:B:414:GLU:O	2:B:417:TYR:HB3	2.14	0.47
2:B:425:PRO:HD2	2:B:428:VAL:CG2	2.39	0.47
3:M:124:ILE:O	3:M:127:CYS:N	2.47	0.47
3:M:245:ASP:N	3:M:472:TYR:CE2	2.82	0.47
3:M:364:VAL:HG12	3:M:372:ILE:HG13	1.95	0.47
4:S:35:VAL:HG12	4:S:68:VAL:CG1	2.44	0.47
4:S:50:PHE:HB2	4:S:75:ILE:O	2.14	0.47

Continued on next page...

Continued from previous page...

Atom-1	Atom-2	Interatomic distance (Å)	Clash overlap (Å)
4:S:50:PHE:HD1	4:S:52:VAL:HG23	1.79	0.47
1:A:180:LYS:NZ	4:S:156:LEU:HD11	2.29	0.47
1:A:213:SER:C	4:S:143:GLU:CD	2.72	0.47
1:A:220:SER:CA	4:S:142:ILE:HG22	2.37	0.47
1:A:291:ILE:H	1:A:291:ILE:CD1	2.28	0.47
1:A:605:GLU:CD	1:A:632:PHE:CE2	2.88	0.47
2:B:42:ILE:HD13	2:B:65:ARG:CD	2.39	0.47
2:B:260:LEU:HA	2:B:291:TYR:CZ	2.49	0.47
2:B:588:ILE:HG21	2:B:618:PHE:HE1	1.79	0.47
3:M:8:THR:CA	3:M:13:LYS:O	2.62	0.47
3:M:223:HIS:N	3:M:239:SER:O	2.46	0.47
3:M:240:ILE:HG21	3:M:444:ALA:O	2.14	0.47
3:M:252:ASP:O	3:M:254:PRO:HD2	2.14	0.47
1:A:182:ILE:HG23	1:A:221:VAL:HG21	1.83	0.47
1:A:588:LEU:O	1:A:590:TYR:N	2.47	0.47
2:B:155:LEU:C	2:B:157:THR:H	2.17	0.47
2:B:277:CYS:SG	2:B:292:GLU:CD	2.91	0.47
2:B:279:LEU:HD12	2:B:288:TYR:HE1	1.79	0.47
2:B:494:ALA:CB	2:B:529:VAL:HG22	2.45	0.47
3:M:8:THR:O	3:M:75:TRP:HB2	2.14	0.47
3:M:226:PHE:CE2	3:M:235:LEU:HB2	2.50	0.47
3:M:235:LEU:CD1	3:M:306:LEU:HD13	2.44	0.47
3:M:284:SER:O	3:M:286:SER:N	2.48	0.47
4:S:4:ALA:CB	4:S:19:PHE:CD2	2.98	0.47
4:S:69:ASN:O	4:S:70:ASN:HB3	2.14	0.47
4:S:101:LEU:HD11	4:S:136:VAL:HG22	1.96	0.47
1:A:270:LEU:CD1	1:A:274:LEU:CD2	2.92	0.47
1:A:633:PHE:CG	2:B:550:VAL:O	2.68	0.47
2:B:127:LEU:CB	2:B:161:LEU:CD1	2.63	0.47
2:B:352:ASN:ND2	2:B:355:ASN:HB2	2.30	0.47
2:B:534:ILE:O	2:B:598:LEU:CD1	2.56	0.47
2:B:566:ALA:HB1	2:B:581:TYR:HA	1.95	0.47
3:M:6:TYR:HD2	3:M:16:PHE:O	1.94	0.47
3:M:218:LEU:HD12	3:M:218:LEU:H	1.80	0.47
4:S:101:LEU:HG	4:S:135:ILE:HG21	1.96	0.47
4:S:127:THR:HG21	4:S:157:ASN:OD1	2.15	0.47
1:A:489:GLU:HB2	1:A:525:LEU:HD21	1.96	0.47
1:A:586:GLU:O	1:A:588:LEU:N	2.44	0.47
2:B:25:VAL:HG22	2:B:36:THR:N	2.30	0.47
2:B:42:ILE:CD1	2:B:65:ARG:CD	2.81	0.47
2:B:42:ILE:HG23	2:B:46:GLN:OE1	2.14	0.47

Continued on next page...

Continued from previous page...

Atom-1	Atom-2	Interatomic distance (Å)	Clash overlap (Å)
2:B:141:ALA:C	2:B:143:SER:H	2.18	0.47
2:B:232:ARG:O	2:B:236:ILE:N	2.43	0.47
2:B:243:TRP:HZ3	3:M:91:THR:CA	2.12	0.47
2:B:274:PRO:CB	2:B:295:ASN:HD21	2.28	0.47
2:B:531:ARG:HG3	2:B:591:MET:CE	2.45	0.47
3:M:62:VAL:O	3:M:64:LYS:HG3	2.15	0.47
3:M:273:HIS:HB3	3:M:276:VAL:HG23	1.97	0.47
1:A:141:VAL:CG2	4:S:156:LEU:C	2.75	0.47
1:A:156:PRO:O	1:A:160:ARG:CG	2.62	0.47
1:A:178:ARG:O	1:A:182:ILE:HG13	2.15	0.47
1:A:220:SER:CB	4:S:142:ILE:HG23	2.27	0.47
1:A:316:LEU:HD21	1:A:341:ILE:HG13	1.95	0.47
1:A:388:VAL:HG22	1:A:435:ILE:HD12	1.96	0.47
1:A:463:ASP:C	2:B:1:MET:CG	2.73	0.47
1:A:563:CYS:O	1:A:566:PHE:CE2	2.68	0.47
1:A:609:LEU:HD12	1:A:625:LEU:HD12	1.96	0.47
2:B:25:VAL:HG22	2:B:35:TYR:C	2.35	0.47
2:B:37:TYR:HE2	2:B:46:GLN:OE1	1.97	0.47
2:B:51:LEU:HD11	2:B:66:ILE:HD13	1.95	0.47
2:B:85:ASP:C	2:B:89:ASN:HD22	2.18	0.47
2:B:117:LEU:O	2:B:120:ILE:HB	2.14	0.47
2:B:140:SER:CB	2:B:172:GLU:OE2	2.55	0.47
2:B:216:LYS:CG	2:B:251:LEU:HA	2.45	0.47
2:B:336:ASN:C	2:B:338:LYS:H	2.17	0.47
2:B:421:SER:O	2:B:422:ALA:C	2.49	0.47
2:B:474:VAL:C	2:B:476:ARG:N	2.66	0.47
2:B:523:PHE:HD1	2:B:559:ASP:OD2	1.97	0.47
2:B:534:ILE:HG12	2:B:595:VAL:CG2	2.44	0.47
2:B:585:GLY:O	2:B:589:SER:CB	2.63	0.47
3:M:215:TYR:CD1	3:M:467:TYR:C	2.88	0.47
3:M:222:PHE:CD2	3:M:439:TYR:CZ	3.02	0.47
3:M:243:ILE:H	3:M:474:THR:HG21	1.64	0.47
3:M:244:VAL:O	3:M:299:LEU:CB	2.62	0.47
3:M:276:VAL:CG1	3:M:299:LEU:HD12	2.45	0.47
3:M:348:LYS:HA	3:M:406:GLY:N	2.30	0.47
3:M:353:VAL:HG11	3:M:356:LEU:HB2	1.97	0.47
3:M:362:PHE:CE1	3:M:374:TYR:CZ	3.03	0.47
4:S:1:MET:H2	4:S:93:GLU:CD	2.18	0.47
4:S:85:PHE:CE2	4:S:109:LEU:CD2	2.85	0.47
4:S:112:CYS:SG	4:S:153:VAL:CG2	2.99	0.47
1:A:67:LYS:O	4:S:166:LYS:HA	2.13	0.47

Continued on next page...

Continued from previous page...

Atom-1	Atom-2	Interatomic distance (Å)	Clash overlap (Å)
1:A:77:LEU:O	1:A:80:TYR:HB2	2.15	0.47
1:A:114:PHE:CE2	1:A:153:ILE:HA	2.50	0.47
1:A:220:SER:HB2	4:S:142:ILE:CA	2.43	0.47
2:B:94:ASP:OD1	2:B:95:THR:N	2.47	0.47
2:B:213:LEU:HD21	2:B:247:TYR:CG	2.50	0.47
2:B:389:ILE:HG21	2:B:427:ASN:HD22	1.80	0.47
2:B:515:PHE:HA	2:B:518:ILE:HG22	1.97	0.47
3:M:72:LEU:HD11	3:M:101:LEU:HD22	1.97	0.47
3:M:101:LEU:HD11	3:M:107:ASP:N	2.30	0.47
1:A:114:PHE:CE1	1:A:153:ILE:HG23	2.50	0.47
1:A:147:LEU:CD1	1:A:166:LEU:HD23	2.44	0.47
1:A:189:PHE:HD2	1:A:225:LEU:CG	2.27	0.47
1:A:530:ASN:OD1	1:A:577:VAL:CG2	2.63	0.47
2:B:139:LEU:O	2:B:176:ALA:HB2	2.14	0.47
2:B:143:SER:O	2:B:144:ASP:C	2.46	0.47
2:B:143:SER:CA	2:B:179:LYS:CB	2.90	0.47
2:B:175:LEU:CD1	2:B:210:CYS:CB	2.89	0.47
2:B:219:TYR:CB	2:B:223:LEU:HD21	2.20	0.47
2:B:245:GLN:CB	2:B:309:LEU:CD1	2.93	0.47
2:B:267:ASP:CB	2:B:289:PRO:HB3	2.45	0.47
2:B:399:LEU:O	2:B:401:THR:N	2.48	0.47
3:M:224:VAL:CB	3:M:479:PHE:CD1	2.98	0.47
1:A:95:MET:C	1:A:127:LEU:HD23	2.33	0.47
1:A:408:ILE:HD12	1:A:410:TYR:HD1	1.77	0.47
1:A:476:GLN:OE1	1:A:476:GLN:HA	2.15	0.47
1:A:594:PHE:CB	2:B:473:ASN:HB3	2.22	0.47
2:B:38:TYR:HB3	2:B:42:ILE:HD12	1.45	0.47
2:B:174:ALA:HB1	2:B:211:ALA:HB1	1.94	0.47
2:B:215:TYR:CE2	2:B:229:HIS:CG	3.02	0.47
2:B:299:LEU:HD21	2:B:328:LEU:HD13	1.98	0.47
2:B:553:ALA:O	2:B:556:LEU:HB2	2.15	0.47
2:B:559:ASP:CG	2:B:582:ASP:OD2	2.53	0.47
3:M:5:PHE:HB2	3:M:125:PHE:HE2	1.77	0.47
3:M:215:TYR:CE1	3:M:468:LYS:CA	2.90	0.47
3:M:270:PRO:CA	3:M:302:TYR:HD1	2.28	0.47
3:M:336:ASP:CG	3:M:415:ILE:O	2.44	0.47
3:M:421:GLY:O	3:M:423:ASN:N	2.48	0.47
3:M:473:LYS:O	3:M:474:THR:HG22	2.15	0.47
1:A:100:LEU:CD1	4:S:161:GLU:H	2.28	0.46
1:A:141:VAL:HB	4:S:159:ALA:CB	2.38	0.46
1:A:219:VAL:HG21	1:A:256:LEU:HD23	1.86	0.46

Continued on next page...

Continued from previous page...

Atom-1	Atom-2	Interatomic distance (Å)	Clash overlap (Å)
1:A:241:TYR:C	1:A:241:TYR:CD2	2.88	0.46
1:A:335:CYS:O	1:A:338:PHE:HB2	2.15	0.46
1:A:491:THR:O	1:A:495:ILE:HG12	2.15	0.46
1:A:557:LYS:CG	2:B:606:ASP:CB	2.93	0.46
1:A:636:TYR:HD2	2:B:513:TRP:HZ2	1.63	0.46
2:B:4:SER:O	2:B:8:ILE:CG1	2.63	0.46
2:B:10:SER:O	2:B:11:ALA:C	2.52	0.46
2:B:196:LEU:HD13	2:B:215:TYR:CE2	2.50	0.46
2:B:212:VAL:N	2:B:233:TYR:CZ	2.83	0.46
2:B:336:ASN:C	2:B:338:LYS:N	2.67	0.46
2:B:570:GLY:O	2:B:571:SER:C	2.51	0.46
3:M:9:ASP:C	3:M:9:ASP:OD1	2.54	0.46
3:M:217:ASP:CB	3:M:471:LYS:N	2.54	0.46
3:M:223:HIS:CA	3:M:479:PHE:CG	2.86	0.46
3:M:375:LYS:CG	3:M:418:GLU:OE1	2.63	0.46
4:S:35:VAL:HG12	4:S:68:VAL:HG11	1.96	0.46
4:S:53:THR:HG22	4:S:57:LEU:CB	2.26	0.46
1:A:170:LEU:HB3	1:A:202:LYS:HG3	1.98	0.46
1:A:226:SER:HB3	1:A:263:LEU:HD21	1.97	0.46
1:A:316:LEU:CD1	1:A:341:ILE:HG21	2.42	0.46
1:A:403:LEU:CD2	1:A:422:GLU:OE2	2.59	0.46
1:A:636:TYR:H	2:B:554:LYS:CE	2.27	0.46
2:B:97:VAL:HG12	2:B:101:ILE:CD1	2.45	0.46
2:B:115:LEU:HD13	2:B:115:LEU:HA	1.83	0.46
2:B:216:LYS:HB2	2:B:251:LEU:HB2	1.96	0.46
2:B:261:PRO:HD2	2:B:293:VAL:HG23	1.97	0.46
2:B:592:TYR:CE1	2:B:615:SER:CB	2.98	0.46
3:M:6:TYR:OH	3:M:17:GLN:CD	2.41	0.46
3:M:290:PHE:CE2	3:M:297:PHE:CE1	3.00	0.46
3:M:325:LEU:HG	3:M:338:PHE:HD1	1.80	0.46
3:M:339:GLU:CG	3:M:412:ARG:HD3	2.39	0.46
4:S:17:VAL:HB	4:S:32:LEU:HD11	1.98	0.46
1:A:105:VAL:N	4:S:167:ILE:HD12	1.98	0.46
1:A:435:ILE:CG2	1:A:441:TYR:CE2	2.98	0.46
1:A:515:CYS:O	1:A:519:LEU:HG	2.15	0.46
2:B:96:LYS:O	2:B:99:ARG:HB3	2.16	0.46
2:B:403:ILE:CG2	2:B:411:ILE:CD1	2.92	0.46
2:B:522:GLU:C	2:B:524:LYS:H	2.18	0.46
2:B:546:CYS:N	2:B:607:ILE:HG21	2.31	0.46
2:B:592:TYR:O	2:B:595:VAL:HB	2.16	0.46
2:B:604:GLU:HB3	2:B:607:ILE:HD12	1.97	0.46

Continued on next page...

Continued from previous page...

Atom-1	Atom-2	Interatomic distance (Å)	Clash overlap (Å)
3:M:253:ASN:O	3:M:254:PRO:C	2.53	0.46
3:M:360:LEU:HD13	3:M:433:VAL:HG23	1.95	0.46
1:A:251:TRP:NE1	4:S:104:THR:HA	2.29	0.46
1:A:287:ALA:HB3	1:A:288:THR:CA	2.45	0.46
1:A:287:ALA:CB	1:A:289:SER:HB3	2.44	0.46
1:A:288:THR:HG1	1:A:291:ILE:CB	2.16	0.46
1:A:469:LEU:O	1:A:470:GLY:C	2.43	0.46
2:B:7:ARG:O	2:B:8:ILE:C	2.53	0.46
2:B:102:HIS:HA	2:B:141:ALA:HB2	1.97	0.46
2:B:307:ASN:O	2:B:309:LEU:N	2.49	0.46
2:B:437:SER:HA	2:B:478:LEU:CG	2.45	0.46
2:B:537:PHE:CG	2:B:598:LEU:HD13	2.36	0.46
2:B:559:ASP:O	2:B:562:ASN:HB2	2.15	0.46
3:M:17:GLN:N	3:M:118:TYR:CE1	2.74	0.46
3:M:220:GLU:HB2	3:M:222:PHE:CE1	2.46	0.46
3:M:220:GLU:OE2	3:M:439:TYR:CG	2.69	0.46
3:M:449:VAL:HG12	3:M:452:ILE:HD12	1.91	0.46
1:A:96:SER:H	1:A:127:LEU:CD2	1.79	0.46
1:A:129:LYS:HG2	1:A:165:ASP:OD2	2.16	0.46
1:A:537:THR:CB	1:A:584:PHE:CZ	2.97	0.46
1:A:607:LEU:C	1:A:609:LEU:H	2.18	0.46
2:B:59:VAL:O	2:B:63:MET:HB2	2.15	0.46
2:B:132:SER:O	2:B:169:VAL:HG22	2.16	0.46
2:B:143:SER:HB2	2:B:179:LYS:CD	2.45	0.46
2:B:169:VAL:HG12	2:B:173:VAL:HG23	1.94	0.46
2:B:223:LEU:HB3	2:B:259:TYR:CB	2.46	0.46
2:B:355:ASN:O	2:B:358:MET:N	2.49	0.46
2:B:513:TRP:H	2:B:551:LEU:HD11	1.61	0.46
2:B:546:CYS:N	2:B:607:ILE:HD13	2.30	0.46
3:M:217:ASP:CB	3:M:470:ALA:CA	2.89	0.46
3:M:224:VAL:HG11	3:M:235:LEU:HD12	1.98	0.46
3:M:240:ILE:CG2	3:M:444:ALA:HA	2.45	0.46
3:M:338:PHE:CD2	3:M:415:ILE:HD11	2.50	0.46
1:A:103:LYS:O	1:A:107:TYR:CG	2.61	0.46
1:A:323:CYS:HB2	1:A:355:LEU:HD21	1.96	0.46
1:A:374:LEU:C	1:A:376:GLU:OE1	2.54	0.46
1:A:409:VAL:O	1:A:409:VAL:CG1	2.63	0.46
1:A:604:LEU:HD23	1:A:604:LEU:C	2.35	0.46
2:B:20:ARG:CD	2:B:35:TYR:OH	2.31	0.46
2:B:59:VAL:O	2:B:59:VAL:HG12	2.16	0.46
2:B:136:CYS:HB3	2:B:172:GLU:HG3	1.97	0.46

Continued on next page...

Continued from previous page...

Atom-1	Atom-2	Interatomic distance (Å)	Clash overlap (Å)
2:B:169:VAL:C	2:B:173:VAL:HG23	2.35	0.46
2:B:199:LEU:O	2:B:201:ALA:CA	2.59	0.46
2:B:219:TYR:HE2	2:B:226:LEU:H	1.48	0.46
2:B:419:VAL:O	2:B:420:ALA:C	2.53	0.46
2:B:461:HIS:CB	2:B:463:LEU:CD2	2.94	0.46
2:B:477:MET:O	2:B:480:GLN:CB	2.58	0.46
2:B:497:LEU:HD13	2:B:503:LEU:HD12	1.97	0.46
2:B:564:LYS:O	2:B:574:ASN:ND2	2.47	0.46
2:B:569:THR:HA	2:B:571:SER:H	1.80	0.46
3:M:130:GLU:OE1	3:M:130:GLU:HA	2.16	0.46
3:M:217:ASP:C	3:M:472:TYR:CE1	2.89	0.46
1:A:95:MET:CB	1:A:127:LEU:HD23	2.46	0.46
1:A:128:LEU:CD1	1:A:150:LEU:HG	2.29	0.46
1:A:219:VAL:CA	1:A:259:LEU:CD1	2.93	0.46
1:A:275:LEU:C	1:A:275:LEU:HD23	2.35	0.46
1:A:287:ALA:N	1:A:288:THR:HA	2.31	0.46
1:A:503:ASN:OD1	3:M:59:ASP:CG	2.52	0.46
2:B:143:SER:CB	2:B:175:LEU:HB3	2.45	0.46
2:B:177:ILE:HB	2:B:196:LEU:CD2	2.26	0.46
2:B:490:ILE:CD1	2:B:518:ILE:HG21	2.45	0.46
2:B:572:GLU:C	2:B:574:ASN:N	2.69	0.46
3:M:235:LEU:HD11	3:M:306:LEU:HB2	1.94	0.46
3:M:290:PHE:HD1	3:M:299:LEU:CD1	2.28	0.46
1:A:166:LEU:HD12	1:A:185:LEU:HD21	1.95	0.46
1:A:166:LEU:HB2	1:A:185:LEU:HD21	1.98	0.46
1:A:320:HIS:N	1:A:338:PHE:HZ	2.14	0.46
1:A:384:LEU:HD21	1:A:441:TYR:CD2	2.49	0.46
2:B:56:SER:O	2:B:97:VAL:HG21	2.15	0.46
2:B:106:LEU:CG	2:B:144:ASP:HB2	2.43	0.46
2:B:120:ILE:HG13	2:B:150:LEU:CD2	2.44	0.46
2:B:208:ILE:C	2:B:210:CYS:N	2.66	0.46
2:B:215:TYR:HB3	2:B:226:LEU:HD13	1.87	0.46
2:B:257:LYS:HA	2:B:260:LEU:HG	1.97	0.46
2:B:389:ILE:HG22	2:B:393:ILE:HD11	1.98	0.46
2:B:487:LEU:CD2	2:B:522:GLU:HB3	2.29	0.46
2:B:556:LEU:HD22	2:B:588:ILE:CG1	2.07	0.46
3:M:2:TYR:O	3:M:80:THR:C	2.54	0.46
3:M:246:VAL:CA	3:M:470:ALA:HB1	2.45	0.46
3:M:258:VAL:CG1	3:M:452:ILE:HG13	2.45	0.46
3:M:435:LEU:HD12	3:M:435:LEU:N	2.31	0.46
4:S:135:ILE:O	4:S:141:VAL:CB	2.62	0.46

Continued on next page...

Continued from previous page...

Atom-1	Atom-2	Interatomic distance (Å)	Clash overlap (Å)
1:A:175:PRO:CB	1:A:211:ASP:CG	2.82	0.46
1:A:252:ILE:HG13	4:S:145:ASN:HA	1.67	0.46
1:A:562:TRP:CE3	1:A:574:ILE:HD12	2.51	0.46
1:A:633:PHE:CB	2:B:554:LYS:HB2	2.45	0.46
2:B:36:THR:O	2:B:40:GLN:CG	2.64	0.46
2:B:77:ILE:HG23	2:B:82:TYR:HE1	1.71	0.46
2:B:267:ASP:CB	2:B:289:PRO:HG3	2.46	0.46
3:M:339:GLU:CD	3:M:412:ARG:HD3	2.35	0.46
3:M:356:LEU:HD23	3:M:358:ILE:HG13	1.93	0.46
4:S:53:THR:HG1	4:S:68:VAL:C	1.98	0.46
4:S:127:THR:CG2	4:S:157:ASN:OD1	2.64	0.46
4:S:160:ALA:HB1	4:S:167:ILE:HG21	1.96	0.46
1:A:67:LYS:CG	1:A:94:VAL:HG22	2.45	0.46
1:A:132:LEU:HD12	1:A:165:ASP:CB	2.45	0.46
2:B:14:THR:O	2:B:16:LYS:N	2.48	0.46
2:B:87:VAL:HG21	2:B:119:SER:HA	1.98	0.46
2:B:157:THR:C	2:B:159:LYS:H	2.18	0.46
2:B:175:LEU:HD21	2:B:210:CYS:O	2.02	0.46
2:B:215:TYR:O	2:B:219:TYR:CD1	2.67	0.46
2:B:252:LEU:CG	2:B:302:PHE:CE1	2.94	0.46
2:B:433:VAL:HG12	2:B:474:VAL:CG1	2.43	0.46
2:B:493:LEU:HD21	2:B:511:ILE:CG1	2.46	0.46
3:M:18:TYR:CE2	3:M:20:LEU:HD23	2.43	0.46
4:S:34:GLN:CG	4:S:58:LEU:HD11	2.45	0.46
4:S:51:LEU:H	4:S:77:TYR:HE1	1.64	0.46
1:A:63:ASP:C	4:S:165:SER:HG	2.11	0.45
1:A:121:LEU:HD13	1:A:155:THR:HG21	1.97	0.45
1:A:128:LEU:CB	1:A:150:LEU:HD21	2.46	0.45
1:A:132:LEU:CD2	1:A:169:MET:SD	3.04	0.45
1:A:200:PHE:CE1	1:A:236:LEU:HG	2.51	0.45
1:A:353:ASP:OD1	1:A:378:ILE:HD12	2.16	0.45
1:A:401:VAL:HG23	1:A:419:ILE:HB	1.98	0.45
1:A:401:VAL:HG12	1:A:402:ILE:N	2.30	0.45
1:A:404:GLN:OE1	2:B:7:ARG:NH1	2.50	0.45
2:B:143:SER:CA	2:B:179:LYS:CD	2.80	0.45
2:B:178:ILE:HG23	2:B:218:CYS:N	2.30	0.45
2:B:523:PHE:CD1	2:B:559:ASP:OD2	2.69	0.45
3:M:67:SER:HB3	3:M:90:PHE:HD1	1.70	0.45
3:M:216:VAL:O	3:M:216:VAL:CG2	2.64	0.45
3:M:228:LYS:NZ	3:M:327:PHE:N	2.63	0.45
3:M:319:SER:HB2	3:M:346:ASN:N	2.26	0.45

Continued on next page...

Continued from previous page...

Atom-1	Atom-2	Interatomic distance (Å)	Clash overlap (Å)
3:M:469:GLY:O	3:M:470:ALA:C	2.53	0.45
4:S:50:PHE:CA	4:S:76:ILE:HA	2.46	0.45
1:A:74:LEU:HD22	1:A:87:CYS:HB3	1.96	0.45
1:A:95:MET:SD	1:A:107:TYR:CE2	3.09	0.45
1:A:105:VAL:CG2	4:S:167:ILE:CG1	2.71	0.45
1:A:171:ASN:CG	1:A:202:LYS:NZ	2.69	0.45
1:A:264:SER:HB2	1:A:271:ARG:HG3	1.92	0.45
1:A:533:ILE:CG1	1:A:562:TRP:HH2	2.12	0.45
1:A:633:PHE:CZ	2:B:513:TRP:HE3	2.13	0.45
2:B:29:LYS:HE2	2:B:30:LEU:CB	2.46	0.45
2:B:106:LEU:HD22	2:B:144:ASP:CB	2.45	0.45
2:B:121:ASN:N	2:B:153:ILE:HD13	2.31	0.45
2:B:215:TYR:CE2	2:B:229:HIS:HB2	2.50	0.45
2:B:381:PHE:O	2:B:383:VAL:N	2.49	0.45
2:B:468:LEU:HD13	2:B:507:ALA:HB2	1.96	0.45
2:B:478:LEU:C	2:B:480:GLN:N	2.63	0.45
2:B:512:VAL:CG2	2:B:548:ILE:HG12	2.39	0.45
2:B:538:SER:N	2:B:598:LEU:HD22	2.32	0.45
3:M:323:MET:CG	3:M:342:LEU:HG	2.46	0.45
1:A:224:GLU:O	1:A:227:LYS:N	2.46	0.45
1:A:320:HIS:CB	1:A:352:PHE:CE2	2.84	0.45
1:A:609:LEU:CG	1:A:628:VAL:CG2	2.92	0.45
1:A:631:SER:HA	2:B:554:LYS:HA	1.98	0.45
2:B:37:TYR:CZ	2:B:46:GLN:CD	2.63	0.45
2:B:127:LEU:HD12	2:B:157:THR:CB	2.43	0.45
2:B:133:GLU:O	2:B:168:MET:SD	2.74	0.45
2:B:193:LEU:HB3	2:B:225:LEU:HD11	0.97	0.45
2:B:381:PHE:HD2	2:B:395:LYS:NZ	2.13	0.45
2:B:418:TYR:CG	2:B:419:VAL:N	2.83	0.45
3:M:1:MET:C	3:M:81:SER:CB	2.84	0.45
3:M:111:ILE:HA	3:M:114:ILE:HD12	1.98	0.45
3:M:222:PHE:HB3	3:M:479:PHE:CE1	2.52	0.45
3:M:347:PHE:CZ	3:M:439:TYR:HD2	2.33	0.45
4:S:135:ILE:HG23	4:S:141:VAL:HG11	1.93	0.45
4:S:151:ALA:O	4:S:154:ASP:HB2	2.16	0.45
1:A:102:GLN:HA	4:S:167:ILE:N	2.32	0.45
1:A:102:GLN:C	4:S:163:THR:HB	2.13	0.45
1:A:287:ALA:H	1:A:288:THR:HA	1.81	0.45
1:A:586:GLU:CA	1:A:604:LEU:CD1	2.94	0.45
2:B:274:PRO:CD	2:B:295:ASN:CG	2.78	0.45
2:B:278:PRO:CB	2:B:289:PRO:O	2.64	0.45

Continued on next page...

Continued from previous page...

Atom-1	Atom-2	Interatomic distance (Å)	Clash overlap (Å)
2:B:325:LEU:HD22	2:B:339:PHE:CE2	2.52	0.45
2:B:588:ILE:O	2:B:591:MET:HB2	2.15	0.45
3:M:128:CYS:O	3:M:131:ALA:HB3	2.16	0.45
3:M:378:ILE:HB	3:M:413:GLY:CA	2.44	0.45
4:S:27:LYS:O	4:S:31:LEU:HG	2.17	0.45
4:S:135:ILE:O	4:S:140:MET:O	2.34	0.45
1:A:88:ASN:OD1	1:A:120:ILE:HG21	2.16	0.45
1:A:129:LYS:CG	1:A:161:ASP:HB3	2.47	0.45
1:A:215:VAL:O	1:A:219:VAL:CG2	2.61	0.45
1:A:225:LEU:CG	1:A:233:PHE:CZ	2.99	0.45
1:A:482:ILE:CG1	1:A:517:TRP:CZ3	2.97	0.45
1:A:555:LEU:HD12	1:A:585:PHE:CZ	2.52	0.45
2:B:63:MET:HG2	2:B:100:LEU:CB	2.46	0.45
2:B:334:MET:HE1	2:B:339:PHE:CE1	2.50	0.45
3:M:215:TYR:CB	3:M:468:LYS:CA	2.92	0.45
3:M:224:VAL:CG1	3:M:235:LEU:HG	2.46	0.45
3:M:290:PHE:CD1	3:M:297:PHE:CD1	2.90	0.45
3:M:319:SER:O	3:M:320:ILE:HD13	2.16	0.45
4:S:53:THR:HG22	4:S:54:PRO:O	2.17	0.45
4:S:70:ASN:HB3	4:S:73:ILE:HB	1.96	0.45
4:S:129:GLU:O	4:S:132:LEU:HB3	2.17	0.45
1:A:71:VAL:HG11	1:A:105:VAL:C	2.37	0.45
1:A:141:VAL:HB	4:S:159:ALA:HB2	1.97	0.45
1:A:150:LEU:HD22	1:A:162:ILE:CD1	2.46	0.45
1:A:179:LYS:HD2	4:S:143:GLU:HB2	1.89	0.45
1:A:232:PRO:O	1:A:235:GLN:HB2	2.16	0.45
1:A:433:ILE:HD11	1:A:473:ILE:CG1	2.47	0.45
1:A:617:ASP:OD1	1:A:618:THR:N	2.45	0.45
2:B:47:LEU:HD21	2:B:66:ILE:N	2.31	0.45
2:B:97:VAL:HG12	2:B:101:ILE:HD12	1.98	0.45
2:B:196:LEU:O	2:B:197:LYS:O	2.35	0.45
2:B:241:ASP:HB2	3:M:274:ASP:OD1	2.16	0.45
2:B:279:LEU:CD1	2:B:288:TYR:CE1	2.99	0.45
2:B:394:TRP:CE3	2:B:397:GLN:CD	2.89	0.45
2:B:416:LYS:HE3	2:B:453:TRP:CZ3	2.52	0.45
2:B:463:LEU:HD13	2:B:467:VAL:HG11	1.99	0.45
2:B:511:ILE:O	2:B:515:PHE:CD1	2.66	0.45
2:B:577:ASN:O	2:B:578:PRO:C	2.39	0.45
3:M:8:THR:H	3:M:75:TRP:C	2.17	0.45
3:M:222:PHE:CE2	3:M:240:ILE:HD13	2.52	0.45
3:M:242:GLY:O	3:M:302:TYR:N	2.50	0.45

Continued on next page...

Continued from previous page...

Atom-1	Atom-2	Interatomic distance (Å)	Clash overlap (Å)
3:M:246:VAL:CA	3:M:470:ALA:CB	2.80	0.45
4:S:14:PRO:CB	4:S:36:TYR:HE1	2.25	0.45
4:S:47:GLN:HG2	4:S:84:TYR:HE2	1.81	0.45
1:A:135:ASP:O	1:A:139:ASP:HB3	2.16	0.45
1:A:170:LEU:HD13	1:A:170:LEU:HA	1.82	0.45
1:A:395:PHE:CD1	1:A:428:MET:HG3	2.51	0.45
1:A:625:LEU:C	2:B:617:LEU:HD11	2.33	0.45
1:A:631:SER:HB2	2:B:557:SER:HB3	1.88	0.45
2:B:79:VAL:HG21	2:B:108:PHE:CE1	2.39	0.45
2:B:117:LEU:CD2	2:B:149:SER:CB	2.85	0.45
2:B:136:CYS:SG	2:B:168:MET:CB	3.05	0.45
2:B:155:LEU:HD21	2:B:191:GLU:HB2	1.97	0.45
2:B:279:LEU:O	2:B:280:PRO:C	2.52	0.45
2:B:467:VAL:HG12	2:B:471:TYR:CD1	2.51	0.45
2:B:498:THR:HG22	2:B:532:ARG:HB3	1.98	0.45
3:M:222:PHE:CD2	3:M:439:TYR:OH	2.70	0.45
1:A:464:ILE:O	1:A:464:ILE:HG12	2.17	0.45
1:A:481:MET:O	1:A:482:ILE:C	2.53	0.45
1:A:600:SER:O	1:A:602:GLU:N	2.50	0.45
2:B:74:ASP:O	2:B:77:ILE:HB	2.17	0.45
2:B:106:LEU:CD2	2:B:144:ASP:HB3	2.42	0.45
2:B:196:LEU:CA	2:B:229:HIS:CE1	2.99	0.45
2:B:257:LYS:HA	2:B:260:LEU:HD11	1.99	0.45
2:B:259:TYR:O	2:B:260:LEU:HB2	2.16	0.45
2:B:266:VAL:HG13	2:B:291:TYR:N	2.24	0.45
2:B:292:GLU:HG3	2:B:295:ASN:O	2.17	0.45
2:B:387:ASP:CB	2:B:388:PRO:HD2	2.45	0.45
2:B:390:VAL:CG1	2:B:394:TRP:NE1	2.80	0.45
2:B:461:HIS:C	2:B:463:LEU:N	2.63	0.45
2:B:544:THR:HG22	2:B:548:ILE:HD11	1.99	0.45
3:M:245:ASP:OD1	3:M:297:PHE:O	2.35	0.45
3:M:331:LEU:HD12	3:M:331:LEU:C	2.36	0.45
3:M:376:ILE:HG22	3:M:379:LEU:HD11	1.97	0.45
4:S:118:GLU:O	4:S:122:ILE:HG13	2.16	0.45
1:A:63:ASP:OD1	1:A:64:LEU:N	2.50	0.45
1:A:282:MET:HE3	1:A:294:SER:CB	2.46	0.45
2:B:139:LEU:CD2	2:B:173:VAL:CG1	2.95	0.45
2:B:425:PRO:HB2	2:B:428:VAL:HG23	1.97	0.45
2:B:513:TRP:HE1	2:B:517:GLU:CG	2.30	0.45
2:B:534:ILE:HD12	2:B:591:MET:HA	1.71	0.45
3:M:215:TYR:HB2	3:M:467:TYR:CB	2.47	0.45

Continued on next page...

Continued from previous page...

Atom-1	Atom-2	Interatomic distance (Å)	Clash overlap (Å)
3:M:344:ILE:O	3:M:406:GLY:HA2	2.17	0.45
4:S:55:PRO:HB3	4:S:71:GLU:CA	2.46	0.45
4:S:85:PHE:HE2	4:S:109:LEU:HD22	1.81	0.45
4:S:93:GLU:HB3	4:S:98:ILE:HD11	1.98	0.45
1:A:67:LYS:CA	4:S:166:LYS:HA	2.45	0.45
1:A:145:ILE:HD11	4:S:156:LEU:HD22	1.93	0.45
1:A:211:ASP:OD2	4:S:148:ARG:CZ	2.57	0.45
1:A:298:ILE:HG21	1:A:312:ALA:HB2	1.99	0.45
1:A:300:LYS:O	1:A:301:GLY:C	2.55	0.45
1:A:566:PHE:CE1	1:A:570:LYS:HB3	2.52	0.45
2:B:132:SER:CB	2:B:169:VAL:HG23	2.45	0.45
2:B:141:ALA:HB1	2:B:145:MET:HE1	1.98	0.45
2:B:281:ASP:OD1	2:B:287:GLU:CD	2.55	0.45
2:B:306:LEU:HD22	2:B:321:CYS:CA	2.45	0.45
2:B:314:ASN:O	2:B:317:VAL:HB	2.17	0.45
3:M:52:ASP:CA	3:M:67:SER:HA	2.46	0.45
3:M:347:PHE:CZ	3:M:352:GLN:O	2.70	0.45
3:M:430:LEU:HD23	3:M:483:LEU:HD13	1.98	0.45
1:A:150:LEU:HD22	1:A:162:ILE:HD11	1.99	0.44
1:A:249:ASN:OD1	1:A:251:TRP:N	2.45	0.44
1:A:292:TYR:O	1:A:292:TYR:HD1	1.96	0.44
1:A:388:VAL:HG22	1:A:435:ILE:CD1	2.47	0.44
1:A:396:VAL:O	1:A:396:VAL:CG1	2.65	0.44
1:A:453:VAL:O	1:A:457:LEU:HG	2.16	0.44
1:A:464:ILE:O	1:A:464:ILE:HG23	2.17	0.44
1:A:594:PHE:O	1:A:598:GLU:HG2	2.17	0.44
2:B:208:ILE:CB	2:B:236:ILE:HG21	2.46	0.44
2:B:279:LEU:CD1	2:B:288:TYR:CD1	3.00	0.44
2:B:419:VAL:HG11	2:B:457:HIS:NE2	2.31	0.44
3:M:218:LEU:HD12	3:M:472:TYR:OH	2.16	0.44
3:M:220:GLU:HG3	3:M:439:TYR:CG	2.51	0.44
3:M:224:VAL:CB	3:M:226:PHE:CE1	2.99	0.44
3:M:253:ASN:OD1	3:M:292:PRO:HD2	2.16	0.44
4:S:15:ARG:NE	4:S:122:ILE:HD11	2.29	0.44
1:A:100:LEU:HB3	1:A:142:LYS:CG	2.47	0.44
1:A:103:LYS:O	1:A:104:ARG:O	2.35	0.44
1:A:141:VAL:HG13	4:S:156:LEU:HA	0.85	0.44
1:A:213:SER:O	4:S:143:GLU:CB	2.63	0.44
1:A:240:LEU:C	1:A:242:GLU:O	2.56	0.44
1:A:281:LEU:O	1:A:282:MET:C	2.36	0.44
1:A:326:GLN:HG2	1:A:331:ARG:NH2	2.32	0.44

Continued on next page...

Continued from previous page...

Atom-1	Atom-2	Interatomic distance (Å)	Clash overlap (Å)
1:A:609:LEU:CG	1:A:628:VAL:HG11	2.48	0.44
1:A:638:LEU:HD13	2:B:519:ALA:HB2	1.37	0.44
2:B:162:VAL:HG13	2:B:198:GLU:HB3	1.98	0.44
2:B:201:ALA:HA	2:B:232:ARG:HG2	1.99	0.44
2:B:374:PHE:CZ	2:B:381:PHE:HD1	2.26	0.44
2:B:436:LEU:HB3	2:B:450:VAL:CG1	2.47	0.44
2:B:526:CYS:HB2	2:B:555:LEU:HD11	1.98	0.44
2:B:592:TYR:CE1	2:B:615:SER:HB3	2.53	0.44
2:B:592:TYR:HE1	2:B:615:SER:HB3	1.82	0.44
3:M:18:TYR:HD1	3:M:122:SER:CB	2.16	0.44
3:M:56:VAL:O	3:M:57:GLY:O	2.34	0.44
3:M:220:GLU:OE1	3:M:222:PHE:CZ	2.71	0.44
3:M:347:PHE:CE1	3:M:350:VAL:HG11	2.52	0.44
3:M:380:ARG:HG2	3:M:410:VAL:HB	1.40	0.44
4:S:17:VAL:HG11	4:S:19:PHE:CE1	2.51	0.44
4:S:51:LEU:HD12	4:S:77:TYR:CZ	2.52	0.44
4:S:107:GLU:HG3	4:S:146:VAL:HG11	2.00	0.44
1:A:114:PHE:HZ	1:A:154:ILE:HG12	1.82	0.44
1:A:481:MET:O	1:A:522:PHE:HZ	2.00	0.44
1:A:581:LEU:HG	1:A:585:PHE:CE2	2.52	0.44
1:A:636:TYR:CD2	2:B:513:TRP:HZ2	2.35	0.44
2:B:17:VAL:O	2:B:18:ILE:C	2.54	0.44
2:B:42:ILE:HG12	2:B:65:ARG:CD	2.35	0.44
2:B:135:ARG:O	2:B:161:LEU:CD2	2.66	0.44
2:B:344:VAL:CG2	2:B:381:PHE:CZ	2.98	0.44
2:B:418:TYR:O	2:B:418:TYR:HD1	1.99	0.44
2:B:556:LEU:CD2	2:B:588:ILE:HD11	2.46	0.44
2:B:559:ASP:CA	2:B:562:ASN:HB2	2.36	0.44
3:M:212:ASN:CB	3:M:250:LEU:HA	2.40	0.44
3:M:217:ASP:N	3:M:472:TYR:CE1	2.86	0.44
4:S:102:ILE:O	4:S:105:PHE:HB3	2.17	0.44
1:A:171:ASN:CG	1:A:202:LYS:HZ1	2.18	0.44
2:B:81:LEU:O	2:B:85:ASP:N	2.37	0.44
2:B:109:ALA:O	2:B:110:GLU:O	2.35	0.44
2:B:120:ILE:O	2:B:153:ILE:HG21	2.18	0.44
2:B:197:LYS:HG2	2:B:198:GLU:N	2.32	0.44
2:B:430:ILE:HG23	2:B:470:ALA:HB1	1.97	0.44
2:B:502:SER:O	2:B:503:LEU:O	2.35	0.44
2:B:520:SER:HA	2:B:523:PHE:CD2	2.53	0.44
3:M:74:TYR:HD1	3:M:76:CYS:SG	2.40	0.44
3:M:222:PHE:CE1	3:M:439:TYR:CD1	3.05	0.44

Continued on next page...

Continued from previous page...

Atom-1	Atom-2	Interatomic distance (Å)	Clash overlap (Å)
3:M:228:LYS:HZ1	3:M:326:HIS:HA	1.81	0.44
3:M:269:ILE:N	3:M:302:TYR:CE1	2.86	0.44
3:M:340:LEU:HG	3:M:342:LEU:CD1	2.48	0.44
3:M:424:PHE:HZ	3:M:428:VAL:HG23	1.82	0.44
4:S:51:LEU:N	4:S:75:ILE:O	2.50	0.44
4:S:80:TYR:CB	4:S:106:VAL:HG11	2.47	0.44
1:A:114:PHE:CD1	1:A:114:PHE:C	2.91	0.44
1:A:219:VAL:O	4:S:140:MET:HE1	2.18	0.44
1:A:365:VAL:O	1:A:366:SER:C	2.46	0.44
2:B:63:MET:N	2:B:66:ILE:HD12	2.32	0.44
2:B:116:THR:O	2:B:150:LEU:HD22	2.17	0.44
2:B:195:ILE:HG22	2:B:196:LEU:N	2.31	0.44
2:B:382:TYR:HE1	2:B:399:LEU:HD21	1.83	0.44
2:B:556:LEU:O	2:B:563:PHE:HD1	2.01	0.44
2:B:589:SER:C	2:B:591:MET:H	2.20	0.44
3:M:258:VAL:CB	3:M:452:ILE:HG13	2.48	0.44
1:A:237:SER:HB2	1:A:270:LEU:HD22	2.00	0.44
1:A:392:MET:HE3	1:A:428:MET:CE	2.46	0.44
1:A:396:VAL:C	1:A:397:ASP:OD1	2.56	0.44
1:A:438:ALA:O	1:A:441:TYR:HD1	1.97	0.44
1:A:603:VAL:O	1:A:606:PHE:CB	2.62	0.44
2:B:42:ILE:HG22	2:B:43:ASN:O	2.17	0.44
2:B:47:LEU:O	2:B:50:LEU:HB3	2.18	0.44
2:B:234:CYS:HB2	2:B:301:LEU:HB3	1.99	0.44
2:B:281:ASP:C	2:B:283:TYR:H	2.20	0.44
2:B:493:LEU:CD2	2:B:511:ILE:HA	2.48	0.44
3:M:223:HIS:CD2	3:M:478:ASN:HB3	2.50	0.44
3:M:437:TYR:CG	3:M:439:TYR:OH	2.70	0.44
4:S:34:GLN:HB3	4:S:58:LEU:CD1	2.48	0.44
1:A:132:LEU:HD13	1:A:165:ASP:HB2	1.95	0.44
1:A:179:LYS:NZ	4:S:149:ILE:CG1	2.77	0.44
2:B:140:SER:OG	2:B:175:LEU:CD1	2.66	0.44
2:B:212:VAL:CG2	2:B:248:LEU:HD23	2.42	0.44
2:B:212:VAL:HG22	2:B:248:LEU:HD23	1.93	0.44
2:B:315:PRO:O	2:B:318:ILE:HB	2.17	0.44
2:B:483:PRO:O	2:B:486:HIS:HB3	2.17	0.44
2:B:556:LEU:CB	2:B:588:ILE:CD1	2.85	0.44
3:M:258:VAL:CG2	3:M:452:ILE:HG23	2.47	0.44
3:M:290:PHE:CD2	3:M:297:PHE:CZ	3.06	0.44
3:M:372:ILE:CD1	3:M:428:VAL:HG13	2.48	0.44
3:M:433:VAL:CG1	3:M:433:VAL:O	2.66	0.44

Continued on next page...

Continued from previous page...

Atom-1	Atom-2	Interatomic distance (Å)	Clash overlap (Å)
4:S:3:HIS:O	4:S:19:PHE:CA	2.62	0.44
4:S:80:TYR:CB	4:S:106:VAL:CG1	2.96	0.44
4:S:148:ARG:O	4:S:151:ALA:N	2.51	0.44
1:A:200:PHE:O	1:A:203:PHE:HB2	2.18	0.44
1:A:392:MET:CE	1:A:428:MET:CE	2.96	0.44
2:B:158:VAL:HG21	2:B:177:ILE:CG1	2.47	0.44
2:B:230:PHE:CD2	2:B:298:ASP:CA	3.01	0.44
2:B:305:SER:O	2:B:309:LEU:HD23	2.17	0.44
2:B:498:THR:CG2	2:B:532:ARG:HB2	2.47	0.44
2:B:537:PHE:O	2:B:539:ASN:N	2.50	0.44
3:M:219:LEU:H	3:M:472:TYR:CB	2.31	0.44
3:M:220:GLU:OE2	3:M:439:TYR:HB2	2.18	0.44
3:M:220:GLU:OE1	3:M:222:PHE:CE1	2.70	0.44
3:M:253:ASN:OD1	3:M:292:PRO:CD	2.65	0.44
3:M:347:PHE:CE2	3:M:352:GLN:O	2.70	0.44
4:S:39:ILE:CG1	4:S:77:TYR:CD2	3.01	0.44
4:S:87:PHE:CD1	4:S:102:ILE:CG1	2.89	0.44
1:A:128:LEU:HD22	1:A:146:ALA:HB1	1.99	0.44
1:A:139:ASP:CG	1:A:177:ILE:CD1	2.86	0.44
1:A:213:SER:CA	4:S:143:GLU:OE1	2.66	0.44
1:A:275:LEU:CD1	1:A:308:ASP:CB	2.95	0.44
1:A:323:CYS:HB3	1:A:355:LEU:HD21	1.97	0.44
1:A:399:ASP:OD1	1:A:420:ILE:CG2	2.66	0.44
1:A:581:LEU:HG	1:A:607:LEU:CD1	2.48	0.44
1:A:594:PHE:CD2	2:B:474:VAL:HG23	2.47	0.44
2:B:162:VAL:HG11	2:B:195:ILE:O	2.18	0.44
2:B:390:VAL:HG12	2:B:394:TRP:NE1	2.32	0.44
2:B:440:GLY:C	2:B:442:LEU:H	2.22	0.44
2:B:490:ILE:HG23	2:B:515:PHE:CZ	2.51	0.44
3:M:3:LEU:O	3:M:20:LEU:HD12	2.18	0.44
3:M:7:ILE:HG21	3:M:114:ILE:HD13	2.00	0.44
3:M:219:LEU:CG	3:M:440:ILE:HG12	2.45	0.44
3:M:223:HIS:CA	3:M:479:PHE:CZ	2.98	0.44
3:M:319:SER:HB3	3:M:346:ASN:CG	2.37	0.44
3:M:323:MET:HE3	3:M:342:LEU:CD2	2.35	0.44
4:S:48:SER:CB	4:S:78:LYS:N	2.76	0.44
4:S:50:PHE:CB	4:S:76:ILE:CD1	2.93	0.44
4:S:75:ILE:HB	4:S:77:TYR:CE1	2.52	0.44
1:A:114:PHE:CZ	1:A:154:ILE:HG12	2.53	0.43
1:A:178:ARG:CB	1:A:214:VAL:CG1	2.95	0.43
1:A:317:GLU:HG2	1:A:351:ARG:HH12	1.83	0.43

Continued on next page...

Continued from previous page...

Atom-1	Atom-2	Interatomic distance (Å)	Clash overlap (Å)
1:A:420:ILE:HG22	1:A:421:PRO:O	2.18	0.43
1:A:429:VAL:HG13	1:A:457:LEU:HD13	1.99	0.43
1:A:509:PRO:HA	1:A:547:VAL:HG21	1.98	0.43
2:B:127:LEU:HD22	2:B:157:THR:CG2	2.48	0.43
2:B:197:LYS:H	2:B:229:HIS:CE1	2.33	0.43
2:B:227:HIS:O	2:B:229:HIS:CA	2.58	0.43
2:B:467:VAL:HG12	2:B:471:TYR:HD1	1.81	0.43
2:B:537:PHE:CZ	2:B:545:ARG:HD3	2.52	0.43
2:B:589:SER:HA	2:B:592:TYR:CD2	2.45	0.43
3:M:380:ARG:HH11	3:M:412:ARG:NH1	2.16	0.43
4:S:6:LEU:HB3	4:S:86:THR:HB	1.99	0.43
4:S:127:THR:HG22	4:S:153:VAL:HG13	2.00	0.43
1:A:150:LEU:HD13	1:A:162:ILE:HG23	2.00	0.43
1:A:179:LYS:HG3	4:S:143:GLU:HG3	1.50	0.43
1:A:249:ASN:HB3	1:A:252:ILE:HD12	2.00	0.43
1:A:486:SER:O	1:A:487:MET:HB2	2.18	0.43
1:A:563:CYS:O	1:A:566:PHE:CD2	2.71	0.43
2:B:171:GLY:H	2:B:207:VAL:HG13	1.66	0.43
2:B:208:ILE:O	2:B:210:CYS:N	2.51	0.43
2:B:340:ILE:CG2	2:B:373:LEU:HG	2.48	0.43
2:B:343:LEU:O	2:B:359:LEU:HD12	2.17	0.43
2:B:398:ILE:C	2:B:400:SER:N	2.69	0.43
2:B:483:PRO:CB	2:B:521:ILE:CG2	2.65	0.43
3:M:100:LEU:HD22	3:M:100:LEU:C	2.36	0.43
3:M:214:LEU:N	3:M:467:TYR:CB	2.81	0.43
3:M:319:SER:O	3:M:320:ILE:HG12	2.18	0.43
3:M:372:ILE:HD12	3:M:428:VAL:CG2	2.48	0.43
3:M:376:ILE:CD1	3:M:415:ILE:HG23	2.48	0.43
3:M:450:GLU:OE1	3:M:450:GLU:HA	2.18	0.43
4:S:15:ARG:HE	4:S:118:GLU:HB2	1.84	0.43
4:S:55:PRO:O	4:S:57:LEU:N	2.51	0.43
1:A:182:ILE:HG23	1:A:221:VAL:CG2	2.39	0.43
1:A:264:SER:HB2	1:A:271:ARG:NE	2.30	0.43
1:A:320:HIS:HA	1:A:338:PHE:HZ	1.82	0.43
1:A:548:GLN:NE2	1:A:588:LEU:HD11	2.32	0.43
1:A:582:ILE:HG23	1:A:604:LEU:HG	2.00	0.43
1:A:621:LEU:O	1:A:621:LEU:HD13	2.18	0.43
1:A:634:ASN:HA	2:B:516:GLY:CA	2.48	0.43
2:B:14:THR:C	2:B:16:LYS:N	2.71	0.43
2:B:237:ILE:HB	2:B:248:LEU:HD13	2.00	0.43
2:B:359:LEU:HD13	2:B:359:LEU:HA	1.88	0.43

Continued on next page...

Continued from previous page...

Atom-1	Atom-2	Interatomic distance (Å)	Clash overlap (Å)
2:B:580:TYR:C	2:B:582:ASP:N	2.72	0.43
2:B:589:SER:O	2:B:592:TYR:N	2.51	0.43
2:B:591:MET:O	2:B:595:VAL:HG23	2.18	0.43
3:M:61:GLU:OE2	3:M:82:LYS:HB2	2.19	0.43
4:S:16:LEU:CB	4:S:125:TRP:CD1	3.01	0.43
1:A:295:VAL:HG22	1:A:315:CYS:CB	2.19	0.43
1:A:309:PHE:CZ	1:A:348:PHE:CE1	3.07	0.43
1:A:532:LEU:O	1:A:535:ILE:N	2.51	0.43
1:A:569:ASP:C	1:A:571:ARG:N	2.65	0.43
1:A:576:MET:O	1:A:579:LYS:HB3	2.18	0.43
1:A:637:GLU:HG2	2:B:512:VAL:O	2.19	0.43
3:M:9:ASP:CG	3:M:13:LYS:HB3	2.38	0.43
3:M:67:SER:CB	3:M:90:PHE:CB	2.96	0.43
3:M:326:HIS:O	3:M:338:PHE:CA	2.50	0.43
3:M:473:LYS:C	3:M:474:THR:CG2	2.86	0.43
1:A:128:LEU:HD12	1:A:150:LEU:HD23	1.69	0.43
1:A:200:PHE:HZ	1:A:235:GLN:HB2	1.82	0.43
1:A:224:GLU:N	4:S:140:MET:HE3	2.31	0.43
1:A:253:ILE:CG2	1:A:281:LEU:CB	2.97	0.43
1:A:552:ILE:CD1	1:A:600:SER:CB	2.96	0.43
1:A:563:CYS:CA	1:A:566:PHE:HD2	2.11	0.43
2:B:24:ALA:HB2	2:B:35:TYR:CZ	2.36	0.43
2:B:63:MET:HG2	2:B:101:ILE:N	2.33	0.43
2:B:158:VAL:HG13	2:B:177:ILE:HG13	1.91	0.43
2:B:178:ILE:HG23	2:B:217:GLU:N	2.33	0.43
2:B:455:ILE:HD11	2:B:489:ILE:HG23	1.99	0.43
2:B:537:PHE:CZ	2:B:598:LEU:C	2.91	0.43
2:B:563:PHE:CE2	2:B:582:ASP:HB2	2.52	0.43
3:M:220:GLU:O	3:M:439:TYR:HD1	2.02	0.43
3:M:355:ASP:O	3:M:356:LEU:C	2.55	0.43
3:M:373:ALA:O	3:M:417:TYR:HA	2.18	0.43
4:S:34:GLN:CD	4:S:58:LEU:CG	2.83	0.43
4:S:34:GLN:C	4:S:36:TYR:H	2.21	0.43
4:S:107:GLU:HG3	4:S:146:VAL:CG1	2.48	0.43
4:S:111:ARG:HB3	4:S:150:VAL:CG2	2.47	0.43
4:S:117:ASN:CG	4:S:120:ASP:OD2	2.57	0.43
1:A:402:ILE:O	1:A:403:LEU:C	2.54	0.43
1:A:631:SER:CA	2:B:554:LYS:HA	2.49	0.43
2:B:37:TYR:CE2	2:B:46:GLN:CD	2.87	0.43
2:B:123:LEU:C	2:B:127:LEU:CD1	2.86	0.43
2:B:151:ALA:CB	2:B:188:TYR:CE1	3.01	0.43

Continued on next page...

Continued from previous page...

Atom-1	Atom-2	Interatomic distance (Å)	Clash overlap (Å)
2:B:159:LYS:CG	2:B:191:GLU:OE1	2.66	0.43
2:B:326:TYR:O	2:B:327:GLN:C	2.56	0.43
2:B:334:MET:CE	2:B:334:MET:CA	2.86	0.43
2:B:404:ASN:C	2:B:408:VAL:HG23	2.36	0.43
2:B:433:VAL:HG11	2:B:474:VAL:CG2	2.35	0.43
3:M:24:ALA:C	3:M:25:PRO:O	2.50	0.43
3:M:118:TYR:HA	3:M:121:ILE:HD12	2.01	0.43
3:M:304:VAL:CG1	3:M:445:SER:HA	2.43	0.43
3:M:374:TYR:CE1	3:M:390:ILE:CD1	3.02	0.43
1:A:104:ARG:CG	1:A:145:ILE:CG1	2.66	0.43
1:A:144:GLY:CA	1:A:180:LYS:HG3	2.49	0.43
1:A:429:VAL:CG2	1:A:469:LEU:HD21	2.40	0.43
1:A:626:SER:O	1:A:630:PRO:CD	2.66	0.43
2:B:116:THR:CG2	2:B:150:LEU:HD21	2.41	0.43
2:B:212:VAL:HG23	2:B:233:TYR:CD2	2.53	0.43
2:B:287:GLU:OE2	2:B:287:GLU:HA	2.18	0.43
2:B:367:SER:CB	2:B:401:THR:OG1	2.67	0.43
2:B:410:GLU:HA	2:B:413:LYS:HE2	2.01	0.43
3:M:103:TYR:CE1	3:M:124:ILE:HG12	2.54	0.43
3:M:271:SER:C	3:M:272:LEU:HD12	2.39	0.43
3:M:380:ARG:HG2	3:M:410:VAL:O	2.19	0.43
4:S:4:ALA:HB1	4:S:19:PHE:CD2	2.53	0.43
4:S:14:PRO:CB	4:S:36:TYR:CE1	3.00	0.43
4:S:75:ILE:CG2	4:S:86:THR:HG21	2.44	0.43
4:S:87:PHE:CZ	4:S:102:ILE:HA	2.53	0.43
1:A:125:THR:CA	1:A:158:LEU:HD13	2.49	0.43
1:A:128:LEU:HB3	1:A:150:LEU:HD11	1.99	0.43
1:A:219:VAL:CG2	1:A:240:LEU:HD22	2.28	0.43
1:A:432:ILE:CG2	1:A:457:LEU:HD11	2.39	0.43
1:A:524:THR:CG2	1:A:565:ASN:ND2	2.81	0.43
2:B:13:ASP:O	2:B:14:THR:O	2.36	0.43
2:B:108:PHE:HE2	2:B:115:LEU:HD23	1.82	0.43
2:B:230:PHE:CG	2:B:298:ASP:HB3	2.42	0.43
2:B:279:LEU:HB3	2:B:280:PRO:HD2	2.01	0.43
2:B:329:ALA:HA	2:B:333:GLN:NE2	2.34	0.43
2:B:367:SER:O	2:B:368:ILE:C	2.55	0.43
2:B:537:PHE:CD2	2:B:537:PHE:O	2.71	0.43
2:B:563:PHE:C	2:B:564:LYS:O	2.56	0.43
2:B:567:GLN:OE1	2:B:618:PHE:CE1	2.71	0.43
3:M:94:GLU:O	3:M:97:ASP:CB	2.64	0.43
3:M:306:LEU:HD13	3:M:317:MET:HE3	1.99	0.43

Continued on next page...

Continued from previous page...

Atom-1	Atom-2	Interatomic distance (Å)	Clash overlap (Å)
3:M:377:LYS:HE3	3:M:416:GLU:HB2	1.97	0.43
4:S:56:SER:C	4:S:60:SER:HB2	2.34	0.43
1:A:107:TYR:HD1	1:A:145:ILE:HG22	1.84	0.43
1:A:478:ARG:HG3	1:A:517:TRP:CE3	2.54	0.43
1:A:589:SER:HB3	1:A:601:VAL:CG2	2.48	0.43
2:B:139:LEU:HD21	2:B:176:ALA:HB3	1.97	0.43
2:B:237:ILE:HD13	2:B:309:LEU:HD21	2.01	0.43
2:B:546:CYS:SG	2:B:607:ILE:CA	3.06	0.43
2:B:556:LEU:HA	2:B:588:ILE:HD11	0.49	0.43
2:B:577:ASN:OD1	2:B:577:ASN:N	2.52	0.43
2:B:588:ILE:HG23	2:B:589:SER:N	2.34	0.43
3:M:343:ASN:ND2	3:M:343:ASN:N	2.56	0.43
3:M:351:SER:O	3:M:352:GLN:HB3	2.19	0.43
3:M:435:LEU:O	3:M:437:TYR:HE1	2.02	0.43
4:S:38:LEU:HB3	4:S:51:LEU:CD1	2.47	0.43
1:A:92:LEU:CD1	1:A:120:ILE:HA	2.15	0.43
1:A:121:LEU:O	1:A:124:ALA:HB3	2.18	0.43
1:A:395:PHE:CZ	1:A:428:MET:HB2	2.54	0.43
2:B:83:PHE:CE2	2:B:119:SER:N	2.84	0.43
2:B:98:LYS:CG	2:B:137:PHE:HB2	2.48	0.43
2:B:123:LEU:CD2	2:B:138:ALA:CA	2.97	0.43
2:B:130:SER:O	2:B:135:ARG:NH2	2.52	0.43
2:B:200:MET:HE2	2:B:229:HIS:HA	1.69	0.43
2:B:374:PHE:HB3	2:B:402:LEU:HD21	2.01	0.43
3:M:96:ILE:HG21	3:M:125:PHE:CE2	2.54	0.43
3:M:226:PHE:CG	3:M:481:VAL:HG22	2.53	0.43
3:M:242:GLY:CA	3:M:474:THR:CG2	2.94	0.43
3:M:374:TYR:CE1	3:M:376:ILE:HD11	2.54	0.43
4:S:16:LEU:HD11	4:S:129:GLU:CG	2.49	0.43
4:S:17:VAL:CG1	4:S:19:PHE:HE1	2.24	0.43
1:A:63:ASP:C	1:A:63:ASP:OD1	2.57	0.42
1:A:226:SER:CB	1:A:263:LEU:HD23	2.49	0.42
1:A:379:VAL:HG22	1:A:380:ASP:N	2.34	0.42
1:A:555:LEU:O	1:A:558:VAL:HB	2.19	0.42
2:B:59:VAL:O	2:B:59:VAL:CG1	2.66	0.42
2:B:60:ARG:O	2:B:100:LEU:HD13	2.18	0.42
2:B:80:GLN:HA	2:B:115:LEU:CD1	2.48	0.42
2:B:83:PHE:HZ	2:B:119:SER:OG	1.94	0.42
2:B:127:LEU:CD2	2:B:139:LEU:HB2	2.49	0.42
2:B:159:LYS:HD2	2:B:191:GLU:CD	2.39	0.42
2:B:261:PRO:C	2:B:290:SER:CB	2.86	0.42

Continued on next page...

Continued from previous page...

Atom-1	Atom-2	Interatomic distance (Å)	Clash overlap (Å)
2:B:285:GLU:N	2:B:285:GLU:OE2	2.52	0.42
2:B:366:LEU:O	2:B:368:ILE:N	2.48	0.42
2:B:397:GLN:O	2:B:400:SER:OG	2.16	0.42
2:B:520:SER:C	2:B:523:PHE:HD2	2.22	0.42
2:B:588:ILE:HG21	2:B:618:PHE:CE1	2.54	0.42
3:M:60:LEU:CD2	3:M:60:LEU:C	2.87	0.42
3:M:215:TYR:HD2	3:M:469:GLY:C	2.23	0.42
3:M:240:ILE:HG21	3:M:444:ALA:HA	2.01	0.42
3:M:466:LEU:HD23	3:M:467:TYR:O	2.18	0.42
1:A:250:ASN:CG	1:A:285:THR:HB	2.40	0.42
1:A:375:VAL:O	1:A:376:GLU:C	2.57	0.42
1:A:419:ILE:O	1:A:419:ILE:HG12	2.20	0.42
1:A:507:GLN:O	1:A:509:PRO:HD2	2.18	0.42
1:A:509:PRO:CA	1:A:547:VAL:HG21	2.49	0.42
1:A:532:LEU:O	1:A:535:ILE:C	2.56	0.42
1:A:552:ILE:CD1	1:A:600:SER:HB2	2.48	0.42
1:A:575:LYS:CE	1:A:611:LEU:HD21	2.46	0.42
2:B:127:LEU:CD2	2:B:157:THR:HG21	2.41	0.42
2:B:167:ALA:O	2:B:202:ASP:CG	2.56	0.42
2:B:174:ALA:C	2:B:214:ALA:HB2	2.40	0.42
2:B:230:PHE:HB3	2:B:298:ASP:CG	2.38	0.42
2:B:230:PHE:HD2	2:B:298:ASP:CA	2.32	0.42
2:B:277:CYS:O	2:B:288:TYR:HA	2.18	0.42
2:B:393:ILE:HG21	2:B:431:MET:HE2	2.01	0.42
2:B:560:ILE:CG2	2:B:561:ASP:N	2.79	0.42
2:B:560:ILE:O	2:B:563:PHE:N	2.51	0.42
3:M:7:ILE:HG23	3:M:75:TRP:C	2.38	0.42
4:S:5:VAL:O	4:S:17:VAL:CA	2.55	0.42
4:S:56:SER:C	4:S:57:LEU:HD12	2.39	0.42
1:A:147:LEU:CG	1:A:166:LEU:HD21	2.45	0.42
1:A:222:ILE:CG2	1:A:233:PHE:HB3	2.42	0.42
1:A:359:LEU:HD22	1:A:367:ILE:CG2	2.48	0.42
1:A:450:TYR:OH	1:A:476:GLN:NE2	2.52	0.42
2:B:139:LEU:CD2	2:B:173:VAL:HG13	2.49	0.42
2:B:549:LEU:CD1	2:B:611:ALA:HB1	2.21	0.42
3:M:226:PHE:CE2	3:M:321:GLY:O	2.69	0.42
3:M:336:ASP:CG	3:M:415:ILE:HB	2.37	0.42
1:A:75:THR:O	1:A:79:MET:HG3	2.19	0.42
1:A:102:GLN:N	4:S:163:THR:C	2.71	0.42
1:A:213:SER:N	4:S:148:ARG:CD	2.57	0.42
1:A:514:GLU:OE1	1:A:514:GLU:HA	2.20	0.42

Continued on next page...

Continued from previous page...

Atom-1	Atom-2	Interatomic distance (Å)	Clash overlap (Å)
2:B:18:ILE:HD13	2:B:18:ILE:N	2.32	0.42
2:B:47:LEU:HD11	2:B:66:ILE:HA	1.98	0.42
2:B:189:HIS:CD2	2:B:222:HIS:CG	3.08	0.42
2:B:245:GLN:HB3	2:B:309:LEU:HD11	2.02	0.42
2:B:292:GLU:CD	2:B:296:ASP:OD2	2.50	0.42
2:B:303:LEU:CD1	2:B:333:GLN:HG2	2.45	0.42
2:B:310:ILE:HG12	2:B:318:ILE:HG23	2.01	0.42
2:B:350:THR:HA	2:B:351:GLU:OE2	2.20	0.42
2:B:403:ILE:CA	2:B:411:ILE:HD12	2.48	0.42
2:B:437:SER:CA	2:B:478:LEU:CD2	2.71	0.42
2:B:589:SER:O	2:B:591:MET:N	2.52	0.42
3:M:220:GLU:O	3:M:439:TYR:CD1	2.73	0.42
3:M:243:ILE:C	3:M:472:TYR:CD2	2.89	0.42
3:M:276:VAL:HG22	3:M:290:PHE:HD1	1.84	0.42
3:M:410:VAL:HG11	3:M:412:ARG:HH11	1.80	0.42
1:A:143:VAL:HG13	1:A:169:MET:HG3	2.00	0.42
1:A:213:SER:HB2	4:S:143:GLU:HB3	2.01	0.42
1:A:288:THR:OG1	1:A:291:ILE:CG1	2.67	0.42
1:A:339:TYR:HB2	1:A:374:LEU:HD11	2.01	0.42
1:A:349:ILE:O	1:A:350:SER:C	2.56	0.42
1:A:483:LYS:O	1:A:484:VAL:CB	2.65	0.42
2:B:37:TYR:C	2:B:39:SER:H	2.20	0.42
2:B:106:LEU:CD2	2:B:144:ASP:HB2	2.48	0.42
2:B:121:ASN:HA	2:B:153:ILE:HG21	2.01	0.42
2:B:256:CYS:O	2:B:260:LEU:N	2.53	0.42
2:B:340:ILE:CD1	2:B:366:LEU:HD13	2.23	0.42
2:B:399:LEU:HD12	2:B:415:LEU:HD23	2.01	0.42
3:M:243:ILE:HD13	3:M:301:GLU:HB3	2.00	0.42
1:A:102:GLN:C	4:S:167:ILE:HG12	2.32	0.42
1:A:200:PHE:CZ	1:A:236:LEU:HG	2.54	0.42
1:A:348:PHE:HB3	1:A:352:PHE:CE1	2.53	0.42
1:A:552:ILE:CG2	1:A:603:VAL:HG21	2.45	0.42
1:A:555:LEU:HD12	1:A:585:PHE:HE1	1.83	0.42
1:A:558:VAL:C	1:A:560:SER:N	2.73	0.42
2:B:50:LEU:CD1	2:B:58:GLU:HB3	2.49	0.42
2:B:178:ILE:CG2	2:B:217:GLU:CB	2.75	0.42
2:B:195:ILE:O	2:B:196:LEU:C	2.55	0.42
2:B:307:ASN:O	2:B:308:CYS:C	2.57	0.42
2:B:418:TYR:CE1	2:B:419:VAL:HG22	2.53	0.42
2:B:475:ILE:HG21	2:B:514:LEU:HD21	1.98	0.42
1:A:171:ASN:HB2	1:A:202:LYS:HE2	2.02	0.42

Continued on next page...

Continued from previous page...

Atom-1	Atom-2	Interatomic distance (Å)	Clash overlap (Å)
1:A:182:ILE:HG21	1:A:217:ALA:C	2.39	0.42
1:A:564:ASN:OD1	1:A:622:PRO:HG2	2.17	0.42
1:A:623:MET:O	2:B:617:LEU:HG	2.19	0.42
2:B:37:TYR:CD2	2:B:42:ILE:HA	2.53	0.42
2:B:62:ALA:C	2:B:66:ILE:HD11	2.34	0.42
2:B:215:TYR:OH	2:B:229:HIS:HB3	2.17	0.42
2:B:396:ILE:HD13	2:B:418:TYR:CZ	2.47	0.42
2:B:534:ILE:HA	2:B:598:LEU:HD12	2.02	0.42
2:B:556:LEU:HD12	2:B:614:ILE:CG2	2.49	0.42
2:B:592:TYR:CD1	2:B:593:ASN:N	2.88	0.42
3:M:2:TYR:C	3:M:81:SER:CB	2.77	0.42
3:M:428:VAL:O	3:M:430:LEU:HA	2.19	0.42
4:S:47:GLN:O	4:S:49:SER:N	2.49	0.42
4:S:50:PHE:CA	4:S:77:TYR:HD1	2.28	0.42
4:S:122:ILE:O	4:S:123:PHE:C	2.56	0.42
1:A:91:ILE:HD12	1:A:109:ALA:HB3	2.01	0.42
1:A:100:LEU:HD22	1:A:138:ASN:CB	2.48	0.42
1:A:183:THR:N	4:S:142:ILE:HD13	2.34	0.42
1:A:274:LEU:O	1:A:277:LYS:N	2.52	0.42
1:A:634:ASN:ND2	2:B:554:LYS:H	2.15	0.42
2:B:47:LEU:HD22	2:B:66:ILE:CA	2.50	0.42
2:B:154:ILE:HG21	2:B:180:LEU:CB	2.50	0.42
2:B:336:ASN:HD21	2:B:338:LYS:HB2	1.80	0.42
2:B:383:VAL:CG2	2:B:384:PHE:N	2.83	0.42
2:B:560:ILE:HG22	2:B:561:ASP:H	1.83	0.42
3:M:219:LEU:H	3:M:472:TYR:HB2	1.84	0.42
3:M:245:ASP:C	3:M:246:VAL:HG23	2.40	0.42
4:S:1:MET:H3	4:S:93:GLU:HB2	1.85	0.42
1:A:66:SER:N	4:S:165:SER:CB	2.66	0.42
1:A:97:SER:CA	1:A:98:ASN:O	2.66	0.42
1:A:219:VAL:CG2	1:A:256:LEU:HD23	2.48	0.42
1:A:271:ARG:HH11	1:A:303:MET:HA	1.85	0.42
1:A:336:ILE:HG23	1:A:340:LYS:HE3	2.02	0.42
1:A:401:VAL:HB	1:A:419:ILE:HB	2.02	0.42
2:B:105:LEU:CB	2:B:145:MET:HE1	2.39	0.42
2:B:114:ASN:O	2:B:117:LEU:CB	2.63	0.42
2:B:216:LYS:HB2	2:B:251:LEU:CB	2.50	0.42
2:B:380:LYS:HZ2	3:M:237:THR:CG2	2.33	0.42
2:B:562:ASN:CG	2:B:580:TYR:CG	2.92	0.42
3:M:74:TYR:CE1	3:M:76:CYS:SG	3.13	0.42
3:M:265:ASN:OD1	3:M:313:SER:HB3	2.15	0.42

Continued on next page...

Continued from previous page...

Atom-1	Atom-2	Interatomic distance (Å)	Clash overlap (Å)
4:S:16:LEU:CD1	4:S:17:VAL:N	2.80	0.42
4:S:47:GLN:NE2	4:S:47:GLN:C	2.73	0.42
1:A:121:LEU:HD11	1:A:155:THR:OG1	2.20	0.42
1:A:213:SER:HB2	4:S:143:GLU:OE1	2.12	0.42
1:A:263:LEU:O	1:A:264:SER:C	2.53	0.42
1:A:288:THR:OG1	1:A:291:ILE:HD13	2.19	0.42
1:A:421:PRO:CB	1:A:424:TYR:CE1	3.03	0.42
1:A:524:THR:CG2	1:A:565:ASN:HD22	2.32	0.42
1:A:552:ILE:HD13	1:A:600:SER:HB2	2.01	0.42
1:A:627:GLU:HA	2:B:617:LEU:HB2	1.18	0.42
2:B:13:ASP:O	2:B:14:THR:C	2.59	0.42
2:B:90:ILE:O	2:B:98:LYS:CE	2.68	0.42
2:B:151:ALA:C	2:B:153:ILE:H	2.24	0.42
2:B:158:VAL:CB	2:B:177:ILE:CG1	2.89	0.42
2:B:172:GLU:OE1	2:B:175:LEU:HD12	2.19	0.42
2:B:176:ALA:O	2:B:178:ILE:C	2.58	0.42
2:B:177:ILE:CD1	2:B:196:LEU:CG	2.96	0.42
2:B:240:LEU:HB3	2:B:241:ASP:H	1.77	0.42
2:B:408:VAL:HG13	2:B:412:PHE:CE2	2.48	0.42
2:B:527:PRO:HB3	2:B:587:ARG:O	2.20	0.42
3:M:218:LEU:N	3:M:218:LEU:CD1	2.82	0.42
3:M:222:PHE:HD1	3:M:222:PHE:N	2.18	0.42
4:S:7:ILE:CG2	4:S:121:LEU:HD23	2.50	0.42
4:S:14:PRO:C	4:S:15:ARG:HG2	2.40	0.42
1:A:134:TYR:CD2	1:A:136:GLY:N	2.84	0.41
1:A:180:LYS:O	1:A:183:THR:N	2.53	0.41
1:A:183:THR:OG1	4:S:142:ILE:CG1	2.65	0.41
1:A:226:SER:HB3	1:A:263:LEU:CD2	2.50	0.41
1:A:253:ILE:HG21	1:A:281:LEU:CB	2.47	0.41
1:A:374:LEU:CA	1:A:376:GLU:OE1	2.64	0.41
1:A:429:VAL:O	1:A:432:ILE:HB	2.19	0.41
1:A:509:PRO:CA	1:A:547:VAL:CG2	2.98	0.41
2:B:106:LEU:HD22	2:B:144:ASP:HB2	2.02	0.41
2:B:131:ASN:O	2:B:135:ARG:HG3	2.20	0.41
2:B:135:ARG:CZ	2:B:164:ASP:CB	2.95	0.41
2:B:135:ARG:O	2:B:161:LEU:HD21	2.20	0.41
2:B:154:ILE:O	2:B:157:THR:HB	2.20	0.41
2:B:227:HIS:ND1	2:B:292:GLU:HG2	2.35	0.41
2:B:306:LEU:CD1	2:B:325:LEU:HD23	2.44	0.41
2:B:424:PHE:HA	2:B:425:PRO:HD3	1.58	0.41
2:B:447:GLU:HG3	2:B:482:ASN:ND2	2.35	0.41

Continued on next page...

Continued from previous page...

Atom-1	Atom-2	Interatomic distance (Å)	Clash overlap (Å)
3:M:95:THR:HG22	3:M:99:ILE:HD11	2.01	0.41
3:M:214:LEU:C	3:M:214:LEU:CD2	2.80	0.41
3:M:283:PHE:CD2	3:M:289:THR:OG1	2.60	0.41
3:M:380:ARG:HB3	3:M:412:ARG:N	2.35	0.41
3:M:381:ASN:HD21	3:M:384:GLY:HA3	1.83	0.41
3:M:386:PHE:HB2	3:M:397:TRP:HD1	1.81	0.41
3:M:445:SER:OG	3:M:447:ILE:HG23	2.19	0.41
4:S:47:GLN:HE22	4:S:78:LYS:C	2.22	0.41
4:S:53:THR:OG1	4:S:68:VAL:CA	2.68	0.41
4:S:101:LEU:HG	4:S:135:ILE:CG2	2.49	0.41
1:A:100:LEU:CB	1:A:142:LYS:CG	2.98	0.41
1:A:140:VAL:CG2	1:A:174:ARG:CB	2.97	0.41
1:A:185:LEU:HD22	1:A:189:PHE:HZ	1.80	0.41
1:A:245:VAL:O	1:A:245:VAL:HG22	2.20	0.41
2:B:153:ILE:O	2:B:154:ILE:C	2.58	0.41
3:M:8:THR:OG1	3:M:75:TRP:CB	2.67	0.41
3:M:16:PHE:CE2	3:M:125:PHE:CZ	3.08	0.41
3:M:218:LEU:H	3:M:218:LEU:CD1	2.33	0.41
3:M:223:HIS:HE1	3:M:476:THR:H	1.61	0.41
3:M:256:VAL:HG11	3:M:290:PHE:HB3	1.95	0.41
3:M:276:VAL:CG1	3:M:299:LEU:CD1	2.98	0.41
3:M:343:ASN:ND2	3:M:343:ASN:H	2.13	0.41
3:M:360:LEU:CG	3:M:362:PHE:CE2	3.03	0.41
1:A:145:ILE:O	1:A:147:LEU:N	2.54	0.41
1:A:170:LEU:HB2	1:A:202:LYS:CG	2.50	0.41
1:A:175:PRO:HG3	1:A:211:ASP:HB2	2.01	0.41
1:A:220:SER:OG	4:S:141:VAL:N	2.26	0.41
1:A:270:LEU:HD12	1:A:274:LEU:CD2	2.50	0.41
1:A:385:LYS:NZ	1:A:445:ASN:OD1	2.52	0.41
1:A:403:LEU:CD2	1:A:421:PRO:O	2.64	0.41
1:A:557:LYS:CD	2:B:606:ASP:HB2	2.50	0.41
1:A:567:GLN:C	1:A:569:ASP:N	2.72	0.41
1:A:589:SER:HB2	1:A:601:VAL:HG22	1.89	0.41
2:B:127:LEU:HD11	2:B:142:LEU:CD1	2.50	0.41
2:B:169:VAL:O	2:B:173:VAL:N	2.47	0.41
2:B:212:VAL:CG1	2:B:248:LEU:CD2	2.97	0.41
2:B:260:LEU:CG	2:B:291:TYR:CE1	2.90	0.41
2:B:285:GLU:N	2:B:285:GLU:CD	2.73	0.41
3:M:244:VAL:CG2	3:M:472:TYR:HE2	2.32	0.41
3:M:246:VAL:CB	3:M:297:PHE:CE1	3.02	0.41
3:M:290:PHE:CD1	3:M:299:LEU:CD1	3.03	0.41

Continued on next page...

Continued from previous page...

Atom-1	Atom-2	Interatomic distance (Å)	Clash overlap (Å)
3:M:306:LEU:HD22	3:M:310:VAL:HG23	2.02	0.41
3:M:311:LYS:O	3:M:312:GLN:C	2.55	0.41
3:M:411:LEU:O	3:M:412:ARG:HG3	2.20	0.41
4:S:58:LEU:HD13	4:S:68:VAL:CG1	2.50	0.41
1:A:73:LYS:O	1:A:76:TYR:HB2	2.21	0.41
1:A:92:LEU:O	1:A:94:VAL:N	2.53	0.41
1:A:104:ARG:N	4:S:167:ILE:HD11	2.35	0.41
1:A:332:TYR:CE2	1:A:336:ILE:HD12	2.55	0.41
1:A:338:PHE:O	1:A:341:ILE:HB	2.21	0.41
1:A:356:ILE:O	1:A:359:LEU:HB2	2.20	0.41
1:A:480:LEU:C	1:A:480:LEU:CD1	2.88	0.41
2:B:252:LEU:CB	2:B:302:PHE:CG	2.99	0.41
2:B:296:ASP:C	2:B:298:ASP:N	2.72	0.41
2:B:389:ILE:O	2:B:393:ILE:CG1	2.68	0.41
2:B:519:ALA:CB	2:B:555:LEU:HD12	2.49	0.41
2:B:528:ASP:HA	2:B:531:ARG:NH1	2.35	0.41
3:M:319:SER:HB2	3:M:346:ASN:C	2.40	0.41
4:S:58:LEU:HA	4:S:68:VAL:HG22	2.02	0.41
1:A:125:THR:OG1	1:A:158:LEU:HB2	2.21	0.41
1:A:175:PRO:HA	1:A:214:VAL:HG21	2.01	0.41
1:A:215:VAL:HG21	1:A:243:ILE:HG23	1.18	0.41
1:A:420:ILE:HA	1:A:421:PRO:HD3	1.83	0.41
1:A:462:GLN:NE2	2:B:1:MET:CE	2.83	0.41
1:A:523:SER:OG	1:A:562:TRP:CD1	2.57	0.41
1:A:626:SER:OG	2:B:616:SER:HB2	2.21	0.41
2:B:63:MET:HB3	2:B:100:LEU:CD1	2.51	0.41
2:B:105:LEU:C	2:B:145:MET:HE1	2.40	0.41
2:B:266:VAL:CA	2:B:289:PRO:HB2	2.48	0.41
2:B:311:TYR:CD2	2:B:342:ALA:HB1	2.53	0.41
2:B:357:GLU:CG	2:B:361:GLN:NE2	2.80	0.41
2:B:458:MET:C	2:B:460:SER:N	2.73	0.41
2:B:533:LEU:O	2:B:536:ASN:N	2.42	0.41
2:B:559:ASP:CG	2:B:563:PHE:CE1	2.92	0.41
3:M:8:THR:OG1	3:M:75:TRP:HB2	2.20	0.41
3:M:223:HIS:C	3:M:479:PHE:CG	2.83	0.41
3:M:374:TYR:O	3:M:390:ILE:CG2	2.68	0.41
1:A:100:LEU:CB	4:S:162:SER:HB2	2.51	0.41
1:A:140:VAL:O	1:A:141:VAL:O	2.38	0.41
1:A:396:VAL:O	1:A:397:ASP:OD1	2.37	0.41
2:B:25:VAL:HG22	2:B:35:TYR:HB3	2.03	0.41
2:B:123:LEU:HB2	2:B:142:LEU:CD1	2.45	0.41

Continued on next page...

Continued from previous page...

Atom-1	Atom-2	Interatomic distance (Å)	Clash overlap (Å)
2:B:135:ARG:HH22	2:B:164:ASP:CB	2.27	0.41
2:B:167:ALA:O	2:B:207:VAL:CG1	2.68	0.41
2:B:537:PHE:CE1	2:B:545:ARG:CB	3.03	0.41
2:B:617:LEU:O	2:B:618:PHE:C	2.58	0.41
3:M:80:THR:O	3:M:81:SER:CB	2.69	0.41
3:M:118:TYR:CD2	3:M:118:TYR:O	2.74	0.41
3:M:221:THR:HG22	3:M:223:HIS:CE1	2.55	0.41
3:M:352:GLN:CB	3:M:401:LYS:O	2.69	0.41
3:M:379:LEU:HD13	3:M:386:PHE:CE1	2.56	0.41
3:M:429:ASP:O	3:M:430:LEU:C	2.57	0.41
1:A:100:LEU:CD1	1:A:141:VAL:HG11	2.49	0.41
1:A:140:VAL:HG13	1:A:176:TYR:HB2	1.87	0.41
1:A:163:ALA:C	1:A:165:ASP:N	2.71	0.41
1:A:222:ILE:HG23	1:A:233:PHE:CB	2.43	0.41
1:A:436:CYS:CB	1:A:450:TYR:CZ	3.04	0.41
1:A:536:MET:HB3	1:A:555:LEU:HD21	2.01	0.41
1:A:559:PHE:CD2	1:A:578:LEU:HD23	2.55	0.41
1:A:609:LEU:C	1:A:611:LEU:N	2.73	0.41
2:B:137:PHE:O	2:B:140:SER:N	2.50	0.41
2:B:159:LYS:CD	2:B:191:GLU:CD	2.89	0.41
2:B:181:TYR:CD2	2:B:218:CYS:CB	2.90	0.41
2:B:212:VAL:CG2	2:B:233:TYR:CD2	3.03	0.41
2:B:257:LYS:O	2:B:260:LEU:HG	2.20	0.41
2:B:319:LEU:C	2:B:321:CYS:N	2.73	0.41
2:B:467:VAL:CG1	2:B:471:TYR:CE1	3.04	0.41
2:B:537:PHE:CA	2:B:598:LEU:HD13	2.37	0.41
3:M:347:PHE:CE2	3:M:350:VAL:CG1	3.04	0.41
1:A:147:LEU:O	1:A:184:ALA:HB1	2.21	0.41
1:A:179:LYS:O	4:S:142:ILE:HD12	2.16	0.41
1:A:212:ILE:CB	4:S:145:ASN:ND2	2.81	0.41
1:A:401:VAL:HG11	1:A:419:ILE:HD12	2.00	0.41
1:A:516:ILE:CD1	1:A:551:LEU:HB2	2.51	0.41
1:A:630:PRO:O	2:B:554:LYS:HB2	2.21	0.41
2:B:78:ASP:OD1	2:B:80:GLN:CB	2.56	0.41
2:B:158:VAL:CB	2:B:177:ILE:HG13	2.48	0.41
2:B:328:LEU:O	2:B:329:ALA:C	2.50	0.41
2:B:344:VAL:HG22	2:B:363:ILE:HD12	1.94	0.41
2:B:520:SER:CA	2:B:523:PHE:CD2	3.03	0.41
2:B:537:PHE:HA	2:B:540:GLU:CD	2.40	0.41
3:M:217:ASP:OD2	3:M:440:ILE:CG2	2.69	0.41
3:M:342:LEU:CD1	3:M:411:LEU:HB3	2.27	0.41

Continued on next page...

Continued from previous page...

Atom-1	Atom-2	Interatomic distance (Å)	Clash overlap (Å)
3:M:410:VAL:CG1	3:M:412:ARG:NH1	2.73	0.41
1:A:99:LYS:HE2	1:A:101:GLN:HB3	2.02	0.41
1:A:375:VAL:O	1:A:378:ILE:N	2.53	0.41
1:A:420:ILE:HG23	1:A:424:TYR:HB2	2.03	0.41
1:A:443:SER:O	1:A:445:ASN:N	2.47	0.41
1:A:461:CYS:SG	1:A:469:LEU:CB	3.02	0.41
1:A:567:GLN:O	1:A:569:ASP:N	2.53	0.41
1:A:588:LEU:C	1:A:590:TYR:N	2.72	0.41
1:A:626:SER:OG	2:B:617:LEU:HG	2.21	0.41
2:B:48:VAL:HG22	2:B:82:TYR:CE2	2.53	0.41
2:B:56:SER:HB3	2:B:92:THR:HG23	1.90	0.41
2:B:106:LEU:HD11	2:B:144:ASP:C	2.29	0.41
2:B:169:VAL:HG12	2:B:173:VAL:HG21	2.01	0.41
2:B:216:LYS:HD3	2:B:251:LEU:CB	2.49	0.41
2:B:231:ARG:C	2:B:233:TYR:H	2.24	0.41
2:B:343:LEU:CD2	2:B:363:ILE:N	2.84	0.41
2:B:444:THR:O	2:B:446:TRP:N	2.53	0.41
2:B:567:GLN:CB	2:B:569:THR:OG1	2.68	0.41
2:B:587:ARG:O	2:B:591:MET:HG2	2.21	0.41
3:M:96:ILE:HD13	3:M:96:ILE:HA	1.94	0.41
3:M:218:LEU:CB	3:M:442:GLN:O	2.69	0.41
3:M:220:GLU:CD	3:M:439:TYR:HB2	2.41	0.41
3:M:222:PHE:CB	3:M:240:ILE:HG12	2.51	0.41
3:M:224:VAL:HB	3:M:479:PHE:HD1	1.84	0.41
3:M:261:ASN:CG	3:M:262:THR:N	2.74	0.41
3:M:356:LEU:HD21	3:M:358:ILE:HG12	1.99	0.41
4:S:4:ALA:HB2	4:S:19:PHE:CD2	2.56	0.41
4:S:117:ASN:CB	4:S:120:ASP:OD2	2.68	0.41
4:S:127:THR:CG2	4:S:153:VAL:HG13	2.51	0.41
1:A:104:ARG:HG3	1:A:145:ILE:CB	2.49	0.41
1:A:154:ILE:HG22	1:A:191:GLN:CG	2.39	0.41
1:A:180:LYS:HE3	4:S:156:LEU:CD1	2.50	0.41
1:A:509:PRO:O	1:A:547:VAL:HG22	2.22	0.41
1:A:563:CYS:SG	1:A:621:LEU:CG	3.08	0.41
2:B:13:ASP:O	2:B:16:LYS:O	2.38	0.41
2:B:95:THR:HA	2:B:134:LEU:HD23	2.02	0.41
2:B:143:SER:O	2:B:179:LYS:CB	2.69	0.41
2:B:151:ALA:HB3	2:B:188:TYR:CE1	2.56	0.41
2:B:178:ILE:CD1	2:B:214:ALA:C	2.82	0.41
2:B:309:LEU:HD13	2:B:309:LEU:HA	1.87	0.41
2:B:336:ASN:CB	2:B:339:PHE:CD1	3.03	0.41

Continued on next page...

Continued from previous page...

Atom-1	Atom-2	Interatomic distance (Å)	Clash overlap (Å)
3:M:323:MET:CE	3:M:342:LEU:CG	2.99	0.41
3:M:436:GLU:HG3	3:M:436:GLU:O	2.21	0.41
1:A:100:LEU:HD23	4:S:162:SER:OG	2.20	0.40
1:A:200:PHE:CZ	1:A:235:GLN:HB2	2.56	0.40
1:A:316:LEU:HD11	1:A:348:PHE:CE2	2.43	0.40
1:A:338:PHE:CE2	1:A:352:PHE:CE2	3.08	0.40
1:A:376:GLU:CD	1:A:376:GLU:N	2.73	0.40
1:A:594:PHE:CE1	2:B:434:LYS:CD	2.98	0.40
2:B:102:HIS:CD2	2:B:123:LEU:HD21	2.56	0.40
2:B:123:LEU:HB2	2:B:142:LEU:HD21	2.03	0.40
2:B:159:LYS:CA	2:B:195:ILE:HD13	2.39	0.40
2:B:170:ARG:O	2:B:171:GLY:C	2.58	0.40
2:B:178:ILE:HA	2:B:178:ILE:HD13	1.89	0.40
2:B:334:MET:C	2:B:336:ASN:N	2.73	0.40
2:B:374:PHE:CD2	2:B:402:LEU:HD21	2.53	0.40
2:B:518:ILE:O	2:B:518:ILE:CG1	2.69	0.40
3:M:12:ASN:OD1	3:M:45:SER:OG	2.35	0.40
3:M:66:PHE:HB3	3:M:77:LEU:HD21	1.78	0.40
3:M:240:ILE:HG22	3:M:444:ALA:CA	2.51	0.40
3:M:446:GLY:C	3:M:448:TYR:H	2.21	0.40
3:M:478:ASN:C	3:M:479:PHE:O	2.59	0.40
1:A:71:VAL:HG11	1:A:105:VAL:CB	2.51	0.40
1:A:78:GLU:OE1	1:A:113:SER:HB3	2.20	0.40
1:A:92:LEU:CD2	1:A:124:ALA:N	2.85	0.40
1:A:92:LEU:HD22	1:A:123:LEU:CB	2.50	0.40
1:A:141:VAL:HB	4:S:159:ALA:CA	2.46	0.40
1:A:145:ILE:C	1:A:147:LEU:N	2.72	0.40
1:A:182:ILE:HD13	1:A:218:ALA:N	2.37	0.40
1:A:251:TRP:CD1	4:S:104:THR:HG23	2.55	0.40
1:A:420:ILE:HG23	1:A:421:PRO:CD	2.48	0.40
1:A:429:VAL:CG2	1:A:469:LEU:HD11	2.42	0.40
1:A:461:CYS:O	1:A:463:ASP:N	2.49	0.40
1:A:629:LEU:O	1:A:631:SER:N	2.52	0.40
2:B:20:ARG:HH11	2:B:35:TYR:HE1	1.67	0.40
2:B:37:TYR:O	2:B:40:GLN:C	2.59	0.40
2:B:139:LEU:HD23	2:B:173:VAL:HA	0.42	0.40
2:B:258:GLN:HA	2:B:258:GLN:OE1	2.21	0.40
2:B:565:GLN:C	2:B:574:ASN:HD21	2.20	0.40
3:M:292:PRO:HA	3:M:293:PRO:HD3	1.53	0.40
3:M:360:LEU:CD1	3:M:433:VAL:CB	2.93	0.40
3:M:433:VAL:O	3:M:435:LEU:HD12	2.21	0.40

Continued on next page...

Continued from previous page...

Atom-1	Atom-2	Interatomic distance (Å)	Clash overlap (Å)
4:S:90:ASP:C	4:S:92:GLN:H	2.25	0.40
4:S:108:SER:OG	4:S:149:ILE:CG2	2.60	0.40
1:A:102:GLN:CG	4:S:164:ASP:C	2.89	0.40
1:A:104:ARG:CG	1:A:145:ILE:CD1	2.95	0.40
1:A:231:GLN:CB	1:A:232:PRO:HD3	2.44	0.40
1:A:247:ILE:HG21	1:A:252:ILE:CG2	2.52	0.40
1:A:247:ILE:HG21	1:A:252:ILE:HB	2.03	0.40
1:A:392:MET:CE	1:A:460:LEU:HD12	2.51	0.40
1:A:535:ILE:O	1:A:536:MET:HG3	2.20	0.40
2:B:208:ILE:HD13	2:B:240:LEU:HD11	2.04	0.40
2:B:286:ILE:CG2	2:B:288:TYR:CE2	2.82	0.40
2:B:306:LEU:O	2:B:309:LEU:HB2	2.21	0.40
2:B:363:ILE:CG2	2:B:398:ILE:HD11	2.47	0.40
2:B:566:ALA:HB2	2:B:581:TYR:CD2	2.56	0.40
3:M:249:TYR:HE1	3:M:467:TYR:CZ	2.37	0.40
3:M:320:ILE:CG2	3:M:439:TYR:OH	2.70	0.40
3:M:376:ILE:HG22	3:M:379:LEU:CD1	2.51	0.40
4:S:85:PHE:HZ	4:S:109:LEU:HD23	1.85	0.40
1:A:84:MET:O	1:A:85:ALA:O	2.36	0.40
1:A:125:THR:HA	1:A:158:LEU:HD13	2.04	0.40
1:A:141:VAL:HG21	4:S:157:ASN:N	2.36	0.40
1:A:149:GLY:C	1:A:151:SER:N	2.72	0.40
1:A:150:LEU:CG	1:A:162:ILE:HG12	2.52	0.40
1:A:174:ARG:HA	1:A:175:PRO:HD2	1.66	0.40
1:A:175:PRO:CG	1:A:211:ASP:OD2	2.69	0.40
1:A:259:LEU:C	1:A:259:LEU:HD23	2.41	0.40
1:A:260:PHE:CZ	1:A:274:LEU:CG	3.04	0.40
1:A:288:THR:HB	1:A:322:PHE:CZ	2.42	0.40
1:A:313:MET:CA	1:A:348:PHE:CZ	3.02	0.40
1:A:326:GLN:HA	1:A:331:ARG:NE	2.36	0.40
1:A:359:LEU:HD22	1:A:367:ILE:HG21	2.03	0.40
1:A:504:ILE:O	1:A:506:LYS:N	2.52	0.40
1:A:557:LYS:HG2	2:B:606:ASP:N	2.36	0.40
2:B:17:VAL:CB	3:M:119:ASP:OD2	2.69	0.40
2:B:41:ASN:HD22	2:B:43:ASN:HA	1.87	0.40
2:B:105:LEU:C	2:B:145:MET:CE	2.90	0.40
2:B:132:SER:C	2:B:169:VAL:HG23	2.41	0.40
2:B:171:GLY:O	2:B:174:ALA:HB3	2.22	0.40
2:B:257:LYS:CA	2:B:260:LEU:HG	2.51	0.40
2:B:278:PRO:HB3	2:B:289:PRO:O	2.22	0.40
2:B:306:LEU:HD22	2:B:321:CYS:CB	2.52	0.40

Continued on next page...

Continued from previous page...

Atom-1	Atom-2	Interatomic distance (Å)	Clash overlap (Å)
2:B:319:LEU:O	2:B:321:CYS:N	2.55	0.40
2:B:353:GLN:HE21	3:M:47:SER:CB	1.91	0.40
2:B:542:PRO:O	2:B:607:ILE:HD13	2.22	0.40
3:M:7:ILE:HG22	3:M:75:TRP:O	2.17	0.40
3:M:104:PHE:O	3:M:105:ASP:CB	2.69	0.40
4:S:87:PHE:CD1	4:S:87:PHE:N	2.89	0.40
1:A:192:TYR:CD2	1:A:192:TYR:O	2.75	0.40
1:A:220:SER:CB	4:S:142:ILE:CA	3.00	0.40
1:A:220:SER:CB	4:S:142:ILE:HA	2.49	0.40
1:A:566:PHE:CZ	1:A:618:THR:HB	2.53	0.40
1:A:637:GLU:HB2	2:B:513:TRP:CG	2.57	0.40
2:B:38:TYR:CA	2:B:42:ILE:H	2.13	0.40
2:B:64:LYS:CG	2:B:100:LEU:CD1	2.98	0.40
2:B:70:MET:CB	2:B:104:TYR:CE1	2.99	0.40
2:B:84:ALA:O	2:B:87:VAL:HB	2.21	0.40
2:B:200:MET:HE3	2:B:232:ARG:N	2.24	0.40
2:B:361:GLN:HG2	2:B:394:TRP:CE2	2.56	0.40
2:B:451:MET:CE	2:B:489:ILE:CG1	2.98	0.40
3:M:220:GLU:HA	3:M:474:THR:CG2	2.50	0.40
3:M:270:PRO:N	3:M:302:TYR:CD1	2.90	0.40

There are no symmetry-related clashes.

5.3 Torsion angles (i)

5.3.1 Protein backbone (i)

In the following table, the Percentiles column shows the percent Ramachandran outliers of the chain as a percentile score with respect to all PDB entries followed by that with respect to all EM entries.

The Analysed column shows the number of residues for which the backbone conformation was analysed, and the total number of residues.

Mol	Chain	Analysed	Favoured	Allowed	Outliers	Percentiles	
1	A	574/964 (60%)	511 (89%)	39 (7%)	24 (4%)	3	22
2	B	619/809 (76%)	474 (77%)	79 (13%)	66 (11%)	0	8
3	M	385/483 (80%)	315 (82%)	52 (14%)	18 (5%)	2	21
4	S	166/194 (86%)	150 (90%)	12 (7%)	4 (2%)	6	33
All	All	1744/2450 (71%)	1450 (83%)	182 (10%)	112 (6%)	3	16

All (112) Ramachandran outliers are listed below:

Mol	Chain	Res	Type
1	A	267	GLU
1	A	278	ILE
1	A	279	LEU
1	A	305	GLU
1	A	401	VAL
1	A	405	THR
1	A	440	ASN
1	A	441	TYR
1	A	449	TRP
1	A	507	GLN
2	B	14	THR
2	B	18	ILE
2	B	19	THR
2	B	43	ASN
2	B	78	ASP
2	B	154	ILE
2	B	156	HIS
2	B	176	ALA
2	B	177	ILE
2	B	179	LYS
2	B	192	LEU
2	B	194	ASP
2	B	196	LEU
2	B	197	LYS
2	B	198	GLU
2	B	200	MET
2	B	215	TYR
2	B	218	CYS
2	B	228	GLY
2	B	261	PRO
2	B	274	PRO
2	B	287	GLU
2	B	296	ASP
2	B	436	LEU
2	B	558	TYR
2	B	560	ILE
2	B	561	ASP
2	B	564	LYS
2	B	565	GLN
2	B	568	VAL
2	B	578	PRO
2	B	583	PHE

Continued on next page...

Continued from previous page...

Mol	Chain	Res	Type
3	M	105	ASP
3	M	282	VAL
3	M	352	GLN
3	M	447	ILE
3	M	470	ALA
4	S	50	PHE
1	A	116	LYS
1	A	126	ASN
1	A	174	ARG
1	A	536	MET
2	B	3	ASP
2	B	29	LYS
2	B	135	ARG
2	B	144	ASP
2	B	158	VAL
2	B	178	ILE
2	B	199	LEU
2	B	227	HIS
2	B	332	LEU
2	B	383	VAL
2	B	432	ALA
3	M	45	SER
3	M	72	LEU
3	M	82	LYS
3	M	316	ARG
3	M	317	MET
3	M	429	ASP
1	A	256	LEU
1	A	421	PRO
1	A	571	ARG
1	A	608	ARG
2	B	15	ALA
2	B	38	TYR
2	B	232	ARG
2	B	337	THR
2	B	503	LEU
2	B	538	SER
2	B	590	GLN
3	M	86	PRO
3	M	254	PRO
3	M	355	ASP
1	A	306	GLU

Continued on next page...

Continued from previous page...

Mol	Chain	Res	Type
1	A	406	GLY
2	B	213	LEU
2	B	222	HIS
2	B	260	LEU
2	B	523	PHE
2	B	600	LYS
3	M	118	TYR
3	M	363	ASN
4	S	15	ARG
4	S	81	ALA
1	A	419	ILE
2	B	20	ARG
2	B	142	LEU
2	B	535	GLN
2	B	573	GLU
3	M	57	GLY
1	A	302	ASN
1	A	439	ASP
2	B	37	TYR
2	B	155	LEU
2	B	372	THR
2	B	42	ILE
2	B	542	PRO
3	M	268	GLY
2	B	8	ILE
2	B	347	VAL
4	S	26	PRO
1	A	601	VAL

5.3.2 Protein sidechains (i)

In the following table, the Percentiles column shows the percent sidechain outliers of the chain as a percentile score with respect to all PDB entries followed by that with respect to all EM entries.

The Analysed column shows the number of residues for which the sidechain conformation was analysed, and the total number of residues.

Mol	Chain	Analysed	Rotameric	Outliers	Percentiles	
1	A	536/898 (60%)	527 (98%)	9 (2%)	60	78
2	B	564/738 (76%)	548 (97%)	16 (3%)	43	65
3	M	353/441 (80%)	342 (97%)	11 (3%)	40	62

Continued on next page...

Continued from previous page...

Mol	Chain	Analysed	Rotameric	Outliers	Percentiles	
4	S	157/175 (90%)	155 (99%)	2 (1%)	69	81
All	All	1610/2252 (72%)	1572 (98%)	38 (2%)	51	69

All (38) residues with a non-rotameric sidechain are listed below:

Mol	Chain	Res	Type
1	A	175	PRO
1	A	186	PHE
1	A	196	LEU
1	A	241	TYR
1	A	288	THR
1	A	292	TYR
1	A	376	GLU
1	A	418	ILE
1	A	439	ASP
2	B	1	MET
2	B	4	SER
2	B	10	SER
2	B	12	LEU
2	B	14	THR
2	B	16	LYS
2	B	19	THR
2	B	29	LYS
2	B	30	LEU
2	B	233	TYR
2	B	261	PRO
2	B	285	GLU
2	B	352	ASN
2	B	418	TYR
2	B	453	TRP
2	B	592	TYR
3	M	68	VAL
3	M	100	LEU
3	M	118	TYR
3	M	214	LEU
3	M	293	PRO
3	M	343	ASN
3	M	352	GLN
3	M	437	TYR
3	M	439	TYR
3	M	472	TYR
3	M	479	PHE

Continued on next page...

Continued from previous page...

Mol	Chain	Res	Type
4	S	38	LEU
4	S	47	GLN

Sometimes sidechains can be flipped to improve hydrogen bonding and reduce clashes. All (26) such sidechains are listed below:

Mol	Chain	Res	Type
1	A	101	GLN
1	A	102	GLN
1	A	199	ASN
1	A	235	GLN
1	A	345	ASN
1	A	440	ASN
1	A	462	GLN
1	A	565	ASN
2	B	41	ASN
2	B	156	HIS
2	B	307	ASN
2	B	333	GLN
2	B	352	ASN
2	B	441	GLN
2	B	486	HIS
2	B	547	GLN
2	B	567	GLN
2	B	574	ASN
3	M	17	GLN
3	M	117	ASN
3	M	241	HIS
3	M	326	HIS
3	M	343	ASN
3	M	346	ASN
4	S	47	GLN
4	S	103	GLN

5.3.3 RNA (i)

There are no RNA molecules in this entry.

5.4 Non-standard residues in protein, DNA, RNA chains (i)

There are no non-standard protein/DNA/RNA residues in this entry.

5.5 Carbohydrates (i)

There are no monosaccharides in this entry.

5.6 Ligand geometry (i)

There are no ligands in this entry.

5.7 Other polymers (i)

There are no such residues in this entry.

5.8 Polymer linkage issues (i)

There are no chain breaks in this entry.

6 Map visualisation (i)

This section contains visualisations of the EMDB entry EMD-13189. These allow visual inspection of the internal detail of the map and identification of artifacts.

No raw map or half-maps were deposited for this entry and therefore no images, graphs, etc. pertaining to the raw map can be shown.

6.1 Orthogonal projections (i)

6.1.1 Primary map

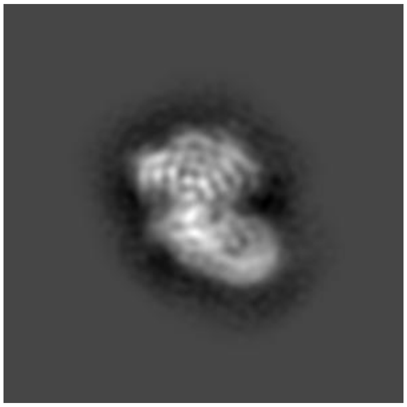
X

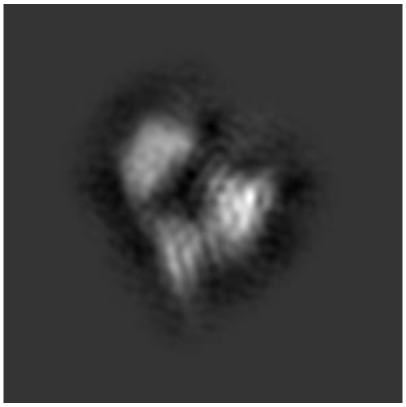
Y

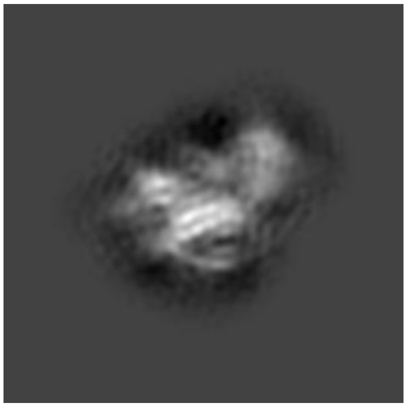
Z

The images above show the map projected in three orthogonal directions.

6.2 Central slices (i)

6.2.1 Primary map

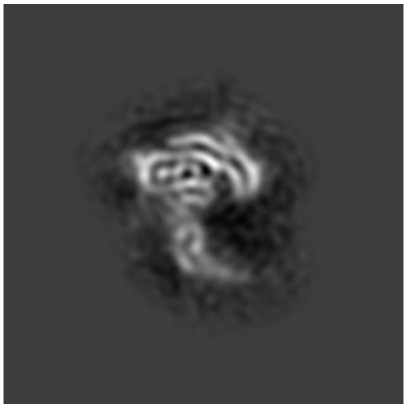
X Index: 132

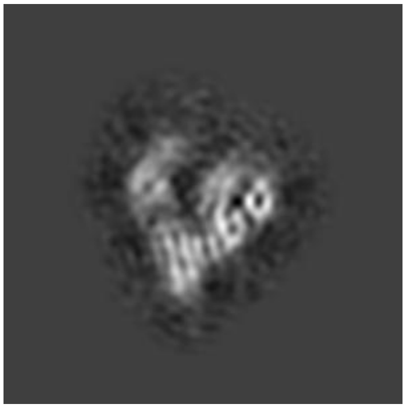
Y Index: 132

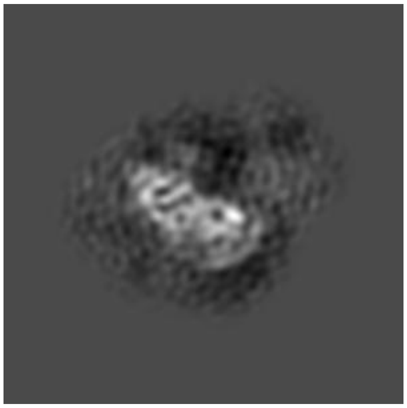
Z Index: 132

The images above show central slices of the map in three orthogonal directions.

6.3 Largest variance slices

6.3.1 Primary map

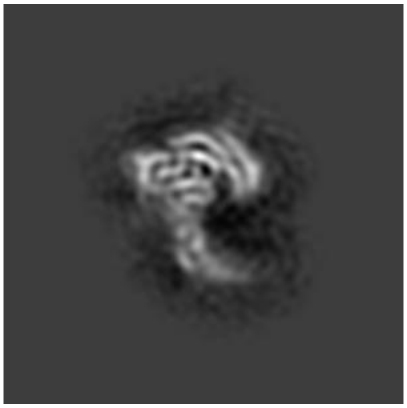

X Index: 134

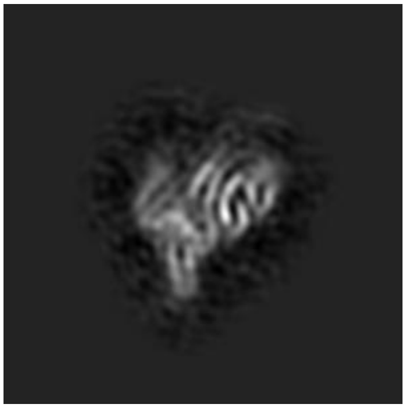

Y Index: 123

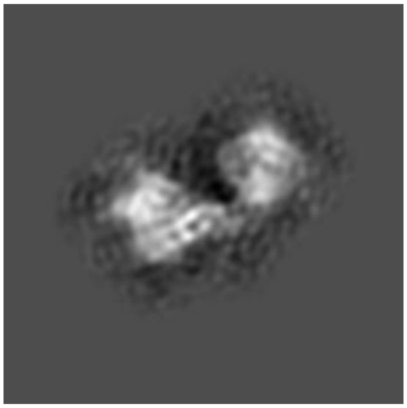

Z Index: 112

The images above show the largest variance slices of the map in three orthogonal directions.

6.4 Orthogonal surface views

6.4.1 Primary map

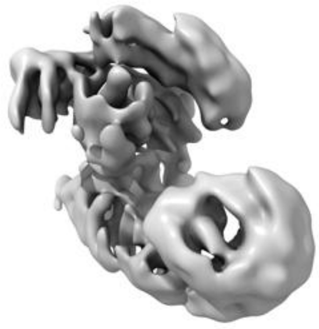

X

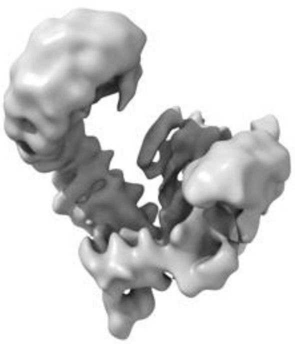

Y

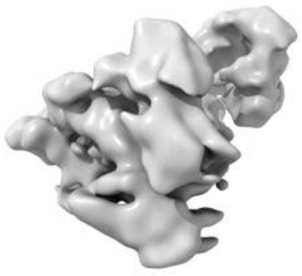

Z

The images above show the 3D surface view of the map at the recommended contour level 0.0155. These images, in conjunction with the slice images, may facilitate assessment of whether an appropriate contour level has been provided.

6.5 Mask visualisation (i)

This section was not generated. No masks/segmentation were deposited.

7 Map analysis

This section contains the results of statistical analysis of the map.

7.1 Map-value distribution

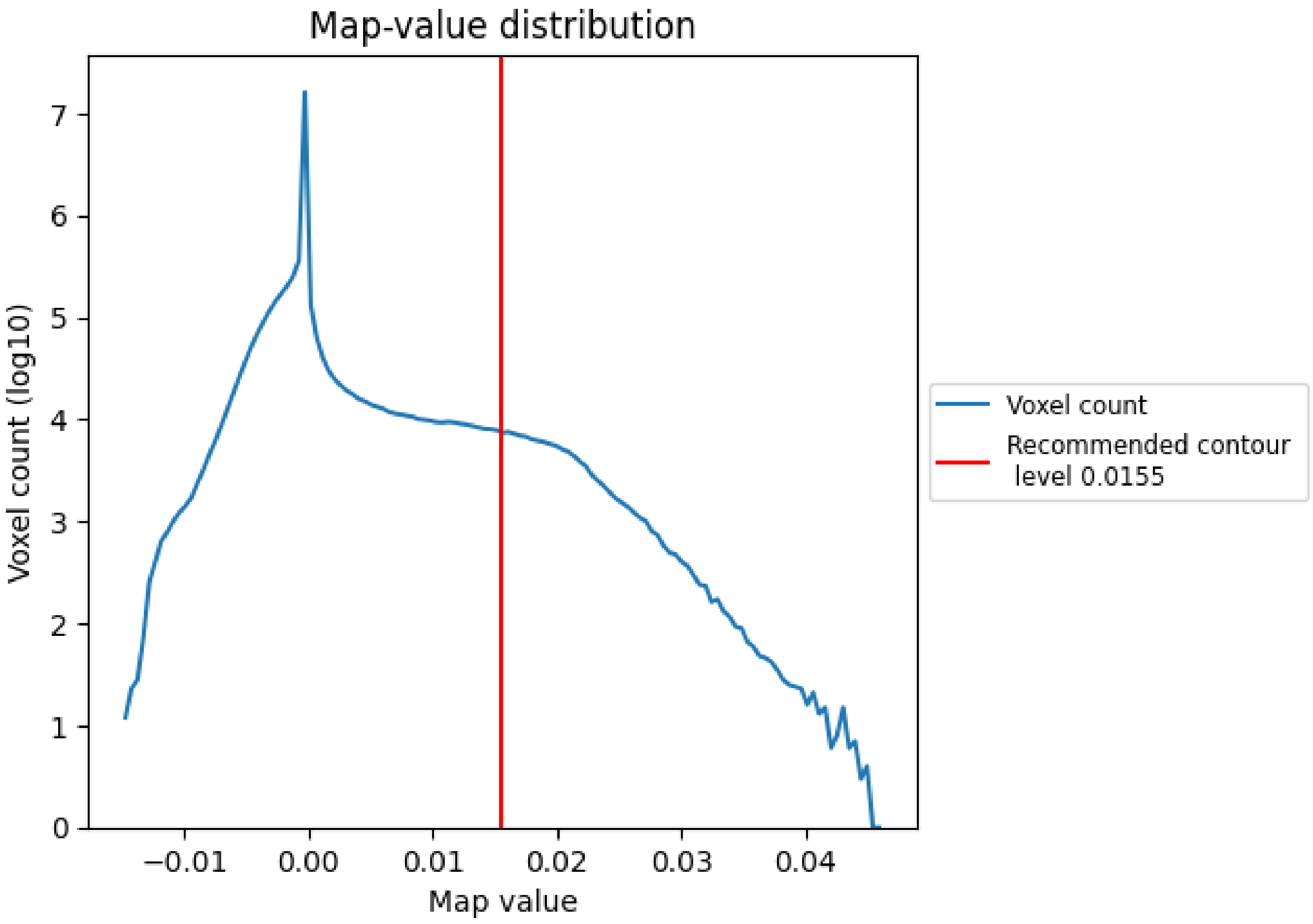

The map-value distribution is plotted in 128 intervals along the x-axis. The y-axis is logarithmic. A spike in this graph at zero usually indicates that the volume has been masked.

7.2 Volume estimate (i)

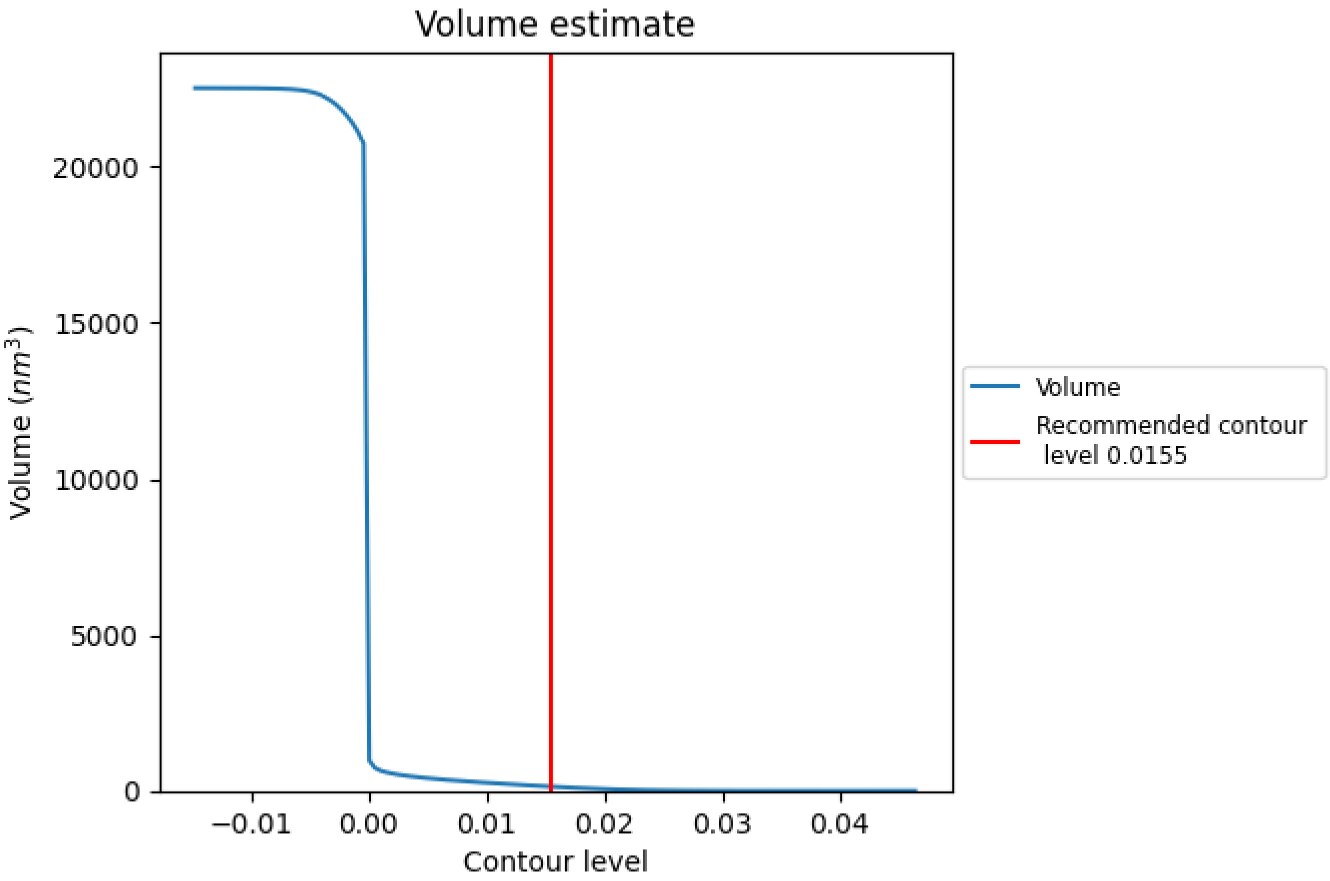

The volume at the recommended contour level is 137 nm^3; this corresponds to an approximate mass of 124 kDa.

The volume estimate graph shows how the enclosed volume varies with the contour level. The recommended contour level is shown as a vertical line and the intersection between the line and the curve gives the volume of the enclosed surface at the given level.

7.3 Rotationally averaged power spectrum (i)

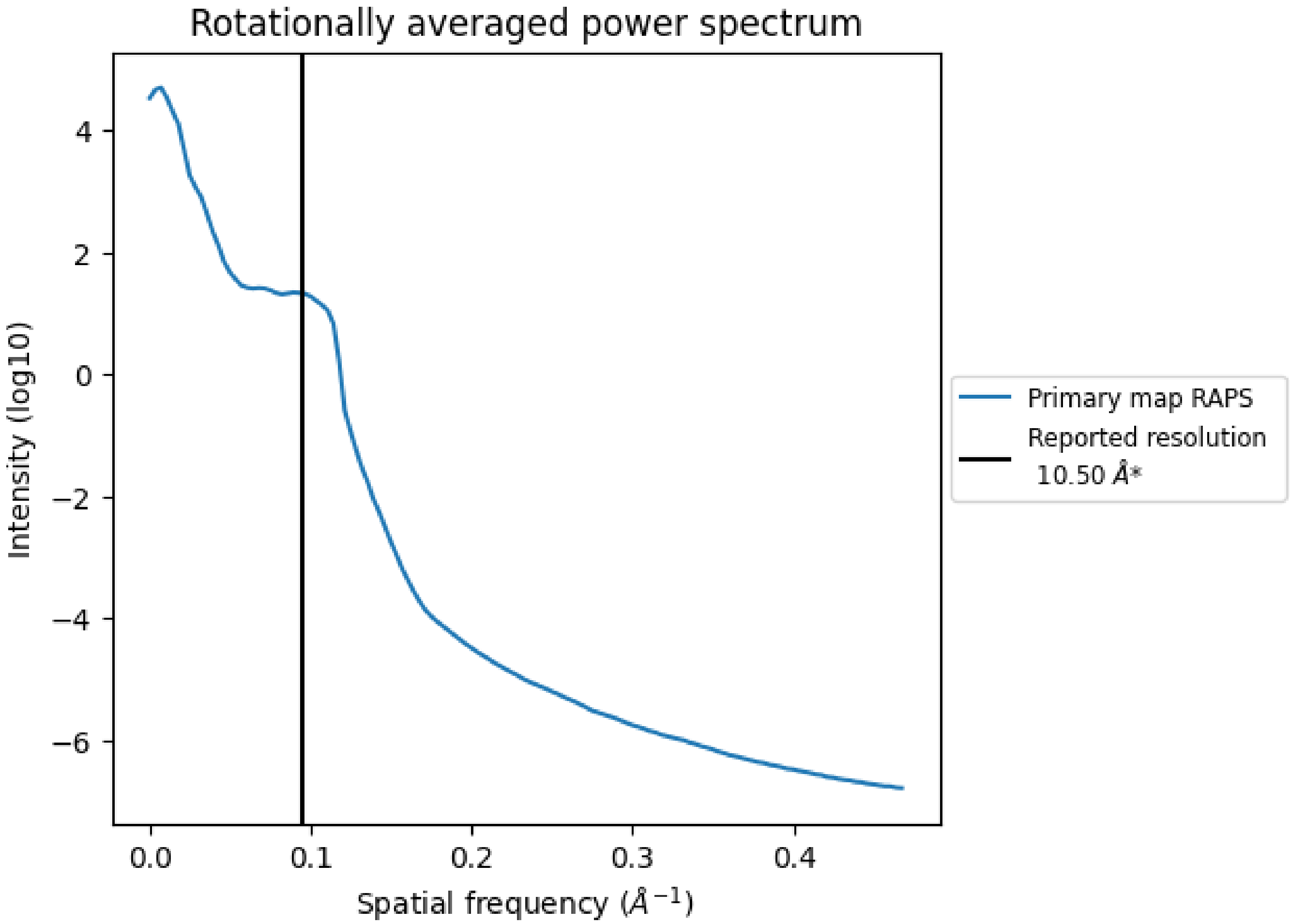

*Reported resolution corresponds to spatial frequency of 0.095 Å^{-1}

8 Fourier-Shell correlation (i)

This section was not generated. No FSC curve or half-maps provided.

9 Map-model fit ⓘ

This section contains information regarding the fit between EMDB map EMD-13189 and PDB model 7P3Z. Per-residue inclusion information can be found in section 3 on page 5.

9.1 Map-model overlay ⓘ

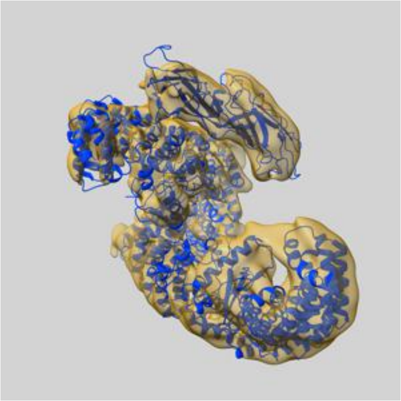

X

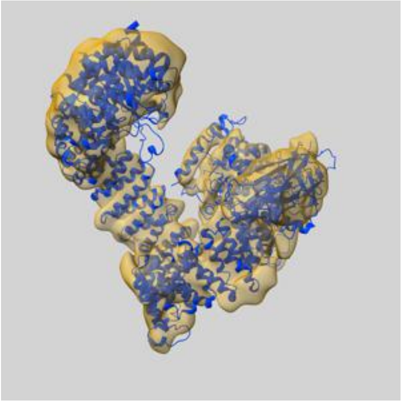

Y

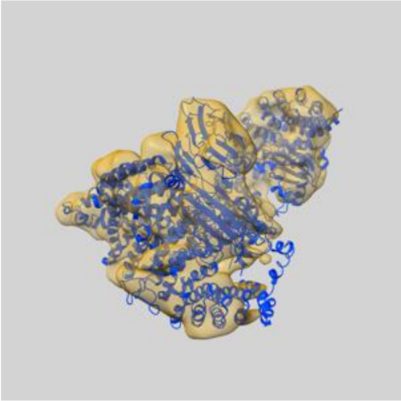

Z

The images above show the 3D surface view of the map at the recommended contour level 0.0155 at 50% transparency in yellow overlaid with a ribbon representation of the model coloured in blue. These images allow for the visual assessment of the quality of fit between the atomic model and the map.

9.2 Atom inclusion (i)

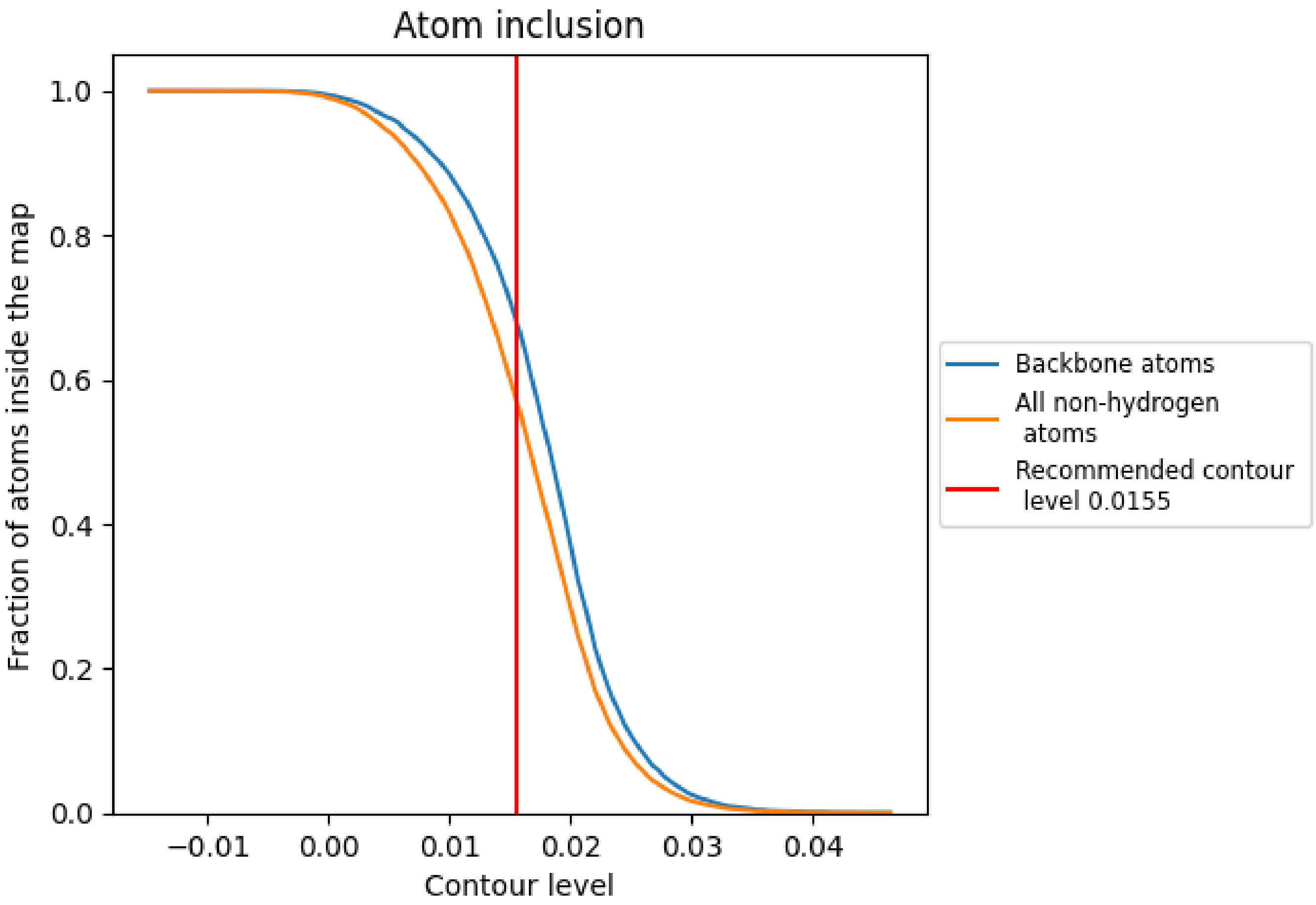

At the recommended contour level, 68% of all backbone atoms, 57% of all non-hydrogen atoms, are inside the map.
